# ABCC Transporter Gene *MoABC-R1* Is Associated with Pyraclostrobin Tolerance in *Magnaporthe oryzae*

**DOI:** 10.3390/jof9090917

**Published:** 2023-09-11

**Authors:** Pei Hu, Yanchen Liu, Xiaoli Zhu, Houxiang Kang

**Affiliations:** State Key Laboratory for Biology of Plant Diseases and Insect Pests, Institute of Plant Protection, Chinese Academy of Agricultural Sciences, Beijing 100193, China; hupei733246@163.com (P.H.); liuyc@big.ac.cn (Y.L.); shirleyz8922@gmail.com (X.Z.)

**Keywords:** rice blast, fungicide tolerance, ABC transporter, pyraclostrobin, pathogenicity

## Abstract

Rice blast is a worldwide fungal disease that poses a threat to food security. Fungicide treatment is one of the most effective methods to control rice blast disease. However, the emergence of fungicide tolerance hampers the control efforts against rice blast. ATP-binding cassette (ABC) transporters have been found to be crucial in multidrug tolerance in various phytopathogenic fungi. This study investigated the association between polymorphisms in 50 ABC transporters and pyraclostrobin sensitivity in 90 strains of rice blast fungus. As a result, we identified *MoABC-R1*, a gene associated with fungicide tolerance. *MoABC-R1* belongs to the ABCC-type transporter families. Deletion mutants of *MoABC-R1*, *abc-r1*, exhibited high sensitivity to pyraclostrobin at the concentration of 0.01 μg/mL. Furthermore, the pathogenicity of *abc-r1* was significantly diminished. These findings indicate that *MoABC-R1* not only plays a pivotal role in fungicide tolerance but also regulates the pathogenicity of rice blast. Interestingly, the combination of *MoABC-R1* deletion with fungicide treatment resulted in a three-fold increase in control efficiency against rice blast. This discovery highlights *MoABC-R1* as a potential target gene for the management of rice blast.

## 1. Introduction

Fungicide treatment is a crucial part of the integrated control system for plant pathogenic fungi diseases. However, it has led to the accelerated emergence of fungicide-tolerant strains in many filamentous fungi, including *Botrytis cinerea* [1], *Mycosphaerella graminicola* [2], and *Penicillium digitatum* [3]. As early as the 1970s, rice blast was found to be tolerant to kasugamycin and Kitazin P (IBP) [4]. Hereafter, several other cases of fungicide tolerance have been discovered in rice blast [5,6,7]. The emergence of fungicide tolerance in rice blast necessitates the alternating use of different fungicides and the development of new fungicides.

Pyraclostrobin, a Qo-inhibiting (QoI) fungicide recently approved in China, can inhibit pathogenic fungi invasion by disrupting their mitochondrial respiration [8]. However, mutation on specific target sites of QoI (Quinone outside Inhibitor) fungicides raises a significant concern regarding the emergence of resistant pathogens [9] such as rice blast, which has been found to resist QoI fungicides like azoxystrobin [10], enestroburin, and SYP-1620 [11]. Previous studies on QoI fungicide tolerance mechanisms in *M. oryzae* mainly focused on the mutation of the target gene *CYTB* [10,12,13], or alternative respiration in response to QoI action [14]. Recently, drug transporters resulting in fungicide tolerance via pumping toxic components out of fungal cells have been reported [15]. The ATP-binding cassette (ABC) transporters are one of the most significant efflux pumps, leading pathogens against all major classes of fungicides [5,16,17]. However, research concerning drug transporters that can efflux fungicides in pathogenic fungi is limited [18], hindering their potential development and utilization as fungicide targets.

ABC transporters, one of the largest transmembrane transporter families, are found in all bacteria, fungi, plants, and animals, and are responsible for a wide spectrum of substrate translocation, driven by the energy of ATP hydrolysis [19]. The typical structure of ABC transporters consists of two transmembrane domains (TMDs) embedded in the plasma membrane or organelle membrane, along with two conserved nucleotide binding domains (NBDs or ABCs) located in the cytoplasm [19,20]. The TMDs form transmembrane channels [21] and exhibit significant variations in sequences, sizes, and 3D structures, which contribute to the polyspecificity or high selectivity of ABC transporters toward substrates [22,23]. The NBDs contain highly conserved motifs, including LSGGQ or C-motif, Walker A, Walker B, and the ABC signature motif (C-loop) [24,25], which are involved in Mg-ATP binding and phosphate-bond hydrolyzing [26,27]. Generally, ABC transporters can be classified as importers or exporters based on whether the substrate is bound in the cytoplasm or released extracellularly [19,28]. Importers are rare in eukaryotes, with exceptions such as ABCA4 [29], CFTR, SUR1, and SUR2 in humans [30]. Exporters, on the other hand, are found in both eukaryotic and prokaryotic organisms [19]. The importer mainly uptakes nutrients and other metabolites, whereas the exporter pumps antibiotics, lipids, and proteins to extracellular or intercellular space [31,32]. In addition to their role in substrate transportation, ABC transporters also play crucial physiological roles as non-transporters in the cytoplasm [21]. These functions include maintaining and repairing DNA [33], participating in mycelium formation in *Candida albicans* [34], regulating the lifespan in *drosophila* [35], and contributing to pathogenesis in various pathogenic fungi [20,36,37]. The diverse functions of ABC transporters highlight their indispensable role within organisms.

ABC transporters have garnered significant attention and thorough understanding due to their involvement in multidrug tolerance in cancer [38,39]. The ABCB (MDR), ABCG (PDR), and ABCC (MRP) subfamilies have been extensively studied for their role in drug tolerance [40,41]. Recently, ABC transporters have also been reported in fungicide tolerance of various filamentous fungi [1,5]. For example, *BcatrB* can be induced by fenpiclonil [1]; *CaABC1* shows high expression levels during iprobenfos, kresoxim-methyl, and thiophanate-methyl treatment [42]; and *FgABCC9* is up-regulated by tebuconazole [37]. However, the functional characterization of ABC transporters in filamentous fungi, particularly in rice blast, remains limited. There are 50 ABC transporters identified in the *M. oryzae* genome, but only six of them have been cloned and characterized [43]. Among these, ABC1 [44], ABC4 [45], and MoABC5 [43] are involved in the pathogenic process, while ABC2 [46], ABC3 [47], and MoABC6 [43] play a role in abiotic stress tolerance.

In this study, we identified a fungicide tolerance-associated gene, *MoABC-R1*, through fungicide sensitivity assays and the genomic sequence analysis of the ABC transporter genes. Structural and functional prediction analyses revealed that *MoABC-R1* encodes an ABCC (MRP) transporter. To investigate the role of *MoABC-R1* in fungicide tolerance, we generated *MoABC-R1* knockout mutants and assessed their phenotypes. Additionally, we found that *MoABC-R1* significantly impacts the pathogenicity of *M. oryzae* via playing a crucial role in appressorium formation. Based on the functional characterization of *MoABC-R1*, we propose and validate an enhanced efficacy of fungicide for rice blast management when *MoABC-R1* is disrupted. This study not only enhances our understanding of fungicide tolerance in *M. oryzae* but also has potential implications for other pathogenic fungi. 

## 2. Materials and Methods

### 2.1. Primers Used in This Study

All of the primer sequences used in this study are listed in Supplementary Table S1.

### 2.2. Fungal Cultivation

The following fungal strains were maintained: YN226, mutants of *MoABC-R1* (*MGG_05044*), and mutants of *MGG_04899* on oatmeal agar medium (OMA) at 25 °C in dark conditions.

### 2.3. Conidia Production

Fungal pieces (5 mm) taken from the petri dish were cultured on the center of the oatmeal agar medium at 25 °C under light conditions for ten days to produce conidia.

### 2.4. Mycelia Collection

Conidial suspensions (5 × 10^4^ conidia/mL) from rice blast isolates were cultured in 100 mL of liquid complete medium at 28 °C for two days in an orbital shaker (200 rpm).

### 2.5. Vegetative Growth Rates

Fungal pieces (5 mm) taken from the petri dish were cultured on the center of CM plates at 25 °C in dark conditions for seven days to measure the vegetative growth rates.

### 2.6. Feature and Function Prediction for MoABC-R1

The protein structure of *MoABC-R1* was obtained from the InterPro database (http://www.ebi.ac.uk/interpro (accessed on 6 July 2016)), NCBI CCD (https://www.ncbi.nlm.nih.gov/Structure/cdd/ (accessed on 6 July 2016)) and TMHMM (https://services.healthtech.dtu.dk/services/TMHMM-2.0/ (accessed on 6 July 2016)). Phylogenetic analysis was performed based on protein sequences using TBtools 1.068 software. The three-dimensional structure of MoABC-R1 was predicted using PyMoL2.5 software.

### 2.7. Generation of MoABC-R1 Disrupted Mutants

*MoABC-R1* and *MGG_04899* knockout mutants were generated via a homologous recombination strategy, which replaced the major part of the coding region of *MoABC-R1* with the hygromycin B in wild-type strain YN226, and putative mutants were confirmed preliminarily with the selecting medium containing hygromycin B, further confirmed via PCR in DNA and cDNA. 

### 2.8. Generation of MoABC-R1 Complement Strains

First, the *MoABC-R1* gene with its native promoter (approximately 1.5 kb) was amplified in the wild-type YN226. The yeast shuttle vectors pYF11, carrying the zeocin (Invitrogen) tolerance gene, were digested with XhoI restriction enzyme (New England Biolabs). Next, the integration of *MoABC-R1* with linearized pYF11 was performed through yeast transformation in Mav203, and the positive fusion constructs were confirmed using a SD-Trp medium [48]. The fusion constructs were then amplified and collected in Escherichia coli. Finally, the fusion constructs were transformed into *MoABC-R1* knockout mutants, and putative complement mutants were preliminarily confirmed using a selected medium containing zeocin. Further confirmation was performed using PCR and qRT-PCR (All primer sequences are listed in Supplementary Table S1).

### 2.9. Appressorium Formation Assay

Approximately 10 μL of spore suspension (5 × 10^4^ conidia/mL) was placed on a hydrophobic surface (Fisherfinest, Premium Cover Glass, Jiangsu, China) and incubated under moist conditions for 8 h. Approximately 200 μL of the sample was used for each assay. The appressorium formation of 500 spores was statistically evaluated under a microscope.

### 2.10. Fungicide and Salt Stress Sensitivity Assay

CM solid media were prepared with a final concentration of 0.01 μg/mL of pyraclostrobin (Huaxia Regent, Chengdu, China), 0.7 M NaCl, or 0.6 M KCl, respectively. Fungal plugs (5 mm) were cultured on the center of CM plates with or without treatment and incubated at 25 °C in dark conditions for seven days. The diameter was determined using the crossing method, with three technical replicates and three biological repeats for each treatment.

### 2.11. MoABC-R1 Response to Fungicide and Salt Stress

CM liquid media were prepared with a final concentration of 0.01 μg/mL pyraclostrobin, 0.7 M NaCl, or 0.6 M KCl, respectively. Wild-type YN226 mycelia were collected and washed with sterilized distilled water, then transferred to the liquid CM with or without various stressors and cultured for different time points (0, 1, 3, 5, 7, 9, 12 h). Disease lesions were harvested, immediately frozen in liquid nitrogen, and stored at −80 °C. The total RNA was isolated using Trizol reagent (Sangon Biotech, Shanghai, China). One microgram of total RNA was used to synthesize cDNA using HiScript II Reverse Transcriptase (Vazyme, Nanjing, China) in a 20 μL reaction system. Five microliters of decuple-diluted cDNA were used to detect the transcript of *MoABC-R1* after treatment, three technical replicates, and three biological repeats for each treatment. 

### 2.12. Pathogenicity Assay

In vivo: two-week-old rice seedlings were selected and spray inoculated with spore suspension (15 × 10^4^ conidia/mL), and incubated under 25 °C and alternations of light and darkness for seven days after 24 h dark conditions. 

In vitro: the second leaves, obtained from two-week-old rice seedlings, were placed in the filters and moistened with 1 mg/L 6-BA, where 4 μL droplets of the spore suspension (1 × 10^5^ conidia/mL) with and without fungicide were inoculated on the wounds of leaves, cultured at 25 °C in dark conditions for 24 h, then the disease symptoms were recorded after exposure to continuous fluorescent light for four days.

## 3. Results

### 3.1. MoABC-R1 Is Required for Fungicide Tolerance in M. oryzae

**Figure 1:**
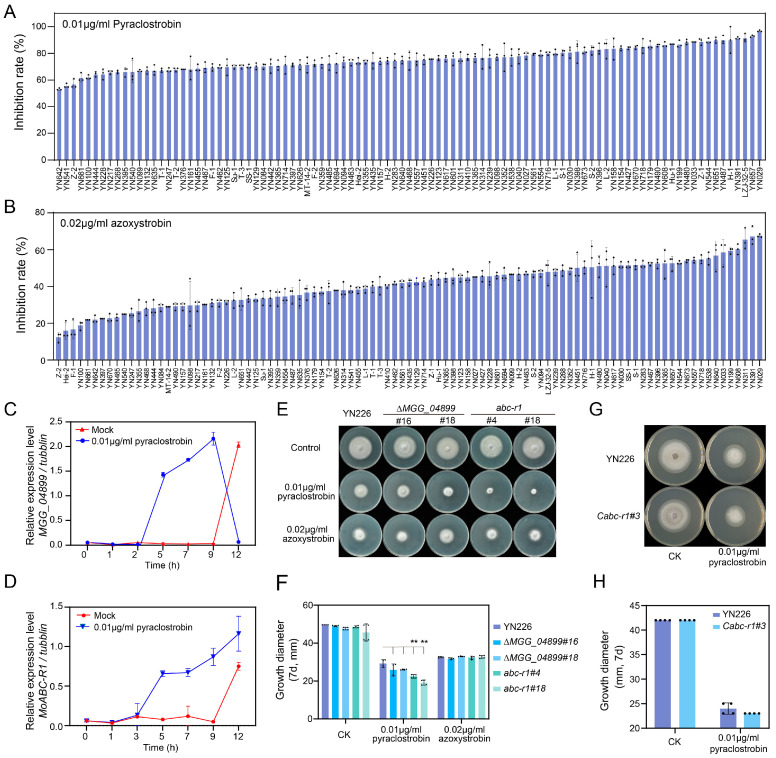
*MoABC-R1* is required for fungicide resistance in *M. oryzae*. (**A**) Inhibition rate for 90 rice blast strains in 0.01 μg/mL of pyraclostrobin condition. Error bars represent the standard deviations. Black dots represent biological replicates; (**B**) inhibition rate for 90 rice blast strains in 0.02 μg/mL of azoxystrobin condition. Error bars represent the standard deviations; (**C**,**D**) the transcription profiles of *MGG_04899* (**C**) and *MoABC-R1* (**D**) in the wild type were analyzed before and after treatment with 0.01 μg/mL of pyraclostrobin at various time points, measured via quantitative real-time polymerase chain reaction (qRT-PCR). Error bars represent the standard deviations; (**E**) comparison of the colony morphology of genes *MoABC-R1* and *MGG_04899* deletion mutants and wild-type strain in the presence of 0.01 μg/mL of pyraclostrobin and 0.02 μg/mL of azoxystrobin; (**F**,**H**) statistical analysis of the colony diameter of indicated strains and treatment. Error bars represent the standard deviations and asterisks represent significant differences (** represent *p* < 0.01). Black dots represent biological replicates; (**G**) comparison of the colony morphology of gene *MoABC-R1* complementary mutants and wild-type strain in the presence of 0.01 μg/mL of pyraclostrobin.

Fungicide treatment is widely employed as an effective method for controlling rice blast disease. Nevertheless, the widespread use of fungicides has resulted in the rapid emergence of fungicide-tolerant strains, leading to the outbreak of rice blast disease. To investigate the mechanism of fungicide tolerance in rice blast, we conducted sensitivity tests on 90 rice blast strains using the fungicides pyraclostrobin and azoxystrobin. Our results revealed that these 90 strains exhibited varying degrees of sensitivity to pyraclostrobin and azoxystrobin, as shown in Figure 1A,B. The inhibition rate of 0.01 μg/mL of pyraclostrobin ranged from 52% to 95% and the 0.02 μg/mL of azoxystrobin had an inhibition rate ranging from 11% to 66% (Figure 1A,B, Supplementary Tables S2 and S3). To further explore the reason for the diversity of fungicide tolerance observed in the 90 rice blast isolates, firstly, we sequenced the *CYTB* gene in the 90 strains and found that all of the 90 strains shared a highly identical sequence of the *CYTB* gene (Supplementary Data S1 and S2). Then, we analyzed the correlation between the fungicide tolerance phenotype and the sequences of all 50 ABC transporters [43] in the 90 strains, and we identified two ABC transporter genes (*p* < 0.01), *MoABC-R1* (*MGG_05044*) and *MGG_04899* (Supplementary Data S3 and S4), which were highly correlated with fungicide tolerance. 

To investigate the role of *MoABC-R1* and *MGG_04899* in fungicide tolerance, we analyzed the gene expression pattern following treatment with 0.01 μg/mL of pyraclostrobin. Our findings revealed that both *MoABC-R1* and *MGG_04899* were induced upon pyraclostrobin treatment, as depicted in Figure 1C,D. To further elucidate the function of these genes in fungicide tolerance, we generated gene deletion mutants of *MoABC-R1* and *MGG_04899* in the YN226 background. Subsequently, the deletion mutants were treated with 0.01 μg/mL of pyraclostrobin and 0.02 μg/mL of azoxystrobin on a complete medium. We observed that the *MoABC-R1* deletion mutants, *abc-r1*, exhibited extreme sensitivity to 0.01 μg/mL of pyraclostrobin, while the other mutants showed no significant differences compared to the wild-type strain YN226 (Figure 1E,F). Furthermore, we generated complementary transformants for *MoABC-R1* (designed as *Cabc-r1*) and confirmed that these transformants were able to restore pyraclostrobin tolerance (Figure 1G,H). The results suggest that *MoABC-R1* plays a critical role in pyraclostrobin tolerance in *M. oryzae*.

### 3.2. MoABC-R1 Encodes a Multidrug Tolerance Protein: ABCC Transporter

**Figure 2:**
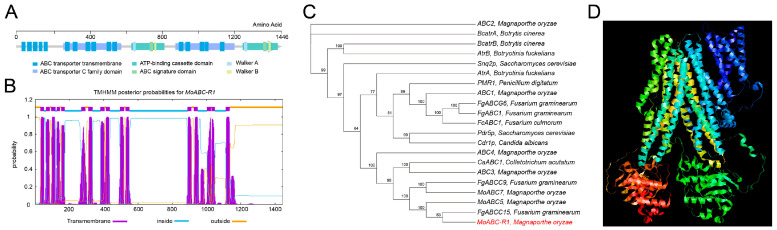
Gene and protein structure of *MoABC-R1*. (**A**) Gene architecture of *MoABC-R1*. The architecture is predicted on Interpro and NCBI CDD web-based programs; (**B**) putative transmembrane regions of MoABC-R1 analyzed on TMHMM web-based program; (**C**) molecular phylogeny of *MoABC-R1* transporter gene and 20 homologous genes from various organisms. The depicted phylogram was obtained via neighbor-joining using TBtools 1.068 software; (**D**) protein 3D structure of MoABC-R1.

The *MoABC-R1* gene has a total length of 5115 bp (3984 bp transcript) and consists of 11 exons and 10 introns. Analysis of the protein sequence of *MoABC-R1* revealed a significant similarity to the ABC transporter C family, with an MDR domain (Figure 2A). Additionally, we observed the presence of the ABC signature motif, ABC transporter transmembrane motifs, Walker A motifs, and Walker B motifs in the protein sequence of *MoABC-R1* (Figure 2A, Supplementary Data S5). Using TMHMM web-based programs, we identified 12 typical transmembrane regions in the MoABC-R1 protein sequence (Figure 2A,B). Phylogenetic tree analysis demonstrated a close genetic relationship between MoABC-R1 and ABCC subfamily transporters, including MoABC5, FgABCC15, FgABCC9, and MoABC7 (Figure 2C, Supplementary Data S5). Furthermore, the 3D structure of MoABC-R1 generated using PyMoL2.5 showed that it is a typical ABC transporter (Figure 2D) [32]. These findings strongly suggest that *MoABC-R1* encodes an ABCC-type transporter.

### 3.3. MoABC-R1 Is Not Essential for Inorganic Salt Transportation

**Figure 3:**
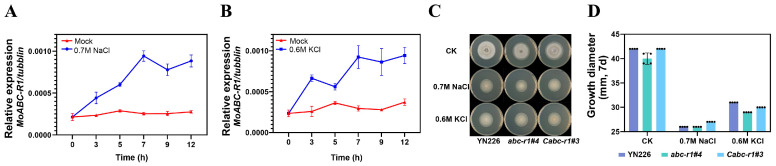
Expression of *MoABC-R1* and growth phenotype of *M. oryzae* under NaCl and KCl conditions. (**A**,**B**) Transcription profiles of *MoABC-R1* in *M. oryzae* before and after treatment with 0.7 M NaCl (**A**) and 0.6 M KCl (**B**) at different time points, measured via quantitative real-time polymerase chain reaction (qRT-PCR). Error bars represent the standard deviations; (**C**) comparing the colony morphology of *MoABC-R1* mutants and the wild-type strain under 0.7 M NaCl or 0.6 M KCl conditions; (**D**) statistical analysis of the colony diameter of indicated strains and treatment. Error bars represent the standard deviations. Black dots represent biological replicates.

Previous studies have demonstrated that ABC transporters can confer tolerance to metal ions, including K^+^ and Na^+^ [45,49]. In this study, we analyzed the *MoABC-R1* expression pattern under 0.7 M of NaCl or 0.6 M of KCl conditions. We observed a significant induction of *MoABC-R1* in the wild-type strain YN226 (Figure 3A,B). Additionally, we evaluated the mycelial growth of the *MoABC-R1* deletion mutants *abc-r1* in the presence of 0.7 M of NaCl or 0.6 M of KCl. Surprisingly, we did not observe any detectable differences in growth diameters between the *abc-r1* mutant and the wild-type strain YN226 (Figure 3C,D). These findings suggest that *MoABC-R1* plays a non-essential role in the transportation of the K^+^ and Na^+^ ions. 

### 3.4. MoABC-R1 Plays a Critical Role in the Pathogenesis of M. oryzae

**Figure 4:**
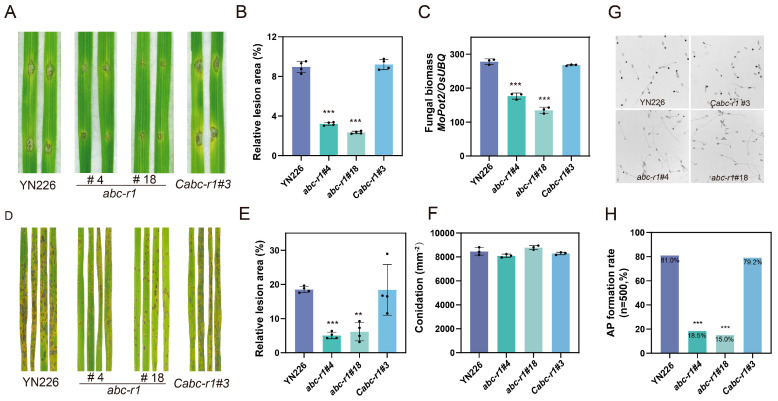
*MoABC-R1* plays an important role in the pathogenicity of *M. oryzae*. (**A**) The pathogenicity assay in vitro. Conidial suspension of strains (5 × 10^4^/mL) were drop inoculated on wounded leaves. Two-week-old seedling Nipponbare was selected. Diseased leaves were photographed after four days of inoculation; (**B**) relative lesion areas of diseased leaves in vitro. Error bars represent the standard deviations and asterisks represent significant differences (*** represent *p* < 0.001). Black dots represent biological replicates; (**C**) quantification of rice blast biomass in diseased leaves (in vitro). Error bars represent the standard deviations and asterisks represent significant differences (*** represent *p* < 0.001). Black dots represent biological replicates; (**D**) the pathogenicity assay in vivo. Conidial suspension of strains (15 × 10^4^/mL) was sprayed onto two-week-old seedling leaves (Nipponbare). Diseased leaves were photographed after seven days of inoculation; (**E**) relative lesion areas of diseased leaves in vivo. Error bars represent the standard deviations and asterisks represent significant differences (** represent *p* < 0.01; *** represent *p* < 0.001). Black dots represent biological replicates; (**F**) statistical analysis of conidia production in an area of 1 mm^2^. Conidia produced on oatmeal agar medium for ten days were collected in a consistent area. Error bars represent the standard deviations. Black dots represent biological replicates; (**G**) comparison of mutants and wild-type strains on appressurium formation. Appressurium formation was observed under a light microscope at 12 h at 25 °C after inoculation on the hydrophobic surface; (**H**) statistical analysis of appressurium formation (n = 500). Asterisks represent significant differences (*** represent *p* < 0.001). Black dots represent biological replicates.

Previous studies have revealed that ABC transporters possess the ability to efflux harmful components, including plant-derived toxins, thereby enhancing their pathogenicity [20,37,44]. Based on previous research, we hypothesized that *MoABC-R1* could increase the pathogenicity of *M. oryzae* by transporting plant-derived toxins out of the cell. To test this hypothesis, we performed spraying and wounding inoculation on Nipponbare (NPB) seedling leaves and assessed the pathogenicity of the *abc-r1* mutants. The spraying inoculation result clearly showed that the disease lesions caused by the *abc-r1* were smaller compared to the wild-type strain YN226 (Figure 4A–C). The wounding inoculation method also yielded consistent results (Figure 4D–F). These findings provide compelling evidences that *MoABC-R1* plays a significant role in the pathogenicity of *M. oryzae*. 

To elucidate the role of *MoABC-R1* in the invasion process of *M. oryzae,* it is essential to differentiate its effects on various biological phenotypes. Initially, we confirmed that *MoABC-R1* has no impact on vegetative growth as shown in Figure 1E. Then, we examined the production of conidia and the formation of appressoria in the *abc-r1* mutants. However, we observed no significant differences in conidia production between *abc-r1* and the wild-type strain YN226 (Figure 1F). Interestingly, the appressorium formation rate of *abc-r1* was significantly reduced compared to YN226 (Figure 4G,H). These findings suggest that *MoABC-R1* interferes with the pathogenicity of *M. oryzae* by disrupting the formation of appressoria.

### 3.5. The Significance of MoABC-R1 in Fungicide-Based Rice Blast Control

**Figure 5:**
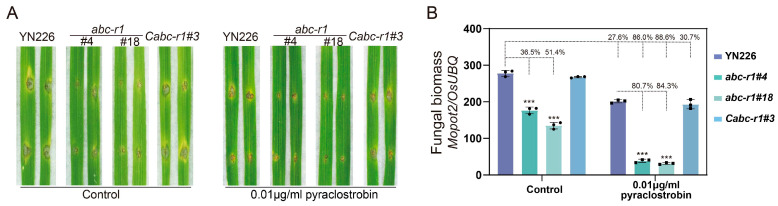
The difference in fungicide efficiency between the wild-type and *abc-r1* mutants of *M. oryzae*. (**A**) Comparison of the efficiency of fungicide in *abc-r1* and wild-type strain. Conidial suspension of strains (5 × 10^4^/mL) with or without 0.01 μg/mL of pyraclostrobin were drop-inoculated on wounded leaves. Two-week-old rice (NPB) seedling leaves were selected. Diseased leaves were photographed four days post-inoculation; (**B**) quantification of rice blast biomass in diseased leaves (in vitro). The percentage numbers represent the proportion of fungal biomass inhibited in mutants compared with the corresponding control. Error bars represent the standard deviations and asterisks represent significant difference (*** represent *p* < 0.001). Black dots represent biological replicates.

In this study, we have demonstrated the role of *MoABC-R1* in both pathogenicity and fungicide tolerance, as shown in Figure 1 and Figure 4. Based on these findings, we propose an approach for the prevention and management of rice blast: targeting and inhibiting the function of *MoABC-R1* in conjunction with fungicide application. To evaluate the feasibility of this approach, we compared the pathogenicity of the *abc-r1* mutant and the wild-type strain YN226 in the presence of 0.01 μg/mL of pyraclostrobin. We observed that the fungicide pyraclostrobin significantly reduced the colonization probability of all tested strains on NPB seedling leaves (Figure 5A). Importantly, the deletion mutant exhibited minimal disease lesions on NPB seedling leaves in the presence of pyraclostrobin. Interestingly, the fungal biomass of *abc-r1* decreased by more than 85%, while the wild-type YN226 only exhibited a reduction of 27.6% when compared to untreated YN226 (Figure 5B). In conclusion, the combination of *MoABC-R1* inactivation (*MoABC-R1* absence) and fungicide treatment resulted in a three-fold increase in control efficiency against rice blast (Figure 5B). These findings suggest that interfering with *MoABC-R1* can improve the effectiveness of fungicide-based rice blast management.

## 4. Discussion

In this study, we discovered that the gene *MoABC-R1* is involved in fungicide tolerance through an association analysis of fungicide tolerance and transporter gene sequences. The induction of *MoABC-R1* expression by pyraclostrobin and the observed growth phenotype on a pyraclostrobin-containing medium provided evidence for its role in fungicide tolerance. Sequence analysis revealed that *MoABC-R1* encodes an ABC transporter, along with a conserved MDR domain that classifies it as an ABCC (MDR) transporter. A related ABCC transporter, FgABCC15, shares 88% protein identity with MoABC-R1 (Figure 2C) and possesses an MRP domain that confers fungicide tolerance in the phytopathogenic fungus *Fusarium graminearum* [37]. Furthermore, several ABC transporters have been implicated in fungicide tolerance, such as FgABCC9 in *F. graminearum* [37], ABC3 in *M. oryzae* [47], and CaABC1 in *Colletotrichum acutatum* [42]. In this study, the ABCC transporter gene *MoABC-R1* was significantly induced in response to pyraclostrobin treatment, and it was associated with vegetative growth upon pyraclostrobin treatment (Figure 1). These results indicate that *MoABC-R1* functions as an exporter involved in detoxifying pyraclostrobin in *M. oryzae*. 

Previous studies have shown that ABC4 (orthologous gene of *MoABC-R1*) has no impact on mycelial growth but leads to a decreased germ tube growth rate in the presence of NaCl [45]. In this study, we observed the significant induction of *MoABC-R1* while treated with K^+^ and Na^+^. However, no differences were observed in mycelial growth in the presence of K^+^ and Na^+^ (Figure 3). Therefore, we proposed that *MoABC-R1* may be involved in Na^+^ and K^+^ transportation in other biological processes.

The ABC proteins containing the MDR domain have been identified as critical for the pathogenicity of phytopathogenic fungi. For instance, deletion mutants of FgABCC9 showed reduced visual disease symptoms in wheat [37]. *FgArb1* was found to regulate pathogenicity by participating in the phosphorylation of downstream gene *FgGpmk1* [20]. Similarly, in *M. oryzae*, ABC1 [44], ABC4 [45], and MoABC5 [43] have been identified as pathogenicity determinants. In our study, we observed that the deletion mutant of *MoABC-R1* exhibited less severe disease lesions (Figure 4A,D), and further investigation revealed that *MoABC-R1* is essential for appressorium formation (Figure 4G). Therefore, we conclude that *MoABC-R1* plays a similar role in the pathogenic process to ABC4 [45] of appressorium-mediated penetration, but establishes colonization within host cells upon successful infection.

QoI fungicides, known for their broad efficacy, have been effective in preventing fungal diseases in various crops. Previous studies on the mechanism of QoI fungicide tolerance in plant pathogens have identified three main modes of tolerance [9]: (i) mutation in the target gene *CYTB*, (ii) the substitution of the target gene with an equivalent gene, and (iii) the overexpression of the target gene. Recently, increased research has revealed the involvement of ABC transporters in mediating fungicide tolerance in phytopathogenic fungi [1,3,41]. Based on the function of *MoABC-R1* in fungicide tolerance and pathogenicity in this study, we propose an approach for the prevention and management of rice blast: targeting and inhibiting the function of *MoABC-R1* in conjunction with fungicide application. To evaluate the feasibility of this approach, the disease intensity of *abc-r1* and wild-type strain YN226 is detected with or without 0.01 μg/mL of pyraclostrobin treatment. We found that the lesion expansion of *abc-r1* is inhibited in the wound spot. Remarkably, the fungal biomass of *abc-r1* decreased by approximately 86%, while YN226 showed a 27.6% reduction compared to YN226 without fungicide treatment (Figure 5). These findings provide strong support for the approach we have proposed.

In summary, the study of *MoABC-R1* and its role in fungicide tolerance in *M. oryzae* underscores the complexity of these tolerance mechanisms and the need for ongoing research in this area. This work opens up new avenues for the development of more effective strategies for managing fungicide tolerance in crop pathogens.

## Data Availability

The data presented in this study are available on request from the corresponding authors.

## Supplementary Materials

The following supporting information can be downloaded at https://www.mdpi.com/article/10.3390/jof9090917/s1, Supplementary Data S1: nucleotide sequence of gene *CYTB*; Supplementary Data S2: amino acid sequence of gene *CYTB*; Supplementary Data S3: protein sequence alignment of gene *MoABC-R1* in 90 rice blast strains; Supplementary Data S4: protein sequence alignment of gene *MGG_04899* in 90 rice blast strains; Supplementary Data S5: sequence alignment for the homology protein of MoABC-R1; Supplementary Table S1: the inhibition rate of 90 rice blasts in the presence of 0.01 μg/mL of pyraclostrobin and 0.02 μg/mL of azoxystrobin; Supplementary Table S2: sequences of primers used in this study; Supplementary Table S3: significant analysis for azoxystrobin and pyraclostrobin treatment.
