# Supplementary material for: ABCC Transporter Gene MoABC-R1 Is Associated with Pyraclostrobin Tolerance in Magnaporthe oryzae
Source: J Fungi (Basel). 2023 Sep 11;9(9):917. doi: 10.3390/jof9090917 (PMC10532721; doi:10.3390/jof9090917)
Supplement: Supplementary file 1 [file jof-09-00917-s001.zip › Supplementary Data S1 Nucleotide sequence of gene CYTB.pdf]

>RB22
ATAACAATTCTAAAGAACATAAAGAGTTATTAGATAAAAATAATTCACCTATACAGTTAATAAAT
CAACTTAAAGGGTATTTTTTCATAAATCCTTTATTAGCTTTAAGTTTAGCTATTACTATTTTCTCTTT
TGCAGGTATTCCTCCTCTTGTAGGGTTCTTTGCTAAACAGATGGTATTAAGCGCGGCTATTGATC
AAGGTTATATCTTTTTATCTTTAGTTGCAATATTAACTAGTGTTATAGGAGGGGTTTATTATTTAAA
TATAATTAAAGAAATGTTCTTTTATTCACCTGACTATAAATTAAACGAAGAAATTAAAAATAATAC
TATTAATGGTCAAATTATTAATAGAAACAATAAAATATTAAATGTTGAATTTAATTATACAAATGT
AGTTATGTCTAGTTCTGTGGCAATAACTATTTCTACTATTACATTAGTAGTTTTATTATTCATGTTT
ATGAATAAAGAATGATTAAGTCTGGGTACTATATTGGTACAATCTTTATTTAGCTATTAATGAGTA
GTATGACATTATTTATAGGGTTTGTATCTGTTATAGCTATTTTATTTTTAGCCATTAATTTTATATTT
GCTCCTCATAATCCTTATCAAGAAAAATATAGTATTTTCGAGTGTGGTTTCCATAGTTTTTTAGGG
CAAAATAGAACACAATTCGGTATAAAATTCTTTATTTTTGCTTTAGTTTATTTACTTTTAGATTTAG
AAATATTATTAACTTTCCCTTTCGCTCTTAGTGAGTATGTTAATGGTATTTATGGTCTTTTAGTTAC
TTTAATTTTTATAGCTATAATAACTATAGGATTTATATTTGAATTAGGTAAAAGCGCTCTTAAAATA
GACAGCAGACAAAAATTATATATACCTAAATTGAACGTTAATTACCATACAGAGTATGTTGGAAT
AGGTAAGGTTTCTAAGTAAAGTTATAGAGGCAGAAAACCAAAAAACCTACCAAAGGGTAGCTA
ATGGGAAGCTATTAAAAATAAAAGATGATAACCTATATATAGTATAGTTACTATATATTTACTATA
ACTAGGATTATTATATATATATATATTATCTTATTGTATATTAAGATTATTATTATTATAAGGTATA
ATTAATATAGTATCTTATTGTATAAGAATATAATATATTAACCTATAATTAATTTTATTTTTTAATTT
TTAATTATAATTTTTTTTTATATCTAGATGCTTACACATCTACAGATGTAGAAGAGAACAAAATAT
TGTTGTTATAGTAGTGGATGATATAGAAAAATATTTTTATATTATTTATTTTTGTTAGGTAGCTTTT
GAAGTGTTTGATAGAGAGGATATATGGACGGTAGGAGGGTATTCATTTTAATGAACAGTGGATA
GTTTAAATTAACCTAGTTATAGTTTATGAATTTAAATTATAATTATATTAATGTAGGTTATGATAGA
ATTATGTATTGATCCTTAAGAGTTAAGAGAGATACGCCCACGTATAATACATATTGGTTTAGGGT
TGAGTATATATACTTAAGAGTTAAGAATATATATATACAATATATAATAAATATAGTAATATATTA
ATGGTATGGACTTAACCAGGTTTATATATTATCATTTGATAAACATTAATTATAATTTTTCTATTTA
TTATTATTATTATACTGTGAGATTAATAATTATTAAAATATAATATTACATAGTACGTGATATTTGG
GGATTTTATCTTATTAATTGGCAATTAATGATTCTAATCAAATTTTATTCTCTTTAGTTTAATGGTA
GAACAATGATCTTCTAATTCATTGGTTTTAGTTCGATTCTAAAAAGAGATGAGTAAATAATTTTCT
AGATCAGAAATACTACTTTTAACTACAAAAAGCTTACGCTTTTTAACATTTTTTTGATAAATAACA
ACTATTGTTAATATTTGGCTGTCTATTGGTTTAACTAACAATTACAAAATTTTTTCAATTTTTATAAT
ATAAATTTAAATGAGAATATTAAAAAGTCATTCATTATTAAAATTAGTGAATTCTTACCTTATCGA
TGCGTCACAACCAAGTAACATTAGTTACTTGTGAAATTTTGGTTCATTATTAGCTGTTTGTTTAAT
AGTACAAATTATTACCGGTATTACATTAGCTATGCATTATAGTCCTAGTGTAATGGAAGCTTTTAA
CTCAATAGAGCATATAATGAGAGATGTTAATAACGGGTGATTAGTTCGTTATCTACATAGTAATA
CAGCTTCTGCTTTCTTTTTCTTAGTGTATTTACACATAGGAAGAGGTATATATTACGGATCATATA
GAGCTCCTCGTACTTTAGTTTGAGCTATTGGTACTGTTATATTAATATTAATGATGGCTATCGGTT
TCCTAGGTTATGTTTTACCTTATGGACAGATGTCATTATGAGGTGCTACAGTTATTACTAATCTTA
TTAGTGCTATACCTTGAATAGGGCAAGATATTGTTGAATTCATTTGAGGTGGTTTTTCTGTTAATA
ATGCCACTTTAAACAGATTTTTTGCATTACATTTTGTATTGCCTTTTGTATTAGCTGCTTTAGTTTTA
ATGCACTTAATTGCACTTCATGATACTGCTGGTTCAAGCAATCCTCTTGGTGTTTCAGGTAATTAC
GATAGAATTACATTTGCTCCATATTTTTTATTTAAAGATTTAATTACTATTTTTATATTTATTTTTGTA
TTAAGTGCTTTTGTATTCTTTATGCCTAATGTTTTAGGGGATAGTGATAATTATATTATGGCTAATC
CTATGCAAACTCCTGCTGCTATTGTACCTGAATGATACTTATTACCTTTCTATGCTATTTTAAGATC

TATACCTAATAAATTATTAGGTGTTATAGCGATGTTTAGTGCTATTTTAGCTATTATGTTATTACCT
GTTACAGATTTAGGTAGATCTAGAGGTTTACAATTTAGACCATTTAGTAAAATAGCTTTCTGAGTT
TTTGTTGCTAATTTCTTAGTTTTAATGCAATTAGGTGCTAAACACGTTGAAGATCCATTTATATTAT
TAGGTCAATTAAGTACTGTATTATACTTTAGTTATTTTGTTGCTATATTACCTTTAGCTAGTTACTT
AGATAATAGTTTAACTGATTTATCTAATAAATCTGAATTATTTTTAAATAAAACTAACTAAATATAT
TAAGATTATTATTTAATATATTTTCTATTTAAGATACTATTAATTTAGTATTTTGGGTTTTCAGTTTA
TAATTTATATTATATTATGCATTACCCTCCACCTTGCTTTGTAGTAAGCTAATCTGTTATTTCCTTTA
GTTTAATGGTAGAACAATGATCTTCTAATTCATTGGTTTTAGTTCGAATCTAAAAAGGAAATAAG
AAATATATTCTTATTATTACTTATATAATAATTATTTCTTAAAAATATACATTTTGCATTATAGCCGT
TTAGCTGTATTAAAATGTAAAATGATATAAAATAGAATAAATATTTAAATTATTCCTATGTTATATT
ATCCTATATTGCAACCATTATCAGAAGTTGTATTAATACTTGTACCTGCCTTATTAGCTGTAGCTT
ATGTTACAGTTGCTGAAAGAAAAACTATGGCTAGTATGCAAAGAAGATTAGGTCCTAATGCTGT
AGGTTACTATGGACTATTGCAAGCATTTGCTGATGCCTTAAAACTTTTATTAAAAGAATATGTAG
CTCCTACACAATCTAATATTGTTCTTTTCTTTTTAGGTCCTGTAATAACTTTAATTTTTGCATTATTA
GGTTACGCTGTTATACCCTATGGTCCTGGTTCAGGGATAAGCGACATGAATTTAGGTATATTTTA
CATGTTAGCTGTGTCATCTTTAGCTACATACGGTATTCTATTAGCTGGTTGAAGTGCGAATAGTA
AATACGCTTTTCTAGGTTCTCTTAGAAGTACAGCTCAATTAATTAGTTATGAATTAATATTAAGTT
CAGCTATATTAATAGTAATTATGATAACAGGAAATTTAAATTTAACTGTTTGTACTGAATCTCAAA
GAGCTATTTGATTTATACTACCTTTATTTCCTGTGTTTATAATATTTTTCATAGGATCTATAGCTGA
GACAAATAGAGCTCCTTTTGATTTAGCCGAGGCTAACCTGCTAATCTGGTTTGGTCTGGTTATAT
GTCACAAATTGCTAGGAAACCTTTTTATTTTAAAAACAAAAGACAATTAGCAGGAAACTTAATTT
AACCTAATTAAAAATATTAGATAATTAAACTCTTCATAGACTAAACGTGACAATTTAATATATATA
TATATTTATTTATATATATGATTAAATAAGATATAGTCAATCATCGGTGTGAATCGACTTAAAAAA
AAAAGCACATGGGTTAAACCCATCTCCCCTTATTAGGGGAATCAGAACTTGTTAGTGGGTTCAT
GACAGAGCATGCTGCCGTAGTTTTCGTATTCTTCTTTTTAGCTGAGTACGGTAGTATTGTACTAAT
GTGTATTTTAACTAGTATATTATTTATTGGTGGTTACTTATTATTTGAAATATCCTATGTTTTTACTG
TGGTAAATTATATTTTCTTTGAATTATTCTTTATAGACTGAGTAACATTTGTAGAGGTACAATCTTT
ATACACTGATTTTTTAAATAATTCTATCATTGAAGGATTATTATATGGGTTTAATCTAGGATTAAA
AAGTTCTTTAATGATATTCACATTTATTTGAGCTAGAGCATCCTTCCCTAGAATACGATTTGATCA
ACTAATGGGCTTCTGTTGAACAGTTTTATTACCTATTAATTTTGCAATTATTATATTAGTACCTTGT
GTTTTATATAGTTTTAACTTATTACCTGTAAATATACCATTGTTCTAGCTCACACACCCGCCCGCC
CTACTGCCACAAGGCTACAGTACATATGAGGAGGGGAACTAAAGATCTAGAACTATCCTAGTTA
ATAATTACACTTAATAGTATACTAAATAGACCATCTATCATACTCGAGAATAGTGATAGTGTAAT
TATACACTATTTTAACTGTATTCTACACTATTAGCATATTATTATCTTTATATGATAATAATTTTATA
ACTTAATTTATTAGGTTACATTAACAAAAAATTCGTTAACTTTTTTCACCACTTTTTAATACAAAAT
ACGAATTTTA
>F-1
ATAACAATTCTAAAGAACATAAAGAGTTATTAGATAAAAATAATTCACCTATACAGTTAATAAAT
CAACTTAAAGGGTATTTTTTCATAAATCCTTTATTAGCTTTAAGTTTAGCTATTACTATTTTCTCTTT
TGCAGGTATTCCTCCTCTTGTAGGGTTCTTTGCTAAACAGATGGTATTAAGCGCGGCTATTGATC
AAGGTTATATCTTTTTATCTTTAGTTGCAATATTAACTAGTGTTATAGGAGGGGTTTATTATTTAAA
TATAATTAAAGAAATGTTCTTTTATTCACCTGACTATAAATTAAACGAAGAAATTAAAAATAATAC
TATTAATGGTCAAATTATTAATAGAAACAATAAAATATTAAATGTTGAATTTAATTATACAAATGT
AGTTATGTCTAGTTCTGTGGCAATAACTATTTCTACTATTACATTAGTAGTTTTATTATTCATGTTT

ATGAATAAAGAATGATTAAGTCTGGGTACTATATTGGTACAATCTTTATTTAGCTATTAATGAGTA
GTATGACATTATTTATAGGGTTTGTATCTGTTATAGCTATTTTATTTTTAGCCATTAATTTTATATTT
GCTCCTCATAATCCTTATCAAGAAAAATATAGTATTTTCGAGTGTGGTTTCCATAGTTTTTTAGGG
CAAAATAGAACACAATTCGGTATAAAATTCTTTATTTTTGCTTTAGTTTATTTACTTTTAGATTTAG
AAATATTATTAACTTTCCCTTTCGCTCTTAGTGAGTATGTTAATGGTATTTATGGTCTTTTAGTTAC
TTTAATTTTTATAGCTATAATAACTATAGGATTTATATTTGAATTAGGTAAAAGCGCTCTTAAAATA
GACAGCAGACAAAAATTATATATACCTAAATTGAACGTTAATTACCATACAGAGTATGTTGGAAT
AGGTAAGGTTTCTAAGTAAAGTTATAGAGGCAGAAAACCAAAAAACCTACCAAAGGGTAGCTA
ATGGGAAGCTATTAAAAATAAAAGATGATAACCTATATATAGTATAGTTACTATATATTTACTATA
ACTAGGATTATTATATATATATATATTATCTTATTGTATATTAAGATTATTATTATTATAAGGTATA
ATTAATATAGTATCTTATTGTATAAGAATATAATATATTAACCTATAATTAATTTTATTTTTTAATTT
TTAATTATAATTTTTTTTTATATCTAGATGCTTACACATCTACAGATGTAGAAGAGAACAAAATAT
TGTTGTTATAGTAGTGGATGATATAGAAAAATATTTTTATATTATTTATTTTTGTTAGGTAGCTTTT
GAAGTGTTTGATAGAGAGGATATATGGACGGTAGGAGGGTATTCATTTTAATGAACAGTGGATA
GTTTAAATTAACCTAGTTATAGTTTATGAATTTAAATTATAATTATATTAATGTAGGTTATGATAGA
ATTATGTATTGATCCTTAAGAGTTAAGAGAGATACGCCCACGTATAATACATATTGGTTTAGGGT
TGAGTATATATACTTAAGAGTTAAGAATATATATATACAATATATAATAAATATAGTAATATATTA
ATGGTATGGACTTAACCAGGTTTATATATTATCATTTGATAAACATTAATTATAATTTTTCTATTTA
TTATTATTATTATACTGTGAGATTAATAATTATTAAAATATAATATTACATAGTACGTGATATTTGG
GGATTTTATCTTATTAATTGGCAATTAATGATTCTAATCAAATTTTATTCTCTTTAGTTTAATGGTA
GAACAATGATCTTCTAATTCATTGGTTTTAGTTCGATTCTAAAAAGAGATGAGTAAATAATTTTCT
AGATCAGAAATACTACTTTTAACTACAAAAAGCTTACGCTTTTTAACATTTTTTTGATAAATAACA
ACTATTGTTAATATTTGGCTGTCTATTGGTTTAACTAACAATTACAAAATTTTTTCAATTTTTATAAT
ATAAATTTAAATGAGAATATTAAAAAGTCATTCATTATTAAAATTAGTGAATTCTTACCTTATCGA
TGCGTCACAACCAAGTAACATTAGTTACTTGTGAAATTTTGGTTCATTATTAGCTGTTTGTTTAAT
AGTACAAATTATTACCGGTATTACATTAGCTATGCATTATAGTCCTAGTGTAATGGAAGCTTTTAA
CTCAATAGAGCATATAATGAGAGATGTTAATAACGGGTGATTAGTTCGTTATCTACATAGTAATA
CAGCTTCTGCTTTCTTTTTCTTAGTGTATTTACACATAGGAAGAGGTATATATTACGGATCATATA
GAGCTCCTCGTACTTTAGTTTGAGCTATTGGTACTGTTATATTAATATTAATGATGGCTATCGGTT
TCCTAGGTTATGTTTTACCTTATGGACAGATGTCATTATGAGGTGCTACAGTTATTACTAATCTTA
TTAGTGCTATACCTTGAATAGGGCAAGATATTGTTGAATTCATTTGAGGTGGTTTTTCTGTTAATA
ATGCCACTTTAAACAGATTTTTTGCATTACATTTTGTATTGCCTTTTGTATTAGCTGCTTTAGTTTTA
ATGCACTTAATTGCACTTCATGATACTGCTGGTTCAAGCAATCCTCTTGGTGTTTCAGGTAATTAC
GATAGAATTACATTTGCTCCATATTTTTTATTTAAAGATTTAATTACTATTTTTATATTTATTTTTGTA
TTAAGTGCTTTTGTATTCTTTATGCCTAATGTTTTAGGGGATAGTGATAATTATATTATGGCTAATC
CTATGCAAACTCCTGCTGCTATTGTACCTGAATGATACTTATTACCTTTCTATGCTATTTTAAGATC
TATACCTAATAAATTATTAGGTGTTATAGCGATGTTTAGTGCTATTTTAGCTATTATGTTATTACCT
GTTACAGATTTAGGTAGATCTAGAGGTTTACAATTTAGACCATTTAGTAAAATAGCTTTCTGAGTT
TTTGTTGCTAATTTCTTAGTTTTAATGCAATTAGGTGCTAAACACGTTGAAGATCCATTTATATTAT
TAGGTCAATTAAGTACTGTATTATACTTTAGTTATTTTGTTGCTATATTACCTTTAGCTAGTTACTT
AGATAATAGTTTAACTGATTTATCTAATAAATCTGAATTATTTTTAAATAAAACTAACTAAATATAT
TAAGATTATTATTTAATATATTTTCTATTTAAGATACTATTAATTTAGTATTTTGGGTTTTCAGTTTA
TAATTTATATTATATTATGCATTACCCTCCACCTTGCTTTGTAGTAAGCTAATCTGTTATTTCCTTTA
GTTTAATGGTAGAACAATGATCTTCTAATTCATTGGTTTTAGTTCGAATCTAAAAAGGAAATAAG

AAATATATTCTTATTATTACTTATATAATAATTATTTCTTAAAAATATACATTTTGCATTATAGCCGT
TTAGCTGTATTAAAATGTAAAATGATATAAAATAGAATAAATATTTAAATTATTCCTATGTTATATT
ATCCTATATTGCAACCATTATCAGAAGTTGTATTAATACTTGTACCTGCCTTATTAGCTGTAGCTT
ATGTTACAGTTGCTGAAAGAAAAACTATGGCTAGTATGCAAAGAAGATTAGGTCCTAATGCTGT
AGGTTACTATGGACTATTGCAAGCATTTGCTGATGCCTTAAAACTTTTATTAAAAGAATATGTAG
CTCCTACACAATCTAATATTGTTCTTTTCTTTTTAGGTCCTGTAATAACTTTAATTTTTGCATTATTA
GGTTACGCTGTTATACCCTATGGTCCTGGTTCAGGGATAAGCGACATGAATTTAGGTATATTTTA
CATGTTAGCTGTGTCATCTTTAGCTACATACGGTATTCTATTAGCTGGTTGAAGTGCGAATAGTA
AATACGCTTTTCTAGGTTCTCTTAGAAGTACAGCTCAATTAATTAGTTATGAATTAATATTAAGTT
CAGCTATATTAATAGTAATTATGATAACAGGAAATTTAAATTTAACTGTTTGTACTGAATCTCAAA
GAGCTATTTGATTTATACTACCTTTATTTCCTGTGTTTATAATATTTTTCATAGGATCTATAGCTGA
GACAAATAGAGCTCCTTTTGATTTAGCCGAGGCTAACCTGCTAATCTGGTTTGGTCTGGTTATAT
GTCACAAATTGCTAGGAAACCTTTTTATTTTAAAAACAAAAGACAATTAGCAGGAAACTTAATTT
AACCTAATTAAAAATATTAGATAATTAAACTCTTCATAGACTAAACGTGACAATTTAATATATATA
TATATTTATTTATATATATGATTAAATAAGATATAGTCAATCATCGGTGTGAATCGACTTAAAAAA
AAAAGCACATGGGTTAAACCCATCTCCCCTTATTAGGGGAATCAGAACTTGTTAGTGGGTTCAT
GACAGAGCATGCTGCCGTAGTTTTCGTATTCTTCTTTTTAGCTGAGTACGGTAGTATTGTACTAAT
GTGTATTTTAACTAGTATATTATTTATTGGTGGTTACTTATTATTTGAAATATCCTATGTTTTTACTG
TGGTAAATTATATTTTCTTTGAATTATTCTTTATAGACTGAGTAACATTTGTAGAGGTACAATCTTT
ATACACTGATTTTTTAAATAATTCTATCATTGAAGGATTATTATATGGGTTTAATCTAGGATTAAA
AAGTTCTTTAATGATATTCACATTTATTTGAGCTAGAGCATCCTTCCCTAGAATACGATTTGATCA
ACTAATGGGCTTCTGTTGAACAGTTTTATTACCTATTAATTTTGCAATTATTATATTAGTACCTTGT
GTTTTATATAGTTTTAACTTATTACCTGTAAATATACCATTGTTCTAGCTCACACACCCGCCCGCC
CTACTGCCACAAGGCTACAGTACATATGAGGAGGGGAACTAAAGATCTAGAACTATCCTAGTTA
ATAATTACACTTAATAGTATACTAAATAGACCATCTATCATACTCGAGAATAGTGATAGTGTAAT
TATACACTATTTTAACTGTATTCTACACTATTAGCATATTATTATCTTTATATGATAATAATTTTATA
ACTTAATTTATTAGGTTACATTAACAAAAAAATTCGTTAACTTTTTTCACCACTTTTTAATACAAAAT
ACGAATTTTA
>F-2
ATAACAATTCTAAAGAACATAAAGAGTTATTAGATAAAAATAATTCACCTATACAGTTAATAAAT
CAACTTAAAGGGTATTTTTTCATAAATCCTTTATTAGCTTTAAGTTTAGCTATTACTATTTTCTCTTT
TGCAGGTATTCCTCCTCTTGTAGGGTTCTTTGCTAAACAGATGGTATTAAGCGCGGCTATTGATC
AAGGTTATATCTTTTTATCTTTAGTTGCAATATTAACTAGTGTTATAGGAGGGGTTTATTATTTAAA
TATAATTAAAGAAATGTTCTTTTATTCACCTGACTATAAATTAAACGAAGAAATTAAAAATAATAC
TATTAATGGTCAAATTATTAATAGAAACAATAAAATATTAAATGTTGAATTTAATTATACAAATGT
AGTTATGTCTAGTTCTGTGGCAATAACTATTTCTACTATTACATTAGTAGTTTTATTATTCATGTTT
ATGAATAAAGAATGATTAAGTCTGGGTACTATATTGGTACAATCTTTATTTAGCTATTAATGAGTA
GTATGACATTATTTATAGGGTTTGTATCTGTTATAGCTATTTTATTTTTAGCCATTAATTTTATATTT
GCTCCTCATAATCCTTATCAAGAAAAATATAGTATTTTCGAGTGTGGTTTCCATAGTTTTTTAGGG
CAAAATAGAACACAATTCGGTATAAAATTCTTTATTTTTGCTTTAGTTTATTTACTTTTAGATTTAG
AAATATTATTAACTTTCCCTTTCGCTCTTAGTGAGTATGTTAATGGTATTTATGGTCTTTTAGTTAC
TTTAATTTTTATAGCTATAATAACTATAGGATTTATATTTGAATTAGGTAAAAGCGCTCTTAAAATA
GACAGCAGACAAAAATTATATATACCTAAATTGAACGTTAATTACCATACAGAGTATGTTGGAAT
AGGTAAGGTTTCTAAGTAAAGTTATAGAGGCAGAAAACCAAAAAACCTACCAAAGGGTAGCTA

ATGGGAAGCTATTAAAAATAAAAGATGATAACCTATATATAGTATAGTTACTATATATTTACTATA
ACTAGGATTATTATATATATATATATTATCTTATTGTATATTAAGATTATTATTATTATAAGGTATA
ATTAATATAGTATCTTATTGTATAAGAATATAATATATTAACCTATAATTAATTTTATTTTTTAATTT
TTAATTATAATTTTTTTTTATATCTAGATGCTTACACATCTACAGATGTAGAAGAGAACAAAATAT
TGTTGTTATAGTAGTGGATGATATAGAAAAATATTTTTATATTATTTATTTTTGTTAGGTAGCTTTT
GAAGTGTTTGATAGAGAGGATATATGGACGGTAGGAGGGTATTCATTTTAATGAACAGTGGATA
GTTTAAATTAACCTAGTTATAGTTTATGAATTTAAATTATAATTATATTAATGTAGGTTATGATAGA
ATTATGTATTGATCCTTAAGAGTTAAGAGAGATACGCCCACGTATAATACATATTGGTTTAGGGT
TGAGTATATATACTTAAGAGTTAAGAATATATATATACAATATATAATAAATATAGTAATATATTA
ATGGTATGGACTTAACCAGGTTTATATATTATCATTTGATAAACATTAATTATAATTTTTCTATTTA
TTATTATTATTATACTGTGAGATTAATAATTATTAAAATATAATATTACATAGTACGTGATATTTGG
GGATTTTATCTTATTAATTGGCAATTAATGATTCTAATCAAATTTTATTCTCTTTAGTTTAATGGTA
GAACAATGATCTTCTAATTCATTGGTTTTAGTTCGATTCTAAAAAGAGATGAGTAAATAATTTTCT
AGATCAGAAATACTACTTTTAACTACAAAAAGCTTACGCTTTTTAACATTTTTTTGATAAATAACA
ACTATTGTTAATATTTGGCTGTCTATTGGTTTAACTAACAATTACAAAATTTTTTCAATTTTTATAAT
ATAAATTTAAATGAGAATATTAAAAAGTCATTCATTATTAAAATTAGTGAATTCTTACCTTATCGA
TGCGTCACAACCAAGTAACATTAGTTACTTGTGAAATTTTGGTTCATTATTAGCTGTTTGTTTAAT
AGTACAAATTATTACCGGTATTACATTAGCTATGCATTATAGTCCTAGTGTAATGGAAGCTTTTAA
CTCAATAGAGCATATAATGAGAGATGTTAATAACGGGTGATTAGTTCGTTATCTACATAGTAATA
CAGCTTCTGCTTTCTTTTTCTTAGTGTATTTACACATAGGAAGAGGTATATATTACGGATCATATA
GAGCTCCTCGTACTTTAGTTTGAGCTATTGGTACTGTTATATTAATATTAATGATGGCTATCGGTT
TCCTAGGTTATGTTTTACCTTATGGACAGATGTCATTATGAGGTGCTACAGTTATTACTAATCTTA
TTAGTGCTATACCTTGAATAGGGCAAGATATTGTTGAATTCATTTGAGGTGGTTTTTCTGTTAATA
ATGCCACTTTAAACAGATTTTTTGCATTACATTTTGTATTGCCTTTTGTATTAGCTGCTTTAGTTTTA
ATGCACTTAATTGCACTTCATGATACTGCTGGTTCAAGCAATCCTCTTGGTGTTTCAGGTAATTAC
GATAGAATTACATTTGCTCCATATTTTTTATTTAAAGATTTAATTACTATTTTTATATTTATTTTTGTA
TTAAGTGCTTTTGTATTCTTTATGCCTAATGTTTTAGGGGATAGTGATAATTATATTATGGCTAATC
CTATGCAAACTCCTGCTGCTATTGTACCTGAATGATACTTATTACCTTTCTATGCTATTTTAAGATC
TATACCTAATAAATTATTAGGTGTTATAGCGATGTTTAGTGCTATTTTAGCTATTATGTTATTACCT
GTTACAGATTTAGGTAGATCTAGAGGTTTACAATTTAGACCATTTAGTAAAATAGCTTTCTGAGTT
TTTGTTGCTAATTTCTTAGTTTTAATGCAATTAGGTGCTAAACACGTTGAAGATCCATTTATATTAT
TAGGTCAATTAAGTACTGTATTATACTTTAGTTATTTTGTTGCTATATTACCTTTAGCTAGTTACTT
AGATAATAGTTTAACTGATTTATCTAATAAATCTGAATTATTTTTAAATAAAACTAACTAAATATAT
TAAGATTATTATTTAATATATTTTCTATTTAAGATACTATTAATTTAGTATTTTGGGTTTTCAGTTTA
TAATTTATATTATATTATGCATTACCCTCCACCTTGCTTTGTAGTAAGCTAATCTGTTATTTCCTTTA
GTTTAATGGTAGAACAATGATCTTCTAATTCATTGGTTTTAGTTCGAATCTAAAAAGGAAATAAG
AAATATATTCTTATTATTACTTATATAATAATTATTTCTTAAAAATATACATTTTGCATTATAGCCGT
TTAGCTGTATTAAAATGTAAAATGATATAAAATAGAATAAATATTTAAATTATTCCTATGTTATATT
ATCCTATATTGCAACCATTATCAGAAGTTGTATTAATACTTGTACCTGCCTTATTAGCTGTAGCTT
ATGTTACAGTTGCTGAAAGAAAAACTATGGCTAGTATGCAAAGAAGATTAGGTCCTAATGCTGT
AGGTTACTATGGACTATTGCAAGCATTTGCTGATGCCTTAAAACTTTTATTAAAAGAATATGTAG
CTCCTACACAATCTAATATTGTTCTTTTCTTTTTAGGTCCTGTAATAACTTTAATTTTTGCATTATTA
GGTTACGCTGTTATACCCTATGGTCCTGGTTCAGGGATAAGCGACATGAATTTAGGTATATTTTA
CATGTTAGCTGTGTCATCTTTAGCTACATACGGTATTCTATTAGCTGGTTGAAGTGCGAATAGTA

AATACGCTTTTCTAGGTTCTCTTAGAAGTACAGCTCAATTAATTAGTTATGAATTAATATTAAGTT
CAGCTATATTAATAGTAATTATGATAACAGGAAATTTAAATTTAACTGTTTGTACTGAATCTCAAA
GAGCTATTTGATTTATACTACCTTTATTTCCTGTGTTTATAATATTTTTCATAGGATCTATAGCTGA
GACAAATAGAGCTCCTTTTGATTTAGCCGAGGCTAACCTGCTAATCTGGTTTGGTCTGGTTATAT
GTCACAAATTGCTAGGAAACCTTTTTATTTTAAAAACAAAAGACAATTAGCAGGAAACTTAATTT
AACCTAATTAAAAATATTAGATAATTAAACTCTTCATAGACTAAACGTGACAATTTAATATATATA
TATATTTATTTATATATATGATTAAATAAGATATAGTCAATCATCGGTGTGAATCGACTTAAAAAA
AAAAGCACATGGGTTAAACCCATCTCCCCTTATTAGGGGAATCAGAACTTGTTAGTGGGTTCAT
GACAGAGCATGCTGCCGTAGTTTTCGTATTCTTCTTTTTAGCTGAGTACGGTAGTATTGTACTAAT
GTGTATTTTAACTAGTATATTATTTATTGGTGGTTACTTATTATTTGAAATATCCTATGTTTTTACTG
TGGTAAATTATATTTTCTTTGAATTATTCTTTATAGACTGAGTAACATTTGTAGAGGTACAATCTTT
ATACACTGATTTTTTAAATAATTCTATCATTGAAGGATTATTATATGGGTTTAATCTAGGATTAAA
AAGTTCTTTAATGATATTCACATTTATTTGAGCTAGAGCATCCTTCCCTAGAATACGATTTGATCA
ACTAATGGGCTTCTGTTGAACAGTTTTATTACCTATTAATTTTGCAATTATTATATTAGTACCTTGT
GTTTTATATAGTTTTAACTTATTACCTGTAAATATACCATTGTTCTAGCTCACACACCCGCCCGCC
CTACTGCCACAAGGCTACAGTACATATGAGGAGGGGAACTAAAGATCTAGAACTATCCTAGTTA
ATAATTACACTTAATAGTATACTAAATAGACCATCTATCATACTCGAGAATAGTGATAGTGTAAT
TATACACTATTTTAACTGTATTCTACACTATTAGCATATTATTATCTTTATATGATAATAATTTTATA
ACTTAATTTATTAGGTTACATTAACAAAAAAATTCGTTAACTTTTTTCACCACTTTTTAATACAAAAT
ACGAATTAAA
>H-2
ATAACAATTCTAAAGAACATAAAGAGTTATTAGATAAAAATAATTCACCTATACAGTTAATAAAT
CAACTTAAAGGGTATTTTTTCATAAATCCTTTATTAGCTTTAAGTTTAGCTATTACTATTTTCTCTTT
TGCAGGTATTCCTCCTCTTGTAGGGTTCTTTGCTAAACAGATGGTATTAAGCGCGGCTATTGATC
AAGGTTATATCTTTTTATCTTTAGTTGCAATATTAACTAGTGTTATAGGAGGGGTTTATTATTTAAA
TATAATTAAAGAAATGTTCTTTTATTCACCTGACTATAAATTAAACGAAGAAATTAAAAATAATAC
TATTAATGGTCAAATTATTAATAGAAACAATAAAATATTAAATGTTGAATTTAATTATACAAATGT
AGTTATGTCTAGTTCTGTGGCAATAACTATTTCTACTATTACATTAGTAGTTTTATTATTCATGTTT
ATGAATAAAGAATGATTAAGTCTGGGTACTATATTGGTACAATCTTTATTTAGCTATTAATGAGTA
GTATGACATTATTTATAGGGTTTGTATCTGTTATAGCTATTTTATTTTTAGCCATTAATTTTATATTT
GCTCCTCATAATCCTTATCAAGAAAAATATAGTATTTTCGAGTGTGGTTTCCATAGTTTTTTAGGG
CAAAATAGAACACAATTCGGTATAAAATTCTTTATTTTTGCTTTAGTTTATTTACTTTTAGATTTAG
AAATATTATTAACTTTCCCTTTCGCTCTTAGTGAGTATGTTAATGGTATTTATGGTCTTTTAGTTAC
TTTAATTTTTATAGCTATAATAACTATAGGATTTATATTTGAATTAGGTAAAAGCGCTCTTAAAATA
GACAGCAGACAAAAATTATATATACCTAAATTGAACGTTAATTACCATACAGAGTATGTTGGAAT
AGGTAAGGTTTCTAAGTAAAGTTATAGAGGCAGAAAACCAAAAAACCTACCAAAGGGTAGCTA
ATGGGAAGCTATTAAAAATAAAAGATGATAACCTATATATAGTATAGTTACTATATATTTACTATA
ACTAGGATTATTATATATATATATATATTATCTTATTGTATATTAAGATTATTATTATTATAAGGTATA
ATTAATATAGTATCTTATTGTATAAGAATATAATATATTAACCTATAATTAATTTTATTTTTTAATTT
TTAATTATAATTTTTTTTTATATCTAGATGCTTACACATCTACAGATGTAGAAGAGAACAAAATAT
TGTTGTTATAGTAGTGGATGATATAGAAAAATATTTTTATATTATTTATTTTTGTTAGGTAGCTTTT
GAAGTGTTTGATAGAGAGGATATATGGACGGTAGGAGGGTATTCATTTTAATGAACAGTGGATA
GTTTAAATTAACCTAGTTATAGTTTATGAATTTAAATTATAATTATATTAATGTAGGTTATGATAGA
ATTATGTATTGATCCTTAAGAGTTAAGAGAGATACGCCCACGTATAATACATATTGGTTTAGGGT

TGAGTATATATACTTAAGAGTTAAGAATATATATATACAATATATAATAAATATAGTAATATATTA
ATGGTATGGACTTAACCAGGTTTATATATTATCATTTGATAAACATTAATTATAATTTTTCTATTTA
TTATTATTATTATACTGTGAGATTAATAATTATTAAAATATAATATTACATAGTACGTGATATTTGG
GGATTTTATCTTATTAATTGGCAATTAATGATTCTAATCAAATTTTATTCTCTTTAGTTTAATGGTA
GAACAATGATCTTCTAATTCATTGGTTTTAGTTCGATTCTAAAAAGAGATGAGTAAATAATTTTCT
AGATCAGAAATACTACTTTTAACTACAAAAAGCTTACGCTTTTTAACATTTTTTTGATAAATAACA
ACTATTGTTAATATTTGGCTGTCTATTGGTTTAACTAACAATTACAAAATTTTTTCAATTTTTATAAT
ATAAATTTAAATGAGAATATTAAAAAGTCATTCATTATTAAAATTAGTGAATTCTTACCTTATCGA
TGCGTCACAACCAAGTAACATTAGTTACTTGTGAAATTTTGGTTCATTATTAGCTGTTTGTTTAAT
AGTACAAATTATTACCGGTATTACATTAGCTATGCATTATAGTCCTAGTGTAATGGAAGCTTTTAA
CTCAATAGAGCATATAATGAGAGATGTTAATAACGGGTGATTAGTTCGTTATCTACATAGTAATA
CAGCTTCTGCTTTCTTTTTCTTAGTGTATTTACACATAGGAAGAGGTATATATTACGGATCATATA
GAGCTCCTCGTACTTTAGTTTGAGCTATTGGTACTGTTATATTAATATTAATGATGGCTATCGGTT
TCCTAGGTTATGTTTTACCTTATGGACAGATGTCATTATGAGGTGCTACAGTTATTACTAATCTTA
TTAGTGCTATACCTTGAATAGGGCAAGATATTGTTGAATTCATTTGAGGTGGTTTTTCTGTTAATA
ATGCCACTTTAAACAGATTTTTTGCATTACATTTTGTATTGCCTTTTGTATTAGCTGCTTTAGTTTTA
ATGCACTTAATTGCACTTCATGATACTGCTGGTTCAAGCAATCCTCTTGGTGTTTCAGGTAATTAC
GATAGAATTACATTTGCTCCATATTTTTTATTTAAAGATTTAATTACTATTTTTATATTTATTTTTGTA
TTAAGTGCTTTTGTATTCTTTATGCCTAATGTTTTAGGGGATAGTGATAATTATATTATGGCTAATC
CTATGCAAACTCCTGCTGCTATTGTACCTGAATGATACTTATTACCTTTCTATGCTATTTTAAGATC
TATACCTAATAAATTATTAGGTGTTATAGCGATGTTTAGTGCTATTTTAGCTATTATGTTATTACCT
GTTACAGATTTAGGTAGATCTAGAGGTTTACAATTTAGACCATTTAGTAAAATAGCTTTCTGAGTT
TTTGTTGCTAATTTCTTAGTTTTAATGCAATTAGGTGCTAAACACGTTGAAGATCCATTTATATTAT
TAGGTCAATTAAGTACTGTATTATACTTTAGTTATTTTGTTGCTATATTACCTTTAGCTAGTTACTT
AGATAATAGTTTAACTGATTTATCTAATAAATCTGAATTATTTTTAAATAAAACTAACTAAATATAT
TAAGATTATTATTTAATATATTTTCTATTTAAGATACTATTAATTTAGTATTTTGGGTTTTCAGTTTA
TAATTTATATTATATTATGCATTACCCTCCACCTTGCTTTGTAGTAAGCTAATCTGTTATTTCCTTTA
GTTTAATGGTAGAACAATGATCTTCTAATTCATTGGTTTTAGTTCGAATCTAAAAAGGAAATAAG
AAATATATTCTTATTATTACTTATATAATAATTATTTCTTAAAAATATACATTTTGCATTATAGCCGT
TTAGCTGTATTAAAATGTAAAATGATATAAAATAGAATAAATATTTAAATTATTCCTATGTTATATT
ATCCTATATTGCAACCATTATCAGAAGTTGTATTAATACTTGTACCTGCCTTATTAGCTGTAGCTT
ATGTTACAGTTGCTGAAAGAAAAACTATGGCTAGTATGCAAAGAAGATTAGGTCCTAATGCTGT
AGGTTACTATGGACTATTGCAAGCATTTGCTGATGCCTTAAAACTTTTATTAAAAGAATATGTAG
CTCCTACACAATCTAATATTGTTCTTTTCTTTTTAGGTCCTGTAATAACTTTAATTTTTGCATTATTA
GGTTACGCTGTTATACCCTATGGTCCTGGTTCAGGGATAAGCGACATGAATTTAGGTATATTTTA
CATGTTAGCTGTGTCATCTTTAGCTACATACGGTATTCTATTAGCTGGTTGAAGTGCGAATAGTA
AATACGCTTTTCTAGGTTCTCTTAGAAGTACAGCTCAATTAATTAGTTATGAATTAATATTAAGTT
CAGCTATATTAATAGTAATTATGATAACAGGAAATTTAAATTTAACTGTTTGTACTGAATCTCAAA
GAGCTATTTGATTTATACTACCTTTATTTCCTGTGTTTATAATATTTTTCATAGGATCTATAGCTGA
GACAAATAGAGCTCCTTTTGATTTAGCCGAGGCTAACCTGCTAATCTGGTTTGGTCTGGTTATAT
GTCACAAATTGCTAGGAAACCTTTTTATTTTAAAAACAAAAGACAATTAGCAGGAAACTTAATTT
AACCTAATTAAAAATATTAGATAATTAAACTCTTCATAGACTAAACGTGACAATTTAATATATATA
TATATTTATTTATATATATGATTAAATAAGATATAGTCAATCATCGGTGTGAATCGACTTAAAAAA
AAAAGCACATGGGTTAAACCCATCTCCCCTTATTAGGGGAATCAGAACTTGTTAGTGGGTTCAT

GACAGAGCATGCTGCCGTAGTTTTCGTATTCTTCTTTTTAGCTGAGTACGGTAGTATTGTACTAAT
GTGTATTTTAACTAGTATATTATTTATTGGTGGTTACTTATTATTTGAAATATCCTATGTTTTTACTG
TGGTAAATTATATTTTCTTTGAATTATTCTTTATAGACTGAGTAACATTTGTAGAGGTACAATCTTT
ATACACTGATTTTTTAAATAATTCTATCATTGAAGGATTATTATATGGGTTTAATCTAGGATTAAA
AAGTTCTTTAATGATATTCACATTTATTTGAGCTAGAGCATCCTTCCCTAGAATACGATTTGATCA
ACTAATGGGCTTCTGTTGAACAGTTTTATTACCTATTAATTTTGCAATTATTATATTAGTACCTTGT
GTTTTATATAGTTTTAACTTATTACCTGTAAATATACCATTGTTCTAGCTCACACACCCGCCCGCC
CTACTGCCACAAGGCTACAGTACATATGAGGAGGGGAACTAAAGATCTAGAACTATCCTAGTTA
ATAATTACACTTAATAGTATACTAAATAGACCATCTATCATACTCGAGAATAGTGATAGTGTAAT
TATACACTATTTTAACTGTATTCTACACTATTAGCATATTATTATCTTTATATGATAATAATTTTATA
ACTTAATTTATTAGGTTACATTAACAAAAAATTCGTTAACTTTTTTCACCACTTTTTAATACAAAAT
ACGAATTTTA
>Hei-1
ATAACAATTCTAAAGAACATAAAGAGTTATTAGATAAAAATAATTCACCTATACAGTTAATAAAT
CAACTTAAAGGGTATTTTTTCATAAATCCTTTATTAGCTTTAAGTTTAGCTATTACTATTTTCTCTTT
TGCAGGTATTCCTCCTCTTGTAGGGTTCTTTGCTAAACAGATGGTATTAAGCGCGGCTATTGATC
AAGGTTATATCTTTTTATCTTTAGTTGCAATATTAACTAGTGTTATAGGAGGGGTTTATTATTTAAA
TATAATTAAAGAAATGTTCTTTTATTCACCTGACTATAAATTAAACGAAGAAATTAAAAATAATAC
TATTAATGGTCAAATTATTAATAGAAACAATAAAATATTAAATGTTGAATTTAATTATACAAATGT
AGTTATGTCTAGTTCTGTGGCAATAACTATTTCTACTATTACATTAGTAGTTTTATTATTCATGTTT
ATGAATAAAGAATGATTAAGTCTGGGTACTATATTGGTACAATCTTTATTTAGCTATTAATGAGTA
GTATGACATTATTTATAGGGTTTGTATCTGTTATAGCTATTTTATTTTTAGCCATTAATTTTATATTT
GCTCCTCATAATCCTTATCAAGAAAAATATAGTATTTTCGAGTGTGGTTTCCATAGTTTTTTAGGG
CAAAATAGAACACAATTCGGTATAAAATTCTTTATTTTTGCTTTAGTTTATTTACTTTTAGATTTAG
AAATATTATTAACTTTCCCTTTCGCTCTTAGTGAGTATGTTAATGGTATTTATGGTCTTTTAGTTAC
TTTAATTTTTATAGCTATAATAACTATAGGATTTATATTTGAATTAGGTAAAAGCGCTCTTAAAATA
GACAGCAGACAAAAATTATATATACCTAAATTGAACGTTAATTACCATACAGAGTATGTTGGAAT
AGGTAAGGTTTCTAAGTAAAGTTATAGAGGCAGAAAACCAAAAAACCTACCAAAGGGTAGCTA
ATGGGAAGCTATTAAAAATAAAAGATGATAACCTATATATAGTATAGTTACTATATATTTACTATA
ACTAGGATTATTATATATATATATATTATCTTATTGTATATTAAGATTATTATTATTATAAGGTATA
ATTAATATAGTATCTTATTGTATAAGAATATAATATATTAACCTATAATTAATTTTATTTTTTAATTT
TTAATTATAATTTTTTTTTATATCTAGATGCTTACACATCTACAGATGTAGAAGAGAACAAAATAT
TGTTGTTATAGTAGTGGATGATATAGAAAAATATTTTTATATTATTTATTTTTGTTAGGTAGCTTTT
GAAGTGTTTGATAGAGAGGATATATGGACGGTAGGAGGGTATTCATTTTAATGAACAGTGGATA
GTTTAAATTAACCTAGTTATAGTTTATGAATTTAAATTATAATTATATTAATGTAGGTTATGATAGA
ATTATGTATTGATCCTTAAGAGTTAAGAGAGATACGCCCACGTATAATACATATTGGTTTAGGGT
TGAGTATATATACTTAAGAGTTAAGAATATATATATACAATATATAATAAATATAGTAATATATTA
ATGGTATGGACTTAACCAGGTTTATATATTATCATTTGATAAACATTAATTATAATTTTTCTATTTA
TTATTATTATTATACTGTGAGATTAATAATTATTAAAATATAATATTACATAGTACGTGATATTTGG
GGATTTTATCTTATTAATTGGCAATTAATGATTCTAATCAAATTTTATTCTCTTTAGTTTAATGGTA
GAACAATGATCTTCTAATTCATTGGTTTTAGTTCGATTCTAAAAAGAGATGAGTAAATAATTTTCT
AGATCAGAAATACTACTTTTAACTACAAAAAGCTTACGCTTTTTAACATTTTTTTGATAAATAACA
ACTATTGTTAATATTTGGCTGTCTATTGGTTTAACTAACAATTACAAAATTTTTTCAATTTTTATAAT
ATAAATTTAAATGAGAATATTAAAAAGTCATTCATTATTAAAATTAGTGAATTCTTACCTTATCGA

TGCGTCACAACCAAGTAACATTAGTTACTTGTGAAATTTTGGTTCATTATTAGCTGTTTGTTTAAT
AGTACAAATTATTACCGGTATTACATTAGCTATGCATTATAGTCCTAGTGTAATGGAAGCTTTTAA
CTCAATAGAGCATATAATGAGAGATGTTAATAACGGGTGATTAGTTCGTTATCTACATAGTAATA
CAGCTTCTGCTTTCTTTTTCTTAGTGTATTTACACATAGGAAGAGGTATATATTACGGATCATATA
GAGCTCCTCGTACTTTAGTTTGAGCTATTGGTACTGTTATATTAATATTAATGATGGCTATCGGTT
TCCTAGGTTATGTTTTACCTTATGGACAGATGTCATTATGAGGTGCTACAGTTATTACTAATCTTA
TTAGTGCTATACCTTGAATAGGGCAAGATATTGTTGAATTCATTTGAGGTGGTTTTTCTGTTAATA
ATGCCACTTTAAACAGATTTTTTGCATTACATTTTGTATTGCCTTTTGTATTAGCTGCTTTAGTTTTA
ATGCACTTAATTGCACTTCATGATACTGCTGGTTCAAGCAATCCTCTTGGTGTTTCAGGTAATTAC
GATAGAATTACATTTGCTCCATATTTTTTATTTAAAGATTTAATTACTATTTTTATATTTATTTTTGTA
TTAAGTGCTTTTGTATTCTTTATGCCTAATGTTTTAGGGGATAGTGATAATTATATTATGGCTAATC
CTATGCAAACTCCTGCTGCTATTGTACCTGAATGATACTTATTACCTTTCTATGCTATTTTAAGATC
TATACCTAATAAATTATTAGGTGTTATAGCGATGTTTAGTGCTATTTTAGCTATTATGTTATTACCT
GTTACAGATTTAGGTAGATCTAGAGGTTTACAATTTAGACCATTTAGTAAAATAGCTTTCTGAGTT
TTTGTTGCTAATTTCTTAGTTTTAATGCAATTAGGTGCTAAACACGTTGAAGATCCATTTATATTAT
TAGGTCAATTAAGTACTGTATTATACTTTAGTTATTTTGTTGCTATATTACCTTTAGCTAGTTACTT
AGATAATAGTTTAACTGATTTATCTAATAAATCTGAATTATTTTTAAATAAAACTAACTAAATATAT
TAAGATTATTATTTAATATATTTTCTATTTAAGATACTATTAATTTAGTATTTTGGGTTTTCAGTTTA
TAATTTATATTATATTATGCATTACCCTCCACCTTGCTTTGTAGTAAGCTAATCTGTTATTTCCTTTA
GTTTAATGGTAGAACAATGATCTTCTAATTCATTGGTTTTAGTTCGAATCTAAAAAGGAAATAAG
AAATATATTCTTATTATTACTTATATAATAATTATTTCTTAAAAATATACATTTTGCATTATAGCCGT
TTAGCTGTATTAAAATGTAAAATGATATAAAATAGAATAAATATTTAAATTATTCCTATGTTATATT
ATCCTATATTGCAACCATTATCAGAAGTTGTATTAATACTTGTACCTGCCTTATTAGCTGTAGCTT
ATGTTACAGTTGCTGAAAGAAAAACTATGGCTAGTATGCAAAGAAGATTAGGTCCTAATGCTGT
AGGTTACTATGGACTATTGCAAGCATTTGCTGATGCCTTAAAACTTTTATTAAAAGAATATGTAG
CTCCTACACAATCTAATATTGTTCTTTTCTTTTTAGGTCCTGTAATAACTTTAATTTTTGCATTATTA
GGTTACGCTGTTATACCCTATGGTCCTGGTTCAGGGATAAGCGACATGAATTTAGGTATATTTTA
CATGTTAGCTGTGTCATCTTTAGCTACATACGGTATTCTATTAGCTGGTTGAAGTGCGAATAGTA
AATACGCTTTTCTAGGTTCTCTTAGAAGTACAGCTCAATTAATTAGTTATGAATTAATATTAAGTT
CAGCTATATTAATAGTAATTATGATAACAGGAAATTTAAATTTAACTGTTTGTACTGAATCTCAAA
GAGCTATTTGATTTATACTACCTTTATTTCCTGTGTTTATAATATTTTTCATAGGATCTATAGCTGA
GACAAATAGAGCTCCTTTTGATTTAGCCGAGGCTAACCTGCTAATCTGGTTTGGTCTGGTTATAT
GTCACAAATTGCTAGGAAACCTTTTTATTTTAAAAACAAAAGACAATTAGCAGGAAACTTAATTT
AACCTAATTAAAAATATTAGATAATTAAACTCTTCATAGACTAAACGTGACAATTTAATATATATA
TATATTTATTTATATATATGATTAAATAAGATATAGTCAATCATCGGTGTGAATCGACTTAAAAAA
AAAAGCACATGGGTTAAACCCATCTCCCCTTATTAGGGGAATCAGAACTTGTTAGTGGGTTCAT
GACAGAGCATGCTGCCGTAGTTTTCGTATTCTTCTTTTTAGCTGAGTACGGTAGTATTGTACTAAT
GTGTATTTTAACTAGTATATTATTTATTGGTGGTTACTTATTATTTGAAATATCCTATGTTTTTACTG
TGGTAAATTATATTTTCTTTGAATTATTCTTTATAGACTGAGTAACATTTGTAGAGGTACAATCTTT
ATACACTGATTTTTTAAATAATTCTATCATTGAAGGATTATTATATGGGTTTAATCTAGGATTAAA
AAGTTCTTTAATGATATTCACATTTATTTGAGCTAGAGCATCCTTCCCTAGAATACGATTTGATCA
ACTAATGGGCTTCTGTTGAACAGTTTTATTACCTATTAATTTTGCAATTATTATATTAGTACCTTGT
GTTTTATATAGTTTTAACTTATTACCTGTAAATATACCATTGTTCTAGCTCACACACCCGCCCGCC
CTACTGCCACAAGGCTACAGTACATATGAGGAGGGGAACTAAAGATCTAGAACTATCCTAGTTA

ATAATTACACTTAATAGTATACTAAATAGACCATCTATCATACTCGAGAATAGTGATAGTGTAAT
TATACACTATTTTAACTGTATTCTACACTATTAGCATATTATTATCTTTATATGATAATAATTTTATA
ACTTAATTTATTAGGTTACATTAACAAAAAATTCGTTAACTTTTTTCACCACTTTTTAATACAAAAT
ACGAATTTTA
>Hei-3
ATAACAATTCTAAAGAACATAAAGAGTTATTAGATAAAAATAATTCACCTATACAGTTAATAAAT
CAACTTAAAGGGTATTTTTTCATAAATCCTTTATTAGCTTTAAGTTTAGCTATTACTATTTTCTCTTT
TGCAGGTATTCCTCCTCTTGTAGGGTTCTTTGCTAAACAGATGGTATTAAGCGCGGCTATTGATC
AAGGTTATATCTTTTTATCTTTAGTTGCAATATTAACTAGTGTTATAGGAGGGGTTTATTATTTAAA
TATAATTAAAGAAATGTTCTTTTATTCACCTGACTATAAATTAAACGAAGAAATTAAAAATAATAC
TATTAATGGTCAAATTATTAATAGAAACAATAAAATATTAAATGTTGAATTTAATTATACAAATGT
AGTTATGTCTAGTTCTGTGGCAATAACTATTTCTACTATTACATTAGTAGTTTTATTATTCATGTTT
ATGAATAAAGAATGATTAAGTCTGGGTACTATATTGGTACAATCTTTATTTAGCTATTAATGAGTA
GTATGACATTATTTATAGGGTTTGTATCTGTTATAGCTATTTTATTTTTAGCCATTAATTTTATATTT
GCTCCTCATAATCCTTATCAAGAAAAATATAGTATTTTCGAGTGTGGTTTCCATAGTTTTTTAGGG
CAAAATAGAACACAATTCGGTATAAAATTCTTTATTTTTGCTTTAGTTTATTTACTTTTAGATTTAG
AAATATTATTAACTTTCCCTTTCGCTCTTAGTGAGTATGTTAATGGTATTTATGGTCTTTTAGTTAC
TTTAATTTTTATAGCTATAATAACTATAGGATTTATATTTGAATTAGGTAAAAGCGCTCTTAAAATA
GACAGCAGACAAAAATTATATATACCTAAATTGAACGTTAATTACCATACAGAGTATGTTGGAAT
AGGTAAGGTTTCTAAGTAAAGTTATAGAGGCAGAAAACCAAAAAACCTACCAAAGGGTAGCTA
ATGGGAAGCTATTAAAAATAAAAGATGATAACCTATATATAGTATAGTTACTATATATTTACTATA
ACTAGGATTATTATATATATATATATTATCTTATTGTATATTAAGATTATTATTATTATAAGGTATA
ATTAATATAGTATCTTATTGTATAAGAATATAATATATTAACCTATAATTAATTTTATTTTTTAATTT
TTAATTATAATTTTTTTTTATATCTAGATGCTTACACATCTACAGATGTAGAAGAGAACAAAATAT
TGTTGTTATAGTAGTGGATGATATAGAAAAATATTTTTATATTATTTATTTTTGTTAGGTAGCTTTT
GAAGTGTTTGATAGAGAGGATATATGGACGGTAGGAGGGTATTCATTTTAATGAACAGTGGATA
GTTTAAATTAACCTAGTTATAGTTTATGAATTTAAATTATAATTATATTAATGTAGGTTATGATAGA
ATTATGTATTGATCCTTAAGAGTTAAGAGAGATACGCCCACGTATAATACATATTGGTTTAGGGT
TGAGTATATATACTTAAGAGTTAAGAATATATATATACAATATATAATAAATATAGTAATATATTA
ATGGTATGGACTTAACCAGGTTTATATATTATCATTTGATAAACATTAATTATAATTTTTCTATTTA
TTATTATTATTATACTGTGAGATTAATAATTATTAAAATATAATATTACATAGTACGTGATATTTGG
GGATTTTATCTTATTAATTGGCAATTAATGATTCTAATCAAATTTTATTCTCTTTAGTTTAATGGTA
GAACAATGATCTTCTAATTCATTGGTTTTAGTTCGATTCTAAAAAGAGATGAGTAAATAATTTTCT
AGATCAGAAATACTACTTTTAACTACAAAAAGCTTACGCTTTTTAACATTTTTTTGATAAATAACA
ACTATTGTTAATATTTGGCTGTCTATTGGTTTAACTAACAATTACAAAATTTTTTCAATTTTTATAAT
ATAAATTTAAATGAGAATATTAAAAAGTCATTCATTATTAAAATTAGTGAATTCTTACCTTATCGA
TGCGTCACAACCAAGTAACATTAGTTACTTGTGAAATTTTGGTTCATTATTAGCTGTTTGTTTAAT
AGTACAAATTATTACCGGTATTACATTAGCTATGCATTATAGTCCTAGTGTAATGGAAGCTTTTAA
CTCAATAGAGCATATAATGAGAGATGTTAATAACGGGTGATTAGTTCGTTATCTACATAGTAATA
CAGCTTCTGCTTTCTTTTTCTTAGTGTATTTACACATAGGAAGAGGTATATATTACGGATCATATA
GAGCTCCTCGTACTTTAGTTTGAGCTATTGGTACTGTTATATTAATATTAATGATGGCTATCGGTT
TCCTAGGTTATGTTTTACCTTATGGACAGATGTCATTATGAGGTGCTACAGTTATTACTAATCTTA
TTAGTGCTATACCTTGAATAGGGCAAGATATTGTTGAATTCATTTGAGGTGGTTTTTCTGTTAATA
ATGCCACTTTAAACAGATTTTTTGCATTACATTTTGTATTGCCTTTTGTATTAGCTGCTTTAGTTTTA

ATGCACTTAATTGCACTTCATGATACTGCTGGTTCAAGCAATCCTCTTGGTGTTTCAGGTAATTAC
GATAGAATTACATTTGCTCCATATTTTTTATTTAAAGATTTAATTACTATTTTTATATTTATTTTTGTA
TTAAGTGCTTTTGTATTCTTTATGCCTAATGTTTTAGGGGATAGTGATAATTATATTATGGCTAATC
CTATGCAAACTCCTGCTGCTATTGTACCTGAATGATACTTATTACCTTTCTATGCTATTTTAAGATC
TATACCTAATAAATTATTAGGTGTTATAGCGATGTTTAGTGCTATTTTAGCTATTATGTTATTACCT
GTTACAGATTTAGGTAGATCTAGAGGTTTACAATTTAGACCATTTAGTAAAATAGCTTTCTGAGTT
TTTGTTGCTAATTTCTTAGTTTTAATGCAATTAGGTGCTAAACACGTTGAAGATCCATTTATATTAT
TAGGTCAATTAAGTACTGTATTATACTTTAGTTATTTTGTTGCTATATTACCTTTAGCTAGTTACTT
AGATAATAGTTTAACTGATTTATCTAATAAATCTGAATTATTTTTAAATAAAACTAACTAAATATAT
TAAGATTATTATTTAATATATTTTCTATTTAAGATACTATTAATTTAGTATTTTGGGTTTTCAGTTTA
TAATTTATATTATATTATGCATTACCCTCCACCTTGCTTTGTAGTAAGCTAATCTGTTATTTCCTTTA
GTTTAATGGTAGAACAATGATCTTCTAATTCATTGGTTTTAGTTCGAATCTAAAAAGGAAATAAG
AAATATATTCTTATTATTACTTATATAATAATTATTTCTTAAAAATATACATTTTGCATTATAGCCGT
TTAGCTGTATTAAAATGTAAAATGATATAAAATAGAATAAATATTTAAATTATTCCTATGTTATATT
ATCCTATATTGCAACCATTATCAGAAGTTGTATTAATACTTGTACCTGCCTTATTAGCTGTAGCTT
ATGTTACAGTTGCTGAAAGAAAAACTATGGCTAGTATGCAAAGAAGATTAGGTCCTAATGCTGT
AGGTTACTATGGACTATTGCAAGCATTTGCTGATGCCTTAAAACTTTTATTAAAAGAATATGTAG
CTCCTACACAATCTAATATTGTTCTTTTCTTTTTAGGTCCTGTAATAACTTTAATTTTTGCATTATTA
GGTTACGCTGTTATACCCTATGGTCCTGGTTCAGGGATAAGCGACATGAATTTAGGTATATTTTA
CATGTTAGCTGTGTCATCTTTAGCTACATACGGTATTCTATTAGCTGGTTGAAGTGCGAATAGTA
AATACGCTTTTCTAGGTTCTCTTAGAAGTACAGCTCAATTAATTAGTTATGAATTAATATTAAGTT
CAGCTATATTAATAGTAATTATGATAACAGGAAATTTAAATTTAACTGTTTGTACTGAATCTCAAA
GAGCTATTTGATTTATACTACCTTTATTTCCTGTGTTTATAATATTTTTCATAGGATCTATAGCTGA
GACAAATAGAGCTCCTTTTGATTTAGCCGAGGCTAACCTGCTAATCTGGTTTGGTCTGGTTATAT
GTCACAAATTGCTAGGAAACCTTTTTATTTTAAAAACAAAAGACAATTAGCAGGAAACTTAATTT
AACCTAATTAAAAATATTAGATAATTAAACTCTTCATAGACTAAACGTGACAATTTAATATATATA
TATATTTATTTATATATATGATTAAATAAGATATAGTCAATCATCGGTGTGAATCGACTTAAAAAA
AAAAGCACATGGGTTAAACCCATCTCCCCTTATTAGGGGAATCAGAACTTGTTAGTGGGTTCAT
GACAGAGCATGCTGCCGTAGTTTTCGTATTCTTCTTTTTAGCTGAGTACGGTAGTATTGTACTAAT
GTGTATTTTAACTAGTATATTATTTATTGGTGGTTACTTATTATTTGAAATATCCTATGTTTTTACTG
TGGTAAATTATATTTTCTTTGAATTATTCTTTATAGACTGAGTAACATTTGTAGAGGTACAATCTTT
ATACACTGATTTTTTAAATAATTCTATCATTGAAGGATTATTATATGGGTTTAATCTAGGATTAAA
AAGTTCTTTAATGATATTCACATTTATTTGAGCTAGAGCATCCTTCCCTAGAATACGATTTGATCA
ACTAATGGGCTTCTGTTGAACAGTTTTATTACCTATTAATTTTGCAATTATTATATTAGTACCTTGT
GTTTTATATAGTTTTAACTTATTACCTGTAAATATACCATTGTTCTAGCTCACACACCCGCCCGCC
CTACTGCCACAAGGCTACAGTACATATGAGGAGGGGAACTAAAGATCTAGAACTATCCTAGTTA
ATAATTACACTTAATAGTATACTAAATAGACCATCTATCATACTCGAGAATAGTGATAGTGTAAT
TATACACTATTTTAACTGTATTCTACACTATTAGCATATTATTATCTTTATATGATAATAATTTTATA
ACTTAATTTATTAGGTTACATTAACAAAAAATTCGTTAACTTTTTTCACCACTTTTTAATACAAAAT
ACGAATTTTA
>Hu-1
ATAACAATTCTAAAGAACATAAAGAGTTATTAGATAAAAATAATTCACCTATACAGTTAATAAAT
CAACTTAAAGGGTATTTTTTCATAAATCCTTTATTAGCTTTAAGTTTAGCTATTACTATTTTCTCTTT
TGCAGGTATTCCTCCTCTTGTAGGGTTCTTTGCTAAACAGATGGTATTAAGCGCGGCTATTGATC

AAGGTTATATCTTTTTATCTTTAGTTGCAATATTAACTAGTGTTATAGGAGGGGTTTATTATTTAAA
TATAATTAAAGAAATGTTCTTTTATTCACCTGACTATAAATTAAACGAAGAAATTAAAAATAATAC
TATTAATGGTCAAATTATTAATAGAAACAATAAAATATTAAATGTTGAATTTAATTATACAAATGT
AGTTATGTCTAGTTCTGTGGCAATAACTATTTCTACTATTACATTAGTAGTTTTATTATTCATGTTT
ATGAATAAAGAATGATTAAGTCTGGGTACTATATTGGTACAATCTTTATTTAGCTATTAATGAGTA
GTATGACATTATTTATAGGGTTTGTATCTGTTATAGCTATTTTATTTTTAGCCATTAATTTTATATTT
GCTCCTCATAATCCTTATCAAGAAAAATATAGTATTTTCGAGTGTGGTTTCCATAGTTTTTTAGGG
CAAAATAGAACACAATTCGGTATAAAATTCTTTATTTTTGCTTTAGTTTATTTACTTTTAGATTTAG
AAATATTATTAACTTTCCCTTTCGCTCTTAGTGAGTATGTTAATGGTATTTATGGTCTTTTAGTTAC
TTTAATTTTTATAGCTATAATAACTATAGGATTTATATTTGAATTAGGTAAAAGCGCTCTTAAAATA
GACAGCAGACAAAAATTATATATACCTAAATTGAACGTTAATTACCATACAGAGTATGTTGGAAT
AGGTAAGGTTTCTAAGTAAAGTTATAGAGGCAGAAAACCAAAAAACCTACCAAAGGGTAGCTA
ATGGGAAGCTATTAAAAATAAAAGATGATAACCTATATATAGTATAGTTACTATATATTTACTATA
ACTAGGATTATTATATATATATATATTATCTTATTGTATATTAAGATTATTATTATTATAAGGTATA
ATTAATATAGTATCTTATTGTATAAGAATATAATATATTAACCTATAATTAATTTTATTTTTTAATTT
TTAATTATAATTTTTTTTTATATCTAGATGCTTACACATCTACAGATGTAGAAGAGAACAAAATAT
TGTTGTTATAGTAGTGGATGATATAGAAAAATATTTTTATATTATTTATTTTTGTTAGGTAGCTTTT
GAAGTGTTTGATAGAGAGGATATATGGACGGTAGGAGGGTATTCATTTTAATGAACAGTGGATA
GTTTAAATTAACCTAGTTATAGTTTATGAATTTAAATTATAATTATATTAATGTAGGTTATGATAGA
ATTATGTATTGATCCTTAAGAGTTAAGAGAGATACGCCCACGTATAATACATATTGGTTTAGGGT
TGAGTATATATACTTAAGAGTTAAGAATATATATATACAATATATAATAAATATAGTAATATATTA
ATGGTATGGACTTAACCAGGTTTATATATTATCATTTGATAAACATTAATTATAATTTTTCTATTTA
TTATTATTATTATACTGTGAGATTAATAATTATTAAAATATAATATTACATAGTACGTGATATTTGG
GGATTTTATCTTATTAATTGGCAATTAATGATTCTAATCAAATTTTATTCTCTTTAGTTTAATGGTA
GAACAATGATCTTCTAATTCATTGGTTTTAGTTCGATTCTAAAAAGAGATGAGTAAATAATTTTCT
AGATCAGAAATACTACTTTTAACTACAAAAAGCTTACGCTTTTTAACATTTTTTTGATAAATAACA
ACTATTGTTAATATTTGGCTGTCTATTGGTTTAACTAACAATTACAAAATTTTTTCAATTTTTATAAT
ATAAATTTAAATGAGAATATTAAAAAGTCATTCATTATTAAAATTAGTGAATTCTTACCTTATCGA
TGCGTCACAACCAAGTAACATTAGTTACTTGTGAAATTTTGGTTCATTATTAGCTGTTTGTTTAAT
AGTACAAATTATTACCGGTATTACATTAGCTATGCATTATAGTCCTAGTGTAATGGAAGCTTTTAA
CTCAATAGAGCATATAATGAGAGATGTTAATAACGGGTGATTAGTTCGTTATCTACATAGTAATA
CAGCTTCTGCTTTCTTTTTCTTAGTGTATTTACACATAGGAAGAGGTATATATTACGGATCATATA
GAGCTCCTCGTACTTTAGTTTGAGCTATTGGTACTGTTATATTAATATTAATGATGGCTATCGGTT
TCCTAGGTTATGTTTTACCTTATGGACAGATGTCATTATGAGGTGCTACAGTTATTACTAATCTTA
TTAGTGCTATACCTTGAATAGGGCAAGATATTGTTGAATTCATTTGAGGTGGTTTTTCTGTTAATA
ATGCCACTTTAAACAGATTTTTTGCATTACATTTTGTATTGCCTTTTGTATTAGCTGCTTTAGTTTTA
ATGCACTTAATTGCACTTCATGATACTGCTGGTTCAAGCAATCCTCTTGGTGTTTCAGGTAATTAC
GATAGAATTACATTTGCTCCATATTTTTTATTTAAAGATTTAATTACTATTTTTATATTTATTTTTGTA
TTAAGTGCTTTTGTATTCTTTATGCCTAATGTTTTAGGGGATAGTGATAATTATATTATGGCTAATC
CTATGCAAACTCCTGCTGCTATTGTACCTGAATGATACTTATTACCTTTCTATGCTATTTTAAGATC
TATACCTAATAAATTATTAGGTGTTATAGCGATGTTTAGTGCTATTTTAGCTATTATGTTATTACCT
GTTACAGATTTAGGTAGATCTAGAGGTTTACAATTTAGACCATTTAGTAAAATAGCTTTCTGAGTT
TTTGTTGCTAATTTCTTAGTTTTAATGCAATTAGGTGCTAAACACGTTGAAGATCCATTTATATTAT
TAGGTCAATTAAGTACTGTATTATACTTTAGTTATTTTGTTGCTATATTACCTTTAGCTAGTTACTT

AGATAATAGTTTAACTGATTTATCTAATAAATCTGAATTATTTTTAAATAAAACTAACTAAATATAT
TAAGATTATTATTTAATATATTTTCTATTTAAGATACTATTAATTTAGTATTTTGGGTTTTCAGTTTA
TAATTTATATTATATTATGCATTACCCTCCACCTTGCTTTGTAGTAAGCTAATCTGTTATTTCCTTTA
GTTTAATGGTAGAACAATGATCTTCTAATTCATTGGTTTTAGTTCGAATCTAAAAAGGAAATAAG
AAATATATTCTTATTATTACTTATATAATAATTATTTCTTAAAAATATACATTTTGCATTATAGCCGT
TTAGCTGTATTAAAATGTAAAATGATATAAAATAGAATAAATATTTAAATTATTCCTATGTTATATT
ATCCTATATTGCAACCATTATCAGAAGTTGTATTAATACTTGTACCTGCCTTATTAGCTGTAGCTT
ATGTTACAGTTGCTGAAAGAAAAACTATGGCTAGTATGCAAAGAAGATTAGGTCCTAATGCTGT
AGGTTACTATGGACTATTGCAAGCATTTGCTGATGCCTTAAAACTTTTATTAAAAGAATATGTAG
CTCCTACACAATCTAATATTGTTCTTTTCTTTTTAGGTCCTGTAATAACTTTAATTTTTGCATTATTA
GGTTACGCTGTTATACCCTATGGTCCTGGTTCAGGGATAAGCGACATGAATTTAGGTATATTTTA
CATGTTAGCTGTGTCATCTTTAGCTACATACGGTATTCTATTAGCTGGTTGAAGTGCGAATAGTA
AATACGCTTTTCTAGGTTCTCTTAGAAGTACAGCTCAATTAATTAGTTATGAATTAATATTAAGTT
CAGCTATATTAATAGTAATTATGATAACAGGAAATTTAAATTTAACTGTTTGTACTGAATCTCAAA
GAGCTATTTGATTTATACTACCTTTATTTCCTGTGTTTATAATATTTTTCATAGGATCTATAGCTGA
GACAAATAGAGCTCCTTTTGATTTAGCCGAGGCTAACCTGCTAATCTGGTTTGGTCTGGTTATAT
GTCACAAATTGCTAGGAAACCTTTTTATTTTAAAAACAAAAGACAATTAGCAGGAAACTTAATTT
AACCTAATTAAAAATATTAGATAATTAAACTCTTCATAGACTAAACGTGACAATTTAATATATATA
TATATTTATTTATATATATGATTAAATAAGATATAGTCAATCATCGGTGTGAATCGACTTAAAAAA
AAAAGCACATGGGTTAAACCCATCTCCCCTTATTAGGGGAATCAGAACTTGTTAGTGGGTTCAT
GACAGAGCATGCTGCCGTAGTTTTCGTATTCTTCTTTTTAGCTGAGTACGGTAGTATTGTACTAAT
GTGTATTTTAACTAGTATATTATTTATTGGTGGTTACTTATTATTTGAAATATCCTATGTTTTTACTG
TGGTAAATTATATTTTCTTTGAATTATTCTTTATAGACTGAGTAACATTTGTAGAGGTACAATCTTT
ATACACTGATTTTTTAAATAATTCTATCATTGAAGGATTATTATATGGGTTTAATCTAGGATTAAA
AAGTTCTTTAATGATATTCACATTTATTTGAGCTAGAGCATCCTTCCCTAGAATACGATTTGATCA
ACTAATGGGCTTCTGTTGAACAGTTTTATTACCTATTAATTTTGCAATTATTATATTAGTACCTTGT
GTTTTATATAGTTTTAACTTATTACCTGTAAATATACCATTGTTCTAGCTCACACACCCGCCCGCC
CTACTGCCACAAGGCTACAGTACATATGAGGAGGGGAACTAAAGATCTAGAACTATCCTAGTTA
ATAATTACACTTAATAGTATACTAAATAGACCATCTATCATACTCGAGAATAGTGATAGTGTAAT
TATACACTATTTTAACTGTATTCTACACTATTAGCATATTATTATCTTTATATGATAATAATTTTATA
ACTTAATTTATTAGGTTACATTAACAAAAAATTCGTTAACTTTTTTCACCACTTTTTAATACAAAAT
ACGAATTTTA
>Hu-2
ATAACAATTCTAAAGAACATAAAGAGTTATTAGATAAAAATAATTCACCTATACAGTTAATAAAT
CAACTTAAAGGGTATTTTTTCATAAATCCTTTATTAGCTTTAAGTTTAGCTATTACTATTTTCTCTTT
TGCAGGTATTCCTCCTCTTGTAGGGTTCTTTGCTAAACAGATGGTATTAAGCGCGGCTATTGATC
AAGGTTATATCTTTTTATCTTTAGTTGCAATATTAACTAGTGTTATAGGAGGGGTTTATTATTTAAA
TATAATTAAAGAAATGTTCTTTTATTCACCTGACTATAAATTAAACGAAGAAATTAAAAATAATAC
TATTAATGGTCAAATTATTAATAGAAACAATAAAATATTAAATGTTGAATTTAATTATACAAATGT
AGTTATGTCTAGTTCTGTGGCAATAACTATTTCTACTATTACATTAGTAGTTTTATTATTCATGTTT
ATGAATAAAGAATGATTAAGTCTGGGTACTATATTGGTACAATCTTTATTTAGCTATTAATGAGTA
GTATGACATTATTTATAGGGTTTGTATCTGTTATAGCTATTTTATTTTTAGCCATTAATTTTATATTT
GCTCCTCATAATCCTTATCAAGAAAAATATAGTATTTTCGAGTGTGGTTTCCATAGTTTTTTAGGG
CAAAATAGAACACAATTCGGTATAAAATTCTTTATTTTTGCTTTAGTTTATTTACTTTTAGATTTAG

AAATATTATTAACTTTCCCTTTCGCTCTTAGTGAGTATGTTAATGGTATTTATGGTCTTTTAGTTAC
TTTAATTTTTATAGCTATAATAACTATAGGATTTATATTTGAATTAGGTAAAAGCGCTCTTAAAATA
GACAGCAGACAAAAATTATATATACCTAAATTGAACGTTAATTACCATACAGAGTATGTTGGAAT
AGGTAAGGTTTCTAAGTAAAGTTATAGAGGCAGAAAACCAAAAAACCTACCAAAGGGTAGCTA
ATGGGAAGCTATTAAAAATAAAAGATGATAACCTATATATAGTATAGTTACTATATATTTACTATA
ACTAGGATTATTATATATATATATATTATCTTATTGTATATTAAGATTATTATTATTATAAGGTATA
ATTAATATAGTATCTTATTGTATAAGAATATAATATATTAACCTATAATTAATTTTATTTTTTAATTT
TTAATTATAATTTTTTTTTATATCTAGATGCTTACACATCTACAGATGTAGAAGAGAACAAAATAT
TGTTGTTATAGTAGTGGATGATATAGAAAAATATTTTTATATTATTTATTTTTGTTAGGTAGCTTTT
GAAGTGTTTGATAGAGAGGATATATGGACGGTAGGAGGGTATTCATTTTAATGAACAGTGGATA
GTTTAAATTAACCTAGTTATAGTTTATGAATTTAAATTATAATTATATTAATGTAGGTTATGATAGA
ATTATGTATTGATCCTTAAGAGTTAAGAGAGATACGCCCACGTATAATACATATTGGTTTAGGGT
TGAGTATATATACTTAAGAGTTAAGAATATATATATACAATATATAATAAATATAGTAATATATTA
ATGGTATGGACTTAACCAGGTTTATATATTATCATTTGATAAACATTAATTATAATTTTTCTATTTA
TTATTATTATTATACTGTGAGATTAATAATTATTAAAATATAATATTACATAGTACGTGATATTTGG
GGATTTTATCTTATTAATTGGCAATTAATGATTCTAATCAAATTTTATTCTCTTTAGTTTAATGGTA
GAACAATGATCTTCTAATTCATTGGTTTTAGTTCGATTCTAAAAAGAGATGAGTAAATAATTTTCT
AGATCAGAAATACTACTTTTAACTACAAAAAGCTTACGCTTTTTAACATTTTTTTGATAAATAACA
ACTATTGTTAATATTTGGCTGTCTATTGGTTTAACTAACAATTACAAAATTTTTTCAATTTTTATAAT
ATAAATTTAAATGAGAATATTAAAAAGTCATTCATTATTAAAATTAGTGAATTCTTACCTTATCGA
TGCGTCACAACCAAGTAACATTAGTTACTTGTGAAATTTTGGTTCATTATTAGCTGTTTGTTTAAT
AGTACAAATTATTACCGGTATTACATTAGCTATGCATTATAGTCCTAGTGTAATGGAAGCTTTTAA
CTCAATAGAGCATATAATGAGAGATGTTAATAACGGGTGATTAGTTCGTTATCTACATAGTAATA
CAGCTTCTGCTTTCTTTTTCTTAGTGTATTTACACATAGGAAGAGGTATATATTACGGATCATATA
GAGCTCCTCGTACTTTAGTTTGAGCTATTGGTACTGTTATATTAATATTAATGATGGCTATCGGTT
TCCTAGGTTATGTTTTACCTTATGGACAGATGTCATTATGAGGTGCTACAGTTATTACTAATCTTA
TTAGTGCTATACCTTGAATAGGGCAAGATATTGTTGAATTCATTTGAGGTGGTTTTTCTGTTAATA
ATGCCACTTTAAACAGATTTTTTGCATTACATTTTGTATTGCCTTTTGTATTAGCTGCTTTAGTTTTA
ATGCACTTAATTGCACTTCATGATACTGCTGGTTCAAGCAATCCTCTTGGTGTTTCAGGTAATTAC
GATAGAATTACATTTGCTCCATATTTTTTATTTAAAGATTTAATTACTATTTTTATATTTATTTTTGTA
TTAAGTGCTTTTGTATTCTTTATGCCTAATGTTTTAGGGGATAGTGATAATTATATTATGGCTAATC
CTATGCAAACTCCTGCTGCTATTGTACCTGAATGATACTTATTACCTTTCTATGCTATTTTAAGATC
TATACCTAATAAATTATTAGGTGTTATAGCGATGTTTAGTGCTATTTTAGCTATTATGTTATTACCT
GTTACAGATTTAGGTAGATCTAGAGGTTTACAATTTAGACCATTTAGTAAAATAGCTTTCTGAGTT
TTTGTTGCTAATTTCTTAGTTTTAATGCAATTAGGTGCTAAACACGTTGAAGATCCATTTATATTAT
TAGGTCAATTAAGTACTGTATTATACTTTAGTTATTTTGTTGCTATATTACCTTTAGCTAGTTACTT
AGATAATAGTTTAACTGATTTATCTAATAAATCTGAATTATTTTTAAATAAAACTAACTAAATATAT
TAAGATTATTATTTAATATATTTTCTATTTAAGATACTATTAATTTAGTATTTTGGGTTTTCAGTTTA
TAATTTATATTATATTATGCATTACCCTCCACCTTGCTTTGTAGTAAGCTAATCTGTTATTTCCTTTA
GTTTAATGGTAGAACAATGATCTTCTAATTCATTGGTTTTAGTTCGAATCTAAAAAGGAAATAAG
AAATATATTCTTATTATTACTTATATAATAATTATTTCTTAAAAATATACATTTTGCATTATAGCCGT
TTAGCTGTATTAAAATGTAAAATGATATAAAATAGAATAAATATTTAAATTATTCCTATGTTATATT
ATCCTATATTGCAACCATTATCAGAAGTTGTATTAATACTTGTACCTGCCTTATTAGCTGTAGCTT
ATGTTACAGTTGCTGAAAGAAAAACTATGGCTAGTATGCAAAGAAGATTAGGTCCTAATGCTGT

AGGTTACTATGGACTATTGCAAGCATTTGCTGATGCCTTAAAACTTTTATTAAAAGAATATGTAG
CTCCTACACAATCTAATATTGTTCTTTTCTTTTTAGGTCCTGTAATAACTTTAATTTTTGCATTATTA
GGTTACGCTGTTATACCCTATGGTCCTGGTTCAGGGATAAGCGACATGAATTTAGGTATATTTTA
CATGTTAGCTGTGTCATCTTTAGCTACATACGGTATTCTATTAGCTGGTTGAAGTGCGAATAGTA
AATACGCTTTTCTAGGTTCTCTTAGAAGTACAGCTCAATTAATTAGTTATGAATTAATATTAAGTT
CAGCTATATTAATAGTAATTATGATAACAGGAAATTTAAATTTAACTGTTTGTACTGAATCTCAAA
GAGCTATTTGATTTATACTACCTTTATTTCCTGTGTTTATAATATTTTTCATAGGATCTATAGCTGA
GACAAATAGAGCTCCTTTTGATTTAGCCGAGGCTAACCTGCTAATCTGGTTTGGTCTGGTTATAT
GTCACAAATTGCTAGGAAACCTTTTTATTTTAAAAACAAAAGACAATTAGCAGGAAACTTAATTT
AACCTAATTAAAAATATTAGATAATTAAACTCTTCATAGACTAAACGTGACAATTTAATATATATA
TATATTTATTTATATATATGATTAAATAAGATATAGTCAATCATCGGTGTGAATCGACTTAAAAAA
AAAAGCACATGGGTTAAACCCATCTCCCCTTATTAGGGGAATCAGAACTTGTTAGTGGGTTCAT
GACAGAGCATGCTGCCGTAGTTTTCGTATTCTTCTTTTTAGCTGAGTACGGTAGTATTGTACTAAT
GTGTATTTTAACTAGTATATTATTTATTGGTGGTTACTTATTATTTGAAATATCCTATGTTTTTACTG
TGGTAAATTATATTTTCTTTGAATTATTCTTTATAGACTGAGTAACATTTGTAGAGGTACAATCTTT
ATACACTGATTTTTTAAATAATTCTATCATTGAAGGATTATTATATGGGTTTAATCTAGGATTAAA
AAGTTCTTTAATGATATTCACATTTATTTGAGCTAGAGCATCCTTCCCTAGAATACGATTTGATCA
ACTAATGGGCTTCTGTTGAACAGTTTTATTACCTATTAATTTTGCAATTATTATATTAGTACCTTGT
GTTTTATATAGTTTTAACTTATTACCTGTAAATATACCATTGTTCTAGCTCACACACCCGCCCGCC
CTACTGCCACAAGGCTACAGTACATATGAGGAGGGGAACTAAAGATCTAGAACTATCCTAGTTA
ATAATTACACTTAATAGTATACTAAATAGACCATCTATCATACTCGAGAATAGTGATAGTGTAAT
TATACACTATTTTAACTGTATTCTACACTATTAGCATATTATTATCTTTATATGATAATAATTTTATA
ACTTAATTTATTAGGTTACATTAACAAAAAAATTCGTTAACTTTTTTCACCACTTTTTAATACAAAAT
ACGAATTTTA
>I-1
ATAACAATTCTAAAGAACATAAAGAGTTATTAGATAAAAATAATTCACCTATACAGTTAATAAAT
CAACTTAAAGGGTATTTTTTCATAAATCCTTTATTAGCTTTAAGTTTAGCTATTACTATTTTCTCTTT
TGCAGGTATTCCTCCTCTTGTAGGGTTCTTTGCTAAACAGATGGTATTAAGCGCGGCTATTGATC
AAGGTTATATCTTTTTATCTTTAGTTGCAATATTAACTAGTGTTATAGGAGGGGTTTATTATTTAAA
TATAATTAAAGAAATGTTCTTTTATTCACCTGACTATAAATTAAACGAAGAAATTAAAAATAATAC
TATTAATGGTCAAATTATTAATAGAAACAATAAAATATTAAATGTTGAATTTAATTATACAAATGT
AGTTATGTCTAGTTCTGTGGCAATAACTATTTCTACTATTACATTAGTAGTTTTATTATTCATGTTT
ATGAATAAAGAATGATTAAGTCTGGGTACTATATTGGTACAATCTTTATTTAGCTATTAATGAGTA
GTATGACATTATTTATAGGGTTTGTATCTGTTATAGCTATTTTATTTTTAGCCATTAATTTTATATTT
GCTCCTCATAATCCTTATCAAGAAAAATATAGTATTTTCGAGTGTGGTTTCCATAGTTTTTTAGGG
CAAAATAGAACACAATTCGGTATAAAATTCTTTATTTTTGCTTTAGTTTATTTACTTTTAGATTTAG
AAATATTATTAACTTTCCCTTTCGCTCTTAGTGAGTATGTTAATGGTATTTATGGTCTTTTAGTTAC
TTTAATTTTTATAGCTATAATAACTATAGGATTTATATTTGAATTAGGTAAAAGCGCTCTTAAAATA
GACAGCAGACAAAAATTATATATACCTAAATTGAACGTTAATTACCATACAGAGTATGTTGGAAT
AGGTAAGGTTTCTAAGTAAAGTTATAGAGGCAGAAAACCAAAAAACCTACCAAAGGGTAGCTA
ATGGGAAGCTATTAAAAATAAAAGATGATAACCTATATATAGTATAGTTACTATATATTTACTATA
ACTAGGATTATTATATATATATATATTATCTTATTGTATATTAAGATTATTATTATTATAAGGTATA
ATTAATATAGTATCTTATTGTATAAGAATATAATATATTAACCTATAATTAATTTTATTTTTTAATTT
TTAATTATAATTTTTTTTTATATCTAGATGCTTACACATCTACAGATGTAGAAGAGAACAAAATAT

TGTTGTTATAGTAGTGGATGATATAGAAAAATATTTTTATATTATTTATTTTTGTTAGGTAGCTTTT
GAAGTGTTTGATAGAGAGGATATATGGACGGTAGGAGGGTATTCATTTTAATGAACAGTGGATA
GTTTAAATTAACCTAGTTATAGTTTATGAATTTAAATTATAATTATATTAATGTAGGTTATGATAGA
ATTATGTATTGATCCTTAAGAGTTAAGAGAGATACGCCCACGTATAATACATATTGGTTTAGGGT
TGAGTATATATACTTAAGAGTTAAGAATATATATATACAATATATAATAAATATAGTAATATATTA
ATGGTATGGACTTAACCAGGTTTATATATTATCATTTGATAAACATTAATTATAATTTTTCTATTTA
TTATTATTATTATACTGTGAGATTAATAATTATTAAAATATAATATTACATAGTACGTGATATTTGG
GGATTTTATCTTATTAATTGGCAATTAATGATTCTAATCAAATTTTATTCTCTTTAGTTTAATGGTA
GAACAATGATCTTCTAATTCATTGGTTTTAGTTCGATTCTAAAAAGAGATGAGTAAATAATTTTCT
AGATCAGAAATACTACTTTTAACTACAAAAAGCTTACGCTTTTTAACATTTTTTTGATAAATAACA
ACTATTGTTAATATTTGGCTGTCTATTGGTTTAACTAACAATTACAAAATTTTTTCAATTTTTATAAT
ATAAATTTAAATGAGAATATTAAAAAGTCATTCATTATTAAAATTAGTGAATTCTTACCTTATCGA
TGCGTCACAACCAAGTAACATTAGTTACTTGTGAAATTTTGGTTCATTATTAGCTGTTTGTTTAAT
AGTACAAATTATTACCGGTATTACATTAGCTATGCATTATAGTCCTAGTGTAATGGAAGCTTTTAA
CTCAATAGAGCATATAATGAGAGATGTTAATAACGGGTGATTAGTTCGTTATCTACATAGTAATA
CAGCTTCTGCTTTCTTTTTCTTAGTGTATTTACACATAGGAAGAGGTATATATTACGGATCATATA
GAGCTCCTCGTACTTTAGTTTGAGCTATTGGTACTGTTATATTAATATTAATGATGGCTATCGGTT
TCCTAGGTTATGTTTTACCTTATGGACAGATGTCATTATGAGGTGCTACAGTTATTACTAATCTTA
TTAGTGCTATACCTTGAATAGGGCAAGATATTGTTGAATTCATTTGAGGTGGTTTTTCTGTTAATA
ATGCCACTTTAAACAGATTTTTTGCATTACATTTTGTATTGCCTTTTGTATTAGCTGCTTTAGTTTTA
ATGCACTTAATTGCACTTCATGATACTGCTGGTTCAAGCAATCCTCTTGGTGTTTCAGGTAATTAC
GATAGAATTACATTTGCTCCATATTTTTTATTTAAAGATTTAATTACTATTTTTATATTTATTTTTGTA
TTAAGTGCTTTTGTATTCTTTATGCCTAATGTTTTAGGGGATAGTGATAATTATATTATGGCTAATC
CTATGCAAACTCCTGCTGCTATTGTACCTGAATGATACTTATTACCTTTCTATGCTATTTTAAGATC
TATACCTAATAAATTATTAGGTGTTATAGCGATGTTTAGTGCTATTTTAGCTATTATGTTATTACCT
GTTACAGATTTAGGTAGATCTAGAGGTTTACAATTTAGACCATTTAGTAAAATAGCTTTCTGAGTT
TTTGTTGCTAATTTCTTAGTTTTAATGCAATTAGGTGCTAAACACGTTGAAGATCCATTTATATTAT
TAGGTCAATTAAGTACTGTATTATACTTTAGTTATTTTGTTGCTATATTACCTTTAGCTAGTTACTT
AGATAATAGTTTAACTGATTTATCTAATAAATCTGAATTATTTTTAAATAAAACTAACTAAATATAT
TAAGATTATTATTTAATATATTTTCTATTTAAGATACTATTAATTTAGTATTTTGGGTTTTCAGTTTA
TAATTTATATTATATTATGCATTACCCTCCACCTTGCTTTGTAGTAAGCTAATCTGTTATTTCCTTTA
GTTTAATGGTAGAACAATGATCTTCTAATTCATTGGTTTTAGTTCGAATCTAAAAAGGAAATAAG
AAATATATTCTTATTATTACTTATATAATAATTATTTCTTAAAAATATACATTTTGCATTATAGCCGT
TTAGCTGTATTAAAATGTAAAATGATATAAAATAGAATAAATATTTAAATTATTCCTATGTTATATT
ATCCTATATTGCAACCATTATCAGAAGTTGTATTAATACTTGTACCTGCCTTATTAGCTGTAGCTT
ATGTTACAGTTGCTGAAAGAAAAACTATGGCTAGTATGCAAAGAAGATTAGGTCCTAATGCTGT
AGGTTACTATGGACTATTGCAAGCATTTGCTGATGCCTTAAAACTTTTATTAAAAGAATATGTAG
CTCCTACACAATCTAATATTGTTCTTTTCTTTTTAGGTCCTGTAATAACTTTAATTTTTGCATTATTA
GGTTACGCTGTTATACCCTATGGTCCTGGTTCAGGGATAAGCGACATGAATTTAGGTATATTTTA
CATGTTAGCTGTGTCATCTTTAGCTACATACGGTATTCTATTAGCTGGTTGAAGTGCGAATAGTA
AATACGCTTTTCTAGGTTCTCTTAGAAGTACAGCTCAATTAATTAGTTATGAATTAATATTAAGTT
CAGCTATATTAATAGTAATTATGATAACAGGAAATTTAAATTTAACTGTTTGTACTGAATCTCAAA
GAGCTATTTGATTTATACTACCTTTATTTCCTGTGTTTATAATATTTTTCATAGGATCTATAGCTGA
GACAAATAGAGCTCCTTTTGATTTAGCCGAGGCTAACCTGCTAATCTGGTTTGGTCTGGTTATAT

GTCACAAATTGCTAGGAAACCTTTTTATTTTAAAAACAAAAGACAATTAGCAGGAAACTTAATTT
AACCTAATTAAAAATATTAGATAATTAAACTCTTCATAGACTAAACGTGACAATTTAATATATATA
TATATTTATTTATATATATGATTAAATAAGATATAGTCAATCATCGGTGTGAATCGACTTAAAAAA
AAAAGCACATGGGTTAAACCCATCTCCCCTTATTAGGGGAATCAGAACTTGTTAGTGGGTTCAT
GACAGAGCATGCTGCCGTAGTTTTCGTATTCTTCTTTTTAGCTGAGTACGGTAGTATTGTACTAAT
GTGTATTTTAACTAGTATATTATTTATTGGTGGTTACTTATTATTTGAAATATCCTATGTTTTTACTG
TGGTAAATTATATTTTCTTTGAATTATTCTTTATAGACTGAGTAACATTTGTAGAGGTACAATCTTT
ATACACTGATTTTTTAAATAATTCTATCATTGAAGGATTATTATATGGGTTTAATCTAGGATTAAA
AAGTTCTTTAATGATATTCACATTTATTTGAGCTAGAGCATCCTTCCCTAGAATACGATTTGATCA
ACTAATGGGCTTCTGTTGAACAGTTTTATTACCTATTAATTTTGCAATTATTATATTAGTACCTTGT
GTTTTATATAGTTTTAACTTATTACCTGTAAATATACCATTGTTCTAGCTCACACACCCGCCCGCC
CTACTGCCACAAGGCTACAGTACATATGAGGAGGGGAACTAAAGATCTAGAACTATCCTAGTTA
ATAATTACACTTAATAGTATACTAAATAGACCATCTATCATACTCGAGAATAGTGATAGTGTAAT
TATACACTATTTTAACTGTATTCTACACTATTAGCATATTATTATCTTTATATGATAATAATTTTATA
ACTTAATTTATTAGGTTACATTAACAAAAAATTCGTTAACTTTTTTCACCACTTTTTAATACAAAAT
ACGAATTTTA
>LZJ-32-5
ATAACAATTCTAAAGAACATAAAGAGTTATTAGATAAAAATAATTCACCTATACAGTTAATAAAT
CAACTTAAAGGGTATTTTTTCATAAATCCTTTATTAGCTTTAAGTTTAGCTATTACTATTTTCTCTTT
TGCAGGTATTCCTCCTCTTGTAGGGTTCTTTGCTAAACAGATGGTATTAAGCGCGGCTATTGATC
AAGGTTATATCTTTTTATCTTTAGTTGCAATATTAACTAGTGTTATAGGAGGGGTTTATTATTTAAA
TATAATTAAAGAAATGTTCTTTTATTCACCTGACTATAAATTAAACGAAGAAATTAAAAATAATAC
TATTAATGGTCAAATTATTAATAGAAACAATAAAATATTAAATGTTGAATTTAATTATACAAATGT
AGTTATGTCTAGTTCTGTGGCAATAACTATTTCTACTATTACATTAGTAGTTTTATTATTCATGTTT
ATGAATAAAGAATGATTAAGTCTGGGTACTATATTGGTACAATCTTTATTTAGCTATTAATGAGTA
GTATGACATTATTTATAGGGTTTGTATCTGTTATAGCTATTTTATTTTTAGCCATTAATTTTATATTT
GCTCCTCATAATCCTTATCAAGAAAAATATAGTATTTTCGAGTGTGGTTTCCATAGTTTTTTAGGG
CAAAATAGAACACAATTCGGTATAAAATTCTTTATTTTTGCTTTAGTTTATTTACTTTTAGATTTAG
AAATATTATTAACTTTCCCTTTCGCTCTTAGTGAGTATGTTAATGGTATTTATGGTCTTTTAGTTAC
TTTAATTTTTATAGCTATAATAACTATAGGATTTATATTTGAATTAGGTAAAAGCGCTCTTAAAATA
GACAGCAGACAAAAATTATATATACCTAAATTGAACGTTAATTACCATACAGAGTATGTTGGAAT
AGGTAAGGTTTCTAAGTAAAGTTATAGAGGCAGAAAACCAAAAAACCTACCAAAGGGTAGCTA
ATGGGAAGCTATTAAAAATAAAAGATGATAACCTATATATAGTATAGTTACTATATATTTACTATA
ACTAGGATTATTATATATATATATATATTATCTTATTGTATATTAAGATTATTATTATTATAAGGTATA
ATTAATATAGTATCTTATTGTATAAGAATATAATATATTAACCTATAATTAATTTTATTTTTTTATTT
TTAATTATAATTTTTTTTTATATCTAGATGCTTACACATCTACAGATGTAGAAGAGAACAAAATAT
TGTTGTTATAGTAGTGGATGATATAGAAAAATATTTTTATATTATTTATTTTTGTTAGGTAGCTTTT
GAAGTGTTTGATAGAGAGGATATATGGACGGTAGGAGGGTATTCATTTAAATGAACAGTGGATA
GTTTAAATTAACCTAGTTATAGTTTATGAATTTAAATTATAATTATATTAATGTAGGTTATGATAGA
ATTATGTATTGATCCTTAAGAGTTAAGAGAGATACGCCCACGTATAATACATATTGGTTTAGGGT
TGAGTATATATACTTAAGAGTTAAGAATATATATATACAATATATAATAAATATAGTAATATATTA
ATGGTATGGACTTAACCAGGTTTATATATTATCATTTGATAAACATTAATTATAATTTTTCTATTTA
TTATTATTATTATACTGTGAGATTAATAATTATTAAAATATAATATTACATAGTACGTGATATTTGG
GGATTTTATCTTATTAATTGGCAATTAATGATTCTAATCAAATTTTATTCTCTTTAGTTTAATGGTA

GAACAATGATCTTCTAATTCATTGGTTTTAGTTCGATTCTAAAAAGAGATGAGTAAATAATTTTCT
AGATCAGAAATACTACTTTTAACTACAAAAAGCTTACGCTTTTTAACATTTTTTTGATAAATAACA
ACTATTGTTAATATTTGGCTGTCTATTGGTTTAACTAACAATTACAAAATTTTTTCAATTTTTATAAT
ATAAATTTAAATGAGAATATTAAAAAGTCATTCATTATTAAAATTAGTGAATTCTTACCTTATCGA
TGCGTCACAACCAAGTAACATTAGTTACTTGTGAAATTTTGGTTCATTATTAGCTGTTTGTTTAAT
AGTACAAATTATTACCGGTATTACATTAGCTATGCATTATAGTCCTAGTGTAATGGAAGCTTTTAA
CTCAATAGAGCATATAATGAGAGATGTTAATAACGGGTGATTAGTTCGTTATCTACATAGTAATA
CAGCTTCTGCTTTCTTTTTCTTAGTGTATTTACACATAGGAAGAGGTATATATTACGGATCATATA
GAGCTCCTCGTACTTTAGTTTGAGCTATTGGTACTGTTATATTAATATTAATGATGGCTATCGGTT
TCCTAGGTTATGTTTTACCTTATGGACAGATGTCATTATGAGGTGCTACAGTTATTACTAATCTTA
TTAGTGCTATACCTTGAATAGGGCAAGATATTGTTGAATTCATTTGAGGTGGTTTTTCTGTTAATA
ATGCCACTTTAAACAGATTTTTTGCATTACATTTTGTATTGCCTTTTGTATTAGCTGCTTTAGTTTTA
ATGCACTTAATTGCACTTCATGATACTGCTGGTTCAAGCAATCCTCTTGGTGTTTCAGGTAATTAC
GATAGAATTACATTTGCTCCATATTATTTATTTAAAGATTTAATTACTATTTTTATATTTATTTTTGT
ATTAAGTGCTTTTGTATTCTTTATGCCTAATGTTTTAGGGGATAGTGATAATTATATTATGGCTAAT
CCTATGCAAACTCCTGCTGCTATTGTACCTGAATGATACTTATTACCTTTCTATGCTATTTTAAGAT
CTATACCTAATAAATTATTAGGTGTTATAGCGATGTTTAGTGCTATTTTAGCTATTATGTTATTACC
TGTTACAGATTTAGGTAGATCTAGAGGTTTACAATTTAGACCATTTAGTAAAATAGCTTTCTGAGT
TTTTGTTGCTAATTTCTTAGTTTTAATGCAATTAGGTGCTAAACACGTTGAAGATCCATTTATATTA
TTAGGTCAATTAAGTACTGTATTATACTTTAGTTATTTTGTTGCTATATTACCTTTAGCTAGTTACTT
AGATAATAGTTTAACTGATTTATCTAATAAATCTGAATTATTTTTAAATAAAACTAACTAAATATAT
TAAGATTATTATTTAATATATTTTCTATTTAAGATACTATTAATTTAGTATTTTGGGTTTTCAGTTTA
TAATTTAGATTATATTATGCATTACCCTCCACCTTGCTTTGTAGTAAGCTAATCTGTTATTTCCTTT
AGTTTAATGGTAGAACAATGATCTTCTAATTCATTGGTTTTAGTTCGAATCTAAAAAGGAAATAA
GAAATATATTCTTATTATTACTTATATAATAATTATTTCTTAAAAATATACATTTTGCATTATAGCC
GTTTAGCTGTATTAAAATGTAAAATGATATAAAATAGAATAAATATTTAAATTATTCCTATGTTAT
ATTATCCTATATTGCAACCATTATCAGAAGTTGTATTAATACTTGTACCTGCCTTATTAGCTGTAG
CTTATGTTATAGTTGCTGAAAGAAAAACTATGGCTAGTATGCAAAGAAGATTAGGTCCTAATGCT
GTAGGTTACTATGGACTATTGCAAGCATTTGCTGATGCCTTAAAACTTTTATTTAAAGAATATGTA
GCTCCTACACAATCTAATATTGTTCTTTTCTTTTTAGGTCCTGTAATAAATTTAATTTTTGCATTATT
AGGTTACGCTGTTATACCCTATGGTCCTGGTTCAGGGATAAGCGACATGAATTTAGGTATATTTT
ACATGTTAGCTGTGTCATCTTTAGCTACATACGGTATTCTATTAGCTGGTTGAAGTGCGAATAGT
AAATACGCTTTTCTAGGTTCTCTTAGAAGTACAGCTCAATTAATTAGTTATGAATTAATATTAAGT
TCAGCTATATTAATAGTAATTATGATAACAGGAAATTTAAATTTAACTGTTTGTACTGAATCTCAA
AGAGCTATTTGATTTATACTACCTTTATTTCCTGTGTTTATAATATTTTTCATAGGATCTATAGCTG
AGACAAATAGAGCTCCTTTTGATTTAGCCGAGGCTAACCTGCTAATCTGGTTTGGTCTGGTTATA
TGTCACAAATTGCTAGGAAACCTTTTTATTTTAAAAACAAAAGACAATTAGCAGGAAACTTAATT
TAACCTAATTAAAAATATTAGATAATTAAACTCTTCATAGACTAAACGTGACAATTTAATATATAT
ATATATTTATTTATATATATGATTAAATAAGATATAGTCAATCATCGGTGTGAATCGACTTAAAAA
AAAAAGCACATGGGTTAAACCCATCTCCCCTTATTAGGGGAATCAGAACTTGTTAGTGGGTTCA
TGACAGAGCATGCTGCCGTAGTTTTCGTATTCTTCTTTTTAGCTGAGTACGGTAGTATTGTACTAA
TGTGTATTTTAACTAGTATATTATTTATTGGTGGTTACTTATTATTTGAAATATCCTATGTTTTTACT
GTGGTAAATTATATTTTCTTTGAATTATTCTTTATAGACTGAGTAACATTTGTAGAGGTACAATCTT
TATACACTGATTTTTTAAATAATTCTATCATTGAAGGATTATTATATGGGTTTAATCTAGGATTAAA

AAGTTCTTTAATGATATTCACATTTATTTGAGCTAGAGCATCCTTCCCTAGAATACGATTTGATCA
ACTAATGGGCTTCTGTTGAACAGTTTTATTACCTATTAATTTTGCAATTATTATATTAGTACCTTGT
GTTTTATATAGTTTTAACTTATTACCTGTAAATATACCATTGTTCTAGCTCACACACCCGCCCGCC
CTACTGCCACAAGGCTACAGTACATATGAGGAGGGGAACTAAAGATCTAGAACTATCCTAGTTA
ATAATTACACTTAATAGTATACTAAATAGACCATCTATCATACTCGAGAATAGTGATAGTGTAAT
TATACACTATTTTAACTGTATTCTACAATATTAGCATATTATTATCTTTATATGATAATAATTTTATA
ACTTAATTTATTAGGTTACATTAACTAAAAATTAGTTAACTTTTTTCACCACTTTTTAATACAAAAT
ACGAATTTTA
>MT-14-2
ATAACAATTCTAAAGAACATAAAGAGTTATTAGATAAAAATAATTCACCAATACAGTTAATAAAT
CAACTTAAAGGGTATTTTTTCATAAATCCTTTATTAGCTTTAAGTTTAGCTATCACTATCTTCTCTTT
TGCAGGGATTCCTCCTCTTGTAGGGTTCTTTGCTAAACAGATGGTATTAAGCGCGGCTATTGATC
AAGGTTATATCTTTTTATCTTTAGTTGCAATATTAACTAGTGTAATAGGAGGAGTTTATTATTTAAA
TATAATAAAAGAAATGTTCTTCTATTCACCTGACTATAAGTTAAACGAAGAAATTAAAAATAATA
CTATTAATGGTCAAATTATTAATAGAAACAATAAAACATTAAATGTAGAATTTAATTATACAAATG
TCGTTATGTCTAGTTCTGTGGCAGTTACTATTTCTACTATAACATTAGTAGTTTTATTATTCATGTTT
ATGAATAAAGAATGATTAAGTCTGGGTACTATATTGGTACAATCTTTATTTAACTATTAATGAGTA
GTATGACATTATTTATAGGGTTTGTATCAGTAATAGCTATATTATTTTTAGCTATTAATTTTATATTT
GCTCCTCATAATCCTTATCAAGAAAAATATAGTATTTTCGAGTGTGGTTTCCATAGTTTTTTAGGG
CAAAATAGAACACAATTCGGTATAAAATTCTTTATATTTGCTTTAGTTTATTTACTTTTAGATTTAG
AAATATTATTAACTTTTCCTTTCGCTGTTAGTGAGTATGTTAATGGTATTTATGGTCTTTTAGTTAC
TTTAATTTTTATAGCTATAATAACTATAGGATTTATATTTGAATTAGGTAAAAGCGCTCTTAAAATA
GATAGCAGACAAAAATTATATATACCTAAAGTAAACGTGAAATACCATACTGAGTACGTTAGAA
TCGGTAAGGTTTCTAAGTAAAGTTATAGAGGCAGAAAACCAAAAAACCTACCAAAGGGTAGCT
AATGGGAAGCTATTAAAAATAAAAGATGATAACCTATATATAGTATAGTTACTATATATTTACTAT
AACTAGGATTATTATATATATATATATTATCTTATTGTATATTAAGATTATTATTATTATAAGGTAT
AATTAATATAGTATCTTATTGTATAAGAATATAATATATTAACCTATAATTAATTTTATTTTTTAATT
TTTAATTATAATTTTTTTTTATATCTAGATGCTTACACATCTACAGATGTAGAAGAGAACAAAATA
TTGTTGTTATAGTAGTGGATGATATAGAAAAATATTTTTATATTATTTATTTTTGTTAGGTAGCTTTT
GAAGTGTTTGATAGAGAGGATATATGGACGGTAGGAGGGTATTCATTTTAATGAACAGTGGATA
GTTTAAATTAACCTAGTTATAGTTTATGAATTTAAATTATAATTATATTAATGTAGGTTATGATAGA
ATTATGTATTGATCCTTAAGAGTTAAGAGAGATACGCCCACGTATAATACATATTGGTTTAGGGT
TGAGTATATATACTTAAGAGTTAAGAATATATATATACAATATATAATAAATATAGTAATATATTA
ATGGTATGGACTTAACCAGGTTTATATATTATCATTTGATAAACATTAATTATAATTTTTCTATTTA
TTATTATTATTATACTGTGAGATTAATAATTATTAAAATATAATATTACATAGTACGTGATATTTGG
GGATTTTATCTTATTAATTGGCCACTAATGAGTCTAATCAAAATTAATTCTCTTTAGTTTAATGGTA
GAACAATGATCTTCTAATTCATTGGTTTTAGTTCGATTCTAAAAAGAGATGAGTAAATAATATTCT
AGCTGAAAAATACTACTTTTAACTACAAAAAGCTTACGCTTTTTAACATTTTTTTGATAAATAACA
ACTATTGTTAATATTTGACTGTTTAGCGGTATAACTTACAATTACAAAATTTAATCAAGTTTTATAA
TCTAAATTTAAATGAGAATATTAAAAAGTCATTCATTATTAAAATTAGTGAATTCTTATCTTATCG
ATGCGTCACAACCAAGTAATATTAGTTACTTATGAAATTTTGGTTCATTATTAGCTGTTTGTTTAAT
AGTACAAATTATTACAGGTATTACATTAGCTATGCATTATAGTCCTAGTGTAATGGAAGCCTTTA
ATTCAATAGAACATATAATGAGAGATGTTAATAACGGATGATTAGTTCGTTATCTACATAGTAAT
ACAGCCTCAGCATTCTTTTTCTTAGTGTATTTACACATAGGTAGAGGTATATATTATGGATCATAT

AGAGCTCCTCGTACTTTAGTATGAGCTATTGGTACTGTAATACTTATATTAATGATGGCCATCGGT
TTCTTAGGTTATGTTTTACCTTATGGACAAATGTCATTATGAGGTGCTACAGTTATTACTAATCTTA
TTAGTGCTATCCCTTGAATAGGACAAGATATTGTTGAATTTATTTGAGGAGGTTTTTCTGTTAATA
ATGCCACTTTAAACAGATTTTTTGCATTACATTTTGTATTACCTTTTGTATTAGCTGCTTTAGTATTA
ATGCACTTAATAGCACTTCACGATACAGCTGGTTCTAGTAACCCTCTAGGTGTTTCAGGTAATTA
TGATAGAATTACATTTGCTCCATATTATTTATTTAAAGATTTAATTACTATTTTTATATTTATATTTG
TATTAAGTGCATTTGTATTCTTTATGCCTAATGTTTTAGGGGATAGTGATAATTATATTATGGCTAA
TCCTATGCAAACTCCTGCTGCTATTGTGCCTGAATGATATTTATTACCTTTCTATGCTATTTTAAGA
TCTATACCTAATAAATTATTAGGAGTTATAGCTATGTTTAGTGCAATTTTAGCTATAATGTTATTAC
CTGTTACAGATTTAGGTAGATCTAGAGGTTTACAATTTAGACCATTTAGTAAAATAGCTTTCTGAG
TTTTTGTAGCTAATTTCTTAGTATTAATGCAATTAGGTGCTAAACACGTAGAAGATCCATTTATAT
TATTAGGTCAATTAAGTACTGTTTTATACTTTAGTTACTTTGTAGCTATATTACCTTTAGCTAGTTA
CTTAGATAATAGTTTAACAGATTTATCTAATAAACCTGAATTATTATTAAATAAAACTAACTAAAT
TTATTAAGATTATTATTTAATATATTTTCTATTTAAGATACTATTAATTTAGTATTTTGGGTTTTCAG
TTTATAATTTATATTATATTATGCATTACCCTCCACCTTGCTTTGTAGTAAGGTAATCCATTATTTC
CTTTAGTTTAATGGTAGAACAATGATCTTCTAATTCATTGGTTTTAGTTCGATTCTAAAAAGGAAA
TAAGAAATAATTGATTATTATTACTTATATAATAATTATTTCTTAAAAATATACATTTTGCATTATA
GCCGTTTAGCTGTATTAAAATGTAAAATGATATAAAATAGAATCAATATTTAAATTATTCCTATGT
TATATTACCCTATTTTACAGCCATTTTCAGAAGTTATATTAATACTTGTACCTGCCTTGTTAGCTGT
AGCTTATGTTACAGTAGCTGAAAGAAAAACTATGGCTAGTATGCAAAGAAGATTAGGTCCTAAC
GCTGTAGGTTATTATGGACTACTACAAGCATTTGCTGATGCCTTAAAACTTTTATTAAAAGAATAT
GTAGCTCCTACACAATCTAATATAGTTCTTTTCTTTTTAGGTCCAGTTATTACTTTAATATTTGCAT
TACTAGGTTATGCTGTTATACCATATGGACCTGGTTCAGGATTAAGCGATATGAATTTAGGTATC
TTCTATATGTTAGCTGTGTCATCTTTAGCTACATACGGTATTTTATTAGCCGGTTGAAGTGCTAAC
AGTAAATATGCATTTCTAGGTTCTCTTAGAAGTACAGCTCAATTAATTAGTTATGAATTAATATTA
AGTTCAGCCATATTAATAGTAATTATGATAACAGGAAATTTAAATTTAACTGTATGTACTGAATCT
CAAAGAGCTGTTTGATTAATATTGCCATTATTTCCTGTATTTATAATATTTTTTATAGGATCTATAG
CTGAAACAAATAGAGCTCCTTTTGATTTAGCCGAGGCTAACCTAATATTCTGGTTTGGTCTGGTT
ATATGTCACAAATTGCTGGGAAACCTTTTTATTTTAAAAACAAAAGACAATTAGCAGGAAACTTA
ATTTAACCTAATTAAAAATATTAGATAATTAAACTCTTCATAGACTAAACGTGACAATTTAATATA
TATATATATTTATTTATATATATGATTAAATAAGATATAGTCAATCATCGGTGTGAATCGACTTAA
AAAAAAAAGCACATGGGTTAAACCCATCTCCCCTTATTAGGGGAATCAGAACTTGTTAGTGGGT
TTATGACAGAACATGCTGCCGTAGTTTTCGTATTCTTTTTTTTAGCAGAATATGGTAGTATTGTATT
AATGTGTATTTTAACTAGTATTTTATTTATAGGTGGTTACTTATTATTTGAAATATCATATGTTTTTA
CTGTTGTAAATTATATTTTCTTTGAATTATTTTTTATAGACTGAGTAACATTTATAGAAATACAATC
TTTATATACGGACTTTTTAAATAATTCTGTTATTGAAGGATTATTATATGGGTTTAATTTAGGATTA
AAAAGCTCTTTAATGATATTTACATTTATTTGAGCTAGAGCTTCTTTCCCTAGAATACGATTTGAT
CAATTAATGGCCTTCTGTTGAACAGTTTTATTACCTATTAATTTTGCTATAATTATTTTAGTACCTT
GTGTATTATATAGTTTTAATTTATTACCAATTAACATCCCATTGTTCTAGCTCACACACCCGCCCG
CCCTACTGCCACAAGGCTACAGTACATATGAGGAGGGGAACTAAAGATCTAGAACTATCCTAGT
TAATAATTACACTTAATAGTATACTAAATAGACCATCTATCATACTCGAGAATAGTGATAGTGTA
ATTATACACTATTTTAACTGTATTCTACACTATTAGCATATTATTATCTTTATATGATAATAATTTTA
TAACTTAATTTATTAGGTTACATTAACAAAAAATTCGTTAACTTTTTTCACCACTTTTTAATACAAA
ATACGAATTTTA

>R01-1
ATAACAATTCTAAAGAACATAAAGAGTTATTAGATAAAAATAATTCACCTATACAGTTAATAAAT
CAACTTAAAGGGTATTTTTTCATAAATCCTTTATTAGCTTTAAGTTTAGCTATTACTATTTTCTCTTT
TGCAGGTATTCCTCCTCTTGTAGGGTTCTTTGCTAAACAGATGGTATTAAGCGCGGCTATTGATC
AAGGTTATATCTTTTTATCTTTAGTTGCAATATTAACTAGTGTTATAGGAGGGGTTTATTATTTAAA
TATAATTAAAGAAATGTTCTTTTATTCACCTGACTATAAATTAAACGAAGAAATTAAAAATAATAC
TATTAATGGTCAAATTATTAATAGAAACAATAAAATATTAAATGTTGAATTTAATTATACAAATGT
AGTTATGTCTAGTTCTGTGGCAATAACTATTTCTACTATTACATTAGTAGTTTTATTATTCATGTTT
ATGAATAAAGAATGATTAAGTCTGGGTACTATATTGGTACAATCTTTATTTAGCTATTAATGAGTA
GTATGACATTATTTATAGGGTTTGTATCTGTTATAGCTATTTTATTTTTAGCCATTAATTTTATATTT
GCTCCTCATAATCCTTATCAAGAAAAATATAGTATTTTCGAGTGTGGTTTCCATAGTTTTTTAGGG
CAAAATAGAACACAATTCGGTATAAAATTCTTTATTTTTGCTTTAGTTTATTTACTTTTAGATTTAG
AAATATTATTAACTTTCCCTTTCGCTCTTAGTGAGTATGTTAATGGTATTTATGGTCTTTTAGTTAC
TTTAATTTTTATAGCTATAATAACTATAGGATTTATATTTGAATTAGGTAAAAGCGCTCTTAAAATA
GACAGCAGACAAAAATTATATATACCTAAATTGAACGTTAATTACCATACAGAGTATGTTGGAAT
AGGTAAGGTTTCTAAGTAAAGTTATAGAGGCAGAAAACCAAAAAACCTACCAAAGGGTAGCTA
ATGGGAAGCTATTAAAAATAAAAGATGATAACCTATATATAGTATAGTTACTATATATTTACTATA
ACTAGGATTATTATATATATATATATTATCTTATTGTATATTAAGATTATTATTATTATAAGGTATA
ATTAATATAGTATCTTATTGTATAAGAATATAATATATTAACCTATAATTAATTTTATTTTTTAATTT
TTAATTATAATTTTTTTTTATATCTAGATGCTTACACATCTACAGATGTAGAAGAGAACAAAATAT
TGTTGTTATAGTAGTGGATGATATAGAAAAATATTTTTATATTATTTATTTTTGTTAGGTAGCTTTT
GAAGTGTTTGATAGAGAGGATATATGGACGGTAGGAGGGTATTCATTTTAATGAACAGTGGATA
GTTTAAATTAACCTAGTTATAGTTTATGAATTTAAATTATAATTATATTAATGTAGGTTATGATAGA
ATTATGTATTGATCCTTAAGAGTTAAGAGAGATACGCCCACGTATAATACATATTGGTTTAGGGT
TGAGTATATATACTTAAGAGTTAAGAATATATATATACAATATATAATAAATATAGTAATATATTA
ATGGTATGGACTTAACCAGGTTTATATATTATCATTTGATAAACATTAATTATAATTTTTCTATTTA
TTATTATTATTATACTGTGAGATTAATAATTATTAAAATATAATATTACATAGTACGTGATATTTGG
GGATTTTATCTTATTAATTGGCAATTAATGATTCTAATCAAATTTTATTCTCTTTAGTTTAATGGTA
GAACAATGATCTTCTAATTCATTGGTTTTAGTTCGATTCTAAAAAGAGATGAGTAAATAATTTTCT
AGATCAGAAATACTACTTTTAACTACAAAAAGCTTACGCTTTTTAACATTTTTTTGATAAATAACA
ACTATTGTTAATATTTGGCTGTCTATTGGTTTAACTAACAATTACAAAATTTTTTCAATTTTTATAAT
ATAAATTTAAATGAGAATATTAAAAAGTCATTCATTATTAAAATTAGTGAATTCTTACCTTATCGA
TGCGTCACAACCAAGTAACATTAGTTACTTGTGAAATTTTGGTTCATTATTAGCTGTTTGTTTAAT
AGTACAAATTATTACCGGTATTACATTAGCTATGCATTATAGTCCTAGTGTAATGGAAGCTTTTAA
CTCAATAGAGCATATAATGAGAGATGTTAATAACGGGTGATTAGTTCGTTATCTACATAGTAATA
CAGCTTCTGCTTTCTTTTTCTTAGTGTATTTACACATAGGAAGAGGTATATATTACGGATCATATA
GAGCTCCTCGTACTTTAGTTTGAGCTATTGGTACTGTTATATTAATATTAATGATGGCTATCGGTT
TCCTAGGTTATGTTTTACCTTATGGACAGATGTCATTATGAGGTGCTACAGTTATTACTAATCTTA
TTAGTGCTATACCTTGAATAGGGCAAGATATTGTTGAATTCATTTGAGGTGGTTTTTCTGTTAATA
ATGCCACTTTAAACAGATTTTTTGCATTACATTTTGTATTGCCTTTTGTATTAGCTGCTTTAGTTTTA
ATGCACTTAATTGCACTTCATGATACTGCTGGTTCAAGCAATCCTCTTGGTGTTTCAGGTAATTAC
GATAGAATTACATTTGCTCCATATTTTTTATTTAAAGATTTAATTACTATTTTTATATTTATTTTTGTA
TTAAGTGCTTTTGTATTCTTTATGCCTAATGTTTTAGGGGATAGTGATAATTATATTATGGCTAATC
CTATGCAAACTCCTGCTGCTATTGTACCTGAATGATACTTATTACCTTTCTATGCTATTTTAAGATC

TATACCTAATAAATTATTAGGTGTTATAGCGATGTTTAGTGCTATTTTAGCTATTATGTTATTACCT
GTTACAGATTTAGGTAGATCTAGAGGTTTACAATTTAGACCATTTAGTAAAATAGCTTTCTGAGTT
TTTGTTGCTAATTTCTTAGTTTTAATGCAATTAGGTGCTAAACACGTTGAAGATCCATTTATATTAT
TAGGTCAATTAAGTACTGTATTATACTTTAGTTATTTTGTTGCTATATTACCTTTAGCTAGTTACTT
AGATAATAGTTTAACTGATTTATCTAATAAATCTGAATTATTTTTAAATAAAACTAACTAAATATAT
TAAGATTATTATTTAATATATTTTCTATTTAAGATACTATTAATTTAGTATTTTGGGTTTTCAGTTTA
TAATTTATATTATATTATGCATTACCCTCCACCTTGCTTTGTAGTAAGCTAATCTGTTATTTCCTTTA
GTTTAATGGTAGAACAATGATCTTCTAATTCATTGGTTTTAGTTCGAATCTAAAAAGGAAATAAG
AAATATATTCTTATTATTACTTATATAATAATTATTTCTTAAAAATATACATTTTGCATTATAGCCGT
TTAGCTGTATTAAAATGTAAAATGATATAAAATAGAATAAATATTTAAATTATTCCTATGTTATATT
ATCCTATATTGCAACCATTATCAGAAGTTGTATTAATACTTGTACCTGCCTTATTAGCTGTAGCTT
ATGTTACAGTTGCTGAAAGAAAAACTATGGCTAGTATGCAAAGAAGATTAGGTCCTAATGCTGT
AGGTTACTATGGACTATTGCAAGCATTTGCTGATGCCTTAAAACTTTTATTAAAAGAATATGTAG
CTCCTACACAATCTAATATTGTTCTTTTCTTTTTAGGTCCTGTAATAACTTTAATTTTTGCATTATTA
GGTTACGCTGTTATACCCTATGGTCCTGGTTCAGGGATAAGCGACATGAATTTAGGTATATTTTA
CATGTTAGCTGTGTCATCTTTAGCTACATACGGTATTCTATTAGCTGGTTGAAGTGCGAATAGTA
AATACGCTTTTCTAGGTTCTCTTAGAAGTACAGCTCAATTAATTAGTTATGAATTAATATTAAGTT
CAGCTATATTAATAGTAATTATGATAACAGGAAATTTAAATTTAACTGTTTGTACTGAATCTCAAA
GAGCTATTTGATTTATACTACCTTTATTTCCTGTGTTTATAATATTTTTCATAGGATCTATAGCTGA
GACAAATAGAGCTCCTTTTGATTTAGCCGAGGCTAACCTGCTAATCTGGTTTGGTCTGGTTATAT
GTCACAAATTGCTAGGAAACCTTTTTATTTTAAAAACAAAAGACAATTAGCAGGAAACTTAATTT
AACCTAATTAAAAATATTAGATAATTAAACTCTTCATAGACTAAACGTGACAATTTAATATATATA
TATATTTATTTATATATATGATTAAATAAGATATAGTCAATCATCGGTGTGAATCGACTTAAAAAA
AAAAGCACATGGGTTAAACCCATCTCCCCTTATTAGGGGAATCAGAACTTGTTAGTGGGTTCAT
GACAGAGCATGCTGCCGTAGTTTTCGTATTCTTCTTTTTAGCTGAGTACGGTAGTATTGTACTAAT
GTGTATTTTAACTAGTATATTATTTATTGGTGGTTACTTATTATTTGAAATATCCTATGTTTTTACTG
TGGTAAATTATATTTTCTTTGAATTATTCTTTATAGACTGAGTAACATTTGTAGAGGTACAATCTTT
ATACACTGATTTTTTAAATAATTCTATCATTGAAGGATTATTATATGGGTTTAATCTAGGATTAAA
AAGTTCTTTAATGATATTCACATTTATTTGAGCTAGAGCATCCTTCCCTAGAATACGATTTGATCA
ACTAATGGGCTTCTGTTGAACAGTTTTATTACCTATTAATTTTGCAATTATTATATTAGTACCTTGT
GTTTTATATAGTTTTAACTTATTACCTGTAAATATACCATTGTTCTAGCTCACACACCCGCCCGCC
CTACTGCCACAAGGCTACAGTACATATGAGGAGGGGAACTAAAGATCTAGAACTATCCTAGTTA
ATAATTACACTTAATAGTATACTAAATAGACCATCTATCATACTCGAGAATAGTGATAGTGTAAT
TATACACTATTTTAACTGTATTCTACACTATTAGCATATTATTATCTTTATATGATAATAATTTTATA
ACTTAATTTATTAGGTTACATTAACAAAAAATTCGTTAACTTTTTTCACCACTTTTTAATACAAAAT
ACGAATTTTA
>S1
ATAACAATTCTAAAGAACATAAAGAGTTATTAGATAAAAATAATTCACCTATACAGTTAATAAAT
CAACTTAAAGGGTATTTTTTCATAAATCCTTTATTAGCTTTAAGTTTAGCTATTACTATTTTCTCTTT
TGCAGGTATTCCTCCTCTTGTAGGGTTCTTTGCTAAACAGATGGTATTAAGCGCGGCTATTGATC
AAGGTTATATCTTTTTATCTTTAGTTGCAATATTAACTAGTGTTATAGGAGGGGTTTATTATTTAAA
TATAATTAAAGAAATGTTCTTTTATTCACCTGACTATAAATTAAACGAAGAAATTAAAAATAATAC
TATTAATGGTCAAATTATTAATAGAAACAATAAAATATTAAATGTTGAATTTAATTATACAAATGT
AGTTATGTCTAGTTCTGTGGCAATAACTATTTCTACTATTACATTAGTAGTTTTATTATTCATGTTT

ATGAATAAAGAATGATTAAGTCTGGGTACTATATTGGTACAATCTTTATTTAGCTATTAATGAGTA
GTATGACATTATTTATAGGGTTTGTATCTGTTATAGCTATTTTATTTTTAGCCATTAATTTTATATTT
GCTCCTCATAATCCTTATCAAGAAAAATATAGTATTTTCGAGTGTGGTTTCCATAGTTTTTTAGGG
CAAAATAGAACACAATTCGGTATAAAATTCTTTATTTTTGCTTTAGTTTATTTACTTTTAGATTTAG
AAATATTATTAACTTTCCCTTTCGCTCTTAGTGAGTATGTTAATGGTATTTATGGTCTTTTAGTTAC
TTTAATTTTTATAGCTATAATAACTATAGGATTTATATTTGAATTAGGTAAAAGCGCTCTTAAAATA
GACAGCAGACAAAAATTATATATACCTAAATTGAACGTTAATTACCATACAGAGTATGTTGGAAT
AGGTAAGGTTTCTAAGTAAAGTTATAGAGGCAGAAAACCAAAAAACCTACCAAAGGGTAGCTA
ATGGGAAGCTATTAAAAATAAAAGATGATAACCTATATATAGTATAGTTACTATATATTTACTATA
ACTAGGATTATTATATATATATATATTATCTTATTGTATATTAAGATTATTATTATTATAAGGTATA
ATTAATATAGTATCTTATTGTATAAGAATATAATATATTAACCTATAATTAATTTTATTTTTTAATTT
TTAATTATAATTTTTTTTTATATCTAGATGCTTACACATCTACAGATGTAGAAGAGAACAAAATAT
TGTTGTTATAGTAGTGGATGATATAGAAAAATATTTTTATATTATTTATTTTTGTTAGGTAGCTTTT
GAAGTGTTTGATAGAGAGGATATATGGACGGTAGGAGGGTATTCATTTTAATGAACAGTGGATA
GTTTAAATTAACCTAGTTATAGTTTATGAATTTAAATTATAATTATATTAATGTAGGTTATGATAGA
ATTATGTATTGATCCTTAAGAGTTAAGAGAGATACGCCCACGTATAATACATATTGGTTTAGGGT
TGAGTATATATACTTAAGAGTTAAGAATATATATATACAATATATAATAAATATAGTAATATATTA
ATGGTATGGACTTAACCAGGTTTATATATTATCATTTGATAAACATTAATTATAATTTTTCTATTTA
TTATTATTATTATACTGTGAGATTAATAATTATTAAAATATAATATTACATAGTACGTGATATTTGG
GGATTTTATCTTATTAATTGGCAATTAATGATTCTAATCAAATTTTATTCTCTTTAGTTTAATGGTA
GAACAATGATCTTCTAATTCATTGGTTTTAGTTCGATTCTAAAAAGAGATGAGTAAATAATTTTCT
AGATCAGAAATACTACTTTTAACTACAAAAAGCTTACGCTTTTTAACATTTTTTTGATAAATAACA
ACTATTGTTAATATTTGGCTGTCTATTGGTTTAACTAACAATTACAAAATTTTTTCAATTTTTATAAT
ATAAATTTAAATGAGAATATTAAAAAGTCATTCATTATTAAAATTAGTGAATTCTTACCTTATCGA
TGCGTCACAACCAAGTAACATTAGTTACTTGTGAAATTTTGGTTCATTATTAGCTGTTTGTTTAAT
AGTACAAATTATTACCGGTATTACATTAGCTATGCATTATAGTCCTAGTGTAATGGAAGCTTTTAA
CTCAATAGAGCATATAATGAGAGATGTTAATAACGGGTGATTAGTTCGTTATCTACATAGTAATA
CAGCTTCTGCTTTCTTTTTCTTAGTGTATTTACACATAGGAAGAGGTATATATTACGGATCATATA
GAGCTCCTCGTACTTTAGTTTGAGCTATTGGTACTGTTATATTAATATTAATGATGGCTATCGGTT
TCCTAGGTTATGTTTTACCTTATGGACAGATGTCATTATGAGGTGCTACAGTTATTACTAATCTTA
TTAGTGCTATACCTTGAATAGGGCAAGATATTGTTGAATTCATTTGAGGTGGTTTTTCTGTTAATA
ATGCCACTTTAAACAGATTTTTTGCATTACATTTTGTATTGCCTTTTGTATTAGCTGCTTTAGTTTTA
ATGCACTTAATTGCACTTCATGATACTGCTGGTTCAAGCAATCCTCTTGGTGTTTCAGGTAATTAC
GATAGAATTACATTTGCTCCATATTATTTATTTAAAGATTTAATTACTATTTTTATATTTATTTTTGT
ATTAAGTGCTTTTGTATTCTTTATGCCTAATGTTTTAGGGGATAGTGATAATTATATTATGGCTAAT
CCTATGCAAACTCCTGCTGCTATTGTACCTGAATGATACTTATTACCTTTCTATGCTATTTTAAGAT
CTATACCTAATAAATTATTAGGTGTTATAGCGATGTTTAGTGCTATTTTAGCTATTATGTTATTACC
TGTTACAGATTTAGGTAGATCTAGAGGTTTACAATTTAGACCATTTAGTAAAATAGCTTTCTGAGT
TTTTGTTGCTAATTTCTTAGTTTTAATGCAATTAGGTGCTAAACACGTTGAAGATCCATTTATATTA
TTAGGTCAATTAAGTACTGTATTATACTTTAGTTATTTTGTTGCTATATTACCTTTAGCTAGTTACTT
AGATAATAGTTTAACTGATTTATCTAATAAATCTGAATTATTTTTAAATAAAACTAACTAAATATAT
TAAGATTATTATTTAATATATTTTCTATTTAAGATACTATTAATTTAGTATTTTGGGTTTTCAGTTTA
TAATTTATATTATATTATGCATTACCCTCCACCTTGCTTTGTAGTAAGCTAATCTGTTATTTCCTTTA
GTTTAATGGTAGAACAATGATCTTCTAATTCATTGGTTTTAGTTCGAATCTAAAAAGGAAATAAG

AAATATATTCTTATTATTACTTATATAATAATTATTTCTTAAAAATATACATTTTGCATTATAGCCGT
TTAGCTGTATTAAAATGTAAAATGATATAAAATAGAATAAATATTTAAATTATTCCTATGTTATATT
ATCCTATATTGCAACCATTATCAGAAGTTGTATTAATACTTGTACCTGCCTTATTAGCTGTAGCTT
ATGTTACAGTTGCTGAAAGAAAAACTATGGCTAGTATGCAAAGAAGATTAGGTCCTAATGCTGT
AGGTTACTATGGACTATTGCAAGCATTTGCTGATGCCTTAAAACTTTTATTAAAAGAATATGTAG
CTCCTACACAATCTAATATTGTTCTTTTCTTTTTAGGTCCTGTAATAACTTTAATTTTTGCATTATTA
GGTTACGCTGTTATACCCTATGGTCCTGGTTCAGGGATAAGCGACATGAATTTAGGTATATTTTA
CATGTTAGCTGTGTCATCTTTAGCTACATACGGTATTCTATTAGCTGGTTGAAGTGCGAATAGTA
AATACGCTTTTCTAGGTTCTCTTAGAAGTACAGCTCAATTAATTAGTTATGAATTAATATTAAGTT
CAGCTATATTAATAGTAATTATGATAACAGGAAATTTAAATTTAACTGTTTGTACTGAATCTCAAA
GAGCTATTTGATTTATACTACCTTTATTTCCTGTGTTTATAATATTTTTCATAGGATCTATAGCTGA
GACAAATAGAGCTCCTTTTGATTTAGCCGAGGCTAACCTGCTAATCTGGTTTGGTCTGGTTATAT
GTCACAAATTGCTAGGAAACCTTTTTATTTTAAAAACAAAAGACAATTAGCAGGAAACTTAATTT
AACCTAATTAAAAATATTAGATAATTAAACTCTTCATAGACTAAACGTGACAATTTAATATATATA
TATATTTATTTATATATATGATTAAATAAGATATAGTCAATCATCGGTGTGAATCGACTTAAAAAA
AAAAGCACATGGGTTAAACCCATCTCCCCTTATTAGGGGAATCAGAACTTGTTAGTGGGTTCAT
GACAGAGCATGCTGCCGTAGTTTTCGTATTCTTCTTTTTAGCTGAGTACGGTAGTATTGTACTAAT
GTGTATTTTAACTAGTATATTATTTATTGGTGGTTACTTATTATTTGAAATATCCTATGTTTTTACTG
TGGTAAATTATATTTTCTTTGAATTATTCTTTATAGACTGAGTAACATTTGTAGAGGTACAATCTTT
ATACACTGATTTTTTAAATAATTCTATCATTGAAGGATTATTATATGGGTTTAATCTAGGATTAAA
AAGTTCTTTAATGATATTCACATTTATTTGAGCTAGAGCATCCTTCCCTAGAATACGATTTGATCA
ACTAATGGGCTTCTGTTGAACAGTTTTATTACCTATTAATTTTGCAATTATTATATTAGTACCTTGT
GTTTTATATAGTTTTAACTTATTACCTGTAAATATACCATTGTTCTAGCTCACACACCCGCCCGCC
CTACTGCCACAAGGCTACAGTACATATGAGGAGGGGAACTAAAGATCTAGAACTATCCTAGTTA
ATAATTACACTTAATAGTATACTAAATAGACCATCTATCATACTCGAGAATAGTGATAGTGTAAT
TATACACTATTTTAACTGTATTCTACACTATTAGCATATTATTATCTTTATATGATAATAATTTTATA
ACTTAATTTATTAGGTTACATTAACAAAAAATTCGTTAACTTTTTTCACCACTTTTTAATACAAAAT
ACGAATTTTA
>Su-1
ATAACAATTCTAAAGAACATAAAGAGTTATTAGATAAAAATAATTCACCTATACAGTTAATAAAT
CAACTTAAAGGGTATTTTTTCATAAATCCTTTATTAGCTTTAAGTTTAGCTATTACTATTTTCTCTTT
TGCAGGTATTCCTCCTCTTGTAGGGTTCTTTGCTAAACAGATGGTATTAAGCGCGGCTATTGATC
AAGGTTATATCTTTTTATCTTTAGTTGCAATATTAACTAGTGTTATAGGAGGGGTTTATTATTTAAA
TATAATTAAAGAAATGTTCTTTTATTCACCTGACTATAAATTAAACGAAGAAATTAAAAATAATAC
TATTAATGGTCAAATTATTAATAGAAACAATAAAATATTAAATGTTGAATTTAATTATACAAATGT
AGTTATGTCTAGTTCTGTGGCAATAACTATTTCTACTATTACATTAGTAGTTTTATTATTCATGTTT
ATGAATAAAGAATGATTAAGTCTGGGTACTATATTGGTACAATCTTTATTTAGCTATTAATGAGTA
GTATGACATTATTTATAGGGTTTGTATCTGTTATAGCTATTTTATTTTTAGCCATTAATTTTATATTT
GCTCCTCATAATCCTTATCAAGAAAAATATAGTATTTTCGAGTGTGGTTTCCATAGTTTTTTAGGG
CAAAATAGAACACAATTCGGTATAAAATTCTTTATTTTTGCTTTAGTTTATTTACTTTTAGATTTAG
AAATATTATTAACTTTCCCTTTCGCTCTTAGTGAGTATGTTAATGGTATTTATGGTCTTTTAGTTAC
TTTAATTTTTATAGCTATAATAACTATAGGATTTATATTTGAATTAGGTAAAAGCGCTCTTAAAATA
GACAGCAGACAAAAATTATATATACCTAAATTGAACGTTAATTACCATACAGAGTATGTTGGAAT
AGGTAAGGTTTCTAAGTAAAGTTATAGAGGCAGAAAACCAAAAAACCTACCAAAGGGTAGCTA

ATGGGAAGCTATTAAAAATAAAAGATGATAACCTATATATAGTATAGTTACTATATATTTACTATA
ACTAGGATTATTATATATATATATATTATCTTATTGTATATTAAGATTATTATTATTATAAGGTATA
ATTAATATAGTATCTTATTGTATAAGAATATAATATATTAACCTATAATTAATTTTATTTTTTAATTT
TTAATTATAATTTTTTTTTATATCTAGATGCTTACACATCTACAGATGTAGAAGAGAACAAAATAT
TGTTGTTATAGTAGTGGATGATATAGAAAAATATTTTTATATTATTTATTTTTGTTAGGTAGCTTTT
GAAGTGTTTGATAGAGAGGATATATGGACGGTAGGAGGGTATTCATTTTAATGAACAGTGGATA
GTTTAAATTAACCTAGTTATAGTTTATGAATTTAAATTATAATTATATTAATGTAGGTTATGATAGA
ATTATGTATTGATCCTTAAGAGTTAAGAGAGATACGCCCACGTATAATACATATTGGTTTAGGGT
TGAGTATATATACTTAAGAGTTAAGAATATATATATACAATATATAATAAATATAGTAATATATTA
ATGGTATGGACTTAACCAGGTTTATATATTATCATTTGATAAACATTAATTATAATTTTTCTATTTA
TTATTATTATTATACTGTGAGATTAATAATTATTAAAATATAATATTACATAGTACGTGATATTTGG
GGATTTTATCTTATTAATTGGCAATTAATGATTCTAATCAAATTTTATTCTCTTTAGTTTAATGGTA
GAACAATGATCTTCTAATTCATTGGTTTTAGTTCGATTCTAAAAAGAGATGAGTAAATAATTTTCT
AGATCAGAAATACTACTTTTAACTACAAAAAGCTTACGCTTTTTAACATTTTTTTGATAAATAACA
ACTATTGTTAATATTTGGCTGTCTATTGGTTTAACTAACAATTACAAAATTTTTTCAATTTTTATAAT
ATAAATTTAAATGAGAATATTAAAAAGTCATTCATTATTAAAATTAGTGAATTCTTACCTTATCGA
TGCGTCACAACCAAGTAACATTAGTTACTTGTGAAATTTTGGTTCATTATTAGCTGTTTGTTTAAT
AGTACAAATTATTACCGGTATTACATTAGCTATGCATTATAGTCCTAGTGTAATGGAAGCTTTTAA
CTCAATAGAGCATATAATGAGAGATGTTAATAACGGGTGATTAGTTCGTTATCTACATAGTAATA
CAGCTTCTGCTTTCTTTTTCTTAGTGTATTTACACATAGGAAGAGGTATATATTACGGATCATATA
GAGCTCCTCGTACTTTAGTTTGAGCTATTGGTACTGTTATATTAATATTAATGATGGCTATCGGTT
TCCTAGGTTATGTTTTACCTTATGGACAGATGTCATTATGAGGTGCTACAGTTATTACTAATCTTA
TTAGTGCTATACCTTGAATAGGGCAAGATATTGTTGAATTCATTTGAGGTGGTTTTTCTGTTAATA
ATGCCACTTTAAACAGATTTTTTGCATTACATTTTGTATTGCCTTTTGTATTAGCTGCTTTAGTTTTA
ATGCACTTAATTGCACTTCATGATACTGCTGGTTCAAGCAATCCTCTTGGTGTTTCAGGTAATTAC
GATAGAATTACATTTGCTCCATATTATTTATTTAAAGATTTAATTACTATTTTTATATTTATTTTTGT
ATTAAGTGCTTTTGTATTCTTTATGCCTAATGTTTTAGGGGATAGTGATAATTATATTATGGCTAAT
CCTATGCAAACTCCTGCTGCTATTGTACCTGAATGATACTTATTACCTTTCTATGCTATTTTAAGAT
CTATACCTAATAAATTATTAGGTGTTATAGCGATGTTTAGTGCTATTTTAGCTATTATGTTATTACC
TGTTACAGATTTAGGTAGATCTAGAGGTTTACAATTTAGACCATTTAGTAAAATAGCTTTCTGAGT
TTTTGTTGCTAATTTCTTAGTTTTAATGCAATTAGGTGCTAAACACGTTGAAGATCCATTTATATTA
TTAGGTCAATTAAGTACTGTATTATACTTTAGTTATTTTGTTGCTATATTACCTTTAGCTAGTTACTT
AGATAATAGTTTAACTGATTTATCTAATAAATCTGAATTATTTTTAAATAAAACTAACTAAATATAT
TAAGATTATTATTTAATATATTTTCTATTTAAGATACTATTAATTTAGTATTTTGGGTTTTCAGTTTA
TAATTTATATTATATTATGCATTACCCTCCACCTTGCTTTGTAGTAAGCTAATCTGTTATTTCCTTTA
GTTTAATGGTAGAACAATGATCTTCTAATTCATTGGTTTTAGTTCGAATCTAAAAAGGAAATAAG
AAATATATTCTTATTATTACTTATATAATAATTATTTCTTAAAAATATACATTTTGCATTATAGCCGT
TTAGCTGTATTAAAATGTAAAATGATATAAAATAGAATAAATATTTAAATTATTCCTATGTTATATT
ATCCTATATTGCAACCATTATCAGAAGTTGTATTAATACTTGTACCTGCCTTATTAGCTGTAGCTT
ATGTTACAGTTGCTGAAAGAAAAACTATGGCTAGTATGCAAAGAAGATTAGGTCCTAATGCTGT
AGGTTACTATGGACTATTGCAAGCATTTGCTGATGCCTTAAAACTTTTATTAAAAGAATATGTAG
CTCCTACACAATCTAATATTGTTCTTTTCTTTTTAGGTCCTGTAATAACTTTAATTTTTGCATTATTA
GGTTACGCTGTTATACCCTATGGTCCTGGTTCAGGGATAAGCGACATGAATTTAGGTATATTTTA
CATGTTAGCTGTGTCATCTTTAGCTACATACGGTATTCTATTAGCTGGTTGAAGTGCGAATAGTA

AATACGCTTTTCTAGGTTCTCTTAGAAGTACAGCTCAATTAATTAGTTATGAATTAATATTAAGTT
CAGCTATATTAATAGTAATTATGATAACAGGAAATTTAAATTTAACTGTTTGTACTGAATCTCAAA
GAGCTATTTGATTTATACTACCTTTATTTCCTGTGTTTATAATATTTTTCATAGGATCTATAGCTGA
GACAAATAGAGCTCCTTTTGATTTAGCCGAGGCTAACCTGCTAATCTGGTTTGGTCTGGTTATAT
GTCACAAATTGCTAGGAAACCTTTTTATTTTAAAAACAAAAGACAATTAGCAGGAAACTTAATTT
AACCTAATTAAAAATATTAGATAATTAAACTCTTCATAGACTAAACGTGACAATTTAATATATATA
TATATTTATTTATATATATGATTAAATAAGATATAGTCAATCATCGGTGTGAATCGACTTAAAAAA
AAAAGCACATGGGTTAAACCCATCTCCCCTTATTAGGGGAATCAGAACTTGTTAGTGGGTTCAT
GACAGAGCATGCTGCCGTAGTTTTCGTATTCTTCTTTTTAGCTGAGTACGGTAGTATTGTACTAAT
GTGTATTTTAACTAGTATATTATTTATTGGTGGTTACTTATTATTTGAAATATCCTATGTTTTTACTG
TGGTAAATTATATTTTCTTTGAATTATTCTTTATAGACTGAGTAACATTTGTAGAGGTACAATCTTT
ATACACTGATTTTTTAAATAATTCTATCATTGAAGGATTATTATATGGGTTTAATCTAGGATTAAA
AAGTTCTTTAATGATATTCACATTTATTTGAGCTAGAGCATCCTTCCCTAGAATACGATTTGATCA
ACTAATGGGCTTCTGTTGAACAGTTTTATTACCTATTAATTTTGCAATTATTATATTAGTACCTTGT
GTTTTATATAGTTTTAACTTATTACCTGTAAATATACCATTGTTCTAGCTCACACACCCGCCCGCC
CTACTGCCACAAGGCTACAGTACATATGAGGAGGGGAACTAAAGATCTAGAACTATCCTAGTTA
ATAATTACACTTAATAGTATACTAAATAGACCATCTATCATACTCGAGAATAGTGATAGTGTAAT
TATACACTATTTTAACTGTATTCTACACTATTAGCATATTATTATCTTTATATGATAATAATTTTATA
ACTTAATTTATTAGGTTACATTAACAAAAAAATTCGTTAACTTTTTTCACCACTTTTTAATACAAAAT
ACGAATTTTA
>T-3
ATAACAATTCTAAAGAACATAAAGAGTTATTAGATAAAAATAATTCACCTATACAGTTAATAAAT
CAACTTAAAGGGTATTTTTTCATAAATCCTTTATTAGCTTTAAGTTTAGCTATTACTATTTTCTCTTT
TGCAGGTATTCCTCCTCTTGTAGGGTTCTTTGCTAAACAGATGGTATTAAGCGCGGCTATTGATC
AAGGTTATATCTTTTTATCTTTAGTTGCAATATTAACTAGTGTTATAGGAGGGGTTTATTATTTAAA
TATAATTAAAGAAATGTTCTTTTATTCACCTGACTATAAATTAAACGAAGAAATTAAAAATAATAC
TATTAATGGTCAAATTATTAATAGAAACAATAAAATATTAAATGTTGAATTTAATTATACAAATGT
AGTTATGTCTAGTTCTGTGGCAATAACTATTTCTACTATTACATTAGTAGTTTTATTATTCATGTTT
ATGAATAAAGAATGATTAAGTCTGGGTACTATATTGGTACAATCTTTATTTAGCTATTAATGAGTA
GTATGACATTATTTATAGGGTTTGTATCTGTTATAGCTATTTTATTTTTAGCCATTAATTTTATATTT
GCTCCTCATAATCCTTATCAAGAAAAATATAGTATTTTCGAGTGTGGTTTCCATAGTTTTTTAGGG
CAAAATAGAACACAATTCGGTATAAAATTCTTTATTTTTGCTTTAGTTTATTTACTTTTAGATTTAG
AAATATTATTAACTTTCCCTTTCGCTCTTAGTGAGTATGTTAATGGTATTTATGGTCTTTTAGTTAC
TTTAATTTTTATAGCTATAATAACTATAGGATTTATATTTGAATTAGGTAAAAGCGCTCTTAAAATA
GACAGCAGACAAAAATTATATATACCTAAATTGAACGTTAATTACCATACAGAGTATGTTGGAAT
AGGTAAGGTTTCTAAGTAAAGTTATAGAGGCAGAAAACCAAAAAACCTACCAAAGGGTAGCTA
ATGGGAAGCTATTAAAAATAAAAGATGATAACCTATATATAGTATAGTTACTATATATTTACTATA
ACTAGGATTATTATATATATATATATATTATCTTATTGTATATTAAGATTATTATTATTATAAGGTATA
ATTAATATAGTATCTTATTGTATAAGAATATAATATATTAACCTATAATTAATTTTATTTTTTAATTT
TTAATTATAATTTTTTTTTATATCTAGATGCTTACACATCTACAGATGTAGAAGAGAACAAAATAT
TGTTGTTATAGTAGTGGATGATATAGAAAAATATTTTTATATTATTTATTTTTGTTAGGTAGCTTTT
GAAGTGTTTGATAGAGAGGATATATGGACGGTAGGAGGGTATTCATTTTAATGAACAGTGGATA
GTTTAAATTAACCTAGTTATAGTTTATGAATTTAAATTATAATTATATTAATGTAGGTTATGATAGA
ATTATGTATTGATCCTTAAGAGTTAAGAGAGATACGCCCACGTATAATACATATTGGTTTAGGGT

TGAGTATATATACTTAAGAGTTAAGAATATATATATACAATATATAATAAATATAGTAATATATTA
ATGGTATGGACTTAACCAGGTTTATATATTATCATTTGATAAACATTAATTATAATTTTTCTATTTA
TTATTATTATTATACTGTGAGATTAATAATTATTAAAATATAATATTACATAGTACGTGATATTTGG
GGATTTTATCTTATTAATTGGCAATTAATGATTCTAATCAAATTTTATTCTCTTTAGTTTAATGGTA
GAACAATGATCTTCTAATTCATTGGTTTTAGTTCGATTCTAAAAAGAGATGAGTAAATAATTTTCT
AGATCAGAAATACTACTTTTAACTACAAAAAGCTTACGCTTTTTAACATTTTTTTGATAAATAACA
ACTATTGTTAATATTTGGCTGTCTATTGGTTTAACTAACAATTACAAAATTTTTTCAATTTTTATAAT
ATAAATTTAAATGAGAATATTAAAAAGTCATTCATTATTAAAATTAGTGAATTCTTACCTTATCGA
TGCGTCACAACCAAGTAACATTAGTTACTTGTGAAATTTTGGTTCATTATTAGCTGTTTGTTTAAT
AGTACAAATTATTACCGGTATTACATTAGCTATGCATTATAGTCCTAGTGTAATGGAAGCTTTTAA
CTCAATAGAGCATATAATGAGAGATGTTAATAACGGGTGATTAGTTCGTTATCTACATAGTAATA
CAGCTTCTGCTTTCTTTTTCTTAGTGTATTTACACATAGGAAGAGGTATATATTACGGATCATATA
GAGCTCCTCGTACTTTAGTTTGAGCTATTGGTACTGTTATATTAATATTAATGATGGCTATCGGTT
TCCTAGGTTATGTTTTACCTTATGGACAGATGTCATTATGAGGTGCTACAGTTATTACTAATCTTA
TTAGTGCTATACCTTGAATAGGGCAAGATATTGTTGAATTCATTTGAGGTGGTTTTTCTGTTAATA
ATGCCACTTTAAACAGATTTTTTGCATTACATTTTGTATTGCCTTTTGTATTAGCTGCTTTAGTTTTA
ATGCACTTAATTGCACTTCATGATACTGCTGGTTCAAGCAATCCTCTTGGTGTTTCAGGTAATTAC
GATAGAATTACATTTGCTCCATATTTTTTATTTAAAGATTTAATTACTATTTTTATATTTATTTTTGTA
TTAAGTGCTTTTGTATTCTTTATGCCTAATGTTTTAGGGGATAGTGATAATTATATTATGGCTAATC
CTATGCAAACTCCTGCTGCTATTGTACCTGAATGATACTTATTACCTTTCTATGCTATTTTAAGATC
TATACCTAATAAATTATTAGGTGTTATAGCGATGTTTAGTGCTATTTTAGCTATTATGTTATTACCT
GTTACAGATTTAGGTAGATCTAGAGGTTTACAATTTAGACCATTTAGTAAAATAGCTTTCTGAGTT
TTTGTTGCTAATTTCTTAGTTTTAATGCAATTAGGTGCTAAACACGTTGAAGATCCATTTATATTAT
TAGGTCAATTAAGTACTGTATTATACTTTAGTTATTTTGTTGCTATATTACCTTTAGCTAGTTACTT
AGATAATAGTTTAACTGATTTATCTAATAAATCTGAATTATTTTTAAATAAAACTAACTAAATATAT
TAAGATTATTATTTAATATATTTTCTATTTAAGATACTATTAATTTAGTATTTTGGGTTTTCAGTTTA
TAATTTATATTATATTATGCATTACCCTCCACCTTGCTTTGTAGTAAGCTAATCTGTTATTTCCTTTA
GTTTAATGGTAGAACAATGATCTTCTAATTCATTGGTTTTAGTTCGAATCTAAAAAGGAAATAAG
AAATATATTCTTATTATTACTTATATAATAATTATTTCTTAAAAATATACATTTTGCATTATAGCCGT
TTAGCTGTATTAAAATGTAAAATGATATAAAATAGAATAAATATTTAAATTATTCCTATGTTATATT
ATCCTATATTGCAACCATTATCAGAAGTTGTATTAATACTTGTACCTGCCTTATTAGCTGTAGCTT
ATGTTACAGTTGCTGAAAGAAAAACTATGGCTAGTATGCAAAGAAGATTAGGTCCTAATGCTGT
AGGTTACTATGGACTATTGCAAGCATTTGCTGATGCCTTAAAACTTTTATTAAAAGAATATGTAG
CTCCTACACAATCTAATATTGTTCTTTTCTTTTTAGGTCCTGTAATAACTTTAATTTTTGCATTATTA
GGTTACGCTGTTATACCCTATGGTCCTGGTTCAGGGATAAGCGACATGAATTTAGGTATATTTTA
CATGTTAGCTGTGTCATCTTTAGCTACATACGGTATTCTATTAGCTGGTTGAAGTGCGAATAGTA
AATACGCTTTTCTAGGTTCTCTTAGAAGTACAGCTCAATTAATTAGTTATGAATTAATATTAAGTT
CAGCTATATTAATAGTAATTATGATAACAGGAAATTTAAATTTAACTGTTTGTACTGAATCTCAAA
GAGCTATTTGATTTATACTACCTTTATTTCCTGTGTTTATAATATTTTTCATAGGATCTATAGCTGA
GACAAATAGAGCTCCTTTTGATTTAGCCGAGGCTAACCTGCTAATCTGGTTTGGTCTGGTTATAT
GTCACAAATTGCTAGGAAACCTTTTTATTTTAAAAACAAAAGACAATTAGCAGGAAACTTAATTT
AACCTAATTAAAAATATTAGATAATTAAACTCTTCATAGACTAAACGTGACAATTTAATATATATA
TATATTTATTTATATATATGATTAAATAAGATATAGTCAATCATCGGTGTGAATCGACTTAAAAAA
AAAAGCACATGGGTTAAACCCATCTCCCCTTATTAGGGGAATCAGAACTTGTTAGTGGGTTCAT

GACAGAGCATGCTGCCGTAGTTTTCGTATTCTTCTTTTTAGCTGAGTACGGTAGTATTGTACTAAT
GTGTATTTTAACTAGTATATTATTTATTGGTGGTTACTTATTATTTGAAATATCCTATGTTTTTACTG
TGGTAAATTATATTTTCTTTGAATTATTCTTTATAGACTGAGTAACATTTGTAGAGGTACAATCTTT
ATACACTGATTTTTTAAATAATTCTATCATTGAAGGATTATTATATGGGTTTAATCTAGGATTAAA
AAGTTCTTTAATGATATTCACATTTATTTGAGCTAGAGCATCCTTCCCTAGAATACGATTTGATCA
ACTAATGGGCTTCTGTTGAACAGTTTTATTACCTATTAATTTTGCAATTATTATATTAGTACCTTGT
GTTTTATATAGTTTTAACTTATTACCTGTAAATATACCATTGTTCTAGCTCACACACCCGCCCGCC
CTACTGCCACAAGGCTACAGTACATATGAGGAGGGGAACTAAAGATCTAGAACTATCCTAGTTA
ATAATTACACTTAATAGTATACTAAATAGACCATCTATCATACTCGAGAATAGTGATAGTGTAAT
TATACACTATTTTAACTGTATTCTACACTATTAGCATATTATTATCTTTATATGATAATAATTTTATA
ACTTAATTTATTAGGTTACATTAACAAAAAATTCGTTAACTTTTTTCACCACTTTTTAATACAAAAT
ACGAATTTTA
>YN010
ATAACAATTCTAAAGAACATAAAGAGTTATTAGATAAAAATAATTCACCTATACAGTTAATAAAT
CAACTTAAAGGGTATTTTTTCATAAATCCTTTATTAGCTTTAAGTTTAGCTATTACTATTTTCTCTTT
TGCAGGTATTCCTCCTCTTGTAGGGTTCTTTGCTAAACAGATGGTATTAAGCGCGGCTATTGATC
AAGGTTATATCTTTTTATCTTTAGTTGCAATATTAACTAGTGTTATAGGAGGGGTTTATTATTTAAA
TATAATTAAAGAAATGTTCTTTTATTCACCTGACTATAAATTAAACGAAGAAATTAAAAATAATAC
TATTAATGGTCAAATTATTAATAGAAACAATAAAATATTAAATGTTGAATTTAATTATACAAATGT
AGTTATGTCTAGTTCTGTGGCAATAACTATTTCTACTATTACATTAGTAGTTTTATTATTCATGTTT
ATGAATAAAGAATGATTAAGTCTGGGTACTATATTGGTACAATCTTTATTTAGCTATTAATGAGTA
GTATGACATTATTTATAGGGTTTGTATCTGTTATAGCTATTTTATTTTTAGCCATTAATTTTATATTT
GCTCCTCATAATCCTTATCAAGAAAAATATAGTATTTTCGAGTGTGGTTTCCATAGTTTTTTAGGG
CAAAATAGAACACAATTCGGTATAAAATTCTTTATTTTTGCTTTAGTTTATTTACTTTTAGATTTAG
AAATATTATTAACTTTCCCTTTCGCTCTTAGTGAGTATGTTAATGGTATTTATGGTCTTTTAGTTAC
TTTAATTTTTATAGCTATAATAACTATAGGATTTATATTTGAATTAGGTAAAAGCGCTCTTAAAATA
GACAGCAGACAAAAATTATATATACCTAAATTGAACGTTAATTACCATACAGAGTATGTTGGAAT
AGGTAAGGTTTCTAAGTAAAGTTATAGAGGCAGAAAACCAAAAAACCTACCAAAGGGTAGCTA
ATGGGAAGCTATTAAAAATAAAAGATGATAACCTATATATAGTATAGTTACTATATATTTACTATA
ACTAGGATTATTATATATATATATATTATCTTATTGTATATTAAGATTATTATTATTATAAGGTATA
ATTAATATAGTATCTTATTGTATAAGAATATAATATATTAACCTATAATTAATTTTATTTTTTAATTT
TTAATTATAATTTTTTTTTATATCTAGATGCTTACACATCTACAGATGTAGAAGAGAACAAAATAT
TGTTGTTATAGTAGTGGATGATATAGAAAAATATTTTTATATTATTTATTTTTGTTAGGTAGCTTTT
GAAGTGTTTGATAGAGAGGATATATGGACGGTAGGAGGGTATTCATTTTAATGAACAGTGGATA
GTTTAAATTAACCTAGTTATAGTTTATGAATTTAAATTATAATTATATTAATGTAGGTTATGATAGA
ATTATGTATTGATCCTTAAGAGTTAAGAGAGATACGCCCACGTATAATACATATTGGTTTAGGGT
TGAGTATATATACTTAAGAGTTAAGAATATATATATACAATATATAATAAATATAGTAATATATTA
ATGGTATGGACTTAACCAGGTTTATATATTATCATTTGATAAACATTAATTATAATTTTTCTATTTA
TTATTATTATTATACTGTGAGATTAATAATTATTAAAATATAATATTACATAGTACGTGATATTTGG
GGATTTTATCTTATTAATTGGCAATTAATGATTCTAATCAAATTTTATTCTCTTTAGTTTAATGGTA
GAACAATGATCTTCTAATTCATTGGTTTTAGTTCGATTCTAAAAAGAGATGAGTAAATAATTTTCT
AGATCAGAAATACTACTTTTAACTACAAAAAGCTTACGCTTTTTAACATTTTTTTGATAAATAACA
ACTATTGTTAATATTTGGCTGTCTATTGGTTTAACTAACAATTACAAAATTTTTTCAATTTTTATAAT
ATAAATTTAAATGAGAATATTAAAAAGTCATTCATTATTAAAATTAGTGAATTCTTACCTTATCGA

TGCGTCACAACCAAGTAACATTAGTTACTTGTGAAATTTTGGTTCATTATTAGCTGTTTGTTTAAT
AGTACAAATTATTACCGGTATTACATTAGCTATGCATTATAGTCCTAGTGTAATGGAAGCTTTTAA
CTCAATAGAGCATATAATGAGAGATGTTAATAACGGGTGATTAGTTCGTTATCTACATAGTAATA
CAGCTTCTGCTTTCTTTTTCTTAGTGTATTTACACATAGGAAGAGGTATATATTACGGATCATATA
GAGCTCCTCGTACTTTAGTTTGAGCTATTGGTACTGTTATATTAATATTAATGATGGCTATCGGTT
TCCTAGGTTATGTTTTACCTTATGGACAGATGTCATTATGAGGTGCTACAGTTATTACTAATCTTA
TTAGTGCTATACCTTGAATAGGGCAAGATATTGTTGAATTCATTTGAGGTGGTTTTTCTGTTAATA
ATGCCACTTTAAACAGATTTTTTGCATTACATTTTGTATTGCCTTTTGTATTAGCTGCTTTAGTTTTA
ATGCACTTAATTGCACTTCATGATACTGCTGGTTCAAGCAATCCTCTTGGTGTTTCAGGTAATTAC
GATAGAATTACATTTGCTCCATATTTTTTATTTAAAGATTTAATTACTATTTTTATATTTATTTTTGTA
TTAAGTGCTTTTGTATTCTTTATGCCTAATGTTTTAGGGGATAGTGATAATTATATTATGGCTAATC
CTATGCAAACTCCTGCTGCTATTGTACCTGAATGATACTTATTACCTTTCTATGCTATTTTAAGATC
TATACCTAATAAATTATTAGGTGTTATAGCGATGTTTAGTGCTATTTTAGCTATTATGTTATTACCT
GTTACAGATTTAGGTAGATCTAGAGGTTTACAATTTAGACCATTTAGTAAAATAGCTTTCTGAGTT
TTTGTTGCTAATTTCTTAGTTTTAATGCAATTAGGTGCTAAACACGTTGAAGATCCATTTATATTAT
TAGGTCAATTAAGTACTGTATTATACTTTAGTTATTTTGTTGCTATATTACCTTTAGCTAGTTACTT
AGATAATAGTTTAACTGATTTATCTAATAAATCTGAATTATTTTTAAATAAAACTAACTAAATATAT
TAAGATTATTATTTAATATATTTTCTATTTAAGATACTATTAATTTAGTATTTTGGGTTTTCAGTTTA
TAATTTATATTATATTATGCATTACCCTCCACCTTGCTTTGTAGTAAGCTAATCTGTTATTTCCTTTA
GTTTAATGGTAGAACAATGATCTTCTAATTCATTGGTTTTAGTTCGAATCTAAAAAGGAAATAAG
AAATATATTCTTATTATTACTTATATAATAATTATTTCTTAAAAATATACATTTTGCATTATAGCCGT
TTAGCTGTATTAAAATGTAAAATGATATAAAATAGAATAAATATTTAAATTATTCCTATGTTATATT
ATCCTATATTGCAACCATTATCAGAAGTTGTATTAATACTTGTACCTGCCTTATTAGCTGTAGCTT
ATGTTACAGTTGCTGAAAGAAAAACTATGGCTAGTATGCAAAGAAGATTAGGTCCTAATGCTGT
AGGTTACTATGGACTATTGCAAGCATTTGCTGATGCCTTAAAACTTTTATTAAAAGAATATGTAG
CTCCTACACAATCTAATATTGTTCTTTTCTTTTTAGGTCCTGTAATAACTTTAATTTTTGCATTATTA
GGTTACGCTGTTATACCCTATGGTCCTGGTTCAGGGATAAGCGACATGAATTTAGGTATATTTTA
CATGTTAGCTGTGTCATCTTTAGCTACATACGGTATTCTATTAGCTGGTTGAAGTGCGAATAGTA
AATACGCTTTTCTAGGTTCTCTTAGAAGTACAGCTCAATTAATTAGTTATGAATTAATATTAAGTT
CAGCTATATTAATAGTAATTATGATAACAGGAAATTTAAATTTAACTGTTTGTACTGAATCTCAAA
GAGCTATTTGATTTATACTACCTTTATTTCCTGTGTTTATAATATTTTTCATAGGATCTATAGCTGA
GACAAATAGAGCTCCTTTTGATTTAGCCGAGGCTAACCTGCTAATCTGGTTTGGTCTGGTTATAT
GTCACAAATTGCTAGGAAACCTTTTTATTTTAAAAACAAAAGACAATTAGCAGGAAACTTAATTT
AACCTAATTAAAAATATTAGATAATTAAACTCTTCATAGACTAAACGTGACAATTTAATATATATA
TATATTTATTTATATATATGATTAAATAAGATATAGTCAATCATCGGTGTGAATCGACTTAAAAAA
AAAAGCACATGGGTTAAACCCATCTCCCCTTATTAGGGGAATCAGAACTTGTTAGTGGGTTCAT
GACAGAGCATGCTGCCGTAGTTTTCGTATTCTTCTTTTTAGCTGAGTACGGTAGTATTGTACTAAT
GTGTATTTTAACTAGTATATTATTTATTGGTGGTTACTTATTATTTGAAATATCCTATGTTTTTACTG
TGGTAAATTATATTTTCTTTGAATTATTCTTTATAGACTGAGTAACATTTGTAGAGGTACAATCTTT
ATACACTGATTTTTTAAATAATTCTATCATTGAAGGATTATTATATGGGTTTAATCTAGGATTAAA
AAGTTCTTTAATGATATTCACATTTATTTGAGCTAGAGCATCCTTCCCTAGAATACGATTTGATCA
ACTAATGGGCTTCTGTTGAACAGTTTTATTACCTATTAATTTTGCAATTATTATATTAGTACCTTGT
GTTTTATATAGTTTTAACTTATTACCTGTAAATATACCATTGTTCTAGCTCACACACCCGCCCGCC
CTACTGCCACAAGGCTACAGTACATATGAGGAGGGGAACTAAAGATCTAGAACTATCCTAGTTA

ATAATTACACTTAATAGTATACTAAATAGACCATCTATCATACTCGAGAATAGTGATAGTGTAAT
TATACACTATTTTAACTGTATTCTACACTATTAGCATATTATTATCTTTATATGATAATAATTTTATA
ACTTAATTTATTAGGTTACATTAACAAAAAATTCGTTAACTTTTTTCACCACTTTTTAATACAAAAT
ACGAATTTTA
>YN029
ATAACAATTCTAAAGAACATAAAGAGTTATTAGATAAAAATAATTCACCTATACAGTTAATAAAT
CAACTTAAAGGGTATTTTTTCATAAATCCTTTATTAGCTTTAAGTTTAGCTATTACTATTTTCTCTTT
TGCAGGTATTCCTCCTCTTGTAGGGTTCTTTGCTAAACAGATGGTATTAAGCGCGGCTATTGATC
AAGGTTATATCTTTTTATCTTTAGTTGCAATATTAACTAGTGTTATAGGAGGGGTTTATTATTTAAA
TATAATTAAAGAAATGTTCTTTTATTCACCTGACTATAAATTAAACGAAGAAATTAAAAATAATAC
TATTAATGGTCAAATTATTAATAGAAACAATAAAATATTAAATGTTGAATTTAATTATACAAATGT
AGTTATGTCTAGTTCTGTGGCAATAACTATTTCTACTATTACATTAGTAGTTTTATTATTCATGTTT
ATGAATAAAGAATGATTAAGTCTGGGTACTATATTGGTACAATCTTTATTTAGCTATTAATGAGTA
GTATGACATTATTTATAGGGTTTGTATCTGTTATAGCTATTTTATTTTTAGCCATTAATTTTATATTT
GCTCCTCATAATCCTTATCAAGAAAAATATAGTATTTTCGAGTGTGGTTTCCATAGTTTTTTAGGG
CAAAATAGAACACAATTCGGTATAAAATTCTTTATTTTTGCTTTAGTTTATTTACTTTTAGATTTAG
AAATATTATTAACTTTCCCTTTCGCTCTTAGTGAGTATGTTAATGGTATTTATGGTCTTTTAGTTAC
TTTAATTTTTATAGCTATAATAACTATAGGATTTATATTTGAATTAGGTAAAAGCGCTCTTAAAATA
GACAGCAGACAAAAATTATATATACCTAAATTGAACGTTAATTACCATACAGAGTATGTTGGAAT
AGGTAAGGTTTCTAAGTAAAGTTATAGAGGCAGAAAACCAAAAAACCTACCAAAGGGTAGCTA
ATGGGAAGCTATTAAAAATAAAAGATGATAACCTATATATAGTATAGTTACTATATATTTACTATA
ACTAGGATTATTATATATATATATATTATCTTATTGTATATTAAGATTATTATTATTATAAGGTATA
ATTAATATAGTATCTTATTGTATAAGAATATAATATATTAACCTATAATTAATTTTATTTTTTAATTT
TTAATTATAATTTTTTTTTATATCTAGATGCTTACACATCTACAGATGTAGAAGAGAACAAAATAT
TGTTGTTATAGTAGTGGATGATATAGAAAAATATTTTTATATTATTTATTTTTGTTAGGTAGCTTTT
GAAGTGTTTGATAGAGAGGATATATGGACGGTAGGAGGGTATTCATTTTAATGAACAGTGGATA
GTTTAAATTAACCTAGTTATAGTTTATGAATTTAAATTATAATTATATTAATGTAGGTTATGATAGA
ATTATGTATTGATCCTTAAGAGTTAAGAGAGATACGCCCACGTATAATACATATTGGTTTAGGGT
TGAGTATATATACTTAAGAGTTAAGAATATATATATACAATATATAATAAATATAGTAATATATTA
ATGGTATGGACTTAACCAGGTTTATATATTATCATTTGATAAACATTAATTATAATTTTTCTATTTA
TTATTATTATTATACTGTGAGATTAATAATTATTAAAATATAATATTACATAGTACGTGATATTTGG
GGATTTTATCTTATTAATTGGCAATTAATGATTCTAATCAAATTTTATTCTCTTTAGTTTAATGGTA
GAACAATGATCTTCTAATTCATTGGTTTTAGTTCGATTCTAAAAAGAGATGAGTAAATAATTTTCT
AGATCAGAAATACTACTTTTAACTACAAAAAGCTTACGCTTTTTAACATTTTTTTGATAAATAACA
ACTATTGTTAATATTTGGCTGTCTATTGGTTTAACTAACAATTACAAAATTTTTTCAATTTTTATAAT
ATAAATTTAAATGAGAATATTAAAAAGTCATTCATTATTAAAATTAGTGAATTCTTACCTTATCGA
TGCGTCACAACCAAGTAACATTAGTTACTTGTGAAATTTTGGTTCATTATTAGCTGTTTGTTTAAT
AGTACAAATTATTACCGGTATTACATTAGCTATGCATTATAGTCCTAGTGTAATGGAAGCTTTTAA
CTCAATAGAGCATATAATGAGAGATGTTAATAACGGGTGATTAGTTCGTTATCTACATAGTAATA
CAGCTTCTGCTTTCTTTTTCTTAGTGTATTTACACATAGGAAGAGGTATATATTACGGATCATATA
GAGCTCCTCGTACTTTAGTTTGAGCTATTGGTACTGTTATATTAATATTAATGATGGCTATCGGTT
TCCTAGGTTATGTTTTACCTTATGGACAGATGTCATTATGAGGTGCTACAGTTATTACTAATCTTA
TTAGTGCTATACCTTGAATAGGGCAAGATATTGTTGAATTCATTTGAGGTGGTTTTTCTGTTAATA
ATGCCACTTTAAACAGATTTTTTGCATTACATTTTGTATTGCCTTTTGTATTAGCTGCTTTAGTTTTA

ATGCACTTAATTGCACTTCATGATACTGCTGGTTCAAGCAATCCTCTTGGTGTTTCAGGTAATTAC
GATAGAATTACATTTGCTCCATATTTTTTATTTAAAGATTTAATTACTATTTTTATATTTATTTTTGTA
TTAAGTGCTTTTGTATTCTTTATGCCTAATGTTTTAGGGGATAGTGATAATTATATTATGGCTAATC
CTATGCAAACTCCTGCTGCTATTGTACCTGAATGATACTTATTACCTTTCTATGCTATTTTAAGATC
TATACCTAATAAATTATTAGGTGTTATAGCGATGTTTAGTGCTATTTTAGCTATTATGTTATTACCT
GTTACAGATTTAGGTAGATCTAGAGGTTTACAATTTAGACCATTTAGTAAAATAGCTTTCTGAGTT
TTTGTTGCTAATTTCTTAGTTTTAATGCAATTAGGTGCTAAACACGTTGAAGATCCATTTATATTAT
TAGGTCAATTAAGTACTGTATTATACTTTAGTTATTTTGTTGCTATATTACCTTTAGCTAGTTACTT
AGATAATAGTTTAACTGATTTATCTAATAAATCTGAATTATTTTTAAATAAAACTAACTAAATATAT
TAAGATTATTATTTAATATATTTTCTATTTAAGATACTATTAATTTAGTATTTTGGGTTTTCAGTTTA
TAATTTATATTATATTATGCATTACCCTCCACCTTGCTTTGTAGTAAGCTAATCTGTTATTTCCTTTA
GTTTAATGGTAGAACAATGATCTTCTAATTCATTGGTTTTAGTTCGAATCTAAAAAGGAAATAAG
AAATATATTCTTATTATTACTTATATAATAATTATTTCTTAAAAATATACATTTTGCATTATAGCCGT
TTAGCTGTATTAAAATGTAAAATGATATAAAATAGAATAAATATTTAAATTATTCCTATGTTATATT
ATCCTATATTGCAACCATTATCAGAAGTTGTATTAATACTTGTACCTGCCTTATTAGCTGTAGCTT
ATGTTACAGTTGCTGAAAGAAAAACTATGGCTAGTATGCAAAGAAGATTAGGTCCTAATGCTGT
AGGTTACTATGGACTATTGCAAGCATTTGCTGATGCCTTAAAACTTTTATTAAAAGAATATGTAG
CTCCTACACAATCTAATATTGTTCTTTTCTTTTTAGGTCCTGTAATAACTTTAATTTTTGCATTATTA
GGTTACGCTGTTATACCCTATGGTCCTGGTTCAGGGATAAGCGACATGAATTTAGGTATATTTTA
CATGTTAGCTGTGTCATCTTTAGCTACATACGGTATTCTATTAGCTGGTTGAAGTGCGAATAGTA
AATACGCTTTTCTAGGTTCTCTTAGAAGTACAGCTCAATTAATTAGTTATGAATTAATATTAAGTT
CAGCTATATTAATAGTAATTATGATAACAGGAAATTTAAATTTAACTGTTTGTACTGAATCTCAAA
GAGCTATTTGATTTATACTACCTTTATTTCCTGTGTTTATAATATTTTTCATAGGATCTATAGCTGA
GACAAATAGAGCTCCTTTTGATTTAGCCGAGGCTAACCTGCTAATCTGGTTTGGTCTGGTTATAT
GTCACAAATTGCTAGGAAACCTTTTTATTTTAAAAACAAAAGACAATTAGCAGGAAACTTAATTT
AACCTAATTAAAAATATTAGATAATTAAACTCTTCATAGACTAAACGTGACAATTTAATATATATA
TATATTTATTTATATATATGATTAAATAAGATATAGTCAATCATCGGTGTGAATCGACTTAAAAAA
AAAAGCACATGGGTTAAACCCATCTCCCCTTATTAGGGGAATCAGAACTTGTTAGTGGGTTCAT
GACAGAGCATGCTGCCGTAGTTTTCGTATTCTTCTTTTTAGCTGAGTACGGTAGTATTGTACTAAT
GTGTATTTTAACTAGTATATTATTTATTGGTGGTTACTTATTATTTGAAATATCCTATGTTTTTACTG
TGGTAAATTATATTTTCTTTGAATTATTCTTTATAGACTGAGTAACATTTGTAGAGGTACAATCTTT
ATACACTGATTTTTTAAATAATTCTATCATTGAAGGATTATTATATGGGTTTAATCTAGGATTAAA
AAGTTCTTTAATGATATTCACATTTATTTGAGCTAGAGCATCCTTCCCTAGAATACGATTTGATCA
ACTAATGGGCTTCTGTTGAACAGTTTTATTACCTATTAATTTTGCAATTATTATATTAGTACCTTGT
GTTTTATATAGTTTTAACTTATTACCTGTAAATATACCATTGTTCTAGCTCACACACCCGCCCGCC
CTACTGCCACAAGGCTACAGTACATATGAGGAGGGGAACTAAAGATCTAGAACTATCCTAGTTA
ATAATTACACTTAATAGTATACTAAATAGACCATCTATCATACTCGAGAATAGTGATAGTGTAAT
TATACACTATTTTAACTGTATTCTACACTATTAGCATATTATTATCTTTATATGATAATAATTTTATA
ACTTAATTTATTAGGTTACATTAACAAAAAATTCGTTAACTTTTTTCACCACTTTTTAATACAAAAT
ACGAATTTTA
>YN030
ATAACAATTCTAAAGAACATAAAGAGTTATTAGATAAAAATAATTCACCTATACAGTTAATAAAT
CAACTTAAAGGGTATTTTTTCATAAATCCTTTATTAGCTTTAAGTTTAGCTATTACTATTTTCTCTTT
TGCAGGTATTCCTCCTCTTGTAGGGTTCTTTGCTAAACAGATGGTATTAAGCGCGGCTATTGATC

AAGGTTATATCTTTTTATCTTTAGTTGCAATATTAACTAGTGTTATAGGAGGGGTTTATTATTTAAA
TATAATTAAAGAAATGTTCTTTTATTCACCTGACTATAAATTAAACGAAGAAATTAAAAATAATAC
TATTAATGGTCAAATTATTAATAGAAACAATAAAATATTAAATGTTGAATTTAATTATACAAATGT
AGTTATGTCTAGTTCTGTGGCAATAACTATTTCTACTATTACATTAGTAGTTTTATTATTCATGTTT
ATGAATAAAGAATGATTAAGTCTGGGTACTATATTGGTACAATCTTTATTTAGCTATTAATGAGTA
GTATGACATTATTTATAGGGTTTGTATCTGTTATAGCTATTTTATTTTTAGCCATTAATTTTATATTT
GCTCCTCATAATCCTTATCAAGAAAAATATAGTATTTTCGAGTGTGGTTTCCATAGTTTTTTAGGG
CAAAATAGAACACAATTCGGTATAAAATTCTTTATTTTTGCTTTAGTTTATTTACTTTTAGATTTAG
AAATATTATTAACTTTCCCTTTCGCTCTTAGTGAGTATGTTAATGGTATTTATGGTCTTTTAGTTAC
TTTAATTTTTATAGCTATAATAACTATAGGATTTATATTTGAATTAGGTAAAAGCGCTCTTAAAATA
GACAGCAGACAAAAATTATATATACCTAAATTGAACGTTAATTACCATACAGAGTATGTTGGAAT
AGGTAAGGTTTCTAAGTAAAGTTATAGAGGCAGAAAACCAAAAAACCTACCAAAGGGTAGCTA
ATGGGAAGCTATTAAAAATAAAAGATGATAACCTATATATAGTATAGTTACTATATATTTACTATA
ACTAGGATTATTATATATATATATATTATCTTATTGTATATTAAGATTATTATTATTATAAGGTATA
ATTAATATAGTATCTTATTGTATAAGAATATAATATATTAACCTATAATTAATTTTATTTTTTAATTT
TTAATTATAATTTTTTTTTATATCTAGATGCTTACACATCTACAGATGTAGAAGAGAACAAAATAT
TGTTGTTATAGTAGTGGATGATATAGAAAAATATTTTTATATTATTTATTTTTGTTAGGTAGCTTTT
GAAGTGTTTGATAGAGAGGATATATGGACGGTAGGAGGGTATTCATTTTAATGAACAGTGGATA
GTTTAAATTAACCTAGTTATAGTTTATGAATTTAAATTATAATTATATTAATGTAGGTTATGATAGA
ATTATGTATTGATCCTTAAGAGTTAAGAGAGATACGCCCACGTATAATACATATTGGTTTAGGGT
TGAGTATATATACTTAAGAGTTAAGAATATATATATACAATATATAATAAATATAGTAATATATTA
ATGGTATGGACTTAACCAGGTTTATATATTATCATTTGATAAACATTAATTATAATTTTTCTATTTA
TTATTATTATTATACTGTGAGATTAATAATTATTAAAATATAATATTACATAGTACGTGATATTTGG
GGATTTTATCTTATTAATTGGCAATTAATGATTCTAATCAAATTTTATTCTCTTTAGTTTAATGGTA
GAACAATGATCTTCTAATTCATTGGTTTTAGTTCGATTCTAAAAAGAGATGAGTAAATAATTTTCT
AGATCAGAAATACTACTTTTAACTACAAAAAGCTTACGCTTTTTAACATTTTTTTGATAAATAACA
ACTATTGTTAATATTTGGCTGTCTATTGGTTTAACTAACAATTACAAAATTTTTTCAATTTTTATAAT
ATAAATTTAAATGAGAATATTAAAAAGTCATTCATTATTAAAATTAGTGAATTCTTACCTTATCGA
TGCGTCACAACCAAGTAACATTAGTTACTTGTGAAATTTTGGTTCATTATTAGCTGTTTGTTTAAT
AGTACAAATTATTACCGGTATTACATTAGCTATGCATTATAGTCCTAGTGTAATGGAAGCTTTTAA
CTCAATAGAGCATATAATGAGAGATGTTAATAACGGGTGATTAGTTCGTTATCTACATAGTAATA
CAGCTTCTGCTTTCTTTTTCTTAGTGTATTTACACATAGGAAGAGGTATATATTACGGATCATATA
GAGCTCCTCGTACTTTAGTTTGAGCTATTGGTACTGTTATATTAATATTAATGATGGCTATCGGTT
TCCTAGGTTATGTTTTACCTTATGGACAGATGTCATTATGAGGTGCTACAGTTATTACTAATCTTA
TTAGTGCTATACCTTGAATAGGGCAAGATATTGTTGAATTCATTTGAGGTGGTTTTTCTGTTAATA
ATGCCACTTTAAACAGATTTTTTGCATTACATTTTGTATTGCCTTTTGTATTAGCTGCTTTAGTTTTA
ATGCACTTAATTGCACTTCATGATACTGCTGGTTCAAGCAATCCTCTTGGTGTTTCAGGTAATTAC
GATAGAATTACATTTGCTCCATATTTTTTATTTAAAGATTTAATTACTATTTTTATATTTATTTTTGTA
TTAAGTGCTTTTGTATTCTTTATGCCTAATGTTTTAGGGGATAGTGATAATTATATTATGGCTAATC
CTATGCAAACTCCTGCTGCTATTGTACCTGAATGATACTTATTACCTTTCTATGCTATTTTAAGATC
TATACCTAATAAATTATTAGGTGTTATAGCGATGTTTAGTGCTATTTTAGCTATTATGTTATTACCT
GTTACAGATTTAGGTAGATCTAGAGGTTTACAATTTAGACCATTTAGTAAAATAGCTTTCTGAGTT
TTTGTTGCTAATTTCTTAGTTTTAATGCAATTAGGTGCTAAACACGTTGAAGATCCATTTATATTAT
TAGGTCAATTAAGTACTGTATTATACTTTAGTTATTTTGTTGCTATATTACCTTTAGCTAGTTACTT

AGATAATAGTTTAACTGATTTATCTAATAAATCTGAATTATTTTTAAATAAAACTAACTAAATATAT
TAAGATTATTATTTAATATATTTTCTATTTAAGATACTATTAATTTAGTATTTTGGGTTTTCAGTTTA
TAATTTATATTATATTATGCATTACCCTCCACCTTGCTTTGTAGTAAGCTAATCTGTTATTTCCTTTA
GTTTAATGGTAGAACAATGATCTTCTAATTCATTGGTTTTAGTTCGAATCTAAAAAGGAAATAAG
AAATATATTCTTATTATTACTTATATAATAATTATTTCTTAAAAATATACATTTTGCATTATAGCCGT
TTAGCTGTATTAAAATGTAAAATGATATAAAATAGAATAAATATTTAAATTATTCCTATGTTATATT
ATCCTATATTGCAACCATTATCAGAAGTTGTATTAATACTTGTACCTGCCTTATTAGCTGTAGCTT
ATGTTACAGTTGCTGAAAGAAAAACTATGGCTAGTATGCAAAGAAGATTAGGTCCTAATGCTGT
AGGTTACTATGGACTATTGCAAGCATTTGCTGATGCCTTAAAACTTTTATTAAAAGAATATGTAG
CTCCTACACAATCTAATATTGTTCTTTTCTTTTTAGGTCCTGTAATAACTTTAATTTTTGCATTATTA
GGTTACGCTGTTATACCCTATGGTCCTGGTTCAGGGATAAGCGACATGAATTTAGGTATATTTTA
CATGTTAGCTGTGTCATCTTTAGCTACATACGGTATTCTATTAGCTGGTTGAAGTGCGAATAGTA
AATACGCTTTTCTAGGTTCTCTTAGAAGTACAGCTCAATTAATTAGTTATGAATTAATATTAAGTT
CAGCTATATTAATAGTAATTATGATAACAGGAAATTTAAATTTAACTGTTTGTACTGAATCTCAAA
GAGCTATTTGATTTATACTACCTTTATTTCCTGTGTTTATAATATTTTTCATAGGATCTATAGCTGA
GACAAATAGAGCTCCTTTTGATTTAGCCGAGGCTAACCTGCTAATCTGGTTTGGTCTGGTTATAT
GTCACAAATTGCTAGGAAACCTTTTTATTTTAAAAACAAAAGACAATTAGCAGGAAACTTAATTT
AACCTAATTAAAAATATTAGATAATTAAACTCTTCATAGACTAAACGTGACAATTTAATATATATA
TATATTTATTTATATATATGATTAAATAAGATATAGTCAATCATCGGTGTGAATCGACTTAAAAAA
AAAAGCACATGGGTTAAACCCATCTCCCCTTATTAGGGGAATCAGAACTTGTTAGTGGGTTCAT
GACAGAGCATGCTGCCGTAGTTTTCGTATTCTTCTTTTTAGCTGAGTACGGTAGTATTGTACTAAT
GTGTATTTTAACTAGTATATTATTTATTGGTGGTTACTTATTATTTGAAATATCCTATGTTTTTACTG
TGGTAAATTATATTTTCTTTGAATTATTCTTTATAGACTGAGTAACATTTGTAGAGGTACAATCTTT
ATACACTGATTTTTTAAATAATTCTATCATTGAAGGATTATTATATGGGTTTAATCTAGGATTAAA
AAGTTCTTTAATGATATTCACATTTATTTGAGCTAGAGCATCCTTCCCTAGAATACGATTTGATCA
ACTAATGGGCTTCTGTTGAACAGTTTTATTACCTATTAATTTTGCAATTATTATATTAGTACCTTGT
GTTTTATATAGTTTTAACTTATTACCTGTAAATATACCATTGTTCTAGCTCACACACCCGCCCGCC
CTACTGCCACAAGGCTACAGTACATATGAGGAGGGGAACTAAAGATCTAGAACTATCCTAGTTA
ATAATTACACTTAATAGTATACTAAATAGACCATCTATCATACTCGAGAATAGTGATAGTGTAAT
TATACACTATTTTAACTGTATTCTACACTATTAGCATATTATTATCTTTATATGATAATAATTTTATA
ACTTAATTTATTAGGTTACATTAACAAAAAATTCGTTAACTTTTTTCACCACTTTTTAATACAAAAT
ACGAATTTTA
>YN040
ATAACAATTCTAAAGAACATAAAGAGTTATTAGATAAAAATAATTCACCTATACAGTTAATAAAT
CAACTTAAAGGGTATTTTTTCATAAATCCTTTATTAGCTTTAAGTTTAGCTATTACTATTTTCTCTTT
TGCAGGTATTCCTCCTCTTGTAGGGTTCTTTGCTAAACAGATGGTATTAAGCGCGGCTATTGATC
AAGGTTATATCTTTTTATCTTTAGTTGCAATATTAACTAGTGTTATAGGAGGGGTTTATTATTTAAA
TATAATTAAAGAAATGTTCTTTTATTCACCTGACTATAAATTAAACGAAGAAATTAAAAATAATAC
TATTAATGGTCAAATTATTAATAGAAACAATAAAATATTAAATGTTGAATTTAATTATACAAATGT
AGTTATGTCTAGTTCTGTGGCAATAACTATTTCTACTATTACATTAGTAGTTTTATTATTCATGTTT
ATGAATAAAGAATGATTAAGTCTGGGTACTATATTGGTACAATCTTTATTTAGCTATTAATGAGTA
GTATGACATTATTTATAGGGTTTGTATCTGTTATAGCTATTTTATTTTTAGCCATTAATTTTATATTT
GCTCCTCATAATCCTTATCAAGAAAAATATAGTATTTTCGAGTGTGGTTTCCATAGTTTTTTAGGG
CAAAATAGAACACAATTCGGTATAAAATTCTTTATTTTTGCTTTAGTTTATTTACTTTTAGATTTAG

AAATATTATTAACTTTCCCTTTCGCTCTTAGTGAGTATGTTAATGGTATTTATGGTCTTTTAGTTAC
TTTAATTTTTATAGCTATAATAACTATAGGATTTATATTTGAATTAGGTAAAAGCGCTCTTAAAATA
GACAGCAGACAAAAATTATATATACCTAAATTGAACGTTAATTACCATACAGAGTATGTTGGAAT
AGGTAAGGTTTCTAAGTAAAGTTATAGAGGCAGAAAACCAAAAAACCTACCAAAGGGTAGCTA
ATGGGAAGCTATTAAAAATAAAAGATGATAACCTATATATAGTATAGTTACTATATATTTACTATA
ACTAGGATTATTATATATATATATATTATCTTATTGTATATTAAGATTATTATTATTATAAGGTATA
ATTAATATAGTATCTTATTGTATAAGAATATAATATATTAACCTATAATTAATTTTATTTTTTAATTT
TTAATTATAATTTTTTTTTATATCTAGATGCTTACACATCTACAGATGTAGAAGAGAACAAAATAT
TGTTGTTATAGTAGTGGATGATATAGAAAAATATTTTTATATTATTTATTTTTGTTAGGTAGCTTTT
GAAGTGTTTGATAGAGAGGATATATGGACGGTAGGAGGGTATTCATTTTAATGAACAGTGGATA
GTTTAAATTAACCTAGTTATAGTTTATGAATTTAAATTATAATTATATTAATGTAGGTTATGATAGA
ATTATGTATTGATCCTTAAGAGTTAAGAGAGATACGCCCACGTATAATACATATTGGTTTAGGGT
TGAGTATATATACTTAAGAGTTAAGAATATATATATACAATATATAATAAATATAGTAATATATTA
ATGGTATGGACTTAACCAGGTTTATATATTATCATTTGATAAACATTAATTATAATTTTTCTATTTA
TTATTATTATTATACTGTGAGATTAATAATTATTAAAATATAATATTACATAGTACGTGATATTTGG
GGATTTTATCTTATTAATTGGCAATTAATGATTCTAATCAAATTTTATTCTCTTTAGTTTAATGGTA
GAACAATGATCTTCTAATTCATTGGTTTTAGTTCGATTCTAAAAAGAGATGAGTAAATAATTTTCT
AGATCAGAAATACTACTTTTAACTACAAAAAGCTTACGCTTTTTAACATTTTTTTGATAAATAACA
ACTATTGTTAATATTTGGCTGTCTATTGGTTTAACTAACAATTACAAAATTTTTTCAATTTTTATAAT
ATAAATTTAAATGAGAATATTAAAAAGTCATTCATTATTAAAATTAGTGAATTCTTACCTTATCGA
TGCGTCACAACCAAGTAACATTAGTTACTTGTGAAATTTTGGTTCATTATTAGCTGTTTGTTTAAT
AGTACAAATTATTACCGGTATTACATTAGCTATGCATTATAGTCCTAGTGTAATGGAAGCTTTTAA
CTCAATAGAGCATATAATGAGAGATGTTAATAACGGGTGATTAGTTCGTTATCTACATAGTAATA
CAGCTTCTGCTTTCTTTTTCTTAGTGTATTTACACATAGGAAGAGGTATATATTACGGATCATATA
GAGCTCCTCGTACTTTAGTTTGAGCTATTGGTACTGTTATATTAATATTAATGATGGCTATCGGTT
TCCTAGGTTATGTTTTACCTTATGGACAGATGTCATTATGAGGTGCTACAGTTATTACTAATCTTA
TTAGTGCTATACCTTGAATAGGGCAAGATATTGTTGAATTCATTTGAGGTGGTTTTTCTGTTAATA
ATGCCACTTTAAACAGATTTTTTGCATTACATTTTGTATTGCCTTTTGTATTAGCTGCTTTAGTTTTA
ATGCACTTAATTGCACTTCATGATACTGCTGGTTCAAGCAATCCTCTTGGTGTTTCAGGTAATTAC
GATAGAATTACATTTGCTCCATATTTTTTATTTAAAGATTTAATTACTATTTTTATATTTATTTTTGTA
TTAAGTGCTTTTGTATTCTTTATGCCTAATGTTTTAGGGGATAGTGATAATTATATTATGGCTAATC
CTATGCAAACTCCTGCTGCTATTGTACCTGAATGATACTTATTACCTTTCTATGCTATTTTAAGATC
TATACCTAATAAATTATTAGGTGTTATAGCGATGTTTAGTGCTATTTTAGCTATTATGTTATTACCT
GTTACAGATTTAGGTAGATCTAGAGGTTTACAATTTAGACCATTTAGTAAAATAGCTTTCTGAGTT
TTTGTTGCTAATTTCTTAGTTTTAATGCAATTAGGTGCTAAACACGTTGAAGATCCATTTATATTAT
TAGGTCAATTAAGTACTGTATTATACTTTAGTTATTTTGTTGCTATATTACCTTTAGCTAGTTACTT
AGATAATAGTTTAACTGATTTATCTAATAAATCTGAATTATTTTTAAATAAAACTAACTAAATATAT
TAAGATTATTATTTAATATATTTTCTATTTAAGATACTATTAATTTAGTATTTTGGGTTTTCAGTTTA
TAATTTATATTATATTATGCATTACCCTCCACCTTGCTTTGTAGTAAGCTAATCTGTTATTTCCTTTA
GTTTAATGGTAGAACAATGATCTTCTAATTCATTGGTTTTAGTTCGAATCTAAAAAGGAAATAAG
AAATATATTCTTATTATTACTTATATAATAATTATTTCTTAAAAATATACATTTTGCATTATAGCCGT
TTAGCTGTATTAAAATGTAAAATGATATAAAATAGAATAAATATTTAAATTATTCCTATGTTATATT
ATCCTATATTGCAACCATTATCAGAAGTTGTATTAATACTTGTACCTGCCTTATTAGCTGTAGCTT
ATGTTACAGTTGCTGAAAGAAAAACTATGGCTAGTATGCAAAGAAGATTAGGTCCTAATGCTGT

AGGTTACTATGGACTATTGCAAGCATTTGCTGATGCCTTAAAACTTTTATTAAAAGAATATGTAG
CTCCTACACAATCTAATATTGTTCTTTTCTTTTTAGGTCCTGTAATAACTTTAATTTTTGCATTATTA
GGTTACGCTGTTATACCCTATGGTCCTGGTTCAGGGATAAGCGACATGAATTTAGGTATATTTTA
CATGTTAGCTGTGTCATCTTTAGCTACATACGGTATTCTATTAGCTGGTTGAAGTGCGAATAGTA
AATACGCTTTTCTAGGTTCTCTTAGAAGTACAGCTCAATTAATTAGTTATGAATTAATATTAAGTT
CAGCTATATTAATAGTAATTATGATAACAGGAAATTTAAATTTAACTGTTTGTACTGAATCTCAAA
GAGCTATTTGATTTATACTACCTTTATTTCCTGTGTTTATAATATTTTTCATAGGATCTATAGCTGA
GACAAATAGAGCTCCTTTTGATTTAGCCGAGGCTAACCTGCTAATCTGGTTTGGTCTGGTTATAT
GTCACAAATTGCTAGGAAACCTTTTTATTTTAAAAACAAAAGACAATTAGCAGGAAACTTAATTT
AACCTAATTAAAAATATTAGATAATTAAACTCTTCATAGACTAAACGTGACAATTTAATATATATA
TATATTTATTTATATATATGATTAAATAAGATATAGTCAATCATCGGTGTGAATCGACTTAAAAAA
AAAAGCACATGGGTTAAACCCATCTCCCCTTATTAGGGGAATCAGAACTTGTTAGTGGGTTCAT
GACAGAGCATGCTGCCGTAGTTTTCGTATTCTTCTTTTTAGCTGAGTACGGTAGTATTGTACTAAT
GTGTATTTTAACTAGTATATTATTTATTGGTGGTTACTTATTATTTGAAATATCCTATGTTTTTACTG
TGGTAAATTATATTTTCTTTGAATTATTCTTTATAGACTGAGTAACATTTGTAGAGGTACAATCTTT
ATACACTGATTTTTTAAATAATTCTATCATTGAAGGATTATTATATGGGTTTAATCTAGGATTAAA
AAGTTCTTTAATGATATTCACATTTATTTGAGCTAGAGCATCCTTCCCTAGAATACGATTTGATCA
ACTAATGGGCTTCTGTTGAACAGTTTTATTACCTATTAATTTTGCAATTATTATATTAGTACCTTGT
GTTTTATATAGTTTTAACTTATTACCTGTAAATATACCATTGTTCTAGCTCACACACCCGCCCGCC
CTACTGCCACAAGGCTACAGTACATATGAGGAGGGGAACTAAAGATCTAGAACTATCCTAGTTA
ATAATTACACTTAATAGTATACTAAATAGACCATCTATCATACTCGAGAATAGTGATAGTGTAAT
TATACACTATTTTAACTGTATTCTACACTATTAGGATATTATTATCTTTATATGATAATAATTTTATA
ACTTAATTTATTAGGTTACATTAACAAAAAATTCGTTAACTTTTTTCACCACTTTTTAATACAAAAT
ACGAATTTTA
>YN047
ATAACAATTCTAAAGAACATAAAGAGTTATTAGATAAAAATAATTCACCTATACAGTTAATAAAT
CAACTTAAAGGGTATTTTTTCATAAATCCTTTATTAGCTTTAAGTTTAGCTATTACTATTTTCTCTTT
TGCAGGTATTCCTCCTCTTGTAGGGTTCTTTGCTAAACAGATGGTATTAAGCGCGGCTATTGATC
AAGGTTATATCTTTTTATCTTTAGTTGCAATATTAACTAGTGTTATAGGAGGGGTTTATTATTTAAA
TATAATTAAAGAAATGTTCTTTTATTCACCTGACTATAAATTAAACGAAGAAATTAAAAATAATAC
TATTAATGGTCAAATTATTAATAGAAACAATAAAATATTAAATGTTGAATTTAATTATACAAATGT
AGTTATGTCTAGTTCTGTGGCAATAACTATTTCTACTATTACATTAGTAGTTTTATTATTCATGTTT
ATGAATAAAGAATGATTAAGTCTGGGTACTATATTGGTACAATCTTTATTTAGCTATTAATGAGTA
GTATGACATTATTTATAGGGTTTGTATCTGTTATAGCTATTTTATTTTTAGCCATTAATTTTATATTT
GCTCCTCATAATCCTTATCAAGAAAAATATAGTATTTTCGAGTGTGGTTTCCATAGTTTTTTAGGG
CAAAATAGAACACAATTCGGTATAAAATTCTTTATTTTTGCTTTAGTTTATTTACTTTTAGATTTAG
AAATATTATTAACTTTCCCTTTCGCTCTTAGTGAGTATGTTAATGGTATTTATGGTCTTTTAGTTAC
TTTAATTTTTATAGCTATAATAACTATAGGATTTATATTTGAATTAGGTAAAAGCGCTCTTAAAATA
GACAGCAGACAAAAATTATATATACCTAAATTGAACGTTAATTACCATACAGAGTATGTTGGAAT
AGGTAAGGTTTCTAAGTAAAGTTATAGAGGCAGAAAACCAAAAAACCTACCAAAGGGTAGCTA
ATGGGAAGCTATTAAAAATAAAAGATGATAACCTATATATAGTATAGTTACTATATATTTACTATA
ACTAGGATTATTATATATATATATATTATCTTATTGTATATTAAGATTATTATTATTATAAGGTATA
ATTAATATAGTATCTTATTGTATAAGAATATAATATATTAACCTATAATTAATTTTATTTTTTAATTT
TTAATTATAATTTTTTTTTATATCTAGATGCTTACACATCTACAGATGTAGAAGAGAACAAAATAT

TGTTGTTATAGTAGTGGATGATATAGAAAAATATTTTTATATTATTTATTTTTGTTAGGTAGCTTTT
GAAGTGTTTGATAGAGAGGATATATGGACGGTAGGAGGGTATTCATTTTAATGAACAGTGGATA
GTTTAAATTAACCTAGTTATAGTTTATGAATTTAAATTATAATTATATTAATGTAGGTTATGATAGA
ATTATGTATTGATCCTTAAGAGTTAAGAGAGATACGCCCACGTATAATACATATTGGTTTAGGGT
TGAGTATATATACTTAAGAGTTAAGAATATATATATACAATATATAATAAATATAGTAATATATTA
ATGGTATGGACTTAACCAGGTTTATATATTATCATTTGATAAACATTAATTATAATTTTTCTATTTA
TTATTATTATTATACTGTGAGATTAATAATTATTAAAATATAATATTACATAGTACGTGATATTTGG
GGATTTTATCTTATTAATTGGCAATTAATGATTCTAATCAAATTTTATTCTCTTTAGTTTAATGGTA
GAACAATGATCTTCTAATTCATTGGTTTTAGTTCGATTCTAAAAAGAGATGAGTAAATAATTTTCT
AGATCAGAAATACTACTTTTAACTACAAAAAGCTTACGCTTTTTAACATTTTTTTGATAAATAACA
ACTATTGTTAATATTTGGCTGTCTATTGGTTTAACTAACAATTACAAAATTTTTTCAATTTTTATAAT
ATAAATTTAAATGAGAATATTAAAAAGTCATTCATTATTAAAATTAGTGAATTCTTACCTTATCGA
TGCGTCACAACCAAGTAACATTAGTTACTTGTGAAATTTTGGTTCATTATTAGCTGTTTGTTTAAT
AGTACAAATTATTACCGGTATTACATTAGCTATGCATTATAGTCCTAGTGTAATGGAAGCTTTTAA
CTCAATAGAGCATATAATGAGAGATGTTAATAACGGGTGATTAGTTCGTTATCTACATAGTAATA
CAGCTTCTGCTTTCTTTTTCTTAGTGTATTTACACATAGGAAGAGGTATATATTACGGATCATATA
GAGCTCCTCGTACTTTAGTTTGAGCTATTGGTACTGTTATATTAATATTAATGATGGCTATCGGTT
TCCTAGGTTATGTTTTACCTTATGGACAGATGTCATTATGAGGTGCTACAGTTATTACTAATCTTA
TTAGTGCTATACCTTGAATAGGGCAAGATATTGTTGAATTCATTTGAGGTGGTTTTTCTGTTAATA
ATGCCACTTTAAACAGATTTTTTGCATTACATTTTGTATTGCCTTTTGTATTAGCTGCTTTAGTTTTA
ATGCACTTAATTGCACTTCATGATACTGCTGGTTCAAGCAATCCTCTTGGTGTTTCAGGTAATTAC
GATAGAATTACATTTGCTCCATATTTTTTATTTAAAGATTTAATTACTATTTTTATATTTATTTTTGTA
TTAAGTGCTTTTGTATTCTTTATGCCTAATGTTTTAGGGGATAGTGATAATTATATTATGGCTAATC
CTATGCAAACTCCTGCTGCTATTGTACCTGAATGATACTTATTACCTTTCTATGCTATTTTAAGATC
TATACCTAATAAATTATTAGGTGTTATAGCGATGTTTAGTGCTATTTTAGCTATTATGTTATTACCT
GTTACAGATTTAGGTAGATCTAGAGGTTTACAATTTAGACCATTTAGTAAAATAGCTTTCTGAGTT
TTTGTTGCTAATTTCTTAGTTTTAATGCAATTAGGTGCTAAACACGTTGAAGATCCATTTATATTAT
TAGGTCAATTAAGTACTGTATTATACTTTAGTTATTTTGTTGCTATATTACCTTTAGCTAGTTACTT
AGATAATAGTTTAACTGATTTATCTAATAAATCTGAATTATTTTTAAATAAAACTAACTAAATATAT
TAAGATTATTATTTAATATATTTTCTATTTAAGATACTATTAATTTAGTATTTTGGGTTTTCAGTTTA
TAATTTATATTATATTATGCATTACCCTCCACCTTGCTTTGTAGTAAGCTAATCTGTTATTTCCTTTA
GTTTAATGGTAGAACAATGATCTTCTAATTCATTGGTTTTAGTTCGATTCTAAAAAGGAAATAAGA
AATATATTCTTATTATTACTTATATAATAATTATTTCTTAAAAATATACATTTTGCATTATAGCCGTT
TAGCTGTATTAAAATGTAAAATGATATAAAATAGAATAAATATTTAAATTATTCCTATGTTATATT
ATCCTATATTGCAACCATTATCAGAAGTTGTATTAATACTTGTACCTGCCTTATTAGCTGTAGCTT
ATGTTACAGTTGCTGAAAGAAAAACTATGGCTAGTATGCAAAGAAGATTAGGTCCTAATGCTGT
AGGTTACTATGGACTATTGCAAGCATTTGCTGATGCCTTAAAACTTTTATTAAAAGAATATGTAG
CTCCTACACAATCTAATATTGTTCTTTTCTTTTTAGGTCCTGTAATAACTTTAATTTTTGCATTATTA
GGTTACGCTGTTATACCCTATGGTCCTGGTTCAGGGATAAGCGACATGAATTTAGGTATATTTTA
CATGTTAGCTGTGTCATCTTTAGCTACATACGGTATTCTATTAGCTGGTTGAAGTGCGAATAGTA
AATACGCTTTTCTAGGTTCTCTTAGAAGTACAGCTCAATTAATTAGTTATGAATTAATATTAAGTT
CAGCTATATTAATAGTAATTATGATAACAGGAAATTTAAATTTAACTGTTTGTACTGAATCTCAAA
GAGCTATTTGATTTATACTACCTTTATTTCCTGTGTTTATAATATTTTTCATAGGATCTATAGCTGA
GACAAATAGAGCTCCTTTTGATTTAGCCGAGGCTAACCTGCTAATCTGGTTTGGTCTGGTTATAT

GTCACAAATTGCTAGGAAACCTTTTTATTTTAAAAACAAAAGACAATTAGCAGGAAACTTAATTT
AACCTAATTAAAAATATTAGATAATTAAACTCTTCATAGACTAAACGTGACAATTTAATATATATA
TATATTTATTTATATATATGATTAAATAAGATATAGTCAATCATCGGTGTGAATCGACTTAAAAAA
AAAAGCACATGGGTTAAACCCATCTCCCCTTATTAGGGGAATCAGAACTTGTTAGTGGGTTCAT
GACAGAGCATGCTGCCGTAGTTTTCGTATTCTTCTTTTTAGCTGAGTACGGTAGTATTGTACTAAT
GTGTATTTTAACTAGTATATTATTTATTGGTGGTTACTTATTATTTGAAATATCCTATGTTTTTACTG
TGGTAAATTATATTTTCTTTGAATTATTCTTTATAGACTGAGTAACATTTGTAGAGGTACAATCTTT
ATACACTGATTTTTTAAATAATTCTATCATTGAAGGATTATTATATGGGTTTAATCTAGGATTAAA
AAGTTCTTTAATGATATTCACATTTATTTGAGCTAGAGCATCCTTCCCTAGAATACGATTTGATCA
ACTAATGGGCTTCTGTTGAACAGTTTTATTACCTATTAATTTTGCAATTATTATATTAGTACCTTGT
GTTTTATATAGTTTTAACTTATTACCTGTAAATATACCATTGTTCTAGCTCACACACCCGCCCGCC
CTACTGCCACAAGGCTACAGTACATATGAGGAGGGGAACTAAAGATCTAGAACTATCCTAGTTA
ATAATTACACTTAATAGTATACTAAATAGACCATCTATCATACTCGAGAATAGTGATAGTGTAAT
TATACACTATTTTAACTGTATTCTACACTATTAGCATATTATTATCTTTATATGATAATAATTTTATA
ACTTAATTTATTAGGTTACATTAACAAAAAAATTCGTTAACTTTTTTCACCACTTTTTAATACAAAAT
ACGAATTTTA
>YN084
ATAACAATTCTAAAGAACATAAAGAGTTATTAGATAAAAATAATTCACCTATACAGTTAATAAAT
CAACTTAAAGGGTATTTTTTCATAAATCCTTTATTAGCTTTAAGTTTAGCTATTACTATTTTCTCTTT
TGCAGGTATTCCTCCTCTTGTAGGGTTCTTTGCTAAACAGATGGTATTAAGCGCGGCTATTGATC
AAGGTTATATCTTTTTATCTTTAGTTGCAATATTAACTAGTGTTATAGGAGGGGTTTATTATTTAAA
TATAATTAAAGAAATGTTCTTTTATTCACCTGACTATAAATTAAACGAAGAAATTAAAAATAATAC
TATTAATGGTCAAATTATTAATAGAAACAATAAAATATTAAATGTTGAATTTAATTATACAAATGT
AGTTATGTCTAGTTCTGTGGCAATAACTATTTCTACTATTACATTAGTAGTTTTATTATTCATGTTT
ATGAATAAAGAATGATTAAGTCTGGGTACTATATTGGTACAATCTTTATTTAGCTATTAATGAGTA
GTATGACATTATTTATAGGGTTTGTATCTGTTATAGCTATTTTATTTTTAGCCATTAATTTTATATTT
GCTCCTCATAATCCTTATCAAGAAAAATATAGTATTTTCGAGTGTGGTTTCCATAGTTTTTTAGGG
CAAAATAGAACACAATTCGGTATAAAATTCTTTATTTTTGCTTTAGTTTATTTACTTTTAGATTTAG
AAATATTATTAACTTTCCCTTTCGCTCTTAGTGAGTATGTTAATGGTATTTATGGTCTTTTAGTTAC
TTTAATTTTTATAGCTATAATAACTATAGGATTTATATTTGAATTAGGTAAAAGCGCTCTTAAAATA
GACAGCAGACAAAAATTATATATACCTAAATTGAACGTTAATTACCATACAGAGTATGTTGGAAT
AGGTAAGGTTTCTAAGTAAAGTTATAGAGGCAGAAAACCAAAAAACCTACCAAAGGGTAGCTA
ATGGGAAGCTATTAAAAATAAAAGATGATAACCTATATATAGTATAGTTACTATATATTTACTATA
ACTAGGATTATTATATATATATATATATTATCTTATTGTATATTAAGATTATTATTATTATAAGGTATA
ATTAATATAGTATCTTATTGTATAAGAATATAATATATTAACCTATAATTAATTTTATTTTTTAATTT
TTAATTATAATTTTTTTTTATATCTAGATGCTTACACATCTACAGATGTAGAAGAGAACAAAATAT
TGTTGTTATAGTAGTGGATGATATAGAAAAATATTTTTATATTATTTATTTTTGTTAGGTAGCTTTT
GAAGTGTTTGATAGAGAGGATATATGGACGGTAGGAGGGTATTCATTTTAATGAACAGTGGATA
GTTTAAATTAACCTAGTTATAGTTTATGAATTTAAATTATAATTATATTAATGTAGGTTATGATAGA
ATTATGTATTGATCCTTAAGAGTTAAGAGAGATACGCCCACGTATAATACATATTGGTTTAGGGT
TGAGTATATATACTTAAGAGTTAAGAATATATATATACAATATATAATAAATATAGTAATATATTA
ATGGTATGGACTTAACCAGGTTTATATATTATCATTTGATAAACATTAATTATAATTTTTCTATTTA
TTATTATTATTATACTGTGAGATTAATAATTATTAAAATATAATATTACATAGTACGTGATATTTGG
GGATTTTATCTTATTAATTGGCAATTAATGATTCTAATCAAATTTTATTCTCTTTAGTTTAATGGTA

GAACAATGATCTTCTAATTCATTGGTTTTAGTTCGATTCTAAAAAGAGATGAGTAAATAATTTTCT
AGATCAGAAATACTACTTTTAACTACAAAAAGCTTACGCTTTTTAACATTTTTTTGATAAATAACA
ACTATTGTTAATATTTGGCTGTCTATTGGTTTAACTAACAATTACAAAATTTTTTCAATTTTTATAAT
ATAAATTTAAATGAGAATATTAAAAAGTCATTCATTATTAAAATTAGTGAATTCTTACCTTATCGA
TGCGTCACAACCAAGTAACATTAGTTACTTGTGAAATTTTGGTTCATTATTAGCTGTTTGTTTAAT
AGTACAAATTATTACCGGTATTACATTAGCTATGCATTATAGTCCTAGTGTAATGGAAGCTTTTAA
CTCAATAGAGCATATAATGAGAGATGTTAATAACGGGTGATTAGTTCGTTATCTACATAGTAATA
CAGCTTCTGCTTTCTTTTTCTTAGTGTATTTACACATAGGAAGAGGTATATATTACGGATCATATA
GAGCTCCTCGTACTTTAGTTTGAGCTATTGGTACTGTTATATTAATATTAATGATGGCTATCGGTT
TCCTAGGTTATGTTTTACCTTATGGACAGATGTCATTATGAGGTGCTACAGTTATTACTAATCTTA
TTAGTGCTATACCTTGAATAGGGCAAGATATTGTTGAATTCATTTGAGGTGGTTTTTCTGTTAATA
ATGCCACTTTAAACAGATTTTTTGCATTACATTTTGTATTGCCTTTTGTATTAGCTGCTTTAGTTTTA
ATGCACTTAATTGCACTTCATGATACTGCTGGTTCAAGCAATCCTCTTGGTGTTTCAGGTAATTAC
GATAGAATTACATTTGCTCCATATTTTTTATTTAAAGATTTAATTACTATTTTTATATTTATTTTTGTA
TTAAGTGCTTTTGTATTCTTTATGCCTAATGTTTTAGGGGATAGTGATAATTATATTATGGCTAATC
CTATGCAAACTCCTGCTGCTATTGTACCTGAATGATACTTATTACCTTTCTATGCTATTTTAAGATC
TATACCTAATAAATTATTAGGTGTTATAGCGATGTTTAGTGCTATTTTAGCTATTATGTTATTACCT
GTTACAGATTTAGGTAGATCTAGAGGTTTACAATTTAGACCATTTAGTAAAATAGCTTTCTGAGTT
TTTGTTGCTAATTTCTTAGTTTTAATGCAATTAGGTGCTAAACACGTTGAAGATCCATTTATATTAT
TAGGTCAATTAAGTACTGTATTATACTTTAGTTATTTTGTTGCTATATTACCTTTAGCTAGTTACTT
AGATAATAGTTTAACTGATTTATCTAATAAATCTGAATTATTTTTAAATAAAACTAACTAAATATAT
TAAGATTATTATTTAATATATTTTCTATTTAAGATACTATTAATTTAGTATTTTGGGTTTTCAGTTTA
TAATTTATATTATATTATGCATTACCCTCCACCTTGCTTTGTAGTAAGCTAATCTGTTATTTCCTTTA
GTTTAATGGTAGAACAATGATCTTCTAATTCATTGGTTTTAGTTCGAATCTAAAAAGGAAATAAG
AAATATATTCTTATTATTACTTATATAATAATTATTTCTTAAAAATATACATTTTGCATTATAGCCGT
TTAGCTGTATTAAAATGTAAAATGATATAAAATAGAATAAATATTTAAATTATTCCTATGTTATATT
ATCCTATATTGCAACCATTATCAGAAGTTGTATTAATACTTGTACCTGCCTTATTAGCTGTAGCTT
ATGTTACAGTTGCTGAAAGAAAAACTATGGCTAGTATGCAAAGAAGATTAGGTCCTAATGCTGT
AGGTTACTATGGACTATTGCAAGCATTTGCTGATGCCTTAAAACTTTTATTAAAAGAATATGTAG
CTCCTACACAATCTAATATTGTTCTTTTCTTTTTAGGTCCTGTAATAACTTTAATTTTTGCATTATTA
GGTTACGCTGTTATACCCTATGGTCCTGGTTCAGGGATAAGCGACATGAATTTAGGTATATTTTA
CATGTTAGCTGTGTCATCTTTAGCTACATACGGTATTCTATTAGCTGGTTGAAGTGCGAATAGTA
AATACGCTTTTCTAGGTTCTCTTAGAAGTACAGCTCAATTAATTAGTTATGAATTAATATTAAGTT
CAGCTATATTAATAGTAATTATGATAACAGGAAATTTAAATTTAACTGTTTGTACTGAATCTCAAA
GAGCTATTTGATTTATACTACCTTTATTTCCTGTGTTTATAATATTTTTCATAGGATCTATAGCTGA
GACAAATAGAGCTCCTTTTGATTTAGCCGAGGCTAACCTGCTAATCTGGTTTGGTCTGGTTATAT
GTCACAAATTGCTAGGAAACCTTTTTATTTTAAAAACAAAAGACAATTAGCAGGAAACTTAATTT
AACCTAATTAAAAATATTAGATAATTAAACTCTTCATAGACTAAACGTGACAATTTAATATATATA
TATATTTATTTATATATATGATTAAATAAGATATAGTCAATCATCGGTGTGAATCGACTTAAAAAA
AAAAGCACATGGGTTAAACCCATCTCCCCTTATTAGGGGAATCAGAACTTGTTAGTGGGTTCAT
GACAGAGCATGCTGCCGTAGTTTTCGTATTCTTCTTTTTAGCTGAGTACGGTAGTATTGTACTAAT
GTGTATTTTAACTAGTATATTATTTATTGGTGGTTACTTATTATTTGAAATATCCTATGTTTTTACTG
TGGTAAATTATATTTTCTTTGAATTATTCTTTATAGACTGAGTAACATTTGTAGAGGTACAATCTTT
ATACACTGATTTTTTAAATAATTCTATCATTGAAGGATTATTATATGGGTTTAATCTAGGATTAAA

AAGTTCTTTAATGATATTCACATTTATTTGAGCTAGAGCATCCTTCCCTAGAATACGATTTGATCA
ACTAATGGGCTTCTGTTGAACAGTTTTATTACCTATTAATTTTGCAATTATTATATTAGTACCTTGT
GTTTTATATAGTTTTAACTTATTACCTGTAAATATACCATTGTTCTAGCTCACACACCCGCCCGCC
CTACTGCCACAAGGCTACAGTACATATGAGGAGGGGAACTAAAGATCTAGAACTATCCTAGTTA
ATAATTACACTTAATAGTATACTAAATAGACCATCTATCATACTCGAGAATAGTGATAGTGTAAT
TATACACTATTTTAACTGTATTCTACACTATTAGCATATTATTATCTTTATATGATAATAATTTTATA
ACTTAATTTATTAGGTTACATTAACAAAAAAATTCGTTAACTTTTTTCACCACTTTTTAATACAAAAT
ACGAATTTTA
>YN093
ATATCAATTCTAAAGAACATAAAGAGTTATTAGATAAAAATAATTCACCTATACAGTTAATAAAT
CAACTTAAAGGGTATTTTTTCATAAATCCTTTATTAGCTTTAAGTTTAGCTATTACTATTTTCTCTTT
TGCAGGTATTCCTCCTCTTGTAGGGTTCTTTGCTAAACAGATGGTATTAAGCGCGGCTATTGATC
AAGGTTATATCTTTTTATCTTTAGTTGCAATATTAACTAGTGTTATAGGAGGGGTTTATTATTTAAA
TATAATTAAAGAAATGTTCTTTTATTCACCTGACTATAAATTAAACGAAGAAATTAAAAATAATAC
TATTAATGGTCAAATTATTAATAGAAACAATAAAATATTAAATGTTGAATTTAATTATACAAATGT
AGTTATGTCTAGTTCTGTGGCAATAACTATTTCTACTATTACATTAGTAGTTTTATTATTCATGTTT
ATGAATAAAGAATGATTAAGTCTGGGTACTATATTGGTACAATCTTTATTTAGCTATTAATGAGTA
GTATGACATTATTTATAGGGTTTGTATCTGTTATAGCTATTTTATTTTTAGCCATTAATTTTATATTT
GCTCCTCATAATCCTTATCAAGAAAAATATAGTATTTTCGAGTGTGGTTTCCATAGTTTTTTAGGG
CAAAATAGAACACAATTCGGTATAAAATTCTTTATTTTTGCTTTAGTTTATTTACTTTTAGATTTAG
AAATATTATTAACTTTCCCTTTCGCTCTTAGTGAGTATGTTAATGGTATTTATGGTCTTTTAGTTAC
TTTAATTTTTATAGCTATAATAACTATAGGATTTATATTTGAATTAGGTAAAAGCGCTCTTAAAATA
GACAGCAGACAAAAATTATATATACCTAAATTGAACGTTAATTACCATACAGAGTATGTTGGAAT
AGGTAAGGTTTCTAAGTAAAGTTATAGAGGCAGAAAACCAAAAAACCTACCAAAGGGTAGCTA
ATGGGAAGCTATTAAAAATAAAAGATGATAACCTATATATAGTATAGTTACTATATATTTACTATA
ACTAGGATTATTATATATATATATATTATCTTATTGTATATTAAGATTATTATTATTATAAGGTATA
ATTAATATAGTATCTTATTGTATAAGAATATAATATATTAACCTATAATTAATTTTATTTTTTAATTT
TTAATTATAATTTTTTTTTATATCTAGATGCTTACACATCTACAGATGTAGAAGAGAACAAAATAT
TGTTGTTATAGTAGTGGATGATATAGAAAAATATTTTTATATTATTTATTTTTGTTAGGTAGCTTTT
GAAGTGTTTGATAGAGAGGATATATGGACGGTAGGAGGGTATTCATTTTAATGAACAGTGGATA
GTTTAAATTAACCTAGTTATAGTTTATGAATTTAAATTATAATTATATTAATGTAGGTTATGATAGA
ATTATGTATTGATCCTTAAGAGTTAAGAGAGATACGCCCACGTATAATACATATTGGTTTAGGGT
TGAGTATATATACTTAAGAGTTAAGAATATATATATACAATATATAATAAATATAGTAATATATTA
ATGGTATGGACTTAACCAGGTTTATATATTATCATTTGATAAACATTAATTATAATTTTTCTATTTA
TTATTATTATTATACTGTGAGATTAATAATTATTAAAATATAATATTACATAGTACGTGATATTTGG
GGATTTTATCTTATTAATTGGCAATTAATGATTCTAATCAAATTTTATTCTCTTTAGTTTAATGGTA
GAACAATGATCTTCTAATTCATTGGTTTTAGTTCGATTCTAAAAAGAGATGAGTAAATAATTTTCT
AGATCAGAAATACTACTTTTAACTACAAAAAGCTTACGCTTTTTAACATTTTTTTGATAAATAACA
ACTATTGTTAATATTTGGCTGTCTATTGGTTTAACTAACAATTACAAAATTTTTTCAATTTTTATAAT
ATAAATTTAAATGAGAATATTAAAAAGTCATTCATTATTAAAATTAGTGAATTCTTACCTTATCGA
TGCGTCACAACCAAGTAACATTAGTTACTTGTGAAATTTTGGTTCATTATTAGCTGTTTGTTTAAT
AGTACAAATTATTACCGGTATTACATTAGCTATGCATTATAGTCCTAGTGTAATGGAAGCTTTTAA
CTCAATAGAGCATATAATGAGAGATGTTAATAACGGGTGATTAGTTCGTTATCTACATAGTAATA
CAGCTTCTGCTTTCTTTTTCTTAGTGTATTTACACATAGGAAGAGGTATATATTACGGATCATATA

GAGCTCCTCGTACTTTAGTTTGAGCTATTGGTACTGTTATATTAATATTAATGATGGCTATCGGTT
TCCTAGGTTATGTTTTACCTTATGGACAGATGTCATTATGAGGTGCTACAGTTATTACTAATCTTA
TTAGTGCTATACCTTGAATAGGGCAAGATATTGTTGAATTCATTTGAGGTGGTTTTTCTGTTAATA
ATGCCACTTTAAACAGATTTTTTGCATTACATTTTGTATTGCCTTTTGTATTAGCTGCTTTAGTTTTA
ATGCACTTAATTGCACTTCATGATACTGCTGGTTCAAGCAATCCTCTTGGTGTTTCAGGTAATTAC
GATAGAATTACATTTGCTCCATATTTTTTATTTAAAGATTTAATTACTATTTTTATATTTATTTTTGTA
TTAAGTGCTTTTGTATTCTTTATGCCTAATGTTTTAGGGGATAGTGATAATTATATTATGGCTAATC
CTATGCAAACTCCTGCTGCTATTGTACCTGAATGATACTTATTACCTTTCTATGCTATTTTAAGATC
TATACCTAATAAATTATTAGGTGTTATAGCGATGTTTAGTGCTATTTTAGCTATTATGTTATTACCT
GTTACAGATTTAGGTAGATCTAGAGGTTTACAATTTAGACCATTTAGTAAAATAGCTTTCTGAGTT
TTTGTTGCTAATTTCTTAGTTTTAATGCAATTAGGTGCTAAACACGTTGAAGATCCATTTATATTAT
TAGGTCAATTAAGTACTGTATTATACTTTAGTTATTTTGTTGCTATATTACCTTTAGCTAGTTACTT
AGATAATAGTTTAACTGATTTATCTAATAAATCTGAATTATTTTTAAATAAAACTAACTAAATATAT
TAAGATTATTATTTAATATATTTTCTATTTAAGATACTATTAATTTAGTATTTTGGGTTTTCAGTTTA
TAATTTATATTATATTATGCATTACCCTCCACCTTGCTTTGTAGTAAGCTAATCTGTTATTTCCTTTA
GTTTAATGGTAGAACAATGATCTTCTAATTCATTGGTTTTAGTTCGAATCTAAAAAGGAAATAAG
AAATATATTCTTATTATTACTTATATAATAATTATTTCTTAAAAATATACATTTTGCATTATAGCCGT
TTAGCTGTATTAAAATGTAAAATGATATAAAATAGAATAAATATTTAAATTATTCCTATGTTATATT
ATCCTATATTGCAACCATTATCAGAAGTTGTATTAATACTTGTACCTGCCTTATTAGCTGTAGCTT
ATGTTACAGTTGCTGAAAGAAAAACTATGGCTAGTATGCAAAGAAGATTAGGTCCTAATGCTGT
AGGTTACTATGGACTATTGCAAGCATTTGCTGATGCCTTAAAACTTTTATTAAAAGAATATGTAG
CTCCTACACAATCTAATATTGTTCTTTTCTTTTTAGGTCCTGTAATAACTTTAATTTTTGCATTATTA
GGTTACGCTGTTATACCCTATGGTCCTGGTTCAGGGATAAGCGACATGAATTTAGGTATATTTTA
CATGTTAGCTGTGTCATCTTTAGCTACATACGGTATTCTATTAGCTGGTTGAAGTGCGAATAGTA
AATACGCTTTTCTAGGTTCTCTTAGAAGTACAGCTCAATTAATTAGTTATGAATTAATATTAAGTT
CAGCTATATTAATAGTAATTATGATAACAGGAAATTTAAATTTAACTGTTTGTACTGAATCTCAAA
GAGCTATTTGATTTATACTACCTTTATTTCCTGTGTTTATAATATTTTTCATAGGATCTATAGCTGA
GACAAATAGAGCTCCTTTTGATTTAGCCGAGGCTAACCTGCTAATCTGGTTTGGTCTGGTTATAT
GTCACAAATTGCTAGGAAACCTTTTTATTTTAAAAACAAAAGACAATTAGCAGGAAACTTAATTT
AACCTAATTAAAAATATTAGATAATTAAACTCTTCATAGACTAAACGTGACAATTTAATATATATA
TATATTTATTTATATATATGATTAAATAAGATATAGTCAATCATCGGTGTGAATCGACTTAAAAAA
AAAAGCACATGGGTTAAACCCATCTCCCCTTATTAGGGGAATCAGAACTTGTTAGTGGGTTCAT
GACAGAGCATGCTGCCGTAGTTTTCGTATTCTTCTTTTTAGCTGAGTACGGTAGTATTGTACTAAT
GTGTATTTTAACTAGTATATTATTTATTGGTGGTTACTTATTATTTGAAATATCCTATGTTTTTACTG
TGGTAAATTATATTTTCTTTGAATTATTCTTTATAGACTGAGTAACATTTGTAGAGGTACAATCTTT
ATACACTGATTTTTTAAATAATTCTATCATTGAAGGATTATTATATGGGTTTAATCTAGGATTAAA
AAGTTCTTTAATGATATTCACATTTATTTGAGCTAGAGCATCCTTCCCTAGAATACGATTTGATCA
ACTAATGGGCTTCTGTTGAACAGTTTTATTACCTATTAATTTTGCAATTATTATATTAGTACCTTGT
GTTTTATATAGTTTTAACTTATTACCTGTAAATATACCATTGTTCTAGCTCACACACCCGCCCGCC
CTACTGCCACAAGGCTACAGTACATATGAGGAGGGGAACTAAAGATCTAGAACTATCCTAGTTA
ATAATTACACTTAATAGTATACTAAATAGACCATCTATCATACTCGAGAATAGTGATAGTGTAAT
TATACACTATTTTAACTGTATTCTACACTATTAGGATATTATTATCTTTATATGATAATAATTTTATA
ACTTAATTTATTAGGTTACATTAACAAAAAATTCGTTAACTTTTTTCACCACTTTTTAATACAAAAT
ACGAATTTTA

>YN094
ATAACAATTCTAAAGAACATAAAGAGTTATTAGATAAAAATAATTCACCTATACAGTTAATAAAT
CAACTTAAAGGGTATTTTTTCATAAATCCTTTATTAGCTTTAAGTTTAGCTATTACTATTTTCTCTTT
TGCAGGTATTCCTCCTCTTGTAGGGTTCTTTGCTAAACAGATGGTATTAAGCGCGGCTATTGATC
AAGGTTATATCTTTTTATCTTTAGTTGCAATATTAACTAGTGTTATAGGAGGGGTTTATTATTTAAA
TATAATTAAAGAAATGTTCTTTTATTCACCTGACTATAAATTAAACGAAGAAATTAAAAATAATAC
TATTAATGGTCAAATTATTAATAGAAACAATAAAATATTAAATGTTGAATTTAATTATACAAATGT
AGTTATGTCTAGTTCTGTGGCAATAACTATTTCTACTATTACATTAGTAGTTTTATTATTCATGTTT
ATGAATAAAGAATGATTAAGTCTGGGTACTATATTGGTACAATCTTTATTTAGCTATTAATGAGTA
GTATGACATTATTTATAGGGTTTGTATCTGTTATAGCTATTTTATTTTTAGCCATTAATTTTATATTT
GCTCCTCATAATCCTTATCAAGAAAAATATAGTATTTTCGAGTGTGGTTTCCATAGTTTTTTAGGG
CAAAATAGAACACAATTCGGTATAAAATTCTTTATTTTTGCTTTAGTTTATTTACTTTTAGATTTAG
AAATATTATTAACTTTCCCTTTCGCTCTTAGTGAGTATGTTAATGGTATTTATGGTCTTTTAGTTAC
TTTAATTTTTATAGCTATAATAACTATAGGATTTATATTTGAATTAGGTAAAAGCGCTCTTAAAATA
GACAGCAGACAAAAATTATATATACCTAAATTGAACGTTAATTACCATACAGAGTATGTTGGAAT
AGGTAAGGTTTCTAAGTAAAGTTATAGAGGCAGAAAACCAAAAAACCTACCAAAGGGTAGCTA
ATGGGAAGCTATTAAAAATAAAAGATGATAACCTATATATAGTATAGTTACTATATATTTACTATA
ACTAGGATTATTATATATATATATATTATCTTATTGTATATTAAGATTATTATTATTATAAGGTATA
ATTAATATAGTATCTTATTGTATAAGAATATAATATATTAACCTATAATTAATTTTATTTTTTAATTT
TTAATTATAATTTTTTTTTATATCTAGATGCTTACACATCTACAGATGTAGAAGAGAACAAAATAT
TGTTGTTATAGTAGTGGATGATATAGAAAAATATTTTTATATTATTTATTTTTGTTAGGTAGCTTTT
GAAGTGTTTGATAGAGAGGATATATGGACGGTAGGAGGGTATTCATTTTAATGAACAGTGGATA
GTTTAAATTAACCTAGTTATAGTTTATGAATTTAAATTATAATTATATTAATGTAGGTTATGATAGA
ATTATGTATTGATCCTTAAGAGTTAAGAGAGATACGCCCACGTATAATACATATTGGTTTAGGGT
TGAGTATATATACTTAAGAGTTAAGAATATATATATACAATATATAATAAATATAGTAATATATTA
ATGGTATGGACTTAACCAGGTTTATATATTATCATTTGATAAACATTAATTATAATTTTTCTATTTA
TTATTATTATTATACTGTGAGATTAATAATTATTAAAATATAATATTACATAGTACGTGATATTTGG
GGATTTTATCTTATTAATTGGCAATTAATGATTCTAATCAAATTTTATTCTCTTTAGTTTAATGGTA
GAACAATGATCTTCTAATTCATTGGTTTTAGTTCGATTCTAAAAAGAGATGAGTAAATAATTTTCT
AGATCAGAAATACTACTTTTAACTACAAAAAGCTTACGCTTTTTAACATTTTTTTGATAAATAACA
ACTATTGTTAATATTTGGCTGTCTATTGGTTTAACTAACAATTACAAAATTTTTTCAATTTTTATAAT
ATAAATTTAAATGAGAATATTAAAAAGTCATTCATTATTAAAATTAGTGAATTCTTACCTTATCGA
TGCGTCACAACCAAGTAACATTAGTTACTTGTGAAATTTTGGTTCATTATTAGCTGTTTGTTTAAT
AGTACAAATTATTACCGGTATTACATTAGCTATGCATTATAGTCCTAGTGTAATGGAAGCTTTTAA
CTCAATAGAGCATATAATGAGAGATGTTAATAACGGGTGATTAGTTCGTTATCTACATAGTAATA
CAGCTTCTGCTTTCTTTTTCTTAGTGTATTTACACATAGGAAGAGGTATATATTACGGATCATATA
GAGCTCCTCGTACTTTAGTTTGAGCTATTGGTACTGTTATATTAATATTAATGATGGCTATCGGTT
TCCTAGGTTATGTTTTACCTTATGGACAGATGTCATTATGAGGTGCTACAGTTATTACTAATCTTA
TTAGTGCTATACCTTGAATAGGGCAAGATATTGTTGAATTCATTTGAGGTGGTTTTTCTGTTAATA
ATGCCACTTTAAACAGATTTTTTGCATTACATTTTGTATTGCCTTTTGTATTAGCTGCTTTAGTTTTA
ATGCACTTAATTGCACTTCATGATACTGCTGGTTCAAGCAATCCTCTTGGTGTTTCAGGTAATTAC
GATAGAATTACATTTGCTCCATATTTTTTATTTAAAGATTTAATTACTATTTTTATATTTATTTTTGTA
TTAAGTGCTTTTGTATTCTTTATGCCTAATGTTTTAGGGGATAGTGATAATTATATTATGGCTAATC
CTATGCAAACTCCTGCTGCTATTGTACCTGAATGATACTTATTACCTTTCTATGCTATTTTAAGATC

TATACCTAATAAATTATTAGGTGTTATAGCGATGTTTAGTGCTATTTTAGCTATTATGTTATTACCT
GTTACAGATTTAGGTAGATCTAGAGGTTTACAATTTAGACCATTTAGTAAAATAGCTTTCTGAGTT
TTTGTTGCTAATTTCTTAGTTTTAATGCAATTAGGTGCTAAACACGTTGAAGATCCATTTATATTAT
TAGGTCAATTAAGTACTGTATTATACTTTAGTTATTTTGTTGCTATATTACCTTTAGCTAGTTACTT
AGATAATAGTTTAACTGATTTATCTAATAAATCTGAATTATTTTTAAATAAAACTAACTAAATATAT
TAAGATTATTATTTAATATATTTTCTATTTAAGATACTATTAATTTAGTATTTTGGGTTTTCAGTTTA
TAATTTATATTATATTATGCATTACCCTCCACCTTGCTTTGTAGTAAGCTAATCTGTTATTTCCTTTA
GTTTAATGGTAGAACAATGATCTTCTAATTCATTGGTTTTAGTTCGAATCTAAAAAGGAAATAAG
AAATATATTCTTATTATTACTTATATAATAATTATTTCTTAAAAATATACATTTTGCATTATAGCCGT
TTAGCTGTATTAAAATGTAAAATGATATAAAATAGAATAAATATTTAAATTATTCCTATGTTATATT
ATCCTATATTGCAACCATTATCAGAAGTTGTATTAATACTTGTACCTGCCTTATTAGCTGTAGCTT
ATGTTACAGTTGCTGAAAGAAAAACTATGGCTAGTATGCAAAGAAGATTAGGTCCTAATGCTGT
AGGTTACTATGGACTATTGCAAGCATTTGCTGATGCCTTAAAACTTTTATTAAAAGAATATGTAG
CTCCTACACAATCTAATATTGTTCTTTTCTTTTTAGGTCCTGTAATAACTTTAATTTTTGCATTATTA
GGTTACGCTGTTATACCCTATGGTCCTGGTTCAGGGATAAGCGACATGAATTTAGGTATATTTTA
CATGTTAGCTGTGTCATCTTTAGCTACATACGGTATTCTATTAGCTGGTTGAAGTGCGAATAGTA
AATACGCTTTTCTAGGTTCTCTTAGAAGTACAGCTCAATTAATTAGTTATGAATTAATATTAAGTT
CAGCTATATTAATAGTAATTATGATAACAGGAAATTTAAATTTAACTGTTTGTACTGAATCTCAAA
GAGCTATTTGATTTATACTACCTTTATTTCCTGTGTTTATAATATTTTTCATAGGATCTATAGCTGA
GACAAATAGAGCTCCTTTTGATTTAGCCGAGGCTAACCTGCTAATCTGGTTTGGTCTGGTTATAT
GTCACAAATTGCTAGGAAACCTTTTTATTTTAAAAACAAAAGACAATTAGCAGGAAACTTAATTT
AACCTAATTAAAAATATTAGATAATTAAACTCTTCATAGACTAAACGTGACAATTTAATATATATA
TATATTTATTTATATATATGATTAAATAAGATATAGTCAATCATCGGTGTGAATCGACTTAAAAAA
AAAAGCACATGGGTTAAACCCATCTCCCCTTATTAGGGGAATCAGAACTTGTTAGTGGGTTCAT
GACAGAGCATGCTGCCGTAGTTTTCGTATTCTTCTTTTTAGCTGAGTACGGTAGTATTGTACTAAT
GTGTATTTTAACTAGTATATTATTTATTGGTGGTTACTTATTATTTGAAATATCCTATGTTTTTACTG
TGGTAAATTATATTTTCTTTGAATTATTCTTTATAGACTGAGTAACATTTGTAGAGGTACAATCTTT
ATACACTGATTTTTTAAATAATTCTATCATTGAAGGATTATTATATGGGTTTAATCTAGGATTAAA
AAGTTCTTTAATGATATTCACATTTATTTGAGCTAGAGCATCCTTCCCTAGAATACGATTTGATCA
ACTAATGGGCTTCTGTTGAACAGTTTTATTACCTATTAATTTTGCAATTATTATATTAGTACCTTGT
GTTTTATATAGTTTTAACTTATTACCTGTAAATATACCATTGTTCTAGCTCACACACCCGCCCGCC
CTACTGCCACAAGGCTACAGTACATATGAGGAGGGGAACTAAAGATCTAGAACTATCCTAGTTA
ATAATTACACTTAATAGTATACTAAATAGACCATCTATCATACTCGAGAATAGTGATAGTGTAAT
TATACACTATTTTAACTGTATTCTACACTATTAGGATATTATTATCTTTATATGATAATAATTTTATA
ACTTAATTTATTAGGTTACATTAACAAAAAATTCGTTAACTTTTTTCACCACTTTTTAATACAAAAT
ACGAATTTTA
>YN097
ATAACAATTCTAAAGAACATAAAGAGTTATTAGATAAAAATAATTCACCTATACAGTTAATAAAT
CAACTTAAAGGGTATTTTTTCATAAATCCTTTATTAGCTTTAAGTTTAGCTATTACTATTTTCTCTTT
TGCAGGTATTCCTCCTCTTGTAGGGTTCTTTGCTAAACAGATGGTATTAAGCGCGGCTATTGATC
AAGGTTATATCTTTTTATCTTTAGTTGCAATATTAACTAGTGTTATAGGAGGGGTTTATTATTTAAA
TATAATTAAAGAAATGTTCTTTTATTCACCTGACTATAAATTAAACGAAGAAATTAAAAATAATAC
TATTAATGGTCAAATTATTAATAGAAACAATAAAATATTAAATGTTGAATTTAATTATACAAATGT
AGTTATGTCTAGTTCTGTGGCAATAACTATTTCTACTATTACATTAGTAGTTTTATTATTCATGTTT

ATGAATAAAGAATGATTAAGTCTGGGTACTATATTGGTACAATCTTTATTTAGCTATTAATGAGTA
GTATGACATTATTTATAGGGTTTGTATCTGTTATAGCTATTTTATTTTTAGCCATTAATTTTATATTT
GCTCCTCATAATCCTTATCAAGAAAAATATAGTATTTTCGAGTGTGGTTTCCATAGTTTTTTAGGG
CAAAATAGAACACAATTCGGTATAAAATTCTTTATTTTTGCTTTAGTTTATTTACTTTTAGATTTAG
AAATATTATTAACTTTCCCTTTCGCTCTTAGTGAGTATGTTAATGGTATTTATGGTCTTTTAGTTAC
TTTAATTTTTATAGCTATAATAACTATAGGATTTATATTTGAATTAGGTAAAAGCGCTCTTAAAATA
GACAGCAGACAAAAATTATATATACCTAAATTGAACGTTAATTACCATACAGAGTATGTTGGAAT
AGGTAAGGTTTCTAAGTAAAGTTATAGAGGCAGAAAACCAAAAAACCTACCAAAGGGTAGCTA
ATGGGAAGCTATTAAAAATAAAAGATGATAACCTATATATAGTATAGTTACTATATATTTACTATA
ACTAGGATTATTATATATATATATATTATCTTATTGTATATTAAGATTATTATTATTATAAGGTATA
ATTAATATAGTATCTTATTGTATAAGAATATAATATATTAACCTATAATTAATTTTATTTTTTAATTT
TTAATTATAATTTTTTTTTATATCTAGATGCTTACACATCTACAGATGTAGAAGAGAACAAAATAT
TGTTGTTATAGTAGTGGATGATATAGAAAAATATTTTTATATTATTTATTTTTGTTAGGTAGCTTTT
GAAGTGTTTGATAGAGAGGATATATGGACGGTAGGAGGGTATTCATTTTAATGAACAGTGGATA
GTTTAAATTAACCTAGTTATAGTTTATGAATTTAAATTATAATTATATTAATGTAGGTTATGATAGA
ATTATGTATTGATCCTTAAGAGTTAAGAGAGATACGCCCACGTATAATACATATTGGTTTAGGGT
TGAGTATATATACTTAAGAGTTAAGAATATATATATACAATATATAATAAATATAGTAATATATTA
ATGGTATGGACTTAACCAGGTTTATATATTATCATTTGATAAACATTAATTATAATTTTTCTATTTA
TTATTATTATTATACTGTGAGATTAATAATTATTAAAATATAATATTACATAGTACGTGATATTTGG
GGATTTTATCTTATTAATTGGCAATTAATGATTCTAATCAAATTTTATTCTCTTTAGTTTAATGGTA
GAACAATGATCTTCTAATTCATTGGTTTTAGTTCGATTCTAAAAAGAGATGAGTAAATAATTTTCT
AGATCAGAAATACTACTTTTAACTACAAAAAGCTTACGCTTTTTAACATTTTTTTGATAAATAACA
ACTATTGTTAATATTTGGCTGTCTATTGGTTTAACTAACAATTACAAAATTTTTTCAATTTTTATAAT
ATAAATTTAAATGAGAATATTAAAAAGTCATTCATTATTAAAATTAGTGAATTCTTACCTTATCGA
TGCGTCACAACCAAGTAACATTAGTTACTTGTGAAATTTTGGTTCATTATTAGCTGTTTGTTTAAT
AGTACAAATTATTACCGGTATTACATTAGCTATGCATTATAGTCCTAGTGTAATGGAAGCTTTTAA
CTCAATAGAGCATATAATGAGAGATGTTAATAACGGGTGATTAGTTCGTTATCTACATAGTAATA
CAGCTTCTGCTTTCTTTTTCTTAGTGTATTTACACATAGGAAGAGGTATATATTACGGATCATATA
GAGCTCCTCGTACTTTAGTTTGAGCTATTGGTACTGTTATATTAATATTAATGATGGCTATCGGTT
TCCTAGGTTATGTTTTACCTTATGGACAGATGTCATTATGAGGTGCTACAGTTATTACTAATCTTA
TTAGTGCTATACCTTGAATAGGGCAAGATATTGTTGAATTCATTTGAGGTGGTTTTTCTGTTAATA
ATGCCACTTTAAACAGATTTTTTGCATTACATTTTGTATTGCCTTTTGTATTAGCTGCTTTAGTTTTA
ATGCACTTAATTGCACTTCATGATACTGCTGGTTCAAGCAATCCTCTTGGTGTTTCAGGTAATTAC
GATAGAATTACATTTGCTCCATATTTTTTATTTAAAGATTTAATTACTATTTTTATATTTATTTTTGTA
TTAAGTGCTTTTGTATTCTTTATGCCTAATGTTTTAGGGGATAGTGATAATTATATTATGGCTAATC
CTATGCAAACTCCTGCTGCTATTGTACCTGAATGATACTTATTACCTTTCTATGCTATTTTAAGATC
TATACCTAATAAATTATTAGGTGTTATAGCGATGTTTAGTGCTATTTTAGCTATTATGTTATTACCT
GTTACAGATTTAGGTAGATCTAGAGGTTTACAATTTAGACCATTTAGTAAAATAGCTTTCTGAGTT
TTTGTTGCTAATTTCTTAGTTTTAATGCAATTAGGTGCTAAACACGTTGAAGATCCATTTATATTAT
TAGGTCAATTAAGTACTGTATTATACTTTAGTTATTTTGTTGCTATATTACCTTTAGCTAGTTACTT
AGATAATAGTTTAACTGATTTATCTAATAAATCTGAATTATTTTTAAATAAAACTAACTAAATATAT
TAAGATTATTATTTAATATATTTTCTATTTAAGATACTATTAATTTAGTATTTTGGGTTTTCAGTTTA
TAATTTATATTATATTATGCATTACCCTCCACCTTGCTTTGTAGTAAGCTAATCTGTTATTTCCTTTA
GTTTAATGGTAGAACAATGATCTTCTAATTCATTGGTTTTAGTTCGAATCTAAAAAGGAAATAAG

AAATATATTCTTATTATTACTTATATAATAATTATTTCTTAAAAATATACATTTTGCATTATAGCCGT
TTAGCTGTATTAAAATGTAAAATGATATAAAATAGAATAAATATTTAAATTATTCCTATGTTATATT
ATCCTATATTGCAACCATTATCAGAAGTTGTATTAATACTTGTACCTGCCTTATTAGCTGTAGCTT
ATGTTACAGTTGCTGAAAGAAAAACTATGGCTAGTATGCAAAGAAGATTAGGTCCTAATGCTGT
AGGTTACTATGGACTATTGCAAGCATTTGCTGATGCCTTAAAACTTTTATTAAAAGAATATGTAG
CTCCTACACAATCTAATATTGTTCTTTTCTTTTTAGGTCCTGTAATAACTTTAATTTTTGCATTATTA
GGTTACGCTGTTATACCCTATGGTCCTGGTTCAGGGATAAGCGACATGAATTTAGGTATATTTTA
CATGTTAGCTGTGTCATCTTTAGCTACATACGGTATTCTATTAGCTGGTTGAAGTGCGAATAGTA
AATACGCTTTTCTAGGTTCTCTTAGAAGTACAGCTCAATTAATTAGTTATGAATTAATATTAAGTT
CAGCTATATTAATAGTAATTATGATAACAGGAAATTTAAATTTAACTGTTTGTACTGAATCTCAAA
GAGCTATTTGATTTATACTACCTTTATTTCCTGTGTTTATAATATTTTTCATAGGATCTATAGCTGA
GACAAATAGAGCTCCTTTTGATTTAGCCGAGGCTAACCTGCTAATCTGGTTTGGTCTGGTTATAT
GTCACAAATTGCTAGGAAACCTTTTTATTTTAAAAACAAAAGACAATTAGCAGGAAACTTAATTT
AACCTAATTAAAAATATTAGATAATTAAACTCTTCATAGACTAAACGTGACAATTTAATATATATA
TATATTTATTTATATATATGATTAAATAAGATATAGTCAATCATCGGTGTGAATCGACTTAAAAAA
AAAAGCACATGGGTTAAACCCATCTCCCCTTATTAGGGGAATCAGAACTTGTTAGTGGGTTCAT
GACAGAGCATGCTGCCGTAGTTTTCGTATTCTTCTTTTTAGCTGAGTACGGTAGTATTGTACTAAT
GTGTATTTTAACTAGTATATTATTTATTGGTGGTTACTTATTATTTGAAATATCCTATGTTTTTACTG
TGGTAAATTATATTTTCTTTGAATTATTCTTTATAGACTGAGTAACATTTGTAGAGGTACAATCTTT
ATACACTGATTTTTTAAATAATTCTATCATTGAAGGATTATTATATGGGTTTAATCTAGGATTAAA
AAGTTCTTTAATGATATTCACATTTATTTGAGCTAGAGCATCCTTCCCTAGAATACGATTTGATCA
ACTAATGGGCTTCTGTTGAACAGTTTTATTACCTATTAATTTTGCAATTATTATATTAGTACCTTGT
GTTTTATATAGTTTTAACTTATTACCTGTAAATATACCATTGTTCTAGCTCACACACCCGCCCGCC
CTACTGCCACAAGGCTACAGTACATATGAGGAGGGGAACTAAAGATCTAGAACTATCCTAGTTA
ATAATTACACTTAATAGTATACTAAATAGACCATCTATCATACTCGAGAATAGTGATAGTGTAAT
TATACACTATTTTAACTGTATTCTACACTATTAGCATATTATTATCTTTATATGATAATAATTTTATA
ACTTAATTTATTAGGTTACATTAACAAAAAATTCGTTAACTTTTTTCACCACTTTTTAATACAAAAT
ACGAATTTTA
>YN137
ATAACAATTCTAAAGAACATAAAGAGTTATTAGATAAAAATAATTCACCTATACAGTTAATAAAT
CAACTTAAAGGGTATTTTTTCATAAATCCTTTATTAGCTTTAAGTTTAGCTATTACTATTTTCTCTTT
TGCAGGTATTCCTCCTCTTGTAGGGTTCTTTGCTAAACAGATGGTATTAAGCGCGGCTATTGATC
AAGGTTATATCTTTTTATCTTTAGTTGCAATATTAACTAGTGTTATAGGAGGGGTTTATTATTTAAA
TATAATTAAAGAAATGTTCTTTTATTCACCTGACTATAAATTAAACGAAGAAATTAAAAATAATAC
TATTAATGGTCAAATTATTAATAGAAACAATAAAATATTAAATGTTGAATTTAATTATACAAATGT
AGTTATGTCTAGTTCTGTGGCAATAACTATTTCTACTATTACATTAGTAGTTTTATTATTCATGTTT
ATGAATAAAGAATGATTAAGTCTGGGTACTATATTGGTACAATCTTTATTTAGCTATTAATGAGTA
GTATGACATTATTTATAGGGTTTGTATCTGTTATAGCTATTTTATTTTTAGCCATTAATTTTATATTT
GCTCCTCATAATCCTTATCAAGAAAAATATAGTATTTTCGAGTGTGGTTTCCATAGTTTTTTAGGG
CAAAATAGAACACAATTCGGTATAAAATTCTTTATTTTTGCTTTAGTTTATTTACTTTTAGATTTAG
AAATATTATTAACTTTCCCTTTCGCTCTTAGTGAGTATGTTAATGGTATTTATGGTCTTTTAGTTAC
TTTAATTTTTATAGCTATAATAACTATAGGATTTATATTTGAATTAGGTAAAAGCGCTCTTAAAATA
GACAGCAGACAAAAATTATATATACCTAAATTGAACGTTAATTACCATACAGAGTATGTTGGAAT
AGGTAAGGTTTCTAAGTAAAGTTATAGAGGCAGAAAACCAAAAAACCTACCAAAGGGTAGCTA

ATGGGAAGCTATTAAAAATAAAAGATGATAACCTATATATAGTATAGTTACTATATATTTACTATA
ACTAGGATTATTATATATATATATATTATCTTATTGTATATTAAGATTATTATTATTATAAGGTATA
ATTAATATAGTATCTTATTGTATAAGAATATAATATATTAACCTATAATTAATTTTATTTTTTAATTT
TTAATTATAATTTTTTTTTATATCTAGATGCTTACACATCTACAGATGTAGAAGAGAACAAAATAT
TGTTGTTATAGTAGTGGATGATATAGAAAAATATTTTTATATTATTTATTTTTGTTAGGTAGCTTTT
GAAGTGTTTGATAGAGAGGATATATGGACGGTAGGAGGGTATTCATTTTAATGAACAGTGGATA
GTTTAAATTAACCTAGTTATAGTTTATGAATTTAAATTATAATTATATTAATGTAGGTTATGATAGA
ATTATGTATTGATCCTTAAGAGTTAAGAGAGATACGCCCACGTATAATACATATTGGTTTAGGGT
TGAGTATATATACTTAAGAGTTAAGAATATATATATACAATATATAATAAATATAGTAATATATTA
ATGGTATGGACTTAACCAGGTTTATATATTATCATTTGATAAACATTAATTATAATTTTTCTATTTA
TTATTATTATTATACTGTGAGATTAATAATTATTAAAATATAATATTACATAGTACGTGATATTTGG
GGATTTTATCTTATTAATTGGCAATTAATGATTCTAATCAAATTTTATTCTCTTTAGTTTAATGGTA
GAACAATGATCTTCTAATTCATTGGTTTTAGTTCGATTCTAAAAAGAGATGAGTAAATAATTTTCT
AGATCAGAAATACTACTTTTAACTACAAAAAGCTTACGCTTTTTAACATTTTTTTGATAAATAACA
ACTATTGTTAATATTTGGCTGTCTATTGGTTTAACTAACAATTACAAAATTTTTTCAATTTTTATAAT
ATAAATTTAAATGAGAATATTAAAAAGTCATTCATTATTAAAATTAGTGAATTCTTACCTTATCGA
TGCGTCACAACCAAGTAACATTAGTTACTTGTGAAATTTTGGTTCATTATTAGCTGTTTGTTTAAT
AGTACAAATTATTACCGGTATTACATTAGCTATGCATTATAGTCCTAGTGTAATGGAAGCTTTTAA
CTCAATAGAGCATATAATGAGAGATGTTAATAACGGGTGATTAGTTCGTTATCTACATAGTAATA
CAGCTTCTGCTTTCTTTTTCTTAGTGTATTTACACATAGGAAGAGGTATATATTACGGATCATATA
GAGCTCCTCGTACTTTAGTTTGAGCTATTGGTACTGTTATATTAATATTAATGATGGCTATCGGTT
TCCTAGGTTATGTTTTACCTTATGGACAGATGTCATTATGAGGTGCTACAGTTATTACTAATCTTA
TTAGTGCTATACCTTGAATAGGGCAAGATATTGTTGAATTCATTTGAGGTGGTTTTTCTGTTAATA
ATGCCACTTTAAACAGATTTTTTGCATTACATTTTGTATTGCCTTTTGTATTAGCTGCTTTAGTTTTA
ATGCACTTAATTGCACTTCATGATACTGCTGGTTCAAGCAATCCTCTTGGTGTTTCAGGTAATTAC
GATAGAATTACATTTGCTCCATATTTTTTATTTAAAGATTTAATTACTATTTTTATATTTATTTTTGTA
TTAAGTGCTTTTGTATTCTTTATGCCTAATGTTTTAGGGGATAGTGATAATTATATTATGGCTAATC
CTATGCAAACTCCTGCTGCTATTGTACCTGAATGATACTTATTACCTTTCTATGCTATTTTAAGATC
TATACCTAATAAATTATTAGGTGTTATAGCGATGTTTAGTGCTATTTTAGCTATTATGTTATTACCT
GTTACAGATTTAGGTAGATCTAGAGGTTTACAATTTAGACCATTTAGTAAAATAGCTTTCTGAGTT
TTTGTTGCTAATTTCTTAGTTTTAATGCAATTAGGTGCTAAACACGTTGAAGATCCATTTATATTAT
TAGGTCAATTAAGTACTGTATTATACTTTAGTTATTTTGTTGCTATATTACCTTTAGCTAGTTACTT
AGATAATAGTTTAACTGATTTATCTAATAAATCTGAATTATTTTTAAATAAAACTAACTAAATATAT
TAAGATTATTATTTAATATATTTTCTATTTAAGATACTATTAATTTAGTATTTTGGGTTTTCAGTTTA
TAATTTATATTATATTATGCATTACCCTCCACCTTGCTTTGTAGTAAGCTAATCTGTTATTTCCTTTA
GTTTAATGGTAGAACAATGATCTTCTAATTCATTGGTTTTAGTTCGAATCTAAAAAGGAAATAAG
AAATATATTCTTATTATTACTTATATAATAATTATTTCTTAAAAATATACATTTTGCATTATAGCCGT
TTAGCTGTATTAAAATGTAAAATGATATAAAATAGAATAAATATTTAAATTATTCCTATGTTATATT
ATCCTATATTGCAACCATTATCAGAAGTTGTATTAATACTTGTACCTGCCTTATTAGCTGTAGCTT
ATGTTACAGTTGCTGAAAGAAAAACTATGGCTAGTATGCAAAGAAGATTAGGTCCTAATGCTGT
AGGTTACTATGGACTATTGCAAGCATTTGCTGATGCCTTAAAACTTTTATTAAAAGAATATGTAG
CTCCTACACAATCTAATATTGTTCTTTTCTTTTTAGGTCCTGTAATAACTTTAATTTTTGCATTATTA
GGTTACGCTGTTATACCCTATGGTCCTGGTTCAGGGATAAGCGACATGAATTTAGGTATATTTTA
CATGTTAGCTGTGTCATCTTTAGCTACATACGGTATTCTATTAGCTGGTTGAAGTGCGAATAGTA

AATACGCTTTTCTAGGTTCTCTTAGAAGTACAGCTCAATTAATTAGTTATGAATTAATATTAAGTT
CAGCTATATTAATAGTAATTATGATAACAGGAAATTTAAATTTAACTGTTTGTACTGAATCTCAAA
GAGCTATTTGATTTATACTACCTTTATTTCCTGTGTTTATAATATTTTTCATAGGATCTATAGCTGA
GACAAATAGAGCTCCTTTTGATTTAGCCGAGGCTAACCTGCTAATCTGGTTTGGTCTGGTTATAT
GTCACAAATTGCTAGGAAACCTTTTTATTTTAAAAACAAAAGACAATTAGCAGGAAACTTAATTT
AACCTAATTAAAAATATTAGATAATTAAACTCTTCATAGACTAAACGTGACAATTTAATATATATA
TATATTTATTTATATATATGATTAAATAAGATATAGTCAATCATCGGTGTGAATCGACTTAAAAAA
AAAAGCACATGGGTTAAACCCATCTCCCCTTATTAGGGGAATCAGAACTTGTTAGTGGGTTCAT
GACAGAGCATGCTGCCGTAGTTTTCGTATTCTTCTTTTTAGCTGAGTACGGTAGTATTGTACTAAT
GTGTATTTTAACTAGTATATTATTTATTGGTGGTTACTTATTATTTGAAATATCCTATGTTTTTACTG
TGGTAAATTATATTTTCTTTGAATTATTCTTTATAGACTGAGTAACATTTGTAGAGGTACAATCTTT
ATACACTGATTTTTTAAATAATTCTATCATTGAAGGATTATTATATGGGTTTAATCTAGGATTAAA
AAGTTCTTTAATGATATTCACATTTATTTGAGCTAGAGCATCCTTCCCTAGAATACGATTTGATCA
ACTAATGGGCTTCTGTTGAACAGTTTTATTACCTATTAATTTTGCAATTATTATATTAGTACCTTGT
GTTTTATATAGTTTTAACTTATTACCTGTAAATATACCATTGTTCTAGCTCACACACCCGCCCGCC
CTACTGCCACAAGGCTACAGTACATATGAGGAGGGGAACTAAAGATCTAGAACTATCCTAGTTA
ATAATTACACTTAATAGTATACTAAATAGACCATCTATCATACTCGAGAATAGTGATAGTGTAAT
TATACACTATTTTAACTGTATTCTACACTATTAGCATATTATTATCTTTATATGATAATAATTTTATA
ACTTAATTTATTAGGTTACATTAACAAAAAAATTCGTTAACTTTTTTCACCACTTTTTAATACAAAAT
ACGAATTATA
>YN153
ATAACAATTCTAAAGAACATAAAGAGTTATTAGATAAAAATAATTCACCTATACAGTTAATAAAT
CAACTTAAAGGGTATTTTTTCATAAATCCTTTATTAGCTTTAAGTTTAGCTATTACTATTTTCTCTTT
TGCAGGTATTCCTCCTCTTGTAGGGTTCTTTGCTAAACAGATGGTATTAAGCGCGGCTATTGATC
AAGGTTATATCTTTTTATCTTTAGTTGCAATATTAACTAGTGTTATAGGAGGGGTTTATTATTTAAA
TATAATTAAAGAAATGTTCTTTTATTCACCTGACTATAAATTAAACGAAGAAATTAAAAATAATAC
TATTAATGGTCAAATTATTAATAGAAACAATAAAATATTAAATGTTGAATTTAATTATACAAATGT
AGTTATGTCTAGTTCTGTGGCAATAACTATTTCTACTATTACATTAGTAGTTTTATTATTCATGTTT
ATGAATAAAGAATGATTAAGTCTGGGTACTATATTGGTACAATCTTTATTTAGCTATTAATGAGTA
GTATGACATTATTTATAGGGTTTGTATCTGTTATAGCTATTTTATTTTTAGCCATTAATTTTATATTT
GCTCCTCATAATCCTTATCAAGAAAAATATAGTATTTTCGAGTGTGGTTTCCATAGTTTTTTAGGG
CAAAATAGAACACAATTCGGTATAAAATTCTTTATTTTTGCTTTAGTTTATTTACTTTTAGATTTAG
AAATATTATTAACTTTCCCTTTCGCTCTTAGTGAGTATGTTAATGGTATTTATGGTCTTTTAGTTAC
TTTAATTTTTATAGCTATAATAACTATAGGATTTATATTTGAATTAGGTAAAAGCGCTCTTAAAATA
GACAGCAGACAAAAATTATATATACCTAAATTGAACGTTAATTACCATACAGAGTATGTTGGAAT
AGGTAAGGTTTCTAAGTAAAGTTATAGAGGCAGAAAACCAAAAAACCTACCAAAGGGTAGCTA
ATGGGAAGCTATTAAAAATAAAAGATGATAACCTATATATAGTATAGTTACTATATATTTACTATA
ACTAGGATTATTATATATATATATATTATCTTATTGTATATTAAGATTATTATTATTATAAGGTATA
ATTAATATAGTATCTTATTGTATAAGAATATAATATATTAACCTATAATTAATTTTATTTTTTAATTT
TTAATTATAATTTTTTTTTATATCTAGATGCTTACACATCTACAGATGTAGAAGAGAACAAAATAT
TGTTGTTATAGTAGTGGATGATATAGAAAAATATTTTTATATTATTTATTTTTGTTAGGTAGCTTTT
GAAGTGTTTGATAGAGAGGATATATGGACGGTAGGAGGGTATTCATTTTAATGAACAGTGGATA
GTTTAAATTAACCTAGTTATAGTTTATGAATTTAAATTATAATTATATTAATGTAGGTTATGATAGA
ATTATGTATTGATCCTTAAGAGTTAAGAGAGATACGCCCACGTATAATACATATTGGTTTAGGGT

TGAGTATATATACTTAAGAGTTAAGAATATATATATACAATATATAATAAATATAGTAATATATTA
ATGGTATGGACTTAACCAGGTTTATATATTATCATTTGATAAACATTAATTATAATTTTTCTATTTA
TTATTATTATTATACTGTGAGATTAATAATTATTAAAATATAATATTACATAGTACGTGATATTTGG
GGATTTTATCTTATTAATTGGCAATTAATGATTCTAATCAAATTTTATTCTCTTTAGTTTAATGGTA
GAACAATGATCTTCTAATTCATTGGTTTTAGTTCGATTCTAAAAAGAGATGAGTAAATAATTTTCT
AGATCAGAAATACTACTTTTAACTACAAAAAGCTTACGCTTTTTAACATTTTTTTGATAAATAACA
ACTATTGTTAATATTTGGCTGTCTATTGGTTTAACTAACAATTACAAAATTTTTTCAATTTTTATAAT
ATAAATTTAAATGAGAATATTAAAAAGTCATTCATTATTAAAATTAGTGAATTCTTACCTTATCGA
TGCGTCACAACCAAGTAACATTAGTTACTTGTGAAATTTTGGTTCATTATTAGCTGTTTGTTTAAT
AGTACAAATTATTACCGGTATTACATTAGCTATGCATTATAGTCCTAGTGTAATGGAAGCTTTTAA
CTCAATAGAGCATATAATGAGAGATGTTAATAACGGGTGATTAGTTCGTTATCTACATAGTAATA
CAGCTTCTGCTTTCTTTTTCTTAGTGTATTTACACATAGGAAGAGGTATATATTACGGATCATATA
GAGCTCCTCGTACTTTAGTTTGAGCTATTGGTACTGTTATATTAATATTAATGATGGCTATCGGTT
TCCTAGGTTATGTTTTACCTTATGGACAGATGTCATTATGAGGTGCTACAGTTATTACTAATCTTA
TTAGTGCTATACCTTGAATAGGGCAAGATATTGTTGAATTCATTTGAGGTGGTTTTTCTGTTAATA
ATGCCACTTTAAACAGATTTTTTGCATTACATTTTGTATTGCCTTTTGTATTAGCTGCTTTAGTTTTA
ATGCACTTAATTGCACTTCATGATACTGCTGGTTCAAGCAATCCTCTTGGTGTTTCAGGTAATTAC
GATAGAATTACATTTGCTCCATATTTTTTATTTAAAGATTTAATTACTATTTTTATATTTATTTTTGTA
TTAAGTGCTTTTGTATTCTTTATGCCTAATGTTTTAGGGGATAGTGATAATTATATTATGGCTAATC
CTATGCAAACTCCTGCTGCTATTGTACCTGAATGATACTTATTACCTTTCTATGCTATTTTAAGATC
TATACCTAATAAATTATTAGGTGTTATAGCGATGTTTAGTGCTATTTTAGCTATTATGTTATTACCT
GTTACAGATTTAGGTAGATCTAGAGGTTTACAATTTAGACCATTTAGTAAAATAGCTTTCTGAGTT
TTTGTTGCTAATTTCTTAGTTTTAATGCAATTAGGTGCTAAACACGTTGAAGATCCATTTATATTAT
TAGGTCAATTAAGTACTGTATTATACTTTAGTTATTTTGTTGCTATATTACCTTTAGCTAGTTACTT
AGATAATAGTTTAACTGATTTATCTAATAAATCTGAATTATTTTTAAATAAAACTAACTAAATATAT
TAAGATTATTATTTAATATATTTTCTATTTAAGATACTATTAATTTAGTATTTTGGGTTTTCAGTTTA
TAATTTATATTATATTATGCATTACCCTCCACCTTGCTTTGTAGTAAGCTAATCTGTTATTTCCTTTA
GTTTAATGGTAGAACAATGATCTTCTAATTCATTGGTTTTAGTTCGAATCTAAAAAGGAAATAAG
AAATATATTCTTATTATTACTTATATAATAATTATTTCTTAAAAATATACATTTTGCATTATAGCCGT
TTAGCTGTATTAAAATGTAAAATGATATAAAATAGAATAAATATTTAAATTATTCCTATGTTATATT
ATCCTATATTGCAACCATTATCAGAAGTTGTATTAATACTTGTACCTGCCTTATTAGCTGTAGCTT
ATGTTACAGTTGCTGAAAGAAAAACTATGGCTAGTATGCAAAGAAGATTAGGTCCTAATGCTGT
AGGTTACTATGGACTATTGCAAGCATTTGCTGATGCCTTAAAACTTTTATTAAAAGAATATGTAG
CTCCTACACAATCTAATATTGTTCTTTTCTTTTTAGGTCCTGTAATAACTTTAATTTTTGCATTATTA
GGTTACGCTGTTATACCCTATGGTCCTGGTTCAGGGATAAGCGACATGAATTTAGGTATATTTTA
CATGTTAGCTGTGTCATCTTTAGCTACATACGGTATTCTATTAGCTGGTTGAAGTGCGAATAGTA
AATACGCTTTTCTAGGTTCTCTTAGAAGTACAGCTCAATTAATTAGTTATGAATTAATATTAAGTT
CAGCTATATTAATAGTAATTATGATAACAGGAAATTTAAATTTAACTGTTTGTACTGAATCTCAAA
GAGCTATTTGATTTATACTACCTTTATTTCCTGTGTTTATAATATTTTTCATAGGATCTATAGCTGA
GACAAATAGAGCTCCTTTTGATTTAGCCGAGGCTAACCTGCTAATCTGGTTTGGTCTGGTTATAT
GTCACAAATTGCTAGGAAACCTTTTTATTTTAAAAACAAAAGACAATTAGCAGGAAACTTAATTT
AACCTAATTAAAAATATTAGATAATTAAACTCTTCATAGACTAAACGTGACAATTTAATATATATA
TATATTTATTTATATATATGATTAAATAAGATATAGTCAATCATCGGTGTGAATCGACTTAAAAAA
AAAAGCACATGGGTTAAACCCATCTCCCCTTATTAGGGGAATCAGAACTTGTTAGTGGGTTCAT

GACAGAGCATGCTGCCGTAGTTTTCGTATTCTTCTTTTTAGCTGAGTACGGTAGTATTGTACTAAT
GTGTATTTTAACTAGTATATTATTTATTGGTGGTTACTTATTATTTGAAATATCCTATGTTTTTACTG
TGGTAAATTATATTTTCTTTGAATTATTCTTTATAGACTGAGTAACATTTGTAGAGGTACAATCTTT
ATACACTGATTTTTTAAATAATTCTATCATTGAAGGATTATTATATGGGTTTAATCTAGGATTAAA
AAGTTCTTTAATGATATTCACATTTATTTGAGCTAGAGCATCCTTCCCTAGAATACGATTTGATCA
ACTAATGGGCTTCTGTTGAACAGTTTTATTACCTATTAATTTTGCAATTATTATATTAGTACCTTGT
GTTTTATATAGTTTTAACTTATTACCTGTAAATATACCATTGTTCTAGCTCACACACCCGCCCGCC
CTACTGCCACAAGGCTACAGTACATATGAGGAGGGGAACTAAAGATCTAGAACTATCCTAGTTA
ATAATTACACTTAATAGTATACTAAATAGACCATCTATCATACTCGAGAATAGTGATAGTGTAAT
TATACACTATTTTAACTGTATTCTACACTATTAGCATATTATTATCTTTATATGATAATAATTTTATA
ACTTAATTTATTAGGTTACATTAACAAAAAATTCGTTAACTTTTTTCACCACTTTTTAATACAAAAT
ACGAATTATA
>YN154
ATAACAATTCTAAAGAACATAAAGAGTTATTAGATAAAAATAATTCACCTATACAGTTAATAAAT
CAACTTAAAGGGTATTTTTTCATAAATCCTTTATTAGCTTTAAGTTTAGCTATTACTATTTTCTCTTT
TGCAGGTATTCCTCCTCTTGTAGGGTTCTTTGCTAAACAGATGGTATTAAGCGCGGCTATTGATC
AAGGTTATATCTTTTTATCTTTAGTTGCAATATTAACTAGTGTTATAGGAGGGGTTTATTATTTAAA
TATAATTAAAGAAATGTTCTTTTATTCACCTGACTATAAATTAAACGAAGAAATTAAAAATAATAC
TATTAATGGTCAAATTATTAATAGAAACAATAAAATATTAAATGTTGAATTTAATTATACAAATGT
AGTTATGTCTAGTTCTGTGGCAATAACTATTTCTACTATTACATTAGTAGTTTTATTATTCATGTTT
ATGAATAAAGAATGATTAAGTCTGGGTACTATATTGGTACAATCTTTATTTAGCTATTAATGAGTA
GTATGACATTATTTATAGGGTTTGTATCTGTTATAGCTATTTTATTTTTAGCCATTAATTTTATATTT
GCTCCTCATAATCCTTATCAAGAAAAATATAGTATTTTCGAGTGTGGTTTCCATAGTTTTTTAGGG
CAAAATAGAACACAATTCGGTATAAAATTCTTTATTTTTGCTTTAGTTTATTTACTTTTAGATTTAG
AAATATTATTAACTTTCCCTTTCGCTCTTAGTGAGTATGTTAATGGTATTTATGGTCTTTTAGTTAC
TTTAATTTTTATAGCTATAATAACTATAGGATTTATATTTGAATTAGGTAAAAGCGCTCTTAAAATA
GACAGCAGACAAAAATTATATATACCTAAATTGAACGTTAATTACCATACAGAGTATGTTGGAAT
AGGTAAGGTTTCTAAGTAAAGTTATAGAGGCAGAAAACCAAAAAACCTACCAAAGGGTAGCTA
ATGGGAAGCTATTAAAAATAAAAGATGATAACCTATATATAGTATAGTTACTATATATTTACTATA
ACTAGGATTATTATATATATATATATTATCTTATTGTATATTAAGATTATTATTATTATAAGGTATA
ATTAATATAGTATCTTATTGTATAAGAATATAATATATTAACCTATAATTAATTTTATTTTTTAATTT
TTAATTATAATTTTTTTTTATATCTAGATGCTTACACATCTACAGATGTAGAAGAGAACAAAATAT
TGTTGTTATAGTAGTGGATGATATAGAAAAATATTTTTATATTATTTATTTTTGTTAGGTAGCTTTT
GAAGTGTTTGATAGAGAGGATATATGGACGGTAGGAGGGTATTCATTTTAATGAACAGTGGATA
GTTTAAATTAACCTAGTTATAGTTTATGAATTTAAATTATAATTATATTAATGTAGGTTATGATAGA
ATTATGTATTGATCCTTAAGAGTTAAGAGAGATACGCCCACGTATAATACATATTGGTTTAGGGT
TGAGTATATATACTTAAGAGTTAAGAATATATATATACAATATATAATAAATATAGTAATATATTA
ATGGTATGGACTTAACCAGGTTTATATATTATCATTTGATAAACATTAATTATAATTTTTCTATTTA
TTATTATTATTATACTGTGAGATTAATAATTATTAAAATATAATATTACATAGTACGTGATATTTGG
GGATTTTATCTTATTAATTGGCAATTAATGATTCTAATCAAATTTTATTCTCTTTAGTTTAATGGTA
GAACAATGATCTTCTAATTCATTGGTTTTAGTTCGATTCTAAAAAGAGATGAGTAAATAATTTTCT
AGATCAGAAATACTACTTTTAACTACAAAAAGCTTACGCTTTTTAACATTTTTTTGATAAATAACA
ACTATTGTTAATATTTGGCTGTCTATTGGTTTAACTAACAATTACAAAATTTTTTCAATTTTTATAAT
ATAAATTTAAATGAGAATATTAAAAAGTCATTCATTATTAAAATTAGTGAATTCTTACCTTATCGA

TGCGTCACAACCAAGTAACATTAGTTACTTGTGAAATTTTGGTTCATTATTAGCTGTTTGTTTAAT
AGTACAAATTATTACCGGTATTACATTAGCTATGCATTATAGTCCTAGTGTAATGGAAGCTTTTAA
CTCAATAGAGCATATAATGAGAGATGTTAATAACGGGTGATTAGTTCGTTATCTACATAGTAATA
CAGCTTCTGCTTTCTTTTTCTTAGTGTATTTACACATAGGAAGAGGTATATATTACGGATCATATA
GAGCTCCTCGTACTTTAGTTTGAGCTATTGGTACTGTTATATTAATATTAATGATGGCTATCGGTT
TCCTAGGTTATGTTTTACCTTATGGACAGATGTCATTATGAGGTGCTACAGTTATTACTAATCTTA
TTAGTGCTATACCTTGAATAGGGCAAGATATTGTTGAATTCATTTGAGGTGGTTTTTCTGTTAATA
ATGCCACTTTAAACAGATTTTTTGCATTACATTTTGTATTGCCTTTTGTATTAGCTGCTTTAGTTTTA
ATGCACTTAATTGCACTTCATGATACTGCTGGTTCAAGCAATCCTCTTGGTGTTTCAGGTAATTAC
GATAGAATTACATTTGCTCCATATTTTTTATTTAAAGATTTAATTACTATTTTTATATTTATTTTTGTA
TTAAGTGCTTTTGTATTCTTTATGCCTAATGTTTTAGGGGATAGTGATAATTATATTATGGCTAATC
CTATGCAAACTCCTGCTGCTATTGTACCTGAATGATACTTATTACCTTTCTATGCTATTTTAAGATC
TATACCTAATAAATTATTAGGTGTTATAGCGATGTTTAGTGCTATTTTAGCTATTATGTTATTACCT
GTTACAGATTTAGGTAGATCTAGAGGTTTACAATTTAGACCATTTAGTAAAATAGCTTTCTGAGTT
TTTGTTGCTAATTTCTTAGTTTTAATGCAATTAGGTGCTAAACACGTTGAAGATCCATTTATATTAT
TAGGTCAATTAAGTACTGTATTATACTTTAGTTATTTTGTTGCTATATTACCTTTAGCTAGTTACTT
AGATAATAGTTTAACTGATTTATCTAATAAATCTGAATTATTTTTAAATAAAACTAACTAAATATAT
TAAGATTATTATTTAATATATTTTCTATTTAAGATACTATTAATTTAGTATTTTGGGTTTTCAGTTTA
TAATTTATATTATATTATGCATTACCCTCCACCTTGCTTTGTAGTAAGCTAATCTGTTATTTCCTTTA
GTTTAATGGTAGAACAATGATCTTCTAATTCATTGGTTTTAGTTCGAATCTAAAAAGGAAATAAG
AAATATATTCTTATTATTACTTATATAATAATTATTTCTTAAAAATATACATTTTGCATTATAGCCGT
TTAGCTGTATTAAAATGTAAAATGATATAAAATAGAATAAATATTTAAATTATTCCTATGTTATATT
ATCCTATATTGCAACCATTATCAGAAGTTGTATTAATACTTGTACCTGCCTTATTAGCTGTAGCTT
ATGTTACAGTTGCTGAAAGAAAAACTATGGCTAGTATGCAAAGAAGATTAGGTCCTAATGCTGT
AGGTTACTATGGACTATTGCAAGCATTTGCTGATGCCTTAAAACTTTTATTAAAAGAATATGTAG
CTCCTACACAATCTAATATTGTTCTTTTCTTTTTAGGTCCTGTAATAACTTTAATTTTTGCATTATTA
GGTTACGCTGTTATACCCTATGGTCCTGGTTCAGGGATAAGCGACATGAATTTAGGTATATTTTA
CATGTTAGCTGTGTCATCTTTAGCTACATACGGTATTCTATTAGCTGGTTGAAGTGCGAATAGTA
AATACGCTTTTCTAGGTTCTCTTAGAAGTACAGCTCAATTAATTAGTTATGAATTAATATTAAGTT
CAGCTATATTAATAGTAATTATGATAACAGGAAATTTAAATTTAACTGTTTGTACTGAATCTCAAA
GAGCTATTTGATTTATACTACCTTTATTTCCTGTGTTTATAATATTTTTCATAGGATCTATAGCTGA
GACAAATAGAGCTCCTTTTGATTTAGCCGAGGCTAACCTGCTAATCTGGTTTGGTCTGGTTATAT
GTCACAAATTGCTAGGAAACCTTTTTATTTTAAAAACAAAAGACAATTAGCAGGAAACTTAATTT
AACCTAATTAAAAATATTAGATAATTAAACTCTTCATAGACTAAACGTGACAATTTAATATATATA
TATATTTATTTATATATATGATTAAATAAGATATAGTCAATCATCGGTGTGAATCGACTTAAAAAA
AAAAGCACATGGGTTAAACCCATCTCCCCTTATTAGGGGAATCAGAACTTGTTAGTGGGTTCAT
GACAGAGCATGCTGCCGTAGTTTTCGTATTCTTCTTTTTAGCTGAGTACGGTAGTATTGTACTAAT
GTGTATTTTAACTAGTATATTATTTATTGGTGGTTACTTATTATTTGAAATATCCTATGTTTTTACTG
TGGTAAATTATATTTTCTTTGAATTATTCTTTATAGACTGAGTAACATTTGTAGAGGTACAATCTTT
ATACACTGATTTTTTAAATAATTCTATCATTGAAGGATTATTATATGGGTTTAATCTAGGATTAAA
AAGTTCTTTAATGATATTCACATTTATTTGAGCTAGAGCATCCTTCCCTAGAATACGATTTGATCA
ACTAATGGGCTTCTGTTGAACAGTTTTATTACCTATTAATTTTGCAATTATTATATTAGTACCTTGT
GTTTTATATAGTTTTAACTTATTACCTGTAAATATACCATTGTTCTAGCTCACACACCCGCCCGCC
CTACTGCCACAAGGCTACAGTACATATGAGGAGGGGAACTAAAGATCTAGAACTATCCTAGTTA

ATAATTACACTTAATAGTATACTAAATAGACCATCTATCATACTCGAGAATAGTGATAGTGTAAT
TATACACTATTTTAACTGTATTCTACACTATTAGCATATTATTATCTTTATATGATAATAATTTTATA
ACTTAATTTATTAGGTTACATTAACAAAAAATTCGTTAACTTTTTTCACCACTTTTTAATACAAAAT
ACGAATTTTA
>YN157
ATAACAATTCTAAAGAACATAAAGAGTTATTAGATAAAAATAATTCACCTATACAGTTAATAAAT
CAACTTAAAGGGTATTTTTTCATAAATCCTTTATTAGCTTTAAGTTTAGCTATTACTATTTTCTCTTT
TGCAGGTATTCCTCCTCTTGTAGGGTTCTTTGCTAAACAGATGGTATTAAGCGCGGCTATTGATC
AAGGTTATATCTTTTTATCTTTAGTTGCAATATTAACTAGTGTTATAGGAGGGGTTTATTATTTAAA
TATAATTAAAGAAATGTTCTTTTATTCACCTGACTATAAATTAAACGAAGAAATTAAAAATAATAC
TATTAATGGTCAAATTATTAATAGAAACAATAAAATATTAAATGTTGAATTTAATTATACAAATGT
AGTTATGTCTAGTTCTGTGGCAATAACTATTTCTACTATTACATTAGTAGTTTTATTATTCATGTTT
ATGAATAAAGAATGATTAAGTCTGGGTACTATATTGGTACAATCTTTATTTAGCTATTAATGAGTA
GTATGACATTATTTATAGGGTTTGTATCTGTTATAGCTATTTTATTTTTAGCCATTAATTTTATATTT
GCTCCTCATAATCCTTATCAAGAAAAATATAGTATTTTCGAGTGTGGTTTCCATAGTTTTTTAGGG
CAAAATAGAACACAATTCGGTATAAAATTCTTTATTTTTGCTTTAGTTTATTTACTTTTAGATTTAG
AAATATTATTAACTTTCCCTTTCGCTCTTAGTGAGTATGTTAATGGTATTTATGGTCTTTTAGTTAC
TTTAATTTTTATAGCTATAATAACTATAGGATTTATATTTGAATTAGGTAAAAGCGCTCTTAAAATA
GACAGCAGACAAAAATTATATATACCTAAATTGAACGTTAATTACCATACAGAGTATGTTGGAAT
AGGTAAGGTTTCTAAGTAAAGTTATAGAGGCAGAAAACCAAAAAACCTACCAAAGGGTAGCTA
ATGGGAAGCTATTAAAAATAAAAGATGATAACCTATATATAGTATAGTTACTATATATTTACTATA
ACTAGGATTATTATATATATATATATTATCTTATTGTATATTAAGATTATTATTATTATAAGGTATA
ATTAATATAGTATCTTATTGTATAAGAATATAATATATTAACCTATAATTAATTTTATTTTTTAATTT
TTAATTATAATTTTTTTTTATATCTAGATGCTTACACATCTACAGATGTAGAAGAGAACAAAATAT
TGTTGTTATAGTAGTGGATGATATAGAAAAATATTTTTATATTATTTATTTTTGTTAGGTAGCTTTT
GAAGTGTTTGATAGAGAGGATATATGGACGGTAGGAGGGTATTCATTTTAATGAACAGTGGATA
GTTTAAATTAACCTAGTTATAGTTTATGAATTTAAATTATAATTATATTAATGTAGGTTATGATAGA
ATTATGTATTGATCCTTAAGAGTTAAGAGAGATACGCCCACGTATAATACATATTGGTTTAGGGT
TGAGTATATATACTTAAGAGTTAAGAATATATATATACAATATATAATAAATATAGTAATATATTA
ATGGTATGGACTTAACCAGGTTTATATATTATCATTTGATAAACATTAATTATAATTTTTCTATTTA
TTATTATTATTATACTGTGAGATTAATAATTATTAAAATATAATATTACATAGTACGTGATATTTGG
GGATTTTATCTTATTAATTGGCAATTAATGATTCTAATCAAATTTTATTCTCTTTAGTTTAATGGTA
GAACAATGATCTTCTAATTCATTGGTTTTAGTTCGATTCTAAAAAGAGATGAGTAAATAATTTTCT
AGATCAGAAATACTACTTTTAACTACAAAAAGCTTACGCTTTTTAACATTTTTTTGATAAATAACA
ACTATTGTTAATATTTGGCTGTCTATTGGTTTAACTAACAATTACAAAATTTTTTCAATTTTTATAAT
ATAAATTTAAATGAGAATATTAAAAAGTCATTCATTATTAAAATTAGTGAATTCTTACCTTATCGA
TGCGTCACAACCAAGTAACATTAGTTACTTGTGAAATTTTGGTTCATTATTAGCTGTTTGTTTAAT
AGTACAAATTATTACCGGTATTACATTAGCTATGCATTATAGTCCTAGTGTAATGGAAGCTTTTAA
CTCAATAGAGCATATAATGAGAGATGTTAATAACGGGTGATTAGTTCGTTATCTACATAGTAATA
CAGCTTCTGCTTTCTTTTTCTTAGTGTATTTACACATAGGAAGAGGTATATATTACGGATCATATA
GAGCTCCTCGTACTTTAGTTTGAGCTATTGGTACTGTTATATTAATATTAATGATGGCTATCGGTT
TCCTAGGTTATGTTTTACCTTATGGACAGATGTCATTATGAGGTGCTACAGTTATTACTAATCTTA
TTAGTGCTATACCTTGAATAGGGCAAGATATTGTTGAATTCATTTGAGGTGGTTTTTCTGTTAATA
ATGCCACTTTAAACAGATTTTTTGCATTACATTTTGTATTGCCTTTTGTATTAGCTGCTTTAGTTTTA

ATGCACTTAATTGCACTTCATGATACTGCTGGTTCAAGCAATCCTCTTGGTGTTTCAGGTAATTAC
GATAGAATTACATTTGCTCCATATTTTTTATTTAAAGATTTAATTACTATTTTTATATTTATTTTTGTA
TTAAGTGCTTTTGTATTCTTTATGCCTAATGTTTTAGGGGATAGTGATAATTATATTATGGCTAATC
CTATGCAAACTCCTGCTGCTATTGTACCTGAATGATACTTATTACCTTTCTATGCTATTTTAAGATC
TATACCTAATAAATTATTAGGTGTTATAGCGATGTTTAGTGCTATTTTAGCTATTATGTTATTACCT
GTTACAGATTTAGGTAGATCTAGAGGTTTACAATTTAGACCATTTAGTAAAATAGCTTTCTGAGTT
TTTGTTGCTAATTTCTTAGTTTTAATGCAATTAGGTGCTAAACACGTTGAAGATCCATTTATATTAT
TAGGTCAATTAAGTACTGTATTATACTTTAGTTATTTTGTTGCTATATTACCTTTAGCTAGTTACTT
AGATAATAGTTTAACTGATTTATCTAATAAATCTGAATTATTTTTAAATAAAACTAACTAAATATAT
TAAGATTATTATTTAATATATTTTCTATTTAAGATACTATTAATTTAGTATTTTGGGTTTTCAGTTTA
TAATTTATATTATATTATGCATTACCCTCCACCTTGCTTTGTAGTAAGCTAATCTGTTATTTCCTTTA
GTTTAATGGTAGAACAATGATCTTCTAATTCATTGGTTTTAGTTCGAATCTAAAAAGGAAATAAG
AAATATATTCTTATTATTACTTATATAATAATTATTTCTTAAAAATATACATTTTGCATTATAGCCGT
TTAGCTGTATTAAAATGTAAAATGATATAAAATAGAATAAATATTTAAATTATTCCTATGTTATATT
ATCCTATATTGCAACCATTATCAGAAGTTGTATTAATACTTGTACCTGCCTTATTAGCTGTAGCTT
ATGTTACAGTTGCTGAAAGAAAAACTATGGCTAGTATGCAAAGAAGATTAGGTCCTAATGCTGT
AGGTTACTATGGACTATTGCAAGCATTTGCTGATGCCTTAAAACTTTTATTAAAAGAATATGTAG
CTCCTACACAATCTAATATTGTTCTTTTCTTTTTAGGTCCTGTAATAACTTTAATTTTTGCATTATTA
GGTTACGCTGTTATACCCTATGGTCCTGGTTCAGGGATAAGCGACATGAATTTAGGTATATTTTA
CATGTTAGCTGTGTCATCTTTAGCTACATACGGTATTCTATTAGCTGGTTGAAGTGCGAATAGTA
AATACGCTTTTCTAGGTTCTCTTAGAAGTACAGCTCAATTAATTAGTTATGAATTAATATTAAGTT
CAGCTATATTAATAGTAATTATGATAACAGGAAATTTAAATTTAACTGTTTGTACTGAATCTCAAA
GAGCTATTTGATTTATACTACCTTTATTTCCTGTGTTTATAATATTTTTCATAGGATCTATAGCTGA
GACAAATAGAGCTCCTTTTGATTTAGCCGAGGCTAACCTGCTAATCTGGTTTGGTCTGGTTATAT
GTCACAAATTGCTAGGAAACCTTTTTATTTTAAAAACAAAAGACAATTAGCAGGAAACTTAATTT
AACCTAATTAAAAATATTAGATAATTAAACTCTTCATAGACTAAACGTGACAATTTAATATATATA
TATATTTATTTATATATATGATTAAATAAGATATAGTCAATCATCGGTGTGAATCGACTTAAAAAA
AAAAGCACATGGGTTAAACCCATCTCCCCTTATTAGGGGAATCAGAACTTGTTAGTGGGTTCAT
GACAGAGCATGCTGCCGTAGTTTTCGTATTCTTCTTTTTAGCTGAGTACGGTAGTATTGTACTAAT
GTGTATTTTAACTAGTATATTATTTATTGGTGGTTACTTATTATTTGAAATATCCTATGTTTTTACTG
TGGTAAATTATATTTTCTTTGAATTATTCTTTATAGACTGAGTAACATTTGTAGAGGTACAATCTTT
ATACACTGATTTTTTAAATAATTCTATCATTGAAGGATTATTATATGGGTTTAATCTAGGATTAAA
AAGTTCTTTAATGATATTCACATTTATTTGAGCTAGAGCATCCTTCCCTAGAATACGATTTGATCA
ACTAATGGGCTTCTGTTGAACAGTTTTATTACCTATTAATTTTGCAATTATTATATTAGTACCTTGT
GTTTTATATAGTTTTAACTTATTACCTGTAAATATACCATTGTTCTAGCTCACACACCCGCCCGCC
CTACTGCCACAAGGCTACAGTACATATGAGGAGGGGAACTAAAGATCTAGAACTATCCTAGTTA
ATAATTACACTTAATAGTATACTAAATAGACCATCTATCATACTCGAGAATAGTGATAGTGTAAT
TATACACTATTTTAACTGTATTCTACACTATTAGCATATTATTATCTTTATATGATAATAATTTTATA
ACTTAATTTATTAGGTTACATTAACAAAAAATTCGTTAACTTTTTTCACCACTTTTTAATACAAAAT
ACGAATTTTA
>YN158
ATAACAATTCTAAAGAACATAAAGAGTTATTAGATAAAAATAATTCACCTATACAGTTAATAAAT
CAACTTAAAGGGTATTTTTTCATAAATCCTTTATTAGCTTTAAGTTTAGCTATTACTATTTTCTCTTT
TGCAGGTATTCCTCCTCTTGTAGGGTTCTTTGCTAAACAGATGGTATTAAGCGCGGCTATTGATC

AAGGTTATATCTTTTTATCTTTAGTTGCAATATTAACTAGTGTTATAGGAGGGGTTTATTATTTAAA
TATAATTAAAGAAATGTTCTTTTATTCACCTGACTATAAATTAAACGAAGAAATTAAAAATAATAC
TATTAATGGTCAAATTATTAATAGAAACAATAAAATATTAAATGTTGAATTTAATTATACAAATGT
AGTTATGTCTAGTTCTGTGGCAATAACTATTTCTACTATTACATTAGTAGTTTTATTATTCATGTTT
ATGAATAAAGAATGATTAAGTCTGGGTACTATATTGGTACAATCTTTATTTAGCTATTAATGAGTA
GTATGACATTATTTATAGGGTTTGTATCTGTTATAGCTATTTTATTTTTAGCCATTAATTTTATATTT
GCTCCTCATAATCCTTATCAAGAAAAATATAGTATTTTCGAGTGTGGTTTCCATAGTTTTTTAGGG
CAAAATAGAACACAATTCGGTATAAAATTCTTTATTTTTGCTTTAGTTTATTTACTTTTAGATTTAG
AAATATTATTAACTTTCCCTTTCGCTCTTAGTGAGTATGTTAATGGTATTTATGGTCTTTTAGTTAC
TTTAATTTTTATAGCTATAATAACTATAGGATTTATATTTGAATTAGGTAAAAGCGCTCTTAAAATA
GACAGCAGACAAAAATTATATATACCTAAATTGAACGTTAATTACCATACAGAGTATGTTGGAAT
AGGTAAGGTTTCTAAGTAAAGTTATAGAGGCAGAAAACCAAAAAACCTACCAAAGGGTAGCTA
ATGGGAAGCTATTAAAAATAAAAGATGATAACCTATATATAGTATAGTTACTATATATTTACTATA
ACTAGGATTATTATATATATATATATTATCTTATTGTATATTAAGATTATTATTATTATAAGGTATA
ATTAATATAGTATCTTATTGTATAAGAATATAATATATTAACCTATAATTAATTTTATTTTTTAATTT
TTAATTATAATTTTTTTTTATATCTAGATGCTTACACATCTACAGATGTAGAAGAGAACAAAATAT
TGTTGTTATAGTAGTGGATGATATAGAAAAATATTTTTATATTATTTATTTTTGTTAGGTAGCTTTT
GAAGTGTTTGATAGAGAGGATATATGGACGGTAGGAGGGTATTCATTTTAATGAACAGTGGATA
GTTTAAATTAACCTAGTTATAGTTTATGAATTTAAATTATAATTATATTAATGTAGGTTATGATAGA
ATTATGTATTGATCCTTAAGAGTTAAGAGAGATACGCCCACGTATAATACATATTGGTTTAGGGT
TGAGTATATATACTTAAGAGTTAAGAATATATATATACAATATATAATAAATATAGTAATATATTA
ATGGTATGGACTTAACCAGGTTTATATATTATCATTTGATAAACATTAATTATAATTTTTCTATTTA
TTATTATTATTATACTGTGAGATTAATAATTATTAAAATATAATATTACATAGTACGTGATATTTGG
GGATTTTATCTTATTAATTGGCAATTAATGATTCTAATCAAATTTTATTCTCTTTAGTTTAATGGTA
GAACAATGATCTTCTAATTCATTGGTTTTAGTTCGATTCTAAAAAGAGATGAGTAAATAATTTTCT
AGATCAGAAATACTACTTTTAACTACAAAAAGCTTACGCTTTTTAACATTTTTTTGATAAATAACA
ACTATTGTTAATATTTGGCTGTCTATTGGTTTAACTAACAATTACAAAATTTTTTCAATTTTTATAAT
ATAAATTTAAATGAGAATATTAAAAAGTCATTCATTATTAAAATTAGTGAATTCTTACCTTATCGA
TGCGTCACAACCAAGTAACATTAGTTACTTGTGAAATTTTGGTTCATTATTAGCTGTTTGTTTAAT
AGTACAAATTATTACCGGTATTACATTAGCTATGCATTATAGTCCTAGTGTAATGGAAGCTTTTAA
CTCAATAGAGCATATAATGAGAGATGTTAATAACGGGTGATTAGTTCGTTATCTACATAGTAATA
CAGCTTCTGCTTTCTTTTTCTTAGTGTATTTACACATAGGAAGAGGTATATATTACGGATCATATA
GAGCTCCTCGTACTTTAGTTTGAGCTATTGGTACTGTTATATTAATATTAATGATGGCTATCGGTT
TCCTAGGTTATGTTTTACCTTATGGACAGATGTCATTATGAGGTGCTACAGTTATTACTAATCTTA
TTAGTGCTATACCTTGAATAGGGCAAGATATTGTTGAATTCATTTGAGGTGGTTTTTCTGTTAATA
ATGCCACTTTAAACAGATTTTTTGCATTACATTTTGTATTGCCTTTTGTATTAGCTGCTTTAGTTTTA
ATGCACTTAATTGCACTTCATGATACTGCTGGTTCAAGCAATCCTCTTGGTGTTTCAGGTAATTAC
GATAGAATTACATTTGCTCCATATTTTTTATTTAAAGATTTAATTACTATTTTTATATTTATTTTTGTA
TTAAGTGCTTTTGTATTCTTTATGCCTAATGTTTTAGGGGATAGTGATAATTATATTATGGCTAATC
CTATGCAAACTCCTGCTGCTATTGTACCTGAATGATACTTATTACCTTTCTATGCTATTTTAAGATC
TATACCTAATAAATTATTAGGTGTTATAGCGATGTTTAGTGCTATTTTAGCTATTATGTTATTACCT
GTTACAGATTTAGGTAGATCTAGAGGTTTACAATTTAGACCATTTAGTAAAATAGCTTTCTGAGTT
TTTGTTGCTAATTTCTTAGTTTTAATGCAATTAGGTGCTAAACACGTTGAAGATCCATTTATATTAT
TAGGTCAATTAAGTACTGTATTATACTTTAGTTATTTTGTTGCTATATTACCTTTAGCTAGTTACTT

AGATAATAGTTTAACTGATTTATCTAATAAATCTGAATTATTTTTAAATAAAACTAACTAAATATAT
TAAGATTATTATTTAATATATTTTCTATTTAAGATACTATTAATTTAGTATTTTGGGTTTTCAGTTTA
TAATTTATATTATATTATGCATTACCCTCCACCTTGCTTTGTAGTAAGCTAATCTGTTATTTCCTTTA
GTTTAATGGTAGAACAATGATCTTCTAATTCATTGGTTTTAGTTCGAATCTAAAAAGGAAATAAG
AAATATATTCTTATTATTACTTATATAATAATTATTTCTTAAAAATATACATTTTGCATTATAGCCGT
TTAGCTGTATTAAAATGTAAAATGATATAAAATAGAATAAATATTTAAATTATTCCTATGTTATATT
ATCCTATATTGCAACCATTATCAGAAGTTGTATTAATACTTGTACCTGCCTTATTAGCTGTAGCTT
ATGTTACAGTTGCTGAAAGAAAAACTATGGCTAGTATGCAAAGAAGATTAGGTCCTAATGCTGT
AGGTTACTATGGACTATTGCAAGCATTTGCTGATGCCTTAAAACTTTTATTAAAAGAATATGTAG
CTCCTACACAATCTAATATTGTTCTTTTCTTTTTAGGTCCTGTAATAACTTTAATTTTTGCATTATTA
GGTTACGCTGTTATACCCTATGGTCCTGGTTCAGGGATAAGCGACATGAATTTAGGTATATTTTA
CATGTTAGCTGTGTCATCTTTAGCTACATACGGTATTCTATTAGCTGGTTGAAGTGCGAATAGTA
AATACGCTTTTCTAGGTTCTCTTAGAAGTACAGCTCAATTAATTAGTTATGAATTAATATTAAGTT
CAGCTATATTAATAGTAATTATGATAACAGGAAATTTAAATTTAACTGTTTGTACTGAATCTCAAA
GAGCTATTTGATTTATACTACCTTTATTTCCTGTGTTTATAATATTTTTCATAGGATCTATAGCTGA
GACAAATAGAGCTCCTTTTGATTTAGCCGAGGCTAACCTGCTAATCTGGTTTGGTCTGGTTATAT
GTCACAAATTGCTAGGAAACCTTTTTATTTTAAAAACAAAAGACAATTAGCAGGAAACTTAATTT
AACCTAATTAAAAATATTAGATAATTAAACTCTTCATAGACTAAACGTGACAATTTAATATATATA
TATATTTATTTATATATATGATTAAATAAGATATAGTCAATCATCGGTGTGAATCGACTTAAAAAA
AAAAGCACATGGGTTAAACCCATCTCCCCTTATTAGGGGAATCAGAACTTGTTAGTGGGTTCAT
GACAGAGCATGCTGCCGTAGTTTTCGTATTCTTCTTTTTAGCTGAGTACGGTAGTATTGTACTAAT
GTGTATTTTAACTAGTATATTATTTATTGGTGGTTACTTATTATTTGAAATATCCTATGTTTTTACTG
TGGTAAATTATATTTTCTTTGAATTATTCTTTATAGACTGAGTAACATTTGTAGAGGTACAATCTTT
ATACACTGATTTTTTAAATAATTCTATCATTGAAGGATTATTATATGGGTTTAATCTAGGATTAAA
AAGTTCTTTAATGATATTCACATTTATTTGAGCTAGAGCATCCTTCCCTAGAATACGATTTGATCA
ACTAATGGGCTTCTGTTGAACAGTTTTATTACCTATTAATTTTGCAATTATTATATTAGTACCTTGT
GTTTTATATAGTTTTAACTTATTACCTGTAAATATACCATTGTTCTAGCTCACACACCCGCCCGCC
CTACTGCCACAAGGCTACAGTACATATGAGGAGGGGAACTAAAGATCTAGAACTATCCTAGTTA
ATAATTACACTTAATAGTATACTAAATAGACCATCTATCATACTCGAGAATAGTGATAGTGTAAT
TATACACTATTTTAACTGTATTCTACACTATTAGCATATTATTATCTTTATATGATAATAATTTTATA
ACTTAATTTATTAGGTTACATTAACAAAAAATTCGTTAACTTTTTTCACCACTTTTTAATACAAAAT
ACGAATTTTA
>YN161
ATAACAATTCTAAAGAACATAAAGAGTTATTAGATAAAAATAATTCACCTATACAGTTAATAAAT
CAACTTAAAGGGTATTTTTTCATAAATCCTTTATTAGCTTTAAGTTTAGCTATTACTATTTTCTCTTT
TGCAGGTATTCCTCCTCTTGTAGGGTTCTTTGCTAAACAGATGGTATTAAGCGCGGCTATTGATC
AAGGTTATATCTTTTTATCTTTAGTTGCAATATTAACTAGTGTTATAGGAGGGGTTTATTATTTAAA
TATAATTAAAGAAATGTTCTTTTATTCACCTGACTATAAATTAAACGAAGAAATTAAAAATAATAC
TATTAATGGTCAAATTATTAATAGAAACAATAAAATATTAAATGTTGAATTTAATTATACAAATGT
AGTTATGTCTAGTTCTGTGGCAATAACTATTTCTACTATTACATTAGTAGTTTTATTATTCATGTTT
ATGAATAAAGAATGATTAAGTCTGGGTACTATATTGGTACAATCTTTATTTAGCTATTAATGAGTA
GTATGACATTATTTATAGGGTTTGTATCTGTTATAGCTATTTTATTTTTAGCCATTAATTTTATATTT
GCTCCTCATAATCCTTATCAAGAAAAATATAGTATTTTCGAGTGTGGTTTCCATAGTTTTTTAGGG
CAAAATAGAACACAATTCGGTATAAAATTCTTTATTTTTGCTTTAGTTTATTTACTTTTAGATTTAG

AAATATTATTAACTTTCCCTTTCGCTCTTAGTGAGTATGTTAATGGTATTTATGGTCTTTTAGTTAC
TTTAATTTTTATAGCTATAATAACTATAGGATTTATATTTGAATTAGGTAAAAGCGCTCTTAAAATA
GACAGCAGACAAAAATTATATATACCTAAATTGAACGTTAATTACCATACAGAGTATGTTGGAAT
AGGTAAGGTTTCTAAGTAAAGTTATAGAGGCAGAAAACCAAAAAACCTACCAAAGGGTAGCTA
ATGGGAAGCTATTAAAAATAAAAGATGATAACCTATATATAGTATAGTTACTATATATTTACTATA
ACTAGGATTATTATATATATATATATATTATCTTATTGTATATTAAGATTATTATTATTATAAGGTATA
ATTAATATAGTATCTTATTGTATAAGAATATAATATATTAACCTATAATTAATTTTATTTTTTAATTT
TTAATTATAATTTTTTTTTATATCTAGATGCTTACACATCTACAGATGTAGAAGAGAACAAAATAT
TGTTGTTATAGTAGTGGATGATATAGAAAAATATTTTTATATTATTTATTTTTGTTAGGTAGCTTTT
GAAGTGTTTGATAGAGAGGATATATGGACGGTAGGAGGGTATTCATTTTAATGAACAGTGGATA
GTTTAAATTAACCTAGTTATAGTTTATGAATTTAAATTATAATTATATTAATGTAGGTTATGATAGA
ATTATGTATTGATCCTTAAGAGTTAAGAGAGATACGCCCACGTATAATACATATTGGTTTAGGGT
TGAGTATATATACTTAAGAGTTAAGAATATATATATACAATATATAATAAATATAGTAATATATTA
ATGGTATGGACTTAACCAGGTTTATATATTATCATTTGATAAACATTAATTATAATTTTTCTATTTA
TTATTATTATTATACTGTGAGATTAATAATTATTAAAATATAATATTACATAGTACGTGATATTTGG
GGATTTTATCTTATTAATTGGCAATTAATGATTCTAATCAAATTTTATTCTCTTTAGTTTAATGGTA
GAACAATGATCTTCTAATTCATTGGTTTTAGTTCGATTCTAAAAAGAGATGAGTAAATAATTTTCT
AGATCAGAAATACTACTTTTAACTACAAAAAGCTTACGCTTTTTAACATTTTTTTGATAAATAACA
ACTATTGTTAATATTTGGCTGTCTATTGGTTTAACTAACAATTACAAAATTTTTTCAATTTTTATAAT
ATAAATTTAAATGAGAATATTAAAAAGTCATTCATTATTAAAATTAGTGAATTCTTACCTTATCGA
TGCGTCACAACCAAGTAACATTAGTTACTTGTGAAATTTTGGTTCATTATTAGCTGTTTGTTTAAT
AGTACAAATTATTACCGGTATTACATTAGCTATGCATTATAGTCCTAGTGTAATGGAAGCTTTTAA
CTCAATAGAGCATATAATGAGAGATGTTAATAACGGGTGATTAGTTCGTTATCTACATAGTAATA
CAGCTTCTGCTTTCTTTTTCTTAGTGTATTTACACATAGGAAGAGGTATATATTACGGATCATATA
GAGCTCCTCGTACTTTAGTTTGAGCTATTGGTACTGTTATATTAATATTAATGATGGCTATCGGTT
TCCTAGGTTATGTTTTACCTTATGGACAGATGTCATTATGAGGTGCTACAGTTATTACTAATCTTA
TTAGTGCTATACCTTGAATAGGGCAAGATATTGTTGAATTCATTTGAGGTGGTTTTTCTGTTAATA
ATGCCACTTTAAACAGATTTTTTGCATTACATTTTGTATTGCCTTTTGTATTAGCTGCTTTAGTTTTA
ATGCACTTAATTGCACTTCATGATACTGCTGGTTCAAGCAATCCTCTTGGTGTTTCAGGTAATTAC
GATAGAATTACATTTGCTCCATATTTTTTATTTAAAGATTTAATTACTATTTTTATATTTATTTTTGTA
TTAAGTGCTTTTGTATTCTTTATGCCTAATGTTTTAGGGGATAGTGATAATTATATTATGGCTAATC
CTATGCAAACTCCTGCTGCTATTGTACCTGAATGATACTTATTACCTTTCTATGCTATTTTAAGATC
TATACCTAATAAATTATTAGGTGTTATAGCGATGTTTAGTGCTATTTTAGCTATTATGTTATTACCT
GTTACAGATTTAGGTAGATCTAGAGGTTTACAATTTAGACCATTTAGTAAAATAGCTTTCTGAGTT
TTTGTTGCTAATTTCTTAGTTTTAATGCAATTAGGTGCTAAACACGTTGAAGATCCATTTATATTAT
TAGGTCAATTAAGTACTGTATTATACTTTAGTTATTTTGTTGCTATATTACCTTTAGCTAGTTACTT
AGATAATAGTTTAACTGATTTATCTAATAAATCTGAATTATTTTTAAATAAAACTAACTAAATATAT
TAAGATTATTATTTAATATATTTTCTATTTAAGATACTATTAATTTAGTATTTTGGGTTTTCAGTTTA
TAATTTATATTATATTATGCATTACCCTCCACCTTGCTTTGTAGTAAGCTAATCTGTTATTTCCTTTA
GTTTAATGGTAGAACAATGATCTTCTAATTCATTGGTTTTAGTTCGAATCTAAAAAGGAAATAAG
AAATATATTCTTATTATTACTTATATAATAATTATTTCTTAAAAATATACATTTTGCATTATAGCCGT
TTAGCTGTATTAAAATGTAAAATGATATAAAATAGAATAAATATTTAAATTATTCCTATGTTATATT
ATCCTATATTGCAACCATTATCAGAAGTTGTATTAATACTTGTACCTGCCTTATTAGCTGTAGCTT
ATGTTACAGTTGCTGAAAGAAAAACTATGGCTAGTATGCAAAGAAGATTAGGTCCTAATGCTGT

AGGTTACTATGGACTATTGCAAGCATTTGCTGATGCCTTAAAACTTTTATTAAAAGAATATGTAG
CTCCTACACAATCTAATATTGTTCTTTTCTTTTTAGGTCCTGTAATAACTTTAATTTTTGCATTATTA
GGTTACGCTGTTATACCCTATGGTCCTGGTTCAGGGATAAGCGACATGAATTTAGGTATATTTTA
CATGTTAGCTGTGTCATCTTTAGCTACATACGGTATTCTATTAGCTGGTTGAAGTGCGAATAGTA
AATACGCTTTTCTAGGTTCTCTTAGAAGTACAGCTCAATTAATTAGTTATGAATTAATATTAAGTT
CAGCTATATTAATAGTAATTATGATAACAGGAAATTTAAATTTAACTGTTTGTACTGAATCTCAAA
GAGCTATTTGATTTATACTACCTTTATTTCCTGTGTTTATAATATTTTTCATAGGATCTATAGCTGA
GACAAATAGAGCTCCTTTTGATTTAGCCGAGGCTAACCTGCTAATCTGGTTTGGTCTGGTTATAT
GTCACAAATTGCTAGGAAACCTTTTTATTTTAAAAACAAAAGACAATTAGCAGGAAACTTAATTT
AACCTAATTAAAAATATTAGATAATTAAACTCTTCATAGACTAAACGTGACAATTTAATATATATA
TATATTTATTTATATATATGATTAAATAAGATATAGTCAATCATCGGTGTGAATCGACTTAAAAAA
AAAAGCACATGGGTTAAACCCATCTCCCCTTATTAGGGGAATCAGAACTTGTTAGTGGGTTCAT
GACAGAGCATGCTGCCGTAGTTTTCGTATTCTTCTTTTTAGCTGAGTACGGTAGTATTGTACTAAT
GTGTATTTTAACTAGTATATTATTTATTGGTGGTTACTTATTATTTGAAATATCCTATGTTTTTACTG
TGGTAAATTATATTTTCTTTGAATTATTCTTTATAGACTGAGTAACATTTGTAGAGGTACAATCTTT
ATACACTGATTTTTTAAATAATTCTATCATTGAAGGATTATTATATGGGTTTAATCTAGGATTAAA
AAGTTCTTTAATGATATTCACATTTATTTGAGCTAGAGCATCCTTCCCTAGAATACGATTTGATCA
ACTAATGGGCTTCTGTTGAACAGTTTTATTACCTATTAATTTTGCAATTATTATATTAGTACCTTGT
GTTTTATATAGTTTTAACTTATTACCTGTAAATATACCATTGTTCTAGCTCACACACCCGCCCGCC
CTACTGCCACAAGGCTACAGTACATATGAGGAGGGGAACTAAAGATCTAGAACTATCCTAGTTA
ATAATTACACTTAATAGTATACTAAATAGACCATCTATCATACTCGAGAATAGTGATAGTGTAAT
TATACACTATTTTAACTGTATTCTACACTATTAGGATATTATTATCTTTATATGATAATAATTTTATA
ACTTAATTTATTAGGTTACATTAACAAAAAATTCGTTAACTTTTTTCACCACTTTTTAATACAAAAT
ACGAATTTTA
>YN166
ATAACAATTCTAAAGAACATAAAGAGTTATTAGATAAAAATAATTCACCTATACAGTTAATAAAT
CAACTTAAAGGGTATTTTTTCATAAATCCTTTATTAGCTTTAAGTTTAGCTATTACTATTTTCTCTTT
TGCAGGTATTCCTCCTCTTGTAGGGTTCTTTGCTAAACAGATGGTATTAAGCGCGGCTATTGATC
AAGGTTATATCTTTTTATCTTTAGTTGCAATATTAACTAGTGTTATAGGAGGGGTTTATTATTTAAA
TATAATTAAAGAAATGTTCTTTTATTCACCTGACTATAAATTAAACGAAGAAATTAAAAATAATAC
TATTAATGGTCAAATTATTAATAGAAACAATAAAATATTAAATGTTGAATTTAATTATACAAATGT
AGTTATGTCTAGTTCTGTGGCAATAACTATTTCTACTATTACATTAGTAGTTTTATTATTCATGTTT
ATGAATAAAGAATGATTAAGTCTGGGTACTATATTGGTACAATCTTTATTTAGCTATTAATGAGTA
GTATGACATTATTTATAGGGTTTGTATCTGTTATAGCTATTTTATTTTTAGCCATTAATTTTATATTT
GCTCCTCATAATCCTTATCAAGAAAAATATAGTATTTTCGAGTGTGGTTTCCATAGTTTTTTAGGG
CAAAATAGAACACAATTCGGTATAAAATTCTTTATTTTTGCTTTAGTTTATTTACTTTTAGATTTAG
AAATATTATTAACTTTCCCTTTCGCTCTTAGTGAGTATGTTAATGGTATTTATGGTCTTTTAGTTAC
TTTAATTTTTATAGCTATAATAACTATAGGATTTATATTTGAATTAGGTAAAAGCGCTCTTAAAATA
GACAGCAGACAAAAATTATATATACCTAAATTGAACGTTAATTACCATACAGAGTATGTTGGAAT
AGGTAAGGTTTCTAAGTAAAGTTATAGAGGCAGAAAACCAAAAAACCTACCAAAGGGTAGCTA
ATGGGAAGCTATTAAAAATAAAAGATGATAACCTATATATAGTATAGTTACTATATATTTACTATA
ACTAGGATTATTATATATATATATATATTATCTTATTGTATATTAAGATTATTATTATTATAAGGTATA
ATTAATATAGTATCTTATTGTATAAGAATATAATATATTAACCTATAATTAATTTTATTTTTTAATTT
TTAATTATAATTTTTTTTTATATCTAGATGCTTACACATCTACAGATGTAGAAGAGAACAAAATAT

TGTTGTTATAGTAGTGGATGATATAGAAAAATATTTTTATATTATTTATTTTTGTTAGGTAGCTTTT
GAAGTGTTTGATAGAGAGGATATATGGACGGTAGGAGGGTATTCATTTTAATGAACAGTGGATA
GTTTAAATTAACCTAGTTATAGTTTATGAATTTAAATTATAATTATATTAATGTAGGTTATGATAGA
ATTATGTATTGATCCTTAAGAGTTAAGAGAGATACGCCCACGTATAATACATATTGGTTTAGGGT
TGAGTATATATACTTAAGAGTTAAGAATATATATATACAATATATAATAAATATAGTAATATATTA
ATGGTATGGACTTAACCAGGTTTATATATTATCATTTGATAAACATTAATTATAATTTTTCTATTTA
TTATTATTATTATACTGTGAGATTAATAATTATTAAAATATAATATTACATAGTACGTGATATTTGG
GGATTTTATCTTATTAATTGGCAATTAATGATTCTAATCAAATTTTATTCTCTTTAGTTTAATGGTA
GAACAATGATCTTCTAATTCATTGGTTTTAGTTCGATTCTAAAAAGAGATGAGTAAATAATTTTCT
AGATCAGAAATACTACTTTTAACTACAAAAAGCTTACGCTTTTTAACATTTTTTTGATAAATAACA
ACTATTGTTAATATTTGGCTGTCTATTGGTTTAACTAACAATTACAAAATTTTTTCAATTTTTATAAT
ATAAATTTAAATGAGAATATTAAAAAGTCATTCATTATTAAAATTAGTGAATTCTTACCTTATCGA
TGCGTCACAACCAAGTAACATTAGTTACTTGTGAAATTTTGGTTCATTATTAGCTGTTTGTTTAAT
AGTACAAATTATTACCGGTATTACATTAGCTATGCATTATAGTCCTAGTGTAATGGAAGCTTTTAA
CTCAATAGAGCATATAATGAGAGATGTTAATAACGGGTGATTAGTTCGTTATCTACATAGTAATA
CAGCTTCTGCTTTCTTTTTCTTAGTGTATTTACACATAGGAAGAGGTATATATTACGGATCATATA
GAGCTCCTCGTACTTTAGTTTGAGCTATTGGTACTGTTATATTAATATTAATGATGGCTATCGGTT
TCCTAGGTTATGTTTTACCTTATGGACAGATGTCATTATGAGGTGCTACAGTTATTACTAATCTTA
TTAGTGCTATACCTTGAATAGGGCAAGATATTGTTGAATTCATTTGAGGTGGTTTTTCTGTTAATA
ATGCCACTTTAAACAGATTTTTTGCATTACATTTTGTATTGCCTTTTGTATTAGCTGCTTTAGTTTTA
ATGCACTTAATTGCACTTCATGATACTGCTGGTTCAAGCAATCCTCTTGGTGTTTCAGGTAATTAC
GATAGAATTACATTTGCTCCATATTTTTTATTTAAAGATTTAATTACTATTTTTATATTTATTTTTGTA
TTAAGTGCTTTTGTATTCTTTATGCCTAATGTTTTAGGGGATAGTGATAATTATATTATGGCTAATC
CTATGCAAACTCCTGCTGCTATTGTACCTGAATGATACTTATTACCTTTCTATGCTATTTTAAGATC
TATACCTAATAAATTATTAGGTGTTATAGCGATGTTTAGTGCTATTTTAGCTATTATGTTATTACCT
GTTACAGATTTAGGTAGATCTAGAGGTTTACAATTTAGACCATTTAGTAAAATAGCTTTCTGAGTT
TTTGTTGCTAATTTCTTAGTTTTAATGCAATTAGGTGCTAAACACGTTGAAGATCCATTTATATTAT
TAGGTCAATTAAGTACTGTATTATACTTTAGTTATTTTGTTGCTATATTACCTTTAGCTAGTTACTT
AGATAATAGTTTAACTGATTTATCTAATAAATCTGAATTATTTTTAAATAAAACTAACTAAATATAT
TAAGATTATTATTTAATATATTTTCTATTTAAGATACTATTAATTTAGTATTTTGGGTTTTCAGTTTA
TAATTTATATTATATTATGCATTACCCTCCACCTTGCTTTGTAGTAAGCTAATCTGTTATTTCCTTTA
GTTTAATGGTAGAACAATGATCTTCTAATTCATTGGTTTTAGTTCGAATCTAAAAAGGAAATAAG
AAATATATTCTTATTATTACTTATATAATAATTATTTCTTAAAAATATACATTTTGCATTATAGCCGT
TTAGCTGTATTAAAATGTAAAATGATATAAAATAGAATAAATATTTAAATTATTCCTATGTTATATT
ATCCTATATTGCAACCATTATCAGAAGTTGTATTAATACTTGTACCTGCCTTATTAGCTGTAGCTT
ATGTTACAGTTGCTGAAAGAAAAACTATGGCTAGTATGCAAAGAAGATTAGGTCCTAATGCTGT
AGGTTACTATGGACTATTGCAAGCATTTGCTGATGCCTTAAAACTTTTATTAAAAGAATATGTAG
CTCCTACACAATCTAATATTGTTCTTTTCTTTTTAGGTCCTGTAATAACTTTAATTTTTGCATTATTA
GGTTACGCTGTTATACCCTATGGTCCTGGTTCAGGGATAAGCGACATGAATTTAGGTATATTTTA
CATGTTAGCTGTGTCATCTTTAGCTACATACGGTATTCTATTAGCTGGTTGAAGTGCGAATAGTA
AATACGCTTTTCTAGGTTCTCTTAGAAGTACAGCTCAATTAATTAGTTATGAATTAATATTAAGTT
CAGCTATATTAATAGTAATTATGATAACAGGAAATTTAAATTTAACTGTTTGTACTGAATCTCAAA
GAGCTATTTGATTTATACTACCTTTATTTCCTGTGTTTATAATATTTTTCATAGGATCTATAGCTGA
GACAAATAGAGCTCCTTTTGATTTAGCCGAGGCTAACCTGCTAATCTGGTTTGGTCTGGTTATAT

GTCACAAATTGCTAGGAAACCTTTTTATTTTAAAAACAAAAGACAATTAGCAGGAAACTTAATTT
AACCTAATTAAAAATATTAGATAATTAAACTCTTCATAGACTAAACGTGACAATTTAATATATATA
TATATTTATTTATATATATGATTAAATAAGATATAGTCAATCATCGGTGTGAATCGACTTAAAAAA
AAAAGCACATGGGTTAAACCCATCTCCCCTTATTAGGGGAATCAGAACTTGTTAGTGGGTTCAT
GACAGAGCATGCTGCCGTAGTTTTCGTATTCTTCTTTTTAGCTGAGTACGGTAGTATTGTACTAAT
GTGTATTTTAACTAGTATATTATTTATTGGTGGTTACTTATTATTTGAAATATCCTATGTTTTTACTG
TGGTAAATTATATTTTCTTTGAATTATTCTTTATAGACTGAGTAACATTTGTAGAGGTACAATCTTT
ATACACTGATTTTTTAAATAATTCTATCATTGAAGGATTATTATATGGGTTTAATCTAGGATTAAA
AAGTTCTTTAATGATATTCACATTTATTTGAGCTAGAGCATCCTTCCCTAGAATACGATTTGATCA
ACTAATGGGCTTCTGTTGAACAGTTTTATTACCTATTAATTTTGCAATTATTATATTAGTACCTTGT
GTTTTATATAGTTTTAACTTATTACCTGTAAATATACCATTGTTCTAGCTCACACACCCGCCCGCC
CTACTGCCACAAGGCTACAGTACATATGAGGAGGGGAACTAAAGATCTAGAACTATCCTAGTTA
ATAATTACACTTAATAGTATACTAAATAGACCATCTATCATACTCGAGAATAGTGATAGTGTAAT
TATACACTATTTTAACTGTATTCTACACTATTAGCATATTATTATCTTTATATGATAATAATTTTATA
ACTTAATTTATTAGGTTACATTAACAAAAAATTCGTTAACTTTTTTCACCACTTTTTAATACAAAAT
ACGAATTTTA
>YN179
ATAACAATTCTAAAGAACATAAAGAGTTATTAGATAAAAATAATTCACCTATACAGTTAATAAAT
CAACTTAAAGGGTATTTTTTCATAAATCCTTTATTAGCTTTAAGTTTAGCTATTACTATTTTCTCTTT
TGCAGGTATTCCTCCTCTTGTAGGGTTCTTTGCTAAACAGATGGTATTAAGCGCGGCTATTGATC
AAGGTTATATCTTTTTATCTTTAGTTGCAATATTAACTAGTGTTATAGGAGGGGTTTATTATTTAAA
TATAATTAAAGAAATGTTCTTTTATTCACCTGACTATAAATTAAACGAAGAAATTAAAAATAATAC
TATTAATGGTCAAATTATTAATAGAAACAATAAAATATTAAATGTTGAATTTAATTATACAAATGT
AGTTATGTCTAGTTCTGTGGCAATAACTATTTCTACTATTACATTAGTAGTTTTATTATTCATGTTT
ATGAATAAAGAATGATTAAGTCTGGGTACTATATTGGTACAATCTTTATTTAGCTATTAATGAGTA
GTATGACATTATTTATAGGGTTTGTATCTGTTATAGCTATTTTATTTTTAGCCATTAATTTTATATTT
GCTCCTCATAATCCTTATCAAGAAAAATATAGTATTTTCGAGTGTGGTTTCCATAGTTTTTTAGGG
CAAAATAGAACACAATTCGGTATAAAATTCTTTATTTTTGCTTTAGTTTATTTACTTTTAGATTTAG
AAATATTATTAACTTTCCCTTTCGCTCTTAGTGAGTATGTTAATGGTATTTATGGTCTTTTAGTTAC
TTTAATTTTTATAGCTATAATAACTATAGGATTTATATTTGAATTAGGTAAAAGCGCTCTTAAAATA
GACAGCAGACAAAAATTATATATACCTAAATTGAACGTTAATTACCATACAGAGTATGTTGGAAT
AGGTAAGGTTTCTAAGTAAAGTTATAGAGGCAGAAAACCAAAAAACCTACCAAAGGGTAGCTA
ATGGGAAGCTATTAAAAATAAAAGATGATAACCTATATATAGTATAGTTACTATATATTTACTATA
ACTAGGATTATTATATATATATATATTATCTTATTGTATATTAAGATTATTATTATTATAAGGTATA
ATTAATATAGTATCTTATTGTATAAGAATATAATATATTAACCTATAATTAATTTTATTTTTTAATTT
TTAATTATAATTTTTTTTTATATCTAGATGCTTACACATCTACAGATGTAGAAGAGAACAAAATAT
TGTTGTTATAGTAGTGGATGATATAGAAAAATATTTTTATATTATTTATTTTTGTTAGGTAGCTTTT
GAAGTGTTTGATAGAGAGGATATATGGACGGTAGGAGGGTATTCATTTTAATGAACAGTGGATA
GTTTAAATTAACCTAGTTATAGTTTATGAATTTAAATTATAATTATATTAATGTAGGTTATGATAGA
ATTATGTATTGATCCTTAAGAGTTAAGAGAGATACGCCCACGTATAATACATATTGGTTTAGGGT
TGAGTATATATACTTAAGAGTTAAGAATATATATATACAATATATAATAAATATAGTAATATATTA
ATGGTATGGACTTAACCAGGTTTATATATTATCATTTGATAAACATTAATTATAATTTTTCTATTTA
TTATTATTATTATACTGTGAGATTAATAATTATTAAAATATAATATTACATAGTACGTGATATTTGG
GGATTTTATCTTATTAATTGGCAATTAATGATTCTAATCAAATTTTATTCTCTTTAGTTTAATGGTA

GAACAATGATCTTCTAATTCATTGGTTTTAGTTCGATTCTAAAAAGAGATGAGTAAATAATTTTCT
AGATCAGAAATACTACTTTTAACTACAAAAAGCTTACGCTTTTTAACATTTTTTTGATAAATAACA
ACTATTGTTAATATTTGGCTGTCTATTGGTTTAACTAACAATTACAAAATTTTTTCAATTTTTATAAT
ATAAATTTAAATGAGAATATTAAAAAGTCATTCATTATTAAAATTAGTGAATTCTTACCTTATCGA
TGCGTCACAACCAAGTAACATTAGTTACTTGTGAAATTTTGGTTCATTATTAGCTGTTTGTTTAAT
AGTACAAATTATTACCGGTATTACATTAGCTATGCATTATAGTCCTAGTGTAATGGAAGCTTTTAA
CTCAATAGAGCATATAATGAGAGATGTTAATAACGGGTGATTAGTTCGTTATCTACATAGTAATA
CAGCTTCTGCTTTCTTTTTCTTAGTGTATTTACACATAGGAAGAGGTATATATTACGGATCATATA
GAGCTCCTCGTACTTTAGTTTGAGCTATTGGTACTGTTATATTAATATTAATGATGGCTATCGGTT
TCCTAGGTTATGTTTTACCTTATGGACAGATGTCATTATGAGGTGCTACAGTTATTACTAATCTTA
TTAGTGCTATACCTTGAATAGGGCAAGATATTGTTGAATTCATTTGAGGTGGTTTTTCTGTTAATA
ATGCCACTTTAAACAGATTTTTTGCATTACATTTTGTATTGCCTTTTGTATTAGCTGCTTTAGTTTTA
ATGCACTTAATTGCACTTCATGATACTGCTGGTTCAAGCAATCCTCTTGGTGTTTCAGGTAATTAC
GATAGAATTACATTTGCTCCATATTTTTTATTTAAAGATTTAATTACTATTTTTATATTTATTTTTGTA
TTAAGTGCTTTTGTATTCTTTATGCCTAATGTTTTAGGGGATAGTGATAATTATATTATGGCTAATC
CTATGCAAACTCCTGCTGCTATTGTACCTGAATGATACTTATTACCTTTCTATGCTATTTTAAGATC
TATACCTAATAAATTATTAGGTGTTATAGCGATGTTTAGTGCTATTTTAGCTATTATGTTATTACCT
GTTACAGATTTAGGTAGATCTAGAGGTTTACAATTTAGACCATTTAGTAAAATAGCTTTCTGAGTT
TTTGTTGCTAATTTCTTAGTTTTAATGCAATTAGGTGCTAAACACGTTGAAGATCCATTTATATTAT
TAGGTCAATTAAGTACTGTATTATACTTTAGTTATTTTGTTGCTATATTACCTTTAGCTAGTTACTT
AGATAATAGTTTAACTGATTTATCTAATAAATCTGAATTATTTTTAAATAAAACTAACTAAATATAT
TAAGATTATTATTTAATATATTTTCTATTTAAGATACTATTAATTTAGTATTTTGGGTTTTCAGTTTA
TAATTTATATTATATTATGCATTACCCTCCACCTTGCTTTGTAGTAAGCTAATCTGTTATTTCCTTTA
GTTTAATGGTAGAACAATGATCTTCTAATTCATTGGTTTTAGTTCGAATCTAAAAAGGAAATAAG
AAATATATTCTTATTATTACTTATATAATAATTATTTCTTAAAAATATACATTTTGCATTATAGCCGT
TTAGCTGTATTAAAATGTAAAATGATATAAAATAGAATAAATATTTAAATTATTCCTATGTTATATT
ATCCTATATTGCAACCATTATCAGAAGTTGTATTAATACTTGTACCTGCCTTATTAGCTGTAGCTT
ATGTTACAGTTGCTGAAAGAAAAACTATGGCTAGTATGCAAAGAAGATTAGGTCCTAATGCTGT
AGGTTACTATGGACTATTGCAAGCATTTGCTGATGCCTTAAAACTTTTATTAAAAGAATATGTAG
CTCCTACACAATCTAATATTGTTCTTTTCTTTTTAGGTCCTGTAATAACTTTAATTTTTGCATTATTA
GGTTACGCTGTTATACCCTATGGTCCTGGTTCAGGGATAAGCGACATGAATTTAGGTATATTTTA
CATGTTAGCTGTGTCATCTTTAGCTACATACGGTATTCTATTAGCTGGTTGAAGTGCGAATAGTA
AATACGCTTTTCTAGGTTCTCTTAGAAGTACAGCTCAATTAATTAGTTATGAATTAATATTAAGTT
CAGCTATATTAATAGTAATTATGATAACAGGAAATTTAAATTTAACTGTTTGTACTGAATCTCAAA
GAGCTATTTGATTTATACTACCTTTATTTCCTGTGTTTATAATATTTTTCATAGGATCTATAGCTGA
GACAAATAGAGCTCCTTTTGATTTAGCCGAGGCTAACCTGCTAATCTGGTTTGGTCTGGTTATAT
GTCACAAATTGCTAGGAAACCTTTTTATTTTAAAAACAAAAGACAATTAGCAGGAAACTTAATTT
AACCTAATTAAAAATATTAGATAATTAAACTCTTCATAGACTAAACGTGACAATTTAATATATATA
TATATTTATTTATATATATGATTAAATAAGATATAGTCAATCATCGGTGTGAATCGACTTAAAAAA
AAAAGCACATGGGTTAAACCCATCTCCCCTTATTAGGGGAATCAGAACTTGTTAGTGGGTTCAT
GACAGAGCATGCTGCCGTAGTTTTCGTATTCTTCTTTTTAGCTGAGTACGGTAGTATTGTACTAAT
GTGTATTTTAACTAGTATATTATTTATTGGTGGTTACTTATTATTTGAAATATCCTATGTTTTTACTG
TGGTAAATTATATTTTCTTTGAATTATTCTTTATAGACTGAGTAACATTTGTAGAGGTACAATCTTT
ATACACTGATTTTTTAAATAATTCTATCATTGAAGGATTATTATATGGGTTTAATCTAGGATTAAA

AAGTTCTTTAATGATATTCACATTTATTTGAGCTAGAGCATCCTTCCCTAGAATACGATTTGATCA
ACTAATGGGCTTCTGTTGAACAGTTTTATTACCTATTAATTTTGCAATTATTATATTAGTACCTTGT
GTTTTATATAGTTTTAACTTATTACCTGTAAATATACCATTGTTCTAGCTCACACACCCGCCCGCC
CTACTGCCACAAGGCTACAGTACATATGAGGAGGGGAACTAAAGATCTAGAACTATCCTAGTTA
ATAATTACACTTAATAGTATACTAAATAGACCATCTATCATACTCGAGAATAGTGATAGTGTAAT
TATACACTATTTTAACTGTATTCTACACTATTAGGATATTATTATCTTTATATGATAATAATTTTATA
ACTTAATTTATTAGGTTACATTAACAAAAAAATTCGTTAACTTTTTTCACCACTTTTTAATACAAAAT
ACGAATTTTA
>YN199
ATAACAATTCTAAAGAACATAAAGAGTTATTAGATAAAAATAATTCACCTATACAGTTAATAAAT
CAACTTAAAGGGTATTTTTTCATAAATCCTTTATTAGCTTTAAGTTTAGCTATTACTATTTTCTCTTT
TGCAGGTATTCCTCCTCTTGTAGGGTTCTTTGCTAAACAGATGGTATTAAGCGCGGCTATTGATC
AAGGTTATATCTTTTTATCTTTAGTTGCAATATTAACTAGTGTTATAGGAGGGGTTTATTATTTAAA
TATAATTAAAGAAATGTTCTTTTATTCACCTGACTATAAATTAAACGAAGAAATTAAAAATAATAC
TATTAATGGTCAAATTATTAATAGAAACAATAAAATATTAAATGTTGAATTTAATTATACAAATGT
AGTTATGTCTAGTTCTGTGGCAATAACTATTTCTACTATTACATTAGTAGTTTTATTATTCATGTTT
ATGAATAAAGAATGATTAAGTCTGGGTACTATATTGGTACAATCTTTATTTAGCTATTAATGAGTA
GTATGACATTATTTATAGGGTTTGTATCTGTTATAGCTATTTTATTTTTAGCCATTAATTTTATATTT
GCTCCTCATAATCCTTATCAAGAAAAATATAGTATTTTCGAGTGTGGTTTCCATAGTTTTTTAGGG
CAAAATAGAACACAATTCGGTATAAAATTCTTTATTTTTGCTTTAGTTTATTTACTTTTAGATTTAG
AAATATTATTAACTTTCCCTTTCGCTCTTAGTGAGTATGTTAATGGTATTTATGGTCTTTTAGTTAC
TTTAATTTTTATAGCTATAATAACTATAGGATTTATATTTGAATTAGGTAAAAGCGCTCTTAAAATA
GACAGCAGACAAAAATTATATATACCTAAATTGAACGTTAATTACCATACAGAGTATGTTGGAAT
AGGTAAGGTTTCTAAGTAAAGTTATAGAGGCAGAAAACCAAAAAACCTACCAAAGGGTAGCTA
ATGGGAAGCTATTAAAAATAAAAGATGATAACCTATATATAGTATAGTTACTATATATTTACTATA
ACTAGGATTATTATATATATATATATTATCTTATTGTATATTAAGATTATTATTATTATAAGGTATA
ATTAATATAGTATCTTATTGTATAAGAATATAATATATTAACCTATAATTAATTTTATTTTTTAATTT
TTAATTATAATTTTTTTTTATATCTAGATGCTTACACATCTACAGATGTAGAAGAGAACAAAATAT
TGTTGTTATAGTAGTGGATGATATAGAAAAATATTTTTATATTATTTATTTTTGTTAGGTAGCTTTT
GAAGTGTTTGATAGAGAGGATATATGGACGGTAGGAGGGTATTCATTTTAATGAACAGTGGATA
GTTTAAATTAACCTAGTTATAGTTTATGAATTTAAATTATAATTATATTAATGTAGGTTATGATAGA
ATTATGTATTGATCCTTAAGAGTTAAGAGAGATACGCCCACGTATAATACATATTGGTTTAGGGT
TGAGTATATATACTTAAGAGTTAAGAATATATATATACAATATATAATAAATATAGTAATATATTA
ATGGTATGGACTTAACCAGGTTTATATATTATCATTTGATAAACATTAATTATAATTTTTCTATTTA
TTATTATTATTATACTGTGAGATTAATAATTATTAAAATATAATATTACATAGTACGTGATATTTGG
GGATTTTATCTTATTAATTGGCAATTAATGATTCTAATCAAATTTTATTCTCTTTAGTTTAATGGTA
GAACAATGATCTTCTAATTCATTGGTTTTAGTTCGATTCTAAAAAGAGATGAGTAAATAATTTTCT
AGATCAGAAATACTACTTTTAACTACAAAAAGCTTACGCTTTTTAACATTTTTTTGATAAATAACA
ACTATTGTTAATATTTGGCTGTCTATTGGTTTAACTAACAATTACAAAATTTTTTCAATTTTTATAAT
ATAAATTTAAATGAGAATATTAAAAAGTCATTCATTATTAAAATTAGTGAATTCTTACCTTATCGA
TGCGTCACAACCAAGTAACATTAGTTACTTGTGAAATTTTGGTTCATTATTAGCTGTTTGTTTAAT
AGTACAAATTATTACCGGTATTACATTAGCTATGCATTATAGTCCTAGTGTAATGGAAGCTTTTAA
CTCAATAGAGCATATAATGAGAGATGTTAATAACGGGTGATTAGTTCGTTATCTACATAGTAATA
CAGCTTCTGCTTTCTTTTTCTTAGTGTATTTACACATAGGAAGAGGTATATATTACGGATCATATA

GAGCTCCTCGTACTTTAGTTTGAGCTATTGGTACTGTTATATTAATATTAATGATGGCTATCGGTT
TCCTAGGTTATGTTTTACCTTATGGACAGATGTCATTATGAGGTGCTACAGTTATTACTAATCTTA
TTAGTGCTATACCTTGAATAGGGCAAGATATTGTTGAATTCATTTGAGGTGGTTTTTCTGTTAATA
ATGCCACTTTAAACAGATTTTTTGCATTACATTTTGTATTGCCTTTTGTATTAGCTGCTTTAGTTTTA
ATGCACTTAATTGCACTTCATGATACTGCTGGTTCAAGCAATCCTCTTGGTGTTTCAGGTAATTAC
GATAGAATTACATTTGCTCCATATTTTTTATTTAAAGATTTAATTACTATTTTTATATTTATTTTTGTA
TTAAGTGCTTTTGTATTCTTTATGCCTAATGTTTTAGGGGATAGTGATAATTATATTATGGCTAATC
CTATGCAAACTCCTGCTGCTATTGTACCTGAATGATACTTATTACCTTTCTATGCTATTTTAAGATC
TATACCTAATAAATTATTAGGTGTTATAGCGATGTTTAGTGCTATTTTAGCTATTATGTTATTACCT
GTTACAGATTTAGGTAGATCTAGAGGTTTACAATTTAGACCATTTAGTAAAATAGCTTTCTGAGTT
TTTGTTGCTAATTTCTTAGTTTTAATGCAATTAGGTGCTAAACACGTTGAAGATCCATTTATATTAT
TAGGTCAATTAAGTACTGTATTATACTTTAGTTATTTTGTTGCTATATTACCTTTAGCTAGTTACTT
AGATAATAGTTTAACTGATTTATCTAATAAATCTGAATTATTTTTAAATAAAACTAACTAAATATAT
TAAGATTATTATTTAATATATTTTCTATTTAAGATACTATTAATTTAGTATTTTGGGTTTTCAGTTTA
TAATTTATATTATATTATGCATTACCCTCCACCTTGCTTTGTAGTAAGCTAATCTGTTATTTCCTTTA
GTTTAATGGTAGAACAATGATCTTCTAATTCATTGGTTTTAGTTCGAATCTAAAAAGGAAATAAG
AAATATATTCTTATTATTACTTATATAATAATTATTTCTTAAAAATATACATTTTGCATTATAGCCGT
TTAGCTGTATTAAAATGTAAAATGATATAAAATAGAATAAATATTTAAATTATTCCTATGTTATATT
ATCCTATATTGCAACCATTATCAGAAGTTGTATTAATACTTGTACCTGCCTTATTAGCTGTAGCTT
ATGTTACAGTTGCTGAAAGAAAAACTATGGCTAGTATGCAAAGAAGATTAGGTCCTAATGCTGT
AGGTTACTATGGACTATTGCAAGCATTTGCTGATGCCTTAAAACTTTTATTAAAAGAATATGTAG
CTCCTACACAATCTAATATTGTTCTTTTCTTTTTAGGTCCTGTAATAACTTTAATTTTTGCATTATTA
GGTTACGCTGTTATACCCTATGGTCCTGGTTCAGGGATAAGCGACATGAATTTAGGTATATTTTA
CATGTTAGCTGTGTCATCTTTAGCTACATACGGTATTCTATTAGCTGGTTGAAGTGCGAATAGTA
AATACGCTTTTCTAGGTTCTCTTAGAAGTACAGCTCAATTAATTAGTTATGAATTAATATTAAGTT
CAGCTATATTAATAGTAATTATGATAACAGGAAATTTAAATTTAACTGTTTGTACTGAATCTCAAA
GAGCTATTTGATTTATACTACCTTTATTTCCTGTGTTTATAATATTTTTCATAGGATCTATAGCTGA
GACAAATAGAGCTCCTTTTGATTTAGCCGAGGCTAACCTGCTAATCTGGTTTGGTCTGGTTATAT
GTCACAAATTGCTAGGAAACCTTTTTATTTTAAAAACAAAAGACAATTAGCAGGAAACTTAATTT
AACCTAATTAAAAATATTAGATAATTAAACTCTTCATAGACTAAACGTGACAATTTAATATATATA
TATATTTATTTATATATATGATTAAATAAGATATAGTCAATCATCGGTGTGAATCGACTTAAAAAA
AAAAGCACATGGGTTAAACCCATCTCCCCTTATTAGGGGAATCAGAACTTGTTAGTGGGTTCAT
GACAGAGCATGCTGCCGTAGTTTTCGTATTCTTCTTTTTAGCTGAGTACGGTAGTATTGTACTAAT
GTGTATTTTAACTAGTATATTATTTATTGGTGGTTACTTATTATTTGAAATATCCTATGTTTTTACTG
TGGTAAATTATATTTTCTTTGAATTATTCTTTATAGACTGAGTAACATTTGTAGAGGTACAATCTTT
ATACACTGATTTTTTAAATAATTCTATCATTGAAGGATTATTATATGGGTTTAATCTAGGATTAAA
AAGTTCTTTAATGATATTCACATTTATTTGAGCTAGAGCATCCTTCCCTAGAATACGATTTGATCA
ACTAATGGGCTTCTGTTGAACAGTTTTATTACCTATTAATTTTGCAATTATTATATTAGTACCTTGT
GTTTTATATAGTTTTAACTTATTACCTGTAAATATACCATTGTTCTAGCTCACACACCCGCCCGCC
CTACTGCCACAAGGCTACAGTACATATGAGGAGGGGAACTAAAGATCTAGAACTATCCTAGTTA
ATAATTACACTTAATAGTATACTAAATAGACCATCTATCATACTCGAGAATAGTGATAGTGTAAT
TATACACTATTTTAACTGTATTCTACACTATTAGCATATTATTATCTTTATATGATAATAATTTTATA
ACTTAATTTATTAGGTTACATTAACAAAAAATTCGTTAACTTTTTTCACCACTTTTTAATACAAAAT
ACGAATTTTA

>YN208
ATAACAATTCTAAAGAACATAAAGAGTTATTAGATAAAAATAATTCACCTATACAGTTAATAAAT
CAACTTAAAGGGTATTTTTTCATAAATCCTTTATTAGCTTTAAGTTTAGCTATTACTATTTTCTCTTT
TGCAGGTATTCCTCCTCTTGTAGGGTTCTTTGCTAAACAGATGGTATTAAGCGCGGCTATTGATC
AAGGTTATATCTTTTTATCTTTAGTTGCAATATTAACTAGTGTTATAGGAGGGGTTTATTATTTAAA
TATAATTAAAGAAATGTTCTTTTATTCACCTGACTATAAATTAAACGAAGAAATTAAAAATAATAC
TATTAATGGTCAAATTATTAATAGAAACAATAAAATATTAAATGTTGAATTTAATTATACAAATGT
AGTTATGTCTAGTTCTGTGGCAATAACTATTTCTACTATTACATTAGTAGTTTTATTATTCATGTTT
ATGAATAAAGAATGATTAAGTCTGGGTACTATATTGGTACAATCTTTATTTAGCTATTAATGAGTA
GTATGACATTATTTATAGGGTTTGTATCTGTTATAGCTATTTTATTTTTAGCCATTAATTTTATATTT
GCTCCTCATAATCCTTATCAAGAAAAATATAGTATTTTCGAGTGTGGTTTCCATAGTTTTTTAGGG
CAAAATAGAACACAATTCGGTATAAAATTCTTTATTTTTGCTTTAGTTTATTTACTTTTAGATTTAG
AAATATTATTAACTTTCCCTTTCGCTCTTAGTGAGTATGTTAATGGTATTTATGGTCTTTTAGTTAC
TTTAATTTTTATAGCTATAATAACTATAGGATTTATATTTGAATTAGGTAAAAGCGCTCTTAAAATA
GACAGCAGACAAAAATTATATATACCTAAATTGAACGTTAATTACCATACAGAGTATGTTGGAAT
AGGTAAGGTTTCTAAGTAAAGTTATAGAGGCAGAAAACCAAAAAACCTACCAAAGGGTAGCTA
ATGGGAAGCTATTAAAAATAAAAGATGATAACCTATATATAGTATAGTTACTATATATTTACTATA
ACTAGGATTATTATATATATATATATTATCTTATTGTATATTAAGATTATTATTATTATAAGGTATA
ATTAATATAGTATCTTATTGTATAAGAATATAATATATTAACCTATAATTAATTTTATTTTTTAATTT
TTAATTATAATTTTTTTTTATATCTAGATGCTTACACATCTACAGATGTAGAAGAGAACAAAATAT
TGTTGTTATAGTAGTGGATGATATAGAAAAATATTTTTATATTATTTATTTTTGTTAGGTAGCTTTT
GAAGTGTTTGATAGAGAGGATATATGGACGGTAGGAGGGTATTCATTTTAATGAACAGTGGATA
GTTTAAATTAACCTAGTTATAGTTTATGAATTTAAATTATAATTATATTAATGTAGGTTATGATAGA
ATTATGTATTGATCCTTAAGAGTTAAGAGAGATACGCCCACGTATAATACATATTGGTTTAGGGT
TGAGTATATATACTTAAGAGTTAAGAATATATATATACAATATATAATAAATATAGTAATATATTA
ATGGTATGGACTTAACCAGGTTTATATATTATCATTTGATAAACATTAATTATAATTTTTCTATTTA
TTATTATTATTATACTGTGAGATTAATAATTATTAAAATATAATATTACATAGTACGTGATATTTGG
GGATTTTATCTTATTAATTGGCAATTAATGATTCTAATCAAATTTTATTCTCTTTAGTTTAATGGTA
GAACAATGATCTTCTAATTCATTGGTTTTAGTTCGATTCTAAAAAGAGATGAGTAAATAATTTTCT
AGATCAGAAATACTACTTTTAACTACAAAAAGCTTACGCTTTTTAACATTTTTTTGATAAATAACA
ACTATTGTTAATATTTGGCTGTCTATTGGTTTAACTAACAATTACAAAATTTTTTCAATTTTTATAAT
ATAAATTTAAATGAGAATATTAAAAAGTCATTCATTATTAAAATTAGTGAATTCTTACCTTATCGA
TGCGTCACAACCAAGTAACATTAGTTACTTGTGAAATTTTGGTTCATTATTAGCTGTTTGTTTAAT
AGTACAAATTATTACCGGTATTACATTAGCTATGCATTATAGTCCTAGTGTAATGGAAGCTTTTAA
CTCAATAGAGCATATAATGAGAGATGTTAATAACGGGTGATTAGTTCGTTATCTACATAGTAATA
CAGCTTCTGCTTTCTTTTTCTTAGTGTATTTACACATAGGAAGAGGTATATATTACGGATCATATA
GAGCTCCTCGTACTTTAGTTTGAGCTATTGGTACTGTTATATTAATATTAATGATGGCTATCGGTT
TCCTAGGTTATGTTTTACCTTATGGACAGATGTCATTATGAGGTGCTACAGTTATTACTAATCTTA
TTAGTGCTATACCTTGAATAGGGCAAGATATTGTTGAATTCATTTGAGGTGGTTTTTCTGTTAATA
ATGCCACTTTAAACAGATTTTTTGCATTACATTTTGTATTGCCTTTTGTATTAGCTGCTTTAGTTTTA
ATGCACTTAATTGCACTTCATGATACTGCTGGTTCAAGCAATCCTCTTGGTGTTTCAGGTAATTAC
GATAGAATTACATTTGCTCCATATTTTTTATTTAAAGATTTAATTACTATTTTTATATTTATTTTTGTA
TTAAGTGCTTTTGTATTCTTTATGCCTAATGTTTTAGGGGATAGTGATAATTATATTATGGCTAATC
CTATGCAAACTCCTGCTGCTATTGTACCTGAATGATACTTATTACCTTTCTATGCTATTTTAAGATC

TATACCTAATAAATTATTAGGTGTTATAGCGATGTTTAGTGCTATTTTAGCTATTATGTTATTACCT
GTTACAGATTTAGGTAGATCTAGAGGTTTACAATTTAGACCATTTAGTAAAATAGCTTTCTGAGTT
TTTGTTGCTAATTTCTTAGTTTTAATGCAATTAGGTGCTAAACACGTTGAAGATCCATTTATATTAT
TAGGTCAATTAAGTACTGTATTATACTTTAGTTATTTTGTTGCTATATTACCTTTAGCTAGTTACTT
AGATAATAGTTTAACTGATTTATCTAATAAATCTGAATTATTTTTAAATAAAACTAACTAAATATAT
TAAGATTATTATTTAATATATTTTCTATTTAAGATACTATTAATTTAGTATTTTGGGTTTTCAGTTTA
TAATTTATATTATATTATGCATTACCCTCCACCTTGCTTTGTAGTAAGCTAATCTGTTATTTCCTTTA
GTTTAATGGTAGAACAATGATCTTCTAATTCATTGGTTTTAGTTCGAATCTAAAAAGGAAATAAG
AAATATATTCTTATTATTACTTATATAATAATTATTTCTTAAAAATATACATTTTGCATTATAGCCGT
TTAGCTGTATTAAAATGTAAAATGATATAAAATAGAATAAATATTTAAATTATTCCTATGTTATATT
ATCCTATATTGCAACCATTATCAGAAGTTGTATTAATACTTGTACCTGCCTTATTAGCTGTAGCTT
ATGTTACAGTTGCTGAAAGAAAAACTATGGCTAGTATGCAAAGAAGATTAGGTCCTAATGCTGT
AGGTTACTATGGACTATTGCAAGCATTTGCTGATGCCTTAAAACTTTTATTAAAAGAATATGTAG
CTCCTACACAATCTAATATTGTTCTTTTCTTTTTAGGTCCTGTAATAACTTTAATTTTTGCATTATTA
GGTTACGCTGTTATACCCTATGGTCCTGGTTCAGGGATAAGCGACATGAATTTAGGTATATTTTA
CATGTTAGCTGTGTCATCTTTAGCTACATACGGTATTCTATTAGCTGGTTGAAGTGCGAATAGTA
AATACGCTTTTCTAGGTTCTCTTAGAAGTACAGCTCAATTAATTAGTTATGAATTAATATTAAGTT
CAGCTATATTAATAGTAATTATGATAACAGGAAATTTAAATTTAACTGTTTGTACTGAATCTCAAA
GAGCTATTTGATTTATACTACCTTTATTTCCTGTGTTTATAATATTTTTCATAGGATCTATAGCTGA
GACAAATAGAGCTCCTTTTGATTTAGCCGAGGCTAACCTGCTAATCTGGTTTGGTCTGGTTATAT
GTCACAAATTGCTAGGAAACCTTTTTATTTTAAAAACAAAAGACAATTAGCAGGAAACTTAATTT
AACCTAATTAAAAATATTAGATAATTAAACTCTTCATAGACTAAACGTGACAATTTAATATATATA
TATATTTATTTATATATATGATTAAATAAGATATAGTCAATCATCGGTGTGAATCGACTTAAAAAA
AAAAGCACATGGGTTAAACCCATCTCCCCTTATTAGGGGAATCAGAACTTGTTAGTGGGTTCAT
GACAGAGCATGCTGCCGTAGTTTTCGTATTCTTCTTTTTAGCTGAGTACGGTAGTATTGTACTAAT
GTGTATTTTAACTAGTATATTATTTATTGGTGGTTACTTATTATTTGAAATATCCTATGTTTTTACTG
TGGTAAATTATATTTTCTTTGAATTATTCTTTATAGACTGAGTAACATTTGTAGAGGTACAATCTTT
ATACACTGATTTTTTAAATAATTCTATCATTGAAGGATTATTATATGGGTTTAATCTAGGATTAAA
AAGTTCTTTAATGATATTCACATTTATTTGAGCTAGAGCATCCTTCCCTAGAATACGATTTGATCA
ACTAATGGGCTTCTGTTGAACAGTTTTATTACCTATTAATTTTGCAATTATTATATTAGTACCTTGT
GTTTTATATAGTTTTAACTTATTACCTGTAAATATACCATTGTTCTAGCTCACACACCCGCCCGCC
CTACTGCCACAAGGCTACAGTACATATGAGGAGGGGAACTAAAGATCTAGAACTATCCTAGTTA
ATAATTACACTTAATAGTATACTAAATAGACCATCTATCATACTCGAGAATAGTGATAGTGTAAT
TATACACTATTTTAACTGTATTCTACACTATTAGCATATTATTATCTTTATATGATAATAATTTTATA
ACTTAATTTATTAGGTTACATTAACAAAAAATTCGTTAACTTTTTTCACCACTTTTTAATACAAAAT
ACGAATTTTA
>YN220
ATAACAATTCTAAAGAACATAAAGAGTTATTAGATAAAAATAATTCACCTATACAGTTAATAAAT
CAACTTAAAGGGTATTTTTTCATAAATCCTTTATTAGCTTTAAGTTTAGCTATTACTATTTTCTCTTT
TGCAGGTATTCCTCCTCTTGTAGGGTTCTTTGCTAAACAGATGGTATTAAGCGCGGCTATTGATC
AAGGTTATATCTTTTTATCTTTAGTTGCAATATTAACTAGTGTTATAGGAGGGGTTTATTATTTAAA
TATAATTAAAGAAATGTTCTTTTATTCACCTGACTATAAATTAAACGAAGAAATTAAAAATAATAC
TATTAATGGTCAAATTATTAATAGAAACAATAAAATATTAAATGTTGAATTTAATTATACAAATGT
AGTTATGTCTAGTTCTGTGGCAATAACTATTTCTACTATTACATTAGTAGTTTTATTATTCATGTTT

ATGAATAAAGAATGATTAAGTCTGGGTACTATATTGGTACAATCTTTATTTAGCTATTAATGAGTA
GTATGACATTATTTATAGGGTTTGTATCTGTTATAGCTATTTTATTTTTAGCCATTAATTTTATATTT
GCTCCTCATAATCCTTATCAAGAAAAATATAGTATTTTCGAGTGTGGTTTCCATAGTTTTTTAGGG
CAAAATAGAACACAATTCGGTATAAAATTCTTTATTTTTGCTTTAGTTTATTTACTTTTAGATTTAG
AAATATTATTAACTTTCCCTTTCGCTCTTAGTGAGTATGTTAATGGTATTTATGGTCTTTTAGTTAC
TTTAATTTTTATAGCTATAATAACTATAGGATTTATATTTGAATTAGGTAAAAGCGCTCTTAAAATA
GACAGCAGACAAAAATTATATATACCTAAATTGAACGTTAATTACCATACAGAGTATGTTGGAAT
AGGTAAGGTTTCTAAGTAAAGTTATAGAGGCAGAAAACCAAAAAACCTACCAAAGGGTAGCTA
ATGGGAAGCTATTAAAAATAAAAGATGATAACCTATATATAGTATAGTTACTATATATTTACTATA
ACTAGGATTATTATATATATATATATATTATCTTATTGTATATTAAGATTATTATTATTATAAGGTATA
ATTAATATAGTATCTTATTGTATAAGAATATAATATATTAACCTATAATTAATTTTATTTTTTAATTT
TTAATTATAATTTTTTTTTATATCTAGATGCTTACACATCTACAGATGTAGAAGAGAACAAAATAT
TGTTGTTATAGTAGTGGATGATATAGAAAAATATTTTTATATTATTTATTTTTGTTAGGTAGCTTTT
GAAGTGTTTGATAGAGAGGATATATGGACGGTAGGAGGGTATTCATTTTAATGAACAGTGGATA
GTTTAAATTAACCTAGTTATAGTTTATGAATTTAAATTATAATTATATTAATGTAGGTTATGATAGA
ATTATGTATTGATCCTTAAGAGTTAAGAGAGATACGCCCACGTATAATACATATTGGTTTAGGGT
TGAGTATATATACTTAAGAGTTAAGAATATATATATACAATATATAATAAATATAGTAATATATTA
ATGGTATGGACTTAACCAGGTTTATATATTATCATTTGATAAACATTAATTATAATTTTTCTATTTA
TTATTATTATTATACTGTGAGATTAATAATTATTAAAATATAATATTACATAGTACGTGATATTTGG
GGATTTTATCTTATTAATTGGCAATTAATGATTCTAATCAAATTTTATTCTCTTTAGTTTAATGGTA
GAACAATGATCTTCTAATTCATTGGTTTTAGTTCGATTCTAAAAAGAGATGAGTAAATAATTTTCT
AGATCAGAAATACTACTTTTAACTACAAAAAGCTTACGCTTTTTAACATTTTTTTGATAAATAACA
ACTATTGTTAATATTTGGCTGTCTATTGGTTTAACTAACAATTACAAAATTTTTTCAATTTTTATAAT
ATAAATTTAAATGAGAATATTAAAAAGTCATTCATTATTAAAATTAGTGAATTCTTACCTTATCGA
TGCGTCACAACCAAGTAACATTAGTTACTTGTGAAATTTTGGTTCATTATTAGCTGTTTGTTTAAT
AGTACAAATTATTACCGGTATTACATTAGCTATGCATTATAGTCCTAGTGTAATGGAAGCTTTTAA
CTCAATAGAGCATATAATGAGAGATGTTAATAACGGGTGATTAGTTCGTTATCTACATAGTAATA
CAGCTTCTGCTTTCTTTTTCTTAGTGTATTTACACATAGGAAGAGGTATATATTACGGATCATATA
GAGCTCCTCGTACTTTAGTTTGAGCTATTGGTACTGTTATATTAATATTAATGATGGCTATCGGTT
TCCTAGGTTATGTTTTACCTTATGGACAGATGTCATTATGAGGTGCTACAGTTATTACTAATCTTA
TTAGTGCTATACCTTGAATAGGGCAAGATATTGTTGAATTCATTTGAGGTGGTTTTTCTGTTAATA
ATGCCACTTTAAACAGATTTTTTGCATTACATTTTGTATTGCCTTTTGTATTAGCTGCTTTAGTTTTA
ATGCACTTAATTGCACTTCATGATACTGCTGGTTCAAGCAATCCTCTTGGTGTTTCAGGTAATTAC
GATAGAATTACATTTGCTCCATATTTTTTATTTAAAGATTTAATTACTATTTTTATATTTATTTTTGTA
TTAAGTGCTTTTGTATTCTTTATGCCTAATGTTTTAGGGGATAGTGATAATTATATTATGGCTAATC
CTATGCAAACTCCTGCTGCTATTGTACCTGAATGATACTTATTACCTTTCTATGCTATTTTAAGATC
TATACCTAATAAATTATTAGGTGTTATAGCGATGTTTAGTGCTATTTTAGCTATTATGTTATTACCT
GTTACAGATTTAGGTAGATCTAGAGGTTTACAATTTAGACCATTTAGTAAAATAGCTTTCTGAGTT
TTTGTTGCTAATTTCTTAGTTTTAATGCAATTAGGTGCTAAACACGTTGAAGATCCATTTATATTAT
TAGGTCAATTAAGTACTGTATTATACTTTAGTTATTTTGTTGCTATATTACCTTTAGCTAGTTACTT
AGATAATAGTTTAACTGATTTATCTAATAAATCTGAATTATTTTTAAATAAAACTAACTAAATATAT
TAAGATTATTATTTAATATATTTTCTATTTAAGATACTATTAATTTAGTATTTTGGGTTTTCAGTTTA
TAATTTATATTATATTATGCATTACCCTCCACCTTGCTTTGTAGTAAGCTAATCTGTTATTTCCTTTA
GTTTAATGGTAGAACAATGATCTTCTAATTCATTGGTTTTAGTTCGAATCTAAAAAGGAAATAAG

AAATATATTCTTATTATTACTTATATAATAATTATTTCTTAAAAATATACATTTTGCATTATAGCCGT
TTAGCTGTATTAAAATGTAAAATGATATAAAATAGAATAAATATTTAAATTATTCCTATGTTATATT
ATCCTATATTGCAACCATTATCAGAAGTTGTATTAATACTTGTACCTGCCTTATTAGCTGTAGCTT
ATGTTACAGTTGCTGAAAGAAAAACTATGGCTAGTATGCAAAGAAGATTAGGTCCTAATGCTGT
AGGTTACTATGGACTATTGCAAGCATTTGCTGATGCCTTAAAACTTTTATTAAAAGAATATGTAG
CTCCTACACAATCTAATATTGTTCTTTTCTTTTTAGGTCCTGTAATAACTTTAATTTTTGCATTATTA
GGTTACGCTGTTATACCCTATGGTCCTGGTTCAGGGATAAGCGACATGAATTTAGGTATATTTTA
CATGTTAGCTGTGTCATCTTTAGCTACATACGGTATTCTATTAGCTGGTTGAAGTGCGAATAGTA
AATACGCTTTTCTAGGTTCTCTTAGAAGTACAGCTCAATTAATTAGTTATGAATTAATATTAAGTT
CAGCTATATTAATAGTAATTATGATAACAGGAAATTTAAATTTAACTGTTTGTACTGAATCTCAAA
GAGCTATTTGATTTATACTACCTTTATTTCCTGTGTTTATAATATTTTTCATAGGATCTATAGCTGA
GACAAATAGAGCTCCTTTTGATTTAGCCGAGGCTAACCTGCTAATCTGGTTTGGTCTGGTTATAT
GTCACAAATTGCTAGGAAACCTTTTTATTTTAAAAACAAAAGACAATTAGCAGGAAACTTAATTT
AACCTAATTAAAAATATTAGATAATTAAACTCTTCATAGACTAAACGTGACAATTTAATATATATA
TATATTTATTTATATATATGATTAAATAAGATATAGTCAATCATCGGTGTGAATCGACTTAAAAAA
AAAAGCACATGGGTTAAACCCATCTCCCCTTATTAGGGGAATCAGAACTTGTTAGTGGGTTCAT
GACAGAGCATGCTGCCGTAGTTTTCGTATTCTTCTTTTTAGCTGAGTACGGTAGTATTGTACTAAT
GTGTATTTTAACTAGTATATTATTTATTGGTGGTTACTTATTATTTGAAATATCCTATGTTTTTACTG
TGGTAAATTATATTTTCTTTGAATTATTCTTTATAGACTGAGTAACATTTGTAGAGGTACAATCTTT
ATACACTGATTTTTTAAATAATTCTATCATTGAAGGATTATTATATGGGTTTAATCTAGGATTAAA
AAGTTCTTTAATGATATTCACATTTATTTGAGCTAGAGCATCCTTCCCTAGAATACGATTTGATCA
ACTAATGGGCTTCTGTTGAACAGTTTTATTACCTATTAATTTTGCAATTATTATATTAGTACCTTGT
GTTTTATATAGTTTTAACTTATTACCTGTAAATATACCATTGTTCTAGCTCACACACCCGCCCGCC
CTACTGCCACAAGGCTACAGTACATATGAGGAGGGGAACTAAAGATCTAGAACTATCCTAGTTA
ATAATTACACTTAATAGTATACTAAATAGACCATCTATCATACTCGAGAATAGTGATAGTGTAAT
TATACACTATTTTAACTGTATTCTACACTATTAGCATATTATTATCTTTATATGATAATAATTTTATA
ACTTAATTTATTAGGTTACATTAACAAAAAATTCGTTAACTTTTTTCACCACTTTTTAATACAAAAT
ACGAATTTTA
>YN226
ATAACAATTCTAAAGAACATAAAGAGTTATTAGATAAAAATAATTCACCTATACAGTTAATAAAT
CAACTTAAAGGGTATTTTTTCATAAATCCTTTATTAGCTTTAAGTTTAGCTATTACTATTTTCTCTTT
TGCAGGTATTCCTCCTCTTGTAGGGTTCTTTGCTAAACAGATGGTATTAAGCGCGGCTATTGATC
AAGGTTATATCTTTTTATCTTTAGTTGCAATATTAACTAGTGTTATAGGAGGGGTTTATTATTTAAA
TATAATTAAAGAAATGTTCTTTTATTCACCTGACTATAAATTAAACGAAGAAATTAAAAATAATAC
TATTAATGGTCAAATTATTAATAGAAACAATAAAATATTAAATGTTGAATTTAATTATACAAATGT
AGTTATGTCTAGTTCTGTGGCAATAACTATTTCTACTATTACATTAGTAGTTTTATTATTCATGTTT
ATGAATAAAGAATGATTAAGTCTGGGTACTATATTGGTACAATCTTTATTTAGCTATTAATGAGTA
GTATGACATTATTTATAGGGTTTGTATCTGTTATAGCTATTTTATTTTTAGCCATTAATTTTATATTT
GCTCCTCATAATCCTTATCAAGAAAAATATAGTATTTTCGAGTGTGGTTTCCATAGTTTTTTAGGG
CAAAATAGAACACAATTCGGTATAAAATTCTTTATTTTTGCTTTAGTTTATTTACTTTTAGATTTAG
AAATATTATTAACTTTCCCTTTCGCTCTTAGTGAGTATGTTAATGGTATTTATGGTCTTTTAGTTAC
TTTAATTTTTATAGCTATAATAACTATAGGATTTATATTTGAATTAGGTAAAAGCGCTCTTAAAATA
GACAGCAGACAAAAATTATATATACCTAAATTGAACGTTAATTACCATACAGAGTATGTTGGAAT
AGGTAAGGTTTCTAAGTAAAGTTATAGAGGCAGAAAACCAAAAAACCTACCAAAGGGTAGCTA

ATGGGAAGCTATTAAAAATAAAAGATGATAACCTATATATAGTATAGTTACTATATATTTACTATA
ACTAGGATTATTATATATATATATATTATCTTATTGTATATTAAGATTATTATTATTATAAGGTATA
ATTAATATAGTATCTTATTGTATAAGAATATAATATATTAACCTATAATTAATTTTATTTTTTAATTT
TTAATTATAATTTTTTTTTATATCTAGATGCTTACACATCTACAGATGTAGAAGAGAACAAAATAT
TGTTGTTATAGTAGTGGATGATATAGAAAAATATTTTTATATTATTTATTTTTGTTAGGTAGCTTTT
GAAGTGTTTGATAGAGAGGATATATGGACGGTAGGAGGGTATTCATTTTAATGAACAGTGGATA
GTTTAAATTAACCTAGTTATAGTTTATGAATTTAAATTATAATTATATTAATGTAGGTTATGATAGA
ATTATGTATTGATCCTTAAGAGTTAAGAGAGATACGCCCACGTATAATACATATTGGTTTAGGGT
TGAGTATATATACTTAAGAGTTAAGAATATATATATACAATATATAATAAATATAGTAATATATTA
ATGGTATGGACTTAACCAGGTTTATATATTATCATTTGATAAACATTAATTATAATTTTTCTATTTA
TTATTATTATTATACTGTGAGATTAATAATTATTAAAATATAATATTACATAGTACGTGATATTTGG
GGATTTTATCTTATTAATTGGCAATTAATGATTCTAATCAAATTTTATTCTCTTTAGTTTAATGGTA
GAACAATGATCTTCTAATTCATTGGTTTTAGTTCGATTCTAAAAAGAGATGAGTAAATAATTTTCT
AGATCAGAAATACTACTTTTAACTACAAAAAGCTTACGCTTTTTAACATTTTTTTGATAAATAACA
ACTATTGTTAATATTTGGCTGTCTATTGGTTTAACTAACAATTACAAAATTTTTTCAATTTTTATAAT
ATAAATTTAAATGAGAATATTAAAAAGTCATTCATTATTAAAATTAGTGAATTCTTACCTTATCGA
TGCGTCACAACCAAGTAACATTAGTTACTTGTGAAATTTTGGTTCATTATTAGCTGTTTGTTTAAT
AGTACAAATTATTACCGGTATTACATTAGCTATGCATTATAGTCCTAGTGTAATGGAAGCTTTTAA
CTCAATAGAGCATATAATGAGAGATGTTAATAACGGGTGATTAGTTCGTTATCTACATAGTAATA
CAGCTTCTGCTTTCTTTTTCTTAGTGTATTTACACATAGGAAGAGGTATATATTACGGATCATATA
GAGCTCCTCGTACTTTAGTTTGAGCTATTGGTACTGTTATATTAATATTAATGATGGCTATCGGTT
TCCTAGGTTATGTTTTACCTTATGGACAGATGTCATTATGAGGTGCTACAGTTATTACTAATCTTA
TTAGTGCTATACCTTGAATAGGGCAAGATATTGTTGAATTCATTTGAGGTGGTTTTTCTGTTAATA
ATGCCACTTTAAACAGATTTTTTGCATTACATTTTGTATTGCCTTTTGTATTAGCTGCTTTAGTTTTA
ATGCACTTAATTGCACTTCATGATACTGCTGGTTCAAGCAATCCTCTTGGTGTTTCAGGTAATTAC
GATAGAATTACATTTGCTCCATATTTTTTATTTAAAGATTTAATTACTATTTTTATATTTATTTTTGTA
TTAAGTGCTTTTGTATTCTTTATGCCTAATGTTTTAGGGGATAGTGATAATTATATTATGGCTAATC
CTATGCAAACTCCTGCTGCTATTGTACCTGAATGATACTTATTACCTTTCTATGCTATTTTAAGATC
TATACCTAATAAATTATTAGGTGTTATAGCGATGTTTAGTGCTATTTTAGCTATTATGTTATTACCT
GTTACAGATTTAGGTAGATCTAGAGGTTTACAATTTAGACCATTTAGTAAAATAGCTTTCTGAGTT
TTTGTTGCTAATTTCTTAGTTTTAATGCAATTAGGTGCTAAACACGTTGAAGATCCATTTATATTAT
TAGGTCAATTAAGTACTGTATTATACTTTAGTTATTTTGTTGCTATATTACCTTTAGCTAGTTACTT
AGATAATAGTTTAACTGATTTATCTAATAAATCTGAATTATTTTTAAATAAAACTAACTAAATATAT
TAAGATTATTATTTAATATATTTTCTATTTAAGATACTATTAATTTAGTATTTTGGGTTTTCAGTTTA
TAATTTATATTATATTATGCATTACCCTCCACCTTGCTTTGTAGTAAGCTAATCTGTTATTTCCTTTA
GTTTAATGGTAGAACAATGATCTTCTAATTCATTGGTTTTAGTTCGAATCTAAAAAGGAAATAAG
AAATATATTCTTATTATTACTTATATAATAATTATTTCTTAAAAATATACATTTTGCATTATAGCCGT
TTAGCTGTATTAAAATGTAAAATGATATAAAATAGAATAAATATTTAAATTATTCCTATGTTATATT
ATCCTATATTGCAACCATTATCAGAAGTTGTATTAATACTTGTACCTGCCTTATTAGCTGTAGCTT
ATGTTACAGTTGCTGAAAGAAAAACTATGGCTAGTATGCAAAGAAGATTAGGTCCTAATGCTGT
AGGTTACTATGGACTATTGCAAGCATTTGCTGATGCCTTAAAACTTTTATTAAAAGAATATGTAG
CTCCTACACAATCTAATATTGTTCTTTTCTTTTTAGGTCCTGTAATAACTTTAATTTTTGCATTATTA
GGTTACGCTGTTATACCCTATGGTCCTGGTTCAGGGATAAGCGACATGAATTTAGGTATATTTTA
CATGTTAGCTGTGTCATCTTTAGCTACATACGGTATTCTATTAGCTGGTTGAAGTGCGAATAGTA

AATACGCTTTTCTAGGTTCTCTTAGAAGTACAGCTCAATTAATTAGTTATGAATTAATATTAAGTT
CAGCTATATTAATAGTAATTATGATAACAGGAAATTTAAATTTAACTGTTTGTACTGAATCTCAAA
GAGCTATTTGATTTATACTACCTTTATTTCCTGTGTTTATAATATTTTTCATAGGATCTATAGCTGA
GACAAATAGAGCTCCTTTTGATTTAGCCGAGGCTAACCTGCTAATCTGGTTTGGTCTGGTTATAT
GTCACAAATTGCTAGGAAACCTTTTTATTTTAAAAACAAAAGACAATTAGCAGGAAACTTAATTT
AACCTAATTAAAAATATTAGATAATTAAACTCTTCATAGACTAAACGTGACAATTTAATATATATA
TATATTTATTTATATATATGATTAAATAAGATATAGTCAATCATCGGTGTGAATCGACTTAAAAAA
AAAAGCACATGGGTTAAACCCATCTCCCCTTATTAGGGGAATCAGAACTTGTTAGTGGGTTCAT
GACAGAGCATGCTGCCGTAGTTTTCGTATTCTTCTTTTTAGCTGAGTACGGTAGTATTGTACTAAT
GTGTATTTTAACTAGTATATTATTTATTGGTGGTTACTTATTATTTGAAATATCCTATGTTTTTACTG
TGGTAAATTATATTTTCTTTGAATTATTCTTTATAGACTGAGTAACATTTGTAGAGGTACAATCTTT
ATACACTGATTTTTTAAATAATTCTATCATTGAAGGATTATTATATGGGTTTAATCTAGGATTAAA
AAGTTCTTTAATGATATTCACATTTATTTGAGCTAGAGCATCCTTCCCTAGAATACGATTTGATCA
ACTAATGGGCTTCTGTTGAACAGTTTTATTACCTATTAATTTTGCAATTATTATATTAGTACCTTGT
GTTTTATATAGTTTTAACTTATTACCTGTAAATATACCATTGTTCTAGCTCACACACCCGCCCGCC
CTACTGCCACAAGGCTACAGTACATATGAGGAGGGGAACTAAAGATCTAGAACTATCCTAGTTA
ATAATTACACTTAATAGTATACTAAATAGACCATCTATCATACTCGAGAATAGTGATAGTGTAAT
TATACACTATTTTAACTGTATTCTACACTATTAGCATATTATTATCTTTATATGATAATAATTTTATA
ACTTAATTTATTAGGTTACATTAACAAAAAAATTCGTTAACTTTTTTCACCACTTTTTAATACAAAAT
ACGAATTAAA
>YN228
ATAACAATTCTAAAGAACATAAAGAGTTATTAGATAAAAATAATTCACCTATACAGTTAATAAAT
CAACTTAAAGGGTATTTTTTCATAAATCCTTTATTAGCTTTAAGTTTAGCTATTACTATTTTCTCTTT
TGCAGGTATTCCTCCTCTTGTAGGGTTCTTTGCTAAACAGATGGTATTAAGCGCGGCTATTGATC
AAGGTTATATCTTTTTATCTTTAGTTGCAATATTAACTAGTGTTATAGGAGGGGTTTATTATTTAAA
TATAATTAAAGAAATGTTCTTTTATTCACCTGACTATAAATTAAACGAAGAAATTAAAAATAATAC
TATTAATGGTCAAATTATTAATAGAAACAATAAAATATTAAATGTTGAATTTAATTATACAAATGT
AGTTATGTCTAGTTCTGTGGCAATAACTATTTCTACTATTACATTAGTAGTTTTATTATTCATGTTT
ATGAATAAAGAATGATTAAGTCTGGGTACTATATTGGTACAATCTTTATTTAGCTATTAATGAGTA
GTATGACATTATTTATAGGGTTTGTATCTGTTATAGCTATTTTATTTTTAGCCATTAATTTTATATTT
GCTCCTCATAATCCTTATCAAGAAAAATATAGTATTTTCGAGTGTGGTTTCCATAGTTTTTTAGGG
CAAAATAGAACACAATTCGGTATAAAATTCTTTATTTTTGCTTTAGTTTATTTACTTTTAGATTTAG
AAATATTATTAACTTTCCCTTTCGCTCTTAGTGAGTATGTTAATGGTATTTATGGTCTTTTAGTTAC
TTTAATTTTTATAGCTATAATAACTATAGGATTTATATTTGAATTAGGTAAAAGCGCTCTTAAAATA
GACAGCAGACAAAAATTATATATACCTAAATTGAACGTTAATTACCATACAGAGTATGTTGGAAT
AGGTAAGGTTTCTAAGTAAAGTTATAGAGGCAGAAAACCAAAAAACCTACCAAAGGGTAGCTA
ATGGGAAGCTATTAAAAATAAAAGATGATAACCTATATATAGTATAGTTACTATATATTTACTATA
ACTAGGATTATTATATATATATATATTATCTTATTGTATATTAAGATTATTATTATTATAAGGTATA
ATTAATATAGTATCTTATTGTATAAGAATATAATATATTAACCTATAATTAATTTTATTTTTTAATTT
TTAATTATAATTTTTTTTTATATCTAGATGCTTACACATCTACAGATGTAGAAGAGAACAAAATAT
TGTTGTTATAGTAGTGGATGATATAGAAAAATATTTTTATATTATTTATTTTTGTTAGGTAGCTTTT
GAAGTGTTTGATAGAGAGGATATATGGACGGTAGGAGGGTATTCATTTTAATGAACAGTGGATA
GTTTAAATTAACCTAGTTATAGTTTATGAATTTAAATTATAATTATATTAATGTAGGTTATGATAGA
ATTATGTATTGATCCTTAAGAGTTAAGAGAGATACGCCCACGTATAATACATATTGGTTTAGGGT

TGAGTATATATACTTAAGAGTTAAGAATATATATATACAATATATAATAAATATAGTAATATATTA
ATGGTATGGACTTAACCAGGTTTATATATTATCATTTGATAAACATTAATTATAATTTTTCTATTTA
TTATTATTATTATACTGTGAGATTAATAATTATTAAAATATAATATTACATAGTACGTGATATTTGG
GGATTTTATCTTATTAATTGGCAATTAATGATTCTAATCAAATTTTATTCTCTTTAGTTTAATGGTA
GAACAATGATCTTCTAATTCATTGGTTTTAGTTCGATTCTAAAAAGAGATGAGTAAATAATTTTCT
AGATCAGAAATACTACTTTTAACTACAAAAAGCTTACGCTTTTTAACATTTTTTTGATAAATAACA
ACTATTGTTAATATTTGGCTGTCTATTGGTTTAACTAACAATTACAAAATTTTTTCAATTTTTATAAT
ATAAATTTAAATGAGAATATTAAAAAGTCATTCATTATTAAAATTAGTGAATTCTTACCTTATCGA
TGCGTCACAACCAAGTAACATTAGTTACTTGTGAAATTTTGGTTCATTATTAGCTGTTTGTTTAAT
AGTACAAATTATTACCGGTATTACATTAGCTATGCATTATAGTCCTAGTGTAATGGAAGCTTTTAA
CTCAATAGAGCATATAATGAGAGATGTTAATAACGGGTGATTAGTTCGTTATCTACATAGTAATA
CAGCTTCTGCTTTCTTTTTCTTAGTGTATTTACACATAGGAAGAGGTATATATTACGGATCATATA
GAGCTCCTCGTACTTTAGTTTGAGCTATTGGTACTGTTATATTAATATTAATGATGGCTATCGGTT
TCCTAGGTTATGTTTTACCTTATGGACAGATGTCATTATGAGGTGCTACAGTTATTACTAATCTTA
TTAGTGCTATACCTTGAATAGGGCAAGATATTGTTGAATTCATTTGAGGTGGTTTTTCTGTTAATA
ATGCCACTTTAAACAGATTTTTTGCATTACATTTTGTATTGCCTTTTGTATTAGCTGCTTTAGTTTTA
ATGCACTTAATTGCACTTCATGATACTGCTGGTTCAAGCAATCCTCTTGGTGTTTCAGGTAATTAC
GATAGAATTACATTTGCTCCATATTTTTTATTTAAAGATTTAATTACTATTTTTATATTTATTTTTGTA
TTAAGTGCTTTTGTATTCTTTATGCCTAATGTTTTAGGGGATAGTGATAATTATATTATGGCTAATC
CTATGCAAACTCCTGCTGCTATTGTACCTGAATGATACTTATTACCTTTCTATGCTATTTTAAGATC
TATACCTAATAAATTATTAGGTGTTATAGCGATGTTTAGTGCTATTTTAGCTATTATGTTATTACCT
GTTACAGATTTAGGTAGATCTAGAGGTTTACAATTTAGACCATTTAGTAAAATAGCTTTCTGAGTT
TTTGTTGCTAATTTCTTAGTTTTAATGCAATTAGGTGCTAAACACGTTGAAGATCCATTTATATTAT
TAGGTCAATTAAGTACTGTATTATACTTTAGTTATTTTGTTGCTATATTACCTTTAGCTAGTTACTT
AGATAATAGTTTAACTGATTTATCTAATAAATCTGAATTATTTTTAAATAAAACTAACTAAATATAT
TAAGATTATTATTTAATATATTTTCTATTTAAGATACTATTAATTTAGTATTTTGGGTTTTCAGTTTA
TAATTTATATTATATTATGCATTACCCTCCACCTTGCTTTGTAGTAAGCTAATCTGTTATTTCCTTTA
GTTTAATGGTAGAACAATGATCTTCTAATTCATTGGTTTTAGTTCGAATCTAAAAAGGAAATAAG
AAATATATTCTTATTATTACTTATATAATAATTATTTCTTAAAAATATACATTTTGCATTATAGCCGT
TTAGCTGTATTAAAATGTAAAATGATATAAAATAGAATAAATATTTAAATTATTCCTATGTTATATT
ATCCTATATTGCAACCATTATCAGAAGTTGTATTAATACTTGTACCTGCCTTATTAGCTGTAGCTT
ATGTTACAGTTGCTGAAAGAAAAACTATGGCTAGTATGCAAAGAAGATTAGGTCCTAATGCTGT
AGGTTACTATGGACTATTGCAAGCATTTGCTGATGCCTTAAAACTTTTATTAAAAGAATATGTAG
CTCCTACACAATCTAATATTGTTCTTTTCTTTTTAGGTCCTGTAATAACTTTAATTTTTGCATTATTA
GGTTACGCTGTTATACCCTATGGTCCTGGTTCAGGGATAAGCGACATGAATTTAGGTATATTTTA
CATGTTAGCTGTGTCATCTTTAGCTACATACGGTATTCTATTAGCTGGTTGAAGTGCGAATAGTA
AATACGCTTTTCTAGGTTCTCTTAGAAGTACAGCTCAATTAATTAGTTATGAATTAATATTAAGTT
CAGCTATATTAATAGTAATTATGATAACAGGAAATTTAAATTTAACTGTTTGTACTGAATCTCAAA
GAGCTATTTGATTTATACTACCTTTATTTCCTGTGTTTATAATATTTTTCATAGGATCTATAGCTGA
GACAAATAGAGCTCCTTTTGATTTAGCCGAGGCTAACCTGCTAATCTGGTTTGGTCTGGTTATAT
GTCACAAATTGCTAGGAAACCTTTTTATTTTAAAAACAAAAGACAATTAGCAGGAAACTTAATTT
AACCTAATTAAAAATATTAGATAATTAAACTCTTCATAGACTAAACGTGACAATTTAATATATATA
TATATTTATTTATATATATGATTAAATAAGATATAGTCAATCATCGGTGTGAATCGACTTAAAAAA
AAAAGCACATGGGTTAAACCCATCTCCCCTTATTAGGGGAATCAGAACTTGTTAGTGGGTTCAT

GACAGAGCATGCTGCCGTAGTTTTCGTATTCTTCTTTTTAGCTGAGTACGGTAGTATTGTACTAAT
GTGTATTTTAACTAGTATATTATTTATTGGTGGTTACTTATTATTTGAAATATCCTATGTTTTTACTG
TGGTAAATTATATTTTCTTTGAATTATTCTTTATAGACTGAGTAACATTTGTAGAGGTACAATCTTT
ATACACTGATTTTTTAAATAATTCTATCATTGAAGGATTATTATATGGGTTTAATCTAGGATTAAA
AAGTTCTTTAATGATATTCACATTTATTTGAGCTAGAGCATCCTTCCCTAGAATACGATTTGATCA
ACTAATGGGCTTCTGTTGAACAGTTTTATTACCTATTAATTTTGCAATTATTATATTAGTACCTTGT
GTTTTATATAGTTTTAACTTATTACCTGTAAATATACCATTGTTCTAGCTCACACACCCGCCCGCC
CTACTGCCACAAGGCTACAGTACATATGAGGAGGGGAACTAAAGATCTAGAACTATCCTAGTTA
ATAATTACACTTAATAGTATACTAAATAGACCATCTATCATACTCGAGAATAGTGATAGTGTAAT
TATACACTATTTTAACTGTATTCTACACTATTAGCATATTATTATCTTTATATGATAATAATTTTATA
ACTTAATTTATTAGGTTACATTAACAAAAAATTCGTTAACTTTTTTCACCACTTTTTAATACAAAAT
ACGAATTATA
>YN232
ATAACAATTCTAAAGAACATAAAGAGTTATTAGATAAAAATAATTCACCTATACAGTTAATAAAT
CAACTTAAAGGGTATTTTTTCATAAATCCTTTATTAGCTTTAAGTTTAGCTATTACTATTTTCTCTTT
TGCAGGTATTCCTCCTCTTGTAGGGTTCTTTGCTAAACAGATGGTATTAAGCGCGGCTATTGATC
AAGGTTATATCTTTTTATCTTTAGTTGCAATATTAACTAGTGTTATAGGAGGGGTTTATTATTTAAA
TATAATTAAAGAAATGTTCTTTTATTCACCTGACTATAAATTAAACGAAGAAATTAAAAATAATAC
TATTAATGGTCAAATTATTAATAGAAACAATAAAATATTAAATGTTGAATTTAATTATACAAATGT
AGTTATGTCTAGTTCTGTGGCAATAACTATTTCTACTATTACATTAGTAGTTTTATTATTCATGTTT
ATGAATAAAGAATGATTAAGTCTGGGTACTATATTGGTACAATCTTTATTTAGCTATTAATGAGTA
GTATGACATTATTTATAGGGTTTGTATCTGTTATAGCTATTTTATTTTTAGCCATTAATTTTATATTT
GCTCCTCATAATCCTTATCAAGAAAAATATAGTATTTTCGAGTGTGGTTTCCATAGTTTTTTAGGG
CAAAATAGAACACAATTCGGTATAAAATTCTTTATTTTTGCTTTAGTTTATTTACTTTTAGATTTAG
AAATATTATTAACTTTCCCTTTCGCTCTTAGTGAGTATGTTAATGGTATTTATGGTCTTTTAGTTAC
TTTAATTTTTATAGCTATAATAACTATAGGATTTATATTTGAATTAGGTAAAAGCGCTCTTAAAATA
GACAGCAGACAAAAATTATATATACCTAAATTGAACGTTAATTACCATACAGAGTATGTTGGAAT
AGGTAAGGTTTCTAAGTAAAGTTATAGAGGCAGAAAACCAAAAAACCTACCAAAGGGTAGCTA
ATGGGAAGCTATTAAAAATAAAAGATGATAACCTATATATAGTATAGTTACTATATATTTACTATA
ACTAGGATTATTATATATATATATATTATCTTATTGTATATTAAGATTATTATTATTATAAGGTATA
ATTAATATAGTATCTTATTGTATAAGAATATAATATATTAACCTATAATTAATTTTATTTTTTAATTT
TTAATTATAATTTTTTTTTATATCTAGATGCTTACACATCTACAGATGTAGAAGAGAACAAAATAT
TGTTGTTATAGTAGTGGATGATATAGAAAAATATTTTTATATTATTTATTTTTGTTAGGTAGCTTTT
GAAGTGTTTGATAGAGAGGATATATGGACGGTAGGAGGGTATTCATTTTAATGAACAGTGGATA
GTTTAAATTAACCTAGTTATAGTTTATGAATTTAAATTATAATTATATTAATGTAGGTTATGATAGA
ATTATGTATTGATCCTTAAGAGTTAAGAGAGATACGCCCACGTATAATACATATTGGTTTAGGGT
TGAGTATATATACTTAAGAGTTAAGAATATATATATACAATATATAATAAATATAGTAATATATTA
ATGGTATGGACTTAACCAGGTTTATATATTATCATTTGATAAACATTAATTATAATTTTTCTATTTA
TTATTATTATTATACTGTGAGATTAATAATTATTAAAATATAATATTACATAGTACGTGATATTTGG
GGATTTTATCTTATTAATTGGCAATTAATGATTCTAATCAAATTTTATTCTCTTTAGTTTAATGGTA
GAACAATGATCTTCTAATTCATTGGTTTTAGTTCGATTCTAAAAAGAGATGAGTAAATAATTTTCT
AGATCAGAAATACTACTTTTAACTACAAAAAGCTTACGCTTTTTAACATTTTTTTGATAAATAACA
ACTATTGTTAATATTTGGCTGTCTATTGGTTTAACTAACAATTACAAAATTTTTTCAATTTTTATAAT
ATAAATTTAAATGAGAATATTAAAAAGTCATTCATTATTAAAATTAGTGAATTCTTACCTTATCGA

TGCGTCACAACCAAGTAACATTAGTTACTTGTGAAATTTTGGTTCATTATTAGCTGTTTGTTTAAT
AGTACAAATTATTACCGGTATTACATTAGCTATGCATTATAGTCCTAGTGTAATGGAAGCTTTTAA
CTCAATAGAGCATATAATGAGAGATGTTAATAACGGGTGATTAGTTCGTTATCTACATAGTAATA
CAGCTTCTGCTTTCTTTTTCTTAGTGTATTTACACATAGGAAGAGGTATATATTACGGATCATATA
GAGCTCCTCGTACTTTAGTTTGAGCTATTGGTACTGTTATATTAATATTAATGATGGCTATCGGTT
TCCTAGGTTATGTTTTACCTTATGGACAGATGTCATTATGAGGTGCTACAGTTATTACTAATCTTA
TTAGTGCTATACCTTGAATAGGGCAAGATATTGTTGAATTCATTTGAGGTGGTTTTTCTGTTAATA
ATGCCACTTTAAACAGATTTTTTGCATTACATTTTGTATTGCCTTTTGTATTAGCTGCTTTAGTTTTA
ATGCACTTAATTGCACTTCATGATACTGCTGGTTCAAGCAATCCTCTTGGTGTTTCAGGTAATTAC
GATAGAATTACATTTGCTCCATATTTTTTATTTAAAGATTTAATTACTATTTTTATATTTATTTTTGTA
TTAAGTGCTTTTGTATTCTTTATGCCTAATGTTTTAGGGGATAGTGATAATTATATTATGGCTAATC
CTATGCAAACTCCTGCTGCTATTGTACCTGAATGATACTTATTACCTTTCTATGCTATTTTAAGATC
TATACCTAATAAATTATTAGGTGTTATAGCGATGTTTAGTGCTATTTTAGCTATTATGTTATTACCT
GTTACAGATTTAGGTAGATCTAGAGGTTTACAATTTAGACCATTTAGTAAAATAGCTTTCTGAGTT
TTTGTTGCTAATTTCTTAGTTTTAATGCAATTAGGTGCTAAACACGTTGAAGATCCATTTATATTAT
TAGGTCAATTAAGTACTGTATTATACTTTAGTTATTTTGTTGCTATATTACCTTTAGCTAGTTACTT
AGATAATAGTTTAACTGATTTATCTAATAAATCTGAATTATTTTTAAATAAAACTAACTAAATATAT
TAAGATTATTATTTAATATATTTTCTATTTAAGATACTATTAATTTAGTATTTTGGGTTTTCAGTTTA
TAATTTATATTATATTATGCATTACCCTCCACCTTGCTTTGTAGTAAGCTAATCTGTTATTTCCTTTA
GTTTAATGGTAGAACAATGATCTTCTAATTCATTGGTTTTAGTTCGAATCTAAAAAGGAAATAAG
AAATATATTCTTATTATTACTTATATAATAATTATTTCTTAAAAATATACATTTTGCATTATAGCCGT
TTAGCTGTATTAAAATGTAAAATGATATAAAATAGAATAAATATTTAAATTATTCCTATGTTATATT
ATCCTATATTGCAACCATTATCAGAAGTTGTATTAATACTTGTACCTGCCTTATTAGCTGTAGCTT
ATGTTACAGTTGCTGAAAGAAAAACTATGGCTAGTATGCAAAGAAGATTAGGTCCTAATGCTGT
AGGTTACTATGGACTATTGCAAGCATTTGCTGATGCCTTAAAACTTTTATTAAAAGAATATGTAG
CTCCTACACAATCTAATATTGTTCTTTTCTTTTTAGGTCCTGTAATAACTTTAATTTTTGCATTATTA
GGTTACGCTGTTATACCCTATGGTCCTGGTTCAGGGATAAGCGACATGAATTTAGGTATATTTTA
CATGTTAGCTGTGTCATCTTTAGCTACATACGGTATTCTATTAGCTGGTTGAAGTGCGAATAGTA
AATACGCTTTTCTAGGTTCTCTTAGAAGTACAGCTCAATTAATTAGTTATGAATTAATATTAAGTT
CAGCTATATTAATAGTAATTATGATAACAGGAAATTTAAATTTAACTGTTTGTACTGAATCTCAAA
GAGCTATTTGATTTATACTACCTTTATTTCCTGTGTTTATAATATTTTTCATAGGATCTATAGCTGA
GACAAATAGAGCTCCTTTTGATTTAGCCGAGGCTAACCTGCTAATCTGGTTTGGTCTGGTTATAT
GTCACAAATTGCTAGGAAACCTTTTTATTTTAAAAACAAAAGACAATTAGCAGGAAACTTAATTT
AACCTAATTAAAAATATTAGATAATTAAACTCTTCATAGACTAAACGTGACAATTTAATATATATA
TATATTTATTTATATATATGATTAAATAAGATATAGTCAATCATCGGTGTGAATCGACTTAAAAAA
AAAAGCACATGGGTTAAACCCATCTCCCCTTATTAGGGGAATCAGAACTTGTTAGTGGGTTCAT
GACAGAGCATGCTGCCGTAGTTTTCGTATTCTTCTTTTTAGCTGAGTACGGTAGTATTGTACTAAT
GTGTATTTTAACTAGTATATTATTTATTGGTGGTTACTTATTATTTGAAATATCCTATGTTTTTACTG
TGGTAAATTATATTTTCTTTGAATTATTCTTTATAGACTGAGTAACATTTGTAGAGGTACAATCTTT
ATACACTGATTTTTTAAATAATTCTATCATTGAAGGATTATTATATGGGTTTAATCTAGGATTAAA
AAGTTCTTTAATGATATTCACATTTATTTGAGCTAGAGCATCCTTCCCTAGAATACGATTTGATCA
ACTAATGGGCTTCTGTTGAACAGTTTTATTACCTATTAATTTTGCAATTATTATATTAGTACCTTGT
GTTTTATATAGTTTTAACTTATTACCTGTAAATATACCATTGTTCTAGCTCACACACCCGCCCGCC
CTACTGCCACAAGGCTACAGTACATATGAGGAGGGGAACTAAAGATCTAGAACTATCCTAGTTA

ATAATTACACTTAATAGTATACTAAATAGACCATCTATCATACTCGAGAATAGTGATAGTGTAAT
TATACACTATTTTAACTGTATTCTACACTATTAGCATATTATTATCTTTATATGATAATAATTTTATA
ACTTAATTTATTAGGTTACATTAACAAAAAATTCGTTAACTTTTTTCACCACTTTTTAATACAAAAT
ACGAATTTTA
>YN239
ATAACAATTCTAAAGAACATAAAGAGTTATTAGATAAAAATAATTCACCTATACAGTTAATAAAT
CAACTTAAAGGGTATTTTTTCATAAATCCTTTATTAGCTTTAAGTTTAGCTATTACTATTTTCTCTTT
TGCAGGTATTCCTCCTCTTGTAGGGTTCTTTGCTAAACAGATGGTATTAAGCGCGGCTATTGATC
AAGGTTATATCTTTTTATCTTTAGTTGCAATATTAACTAGTGTTATAGGAGGGGTTTATTATTTAAA
TATAATTAAAGAAATGTTCTTTTATTCACCTGACTATAAATTAAACGAAGAAATTAAAAATAATAC
TATTAATGGTCAAATTATTAATAGAAACAATAAAATATTAAATGTTGAATTTAATTATACAAATGT
AGTTATGTCTAGTTCTGTGGCAATAACTATTTCTACTATTACATTAGTAGTTTTATTATTCATGTTT
ATGAATAAAGAATGATTAAGTCTGGGTACTATATTGGTACAATCTTTATTTAGCTATTAATGAGTA
GTATGACATTATTTATAGGGTTTGTATCTGTTATAGCTATTTTATTTTTAGCCATTAATTTTATATTT
GCTCCTCATAATCCTTATCAAGAAAAATATAGTATTTTCGAGTGTGGTTTCCATAGTTTTTTAGGG
CAAAATAGAACACAATTCGGTATAAAATTCTTTATTTTTGCTTTAGTTTATTTACTTTTAGATTTAG
AAATATTATTAACTTTCCCTTTCGCTCTTAGTGAGTATGTTAATGGTATTTATGGTCTTTTAGTTAC
TTTAATTTTTATAGCTATAATAACTATAGGATTTATATTTGAATTAGGTAAAAGCGCTCTTAAAATA
GACAGCAGACAAAAAATTATATATACCTAAATTGAACGTTAATTACCATACAGAGTATGTTGGAAT
AGGTAAGGTTTCTAAGTAAAGTTATAGAGGCAGAAAACCAAAAAACCTACCAAAGGGTAGCTA
ATGGGAAGCTATTAAAAATAAAAGATGATAACCTATATATAGTATAGTTACTATATATTTACTATA
ACTAGGATTATTATATATATATATATTATCTTATTGTATATTAAGATTATTATTATTATAAGGTATA
ATTAATATAGTATCTTATTGTATAAGAATATAATATATTAACCTATAATTAATTTTATTTTTTAATTT
TTAATTATAATTTTTTTTTATATCTAGATGCTTACACATCTACAGATGTAGAAGAGAACAAAATAT
TGTTGTTATAGTAGTGGATGATATAGAAAAATATTTTTATATTATTTATTTTTGTTAGGTAGCTTTT
GAAGTGTTTGATAGAGAGGATATATGGACGGTAGGAGGGTATTCATTTTAATGAACAGTGGATA
GTTTAAATTAACCTAGTTATAGTTTATGAATTTAAATTATAATTATATTAATGTAGGTTATGATAGA
ATTATGTATTGATCCTTAAGAGTTAAGAGAGATACGCCCACGTATAATACATATTGGTTTAGGGT
TGAGTATATATACTTAAGAGTTAAGAATATATATATACAATATATAATAAATATAGTAATATATTA
ATGGTATGGACTTAACCAGGTTTATATATTATCATTTGATAAACATTAATTATAATTTTTCTATTTA
TTATTATTATTATACTGTGAGATTAATAATTATTAAAATATAATATTACATAGTACGTGATATTTGG
GGATTTTATCTTATTAATTGGCAATTAATGATTCTAATCAAATTTTATTCTCTTTAGTTTAATGGTA
GAACAATGATCTTCTAATTCATTGGTTTTAGTTCGATTCTAAAAAGAGATGAGTAAATAATTTTCT
AGATCAGAAATACTACTTTTAACTACAAAAAGCTTACGCTTTTTAACATTTTTTTGATAAATAACA
ACTATTGTTAATATTTGGCTGTCTATTGGTTTAACTAACAATTACAAAATTTTTTCAATTTTTATAAT
ATAAATTTAAATGAGAATATTAAAAAGTCATTCATTATTAAAATTAGTGAATTCTTACCTTATCGA
TGCGTCACAACCAAGTAACATTAGTTACTTGTGAAATTTTGGTTCATTATTAGCTGTTTGTTTAAT
AGTACAAATTATTACCGGTATTACATTAGCTATGCATTATAGTCCTAGTGTAATGGAAGCTTTTAA
CTCAATAGAGCATATAATGAGAGATGTTAATAACGGGTGATTAGTTCGTTATCTACATAGTAATA
CAGCTTCTGCTTTCTTTTTCTTAGTGTATTTACACATAGGAAGAGGTATATATTACGGATCATATA
GAGCTCCTCGTACTTTAGTTTGAGCTATTGGTACTGTTATATTAATATTAATGATGGCTATCGGTT
TCCTAGGTTATGTTTTACCTTATGGACAGATGTCATTATGAGGTGCTACAGTTATTACTAATCTTA
TTAGTGCTATACCTTGAATAGGGCAAGATATTGTTGAATTCATTTGAGGTGGTTTTTCTGTTAATA
ATGCCACTTTAAACAGATTTTTTGCATTACATTTTGTATTGCCTTTTGTATTAGCTGCTTTAGTTTTA

ATGCACTTAATTGCACTTCATGATACTGCTGGTTCAAGCAATCCTCTTGGTGTTTCAGGTAATTAC
GATAGAATTACATTTGCTCCATATTTTTTATTTAAAGATTTAATTACTATTTTTATATTTATTTTTGTA
TTAAGTGCTTTTGTATTCTTTATGCCTAATGTTTTAGGGGATAGTGATAATTATATTATGGCTAATC
CTATGCAAACTCCTGCTGCTATTGTACCTGAATGATACTTATTACCTTTCTATGCTATTTTAAGATC
TATACCTAATAAATTATTAGGTGTTATAGCGATGTTTAGTGCTATTTTAGCTATTATGTTATTACCT
GTTACAGATTTAGGTAGATCTAGAGGTTTACAATTTAGACCATTTAGTAAAATAGCTTTCTGAGTT
TTTGTTGCTAATTTCTTAGTTTTAATGCAATTAGGTGCTAAACACGTTGAAGATCCATTTATATTAT
TAGGTCAATTAAGTACTGTATTATACTTTAGTTATTTTGTTGCTATATTACCTTTAGCTAGTTACTT
AGATAATAGTTTAACTGATTTATCTAATAAATCTGAATTATTTTTAAATAAAACTAACTAAATATAT
TAAGATTATTATTTAATATATTTTCTATTTAAGATACTATTAATTTAGTATTTTGGGTTTTCAGTTTA
TAATTTATATTATATTATGCATTACCCTCCACCTTGCTTTGTAGTAAGCTAATCTGTTATTTCCTTTA
GTTTAATGGTAGAACAATGATCTTCTAATTCATTGGTTTTAGTTCGAATCTAAAAAGGAAATAAG
AAATATATTCTTATTATTACTTATATAATAATTATTTCTTAAAAATATACATTTTGCATTATAGCCGT
TTAGCTGTATTAAAATGTAAAATGATATAAAATAGAATAAATATTTAAATTATTCCTATGTTATATT
ATCCTATATTGCAACCATTATCAGAAGTTGTATTAATACTTGTACCTGCCTTATTAGCTGTAGCTT
ATGTTACAGTTGCTGAAAGAAAAACTATGGCTAGTATGCAAAGAAGATTAGGTCCTAATGCTGT
AGGTTACTATGGACTATTGCAAGCATTTGCTGATGCCTTAAAACTTTTATTAAAAGAATATGTAG
CTCCTACACAATCTAATATTGTTCTTTTCTTTTTAGGTCCTGTAATAACTTTAATTTTTGCATTATTA
GGTTACGCTGTTATACCCTATGGTCCTGGTTCAGGGATAAGCGACATGAATTTAGGTATATTTTA
CATGTTAGCTGTGTCATCTTTAGCTACATACGGTATTCTATTAGCTGGTTGAAGTGCGAATAGTA
AATACGCTTTTCTAGGTTCTCTTAGAAGTACAGCTCAATTAATTAGTTATGAATTAATATTAAGTT
CAGCTATATTAATAGTAATTATGATAACAGGAAATTTAAATTTAACTGTTTGTACTGAATCTCAAA
GAGCTATTTGATTTATACTACCTTTATTTCCTGTGTTTATAATATTTTTCATAGGATCTATAGCTGA
GACAAATAGAGCTCCTTTTGATTTAGCCGAGGCTAACCTGCTAATCTGGTTTGGTCTGGTTATAT
GTCACAAATTGCTAGGAAACCTTTTTATTTTAAAAACAAAAGACAATTAGCAGGAAACTTAATTT
AACCTAATTAAAAATATTAGATAATTAAACTCTTCATAGACTAAACGTGACAATTTAATATATATA
TATATTTATTTATATATATGATTAAATAAGATATAGTCAATCATCGGTGTGAATCGACTTAAAAAA
AAAAGCACATGGGTTAAACCCATCTCCCCTTATTAGGGGAATCAGAACTTGTTAGTGGGTTCAT
GACAGAGCATGCTGCCGTAGTTTTCGTATTCTTCTTTTTAGCTGAGTACGGTAGTATTGTACTAAT
GTGTATTTTAACTAGTATATTATTTATTGGTGGTTACTTATTATTTGAAATATCCTATGTTTTTACTG
TGGTAAATTATATTTTCTTTGAATTATTCTTTATAGACTGAGTAACATTTGTAGAGGTACAATCTTT
ATACACTGATTTTTTAAATAATTCTATCATTGAAGGATTATTATATGGGTTTAATCTAGGATTAAA
AAGTTCTTTAATGATATTCACATTTATTTGAGCTAGAGCATCCTTCCCTAGAATACGATTTGATCA
ACTAATGGGCTTCTGTTGAACAGTTTTATTACCTATTAATTTTGCAATTATTATATTAGTACCTTGT
GTTTTATATAGTTTTAACTTATTACCTGTAAATATACCATTGTTCTAGCTCACACACCCGCCCGCC
CTACTGCCACAAGGCTACAGTACATATGAGGAGGGGAACTAAAGATCTAGAACTATCCTAGTTA
ATAATTACACTTAATAGTATACTAAATAGACCATCTATCATACTCGAGAATAGTGATAGTGTAAT
TATACACTATTTTAACTGTATTCTACACTATTAGCATATTATTATCTTTATATGATAATAATTTTATA
ACTTAATTTATTAGGTTACATTAACAAAAAATTCGTTAACTTTTTTCACCACTTTTTAATACAAAAT
ACGAATTTTA
>YN247
ATAACAATTCTAAAGAACATAAAGAGTTATTAGATAAAAATAATTCACCTATACAGTTAATAAAT
CAACTTAAAGGGTATTTTTTCATAAATCCTTTATTAGCTTTAAGTTTAGCTATTACTATTTTCTCTTT
TGCAGGTATTCCTCCTCTTGTAGGGTTCTTTGCTAAACAGATGGTATTAAGCGCGGCTATTGATC

AAGGTTATATCTTTTTATCTTTAGTTGCAATATTAACTAGTGTTATAGGAGGGGTTTATTATTTAAA
TATAATTAAAGAAATGTTCTTTTATTCACCTGACTATAAATTAAACGAAGAAATTAAAAATAATAC
TATTAATGGTCAAATTATTAATAGAAACAATAAAATATTAAATGTTGAATTTAATTATACAAATGT
AGTTATGTCTAGTTCTGTGGCAATAACTATTTCTACTATTACATTAGTAGTTTTATTATTCATGTTT
ATGAATAAAGAATGATTAAGTCTGGGTACTATATTGGTACAATCTTTATTTAGCTATTAATGAGTA
GTATGACATTATTTATAGGGTTTGTATCTGTTATAGCTATTTTATTTTTAGCCATTAATTTTATATTT
GCTCCTCATAATCCTTATCAAGAAAAATATAGTATTTTCGAGTGTGGTTTCCATAGTTTTTTAGGG
CAAAATAGAACACAATTCGGTATAAAATTCTTTATTTTTGCTTTAGTTTATTTACTTTTAGATTTAG
AAATATTATTAACTTTCCCTTTCGCTCTTAGTGAGTATGTTAATGGTATTTATGGTCTTTTAGTTAC
TTTAATTTTTATAGCTATAATAACTATAGGATTTATATTTGAATTAGGTAAAAGCGCTCTTAAAATA
GACAGCAGACAAAAATTATATATACCTAAATTGAACGTTAATTACCATACAGAGTATGTTGGAAT
AGGTAAGGTTTCTAAGTAAAGTTATAGAGGCAGAAAACCAAAAAACCTACCAAAGGGTAGCTA
ATGGGAAGCTATTAAAAATAAAAGATGATAACCTATATATAGTATAGTTACTATATATTTACTATA
ACTAGGATTATTATATATATATATATTATCTTATTGTATATTAAGATTATTATTATTATAAGGTATA
ATTAATATAGTATCTTATTGTATAAGAATATAATATATTAACCTATAATTAATTTTATTTTTTAATTT
TTAATTATAATTTTTTTTTATATCTAGATGCTTACACATCTACAGATGTAGAAGAGAACAAAATAT
TGTTGTTATAGTAGTGGATGATATAGAAAAATATTTTTATATTATTTATTTTTGTTAGGTAGCTTTT
GAAGTGTTTGATAGAGAGGATATATGGACGGTAGGAGGGTATTCATTTTAATGAACAGTGGATA
GTTTAAATTAACCTAGTTATAGTTTATGAATTTAAATTATAATTATATTAATGTAGGTTATGATAGA
ATTATGTATTGATCCTTAAGAGTTAAGAGAGATACGCCCACGTATAATACATATTGGTTTAGGGT
TGAGTATATATACTTAAGAGTTAAGAATATATATATACAATATATAATAAATATAGTAATATATTA
ATGGTATGGACTTAACCAGGTTTATATATTATCATTTGATAAACATTAATTATAATTTTTCTATTTA
TTATTATTATTATACTGTGAGATTAATAATTATTAAAATATAATATTACATAGTACGTGATATTTGG
GGATTTTATCTTATTAATTGGCAATTAATGATTCTAATCAAATTTTATTCTCTTTAGTTTAATGGTA
GAACAATGATCTTCTAATTCATTGGTTTTAGTTCGATTCTAAAAAGAGATGAGTAAATAATTTTCT
AGATCAGAAATACTACTTTTAACTACAAAAAGCTTACGCTTTTTAACATTTTTTTGATAAATAACA
ACTATTGTTAATATTTGGCTGTCTATTGGTTTAACTAACAATTACAAAATTTTTTCAATTTTTATAAT
ATAAATTTAAATGAGAATATTAAAAAGTCATTCATTATTAAAATTAGTGAATTCTTACCTTATCGA
TGCGTCACAACCAAGTAACATTAGTTACTTGTGAAATTTTGGTTCATTATTAGCTGTTTGTTTAAT
AGTACAAATTATTACCGGTATTACATTAGCTATGCATTATAGTCCTAGTGTAATGGAAGCTTTTAA
CTCAATAGAGCATATAATGAGAGATGTTAATAACGGGTGATTAGTTCGTTATCTACATAGTAATA
CAGCTTCTGCTTTCTTTTTCTTAGTGTATTTACACATAGGAAGAGGTATATATTACGGATCATATA
GAGCTCCTCGTACTTTAGTTTGAGCTATTGGTACTGTTATATTAATATTAATGATGGCTATCGGTT
TCCTAGGTTATGTTTTACCTTATGGACAGATGTCATTATGAGGTGCTACAGTTATTACTAATCTTA
TTAGTGCTATACCTTGAATAGGGCAAGATATTGTTGAATTCATTTGAGGTGGTTTTTCTGTTAATA
ATGCCACTTTAAACAGATTTTTTGCATTACATTTTGTATTGCCTTTTGTATTAGCTGCTTTAGTTTTA
ATGCACTTAATTGCACTTCATGATACTGCTGGTTCAAGCAATCCTCTTGGTGTTTCAGGTAATTAC
GATAGAATTACATTTGCTCCATATTTTTTATTTAAAGATTTAATTACTATTTTTATATTTATTTTTGTA
TTAAGTGCTTTTGTATTCTTTATGCCTAATGTTTTAGGGGATAGTGATAATTATATTATGGCTAATC
CTATGCAAACTCCTGCTGCTATTGTACCTGAATGATACTTATTACCTTTCTATGCTATTTTAAGATC
TATACCTAATAAATTATTAGGTGTTATAGCGATGTTTAGTGCTATTTTAGCTATTATGTTATTACCT
GTTACAGATTTAGGTAGATCTAGAGGTTTACAATTTAGACCATTTAGTAAAATAGCTTTCTGAGTT
TTTGTTGCTAATTTCTTAGTTTTAATGCAATTAGGTGCTAAACACGTTGAAGATCCATTTATATTAT
TAGGTCAATTAAGTACTGTATTATACTTTAGTTATTTTGTTGCTATATTACCTTTAGCTAGTTACTT

AGATAATAGTTTAACTGATTTATCTAATAAATCTGAATTATTTTTAAATAAAACTAACTAAATATAT
TAAGATTATTATTTAATATATTTTCTATTTAAGATACTATTAATTTAGTATTTTGGGTTTTCAGTTTA
TAATTTATATTATATTATGCATTACCCTCCACCTTGCTTTGTAGTAAGCTAATCTGTTATTTCCTTTA
GTTTAATGGTAGAACAATGATCTTCTAATTCATTGGTTTTAGTTCGAATCTAAAAAGGAAATAAG
AAATATATTCTTATTATTACTTATATAATAATTATTTCTTAAAAATATACATTTTGCATTATAGCCGT
TTAGCTGTATTAAAATGTAAAATGATATAAAATAGAATAAATATTTAAATTATTCCTATGTTATATT
ATCCTATATTGCAACCATTATCAGAAGTTGTATTAATACTTGTACCTGCCTTATTAGCTGTAGCTT
ATGTTACAGTTGCTGAAAGAAAAACTATGGCTAGTATGCAAAGAAGATTAGGTCCTAATGCTGT
AGGTTACTATGGACTATTGCAAGCATTTGCTGATGCCTTAAAACTTTTATTAAAAGAATATGTAG
CTCCTACACAATCTAATATTGTTCTTTTCTTTTTAGGTCCTGTAATAACTTTAATTTTTGCATTATTA
GGTTACGCTGTTATACCCTATGGTCCTGGTTCAGGGATAAGCGACATGAATTTAGGTATATTTTA
CATGTTAGCTGTGTCATCTTTAGCTACATACGGTATTCTATTAGCTGGTTGAAGTGCGAATAGTA
AATACGCTTTTCTAGGTTCTCTTAGAAGTACAGCTCAATTAATTAGTTATGAATTAATATTAAGTT
CAGCTATATTAATAGTAATTATGATAACAGGAAATTTAAATTTAACTGTTTGTACTGAATCTCAAA
GAGCTATTTGATTTATACTACCTTTATTTCCTGTGTTTATAATATTTTTCATAGGATCTATAGCTGA
GACAAATAGAGCTCCTTTTGATTTAGCCGAGGCTAACCTGCTAATCTGGTTTGGTCTGGTTATAT
GTCACAAATTGCTAGGAAACCTTTTTATTTTAAAAACAAAAGACAATTAGCAGGAAACTTAATTT
AACCTAATTAAAAATATTAGATAATTAAACTCTTCATAGACTAAACGTGACAATTTAATATATATA
TATATTTATTTATATATATGATTAAATAAGATATAGTCAATCATCGGTGTGAATCGACTTAAAAAA
AAAAGCACATGGGTTAAACCCATCTCCCCTTATTAGGGGAATCAGAACTTGTTAGTGGGTTCAT
GACAGAGCATGCTGCCGTAGTTTTCGTATTCTTCTTTTTAGCTGAGTACGGTAGTATTGTACTAAT
GTGTATTTTAACTAGTATATTATTTATTGGTGGTTACTTATTATTTGAAATATCCTATGTTTTTACTG
TGGTAAATTATATTTTCTTTGAATTATTCTTTATAGACTGAGTAACATTTGTAGAGGTACAATCTTT
ATACACTGATTTTTTAAATAATTCTATCATTGAAGGATTATTATATGGGTTTAATCTAGGATTAAA
AAGTTCTTTAATGATATTCACATTTATTTGAGCTAGAGCATCCTTCCCTAGAATACGATTTGATCA
ACTAATGGGCTTCTGTTGAACAGTTTTATTACCTATTAATTTTGCAATTATTATATTAGTACCTTGT
GTTTTATATAGTTTTAACTTATTACCTGTAAATATACCATTGTTCTAGCTCACACACCCGCCCGCC
CTACTGCCACAAGGCTACAGTACATATGAGGAGGGGAACTAAAGATCTAGAACTATCCTAGTTA
ATAATTACACTTAATAGTATACTAAATAGACCATCTATCATACTCGAGAATAGTGATAGTGTAAT
TATACACTATTTTAACTGTATTCTACACTATTAGCATATTATTATCTTTATATGATAATAATTTTATA
ACTTAATTTATTAGGTTACATTAACAAAAAATTCGTTAACTTTTTTCACCACTTTTTAATACAAAAT
ACGAATTTTA
>YN268
ATAACAATTCTAAAGAACATAAAGAGTTATTAGATAAAAATAATTCACCTATACAGTTAATAAAT
CAACTTAAAGGGTATTTTTTCATAAATCCTTTATTAGCTTTAAGTTTAGCTATTACTATTTTCTCTTT
TGCAGGTATTCCTCCTCTTGTAGGGTTCTTTGCTAAACAGATGGTATTAAGCGCGGCTATTGATC
AAGGTTATATCTTTTTATCTTTAGTTGCAATATTAACTAGTGTTATAGGAGGGGTTTATTATTTAAA
TATAATTAAAGAAATGTTCTTTTATTCACCTGACTATAAATTAAACGAAGAAATTAAAAATAATAC
TATTAATGGTCAAATTATTAATAGAAACAATAAAATATTAAATGTTGAATTTAATTATACAAATGT
AGTTATGTCTAGTTCTGTGGCAATAACTATTTCTACTATTACATTAGTAGTTTTATTATTCATGTTT
ATGAATAAAGAATGATTAAGTCTGGGTACTATATTGGTACAATCTTTATTTAGCTATTAATGAGTA
GTATGACATTATTTATAGGGTTTGTATCTGTTATAGCTATTTTATTTTTAGCCATTAATTTTATATTT
GCTCCTCATAATCCTTATCAAGAAAAATATAGTATTTTCGAGTGTGGTTTCCATAGTTTTTTAGGG
CAAAATAGAACACAATTCGGTATAAAATTCTTTATTTTTGCTTTAGTTTATTTACTTTTAGATTTAG

AAATATTATTAACTTTCCCTTTCGCTCTTAGTGAGTATGTTAATGGTATTTATGGTCTTTTAGTTAC
TTTAATTTTTATAGCTATAATAACTATAGGATTTATATTTGAATTAGGTAAAAGCGCTCTTAAAATA
GACAGCAGACAAAAATTATATATACCTAAATTGAACGTTAATTACCATACAGAGTATGTTGGAAT
AGGTAAGGTTTCTAAGTAAAGTTATAGAGGCAGAAAACCAAAAAACCTACCAAAGGGTAGCTA
ATGGGAAGCTATTAAAAATAAAAGATGATAACCTATATATAGTATAGTTACTATATATTTACTATA
ACTAGGATTATTATATATATATATATTATCTTATTGTATATTAAGATTATTATTATTATAAGGTATA
ATTAATATAGTATCTTATTGTATAAGAATATAATATATTAACCTATAATTAATTTTATTTTTTAATTT
TTAATTATAATTTTTTTTTATATCTAGATGCTTACACATCTACAGATGTAGAAGAGAACAAAATAT
TGTTGTTATAGTAGTGGATGATATAGAAAAATATTTTTATATTATTTATTTTTGTTAGGTAGCTTTT
GAAGTGTTTGATAGAGAGGATATATGGACGGTAGGAGGGTATTCATTTTAATGAACAGTGGATA
GTTTAAATTAACCTAGTTATAGTTTATGAATTTAAATTATAATTATATTAATGTAGGTTATGATAGA
ATTATGTATTGATCCTTAAGAGTTAAGAGAGATACGCCCACGTATAATACATATTGGTTTAGGGT
TGAGTATATATACTTAAGAGTTAAGAATATATATATACAATATATAATAAATATAGTAATATATTA
ATGGTATGGACTTAACCAGGTTTATATATTATCATTTGATAAACATTAATTATAATTTTTCTATTTA
TTATTATTATTATACTGTGAGATTAATAATTATTAAAATATAATATTACATAGTACGTGATATTTGG
GGATTTTATCTTATTAATTGGCAATTAATGATTCTAATCAAATTTTATTCTCTTTAGTTTAATGGTA
GAACAATGATCTTCTAATTCATTGGTTTTAGTTCGATTCTAAAAAGAGATGAGTAAATAATTTTCT
AGATCAGAAATACTACTTTTAACTACAAAAAGCTTACGCTTTTTAACATTTTTTTGATAAATAACA
ACTATTGTTAATATTTGGCTGTCTATTGGTTTAACTAACAATTACAAAATTTTTTCAATTTTTATAAT
ATAAATTTAAATGAGAATATTAAAAAGTCATTCATTATTAAAATTAGTGAATTCTTACCTTATCGA
TGCGTCACAACCAAGTAACATTAGTTACTTGTGAAATTTTGGTTCATTATTAGCTGTTTGTTTAAT
AGTACAAATTATTACCGGTATTACATTAGCTATGCATTATAGTCCTAGTGTAATGGAAGCTTTTAA
CTCAATAGAGCATATAATGAGAGATGTTAATAACGGGTGATTAGTTCGTTATCTACATAGTAATA
CAGCTTCTGCTTTCTTTTTCTTAGTGTATTTACACATAGGAAGAGGTATATATTACGGATCATATA
GAGCTCCTCGTACTTTAGTTTGAGCTATTGGTACTGTTATATTAATATTAATGATGGCTATCGGTT
TCCTAGGTTATGTTTTACCTTATGGACAGATGTCATTATGAGGTGCTACAGTTATTACTAATCTTA
TTAGTGCTATACCTTGAATAGGGCAAGATATTGTTGAATTCATTTGAGGTGGTTTTTCTGTTAATA
ATGCCACTTTAAACAGATTTTTTGCATTACATTTTGTATTGCCTTTTGTATTAGCTGCTTTAGTTTTA
ATGCACTTAATTGCACTTCATGATACTGCTGGTTCAAGCAATCCTCTTGGTGTTTCAGGTAATTAC
GATAGAATTACATTTGCTCCATATTTTTTATTTAAAGATTTAATTACTATTTTTATATTTATTTTTGTA
TTAAGTGCTTTTGTATTCTTTATGCCTAATGTTTTAGGGGATAGTGATAATTATATTATGGCTAATC
CTATGCAAACTCCTGCTGCTATTGTACCTGAATGATACTTATTACCTTTCTATGCTATTTTAAGATC
TATACCTAATAAATTATTAGGTGTTATAGCGATGTTTAGTGCTATTTTAGCTATTATGTTATTACCT
GTTACAGATTTAGGTAGATCTAGAGGTTTACAATTTAGACCATTTAGTAAAATAGCTTTCTGAGTT
TTTGTTGCTAATTTCTTAGTTTTAATGCAATTAGGTGCTAAACACGTTGAAGATCCATTTATATTAT
TAGGTCAATTAAGTACTGTATTATACTTTAGTTATTTTGTTGCTATATTACCTTTAGCTAGTTACTT
AGATAATAGTTTAACTGATTTATCTAATAAATCTGAATTATTTTTAAATAAAACTAACTAAATATAT
TAAGATTATTATTTAATATATTTTCTATTTAAGATACTATTAATTTAGTATTTTGGGTTTTCAGTTTA
TAATTTATATTATATTATGCATTACCCTCCACCTTGCTTTGTAGTAAGCTAATCTGTTATTTCCTTTA
GTTTAATGGTAGAACAATGATCTTCTAATTCATTGGTTTTAGTTCGAATCTAAAAAGGAAATAAG
AAATATATTCTTATTATTACTTATATAATAATTATTTCTTAAAAATATACATTTTGCATTATAGCCGT
TTAGCTGTATTAAAATGTAAAATGATATAAAATAGAATAAATATTTAAATTATTCCTATGTTATATT
ATCCTATATTGCAACCATTATCAGAAGTTGTATTAATACTTGTACCTGCCTTATTAGCTGTAGCTT
ATGTTACAGTTGCTGAAAGAAAAACTATGGCTAGTATGCAAAGAAGATTAGGTCCTAATGCTGT

AGGTTACTATGGACTATTGCAAGCATTTGCTGATGCCTTAAAACTTTTATTAAAAGAATATGTAG
CTCCTACACAATCTAATATTGTTCTTTTCTTTTTAGGTCCTGTAATAACTTTAATTTTTGCATTATTA
GGTTACGCTGTTATACCCTATGGTCCTGGTTCAGGGATAAGCGACATGAATTTAGGTATATTTTA
CATGTTAGCTGTGTCATCTTTAGCTACATACGGTATTCTATTAGCTGGTTGAAGTGCGAATAGTA
AATACGCTTTTCTAGGTTCTCTTAGAAGTACAGCTCAATTAATTAGTTATGAATTAATATTAAGTT
CAGCTATATTAATAGTAATTATGATAACAGGAAATTTAAATTTAACTGTTTGTACTGAATCTCAAA
GAGCTATTTGATTTATACTACCTTTATTTCCTGTGTTTATAATATTTTTCATAGGATCTATAGCTGA
GACAAATAGAGCTCCTTTTGATTTAGCCGAGGCTAACCTGCTAATCTGGTTTGGTCTGGTTATAT
GTCACAAATTGCTAGGAAACCTTTTTATTTTAAAAACAAAAGACAATTAGCAGGAAACTTAATTT
AACCTAATTAAAAATATTAGATAATTAAACTCTTCATAGACTAAACGTGACAATTTAATATATATA
TATATTTATTTATATATATGATTAAATAAGATATAGTCAATCATCGGTGTGAATCGACTTAAAAAA
AAAAGCACATGGGTTAAACCCATCTCCCCTTATTAGGGGAATCAGAACTTGTTAGTGGGTTCAT
GACAGAGCATGCTGCCGTAGTTTTCGTATTCTTCTTTTTAGCTGAGTACGGTAGTATTGTACTAAT
GTGTATTTTAACTAGTATATTATTTATTGGTGGTTACTTATTATTTGAAATATCCTATGTTTTTACTG
TGGTAAATTATATTTTCTTTGAATTATTCTTTATAGACTGAGTAACATTTGTAGAGGTACAATCTTT
ATACACTGATTTTTTAAATAATTCTATCATTGAAGGATTATTATATGGGTTTAATCTAGGATTAAA
AAGTTCTTTAATGATATTCACATTTATTTGAGCTAGAGCATCCTTCCCTAGAATACGATTTGATCA
ACTAATGGGCTTCTGTTGAACAGTTTTATTACCTATTAATTTTGCAATTATTATATTAGTACCTTGT
GTTTTATATAGTTTTAACTTATTACCTGTAAATATACCATTGTTCTAGCTCACACACCCGCCCGCC
CTACTGCCACAAGGCTACAGTACATATGAGGAGGGGAACTAAAGATCTAGAACTATCCTAGTTA
ATAATTACACTTAATAGTATACTAAATAGACCATCTATCATACTCGAGAATAGTGATAGTGTAAT
TATACACTATTTTAACTGTATTCTACACTATTAGCATATTATTATCTTTATATGATAATAATTTTATA
ACTTAATTTATTAGGTTACATTAACAAAAAATTCGTTAACTTTTTTCACCACTTTTTAATACAAAAT
ACGAATTTTA
>YN271
ATAACAATTCTAAAGAACATAAAGAGTTATTAGATAAAAATAATTCACCTATACAGTTAATAAAT
CAACTTAAAGGGTATTTTTTCATAAATCCTTTATTAGCTTTAAGTTTAGCTATTACTATTTTCTCTTT
TGCAGGTATTCCTCCTCTTGTAGGGTTCTTTGCTAAACAGATGGTATTAAGCGCGGCTATTGATC
AAGGTTATATCTTTTTATCTTTAGTTGCAATATTAACTAGTGTTATAGGAGGGGTTTATTATTTAAA
TATAATTAAAGAAATGTTCTTTTATTCACCTGACTATAAATTAAACGAAGAAATTAAAAATAATAC
TATTAATGGTCAAATTATTAATAGAAACAATAAAATATTAAATGTTGAATTTAATTATACAAATGT
AGTTATGTCTAGTTCTGTGGCAATAACTATTTCTACTATTACATTAGTAGTTTTATTATTCATGTTT
ATGAATAAAGAATGATTAAGTCTGGGTACTATATTGGTACAATCTTTATTTAGCTATTAATGAGTA
GTATGACATTATTTATAGGGTTTGTATCTGTTATAGCTATTTTATTTTTAGCCATTAATTTTATATTT
GCTCCTCATAATCCTTATCAAGAAAAATATAGTATTTTCGAGTGTGGTTTCCATAGTTTTTTAGGG
CAAAATAGAACACAATTCGGTATAAAATTCTTTATTTTTGCTTTAGTTTATTTACTTTTAGATTTAG
AAATATTATTAACTTTCCCTTTCGCTCTTAGTGAGTATGTTAATGGTATTTATGGTCTTTTAGTTAC
TTTAATTTTTATAGCTATAATAACTATAGGATTTATATTTGAATTAGGTAAAAGCGCTCTTAAAATA
GACAGCAGACAAAAATTATATATACCTAAATTGAACGTTAATTACCATACAGAGTATGTTGGAAT
AGGTAAGGTTTCTAAGTAAAGTTATAGAGGCAGAAAACCAAAAAACCTACCAAAGGGTAGCTA
ATGGGAAGCTATTAAAAATAAAAGATGATAACCTATATATAGTATAGTTACTATATATTTACTATA
ACTAGGATTATTATATATATATATATTATCTTATTGTATATTAAGATTATTATTATTATAAGGTATA
ATTAATATAGTATCTTATTGTATAAGAATATAATATATTAACCTATAATTAATTTTATTTTTTAATTT
TTAATTATAATTTTTTTTTATATCTAGATGCTTACACATCTACAGATGTAGAAGAGAACAAAATAT

TGTTGTTATAGTAGTGGATGATATAGAAAAATATTTTTATATTATTTATTTTTGTTAGGTAGCTTTT
GAAGTGTTTGATAGAGAGGATATATGGACGGTAGGAGGGTATTCATTTTAATGAACAGTGGATA
GTTTAAATTAACCTAGTTATAGTTTATGAATTTAAATTATAATTATATTAATGTAGGTTATGATAGA
ATTATGTATTGATCCTTAAGAGTTAAGAGAGATACGCCCACGTATAATACATATTGGTTTAGGGT
TGAGTATATATACTTAAGAGTTAAGAATATATATATACAATATATAATAAATATAGTAATATATTA
ATGGTATGGACTTAACCAGGTTTATATATTATCATTTGATAAACATTAATTATAATTTTTCTATTTA
TTATTATTATTATACTGTGAGATTAATAATTATTAAAATATAATATTACATAGTACGTGATATTTGG
GGATTTTATCTTATTAATTGGCAATTAATGATTCTAATCAAATTTTATTCTCTTTAGTTTAATGGTA
GAACAATGATCTTCTAATTCATTGGTTTTAGTTCGATTCTAAAAAGAGATGAGTAAATAATTTTCT
AGATCAGAAATACTACTTTTAACTACAAAAAGCTTACGCTTTTTAACATTTTTTTGATAAATAACA
ACTATTGTTAATATTTGGCTGTCTATTGGTTTAACTAACAATTACAAAATTTTTTCAATTTTTATAAT
ATAAATTTAAATGAGAATATTAAAAAGTCATTCATTATTAAAATTAGTGAATTCTTACCTTATCGA
TGCGTCACAACCAAGTAACATTAGTTACTTGTGAAATTTTGGTTCATTATTAGCTGTTTGTTTAAT
AGTACAAATTATTACCGGTATTACATTAGCTATGCATTATAGTCCTAGTGTAATGGAAGCTTTTAA
CTCAATAGAGCATATAATGAGAGATGTTAATAACGGGTGATTAGTTCGTTATCTACATAGTAATA
CAGCTTCTGCTTTCTTTTTCTTAGTGTATTTACACATAGGAAGAGGTATATATTACGGATCATATA
GAGCTCCTCGTACTTTAGTTTGAGCTATTGGTACTGTTATATTAATATTAATGATGGCTATCGGTT
TCCTAGGTTATGTTTTACCTTATGGACAGATGTCATTATGAGGTGCTACAGTTATTACTAATCTTA
TTAGTGCTATACCTTGAATAGGGCAAGATATTGTTGAATTCATTTGAGGTGGTTTTTCTGTTAATA
ATGCCACTTTAAACAGATTTTTTGCATTACATTTTGTATTGCCTTTTGTATTAGCTGCTTTAGTTTTA
ATGCACTTAATTGCACTTCATGATACTGCTGGTTCAAGCAATCCTCTTGGTGTTTCAGGTAATTAC
GATAGAATTACATTTGCTCCATATTTTTTATTTAAAGATTTAATTACTATTTTTATATTTATTTTTGTA
TTAAGTGCTTTTGTATTCTTTATGCCTAATGTTTTAGGGGATAGTGATAATTATATTATGGCTAATC
CTATGCAAACTCCTGCTGCTATTGTACCTGAATGATACTTATTACCTTTCTATGCTATTTTAAGATC
TATACCTAATAAATTATTAGGTGTTATAGCGATGTTTAGTGCTATTTTAGCTATTATGTTATTACCT
GTTACAGATTTAGGTAGATCTAGAGGTTTACAATTTAGACCATTTAGTAAAATAGCTTTCTGAGTT
TTTGTTGCTAATTTCTTAGTTTTAATGCAATTAGGTGCTAAACACGTTGAAGATCCATTTATATTAT
TAGGTCAATTAAGTACTGTATTATACTTTAGTTATTTTGTTGCTATATTACCTTTAGCTAGTTACTT
AGATAATAGTTTAACTGATTTATCTAATAAATCTGAATTATTTTTAAATAAAACTAACTAAATATAT
TAAGATTATTATTTAATATATTTTCTATTTAAGATACTATTAATTTAGTATTTTGGGTTTTCAGTTTA
TAATTTATATTATATTATGCATTACCCTCCACCTTGCTTTGTAGTAAGCTAATCTGTTATTTCCTTTA
GTTTAATGGTAGAACAATGATCTTCTAATTCATTGGTTTTAGTTCGAATCTAAAAAGGAAATAAG
AAATATATTCTTATTATTACTTATATAATAATTATTTCTTAAAAATATACATTTTGCATTATAGCCGT
TTAGCTGTATTAAAATGTAAAATGATATAAAATAGAATAAATATTTAAATTATTCCTATGTTATATT
ATCCTATATTGCAACCATTATCAGAAGTTGTATTAATACTTGTACCTGCCTTATTAGCTGTAGCTT
ATGTTACAGTTGCTGAAAGAAAAACTATGGCTAGTATGCAAAGAAGATTAGGTCCTAATGCTGT
AGGTTACTATGGACTATTGCAAGCATTTGCTGATGCCTTAAAACTTTTATTAAAAGAATATGTAG
CTCCTACACAATCTAATATTGTTCTTTTCTTTTTAGGTCCTGTAATAACTTTAATTTTTGCATTATTA
GGTTACGCTGTTATACCCTATGGTCCTGGTTCAGGGATAAGCGACATGAATTTAGGTATATTTTA
CATGTTAGCTGTGTCATCTTTAGCTACATACGGTATTCTATTAGCTGGTTGAAGTGCGAATAGTA
AATACGCTTTTCTAGGTTCTCTTAGAAGTACAGCTCAATTAATTAGTTATGAATTAATATTAAGTT
CAGCTATATTAATAGTAATTATGATAACAGGAAATTTAAATTTAACTGTTTGTACTGAATCTCAAA
GAGCTATTTGATTTATACTACCTTTATTTCCTGTGTTTATAATATTTTTCATAGGATCTATAGCTGA
GACAAATAGAGCTCCTTTTGATTTAGCCGAGGCTAACCTGCTAATCTGGTTTGGTCTGGTTATAT

GTCACAAATTGCTAGGAAACCTTTTTATTTTAAAAACAAAAGACAATTAGCAGGAAACTTAATTT
AACCTAATTAAAAATATTAGATAATTAAACTCTTCATAGACTAAACGTGACAATTTAATATATATA
TATATTTATTTATATATATGATTAAATAAGATATAGTCAATCATCGGTGTGAATCGACTTAAAAAA
AAAAGCACATGGGTTAAACCCATCTCCCCTTATTAGGGGAATCAGAACTTGTTAGTGGGTTCAT
GACAGAGCATGCTGCCGTAGTTTTCGTATTCTTCTTTTTAGCTGAGTACGGTAGTATTGTACTAAT
GTGTATTTTAACTAGTATATTATTTATTGGTGGTTACTTATTATTTGAAATATCCTATGTTTTTACTG
TGGTAAATTATATTTTCTTTGAATTATTCTTTATAGACTGAGTAACATTTGTAGAGGTACAATCTTT
ATACACTGATTTTTTAAATAATTCTATCATTGAAGGATTATTATATGGGTTTAATCTAGGATTAAA
AAGTTCTTTAATGATATTCACATTTATTTGAGCTAGAGCATCCTTCCCTAGAATACGATTTGATCA
ACTAATGGGCTTCTGTTGAACAGTTTTATTACCTATTAATTTTGCAATTATTATATTAGTACCTTGT
GTTTTATATAGTTTTAACTTATTACCTGTAAATATACCATTGTTCTAGCTCACACACCCGCCCGCC
CTACTGCCACAAGGCTACAGTACATATGAGGAGGGGAACTAAAGATCTAGAACTATCCTAGTTA
ATAATTACACTTAATAGTATACTAAATAGACCATCTATCATACTCGAGAATAGTGATAGTGTAAT
TATACACTATTTTAACTGTATTCTACACTATTAGCATATTATTATCTTTATATGATAATAATTTTATA
ACTTAATTTATTAGGTTACATTAACAAAAAAATTCGTTAACTTTTTTCACCACTTTTTAATACAAAAT
ACGAATTTTA
>YN283
ATAACAATTCTAAAGAACATAAAGAGTTATTAGATAAAAATAATTCACCTATACAGTTAATAAAT
CAACTTAAAGGGTATTTTTTCATAAATCCTTTATTAGCTTTAAGTTTAGCTATTACTATTTTCTCTTT
TGCAGGTATTCCTCCTCTTGTAGGGTTCTTTGCTAAACAGATGGTATTAAGCGCGGCTATTGATC
AAGGTTATATCTTTTTATCTTTAGTTGCAATATTAACTAGTGTTATAGGAGGGGTTTATTATTTAAA
TATAATTAAAGAAATGTTCTTTTATTCACCTGACTATAAATTAAACGAAGAAATTAAAAATAATAC
TATTAATGGTCAAATTATTAATAGAAACAATAAAATATTAAATGTTGAATTTAATTATACAAATGT
AGTTATGTCTAGTTCTGTGGCAATAACTATTTCTACTATTACATTAGTAGTTTTATTATTCATGTTT
ATGAATAAAGAATGATTAAGTCTGGGTACTATATTGGTACAATCTTTATTTAGCTATTAATGAGTA
GTATGACATTATTTATAGGGTTTGTATCTGTTATAGCTATTTTATTTTTAGCCATTAATTTTATATTT
GCTCCTCATAATCCTTATCAAGAAAAATATAGTATTTTCGAGTGTGGTTTCCATAGTTTTTTAGGG
CAAAATAGAACACAATTCGGTATAAAATTCTTTATTTTTGCTTTAGTTTATTTACTTTTAGATTTAG
AAATATTATTAACTTTCCCTTTCGCTCTTAGTGAGTATGTTAATGGTATTTATGGTCTTTTAGTTAC
TTTAATTTTTATAGCTATAATAACTATAGGATTTATATTTGAATTAGGTAAAAGCGCTCTTAAAATA
GACAGCAGACAAAAATTATATATACCTAAATTGAACGTTAATTACCATACAGAGTATGTTGGAAT
AGGTAAGGTTTCTAAGTAAAGTTATAGAGGCAGAAAACCAAAAAACCTACCAAAGGGTAGCTA
ATGGGAAGCTATTAAAAATAAAAGATGATAACCTATATATAGTATAGTTACTATATATTTACTATA
ACTAGGATTATTATATATATATATATTATCTTATTGTATATTAAGATTATTATTATTATAAGGTATA
ATTAATATAGTATCTTATTGTATAAGAATATAATATATTAACCTATAATTAATTTTATTTTTTAATTT
TTAATTATAATTTTTTTTTATATCTAGATGCTTACACATCTACAGATGTAGAAGAGAACAAAATAT
TGTTGTTATAGTAGTGGATGATATAGAAAAATATTTTTATATTATTTATTTTTGTTAGGTAGCTTTT
GAAGTGTTTGATAGAGAGGATATATGGACGGTAGGAGGGTATTCATTTTAATGAACAGTGGATA
GTTTAAATTAACCTAGTTATAGTTTATGAATTTAAATTATAATTATATTAATGTAGGTTATGATAGA
ATTATGTATTGATCCTTAAGAGTTAAGAGAGATACGCCCACGTATAATACATATTGGTTTAGGGT
TGAGTATATATACTTAAGAGTTAAGAATATATATATACAATATATAATAAATATAGTAATATATTA
ATGGTATGGACTTAACCAGGTTTATATATTATCATTTGATAAACATTAATTATAATTTTTCTATTTA
TTATTATTATTATACTGTGAGATTAATAATTATTAAAATATAATATTACATAGTACGTGATATTTGG
GGATTTTATCTTATTAATTGGCAATTAATGATTCTAATCAAATTTTATTCTCTTTAGTTTAATGGTA

GAACAATGATCTTCTAATTCATTGGTTTTAGTTCGATTCTAAAAAGAGATGAGTAAATAATTTTCT
AGATCAGAAATACTACTTTTAACTACAAAAAGCTTACGCTTTTTAACATTTTTTTGATAAATAACA
ACTATTGTTAATATTTGGCTGTCTATTGGTTTAACTAACAATTACAAAATTTTTTCAATTTTTATAAT
ATAAATTTAAATGAGAATATTAAAAAGTCATTCATTATTAAAATTAGTGAATTCTTACCTTATCGA
TGCGTCACAACCAAGTAACATTAGTTACTTGTGAAATTTTGGTTCATTATTAGCTGTTTGTTTAAT
AGTACAAATTATTACCGGTATTACATTAGCTATGCATTATAGTCCTAGTGTAATGGAAGCTTTTAA
CTCAATAGAGCATATAATGAGAGATGTTAATAACGGGTGATTAGTTCGTTATCTACATAGTAATA
CAGCTTCTGCTTTCTTTTTCTTAGTGTATTTACACATAGGAAGAGGTATATATTACGGATCATATA
GAGCTCCTCGTACTTTAGTTTGAGCTATTGGTACTGTTATATTAATATTAATGATGGCTATCGGTT
TCCTAGGTTATGTTTTACCTTATGGACAGATGTCATTATGAGGTGCTACAGTTATTACTAATCTTA
TTAGTGCTATACCTTGAATAGGGCAAGATATTGTTGAATTCATTTGAGGTGGTTTTTCTGTTAATA
ATGCCACTTTAAACAGATTTTTTGCATTACATTTTGTATTGCCTTTTGTATTAGCTGCTTTAGTTTTA
ATGCACTTAATTGCACTTCATGATACTGCTGGTTCAAGCAATCCTCTTGGTGTTTCAGGTAATTAC
GATAGAATTACATTTGCTCCATATTTTTTATTTAAAGATTTAATTACTATTTTTATATTTATTTTTGTA
TTAAGTGCTTTTGTATTCTTTATGCCTAATGTTTTAGGGGATAGTGATAATTATATTATGGCTAATC
CTATGCAAACTCCTGCTGCTATTGTACCTGAATGATACTTATTACCTTTCTATGCTATTTTAAGATC
TATACCTAATAAATTATTAGGTGTTATAGCGATGTTTAGTGCTATTTTAGCTATTATGTTATTACCT
GTTACAGATTTAGGTAGATCTAGAGGTTTACAATTTAGACCATTTAGTAAAATAGCTTTCTGAGTT
TTTGTTGCTAATTTCTTAGTTTTAATGCAATTAGGTGCTAAACACGTTGAAGATCCATTTATATTAT
TAGGTCAATTAAGTACTGTATTATACTTTAGTTATTTTGTTGCTATATTACCTTTAGCTAGTTACTT
AGATAATAGTTTAACTGATTTATCTAATAAATCTGAATTATTTTTAAATAAAACTAACTAAATATAT
TAAGATTATTATTTAATATATTTTCTATTTAAGATACTATTAATTTAGTATTTTGGGTTTTCAGTTTA
TAATTTATATTATATTATGCATTACCCTCCACCTTGCTTTGTAGTAAGCTAATCTGTTATTTCCTTTA
GTTTAATGGTAGAACAATGATCTTCTAATTCATTGGTTTTAGTTCGAATCTAAAAAGGAAATAAG
AAATATATTCTTATTATTACTTATATAATAATTATTTCTTAAAAATATACATTTTGCATTATAGCCGT
TTAGCTGTATTAAAATGTAAAATGATATAAAATAGAATAAATATTTAAATTATTCCTATGTTATATT
ATCCTATATTGCAACCATTATCAGAAGTTGTATTAATACTTGTACCTGCCTTATTAGCTGTAGCTT
ATGTTACAGTTGCTGAAAGAAAAACTATGGCTAGTATGCAAAGAAGATTAGGTCCTAATGCTGT
AGGTTACTATGGACTATTGCAAGCATTTGCTGATGCCTTAAAACTTTTATTAAAAGAATATGTAG
CTCCTACACAATCTAATATTGTTCTTTTCTTTTTAGGTCCTGTAATAACTTTAATTTTTGCATTATTA
GGTTACGCTGTTATACCCTATGGTCCTGGTTCAGGGATAAGCGACATGAATTTAGGTATATTTTA
CATGTTAGCTGTGTCATCTTTAGCTACATACGGTATTCTATTAGCTGGTTGAAGTGCGAATAGTA
AATACGCTTTTCTAGGTTCTCTTAGAAGTACAGCTCAATTAATTAGTTATGAATTAATATTAAGTT
CAGCTATATTAATAGTAATTATGATAACAGGAAATTTAAATTTAACTGTTTGTACTGAATCTCAAA
GAGCTATTTGATTTATACTACCTTTATTTCCTGTGTTTATAATATTTTTCATAGGATCTATAGCTGA
GACAAATAGAGCTCCTTTTGATTTAGCCGAGGCTAACCTGCTAATCTGGTTTGGTCTGGTTATAT
GTCACAAATTGCTAGGAAACCTTTTTATTTTAAAAACAAAAGACAATTAGCAGGAAACTTAATTT
AACCTAATTAAAAATATTAGATAATTAAACTCTTCATAGACTAAACGTGACAATTTAATATATATA
TATATTTATTTATATATATGATTAAATAAGATATAGTCAATCATCGGTGTGAATCGACTTAAAAAA
AAAAGCACATGGGTTAAACCCATCTCCCCTTATTAGGGGAATCAGAACTTGTTAGTGGGTTCAT
GACAGAGCATGCTGCCGTAGTTTTCGTATTCTTCTTTTTAGCTGAGTACGGTAGTATTGTACTAAT
GTGTATTTTAACTAGTATATTATTTATTGGTGGTTACTTATTATTTGAAATATCCTATGTTTTTACTG
TGGTAAATTATATTTTCTTTGAATTATTCTTTATAGACTGAGTAACATTTGTAGAGGTACAATCTTT
ATACACTGATTTTTTAAATAATTCTATCATTGAAGGATTATTATATGGGTTTAATCTAGGATTAAA

AAGTTCTTTAATGATATTCACATTTATTTGAGCTAGAGCATCCTTCCCTAGAATACGATTTGATCA
ACTAATGGGCTTCTGTTGAACAGTTTTATTACCTATTAATTTTGCAATTATTATATTAGTACCTTGT
GTTTTATATAGTTTTAACTTATTACCTGTAAATATACCATTGTTCTAGCTCACACACCCGCCCGCC
CTACTGCCACAAGGCTACAGTACATATGAGGAGGGGAACTAAAGATCTAGAACTATCCTAGTTA
ATAATTACACTTAATAGTATACTAAATAGACCATCTATCATACTCGAGAATAGTGATAGTGTAAT
TATACACTATTTTAACTGTATTCTACACTATTAGCATATTATTATCTTTATATGATAATAATTTTATA
ACTTAATTTATTAGGTTACATTAACAAAAAAATTCGTTAACTTTTTTCACCACTTTTTAATACAAAAT
ACGAATTTTA
>YN311
ATAACAATTCTAAAGAACATAAAGAGTTATTAGATAAAAATAATTCACCTATACAGTTAATAAAT
CAACTTAAAGGGTATTTTTTCATAAATCCTTTATTAGCTTTAAGTTTAGCTATTACTATTTTCTCTTT
TGCAGGTATTCCTCCTCTTGTAGGGTTCTTTGCTAAACAGATGGTATTAAGCGCGGCTATTGATC
AAGGTTATATCTTTTTATCTTTAGTTGCAATATTAACTAGTGTTATAGGAGGGGTTTATTATTTAAA
TATAATTAAAGAAATGTTCTTTTATTCACCTGACTATAAATTAAACGAAGAAATTAAAAATAATAC
TATTAATGGTCAAATTATTAATAGAAACAATAAAATATTAAATGTTGAATTTAATTATACAAATGT
AGTTATGTCTAGTTCTGTGGCAATAACTATTTCTACTATTACATTAGTAGTTTTATTATTCATGTTT
ATGAATAAAGAATGATTAAGTCTGGGTACTATATTGGTACAATCTTTATTTAGCTATTAATGAGTA
GTATGACATTATTTATAGGGTTTGTATCTGTTATAGCTATTTTATTTTTAGCCATTAATTTTATATTT
GCTCCTCATAATCCTTATCAAGAAAAATATAGTATTTTCGAGTGTGGTTTCCATAGTTTTTTAGGG
CAAAATAGAACACAATTCGGTATAAAATTCTTTATTTTTGCTTTAGTTTATTTACTTTTAGATTTAG
AAATATTATTAACTTTCCCTTTCGCTCTTAGTGAGTATGTTAATGGTATTTATGGTCTTTTAGTTAC
TTTAATTTTTATAGCTATAATAACTATAGGATTTATATTTGAATTAGGTAAAAGCGCTCTTAAAATA
GACAGCAGACAAAAATTATATATACCTAAATTGAACGTTAATTACCATACAGAGTATGTTGGAAT
AGGTAAGGTTTCTAAGTAAAGTTATAGAGGCAGAAAACCAAAAAACCTACCAAAGGGTAGCTA
ATGGGAAGCTATTAAAAATAAAAGATGATAACCTATATATAGTATAGTTACTATATATTTACTATA
ACTAGGATTATTATATATATATATATTATCTTATTGTATATTAAGATTATTATTATTATAAGGTATA
ATTAATATAGTATCTTATTGTATAAGAATATAATATATTAACCTATAATTAATTTTATTTTTTAATTT
TTAATTATAATTTTTTTTTATATCTAGATGCTTACACATCTACAGATGTAGAAGAGAACAAAATAT
TGTTGTTATAGTAGTGGATGATATAGAAAAATATTTTTATATTATTTATTTTTGTTAGGTAGCTTTT
GAAGTGTTTGATAGAGAGGATATATGGACGGTAGGAGGGTATTCATTTTAATGAACAGTGGATA
GTTTAAATTAACCTAGTTATAGTTTATGAATTTAAATTATAATTATATTAATGTAGGTTATGATAGA
ATTATGTATTGATCCTTAAGAGTTAAGAGAGATACGCCCACGTATAATACATATTGGTTTAGGGT
TGAGTATATATACTTAAGAGTTAAGAATATATATATACAATATATAATAAATATAGTAATATATTA
ATGGTATGGACTTAACCAGGTTTATATATTATCATTTGATAAACATTAATTATAATTTTTCTATTTA
TTATTATTATTATACTGTGAGATTAATAATTATTAAAATATAATATTACATAGTACGTGATATTTGG
GGATTTTATCTTATTAATTGGCAATTAATGATTCTAATCAAATTTTATTCTCTTTAGTTTAATGGTA
GAACAATGATCTTCTAATTCATTGGTTTTAGTTCGATTCTAAAAAGAGATGAGTAAATAATTTTCT
AGATCAGAAATACTACTTTTAACTACAAAAAGCTTACGCTTTTTAACATTTTTTTGATAAATAACA
ACTATTGTTAATATTTGGCTGTCTATTGGTTTAACTAACAATTACAAAATTTTTTCAATTTTTATAAT
ATAAATTTAAATGAGAATATTAAAAAGTCATTCATTATTAAAATTAGTGAATTCTTACCTTATCGA
TGCGTCACAACCAAGTAACATTAGTTACTTGTGAAATTTTGGTTCATTATTAGCTGTTTGTTTAAT
AGTACAAATTATTACCGGTATTACATTAGCTATGCATTATAGTCCTAGTGTAATGGAAGCTTTTAA
CTCAATAGAGCATATAATGAGAGATGTTAATAACGGGTGATTAGTTCGTTATCTACATAGTAATA
CAGCTTCTGCTTTCTTTTTCTTAGTGTATTTACACATAGGAAGAGGTATATATTACGGATCATATA

GAGCTCCTCGTACTTTAGTTTGAGCTATTGGTACTGTTATATTAATATTAATGATGGCTATCGGTT
TCCTAGGTTATGTTTTACCTTATGGACAGATGTCATTATGAGGTGCTACAGTTATTACTAATCTTA
TTAGTGCTATACCTTGAATAGGGCAAGATATTGTTGAATTCATTTGAGGTGGTTTTTCTGTTAATA
ATGCCACTTTAAACAGATTTTTTGCATTACATTTTGTATTGCCTTTTGTATTAGCTGCTTTAGTTTTA
ATGCACTTAATTGCACTTCATGATACTGCTGGTTCAAGCAATCCTCTTGGTGTTTCAGGTAATTAC
GATAGAATTACATTTGCTCCATATTTTTTATTTAAAGATTTAATTACTATTTTTATATTTATTTTTGTA
TTAAGTGCTTTTGTATTCTTTATGCCTAATGTTTTAGGGGATAGTGATAATTATATTATGGCTAATC
CTATGCAAACTCCTGCTGCTATTGTACCTGAATGATACTTATTACCTTTCTATGCTATTTTAAGATC
TATACCTAATAAATTATTAGGTGTTATAGCGATGTTTAGTGCTATTTTAGCTATTATGTTATTACCT
GTTACAGATTTAGGTAGATCTAGAGGTTTACAATTTAGACCATTTAGTAAAATAGCTTTCTGAGTT
TTTGTTGCTAATTTCTTAGTTTTAATGCAATTAGGTGCTAAACACGTTGAAGATCCATTTATATTAT
TAGGTCAATTAAGTACTGTATTATACTTTAGTTATTTTGTTGCTATATTACCTTTAGCTAGTTACTT
AGATAATAGTTTAACTGATTTATCTAATAAATCTGAATTATTTTTAAATAAAACTAACTAAATATAT
TAAGATTATTATTTAATATATTTTCTATTTAAGATACTATTAATTTAGTATTTTGGGTTTTCAGTTTA
TAATTTATATTATATTATGCATTACCCTCCACCTTGCTTTGTAGTAAGCTAATCTGTTATTTCCTTTA
GTTTAATGGTAGAACAATGATCTTCTAATTCATTGGTTTAGTTCGAATCTAAAAAGGAAATAAG
AAATATATTCTTATTATTACTTATATAATAATTATTTCTTAAAAATATACATTTTGCATTATAGCCGT
TTAGCTGTATTAAAATGTAAAATGATATAAAATAGAATAAATATTTAAATTATTCCTATGTTATATT
ATCCTATATTGCAACCATTATCAGAAGTTGTATTAATACTTGTACCTGCCTTATTAGCTGTAGCTT
ATGTTACAGTTGCTGAAAGAAAAACTATGGCTAGTATGCAAAGAAGATTAGGTCCTAATGCTGT
AGGTTACTATGGACTATTGCAAGCATTTGCTGATGCCTTAAAACTTTTATTAAAAGAATATGTAG
CTCCTACACAATCTAATATTGTTCTTTTCTTTTTAGGTCCTGTAATAACTTTAATTTTTGCATTATTA
GGTTACGCTGTTATACCCTATGGTCCTGGTTCAGGGATAAGCGACATGAATTTAGGTATATTTTA
CATGTTAGCTGTGTCATCTTTAGCTACATACGGTATTCTATTAGCTGGTTGAAGTGCGAATAGTA
AATACGCTTTTCTAGGTTCTCTTAGAAGTACAGCTCAATTAATTAGTTATGAATTAATATTAAGTT
CAGCTATATTAATAGTAATTATGATAACAGGAAATTTAAATTTAACTGTTTGTACTGAATCTCAAA
GAGCTATTTGATTTATACTACCTTTATTTCCTGTGTTTATAATATTTTTCATAGGATCTATAGCTGA
GACAAATAGAGCTCCTTTTGATTTAGCCGAGGCTAACCTGCTAATCTGGTTTGGTCTGGTTATAT
GTCACAAATTGCTAGGAAACCTTTTTATTTTAAAAACAAAAGACAATTAGCAGGAAACTTAATTT
AACCTAATTAAAAATATTAGATAATTAAACTCTTCATAGACTAAACGTGACAATTTAATATATATA
TATATTTATTTATATATATGATTAAATAAGATATAGTCAATCATCGGTGTGAATCGACTTAAAAAA
AAAAGCACATGGGTTAAACCCATCTCCCCTTATTAGGGGAATCAGAACTTGTTAGTGGGTTCAT
GACAGAGCATGCTGCCGTAGTTTTCGTATTCTTCTTTTTAGCTGAGTACGGTAGTATTGTACTAAT
GTGTATTTTAACTAGTATATTATTTATTGGTGGTTACTTATTATTTGAAATATCCTATGTTTTTACTG
TGGTAAATTATATTTTCTTTGAATTATTCTTTATAGACTGAGTAACATTTGTAGAGGTACAATCTTT
ATACACTGATTTTTTAAATAATTCTATCATTGAAGGATTATTATATGGGTTTAATCTAGGATTAAA
AAGTTCTTTAATGATATTCACATTTATTTGAGCTAGAGCATCCTTCCCTAGAATACGATTTGATCA
ACTAATGGGCTTCTGTTGAACAGTTTTATTACCTATTAATTTTGCAATTATTATATTAGTACCTTGT
GTTTTATATAGTTTTAACTTATTACCTGTAAATATACCATTGTTCTAGCTCACACACCCGCCCGCC
CTACTGCCACAAGGCTACAGTACATATGAGGAGGGGAACTAAAGATCTAGAACTATCCTAGTTA
ATAATTACACTTAATAGTATACTAAATAGACCATCTATCATACTCGAGAATAGTGATAGTGTAAT
TATACACTATTTTAACTGTATTCTACACTATTAGCATATTATTATCTTTATATGATAATAATTTTATA
ACTTAATTTATTAGGTTACATTAACAAAAAATTCGTTAACTTTTTTCACCACTTTTTAATACAAAAT
ACGAATTTTA

>YN314
ATAACAATTCTAAAGAACATAAAGAGTTATTAGATAAAAATAATTCACCTATACAGTTAATAAAT
CAACTTAAAGGGTATTTTTTCATAAATCCTTTATTAGCTTTAAGTTTAGCTATTACTATTTTCTCTTT
TGCAGGTATTCCTCCTCTTGTAGGGTTCTTTGCTAAACAGATGGTATTAAGCGCGGCTATTGATC
AAGGTTATATCTTTTTATCTTTAGTTGCAATATTAACTAGTGTTATAGGAGGGGTTTATTATTTAAA
TATAATTAAAGAAATGTTCTTTTATTCACCTGACTATAAATTAAACGAAGAAATTAAAAATAATAC
TATTAATGGTCAAATTATTAATAGAAACAATAAAATATTAAATGTTGAATTTAATTATACAAATGT
AGTTATGTCTAGTTCTGTGGCAATAACTATTTCTACTATTACATTAGTAGTTTTATTATTCATGTTT
ATGAATAAAGAATGATTAAGTCTGGGTACTATATTGGTACAATCTTTATTTAGCTATTAATGAGTA
GTATGACATTATTTATAGGGTTTGTATCTGTTATAGCTATTTTATTTTTAGCCATTAATTTTATATTT
GCTCCTCATAATCCTTATCAAGAAAAATATAGTATTTTCGAGTGTGGTTTCCATAGTTTTTTAGGG
CAAAATAGAACACAATTCGGTATAAAATTCTTTATTTTTGCTTTAGTTTATTTACTTTTAGATTTAG
AAATATTATTAACTTTCCCTTTCGCTCTTAGTGAGTATGTTAATGGTATTTATGGTCTTTTAGTTAC
TTTAATTTTTATAGCTATAATAACTATAGGATTTATATTTGAATTAGGTAAAAGCGCTCTTAAAATA
GACAGCAGACAAAAATTATATATACCTAAATTGAACGTTAATTACCATACAGAGTATGTTGGAAT
AGGTAAGGTTTCTAAGTAAAGTTATAGAGGCAGAAAACCAAAAAACCTACCAAAGGGTAGCTA
ATGGGAAGCTATTAAAAATAAAAGATGATAACCTATATATAGTATAGTTACTATATATTTACTATA
ACTAGGATTATTATATATATATATATTATCTTATTGTATATTAAGATTATTATTATTATAAGGTATA
ATTAATATAGTATCTTATTGTATAAGAATATAATATATTAACCTATAATTAATTTTATTTTTTAATTT
TTAATTATAATTTTTTTTTATATCTAGATGCTTACACATCTACAGATGTAGAAGAGAACAAAATAT
TGTTGTTATAGTAGTGGATGATATAGAAAAATATTTTTATATTATTTATTTTTGTTAGGTAGCTTTT
GAAGTGTTTGATAGAGAGGATATATGGACGGTAGGAGGGTATTCATTTTAATGAACAGTGGATA
GTTTAAATTAACCTAGTTATAGTTTATGAATTTAAATTATAATTATATTAATGTAGGTTATGATAGA
ATTATGTATTGATCCTTAAGAGTTAAGAGAGATACGCCCACGTATAATACATATTGGTTTAGGGT
TGAGTATATATACTTAAGAGTTAAGAATATATATATACAATATATAATAAATATAGTAATATATTA
ATGGTATGGACTTAACCAGGTTTATATATTATCATTTGATAAACATTAATTATAATTTTTCTATTTA
TTATTATTATTATACTGTGAGATTAATAATTATTAAAATATAATATTACATAGTACGTGATATTTGG
GGATTTTATCTTATTAATTGGCAATTAATGATTCTAATCAAATTTTATTCTCTTTAGTTTAATGGTA
GAACAATGATCTTCTAATTCATTGGTTTTAGTTCGATTCTAAAAAGAGATGAGTAAATAATTTTCT
AGATCAGAAATACTACTTTTAACTACAAAAAGCTTACGCTTTTTAACATTTTTTTGATAAATAACA
ACTATTGTTAATATTTGGCTGTCTATTGGTTTAACTAACAATTACAAAATTTTTTCAATTTTTATAAT
ATAAATTTAAATGAGAATATTAAAAAGTCATTCATTATTAAAATTAGTGAATTCTTACCTTATCGA
TGCGTCACAACCAAGTAACATTAGTTACTTGTGAAATTTTGGTTCATTATTAGCTGTTTGTTTAAT
AGTACAAATTATTACCGGTATTACATTAGCTATGCATTATAGTCCTAGTGTAATGGAAGCTTTTAA
CTCAATAGAGCATATAATGAGAGATGTTAATAACGGGTGATTAGTTCGTTATCTACATAGTAATA
CAGCTTCTGCTTTCTTTTTCTTAGTGTATTTACACATAGGAAGAGGTATATATTACGGATCATATA
GAGCTCCTCGTACTTTAGTTTGAGCTATTGGTACTGTTATATTAATATTAATGATGGCTATCGGTT
TCCTAGGTTATGTTTTACCTTATGGACAGATGTCATTATGAGGTGCTACAGTTATTACTAATCTTA
TTAGTGCTATACCTTGAATAGGGCAAGATATTGTTGAATTCATTTGAGGTGGTTTTTCTGTTAATA
ATGCCACTTTAAACAGATTTTTTGCATTACATTTTGTATTGCCTTTTGTATTAGCTGCTTTAGTTTTA
ATGCACTTAATTGCACTTCATGATACTGCTGGTTCAAGCAATCCTCTTGGTGTTTCAGGTAATTAC
GATAGAATTACATTTGCTCCATATTTTTTATTTAAAGATTTAATTACTATTTTTATATTTATTTTTGTA
TTAAGTGCTTTTGTATTCTTTATGCCTAATGTTTTAGGGGATAGTGATAATTATATTATGGCTAATC
CTATGCAAACTCCTGCTGCTATTGTACCTGAATGATACTTATTACCTTTCTATGCTATTTTAAGATC

TATACCTAATAAATTATTAGGTGTTATAGCGATGTTTAGTGCTATTTTAGCTATTATGTTATTACCT
GTTACAGATTTAGGTAGATCTAGAGGTTTACAATTTAGACCATTTAGTAAAATAGCTTTCTGAGTT
TTTGTTGCTAATTTCTTAGTTTTAATGCAATTAGGTGCTAAACACGTTGAAGATCCATTTATATTAT
TAGGTCAATTAAGTACTGTATTATACTTTAGTTATTTTGTTGCTATATTACCTTTAGCTAGTTACTT
AGATAATAGTTTAACTGATTTATCTAATAAATCTGAATTATTTTTAAATAAAACTAACTAAATATAT
TAAGATTATTATTTAATATATTTTCTATTTAAGATACTATTAATTTAGTATTTTGGGTTTTCAGTTTA
TAATTTATATTATATTATGCATTACCCTCCACCTTGCTTTGTAGTAAGCTAATCTGTTATTTCCTTTA
GTTTAATGGTAGAACAATGATCTTCTAATTCATTGGTTTTAGTTCGAATCTAAAAAGGAAATAAG
AAATATATTCTTATTATTACTTATATAATAATTATTTCTTAAAAATATACATTTTGCATTATAGCCGT
TTAGCTGTATTAAAATGTAAAATGATATAAAATAGAATAAATATTTAAATTATTCCTATGTTATATT
ATCCTATATTGCAACCATTATCAGAAGTTGTATTAATACTTGTACCTGCCTTATTAGCTGTAGCTT
ATGTTACAGTTGCTGAAAGAAAAACTATGGCTAGTATGCAAAGAAGATTAGGTCCTAATGCTGT
AGGTTACTATGGACTATTGCAAGCATTTGCTGATGCCTTAAAACTTTTATTAAAAGAATATGTAG
CTCCTACACAATCTAATATTGTTCTTTTCTTTTTAGGTCCTGTAATAACTTTAATTTTTGCATTATTA
GGTTACGCTGTTATACCCTATGGTCCTGGTTCAGGGATAAGCGACATGAATTTAGGTATATTTTA
CATGTTAGCTGTGTCATCTTTAGCTACATACGGTATTCTATTAGCTGGTTGAAGTGCGAATAGTA
AATACGCTTTTCTAGGTTCTCTTAGAAGTACAGCTCAATTAATTAGTTATGAATTAATATTAAGTT
CAGCTATATTAATAGTAATTATGATAACAGGAAATTTAAATTTAACTGTTTGTACTGAATCTCAAA
GAGCTATTTGATTTATACTACCTTTATTTCCTGTGTTTATAATATTTTTCATAGGATCTATAGCTGA
GACAAATAGAGCTCCTTTTGATTTAGCCGAGGCTAACCTGCTAATCTGGTTTGGTCTGGTTATAT
GTCACAAATTGCTAGGAAACCTTTTTATTTTAAAAACAAAAGACAATTAGCAGGAAACTTAATTT
AACCTAATTAAAAATATTAGATAATTAAACTCTTCATAGACTAAACGTGACAATTTAATATATATA
TATATTTATTTATATATATGATTAAATAAGATATAGTCAATCATCGGTGTGAATCGACTTAAAAAA
AAAAGCACATGGGTTAAACCCATCTCCCCTTATTAGGGGAATCAGAACTTGTTAGTGGGTTCAT
GACAGAGCATGCTGCCGTAGTTTTCGTATTCTTCTTTTTAGCTGAGTACGGTAGTATTGTACTAAT
GTGTATTTTAACTAGTATATTATTTATTGGTGGTTACTTATTATTTGAAATATCCTATGTTTTTACTG
TGGTAAATTATATTTTCTTTGAATTATTCTTTATAGACTGAGTAACATTTGTAGAGGTACAATCTTT
ATACACTGATTTTTTAAATAATTCTATCATTGAAGGATTATTATATGGGTTTAATCTAGGATTAAA
AAGTTCTTTAATGATATTCACATTTATTTGAGCTAGAGCATCCTTCCCTAGAATACGATTTGATCA
ACTAATGGGCTTCTGTTGAACAGTTTTATTACCTATTAATTTTGCAATTATTATATTAGTACCTTGT
GTTTTATATAGTTTTAACTTATTACCTGTAAATATACCATTGTTCTAGCTCACACACCCGCCCGCC
CTACTGCCACAAGGCTACAGTACATATGAGGAGGGGAACTAAAGATCTAGAACTATCCTAGTTA
ATAATTACACTTAATAGTATACTAAATAGACCATCTATCATACTCGAGAATAGTGATAGTGTAAT
TATACACTATTTTAACTGTATTCTACACTATTAGCATATTATTATCTTTATATGATAATAATTTTATA
ACTTAATTTATTAGGTTACATTAACAAAAAATTCGTTAACTTTTTTCACCACTTTTTAATACAAAAT
ACGAATTATA
>YN352
ATAACAATTCTAAAGAACATAAAGAGTTATTAGATAAAAATAATTCACCTATACAGTTAATAAAT
CAACTTAAAGGGTATTTTTTCATAAATCCTTTATTAGCTTTAAGTTTAGCTATTACTATTTTCTCTTT
TGCAGGTATTCCTCCTCTTGTAGGGTTCTTTGCTAAACAGATGGTATTAAGCGCGGCTATTGATC
AAGGTTATATCTTTTTATCTTTAGTTGCAATATTAACTAGTGTTATAGGAGGGGTTTATTATTTAAA
TATAATTAAAGAAATGTTCTTTTATTCACCTGACTATAAATTAAACGAAGAAATTAAAAATAATAC
TATTAATGGTCAAATTATTAATAGAAACAATAAAATATTAAATGTTGAATTTAATTATACAAATGT
AGTTATGTCTAGTTCTGTGGCAATAACTATTTCTACTATTACATTAGTAGTTTTATTATTCATGTTT

ATGAATAAAGAATGATTAAGTCTGGGTACTATATTGGTACAATCTTTATTTAGCTATTAATGAGTA
GTATGACATTATTTATAGGGTTTGTATCTGTTATAGCTATTTTATTTTTAGCCATTAATTTTATATTT
GCTCCTCATAATCCTTATCAAGAAAAATATAGTATTTTCGAGTGTGGTTTCCATAGTTTTTTAGGG
CAAAATAGAACACAATTCGGTATAAAATTCTTTATTTTTGCTTTAGTTTATTTACTTTTAGATTTAG
AAATATTATTAACTTTCCCTTTCGCTCTTAGTGAGTATGTTAATGGTATTTATGGTCTTTTAGTTAC
TTTAATTTTTATAGCTATAATAACTATAGGATTTATATTTGAATTAGGTAAAAGCGCTCTTAAAATA
GACAGCAGACAAAAATTATATATACCTAAATTGAACGTTAATTACCATACAGAGTATGTTGGAAT
AGGTAAGGTTTCTAAGTAAAGTTATAGAGGCAGAAAACCAAAAAACCTACCAAAGGGTAGCTA
ATGGGAAGCTATTAAAAATAAAAGATGATAACCTATATATAGTATAGTTACTATATATTTACTATA
ACTAGGATTATTATATATATATATATATTATCTTATTGTATATTAAGATTATTATTATTATAAGGTATA
ATTAATATAGTATCTTATTGTATAAGAATATAATATATTAACCTATAATTAATTTTATTTTTTAATTT
TTAATTATAATTTTTTTTTATATCTAGATGCTTACACATCTACAGATGTAGAAGAGAACAAAATAT
TGTTGTTATAGTAGTGGATGATATAGAAAAATATTTTTATATTATTTATTTTTGTTAGGTAGCTTTT
GAAGTGTTTGATAGAGAGGATATATGGACGGTAGGAGGGTATTCATTTTAATGAACAGTGGATA
GTTTAAATTAACCTAGTTATAGTTTATGAATTTAAATTATAATTATATTAATGTAGGTTATGATAGA
ATTATGTATTGATCCTTAAGAGTTAAGAGAGATACGCCCACGTATAATACATATTGGTTTAGGGT
TGAGTATATATACTTAAGAGTTAAGAATATATATATACAATATATAATAAATATAGTAATATATTA
ATGGTATGGACTTAACCAGGTTTATATATTATCATTTGATAAACATTAATTATAATTTTTCTATTTA
TTATTATTATTATACTGTGAGATTAATAATTATTAAAATATAATATTACATAGTACGTGATATTTGG
GGATTTTATCTTATTAATTGGCAATTAATGATTCTAATCAAATTTTATTCTCTTTAGTTTAATGGTA
GAACAATGATCTTCTAATTCATTGGTTTTAGTTCGATTCTAAAAAGAGATGAGTAAATAATTTTCT
AGATCAGAAATACTACTTTTAACTACAAAAAGCTTACGCTTTTTAACATTTTTTTGATAAATAACA
ACTATTGTTAATATTTGGCTGTCTATTGGTTTAACTAACAATTACAAAATTTTTTCAATTTTTATAAT
ATAAATTTAAATGAGAATATTAAAAAGTCATTCATTATTAAAATTAGTGAATTCTTACCTTATCGA
TGCGTCACAACCAAGTAACATTAGTTACTTGTGAAATTTTGGTTCATTATTAGCTGTTTGTTTAAT
AGTACAAATTATTACCGGTATTACATTAGCTATGCATTATAGTCCTAGTGTAATGGAAGCTTTTAA
CTCAATAGAGCATATAATGAGAGATGTTAATAACGGGTGATTAGTTCGTTATCTACATAGTAATA
CAGCTTCTGCTTTCTTTTTCTTAGTGTATTTACACATAGGAAGAGGTATATATTACGGATCATATA
GAGCTCCTCGTACTTTAGTTTGAGCTATTGGTACTGTTATATTAATATTAATGATGGCTATCGGTT
TCCTAGGTTATGTTTTACCTTATGGACAGATGTCATTATGAGGTGCTACAGTTATTACTAATCTTA
TTAGTGCTATACCTTGAATAGGGCAAGATATTGTTGAATTCATTTGAGGTGGTTTTTCTGTTAATA
ATGCCACTTTAAACAGATTTTTTGCATTACATTTTGTATTGCCTTTTGTATTAGCTGCTTTAGTTTTA
ATGCACTTAATTGCACTTCATGATACTGCTGGTTCAAGCAATCCTCTTGGTGTTTCAGGTAATTAC
GATAGAATTACATTTGCTCCATATTTTTTATTTAAAGATTTAATTACTATTTTTATATTTATTTTTGTA
TTAAGTGCTTTTGTATTCTTTATGCCTAATGTTTTAGGGGATAGTGATAATTATATTATGGCTAATC
CTATGCAAACTCCTGCTGCTATTGTACCTGAATGATACTTATTACCTTTCTATGCTATTTTAAGATC
TATACCTAATAAATTATTAGGTGTTATAGCGATGTTTAGTGCTATTTTAGCTATTATGTTATTACCT
GTTACAGATTTAGGTAGATCTAGAGGTTTACAATTTAGACCATTTAGTAAAATAGCTTTCTGAGTT
TTTGTTGCTAATTTCTTAGTTTTAATGCAATTAGGTGCTAAACACGTTGAAGATCCATTTATATTAT
TAGGTCAATTAAGTACTGTATTATACTTTAGTTATTTTGTTGCTATATTACCTTTAGCTAGTTACTT
AGATAATAGTTTAACTGATTTATCTAATAAATCTGAATTATTTTTAAATAAAACTAACTAAATATAT
TAAGATTATTATTTAATATATTTTCTATTTAAGATACTATTAATTTAGTATTTTGGGTTTTCAGTTTA
TAATTTATATTATATTATGCATTACCCTCCACCTTGCTTTGTAGTAAGCTAATCTGTTATTTCCTTTA
GTTTAATGGTAGAACAATGATCTTCTAATTCATTGGTTTTAGTTCGAATCTAAAAAGGAAATAAG

AAATATATTCTTATTATTACTTATATAATAATTATTTCTTAAAAATATACATTTTGCATTATAGCCGT
TTAGCTGTATTAAAATGTAAAATGATATAAAATAGAATAAATATTTAAATTATTCCTATGTTATATT
ATCCTATATTGCAACCATTATCAGAAGTTGTATTAATACTTGTACCTGCCTTATTAGCTGTAGCTT
ATGTTACAGTTGCTGAAAGAAAAACTATGGCTAGTATGCAAAGAAGATTAGGTCCTAATGCTGT
AGGTTACTATGGACTATTGCAAGCATTTGCTGATGCCTTAAAACTTTTATTAAAAGAATATGTAG
CTCCTACACAATCTAATATTGTTCTTTTCTTTTTAGGTCCTGTAATAACTTTAATTTTTGCATTATTA
GGTTACGCTGTTATACCCTATGGTCCTGGTTCAGGGATAAGCGACATGAATTTAGGTATATTTTA
CATGTTAGCTGTGTCATCTTTAGCTACATACGGTATTCTATTAGCTGGTTGAAGTGCGAATAGTA
AATACGCTTTTCTAGGTTCTCTTAGAAGTACAGCTCAATTAATTAGTTATGAATTAATATTAAGTT
CAGCTATATTAATAGTAATTATGATAACAGGAAATTTAAATTTAACTGTTTGTACTGAATCTCAAA
GAGCTATTTGATTTATACTACCTTTATTTCCTGTGTTTATAATATTTTTCATAGGATCTATAGCTGA
GACAAATAGAGCTCCTTTTGATTTAGCCGAGGCTAACCTGCTAATCTGGTTTGGTCTGGTTATAT
GTCACAAATTGCTAGGAAACCTTTTTATTTTAAAAACAAAAGACAATTAGCAGGAAACTTAATTT
AACCTAATTAAAAATATTAGATAATTAAACTCTTCATAGACTAAACGTGACAATTTAATATATATA
TATATTTATTTATATATATGATTAAATAAGATATAGTCAATCATCGGTGTGAATCGACTTAAAAAA
AAAAGCACATGGGTTAAACCCATCTCCCCTTATTAGGGGAATCAGAACTTGTTAGTGGGTTCAT
GACAGAGCATGCTGCCGTAGTTTTCGTATTCTTCTTTTTAGCTGAGTACGGTAGTATTGTACTAAT
GTGTATTTTAACTAGTATATTATTTATTGGTGGTTACTTATTATTTGAAATATCCTATGTTTTTACTG
TGGTAAATTATATTTTCTTTGAATTATTCTTTATAGACTGAGTAACATTTGTAGAGGTACAATCTTT
ATACACTGATTTTTTAAATAATTCTATCATTGAAGGATTATTATATGGGTTTAATCTAGGATTAAA
AAGTTCTTTAATGATATTCACATTTATTTGAGCTAGAGCATCCTTCCCTAGAATACGATTTGATCA
ACTAATGGGCTTCTGTTGAACAGTTTTATTACCTATTAATTTTGCAATTATTATATTAGTACCTTGT
GTTTTATATAGTTTTAACTTATTACCTGTAAATATACCATTGTTCTAGCTCACACACCCGCCCGCC
CTACTGCCACAAGGCTACAGTACATATGAGGAGGGGAACTAAAGATCTAGAACTATCCTAGTTA
ATAATTACACTTAATAGTATACTAAATAGACCATCTATCATACTCGAGAATAGTGATAGTGTAAT
TATACACTATTTTAACTGTATTCTACACTATTAGCATATTATTATCTTTATATGATAATAATTTTATA
ACTTAATTTATTAGGTTACATTAACAAAAAATTCGTTAACTTTTTTCACCACTTTTTAATACAAAAT
ACGAATTTTA
>YN355
ATAACAATTCTAAAGAACATAAAGAGTTATTAGATAAAAATAATTCACCTATACAGTTAATAAAT
CAACTTAAAGGGTATTTTTTCATAAATCCTTTATTAGCTTTAAGTTTAGCTATTACTATTTTCTCTTT
TGCAGGTATTCCTCCTCTTGTAGGGTTCTTTGCTAAACAGATGGTATTAAGCGCGGCTATTGATC
AAGGTTATATCTTTTTATCTTTAGTTGCAATATTAACTAGTGTTATAGGAGGGGTTTATTATTTAAA
TATAATTAAAGAAATGTTCTTTTATTCACCTGACTATAAATTAAACGAAGAAATTAAAAATAATAC
TATTAATGGTCAAATTATTAATAGAAACAATAAAATATTAAATGTTGAATTTAATTATACAAATGT
AGTTATGTCTAGTTCTGTGGCAATAACTATTTCTACTATTACATTAGTAGTTTTATTATTCATGTTT
ATGAATAAAGAATGATTAAGTCTGGGTACTATATTGGTACAATCTTTATTTAGCTATTAATGAGTA
GTATGACATTATTTATAGGGTTTGTATCTGTTATAGCTATTTTATTTTTAGCCATTAATTTTATATTT
GCTCCTCATAATCCTTATCAAGAAAAATATAGTATTTTCGAGTGTGGTTTCCATAGTTTTTTAGGG
CAAAATAGAACACAATTCGGTATAAAATTCTTTATTTTTGCTTTAGTTTATTTACTTTTAGATTTAG
AAATATTATTAACTTTCCCTTTCGCTCTTAGTGAGTATGTTAATGGTATTTATGGTCTTTTAGTTAC
TTTAATTTTTATAGCTATAATAACTATAGGATTTATATTTGAATTAGGTAAAAGCGCTCTTAAAATA
GACAGCAGACAAAAATTATATATACCTAAATTGAACGTTAATTACCATACAGAGTATGTTGGAAT
AGGTAAGGTTTCTAAGTAAAGTTATAGAGGCAGAAAACCAAAAAACCTACCAAAGGGTAGCTA

ATGGGAAGCTATTAAAAATAAAAGATGATAACCTATATATAGTATAGTTACTATATATTTACTATA
ACTAGGATTATTATATATATATATATTATCTTATTGTATATTAAGATTATTATTATTATAAGGTATA
ATTAATATAGTATCTTATTGTATAAGAATATAATATATTAACCTATAATTAATTTTATTTTTTAATTT
TTAATTATAATTTTTTTTTATATCTAGATGCTTACACATCTACAGATGTAGAAGAGAACAAAATAT
TGTTGTTATAGTAGTGGATGATATAGAAAAATATTTTTATATTATTTATTTTTGTTAGGTAGCTTTT
GAAGTGTTTGATAGAGAGGATATATGGACGGTAGGAGGGTATTCATTTTAATGAACAGTGGATA
GTTTAAATTAACCTAGTTATAGTTTATGAATTTAAATTATAATTATATTAATGTAGGTTATGATAGA
ATTATGTATTGATCCTTAAGAGTTAAGAGAGATACGCCCACGTATAATACATATTGGTTTAGGGT
TGAGTATATATACTTAAGAGTTAAGAATATATATATACAATATATAATAAATATAGTAATATATTA
ATGGTATGGACTTAACCAGGTTTATATATTATCATTTGATAAACATTAATTATAATTTTTCTATTTA
TTATTATTATTATACTGTGAGATTAATAATTATTAAAATATAATATTACATAGTACGTGATATTTGG
GGATTTTATCTTATTAATTGGCAATTAATGATTCTAATCAAATTTTATTCTCTTTAGTTTAATGGTA
GAACAATGATCTTCTAATTCATTGGTTTTAGTTCGATTCTAAAAAGAGATGAGTAAATAATTTTCT
AGATCAGAAATACTACTTTTAACTACAAAAAGCTTACGCTTTTTAACATTTTTTTGATAAATAACA
ACTATTGTTAATATTTGGCTGTCTATTGGTTTAACTAACAATTACAAAATTTTTTCAATTTTTATAAT
ATAAATTTAAATGAGAATATTAAAAAGTCATTCATTATTAAAATTAGTGAATTCTTACCTTATCGA
TGCGTCACAACCAAGTAACATTAGTTACTTGTGAAATTTTGGTTCATTATTAGCTGTTTGTTTAAT
AGTACAAATTATTACCGGTATTACATTAGCTATGCATTATAGTCCTAGTGTAATGGAAGCTTTTAA
CTCAATAGAGCATATAATGAGAGATGTTAATAACGGGTGATTAGTTCGTTATCTACATAGTAATA
CAGCTTCTGCTTTCTTTTTCTTAGTGTATTTACACATAGGAAGAGGTATATATTACGGATCATATA
GAGCTCCTCGTACTTTAGTTTGAGCTATTGGTACTGTTATATTAATATTAATGATGGCTATCGGTT
TCCTAGGTTATGTTTTACCTTATGGACAGATGTCATTATGAGGTGCTACAGTTATTACTAATCTTA
TTAGTGCTATACCTTGAATAGGGCAAGATATTGTTGAATTCATTTGAGGTGGTTTTTCTGTTAATA
ATGCCACTTTAAACAGATTTTTTGCATTACATTTTGTATTGCCTTTTGTATTAGCTGCTTTAGTTTTA
ATGCACTTAATTGCACTTCATGATACTGCTGGTTCAAGCAATCCTCTTGGTGTTTCAGGTAATTAC
GATAGAATTACATTTGCTCCATATTTTTTATTTAAAGATTTAATTACTATTTTTATATTTATTTTTGTA
TTAAGTGCTTTTGTATTCTTTATGCCTAATGTTTTAGGGGATAGTGATAATTATATTATGGCTAATC
CTATGCAAACTCCTGCTGCTATTGTACCTGAATGATACTTATTACCTTTCTATGCTATTTTAAGATC
TATACCTAATAAATTATTAGGTGTTATAGCGATGTTTAGTGCTATTTTAGCTATTATGTTATTACCT
GTTACAGATTTAGGTAGATCTAGAGGTTTACAATTTAGACCATTTAGTAAAATAGCTTTCTGAGTT
TTTGTTGCTAATTTCTTAGTTTTAATGCAATTAGGTGCTAAACACGTTGAAGATCCATTTATATTAT
TAGGTCAATTAAGTACTGTATTATACTTTAGTTATTTTGTTGCTATATTACCTTTAGCTAGTTACTT
AGATAATAGTTTAACTGATTTATCTAATAAATCTGAATTATTTTTAAATAAAACTAACTAAATATAT
TAAGATTATTATTTAATATATTTTCTATTTAAGATACTATTAATTTAGTATTTTGGGTTTTCAGTTTA
TAATTTATATTATATTATGCATTACCCTCCACCTTGCTTTGTAGTAAGCTAATCTGTTATTTCCTTTA
GTTTAATGGTAGAACAATGATCTTCTAATTCATTGGTTTTAGTTCGAATCTAAAAAGGAAATAAG
AAATATATTCTTATTATTACTTATATAATAATTATTTCTTAAAAATATACATTTTGCATTATAGCCGT
TTAGCTGTATTAAAATGTAAAATGATATAAAATAGAATAAATATTTAAATTATTCCTATGTTATATT
ATCCTATATTGCAACCATTATCAGAAGTTGTATTAATACTTGTACCTGCCTTATTAGCTGTAGCTT
ATGTTACAGTTGCTGAAAGAAAAACTATGGCTAGTATGCAAAGAAGATTAGGTCCTAATGCTGT
AGGTTACTATGGACTATTGCAAGCATTTGCTGATGCCTTAAAACTTTTATTAAAAGAATATGTAG
CTCCTACACAATCTAATATTGTTCTTTTCTTTTTAGGTCCTGTAATAACTTTAATTTTTGCATTATTA
GGTTACGCTGTTATACCCTATGGTCCTGGTTCAGGGATAAGCGACATGAATTTAGGTATATTTTA
CATGTTAGCTGTGTCATCTTTAGCTACATACGGTATTCTATTAGCTGGTTGAAGTGCGAATAGTA

AATACGCTTTTCTAGGTTCTCTTAGAAGTACAGCTCAATTAATTAGTTATGAATTAATATTAAGTT
CAGCTATATTAATAGTAATTATGATAACAGGAAATTTAAATTTAACTGTTTGTACTGAATCTCAAA
GAGCTATTTGATTTATACTACCTTTATTTCCTGTGTTTATAATATTTTTCATAGGATCTATAGCTGA
GACAAATAGAGCTCCTTTTGATTTAGCCGAGGCTAACCTGCTAATCTGGTTTGGTCTGGTTATAT
GTCACAAATTGCTAGGAAACCTTTTTATTTTAAAAACAAAAGACAATTAGCAGGAAACTTAATTT
AACCTAATTAAAAATATTAGATAATTAAACTCTTCATAGACTAAACGTGACAATTTAATATATATA
TATATTTATTTATATATATGATTAAATAAGATATAGTCAATCATCGGTGTGAATCGACTTAAAAAA
AAAAGCACATGGGTTAAACCCATCTCCCCTTATTAGGGGAATCAGAACTTGTTAGTGGGTTCAT
GACAGAGCATGCTGCCGTAGTTTTCGTATTCTTCTTTTTAGCTGAGTACGGTAGTATTGTACTAAT
GTGTATTTTAACTAGTATATTATTTATTGGTGGTTACTTATTATTTGAAATATCCTATGTTTTTACTG
TGGTAAATTATATTTTCTTTGAATTATTCTTTATAGACTGAGTAACATTTGTAGAGGTACAATCTTT
ATACACTGATTTTTTAAATAATTCTATCATTGAAGGATTATTATATGGGTTTAATCTAGGATTAAA
AAGTTCTTTAATGATATTCACATTTATTTGAGCTAGAGCATCCTTCCCTAGAATACGATTTGATCA
ACTAATGGGCTTCTGTTGAACAGTTTTATTACCTATTAATTTTGCAATTATTATATTAGTACCTTGT
GTTTTATATAGTTTTAACTTATTACCTGTAAATATACCATTGTTCTAGCTCACACACCCGCCCGCC
CTACTGCCACAAGGCTACAGTACATATGAGGAGGGGAACTAAAGATCTAGAACTATCCTAGTTA
ATAATTACACTTAATAGTATACTAAATAGACCATCTATCATACTCGAGAATAGTGATAGTGTAAT
TATACACTATTTTAACTGTATTCTACACTATTAGCATATTATTATCTTTATATGATAATAATTTTATA
ACTTAATTTATTAGGTTACATTAACAAAAAAATTCGTTAACTTTTTTCACCACTTTTTAATACAAAAT
ACGAATTTTA
>YN358
ATAACAATTCTAAAGAACATAAAGAGTTATTAGATAAAAATAATTCACCTATACAGTTAATAAAT
CAACTTAAAGGGTATTTTTTCATAAATCCTTTATTAGCTTTAAGTTTAGCTATTACTATTTTCTCTTT
TGCAGGTATTCCTCCTCTTGTAGGGTTCTTTGCTAAACAGATGGTATTAAGCGCGGCTATTGATC
AAGGTTATATCTTTTTATCTTTAGTTGCAATATTAACTAGTGTTATAGGAGGGGTTTATTATTTAAA
TATAATTAAAGAAATGTTCTTTTATTCACCTGACTATAAATTAAACGAAGAAATTAAAAATAATAC
TATTAATGGTCAAATTATTAATAGAAACAATAAAATATTAAATGTTGAATTTAATTATACAAATGT
AGTTATGTCTAGTTCTGTGGCAATAACTATTTCTACTATTACATTAGTAGTTTTATTATTCATGTTT
ATGAATAAAGAATGATTAAGTCTGGGTACTATATTGGTACAATCTTTATTTAGCTATTAATGAGTA
GTATGACATTATTTATAGGGTTTGTATCTGTTATAGCTATTTTATTTTTAGCCATTAATTTTATATTT
GCTCCTCATAATCCTTATCAAGAAAAATATAGTATTTTCGAGTGTGGTTTCCATAGTTTTTTAGGG
CAAAATAGAACACAATTCGGTATAAAATTCTTTATTTTTGCTTTAGTTTATTTACTTTTAGATTTAG
AAATATTATTAACTTTCCCTTTCGCTCTTAGTGAGTATGTTAATGGTATTTATGGTCTTTTAGTTAC
TTTAATTTTTATAGCTATAATAACTATAGGATTTATATTTGAATTAGGTAAAAGCGCTCTTAAAATA
GACAGCAGACAAAAATTATATATACCTAAATTGAACGTTAATTACCATACAGAGTATGTTGGAAT
AGGTAAGGTTTCTAAGTAAAGTTATAGAGGCAGAAAACCAAAAAACCTACCAAAGGGTAGCTA
ATGGGAAGCTATTAAAAATAAAAGATGATAACCTATATATAGTATAGTTACTATATATTTACTATA
ACTAGGATTATTATATATATATATATTATCTTATTGTATATTAAGATTATTATTATTATAAGGTATA
ATTAATATAGTATCTTATTGTATAAGAATATAATATATTAACCTATAATTAATTTTATTTTTTAATTT
TTAATTATAATTTTTTTTTATATCTAGATGCTTACACATCTACAGATGTAGAAGAGAACAAAATAT
TGTTGTTATAGTAGTGGATGATATAGAAAAATATTTTTATATTATTTATTTTTGTTAGGTAGCTTTT
GAAGTGTTTGATAGAGAGGATATATGGACGGTAGGAGGGTATTCATTTTAATGAACAGTGGATA
GTTTAAATTAACCTAGTTATAGTTTATGAATTTAAATTATAATTATATTAATGTAGGTTATGATAGA
ATTATGTATTGATCCTTAAGAGTTAAGAGAGATACGCCCACGTATAATACATATTGGTTTAGGGT

TGAGTATATATACTTAAGAGTTAAGAATATATATATACAATATATAATAAATATAGTAATATATTA
ATGGTATGGACTTAACCAGGTTTATATATTATCATTTGATAAACATTAATTATAATTTTTCTATTTA
TTATTATTATTATACTGTGAGATTAATAATTATTAAAATATAATATTACATAGTACGTGATATTTGG
GGATTTTATCTTATTAATTGGCAATTAATGATTCTAATCAAATTTTATTCTCTTTAGTTTAATGGTA
GAACAATGATCTTCTAATTCATTGGTTTTAGTTCGATTCTAAAAAGAGATGAGTAAATAATTTTCT
AGATCAGAAATACTACTTTTAACTACAAAAAGCTTACGCTTTTTAACATTTTTTTGATAAATAACA
ACTATTGTTAATATTTGGCTGTCTATTGGTTTAACTAACAATTACAAAATTTTTTCAATTTTTATAAT
ATAAATTTAAATGAGAATATTAAAAAGTCATTCATTATTAAAATTAGTGAATTCTTACCTTATCGA
TGCGTCACAACCAAGTAACATTAGTTACTTGTGAAATTTTGGTTCATTATTAGCTGTTTGTTTAAT
AGTACAAATTATTACCGGTATTACATTAGCTATGCATTATAGTCCTAGTGTAATGGAAGCTTTTAA
CTCAATAGAGCATATAATGAGAGATGTTAATAACGGGTGATTAGTTCGTTATCTACATAGTAATA
CAGCTTCTGCTTTCTTTTTCTTAGTGTATTTACACATAGGAAGAGGTATATATTACGGATCATATA
GAGCTCCTCGTACTTTAGTTTGAGCTATTGGTACTGTTATATTAATATTAATGATGGCTATCGGTT
TCCTAGGTTATGTTTTACCTTATGGACAGATGTCATTATGAGGTGCTACAGTTATTACTAATCTTA
TTAGTGCTATACCTTGAATAGGGCAAGATATTGTTGAATTCATTTGAGGTGGTTTTTCTGTTAATA
ATGCCACTTTAAACAGATTTTTTGCATTACATTTTGTATTGCCTTTTGTATTAGCTGCTTTAGTTTTA
ATGCACTTAATTGCACTTCATGATACTGCTGGTTCAAGCAATCCTCTTGGTGTTTCAGGTAATTAC
GATAGAATTACATTTGCTCCATATTTTTTATTTAAAGATTTAATTACTATTTTTATATTTATTTTTGTA
TTAAGTGCTTTTGTATTCTTTATGCCTAATGTTTTAGGGGATAGTGATAATTATATTATGGCTAATC
CTATGCAAACTCCTGCTGCTATTGTACCTGAATGATACTTATTACCTTTCTATGCTATTTTAAGATC
TATACCTAATAAATTATTAGGTGTTATAGCGATGTTTAGTGCTATTTTAGCTATTATGTTATTACCT
GTTACAGATTTAGGTAGATCTAGAGGTTTACAATTTAGACCATTTAGTAAAATAGCTTTCTGAGTT
TTTGTTGCTAATTTCTTAGTTTTAATGCAATTAGGTGCTAAACACGTTGAAGATCCATTTATATTAT
TAGGTCAATTAAGTACTGTATTATACTTTAGTTATTTTGTTGCTATATTACCTTTAGCTAGTTACTT
AGATAATAGTTTAACTGATTTATCTAATAAATCTGAATTATTTTTAAATAAAACTAACTAAATATAT
TAAGATTATTATTTAATATATTTTCTATTTAAGATACTATTAATTTAGTATTTTGGGTTTTCAGTTTA
TAATTTATATTATATTATGCATTACCCTCCACCTTGCTTTGTAGTAAGCTAATCTGTTATTTCCTTTA
GTTTAATGGTAGAACAATGATCTTCTAATTCATTGGTTTTAGTTCGAATCTAAAAAGGAAATAAG
AAATATATTCTTATTATTACTTATATAATAATTATTTCTTAAAAATATACATTTTGCATTATAGCCGT
TTAGCTGTATTAAAATGTAAAATGATATAAAATAGAATAAATATTTAAATTATTCCTATGTTATATT
ATCCTATATTGCAACCATTATCAGAAGTTGTATTAATACTTGTACCTGCCTTATTAGCTGTAGCTT
ATGTTACAGTTGCTGAAAGAAAAACTATGGCTAGTATGCAAAGAAGATTAGGTCCTAATGCTGT
AGGTTACTATGGACTATTGCAAGCATTTGCTGATGCCTTAAAACTTTTATTAAAAGAATATGTAG
CTCCTACACAATCTAATATTGTTCTTTTCTTTTTAGGTCCTGTAATAACTTTAATTTTTGCATTATTA
GGTTACGCTGTTATACCCTATGGTCCTGGTTCAGGGATAAGCGACATGAATTTAGGTATATTTTA
CATGTTAGCTGTGTCATCTTTAGCTACATACGGTATTCTATTAGCTGGTTGAAGTGCGAATAGTA
AATACGCTTTTCTAGGTTCTCTTAGAAGTACAGCTCAATTAATTAGTTATGAATTAATATTAAGTT
CAGCTATATTAATAGTAATTATGATAACAGGAAATTTAAATTTAACTGTTTGTACTGAATCTCAAA
GAGCTATTTGATTTATACTACCTTTATTTCCTGTGTTTATAATATTTTTCATAGGATCTATAGCTGA
GACAAATAGAGCTCCTTTTGATTTAGCCGAGGCTAACCTGCTAATCTGGTTTGGTCTGGTTATAT
GTCACAAATTGCTAGGAAACCTTTTTATTTTAAAAACAAAAGACAATTAGCAGGAAACTTAATTT
AACCTAATTAAAAATATTAGATAATTAAACTCTTCATAGACTAAACGTGACAATTTAATATATATA
TATATTTATTTATATATATGATTAAATAAGATATAGTCAATCATCGGTGTGAATCGACTTAAAAAA
AAAAGCACATGGGTTAAACCCATCTCCCCTTATTAGGGGAATCAGAACTTGTTAGTGGGTTCAT

GACAGAGCATGCTGCCGTAGTTTTCGTATTCTTCTTTTTAGCTGAGTACGGTAGTATTGTACTAAT
GTGTATTTTAACTAGTATATTATTTATTGGTGGTTACTTATTATTTGAAATATCCTATGTTTTTACTG
TGGTAAATTATATTTTCTTTGAATTATTCTTTATAGACTGAGTAACATTTGTAGAGGTACAATCTTT
ATACACTGATTTTTTAAATAATTCTATCATTGAAGGATTATTATATGGGTTTAATCTAGGATTAAA
AAGTTCTTTAATGATATTCACATTTATTTGAGCTAGAGCATCCTTCCCTAGAATACGATTTGATCA
ACTAATGGGCTTCTGTTGAACAGTTTTATTACCTATTAATTTTGCAATTATTATATTAGTACCTTGT
GTTTTATATAGTTTTAACTTATTACCTGTAAATATACCATTGTTCTAGCTCACACACCCGCCCGCC
CTACTGCCACAAGGCTACAGTACATATGAGGAGGGGAACTAAAGATCTAGAACTATCCTAGTTA
ATAATTACACTTAATAGTATACTAAATAGACCATCTATCATACTCGAGAATAGTGATAGTGTAAT
TATACACTATTTTAACTGTATTCTACACTATTAGCATATTATTATCTTTATATGATAATAATTTTATA
ACTTAATTTATTAGGTTACATTAACAAAAAATTCGTTAACTTTTTTCACCACTTTTTAATACAAAAT
ACGAATTTTA
>YN359
ATAACAATTCTAAAGAACATAAAGAGTTATTAGATAAAAATAATTCACCTATACAGTTAATAAAT
CAACTTAAAGGGTATTTTTTCATAAATCCTTTATTAGCTTTAAGTTTAGCTATTACTATTTTCTCTTT
TGCAGGTATTCCTCCTCTTGTAGGGTTCTTTGCTAAACAGATGGTATTAAGCGCGGCTATTGATC
AAGGTTATATCTTTTTATCTTTAGTTGCAATATTAACTAGTGTTATAGGAGGGGTTTATTATTTAAA
TATAATTAAAGAAATGTTCTTTTATTCACCTGACTATAAATTAAACGAAGAAATTAAAAATAATAC
TATTAATGGTCAAATTATTAATAGAAACAATAAAATATTAAATGTTGAATTTAATTATACAAATGT
AGTTATGTCTAGTTCTGTGGCAATAACTATTTCTACTATTACATTAGTAGTTTTATTATTCATGTTT
ATGAATAAAGAATGATTAAGTCTGGGTACTATATTGGTACAATCTTTATTTAGCTATTAATGAGTA
GTATGACATTATTTATAGGGTTTGTATCTGTTATAGCTATTTTATTTTTAGCCATTAATTTTATATTT
GCTCCTCATAATCCTTATCAAGAAAAATATAGTATTTTCGAGTGTGGTTTCCATAGTTTTTTAGGG
CAAAATAGAACACAATTCGGTATAAAATTCTTTATTTTTGCTTTAGTTTATTTACTTTTAGATTTAG
AAATATTATTAACTTTCCCTTTCGCTCTTAGTGAGTATGTTAATGGTATTTATGGTCTTTTAGTTAC
TTTAATTTTTATAGCTATAATAACTATAGGATTTATATTTGAATTAGGTAAAAGCGCTCTTAAAATA
GACAGCAGACAAAAATTATATATACCTAAATTGAACGTTAATTACCATACAGAGTATGTTGGAAT
AGGTAAGGTTTCTAAGTAAAGTTATAGAGGCAGAAAACCAAAAAACCTACCAAAGGGTAGCTA
ATGGGAAGCTATTAAAAATAAAAGATGATAACCTATATATAGTATAGTTACTATATATTTACTATA
ACTAGGATTATTATATATATATATATTATCTTATTGTATATTAAGATTATTATTATTATAAGGTATA
ATTAATATAGTATCTTATTGTATAAGAATATAATATATTAACCTATAATTAATTTTATTTTTTAATTT
TTAATTATAATTTTTTTTTATATCTAGATGCTTACACATCTACAGATGTAGAAGAGAACAAAATAT
TGTTGTTATAGTAGTGGATGATATAGAAAAATATTTTTATATTATTTATTTTTGTTAGGTAGCTTTT
GAAGTGTTTGATAGAGAGGATATATGGACGGTAGGAGGGTATTCATTTTAATGAACAGTGGATA
GTTTAAATTAACCTAGTTATAGTTTATGAATTTAAATTATAATTATATTAATGTAGGTTATGATAGA
ATTATGTATTGATCCTTAAGAGTTAAGAGAGATACGCCCACGTATAATACATATTGGTTTAGGGT
TGAGTATATATACTTAAGAGTTAAGAATATATATATACAATATATAATAAATATAGTAATATATTA
ATGGTATGGACTTAACCAGGTTTATATATTATCATTTGATAAACATTAATTATAATTTTTCTATTTA
TTATTATTATTATACTGTGAGATTAATAATTATTAAAATATAATATTACATAGTACGTGATATTTGG
GGATTTTATCTTATTAATTGGCAATTAATGATTCTAATCAAATTTTATTCTCTTTAGTTTAATGGTA
GAACAATGATCTTCTAATTCATTGGTTTTAGTTCGATTCTAAAAAGAGATGAGTAAATAATTTTCT
AGATCAGAAATACTACTTTTAACTACAAAAAGCTTACGCTTTTTAACATTTTTTTGATAAATAACA
ACTATTGTTAATATTTGGCTGTCTATTGGTTTAACTAACAATTACAAAATTTTTTCAATTTTTATAAT
ATAAATTTAAATGAGAATATTAAAAAGTCATTCATTATTAAAATTAGTGAATTCTTACCTTATCGA

TGCGTCACAACCAAGTAACATTAGTTACTTGTGAAATTTTGGTTCATTATTAGCTGTTTGTTTAAT
AGTACAAATTATTACCGGTATTACATTAGCTATGCATTATAGTCCTAGTGTAATGGAAGCTTTTAA
CTCAATAGAGCATATAATGAGAGATGTTAATAACGGGTGATTAGTTCGTTATCTACATAGTAATA
CAGCTTCTGCTTTCTTTTTCTTAGTGTATTTACACATAGGAAGAGGTATATATTACGGATCATATA
GAGCTCCTCGTACTTTAGTTTGAGCTATTGGTACTGTTATATTAATATTAATGATGGCTATCGGTT
TCCTAGGTTATGTTTTACCTTATGGACAGATGTCATTATGAGGTGCTACAGTTATTACTAATCTTA
TTAGTGCTATACCTTGAATAGGGCAAGATATTGTTGAATTCATTTGAGGTGGTTTTTCTGTTAATA
ATGCCACTTTAAACAGATTTTTTGCATTACATTTTGTATTGCCTTTTGTATTAGCTGCTTTAGTTTTA
ATGCACTTAATTGCACTTCATGATACTGCTGGTTCAAGCAATCCTCTTGGTGTTTCAGGTAATTAC
GATAGAATTACATTTGCTCCATATTTTTTATTTAAAGATTTAATTACTATTTTTATATTTATTTTTGTA
TTAAGTGCTTTTGTATTCTTTATGCCTAATGTTTTAGGGGATAGTGATAATTATATTATGGCTAATC
CTATGCAAACTCCTGCTGCTATTGTACCTGAATGATACTTATTACCTTTCTATGCTATTTTAAGATC
TATACCTAATAAATTATTAGGTGTTATAGCGATGTTTAGTGCTATTTTAGCTATTATGTTATTACCT
GTTACAGATTTAGGTAGATCTAGAGGTTTACAATTTAGACCATTTAGTAAAATAGCTTTCTGAGTT
TTTGTTGCTAATTTCTTAGTTTTAATGCAATTAGGTGCTAAACACGTTGAAGATCCATTTATATTAT
TAGGTCAATTAAGTACTGTATTATACTTTAGTTATTTTGTTGCTATATTACCTTTAGCTAGTTACTT
AGATAATAGTTTAACTGATTTATCTAATAAATCTGAATTATTTTTAAATAAAACTAACTAAATATAT
TAAGATTATTATTTAATATATTTTCTATTTAAGATACTATTAATTTAGTATTTTGGGTTTTCAGTTTA
TAATTTATATTATATTATGCATTACCCTCCACCTTGCTTTGTAGTAAGCTAATCTGTTATTTCCTTTA
GTTTAATGGTAGAACAATGATCTTCTAATTCATTGGTTTTAGTTCGAATCTAAAAAGGAAATAAG
AAATATATTCTTATTATTACTTATATAATAATTATTTCTTAAAAATATACATTTTGCATTATAGCCGT
TTAGCTGTATTAAAATGTAAAATGATATAAAATAGAATAAATATTTAAATTATTCCTATGTTATATT
ATCCTATATTGCAACCATTATCAGAAGTTGTATTAATACTTGTACCTGCCTTATTAGCTGTAGCTT
ATGTTACAGTTGCTGAAAGAAAAACTATGGCTAGTATGCAAAGAAGATTAGGTCCTAATGCTGT
AGGTTACTATGGACTATTGCAAGCATTTGCTGATGCCTTAAAACTTTTATTAAAAGAATATGTAG
CTCCTACACAATCTAATATTGTTCTTTTCTTTTTAGGTCCTGTAATAACTTTAATTTTTGCATTATTA
GGTTACGCTGTTATACCCTATGGTCCTGGTTCAGGGATAAGCGACATGAATTTAGGTATATTTTA
CATGTTAGCTGTGTCATCTTTAGCTACATACGGTATTCTATTAGCTGGTTGAAGTGCGAATAGTA
AATACGCTTTTCTAGGTTCTCTTAGAAGTACAGCTCAATTAATTAGTTATGAATTAATATTAAGTT
CAGCTATATTAATAGTAATTATGATAACAGGAAATTTAAATTTAACTGTTTGTACTGAATCTCAAA
GAGCTATTTGATTTATACTACCTTTATTTCCTGTGTTTATAATATTTTTCATAGGATCTATAGCTGA
GACAAATAGAGCTCCTTTTGATTTAGCCGAGGCTAACCTGCTAATCTGGTTTGGTCTGGTTATAT
GTCACAAATTGCTAGGAAACCTTTTTATTTTAAAAACAAAAGACAATTAGCAGGAAACTTAATTT
AACCTAATTAAAAATATTAGATAATTAAACTCTTCATAGACTAAACGTGACAATTTAATATATATA
TATATTTATTTATATATATGATTAAATAAGATATAGTCAATCATCGGTGTGAATCGACTTAAAAAA
AAAAGCACATGGGTTAAACCCATCTCCCCTTATTAGGGGAATCAGAACTTGTTAGTGGGTTCAT
GACAGAGCATGCTGCCGTAGTTTTCGTATTCTTCTTTTTAGCTGAGTACGGTAGTATTGTACTAAT
GTGTATTTTAACTAGTATATTATTTATTGGTGGTTACTTATTATTTGAAATATCCTATGTTTTTACTG
TGGTAAATTATATTTTCTTTGAATTATTCTTTATAGACTGAGTAACATTTGTAGAGGTACAATCTTT
ATACACTGATTTTTTAAATAATTCTATCATTGAAGGATTATTATATGGGTTTAATCTAGGATTAAA
AAGTTCTTTAATGATATTCACATTTATTTGAGCTAGAGCATCCTTCCCTAGAATACGATTTGATCA
ACTAATGGGCTTCTGTTGAACAGTTTTATTACCTATTAATTTTGCAATTATTATATTAGTACCTTGT
GTTTTATATAGTTTTAACTTATTACCTGTAAATATACCATTGTTCTAGCTCACACACCCGCCCGCC
CTACTGCCACAAGGCTACAGTACATATGAGGAGGGGAACTAAAGATCTAGAACTATCCTAGTTA

ATAATTACACTTAATAGTATACTAAATAGACCATCTATCATACTCGAGAATAGTGATAGTGTAAT
TATACACTATTTTAACTGTATTCTACACTATTAGCATATTATTATCTTTATATGATAATAATTTTATA
ACTTAATTTATTAGGTTACATTAACAAAAAATTCGTTAACTTTTTTCACCACTTTTTAATACAAAAT
ACGAATTTTA
>YN365
ATAACAATTCTAAAGAACATAAAGAGTTATTAGATAAAAATAATTCACCTATACAGTTAATAAAT
CAACTTAAAGGGTATTTTTTCATAAATCCTTTATTAGCTTTAAGTTTAGCTATTACTATTTTCTCTTT
TGCAGGTATTCCTCCTCTTGTAGGGTTCTTTGCTAAACAGATGGTATTAAGCGCGGCTATTGATC
AAGGTTATATCTTTTTATCTTTAGTTGCAATATTAACTAGTGTTATAGGAGGGGTTTATTATTTAAA
TATAATTAAAGAAATGTTCTTTTATTCACCTGACTATAAATTAAACGAAGAAATTAAAAATAATAC
TATTAATGGTCAAATTATTAATAGAAACAATAAAATATTAAATGTTGAATTTAATTATACAAATGT
AGTTATGTCTAGTTCTGTGGCAATAACTATTTCTACTATTACATTAGTAGTTTTATTATTCATGTTT
ATGAATAAAGAATGATTAAGTCTGGGTACTATATTGGTACAATCTTTATTTAGCTATTAATGAGTA
GTATGACATTATTTATAGGGTTTGTATCTGTTATAGCTATTTTATTTTTAGCCATTAATTTTATATTT
GCTCCTCATAATCCTTATCAAGAAAAATATAGTATTTTCGAGTGTGGTTTCCATAGTTTTTTAGGG
CAAAATAGAACACAATTCGGTATAAAATTCTTTATTTTTGCTTTAGTTTATTTACTTTTAGATTTAG
AAATATTATTAACTTTCCCTTTCGCTCTTAGTGAGTATGTTAATGGTATTTATGGTCTTTTAGTTAC
TTTAATTTTTATAGCTATAATAACTATAGGATTTATATTTGAATTAGGTAAAAGCGCTCTTAAAATA
GACAGCAGACAAAAATTATATATACCTAAATTGAACGTTAATTACCATACAGAGTATGTTGGAAT
AGGTAAGGTTTCTAAGTAAAGTTATAGAGGCAGAAAACCAAAAAACCTACCAAAGGGTAGCTA
ATGGGAAGCTATTAAAAATAAAAGATGATAACCTATATATAGTATAGTTACTATATATTTACTATA
ACTAGGATTATTATATATATATATATTATCTTATTGTATATTAAGATTATTATTATTATAAGGTATA
ATTAATATAGTATCTTATTGTATAAGAATATAATATATTAACCTATAATTAATTTTATTTTTTAATTT
TTAATTATAATTTTTTTTTATATCTAGATGCTTACACATCTACAGATGTAGAAGAGAACAAAATAT
TGTTGTTATAGTAGTGGATGATATAGAAAAATATTTTTATATTATTTATTTTTGTTAGGTAGCTTTT
GAAGTGTTTGATAGAGAGGATATATGGACGGTAGGAGGGTATTCATTTTAATGAACAGTGGATA
GTTTAAATTAACCTAGTTATAGTTTATGAATTTAAATTATAATTATATTAATGTAGGTTATGATAGA
ATTATGTATTGATCCTTAAGAGTTAAGAGAGATACGCCCACGTATAATACATATTGGTTTAGGGT
TGAGTATATATACTTAAGAGTTAAGAATATATATATACAATATATAATAAATATAGTAATATATTA
ATGGTATGGACTTAACCAGGTTTATATATTATCATTTGATAAACATTAATTATAATTTTTCTATTTA
TTATTATTATTATACTGTGAGATTAATAATTATTAAAATATAATATTACATAGTACGTGATATTTGG
GGATTTTATCTTATTAATTGGCAATTAATGATTCTAATCAAATTTTATTCTCTTTAGTTTAATGGTA
GAACAATGATCTTCTAATTCATTGGTTTTAGTTCGATTCTAAAAAGAGATGAGTAAATAATTTTCT
AGATCAGAAATACTACTTTTAACTACAAAAAGCTTACGCTTTTTAACATTTTTTTGATAAATAACA
ACTATTGTTAATATTTGGCTGTCTATTGGTTTAACTAACAATTACAAAATTTTTTCAATTTTTATAAT
ATAAATTTAAATGAGAATATTAAAAAGTCATTCATTATTAAAATTAGTGAATTCTTACCTTATCGA
TGCGTCACAACCAAGTAACATTAGTTACTTGTGAAATTTTGGTTCATTATTAGCTGTTTGTTTAAT
AGTACAAATTATTACCGGTATTACATTAGCTATGCATTATAGTCCTAGTGTAATGGAAGCTTTTAA
CTCAATAGAGCATATAATGAGAGATGTTAATAACGGGTGATTAGTTCGTTATCTACATAGTAATA
CAGCTTCTGCTTTCTTTTTCTTAGTGTATTTACACATAGGAAGAGGTATATATTACGGATCATATA
GAGCTCCTCGTACTTTAGTTTGAGCTATTGGTACTGTTATATTAATATTAATGATGGCTATCGGTT
TCCTAGGTTATGTTTTACCTTATGGACAGATGTCATTATGAGGTGCTACAGTTATTACTAATCTTA
TTAGTGCTATACCTTGAATAGGGCAAGATATTGTTGAATTCATTTGAGGTGGTTTTTCTGTTAATA
ATGCCACTTTAAACAGATTTTTTGCATTACATTTTGTATTGCCTTTTGTATTAGCTGCTTTAGTTTTA

ATGCACTTAATTGCACTTCATGATACTGCTGGTTCAAGCAATCCTCTTGGTGTTTCAGGTAATTAC
GATAGAATTACATTTGCTCCATATTTTTTATTTAAAGATTTAATTACTATTTTTATATTTATTTTTGTA
TTAAGTGCTTTTGTATTCTTTATGCCTAATGTTTTAGGGGATAGTGATAATTATATTATGGCTAATC
CTATGCAAACTCCTGCTGCTATTGTACCTGAATGATACTTATTACCTTTCTATGCTATTTTAAGATC
TATACCTAATAAATTATTAGGTGTTATAGCGATGTTTAGTGCTATTTTAGCTATTATGTTATTACCT
GTTACAGATTTAGGTAGATCTAGAGGTTTACAATTTAGACCATTTAGTAAAATAGCTTTCTGAGTT
TTTGTTGCTAATTTCTTAGTTTTAATGCAATTAGGTGCTAAACACGTTGAAGATCCATTTATATTAT
TAGGTCAATTAAGTACTGTATTATACTTTAGTTATTTTGTTGCTATATTACCTTTAGCTAGTTACTT
AGATAATAGTTTAACTGATTTATCTAATAAATCTGAATTATTTTTAAATAAAACTAACTAAATATAT
TAAGATTATTATTTAATATATTTTCTATTTAAGATACTATTAATTTAGTATTTTGGGTTTTCAGTTTA
TAATTTATATTATATTATGCATTACCCTCCACCTTGCTTTGTAGTAAGCTAATCTGTTATTTCCTTTA
GTTTAATGGTAGAACAATGATCTTCTAATTCATTGGTTTTAGTTCGAATCTAAAAAGGAAATAAG
AAATATATTCTTATTATTACTTATATAATAATTATTTCTTAAAAATATACATTTTGCATTATAGCCGT
TTAGCTGTATTAAAATGTAAAATGATATAAAATAGAATAAATATTTAAATTATTCCTATGTTATATT
ATCCTATATTGCAACCATTATCAGAAGTTGTATTAATACTTGTACCTGCCTTATTAGCTGTAGCTT
ATGTTACAGTTGCTGAAAGAAAAACTATGGCTAGTATGCAAAGAAGATTAGGTCCTAATGCTGT
AGGTTACTATGGACTATTGCAAGCATTTGCTGATGCCTTAAAACTTTTATTAAAAGAATATGTAG
CTCCTACACAATCTAATATTGTTCTTTTCTTTTTAGGTCCTGTAATAACTTTAATTTTTGCATTATTA
GGTTACGCTGTTATACCCTATGGTCCTGGTTCAGGGATAAGCGACATGAATTTAGGTATATTTTA
CATGTTAGCTGTGTCATCTTTAGCTACATACGGTATTCTATTAGCTGGTTGAAGTGCGAATAGTA
AATACGCTTTTCTAGGTTCTCTTAGAAGTACAGCTCAATTAATTAGTTATGAATTAATATTAAGTT
CAGCTATATTAATAGTAATTATGATAACAGGAAATTTAAATTTAACTGTTTGTACTGAATCTCAAA
GAGCTATTTGATTTATACTACCTTTATTTCCTGTGTTTATAATATTTTTCATAGGATCTATAGCTGA
GACAAATAGAGCTCCTTTTGATTTAGCCGAGGCTAACCTGCTAATCTGGTTTGGTCTGGTTATAT
GTCACAAATTGCTAGGAAACCTTTTTATTTTAAAAACAAAAGACAATTAGCAGGAAACTTAATTT
AACCTAATTAAAAATATTAGATAATTAAACTCTTCATAGACTAAACGTGACAATTTAATATATATA
TATATTTATTTATATATATGATTAAATAAGATATAGTCAATCATCGGTGTGAATCGACTTAAAAAA
AAAAGCACATGGGTTAAACCCATCTCCCCTTATTAGGGGAATCAGAACTTGTTAGTGGGTTCAT
GACAGAGCATGCTGCCGTAGTTTTCGTATTCTTCTTTTTAGCTGAGTACGGTAGTATTGTACTAAT
GTGTATTTTAACTAGTATATTATTTATTGGTGGTTACTTATTATTTGAAATATCCTATGTTTTTACTG
TGGTAAATTATATTTTCTTTGAATTATTCTTTATAGACTGAGTAACATTTGTAGAGGTACAATCTTT
ATACACTGATTTTTTAAATAATTCTATCATTGAAGGATTATTATATGGGTTTAATCTAGGATTAAA
AAGTTCTTTAATGATATTCACATTTATTTGAGCTAGAGCATCCTTCCCTAGAATACGATTTGATCA
ACTAATGGGCTTCTGTTGAACAGTTTTATTACCTATTAATTTTGCAATTATTATATTAGTACCTTGT
GTTTTATATAGTTTTAACTTATTACCTGTAAATATACCATTGTTCTAGCTCACACACCCGCCCGCC
CTACTGCCACAAGGCTACAGTACATATGAGGAGGGGAACTAAAGATCTAGAACTATCCTAGTTA
ATAATTACACTTAATAGTATACTAAATAGACCATCTATCATACTCGAGAATAGTGATAGTGTAAT
TATACACTATTTTAACTGTATTCTACACTATTAGCATATTATTATCTTTATATGATAATAATTTTATA
ACTTAATTTATTAGGTTACATTAACAAAAAATTCGTTAACTTTTTTCACCACTTTTTAATACAAAAT
ACGAATTTTA
>YN376
ATAACAATTCTAAAGAACATAAAGAGTTATTAGATAAAAATAATTCACCTATACAGTTAATAAAT
CAACTTAAAGGGTATTTTTTCATAAATCCTTTATTAGCTTTAAGTTTAGCTATTACTATTTTCTCTTT
TGCAGGTATTCCTCCTCTTGTAGGGTTCTTTGCTAAACAGATGGTATTAAGCGCGGCTATTGATC

AAGGTTATATCTTTTTATCTTTAGTTGCAATATTAACTAGTGTTATAGGAGGGGTTTATTATTTAAA
TATAATTAAAGAAATGTTCTTTTATTCACCTGACTATAAATTAAACGAAGAAATTAAAAATAATAC
TATTAATGGTCAAATTATTAATAGAAACAATAAAATATTAAATGTTGAATTTAATTATACAAATGT
AGTTATGTCTAGTTCTGTGGCAATAACTATTTCTACTATTACATTAGTAGTTTTATTATTCATGTTT
ATGAATAAAGAATGATTAAGTCTGGGTACTATATTGGTACAATCTTTATTTAGCTATTAATGAGTA
GTATGACATTATTTATAGGGTTTGTATCTGTTATAGCTATTTTATTTTTAGCCATTAATTTTATATTT
GCTCCTCATAATCCTTATCAAGAAAAATATAGTATTTTCGAGTGTGGTTTCCATAGTTTTTTAGGG
CAAAATAGAACACAATTCGGTATAAAATTCTTTATTTTTGCTTTAGTTTATTTACTTTTAGATTTAG
AAATATTATTAACTTTCCCTTTCGCTCTTAGTGAGTATGTTAATGGTATTTATGGTCTTTTAGTTAC
TTTAATTTTTATAGCTATAATAACTATAGGATTTATATTTGAATTAGGTAAAAGCGCTCTTAAAATA
GACAGCAGACAAAAATTATATATACCTAAATTGAACGTTAATTACCATACAGAGTATGTTGGAAT
AGGTAAGGTTTCTAAGTAAAGTTATAGAGGCAGAAAACCAAAAAACCTACCAAAGGGTAGCTA
ATGGGAAGCTATTAAAAATAAAAGATGATAACCTATATATAGTATAGTTACTATATATTTACTATA
ACTAGGATTATTATATATATATATATTATCTTATTGTATATTAAGATTATTATTATTATAAGGTATA
ATTAATATAGTATCTTATTGTATAAGAATATAATATATTAACCTATAATTAATTTTATTTTTTAATTT
TTAATTATAATTTTTTTTTATATCTAGATGCTTACACATCTACAGATGTAGAAGAGAACAAAATAT
TGTTGTTATAGTAGTGGATGATATAGAAAAATATTTTTATATTATTTATTTTTGTTAGGTAGCTTTT
GAAGTGTTTGATAGAGAGGATATATGGACGGTAGGAGGGTATTCATTTTAATGAACAGTGGATA
GTTTAAATTAACCTAGTTATAGTTTATGAATTTAAATTATAATTATATTAATGTAGGTTATGATAGA
ATTATGTATTGATCCTTAAGAGTTAAGAGAGATACGCCCACGTATAATACATATTGGTTTAGGGT
TGAGTATATATACTTAAGAGTTAAGAATATATATATACAATATATAATAAATATAGTAATATATTA
ATGGTATGGACTTAACCAGGTTTATATATTATCATTTGATAAACATTAATTATAATTTTTCTATTTA
TTATTATTATTATACTGTGAGATTAATAATTATTAAAATATAATATTACATAGTACGTGATATTTGG
GGATTTTATCTTATTAATTGGCAATTAATGATTCTAATCAAATTTTATTCTCTTTAGTTTAATGGTA
GAACAATGATCTTCTAATTCATTGGTTTTAGTTCGATTCTAAAAAGAGATGAGTAAATAATTTTCT
AGATCAGAAATACTACTTTTAACTACAAAAAGCTTACGCTTTTTAACATTTTTTTGATAAATAACA
ACTATTGTTAATATTTGGCTGTCTATTGGTTTAACTAACAATTACAAAATTTTTTCAATTTTTATAAT
ATAAATTTAAATGAGAATATTAAAAAGTCATTCATTATTAAAATTAGTGAATTCTTACCTTATCGA
TGCGTCACAACCAAGTAACATTAGTTACTTGTGAAATTTTGGTTCATTATTAGCTGTTTGTTTAAT
AGTACAAATTATTACCGGTATTACATTAGCTATGCATTATAGTCCTAGTGTAATGGAAGCTTTTAA
CTCAATAGAGCATATAATGAGAGATGTTAATAACGGGTGATTAGTTCGTTATCTACATAGTAATA
CAGCTTCTGCTTTCTTTTTCTTAGTGTATTTACACATAGGAAGAGGTATATATTACGGATCATATA
GAGCTCCTCGTACTTTAGTTTGAGCTATTGGTACTGTTATATTAATATTAATGATGGCTATCGGTT
TCCTAGGTTATGTTTTACCTTATGGACAGATGTCATTATGAGGTGCTACAGTTATTACTAATCTTA
TTAGTGCTATACCTTGAATAGGGCAAGATATTGTTGAATTCATTTGAGGTGGTTTTTCTGTTAATA
ATGCCACTTTAAACAGATTTTTTGCATTACATTTTGTATTGCCTTTTGTATTAGCTGCTTTAGTTTTA
ATGCACTTAATTGCACTTCATGATACTGCTGGTTCAAGCAATCCTCTTGGTGTTTCAGGTAATTAC
GATAGAATTACATTTGCTCCATATTTTTTATTTAAAGATTTAATTACTATTTTTATATTTATTTTTGTA
TTAAGTGCTTTTGTATTCTTTATGCCTAATGTTTTAGGGGATAGTGATAATTATATTATGGCTAATC
CTATGCAAACTCCTGCTGCTATTGTACCTGAATGATACTTATTACCTTTCTATGCTATTTTAAGATC
TATACCTAATAAATTATTAGGTGTTATAGCGATGTTTAGTGCTATTTTAGCTATTATGTTATTACCT
GTTACAGATTTAGGTAGATCTAGAGGTTTACAATTTAGACCATTTAGTAAAATAGCTTTCTGAGTT
TTTGTTGCTAATTTCTTAGTTTTAATGCAATTAGGTGCTAAACACGTTGAAGATCCATTTATATTAT
TAGGTCAATTAAGTACTGTATTATACTTTAGTTATTTTGTTGCTATATTACCTTTAGCTAGTTACTT

AGATAATAGTTTAACTGATTTATCTAATAAATCTGAATTATTTTTAAATAAAACTAACTAAATATAT
TAAGATTATTATTTAATATATTTTCTATTTAAGATACTATTAATTTAGTATTTTGGGTTTTCAGTTTA
TAATTTATATTATATTATGCATTACCCTCCACCTTGCTTTGTAGTAAGCTAATCTGTTATTTCCTTTA
GTTTAATGGTAGAACAATGATCTTCTAATTCATTGGTTTTAGTTCGAATCTAAAAAGGAAATAAG
AAATATATTCTTATTATTACTTATATAATAATTATTTCTTAAAAATATACATTTTGCATTATAGCCGT
TTAGCTGTATTAAAATGTAAAATGATATAAAATAGAATAAATATTTAAATTATTCCTATGTTATATT
ATCCTATATTGCAACCATTATCAGAAGTTGTATTAATACTTGTACCTGCCTTATTAGCTGTAGCTT
ATGTTACAGTTGCTGAAAGAAAAACTATGGCTAGTATGCAAAGAAGATTAGGTCCTAATGCTGT
AGGTTACTATGGACTATTGCAAGCATTTGCTGATGCCTTAAAACTTTTATTAAAAGAATATGTAG
CTCCTACACAATCTAATATTGTTCTTTTCTTTTTAGGTCCTGTAATAACTTTAATTTTTGCATTATTA
GGTTACGCTGTTATACCCTATGGTCCTGGTTCAGGGATAAGCGACATGAATTTAGGTATATTTTA
CATGTTAGCTGTGTCATCTTTAGCTACATACGGTATTCTATTAGCTGGTTGAAGTGCGAATAGTA
AATACGCTTTTCTAGGTTCTCTTAGAAGTACAGCTCAATTAATTAGTTATGAATTAATATTAAGTT
CAGCTATATTAATAGTAATTATGATAACAGGAAATTTAAATTTAACTGTTTGTACTGAATCTCAAA
GAGCTATTTGATTTATACTACCTTTATTTCCTGTGTTTATAATATTTTTCATAGGATCTATAGCTGA
GACAAATAGAGCTCCTTTTGATTTAGCCGAGGCTAACCTGCTAATCTGGTTTGGTCTGGTTATAT
GTCACAAATTGCTAGGAAACCTTTTTATTTTAAAAACAAAAGACAATTAGCAGGAAACTTAATTT
AACCTAATTAAAAATATTAGATAATTAAACTCTTCATAGACTAAACGTGACAATTTAATATATATA
TATATTTATTTATATATATGATTAAATAAGATATAGTCAATCATCGGTGTGAATCGACTTAAAAAA
AAAAGCACATGGGTTAAACCCATCTCCCCTTATTAGGGGAATCAGAACTTGTTAGTGGGTTCAT
GACAGAGCATGCTGCCGTAGTTTTCGTATTCTTCTTTTTAGCTGAGTACGGTAGTATTGTACTAAT
GTGTATTTTAACTAGTATATTATTTATTGGTGGTTACTTATTATTTGAAATATCCTATGTTTTTACTG
TGGTAAATTATATTTTCTTTGAATTATTCTTTATAGACTGAGTAACATTTGTAGAGGTACAATCTTT
ATACACTGATTTTTTAAATAATTCTATCATTGAAGGATTATTATATGGGTTTAATCTAGGATTAAA
AAGTTCTTTAATGATATTCACATTTATTTGAGCTAGAGCATCCTTCCCTAGAATACGATTTGATCA
ACTAATGGGCTTCTGTTGAACAGTTTTATTACCTATTAATTTTGCAATTATTATATTAGTACCTTGT
GTTTTATATAGTTTTAACTTATTACCTGTAAATATACCATTGTTCTAGCTCACACACCCGCCCGCC
CTACTGCCACAAGGCTACAGTACATATGAGGAGGGGAACTAAAGATCTAGAACTATCCTAGTTA
ATAATTACACTTAATAGTATACTAAATAGACCATCTATCATACTCGAGAATAGTGATAGTGTAAT
TATACACTATTTTAACTGTATTCTACACTATTAGCATATTATTATCTTTATATGATAATAATTTTATA
ACTTAATTTATTAGGTTACATTAACAAAAAATTCGTTAACTTTTTTCACCACTTTTTAATACAAAAT
ACGAATTTTA
>YN390
ATAACAATTCTAAAGAACATAAAGAGTTATTAGATAAAAATAATTCACCTATACAGTTAATAAAT
CAACTTAAAGGGTATTTTTTCATAAATCCTTTATTAGCTTTAAGTTTAGCTATTACTATTTTCTCTTT
TGCAGGTATTCCTCCTCTTGTAGGGTTCTTTGCTAAACAGATGGTATTAAGCGCGGCTATTGATC
AAGGTTATATCTTTTTATCTTTAGTTGCAATATTAACTAGTGTTATAGGAGGGGTTTATTATTTAAA
TATAATTAAAGAAATGTTCTTTTATTCACCTGACTATAAATTAAACGAAGAAATTAAAAATAATAC
TATTAATGGTCAAATTATTAATAGAAACAATAAAATATTAAATGTTGAATTTAATTATACAAATGT
AGTTATGTCTAGTTCTGTGGCAATAACTATTTCTACTATTACATTAGTAGTTTTATTATTCATGTTT
ATGAATAAAGAATGATTAAGTCTGGGTACTATATTGGTACAATCTTTATTTAGCTATTAATGAGTA
GTATGACATTATTTATAGGGTTTGTATCTGTTATAGCTATTTTATTTTTAGCCATTAATTTTATATTT
GCTCCTCATAATCCTTATCAAGAAAAATATAGTATTTTCGAGTGTGGTTTCCATAGTTTTTTAGGG
CAAAATAGAACACAATTCGGTATAAAATTCTTTATTTTTGCTTTAGTTTATTTACTTTTAGATTTAG

AAATATTATTAACTTTCCCTTTCGCTCTTAGTGAGTATGTTAATGGTATTTATGGTCTTTTAGTTAC
TTTAATTTTTATAGCTATAATAACTATAGGATTTATATTTGAATTAGGTAAAAGCGCTCTTAAAATA
GACAGCAGACAAAAATTATATATACCTAAATTGAACGTTAATTACCATACAGAGTATGTTGGAAT
AGGTAAGGTTTCTAAGTAAAGTTATAGAGGCAGAAAACCAAAAAACCTACCAAAGGGTAGCTA
ATGGGAAGCTATTAAAAATAAAAGATGATAACCTATATATAGTATAGTTACTATATATTTACTATA
ACTAGGATTATTATATATATATATATTATCTTATTGTATATTAAGATTATTATTATTATAAGGTATA
ATTAATATAGTATCTTATTGTATAAGAATATAATATATTAACCTATAATTAATTTTATTTTTTAATTT
TTAATTATAATTTTTTTTTATATCTAGATGCTTACACATCTACAGATGTAGAAGAGAACAAAATAT
TGTTGTTATAGTAGTGGATGATATAGAAAAATATTTTTATATTATTTATTTTTGTTAGGTAGCTTTT
GAAGTGTTTGATAGAGAGGATATATGGACGGTAGGAGGGTATTCATTTTAATGAACAGTGGATA
GTTTAAATTAACCTAGTTATAGTTTATGAATTTAAATTATAATTATATTAATGTAGGTTATGATAGA
ATTATGTATTGATCCTTAAGAGTTAAGAGAGATACGCCCACGTATAATACATATTGGTTTAGGGT
TGAGTATATATACTTAAGAGTTAAGAATATATATATACAATATATAATAAATATAGTAATATATTA
ATGGTATGGACTTAACCAGGTTTATATATTATCATTTGATAAACATTAATTATAATTTTTCTATTTA
TTATTATTATTATACTGTGAGATTAATAATTATTAAAATATAATATTACATAGTACGTGATATTTGG
GGATTTTATCTTATTAATTGGCAATTAATGATTCTAATCAAATTTTATTCTCTTTAGTTTAATGGTA
GAACAATGATCTTCTAATTCATTGGTTTTAGTTCGATTCTAAAAAGAGATGAGTAAATAATTTTCT
AGATCAGAAATACTACTTTTAACTACAAAAAGCTTACGCTTTTTAACATTTTTTTGATAAATAACA
ACTATTGTTAATATTTGGCTGTCTATTGGTTTAACTAACAATTACAAAATTTTTTCAATTTTTATAAT
ATAAATTTAAATGAGAATATTAAAAAGTCATTCATTATTAAAATTAGTGAATTCTTACCTTATCGA
TGCGTCACAACCAAGTAACATTAGTTACTTGTGAAATTTTGGTTCATTATTAGCTGTTTGTTTAAT
AGTACAAATTATTACCGGTATTACATTAGCTATGCATTATAGTCCTAGTGTAATGGAAGCTTTTAA
CTCAATAGAGCATATAATGAGAGATGTTAATAACGGGTGATTAGTTCGTTATCTACATAGTAATA
CAGCTTCTGCTTTCTTTTTCTTAGTGTATTTACACATAGGAAGAGGTATATATTACGGATCATATA
GAGCTCCTCGTACTTTAGTTTGAGCTATTGGTACTGTTATATTAATATTAATGATGGCTATCGGTT
TCCTAGGTTATGTTTTACCTTATGGACAGATGTCATTATGAGGTGCTACAGTTATTACTAATCTTA
TTAGTGCTATACCTTGAATAGGGCAAGATATTGTTGAATTCATTTGAGGTGGTTTTTCTGTTAATA
ATGCCACTTTAAACAGATTTTTTGCATTACATTTTGTATTGCCTTTTGTATTAGCTGCTTTAGTTTTA
ATGCACTTAATTGCACTTCATGATACTGCTGGTTCAAGCAATCCTCTTGGTGTTTCAGGTAATTAC
GATAGAATTACATTTGCTCCATATTTTTTATTTAAAGATTTAATTACTATTTTTATATTTATTTTTGTA
TTAAGTGCTTTTGTATTCTTTATGCCTAATGTTTTAGGGGATAGTGATAATTATATTATGGCTAATC
CTATGCAAACTCCTGCTGCTATTGTACCTGAATGATACTTATTACCTTTCTATGCTATTTTAAGATC
TATACCTAATAAATTATTAGGTGTTATAGCGATGTTTAGTGCTATTTTAGCTATTATGTTATTACCT
GTTACAGATTTAGGTAGATCTAGAGGTTTACAATTTAGACCATTTAGTAAAATAGCTTTCTGAGTT
TTTGTTGCTAATTTCTTAGTTTTAATGCAATTAGGTGCTAAACACGTTGAAGATCCATTTATATTAT
TAGGTCAATTAAGTACTGTATTATACTTTAGTTATTTTGTTGCTATATTACCTTTAGCTAGTTACTT
AGATAATAGTTTAACTGATTTATCTAATAAATCTGAATTATTTTTAAATAAAACTAACTAAATATAT
TAAGATTATTATTTAATATATTTTCTATTTAAGATACTATTAATTTAGTATTTTGGGTTTTCAGTTTA
TAATTTATATTATATTATGCATTACCCTCCACCTTGCTTTGTAGTAAGCTAATCTGTTATTTCCTTTA
GTTTAATGGTAGAACAATGATCTTCTAATTCATTGGTTTTAGTTCGAATCTAAAAAGGAAATAAG
AAATATATTCTTATTATTACTTATATAATAATTATTTCTTAAAAATATACATTTTGCATTATAGCCGT
TTAGCTGTATTAAAATGTAAAATGATATAAAATAGAATAAATATTTAAATTATTCCTATGTTATATT
ATCCTATATTGCAACCATTATCAGAAGTTGTATTAATACTTGTACCTGCCTTATTAGCTGTAGCTT
ATGTTACAGTTGCTGAAAGAAAAACTATGGCTAGTATGCAAAGAAGATTAGGTCCTAATGCTGT

AGGTTACTATGGACTATTGCAAGCATTTGCTGATGCCTTAAAACTTTTATTAAAAGAATATGTAG
CTCCTACACAATCTAATATTGTTCTTTTCTTTTTAGGTCCTGTAATAACTTTAATTTTTGCATTATTA
GGTTACGCTGTTATACCCTATGGTCCTGGTTCAGGGATAAGCGACATGAATTTAGGTATATTTTA
CATGTTAGCTGTGTCATCTTTAGCTACATACGGTATTCTATTAGCTGGTTGAAGTGCGAATAGTA
AATACGCTTTTCTAGGTTCTCTTAGAAGTACAGCTCAATTAATTAGTTATGAATTAATATTAAGTT
CAGCTATATTAATAGTAATTATGATAACAGGAAATTTAAATTTAACTGTTTGTACTGAATCTCAAA
GAGCTATTTGATTTATACTACCTTTATTTCCTGTGTTTATAATATTTTTCATAGGATCTATAGCTGA
GACAAATAGAGCTCCTTTTGATTTAGCCGAGGCTAACCTGCTAATCTGGTTTGGTCTGGTTATAT
GTCACAAATTGCTAGGAAACCTTTTTATTTTAAAAACAAAAGACAATTAGCAGGAAACTTAATTT
AACCTAATTAAAAATATTAGATAATTAAACTCTTCATAGACTAAACGTGACAATTTAATATATATA
TATATTTATTTATATATATGATTAAATAAGATATAGTCAATCATCGGTGTGAATCGACTTAAAAAA
AAAAGCACATGGGTTAAACCCATCTCCCCTTATTAGGGGAATCAGAACTTGTTAGTGGGTTCAT
GACAGAGCATGCTGCCGTAGTTTTCGTATTCTTCTTTTTAGCTGAGTACGGTAGTATTGTACTAAT
GTGTATTTTAACTAGTATATTATTTATTGGTGGTTACTTATTATTTGAAATATCCTATGTTTTTACTG
TGGTAAATTATATTTTCTTTGAATTATTCTTTATAGACTGAGTAACATTTGTAGAGGTACAATCTTT
ATACACTGATTTTTTAAATAATTCTATCATTGAAGGATTATTATATGGGTTTAATCTAGGATTAAA
AAGTTCTTTAATGATATTCACATTTATTTGAGCTAGAGCATCCTTCCCTAGAATACGATTTGATCA
ACTAATGGGCTTCTGTTGAACAGTTTTATTACCTATTAATTTTGCAATTATTATATTAGTACCTTGT
GTTTTATATAGTTTTAACTTATTACCTGTAAATATACCATTGTTCTAGCTCACACACCCGCCCGCC
CTACTGCCACAAGGCTACAGTACATATGAGGAGGGGAACTAAAGATCTAGAACTATCCTAGTTA
ATAATTACACTTAATAGTATACTAAATAGACCATCTATCATACTCGAGAATAGTGATAGTGTAAT
TATACACTATTTTAACTGTATTCTACACTATTAGGATATTATTATCTTTATATGATAATAATTTTATA
ACTTAATTTATTAGGTTACATTAACAAAAAAATTCGTTAACTTTTTTCACCACTTTTTAATACAAAAT
ACGAATTTTA
>YN391
ATAACAATTCTAAAGAACATAAAGAGTTATTAGATAAAAATAATTCACCTATACAGTTAATAAAT
CAACTTAAAGGGTATTTTTTCATAAATCCTTTATTAGCTTTAAGTTTAGCTATTACTATTTTCTCTTT
TGCAGGTATTCCTCCTCTTGTAGGGTTCTTTGCTAAACAGATGGTATTAAGCGCGGCTATTGATC
AAGGTTATATCTTTTTATCTTTAGTTGCAATATTAACTAGTGTTATAGGAGGGGTTTATTATTTAAA
TATAATTAAAGAAATGTTCTTTTATTCACCTGACTATAAATTAAACGAAGAAATTAAAAATAATAC
TATTAATGGTCAAATTATTAATAGAAACAATAAAATATTAAATGTTGAATTTAATTATACAAATGT
AGTTATGTCTAGTTCTGTGGCAATAACTATTTCTACTATTACATTAGTAGTTTTATTATTCATGTTT
ATGAATAAAGAATGATTAAGTCTGGGTACTATATTGGTACAATCTTTATTTAGCTATTAATGAGTA
GTATGACATTATTTATAGGGTTTGTATCTGTTATAGCTATTTTATTTTTAGCCATTAATTTTATATTT
GCTCCTCATAATCCTTATCAAGAAAAATATAGTATTTTCGAGTGTGGTTTCCATAGTTTTTTAGGG
CAAAATAGAACACAATTCGGTATAAAATTCTTTATTTTTGCTTTAGTTTATTTACTTTTAGATTTAG
AAATATTATTAACTTTCCCTTTCGCTCTTAGTGAGTATGTTAATGGTATTTATGGTCTTTTAGTTAC
TTTAATTTTTATAGCTATAATAACTATAGGATTTATATTTGAATTAGGTAAAAGCGCTCTTAAAATA
GACAGCAGACAAAAATTATATATACCTAAATTGAACGTTAATTACCATACAGAGTATGTTGGAAT
AGGTAAGGTTTCTAAGTAAAGTTATAGAGGCAGAAAACCAAAAAACCTACCAAAGGGTAGCTA
ATGGGAAGCTATTAAAAATAAAAGATGATAACCTATATATAGTATAGTTACTATATATTTACTATA
ACTAGGATTATTATATATATATATATATTATCTTATTGTATATTAAGATTATTATTATTATAAGGTATA
ATTAATATAGTATCTTATTGTATAAGAATATAATATATTAACCTATAATTAATTTTATTTTTTAATTT
TTAATTATAATTTTTTTTTATATCTAGATGCTTACACATCTACAGATGTAGAAGAGAACAAAATAT

TGTTGTTATAGTAGTGGATGATATAGAAAAATATTTTTATATTATTTATTTTTGTTAGGTAGCTTTT
GAAGTGTTTGATAGAGAGGATATATGGACGGTAGGAGGGTATTCATTTTAATGAACAGTGGATA
GTTTAAATTAACCTAGTTATAGTTTATGAATTTAAATTATAATTATATTAATGTAGGTTATGATAGA
ATTATGTATTGATCCTTAAGAGTTAAGAGAGATACGCCCACGTATAATACATATTGGTTTAGGGT
TGAGTATATATACTTAAGAGTTAAGAATATATATATACAATATATAATAAATATAGTAATATATTA
ATGGTATGGACTTAACCAGGTTTATATATTATCATTTGATAAACATTAATTATAATTTTTCTATTTA
TTATTATTATTATACTGTGAGATTAATAATTATTAAAATATAATATTACATAGTACGTGATATTTGG
GGATTTTATCTTATTAATTGGCAATTAATGATTCTAATCAAATTTTATTCTCTTTAGTTTAATGGTA
GAACAATGATCTTCTAATTCATTGGTTTTAGTTCGATTCTAAAAAGAGATGAGTAAATAATTTTCT
AGATCAGAAATACTACTTTTAACTACAAAAAGCTTACGCTTTTTAACATTTTTTTGATAAATAACA
ACTATTGTTAATATTTGGCTGTCTATTGGTTTAACTAACAATTACAAAATTTTTTCAATTTTTATAAT
ATAAATTTAAATGAGAATATTAAAAAGTCATTCATTATTAAAATTAGTGAATTCTTACCTTATCGA
TGCGTCACAACCAAGTAACATTAGTTACTTGTGAAATTTTGGTTCATTATTAGCTGTTTGTTTAAT
AGTACAAATTATTACCGGTATTACATTAGCTATGCATTATAGTCCTAGTGTAATGGAAGCTTTTAA
CTCAATAGAGCATATAATGAGAGATGTTAATAACGGGTGATTAGTTCGTTATCTACATAGTAATA
CAGCTTCTGCTTTCTTTTTCTTAGTGTATTTACACATAGGAAGAGGTATATATTACGGATCATATA
GAGCTCCTCGTACTTTAGTTTGAGCTATTGGTACTGTTATATTAATATTAATGATGGCTATCGGTT
TCCTAGGTTATGTTTTACCTTATGGACAGATGTCATTATGAGGTGCTACAGTTATTACTAATCTTA
TTAGTGCTATACCTTGAATAGGGCAAGATATTGTTGAATTCATTTGAGGTGGTTTTTCTGTTAATA
ATGCCACTTTAAACAGATTTTTTGCATTACATTTTGTATTGCCTTTTGTATTAGCTGCTTTAGTTTTA
ATGCACTTAATTGCACTTCATGATACTGCTGGTTCAAGCAATCCTCTTGGTGTTTCAGGTAATTAC
GATAGAATTACATTTGCTCCATATTTTTTATTTAAAGATTTAATTACTATTTTTATATTTATTTTTGTA
TTAAGTGCTTTTGTATTCTTTATGCCTAATGTTTTAGGGGATAGTGATAATTATATTATGGCTAATC
CTATGCAAACTCCTGCTGCTATTGTACCTGAATGATACTTATTACCTTTCTATGCTATTTTAAGATC
TATACCTAATAAATTATTAGGTGTTATAGCGATGTTTAGTGCTATTTTAGCTATTATGTTATTACCT
GTTACAGATTTAGGTAGATCTAGAGGTTTACAATTTAGACCATTTAGTAAAATAGCTTTCTGAGTT
TTTGTTGCTAATTTCTTAGTTTTAATGCAATTAGGTGCTAAACACGTTGAAGATCCATTTATATTAT
TAGGTCAATTAAGTACTGTATTATACTTTAGTTATTTTGTTGCTATATTACCTTTAGCTAGTTACTT
AGATAATAGTTTAACTGATTTATCTAATAAATCTGAATTATTTTTAAATAAAACTAACTAAATATAT
TAAGATTATTATTTAATATATTTTCTATTTAAGATACTATTAATTTAGTATTTTGGGTTTTCAGTTTA
TAATTTATATTATATTATGCATTACCCTCCACCTTGCTTTGTAGTAAGCTAATCTGTTATTTCCTTTA
GTTTAATGGTAGAACAATGATCTTCTAATTCATTGGTTTTAGTTCGAATCTAAAAAGGAAATAAG
AAATATATTCTTATTATTACTTATATAATAATTATTTCTTAAAAATATACATTTTGCATTATAGCCGT
TTAGCTGTATTAAAATGTAAAATGATATAAAATAGAATAAATATTTAAATTATTCCTATGTTATATT
ATCCTATATTGCAACCATTATCAGAAGTTGTATTAATACTTGTACCTGCCTTATTAGCTGTAGCTT
ATGTTACAGTTGCTGAAAGAAAAACTATGGCTAGTATGCAAAGAAGATTAGGTCCTAATGCTGT
AGGTTACTATGGACTATTGCAAGCATTTGCTGATGCCTTAAAACTTTTATTAAAAGAATATGTAG
CTCCTACACAATCTAATATTGTTCTTTTCTTTTTAGGTCCTGTAATAACTTTAATTTTTGCATTATTA
GGTTACGCTGTTATACCCTATGGTCCTGGTTCAGGGATAAGCGACATGAATTTAGGTATATTTTA
CATGTTAGCTGTGTCATCTTTAGCTACATACGGTATTCTATTAGCTGGTTGAAGTGCGAATAGTA
AATACGCTTTTCTAGGTTCTCTTAGAAGTACAGCTCAATTAATTAGTTATGAATTAATATTAAGTT
CAGCTATATTAATAGTAATTATGATAACAGGAAATTTAAATTTAACTGTTTGTACTGAATCTCAAA
GAGCTATTTGATTTATACTACCTTTATTTCCTGTGTTTATAATATTTTTCATAGGATCTATAGCTGA
GACAAATAGAGCTCCTTTTGATTTAGCCGAGGCTAACCTGCTAATCTGGTTTGGTCTGGTTATAT

GTCACAAATTGCTAGGAAACCTTTTTATTTTAAAAACAAAAGACAATTAGCAGGAAACTTAATTT
AACCTAATTAAAAATATTAGATAATTAAACTCTTCATAGACTAAACGTGACAATTTAATATATATA
TATATTTATTTATATATATGATTAAATAAGATATAGTCAATCATCGGTGTGAATCGACTTAAAAAA
AAAAGCACATGGGTTAAACCCATCTCCCCTTATTAGGGGAATCAGAACTTGTTAGTGGGTTCAT
GACAGAGCATGCTGCCGTAGTTTTCGTATTCTTCTTTTTAGCTGAGTACGGTAGTATTGTACTAAT
GTGTATTTTAACTAGTATATTATTTATTGGTGGTTACTTATTATTTGAAATATCCTATGTTTTTACTG
TGGTAAATTATATTTTCTTTGAATTATTCTTTATAGACTGAGTAACATTTGTAGAGGTACAATCTTT
ATACACTGATTTTTTAAATAATTCTATCATTGAAGGATTATTATATGGGTTTAATCTAGGATTAAA
AAGTTCTTTAATGATATTCACATTTATTTGAGCTAGAGCATCCTTCCCTAGAATACGATTTGATCA
ACTAATGGGCTTCTGTTGAACAGTTTTATTACCTATTAATTTTGCAATTATTATATTAGTACCTTGT
GTTTTATATAGTTTTAACTTATTACCTGTAAATATACCATTGTTCTAGCTCACACACCCGCCCGCC
CTACTGCCACAAGGCTACAGTACATATGAGGAGGGGAACTAAAGATCTAGAACTATCCTAGTTA
ATAATTACACTTAATAGTATACTAAATAGACCATCTATCATACTCGAGAATAGTGATAGTGTAAT
TATACACTATTTTAACTGTATTCTACACTATTAGCATATTATTATCTTTATATGATAATAATTTTATA
ACTTAATTTATTAGGTTACATTAACAAAAAATTCGTTAACTTTTTTCACCACTTTTTAATACAAAAT
ACGAATTTTA
>YN397
ATAACAATTCTAAAGAACATAAAGAGTTATTAGATAAAAATAATTCACCTATACAGTTAATAAAT
CAACTTAAAGGGTATTTTTTCATAAATCCTTTATTAGCTTTAAGTTTAGCTATTACTATTTTCTCTTT
TGCAGGTATTCCTCCTCTTGTAGGGTTCTTTGCTAAACAGATGGTATTAAGCGCGGCTATTGATC
AAGGTTATATCTTTTTATCTTTAGTTGCAATATTAACTAGTGTTATAGGAGGGGTTTATTATTTAAA
TATAATTAAAGAAATGTTCTTTTATTCACCTGACTATAAATTAAACGAAGAAATTAAAAATAATAC
TATTAATGGTCAAATTATTAATAGAAACAATAAAATATTAAATGTTGAATTTAATTATACAAATGT
AGTTATGTCTAGTTCTGTGGCAATAACTATTTCTACTATTACATTAGTAGTTTTATTATTCATGTTT
ATGAATAAAGAATGATTAAGTCTGGGTACTATATTGGTACAATCTTTATTTAGCTATTAATGAGTA
GTATGACATTATTTATAGGGTTTGTATCTGTTATAGCTATTTTATTTTTAGCCATTAATTTTATATTT
GCTCCTCATAATCCTTATCAAGAAAAATATAGTATTTTCGAGTGTGGTTTCCATAGTTTTTTAGGG
CAAAATAGAACACAATTCGGTATAAAATTCTTTATTTTTGCTTTAGTTTATTTACTTTTAGATTTAG
AAATATTATTAACTTTCCCTTTCGCTCTTAGTGAGTATGTTAATGGTATTTATGGTCTTTTAGTTAC
TTTAATTTTTATAGCTATAATAACTATAGGATTTATATTTGAATTAGGTAAAAGCGCTCTTAAAATA
GACAGCAGACAAAAATTATATATACCTAAATTGAACGTTAATTACCATACAGAGTATGTTGGAAT
AGGTAAGGTTTCTAAGTAAAGTTATAGAGGCAGAAAACCAAAAAACCTACCAAAGGGTAGCTA
ATGGGAAGCTATTAAAAATAAAAGATGATAACCTATATATAGTATAGTTACTATATATTTACTATA
ACTAGGATTATTATATATATATATATATTATCTTATTGTATATTAAGATTATTATTATTATAAGGTATA
ATTAATATAGTATCTTATTGTATAAGAATATAATATATTAACCTATAATTAATTTTATTTTTTAATTT
TTAATTATAATTTTTTTTTATATCTAGATGCTTACACATCTACAGATGTAGAAGAGAACAAAATAT
TGTTGTTATAGTAGTGGATGATATAGAAAAATATTTTTATATTATTTATTTTTGTTAGGTAGCTTTT
GAAGTGTTTGATAGAGAGGATATATGGACGGTAGGAGGGTATTCATTTTAATGAACAGTGGATA
GTTTAAATTAACCTAGTTATAGTTTATGAATTTAAATTATAATTATATTAATGTAGGTTATGATAGA
ATTATGTATTGATCCTTAAGAGTTAAGAGAGATACGCCCACGTATAATACATATTGGTTTAGGGT
TGAGTATATATACTTAAGAGTTAAGAATATATATATACAATATATAATAAATATAGTAATATATTA
ATGGTATGGACTTAACCAGGTTTATATATTATCATTTGATAAACATTAATTATAATTTTTCTATTTA
TTATTATTATTATACTGTGAGATTAATAATTATTAAAATATAATATTACATAGTACGTGATATTTGG
GGATTTTATCTTATTAATTGGCAATTAATGATTCTAATCAAATTTTATTCTCTTTAGTTTAATGGTA

GAACAATGATCTTCTAATTCATTGGTTTTAGTTCGATTCTAAAAAGAGATGAGTAAATAATTTTCT
AGATCAGAAATACTACTTTTAACTACAAAAAGCTTACGCTTTTTAACATTTTTTTGATAAATAACA
ACTATTGTTAATATTTGGCTGTCTATTGGTTTAACTAACAATTACAAAATTTTTTCAATTTTTATAAT
ATAAATTTAAATGAGAATATTAAAAAGTCATTCATTATTAAAATTAGTGAATTCTTACCTTATCGA
TGCGTCACAACCAAGTAACATTAGTTACTTGTGAAATTTTGGTTCATTATTAGCTGTTTGTTTAAT
AGTACAAATTATTACCGGTATTACATTAGCTATGCATTATAGTCCTAGTGTAATGGAAGCTTTTAA
CTCAATAGAGCATATAATGAGAGATGTTAATAACGGGTGATTAGTTCGTTATCTACATAGTAATA
CAGCTTCTGCTTTCTTTTTCTTAGTGTATTTACACATAGGAAGAGGTATATATTACGGATCATATA
GAGCTCCTCGTACTTTAGTTTGAGCTATTGGTACTGTTATATTAATATTAATGATGGCTATCGGTT
TCCTAGGTTATGTTTTACCTTATGGACAGATGTCATTATGAGGTGCTACAGTTATTACTAATCTTA
TTAGTGCTATACCTTGAATAGGGCAAGATATTGTTGAATTCATTTGAGGTGGTTTTTCTGTTAATA
ATGCCACTTTAAACAGATTTTTTGCATTACATTTTGTATTGCCTTTTGTATTAGCTGCTTTAGTTTTA
ATGCACTTAATTGCACTTCATGATACTGCTGGTTCAAGCAATCCTCTTGGTGTTTCAGGTAATTAC
GATAGAATTACATTTGCTCCATATTTTTTATTTAAAGATTTAATTACTATTTTTATATTTATTTTTGTA
TTAAGTGCTTTTGTATTCTTTATGCCTAATGTTTTAGGGGATAGTGATAATTATATTATGGCTAATC
CTATGCAAACTCCTGCTGCTATTGTACCTGAATGATACTTATTACCTTTCTATGCTATTTTAAGATC
TATACCTAATAAATTATTAGGTGTTATAGCGATGTTTAGTGCTATTTTAGCTATTATGTTATTACCT
GTTACAGATTTAGGTAGATCTAGAGGTTTACAATTTAGACCATTTAGTAAAATAGCTTTCTGAGTT
TTTGTTGCTAATTTCTTAGTTTTAATGCAATTAGGTGCTAAACACGTTGAAGATCCATTTATATTAT
TAGGTCAATTAAGTACTGTATTATACTTTAGTTATTTTGTTGCTATATTACCTTTAGCTAGTTACTT
AGATAATAGTTTAACTGATTTATCTAATAAATCTGAATTATTTTTAAATAAAACTAACTAAATATAT
TAAGATTATTATTTAATATATTTTCTATTTAAGATACTATTAATTTAGTATTTTGGGTTTTCAGTTTA
TAATTTATATTATATTATGCATTACCCTCCACCTTGCTTTGTAGTAAGCTAATCTGTTATTTCCTTTA
GTTTAATGGTAGAACAATGATCTTCTAATTCATTGGTTTTAGTTCGAATCTAAAAAGGAAATAAG
AAATATATTCTTATTATTACTTATATAATAATTATTTCTTAAAAATATACATTTTGCATTATAGCCGT
TTAGCTGTATTAAAATGTAAAATGATATAAAATAGAATAAATATTTAAATTATTCCTATGTTATATT
ATCCTATATTGCAACCATTATCAGAAGTTGTATTAATACTTGTACCTGCCTTATTAGCTGTAGCTT
ATGTTACAGTTGCTGAAAGAAAAACTATGGCTAGTATGCAAAGAAGATTAGGTCCTAATGCTGT
AGGTTACTATGGACTATTGCAAGCATTTGCTGATGCCTTAAAACTTTTATTAAAAGAATATGTAG
CTCCTACACAATCTAATATTGTTCTTTTCTTTTTAGGTCCTGTAATAACTTTAATTTTTGCATTATTA
GGTTACGCTGTTATACCCTATGGTCCTGGTTCAGGGATAAGCGACATGAATTTAGGTATATTTTA
CATGTTAGCTGTGTCATCTTTAGCTACATACGGTATTCTATTAGCTGGTTGAAGTGCGAATAGTA
AATACGCTTTTCTAGGTTCTCTTAGAAGTACAGCTCAATTAATTAGTTATGAATTAATATTAAGTT
CAGCTATATTAATAGTAATTATGATAACAGGAAATTTAAATTTAACTGTTTGTACTGAATCTCAAA
GAGCTATTTGATTTATACTACCTTTATTTCCTGTGTTTATAATATTTTTCATAGGATCTATAGCTGA
GACAAATAGAGCTCCTTTTGATTTAGCCGAGGCTAACCTGCTAATCTGGTTTGGTCTGGTTATAT
GTCACAAATTGCTAGGAAACCTTTTTATTTTAAAAACAAAAGACAATTAGCAGGAAACTTAATTT
AACCTAATTAAAAATATTAGATAATTAAACTCTTCATAGACTAAACGTGACAATTTAATATATATA
TATATTTATTTATATATATGATTAAATAAGATATAGTCAATCATCGGTGTGAATCGACTTAAAAAA
AAAAGCACATGGGTTAAACCCATCTCCCCTTATTAGGGGAATCAGAACTTGTTAGTGGGTTCAT
GACAGAGCATGCTGCCGTAGTTTTCGTATTCTTCTTTTTAGCTGAGTACGGTAGTATTGTACTAAT
GTGTATTTTAACTAGTATATTATTTATTGGTGGTTACTTATTATTTGAAATATCCTATGTTTTTACTG
TGGTAAATTATATTTTCTTTGAATTATTCTTTATAGACTGAGTAACATTTGTAGAGGTACAATCTTT
ATACACTGATTTTTTAAATAATTCTATCATTGAAGGATTATTATATGGGTTTAATCTAGGATTAAA

AAGTTCTTTAATGATATTCACATTTATTTGAGCTAGAGCATCCTTCCCTAGAATACGATTTGATCA
ACTAATGGGCTTCTGTTGAACAGTTTTATTACCTATTAATTTTGCAATTATTATATTAGTACCTTGT
GTTTTATATAGTTTTAACTTATTACCTGTAAATATACCATTGTTCTAGCTCACACACCCGCCCGCC
CTACTGCCACAAGGCTACAGTACATATGAGGAGGGGAACTAAAGATCTAGAACTATCCTAGTTA
ATAATTACACTTAATAGTATACTAAATAGACCATCTATCATACTCGAGAATAGTGATAGTGTAAT
TATACACTATTTTAACTGTATTCTACACTATTAGCATATTATTATCTTTATATGATAATAATTTTATA
ACTTAATTTATTAGGTTACATTAACAAAAAAATTCGTTAACTTTTTTCACCACTTTTTAATACAAAAT
ACGAATTTTA
>YN398
ATAACAATTCTAAAGAACATAAAGAGTTATTAGATAAAAATAATTCACCTATACAGTTAATAAAT
CAACTTAAAGGGTATTTTTTCATAAATCCTTTATTAGCTTTAAGTTTAGCTATTACTATTTTCTCTTT
TGCAGGTATTCCTCCTCTTGTAGGGTTCTTTGCTAAACAGATGGTATTAAGCGCGGCTATTGATC
AAGGTTATATCTTTTTATCTTTAGTTGCAATATTAACTAGTGTTATAGGAGGGGTTTATTATTTAAA
TATAATTAAAGAAATGTTCTTTTATTCACCTGACTATAAATTAAACGAAGAAATTAAAAATAATAC
TATTAATGGTCAAATTATTAATAGAAACAATAAAATATTAAATGTTGAATTTAATTATACAAATGT
AGTTATGTCTAGTTCTGTGGCAATAACTATTTCTACTATTACATTAGTAGTTTTATTATTCATGTTT
ATGAATAAAGAATGATTAAGTCTGGGTACTATATTGGTACAATCTTTATTTAGCTATTAATGAGTA
GTATGACATTATTTATAGGGTTTGTATCTGTTATAGCTATTTTATTTTTAGCCATTAATTTTATATTT
GCTCCTCATAATCCTTATCAAGAAAAATATAGTATTTTCGAGTGTGGTTTCCATAGTTTTTTAGGG
CAAAATAGAACACAATTCGGTATAAAATTCTTTATTTTTGCTTTAGTTTATTTACTTTTAGATTTAG
AAATATTATTAACTTTCCCTTTCGCTCTTAGTGAGTATGTTAATGGTATTTATGGTCTTTTAGTTAC
TTTAATTTTTATAGCTATAATAACTATAGGATTTATATTTGAATTAGGTAAAAGCGCTCTTAAAATA
GACAGCAGACAAAAATTATATATACCTAAATTGAACGTTAATTACCATACAGAGTATGTTGGAAT
AGGTAAGGTTTCTAAGTAAAGTTATAGAGGCAGAAAACCAAAAAACCTACCAAAGGGTAGCTA
ATGGGAAGCTATTAAAAATAAAAGATGATAACCTATATATAGTATAGTTACTATATATTTACTATA
ACTAGGATTATTATATATATATATATTATCTTATTGTATATTAAGATTATTATTATTATAAGGTATA
ATTAATATAGTATCTTATTGTATAAGAATATAATATATTAACCTATAATTAATTTTATTTTTTAATTT
TTAATTATAATTTTTTTTTATATCTAGATGCTTACACATCTACAGATGTAGAAGAGAACAAAATAT
TGTTGTTATAGTAGTGGATGATATAGAAAAATATTTTTATATTATTTATTTTTGTTAGGTAGCTTTT
GAAGTGTTTGATAGAGAGGATATATGGACGGTAGGAGGGTATTCATTTTAATGAACAGTGGATA
GTTTAAATTAACCTAGTTATAGTTTATGAATTTAAATTATAATTATATTAATGTAGGTTATGATAGA
ATTATGTATTGATCCTTAAGAGTTAAGAGAGATACGCCCACGTATAATACATATTGGTTTAGGGT
TGAGTATATATACTTAAGAGTTAAGAATATATATATACAATATATAATAAATATAGTAATATATTA
ATGGTATGGACTTAACCAGGTTTATATATTATCATTTGATAAACATTAATTATAATTTTTCTATTTA
TTATTATTATTATACTGTGAGATTAATAATTATTAAAATATAATATTACATAGTACGTGATATTTGG
GGATTTTATCTTATTAATTGGCAATTAATGATTCTAATCAAATTTTATTCTCTTTAGTTTAATGGTA
GAACAATGATCTTCTAATTCATTGGTTTTAGTTCGATTCTAAAAAGAGATGAGTAAATAATTTTCT
AGATCAGAAATACTACTTTTAACTACAAAAAGCTTACGCTTTTTAACATTTTTTTGATAAATAACA
ACTATTGTTAATATTTGGCTGTCTATTGGTTTAACTAACAATTACAAAATTTTTTCAATTTTTATAAT
ATAAATTTAAATGAGAATATTAAAAAGTCATTCATTATTAAAATTAGTGAATTCTTACCTTATCGA
TGCGTCACAACCAAGTAACATTAGTTACTTGTGAAATTTTGGTTCATTATTAGCTGTTTGTTTAAT
AGTACAAATTATTACCGGTATTACATTAGCTATGCATTATAGTCCTAGTGTAATGGAAGCTTTTAA
CTCAATAGAGCATATAATGAGAGATGTTAATAACGGGTGATTAGTTCGTTATCTACATAGTAATA
CAGCTTCTGCTTTCTTTTTCTTAGTGTATTTACACATAGGAAGAGGTATATATTACGGATCATATA

GAGCTCCTCGTACTTTAGTTTGAGCTATTGGTACTGTTATATTAATATTAATGATGGCTATCGGTT
TCCTAGGTTATGTTTTACCTTATGGACAGATGTCATTATGAGGTGCTACAGTTATTACTAATCTTA
TTAGTGCTATACCTTGAATAGGGCAAGATATTGTTGAATTCATTTGAGGTGGTTTTTCTGTTAATA
ATGCCACTTTAAACAGATTTTTTGCATTACATTTTGTATTGCCTTTTGTATTAGCTGCTTTAGTTTTA
ATGCACTTAATTGCACTTCATGATACTGCTGGTTCAAGCAATCCTCTTGGTGTTTCAGGTAATTAC
GATAGAATTACATTTGCTCCATATTTTTTATTTAAAGATTTAATTACTATTTTTATATTTATTTTTGTA
TTAAGTGCTTTTGTATTCTTTATGCCTAATGTTTTAGGGGATAGTGATAATTATATTATGGCTAATC
CTATGCAAACTCCTGCTGCTATTGTACCTGAATGATACTTATTACCTTTCTATGCTATTTTAAGATC
TATACCTAATAAATTATTAGGTGTTATAGCGATGTTTAGTGCTATTTTAGCTATTATGTTATTACCT
GTTACAGATTTAGGTAGATCTAGAGGTTTACAATTTAGACCATTTAGTAAAATAGCTTTCTGAGTT
TTTGTTGCTAATTTCTTAGTTTTAATGCAATTAGGTGCTAAACACGTTGAAGATCCATTTATATTAT
TAGGTCAATTAAGTACTGTATTATACTTTAGTTATTTTGTTGCTATATTACCTTTAGCTAGTTACTT
AGATAATAGTTTAACTGATTTATCTAATAAATCTGAATTATTTTTAAATAAAACTAACTAAATATAT
TAAGATTATTATTTAATATATTTTCTATTTAAGATACTATTAATTTAGTATTTTGGGTTTTCAGTTTA
TAATTTATATTATATTATGCATTACCCTCCACCTTGCTTTGTAGTAAGCTAATCTGTTATTTCCTTTA
GTTTAATGGTAGAACAATGATCTTCTAATTCATTGGTTTTAGTTCGAATCTAAAAAGGAAATAAG
AAATATATTCTTATTATTACTTATATAATAATTATTTCTTAAAAATATACATTTTGCATTATAGCCGT
TTAGCTGTATTAAAATGTAAAATGATATAAAATAGAATAAATATTTAAATTATTCCTATGTTATATT
ATCCTATATTGCAACCATTATCAGAAGTTGTATTAATACTTGTACCTGCCTTATTAGCTGTAGCTT
ATGTTACAGTTGCTGAAAGAAAAACTATGGCTAGTATGCAAAGAAGATTAGGTCCTAATGCTGT
AGGTTACTATGGACTATTGCAAGCATTTGCTGATGCCTTAAAACTTTTATTAAAAGAATATGTAG
CTCCTACACAATCTAATATTGTTCTTTTCTTTTTAGGTCCTGTAATAACTTTAATTTTTGCATTATTA
GGTTACGCTGTTATACCCTATGGTCCTGGTTCAGGGATAAGCGACATGAATTTAGGTATATTTTA
CATGTTAGCTGTGTCATCTTTAGCTACATACGGTATTCTATTAGCTGGTTGAAGTGCGAATAGTA
AATACGCTTTTCTAGGTTCTCTTAGAAGTACAGCTCAATTAATTAGTTATGAATTAATATTAAGTT
CAGCTATATTAATAGTAATTATGATAACAGGAAATTTAAATTTAACTGTTTGTACTGAATCTCAAA
GAGCTATTTGATTTATACTACCTTTATTTCCTGTGTTTATAATATTTTTCATAGGATCTATAGCTGA
GACAAATAGAGCTCCTTTTGATTTAGCCGAGGCTAACCTGCTAATCTGGTTTGGTCTGGTTATAT
GTCACAAATTGCTAGGAAACCTTTTTATTTTAAAAACAAAAGACAATTAGCAGGAAACTTAATTT
AACCTAATTAAAAATATTAGATAATTAAACTCTTCATAGACTAAACGTGACAATTTAATATATATA
TATATTTATTTATATATATGATTAAATAAGATATAGTCAATCATCGGTGTGAATCGACTTAAAAAA
AAAAGCACATGGGTTAAACCCATCTCCCCTTATTAGGGGAATCAGAACTTGTTAGTGGGTTCAT
GACAGAGCATGCTGCCGTAGTTTTCGTATTCTTCTTTTTAGCTGAGTACGGTAGTATTGTACTAAT
GTGTATTTTAACTAGTATATTATTTATTGGTGGTTACTTATTATTTGAAATATCCTATGTTTTTACTG
TGGTAAATTATATTTTCTTTGAATTATTCTTTATAGACTGAGTAACATTTGTAGAGGTACAATCTTT
ATACACTGATTTTTTAAATAATTCTATCATTGAAGGATTATTATATGGGTTTAATCTAGGATTAAA
AAGTTCTTTAATGATATTCACATTTATTTGAGCTAGAGCATCCTTCCCTAGAATACGATTTGATCA
ACTAATGGGCTTCTGTTGAACAGTTTTATTACCTATTAATTTTGCAATTATTATATTAGTACCTTGT
GTTTTATATAGTTTTAACTTATTACCTGTAAATATACCATTGTTCTAGCTCACACACCCGCCCGCC
CTACTGCCACAAGGCTACAGTACATATGAGGAGGGGAACTAAAGATCTAGAACTATCCTAGTTA
ATAATTACACTTAATAGTATACTAAATAGACCATCTATCATACTCGAGAATAGTGATAGTGTAAT
TATACACTATTTTAACTGTATTCTACACTATTAGCATATTATTATCTTTATATGATAATAATTTTATA
ACTTAATTTATTAGGTTACATTAACAAAAAATTCGTTAACTTTTTCACCACTTTTTAATACAAAAT
ACGAATTTTA

>YN406
ATAACAATTCTAAAGAACATAAAGAGTTATTAGATAAAAATAATTCACCTATACAGTTAATAAAT
CAACTTAAAGGGTATTTTTTCATAAATCCTTTATTAGCTTTAAGTTTAGCTATTACTATTTTCTCTTT
TGCAGGTATTCCTCCTCTTGTAGGGTTCTTTGCTAAACAGATGGTATTAAGCGCGGCTATTGATC
AAGGTTATATCTTTTTATCTTTAGTTGCAATATTAACTAGTGTTATAGGAGGGGTTTATTATTTAAA
TATAATTAAAGAAATGTTCTTTTATTCACCTGACTATAAATTAAACGAAGAAATTAAAAATAATAC
TATTAATGGTCAAATTATTAATAGAAACAATAAAATATTAAATGTTGAATTTAATTATACAAATGT
AGTTATGTCTAGTTCTGTGGCAATAACTATTTCTACTATTACATTAGTAGTTTTATTATTCATGTTT
ATGAATAAAGAATGATTAAGTCTGGGTACTATATTGGTACAATCTTTATTTAGCTATTAATGAGTA
GTATGACATTATTTATAGGGTTTGTATCTGTTATAGCTATTTTATTTTTAGCCATTAATTTTATATTT
GCTCCTCATAATCCTTATCAAGAAAAATATAGTATTTTCGAGTGTGGTTTCCATAGTTTTTTAGGG
CAAAATAGAACACAATTCGGTATAAAATTCTTTATTTTTGCTTTAGTTTATTTACTTTTAGATTTAG
AAATATTATTAACTTTCCCTTTCGCTCTTAGTGAGTATGTTAATGGTATTTATGGTCTTTTAGTTAC
TTTAATTTTTATAGCTATAATAACTATAGGATTTATATTTGAATTAGGTAAAAGCGCTCTTAAAATA
GACAGCAGACAAAAATTATATATACCTAAATTGAACGTTAATTACCATACAGAGTATGTTGGAAT
AGGTAAGGTTTCTAAGTAAAGTTATAGAGGCAGAAAACCAAAAAACCTACCAAAGGGTAGCTA
ATGGGAAGCTATTAAAAATAAAAGATGATAACCTATATATAGTATAGTTACTATATATTTACTATA
ACTAGGATTATTATATATATATATATTATCTTATTGTATATTAAGATTATTATTATTATAAGGTATA
ATTAATATAGTATCTTATTGTATAAGAATATAATATATTAACCTATAATTAATTTTATTTTTTAATTT
TTAATTATAATTTTTTTTTATATCTAGATGCTTACACATCTACAGATGTAGAAGAGAACAAAATAT
TGTTGTTATAGTAGTGGATGATATAGAAAAATATTTTTATATTATTTATTTTTGTTAGGTAGCTTTT
GAAGTGTTTGATAGAGAGGATATATGGACGGTAGGAGGGTATTCATTTTAATGAACAGTGGATA
GTTTAAATTAACCTAGTTATAGTTTATGAATTTAAATTATAATTATATTAATGTAGGTTATGATAGA
ATTATGTATTGATCCTTAAGAGTTAAGAGAGATACGCCCACGTATAATACATATTGGTTTAGGGT
TGAGTATATATACTTAAGAGTTAAGAATATATATATACAATATATAATAAATATAGTAATATATTA
ATGGTATGGACTTAACCAGGTTTATATATTATCATTTGATAAACATTAATTATAATTTTTCTATTTA
TTATTATTATTATACTGTGAGATTAATAATTATTAAAATATAATATTACATAGTACGTGATATTTGG
GGATTTTATCTTATTAATTGGCAATTAATGATTCTAATCAAATTTTATTCTCTTTAGTTTAATGGTA
GAACAATGATCTTCTAATTCATTGGTTTTAGTTCGATTCTAAAAAGAGATGAGTAAATAATTTTCT
AGATCAGAAATACTACTTTTAACTACAAAAAGCTTACGCTTTTTAACATTTTTTTGATAAATAACA
ACTATTGTTAATATTTGGCTGTCTATTGGTTTAACTAACAATTACAAAATTTTTTCAATTTTTATAAT
ATAAATTTAAATGAGAATATTAAAAAGTCATTCATTATTAAAATTAGTGAATTCTTACCTTATCGA
TGCGTCACAACCAAGTAACATTAGTTACTTGTGAAATTTTGGTTCATTATTAGCTGTTTGTTTAAT
AGTACAAATTATTACCGGTATTACATTAGCTATGCATTATAGTCCTAGTGTAATGGAAGCTTTTAA
CTCAATAGAGCATATAATGAGAGATGTTAATAACGGGTGATTAGTTCGTTATCTACATAGTAATA
CAGCTTCTGCTTTCTTTTTCTTAGTGTATTTACACATAGGAAGAGGTATATATTACGGATCATATA
GAGCTCCTCGTACTTTAGTTTGAGCTATTGGTACTGTTATATTAATATTAATGATGGCTATCGGTT
TCCTAGGTTATGTTTTACCTTATGGACAGATGTCATTATGAGGTGCTACAGTTATTACTAATCTTA
TTAGTGCTATACCTTGAATAGGGCAAGATATTGTTGAATTCATTTGAGGTGGTTTTTCTGTTAATA
ATGCCACTTTAAACAGATTTTTTGCATTACATTTTGTATTGCCTTTTGTATTAGCTGCTTTAGTTTTA
ATGCACTTAATTGCACTTCATGATACTGCTGGTTCAAGCAATCCTCTTGGTGTTTCAGGTAATTAC
GATAGAATTACATTTGCTCCATATTTTTTATTTAAAGATTTAATTACTATTTTTATATTTATTTTTGTA
TTAAGTGCTTTTGTATTCTTTATGCCTAATGTTTTAGGGGATAGTGATAATTATATTATGGCTAATC
CTATGCAAACTCCTGCTGCTATTGTACCTGAATGATACTTATTACCTTTCTATGCTATTTTAAGATC

TATACCTAATAAATTATTAGGTGTTATAGCGATGTTTAGTGCTATTTTAGCTATTATGTTATTACCT
GTTACAGATTTAGGTAGATCTAGAGGTTTACAATTTAGACCATTTAGTAAAATAGCTTTCTGAGTT
TTTGTTGCTAATTTCTTAGTTTTAATGCAATTAGGTGCTAAACACGTTGAAGATCCATTTATATTAT
TAGGTCAATTAAGTACTGTATTATACTTTAGTTATTTTGTTGCTATATTACCTTTAGCTAGTTACTT
AGATAATAGTTTAACTGATTTATCTAATAAATCTGAATTATTTTTAAATAAAACTAACTAAATATAT
TAAGATTATTATTTAATATATTTTCTATTTAAGATACTATTAATTTAGTATTTTGGGTTTTCAGTTTA
TAATTTATATTATATTATGCATTACCCTCCACCTTGCTTTGTAGTAAGCTAATCTGTTATTTCCTTTA
GTTTAATGGTAGAACAATGATCTTCTAATTCATTGGTTTTAGTTCGAATCTAAAAAGGAAATAAG
AAATATATTCTTATTATTACTTATATAATAATTATTTCTTAAAAATATACATTTTGCATTATAGCCGT
TTAGCTGTATTAAAATGTAAAATGATATAAAATAGAATAAATATTTAAATTATTCCTATGTTATATT
ATCCTATATTGCAACCATTATCAGAAGTTGTATTAATACTTGTACCTGCCTTATTAGCTGTAGCTT
ATGTTACAGTTGCTGAAAGAAAAACTATGGCTAGTATGCAAAGAAGATTAGGTCCTAATGCTGT
AGGTTACTATGGACTATTGCAAGCATTTGCTGATGCCTTAAAACTTTTATTAAAAGAATATGTAG
CTCCTACACAATCTAATATTGTTCTTTTCTTTTTAGGTCCTGTAATAACTTTAATTTTTGCATTATTA
GGTTACGCTGTTATACCCTATGGTCCTGGTTCAGGGATAAGCGACATGAATTTAGGTATATTTTA
CATGTTAGCTGTGTCATCTTTAGCTACATACGGTATTCTATTAGCTGGTTGAAGTGCGAATAGTA
AATACGCTTTTCTAGGTTCTCTTAGAAGTACAGCTCAATTAATTAGTTATGAATTAATATTAAGTT
CAGCTATATTAATAGTAATTATGATAACAGGAAATTTAAATTTAACTGTTTGTACTGAATCTCAAA
GAGCTATTTGATTTATACTACCTTTATTTCCTGTGTTTATAATATTTTTCATAGGATCTATAGCTGA
GACAAATAGAGCTCCTTTTGATTTAGCCGAGGCTAACCTGCTAATCTGGTTTGGTCTGGTTATAT
GTCACAAATTGCTAGGAAACCTTTTTATTTTAAAAACAAAAGACAATTAGCAGGAAACTTAATTT
AACCTAATTAAAAATATTAGATAATTAAACTCTTCATAGACTAAACGTGACAATTTAATATATATA
TATATTTATTTATATATATGATTAAATAAGATATAGTCAATCATCGGTGTGAATCGACTTAAAAAA
AAAAGCACATGGGTTAAACCCATCTCCCCTTATTAGGGGAATCAGAACTTGTTAGTGGGTTCAT
GACAGAGCATGCTGCCGTAGTTTTCGTATTCTTCTTTTTAGCTGAGTACGGTAGTATTGTACTAAT
GTGTATTTTAACTAGTATATTATTTATTGGTGGTTACTTATTATTTGAAATATCCTATGTTTTTACTG
TGGTAAATTATATTTTCTTTGAATTATTCTTTATAGACTGAGTAACATTTGTAGAGGTACAATCTTT
ATACACTGATTTTTTAAATAATTCTATCATTGAAGGATTATTATATGGGTTTAATCTAGGATTAAA
AAGTTCTTTAATGATATTCACATTTATTTGAGCTAGAGCATCCTTCCCTAGAATACGATTTGATCA
ACTAATGGGCTTCTGTTGAACAGTTTTATTACCTATTAATTTTGCAATTATTATATTAGTACCTTGT
GTTTTATATAGTTTTAACTTATTACCTGTAAATATACCATTGTTCTAGCTCACACACCCGCCCGCC
CTACTGCCACAAGGCTACAGTACATATGAGGAGGGGAACTAAAGATCTAGAACTATCCTAGTTA
ATAATTACACTTAATAGTATACTAAATAGACCATCTATCATACTCGAGAATAGTGATAGTGTAAT
TATACACTATTTTAACTGTATTCTACACTATTAGCATATTATTATCTTTATATGATAATAATTTTATA
ACTTAATTTATTAGGTTACATTAACAAAAAATTCGTTAACTTTTTTCACCACTTTTTAATACAAAAT
ACGAATTATA
>YN410
ATAACAATTCTAAAGAACATAAAGAGTTATTAGATAAAAATAATTCACCTATACAGTTAATAAAT
CAACTTAAAGGGTATTTTTTCATAAATCCTTTATTAGCTTTAAGTTTAGCTATTACTATTTTCTCTTT
TGCAGGTATTCCTCCTCTTGTAGGGTTCTTTGCTAAACAGATGGTATTAAGCGCGGCTATTGATC
AAGGTTATATCTTTTTATCTTTAGTTGCAATATTAACTAGTGTTATAGGAGGGGTTTATTATTTAAA
TATAATTAAAGAAATGTTCTTTTATTCACCTGACTATAAATTAAACGAAGAAATTAAAAATAATAC
TATTAATGGTCAAATTATTAATAGAAACAATAAAATATTAAATGTTGAATTTAATTATACAAATGT
AGTTATGTCTAGTTCTGTGGCAATAACTATTTCTACTATTACATTAGTAGTTTTATTATTCATGTTT

ATGAATAAAGAATGATTAAGTCTGGGTACTATATTGGTACAATCTTTATTTAGCTATTAATGAGTA
GTATGACATTATTTATAGGGTTTGTATCTGTTATAGCTATTTTATTTTTAGCCATTAATTTTATATTT
GCTCCTCATAATCCTTATCAAGAAAAATATAGTATTTTCGAGTGTGGTTTCCATAGTTTTTTAGGG
CAAAATAGAACACAATTCGGTATAAAATTCTTTATTTTTGCTTTAGTTTATTTACTTTTAGATTTAG
AAATATTATTAACTTTCCCTTTCGCTCTTAGTGAGTATGTTAATGGTATTTATGGTCTTTTAGTTAC
TTTAATTTTTATAGCTATAATAACTATAGGATTTATATTTGAATTAGGTAAAAGCGCTCTTAAAATA
GACAGCAGACAAAAATTATATATACCTAAATTGAACGTTAATTACCATACAGAGTATGTTGGAAT
AGGTAAGGTTTCTAAGTAAAGTTATAGAGGCAGAAAACCAAAAAACCTACCAAAGGGTAGCTA
ATGGGAAGCTATTAAAAATAAAAGATGATAACCTATATATAGTATAGTTACTATATATTTACTATA
ACTAGGATTATTATATATATATATATATTATCTTATTGTATATTAAGATTATTATTATTATAAGGTATA
ATTAATATAGTATCTTATTGTATAAGAATATAATATATTAACCTATAATTAATTTTATTTTTTAATTT
TTAATTATAATTTTTTTTTATATCTAGATGCTTACACATCTACAGATGTAGAAGAGAACAAAATAT
TGTTGTTATAGTAGTGGATGATATAGAAAAATATTTTTATATTATTTATTTTTGTTAGGTAGCTTTT
GAAGTGTTTGATAGAGAGGATATATGGACGGTAGGAGGGTATTCATTTTAATGAACAGTGGATA
GTTTAAATTAACCTAGTTATAGTTTATGAATTTAAATTATAATTATATTAATGTAGGTTATGATAGA
ATTATGTATTGATCCTTAAGAGTTAAGAGAGATACGCCCACGTATAATACATATTGGTTTAGGGT
TGAGTATATATACTTAAGAGTTAAGAATATATATATACAATATATAATAAATATAGTAATATATTA
ATGGTATGGACTTAACCAGGTTTATATATTATCATTTGATAAACATTAATTATAATTTTTCTATTTA
TTATTATTATTATACTGTGAGATTAATAATTATTAAAATATAATATTACATAGTACGTGATATTTGG
GGATTTTATCTTATTAATTGGCAATTAATGATTCTAATCAAATTTTATTCTCTTTAGTTTAATGGTA
GAACAATGATCTTCTAATTCATTGGTTTTAGTTCGATTCTAAAAAGAGATGAGTAAATAATTTTCT
AGATCAGAAATACTACTTTTAACTACAAAAAGCTTACGCTTTTTAACATTTTTTTGATAAATAACA
ACTATTGTTAATATTTGGCTGTCTATTGGTTTAACTAACAATTACAAAATTTTTTCAATTTTTATAAT
ATAAATTTAAATGAGAATATTAAAAAGTCATTCATTATTAAAATTAGTGAATTCTTACCTTATCGA
TGCGTCACAACCAAGTAACATTAGTTACTTGTGAAATTTTGGTTCATTATTAGCTGTTTGTTTAAT
AGTACAAATTATTACCGGTATTACATTAGCTATGCATTATAGTCCTAGTGTAATGGAAGCTTTTAA
CTCAATAGAGCATATAATGAGAGATGTTAATAACGGGTGATTAGTTCGTTATCTACATAGTAATA
CAGCTTCTGCTTTCTTTTTCTTAGTGTATTTACACATAGGAAGAGGTATATATTACGGATCATATA
GAGCTCCTCGTACTTTAGTTTGAGCTATTGGTACTGTTATATTAATATTAATGATGGCTATCGGTT
TCCTAGGTTATGTTTTACCTTATGGACAGATGTCATTATGAGGTGCTACAGTTATTACTAATCTTA
TTAGTGCTATACCTTGAATAGGGCAAGATATTGTTGAATTCATTTGAGGTGGTTTTTCTGTTAATA
ATGCCACTTTAAACAGATTTTTTGCATTACATTTTGTATTGCCTTTTGTATTAGCTGCTTTAGTTTTA
ATGCACTTAATTGCACTTCATGATACTGCTGGTTCAAGCAATCCTCTTGGTGTTTCAGGTAATTAC
GATAGAATTACATTTGCTCCATATTTTTTATTTAAAGATTTAATTACTATTTTTATATTTATTTTTGTA
TTAAGTGCTTTTGTATTCTTTATGCCTAATGTTTTAGGGGATAGTGATAATTATATTATGGCTAATC
CTATGCAAACTCCTGCTGCTATTGTACCTGAATGATACTTATTACCTTTCTATGCTATTTTAAGATC
TATACCTAATAAATTATTAGGTGTTATAGCGATGTTTAGTGCTATTTTAGCTATTATGTTATTACCT
GTTACAGATTTAGGTAGATCTAGAGGTTTACAATTTAGACCATTTAGTAAAATAGCTTTCTGAGTT
TTTGTTGCTAATTTCTTAGTTTTAATGCAATTAGGTGCTAAACACGTTGAAGATCCATTTATATTAT
TAGGTCAATTAAGTACTGTATTATACTTTAGTTATTTTGTTGCTATATTACCTTTAGCTAGTTACTT
AGATAATAGTTTAACTGATTTATCTAATAAATCTGAATTATTTTTAAATAAAACTAACTAAATATAT
TAAGATTATTATTTAATATATTTTCTATTTAAGATACTATTAATTTAGTATTTTGGGTTTTCAGTTTA
TAATTTATATTATATTATGCATTACCCTCCACCTTGCTTTGTAGTAAGCTAATCTGTTATTTCCTTTA
GTTTAATGGTAGAACAATGATCTTCTAATTCATTGGTTTTAGTTCGAATCTAAAAAGGAAATAAG

AAATATATTCTTATTATTACTTATATAATAATTATTTCTTAAAAATATACATTTTGCATTATAGCCGT
TTAGCTGTATTAAAATGTAAAATGATATAAAATAGAATAAATATTTAAATTATTCCTATGTTATATT
ATCCTATATTGCAACCATTATCAGAAGTTGTATTAATACTTGTACCTGCCTTATTAGCTGTAGCTT
ATGTTACAGTTGCTGAAAGAAAAACTATGGCTAGTATGCAAAGAAGATTAGGTCCTAATGCTGT
AGGTTACTATGGACTATTGCAAGCATTTGCTGATGCCTTAAAACTTTTATTAAAAGAATATGTAG
CTCCTACACAATCTAATATTGTTCTTTTCTTTTTAGGTCCTGTAATAACTTTAATTTTTGCATTATTA
GGTTACGCTGTTATACCCTATGGTCCTGGTTCAGGGATAAGCGACATGAATTTAGGTATATTTTA
CATGTTAGCTGTGTCATCTTTAGCTACATACGGTATTCTATTAGCTGGTTGAAGTGCGAATAGTA
AATACGCTTTTCTAGGTTCTCTTAGAAGTACAGCTCAATTAATTAGTTATGAATTAATATTAAGTT
CAGCTATATTAATAGTAATTATGATAACAGGAAATTTAAATTTAACTGTTTGTACTGAATCTCAAA
GAGCTATTTGATTTATACTACCTTTATTTCCTGTGTTTATAATATTTTTCATAGGATCTATAGCTGA
GACAAATAGAGCTCCTTTTGATTTAGCCGAGGCTAACCTGCTAATCTGGTTTGGTCTGGTTATAT
GTCACAAATTGCTAGGAAACCTTTTTATTTTAAAAACAAAAGACAATTAGCAGGAAACTTAATTT
AACCTAATTAAAAATATTAGATAATTAAACTCTTCATAGACTAAACGTGACAATTTAATATATATA
TATATTTATTTATATATATGATTAAATAAGATATAGTCAATCATCGGTGTGAATCGACTTAAAAAA
AAAAGCACATGGGTTAAACCCATCTCCCCTTATTAGGGGAATCAGAACTTGTTAGTGGGTTCAT
GACAGAGCATGCTGCCGTAGTTTTCGTATTCTTCTTTTTAGCTGAGTACGGTAGTATTGTACTAAT
GTGTATTTTAACTAGTATATTATTTATTGGTGGTTACTTATTATTTGAAATATCCTATGTTTTTACTG
TGGTAAATTATATTTTCTTTGAATTATTCTTTATAGACTGAGTAACATTTGTAGAGGTACAATCTTT
ATACACTGATTTTTTAAATAATTCTATCATTGAAGGATTATTATATGGGTTTAATCTAGGATTAAA
AAGTTCTTTAATGATATTCACATTTATTTGAGCTAGAGCATCCTTCCCTAGAATACGATTTGATCA
ACTAATGGGCTTCTGTTGAACAGTTTTATTACCTATTAATTTTGCAATTATTATATTAGTACCTTGT
GTTTTATATAGTTTTAACTTATTACCTGTAAATATACCATTGTTCTAGCTCACACACCCGCCCGCC
CTACTGCCACAAGGCTACAGTACATATGAGGAGGGGAACTAAAGATCTAGAACTATCCTAGTTA
ATAATTACACTTAATAGTATACTAAATAGACCATCTATCATACTCGAGAATAGTGATAGTGTAAT
TATACACTATTTTAACTGTATTCTACACTATTAGCATATTATTATCTTTATATGATAATAATTTTATA
ACTTAATTTATTAGGTTACATTAACAAAAAATTCGTTAACTTTTTTCACCACTTTTTAATACAAAAT
ACGAATTTTA
>YN427
ATAACAATTCTAAAGAACATAAAGAGTTATTAGATAAAAATAATTCACCTATACAGTTAATAAAT
CAACTTAAAGGGTATTTTTTCATAAATCCTTTATTAGCTTTAAGTTTAGCTATTACTATTTTCTCTTT
TGCAGGTATTCCTCCTCTTGTAGGGTTCTTTGCTAAACAGATGGTATTAAGCGCGGCTATTGATC
AAGGTTATATCTTTTTATCTTTAGTTGCAATATTAACTAGTGTTATAGGAGGGGTTTATTATTTAAA
TATAATTAAAGAAATGTTCTTTTATTCACCTGACTATAAATTAAACGAAGAAATTAAAAATAATAC
TATTAATGGTCAAATTATTAATAGAAACAATAAAATATTAAATGTTGAATTTAATTATACAAATGT
AGTTATGTCTAGTTCTGTGGCAATAACTATTTCTACTATTACATTAGTAGTTTTATTATTCATGTTT
ATGAATAAAGAATGATTAAGTCTGGGTACTATATTGGTACAATCTTTATTTAGCTATTAATGAGTA
GTATGACATTATTTATAGGGTTTGTATCTGTTATAGCTATTTTATTTTTAGCCATTAATTTTATATTT
GCTCCTCATAATCCTTATCAAGAAAAATATAGTATTTTCGAGTGTGGTTTCCATAGTTTTTTAGGG
CAAAATAGAACACAATTCGGTATAAAATTCTTTATTTTTGCTTTAGTTTATTTACTTTTAGATTTAG
AAATATTATTAACTTTCCCTTTCGCTCTTAGTGAGTATGTTAATGGTATTTATGGTCTTTTAGTTAC
TTTAATTTTTATAGCTATAATAACTATAGGATTTATATTTGAATTAGGTAAAAGCGCTCTTAAAATA
GACAGCAGACAAAAATTATATATACCTAAATTGAACGTTAATTACCATACAGAGTATGTTGGAAT
AGGTAAGGTTTCTAAGTAAAGTTATAGAGGCAGAAAACCAAAAAACCTACCAAAGGGTAGCTA

ATGGGAAGCTATTAAAAATAAAAGATGATAACCTATATATAGTATAGTTACTATATATTTACTATA
ACTAGGATTATTATATATATATATATTATCTTATTGTATATTAAGATTATTATTATTATAAGGTATA
ATTAATATAGTATCTTATTGTATAAGAATATAATATATTAACCTATAATTAATTTTATTTTTTAATTT
TTAATTATAATTTTTTTTTATATCTAGATGCTTACACATCTACAGATGTAGAAGAGAACAAAATAT
TGTTGTTATAGTAGTGGATGATATAGAAAAATATTTTTATATTATTTATTTTTGTTAGGTAGCTTTT
GAAGTGTTTGATAGAGAGGATATATGGACGGTAGGAGGGTATTCATTTTAATGAACAGTGGATA
GTTTAAATTAACCTAGTTATAGTTTATGAATTTAAATTATAATTATATTAATGTAGGTTATGATAGA
ATTATGTATTGATCCTTAAGAGTTAAGAGAGATACGCCCACGTATAATACATATTGGTTTAGGGT
TGAGTATATATACTTAAGAGTTAAGAATATATATATACAATATATAATAAATATAGTAATATATTA
ATGGTATGGACTTAACCAGGTTTATATATTATCATTTGATAAACATTAATTATAATTTTTCTATTTA
TTATTATTATTATACTGTGAGATTAATAATTATTAAAATATAATATTACATAGTACGTGATATTTGG
GGATTTTATCTTATTAATTGGCAATTAATGATTCTAATCAAATTTTATTCTCTTTAGTTTAATGGTA
GAACAATGATCTTCTAATTCATTGGTTTTAGTTCGATTCTAAAAAGAGATGAGTAAATAATTTTCT
AGATCAGAAATACTACTTTTAACTACAAAAAGCTTACGCTTTTTAACATTTTTTTGATAAATAACA
ACTATTGTTAATATTTGGCTGTCTATTGGTTTAACTAACAATTACAAAATTTTTTCAATTTTTATAAT
ATAAATTTAAATGAGAATATTAAAAAGTCATTCATTATTAAAATTAGTGAATTCTTACCTTATCGA
TGCGTCACAACCAAGTAACATTAGTTACTTGTGAAATTTTGGTTCATTATTAGCTGTTTGTTTAAT
AGTACAAATTATTACCGGTATTACATTAGCTATGCATTATAGTCCTAGTGTAATGGAAGCTTTTAA
CTCAATAGAGCATATAATGAGAGATGTTAATAACGGGTGATTAGTTCGTTATCTACATAGTAATA
CAGCTTCTGCTTTCTTTTTCTTAGTGTATTTACACATAGGAAGAGGTATATATTACGGATCATATA
GAGCTCCTCGTACTTTAGTTTGAGCTATTGGTACTGTTATATTAATATTAATGATGGCTATCGGTT
TCCTAGGTTATGTTTTACCTTATGGACAGATGTCATTATGAGGTGCTACAGTTATTACTAATCTTA
TTAGTGCTATACCTTGAATAGGGCAAGATATTGTTGAATTCATTTGAGGTGGTTTTTCTGTTAATA
ATGCCACTTTAAACAGATTTTTTGCATTACATTTTGTATTGCCTTTTGTATTAGCTGCTTTAGTTTTA
ATGCACTTAATTGCACTTCATGATACTGCTGGTTCAAGCAATCCTCTTGGTGTTTCAGGTAATTAC
GATAGAATTACATTTGCTCCATATTTTTTATTTAAAGATTTAATTACTATTTTTATATTTATTTTTGTA
TTAAGTGCTTTTGTATTCTTTATGCCTAATGTTTTAGGGGATAGTGATAATTATATTATGGCTAATC
CTATGCAAACTCCTGCTGCTATTGTACCTGAATGATACTTATTACCTTTCTATGCTATTTTAAGATC
TATACCTAATAAATTATTAGGTGTTATAGCGATGTTTAGTGCTATTTTAGCTATTATGTTATTACCT
GTTACAGATTTAGGTAGATCTAGAGGTTTACAATTTAGACCATTTAGTAAAATAGCTTTCTGAGTT
TTTGTTGCTAATTTCTTAGTTTTAATGCAATTAGGTGCTAAACACGTTGAAGATCCATTTATATTAT
TAGGTCAATTAAGTACTGTATTATACTTTAGTTATTTTGTTGCTATATTACCTTTAGCTAGTTACTT
AGATAATAGTTTAACTGATTTATCTAATAAATCTGAATTATTTTTAAATAAAACTAACTAAATATAT
TAAGATTATTATTTAATATATTTTCTATTTAAGATACTATTAATTTAGTATTTTGGGTTTTCAGTTTA
TAATTTATATTATATTATGCATTACCCTCCACCTTGCTTTGTAGTAAGCTAATCTGTTATTTCCTTTA
GTTTAATGGTAGAACAATGATCTTCTAATTCATTGGTTTTAGTTCGAATCTAAAAAGGAAATAAG
AAATATATTCTTATTATTACTTATATAATAATTATTTCTTAAAAATATACATTTTGCATTATAGCCGT
TTAGCTGTATTAAAATGTAAAATGATATAAAATAGAATAAATATTTAAATTATTCCTATGTTATATT
ATCCTATATTGCAACCATTATCAGAAGTTGTATTAATACTTGTACCTGCCTTATTAGCTGTAGCTT
ATGTTACAGTTGCTGAAAGAAAAACTATGGCTAGTATGCAAAGAAGATTAGGTCCTAATGCTGT
AGGTTACTATGGACTATTGCAAGCATTTGCTGATGCCTTAAAACTTTTATTAAAAGAATATGTAG
CTCCTACACAATCTAATATTGTTCTTTTCTTTTTAGGTCCTGTAATAACTTTAATTTTTGCATTATTA
GGTTACGCTGTTATACCCTATGGTCCTGGTTCAGGGATAAGCGACATGAATTTAGGTATATTTTA
CATGTTAGCTGTGTCATCTTTAGCTACATACGGTATTCTATTAGCTGGTTGAAGTGCGAATAGTA

AATACGCTTTTCTAGGTTCTCTTAGAAGTACAGCTCAATTAATTAGTTATGAATTAATATTAAGTT
CAGCTATATTAATAGTAATTATGATAACAGGAAATTTAAATTTAACTGTTTGTACTGAATCTCAAA
GAGCTATTTGATTTATACTACCTTTATTTCCTGTGTTTATAATATTTTTCATAGGATCTATAGCTGA
GACAAATAGAGCTCCTTTTGATTTAGCCGAGGCTAACCTGCTAATCTGGTTTGGTCTGGTTATAT
GTCACAAATTGCTAGGAAACCTTTTTATTTTAAAAACAAAAGACAATTAGCAGGAAACTTAATTT
AACCTAATTAAAAATATTAGATAATTAAACTCTTCATAGACTAAACGTGACAATTTAATATATATA
TATATTTATTTATATATATGATTAAATAAGATATAGTCAATCATCGGTGTGAATCGACTTAAAAAA
AAAAGCACATGGGTTAAACCCATCTCCCCTTATTAGGGGAATCAGAACTTGTTAGTGGGTTCAT
GACAGAGCATGCTGCCGTAGTTTTCGTATTCTTCTTTTTAGCTGAGTACGGTAGTATTGTACTAAT
GTGTATTTTAACTAGTATATTATTTATTGGTGGTTACTTATTATTTGAAATATCCTATGTTTTTACTG
TGGTAAATTATATTTTCTTTGAATTATTCTTTATAGACTGAGTAACATTTGTAGAGGTACAATCTTT
ATACACTGATTTTTTAAATAATTCTATCATTGAAGGATTATTATATGGGTTTAATCTAGGATTAAA
AAGTTCTTTAATGATATTCACATTTATTTGAGCTAGAGCATCCTTCCCTAGAATACGATTTGATCA
ACTAATGGGCTTCTGTTGAACAGTTTTATTACCTATTAATTTTGCAATTATTATATTAGTACCTTGT
GTTTTATATAGTTTTAACTTATTACCTGTAAATATACCATTGTTCTAGCTCACACACCCGCCCGCC
CTACTGCCACAAGGCTACAGTACATATGAGGAGGGGAACTAAAGATCTAGAACTATCCTAGTTA
ATAATTACACTTAATAGTATACTAAATAGACCATCTATCATACTCGAGAATAGTGATAGTGTAAT
TATACACTATTTTAACTGTATTCTACACTATTAGCATATTATTATCTTTATATGATAATAATTTTATA
ACTTAATTTATTAGGTTACATTAACAAAAAAATTCGTTAACTTTTTTCACCACTTTTTAATACAAAAT
ACGAATTTTA
>YN435
ATAACAATTCTAAAGAACATAAAGAGTTATTAGATAAAAATAATTCACCTATACAGTTAATAAAT
CAACTTAAAGGGTATTTTTTCATAAATCCTTTATTAGCTTTAAGTTTAGCTATTACTATTTTCTCTTT
TGCAGGTATTCCTCCTCTTGTAGGGTTCTTTGCTAAACAGATGGTATTAAGCGCGGCTATTGATC
AAGGTTATATCTTTTTATCTTTAGTTGCAATATTAACTAGTGTTATAGGAGGGGTTTATTATTTAAA
TATAATTAAAGAAATGTTCTTTTATTCACCTGACTATAAATTAAACGAAGAAATTAAAAATAATAC
TATTAATGGTCAAATTATTAATAGAAACAATAAAATATTAAATGTTGAATTTAATTATACAAATGT
AGTTATGTCTAGTTCTGTGGCAATAACTATTTCTACTATTACATTAGTAGTTTTATTATTCATGTTT
ATGAATAAAGAATGATTAAGTCTGGGTACTATATTGGTACAATCTTTATTTAGCTATTAATGAGTA
GTATGACATTATTTATAGGGTTTGTATCTGTTATAGCTATTTTATTTTTAGCCATTAATTTTATATTT
GCTCCTCATAATCCTTATCAAGAAAAATATAGTATTTTCGAGTGTGGTTTCCATAGTTTTTTAGGG
CAAAATAGAACACAATTCGGTATAAAATTCTTTATTTTTGCTTTAGTTTATTTACTTTTAGATTTAG
AAATATTATTAACTTTCCCTTTCGCTCTTAGTGAGTATGTTAATGGTATTTATGGTCTTTTAGTTAC
TTTAATTTTTATAGCTATAATAACTATAGGATTTATATTTGAATTAGGTAAAAGCGCTCTTAAAATA
GACAGCAGACAAAAATTATATATACCTAAATTGAACGTTAATTACCATACAGAGTATGTTGGAAT
AGGTAAGGTTTCTAAGTAAAGTTATAGAGGCAGAAAACCAAAAAACCTACCAAAGGGTAGCTA
ATGGGAAGCTATTAAAAATAAAAGATGATAACCTATATATAGTATAGTTACTATATATTTACTATA
ACTAGGATTATTATATATATATATATATTATCTTATTGTATATTAAGATTATTATTATTATAAGGTATA
ATTAATATAGTATCTTATTGTATAAGAATATAATATATTAACCTATAATTAATTTTATTTTTTAATTT
TTAATTATAATTTTTTTTTATATCTAGATGCTTACACATCTACAGATGTAGAAGAGAACAAAATAT
TGTTGTTATAGTAGTGGATGATATAGAAAAATATTTTTATATTATTTATTTTTGTTAGGTAGCTTTT
GAAGTGTTTGATAGAGAGGATATATGGACGGTAGGAGGGTATTCATTTTAATGAACAGTGGATA
GTTTAAATTAACCTAGTTATAGTTTATGAATTTAAATTATAATTATATTAATGTAGGTTATGATAGA
ATTATGTATTGATCCTTAAGAGTTAAGAGAGATACGCCCACGTATAATACATATTGGTTTAGGGT

TGAGTATATATACTTAAGAGTTAAGAATATATATATACAATATATAATAAATATAGTAATATATTA
ATGGTATGGACTTAACCAGGTTTATATATTATCATTTGATAAACATTAATTATAATTTTTCTATTTA
TTATTATTATTATACTGTGAGATTAATAATTATTAAAATATAATATTACATAGTACGTGATATTTGG
GGATTTTATCTTATTAATTGGCAATTAATGATTCTAATCAAATTTTATTCTCTTTAGTTTAATGGTA
GAACAATGATCTTCTAATTCATTGGTTTTAGTTCGATTCTAAAAAGAGATGAGTAAATAATTTTCT
AGATCAGAAATACTACTTTTAACTACAAAAAGCTTACGCTTTTTAACATTTTTTTGATAAATAACA
ACTATTGTTAATATTTGGCTGTCTATTGGTTTAACTAACAATTACAAAATTTTTTCAATTTTTATAAT
ATAAATTTAAATGAGAATATTAAAAAGTCATTCATTATTAAAATTAGTGAATTCTTACCTTATCGA
TGCGTCACAACCAAGTAACATTAGTTACTTGTGAAATTTTGGTTCATTATTAGCTGTTTGTTTAAT
AGTACAAATTATTACCGGTATTACATTAGCTATGCATTATAGTCCTAGTGTAATGGAAGCTTTTAA
CTCAATAGAGCATATAATGAGAGATGTTAATAACGGGTGATTAGTTCGTTATCTACATAGTAATA
CAGCTTCTGCTTTCTTTTTCTTAGTGTATTTACACATAGGAAGAGGTATATATTACGGATCATATA
GAGCTCCTCGTACTTTAGTTTGAGCTATTGGTACTGTTATATTAATATTAATGATGGCTATCGGTT
TCCTAGGTTATGTTTTACCTTATGGACAGATGTCATTATGAGGTGCTACAGTTATTACTAATCTTA
TTAGTGCTATACCTTGAATAGGGCAAGATATTGTTGAATTCATTTGAGGTGGTTTTTCTGTTAATA
ATGCCACTTTAAACAGATTTTTTGCATTACATTTTGTATTGCCTTTTGTATTAGCTGCTTTAGTTTTA
ATGCACTTAATTGCACTTCATGATACTGCTGGTTCAAGCAATCCTCTTGGTGTTTCAGGTAATTAC
GATAGAATTACATTTGCTCCATATTTTTTATTTAAAGATTTAATTACTATTTTTATATTTATTTTTGTA
TTAAGTGCTTTTGTATTCTTTATGCCTAATGTTTTAGGGGATAGTGATAATTATATTATGGCTAATC
CTATGCAAACTCCTGCTGCTATTGTACCTGAATGATACTTATTACCTTTCTATGCTATTTTAAGATC
TATACCTAATAAATTATTAGGTGTTATAGCGATGTTTAGTGCTATTTTAGCTATTATGTTATTACCT
GTTACAGATTTAGGTAGATCTAGAGGTTTACAATTTAGACCATTTAGTAAAATAGCTTTCTGAGTT
TTTGTTGCTAATTTCTTAGTTTTAATGCAATTAGGTGCTAAACACGTTGAAGATCCATTTATATTAT
TAGGTCAATTAAGTACTGTATTATACTTTAGTTATTTTGTTGCTATATTACCTTTAGCTAGTTACTT
AGATAATAGTTTAACTGATTTATCTAATAAATCTGAATTATTTTTAAATAAAACTAACTAAATATAT
TAAGATTATTATTTAATATATTTTCTATTTAAGATACTATTAATTTAGTATTTTGGGTTTTCAGTTTA
TAATTTATATTATATTATGCATTACCCTCCACCTTGCTTTGTAGTAAGCTAATCTGTTATTTCCTTTA
GTTTAATGGTAGAACAATGATCTTCTAATTCATTGGTTTTAGTTCGAATCTAAAAAGGAAATAAG
AAATATATTCTTATTATTACTTATATAATAATTATTTCTTAAAAATATACATTTTGCATTATAGCCGT
TTAGCTGTATTAAAATGTAAAATGATATAAAATAGAATAAATATTTAAATTATTCCTATGTTATATT
ATCCTATATTGCAACCATTATCAGAAGTTGTATTAATACTTGTACCTGCCTTATTAGCTGTAGCTT
ATGTTACAGTTGCTGAAAGAAAAACTATGGCTAGTATGCAAAGAAGATTAGGTCCTAATGCTGT
AGGTTACTATGGACTATTGCAAGCATTTGCTGATGCCTTAAAACTTTTATTAAAAGAATATGTAG
CTCCTACACAATCTAATATTGTTCTTTTCTTTTTAGGTCCTGTAATAACTTTAATTTTTGCATTATTA
GGTTACGCTGTTATACCCTATGGTCCTGGTTCAGGGATAAGCGACATGAATTTAGGTATATTTTA
CATGTTAGCTGTGTCATCTTTAGCTACATACGGTATTCTATTAGCTGGTTGAAGTGCGAATAGTA
AATACGCTTTTCTAGGTTCTCTTAGAAGTACAGCTCAATTAATTAGTTATGAATTAATATTAAGTT
CAGCTATATTAATAGTAATTATGATAACAGGAAATTTAAATTTAACTGTTTGTACTGAATCTCAAA
GAGCTATTTGATTTATACTACCTTTATTTCCTGTGTTTATAATATTTTTCATAGGATCTATAGCTGA
GACAAATAGAGCTCCTTTTGATTTAGCCGAGGCTAACCTGCTAATCTGGTTTGGTCTGGTTATAT
GTCACAAATTGCTAGGAAACCTTTTTATTTTAAAAACAAAAGACAATTAGCAGGAAACTTAATTT
AACCTAATTAAAAATATTAGATAATTAAACTCTTCATAGACTAAACGTGACAATTTAATATATATA
TATATTTATTTATATATATGATTAAATAAGATATAGTCAATCATCGGTGTGAATCGACTTAAAAAA
AAAAGCACATGGGTTAAACCCATCTCCCCTTATTAGGGGAATCAGAACTTGTTAGTGGGTTCAT

GACAGAGCATGCTGCCGTAGTTTTCGTATTCTTCTTTTTAGCTGAGTACGGTAGTATTGTACTAAT
GTGTATTTTAACTAGTATATTATTTATTGGTGGTTACTTATTATTTGAAATATCCTATGTTTTTACTG
TGGTAAATTATATTTTCTTTGAATTATTCTTTATAGACTGAGTAACATTTGTAGAGGTACAATCTTT
ATACACTGATTTTTTAAATAATTCTATCATTGAAGGATTATTATATGGGTTTAATCTAGGATTAAA
AAGTTCTTTAATGATATTCACATTTATTTGAGCTAGAGCATCCTTCCCTAGAATACGATTTGATCA
ACTAATGGGCTTCTGTTGAACAGTTTTATTACCTATTAATTTTGCAATTATTATATTAGTACCTTGT
GTTTTATATAGTTTTAACTTATTACCTGTAAATATACCATTGTTCTAGCTCACACACCCGCCCGCC
CTACTGCCACAAGGCTACAGTACATATGAGGAGGGGAACTAAAGATCTAGAACTATCCTAGTTA
ATAATTACACTTAATAGTATACTAAATAGACCATCTATCATACTCGAGAATAGTGATAGTGTAAT
TATACACTATTTTAACTGTATTCTACACTATTAGCATATTATTATCTTTATATGATAATAATTTTATA
ACTTAATTTATTAGGTTACATTAACAAAAAATTCGTTAACTTTTTTCACCACTTTTTAATACAAAAT
ACGAATTTTA
>YN442
ATAACAATTCTAAAGAACATAAAGAGTTATTAGATAAAAATAATTCACCTATACAGTTAATAAAT
CAACTTAAAGGGTATTTTTTCATAAATCCTTTATTAGCTTTAAGTTTAGCTATTACTATTTTCTCTTT
TGCAGGTATTCCTCCTCTTGTAGGGTTCTTTGCTAAACAGATGGTATTAAGCGCGGCTATTGATC
AAGGTTATATCTTTTTATCTTTAGTTGCAATATTAACTAGTGTTATAGGAGGGGTTTATTATTTAAA
TATAATTAAAGAAATGTTCTTTTATTCACCTGACTATAAATTAAACGAAGAAATTAAAAATAATAC
TATTAATGGTCAAATTATTAATAGAAACAATAAAATATTAAATGTTGAATTTAATTATACAAATGT
AGTTATGTCTAGTTCTGTGGCAATAACTATTTCTACTATTACATTAGTAGTTTTATTATTCATGTTT
ATGAATAAAGAATGATTAAGTCTGGGTACTATATTGGTACAATCTTTATTTAGCTATTAATGAGTA
GTATGACATTATTTATAGGGTTTGTATCTGTTATAGCTATTTTATTTTTAGCCATTAATTTTATATTT
GCTCCTCATAATCCTTATCAAGAAAAATATAGTATTTTCGAGTGTGGTTTCCATAGTTTTTTAGGG
CAAAATAGAACACAATTCGGTATAAAATTCTTTATTTTTGCTTTAGTTTATTTACTTTTAGATTTAG
AAATATTATTAACTTTCCCTTTCGCTCTTAGTGAGTATGTTAATGGTATTTATGGTCTTTTAGTTAC
TTTAATTTTTATAGCTATAATAACTATAGGATTTATATTTGAATTAGGTAAAAGCGCTCTTAAAATA
GACAGCAGACAAAAATTATATATACCTAAATTGAACGTTAATTACCATACAGAGTATGTTGGAAT
AGGTAAGGTTTCTAAGTAAAGTTATAGAGGCAGAAAACCAAAAAACCTACCAAAGGGTAGCTA
ATGGGAAGCTATTAAAAATAAAAGATGATAACCTATATATAGTATAGTTACTATATATTTACTATA
ACTAGGATTATTATATATATATATATTATCTTATTGTATATTAAGATTATTATTATTATAAGGTATA
ATTAATATAGTATCTTATTGTATAAGAATATAATATATTAACCTATAATTAATTTTATTTTTTAATTT
TTAATTATAATTTTTTTTTATATCTAGATGCTTACACATCTACAGATGTAGAAGAGAACAAAATAT
TGTTGTTATAGTAGTGGATGATATAGAAAAATATTTTTATATTATTTATTTTTGTTAGGTAGCTTTT
GAAGTGTTTGATAGAGAGGATATATGGACGGTAGGAGGGTATTCATTTTAATGAACAGTGGATA
GTTTAAATTAACCTAGTTATAGTTTATGAATTTAAATTATAATTATATTAATGTAGGTTATGATAGA
ATTATGTATTGATCCTTAAGAGTTAAGAGAGATACGCCCACGTATAATACATATTGGTTTAGGGT
TGAGTATATATACTTAAGAGTTAAGAATATATATATACAATATATAATAAATATAGTAATATATTA
ATGGTATGGACTTAACCAGGTTTATATATTATCATTTGATAAACATTAATTATAATTTTTCTATTTA
TTATTATTATTATACTGTGAGATTAATAATTATTAAAATATAATATTACATAGTACGTGATATTTGG
GGATTTTATCTTATTAATTGGCAATTAATGATTCTAATCAAATTTTATTCTCTTTAGTTTAATGGTA
GAACAATGATCTTCTAATTCATTGGTTTTAGTTCGATTCTAAAAAGAGATGAGTAAATAATTTTCT
AGATCAGAAATACTACTTTTAACTACAAAAAGCTTACGCTTTTTAACATTTTTTTGATAAATAACA
ACTATTGTTAATATTTGGCTGTCTATTGGTTTAACTAACAATTACAAAATTTTTTCAATTTTTATAAT
ATAAATTTAAATGAGAATATTAAAAAGTCATTCATTATTAAAATTAGTGAATTCTTACCTTATCGA

TGCGTCACAACCAAGTAACATTAGTTACTTGTGAAATTTTGGTTCATTATTAGCTGTTTGTTTAAT
AGTACAAATTATTACCGGTATTACATTAGCTATGCATTATAGTCCTAGTGTAATGGAAGCTTTTAA
CTCAATAGAGCATATAATGAGAGATGTTAATAACGGGTGATTAGTTCGTTATCTACATAGTAATA
CAGCTTCTGCTTTCTTTTTCTTAGTGTATTTACACATAGGAAGAGGTATATATTACGGATCATATA
GAGCTCCTCGTACTTTAGTTTGAGCTATTGGTACTGTTATATTAATATTAATGATGGCTATCGGTT
TCCTAGGTTATGTTTTACCTTATGGACAGATGTCATTATGAGGTGCTACAGTTATTACTAATCTTA
TTAGTGCTATACCTTGAATAGGGCAAGATATTGTTGAATTCATTTGAGGTGGTTTTTCTGTTAATA
ATGCCACTTTAAACAGATTTTTTGCATTACATTTTGTATTGCCTTTTGTATTAGCTGCTTTAGTTTTA
ATGCACTTAATTGCACTTCATGATACTGCTGGTTCAAGCAATCCTCTTGGTGTTTCAGGTAATTAC
GATAGAATTACATTTGCTCCATATTTTTTATTTAAAGATTTAATTACTATTTTTATATTTATTTTTGTA
TTAAGTGCTTTTGTATTCTTTATGCCTAATGTTTTAGGGGATAGTGATAATTATATTATGGCTAATC
CTATGCAAACTCCTGCTGCTATTGTACCTGAATGATACTTATTACCTTTCTATGCTATTTTAAGATC
TATACCTAATAAATTATTAGGTGTTATAGCGATGTTTAGTGCTATTTTAGCTATTATGTTATTACCT
GTTACAGATTTAGGTAGATCTAGAGGTTTACAATTTAGACCATTTAGTAAAATAGCTTTCTGAGTT
TTTGTTGCTAATTTCTTAGTTTTAATGCAATTAGGTGCTAAACACGTTGAAGATCCATTTATATTAT
TAGGTCAATTAAGTACTGTATTATACTTTAGTTATTTTGTTGCTATATTACCTTTAGCTAGTTACTT
AGATAATAGTTTAACTGATTTATCTAATAAATCTGAATTATTTTTAAATAAAACTAACTAAATATAT
TAAGATTATTATTTAATATATTTTCTATTTAAGATACTATTAATTTAGTATTTTGGGTTTTCAGTTTA
TAATTTATATTATATTATGCATTACCCTCCACCTTGCTTTGTAGTAAGCTAATCTGTTATTTCCTTTA
GTTTAATGGTAGAACAATGATCTTCTAATTCATTGGTTTTAGTTCGAATCTAAAAAGGAAATAAG
AAATATATTCTTATTATTACTTATATAATAATTATTTCTTAAAAATATACATTTTGCATTATAGCCGT
TTAGCTGTATTAAAATGTAAAATGATATAAAATAGAATAAATATTTAAATTATTCCTATGTTATATT
ATCCTATATTGCAACCATTATCAGAAGTTGTATTAATACTTGTACCTGCCTTATTAGCTGTAGCTT
ATGTTACAGTTGCTGAAAGAAAAACTATGGCTAGTATGCAAAGAAGATTAGGTCCTAATGCTGT
AGGTTACTATGGACTATTGCAAGCATTTGCTGATGCCTTAAAACTTTTATTAAAAGAATATGTAG
CTCCTACACAATCTAATATTGTTCTTTTCTTTTTAGGTCCTGTAATAACTTTAATTTTTGCATTATTA
GGTTACGCTGTTATACCCTATGGTCCTGGTTCAGGGATAAGCGACATGAATTTAGGTATATTTTA
CATGTTAGCTGTGTCATCTTTAGCTACATACGGTATTCTATTAGCTGGTTGAAGTGCGAATAGTA
AATACGCTTTTCTAGGTTCTCTTAGAAGTACAGCTCAATTAATTAGTTATGAATTAATATTAAGTT
CAGCTATATTAATAGTAATTATGATAACAGGAAATTTAAATTTAACTGTTTGTACTGAATCTCAAA
GAGCTATTTGATTTATACTACCTTTATTTCCTGTGTTTATAATATTTTTCATAGGATCTATAGCTGA
GACAAATAGAGCTCCTTTTGATTTAGCCGAGGCTAACCTGCTAATCTGGTTTGGTCTGGTTATAT
GTCACAAATTGCTAGGAAACCTTTTTATTTTAAAAACAAAAGACAATTAGCAGGAAACTTAATTT
AACCTAATTAAAAATATTAGATAATTAAACTCTTCATAGACTAAACGTGACAATTTAATATATATA
TATATTTATTTATATATATGATTAAATAAGATATAGTCAATCATCGGTGTGAATCGACTTAAAAAA
AAAAGCACATGGGTTAAACCCATCTCCCCTTATTAGGGGAATCAGAACTTGTTAGTGGGTTCAT
GACAGAGCATGCTGCCGTAGTTTTCGTATTCTTCTTTTTAGCTGAGTACGGTAGTATTGTACTAAT
GTGTATTTTAACTAGTATATTATTTATTGGTGGTTACTTATTATTTGAAATATCCTATGTTTTTACTG
TGGTAAATTATATTTTCTTTGAATTATTCTTTATAGACTGAGTAACATTTGTAGAGGTACAATCTTT
ATACACTGATTTTTTAAATAATTCTATCATTGAAGGATTATTATATGGGTTTAATCTAGGATTAAA
AAGTTCTTTAATGATATTCACATTTATTTGAGCTAGAGCATCCTTCCCTAGAATACGATTTGATCA
ACTAATGGGCTTCTGTTGAACAGTTTTATTACCTATTAATTTTGCAATTATTATATTAGTACCTTGT
GTTTTATATAGTTTTAACTTATTACCTGTAAATATACCATTGTTCTAGCTCACACACCCGCCCGCC
CTACTGCCACAAGGCTACAGTACATATGAGGAGGGGAACTAAAGATCTAGAACTATCCTAGTTA

ATAATTACACTTAATAGTATACTAAATAGACCATCTATCATACTCGAGAATAGTGATAGTGTAAT
TATACACTATTTTAACTGTATTCTACACTATTAGCATATTATTATCTTTATATGATAATAATTTTATA
ACTTAATTTATTAGGTTACATTAACAAAAAATTCGTTAACTTTTTTCACCACTTTTTAATACAAAAT
ACGAATTTTA
>YN455
ATAACAATTCTAAAGAACATAAAGAGTTATTAGATAAAAATAATTCACCTATACAGTTAATAAAT
CAACTTAAAGGGTATTTTTTCATAAATCCTTTATTAGCTTTAAGTTTAGCTATTACTATTTTCTCTTT
TGCAGGTATTCCTCCTCTTGTAGGGTTCTTTGCTAAACAGATGGTATTAAGCGCGGCTATTGATC
AAGGTTATATCTTTTTATCTTTAGTTGCAATATTAACTAGTGTTATAGGAGGGGTTTATTATTTAAA
TATAATTAAAGAAATGTTCTTTTATTCACCTGACTATAAATTAAACGAAGAAATTAAAAATAATAC
TATTAATGGTCAAATTATTAATAGAAACAATAAAATATTAAATGTTGAATTTAATTATACAAATGT
AGTTATGTCTAGTTCTGTGGCAATAACTATTTCTACTATTACATTAGTAGTTTTATTATTCATGTTT
ATGAATAAAGAATGATTAAGTCTGGGTACTATATTGGTACAATCTTTATTTAGCTATTAATGAGTA
GTATGACATTATTTATAGGGTTTGTATCTGTTATAGCTATTTTATTTTTAGCCATTAATTTTATATTT
GCTCCTCATAATCCTTATCAAGAAAAATATAGTATTTTCGAGTGTGGTTTCCATAGTTTTTTAGGG
CAAAATAGAACACAATTCGGTATAAAATTCTTTATTTTTGCTTTAGTTTATTTACTTTTAGATTTAG
AAATATTATTAACTTTCCCTTTCGCTCTTAGTGAGTATGTTAATGGTATTTATGGTCTTTTAGTTAC
TTTAATTTTTATAGCTATAATAACTATAGGATTTATATTTGAATTAGGTAAAAGCGCTCTTAAAATA
GACAGCAGACAAAAATTATATATACCTAAATTGAACGTTAATTACCATACAGAGTATGTTGGAAT
AGGTAAGGTTTCTAAGTAAAGTTATAGAGGCAGAAAACCAAAAAACCTACCAAAGGGTAGCTA
ATGGGAAGCTATTAAAAATAAAAGATGATAACCTATATATAGTATAGTTACTATATATTTACTATA
ACTAGGATTATTATATATATATATATTATCTTATTGTATATTAAGATTATTATTATTATAAGGTATA
ATTAATATAGTATCTTATTGTATAAGAATATAATATATTAACCTATAATTAATTTTATTTTTTAATTT
TTAATTATAATTTTTTTTTATATCTAGATGCTTACACATCTACAGATGTAGAAGAGAACAAAATAT
TGTTGTTATAGTAGTGGATGATATAGAAAAATATTTTTATATTATTTATTTTTGTTAGGTAGCTTTT
GAAGTGTTTGATAGAGAGGATATATGGACGGTAGGAGGGTATTCATTTTAATGAACAGTGGATA
GTTTAAATTAACCTAGTTATAGTTTATGAATTTAAATTATAATTATATTAATGTAGGTTATGATAGA
ATTATGTATTGATCCTTAAGAGTTAAGAGAGATACGCCCACGTATAATACATATTGGTTTAGGGT
TGAGTATATATACTTAAGAGTTAAGAATATATATATACAATATATAATAAATATAGTAATATATTA
ATGGTATGGACTTAACCAGGTTTATATATTATCATTTGATAAACATTAATTATAATTTTTCTATTTA
TTATTATTATTATACTGTGAGATTAATAATTATTAAAATATAATATTACATAGTACGTGATATTTGG
GGATTTTATCTTATTAATTGGCAATTAATGATTCTAATCAAATTTTATTCTCTTTAGTTTAATGGTA
GAACAATGATCTTCTAATTCATTGGTTTTAGTTCGATTCTAAAAAGAGATGAGTAAATAATTTTCT
AGATCAGAAATACTACTTTTAACTACAAAAAGCTTACGCTTTTTAACATTTTTTTGATAAATAACA
ACTATTGTTAATATTTGGCTGTCTATTGGTTTAACTAACAATTACAAAATTTTTTCAATTTTTATAAT
ATAAATTTAAATGAGAATATTAAAAAGTCATTCATTATTAAAATTAGTGAATTCTTACCTTATCGA
TGCGTCACAACCAAGTAACATTAGTTACTTGTGAAATTTTGGTTCATTATTAGCTGTTTGTTTAAT
AGTACAAATTATTACCGGTATTACATTAGCTATGCATTATAGTCCTAGTGTAATGGAAGCTTTTAA
CTCAATAGAGCATATAATGAGAGATGTTAATAACGGGTGATTAGTTCGTTATCTACATAGTAATA
CAGCTTCTGCTTTCTTTTTCTTAGTGTATTTACACATAGGAAGAGGTATATATTACGGATCATATA
GAGCTCCTCGTACTTTAGTTTGAGCTATTGGTACTGTTATATTAATATTAATGATGGCTATCGGTT
TCCTAGGTTATGTTTTACCTTATGGACAGATGTCATTATGAGGTGCTACAGTTATTACTAATCTTA
TTAGTGCTATACCTTGAATAGGGCAAGATATTGTTGAATTCATTTGAGGTGGTTTTTCTGTTAATA
ATGCCACTTTAAACAGATTTTTTGCATTACATTTTGTATTGCCTTTTGTATTAGCTGCTTTAGTTTTA

ATGCACTTAATTGCACTTCATGATACTGCTGGTTCAAGCAATCCTCTTGGTGTTTCAGGTAATTAC
GATAGAATTACATTTGCTCCATATTTTTTATTTAAAGATTTAATTACTATTTTTATATTTATTTTTGTA
TTAAGTGCTTTTGTATTCTTTATGCCTAATGTTTTAGGGGATAGTGATAATTATATTATGGCTAATC
CTATGCAAACTCCTGCTGCTATTGTACCTGAATGATACTTATTACCTTTCTATGCTATTTTAAGATC
TATACCTAATAAATTATTAGGTGTTATAGCGATGTTTAGTGCTATTTTAGCTATTATGTTATTACCT
GTTACAGATTTAGGTAGATCTAGAGGTTTACAATTTAGACCATTTAGTAAAATAGCTTTCTGAGTT
TTTGTTGCTAATTTCTTAGTTTTAATGCAATTAGGTGCTAAACACGTTGAAGATCCATTTATATTAT
TAGGTCAATTAAGTACTGTATTATACTTTAGTTATTTTGTTGCTATATTACCTTTAGCTAGTTACTT
AGATAATAGTTTAACTGATTTATCTAATAAATCTGAATTATTTTTAAATAAAACTAACTAAATATAT
TAAGATTATTATTTAATATATTTTCTATTTAAGATACTATTAATTTAGTATTTTGGGTTTTCAGTTTA
TAATTTATATTATATTATGCATTACCCTCCACCTTGCTTTGTAGTAAGCTAATCTGTTATTTCCTTTA
GTTTAATGGTAGAACAATGATCTTCTAATTCATTGGTTTTAGTTCGAATCTAAAAAGGAAATAAG
AAATATATTCTTATTATTACTTATATAATAATTATTTCTTAAAAATATACATTTTGCATTATAGCCGT
TTAGCTGTATTAAAATGTAAAATGATATAAAATAGAATAAATATTTAAATTATTCCTATGTTATATT
ATCCTATATTGCAACCATTATCAGAAGTTGTATTAATACTTGTACCTGCCTTATTAGCTGTAGCTT
ATGTTACAGTTGCTGAAAGAAAAACTATGGCTAGTATGCAAAGAAGATTAGGTCCTAATGCTGT
AGGTTACTATGGACTATTGCAAGCATTTGCTGATGCCTTAAAACTTTTATTAAAAGAATATGTAG
CTCCTACACAATCTAATATTGTTCTTTTCTTTTTAGGTCCTGTAATAACTTTAATTTTTGCATTATTA
GGTTACGCTGTTATACCCTATGGTCCTGGTTCAGGGATAAGCGACATGAATTTAGGTATATTTTA
CATGTTAGCTGTGTCATCTTTAGCTACATACGGTATTCTATTAGCTGGTTGAAGTGCGAATAGTA
AATACGCTTTTCTAGGTTCTCTTAGAAGTACAGCTCAATTAATTAGTTATGAATTAATATTAAGTT
CAGCTATATTAATAGTAATTATGATAACAGGAAATTTAAATTTAACTGTTTGTACTGAATCTCAAA
GAGCTATTTGATTTATACTACCTTTATTTCCTGTGTTTATAATATTTTTCATAGGATCTATAGCTGA
GACAAATAGAGCTCCTTTTGATTTAGCCGAGGCTAACCTGCTAATCTGGTTTGGTCTGGTTATAT
GTCACAAATTGCTAGGAAACCTTTTTATTTTAAAAACAAAAGACAATTAGCAGGAAACTTAATTT
AACCTAATTAAAAATATTAGATAATTAAACTCTTCATAGACTAAACGTGACAATTTAATATATATA
TATATTTATTTATATATATGATTAAATAAGATATAGTCAATCATCGGTGTGAATCGACTTAAAAAA
AAAAGCACATGGGTTAAACCCATCTCCCCTTATTAGGGGAATCAGAACTTGTTAGTGGGTTCAT
GACAGAGCATGCTGCCGTAGTTTTCGTATTCTTCTTTTTAGCTGAGTACGGTAGTATTGTACTAAT
GTGTATTTTAACTAGTATATTATTTATTGGTGGTTACTTATTATTTGAAATATCCTATGTTTTTACTG
TGGTAAATTATATTTTCTTTGAATTATTCTTTATAGACTGAGTAACATTTGTAGAGGTACAATCTTT
ATACACTGATTTTTTAAATAATTCTATCATTGAAGGATTATTATATGGGTTTAATCTAGGATTAAA
AAGTTCTTTAATGATATTCACATTTATTTGAGCTAGAGCATCCTTCCCTAGAATACGATTTGATCA
ACTAATGGGCTTCTGTTGAACAGTTTTATTACCTATTAATTTTGCAATTATTATATTAGTACCTTGT
GTTTTATATAGTTTTAACTTATTACCTGTAAATATACCATTGTTCTAGCTCACACACCCGCCCGCC
CTACTGCCACAAGGCTACAGTACATATGAGGAGGGGAACTAAAGATCTAGAACTATCCTAGTTA
ATAATTACACTTAATAGTATACTAAATAGACCATCTATCATACTCGAGAATAGTGATAGTGTAAT
TATACACTATTTTAACTGTATTCTACACTATTAGCATATTATTATCTTTATATGATAATAATTTTATA
ACTTAATTTATTAGGTTACATTAACAAAAAATTCGTTAACTTTTTTCACCACTTTTTAATACAAAAT
ACGAATTTTA
>YN460
ATAACAATTCTAAAGAACATAAAGAGTTATTAGATAAAAATAATTCACCTATACAGTTAATAAAT
CAACTTAAAGGGTATTTTTTCATAAATCCTTTATTAGCTTTAAGTTTAGCTATTACTATTTTCTCTTT
TGCAGGTATTCCTCCTCTTGTAGGGTTCTTTGCTAAACAGATGGTATTAAGCGCGGCTATTGATC

AAGGTTATATCTTTTTATCTTTAGTTGCAATATTAACTAGTGTTATAGGAGGGGTTTATTATTTAAA
TATAATTAAAGAAATGTTCTTTTATTCACCTGACTATAAATTAAACGAAGAAATTAAAAATAATAC
TATTAATGGTCAAATTATTAATAGAAACAATAAAATATTAAATGTTGAATTTAATTATACAAATGT
AGTTATGTCTAGTTCTGTGGCAATAACTATTTCTACTATTACATTAGTAGTTTTATTATTCATGTTT
ATGAATAAAGAATGATTAAGTCTGGGTACTATATTGGTACAATCTTTATTTAGCTATTAATGAGTA
GTATGACATTATTTATAGGGTTTGTATCTGTTATAGCTATTTTATTTTTAGCCATTAATTTTATATTT
GCTCCTCATAATCCTTATCAAGAAAAATATAGTATTTTCGAGTGTGGTTTCCATAGTTTTTTAGGG
CAAAATAGAACACAATTCGGTATAAAATTCTTTATTTTTGCTTTAGTTTATTTACTTTTAGATTTAG
AAATATTATTAACTTTCCCTTTCGCTCTTAGTGAGTATGTTAATGGTATTTATGGTCTTTTAGTTAC
TTTAATTTTTATAGCTATAATAACTATAGGATTTATATTTGAATTAGGTAAAAGCGCTCTTAAAATA
GACAGCAGACAAAAATTATATATACCTAAATTGAACGTTAATTACCATACAGAGTATGTTGGAAT
AGGTAAGGTTTCTAAGTAAAGTTATAGAGGCAGAAAACCAAAAAACCTACCAAAGGGTAGCTA
ATGGGAAGCTATTAAAAATAAAAGATGATAACCTATATATAGTATAGTTACTATATATTTACTATA
ACTAGGATTATTATATATATATATATTATCTTATTGTATATTAAGATTATTATTATTATAAGGTATA
ATTAATATAGTATCTTATTGTATAAGAATATAATATATTAACCTATAATTAATTTTATTTTTTAATTT
TTAATTATAATTTTTTTTTATATCTAGATGCTTACACATCTACAGATGTAGAAGAGAACAAAATAT
TGTTGTTATAGTAGTGGATGATATAGAAAAATATTTTTATATTATTTATTTTTGTTAGGTAGCTTTT
GAAGTGTTTGATAGAGAGGATATATGGACGGTAGGAGGGTATTCATTTTAATGAACAGTGGATA
GTTTAAATTAACCTAGTTATAGTTTATGAATTTAAATTATAATTATATTAATGTAGGTTATGATAGA
ATTATGTATTGATCCTTAAGAGTTAAGAGAGATACGCCCACGTATAATACATATTGGTTTAGGGT
TGAGTATATATACTTAAGAGTTAAGAATATATATATACAATATATAATAAATATAGTAATATATTA
ATGGTATGGACTTAACCAGGTTTATATATTATCATTTGATAAACATTAATTATAATTTTTCTATTTA
TTATTATTATTATACTGTGAGATTAATAATTATTAAAATATAATATTACATAGTACGTGATATTTGG
GGATTTTATCTTATTAATTGGCAATTAATGATTCTAATCAAATTTTATTCTCTTTAGTTTAATGGTA
GAACAATGATCTTCTAATTCATTGGTTTTAGTTCGATTCTAAAAAGAGATGAGTAAATAATTTTCT
AGATCAGAAATACTACTTTTAACTACAAAAAGCTTACGCTTTTTAACATTTTTTTGATAAATAACA
ACTATTGTTAATATTTGGCTGTCTATTGGTTTAACTAACAATTACAAAATTTTTTCAATTTTTATAAT
ATAAATTTAAATGAGAATATTAAAAAGTCATTCATTATTAAAATTAGTGAATTCTTACCTTATCGA
TGCGTCACAACCAAGTAACATTAGTTACTTGTGAAATTTTGGTTCATTATTAGCTGTTTGTTTAAT
AGTACAAATTATTACCGGTATTACATTAGCTATGCATTATAGTCCTAGTGTAATGGAAGCTTTTAA
CTCAATAGAGCATATAATGAGAGATGTTAATAACGGGTGATTAGTTCGTTATCTACATAGTAATA
CAGCTTCTGCTTTCTTTTTCTTAGTGTATTTACACATAGGAAGAGGTATATATTACGGATCATATA
GAGCTCCTCGTACTTTAGTTTGAGCTATTGGTACTGTTATATTAATATTAATGATGGCTATCGGTT
TCCTAGGTTATGTTTTACCTTATGGACAGATGTCATTATGAGGTGCTACAGTTATTACTAATCTTA
TTAGTGCTATACCTTGAATAGGGCAAGATATTGTTGAATTCATTTGAGGTGGTTTTTCTGTTAATA
ATGCCACTTTAAACAGATTTTTTGCATTACATTTTGTATTGCCTTTTGTATTAGCTGCTTTAGTTTTA
ATGCACTTAATTGCACTTCATGATACTGCTGGTTCAAGCAATCCTCTTGGTGTTTCAGGTAATTAC
GATAGAATTACATTTGCTCCATATTTTTTATTTAAAGATTTAATTACTATTTTTATATTTATTTTTGTA
TTAAGTGCTTTTGTATTCTTTATGCCTAATGTTTTAGGGGATAGTGATAATTATATTATGGCTAATC
CTATGCAAACTCCTGCTGCTATTGTACCTGAATGATACTTATTACCTTTCTATGCTATTTTAAGATC
TATACCTAATAAATTATTAGGTGTTATAGCGATGTTTAGTGCTATTTTAGCTATTATGTTATTACCT
GTTACAGATTTAGGTAGATCTAGAGGTTTACAATTTAGACCATTTAGTAAAATAGCTTTCTGAGTT
TTTGTTGCTAATTTCTTAGTTTTAATGCAATTAGGTGCTAAACACGTTGAAGATCCATTTATATTAT
TAGGTCAATTAAGTACTGTATTATACTTTAGTTATTTTGTTGCTATATTACCTTTAGCTAGTTACTT

AGATAATAGTTTAACTGATTTATCTAATAAATCTGAATTATTTTTAAATAAAACTAACTAAATATAT
TAAGATTATTATTTAATATATTTTCTATTTAAGATACTATTAATTTAGTATTTTGGGTTTTCAGTTTA
TAATTTATATTATATTATGCATTACCCTCCACCTTGCTTTGTAGTAAGCTAATCTGTTATTTCCTTTA
GTTTAATGGTAGAACAATGATCTTCTAATTCATTGGTTTTAGTTCGAATCTAAAAAGGAAATAAG
AAATATATTCTTATTATTACTTATATAATAATTATTTCTTAAAAATATACATTTTGCATTATAGCCGT
TTAGCTGTATTAAAATGTAAAATGATATAAAATAGAATAAATATTTAAATTATTCCTATGTTATATT
ATCCTATATTGCAACCATTATCAGAAGTTGTATTAATACTTGTACCTGCCTTATTAGCTGTAGCTT
ATGTTACAGTTGCTGAAAGAAAAACTATGGCTAGTATGCAAAGAAGATTAGGTCCTAATGCTGT
AGGTTACTATGGACTATTGCAAGCATTTGCTGATGCCTTAAAACTTTTATTAAAAGAATATGTAG
CTCCTACACAATCTAATATTGTTCTTTTCTTTTTAGGTCCTGTAATAACTTTAATTTTTGCATTATTA
GGTTACGCTGTTATACCCTATGGTCCTGGTTCAGGGATAAGCGACATGAATTTAGGTATATTTTA
CATGTTAGCTGTGTCATCTTTAGCTACATACGGTATTCTATTAGCTGGTTGAAGTGCGAATAGTA
AATACGCTTTTCTAGGTTCTCTTAGAAGTACAGCTCAATTAATTAGTTATGAATTAATATTAAGTT
CAGCTATATTAATAGTAATTATGATAACAGGAAATTTAAATTTAACTGTTTGTACTGAATCTCAAA
GAGCTATTTGATTTATACTACCTTTATTTCCTGTGTTTATAATATTTTTCATAGGATCTATAGCTGA
GACAAATAGAGCTCCTTTTGATTTAGCCGAGGCTAACCTGCTAATCTGGTTTGGTCTGGTTATAT
GTCACAAATTGCTAGGAAACCTTTTTATTTTAAAAACAAAAGACAATTAGCAGGAAACTTAATTT
AACCTAATTAAAAATATTAGATAATTAAACTCTTCATAGACTAAACGTGACAATTTAATATATATA
TATATTTATTTATATATATGATTAAATAAGATATAGTCAATCATCGGTGTGAATCGACTTAAAAAA
AAAAGCACATGGGTTAAACCCATCTCCCCTTATTAGGGGAATCAGAACTTGTTAGTGGGTTCAT
GACAGAGCATGCTGCCGTAGTTTTCGTATTCTTCTTTTTAGCTGAGTACGGTAGTATTGTACTAAT
GTGTATTTTAACTAGTATATTATTTATTGGTGGTTACTTATTATTTGAAATATCCTATGTTTTTACTG
TGGTAAATTATATTTTCTTTGAATTATTCTTTATAGACTGAGTAACATTTGTAGAGGTACAATCTTT
ATACACTGATTTTTTAAATAATTCTATCATTGAAGGATTATTATATGGGTTTAATCTAGGATTAAA
AAGTTCTTTAATGATATTCACATTTATTTGAGCTAGAGCATCCTTCCCTAGAATACGATTTGATCA
ACTAATGGGCTTCTGTTGAACAGTTTTATTACCTATTAATTTTGCAATTATTATATTAGTACCTTGT
GTTTTATATAGTTTTAACTTATTACCTGTAAATATACCATTGTTCTAGCTCACACACCCGCCCGCC
CTACTGCCACAAGGCTACAGTACATATGAGGAGGGGAACTAAAGATCTAGAACTATCCTAGTTA
ATAATTACACTTAATAGTATACTAAATAGACCATCTATCATACTCGAGAATAGTGATAGTGTAAT
TATACACTATTTTAACTGTATTCTACACTATTAGCATATTATTATCTTTATATGATAATAATTTTATA
ACTTAATTTATTAGGTTACATTAACAAAAAATTCGTTAACTTTTTTCACCACTTTTTAATACAAAAT
ACGAATTTTA
>YN462
ATAACAATTCTAAAGAACATAAAGAGTTATTAGATAAAAATAATTCACCTATACAGTTAATAAAT
CAACTTAAAGGGTATTTTTTCATAAATCCTTTATTAGCTTTAAGTTTAGCTATTACTATTTTCTCTTT
TGCAGGTATTCCTCCTCTTGTAGGGTTCTTTGCTAAACAGATGGTATTAAGCGCGGCTATTGATC
AAGGTTATATCTTTTTATCTTTAGTTGCAATATTAACTAGTGTTATAGGAGGGGTTTATTATTTAAA
TATAATTAAAGAAATGTTCTTTTATTCACCTGACTATAAATTAAACGAAGAAATTAAAAATAATAC
TATTAATGGTCAAATTATTAATAGAAACAATAAAATATTAAATGTTGAATTTAATTATACAAATGT
AGTTATGTCTAGTTCTGTGGCAATAACTATTTCTACTATTACATTAGTAGTTTTATTATTCATGTTT
ATGAATAAAGAATGATTAAGTCTGGGTACTATATTGGTACAATCTTTATTTAGCTATTAATGAGTA
GTATGACATTATTTATAGGGTTTGTATCTGTTATAGCTATTTTATTTTTAGCCATTAATTTTATATTT
GCTCCTCATAATCCTTATCAAGAAAAATATAGTATTTTCGAGTGTGGTTTCCATAGTTTTTTAGGG
CAAAATAGAACACAATTCGGTATAAAATTCTTTATTTTTGCTTTAGTTTATTTACTTTTAGATTTAG

AAATATTATTAACTTTCCCTTTCGCTCTTAGTGAGTATGTTAATGGTATTTATGGTCTTTTAGTTAC
TTTAATTTTTATAGCTATAATAACTATAGGATTTATATTTGAATTAGGTAAAAGCGCTCTTAAAATA
GACAGCAGACAAAAATTATATATACCTAAATTGAACGTTAATTACCATACAGAGTATGTTGGAAT
AGGTAAGGTTTCTAAGTAAAGTTATAGAGGCAGAAAACCAAAAAACCTACCAAAGGGTAGCTA
ATGGGAAGCTATTAAAAATAAAAGATGATAACCTATATATAGTATAGTTACTATATATTTACTATA
ACTAGGATTATTATATATATATATATTATCTTATTGTATATTAAGATTATTATTATTATAAGGTATA
ATTAATATAGTATCTTATTGTATAAGAATATAATATATTAACCTATAATTAATTTTATTTTTTAATTT
TTAATTATAATTTTTTTTTATATCTAGATGCTTACACATCTACAGATGTAGAAGAGAACAAAATAT
TGTTGTTATAGTAGTGGATGATATAGAAAAATATTTTTATATTATTTATTTTTGTTAGGTAGCTTTT
GAAGTGTTTGATAGAGAGGATATATGGACGGTAGGAGGGTATTCATTTTAATGAACAGTGGATA
GTTTAAATTAACCTAGTTATAGTTTATGAATTTAAATTATAATTATATTAATGTAGGTTATGATAGA
ATTATGTATTGATCCTTAAGAGTTAAGAGAGATACGCCCACGTATAATACATATTGGTTTAGGGT
TGAGTATATATACTTAAGAGTTAAGAATATATATATACAATATATAATAAATATAGTAATATATTA
ATGGTATGGACTTAACCAGGTTTATATATTATCATTTGATAAACATTAATTATAATTTTTCTATTTA
TTATTATTATTATACTGTGAGATTAATAATTATTAAAATATAATATTACATAGTACGTGATATTTGG
GGATTTTATCTTATTAATTGGCAATTAATGATTCTAATCAAATTTTATTCTCTTTAGTTTAATGGTA
GAACAATGATCTTCTAATTCATTGGTTTTAGTTCGATTCTAAAAAGAGATGAGTAAATAATTTTCT
AGATCAGAAATACTACTTTTAACTACAAAAAGCTTACGCTTTTTAACATTTTTTTGATAAATAACA
ACTATTGTTAATATTTGGCTGTCTATTGGTTTAACTAACAATTACAAAATTTTTTCAATTTTTATAAT
ATAAATTTAAATGAGAATATTAAAAAGTCATTCATTATTAAAATTAGTGAATTCTTACCTTATCGA
TGCGTCACAACCAAGTAACATTAGTTACTTGTGAAATTTTGGTTCATTATTAGCTGTTTGTTTAAT
AGTACAAATTATTACCGGTATTACATTAGCTATGCATTATAGTCCTAGTGTAATGGAAGCTTTTAA
CTCAATAGAGCATATAATGAGAGATGTTAATAACGGGTGATTAGTTCGTTATCTACATAGTAATA
CAGCTTCTGCTTTCTTTTTCTTAGTGTATTTACACATAGGAAGAGGTATATATTACGGATCATATA
GAGCTCCTCGTACTTTAGTTTGAGCTATTGGTACTGTTATATTAATATTAATGATGGCTATCGGTT
TCCTAGGTTATGTTTTACCTTATGGACAGATGTCATTATGAGGTGCTACAGTTATTACTAATCTTA
TTAGTGCTATACCTTGAATAGGGCAAGATATTGTTGAATTCATTTGAGGTGGTTTTTCTGTTAATA
ATGCCACTTTAAACAGATTTTTTGCATTACATTTTGTATTGCCTTTTGTATTAGCTGCTTTAGTTTTA
ATGCACTTAATTGCACTTCATGATACTGCTGGTTCAAGCAATCCTCTTGGTGTTTCAGGTAATTAC
GATAGAATTACATTTGCTCCATATTTTTTATTTAAAGATTTAATTACTATTTTTATATTTATTTTTGTA
TTAAGTGCTTTTGTATTCTTTATGCCTAATGTTTTAGGGGATAGTGATAATTATATTATGGCTAATC
CTATGCAAACTCCTGCTGCTATTGTACCTGAATGATACTTATTACCTTTCTATGCTATTTTAAGATC
TATACCTAATAAATTATTAGGTGTTATAGCGATGTTTAGTGCTATTTTAGCTATTATGTTATTACCT
GTTACAGATTTAGGTAGATCTAGAGGTTTACAATTTAGACCATTTAGTAAAATAGCTTTCTGAGTT
TTTGTTGCTAATTTCTTAGTTTTAATGCAATTAGGTGCTAAACACGTTGAAGATCCATTTATATTAT
TAGGTCAATTAAGTACTGTATTATACTTTAGTTATTTTGTTGCTATATTACCTTTAGCTAGTTACTT
AGATAATAGTTTAACTGATTTATCTAATAAATCTGAATTATTTTTAAATAAAACTAACTAAATATAT
TAAGATTATTATTTAATATATTTTCTATTTAAGATACTATTAATTTAGTATTTTGGGTTTTCAGTTTA
TAATTTATATTATATTATGCATTACCCTCCACCTTGCTTTGTAGTAAGCTAATCTGTTATTTCCTTTA
GTTTAATGGTAGAACAATGATCTTCTAATTCATTGGTTTTAGTTCGAATCTAAAAAGGAAATAAG
AAATATATTCTTATTATTACTTATATAATAATTATTTCTTAAAAATATACATTTTGCATTATAGCCGT
TTAGCTGTATTAAAATGTAAAATGATATAAAATAGAATAAATATTTAAATTATTCCTATGTTATATT
ATCCTATATTGCAACCATTATCAGAAGTTGTATTAATACTTGTACCTGCCTTATTAGCTGTAGCTT
ATGTTACAGTTGCTGAAAGAAAAACTATGGCTAGTATGCAAAGAAGATTAGGTCCTAATGCTGT

AGGTTACTATGGACTATTGCAAGCATTTGCTGATGCCTTAAAACTTTTATTAAAAGAATATGTAG
CTCCTACACAATCTAATATTGTTCTTTTCTTTTTAGGTCCTGTAATAACTTTAATTTTTGCATTATTA
GGTTACGCTGTTATACCCTATGGTCCTGGTTCAGGGATAAGCGACATGAATTTAGGTATATTTTA
CATGTTAGCTGTGTCATCTTTAGCTACATACGGTATTCTATTAGCTGGTTGAAGTGCGAATAGTA
AATACGCTTTTCTAGGTTCTCTTAGAAGTACAGCTCAATTAATTAGTTATGAATTAATATTAAGTT
CAGCTATATTAATAGTAATTATGATAACAGGAAATTTAAATTTAACTGTTTGTACTGAATCTCAAA
GAGCTATTTGATTTATACTACCTTTATTTCCTGTGTTTATAATATTTTTCATAGGATCTATAGCTGA
GACAAATAGAGCTCCTTTTGATTTAGCCGAGGCTAACCTGCTAATCTGGTTTGGTCTGGTTATAT
GTCACAAATTGCTAGGAAACCTTTTTATTTTAAAAACAAAAGACAATTAGCAGGAAACTTAATTT
AACCTAATTAAAAATATTAGATAATTAAACTCTTCATAGACTAAACGTGACAATTTAATATATATA
TATATTTATTTATATATATGATTAAATAAGATATAGTCAATCATCGGTGTGAATCGACTTAAAAAA
AAAAGCACATGGGTTAAACCCATCTCCCCTTATTAGGGGAATCAGAACTTGTTAGTGGGTTCAT
GACAGAGCATGCTGCCGTAGTTTTCGTATTCTTCTTTTTAGCTGAGTACGGTAGTATTGTACTAAT
GTGTATTTTAACTAGTATATTATTTATTGGTGGTTACTTATTATTTGAAATATCCTATGTTTTTACTG
TGGTAAATTATATTTTCTTTGAATTATTCTTTATAGACTGAGTAACATTTGTAGAGGTACAATCTTT
ATACACTGATTTTTTAAATAATTCTATCATTGAAGGATTATTATATGGGTTTAATCTAGGATTAAA
AAGTTCTTTAATGATATTCACATTTATTTGAGCTAGAGCATCCTTCCCTAGAATACGATTTGATCA
ACTAATGGGCTTCTGTTGAACAGTTTTATTACCTATTAATTTTGCAATTATTATATTAGTACCTTGT
GTTTTATATAGTTTTAACTTATTACCTGTAAATATACCATTGTTCTAGCTCACACACCCGCCCGCC
CTACTGCCACAAGGCTACAGTACATATGAGGAGGGGAACTAAAGATCTAGAACTATCCTAGTTA
ATAATTACACTTAATAGTATACTAAATAGACCATCTATCATACTCGAGAATAGTGATAGTGTAAT
TATACACTATTTTAACTGTATTCTACACTATTAGCATATTATTATCTTTATATGATAATAATTTTATA
ACTTAATTTATTAGGTTACATTAACAAAAAATTCGTTAACTTTTTTCACCACTTTTTAATACAAAAT
ACGAATTTTA
>YN463
ATAACAATTCTAAAGAACATAAAGAGTTATTAGATAAAAATAATTCACCTATACAGTTAATAAAT
CAACTTAAAGGGTATTTTTTCATAAATCCTTTATTAGCTTTAAGTTTAGCTATTACTATTTTCTCTTT
TGCAGGTATTCCTCCTCTTGTAGGGTTCTTTGCTAAACAGATGGTATTAAGCGCGGCTATTGATC
AAGGTTATATCTTTTTATCTTTAGTTGCAATATTAACTAGTGTTATAGGAGGGGTTTATTATTTAAA
TATAATTAAAGAAATGTTCTTTTATTCACCTGACTATAAATTAAACGAAGAAATTAAAAATAATAC
TATTAATGGTCAAATTATTAATAGAAACAATAAAATATTAAATGTTGAATTTAATTATACAAATGT
AGTTATGTCTAGTTCTGTGGCAATAACTATTTCTACTATTACATTAGTAGTTTTATTATTCATGTTT
ATGAATAAAGAATGATTAAGTCTGGGTACTATATTGGTACAATCTTTATTTAGCTATTAATGAGTA
GTATGACATTATTTATAGGGTTTGTATCTGTTATAGCTATTTTATTTTTAGCCATTAATTTTATATTT
GCTCCTCATAATCCTTATCAAGAAAAATATAGTATTTTCGAGTGTGGTTTCCATAGTTTTTTAGGG
CAAAATAGAACACAATTCGGTATAAAATTCTTTATTTTTGCTTTAGTTTATTTACTTTTAGATTTAG
AAATATTATTAACTTTCCCTTTCGCTCTTAGTGAGTATGTTAATGGTATTTATGGTCTTTTAGTTAC
TTTAATTTTTATAGCTATAATAACTATAGGATTTATATTTGAATTAGGTAAAAGCGCTCTTAAAATA
GACAGCAGACAAAAATTATATATACCTAAATTGAACGTTAATTACCATACAGAGTATGTTGGAAT
AGGTAAGGTTTCTAAGTAAAGTTATAGAGGCAGAAAACCAAAAAACCTACCAAAGGGTAGCTA
ATGGGAAGCTATTAAAAATAAAAGATGATAACCTATATATAGTATAGTTACTATATATTTACTATA
ACTAGGATTATTATATATATATATATATTATCTTATTGTATATTAAGATTATTATTATTATAAGGTATA
ATTAATATAGTATCTTATTGTATAAGAATATAATATATTAACCTATAATTAATTTTATTTTTTAATTT
TTAATTATAATTTTTTTTTATATCTAGATGCTTACACATCTACAGATGTAGAAGAGAACAAAATAT

TGTTGTTATAGTAGTGGATGATATAGAAAAATATTTTTATATTATTTATTTTTGTTAGGTAGCTTTT
GAAGTGTTTGATAGAGAGGATATATGGACGGTAGGAGGGTATTCATTTTAATGAACAGTGGATA
GTTTAAATTAACCTAGTTATAGTTTATGAATTTAAATTATAATTATATTAATGTAGGTTATGATAGA
ATTATGTATTGATCCTTAAGAGTTAAGAGAGATACGCCCACGTATAATACATATTGGTTTAGGGT
TGAGTATATATACTTAAGAGTTAAGAATATATATATACAATATATAATAAATATAGTAATATATTA
ATGGTATGGACTTAACCAGGTTTATATATTATCATTTGATAAACATTAATTATAATTTTTCTATTTA
TTATTATTATTATACTGTGAGATTAATAATTATTAAAATATAATATTACATAGTACGTGATATTTGG
GGATTTTATCTTATTAATTGGCAATTAATGATTCTAATCAAATTTTATTCTCTTTAGTTTAATGGTA
GAACAATGATCTTCTAATTCATTGGTTTTAGTTCGATTCTAAAAAGAGATGAGTAAATAATTTTCT
AGATCAGAAATACTACTTTTAACTACAAAAAGCTTACGCTTTTTAACATTTTTTTGATAAATAACA
ACTATTGTTAATATTTGGCTGTCTATTGGTTTAACTAACAATTACAAAATTTTTTCAATTTTTATAAT
ATAAATTTAAATGAGAATATTAAAAAGTCATTCATTATTAAAATTAGTGAATTCTTACCTTATCGA
TGCGTCACAACCAAGTAACATTAGTTACTTGTGAAATTTTGGTTCATTATTAGCTGTTTGTTTAAT
AGTACAAATTATTACCGGTATTACATTAGCTATGCATTATAGTCCTAGTGTAATGGAAGCTTTTAA
CTCAATAGAGCATATAATGAGAGATGTTAATAACGGGTGATTAGTTCGTTATCTACATAGTAATA
CAGCTTCTGCTTTCTTTTTCTTAGTGTATTTACACATAGGAAGAGGTATATATTACGGATCATATA
GAGCTCCTCGTACTTTAGTTTGAGCTATTGGTACTGTTATATTAATATTAATGATGGCTATCGGTT
TCCTAGGTTATGTTTTACCTTATGGACAGATGTCATTATGAGGTGCTACAGTTATTACTAATCTTA
TTAGTGCTATACCTTGAATAGGGCAAGATATTGTTGAATTCATTTGAGGTGGTTTTTCTGTTAATA
ATGCCACTTTAAACAGATTTTTTGCATTACATTTTGTATTGCCTTTTGTATTAGCTGCTTTAGTTTTA
ATGCACTTAATTGCACTTCATGATACTGCTGGTTCAAGCAATCCTCTTGGTGTTTCAGGTAATTAC
GATAGAATTACATTTGCTCCATATTTTTTATTTAAAGATTTAATTACTATTTTTATATTTATTTTTGTA
TTAAGTGCTTTTGTATTCTTTATGCCTAATGTTTTAGGGGATAGTGATAATTATATTATGGCTAATC
CTATGCAAACTCCTGCTGCTATTGTACCTGAATGATACTTATTACCTTTCTATGCTATTTTAAGATC
TATACCTAATAAATTATTAGGTGTTATAGCGATGTTTAGTGCTATTTTAGCTATTATGTTATTACCT
GTTACAGATTTAGGTAGATCTAGAGGTTTACAATTTAGACCATTTAGTAAAATAGCTTTCTGAGTT
TTTGTTGCTAATTTCTTAGTTTTAATGCAATTAGGTGCTAAACACGTTGAAGATCCATTTATATTAT
TAGGTCAATTAAGTACTGTATTATACTTTAGTTATTTTGTTGCTATATTACCTTTAGCTAGTTACTT
AGATAATAGTTTAACTGATTTATCTAATAAATCTGAATTATTTTTAAATAAAACTAACTAAATATAT
TAAGATTATTATTTAATATATTTTCTATTTAAGATACTATTAATTTAGTATTTTGGGTTTTCAGTTTA
TAATTTATATTATATTATGCATTACCCTCCACCTTGCTTTGTAGTAAGCTAATCTGTTATTTCCTTTA
GTTTAATGGTAGAACAATGATCTTCTAATTCATTGGTTTTAGTTCGAATCTAAAAAGGAAATAAG
AAATATATTCTTATTATTACTTATATAATAATTATTTCTTAAAAATATACATTTTGCATTATAGCCGT
TTAGCTGTATTAAAATGTAAAATGATATAAAATAGAATAAATATTTAAATTATTCCTATGTTATATT
ATCCTATATTGCAACCATTATCAGAAGTTGTATTAATACTTGTACCTGCCTTATTAGCTGTAGCTT
ATGTTACAGTTGCTGAAAGAAAAACTATGGCTAGTATGCAAAGAAGATTAGGTCCTAATGCTGT
AGGTTACTATGGACTATTGCAAGCATTTGCTGATGCCTTAAAACTTTTATTAAAAGAATATGTAG
CTCCTACACAATCTAATATTGTTCTTTTCTTTTTAGGTCCTGTAATAACTTTAATTTTTGCATTATTA
GGTTACGCTGTTATACCCTATGGTCCTGGTTCAGGGATAAGCGACATGAATTTAGGTATATTTTA
CATGTTAGCTGTGTCATCTTTAGCTACATACGGTATTCTATTAGCTGGTTGAAGTGCGAATAGTA
AATACGCTTTTCTAGGTTCTCTTAGAAGTACAGCTCAATTAATTAGTTATGAATTAATATTAAGTT
CAGCTATATTAATAGTAATTATGATAACAGGAAATTTAAATTTAACTGTTTGTACTGAATCTCAAA
GAGCTATTTGATTTATACTACCTTTATTTCCTGTGTTTATAATATTTTTCATAGGATCTATAGCTGA
GACAAATAGAGCTCCTTTTGATTTAGCCGAGGCTAACCTGCTAATCTGGTTTGGTCTGGTTATAT

GTCACAAATTGCTAGGAAACCTTTTTATTTTAAAAACAAAAGACAATTAGCAGGAAACTTAATTT
AACCTAATTAAAAATATTAGATAATTAAACTCTTCATAGACTAAACGTGACAATTTAATATATATA
TATATTTATTTATATATATGATTAAATAAGATATAGTCAATCATCGGTGTGAATCGACTTAAAAAA
AAAAGCACATGGGTTAAACCCATCTCCCCTTATTAGGGGAATCAGAACTTGTTAGTGGGTTCAT
GACAGAGCATGCTGCCGTAGTTTTCGTATTCTTCTTTTTAGCTGAGTACGGTAGTATTGTACTAAT
GTGTATTTTAACTAGTATATTATTTATTGGTGGTTACTTATTATTTGAAATATCCTATGTTTTTACTG
TGGTAAATTATATTTTCTTTGAATTATTCTTTATAGACTGAGTAACATTTGTAGAGGTACAATCTTT
ATACACTGATTTTTTAAATAATTCTATCATTGAAGGATTATTATATGGGTTTAATCTAGGATTAAA
AAGTTCTTTAATGATATTCACATTTATTTGAGCTAGAGCATCCTTCCCTAGAATACGATTTGATCA
ACTAATGGGCTTCTGTTGAACAGTTTTATTACCTATTAATTTTGCAATTATTATATTAGTACCTTGT
GTTTTATATAGTTTTAACTTATTACCTGTAAATATACCATTGTTCTAGCTCACACACCCGCCCGCC
CTACTGCCACAAGGCTACAGTACATATGAGGAGGGGAACTAAAGATCTAGAACTATCCTAGTTA
ATAATTACACTTAATAGTATACTAAATAGACCATCTATCATACTCGAGAATAGTGATAGTGTAAT
TATACACTATTTTAACTGTATTCTACACTATTAGCATATTATTATCTTTATATGATAATAATTTTATA
ACTTAATTTATTAGGTTACATTAACAAAAAATTCGTTAACTTTTTTCACCACTTTTTAATACAAAAT
ACGAATTTTA
>YN468
ATAACAATTCTAAAGAACATAAAGAGTTATTAGATAAAAATAATTCACCTATACAGTTAATAAAT
CAACTTAAAGGGTATTTTTTCATAAATCCTTTATTAGCTTTAAGTTTAGCTATTACTATTTTCTCTTT
TGCAGGTATTCCTCCTCTTGTAGGGTTCTTTGCTAAACAGATGGTATTAAGCGCGGCTATTGATC
AAGGTTATATCTTTTTATCTTTAGTTGCAATATTAACTAGTGTTATAGGAGGGGTTTATTATTTAAA
TATAATTAAAGAAATGTTCTTTTATTCACCTGACTATAAATTAAACGAAGAAATTAAAAATAATAC
TATTAATGGTCAAATTATTAATAGAAACAATAAAATATTAAATGTTGAATTTAATTATACAAATGT
AGTTATGTCTAGTTCTGTGGCAATAACTATTTCTACTATTACATTAGTAGTTTTATTATTCATGTTT
ATGAATAAAGAATGATTAAGTCTGGGTACTATATTGGTACAATCTTTATTTAGCTATTAATGAGTA
GTATGACATTATTTATAGGGTTTGTATCTGTTATAGCTATTTTATTTTTAGCCATTAATTTTATATTT
GCTCCTCATAATCCTTATCAAGAAAAATATAGTATTTTCGAGTGTGGTTTCCATAGTTTTTTAGGG
CAAAATAGAACACAATTCGGTATAAAATTCTTTATTTTTGCTTTAGTTTATTTACTTTTAGATTTAG
AAATATTATTAACTTTCCCTTTCGCTCTTAGTGAGTATGTTAATGGTATTTATGGTCTTTTAGTTAC
TTTAATTTTTATAGCTATAATAACTATAGGATTTATATTTGAATTAGGTAAAAGCGCTCTTAAAATA
GACAGCAGACAAAAATTATATATACCTAAATTGAACGTTAATTACCATACAGAGTATGTTGGAAT
AGGTAAGGTTTCTAAGTAAAGTTATAGAGGCAGAAAACCAAAAAACCTACCAAAGGGTAGCTA
ATGGGAAGCTATTAAAAATAAAAGATGATAACCTATATATAGTATAGTTACTATATATTTACTATA
ACTAGGATTATTATATATATATATATTATCTTATTGTATATTAAGATTATTATTATTATAAGGTATA
ATTAATATAGTATCTTATTGTATAAGAATATAATATATTAACCTATAATTAATTTTATTTTTTAATTT
TTAATTATAATTTTTTTTTATATCTAGATGCTTACACATCTACAGATGTAGAAGAGAACAAAATAT
TGTTGTTATAGTAGTGGATGATATAGAAAAATATTTTTATATTATTTATTTTTGTTAGGTAGCTTTT
GAAGTGTTTGATAGAGAGGATATATGGACGGTAGGAGGGTATTCATTTTAATGAACAGTGGATA
GTTTAAATTAACCTAGTTATAGTTTATGAATTTAAATTATAATTATATTAATGTAGGTTATGATAGA
ATTATGTATTGATCCTTAAGAGTTAAGAGAGATACGCCCACGTATAATACATATTGGTTTAGGGT
TGAGTATATATACTTAAGAGTTAAGAATATATATATACAATATATAATAAATATAGTAATATATTA
ATGGTATGGACTTAACCAGGTTTATATATTATCATTTGATAAACATTAATTATAATTTTTCTATTTA
TTATTATTATTATACTGTGAGATTAATAATTATTAAAATATAATATTACATAGTACGTGATATTTGG
GGATTTTATCTTATTAATTGGCAATTAATGATTCTAATCAAATTTTATTCTCTTTAGTTTAATGGTA

GAACAATGATCTTCTAATTCATTGGTTTTAGTTCGATTCTAAAAAGAGATGAGTAAATAATTTTCT
AGATCAGAAATACTACTTTTAACTACAAAAAGCTTACGCTTTTTAACATTTTTTTGATAAATAACA
ACTATTGTTAATATTTGGCTGTCTATTGGTTTAACTAACAATTACAAAATTTTTTCAATTTTTATAAT
ATAAATTTAAATGAGAATATTAAAAAGTCATTCATTATTAAAATTAGTGAATTCTTACCTTATCGA
TGCGTCACAACCAAGTAACATTAGTTACTTGTGAAATTTTGGTTCATTATTAGCTGTTTGTTTAAT
AGTACAAATTATTACCGGTATTACATTAGCTATGCATTATAGTCCTAGTGTAATGGAAGCTTTTAA
CTCAATAGAGCATATAATGAGAGATGTTAATAACGGGTGATTAGTTCGTTATCTACATAGTAATA
CAGCTTCTGCTTTCTTTTTCTTAGTGTATTTACACATAGGAAGAGGTATATATTACGGATCATATA
GAGCTCCTCGTACTTTAGTTTGAGCTATTGGTACTGTTATATTAATATTAATGATGGCTATCGGTT
TCCTAGGTTATGTTTTACCTTATGGACAGATGTCATTATGAGGTGCTACAGTTATTACTAATCTTA
TTAGTGCTATACCTTGAATAGGGCAAGATATTGTTGAATTCATTTGAGGTGGTTTTTCTGTTAATA
ATGCCACTTTAAACAGATTTTTTGCATTACATTTTGTATTGCCTTTTGTATTAGCTGCTTTAGTTTTA
ATGCACTTAATTGCACTTCATGATACTGCTGGTTCAAGCAATCCTCTTGGTGTTTCAGGTAATTAC
GATAGAATTACATTTGCTCCATATTTTTTATTTAAAGATTTAATTACTATTTTTATATTTATTTTTGTA
TTAAGTGCTTTTGTATTCTTTATGCCTAATGTTTTAGGGGATAGTGATAATTATATTATGGCTAATC
CTATGCAAACTCCTGCTGCTATTGTACCTGAATGATACTTATTACCTTTCTATGCTATTTTAAGATC
TATACCTAATAAATTATTAGGTGTTATAGCGATGTTTAGTGCTATTTTAGCTATTATGTTATTACCT
GTTACAGATTTAGGTAGATCTAGAGGTTTACAATTTAGACCATTTAGTAAAATAGCTTTCTGAGTT
TTTGTTGCTAATTTCTTAGTTTTAATGCAATTAGGTGCTAAACACGTTGAAGATCCATTTATATTAT
TAGGTCAATTAAGTACTGTATTATACTTTAGTTATTTTGTTGCTATATTACCTTTAGCTAGTTACTT
AGATAATAGTTTAACTGATTTATCTAATAAATCTGAATTATTTTTAAATAAAACTAACTAAATATAT
TAAGATTATTATTTAATATATTTTCTATTTAAGATACTATTAATTTAGTATTTTGGGTTTTCAGTTTA
TAATTTATATTATATTATGCATTACCCTCCACCTTGCTTTGTAGTAAGCTAATCTGTTATTTCCTTTA
GTTTAATGGTAGAACAATGATCTTCTAATTCATTGGTTTTAGTTCGAATCTAAAAAGGAAATAAG
AAATATATTCTTATTATTACTTATATAATAATTATTTCTTAAAAATATACATTTTGCATTATAGCCGT
TTAGCTGTATTAAAATGTAAAATGATATAAAATAGAATAAATATTTAAATTATTCCTATGTTATATT
ATCCTATATTGCAACCATTATCAGAAGTTGTATTAATACTTGTACCTGCCTTATTAGCTGTAGCTT
ATGTTACAGTTGCTGAAAGAAAAACTATGGCTAGTATGCAAAGAAGATTAGGTCCTAATGCTGT
AGGTTACTATGGACTATTGCAAGCATTTGCTGATGCCTTAAAACTTTTATTAAAAGAATATGTAG
CTCCTACACAATCTAATATTGTTCTTTTCTTTTTAGGTCCTGTAATAACTTTAATTTTTGCATTATTA
GGTTACGCTGTTATACCCTATGGTCCTGGTTCAGGGATAAGCGACATGAATTTAGGTATATTTTA
CATGTTAGCTGTGTCATCTTTAGCTACATACGGTATTCTATTAGCTGGTTGAAGTGCGAATAGTA
AATACGCTTTTCTAGGTTCTCTTAGAAGTACAGCTCAATTAATTAGTTATGAATTAATATTAAGTT
CAGCTATATTAATAGTAATTATGATAACAGGAAATTTAAATTTAACTGTTTGTACTGAATCTCAAA
GAGCTATTTGATTTATACTACCTTTATTTCCTGTGTTTATAATATTTTTCATAGGATCTATAGCTGA
GACAAATAGAGCTCCTTTTGATTTAGCCGAGGCTAACCTGCTAATCTGGTTTGGTCTGGTTATAT
GTCACAAATTGCTAGGAAACCTTTTTATTTTAAAAACAAAAGACAATTAGCAGGAAACTTAATTT
AACCTAATTAAAAATATTAGATAATTAAACTCTTCATAGACTAAACGTGACAATTTAATATATATA
TATATTTATTTATATATATGATTAAATAAGATATAGTCAATCATCGGTGTGAATCGACTTAAAAAA
AAAAGCACATGGGTTAAACCCATCTCCCCTTATTAGGGGAATCAGAACTTGTTAGTGGGTTCAT
GACAGAGCATGCTGCCGTAGTTTTCGTATTCTTCTTTTTAGCTGAGTACGGTAGTATTGTACTAAT
GTGTATTTTAACTAGTATATTATTTATTGGTGGTTACTTATTATTTGAAATATCCTATGTTTTTACTG
TGGTAAATTATATTTTCTTTGAATTATTCTTTATAGACTGAGTAACATTTGTAGAGGTACAATCTTT
ATACACTGATTTTTTAAATAATTCTATCATTGAAGGATTATTATATGGGTTTAATCTAGGATTAAA

AAGTTCTTTAATGATATTCACATTTATTTGAGCTAGAGCATCCTTCCCTAGAATACGATTTGATCA
ACTAATGGGCTTCTGTTGAACAGTTTTATTACCTATTAATTTTGCAATTATTATATTAGTACCTTGT
GTTTTATATAGTTTTAACTTATTACCTGTAAATATACCATTGTTCTAGCTCACACACCCGCCCGCC
CTACTGCCACAAGGCTACAGTACATATGAGGAGGGGAACTAAAGATCTAGAACTATCCTAGTTA
ATAATTACACTTAATAGTATACTAAATAGACCATCTATCATACTCGAGAATAGTGATAGTGTAAT
TATACACTATTTTAACTGTATTCTACACTATTAGCATATTATTATCTTTATATGATAATAATTTTATA
ACTTAATTTATTAGGTTACATTAACAAAAAATTCGTTAACTTTTTTCACCACTTTTTAATACAAAAT
ACGAATTTTA
>YN476
ATAACAATTCTAAAGAACATAAAGAGTTATTAGATAAAAATAATTCACCTATACAGTTAATAAAT
CAACTTAAAGGGTATTTTTTCATAAATCCTTTATTAGCTTTAAGTTTAGCTATTACTATTTTCTCTTT
TGCAGGTATTCCTCCTCTTGTAGGGTTCTTTGCTAAACAGATGGTATTAAGCGCGGCTATTGATC
AAGGTTATATCTTTTTATCTTTAGTTGCAATATTAACTAGTGTTATAGGAGGGGTTTATTATTTAAA
TATAATTAAAGAAATGTTCTTTTATTCACCTGACTATAAATTAAACGAAGAAATTAAAAATAATAC
TATTAATGGTCAAATTATTAATAGAAACAATAAAATATTAAATGTTGAATTTAATTATACAAATGT
AGTTATGTCTAGTTCTGTGGCAATAACTATTTCTACTATTACATTAGTAGTTTTATTATTCATGTTT
ATGAATAAAGAATGATTAAGTCTGGGTACTATATTGGTACAATCTTTATTTAGCTATTAATGAGTA
GTATGACATTATTTATAGGGTTTGTATCTGTTATAGCTATTTTATTTTTAGCCATTAATTTTATATTT
GCTCCTCATAATCCTTATCAAGAAAAATATAGTATTTTCGAGTGTGGTTTCCATAGTTTTTTAGGG
CAAAATAGAACACAATTCGGTATAAAATTCTTTATTTTTGCTTTAGTTTATTTACTTTTAGATTTAG
AAATATTATTAACTTTCCCTTTCGCTCTTAGTGAGTATGTTAATGGTATTTATGGTCTTTTAGTTAC
TTTAATTTTTATAGCTATAATAACTATAGGATTTATATTTGAATTAGGTAAAAGCGCTCTTAAAATA
GACAGCAGACAAAAATTATATATACCTAAATTGAACGTTAATTACCATACAGAGTATGTTGGAAT
AGGTAAGGTTTCTAAGTAAAGTTATAGAGGCAGAAAACCAAAAAACCTACCAAAGGGTAGCTA
ATGGGAAGCTATTAAAAATAAAAGATGATAACCTATATATAGTATAGTTACTATATATTTACTATA
ACTAGGATTATTATATATATATATATTATCTTATTGTATATTAAGATTATTATTATTATAAGGTATA
ATTAATATAGTATCTTATTGTATAAGAATATAATATATTAACCTATAATTAATTTTATTTTTTAATTT
TTAATTATAATTTTTTTTTATATCTAGATGCTTACACATCTACAGATGTAGAAGAGAACAAAATAT
TGTTGTTATAGTAGTGGATGATATAGAAAAATATTTTTATATTATTTATTTTTGTTAGGTAGCTTTT
GAAGTGTTTGATAGAGAGGATATATGGACGGTAGGAGGGTATTCATTTTAATGAACAGTGGATA
GTTTAAATTAACCTAGTTATAGTTTATGAATTTAAATTATAATTATATTAATGTAGGTTATGATAGA
ATTATGTATTGATCCTTAAGAGTTAAGAGAGATACGCCCACGTATAATACATATTGGTTTAGGGT
TGAGTATATATACTTAAGAGTTAAGAATATATATATACAATATATAATAAATATAGTAATATATTA
ATGGTATGGACTTAACCAGGTTTATATATTATCATTTGATAAACATTAATTATAATTTTTCTATTTA
TTATTATTATTATACTGTGAGATTAATAATTATTAAAATATAATATTACATAGTACGTGATATTTGG
GGATTTTATCTTATTAATTGGCAATTAATGATTCTAATCAAATTTTATTCTCTTTAGTTTAATGGTA
GAACAATGATCTTCTAATTCATTGGTTTTAGTTCGATTCTAAAAAGAGATGAGTAAATAATTTTCT
AGATCAGAAATACTACTTTTAACTACAAAAAGCTTACGCTTTTTAACATTTTTTTGATAAATAACA
ACTATTGTTAATATTTGGCTGTCTATTGGTTTAACTAACAATTACAAAATTTTTTCAATTTTTATAAT
ATAAATTTAAATGAGAATATTAAAAAGTCATTCATTATTAAAATTAGTGAATTCTTACCTTATCGA
TGCGTCACAACCAAGTAACATTAGTTACTTGTGAAATTTTGGTTCATTATTAGCTGTTTGTTTAAT
AGTACAAATTATTACCGGTATTACATTAGCTATGCATTATAGTCCTAGTGTAATGGAAGCTTTTAA
CTCAATAGAGCATATAATGAGAGATGTTAATAACGGGTGATTAGTTCGTTATCTACATAGTAATA
CAGCTTCTGCTTTCTTTTTCTTAGTGTATTTACACATAGGAAGAGGTATATATTACGGATCATATA

GAGCTCCTCGTACTTTAGTTTGAGCTATTGGTACTGTTATATTAATATTAATGATGGCTATCGGTT
TCCTAGGTTATGTTTTACCTTATGGACAGATGTCATTATGAGGTGCTACAGTTATTACTAATCTTA
TTAGTGCTATACCTTGAATAGGGCAAGATATTGTTGAATTCATTTGAGGTGGTTTTTCTGTTAATA
ATGCCACTTTAAACAGATTTTTTGCATTACATTTTGTATTGCCTTTTGTATTAGCTGCTTTAGTTTTA
ATGCACTTAATTGCACTTCATGATACTGCTGGTTCAAGCAATCCTCTTGGTGTTTCAGGTAATTAC
GATAGAATTACATTTGCTCCATATTTTTTATTTAAAGATTTAATTACTATTTTTATATTTATTTTTGTA
TTAAGTGCTTTTGTATTCTTTATGCCTAATGTTTTAGGGGATAGTGATAATTATATTATGGCTAATC
CTATGCAAACTCCTGCTGCTATTGTACCTGAATGATACTTATTACCTTTCTATGCTATTTTAAGATC
TATACCTAATAAATTATTAGGTGTTATAGCGATGTTTAGTGCTATTTTAGCTATTATGTTATTACCT
GTTACAGATTTAGGTAGATCTAGAGGTTTACAATTTAGACCATTTAGTAAAATAGCTTTCTGAGTT
TTTGTTGCTAATTTCTTAGTTTTAATGCAATTAGGTGCTAAACACGTTGAAGATCCATTTATATTAT
TAGGTCAATTAAGTACTGTATTATACTTTAGTTATTTTGTTGCTATATTACCTTTAGCTAGTTACTT
AGATAATAGTTTAACTGATTTATCTAATAAATCTGAATTATTTTTAAATAAAACTAACTAAATATAT
TAAGATTATTATTTAATATATTTTCTATTTAAGATACTATTAATTTAGTATTTTGGGTTTTCAGTTTA
TAATTTATATTATATTATGCATTACCCTCCACCTTGCTTTGTAGTAAGCTAATCTGTTATTTCCTTTA
GTTTAATGGTAGAACAATGATCTTCTAATTCATTGGTTTTAGTTCGAATCTAAAAAGGAAATAAG
AAATATATTCTTATTATTACTTATATAATAATTATTTCTTAAAAATATACATTTTGCATTATAGCCGT
TTAGCTGTATTAAAATGTAAAATGATATAAAATAGAATAAATATTTAAATTATTCCTATGTTATATT
ATCCTATATTGCAACCATTATCAGAAGTTGTATTAATACTTGTACCTGCCTTATTAGCTGTAGCTT
ATGTTACAGTTGCTGAAAGAAAAACTATGGCTAGTATGCAAAGAAGATTAGGTCCTAATGCTGT
AGGTTACTATGGACTATTGCAAGCATTTGCTGATGCCTTAAAACTTTTATTAAAAGAATATGTAG
CTCCTACACAATCTAATATTGTTCTTTTCTTTTTAGGTCCTGTAATAACTTTAATTTTTGCATTATTA
GGTTACGCTGTTATACCCTATGGTCCTGGTTCAGGGATAAGCGACATGAATTTAGGTATATTTTA
CATGTTAGCTGTGTCATCTTTAGCTACATACGGTATTCTATTAGCTGGTTGAAGTGCGAATAGTA
AATACGCTTTTCTAGGTTCTCTTAGAAGTACAGCTCAATTAATTAGTTATGAATTAATATTAAGTT
CAGCTATATTAATAGTAATTATGATAACAGGAAATTTAAATTTAACTGTTTGTACTGAATCTCAAA
GAGCTATTTGATTTATACTACCTTTATTTCCTGTGTTTATAATATTTTTCATAGGATCTATAGCTGA
GACAAATAGAGCTCCTTTTGATTTAGCCGAGGCTAACCTGCTAATCTGGTTTGGTCTGGTTATAT
GTCACAAATTGCTAGGAAACCTTTTTATTTTAAAAACAAAAGACAATTAGCAGGAAACTTAATTT
AACCTAATTAAAAATATTAGATAATTAAACTCTTCATAGACTAAACGTGACAATTTAATATATATA
TATATTTATTTATATATATGATTAAATAAGATATAGTCAATCATCGGTGTGAATCGACTTAAAAAA
AAAAGCACATGGGTTAAACCCATCTCCCCTTATTAGGGGAATCAGAACTTGTTAGTGGGTTCAT
GACAGAGCATGCTGCCGTAGTTTTCGTATTCTTCTTTTTAGCTGAGTACGGTAGTATTGTACTAAT
GTGTATTTTAACTAGTATATTATTTATTGGTGGTTACTTATTATTTGAAATATCCTATGTTTTTACTG
TGGTAAATTATATTTTCTTTGAATTATTCTTTATAGACTGAGTAACATTTGTAGAGGTACAATCTTT
ATACACTGATTTTTTAAATAATTCTATCATTGAAGGATTATTATATGGGTTTAATCTAGGATTAAA
AAGTTCTTTAATGATATTCACATTTATTTGAGCTAGAGCATCCTTCCCTAGAATACGATTTGATCA
ACTAATGGGCTTCTGTTGAACAGTTTTATTACCTATTAATTTTGCAATTATTATATTAGTACCTTGT
GTTTTATATAGTTTTAACTTATTACCTGTAAATATACCATTGTTCTAGCTCACACACCCGCCCGCC
CTACTGCCACAAGGCTACAGTACATATGAGGAGGGGAACTAAAGATCTAGAACTATCCTAGTTA
ATAATTACACTTAATAGTATACTAAATAGACCATCTATCATACTCGAGAATAGTGATAGTGTAAT
TATACACTATTTTAACTGTATTCTACACTATTAGGATATTATTATCTTTATATGATAATAATTTTATA
ACTTAATTTATTAGGTTACATTAACAAAAAATTCGTTAACTTTTTTCACCACTTTTTAATACAAAAT
ACGAATTTTA

>YN477
ATAACAATTCTAAAGAACATAAAGAGTTATTAGATAAAAATAATTCACCTATACAGTTAATAAAT
CAACTTAAAGGGTATTTTTTCATAAATCCTTTATTAGCTTTAAGTTTAGCTATTACTATTTTCTCTTT
TGCAGGTATTCCTCCTCTTGTAGGGTTCTTTGCTAAACAGATGGTATTAAGCGCGGCTATTGATC
AAGGTTATATCTTTTTATCTTTAGTTGCAATATTAACTAGTGTTATAGGAGGGGTTTATTATTTAAA
TATAATTAAAGAAATGTTCTTTTATTCACCTGACTATAAATTAAACGAAGAAATTAAAAATAATAC
TATTAATGGTCAAATTATTAATAGAAACAATAAAATATTAAATGTTGAATTTAATTATACAAATGT
AGTTATGTCTAGTTCTGTGGCAATAACTATTTCTACTATTACATTAGTAGTTTTATTATTCATGTTT
ATGAATAAAGAATGATTAAGTCTGGGTACTATATTGGTACAATCTTTATTTAGCTATTAATGAGTA
GTATGACATTATTTATAGGGTTTGTATCTGTTATAGCTATTTTATTTTTAGCCATTAATTTTATATTT
GCTCCTCATAATCCTTATCAAGAAAAATATAGTATTTTCGAGTGTGGTTTCCATAGTTTTTTAGGG
CAAAATAGAACACAATTCGGTATAAAATTCTTTATTTTTGCTTTAGTTTATTTACTTTTAGATTTAG
AAATATTATTAACTTTCCCTTTCGCTCTTAGTGAGTATGTTAATGGTATTTATGGTCTTTTAGTTAC
TTTAATTTTTATAGCTATAATAACTATAGGATTTATATTTGAATTAGGTAAAAGCGCTCTTAAAATA
GACAGCAGACAAAAATTATATATACCTAAATTGAACGTTAATTACCATACAGAGTATGTTGGAAT
AGGTAAGGTTTCTAAGTAAAGTTATAGAGGCAGAAAACCAAAAAACCTACCAAAGGGTAGCTA
ATGGGAAGCTATTAAAAATAAAAGATGATAACCTATATATAGTATAGTTACTATATATTTACTATA
ACTAGGATTATTATATATATATATATTATCTTATTGTATATTAAGATTATTATTATTATAAGGTATA
ATTAATATAGTATCTTATTGTATAAGAATATAATATATTAACCTATAATTAATTTTATTTTTTAATTT
TTAATTATAATTTTTTTTTATATCTAGATGCTTACACATCTACAGATGTAGAAGAGAACAAAATAT
TGTTGTTATAGTAGTGGATGATATAGAAAAATATTTTTATATTATTTATTTTTGTTAGGTAGCTTTT
GAAGTGTTTGATAGAGAGGATATATGGACGGTAGGAGGGTATTCATTTTAATGAACAGTGGATA
GTTTAAATTAACCTAGTTATAGTTTATGAATTTAAATTATAATTATATTAATGTAGGTTATGATAGA
ATTATGTATTGATCCTTAAGAGTTAAGAGAGATACGCCCACGTATAATACATATTGGTTTAGGGT
TGAGTATATATACTTAAGAGTTAAGAATATATATATACAATATATAATAAATATAGTAATATATTA
ATGGTATGGACTTAACCAGGTTTATATATTATCATTTGATAAACATTAATTATAATTTTTCTATTTA
TTATTATTATTATACTGTGAGATTAATAATTATTAAAATATAATATTACATAGTACGTGATATTTGG
GGATTTTATCTTATTAATTGGCAATTAATGATTCTAATCAAATTTTATTCTCTTTAGTTTAATGGTA
GAACAATGATCTTCTAATTCATTGGTTTTAGTTCGATTCTAAAAAGAGATGAGTAAATAATTTTCT
AGATCAGAAATACTACTTTTAACTACAAAAAGCTTACGCTTTTTAACATTTTTTTGATAAATAACA
ACTATTGTTAATATTTGGCTGTCTATTGGTTTAACTAACAATTACAAAATTTTTTCAATTTTTATAAT
ATAAATTTAAATGAGAATATTAAAAAGTCATTCATTATTAAAATTAGTGAATTCTTACCTTATCGA
TGCGTCACAACCAAGTAACATTAGTTACTTGTGAAATTTTGGTTCATTATTAGCTGTTTGTTTAAT
AGTACAAATTATTACCGGTATTACATTAGCTATGCATTATAGTCCTAGTGTAATGGAAGCTTTTAA
CTCAATAGAGCATATAATGAGAGATGTTAATAACGGGTGATTAGTTCGTTATCTACATAGTAATA
CAGCTTCTGCTTTCTTTTTCTTAGTGTATTTACACATAGGAAGAGGTATATATTACGGATCATATA
GAGCTCCTCGTACTTTAGTTTGAGCTATTGGTACTGTTATATTAATATTAATGATGGCTATCGGTT
TCCTAGGTTATGTTTTACCTTATGGACAGATGTCATTATGAGGTGCTACAGTTATTACTAATCTTA
TTAGTGCTATACCTTGAATAGGGCAAGATATTGTTGAATTCATTTGAGGTGGTTTTTCTGTTAATA
ATGCCACTTTAAACAGATTTTTTGCATTACATTTTGTATTGCCTTTTGTATTAGCTGCTTTAGTTTTA
ATGCACTTAATTGCACTTCATGATACTGCTGGTTCAAGCAATCCTCTTGGTGTTTCAGGTAATTAC
GATAGAATTACATTTGCTCCATATTTTTTATTTAAAGATTTAATTACTATTTTTATATTTATTTTTGTA
TTAAGTGCTTTTGTATTCTTTATGCCTAATGTTTTAGGGGATAGTGATAATTATATTATGGCTAATC
CTATGCAAACTCCTGCTGCTATTGTACCTGAATGATACTTATTACCTTTCTATGCTATTTTAAGATC

TATACCTAATAAATTATTAGGTGTTATAGCGATGTTTAGTGCTATTTTAGCTATTATGTTATTACCT
GTTACAGATTTAGGTAGATCTAGAGGTTTACAATTTAGACCATTTAGTAAAATAGCTTTCTGAGTT
TTTGTTGCTAATTTCTTAGTTTTAATGCAATTAGGTGCTAAACACGTTGAAGATCCATTTATATTAT
TAGGTCAATTAAGTACTGTATTATACTTTAGTTATTTTGTTGCTATATTACCTTTAGCTAGTTACTT
AGATAATAGTTTAACTGATTTATCTAATAAATCTGAATTATTTTTAAATAAAACTAACTAAATATAT
TAAGATTATTATTTAATATATTTTCTATTTAAGATACTATTAATTTAGTATTTTGGGTTTTCAGTTTA
TAATTTATATTATATTATGCATTACCCTCCACCTTGCTTTGTAGTAAGCTAATCTGTTATTTCCTTTA
GTTTAATGGTAGAACAATGATCTTCTAATTCATTGGTTTTAGTTCGAATCTAAAAAGGAAATAAG
AAATATATTCTTATTATTACTTATATAATAATTATTTCTTAAAAATATACATTTTGCATTATAGCCGT
TTAGCTGTATTAAAATGTAAAATGATATAAAATAGAATAAATATTTAAATTATTCCTATGTTATATT
ATCCTATATTGCAACCATTATCAGAAGTTGTATTAATACTTGTACCTGCCTTATTAGCTGTAGCTT
ATGTTACAGTTGCTGAAAGAAAAACTATGGCTAGTATGCAAAGAAGATTAGGTCCTAATGCTGT
AGGTTACTATGGACTATTGCAAGCATTTGCTGATGCCTTAAAACTTTTATTAAAAGAATATGTAG
CTCCTACACAATCTAATATTGTTCTTTTCTTTTTAGGTCCTGTAATAACTTTAATTTTTGCATTATTA
GGTTACGCTGTTATACCCTATGGTCCTGGTTCAGGGATAAGCGACATGAATTTAGGTATATTTTA
CATGTTAGCTGTGTCATCTTTAGCTACATACGGTATTCTATTAGCTGGTTGAAGTGCGAATAGTA
AATACGCTTTTCTAGGTTCTCTTAGAAGTACAGCTCAATTAATTAGTTATGAATTAATATTAAGTT
CAGCTATATTAATAGTAATTATGATAACAGGAAATTTAAATTTAACTGTTTGTACTGAATCTCAAA
GAGCTATTTGATTTATACTACCTTTATTTCCTGTGTTTATAATATTTTTCATAGGATCTATAGCTGA
GACAAATAGAGCTCCTTTTGATTTAGCCGAGGCTAACCTGCTAATCTGGTTTGGTCTGGTTATAT
GTCACAAATTGCTAGGAAACCTTTTTATTTTAAAAACAAAAGACAATTAGCAGGAAACTTAATTT
AACCTAATTAAAAATATTAGATAATTAAACTCTTCATAGACTAAACGTGACAATTTAATATATATA
TATATTTATTTATATATATGATTAAATAAGATATAGTCAATCATCGGTGTGAATCGACTTAAAAAA
AAAAGCACATGGGTTAAACCCATCTCCCCTTATTAGGGGAATCAGAACTTGTTAGTGGGTTCAT
GACAGAGCATGCTGCCGTAGTTTTCGTATTCTTCTTTTTAGCTGAGTACGGTAGTATTGTACTAAT
GTGTATTTTAACTAGTATATTATTTATTGGTGGTTACTTATTATTTGAAATATCCTATGTTTTTACTG
TGGTAAATTATATTTTCTTTGAATTATTCTTTATAGACTGAGTAACATTTGTAGAGGTACAATCTTT
ATACACTGATTTTTTAAATAATTCTATCATTGAAGGATTATTATATGGGTTTAATCTAGGATTAAA
AAGTTCTTTAATGATATTCACATTTATTTGAGCTAGAGCATCCTTCCCTAGAATACGATTTGATCA
ACTAATGGGCTTCTGTTGAACAGTTTTATTACCTATTAATTTTGCAATTATTATATTAGTACCTTGT
GTTTTATATAGTTTTAACTTATTACCTGTAAATATACCATTGTTCTAGCTCACACACCCGCCCGCC
CTACTGCCACAAGGCTACAGTACATATGAGGAGGGGAACTAAAGATCTAGAACTATCCTAGTTA
ATAATTACACTTAATAGTATACTAAATAGACCATCTATCATACTCGAGAATAGTGATAGTGTAAT
TATACACTATTTTAACTGTATTCTACACTATTAGGATATTATTATCTTTATATGATAATAATTTTATA
ACTTAATTTATTAGGTTACATTAACAAAAAATTCGTTAACTTTTTTCACCACTTTTTAATACAAAAT
ACGAATTTTA
>YN480
ATAACAATTCTAAAGAACATAAAGAGTTATTAGATAAAAATAATTCACCTATACAGTTAATAAAT
CAACTTAAAGGGTATTTTTTCATAAATCCTTTATTAGCTTTAAGTTTAGCTATTACTATTTTCTCTTT
TGCAGGTATTCCTCCTCTTGTAGGGTTCTTTGCTAAACAGATGGTATTAAGCGCGGCTATTGATC
AAGGTTATATCTTTTTATCTTTAGTTGCAATATTAACTAGTGTTATAGGAGGGGTTTATTATTTAAA
TATAATTAAAGAAATGTTCTTTTATTCACCTGACTATAAATTAAACGAAGAAATTAAAAATAATAC
TATTAATGGTCAAATTATTAATAGAAACAATAAAATATTAAATGTTGAATTTAATTATACAAATGT
AGTTATGTCTAGTTCTGTGGCAATAACTATTTCTACTATTACATTAGTAGTTTTATTATTCATGTTT

ATGAATAAAGAATGATTAAGTCTGGGTACTATATTGGTACAATCTTTATTTAGCTATTAATGAGTA
GTATGACATTATTTATAGGGTTTGTATCTGTTATAGCTATTTTATTTTTAGCCATTAATTTTATATTT
GCTCCTCATAATCCTTATCAAGAAAAATATAGTATTTTCGAGTGTGGTTTCCATAGTTTTTTAGGG
CAAAATAGAACACAATTCGGTATAAAATTCTTTATTTTTGCTTTAGTTTATTTACTTTTAGATTTAG
AAATATTATTAACTTTCCCTTTCGCTCTTAGTGAGTATGTTAATGGTATTTATGGTCTTTTAGTTAC
TTTAATTTTTATAGCTATAATAACTATAGGATTTATATTTGAATTAGGTAAAAGCGCTCTTAAAATA
GACAGCAGACAAAAATTATATATACCTAAATTGAACGTTAATTACCATACAGAGTATGTTGGAAT
AGGTAAGGTTTCTAAGTAAAGTTATAGAGGCAGAAAACCAAAAAACCTACCAAAGGGTAGCTA
ATGGGAAGCTATTAAAAATAAAAGATGATAACCTATATATAGTATAGTTACTATATATTTACTATA
ACTAGGATTATTATATATATATATATTATCTTATTGTATATTAAGATTATTATTATTATAAGGTATA
ATTAATATAGTATCTTATTGTATAAGAATATAATATATTAACCTATAATTAATTTTATTTTTTAATTT
TTAATTATAATTTTTTTTTATATCTAGATGCTTACACATCTACAGATGTAGAAGAGAACAAAATAT
TGTTGTTATAGTAGTGGATGATATAGAAAAATATTTTTATATTATTTATTTTTGTTAGGTAGCTTTT
GAAGTGTTTGATAGAGAGGATATATGGACGGTAGGAGGGTATTCATTTTAATGAACAGTGGATA
GTTTAAATTAACCTAGTTATAGTTTATGAATTTAAATTATAATTATATTAATGTAGGTTATGATAGA
ATTATGTATTGATCCTTAAGAGTTAAGAGAGATACGCCCACGTATAATACATATTGGTTTAGGGT
TGAGTATATATACTTAAGAGTTAAGAATATATATATACAATATATAATAAATATAGTAATATATTA
ATGGTATGGACTTAACCAGGTTTATATATTATCATTTGATAAACATTAATTATAATTTTTCTATTTA
TTATTATTATTATACTGTGAGATTAATAATTATTAAAATATAATATTACATAGTACGTGATATTTGG
GGATTTTATCTTATTAATTGGCAATTAATGATTCTAATCAAATTTTATTCTCTTTAGTTTAATGGTA
GAACAATGATCTTCTAATTCATTGGTTTTAGTTCGATTCTAAAAAGAGATGAGTAAATAATTTTCT
AGATCAGAAATACTACTTTTAACTACAAAAAGCTTACGCTTTTTAACATTTTTTTGATAAATAACA
ACTATTGTTAATATTTGGCTGTCTATTGGTTTAACTAACAATTACAAAATTTTTTCAATTTTTATAAT
ATAAATTTAAATGAGAATATTAAAAAGTCATTCATTATTAAAATTAGTGAATTCTTACCTTATCGA
TGCGTCACAACCAAGTAACATTAGTTACTTGTGAAATTTTGGTTCATTATTAGCTGTTTGTTTAAT
AGTACAAATTATTACCGGTATTACATTAGCTATGCATTATAGTCCTAGTGTAATGGAAGCTTTTAA
CTCAATAGAGCATATAATGAGAGATGTTAATAACGGGTGATTAGTTCGTTATCTACATAGTAATA
CAGCTTCTGCTTTCTTTTTCTTAGTGTATTTACACATAGGAAGAGGTATATATTACGGATCATATA
GAGCTCCTCGTACTTTAGTTTGAGCTATTGGTACTGTTATATTAATATTAATGATGGCTATCGGTT
TCCTAGGTTATGTTTTACCTTATGGACAGATGTCATTATGAGGTGCTACAGTTATTACTAATCTTA
TTAGTGCTATACCTTGAATAGGGCAAGATATTGTTGAATTCATTTGAGGTGGTTTTTCTGTTAATA
ATGCCACTTTAAACAGATTTTTTGCATTACATTTTGTATTGCCTTTTGTATTAGCTGCTTTAGTTTTA
ATGCACTTAATTGCACTTCATGATACTGCTGGTTCAAGCAATCCTCTTGGTGTTTCAGGTAATTAC
GATAGAATTACATTTGCTCCATATTTTTTATTTAAAGATTTAATTACTATTTTTATATTTATTTTTGTA
TTAAGTGCTTTTGTATTCTTTATGCCTAATGTTTTAGGGGATAGTGATAATTATATTATGGCTAATC
CTATGCAAACTCCTGCTGCTATTGTACCTGAATGATACTTATTACCTTTCTATGCTATTTTAAGATC
TATACCTAATAAATTATTAGGTGTTATAGCGATGTTTAGTGCTATTTTAGCTATTATGTTATTACCT
GTTACAGATTTAGGTAGATCTAGAGGTTTACAATTTAGACCATTTAGTAAAATAGCTTTCTGAGTT
TTTGTTGCTAATTTCTTAGTTTTAATGCAATTAGGTGCTAAACACGTTGAAGATCCATTTATATTAT
TAGGTCAATTAAGTACTGTATTATACTTTAGTTATTTTGTTGCTATATTACCTTTAGCTAGTTACTT
AGATAATAGTTTAACTGATTTATCTAATAAATCTGAATTATTTTTAAATAAAACTAACTAAATATAT
TAAGATTATTATTTAATATATTTTCTATTTAAGATACTATTAATTTAGTATTTTGGGTTTTCAGTTTA
TAATTTATATTATATTATGCATTACCCTCCACCTTGCTTTGTAGTAAGCTAATCTGTTATTTCCTTTA
GTTTAATGGTAGAACAATGATCTTCTAATTCATTGGTTTTAGTTCGAATCTAAAAAGGAAATAAG

AAATATATTCTTATTATTACTTATATAATAATTATTTCTTAAAAATATACATTTTGCATTATAGCCGT
TTAGCTGTATTAAAATGTAAAATGATATAAAATAGAATAAATATTTAAATTATTCCTATGTTATATT
ATCCTATATTGCAACCATTATCAGAAGTTGTATTAATACTTGTACCTGCCTTATTAGCTGTAGCTT
ATGTTACAGTTGCTGAAAGAAAAACTATGGCTAGTATGCAAAGAAGATTAGGTCCTAATGCTGT
AGGTTACTATGGACTATTGCAAGCATTTGCTGATGCCTTAAAACTTTTATTAAAAGAATATGTAG
CTCCTACACAATCTAATATTGTTCTTTTCTTTTTAGGTCCTGTAATAACTTTAATTTTTGCATTATTA
GGTTACGCTGTTATACCCTATGGTCCTGGTTCAGGGATAAGCGACATGAATTTAGGTATATTTTA
CATGTTAGCTGTGTCATCTTTAGCTACATACGGTATTCTATTAGCTGGTTGAAGTGCGAATAGTA
AATACGCTTTTCTAGGTTCTCTTAGAAGTACAGCTCAATTAATTAGTTATGAATTAATATTAAGTT
CAGCTATATTAATAGTAATTATGATAACAGGAAATTTAAATTTAACTGTTTGTACTGAATCTCAAA
GAGCTATTTGATTTATACTACCTTTATTTCCTGTGTTTATAATATTTTTCATAGGATCTATAGCTGA
GACAAATAGAGCTCCTTTTGATTTAGCCGAGGCTAACCTGCTAATCTGGTTTGGTCTGGTTATAT
GTCACAAATTGCTAGGAAACCTTTTTATTTTAAAAACAAAAGACAATTAGCAGGAAACTTAATTT
AACCTAATTAAAAATATTAGATAATTAAACTCTTCATAGACTAAACGTGACAATTTAATATATATA
TATATTTATTTATATATATGATTAAATAAGATATAGTCAATCATCGGTGTGAATCGACTTAAAAAA
AAAAGCACATGGGTTAAACCCATCTCCCCTTATTAGGGGAATCAGAACTTGTTAGTGGGTTCAT
GACAGAGCATGCTGCCGTAGTTTTCGTATTCTTCTTTTTAGCTGAGTACGGTAGTATTGTACTAAT
GTGTATTTTAACTAGTATATTATTTATTGGTGGTTACTTATTATTTGAAATATCCTATGTTTTTACTG
TGGTAAATTATATTTTCTTTGAATTATTCTTTATAGACTGAGTAACATTTGTAGAGGTACAATCTTT
ATACACTGATTTTTTAAATAATTCTATCATTGAAGGATTATTATATGGGTTTAATCTAGGATTAAA
AAGTTCTTTAATGATATTCACATTTATTTGAGCTAGAGCATCCTTCCCTAGAATACGATTTGATCA
ACTAATGGGCTTCTGTTGAACAGTTTTATTACCTATTAATTTTGCAATTATTATATTAGTACCTTGT
GTTTTATATAGTTTTAACTTATTACCTGTAAATATACCATTGTTCTAGCTCACACACCCGCCCGCC
CTACTGCCACAAGGCTACAGTACATATGAGGAGGGGAACTAAAGATCTAGAACTATCCTAGTTA
ATAATTACACTTAATAGTATACTAAATAGACCATCTATCATACTCGAGAATAGTGATAGTGTAAT
TATACACTATTTTAACTGTATTCTACACTATTAGCATATTATTATCTTTATATGATAATAATTTTATA
ACTTAATTTATTAGGTTACATTAACAAAAAATTCGTTAACTTTTTTCACCACTTTTTAATACAAAAT
ACGAATTTTA
>YN485
ATAACAATTCTAAAGAACATAAAGAGTTATTAGATAAAAATAATTCACCTATACAGTTAATAAAT
CAACTTAAAGGGTATTTTTTCATAAATCCTTTATTAGCTTTAAGTTTAGCTATTACTATTTTCTCTTT
TGCAGGTATTCCTCCTCTTGTAGGGTTCTTTGCTAAACAGATGGTATTAAGCGCGGCTATTGATC
AAGGTTATATCTTTTTATCTTTAGTTGCAATATTAACTAGTGTTATAGGAGGGGTTTATTATTTAAA
TATAATTAAAGAAATGTTCTTTTATTCACCTGACTATAAATTAAACGAAGAAATTAAAAATAATAC
TATTAATGGTCAAATTATTAATAGAAACAATAAAATATTAAATGTTGAATTTAATTATACAAATGT
AGTTATGTCTAGTTCTGTGGCAATAACTATTTCTACTATTACATTAGTAGTTTTATTATTCATGTTT
ATGAATAAAGAATGATTAAGTCTGGGTACTATATTGGTACAATCTTTATTTAGCTATTAATGAGTA
GTATGACATTATTTATAGGGTTTGTATCTGTTATAGCTATTTTATTTTTAGCCATTAATTTTATATTT
GCTCCTCATAATCCTTATCAAGAAAAATATAGTATTTTCGAGTGTGGTTTCCATAGTTTTTTAGGG
CAAAATAGAACACAATTCGGTATAAAATTCTTTATTTTTGCTTTAGTTTATTTACTTTTAGATTTAG
AAATATTATTAACTTTCCCTTTCGCTCTTAGTGAGTATGTTAATGGTATTTATGGTCTTTTAGTTAC
TTTAATTTTTATAGCTATAATAACTATAGGATTTATATTTGAATTAGGTAAAAGCGCTCTTAAAATA
GACAGCAGACAAAAATTATATATACCTAAATTGAACGTTAATTACCATACAGAGTATGTTGGAAT
AGGTAAGGTTTCTAAGTAAAGTTATAGAGGCAGAAAACCAAAAAACCTACCAAAGGGTAGCTA

ATGGGAAGCTATTAAAAATAAAAGATGATAACCTATATATAGTATAGTTACTATATATTTACTATA
ACTAGGATTATTATAAATATATATATTATCTTATTGTATATTAAGATTATTATTATTATAAGGTATA
ATTAATATAGTATCTTATTGTATAAGAATATAATATATTAACCTATAATTAATTTTATTTTTTAATTT
TTAATTATAATTTTTTTTTATATCTAGATGCTTACACATCTACAGATGTAGAAGAGAACAAAATAT
TGTTGTTATAGTAGTGGATGATATAGAAAAATATTTTTATATTATTTATTTTTGTTAGGTAGCTTTT
GAAGTGTTTGATAGAGAGGATATATGGACGGTAGGAGGGTATTCATTTTAATGAACAGTGGATA
GTTTAAATTAACCTAGTTATAGTTTATGAATTTAAATTATAATTATATTAATGTAGGTTATGATAGA
ATTATGTATTGATCCTTAAGAGTTAAGAGAGATACGCCCACGTATAATACATATTGGTTTAGGGT
TGAGTATATATACTTAAGAGTTAAGAATATATATATACAATATATAATAAATATAGTAATATATTA
ATGGTATGGACTTAACCAGGTTTATATATTATCATTTGATAAACATTAATTATAATTTTTCTATTTA
TTATTATTATTATACTGTGAGATTAATAATTATTAAAATATAATATTACATAGTACGTGATATTTGG
GGATTTTATCTTATTAATTGGCAATTAATGATTCTAATCAAATTTTATTCTCTTTAGTTTAATGGTA
GAACAATGATCTTCTAATTCATTGGTTTTAGTTCGATTCTAAAAAGAGATGAGTAAATAATTTTCT
AGATCAGAAATACTACTTTTAACTACAAAAAGCTTACGCTTTTTAACATTTTTTTGATAAATAACA
ACTATTGTTAATATTTGGCTGTCTATTGGTTTAACTAACAATTACAAAATTTTTTCAATTTTTATAAT
ATAAATTTAAATGAGAATATTAAAAAGTCATTCATTATTAAAATTAGTGAATTCTTACCTTATCGA
TGCGTCACAACCAAGTAACATTAGTTACTTGTGAAATTTTGGTTCATTATTAGCTGTTTGTTTAAT
AGTACAAATTATTACCGGTATTACATTAGCTATGCATTATAGTCCTAGTGTAATGGAAGCTTTTAA
CTCAATAGAGCATATAATGAGAGATGTTAATAACGGGTGATTAGTTCGTTATCTACATAGTAATA
CAGCTTCTGCTTTCTTTTTCTTAGTGTATTTACACATAGGAAGAGGTATATATTACGGATCATATA
GAGCTCCTCGTACTTTAGTTTGAGCTATTGGTACTGTTATATTAATATTAATGATGGCTATCGGTT
TCCTAGGTTATGTTTTACCTTATGGACAGATGTCATTATGAGGTGCTACAGTTATTACTAATCTTA
TTAGTGCTATACCTTGAATAGGGCAAGATATTGTTGAATTCATTTGAGGTGGTTTTTCTGTTAATA
ATGCCACTTTAAACAGATTTTTTGCATTACATTTTGTATTGCCTTTTGTATTAGCTGCTTTAGTTTTA
ATGCACTTAATTGCACTTCATGATACTGCTGGTTCAAGCAATCCTCTTGGTGTTTCAGGTAATTAC
GATAGAATTACATTTGCTCCATATTTTTTATTTAAAGATTTAATTACTATTTTTATATTTATTTTTGTA
TTAAGTGCTTTTGTATTCTTTATGCCTAATGTTTTAGGGGATAGTGATAATTATATTATGGCTAATC
CTATGCAAACTCCTGCTGCTATTGTACCTGAATGATACTTATTACCTTTCTATGCTATTTTAAGATC
TATACCTAATAAATTATTAGGTGTTATAGCGATGTTTAGTGCTATTTTAGCTATTATGTTATTACCT
GTTACAGATTTAGGTAGATCTAGAGGTTTACAATTTAGACCATTTAGTAAAATAGCTTTCTGAGTT
TTTGTTGCTAATTTCTTAGTTTTAATGCAATTAGGTGCTAAACACGTTGAAGATCCATTTATATTAT
TAGGTCAATTAAGTACTGTATTATACTTTAGTTATTTTGTTGCTATATTACCTTTAGCTAGTTACTT
AGATAATAGTTTAACTGATTTATCTAATAAATCTGAATTATTTTTAAATAAAACTAACTAAATATAT
TAAGATTATTATTTAATATATTTTCTATTTAAGATACTATTAATTTAGTATTTTGGGTTTTCAGTTTA
TAATTTATATTATATTATGCATTACCCTCCACCTTGCTTTGTAGTAAGCTAATCTGTTATTTCCTTTA
GTTTAATGGTAGAACAATGATCTTCTAATTCATTGGTTTTAGTTCGAATCTAAAAAGGAAATAAG
AAATATATTCTTATTATTACTTATATAATAATTATTTCTTAAAAATATACATTTTGCATTATAGCCGT
TTAGCTGTATTAAAATGTAAAATGATATAAAATAGAATAAATATTTAAATTATTCCTATGTTATATT
ATCCTATATTGCAACCATTATCAGAAGTTGTATTAATACTTGTACCTGCCTTATTAGCTGTAGCTT
ATGTTACAGTTGCTGAAAGAAAAACTATGGCTAGTATGCAAAGAAGATTAGGTCCTAATGCTGT
AGGTTACTATGGACTATTGCAAGCATTTGCTGATGCCTTAAAACTTTTATTAAAAGAATATGTAG
CTCCTACACAATCTAATATTGTTCTTTTCTTTTTAGGTCCTGTAATAACTTTAATTTTTGCATTATTA
GGTTACGCTGTTATACCCTATGGTCCTGGTTCAGGGATAAGCGACATGAATTTAGGTATATTTTA
CATGTTAGCTGTGTCATCTTTAGCTACATACGGTATTCTATTAGCTGGTTGAAGTGCGAATAGTA

AATACGCTTTTCTAGGTTCTCTTAGAAGTACAGCTCAATTAATTAGTTATGAATTAATATTAAGTT
CAGCTATATTAATAGTAATTATGATAACAGGAAATTTAAATTTAACTGTTTGTACTGAATCTCAAA
GAGCTATTTGATTTATACTACCTTTATTTCCTGTGTTTATAATATTTTTCATAGGATCTATAGCTGA
GACAAATAGAGCTCCTTTTGATTTAGCCGAGGCTAACCTGCTAATCTGGTTTGGTCTGGTTATAT
GTCACAAATTGCTAGGAAACCTTTTTATTTTAAAAACAAAAGACAATTAGCAGGAAACTTAATTT
AACCTAATTAAAAATATTAGATAATTAAACTCTTCATAGACTAAACGTGACAATTTAATATATATA
TATATTTATTTATATATATGATTAAATAAGATATAGTCAATCATCGGTGTGAATCGACTTAAAAAA
AAAAGCACATGGGTTAAACCCATCTCCCCTTATTAGGGGAATCAGAACTTGTTAGTGGGTTCAT
GACAGAGCATGCTGCCGTAGTTTTCGTATTCTTCTTTTTAGCTGAGTACGGTAGTATTGTACTAAT
GTGTATTTTAACTAGTATATTATTTATTGGTGGTTACTTATTATTTGAAATATCCTATGTTTTTACTG
TGGTAAATTATATTTTCTTTGAATTATTCTTTATAGACTGAGTAACATTTGTAGAGGTACAATCTTT
ATACACTGATTTTTTAAATAATTCTATCATTGAAGGATTATTATATGGGTTTAATCTAGGATTAAA
AAGTTCTTTAATGATATTCACATTTATTTGAGCTAGAGCATCCTTCCCTAGAATACGATTTGATCA
ACTAATGGGCTTCTGTTGAACAGTTTTATTACCTATTAATTTTGCAATTATTATATTAGTACCTTGT
GTTTTATATAGTTTTAACTTATTACCTGTAAATATACCATTGTTCTAGCTCACACACCCGCCCGCC
CTACTGCCACAAGGCTACAGTACATATGAGGAGGGGAACTAAAGATCTAGAACTATCCTAGTTA
ATAATTACACTTAATAGTATACTAAATAGACCATCTATCATACTCGAGAATAGTGATAGTGTAAT
TATACACTATTTTAACTGTATTCTACACTATTAGCATATTATTATCTTTATATGATAATAATTTTATA
ACTTAATTTATTAGGTTACATTAACAAAAAAATTCGTTAACTTTTTTCACCACTTTTTAATACAAAAT
ACGAATTTTA
>YN487
ATAACAATTCTAAAGAACATAAAGAGTTATTAGATAAAAATAATTCACCTATACAGTTAATAAAT
CAACTTAAAGGGTATTTTTTCATAAATCCTTTATTAGCTTTAAGTTTAGCTATTACTATTTTCTCTTT
TGCAGGTATTCCTCCTCTTGTAGGGTTCTTTGCTAAACAGATGGTATTAAGCGCGGCTATTGATC
AAGGTTATATCTTTTTATCTTTAGTTGCAATATTAACTAGTGTTATAGGAGGGGTTTATTATTTAAA
TATAATTAAAGAAATGTTCTTTTATTCACCTGACTATAAATTAAACGAAGAAATTAAAAATAATAC
TATTAATGGTCAAATTATTAATAGAAACAATAAAATATTAAATGTTGAATTTAATTATACAAATGT
AGTTATGTCTAGTTCTGTGGCAATAACTATTTCTACTATTACATTAGTAGTTTTATTATTCATGTTT
ATGAATAAAGAATGATTAAGTCTGGGTACTATATTGGTACAATCTTTATTTAGCTATTAATGAGTA
GTATGACATTATTTATAGGGTTTGTATCTGTTATAGCTATTTTATTTTTAGCCATTAATTTTATATTT
GCTCCTCATAATCCTTATCAAGAAAAATATAGTATTTTCGAGTGTGGTTTCCATAGTTTTTTAGGG
CAAAATAGAACACAATTCGGTATAAAATTCTTTATTTTTGCTTTAGTTTATTTACTTTTAGATTTAG
AAATATTATTAACTTTCCCTTTCGCTCTTAGTGAGTATGTTAATGGTATTTATGGTCTTTTAGTTAC
TTTAATTTTTATAGCTATAATAACTATAGGATTTATATTTGAATTAGGTAAAAGCGCTCTTAAAATA
GACAGCAGACAAAAATTATATATACCTAAATTGAACGTTAATTACCATACAGAGTATGTTGGAAT
AGGTAAGGTTTCTAAGTAAAGTTATAGAGGCAGAAAACCAAAAAACCTACCAAAGGGTAGCTA
ATGGGAAGCTATTAAAAATAAAAGATGATAACCTATATATAGTATAGTTACTATATATTTACTATA
ACTAGGATTATTATATATATATATATTATCTTATTGTATATTAAGATTATTATTATTATAAGGTATA
ATTAATATAGTATCTTATTGTATAAGAATATAATATATTAACCTATAATTAATTTTATTTTTTAATTT
TTAATTATAATTTTTTTTTATATCTAGATGCTTACACATCTACAGATGTAGAAGAGAACAAAATAT
TGTTGTTATAGTAGTGGATGATATAGAAAAATATTTTTATATTATTTATTTTTGTTAGGTAGCTTTT
GAAGTGTTTGATAGAGAGGATATATGGACGGTAGGAGGGTATTCATTTTAATGAACAGTGGATA
GTTTAAATTAACCTAGTTATAGTTTATGAATTTAAATTATAATTATATTAATGTAGGTTATGATAGA
ATTATGTATTGATCCTTAAGAGTTAAGAGAGATACGCCCACGTATAATACATATTGGTTTAGGGT

TGAGTATATATACTTAAGAGTTAAGAATATATATATACAATATATAATAAATATAGTAATATATTA
ATGGTATGGACTTAACCAGGTTTATATATTATCATTTGATAAACATTAATTATAATTTTTCTATTTA
TTATTATTATTATACTGTGAGATTAATAATTATTAAAATATAATATTACATAGTACGTGATATTTGG
GGATTTTATCTTATTAATTGGCAATTAATGATTCTAATCAAATTTTATTCTCTTTAGTTTAATGGTA
GAACAATGATCTTCTAATTCATTGGTTTTAGTTCGATTCTAAAAAGAGATGAGTAAATAATTTTCT
AGATCAGAAATACTACTTTTAACTACAAAAAGCTTACGCTTTTTAACATTTTTTTGATAAATAACA
ACTATTGTTAATATTTGGCTGTCTATTGGTTTAACTAACAATTACAAAATTTTTTCAATTTTTATAAT
ATAAATTTAAATGAGAATATTAAAAAGTCATTCATTATTAAAATTAGTGAATTCTTACCTTATCGA
TGCGTCACAACCAAGTAACATTAGTTACTTGTGAAATTTTGGTTCATTATTAGCTGTTTGTTTAAT
AGTACAAATTATTACCGGTATTACATTAGCTATGCATTATAGTCCTAGTGTAATGGAAGCTTTTAA
CTCAATAGAGCATATAATGAGAGATGTTAATAACGGGTGATTAGTTCGTTATCTACATAGTAATA
CAGCTTCTGCTTTCTTTTTCTTAGTGTATTTACACATAGGAAGAGGTATATATTACGGATCATATA
GAGCTCCTCGTACTTTAGTTTGAGCTATTGGTACTGTTATATTAATATTAATGATGGCTATCGGTT
TCCTAGGTTATGTTTTACCTTATGGACAGATGTCATTATGAGGTGCTACAGTTATTACTAATCTTA
TTAGTGCTATACCTTGAATAGGGCAAGATATTGTTGAATTCATTTGAGGTGGTTTTTCTGTTAATA
ATGCCACTTTAAACAGATTTTTTGCATTACATTTTGTATTGCCTTTTGTATTAGCTGCTTTAGTTTTA
ATGCACTTAATTGCACTTCATGATACTGCTGGTTCAAGCAATCCTCTTGGTGTTTCAGGTAATTAC
GATAGAATTACATTTGCTCCATATTTTTTATTTAAAGATTTAATTACTATTTTTATATTTATTTTTGTA
TTAAGTGCTTTTGTATTCTTTATGCCTAATGTTTTAGGGGATAGTGATAATTATATTATGGCTAATC
CTATGCAAACTCCTGCTGCTATTGTACCTGAATGATACTTATTACCTTTCTATGCTATTTTAAGATC
TATACCTAATAAATTATTAGGTGTTATAGCGATGTTTAGTGCTATTTTAGCTATTATGTTATTACCT
GTTACAGATTTAGGTAGATCTAGAGGTTTACAATTTAGACCATTTAGTAAAATAGCTTTCTGAGTT
TTTGTTGCTAATTTCTTAGTTTTAATGCAATTAGGTGCTAAACACGTTGAAGATCCATTTATATTAT
TAGGTCAATTAAGTACTGTATTATACTTTAGTTATTTTGTTGCTATATTACCTTTAGCTAGTTACTT
AGATAATAGTTTAACTGATTTATCTAATAAATCTGAATTATTTTTAAATAAAACTAACTAAATATAT
TAAGATTATTATTTAATATATTTTCTATTTAAGATACTATTAATTTAGTATTTTGGGTTTTCAGTTTA
TAATTTATATTATATTATGCATTACCCTCCACCTTGCTTTGTAGTAAGCTAATCTGTTATTTCCTTTA
GTTTAATGGTAGAACAATGATCTTCTAATTCATTGGTTTTAGTTCGAATCTAAAAAGGAAATAAG
AAATATATTCTTATTATTACTTATATAATAATTATTTCTTAAAAATATACATTTTGCATTATAGCCGT
TTAGCTGTATTAAAATGTAAAATGATATAAAATAGAATAAATATTTAAATTATTCCTATGTTATATT
ATCCTATATTGCAACCATTATCAGAAGTTGTATTAATACTTGTACCTGCCTTATTAGCTGTAGCTT
ATGTTACAGTTGCTGAAAGAAAAACTATGGCTAGTATGCAAAGAAGATTAGGTCCTAATGCTGT
AGGTTACTATGGACTATTGCAAGCATTTGCTGATGCCTTAAAACTTTTATTAAAAGAATATGTAG
CTCCTACACAATCTAATATTGTTCTTTTCTTTTTAGGTCCTGTAATAACTTTAATTTTTGCATTATTA
GGTTACGCTGTTATACCCTATGGTCCTGGTTCAGGGATAAGCGACATGAATTTAGGTATATTTTA
CATGTTAGCTGTGTCATCTTTAGCTACATACGGTATTCTATTAGCTGGTTGAAGTGCGAATAGTA
AATACGCTTTTCTAGGTTCTCTTAGAAGTACAGCTCAATTAATTAGTTATGAATTAATATTAAGTT
CAGCTATATTAATAGTAATTATGATAACAGGAAATTTAAATTTAACTGTTTGTACTGAATCTCAAA
GAGCTATTTGATTTATACTACCTTTATTTCCTGTGTTTATAATATTTTTCATAGGATCTATAGCTGA
GACAAATAGAGCTCCTTTTGATTTAGCCGAGGCTAACCTGCTAATCTGGTTTGGTCTGGTTATAT
GTCACAAATTGCTAGGAAACCTTTTTATTTTAAAAACAAAAGACAATTAGCAGGAAACTTAATTT
AACCTAATTAAAAATATTAGATAATTAAACTCTTCATAGACTAAACGTGACAATTTAATATATATA
TATATTTATTTATATATATGATTAAATAAGATATAGTCAATCATCGGTGTGAATCGACTTAAAAAA
AAAAGCACATGGGTTAAACCCATCTCCCCTTATTAGGGGAATCAGAACTTGTTAGTGGGTTCAT

GACAGAGCATGCTGCCGTAGTTTTCGTATTCTTCTTTTTAGCTGAGTACGGTAGTATTGTACTAAT
GTGTATTTTAACTAGTATATTATTTATTGGTGGTTACTTATTATTTGAAATATCCTATGTTTTTACTG
TGGTAAATTATATTTTCTTTGAATTATTCTTTATAGACTGAGTAACATTTGTAGAGGTACAATCTTT
ATACACTGATTTTTTAAATAATTCTATCATTGAAGGATTATTATATGGGTTTAATCTAGGATTAAA
AAGTTCTTTAATGATATTCACATTTATTTGAGCTAGAGCATCCTTCCCTAGAATACGATTTGATCA
ACTAATGGGCTTCTGTTGAACAGTTTTATTACCTATTAATTTTGCAATTATTATATTAGTACCTTGT
GTTTTATATAGTTTTAACTTATTACCTGTAAATATACCATTGTTCTAGCTCACACACCCGCCCGCC
CTACTGCCACAAGGCTACAGTACATATGAGGAGGGGAACTAAAGATCTAGAACTATCCTAGTTA
ATAATTACACTTAATAGTATACTAAATAGACCATCTATCATACTCGAGAATAGTGATAGTGTAAT
TATACACTATTTTAACTGTATTCTACACTATTAGCATATTATTATCTTTATATGATAATAATTTTATA
ACTTAATTTATTAGGTTACATTAACAAAAAATTCGTTAACTTTTTTCACCACTTTTTAATACAAAAT
ACGAATTTTA
>YN490
ATAACAATTCTAAAGAACATAAAGAGTTATTAGATAAAAATAATTCACCTATACAGTTAATAAAT
CAACTTAAAGGGTATTTTTTCATAAATCCTTTATTAGCTTTAAGTTTAGCTATTACTATTTTCTCTTT
TGCAGGTATTCCTCCTCTTGTAGGGTTCTTTGCTAAACAGATGGTATTAAGCGCGGCTATTGATC
AAGGTTATATCTTTTTATCTTTAGTTGCAATATTAACTAGTGTTATAGGAGGGGTTTATTATTTAAA
TATAATTAAAGAAATGTTCTTTTATTCACCTGACTATAAATTAAACGAAGAAATTAAAAATAATAC
TATTAATGGTCAAATTATTAATAGAAACAATAAAATATTAAATGTTGAATTTAATTATACAAATGT
AGTTATGTCTAGTTCTGTGGCAATAACTATTTCTACTATTACATTAGTAGTTTTATTATTCATGTTT
ATGAATAAAGAATGATTAAGTCTGGGTACTATATTGGTACAATCTTTATTTAGCTATTAATGAGTA
GTATGACATTATTTATAGGGTTTGTATCTGTTATAGCTATTTTATTTTTAGCCATTAATTTTATATTT
GCTCCTCATAATCCTTATCAAGAAAAATATAGTATTTTCGAGTGTGGTTTCCATAGTTTTTTAGGG
CAAAATAGAACACAATTCGGTATAAAATTCTTTATTTTTGCTTTAGTTTATTTACTTTTAGATTTAG
AAATATTATTAACTTTCCCTTTCGCTCTTAGTGAGTATGTTAATGGTATTTATGGTCTTTTAGTTAC
TTTAATTTTTATAGCTATAATAACTATAGGATTTATATTTGAATTAGGTAAAAGCGCTCTTAAAATA
GACAGCAGACAAAAATTATATATACCTAAATTGAACGTTAATTACCATACAGAGTATGTTGGAAT
AGGTAAGGTTTCTAAGTAAAGTTATAGAGGCAGAAAACCAAAAAACCTACCAAAGGGTAGCTA
ATGGGAAGCTATTAAAAATAAAAGATGATAACCTATATATAGTATAGTTACTATATATTTACTATA
ACTAGGATTATTATATATATATATATTATCTTATTGTATATTAAGATTATTATTATTATAAGGTATA
ATTAATATAGTATCTTATTGTATAAGAATATAATATATTAACCTATAATTAATTTTATTTTTTAATTT
TTAATTATAATTTTTTTTTATATCTAGATGCTTACACATCTACAGATGTAGAAGAGAACAAAATAT
TGTTGTTATAGTAGTGGATGATATAGAAAAATATTTTTATATTATTTATTTTTGTTAGGTAGCTTTT
GAAGTGTTTGATAGAGAGGATATATGGACGGTAGGAGGGTATTCATTTTAATGAACAGTGGATA
GTTTAAATTAACCTAGTTATAGTTTATGAATTTAAATTATAATTATATTAATGTAGGTTATGATAGA
ATTATGTATTGATCCTTAAGAGTTAAGAGAGATACGCCCACGTATAATACATATTGGTTTAGGGT
TGAGTATATATACTTAAGAGTTAAGAATATATATATACAATATATAATAAATATAGTAATATATTA
ATGGTATGGACTTAACCAGGTTTATATATTATCATTTGATAAACATTAATTATAATTTTTCTATTTA
TTATTATTATTATACTGTGAGATTAATAATTATTAAAATATAATATTACATAGTACGTGATATTTGG
GGATTTTATCTTATTAATTGGCAATTAATGATTCTAATCAAATTTTATTCTCTTTAGTTTAATGGTA
GAACAATGATCTTCTAATTCATTGGTTTTAGTTCGATTCTAAAAAGAGATGAGTAAATAATTTTCT
AGATCAGAAATACTACTTTTAACTACAAAAAGCTTACGCTTTTTAACATTTTTTTGATAAATAACA
ACTATTGTTAATATTTGGCTGTCTATTGGTTTAACTAACAATTACAAAATTTTTTCAATTTTTATAAT
ATAAATTTAAATGAGAATATTAAAAAGTCATTCATTATTAAAATTAGTGAATTCTTACCTTATCGA

TGCGTCACAACCAAGTAACATTAGTTACTTGTGAAATTTTGGTTCATTATTAGCTGTTTGTTTAAT
AGTACAAATTATTACCGGTATTACATTAGCTATGCATTATAGTCCTAGTGTAATGGAAGCTTTTAA
CTCAATAGAGCATATAATGAGAGATGTTAATAACGGGTGATTAGTTCGTTATCTACATAGTAATA
CAGCTTCTGCTTTCTTTTTCTTAGTGTATTTACACATAGGAAGAGGTATATATTACGGATCATATA
GAGCTCCTCGTACTTTAGTTTGAGCTATTGGTACTGTTATATTAATATTAATGATGGCTATCGGTT
TCCTAGGTTATGTTTTACCTTATGGACAGATGTCATTATGAGGTGCTACAGTTATTACTAATCTTA
TTAGTGCTATACCTTGAATAGGGCAAGATATTGTTGAATTCATTTGAGGTGGTTTTTCTGTTAATA
ATGCCACTTTAAACAGATTTTTTGCATTACATTTTGTATTGCCTTTTGTATTAGCTGCTTTAGTTTTA
ATGCACTTAATTGCACTTCATGATACTGCTGGTTCAAGCAATCCTCTTGGTGTTTCAGGTAATTAC
GATAGAATTACATTTGCTCCATATTTTTTATTTAAAGATTTAATTACTATTTTTATATTTATTTTTGTA
TTAAGTGCTTTTGTATTCTTTATGCCTAATGTTTTAGGGGATAGTGATAATTATATTATGGCTAATC
CTATGCAAACTCCTGCTGCTATTGTACCTGAATGATACTTATTACCTTTCTATGCTATTTTAAGATC
TATACCTAATAAATTATTAGGTGTTATAGCGATGTTTAGTGCTATTTTAGCTATTATGTTATTACCT
GTTACAGATTTAGGTAGATCTAGAGGTTTACAATTTAGACCATTTAGTAAAATAGCTTTCTGAGTT
TTTGTTGCTAATTTCTTAGTTTTAATGCAATTAGGTGCTAAACACGTTGAAGATCCATTTATATTAT
TAGGTCAATTAAGTACTGTATTATACTTTAGTTATTTTGTTGCTATATTACCTTTAGCTAGTTACTT
AGATAATAGTTTAACTGATTTATCTAATAAATCTGAATTATTTTTAAATAAAACTAACTAAATATAT
TAAGATTATTATTTAATATATTTTCTATTTAAGATACTATTAATTTAGTATTTTGGGTTTTCAGTTTA
TAATTTATATTATATTATGCATTACCCTCCACCTTGCTTTGTAGTAAGCTAATCTGTTATTTCCTTTA
GTTTAATGGTAGAACAATGATCTTCTAATTCATTGGTTTTAGTTCGAATCTAAAAAGGAAATAAG
AAATATATTCTTATTATTACTTATATAATAATTATTTCTTAAAAATATACATTTTGCATTATAGCCGT
TTAGCTGTATTAAAATGTAAAATGATATAAAATAGAATAAATATTTAAATTATTCCTATGTTATATT
ATCCTATATTGCAACCATTATCAGAAGTTGTATTAATACTTGTACCTGCCTTATTAGCTGTAGCTT
ATGTTACAGTTGCTGAAAGAAAAACTATGGCTAGTATGCAAAGAAGATTAGGTCCTAATGCTGT
AGGTTACTATGGACTATTGCAAGCATTTGCTGATGCCTTAAAACTTTTATTAAAAGAATATGTAG
CTCCTACACAATCTAATATTGTTCTTTTCTTTTTAGGTCCTGTAATAACTTTAATTTTTGCATTATTA
GGTTACGCTGTTATACCCTATGGTCCTGGTTCAGGGATAAGCGACATGAATTTAGGTATATTTTA
CATGTTAGCTGTGTCATCTTTAGCTACATACGGTATTCTATTAGCTGGTTGAAGTGCGAATAGTA
AATACGCTTTTCTAGGTTCTCTTAGAAGTACAGCTCAATTAATTAGTTATGAATTAATATTAAGTT
CAGCTATATTAATAGTAATTATGATAACAGGAAATTTAAATTTAACTGTTTGTACTGAATCTCAAA
GAGCTATTTGATTTATACTACCTTTATTTCCTGTGTTTATAATATTTTTCATAGGATCTATAGCTGA
GACAAATAGAGCTCCTTTTGATTTAGCCGAGGCTAACCTGCTAATCTGGTTTGGTCTGGTTATAT
GTCACAAATTGCTAGGAAACCTTTTTATTTTAAAAACAAAAGACAATTAGCAGGAAACTTAATTT
AACCTAATTAAAAATATTAGATAATTAAACTCTTCATAGACTAAACGTGACAATTTAATATATATA
TATATTTATTTATATATATGATTAAATAAGATATAGTCAATCATCGGTGTGAATCGACTTAAAAAA
AAAAGCACATGGGTTAAACCCATCTCCCCTTATTAGGGGAATCAGAACTTGTTAGTGGGTTCAT
GACAGAGCATGCTGCCGTAGTTTTCGTATTCTTCTTTTTAGCTGAGTACGGTAGTATTGTACTAAT
GTGTATTTTAACTAGTATATTATTTATTGGTGGTTACTTATTATTTGAAATATCCTATGTTTTTACTG
TGGTAAATTATATTTTCTTTGAATTATTCTTTATAGACTGAGTAACATTTGTAGAGGTACAATCTTT
ATACACTGATTTTTTAAATAATTCTATCATTGAAGGATTATTATATGGGTTTAATCTAGGATTAAA
AAGTTCTTTAATGATATTCACATTTATTTGAGCTAGAGCATCCTTCCCTAGAATACGATTTGATCA
ACTAATGGGCTTCTGTTGAACAGTTTTATTACCTATTAATTTTGCAATTATTATATTAGTACCTTGT
GTTTTATATAGTTTTAACTTATTACCTGTAAATATACCATTGTTCTAGCTCACACACCCGCCCGCC
CTACTGCCACAAGGCTACAGTACATATGAGGAGGGGAACTAAAGATCTAGAACTATCCTAGTTA

ATAATTACACTTAATAGTATACTAAATAGACCATCTATCATACTCGAGAATAGTGATAGTGTAAT
TATACACTATTTTAACTGTATTCTACACTATTAGCATATTATTATCTTTATATGATAATAATTTTATA
ACTTAATTTATTAGGTTACATTAACAAAAAATTCGTTAACTTTTTTCACCACTTTTTAATACAAAAT
ACGAATTTTA
>YN540
ATAACAATTCTAAAGAACATAAAGAGTTATTAGATAAAAATAATTCACCTATACAGTTAATAAAT
CAACTTAAAGGGTATTTTTTCATAAATCCTTTATTAGCTTTAAGTTTAGCTATTACTATTTTCTCTTT
TGCAGGTATTCCTCCTCTTGTAGGGTTCTTTGCTAAACAGATGGTATTAAGCGCGGCTATTGATC
AAGGTTATATCTTTTTATCTTTAGTTGCAATATTAACTAGTGTTATAGGAGGGGTTTATTATTTAAA
TATAATTAAAGAAATGTTCTTTTATTCACCTGACTATAAATTAAACGAAGAAATTAAAAATAATAC
TATTAATGGTCAAATTATTAATAGAAACAATAAAATATTAAATGTTGAATTTAATTATACAAATGT
AGTTATGTCTAGTTCTGTGGCAATAACTATTTCTACTATTACATTAGTAGTTTTATTATTCATGTTT
ATGAATAAAGAATGATTAAGTCTGGGTACTATATTGGTACAATCTTTATTTAGCTATTAATGAGTA
GTATGACATTATTTATAGGGTTTGTATCTGTTATAGCTATTTTATTTTTAGCCATTAATTTTATATTT
GCTCCTCATAATCCTTATCAAGAAAAATATAGTATTTTCGAGTGTGGTTTCCATAGTTTTTTAGGG
CAAAATAGAACACAATTCGGTATAAAATTCTTTATTTTTGCTTTAGTTTATTTACTTTTAGATTTAG
AAATATTATTAACTTTCCCTTTCGCTCTTAGTGAGTATGTTAATGGTATTTATGGTCTTTTAGTTAC
TTTAATTTTTATAGCTATAATAACTATAGGATTTATATTTGAATTAGGTAAAAGCGCTCTTAAAATA
GACAGCAGACAAAAATTATATATACCTAAATTGAACGTTAATTACCATACAGAGTATGTTGGAAT
AGGTAAGGTTTCTAAGTAAAGTTATAGAGGCAGAAAACCAAAAAACCTACCAAAGGGTAGCTA
ATGGGAAGCTATTAAAAATAAAAGATGATAACCTATATATAGTATAGTTACTATATATTTACTATA
ACTAGGATTATTATATATATATATATTATCTTATTGTATATTAAGATTATTATTATTATAAGGTATA
ATTAATATAGTATCTTATTGTATAAGAATATAATATATTAACCTATAATTAATTTTATTTTTTAATTT
TTAATTATAATTTTTTTTTATATCTAGATGCTTACACATCTACAGATGTAGAAGAGAACAAAATAT
TGTTGTTATAGTAGTGGATGATATAGAAAAATATTTTTATATTATTTATTTTTGTTAGGTAGCTTTT
GAAGTGTTTGATAGAGAGGATATATGGACGGTAGGAGGGTATTCATTTTAATGAACAGTGGATA
GTTTAAATTAACCTAGTTATAGTTTATGAATTTAAATTATAATTATATTAATGTAGGTTATGATAGA
ATTATGTATTGATCCTTAAGAGTTAAGAGAGATACGCCCACGTATAATACATATTGGTTTAGGGT
TGAGTATATATACTTAAGAGTTAAGAATATATATATACAATATATAATAAATATAGTAATATATTA
ATGGTATGGACTTAACCAGGTTTATATATTATCATTTGATAAACATTAATTATAATTTTTCTATTTA
TTATTATTATTATACTGTGAGATTAATAATTATTAAAATATAATATTACATAGTACGTGATATTTGG
GGATTTTATCTTATTAATTGGCAATTAATGATTCTAATCAAATTTTATTCTCTTTAGTTTAATGGTA
GAACAATGATCTTCTAATTCATTGGTTTTAGTTCGATTCTAAAAAGAGATGAGTAAATAATTTTCT
AGATCAGAAATACTACTTTTAACTACAAAAAGCTTACGCTTTTTAACATTTTTTTGATAAATAACA
ACTATTGTTAATATTTGGCTGTCTATTGGTTTAACTAACAATTACAAAATTTTTTCAATTTTTATAAT
ATAAATTTAAATGAGAATATTAAAAAGTCATTCATTATTAAAATTAGTGAATTCTTACCTTATCGA
TGCGTCACAACCAAGTAACATTAGTTACTTGTGAAATTTTGGTTCATTATTAGCTGTTTGTTTAAT
AGTACAAATTATTACCGGTATTACATTAGCTATGCATTATAGTCCTAGTGTAATGGAAGCTTTTAA
CTCAATAGAGCATATAATGAGAGATGTTAATAACGGGTGATTAGTTCGTTATCTACATAGTAATA
CAGCTTCTGCTTTCTTTTTCTTAGTGTATTTACACATAGGAAGAGGTATATATTACGGATCATATA
GAGCTCCTCGTACTTTAGTTTGAGCTATTGGTACTGTTATATTAATATTAATGATGGCTATCGGTT
TCCTAGGTTATGTTTTACCTTATGGACAGATGTCATTATGAGGTGCTACAGTTATTACTAATCTTA
TTAGTGCTATACCTTGAATAGGGCAAGATATTGTTGAATTCATTTGAGGTGGTTTTTCTGTTAATA
ATGCCACTTTAAACAGATTTTTTGCATTACATTTTGTATTGCCTTTTGTATTAGCTGCTTTAGTTTTA

ATGCACTTAATTGCACTTCATGATACTGCTGGTTCAAGCAATCCTCTTGGTGTTTCAGGTAATTAC
GATAGAATTACATTTGCTCCATATTTTTTATTTAAAGATTTAATTACTATTTTTATATTTATTTTTGTA
TTAAGTGCTTTTGTATTCTTTATGCCTAATGTTTTAGGGGATAGTGATAATTATATTATGGCTAATC
CTATGCAAACTCCTGCTGCTATTGTACCTGAATGATACTTATTACCTTTCTATGCTATTTTAAGATC
TATACCTAATAAATTATTAGGTGTTATAGCGATGTTTAGTGCTATTTTAGCTATTATGTTATTACCT
GTTACAGATTTAGGTAGATCTAGAGGTTTACAATTTAGACCATTTAGTAAAATAGCTTTCTGAGTT
TTTGTTGCTAATTTCTTAGTTTTAATGCAATTAGGTGCTAAACACGTTGAAGATCCATTTATATTAT
TAGGTCAATTAAGTACTGTATTATACTTTAGTTATTTTGTTGCTATATTACCTTTAGCTAGTTACTT
AGATAATAGTTTAACTGATTTATCTAATAAATCTGAATTATTTTTAAATAAAACTAACTAAATATAT
TAAGATTATTATTTAATATATTTTCTATTTAAGATACTATTAATTTAGTATTTTGGGTTTTCAGTTTA
TAATTTATATTATATTATGCATTACCCTCCACCTTGCTTTGTAGTAAGCTAATCTGTTATTTCCTTTA
GTTTAATGGTAGAACAATGATCTTCTAATTCATTGGTTTTAGTTCGAATCTAAAAAGGAAATAAG
AAATATATTCTTATTATTACTTATATAATAATTATTTCTTAAAAATATACATTTTGCATTATAGCCGT
TTAGCTGTATTAAAATGTAAAATGATATAAAATAGAATAAATATTTAAATTATTCCTATGTTATATT
ATCCTATATTGCAACCATTATCAGAAGTTGTATTAATACTTGTACCTGCCTTATTAGCTGTAGCTT
ATGTTACAGTTGCTGAAAGAAAAACTATGGCTAGTATGCAAAGAAGATTAGGTCCTAATGCTGT
AGGTTACTATGGACTATTGCAAGCATTTGCTGATGCCTTAAAACTTTTATTAAAAGAATATGTAG
CTCCTACACAATCTAATATTGTTCTTTTCTTTTTAGGTCCTGTAATAACTTTAATTTTTGCATTATTA
GGTTACGCTGTTATACCCTATGGTCCTGGTTCAGGGATAAGCGACATGAATTTAGGTATATTTTA
CATGTTAGCTGTGTCATCTTTAGCTACATACGGTATTCTATTAGCTGGTTGAAGTGCGAATAGTA
AATACGCTTTTCTAGGTTCTCTTAGAAGTACAGCTCAATTAATTAGTTATGAATTAATATTAAGTT
CAGCTATATTAATAGTAATTATGATAACAGGAAATTTAAATTTAACTGTTTGTACTGAATCTCAAA
GAGCTATTTGATTTATACTACCTTTATTTCCTGTGTTTATAATATTTTTCATAGGATCTATAGCTGA
GACAAATAGAGCTCCTTTTGATTTAGCCGAGGCTAACCTGCTAATCTGGTTTGGTCTGGTTATAT
GTCACAAATTGCTAGGAAACCTTTTTATTTTAAAAACAAAAGACAATTAGCAGGAAACTTAATTT
AACCTAATTAAAAATATTAGATAATTAAACTCTTCATAGACTAAACGTGACAATTTAATATATATA
TATATTTATTTATATATATGATTAAATAAGATATAGTCAATCATCGGTGTGAATCGACTTAAAAAA
AAAAGCACATGGGTTAAACCCATCTCCCCTTATTAGGGGAATCAGAACTTGTTAGTGGGTTCAT
GACAGAGCATGCTGCCGTAGTTTTCGTATTCTTCTTTTTAGCTGAGTACGGTAGTATTGTACTAAT
GTGTATTTTAACTAGTATATTATTTATTGGTGGTTACTTATTATTTGAAATATCCTATGTTTTTACTG
TGGTAAATTATATTTTCTTTGAATTATTCTTTATAGACTGAGTAACATTTGTAGAGGTACAATCTTT
ATACACTGATTTTTTAAATAATTCTATCATTGAAGGATTATTATATGGGTTTAATCTAGGATTAAA
AAGTTCTTTAATGATATTCACATTTATTTGAGCTAGAGCATCCTTCCCTAGAATACGATTTGATCA
ACTAATGGGCTTCTGTTGAACAGTTTTATTACCTATTAATTTTGCAATTATTATATTAGTACCTTGT
GTTTTATATAGTTTTAACTTATTACCTGTAAATATACCATTGTTCTAGCTCACACACCCGCCCGCC
CTACTGCCACAAGGCTACAGTACATATGAGGAGGGGAACTAAAGATCTAGAACTATCCTAGTTA
ATAATTACACTTAATAGTATACTAAATAGACCATCTATCATACTCGAGAATAGTGATAGTGTAAT
TATACACTATTTTAACTGTATTCTACACTATTAGCATATTATTATCTTTATATGATAATAATTTTATA
ACTTAATTTATTAGGTTACATTAACAAAAAATTCGTTAACTTTTTTCACCACTTTTTAATACAAAAT
ACGAATAATA
>YN541
ATAACAATTCTAAAGAACATAAAGAGTTATTAGATAAAAATAATTCACCTATACAGTTAATAAAT
CAACTTAAAGGGTATTTTTTCATAAATCCTTTATTAGCTTTAAGTTTAGCTATTACTATTTTCTCTTT
TGCAGGTATTCCTCCTCTTGTAGGGTTCTTTGCTAAACAGATGGTATTAAGCGCGGCTATTGATC

AAGGTTATATCTTTTTATCTTTAGTTGCAATATTAACTAGTGTTATAGGAGGGGTTTATTATTTAAA
TATAATTAAAGAAATGTTCTTTTATTCACCTGACTATAAATTAAACGAAGAAATTAAAAATAATAC
TATTAATGGTCAAATTATTAATAGAAACAATAAAATATTAAATGTTGAATTTAATTATACAAATGT
AGTTATGTCTAGTTCTGTGGCAATAACTATTTCTACTATTACATTAGTAGTTTTATTATTCATGTTT
ATGAATAAAGAATGATTAAGTCTGGGTACTATATTGGTACAATCTTTATTTAGCTATTAATGAGTA
GTATGACATTATTTATAGGGTTTGTATCTGTTATAGCTATTTTATTTTTAGCCATTAATTTTATATTT
GCTCCTCATAATCCTTATCAAGAAAAATATAGTATTTTCGAGTGTGGTTTCCATAGTTTTTTAGGG
CAAAATAGAACACAATTCGGTATAAAATTCTTTATTTTTGCTTTAGTTTATTTACTTTTAGATTTAG
AAATATTATTAACTTTCCCTTTCGCTCTTAGTGAGTATGTTAATGGTATTTATGGTCTTTTAGTTAC
TTTAATTTTTATAGCTATAATAACTATAGGATTTATATTTGAATTAGGTAAAAGCGCTCTTAAAATA
GACAGCAGACAAAAATTATATATACCTAAATTGAACGTTAATTACCATACAGAGTATGTTGGAAT
AGGTAAGGTTTCTAAGTAAAGTTATAGAGGCAGAAAACCAAAAAACCTACCAAAGGGTAGCTA
ATGGGAAGCTATTAAAAATAAAAGATGATAACCTATATATAGTATAGTTACTATATATTTACTATA
ACTAGGATTATTATATATATATATATTATCTTATTGTATATTAAGATTATTATTATTATAAGGTATA
ATTAATATAGTATCTTATTGTATAAGAATATAATATATTAACCTATAATTAATTTTATTTTTTAATTT
TTAATTATAATTTTTTTTTATATCTAGATGCTTACACATCTACAGATGTAGAAGAGAACAAAATAT
TGTTGTTATAGTAGTGGATGATATAGAAAAATATTTTTATATTATTTATTTTTGTTAGGTAGCTTTT
GAAGTGTTTGATAGAGAGGATATATGGACGGTAGGAGGGTATTCATTTTAATGAACAGTGGATA
GTTTAAATTAACCTAGTTATAGTTTATGAATTTAAATTATAATTATATTAATGTAGGTTATGATAGA
ATTATGTATTGATCCTTAAGAGTTAAGAGAGATACGCCCACGTATAATACATATTGGTTTAGGGT
TGAGTATATATACTTAAGAGTTAAGAATATATATATACAATATATAATAAATATAGTAATATATTA
ATGGTATGGACTTAACCAGGTTTATATATTATCATTTGATAAACATTAATTATAATTTTTCTATTTA
TTATTATTATTATACTGTGAGATTAATAATTATTAAAATATAATATTACATAGTACGTGATATTTGG
GGATTTTATCTTATTAATTGGCAATTAATGATTCTAATCAAATTTTATTCTCTTTAGTTTAATGGTA
GAACAATGATCTTCTAATTCATTGGTTTTAGTTCGATTCTAAAAAGAGATGAGTAAATAATTTTCT
AGATCAGAAATACTACTTTTAACTACAAAAAGCTTACGCTTTTTAACATTTTTTTGATAAATAACA
ACTATTGTTAATATTTGGCTGTCTATTGGTTTAACTAACAATTACAAAATTTTTTCAATTTTTATAAT
ATAAATTTAAATGAGAATATTAAAAAGTCATTCATTATTAAAATTAGTGAATTCTTACCTTATCGA
TGCGTCACAACCAAGTAACATTAGTTACTTGTGAAATTTTGGTTCATTATTAGCTGTTTGTTTAAT
AGTACAAATTATTACCGGTATTACATTAGCTATGCATTATAGTCCTAGTGTAATGGAAGCTTTTAA
CTCAATAGAGCATATAATGAGAGATGTTAATAACGGGTGATTAGTTCGTTATCTACATAGTAATA
CAGCTTCTGCTTTCTTTTTCTTAGTGTATTTACACATAGGAAGAGGTATATATTACGGATCATATA
GAGCTCCTCGTACTTTAGTTTGAGCTATTGGTACTGTTATATTAATATTAATGATGGCTATCGGTT
TCCTAGGTTATGTTTTACCTTATGGACAGATGTCATTATGAGGTGCTACAGTTATTACTAATCTTA
TTAGTGCTATACCTTGAATAGGGCAAGATATTGTTGAATTCATTTGAGGTGGTTTTTCTGTTAATA
ATGCCACTTTAAACAGATTTTTTGCATTACATTTTGTATTGCCTTTTGTATTAGCTGCTTTAGTTTTA
ATGCACTTAATTGCACTTCATGATACTGCTGGTTCAAGCAATCCTCTTGGTGTTTCAGGTAATTAC
GATAGAATTACATTTGCTCCATATTTTTTATTTAAAGATTTAATTACTATTTTTATATTTATTTTTGTA
TTAAGTGCTTTTGTATTCTTTATGCCTAATGTTTTAGGGGATAGTGATAATTATATTATGGCTAATC
CTATGCAAACTCCTGCTGCTATTGTACCTGAATGATACTTATTACCTTTCTATGCTATTTTAAGATC
TATACCTAATAAATTATTAGGTGTTATAGCGATGTTTAGTGCTATTTTAGCTATTATGTTATTACCT
GTTACAGATTTAGGTAGATCTAGAGGTTTACAATTTAGACCATTTAGTAAAATAGCTTTCTGAGTT
TTTGTTGCTAATTTCTTAGTTTTAATGCAATTAGGTGCTAAACACGTTGAAGATCCATTTATATTAT
TAGGTCAATTAAGTACTGTATTATACTTTAGTTATTTTGTTGCTATATTACCTTTAGCTAGTTACTT

AGATAATAGTTTAACTGATTTATCTAATAAATCTGAATTATTTTTAAATAAAACTAACTAAATATAT
TAAGATTATTATTTAATATATTTTCTATTTAAGATACTATTAATTTAGTATTTTGGGTTTTCAGTTTA
TAATTTATATTATATTATGCATTACCCTCCACCTTGCTTTGTAGTAAGCTAATCTGTTATTTCCTTTA
GTTTAATGGTAGAACAATGATCTTCTAATTCATTGGTTTTAGTTCGAATCTAAAAAGGAAATAAG
AAATATATTCTTATTATTACTTATATAATAATTATTTCTTAAAAATATACATTTTGCATTATAGCCGT
TTAGCTGTATTAAAATGTAAAATGATATAAAATAGAATAAATATTTAAATTATTCCTATGTTATATT
ATCCTATATTGCAACCATTATCAGAAGTTGTATTAATACTTGTACCTGCCTTATTAGCTGTAGCTT
ATGTTACAGTTGCTGAAAGAAAAACTATGGCTAGTATGCAAAGAAGATTAGGTCCTAATGCTGT
AGGTTACTATGGACTATTGCAAGCATTTGCTGATGCCTTAAAACTTTTATTAAAAGAATATGTAG
CTCCTACACAATCTAATATTGTTCTTTTCTTTTTAGGTCCTGTAATAACTTTAATTTTTGCATTATTA
GGTTACGCTGTTATACCCTATGGTCCTGGTTCAGGGATAAGCGACATGAATTTAGGTATATTTTA
CATGTTAGCTGTGTCATCTTTAGCTACATACGGTATTCTATTAGCTGGTTGAAGTGCGAATAGTA
AATACGCTTTTCTAGGTTCTCTTAGAAGTACAGCTCAATTAATTAGTTATGAATTAATATTAAGTT
CAGCTATATTAATAGTAATTATGATAACAGGAAATTTAAATTTAACTGTTTGTACTGAATCTCAAA
GAGCTATTTGATTTATACTACCTTTATTTCCTGTGTTTATAATATTTTTCATAGGATCTATAGCTGA
GACAAATAGAGCTCCTTTTGATTTAGCCGAGGCTAACCTGCTAATCTGGTTTGGTCTGGTTATAT
GTCACAAATTGCTAGGAAACCTTTTTATTTTAAAAACAAAAGACAATTAGCAGGAAACTTAATTT
AACCTAATTAAAAATATTAGATAATTAAACTCTTCATAGACTAAACGTGACAATTTAATATATATA
TATATTTATTTATATATATGATTAAATAAGATATAGTCAATCATCGGTGTGAATCGACTTAAAAAA
AAAAGCACATGGGTTAAACCCATCTCCCCTTATTAGGGGAATCAGAACTTGTTAGTGGGTTCAT
GACAGAGCATGCTGCCGTAGTTTTCGTATTCTTCTTTTTAGCTGAGTACGGTAGTATTGTACTAAT
GTGTATTTTAACTAGTATATTATTTATTGGTGGTTACTTATTATTTGAAATATCCTATGTTTTTACTG
TGGTAAATTATATTTTCTTTGAATTATTCTTTATAGACTGAGTAACATTTGTAGAGGTACAATCTTT
ATACACTGATTTTTTAAATAATTCTATCATTGAAGGATTATTATATGGGTTTAATCTAGGATTAAA
AAGTTCTTTAATGATATTCACATTTATTTGAGCTAGAGCATCCTTCCCTAGAATACGATTTGATCA
ACTAATGGGCTTCTGTTGAACAGTTTTATTACCTATTAATTTTGCAATTATTATATTAGTACCTTGT
GTTTTATATAGTTTTAACTTATTACCTGTAAATATACCATTGTTCTAGCTCACACACCCGCCCGCC
CTACTGCCACAAGGCTACAGTACATATGAGGAGGGGAACTAAAGATCTAGAACTATCCTAGTTA
ATAATTACACTTAATAGTATACTAAATAGACCATCTATCATACTCGAGAATAGTGATAGTGTAAT
TATACACTATTTTAACTGTATTCTACACTATTAGCATATTATTATCTTTATATGATAATAATTTTATA
ACTTAATTTATTAGGTTACATTAACAAAAAATTCGTTAACTTTTTTCACCACTTTTTAATACAAAAT
ACGAATTTTA
>YN544
ATAACAATTCTAAAGAACATAAAGAGTTATTAGATAAAAATAATTCACCTATACAGTTAATAAAT
CAACTTAAAGGGTATTTTTTCATAAATCCTTTATTAGCTTTAAGTTTAGCTATTACTATTTTCTCTTT
TGCAGGTATTCCTCCTCTTGTAGGGTTCTTTGCTAAACAGATGGTATTAAGCGCGGCTATTGATC
AAGGTTATATCTTTTTATCTTTAGTTGCAATATTAACTAGTGTTATAGGAGGGGTTTATTATTTAAA
TATAATTAAAGAAATGTTCTTTTATTCACCTGACTATAAATTAAACGAAGAAATTAAAAATAATAC
TATTAATGGTCAAATTATTAATAGAAACAATAAAATATTAAATGTTGAATTTAATTATACAAATGT
AGTTATGTCTAGTTCTGTGGCAATAACTATTTCTACTATTACATTAGTAGTTTTATTATTCATGTTT
ATGAATAAAGAATGATTAAGTCTGGGTACTATATTGGTACAATCTTTATTTAGCTATTAATGAGTA
GTATGACATTATTTATAGGGTTTGTATCTGTTATAGCTATTTTATTTTTAGCCATTAATTTTATATTT
GCTCCTCATAATCCTTATCAAGAAAAATATAGTATTTTCGAGTGTGGTTTCCATAGTTTTTTAGGG
CAAAATAGAACACAATTCGGTATAAAATTCTTTATTTTTGCTTTAGTTTATTTACTTTTAGATTTAG

AAATATTATTAACTTTCCCTTTCGCTCTTAGTGAGTATGTTAATGGTATTTATGGTCTTTTAGTTAC
TTTAATTTTTATAGCTATAATAACTATAGGATTTATATTTGAATTAGGTAAAAGCGCTCTTAAAATA
GACAGCAGACAAAAATTATATATACCTAAATTGAACGTTAATTACCATACAGAGTATGTTGGAAT
AGGTAAGGTTTCTAAGTAAAGTTATAGAGGCAGAAAACCAAAAAACCTACCAAAGGGTAGCTA
ATGGGAAGCTATTAAAAATAAAAGATGATAACCTATATATAGTATAGTTACTATATATTTACTATA
ACTAGGATTATTATATATATATATATTATCTTATTGTATATTAAGATTATTATTATTATAAGGTATA
ATTAATATAGTATCTTATTGTATAAGAATATAATATATTAACCTATAATTAATTTTATTTTTTAATTT
TTAATTATAATTTTTTTTTATATCTAGATGCTTACACATCTACAGATGTAGAAGAGAACAAAATAT
TGTTGTTATAGTAGTGGATGATATAGAAAAATATTTTTATATTATTTATTTTTGTTAGGTAGCTTTT
GAAGTGTTTGATAGAGAGGATATATGGACGGTAGGAGGGTATTCATTTTAATGAACAGTGGATA
GTTTAAATTAACCTAGTTATAGTTTATGAATTTAAATTATAATTATATTAATGTAGGTTATGATAGA
ATTATGTATTGATCCTTAAGAGTTAAGAGAGATACGCCCACGTATAATACATATTGGTTTAGGGT
TGAGTATATATACTTAAGAGTTAAGAATATATATATACAATATATAATAAATATAGTAATATATTA
ATGGTATGGACTTAACCAGGTTTATATATTATCATTTGATAAACATTAATTATAATTTTTCTATTTA
TTATTATTATTATACTGTGAGATTAATAATTATTAAAATATAATATTACATAGTACGTGATATTTGG
GGATTTTATCTTATTAATTGGCAATTAATGATTCTAATCAAATTTTATTCTCTTTAGTTTAATGGTA
GAACAATGATCTTCTAATTCATTGGTTTTAGTTCGATTCTAAAAAGAGATGAGTAAATAATTTTCT
AGATCAGAAATACTACTTTTAACTACAAAAAGCTTACGCTTTTTAACATTTTTTTGATAAATAACA
ACTATTGTTAATATTTGGCTGTCTATTGGTTTAACTAACAATTACAAAATTTTTTCAATTTTTATAAT
ATAAATTTAAATGAGAATATTAAAAAGTCATTCATTATTAAAATTAGTGAATTCTTACCTTATCGA
TGCGTCACAACCAAGTAACATTAGTTACTTGTGAAATTTTGGTTCATTATTAGCTGTTTGTTTAAT
AGTACAAATTATTACCGGTATTACATTAGCTATGCATTATAGTCCTAGTGTAATGGAAGCTTTTAA
CTCAATAGAGCATATAATGAGAGATGTTAATAACGGGTGATTAGTTCGTTATCTACATAGTAATA
CAGCTTCTGCTTTCTTTTTCTTAGTGTATTTACACATAGGAAGAGGTATATATTACGGATCATATA
GAGCTCCTCGTACTTTAGTTTGAGCTATTGGTACTGTTATATTAATATTAATGATGGCTATCGGTT
TCCTAGGTTATGTTTTACCTTATGGACAGATGTCATTATGAGGTGCTACAGTTATTACTAATCTTA
TTAGTGCTATACCTTGAATAGGGCAAGATATTGTTGAATTCATTTGAGGTGGTTTTTCTGTTAATA
ATGCCACTTTAAACAGATTTTTTGCATTACATTTTGTATTGCCTTTTGTATTAGCTGCTTTAGTTTTA
ATGCACTTAATTGCACTTCATGATACTGCTGGTTCAAGCAATCCTCTTGGTGTTTCAGGTAATTAC
GATAGAATTACATTTGCTCCATATTTTTTATTTAAAGATTTAATTACTATTTTTATATTTATTTTTGTA
TTAAGTGCTTTTGTATTCTTTATGCCTAATGTTTTAGGGGATAGTGATAATTATATTATGGCTAATC
CTATGCAAACTCCTGCTGCTATTGTACCTGAATGATACTTATTACCTTTCTATGCTATTTTAAGATC
TATACCTAATAAATTATTAGGTGTTATAGCGATGTTTAGTGCTATTTTAGCTATTATGTTATTACCT
GTTACAGATTTAGGTAGATCTAGAGGTTTACAATTTAGACCATTTAGTAAAATAGCTTTCTGAGTT
TTTGTTGCTAATTTCTTAGTTTTAATGCAATTAGGTGCTAAACACGTTGAAGATCCATTTATATTAT
TAGGTCAATTAAGTACTGTATTATACTTTAGTTATTTTGTTGCTATATTACCTTTAGCTAGTTACTT
AGATAATAGTTTAACTGATTTATCTAATAAATCTGAATTATTTTTAAATAAAACTAACTAAATATAT
TAAGATTATTATTTAATATATTTTCTATTTAAGATACTATTAATTTAGTATTTTGGGTTTTCAGTTTA
TAATTTATATTATATTATGCATTACCCTCCACCTTGCTTTGTAGTAAGCTAATCTGTTATTTCCTTTA
GTTTAATGGTAGAACAATGATCTTCTAATTCATTGGTTTTAGTTCGAATCTAAAAAGGAAATAAG
AAATATATTCTTATTATTACTTATATAATAATTATTTCTTAAAAATATACATTTTGCATTATAGCCGT
TTAGCTGTATTAAAATGTAAAATGATATAAAATAGAATAAATATTTAAATTATTCCTATGTTATATT
ATCCTATATTGCAACCATTATCAGAAGTTGTATTAATACTTGTACCTGCCTTATTAGCTGTAGCTT
ATGTTACAGTTGCTGAAAGAAAAACTATGGCTAGTATGCAAAGAAGATTAGGTCCTAATGCTGT

AGGTTACTATGGACTATTGCAAGCATTTGCTGATGCCTTAAAACTTTTATTAAAAGAATATGTAG
CTCCTACACAATCTAATATTGTTCTTTTCTTTTTAGGTCCTGTAATAACTTTAATTTTTGCATTATTA
GGTTACGCTGTTATACCCTATGGTCCTGGTTCAGGGATAAGCGACATGAATTTAGGTATATTTTA
CATGTTAGCTGTGTCATCTTTAGCTACATACGGTATTCTATTAGCTGGTTGAAGTGCGAATAGTA
AATACGCTTTTCTAGGTTCTCTTAGAAGTACAGCTCAATTAATTAGTTATGAATTAATATTAAGTT
CAGCTATATTAATAGTAATTATGATAACAGGAAATTTAAATTTAACTGTTTGTACTGAATCTCAAA
GAGCTATTTGATTTATACTACCTTTATTTCCTGTGTTTATAATATTTTTCATAGGATCTATAGCTGA
GACAAATAGAGCTCCTTTTGATTTAGCCGAGGCTAACCTGCTAATCTGGTTTGGTCTGGTTATAT
GTCACAAATTGCTAGGAAACCTTTTTATTTTAAAAACAAAAGACAATTAGCAGGAAACTTAATTT
AACCTAATTAAAAATATTAGATAATTAAACTCTTCATAGACTAAACGTGACAATTTAATATATATA
TATATTTATTTATATATATGATTAAATAAGATATAGTCAATCATCGGTGTGAATCGACTTAAAAAA
AAAAGCACATGGGTTAAACCCATCTCCCCTTATTAGGGGAATCAGAACTTGTTAGTGGGTTCAT
GACAGAGCATGCTGCCGTAGTTTTCGTATTCTTCTTTTTAGCTGAGTACGGTAGTATTGTACTAAT
GTGTATTTTAACTAGTATATTATTTATTGGTGGTTACTTATTATTTGAAATATCCTATGTTTTTACTG
TGGTAAATTATATTTTCTTTGAATTATTCTTTATAGACTGAGTAACATTTGTAGAGGTACAATCTTT
ATACACTGATTTTTTAAATAATTCTATCATTGAAGGATTATTATATGGGTTTAATCTAGGATTAAA
AAGTTCTTTAATGATATTCACATTTATTTGAGCTAGAGCATCCTTCCCTAGAATACGATTTGATCA
ACTAATGGGCTTCTGTTGAACAGTTTTATTACCTATTAATTTTGCAATTATTATATTAGTACCTTGT
GTTTTATATAGTTTTAACTTATTACCTGTAAATATACCATTGTTCTAGCTCACACACCCGCCCGCC
CTACTGCCACAAGGCTACAGTACATATGAGGAGGGGAACTAAAGATCTAGAACTATCCTAGTTA
ATAATTACACTTAATAGTATACTAAATAGACCATCTATCATACTCGAGAATAGTGATAGTGTAAT
TATACACTATTTTAACTGTATTCTACACTATTAGCATATTATTATCTTTATATGATAATAATTTTATA
ACTTAATTTATTAGGTTACATTAACAAAAAAATTCGTTAACTTTTTTCACCACTTTTTAATACAAAAT
ACGAATTTTA
>YN554
ATAACAATTCTAAAGAACATAAAGAGTTATTAGATAAAAATAATTCACCTATACAGTTAATAAAT
CAACTTAAAGGGTATTTTTTCATAAATCCTTTATTAGCTTTAAGTTTAGCTATTACTATTTTCTCTTT
TGCAGGTATTCCTCCTCTTGTAGGGTTCTTTGCTAAACAGATGGTATTAAGCGCGGCTATTGATC
AAGGTTATATCTTTTTATCTTTAGTTGCAATATTAACTAGTGTTATAGGAGGGGTTTATTATTTAAA
TATAATTAAAGAAATGTTCTTTTATTCACCTGACTATAAATTAAACGAAGAAATTAAAAATAATAC
TATTAATGGTCAAATTATTAATAGAAACAATAAAATATTAAATGTTGAATTTAATTATACAAATGT
AGTTATGTCTAGTTCTGTGGCAATAACTATTTCTACTATTACATTAGTAGTTTTATTATTCATGTTT
ATGAATAAAGAATGATTAAGTCTGGGTACTATATTGGTACAATCTTTATTTAGCTATTAATGAGTA
GTATGACATTATTTATAGGGTTTGTATCTGTTATAGCTATTTTATTTTTAGCCATTAATTTTATATTT
GCTCCTCATAATCCTTATCAAGAAAAATATAGTATTTTCGAGTGTGGTTTCCATAGTTTTTTAGGG
CAAAATAGAACACAATTCGGTATAAAATTCTTTATTTTTGCTTTAGTTTATTTACTTTTAGATTTAG
AAATATTATTAACTTTCCCTTTCGCTCTTAGTGAGTATGTTAATGGTATTTATGGTCTTTTAGTTAC
TTTAATTTTTATAGCTATAATAACTATAGGATTTATATTTGAATTAGGTAAAAGCGCTCTTAAAATA
GACAGCAGACAAAAATTATATATACCTAAATTGAACGTTAATTACCATACAGAGTATGTTGGAAT
AGGTAAGGTTTCTAAGTAAAGTTATAGAGGCAGAAAACCAAAAAACCTACCAAAGGGTAGCTA
ATGGGAAGCTATTAAAAATAAAAGATGATAACCTATATATAGTATAGTTACTATATATTTACTATA
ACTAGGATTATTATATATATATATATTATCTTATTGTATATTAAGATTATTATTATTATAAGGTATA
ATTAATATAGTATCTTATTGTATAAGAATATAATATATTAACCTATAATTAATTTTATTTTTTAATTT
TTAATTATAATTTTTTTTTATATCTAGATGCTTACACATCTACAGATGTAGAAGAGAACAAAATAT

TGTTGTTATAGTAGTGGATGATATAGAAAAATATTTTTATATTATTTATTTTTGTTAGGTAGCTTTT
GAAGTGTTTGATAGAGAGGATATATGGACGGTAGGAGGGTATTCATTTTAATGAACAGTGGATA
GTTTAAATTAACCTAGTTATAGTTTATGAATTTAAATTATAATTATATTAATGTAGGTTATGATAGA
ATTATGTATTGATCCTTAAGAGTTAAGAGAGATACGCCCACGTATAATACATATTGGTTTAGGGT
TGAGTATATATACTTAAGAGTTAAGAATATATATATACAATATATAATAAATATAGTAATATATTA
ATGGTATGGACTTAACCAGGTTTATATATTATCATTTGATAAACATTAATTATAATTTTTCTATTTA
TTATTATTATTATACTGTGAGATTAATAATTATTAAAATATAATATTACATAGTACGTGATATTTGG
GGATTTTATCTTATTAATTGGCAATTAATGATTCTAATCAAATTTTATTCTCTTTAGTTTAATGGTA
GAACAATGATCTTCTAATTCATTGGTTTTAGTTCGATTCTAAAAAGAGATGAGTAAATAATTTTCT
AGATCAGAAATACTACTTTTAACTACAAAAAGCTTACGCTTTTTAACATTTTTTTGATAAATAACA
ACTATTGTTAATATTTGGCTGTCTATTGGTTTAACTAACAATTACAAAATTTTTTCAATTTTTATAAT
ATAAATTTAAATGAGAATATTAAAAAGTCATTCATTATTAAAATTAGTGAATTCTTACCTTATCGA
TGCGTCACAACCAAGTAACATTAGTTACTTGTGAAATTTTGGTTCATTATTAGCTGTTTGTTTAAT
AGTACAAATTATTACCGGTATTACATTAGCTATGCATTATAGTCCTAGTGTAATGGAAGCTTTTAA
CTCAATAGAGCATATAATGAGAGATGTTAATAACGGGTGATTAGTTCGTTATCTACATAGTAATA
CAGCTTCTGCTTTCTTTTTCTTAGTGTATTTACACATAGGAAGAGGTATATATTACGGATCATATA
GAGCTCCTCGTACTTTAGTTTGAGCTATTGGTACTGTTATATTAATATTAATGATGGCTATCGGTT
TCCTAGGTTATGTTTTACCTTATGGACAGATGTCATTATGAGGTGCTACAGTTATTACTAATCTTA
TTAGTGCTATACCTTGAATAGGGCAAGATATTGTTGAATTCATTTGAGGTGGTTTTTCTGTTAATA
ATGCCACTTTAAACAGATTTTTTGCATTACATTTTGTATTGCCTTTTGTATTAGCTGCTTTAGTTTTA
ATGCACTTAATTGCACTTCATGATACTGCTGGTTCAAGCAATCCTCTTGGTGTTTCAGGTAATTAC
GATAGAATTACATTTGCTCCATATTTTTTATTTAAAGATTTAATTACTATTTTTATATTTATTTTTGTA
TTAAGTGCTTTTGTATTCTTTATGCCTAATGTTTTAGGGGATAGTGATAATTATATTATGGCTAATC
CTATGCAAACTCCTGCTGCTATTGTACCTGAATGATACTTATTACCTTTCTATGCTATTTTAAGATC
TATACCTAATAAATTATTAGGTGTTATAGCGATGTTTAGTGCTATTTTAGCTATTATGTTATTACCT
GTTACAGATTTAGGTAGATCTAGAGGTTTACAATTTAGACCATTTAGTAAAATAGCTTTCTGAGTT
TTTGTTGCTAATTTCTTAGTTTTAATGCAATTAGGTGCTAAACACGTTGAAGATCCATTTATATTAT
TAGGTCAATTAAGTACTGTATTATACTTTAGTTATTTTGTTGCTATATTACCTTTAGCTAGTTACTT
AGATAATAGTTTAACTGATTTATCTAATAAATCTGAATTATTTTTAAATAAAACTAACTAAATATAT
TAAGATTATTATTTAATATATTTTCTATTTAAGATACTATTAATTTAGTATTTTGGGTTTTCAGTTTA
TAATTTATATTATATTATGCATTACCCTCCACCTTGCTTTGTAGTAAGCTAATCTGTTATTTCCTTTA
GTTTAATGGTAGAACAATGATCTTCTAATTCATTGGTTTTAGTTCGATTCTAAAAAGGAAATAAGA
AATATATTCTTATTATTACTTATATAATAATTATTTCTTAAAAATATACATTTTGCATTATAGCCGTT
TAGCTGTATTAAAATGTAAAATGATATAAAATAGAATAAATATTTAAATTATTCCTATGTTATATT
ATCCTATATTGCAACCATTATCAGAAGTTGTATTAATACTTGTACCTGCCTTATTAGCTGTAGCTT
ATGTTACAGTTGCTGAAAGAAAAACTATGGCTAGTATGCAAAGAAGATTAGGTCCTAATGCTGT
AGGTTACTATGGACTATTGCAAGCATTTGCTGATGCCTTAAAACTTTTATTAAAAGAATATGTAG
CTCCTACACAATCTAATATTGTTCTTTTCTTTTTAGGTCCTGTAATAACTTTAATTTTTGCATTATTA
GGTTACGCTGTTATACCCTATGGTCCTGGTTCAGGGATAAGCGACATGAATTTAGGTATATTTTA
CATGTTAGCTGTGTCATCTTTAGCTACATACGGTATTCTATTAGCTGGTTGAAGTGCGAATAGTA
AATACGCTTTTCTAGGTTCTCTTAGAAGTACAGCTCAATTAATTAGTTATGAATTAATATTAAGTT
CAGCTATATTAATAGTAATTATGATAACAGGAAATTTAAATTTAACTGTTTGTACTGAATCTCAAA
GAGCTATTTGATTTATACTACCTTTATTTCCTGTGTTTATAATATTTTTCATAGGATCTATAGCTGA
GACAAATAGAGCTCCTTTTGATTTAGCCGAGGCTAACCTGCTAATCTGGTTTGGTCTGGTTATAT

GTCACAAATTGCTAGGAAACCTTTTTATTTTAAAAACAAAAGACAATTAGCAGGAAACTTAATTT
AACCTAATTAAAAATATTAGATAATTAAACTCTTCATAGACTAAACGTGACAATTTAATATATATA
TATATTTATTTATATATATGATTAAATAAGATATAGTCAATCATCGGTGTGAATCGACTTAAAAAA
AAAAGCACATGGGTTAAACCCATCTCCCCTTATTAGGGGAATCAGAACTTGTTAGTGGGTTCAT
GACAGAGCATGCTGCCGTAGTTTTCGTATTCTTCTTTTTAGCTGAGTACGGTAGTATTGTACTAAT
GTGTATTTTAACTAGTATATTATTTATTGGTGGTTACTTATTATTTGAAATATCCTATGTTTTTACTG
TGGTAAATTATATTTTCTTTGAATTATTCTTTATAGACTGAGTAACATTTGTAGAGGTACAATCTTT
ATACACTGATTTTTTAAATAATTCTATCATTGAAGGATTATTATATGGGTTTAATCTAGGATTAAA
AAGTTCTTTAATGATATTCACATTTATTTGAGCTAGAGCATCCTTCCCTAGAATACGATTTGATCA
ACTAATGGGCTTCTGTTGAACAGTTTTATTACCTATTAATTTTGCAATTATTATATTAGTACCTTGT
GTTTTATATAGTTTTAACTTATTACCTGTAAATATACCATTGTTCTAGCTCACACACCCGCCCGCC
CTACTGCCACAAGGCTACAGTACATATGAGGAGGGGAACTAAAGATCTAGAACTATCCTAGTTA
ATAATTACACTTAATAGTATACTAAATAGACCATCTATCATACTCGAGAATAGTGATAGTGTAAT
TATACACTATTTTAACTGTATTCTACACTATTAGCATATTATTATCTTTATATGATAATAATTTTATA
ACTTAATTTATTAGGTTACATTAACAAAAAAATTCGTTAACTTTTTTCACCACTTTTTAATACAAAAT
ACGAATTTTA
>YN557
ATAACAATTCTAAAGAACATAAAGAGTTATTAGATAAAAATAATTCACCTATACAGTTAATAAAT
CAACTTAAAGGGTATTTTTTCATAAATCCTTTATTAGCTTTAAGTTTAGCTATTACTATTTTCTCTTT
TGCAGGTATTCCTCCTCTTGTAGGGTTCTTTGCTAAACAGATGGTATTAAGCGCGGCTATTGATC
AAGGTTATATCTTTTTATCTTTAGTTGCAATATTAACTAGTGTTATAGGAGGGGTTTATTATTTAAA
TATAATTAAAGAAATGTTCTTTTATTCACCTGACTATAAATTAAACGAAGAAATTAAAAATAATAC
TATTAATGGTCAAATTATTAATAGAAACAATAAAATATTAAATGTTGAATTTAATTATACAAATGT
AGTTATGTCTAGTTCTGTGGCAATAACTATTTCTACTATTACATTAGTAGTTTTATTATTCATGTTT
ATGAATAAAGAATGATTAAGTCTGGGTACTATATTGGTACAATCTTTATTTAGCTATTAATGAGTA
GTATGACATTATTTATAGGGTTTGTATCTGTTATAGCTATTTTATTTTTAGCCATTAATTTTATATTT
GCTCCTCATAATCCTTATCAAGAAAAATATAGTATTTTCGAGTGTGGTTTCCATAGTTTTTTAGGG
CAAAATAGAACACAATTCGGTATAAAATTCTTTATTTTTGCTTTAGTTTATTTACTTTTAGATTTAG
AAATATTATTAACTTTCCCTTTCGCTCTTAGTGAGTATGTTAATGGTATTTATGGTCTTTTAGTTAC
TTTAATTTTTATAGCTATAATAACTATAGGATTTATATTTGAATTAGGTAAAAGCGCTCTTAAAATA
GACAGCAGACAAAAATTATATATACCTAAATTGAACGTTAATTACCATACAGAGTATGTTGGAAT
AGGTAAGGTTTCTAAGTAAAGTTATAGAGGCAGAAAACCAAAAAACCTACCAAAGGGTAGCTA
ATGGGAAGCTATTAAAAATAAAAGATGATAACCTATATATAGTATAGTTACTATATATTTACTATA
ACTAGGATTATTATATATATATATATTATCTTATTGTATATTAAGATTATTATTATTATAAGGTATA
ATTAATATAGTATCTTATTGTATAAGAATATAATATATTAACCTATAATTAATTTTATTTTTTAATTT
TTAATTATAATTTTTTTTTATATCTAGATGCTTACACATCTACAGATGTAGAAGAGAACAAAATAT
TGTTGTTATAGTAGTGGATGATATAGAAAAATATTTTTATATTATTTATTTTTGTTAGGTAGCTTTT
GAAGTGTTTGATAGAGAGGATATATGGACGGTAGGAGGGTATTCATTTTAATGAACAGTGGATA
GTTTAAATTAACCTAGTTATAGTTTATGAATTTAAATTATAATTATATTAATGTAGGTTATGATAGA
ATTATGTATTGATCCTTAAGAGTTAAGAGAGATACGCCCACGTATAATACATATTGGTTTAGGGT
TGAGTATATATACTTAAGAGTTAAGAATATATATATACAATATATAATAAATATAGTAATATATTA
ATGGTATGGACTTAACCAGGTTTATATATTATCATTTGATAAACATTAATTATAATTTTTCTATTTA
TTATTATTATTATACTGTGAGATTAATAATTATTAAAATATAATATTACATAGTACGTGATATTTGG
GGATTTTATCTTATTAATTGGCAATTAATGATTCTAATCAAATTTTATTCTCTTTAGTTTAATGGTA

GAACAATGATCTTCTAATTCATTGGTTTTAGTTCGATTCTAAAAAGAGATGAGTAAATAATTTTCT
AGATCAGAAATACTACTTTTAACTACAAAAAGCTTACGCTTTTTAACATTTTTTTGATAAATAACA
ACTATTGTTAATATTTGGCTGTCTATTGGTTTAACTAACAATTACAAAATTTTTTCAATTTTTATAAT
ATAAATTTAAATGAGAATATTAAAAAGTCATTCATTATTAAAATTAGTGAATTCTTACCTTATCGA
TGCGTCACAACCAAGTAACATTAGTTACTTGTGAAATTTTGGTTCATTATTAGCTGTTTGTTTAAT
AGTACAAATTATTACCGGTATTACATTAGCTATGCATTATAGTCCTAGTGTAATGGAAGCTTTTAA
CTCAATAGAGCATATAATGAGAGATGTTAATAACGGGTGATTAGTTCGTTATCTACATAGTAATA
CAGCTTCTGCTTTCTTTTTCTTAGTGTATTTACACATAGGAAGAGGTATATATTACGGATCATATA
GAGCTCCTCGTACTTTAGTTTGAGCTATTGGTACTGTTATATTAATATTAATGATGGCTATCGGTT
TCCTAGGTTATGTTTTACCTTATGGACAGATGTCATTATGAGGTGCTACAGTTATTACTAATCTTA
TTAGTGCTATACCTTGAATAGGGCAAGATATTGTTGAATTCATTTGAGGTGGTTTTTCTGTTAATA
ATGCCACTTTAAACAGATTTTTTGCATTACATTTTGTATTGCCTTTTGTATTAGCTGCTTTAGTTTTA
ATGCACTTAATTGCACTTCATGATACTGCTGGTTCAAGCAATCCTCTTGGTGTTTCAGGTAATTAC
GATAGAATTACATTTGCTCCATATTTTTTATTTAAAGATTTAATTACTATTTTTATATTTATTTTTGTA
TTAAGTGCTTTTGTATTCTTTATGCCTAATGTTTTAGGGGATAGTGATAATTATATTATGGCTAATC
CTATGCAAACTCCTGCTGCTATTGTACCTGAATGATACTTATTACCTTTCTATGCTATTTTAAGATC
TATACCTAATAAATTATTAGGTGTTATAGCGATGTTTAGTGCTATTTTAGCTATTATGTTATTACCT
GTTACAGATTTAGGTAGATCTAGAGGTTTACAATTTAGACCATTTAGTAAAATAGCTTTCTGAGTT
TTTGTTGCTAATTTCTTAGTTTTAATGCAATTAGGTGCTAAACACGTTGAAGATCCATTTATATTAT
TAGGTCAATTAAGTACTGTATTATACTTTAGTTATTTTGTTGCTATATTACCTTTAGCTAGTTACTT
AGATAATAGTTTAACTGATTTATCTAATAAATCTGAATTATTTTTAAATAAAACTAACTAAATATAT
TAAGATTATTATTTAATATATTTTCTATTTAAGATACTATTAATTTAGTATTTTGGGTTTTCAGTTTA
TAATTTATATTATATTATGCATTACCCTCCACCTTGCTTTGTAGTAAGCTAATCTGTTATTTCCTTTA
GTTTAATGGTAGAACAATGATCTTCTAATTCATTGGTTTTAGTTCGAATCTAAAAAGGAAATAAG
AAATATATTCTTATTATTACTTATATAATAATTATTTCTTAAAAATATACATTTTGCATTATAGCCGT
TTAGCTGTATTAAAATGTAAAATGATATAAAATAGAATAAATATTTAAATTATTCCTATGTTATATT
ATCCTATATTGCAACCATTATCAGAAGTTGTATTAATACTTGTACCTGCCTTATTAGCTGTAGCTT
ATGTTACAGTTGCTGAAAGAAAAACTATGGCTAGTATGCAAAGAAGATTAGGTCCTAATGCTGT
AGGTTACTATGGACTATTGCAAGCATTTGCTGATGCCTTAAAACTTTTATTAAAAGAATATGTAG
CTCCTACACAATCTAATATTGTTCTTTTCTTTTTAGGTCCTGTAATAACTTTAATTTTTGCATTATTA
GGTTACGCTGTTATACCCTATGGTCCTGGTTCAGGGATAAGCGACATGAATTTAGGTATATTTTA
CATGTTAGCTGTGTCATCTTTAGCTACATACGGTATTCTATTAGCTGGTTGAAGTGCGAATAGTA
AATACGCTTTTCTAGGTTCTCTTAGAAGTACAGCTCAATTAATTAGTTATGAATTAATATTAAGTT
CAGCTATATTAATAGTAATTATGATAACAGGAAATTTAAATTTAACTGTTTGTACTGAATCTCAAA
GAGCTATTTGATTTATACTACCTTTATTTCCTGTGTTTATAATATTTTTCATAGGATCTATAGCTGA
GACAAATAGAGCTCCTTTTGATTTAGCCGAGGCTAACCTGCTAATCTGGTTTGGTCTGGTTATAT
GTCACAAATTGCTAGGAAACCTTTTTATTTTAAAAACAAAAGACAATTAGCAGGAAACTTAATTT
AACCTAATTAAAAATATTAGATAATTAAACTCTTCATAGACTAAACGTGACAATTTAATATATATA
TATATTTATTTATATATATGATTAAATAAGATATAGTCAATCATCGGTGTGAATCGACTTAAAAAA
AAAAGCACATGGGTTAAACCCATCTCCCCTTATTAGGGGAATCAGAACTTGTTAGTGGGTTCAT
GACAGAGCATGCTGCCGTAGTTTTCGTATTCTTCTTTTTAGCTGAGTACGGTAGTATTGTACTAAT
GTGTATTTTAACTAGTATATTATTTATTGGTGGTTACTTATTATTTGAAATATCCTATGTTTTTACTG
TGGTAAATTATATTTTCTTTGAATTATTCTTTATAGACTGAGTAACATTTGTAGAGGTACAATCTTT
ATACACTGATTTTTTAAATAATTCTATCATTGAAGGATTATTATATGGGTTTAATCTAGGATTAAA

AAGTTCTTTAATGATATTCACATTTATTTGAGCTAGAGCATCCTTCCCTAGAATACGATTTGATCA
ACTAATGGGCTTCTGTTGAACAGTTTTATTACCTATTAATTTTGCAATTATTATATTAGTACCTTGT
GTTTTATATAGTTTTAACTTATTACCTGTAAATATACCATTGTTCTAGCTCACACACCCGCCCGCC
CTACTGCCACAAGGCTACAGTACATATGAGGAGGGGAACTAAAGATCTAGAACTATCCTAGTTA
ATAATTACACTTAATAGTATACTAAATAGACCATCTATCATACTCGAGAATAGTGATAGTGTAAT
TATACACTATTTTAACTGTATTCTACACTATTAGCATATTATTATCTTTATATGATAATAATTTTATA
ACTTAATTTATTAGGTTACATTAACAAAAAAATTCGTTAACTTTTTTCACCACTTTTTAATACAAAAT
ACGAATTTTA
>YN561
ATAACAATTCTAAAGAACATAAAGAGTTATTAGATAAAAATAATTCACCTATACAGTTAATAAAT
CAACTTAAAGGGTATTTTTTCATAAATCCTTTATTAGCTTTAAGTTTAGCTATTACTATTTTCTCTTT
TGCAGGTATTCCTCCTCTTGTAGGGTTCTTTGCTAAACAGATGGTATTAAGCGCGGCTATTGATC
AAGGTTATATCTTTTTATCTTTAGTTGCAATATTAACTAGTGTTATAGGAGGGGTTTATTATTTAAA
TATAATTAAAGAAATGTTCTTTTATTCACCTGACTATAAATTAAACGAAGAAATTAAAAATAATAC
TATTAATGGTCAAATTATTAATAGAAACAATAAAATATTAAATGTTGAATTTAATTATACAAATGT
AGTTATGTCTAGTTCTGTGGCAATAACTATTTCTACTATTACATTAGTAGTTTTATTATTCATGTTT
ATGAATAAAGAATGATTAAGTCTGGGTACTATATTGGTACAATCTTTATTTAGCTATTAATGAGTA
GTATGACATTATTTATAGGGTTTGTATCTGTTATAGCTATTTTATTTTTAGCCATTAATTTTATATTT
GCTCCTCATAATCCTTATCAAGAAAAATATAGTATTTTCGAGTGTGGTTTCCATAGTTTTTTAGGG
CAAAATAGAACACAATTCGGTATAAAATTCTTTATTTTTGCTTTAGTTTATTTACTTTTAGATTTAG
AAATATTATTAACTTTCCCTTTCGCTCTTAGTGAGTATGTTAATGGTATTTATGGTCTTTTAGTTAC
TTTAATTTTTATAGCTATAATAACTATAGGATTTATATTTGAATTAGGTAAAAGCGCTCTTAAAATA
GACAGCAGACAAAAATTATATATACCTAAATTGAACGTTAATTACCATACAGAGTATGTTGGAAT
AGGTAAGGTTTCTAAGTAAAGTTATAGAGGCAGAAAACCAAAAAACCTACCAAAGGGTAGCTA
ATGGGAAGCTATTAAAAATAAAAGATGATAACCTATATATAGTATAGTTACTATATATTTACTATA
ACTAGGATTATTATATATATATATATTATCTTATTGTATATTAAGATTATTATTATTATAAGGTATA
ATTAATATAGTATCTTATTGTATAAGAATATAATATATTAACCTATAATTAATTTTATTTTTTAATTT
TTAATTATAATTTTTTTTTATATCTAGATGCTTACACATCTACAGATGTAGAAGAGAACAAAATAT
TGTTGTTATAGTAGTGGATGATATAGAAAAATATTTTTATATTATTTATTTTTGTTAGGTAGCTTTT
GAAGTGTTTGATAGAGAGGATATATGGACGGTAGGAGGGTATTCATTTTAATGAACAGTGGATA
GTTTAAATTAACCTAGTTATAGTTTATGAATTTAAATTATAATTATATTAATGTAGGTTATGATAGA
ATTATGTATTGATCCTTAAGAGTTAAGAGAGATACGCCCACGTATAATACATATTGGTTTAGGGT
TGAGTATATATACTTAAGAGTTAAGAATATATATATACAATATATAATAAATATAGTAATATATTA
ATGGTATGGACTTAACCAGGTTTATATATTATCATTTGATAAACATTAATTATAATTTTTCTATTTA
TTATTATTATTATACTGTGAGATTAATAATTATTAAAATATAATATTACATAGTACGTGATATTTGG
GGATTTTATCTTATTAATTGGCAATTAATGATTCTAATCAAATTTTATTCTCTTTAGTTTAATGGTA
GAACAATGATCTTCTAATTCATTGGTTTTAGTTCGATTCTAAAAAGAGATGAGTAAATAATTTTCT
AGATCAGAAATACTACTTTTAACTACAAAAAGCTTACGCTTTTTAACATTTTTTTGATAAATAACA
ACTATTGTTAATATTTGGCTGTCTATTGGTTTAACTAACAATTACAAAATTTTTTCAATTTTTATAAT
ATAAATTTAAATGAGAATATTAAAAAGTCATTCATTATTAAAATTAGTGAATTCTTACCTTATCGA
TGCGTCACAACCAAGTAACATTAGTTACTTGTGAAATTTTGGTTCATTATTAGCTGTTTGTTTAAT
AGTACAAATTATTACCGGTATTACATTAGCTATGCATTATAGTCCTAGTGTAATGGAAGCTTTTAA
CTCAATAGAGCATATAATGAGAGATGTTAATAACGGGTGATTAGTTCGTTATCTACATAGTAATA
CAGCTTCTGCTTTCTTTTTCTTAGTGTATTTACACATAGGAAGAGGTATATATTACGGATCATATA

GAGCTCCTCGTACTTTAGTTTGAGCTATTGGTACTGTTATATTAATATTAATGATGGCTATCGGTT
TCCTAGGTTATGTTTTACCTTATGGACAGATGTCATTATGAGGTGCTACAGTTATTACTAATCTTA
TTAGTGCTATACCTTGAATAGGGCAAGATATTGTTGAATTCATTTGAGGTGGTTTTTCTGTTAATA
ATGCCACTTTAAACAGATTTTTTGCATTACATTTTGTATTGCCTTTTGTATTAGCTGCTTTAGTTTTA
ATGCACTTAATTGCACTTCATGATACTGCTGGTTCAAGCAATCCTCTTGGTGTTTCAGGTAATTAC
GATAGAATTACATTTGCTCCATATTTTTTATTTAAAGATTTAATTACTATTTTTATATTTATTTTTGTA
TTAAGTGCTTTTGTATTCTTTATGCCTAATGTTTTAGGGGATAGTGATAATTATATTATGGCTAATC
CTATGCAAACTCCTGCTGCTATTGTACCTGAATGATACTTATTACCTTTCTATGCTATTTTAAGATC
TATACCTAATAAATTATTAGGTGTTATAGCGATGTTTAGTGCTATTTTAGCTATTATGTTATTACCT
GTTACAGATTTAGGTAGATCTAGAGGTTTACAATTTAGACCATTTAGTAAAATAGCTTTCTGAGTT
TTTGTTGCTAATTTCTTAGTTTTAATGCAATTAGGTGCTAAACACGTTGAAGATCCATTTATATTAT
TAGGTCAATTAAGTACTGTATTATACTTTAGTTATTTTGTTGCTATATTACCTTTAGCTAGTTACTT
AGATAATAGTTTAACTGATTTATCTAATAAATCTGAATTATTTTTAAATAAAACTAACTAAATATAT
TAAGATTATTATTTAATATATTTTCTATTTAAGATACTATTAATTTAGTATTTTGGGTTTTCAGTTTA
TAATTTATATTATATTATGCATTACCCTCCACCTTGCTTTGTAGTAAGCTAATCTGTTATTTCCTTTA
GTTTAATGGTAGAACAATGATCTTCTAATTCATTGGTTTTAGTTCGAATCTAAAAAGGAAATAAG
AAATATATTCTTATTATTACTTATATAATAATTATTTCTTAAAAATATACATTTTGCATTATAGCCGT
TTAGCTGTATTAAAATGTAAAATGATATAAAATAGAATAAATATTTAAATTATTCCTATGTTATATT
ATCCTATATTGCAACCATTATCAGAAGTTGTATTAATACTTGTACCTGCCTTATTAGCTGTAGCTT
ATGTTACAGTTGCTGAAAGAAAAACTATGGCTAGTATGCAAAGAAGATTAGGTCCTAATGCTGT
AGGTTACTATGGACTATTGCAAGCATTTGCTGATGCCTTAAAACTTTTATTAAAAGAATATGTAG
CTCCTACACAATCTAATATTGTTCTTTTCTTTTTAGGTCCTGTAATAACTTTAATTTTTGCATTATTA
GGTTACGCTGTTATACCCTATGGTCCTGGTTCAGGGATAAGCGACATGAATTTAGGTATATTTTA
CATGTTAGCTGTGTCATCTTTAGCTACATACGGTATTCTATTAGCTGGTTGAAGTGCGAATAGTA
AATACGCTTTTCTAGGTTCTCTTAGAAGTACAGCTCAATTAATTAGTTATGAATTAATATTAAGTT
CAGCTATATTAATAGTAATTATGATAACAGGAAATTTAAATTTAACTGTTTGTACTGAATCTCAAA
GAGCTATTTGATTTATACTACCTTTATTTCCTGTGTTTATAATATTTTTCATAGGATCTATAGCTGA
GACAAATAGAGCTCCTTTTGATTTAGCCGAGGCTAACCTGCTAATCTGGTTTGGTCTGGTTATAT
GTCACAAATTGCTAGGAAACCTTTTTATTTTAAAAACAAAAGACAATTAGCAGGAAACTTAATTT
AACCTAATTAAAAATATTAGATAATTAAACTCTTCATAGACTAAACGTGACAATTTAATATATATA
TATATTTATTTATATATATGATTAAATAAGATATAGTCAATCATCGGTGTGAATCGACTTAAAAAA
AAAAGCACATGGGTTAAACCCATCTCCCCTTATTAGGGGAATCAGAACTTGTTAGTGGGTTCAT
GACAGAGCATGCTGCCGTAGTTTTCGTATTCTTCTTTTTAGCTGAGTACGGTAGTATTGTACTAAT
GTGTATTTTAACTAGTATATTATTTATTGGTGGTTACTTATTATTTGAAATATCCTATGTTTTTACTG
TGGTAAATTATATTTTCTTTGAATTATTCTTTATAGACTGAGTAACATTTGTAGAGGTACAATCTTT
ATACACTGATTTTTTAAATAATTCTATCATTGAAGGATTATTATATGGGTTTAATCTAGGATTAAA
AAGTTCTTTAATGATATTCACATTTATTTGAGCTAGAGCATCCTTCCCTAGAATACGATTTGATCA
ACTAATGGGCTTCTGTTGAACAGTTTTATTACCTATTAATTTTGCAATTATTATATTAGTACCTTGT
GTTTTATATAGTTTTAACTTATTACCTGTAAATATACCATTGTTCTAGCTCACACACCCGCCCGCC
CTACTGCCACAAGGCTACAGTACATATGAGGAGGGGAACTAAAGATCTAGAACTATCCTAGTTA
ATAATTACACTTAATAGTATACTAAATAGACCATCTATCATACTCGAGAATAGTGATAGTGTAAT
TATACACTATTTTAACTGTATTCTACACTATTAGCATATTATTATCTTTATATGATAATAATTTTATA
ACTTAATTTATTAGGTTACATTAACAAAAAATTCGTTAACTTTTTTCACCACTTTTTAATACAAAAT
ACGAATTATA

>YN567
ATAACAATTCTAAAGAACATAAAGAGTTATTAGATAAAAATAATTCACCTATACAGTTAATAAAT
CAACTTAAAGGGTATTTTTTCATAAATCCTTTATTAGCTTTAAGTTTAGCTATTACTATTTTCTCTTT
TGCAGGTATTCCTCCTCTTGTAGGGTTCTTTGCTAAACAGATGGTATTAAGCGCGGCTATTGATC
AAGGTTATATCTTTTTATCTTTAGTTGCAATATTAACTAGTGTTATAGGAGGGGTTTATTATTTAAA
TATAATTAAAGAAATGTTCTTTTATTCACCTGACTATAAATTAAACGAAGAAATTAAAAATAATAC
TATTAATGGTCAAATTATTAATAGAAACAATAAAATATTAAATGTTGAATTTAATTATACAAATGT
AGTTATGTCTAGTTCTGTGGCAATAACTATTTCTACTATTACATTAGTAGTTTTATTATTCATGTTT
ATGAATAAAGAATGATTAAGTCTGGGTACTATATTGGTACAATCTTTATTTAGCTATTAATGAGTA
GTATGACATTATTTATAGGGTTTGTATCTGTTATAGCTATTTTATTTTTAGCCATTAATTTTATATTT
GCTCCTCATAATCCTTATCAAGAAAAATATAGTATTTTCGAGTGTGGTTTCCATAGTTTTTTAGGG
CAAAATAGAACACAATTCGGTATAAAATTCTTTATTTTTGCTTTAGTTTATTTACTTTTAGATTTAG
AAATATTATTAACTTTCCCTTTCGCTCTTAGTGAGTATGTTAATGGTATTTATGGTCTTTTAGTTAC
TTTAATTTTTATAGCTATAATAACTATAGGATTTATATTTGAATTAGGTAAAAGCGCTCTTAAAATA
GACAGCAGACAAAAATTATATATACCTAAATTGAACGTTAATTACCATACAGAGTATGTTGGAAT
AGGTAAGGTTTCTAAGTAAAGTTATAGAGGCAGAAAACCAAAAAACCTACCAAAGGGTAGCTA
ATGGGAAGCTATTAAAAATAAAAGATGATAACCTATATATAGTATAGTTACTATATATTTACTATA
ACTAGGATTATTATATATATATATATTATCTTATTGTATATTAAGATTATTATTATTATAAGGTATA
ATTAATATAGTATCTTATTGTATAAGAATATAATATATTAACCTATAATTAATTTTATTTTTTAATTT
TTAATTATAATTTTTTTTTATATCTAGATGCTTACACATCTACAGATGTAGAAGAGAACAAAATAT
TGTTGTTATAGTAGTGGATGATATAGAAAAATATTTTTATATTATTTATTTTTGTTAGGTAGCTTTT
GAAGTGTTTGATAGAGAGGATATATGGACGGTAGGAGGGTATTCATTTTAATGAACAGTGGATA
GTTTAAATTAACCTAGTTATAGTTTATGAATTTAAATTATAATTATATTAATGTAGGTTATGATAGA
ATTATGTATTGATCCTTAAGAGTTAAGAGAGATACGCCCACGTATAATACATATTGGTTTAGGGT
TGAGTATATATACTTAAGAGTTAAGAATATATATATACAATATATAATAAATATAGTAATATATTA
ATGGTATGGACTTAACCAGGTTTATATATTATCATTTGATAAACATTAATTATAATTTTTCTATTTA
TTATTATTATTATACTGTGAGATTAATAATTATTAAAATATAATATTACATAGTACGTGATATTTGG
GGATTTTATCTTATTAATTGGCAATTAATGATTCTAATCAAATTTTATTCTCTTTAGTTTAATGGTA
GAACAATGATCTTCTAATTCATTGGTTTTAGTTCGATTCTAAAAAGAGATGAGTAAATAATTTTCT
AGATCAGAAATACTACTTTTAACTACAAAAAGCTTACGCTTTTTAACATTTTTTTGATAAATAACA
ACTATTGTTAATATTTGGCTGTCTATTGGTTTAACTAACAATTACAAAATTTTTTCAATTTTTATAAT
ATAAATTTAAATGAGAATATTAAAAAGTCATTCATTATTAAAATTAGTGAATTCTTACCTTATCGA
TGCGTCACAACCAAGTAACATTAGTTACTTGTGAAATTTTGGTTCATTATTAGCTGTTTGTTTAAT
AGTACAAATTATTACCGGTATTACATTAGCTATGCATTATAGTCCTAGTGTAATGGAAGCTTTTAA
CTCAATAGAGCATATAATGAGAGATGTTAATAACGGGTGATTAGTTCGTTATCTACATAGTAATA
CAGCTTCTGCTTTCTTTTTCTTAGTGTATTTACACATAGGAAGAGGTATATATTACGGATCATATA
GAGCTCCTCGTACTTTAGTTTGAGCTATTGGTACTGTTATATTAATATTAATGATGGCTATCGGTT
TCCTAGGTTATGTTTTACCTTATGGACAGATGTCATTATGAGGTGCTACAGTTATTACTAATCTTA
TTAGTGCTATACCTTGAATAGGGCAAGATATTGTTGAATTCATTTGAGGTGGTTTTTCTGTTAATA
ATGCCACTTTAAACAGATTTTTTGCATTACATTTTGTATTGCCTTTTGTATTAGCTGCTTTAGTTTTA
ATGCACTTAATTGCACTTCATGATACTGCTGGTTCAAGCAATCCTCTTGGTGTTTCAGGTAATTAC
GATAGAATTACATTTGCTCCATATTTTTTATTTAAAGATTTAATTACTATTTTTATATTTATTTTTGTA
TTAAGTGCTTTTGTATTCTTTATGCCTAATGTTTTAGGGGATAGTGATAATTATATTATGGCTAATC
CTATGCAAACTCCTGCTGCTATTGTACCTGAATGATACTTATTACCTTTCTATGCTATTTTAAGATC

TATACCTAATAAATTATTAGGTGTTATAGCGATGTTTAGTGCTATTTTAGCTATTATGTTATTACCT
GTTACAGATTTAGGTAGATCTAGAGGTTTACAATTTAGACCATTTAGTAAAATAGCTTTCTGAGTT
TTTGTTGCTAATTTCTTAGTTTTAATGCAATTAGGTGCTAAACACGTTGAAGATCCATTTATATTAT
TAGGTCAATTAAGTACTGTATTATACTTTAGTTATTTTGTTGCTATATTACCTTTAGCTAGTTACTT
AGATAATAGTTTAACTGATTTATCTAATAAATCTGAATTATTTTTAAATAAAACTAACTAAATATAT
TAAGATTATTATTTAATATATTTTCTATTTAAGATACTATTAATTTAGTATTTTGGGTTTTCAGTTTA
TAATTTATATTATATTATGCATTACCCTCCACCTTGCTTTGTAGTAAGCTAATCTGTTATTTCCTTTA
GTTTAATGGTAGAACAATGATCTTCTAATTCATTGGTTTTAGTTCGAATCTAAAAAGGAAATAAG
AAATATATTCTTATTATTACTTATATAATAATTATTTCTTAAAAATATACATTTTGCATTATAGCCGT
TTAGCTGTATTAAAATGTAAAATGATATAAAATAGAATAAATATTTAAATTATTCCTATGTTATATT
ATCCTATATTGCAACCATTATCAGAAGTTGTATTAATACTTGTACCTGCCTTATTAGCTGTAGCTT
ATGTTACAGTTGCTGAAAGAAAAACTATGGCTAGTATGCAAAGAAGATTAGGTCCTAATGCTGT
AGGTTACTATGGACTATTGCAAGCATTTGCTGATGCCTTAAAACTTTTATTAAAAGAATATGTAG
CTCCTACACAATCTAATATTGTTCTTTTCTTTTTAGGTCCTGTAATAACTTTAATTTTTGCATTATTA
GGTTACGCTGTTATACCCTATGGTCCTGGTTCAGGGATAAGCGACATGAATTTAGGTATATTTTA
CATGTTAGCTGTGTCATCTTTAGCTACATACGGTATTCTATTAGCTGGTTGAAGTGCGAATAGTA
AATACGCTTTTCTAGGTTCTCTTAGAAGTACAGCTCAATTAATTAGTTATGAATTAATATTAAGTT
CAGCTATATTAATAGTAATTATGATAACAGGAAATTTAAATTTAACTGTTTGTACTGAATCTCAAA
GAGCTATTTGATTTATACTACCTTTATTTCCTGTGTTTATAATATTTTTCATAGGATCTATAGCTGA
GACAAATAGAGCTCCTTTTGATTTAGCCGAGGCTAACCTGCTAATCTGGTTTGGTCTGGTTATAT
GTCACAAATTGCTAGGAAACCTTTTTATTTTAAAAACAAAAGACAATTAGCAGGAAACTTAATTT
AACCTAATTAAAAATATTAGATAATTAAACTCTTCATAGACTAAACGTGACAATTTAATATATATA
TATATTTATTTATATATATGATTAAATAAGATATAGTCAATCATCGGTGTGAATCGACTTAAAAAA
AAAAGCACATGGGTTAAACCCATCTCCCCTTATTAGGGGAATCAGAACTTGTTAGTGGGTTCAT
GACAGAGCATGCTGCCGTAGTTTTCGTATTCTTCTTTTTAGCTGAGTACGGTAGTATTGTACTAAT
GTGTATTTTAACTAGTATATTATTTATTGGTGGTTACTTATTATTTGAAATATCCTATGTTTTTACTG
TGGTAAATTATATTTTCTTTGAATTATTCTTTATAGACTGAGTAACATTTGTAGAGGTACAATCTTT
ATACACTGATTTTTTAAATAATTCTATCATTGAAGGATTATTATATGGGTTTAATCTAGGATTAAA
AAGTTCTTTAATGATATTCACATTTATTTGAGCTAGAGCATCCTTCCCTAGAATACGATTTGATCA
ACTAATGGGCTTCTGTTGAACAGTTTTATTACCTATTAATTTTGCAATTATTATATTAGTACCTTGT
GTTTTATATAGTTTTAACTTATTACCTGTAAATATACCATTGTTCTAGCTCACACACCCGCCCGCC
CTACTGCCACAAGGCTACAGTACATATGAGGAGGGGAACTAAAGATCTAGAACTATCCTAGTTA
ATAATTACACTTAATAGTATACTAAATAGACCATCTATCATACTCGAGAATAGTGATAGTGTAAT
TATACACTATTTTAACTGTATTCTACACTATTAGCATATTATTATCTTTATATGATAATAATTTTATA
ACTTAATTTATTAGGTTACATTAACAAAAAATTCGTTAACTTTTTTCACCACTTTTTAATACAAAAT
ACGAATTTTA
>YN604
ATAACAATTCTAAAGAACATAAAGAGTTATTAGATAAAAATAATTCACCTATACAGTTAATAAAT
CAACTTAAAGGGTATTTTTTCATAAATCCTTTATTAGCTTTAAGTTTAGCTATTACTATTTTCTCTTT
TGCAGGTATTCCTCCTCTTGTAGGGTTCTTTGCTAAACAGATGGTATTAAGCGCGGCTATTGATC
AAGGTTATATCTTTTTATCTTTAGTTGCAATATTAACTAGTGTTATAGGAGGGGTTTATTATTTAAA
TATAATTAAAGAAATGTTCTTTTATTCACCTGACTATAAATTAAACGAAGAAATTAAAAATAATAC
TATTAATGGTCAAATTATTAATAGAAACAATAAAATATTAAATGTTGAATTTAATTATACAAATGT
AGTTATGTCTAGTTCTGTGGCAATAACTATTTCTACTATTACATTAGTAGTTTTATTATTCATGTTT

ATGAATAAAGAATGATTAAGTCTGGGTACTATATTGGTACAATCTTTATTTAGCTATTAATGAGTA
GTATGACATTATTTATAGGGTTTGTATCTGTTATAGCTATTTTATTTTTAGCCATTAATTTTATATTT
GCTCCTCATAATCCTTATCAAGAAAAATATAGTATTTTCGAGTGTGGTTTCCATAGTTTTTTAGGG
CAAAATAGAACACAATTCGGTATAAAATTCTTTATTTTTGCTTTAGTTTATTTACTTTTAGATTTAG
AAATATTATTAACTTTCCCTTTCGCTCTTAGTGAGTATGTTAATGGTATTTATGGTCTTTTAGTTAC
TTTAATTTTTATAGCTATAATAACTATAGGATTTATATTTGAATTAGGTAAAAGCGCTCTTAAAATA
GACAGCAGACAAAAATTATATATACCTAAATTGAACGTTAATTACCATACAGAGTATGTTGGAAT
AGGTAAGGTTTCTAAGTAAAGTTATAGAGGCAGAAAACCAAAAAACCTACCAAAGGGTAGCTA
ATGGGAAGCTATTAAAAATAAAAGATGATAACCTATATATAGTATAGTTACTATATATTTACTATA
ACTAGGATTATTATATATATATATATTATCTTATTGTATATTAAGATTATTATTATTATAAGGTATA
ATTAATATAGTATCTTATTGTATAAGAATATAATATATTAACCTATAATTAATTTTATTTTTTAATTT
TTAATTATAATTTTTTTTTATATCTAGATGCTTACACATCTACAGATGTAGAAGAGAACAAAATAT
TGTTGTTATAGTAGTGGATGATATAGAAAAATATTTTTATATTATTTATTTTTGTTAGGTAGCTTTT
GAAGTGTTTGATAGAGAGGATATATGGACGGTAGGAGGGTATTCATTTTAATGAACAGTGGATA
GTTTAAATTAACCTAGTTATAGTTTATGAATTTAAATTATAATTATATTAATGTAGGTTATGATAGA
ATTATGTATTGATCCTTAAGAGTTAAGAGAGATACGCCCACGTATAATACATATTGGTTTAGGGT
TGAGTATATATACTTAAGAGTTAAGAATATATATATACAATATATAATAAATATAGTAATATATTA
ATGGTATGGACTTAACCAGGTTTATATATTATCATTTGATAAACATTAATTATAATTTTTCTATTTA
TTATTATTATTATACTGTGAGATTAATAATTATTAAAATATAATATTACATAGTACGTGATATTTGG
GGATTTTATCTTATTAATTGGCAATTAATGATTCTAATCAAATTTTATTCTCTTTAGTTTAATGGTA
GAACAATGATCTTCTAATTCATTGGTTTTAGTTCGATTCTAAAAAGAGATGAGTAAATAATTTTCT
AGATCAGAAATACTACTTTTAACTACAAAAAGCTTACGCTTTTTAACATTTTTTTGATAAATAACA
ACTATTGTTAATATTTGGCTGTCTATTGGTTTAACTAACAATTACAAAATTTTTTCAATTTTTATAAT
ATAAATTTAAATGAGAATATTAAAAAGTCATTCATTATTAAAATTAGTGAATTCTTACCTTATCGA
TGCGTCACAACCAAGTAACATTAGTTACTTGTGAAATTTTGGTTCATTATTAGCTGTTTGTTTAAT
AGTACAAATTATTACCGGTATTACATTAGCTATGCATTATAGTCCTAGTGTAATGGAAGCTTTTAA
CTCAATAGAGCATATAATGAGAGATGTTAATAACGGGTGATTAGTTCGTTATCTACATAGTAATA
CAGCTTCTGCTTTCTTTTTCTTAGTGTATTTACACATAGGAAGAGGTATATATTACGGATCATATA
GAGCTCCTCGTACTTTAGTTTGAGCTATTGGTACTGTTATATTAATATTAATGATGGCTATCGGTT
TCCTAGGTTATGTTTTACCTTATGGACAGATGTCATTATGAGGTGCTACAGTTATTACTAATCTTA
TTAGTGCTATACCTTGAATAGGGCAAGATATTGTTGAATTCATTTGAGGTGGTTTTTCTGTTAATA
ATGCCACTTTAAACAGATTTTTTGCATTACATTTTGTATTGCCTTTTGTATTAGCTGCTTTAGTTTTA
ATGCACTTAATTGCACTTCATGATACTGCTGGTTCAAGCAATCCTCTTGGTGTTTCAGGTAATTAC
GATAGAATTACATTTGCTCCATATTTTTTATTTAAAGATTTAATTACTATTTTTATATTTATTTTTGTA
TTAAGTGCTTTTGTATTCTTTATGCCTAATGTTTTAGGGGATAGTGATAATTATATTATGGCTAATC
CTATGCAAACTCCTGCTGCTATTGTACCTGAATGATACTTATTACCTTTCTATGCTATTTTAAGATC
TATACCTAATAAATTATTAGGTGTTATAGCGATGTTTAGTGCTATTTTAGCTATTATGTTATTACCT
GTTACAGATTTAGGTAGATCTAGAGGTTTACAATTTAGACCATTTAGTAAAATAGCTTTCTGAGTT
TTTGTTGCTAATTTCTTAGTTTTAATGCAATTAGGTGCTAAACACGTTGAAGATCCATTTATATTAT
TAGGTCAATTAAGTACTGTATTATACTTTAGTTATTTTGTTGCTATATTACCTTTAGCTAGTTACTT
AGATAATAGTTTAACTGATTTATCTAATAAATCTGAATTATTTTTAAATAAAACTAACTAAATATAT
TAAGATTATTATTTAATATATTTTCTATTTAAGATACTATTAATTTAGTATTTTGGGTTTTCAGTTTA
TAATTTATATTATATTATGCATTACCCTCCACCTTGCTTTGTAGTAAGCTAATCTGTTATTTCCTTTA
GTTTAATGGTAGAACAATGATCTTCTAATTCATTGGTTTTAGTTCGAATCTAAAAAGGAAATAAG

AAATATATTCTTATTATTACTTATATAATAATTATTTCTTAAAAATATACATTTTGCATTATAGCCGT
TTAGCTGTATTAAAATGTAAAATGATATAAAATAGAATAAATATTTAAATTATTCCTATGTTATATT
ATCCTATATTGCAACCATTATCAGAAGTTGTATTAATACTTGTACCTGCCTTATTAGCTGTAGCTT
ATGTTACAGTTGCTGAAAGAAAAACTATGGCTAGTATGCAAAGAAGATTAGGTCCTAATGCTGT
AGGTTACTATGGACTATTGCAAGCATTTGCTGATGCCTTAAAACTTTTATTAAAAGAATATGTAG
CTCCTACACAATCTAATATTGTTCTTTTCTTTTTAGGTCCTGTAATAACTTTAATTTTTGCATTATTA
GGTTACGCTGTTATACCCTATGGTCCTGGTTCAGGGATAAGCGACATGAATTTAGGTATATTTTA
CATGTTAGCTGTGTCATCTTTAGCTACATACGGTATTCTATTAGCTGGTTGAAGTGCGAATAGTA
AATACGCTTTTCTAGGTTCTCTTAGAAGTACAGCTCAATTAATTAGTTATGAATTAATATTAAGTT
CAGCTATATTAATAGTAATTATGATAACAGGAAATTTAAATTTAACTGTTTGTACTGAATCTCAAA
GAGCTATTTGATTTATACTACCTTTATTTCCTGTGTTTATAATATTTTTCATAGGATCTATAGCTGA
GACAAATAGAGCTCCTTTTGATTTAGCCGAGGCTAACCTGCTAATCTGGTTTGGTCTGGTTATAT
GTCACAAATTGCTAGGAAACCTTTTTATTTTAAAAACAAAAGACAATTAGCAGGAAACTTAATTT
AACCTAATTAAAAATATTAGATAATTAAACTCTTCATAGACTAAACGTGACAATTTAATATATATA
TATATTTATTTATATATATGATTAAATAAGATATAGTCAATCATCGGTGTGAATCGACTTAAAAAA
AAAAGCACATGGGTTAAACCCATCTCCCCTTATTAGGGGAATCAGAACTTGTTAGTGGGTTCAT
GACAGAGCATGCTGCCGTAGTTTTCGTATTCTTCTTTTTAGCTGAGTACGGTAGTATTGTACTAAT
GTGTATTTTAACTAGTATATTATTTATTGGTGGTTACTTATTATTTGAAATATCCTATGTTTTTACTG
TGGTAAATTATATTTTCTTTGAATTATTCTTTATAGACTGAGTAACATTTGTAGAGGTACAATCTTT
ATACACTGATTTTTTAAATAATTCTATCATTGAAGGATTATTATATGGGTTTAATCTAGGATTAAA
AAGTTCTTTAATGATATTCACATTTATTTGAGCTAGAGCATCCTTCCCTAGAATACGATTTGATCA
ACTAATGGGCTTCTGTTGAACAGTTTTATTACCTATTAATTTTGCAATTATTATATTAGTACCTTGT
GTTTTATATAGTTTTAACTTATTACCTGTAAATATACCATTGTTCTAGCTCACACACCCGCCCGCC
CTACTGCCACAAGGCTACAGTACATATGAGGAGGGGAACTAAAGATCTAGAACTATCCTAGTTA
ATAATTACACTTAATAGTATACTAAATAGACCATCTATCATACTCGAGAATAGTGATAGTGTAAT
TATACACTATTTTAACTGTATTCTACACTATTAGCATATTATTATCTTTATATGATAATAATTTTATA
ACTTAATTTATTAGGTTACATTAACAAAAAATTCGTTAACTTTTTTCACCACTTTTTAATACAAAAT
ACGAATTTTA
>YN608
ATAACAATTCTAAAGAACATAAAGAGTTATTAGATAAAAATAATTCACCTATACAGTTAATAAAT
CAACTTAAAGGGTATTTTTTCATAAATCCTTTATTAGCTTTAAGTTTAGCTATTACTATTTTCTCTTT
TGCAGGTATTCCTCCTCTTGTAGGGTTCTTTGCTAAACAGATGGTATTAAGCGCGGCTATTGATC
AAGGTTATATCTTTTTATCTTTAGTTGCAATATTAACTAGTGTTATAGGAGGGGTTTATTATTTAAA
TATAATTAAAGAAATGTTCTTTTATTCACCTGACTATAAATTAAACGAAGAAATTAAAAATAATAC
TATTAATGGTCAAATTATTAATAGAAACAATAAAATATTAAATGTTGAATTTAATTATACAAATGT
AGTTATGTCTAGTTCTGTGGCAATAACTATTTCTACTATTACATTAGTAGTTTTATTATTCATGTTT
ATGAATAAAGAATGATTAAGTCTGGGTACTATATTGGTACAATCTTTATTTAGCTATTAATGAGTA
GTATGACATTATTTATAGGGTTTGTATCTGTTATAGCTATTTTATTTTTAGCCATTAATTTTATATTT
GCTCCTCATAATCCTTATCAAGAAAAATATAGTATTTTCGAGTGTGGTTTCCATAGTTTTTTAGGG
CAAAATAGAACACAATTCGGTATAAAATTCTTTATTTTTGCTTTAGTTTATTTACTTTTAGATTTAG
AAATATTATTAACTTTCCCTTTCGCTCTTAGTGAGTATGTTAATGGTATTTATGGTCTTTTAGTTAC
TTTAATTTTTATAGCTATAATAACTATAGGATTTATATTTGAATTAGGTAAAAGCGCTCTTAAAATA
GACAGCAGACAAAAATTATATATACCTAAATTGAACGTTAATTACCATACAGAGTATGTTGGAAT
AGGTAAGGTTTCTAAGTAAAGTTATAGAGGCAGAAAACCAAAAAACCTACCAAAGGGTAGCTA

ATGGGAAGCTATTAAAAATAAAAGATGATAACCTATATATAGTATAGTTACTATATATTTACTATA
ACTAGGATTATTATATATATATATATTATCTTATTGTATATTAAGATTATTATTATTATAAGGTATA
ATTAATATAGTATCTTATTGTATAAGAATATAATATATTAACCTATAATTAATTTTATTTTTTAATTT
TTAATTATAATTTTTTTTTATATCTAGATGCTTACACATCTACAGATGTAGAAGAGAACAAAATAT
TGTTGTTATAGTAGTGGATGATATAGAAAAATATTTTTATATTATTTATTTTTGTTAGGTAGCTTTT
GAAGTGTTTGATAGAGAGGATATATGGACGGTAGGAGGGTATTCATTTTAATGAACAGTGGATA
GTTTAAATTAACCTAGTTATAGTTTATGAATTTAAATTATAATTATATTAATGTAGGTTATGATAGA
ATTATGTATTGATCCTTAAGAGTTAAGAGAGATACGCCCACGTATAATACATATTGGTTTAGGGT
TGAGTATATATACTTAAGAGTTAAGAATATATATATACAATATATAATAAATATAGTAATATATTA
ATGGTATGGACTTAACCAGGTTTATATATTATCATTTGATAAACATTAATTATAATTTTTCTATTTA
TTATTATTATTATACTGTGAGATTAATAATTATTAAAATATAATATTACATAGTACGTGATATTTGG
GGATTTTATCTTATTAATTGGCAATTAATGATTCTAATCAAATTTTATTCTCTTTAGTTTAATGGTA
GAACAATGATCTTCTAATTCATTGGTTTTAGTTCGATTCTAAAAAGAGATGAGTAAATAATTTTCT
AGATCAGAAATACTACTTTTAACTACAAAAAGCTTACGCTTTTTAACATTTTTTTGATAAATAACA
ACTATTGTTAATATTTGGCTGTCTATTGGTTTAACTAACAATTACAAAATTTTTTCAATTTTTATAAT
ATAAATTTAAATGAGAATATTAAAAAGTCATTCATTATTAAAATTAGTGAATTCTTACCTTATCGA
TGCGTCACAACCAAGTAACATTAGTTACTTGTGAAATTTTGGTTCATTATTAGCTGTTTGTTTAAT
AGTACAAATTATTACCGGTATTACATTAGCTATGCATTATAGTCCTAGTGTAATGGAAGCTTTTAA
CTCAATAGAGCATATAATGAGAGATGTTAATAACGGGTGATTAGTTCGTTATCTACATAGTAATA
CAGCTTCTGCTTTCTTTTTCTTAGTGTATTTACACATAGGAAGAGGTATATATTACGGATCATATA
GAGCTCCTCGTACTTTAGTTTGAGCTATTGGTACTGTTATATTAATATTAATGATGGCTATCGGTT
TCCTAGGTTATGTTTTACCTTATGGACAGATGTCATTATGAGGTGCTACAGTTATTACTAATCTTA
TTAGTGCTATACCTTGAATAGGGCAAGATATTGTTGAATTCATTTGAGGTGGTTTTTCTGTTAATA
ATGCCACTTTAAACAGATTTTTTGCATTACATTTTGTATTGCCTTTTGTATTAGCTGCTTTAGTTTTA
ATGCACTTAATTGCACTTCATGATACTGCTGGTTCAAGCAATCCTCTTGGTGTTTCAGGTAATTAC
GATAGAATTACATTTGCTCCATATTTTTTATTTAAAGATTTAATTACTATTTTTATATTTATTTTTGTA
TTAAGTGCTTTTGTATTCTTTATGCCTAATGTTTTAGGGGATAGTGATAATTATATTATGGCTAATC
CTATGCAAACTCCTGCTGCTATTGTACCTGAATGATACTTATTACCTTTCTATGCTATTTTAAGATC
TATACCTAATAAATTATTAGGTGTTATAGCGATGTTTAGTGCTATTTTAGCTATTATGTTATTACCT
GTTACAGATTTAGGTAGATCTAGAGGTTTACAATTTAGACCATTTAGTAAAATAGCTTTCTGAGTT
TTTGTTGCTAATTTCTTAGTTTTAATGCAATTAGGTGCTAAACACGTTGAAGATCCATTTATATTAT
TAGGTCAATTAAGTACTGTATTATACTTTAGTTATTTTGTTGCTATATTACCTTTAGCTAGTTACTT
AGATAATAGTTTAACTGATTTATCTAATAAATCTGAATTATTTTTAAATAAAACTAACTAAATATAT
TAAGATTATTATTTAATATATTTTCTATTTAAGATACTATTAATTTAGTATTTTGGGTTTTCAGTTTA
TAATTTATATTATATTATGCATTACCCTCCACCTTGCTTTGTAGTAAGCTAATCTGTTATTTCCTTTA
GTTTAATGGTAGAACAATGATCTTCTAATTCATTGGTTTTAGTTCGAATCTAAAAAGGAAATAAG
AAATATATTCTTATTATTACTTATATAATAATTATTTCTTAAAAATATACATTTTGCATTATAGCCGT
TTAGCTGTATTAAAATGTAAAATGATATAAAATAGAATAAATATTTAAATTATTCCTATGTTATATT
ATCCTATATTGCAACCATTATCAGAAGTTGTATTAATACTTGTACCTGCCTTATTAGCTGTAGCTT
ATGTTACAGTTGCTGAAAGAAAAACTATGGCTAGTATGCAAAGAAGATTAGGTCCTAATGCTGT
AGGTTACTATGGACTATTGCAAGCATTTGCTGATGCCTTAAAACTTTTATTAAAAGAATATGTAG
CTCCTACACAATCTAATATTGTTCTTTTCTTTTTAGGTCCTGTAATAACTTTAATTTTTGCATTATTA
GGTTACGCTGTTATACCCTATGGTCCTGGTTCAGGGATAAGCGACATGAATTTAGGTATATTTTA
CATGTTAGCTGTGTCATCTTTAGCTACATACGGTATTCTATTAGCTGGTTGAAGTGCGAATAGTA

AATACGCTTTTCTAGGTTCTCTTAGAAGTACAGCTCAATTAATTAGTTATGAATTAATATTAAGTT
CAGCTATATTAATAGTAATTATGATAACAGGAAATTTAAATTTAACTGTTTGTACTGAATCTCAAA
GAGCTATTTGATTTATACTACCTTTATTTCCTGTGTTTATAATATTTTTCATAGGATCTATAGCTGA
GACAAATAGAGCTCCTTTTGATTTAGCCGAGGCTAACCTGCTAATCTGGTTTGGTCTGGTTATAT
GTCACAAATTGCTAGGAAACCTTTTTATTTTAAAAACAAAAGACAATTAGCAGGAAACTTAATTT
AACCTAATTAAAAATATTAGATAATTAAACTCTTCATAGACTAAACGTGACAATTTAATATATATA
TATATTTATTTATATATATGATTAAATAAGATATAGTCAATCATCGGTGTGAATCGACTTAAAAAA
AAAAGCACATGGGTTAAACCCATCTCCCCTTATTAGGGGAATCAGAACTTGTTAGTGGGTTCAT
GACAGAGCATGCTGCCGTAGTTTTCGTATTCTTCTTTTTAGCTGAGTACGGTAGTATTGTACTAAT
GTGTATTTTAACTAGTATATTATTTATTGGTGGTTACTTATTATTTGAAATATCCTATGTTTTTACTG
TGGTAAATTATATTTTCTTTGAATTATTCTTTATAGACTGAGTAACATTTGTAGAGGTACAATCTTT
ATACACTGATTTTTTAAATAATTCTATCATTGAAGGATTATTATATGGGTTTAATCTAGGATTAAA
AAGTTCTTTAATGATATTCACATTTATTTGAGCTAGAGCATCCTTCCCTAGAATACGATTTGATCA
ACTAATGGGCTTCTGTTGAACAGTTTTATTACCTATTAATTTTGCAATTATTATATTAGTACCTTGT
GTTTTATATAGTTTTAACTTATTACCTGTAAATATACCATTGTTCTAGCTCACACACCCGCCCGCC
CTACTGCCACAAGGCTACAGTACATATGAGGAGGGGAACTAAAGATCTAGAACTATCCTAGTTA
ATAATTACACTTAATAGTATACTAAATAGACCATCTATCATACTCGAGAATAGTGATAGTGTAAT
TATACACTATTTTAACTGTATTCTACACTATTAGCATATTATTATCTTTATATGATAATAATTTTATA
ACTTAATTTATTAGGTTACATTAACAAAAAATTCGTTAACTTTTTTCACCACTTTTTAATACAAAAT
ACGAATTTTA
>YN617
ATAACAATTCTAAAGAACATAAAGAGTTATTAGATAAAAATAATTCACCTATACAGTTAATAAAT
CAACTTAAAGGGTATTTTTTCATAAATCCTTTATTAGCTTTAAGTTTAGCTATTACTATTTTCTCTTT
TGCAGGTATTCCTCCTCTTGTAGGGTTCTTTGCTAAACAGATGGTATTAAGCGCGGCTATTGATC
AAGGTTATATCTTTTTATCTTTAGTTGCAATATTAACTAGTGTTATAGGAGGGGTTTATTATTTAAA
TATAATTAAAGAAATGTTCTTTTATTCACCTGACTATAAATTAAACGAAGAAATTAAAAATAATAC
TATTAATGGTCAAATTATTAATAGAAACAATAAAATATTAAATGTTGAATTTAATTATACAAATGT
AGTTATGTCTAGTTCTGTGGCAATAACTATTTCTACTATTACATTAGTAGTTTTATTATTCATGTTT
ATGAATAAAGAATGATTAAGTCTGGGTACTATATTGGTACAATCTTTATTTAGCTATTAATGAGTA
GTATGACATTATTTATAGGGTTTGTATCTGTTATAGCTATTTTATTTTTAGCCATTAATTTTATATTT
GCTCCTCATAATCCTTATCAAGAAAAATATAGTATTTTCGAGTGTGGTTTCCATAGTTTTTTAGGG
CAAAATAGAACACAATTCGGTATAAAATTCTTTATTTTTGCTTTAGTTTATTTACTTTTAGATTTAG
AAATATTATTAACTTTCCCTTTCGCTCTTAGTGAGTATGTTAATGGTATTTATGGTCTTTTAGTTAC
TTTAATTTTTATAGCTATAATAACTATAGGATTTATATTTGAATTAGGTAAAAGCGCTCTTAAAATA
GACAGCAGACAAAAATTATATATACCTAAATTGAACGTTAATTACCATACAGAGTATGTTGGAAT
AGGTAAGGTTTCTAAGTAAAGTTATAGAGGCAGAAAACCAAAAAACCTACCAAAGGGTAGCTA
ATGGGAAGCTATTAAAAATAAAAGATGATAACCTATATATAGTATAGTTACTATATATTTACTATA
ACTAGGATTATTATATATATATATATTATCTTATTGTATATTAAGATTATTATTATTATAAGGTATA
ATTAATATAGTATCTTATTGTATAAGAATATAATATATTAACCTATAATTAATTTTATTTTTTAATTT
TTAATTATAATTTTTTTTTATATCTAGATGCTTACACATCTACAGATGTAGAAGAGAACAAAATAT
TGTTGTTATAGTAGTGGATGATATAGAAAAATATTTTTATATTATTTATTTTTGTTAGGTAGCTTTT
GAAGTGTTTGATAGAGAGGATATATGGACGGTAGGAGGGTATTCATTTTAATGAACAGTGGATA
GTTTAAATTAACCTAGTTATAGTTTATGAATTTAAATTATAATTATATTAATGTAGGTTATGATAGA
ATTATGTATTGATCCTTAAGAGTTAAGAGAGATACGCCCACGTATAATACATATTGGTTTAGGGT

TGAGTATATATACTTAAGAGTTAAGAATATATATATACAATATATAATAAATATAGTAATATATTA
ATGGTATGGACTTAACCAGGTTTATATATTATCATTTGATAAACATTAATTATAATTTTTCTATTTA
TTATTATTATTATACTGTGAGATTAATAATTATTAAAATATAATATTACATAGTACGTGATATTTGG
GGATTTTATCTTATTAATTGGCAATTAATGATTCTAATCAAATTTTATTCTCTTTAGTTTAATGGTA
GAACAATGATCTTCTAATTCATTGGTTTTAGTTCGATTCTAAAAAGAGATGAGTAAATAATTTTCT
AGATCAGAAATACTACTTTTAACTACAAAAAGCTTACGCTTTTTAACATTTTTTTGATAAATAACA
ACTATTGTTAATATTTGGCTGTCTATTGGTTTAACTAACAATTACAAAATTTTTTCAATTTTTATAAT
ATAAATTTAAATGAGAATATTAAAAAGTCATTCATTATTAAAATTAGTGAATTCTTACCTTATCGA
TGCGTCACAACCAAGTAACATTAGTTACTTGTGAAATTTTGGTTCATTATTAGCTGTTTGTTTAAT
AGTACAAATTATTACCGGTATTACATTAGCTATGCATTATAGTCCTAGTGTAATGGAAGCTTTTAA
CTCAATAGAGCATATAATGAGAGATGTTAATAACGGGTGATTAGTTCGTTATCTACATAGTAATA
CAGCTTCTGCTTTCTTTTTCTTAGTGTATTTACACATAGGAAGAGGTATATATTACGGATCATATA
GAGCTCCTCGTACTTTAGTTTGAGCTATTGGTACTGTTATATTAATATTAATGATGGCTATCGGTT
TCCTAGGTTATGTTTTACCTTATGGACAGATGTCATTATGAGGTGCTACAGTTATTACTAATCTTA
TTAGTGCTATACCTTGAATAGGGCAAGATATTGTTGAATTCATTTGAGGTGGTTTTTCTGTTAATA
ATGCCACTTTAAACAGATTTTTTGCATTACATTTTGTATTGCCTTTTGTATTAGCTGCTTTAGTTTTA
ATGCACTTAATTGCACTTCATGATACTGCTGGTTCAAGCAATCCTCTTGGTGTTTCAGGTAATTAC
GATAGAATTACATTTGCTCCATATTTTTTATTTAAAGATTTAATTACTATTTTTATATTTATTTTTGTA
TTAAGTGCTTTTGTATTCTTTATGCCTAATGTTTTAGGGGATAGTGATAATTATATTATGGCTAATC
CTATGCAAACTCCTGCTGCTATTGTACCTGAATGATACTTATTACCTTTCTATGCTATTTTAAGATC
TATACCTAATAAATTATTAGGTGTTATAGCGATGTTTAGTGCTATTTTAGCTATTATGTTATTACCT
GTTACAGATTTAGGTAGATCTAGAGGTTTACAATTTAGACCATTTAGTAAAATAGCTTTCTGAGTT
TTTGTTGCTAATTTCTTAGTTTTAATGCAATTAGGTGCTAAACACGTTGAAGATCCATTTATATTAT
TAGGTCAATTAAGTACTGTATTATACTTTAGTTATTTTGTTGCTATATTACCTTTAGCTAGTTACTT
AGATAATAGTTTAACTGATTTATCTAATAAATCTGAATTATTTTTAAATAAAACTAACTAAATATAT
TAAGATTATTATTTAATATATTTTCTATTTAAGATACTATTAATTTAGTATTTTGGGTTTTCAGTTTA
TAATTTATATTATATTATGCATTACCCTCCACCTTGCTTTGTAGTAAGCTAATCTGTTATTTCCTTTA
GTTTAATGGTAGAACAATGATCTTCTAATTCATTGGTTTTAGTTCGAATCTAAAAAGGAAATAAG
AAATATATTCTTATTATTACTTATATAATAATTATTTCTTAAAAATATACATTTTGCATTATAGCCGT
TTAGCTGTATTAAAATGTAAAATGATATAAAATAGAATAAATATTTAAATTATTCCTATGTTATATT
ATCCTATATTGCAACCATTATCAGAAGTTGTATTAATACTTGTACCTGCCTTATTAGCTGTAGCTT
ATGTTACAGTTGCTGAAAGAAAAACTATGGCTAGTATGCAAAGAAGATTAGGTCCTAATGCTGT
AGGTTACTATGGACTATTGCAAGCATTTGCTGATGCCTTAAAACTTTTATTAAAAGAATATGTAG
CTCCTACACAATCTAATATTGTTCTTTTCTTTTTAGGTCCTGTAATAACTTTAATTTTTGCATTATTA
GGTTACGCTGTTATACCCTATGGTCCTGGTTCAGGGATAAGCGACATGAATTTAGGTATATTTTA
CATGTTAGCTGTGTCATCTTTAGCTACATACGGTATTCTATTAGCTGGTTGAAGTGCGAATAGTA
AATACGCTTTTCTAGGTTCTCTTAGAAGTACAGCTCAATTAATTAGTTATGAATTAATATTAAGTT
CAGCTATATTAATAGTAATTATGATAACAGGAAATTTAAATTTAACTGTTTGTACTGAATCTCAAA
GAGCTATTTGATTTATACTACCTTTATTTCCTGTGTTTATAATATTTTTCATAGGATCTATAGCTGA
GACAAATAGAGCTCCTTTTGATTTAGCCGAGGCTAACCTGCTAATCTGGTTTGGTCTGGTTATAT
GTCACAAATTGCTAGGAAACCTTTTTATTTTAAAAACAAAAGACAATTAGCAGGAAACTTAATTT
AACCTAATTAAAAATATTAGATAATTAAACTCTTCATAGACTAAACGTGACAATTTAATATATATA
TATATTTATTTATATATATGATTAAATAAGATATAGTCAATCATCGGTGTGAATCGACTTAAAAAA
AAAAGCACATGGGTTAAACCCATCTCCCCTTATTAGGGGAATCAGAACTTGTTAGTGGGTTCAT

GACAGAGCATGCTGCCGTAGTTTTCGTATTCTTCTTTTTAGCTGAGTACGGTAGTATTGTACTAAT
GTGTATTTTAACTAGTATATTATTTATTGGTGGTTACTTATTATTTGAAATATCCTATGTTTTTACTG
TGGTAAATTATATTTTCTTTGAATTATTCTTTATAGACTGAGTAACATTTGTAGAGGTACAATCTTT
ATACACTGATTTTTTAAATAATTCTATCATTGAAGGATTATTATATGGGTTTAATCTAGGATTAAA
AAGTTCTTTAATGATATTCACATTTATTTGAGCTAGAGCATCCTTCCCTAGAATACGATTTGATCA
ACTAATGGGCTTCTGTTGAACAGTTTTATTACCTATTAATTTTGCAATTATTATATTAGTACCTTGT
GTTTTATATATAGTTTTAACTTATTACCTGTAAATATACCATTGTTCTAGCTCACACACCCGCCCGCC
CTACTGCCACAAGGCTACAGTACATATGAGGAGGGGAACTAAAGATCTAGAACTATCCTAGTTA
ATAATTACACTTAATAGTATACTAAATAGACCATCTATCATACTCGAGAATAGTGATAGTGTAAT
TATACACTATTTTAACTGTATTCTACACTATTAGCATATTATTATCTTTATATGATAATAATTTTATA
ACTTAATTTATTAGGTTACATTAACAAAAAAATTCGTTAACTTTTTTCACCACTTTTTAATACAAAAT
ACGAATTAAA
>YN626
ATAACAATTCTAAAGAACATAAAGAGTTATTAGATAAAAATAATTCACCTATACAGTTAATAAAT
CAACTTAAAGGGTATTTTTTCATAAATCCTTTATTAGCTTTAAGTTTAGCTATTACTATTTTCTCTTT
TGCAGGTATTCCTCCTCTTGTAGGGTTCTTTGCTAAACAGATGGTATTAAGCGCGGCTATTGATC
AAGGTTATATCTTTTTATCTTTAGTTGCAATATTAACTAGTGTTATAGGAGGGGTTTATTATTTAAA
TATAATTAAAGAAATGTTCTTTTATTCACCTGACTATAAATTAAACGAAGAAATTAAAAATAATAC
TATTAATGGTCAAATTATTAATAGAAACAATAAAATATTAAATGTTGAATTTAATTATACAAATGT
AGTTATGTCTAGTTCTGTGGCAATAACTATTTCTACTATTACATTAGTAGTTTTATTATTCATGTTT
ATGAATAAAGAATGATTAAGTCTGGGTACTATATTGGTACAATCTTTATTTAGCTATTAATGAGTA
GTATGACATTATTTATAGGGTTTGTATCTGTTATAGCTATTTTATTTTTAGCCATTAATTTTATATTT
GCTCCTCATAATCCTTATCAAGAAAAATATAGTATTTTCGAGTGTGGTTTCCATAGTTTTTTAGGG
CAAAATAGAACACAATTCGGTATAAAATTCTTTATTTTTGCTTTAGTTTATTTACTTTTAGATTTAG
AAATATTATTAACTTTCCCTTTCGCTCTTAGTGAGTATGTTAATGGTATTTATGGTCTTTTAGTTAC
TTTAATTTTTATAGCTATAATAACTATAGGATTTATATTTGAATTAGGTAAAAGCGCTCTTAAAATA
GACAGCAGACAAAAATTATATATACCTAAATTGAACGTTAATTACCATACAGAGTATGTTGGAAT
AGGTAAGGTTTCTAAGTAAAGTTATAGAGGCAGAAAACCAAAAAACCTACCAAAGGGTAGCTA
ATGGGAAGCTATTAAAAATAAAAGATGATAACCTATATATAGTATAGTTACTATATATTTACTATA
ACTAGGATTATTATATATATATATATATTATCTTATTGTATATTAAGATTATTATTATTATAAGGTATA
ATTAATATAGTATCTTATTGTATAAGAATATAATATATTAACCTATAATTAATTTTATTTTTTAATTT
TTAATTATAATTTTTTTTTATATCTAGATGCTTACACATCTACAGATGTAGAAGAGAACAAAATAT
TGTTGTTATAGTAGTGGATGATATAGAAAAATATTTTTATATTATTTATTTTTGTTAGGTAGCTTTT
GAAGTGTTTGATAGAGAGGATATATGGACGGTAGGAGGGTATTCATTTTAATGAACAGTGGATA
GTTTAAATTAACCTAGTTATAGTTTATGAATTTAAATTATAATTATATTAATGTAGGTTATGATAGA
ATTATGTATTGATCCTTAAGAGTTAAGAGAGATACGCCCACGTATAATACATATTGGTTTAGGGT
TGAGTATATATACTTAAGAGTTAAGAATATATATATACAATATATAATAAATATAGTAATATATTA
ATGGTATGGACTTAACCAGGTTTATATATTATCATTTGATAAACATTAATTATAATTTTTCTATTTA
TTATTATTATTATACTGTGAGATTAATAATTATTAAAATATAATATTACATAGTACGTGATATTTGG
GGATTTTATCTTATTAATTGGCAATTAATGATTCTAATCAAATTTTATTCTCTTTAGTTTAATGGTA
GAACAATGATCTTCTAATTCATTGGTTTTAGTTCGATTCTAAAAAGAGATGAGTAAATAATTTTCT
AGATCAGAAATACTACTTTTAACTACAAAAAGCTTACGCTTTTTAACATTTTTTTGATAAATAACA
ACTATTGTTAATATTTGGCTGTCTATTGGTTTAACTAACAATTACAAAATTTTTTCAATTTTTATAAT
ATAAATTTAAATGAGAATATTAAAAAGTCATTCATTATTAAAATTAGTGAATTCTTACCTTATCGA

TGCGTCACAACCAAGTAACATTAGTTACTTGTGAAATTTTGGTTCATTATTAGCTGTTTGTTTAAT
AGTACAAATTATTACCGGTATTACATTAGCTATGCATTATAGTCCTAGTGTAATGGAAGCTTTTAA
CTCAATAGAGCATATAATGAGAGATGTTAATAACGGGTGATTAGTTCGTTATCTACATAGTAATA
CAGCTTCTGCTTTCTTTTTCTTAGTGTATTTACACATAGGAAGAGGTATATATTACGGATCATATA
GAGCTCCTCGTACTTTAGTTTGAGCTATTGGTACTGTTATATTAATATTAATGATGGCTATCGGTT
TCCTAGGTTATGTTTTACCTTATGGACAGATGTCATTATGAGGTGCTACAGTTATTACTAATCTTA
TTAGTGCTATACCTTGAATAGGGCAAGATATTGTTGAATTCATTTGAGGTGGTTTTTCTGTTAATA
ATGCCACTTTAAACAGATTTTTTGCATTACATTTTGTATTGCCTTTTGTATTAGCTGCTTTAGTTTTA
ATGCACTTAATTGCACTTCATGATACTGCTGGTTCAAGCAATCCTCTTGGTGTTTCAGGTAATTAC
GATAGAATTACATTTGCTCCATATTTTTTATTTAAAGATTTAATTACTATTTTTATATTTATTTTTGTA
TTAAGTGCTTTTGTATTCTTTATGCCTAATGTTTTAGGGGATAGTGATAATTATATTATGGCTAATC
CTATGCAAACTCCTGCTGCTATTGTACCTGAATGATACTTATTACCTTTCTATGCTATTTTAAGATC
TATACCTAATAAATTATTAGGTGTTATAGCGATGTTTAGTGCTATTTTAGCTATTATGTTATTACCT
GTTACAGATTTAGGTAGATCTAGAGGTTTACAATTTAGACCATTTAGTAAAATAGCTTTCTGAGTT
TTTGTTGCTAATTTCTTAGTTTTAATGCAATTAGGTGCTAAACACGTTGAAGATCCATTTATATTAT
TAGGTCAATTAAGTACTGTATTATACTTTAGTTATTTTGTTGCTATATTACCTTTAGCTAGTTACTT
AGATAATAGTTTAACTGATTTATCTAATAAATCTGAATTATTTTTAAATAAAACTAACTAAATATAT
TAAGATTATTATTTAATATATTTTCTATTTAAGATACTATTAATTTAGTATTTTGGGTTTTCAGTTTA
TAATTTATATTATATTATGCATTACCCTCCACCTTGCTTTGTAGTAAGCTAATCTGTTATTTCCTTTA
GTTTAATGGTAGAACAATGATCTTCTAATTCATTGGTTTTAGTTCGAATCTAAAAAGGAAATAAG
AAATATATTCTTATTATTACTTATATAATAATTATTTCTTAAAAATATACATTTTGCATTATAGCCGT
TTAGCTGTATTAAAATGTAAAATGATATAAAATAGAATAAATATTTAAATTATTCCTATGTTATATT
ATCCTATATTGCAACCATTATCAGAAGTTGTATTAATACTTGTACCTGCCTTATTAGCTGTAGCTT
ATGTTACAGTTGCTGAAAGAAAAACTATGGCTAGTATGCAAAGAAGATTAGGTCCTAATGCTGT
AGGTTACTATGGACTATTGCAAGCATTTGCTGATGCCTTAAAACTTTTATTAAAAGAATATGTAG
CTCCTACACAATCTAATATTGTTCTTTTCTTTTTAGGTCCTGTAATAACTTTAATTTTTGCATTATTA
GGTTACGCTGTTATACCCTATGGTCCTGGTTCAGGGATAAGCGACATGAATTTAGGTATATTTTA
CATGTTAGCTGTGTCATCTTTAGCTACATACGGTATTCTATTAGCTGGTTGAAGTGCGAATAGTA
AATACGCTTTTCTAGGTTCTCTTAGAAGTACAGCTCAATTAATTAGTTATGAATTAATATTAAGTT
CAGCTATATTAATAGTAATTATGATAACAGGAAATTTAAATTTAACTGTTTGTACTGAATCTCAAA
GAGCTATTTGATTTATACTACCTTTATTTCCTGTGTTTATAATATTTTTCATAGGATCTATAGCTGA
GACAAATAGAGCTCCTTTTGATTTAGCCGAGGCTAACCTGCTAATCTGGTTTGGTCTGGTTATAT
GTCACAAATTGCTAGGAAACCTTTTTATTTTAAAAACAAAAGACAATTAGCAGGAAACTTAATTT
AACCTAATTAAAAATATTAGATAATTAAACTCTTCATAGACTAAACGTGACAATTTAATATATATA
TATATTTATTTATATATATGATTAAATAAGATATAGTCAATCATCGGTGTGAATCGACTTAAAAAA
AAAAGCACATGGGTTAAACCCATCTCCCCTTATTAGGGGAATCAGAACTTGTTAGTGGGTTCAT
GACAGAGCATGCTGCCGTAGTTTTCGTATTCTTCTTTTTAGCTGAGTACGGTAGTATTGTACTAAT
GTGTATTTTAACTAGTATATTATTTATTGGTGGTTACTTATTATTTGAAATATCCTATGTTTTTACTG
TGGTAAATTATATTTTCTTTGAATTATTCTTTATAGACTGAGTAACATTTGTAGAGGTACAATCTTT
ATACACTGATTTTTTAAATAATTCTATCATTGAAGGATTATTATATGGGTTTAATCTAGGATTAAA
AAGTTCTTTAATGATATTCACATTTATTTGAGCTAGAGCATCCTTCCCTAGAATACGATTTGATCA
ACTAATGGGCTTCTGTTGAACAGTTTTATTACCTATTAATTTTGCAATTATTATATTAGTACCTTGT
GTTTTATATAGTTTTAACTTATTACCTGTAAATATACCATTGTTCTAGCTCACACACCCGCCCGCC
CTACTGCCACAAGGCTACAGTACATATGAGGAGGGGAACTAAAGATCTAGAACTATCCTAGTTA

ATAATTACACTTAATAGTATACTAAATAGACCATCTATCATACTCGAGAATAGTGATAGTGTAAT
TATACACTATTTTAACTGTATTCTACACTATTAGCATATTATTATCTTTATATGATAATAATTTTATA
ACTTAATTTATTAGGTTACATTAACAAAAAATTCGTTAACTTTTTTCACCACTTTTTAATACAAAAT
ACGAATTTTA
>YN634
ATAACAATTCTAAAGAACATAAAGAGTTATTAGATAAAAATAATTCACCTATACAGTTAATAAAT
CAACTTAAAGGGTATTTTTTCATAAATCCTTTATTAGCTTTAAGTTTAGCTATTACTATTTTCTCTTT
TGCAGGTATTCCTCCTCTTGTAGGGTTCTTTGCTAAACAGATGGTATTAAGCGCGGCTATTGATC
AAGGTTATATCTTTTTATCTTTAGTTGCAATATTAACTAGTGTTATAGGAGGGGTTTATTATTTAAA
TATAATTAAAGAAATGTTCTTTTATTCACCTGACTATAAATTAAACGAAGAAATTAAAAATAATAC
TATTAATGGTCAAATTATTAATAGAAACAATAAAATATTAAATGTTGAATTTAATTATACAAATGT
AGTTATGTCTAGTTCTGTGGCAATAACTATTTCTACTATTACATTAGTAGTTTTATTATTCATGTTT
ATGAATAAAGAATGATTAAGTCTGGGTACTATATTGGTACAATCTTTATTTAGCTATTAATGAGTA
GTATGACATTATTTATAGGGTTTGTATCTGTTATAGCTATTTTATTTTTAGCCATTAATTTTATATTT
GCTCCTCATAATCCTTATCAAGAAAAATATAGTATTTTCGAGTGTGGTTTCCATAGTTTTTTAGGG
CAAAATAGAACACAATTCGGTATAAAATTCTTTATTTTTGCTTTAGTTTATTTACTTTTAGATTTAG
AAATATTATTAACTTTCCCTTTCGCTCTTAGTGAGTATGTTAATGGTATTTATGGTCTTTTAGTTAC
TTTAATTTTTATAGCTATAATAACTATAGGATTTATATTTGAATTAGGTAAAAGCGCTCTTAAAATA
GACAGCAGACAAAAATTATATATACCTAAATTGAACGTTAATTACCATACAGAGTATGTTGGAAT
AGGTAAGGTTTCTAAGTAAAGTTATAGAGGCAGAAAACCAAAAAACCTACCAAAGGGTAGCTA
ATGGGAAGCTATTAAAAATAAAAGATGATAACCTATATATAGTATAGTTACTATATATTTACTATA
ACTAGGATTATTATATATATATATATTATCTTATTGTATATTAAGATTATTATTATTATAAGGTATA
ATTAATATAGTATCTTATTGTATAAGAATATAATATATTAACCTATAATTAATTTTATTTTTTAATTT
TTAATTATAATTTTTTTTTATATCTAGATGCTTACACATCTACAGATGTAGAAGAGAACAAAATAT
TGTTGTTATAGTAGTGGATGATATAGAAAAATATTTTTATATTATTTATTTTTGTTAGGTAGCTTTT
GAAGTGTTTGATAGAGAGGATATATGGACGGTAGGAGGGTATTCATTTTAATGAACAGTGGATA
GTTTAAATTAACCTAGTTATAGTTTATGAATTTAAATTATAATTATATTAATGTAGGTTATGATAGA
ATTATGTATTGATCCTTAAGAGTTAAGAGAGATACGCCCACGTATAATACATATTGGTTTAGGGT
TGAGTATATATACTTAAGAGTTAAGAATATATATATACAATATATAATAAATATAGTAATATATTA
ATGGTATGGACTTAACCAGGTTTATATATTATCATTTGATAAACATTAATTATAATTTTTCTATTTA
TTATTATTATTATACTGTGAGATTAATAATTATTAAAATATAATATTACATAGTACGTGATATTTGG
GGATTTTATCTTATTAATTGGCAATTAATGATTCTAATCAAATTTTATTCTCTTTAGTTTAATGGTA
GAACAATGATCTTCTAATTCATTGGTTTTAGTTCGATTCTAAAAAGAGATGAGTAAATAATTTTCT
AGATCAGAAATACTACTTTTAACTACAAAAAGCTTACGCTTTTTAACATTTTTTTGATAAATAACA
ACTATTGTTAATATTTGGCTGTCTATTGGTTTAACTAACAATTACAAAATTTTTTCAATTTTTATAAT
ATAAATTTAAATGAGAATATTAAAAAGTCATTCATTATTAAAATTAGTGAATTCTTACCTTATCGA
TGCGTCACAACCAAGTAACATTAGTTACTTGTGAAATTTTGGTTCATTATTAGCTGTTTGTTTAAT
AGTACAAATTATTACCGGTATTACATTAGCTATGCATTATAGTCCTAGTGTAATGGAAGCTTTTAA
CTCAATAGAGCATATAATGAGAGATGTTAATAACGGGTGATTAGTTCGTTATCTACATAGTAATA
CAGCTTCTGCTTTCTTTTTCTTAGTGTATTTACACATAGGAAGAGGTATATATTACGGATCATATA
GAGCTCCTCGTACTTTAGTTTGAGCTATTGGTACTGTTATATTAATATTAATGATGGCTATCGGTT
TCCTAGGTTATGTTTTACCTTATGGACAGATGTCATTATGAGGTGCTACAGTTATTACTAATCTTA
TTAGTGCTATACCTTGAATAGGGCAAGATATTGTTGAATTCATTTGAGGTGGTTTTTCTGTTAATA
ATGCCACTTTAAACAGATTTTTTGCATTACATTTTGTATTGCCTTTTGTATTAGCTGCTTTAGTTTTA

ATGCACTTAATTGCACTTCATGATACTGCTGGTTCAAGCAATCCTCTTGGTGTTTCAGGTAATTAC
GATAGAATTACATTTGCTCCATATTTTTTATTTAAAGATTTAATTACTATTTTTATATTTATTTTTGTA
TTAAGTGCTTTTGTATTCTTTATGCCTAATGTTTTAGGGGATAGTGATAATTATATTATGGCTAATC
CTATGCAAACTCCTGCTGCTATTGTACCTGAATGATACTTATTACCTTTCTATGCTATTTTAAGATC
TATACCTAATAAATTATTAGGTGTTATAGCGATGTTTAGTGCTATTTTAGCTATTATGTTATTACCT
GTTACAGATTTAGGTAGATCTAGAGGTTTACAATTTAGACCATTTAGTAAAATAGCTTTCTGAGTT
TTTGTTGCTAATTTCTTAGTTTTAATGCAATTAGGTGCTAAACACGTTGAAGATCCATTTATATTAT
TAGGTCAATTAAGTACTGTATTATACTTTAGTTATTTTGTTGCTATATTACCTTTAGCTAGTTACTT
AGATAATAGTTTAACTGATTTATCTAATAAATCTGAATTATTTTTAAATAAAACTAACTAAATATAT
TAAGATTATTATTTAATATATTTTCTATTTAAGATACTATTAATTTAGTATTTTGGGTTTTCAGTTTA
TAATTTATATTATATTATGCATTACCCTCCACCTTGCTTTGTAGTAAGCTAATCTGTTATTTCCTTTA
GTTTAATGGTAGAACAATGATCTTCTAATTCATTGGTTTTAGTTCGAATCTAAAAAGGAAATAAG
AAATATATTCTTATTATTACTTATATAATAATTATTTCTTAAAAATATACATTTTGCATTATAGCCGT
TTAGCTGTATTAAAATGTAAAATGATATAAAATAGAATAAATATTTAAATTATTCCTATGTTATATT
ATCCTATATTGCAACCATTATCAGAAGTTGTATTAATACTTGTACCTGCCTTATTAGCTGTAGCTT
ATGTTACAGTTGCTGAAAGAAAAACTATGGCTAGTATGCAAAGAAGATTAGGTCCTAATGCTGT
AGGTTACTATGGACTATTGCAAGCATTTGCTGATGCCTTAAAACTTTTATTAAAAGAATATGTAG
CTCCTACACAATCTAATATTGTTCTTTTCTTTTTAGGTCCTGTAATAACTTTAATTTTTGCATTATTA
GGTTACGCTGTTATACCCTATGGTCCTGGTTCAGGGATAAGCGACATGAATTTAGGTATATTTTA
CATGTTAGCTGTGTCATCTTTAGCTACATACGGTATTCTATTAGCTGGTTGAAGTGCGAATAGTA
AATACGCTTTTCTAGGTTCTCTTAGAAGTACAGCTCAATTAATTAGTTATGAATTAATATTAAGTT
CAGCTATATTAATAGTAATTATGATAACAGGAAATTTAAATTTAACTGTTTGTACTGAATCTCAAA
GAGCTATTTGATTTATACTACCTTTATTTCCTGTGTTTATAATATTTTTCATAGGATCTATAGCTGA
GACAAATAGAGCTCCTTTTGATTTAGCCGAGGCTAACCTGCTAATCTGGTTTGGTCTGGTTATAT
GTCACAAATTGCTAGGAAACCTTTTTATTTTAAAAACAAAAGACAATTAGCAGGAAACTTAATTT
AACCTAATTAAAAATATTAGATAATTAAACTCTTCATAGACTAAACGTGACAATTTAATATATATA
TATATTTATTTATATATATGATTAAATAAGATATAGTCAATCATCGGTGTGAATCGACTTAAAAAA
AAAAGCACATGGGTTAAACCCATCTCCCCTTATTAGGGGAATCAGAACTTGTTAGTGGGTTCAT
GACAGAGCATGCTGCCGTAGTTTTCGTATTCTTCTTTTTAGCTGAGTACGGTAGTATTGTACTAAT
GTGTATTTTAACTAGTATATTATTTATTGGTGGTTACTTATTATTTGAAATATCCTATGTTTTTACTG
TGGTAAATTATATTTTCTTTGAATTATTCTTTATAGACTGAGTAACATTTGTAGAGGTACAATCTTT
ATACACTGATTTTTTAAATAATTCTATCATTGAAGGATTATTATATGGGTTTAATCTAGGATTAAA
AAGTTCTTTAATGATATTCACATTTATTTGAGCTAGAGCATCCTTCCCTAGAATACGATTTGATCA
ACTAATGGGCTTCTGTTGAACAGTTTTATTACCTATTAATTTTGCAATTATTATATTAGTACCTTGT
GTTTTATATAGTTTTAACTTATTACCTGTAAATATACCATTGTTCTAGCTCACACACCCGCCCGCC
CTACTGCCACAAGGCTACAGTACATATGAGGAGGGGAACTAAAGATCTAGAACTATCCTAGTTA
ATAATTACACTTAATAGTATACTAAATAGACCATCTATCATACTCGAGAATAGTGATAGTGTAAT
TATACACTATTTTAACTGTATTCTACACTATTAGCATATTATTATCTTTATATGATAATAATTTTATA
ACTTAATTTATTAGGTTACATTAACAAAAAATTCGTTAACTTTTTTCACCACTTTTTAATACAAAAT
ACGAATTTTA
>YN635
ATAACAATTCTAAAGAACATAAAGAGTTATTAGATAAAAATAATTCACCTATACAGTTAATAAAT
CAACTTAAAGGGTATTTTTTCATAAATCCTTTATTAGCTTTAAGTTTAGCTATTACTATTTTCTCTTT
TGCAGGTATTCCTCCTCTTGTAGGGTTCTTTGCTAAACAGATGGTATTAAGCGCGGCTATTGATC

AAGGTTATATCTTTTTATCTTTAGTTGCAATATTAACTAGTGTTATAGGAGGGGTTTATTATTTAAA
TATAATTAAAGAAATGTTCTTTTATTCACCTGACTATAAATTAAACGAAGAAATTAAAAATAATAC
TATTAATGGTCAAATTATTAATAGAAACAATAAAATATTAAATGTTGAATTTAATTATACAAATGT
AGTTATGTCTAGTTCTGTGGCAATAACTATTTCTACTATTACATTAGTAGTTTTATTATTCATGTTT
ATGAATAAAGAATGATTAAGTCTGGGTACTATATTGGTACAATCTTTATTTAGCTATTAATGAGTA
GTATGACATTATTTATAGGGTTTGTATCTGTTATAGCTATTTTATTTTTAGCCATTAATTTTATATTT
GCTCCTCATAATCCTTATCAAGAAAAATATAGTATTTTCGAGTGTGGTTTCCATAGTTTTTTAGGG
CAAAATAGAACACAATTCGGTATAAAATTCTTTATTTTTGCTTTAGTTTATTTACTTTTAGATTTAG
AAATATTATTAACTTTCCCTTTCGCTCTTAGTGAGTATGTTAATGGTATTTATGGTCTTTTAGTTAC
TTTAATTTTTATAGCTATAATAACTATAGGATTTATATTTGAATTAGGTAAAAGCGCTCTTAAAATA
GACAGCAGACAAAAATTATATATACCTAAATTGAACGTTAATTACCATACAGAGTATGTTGGAAT
AGGTAAGGTTTCTAAGTAAAGTTATAGAGGCAGAAAACCAAAAAACCTACCAAAGGGTAGCTA
ATGGGAAGCTATTAAAAATAAAAGATGATAACCTATATATAGTATAGTTACTATATATTTACTATA
ACTAGGATTATTATATATATATATATTATCTTATTGTATATTAAGATTATTATTATTATAAGGTATA
ATTAATATAGTATCTTATTGTATAAGAATATAATATATTAACCTATAATTAATTTTATTTTTTAATTT
TTAATTATAATTTTTTTTTATATCTAGATGCTTACACATCTACAGATGTAGAAGAGAACAAAATAT
TGTTGTTATAGTAGTGGATGATATAGAAAAATATTTTTATATTATTTATTTTTGTTAGGTAGCTTTT
GAAGTGTTTGATAGAGAGGATATATGGACGGTAGGAGGGTATTCATTTTAATGAACAGTGGATA
GTTTAAATTAACCTAGTTATAGTTTATGAATTTAAATTATAATTATATTAATGTAGGTTATGATAGA
ATTATGTATTGATCCTTAAGAGTTAAGAGAGATACGCCCACGTATAATACATATTGGTTTAGGGT
TGAGTATATATACTTAAGAGTTAAGAATATATATATACAATATATAATAAATATAGTAATATATTA
ATGGTATGGACTTAACCAGGTTTATATATTATCATTTGATAAACATTAATTATAATTTTTCTATTTA
TTATTATTATTATACTGTGAGATTAATAATTATTAAAATATAATATTACATAGTACGTGATATTTGG
GGATTTTATCTTATTAATTGGCAATTAATGATTCTAATCAAATTTTATTCTCTTTAGTTTAATGGTA
GAACAATGATCTTCTAATTCATTGGTTTTAGTTCGATTCTAAAAAGAGATGAGTAAATAATTTTCT
AGATCAGAAATACTACTTTTAACTACAAAAAGCTTACGCTTTTTAACATTTTTTTGATAAATAACA
ACTATTGTTAATATTTGGCTGTCTATTGGTTTAACTAACAATTACAAAATTTTTTCAATTTTTATAAT
ATAAATTTAAATGAGAATATTAAAAAGTCATTCATTATTAAAATTAGTGAATTCTTACCTTATCGA
TGCGTCACAACCAAGTAACATTAGTTACTTGTGAAATTTTGGTTCATTATTAGCTGTTTGTTTAAT
AGTACAAATTATTACCGGTATTACATTAGCTATGCATTATAGTCCTAGTGTAATGGAAGCTTTTAA
CTCAATAGAGCATATAATGAGAGATGTTAATAACGGGTGATTAGTTCGTTATCTACATAGTAATA
CAGCTTCTGCTTTCTTTTTCTTAGTGTATTTACACATAGGAAGAGGTATATATTACGGATCATATA
GAGCTCCTCGTACTTTAGTTTGAGCTATTGGTACTGTTATATTAATATTAATGATGGCTATCGGTT
TCCTAGGTTATGTTTTACCTTATGGACAGATGTCATTATGAGGTGCTACAGTTATTACTAATCTTA
TTAGTGCTATACCTTGAATAGGGCAAGATATTGTTGAATTCATTTGAGGTGGTTTTTCTGTTAATA
ATGCCACTTTAAACAGATTTTTTGCATTACATTTTGTATTGCCTTTTGTATTAGCTGCTTTAGTTTTA
ATGCACTTAATTGCACTTCATGATACTGCTGGTTCAAGCAATCCTCTTGGTGTTTCAGGTAATTAC
GATAGAATTACATTTGCTCCATATTTTTTATTTAAAGATTTAATTACTATTTTTATATTTATTTTTGTA
TTAAGTGCTTTTGTATTCTTTATGCCTAATGTTTTAGGGGATAGTGATAATTATATTATGGCTAATC
CTATGCAAACTCCTGCTGCTATTGTACCTGAATGATACTTATTACCTTTCTATGCTATTTTAAGATC
TATACCTAATAAATTATTAGGTGTTATAGCGATGTTTAGTGCTATTTTAGCTATTATGTTATTACCT
GTTACAGATTTAGGTAGATCTAGAGGTTTACAATTTAGACCATTTAGTAAAATAGCTTTCTGAGTT
TTTGTTGCTAATTTCTTAGTTTTAATGCAATTAGGTGCTAAACACGTTGAAGATCCATTTATATTAT
TAGGTCAATTAAGTACTGTATTATACTTTAGTTATTTTGTTGCTATATTACCTTTAGCTAGTTACTT

AGATAATAGTTTAACTGATTTATCTAATAAATCTGAATTATTTTTAAATAAAACTAACTAAATATAT
TAAGATTATTATTTAATATATTTTCTATTTAAGATACTATTAATTTAGTATTTTGGGTTTTCAGTTTA
TAATTTATATTATATTATGCATTACCCTCCACCTTGCTTTGTAGTAAGCTAATCTGTTATTTCCTTTA
GTTTAATGGTAGAACAATGATCTTCTAATTCATTGGTTTTAGTTCGAATCTAAAAAGGAAATAAG
AAATATATTCTTATTATTACTTATATAATAATTATTTCTTAAAAATATACATTTTGCATTATAGCCGT
TTAGCTGTATTAAAATGTAAAATGATATAAAATAGAATAAATATTTAAATTATTCCTATGTTATATT
ATCCTATATTGCAACCATTATCAGAAGTTGTATTAATACTTGTACCTGCCTTATTAGCTGTAGCTT
ATGTTACAGTTGCTGAAAGAAAAACTATGGCTAGTATGCAAAGAAGATTAGGTCCTAATGCTGT
AGGTTACTATGGACTATTGCAAGCATTTGCTGATGCCTTAAAACTTTTATTAAAAGAATATGTAG
CTCCTACACAATCTAATATTGTTCTTTTCTTTTTAGGTCCTGTAATAACTTTAATTTTTGCATTATTA
GGTTACGCTGTTATACCCTATGGTCCTGGTTCAGGGATAAGCGACATGAATTTAGGTATATTTTA
CATGTTAGCTGTGTCATCTTTAGCTACATACGGTATTCTATTAGCTGGTTGAAGTGCGAATAGTA
AATACGCTTTTCTAGGTTCTCTTAGAAGTACAGCTCAATTAATTAGTTATGAATTAATATTAAGTT
CAGCTATATTAATAGTAATTATGATAACAGGAAATTTAAATTTAACTGTTTGTACTGAATCTCAAA
GAGCTATTTGATTTATACTACCTTTATTTCCTGTGTTTATAATATTTTTCATAGGATCTATAGCTGA
GACAAATAGAGCTCCTTTTGATTTAGCCGAGGCTAACCTGCTAATCTGGTTTGGTCTGGTTATAT
GTCACAAATTGCTAGGAAACCTTTTTATTTTAAAAACAAAAGACAATTAGCAGGAAACTTAATTT
AACCTAATTAAAAATATTAGATAATTAAACTCTTCATAGACTAAACGTGACAATTTAATATATATA
TATATTTATTTATATATATGATTAAATAAGATATAGTCAATCATCGGTGTGAATCGACTTAAAAAA
AAAAGCACATGGGTTAAACCCATCTCCCCTTATTAGGGGAATCAGAACTTGTTAGTGGGTTCAT
GACAGAGCATGCTGCCGTAGTTTTCGTATTCTTCTTTTTAGCTGAGTACGGTAGTATTGTACTAAT
GTGTATTTTAACTAGTATATTATTTATTGGTGGTTACTTATTATTTGAAATATCCTATGTTTTTACTG
TGGTAAATTATATTTTCTTTGAATTATTCTTTATAGACTGAGTAACATTTGTAGAGGTACAATCTTT
ATACACTGATTTTTTAAATAATTCTATCATTGAAGGATTATTATATGGGTTTAATCTAGGATTAAA
AAGTTCTTTAATGATATTCACATTTATTTGAGCTAGAGCATCCTTCCCTAGAATACGATTTGATCA
ACTAATGGGCTTCTGTTGAACAGTTTTATTACCTATTAATTTTGCAATTATTATATTAGTACCTTGT
GTTTTATATAGTTTTAACTTATTACCTGTAAATATACCATTGTTCTAGCTCACACACCCGCCCGCC
CTACTGCCACAAGGCTACAGTACATATGAGGAGGGGAACTAAAGATCTAGAACTATCCTAGTTA
ATAATTACACTTAATAGTATACTAAATAGACCATCTATCATACTCGAGAATAGTGATAGTGTAAT
TATACACTATTTTAACTGTATTCTACACTATTAGCATATTATTATCTTTATATGATAATAATTTTATA
ACTTAATTTATTAGGTTACATTAACAAAAAATTCGTTAACTTTTTTCACCACTTTTTAATACAAAAT
ACGAATTTTA
>YN640
ATAACAATTCTAAAGAACATAAAGAGTTATTAGATAAAAATAATTCACCTATACAGTTAATAAAT
CAACTTAAAGGGTATTTTTTCATAAATCCTTTATTAGCTTTAAGTTTAGCTATTACTATTTTCTCTTT
TGCAGGTATTCCTCCTCTTGTAGGGTTCTTTGCTAAACAGATGGTATTAAGCGCGGCTATTGATC
AAGGTTATATCTTTTTATCTTTAGTTGCAATATTAACTAGTGTTATAGGAGGGGTTTATTATTTAAA
TATAATTAAAGAAATGTTCTTTTATTCACCTGACTATAAATTAAACGAAGAAATTAAAAATAATAC
TATTAATGGTCAAATTATTAATAGAAACAATAAAATATTAAATGTTGAATTTAATTATACAAATGT
AGTTATGTCTAGTTCTGTGGCAATAACTATTTCTACTATTACATTAGTAGTTTTATTATTCATGTTT
ATGAATAAAGAATGATTAAGTCTGGGTACTATATTGGTACAATCTTTATTTAGCTATTAATGAGTA
GTATGACATTATTTATAGGGTTTGTATCTGTTATAGCTATTTTATTTTTAGCCATTAATTTTATATTT
GCTCCTCATAATCCTTATCAAGAAAAATATAGTATTTTCGAGTGTGGTTTCCATAGTTTTTTAGGG
CAAAATAGAACACAATTCGGTATAAAATTCTTTATTTTTGCTTTAGTTTATTTACTTTTAGATTTAG

AAATATTATTAACTTTCCCTTTCGCTCTTAGTGAGTATGTTAATGGTATTTATGGTCTTTTAGTTAC
TTTAATTTTTATAGCTATAATAACTATAGGATTTATATTTGAATTAGGTAAAAGCGCTCTTAAAATA
GACAGCAGACAAAAATTATATATACCTAAATTGAACGTTAATTACCATACAGAGTATGTTGGAAT
AGGTAAGGTTTCTAAGTAAAGTTATAGAGGCAGAAAACCAAAAAACCTACCAAAGGGTAGCTA
ATGGGAAGCTATTAAAAATAAAAGATGATAACCTATATATAGTATAGTTACTATATATTTACTATA
ACTAGGATTATTATATATATATATATTATCTTATTGTATATTAAGATTATTATTATTATAAGGTATA
ATTAATATAGTATCTTATTGTATAAGAATATAATATATTAACCTATAATTAATTTTATTTTTTAATTT
TTAATTATAATTTTTTTTTATATCTAGATGCTTACACATCTACAGATGTAGAAGAGAACAAAATAT
TGTTGTTATAGTAGTGGATGATATAGAAAAATATTTTTATATTATTTATTTTTGTTAGGTAGCTTTT
GAAGTGTTTGATAGAGAGGATATATGGACGGTAGGAGGGTATTCATTTTAATGAACAGTGGATA
GTTTAAATTAACCTAGTTATAGTTTATGAATTTAAATTATAATTATATTAATGTAGGTTATGATAGA
ATTATGTATTGATCCTTAAGAGTTAAGAGAGATACGCCCACGTATAATACATATTGGTTTAGGGT
TGAGTATATATACTTAAGAGTTAAGAATATATATATACAATATATAATAAATATAGTAATATATTA
ATGGTATGGACTTAACCAGGTTTATATATTATCATTTGATAAACATTAATTATAATTTTTCTATTTA
TTATTATTATTATACTGTGAGATTAATAATTATTAAAATATAATATTACATAGTACGTGATATTTGG
GGATTTTATCTTATTAATTGGCAATTAATGATTCTAATCAAATTTTATTCTCTTTAGTTTAATGGTA
GAACAATGATCTTCTAATTCATTGGTTTTAGTTCGATTCTAAAAAGAGATGAGTAAATAATTTTCT
AGATCAGAAATACTACTTTTAACTACAAAAAGCTTACGCTTTTTAACATTTTTTTGATAAATAACA
ACTATTGTTAATATTTGGCTGTCTATTGGTTTAACTAACAATTACAAAATTTTTTCAATTTTTATAAT
ATAAATTTAAATGAGAATATTAAAAAGTCATTCATTATTAAAATTAGTGAATTCTTACCTTATCGA
TGCGTCACAACCAAGTAACATTAGTTACTTGTGAAATTTTGGTTCATTATTAGCTGTTTGTTTAAT
AGTACAAATTATTACCGGTATTACATTAGCTATGCATTATAGTCCTAGTGTAATGGAAGCTTTTAA
CTCAATAGAGCATATAATGAGAGATGTTAATAACGGGTGATTAGTTCGTTATCTACATAGTAATA
CAGCTTCTGCTTTCTTTTTCTTAGTGTATTTACACATAGGAAGAGGTATATATTACGGATCATATA
GAGCTCCTCGTACTTTAGTTTGAGCTATTGGTACTGTTATATTAATATTAATGATGGCTATCGGTT
TCCTAGGTTATGTTTTACCTTATGGACAGATGTCATTATGAGGTGCTACAGTTATTACTAATCTTA
TTAGTGCTATACCTTGAATAGGGCAAGATATTGTTGAATTCATTTGAGGTGGTTTTTCTGTTAATA
ATGCCACTTTAAACAGATTTTTTGCATTACATTTTGTATTGCCTTTTGTATTAGCTGCTTTAGTTTTA
ATGCACTTAATTGCACTTCATGATACTGCTGGTTCAAGCAATCCTCTTGGTGTTTCAGGTAATTAC
GATAGAATTACATTTGCTCCATATTTTTTATTTAAAGATTTAATTACTATTTTTATATTTATTTTTGTA
TTAAGTGCTTTTGTATTCTTTATGCCTAATGTTTTAGGGGATAGTGATAATTATATTATGGCTAATC
CTATGCAAACTCCTGCTGCTATTGTACCTGAATGATACTTATTACCTTTCTATGCTATTTTAAGATC
TATACCTAATAAATTATTAGGTGTTATAGCGATGTTTAGTGCTATTTTAGCTATTATGTTATTACCT
GTTACAGATTTAGGTAGATCTAGAGGTTTACAATTTAGACCATTTAGTAAAATAGCTTTCTGAGTT
TTTGTTGCTAATTTCTTAGTTTTAATGCAATTAGGTGCTAAACACGTTGAAGATCCATTTATATTAT
TAGGTCAATTAAGTACTGTATTATACTTTAGTTATTTTGTTGCTATATTACCTTTAGCTAGTTACTT
AGATAATAGTTTAACTGATTTATCTAATAAATCTGAATTATTTTTAAATAAAACTAACTAAATATAT
TAAGATTATTATTTAATATATTTTCTATTTAAGATACTATTAATTTAGTATTTTGGGTTTTCAGTTTA
TAATTTATATTATATTATGCATTACCCTCCACCTTGCTTTGTAGTAAGCTAATCTGTTATTTCCTTTA
GTTTAATGGTAGAACAATGATCTTCTAATTCATTGGTTTTAGTTCGAATCTAAAAAGGAAATAAG
AAATATATTCTTATTATTACTTATATAATAATTATTTCTTAAAAATATACATTTTGCATTATAGCCGT
TTAGCTGTATTAAAATGTAAAATGATATAAAATAGAATAAATATTTAAATTATTCCTATGTTATATT
ATCCTATATTGCAACCATTATCAGAAGTTGTATTAATACTTGTACCTGCCTTATTAGCTGTAGCTT
ATGTTACAGTTGCTGAAAGAAAAACTATGGCTAGTATGCAAAGAAGATTAGGTCCTAATGCTGT

AGGTTACTATGGACTATTGCAAGCATTTGCTGATGCCTTAAAACTTTTATTAAAAGAATATGTAG
CTCCTACACAATCTAATATTGTTCTTTTCTTTTTAGGTCCTGTAATAACTTTAATTTTTGCATTATTA
GGTTACGCTGTTATACCCTATGGTCCTGGTTCAGGGATAAGCGACATGAATTTAGGTATATTTTA
CATGTTAGCTGTGTCATCTTTAGCTACATACGGTATTCTATTAGCTGGTTGAAGTGCGAATAGTA
AATACGCTTTTCTAGGTTCTCTTAGAAGTACAGCTCAATTAATTAGTTATGAATTAATATTAAGTT
CAGCTATATTAATAGTAATTATGATAACAGGAAATTTAAATTTAACTGTTTGTACTGAATCTCAAA
GAGCTATTTGATTTATACTACCTTTATTTCCTGTGTTTATAATATTTTTCATAGGATCTATAGCTGA
GACAAATAGAGCTCCTTTTGATTTAGCCGAGGCTAACCTGCTAATCTGGTTTGGTCTGGTTATAT
GTCACAAATTGCTAGGAAACCTTTTTATTTTAAAAACAAAAGACAATTAGCAGGAAACTTAATTT
AACCTAATTAAAAATATTAGATAATTAAACTCTTCATAGACTAAACGTGACAATTTAATATATATA
TATATTTATTTATATATATGATTAAATAAGATATAGTCAATCATCGGTGTGAATCGACTTAAAAAA
AAAAGCACATGGGTTAAACCCATCTCCCCTTATTAGGGGAATCAGAACTTGTTAGTGGGTTCAT
GACAGAGCATGCTGCCGTAGTTTTCGTATTCTTCTTTTTAGCTGAGTACGGTAGTATTGTACTAAT
GTGTATTTTAACTAGTATATTATTTATTGGTGGTTACTTATTATTTGAAATATCCTATGTTTTTACTG
TGGTAAATTATATTTTCTTTGAATTATTCTTTATAGACTGAGTAACATTTGTAGAGGTACAATCTTT
ATACACTGATTTTTTAAATAATTCTATCATTGAAGGATTATTATATGGGTTTAATCTAGGATTAAA
AAGTTCTTTAATGATATTCACATTTATTTGAGCTAGAGCATCCTTCCCTAGAATACGATTTGATCA
ACTAATGGGCTTCTGTTGAACAGTTTTATTACCTATTAATTTTGCAATTATTATATTAGTACCTTGT
GTTTTATATAGTTTTAACTTATTACCTGTAAATATACCATTGTTCTAGCTCACACACCCGCCCGCC
CTACTGCCACAAGGCTACAGTACATATGAGGAGGGGAACTAAAGATCTAGAACTATCCTAGTTA
ATAATTACACTTAATAGTATACTAAATAGACCATCTATCATACTCGAGAATAGTGATAGTGTAAT
TATACACTATTTTAACTGTATTCTACACTATTAGCATATTATTATCTTTATATGATAATAATTTTATA
ACTTAATTTATTAGGTTACATTAACAAAAAATTCGTTAACTTTTTTCACCACTTTTTAATACAAAAT
ACGAATTTTA
>YN643
ATAACAATTCTAAAGAACATAAAGAGTTATTAGATAAAAATAATTCACCTATACAGTTAATAAAT
CAACTTAAAGGGTATTTTTTCATAAATCCTTTATTAGCTTTAAGTTTAGCTATTACTATTTTCTCTTT
TGCAGGTATTCCTCCTCTTGTAGGGTTCTTTGCTAAACAGATGGTATTAAGCGCGGCTATTGATC
AAGGTTATATCTTTTTATCTTTAGTTGCAATATTAACTAGTGTTATAGGAGGGGTTTATTATTTAAA
TATAATTAAAGAAATGTTCTTTTATTCACCTGACTATAAATTAAACGAAGAAATTAAAAATAATAC
TATTAATGGTCAAATTATTAATAGAAACAATAAAATATTAAATGTTGAATTTAATTATACAAATGT
AGTTATGTCTAGTTCTGTGGCAATAACTATTTCTACTATTACATTAGTAGTTTTATTATTCATGTTT
ATGAATAAAGAATGATTAAGTCTGGGTACTATATTGGTACAATCTTTATTTAGCTATTAATGAGTA
GTATGACATTATTTATAGGGTTTGTATCTGTTATAGCTATTTTATTTTTAGCCATTAATTTTATATTT
GCTCCTCATAATCCTTATCAAGAAAAATATAGTATTTTCGAGTGTGGTTTCCATAGTTTTTTAGGG
CAAAATAGAACACAATTCGGTATAAAATTCTTTATTTTTGCTTTAGTTTATTTACTTTTAGATTTAG
AAATATTATTAACTTTCCCTTTCGCTCTTAGTGAGTATGTTAATGGTATTTATGGTCTTTTAGTTAC
TTTAATTTTTATAGCTATAATAACTATAGGATTTATATTTGAATTAGGTAAAAGCGCTCTTAAAATA
GACAGCAGACAAAAATTATATATACCTAAATTGAACGTTAATTACCATACAGAGTATGTTGGAAT
AGGTAAGGTTTCTAAGTAAAGTTATAGAGGCAGAAAACCAAAAAACCTACCAAAGGGTAGCTA
ATGGGAAGCTATTAAAAATAAAAGATGATAACCTATATATAGTATAGTTACTATATATTTACTATA
ACTAGGATTATTATATATATATATATTATCTTATTGTATATTAAGATTATTATTATTATAAGGTATA
ATTAATATAGTATCTTATTGTATAAGAATATAATATATTAACCTATAATTAATTTTATTTTTTAATTT
TTAATTATAATTTTTTTTTATATCTAGATGCTTACACATCTACAGATGTAGAAGAGAACAAAATAT

TGTTGTTATAGTAGTGGATGATATAGAAAAATATTTTTATATTATTTATTTTTGTTAGGTAGCTTTT
GAAGTGTTTGATAGAGAGGATATATGGACGGTAGGAGGGTATTCATTTTAATGAACAGTGGATA
GTTTAAATTAACCTAGTTATAGTTTATGAATTTAAATTATAATTATATTAATGTAGGTTATGATAGA
ATTATGTATTGATCCTTAAGAGTTAAGAGAGATACGCCCACGTATAATACATATTGGTTTAGGGT
TGAGTATATATACTTAAGAGTTAAGAATATATATATACAATATATAATAAATATAGTAATATATTA
ATGGTATGGACTTAACCAGGTTTATATATTATCATTTGATAAACATTAATTATAATTTTTCTATTTA
TTATTATTATTATACTGTGAGATTAATAATTATTAAAATATAATATTACATAGTACGTGATATTTGG
GGATTTTATCTTATTAATTGGCAATTAATGATTCTAATCAAATTTTATTCTCTTTAGTTTAATGGTA
GAACAATGATCTTCTAATTCATTGGTTTTAGTTCGATTCTAAAAAGAGATGAGTAAATAATTTTCT
AGATCAGAAATACTACTTTTAACTACAAAAAGCTTACGCTTTTTAACATTTTTTTGATAAATAACA
ACTATTGTTAATATTTGGCTGTCTATTGGTTTAACTAACAATTACAAAATTTTTTCAATTTTTATAAT
ATAAATTTAAATGAGAATATTAAAAAGTCATTCATTATTAAAATTAGTGAATTCTTACCTTATCGA
TGCGTCACAACCAAGTAACATTAGTTACTTGTGAAATTTTGGTTCATTATTAGCTGTTTGTTTAAT
AGTACAAATTATTACCGGTATTACATTAGCTATGCATTATAGTCCTAGTGTAATGGAAGCTTTTAA
CTCAATAGAGCATATAATGAGAGATGTTAATAACGGGTGATTAGTTCGTTATCTACATAGTAATA
CAGCTTCTGCTTTCTTTTTCTTAGTGTATTTACACATAGGAAGAGGTATATATTACGGATCATATA
GAGCTCCTCGTACTTTAGTTTGAGCTATTGGTACTGTTATATTAATATTAATGATGGCTATCGGTT
TCCTAGGTTATGTTTTACCTTATGGACAGATGTCATTATGAGGTGCTACAGTTATTACTAATCTTA
TTAGTGCTATACCTTGAATAGGGCAAGATATTGTTGAATTCATTTGAGGTGGTTTTTCTGTTAATA
ATGCCACTTTAAACAGATTTTTTGCATTACATTTTGTATTGCCTTTTGTATTAGCTGCTTTAGTTTTA
ATGCACTTAATTGCACTTCATGATACTGCTGGTTCAAGCAATCCTCTTGGTGTTTCAGGTAATTAC
GATAGAATTACATTTGCTCCATATTTTTTATTTAAAGATTTAATTACTATTTTTATATTTATTTTTGTA
TTAAGTGCTTTTGTATTCTTTATGCCTAATGTTTTAGGGGATAGTGATAATTATATTATGGCTAATC
CTATGCAAACTCCTGCTGCTATTGTACCTGAATGATACTTATTACCTTTCTATGCTATTTTAAGATC
TATACCTAATAAATTATTAGGTGTTATAGCGATGTTTAGTGCTATTTTAGCTATTATGTTATTACCT
GTTACAGATTTAGGTAGATCTAGAGGTTTACAATTTAGACCATTTAGTAAAATAGCTTTCTGAGTT
TTTGTTGCTAATTTCTTAGTTTTAATGCAATTAGGTGCTAAACACGTTGAAGATCCATTTATATTAT
TAGGTCAATTAAGTACTGTATTATACTTTAGTTATTTTGTTGCTATATTACCTTTAGCTAGTTACTT
AGATAATAGTTTAACTGATTTATCTAATAAATCTGAATTATTTTTAAATAAAACTAACTAAATATAT
TAAGATTATTATTTAATATATTTTCTATTTAAGATACTATTAATTTAGTATTTTGGGTTTTCAGTTTA
TAATTTATATTATATTATGCATTACCCTCCACCTTGCTTTGTAGTAAGCTAATCTGTTATTTCCTTTA
GTTTAATGGTAGAACAATGATCTTCTAATTCATTGGTTTTAGTTCGAATCTAAAAAGGAAATAAG
AAATATATTCTTATTATTACTTATATAATAATTATTTCTTAAAAATATACATTTTGCATTATAGCCGT
TTAGCTGTATTAAAATGTAAAATGATATAAAATAGAATAAATATTTAAATTATTCCTATGTTATATT
ATCCTATATTGCAACCATTATCAGAAGTTGTATTAATACTTGTACCTGCCTTATTAGCTGTAGCTT
ATGTTACAGTTGCTGAAAGAAAAACTATGGCTAGTATGCAAAGAAGATTAGGTCCTAATGCTGT
AGGTTACTATGGACTATTGCAAGCATTTGCTGATGCCTTAAAACTTTTATTAAAAGAATATGTAG
CTCCTACACAATCTAATATTGTTCTTTTCTTTTTAGGTCCTGTAATAACTTTAATTTTTGCATTATTA
GGTTACGCTGTTATACCCTATGGTCCTGGTTCAGGGATAAGCGACATGAATTTAGGTATATTTTA
CATGTTAGCTGTGTCATCTTTAGCTACATACGGTATTCTATTAGCTGGTTGAAGTGCGAATAGTA
AATACGCTTTTCTAGGTTCTCTTAGAAGTACAGCTCAATTAATTAGTTATGAATTAATATTAAGTT
CAGCTATATTAATAGTAATTATGATAACAGGAAATTTAAATTTAACTGTTTGTACTGAATCTCAAA
GAGCTATTTGATTTATACTACCTTTATTTCCTGTGTTTATAATATTTTTCATAGGATCTATAGCTGA
GACAAATAGAGCTCCTTTTGATTTAGCCGAGGCTAACCTGCTAATCTGGTTTGGTCTGGTTATAT

GTCACAAATTGCTAGGAAACCTTTTTATTTTAAAAACAAAAGACAATTAGCAGGAAACTTAATTT
AACCTAATTAAAAATATTAGATAATTAAACTCTTCATAGACTAAACGTGACAATTTAATATATATA
TATATTTATTTATATATATGATTAAATAAGATATAGTCAATCATCGGTGTGAATCGACTTAAAAAA
AAAAGCACATGGGTTAAACCCATCTCCCCTTATTAGGGGAATCAGAACTTGTTAGTGGGTTCAT
GACAGAGCATGCTGCCGTAGTTTTCGTATTCTTCTTTTTAGCTGAGTACGGTAGTATTGTACTAAT
GTGTATTTTAACTAGTATATTATTTATTGGTGGTTACTTATTATTTGAAATATCCTATGTTTTTACTG
TGGTAAATTATATTTTCTTTGAATTATTCTTTATAGACTGAGTAACATTTGTAGAGGTACAATCTTT
ATACACTGATTTTTTAAATAATTCTATCATTGAAGGATTATTATATGGGTTTAATCTAGGATTAAA
AAGTTCTTTAATGATATTCACATTTATTTGAGCTAGAGCATCCTTCCCTAGAATACGATTTGATCA
ACTAATGGGCTTCTGTTGAACAGTTTTATTACCTATTAATTTTGCAATTATTATATTAGTACCTTGT
GTTTTATATAGTTTTAACTTATTACCTGTAAATATACCATTGTTCTAGCTCACACACCCGCCCGCC
CTACTGCCACAAGGCTACAGTACATATGAGGAGGGGAACTAAAGATCTAGAACTATCCTAGTTA
ATAATTACACTTAATAGTATACTAAATAGACCATCTATCATACTCGAGAATAGTGATAGTGTAAT
TATACACTATTTTAACTGTATTCTACACTATTAGCATATTATTATCTTTATATGATAATAATTTTATA
ACTTAATTTATTAGGTTACATTAACAAAAAAATTCGTTAACTTTTTTCACCACTTTTTAATACAAAAT
ACGAATTTTA
>YN651
ATAACAATTCTAAAGAACATAAAGAGTTATTAGATAAAAATAATTCACCTATACAGTTAATAAAT
CAACTTAAAGGGTATTTTTTCATAAATCCTTTATTAGCTTTAAGTTTAGCTATTACTATTTTCTCTTT
TGCAGGTATTCCTCCTCTTGTAGGGTTCTTTGCTAAACAGATGGTATTAAGCGCGGCTATTGATC
AAGGTTATATCTTTTTATCTTTAGTTGCAATATTAACTAGTGTTATAGGAGGGGTTTATTATTTAAA
TATAATTAAAGAAATGTTCTTTTATTCACCTGACTATAAATTAAACGAAGAAATTAAAAATAATAC
TATTAATGGTCAAATTATTAATAGAAACAATAAAATATTAAATGTTGAATTTAATTATACAAATGT
AGTTATGTCTAGTTCTGTGGCAATAACTATTTCTACTATTACATTAGTAGTTTTATTATTCATGTTT
ATGAATAAAGAATGATTAAGTCTGGGTACTATATTGGTACAATCTTTATTTAGCTATTAATGAGTA
GTATGACATTATTTATAGGGTTTGTATCTGTTATAGCTATTTTATTTTTAGCCATTAATTTTATATTT
GCTCCTCATAATCCTTATCAAGAAAAATATAGTATTTTCGAGTGTGGTTTCCATAGTTTTTTAGGG
CAAAATAGAACACAATTCGGTATAAAATTCTTTATTTTTGCTTTAGTTTATTTACTTTTAGATTTAG
AAATATTATTAACTTTCCCTTTCGCTCTTAGTGAGTATGTTAATGGTATTTATGGTCTTTTAGTTAC
TTTAATTTTTATAGCTATAATAACTATAGGATTTATATTTGAATTAGGTAAAAGCGCTCTTAAAATA
GACAGCAGACAAAAATTATATATACCTAAATTGAACGTTAATTACCATACAGAGTATGTTGGAAT
AGGTAAGGTTTCTAAGTAAAGTTATAGAGGCAGAAAACCAAAAAACCTACCAAAGGGTAGCTA
ATGGGAAGCTATTAAAAATAAAAGATGATAACCTATATATAGTATAGTTACTATATATTTACTATA
ACTAGGATTATTATATATATATATATTATCTTATTGTATATTAAGATTATTATTATTATAAGGTATA
ATTAATATAGTATCTTATTGTATAAGAATATAATATATTAACCTATAATTAATTTTATTTTTTAATTT
TTAATTATAATTTTTTTTTATATCTAGATGCTTACACATCTACAGATGTAGAAGAGAACAAAATAT
TGTTGTTATAGTAGTGGATGATATAGAAAAATATTTTTATATTATTTATTTTTGTTAGGTAGCTTTT
GAAGTGTTTGATAGAGAGGATATATGGACGGTAGGAGGGTATTCATTTTAATGAACAGTGGATA
GTTTAAATTAACCTAGTTATAGTTTATGAATTTAAATTATAATTATATTAATGTAGGTTATGATAGA
ATTATGTATTGATCCTTAAGAGTTAAGAGAGATACGCCCACGTATAATACATATTGGTTTAGGGT
TGAGTATATATACTTAAGAGTTAAGAATATATATATACAATATATAATAAATATAGTAATATATTA
ATGGTATGGACTTAACCAGGTTTATATATTATCATTTGATAAACATTAATTATAATTTTTCTATTTA
TTATTATTATTATACTGTGAGATTAATAATTATTAAAATATAATATTACATAGTACGTGATATTTGG
GGATTTTATCTTATTAATTGGCAATTAATGATTCTAATCAAATTTTATTCTCTTTAGTTTAATGGTA

GAACAATGATCTTCTAATTCATTGGTTTTAGTTCGATTCTAAAAAGAGATGAGTAAATAATTTTCT
AGATCAGAAATACTACTTTTAACTACAAAAAGCTTACGCTTTTTAACATTTTTTTGATAAATAACA
ACTATTGTTAATATTTGGCTGTCTATTGGTTTAACTAACAATTACAAAATTTTTTCAATTTTTATAAT
ATAAATTTAAATGAGAATATTAAAAAGTCATTCATTATTAAAATTAGTGAATTCTTACCTTATCGA
TGCGTCACAACCAAGTAACATTAGTTACTTGTGAAATTTTGGTTCATTATTAGCTGTTTGTTTAAT
AGTACAAATTATTACCGGTATTACATTAGCTATGCATTATAGTCCTAGTGTAATGGAAGCTTTTAA
CTCAATAGAGCATATAATGAGAGATGTTAATAACGGGTGATTAGTTCGTTATCTACATAGTAATA
CAGCTTCTGCTTTCTTTTTCTTAGTGTATTTACACATAGGAAGAGGTATATATTACGGATCATATA
GAGCTCCTCGTACTTTAGTTTGAGCTATTGGTACTGTTATATTAATATTAATGATGGCTATCGGTT
TCCTAGGTTATGTTTTACCTTATGGACAGATGTCATTATGAGGTGCTACAGTTATTACTAATCTTA
TTAGTGCTATACCTTGAATAGGGCAAGATATTGTTGAATTCATTTGAGGTGGTTTTTCTGTTAATA
ATGCCACTTTAAACAGATTTTTTGCATTACATTTTGTATTGCCTTTTGTATTAGCTGCTTTAGTTTTA
ATGCACTTAATTGCACTTCATGATACTGCTGGTTCAAGCAATCCTCTTGGTGTTTCAGGTAATTAC
GATAGAATTACATTTGCTCCATATTTTTTATTTAAAGATTTAATTACTATTTTTATATTTATTTTTGTA
TTAAGTGCTTTTGTATTCTTTATGCCTAATGTTTTAGGGGATAGTGATAATTATATTATGGCTAATC
CTATGCAAACTCCTGCTGCTATTGTACCTGAATGATACTTATTACCTTTCTATGCTATTTTAAGATC
TATACCTAATAAATTATTAGGTGTTATAGCGATGTTTAGTGCTATTTTAGCTATTATGTTATTACCT
GTTACAGATTTAGGTAGATCTAGAGGTTTACAATTTAGACCATTTAGTAAAATAGCTTTCTGAGTT
TTTGTTGCTAATTTCTTAGTTTTAATGCAATTAGGTGCTAAACACGTTGAAGATCCATTTATATTAT
TAGGTCAATTAAGTACTGTATTATACTTTAGTTATTTTGTTGCTATATTACCTTTAGCTAGTTACTT
AGATAATAGTTTAACTGATTTATCTAATAAATCTGAATTATTTTTAAATAAAACTAACTAAATATAT
TAAGATTATTATTTAATATATTTTCTATTTAAGATACTATTAATTTAGTATTTTGGGTTTTCAGTTTA
TAATTTATATTATATTATGCATTACCCTCCACCTTGCTTTGTAGTAAGCTAATCTGTTATTTCCTTTA
GTTTAATGGTAGAACAATGATCTTCTAATTCATTGGTTTTAGTTCGAATCTAAAAAGGAAATAAG
AAATATATTCTTATTATTACTTATATAATAATTATTTCTTAAAAATATACATTTTGCATTATAGCCGT
TTAGCTGTATTAAAATGTAAAATGATATAAAATAGAATAAATATTTAAATTATTCCTATGTTATATT
ATCCTATATTGCAACCATTATCAGAAGTTGTATTAATACTTGTACCTGCCTTATTAGCTGTAGCTT
ATGTTACAGTTGCTGAAAGAAAAACTATGGCTAGTATGCAAAGAAGATTAGGTCCTAATGCTGT
AGGTTACTATGGACTATTGCAAGCATTTGCTGATGCCTTAAAACTTTTATTAAAAGAATATGTAG
CTCCTACACAATCTAATATTGTTCTTTTCTTTTTAGGTCCTGTAATAACTTTAATTTTTGCATTATTA
GGTTACGCTGTTATACCCTATGGTCCTGGTTCAGGGATAAGCGACATGAATTTAGGTATATTTTA
CATGTTAGCTGTGTCATCTTTAGCTACATACGGTATTCTATTAGCTGGTTGAAGTGCGAATAGTA
AATACGCTTTTCTAGGTTCTCTTAGAAGTACAGCTCAATTAATTAGTTATGAATTAATATTAAGTT
CAGCTATATTAATAGTAATTATGATAACAGGAAATTTAAATTTAACTGTTTGTACTGAATCTCAAA
GAGCTATTTGATTTATACTACCTTTATTTCCTGTGTTTATAATATTTTTCATAGGATCTATAGCTGA
GACAAATAGAGCTCCTTTTGATTTAGCCGAGGCTAACCTGCTAATCTGGTTTGGTCTGGTTATAT
GTCACAAATTGCTAGGAAACCTTTTTATTTTAAAAACAAAAGACAATTAGCAGGAAACTTAATTT
AACCTAATTAAAAATATTAGATAATTAAACTCTTCATAGACTAAACGTGACAATTTAATATATATA
TATATTTATTTATATATATGATTAAATAAGATATAGTCAATCATCGGTGTGAATCGACTTAAAAAA
AAAAGCACATGGGTTAAACCCATCTCCCCTTATTAGGGGAATCAGAACTTGTTAGTGGGTTCAT
GACAGAGCATGCTGCCGTAGTTTTCGTATTCTTCTTTTTAGCTGAGTACGGTAGTATTGTACTAAT
GTGTATTTTAACTAGTATATTATTTATTGGTGGTTACTTATTATTTGAAATATCCTATGTTTTTACTG
TGGTAAATTATATTTTCTTTGAATTATTCTTTATAGACTGAGTAACATTTGTAGAGGTACAATCTTT
ATACACTGATTTTTTAAATAATTCTATCATTGAAGGATTATTATATGGGTTTAATCTAGGATTAAA

AAGTTCTTTAATGATATTCACATTTATTTGAGCTAGAGCATCCTTCCCTAGAATACGATTTGATCA
ACTAATGGGCTTCTGTTGAACAGTTTTATTACCTATTAATTTTGCAATTATTATATTAGTACCTTGT
GTTTTATATAGTTTTAACTTATTACCTGTAAATATACCATTGTTCTAGCTCACACACCCGCCCGCC
CTACTGCCACAAGGCTACAGTACATATGAGGAGGGGAACTAAAGATCTAGAACTATCCTAGTTA
ATAATTACACTTAATAGTATACTAAATAGACCATCTATCATACTCGAGAATAGTGATAGTGTAAT
TATACACTATTTTAACTGTATTCTACACTATTAGCATATTATTATCTTTATATGATAATAATTTTATA
ACTTAATTTATTAGGTTACATTAACAAAAAAATTCGTTAACTTTTTTCACCACTTTTTAATACAAAAT
ACGAATTTTA
>YN657
ATAACAATTCTAAAGAACATAAAGAGTTATTAGATAAAAATAATTCACCTATACAGTTAATAAAT
CAACTTAAAGGGTATTTTTTCATAAATCCTTTATTAGCTTTAAGTTTAGCTATTACTATTTTCTCTTT
TGCAGGTATTCCTCCTCTTGTAGGGTTCTTTGCTAAACAGATGGTATTAAGCGCGGCTATTGATC
AAGGTTATATCTTTTTATCTTTAGTTGCAATATTAACTAGTGTTATAGGAGGGGTTTATTATTTAAA
TATAATTAAAGAAATGTTCTTTTATTCACCTGACTATAAATTAAACGAAGAAATTAAAAATAATAC
TATTAATGGTCAAATTATTAATAGAAACAATAAAATATTAAATGTTGAATTTAATTATACAAATGT
AGTTATGTCTAGTTCTGTGGCAATAACTATTTCTACTATTACATTAGTAGTTTTATTATTCATGTTT
ATGAATAAAGAATGATTAAGTCTGGGTACTATATTGGTACAATCTTTATTTAGCTATTAATGAGTA
GTATGACATTATTTATAGGGTTTGTATCTGTTATAGCTATTTTATTTTTAGCCATTAATTTTATATTT
GCTCCTCATAATCCTTATCAAGAAAAATATAGTATTTTCGAGTGTGGTTTCCATAGTTTTTTAGGG
CAAAATAGAACACAATTCGGTATAAAATTCTTTATTTTTGCTTTAGTTTATTTACTTTTAGATTTAG
AAATATTATTAACTTTCCCTTTCGCTCTTAGTGAGTATGTTAATGGTATTTATGGTCTTTTAGTTAC
TTTAATTTTTATAGCTATAATAACTATAGGATTTATATTTGAATTAGGTAAAAGCGCTCTTAAAATA
GACAGCAGACAAAAATTATATATACCTAAATTGAACGTTAATTACCATACAGAGTATGTTGGAAT
AGGTAAGGTTTCTAAGTAAAGTTATAGAGGCAGAAAACCAAAAAACCTACCAAAGGGTAGCTA
ATGGGAAGCTATTAAAAATAAAAGATGATAACCTATATATAGTATAGTTACTATATATTTACTATA
ACTAGGATTATTATATATATATATATTATCTTATTGTATATTAAGATTATTATTATTATAAGGTATA
ATTAATATAGTATCTTATTGTATAAGAATATAATATATTAACCTATAATTAATTTTATTTTTTAATTT
TTAATTATAATTTTTTTTTATATCTAGATGCTTACACATCTACAGATGTAGAAGAGAACAAAATAT
TGTTGTTATAGTAGTGGATGATATAGAAAAATATTTTTATATTATTTATTTTTGTTAGGTAGCTTTT
GAAGTGTTTGATAGAGAGGATATATGGACGGTAGGAGGGTATTCATTTTAATGAACAGTGGATA
GTTTAAATTAACCTAGTTATAGTTTATGAATTTAAATTATAATTATATTAATGTAGGTTATGATAGA
ATTATGTATTGATCCTTAAGAGTTAAGAGAGATACGCCCACGTATAATACATATTGGTTTAGGGT
TGAGTATATATACTTAAGAGTTAAGAATATATATATACAATATATAATAAATATAGTAATATATTA
ATGGTATGGACTTAACCAGGTTTATATATTATCATTTGATAAACATTAATTATAATTTTTCTATTTA
TTATTATTATTATACTGTGAGATTAATAATTATTAAAATATAATATTACATAGTACGTGATATTTGG
GGATTTTATCTTATTAATTGGCAATTAATGATTCTAATCAAATTTTATTCTCTTTAGTTTAATGGTA
GAACAATGATCTTCTAATTCATTGGTTTTAGTTCGATTCTAAAAAGAGATGAGTAAATAATTTTCT
AGATCAGAAATACTACTTTTAACTACAAAAAGCTTACGCTTTTTAACATTTTTTTGATAAATAACA
ACTATTGTTAATATTTGGCTGTCTATTGGTTTAACTAACAATTACAAAATTTTTTCAATTTTTATAAT
ATAAATTTAAATGAGAATATTAAAAAGTCATTCATTATTAAAATTAGTGAATTCTTACCTTATCGA
TGCGTCACAACCAAGTAACATTAGTTACTTGTGAAATTTTGGTTCATTATTAGCTGTTTGTTTAAT
AGTACAAATTATTACCGGTATTACATTAGCTATGCATTATAGTCCTAGTGTAATGGAAGCTTTTAA
CTCAATAGAGCATATAATGAGAGATGTTAATAACGGGTGATTAGTTCGTTATCTACATAGTAATA
CAGCTTCTGCTTTCTTTTTCTTAGTGTATTTACACATAGGAAGAGGTATATATTACGGATCATATA

GAGCTCCTCGTACTTTAGTTTGAGCTATTGGTACTGTTATATTAATATTAATGATGGCTATCGGTT
TCCTAGGTTATGTTTTACCTTATGGACAGATGTCATTATGAGGTGCTACAGTTATTACTAATCTTA
TTAGTGCTATACCTTGAATAGGGCAAGATATTGTTGAATTCATTTGAGGTGGTTTTTCTGTTAATA
ATGCCACTTTAAACAGATTTTTTGCATTACATTTTGTATTGCCTTTTGTATTAGCTGCTTTAGTTTTA
ATGCACTTAATTGCACTTCATGATACTGCTGGTTCAAGCAATCCTCTTGGTGTTTCAGGTAATTAC
GATAGAATTACATTTGCTCCATATTTTTTATTTAAAGATTTAATTACTATTTTTATATTTATTTTTGTA
TTAAGTGCTTTTGTATTCTTTATGCCTAATGTTTTAGGGGATAGTGATAATTATATTATGGCTAATC
CTATGCAAACTCCTGCTGCTATTGTACCTGAATGATACTTATTACCTTTCTATGCTATTTTAAGATC
TATACCTAATAAATTATTAGGTGTTATAGCGATGTTTAGTGCTATTTTAGCTATTATGTTATTACCT
GTTACAGATTTAGGTAGATCTAGAGGTTTACAATTTAGACCATTTAGTAAAATAGCTTTCTGAGTT
TTTGTTGCTAATTTCTTAGTTTTAATGCAATTAGGTGCTAAACACGTTGAAGATCCATTTATATTAT
TAGGTCAATTAAGTACTGTATTATACTTTAGTTATTTTGTTGCTATATTACCTTTAGCTAGTTACTT
AGATAATAGTTTAACTGATTTATCTAATAAATCTGAATTATTTTTAAATAAAACTAACTAAATATAT
TAAGATTATTATTTAATATATTTTCTATTTAAGATACTATTAATTTAGTATTTTGGGTTTTCAGTTTA
TAATTTATATTATATTATGCATTACCCTCCACCTTGCTTTGTAGTAAGCTAATCTGTTATTTCCTTTA
GTTTAATGGTAGAACAATGATCTTCTAATTCATTGGTTTTAGTTCGAATCTAAAAAGGAAATAAG
AAATATATTCTTATTATTACTTATATAATAATTATTTCTTAAAAATATACATTTTGCATTATAGCCGT
TTAGCTGTATTAAAATGTAAAATGATATAAAATAGAATAAATATTTAAATTATTCCTATGTTATATT
ATCCTATATTGCAACCATTATCAGAAGTTGTATTAATACTTGTACCTGCCTTATTAGCTGTAGCTT
ATGTTACAGTTGCTGAAAGAAAAACTATGGCTAGTATGCAAAGAAGATTAGGTCCTAATGCTGT
AGGTTACTATGGACTATTGCAAGCATTTGCTGATGCCTTAAAACTTTTATTAAAAGAATATGTAG
CTCCTACACAATCTAATATTGTTCTTTTCTTTTTAGGTCCTGTAATAACTTTAATTTTTGCATTATTA
GGTTACGCTGTTATACCCTATGGTCCTGGTTCAGGGATAAGCGACATGAATTTAGGTATATTTTA
CATGTTAGCTGTGTCATCTTTAGCTACATACGGTATTCTATTAGCTGGTTGAAGTGCGAATAGTA
AATACGCTTTTCTAGGTTCTCTTAGAAGTACAGCTCAATTAATTAGTTATGAATTAATATTAAGTT
CAGCTATATTAATAGTAATTATGATAACAGGAAATTTAAATTTAACTGTTTGTACTGAATCTCAAA
GAGCTATTTGATTTATACTACCTTTATTTCCTGTGTTTATAATATTTTTCATAGGATCTATAGCTGA
GACAAATAGAGCTCCTTTTGATTTAGCCGAGGCTAACCTGCTAATCTGGTTTGGTCTGGTTATAT
GTCACAAATTGCTAGGAAACCTTTTTATTTTAAAAACAAAAGACAATTAGCAGGAAACTTAATTT
AACCTAATTAAAAATATTAGATAATTAAACTCTTCATAGACTAAACGTGACAATTTAATATATATA
TATATTTATTTATATATATGATTAAATAAGATATAGTCAATCATCGGTGTGAATCGACTTAAAAAA
AAAAGCACATGGGTTAAACCCATCTCCCCTTATTAGGGGAATCAGAACTTGTTAGTGGGTTCAT
GACAGAGCATGCTGCCGTAGTTTTCGTATTCTTCTTTTTAGCTGAGTACGGTAGTATTGTACTAAT
GTGTATTTTAACTAGTATATTATTTATTGGTGGTTACTTATTATTTGAAATATCCTATGTTTTTACTG
TGGTAAATTATATTTTCTTTGAATTATTCTTTATAGACTGAGTAACATTTGTAGAGGTACAATCTTT
ATACACTGATTTTTTAAATAATTCTATCATTGAAGGATTATTATATGGGTTTAATCTAGGATTAAA
AAGTTCTTTAATGATATTCACATTTATTTGAGCTAGAGCATCCTTCCCTAGAATACGATTTGATCA
ACTAATGGGCTTCTGTTGAACAGTTTTATTACCTATTAATTTTGCAATTATTATATTAGTACCTTGT
GTTTTATATAGTTTTAACTTATTACCTGTAAATATACCATTGTTCTAGCTCACACACCCGCCCGCC
CTACTGCCACAAGGCTACAGTACATATGAGGAGGGGAACTAAAGATCTAGAACTATCCTAGTTA
ATAATTACACTTAATAGTATACTAAATAGACCATCTATCATACTCGAGAATAGTGATAGTGTAAT
TATACACTATTTTAACTGTATTCTACACTATTAGCATATTATTATCTTTATATGATAATAATTTTATA
ACTTAATTTATTAGGTTACATTAACAAAAAATTCGTTAACTTTTTTCACCACTTTTTAATACAAAAT
ACGAATATTA

>YN661
ATAACAATTCTAAAGAACATAAAGAGTTATTAGATAAAAATAATTCACCTATACAGTTAATAAAT
CAACTTAAAGGGTATTTTTTCATAAATCCTTTATTAGCTTTAAGTTTAGCTATTACTATTTTCTCTTT
TGCAGGTATTCCTCCTCTTGTAGGGTTCTTTGCTAAACAGATGGTATTAAGCGCGGCTATTGATC
AAGGTTATATCTTTTTATCTTTAGTTGCAATATTAACTAGTGTTATAGGAGGGGTTTATTATTTAAA
TATAATTAAAGAAATGTTCTTTTATTCACCTGACTATAAATTAAACGAAGAAATTAAAAATAATAC
TATTAATGGTCAAATTATTAATAGAAACAATAAAATATTAAATGTTGAATTTAATTATACAAATGT
AGTTATGTCTAGTTCTGTGGCAATAACTATTTCTACTATTACATTAGTAGTTTTATTATTCATGTTT
ATGAATAAAGAATGATTAAGTCTGGGTACTATATTGGTACAATCTTTATTTAGCTATTAATGAGTA
GTATGACATTATTTATAGGGTTTGTATCTGTTATAGCTATTTTATTTTTAGCCATTAATTTTATATTT
GCTCCTCATAATCCTTATCAAGAAAAATATAGTATTTTCGAGTGTGGTTTCCATAGTTTTTTAGGG
CAAAATAGAACACAATTCGGTATAAAATTCTTTATTTTTGCTTTAGTTTATTTACTTTTAGATTTAG
AAATATTATTAACTTTCCCTTTCGCTCTTAGTGAGTATGTTAATGGTATTTATGGTCTTTTAGTTAC
TTTAATTTTTATAGCTATAATAACTATAGGATTTATATTTGAATTAGGTAAAAGCGCTCTTAAAATA
GACAGCAGACAAAAATTATATATACCTAAATTGAACGTTAATTACCATACAGAGTATGTTGGAAT
AGGTAAGGTTTCTAAGTAAAGTTATAGAGGCAGAAAACCAAAAAACCTACCAAAGGGTAGCTA
ATGGGAAGCTATTAAAAATAAAAGATGATAACCTATATATAGTATAGTTACTATATATTTACTATA
ACTAGGATTATTATATATATATATATTATCTTATTGTATATTAAGATTATTATTATTATAAGGTATA
ATTAATATAGTATCTTATTGTATAAGAATATAATATATTAACCTATAATTAATTTTATTTTTTAATTT
TTAATTATAATTTTTTTTTATATCTAGATGCTTACACATCTACAGATGTAGAAGAGAACAAAATAT
TGTTGTTATAGTAGTGGATGATATAGAAAAATATTTTTATATTATTTATTTTTGTTAGGTAGCTTTT
GAAGTGTTTGATAGAGAGGATATATGGACGGTAGGAGGGTATTCATTTTAATGAACAGTGGATA
GTTTAAATTAACCTAGTTATAGTTTATGAATTTAAATTATAATTATATTAATGTAGGTTATGATAGA
ATTATGTATTGATCCTTAAGAGTTAAGAGAGATACGCCCACGTATAATACATATTGGTTTAGGGT
TGAGTATATATACTTAAGAGTTAAGAATATATATATACAATATATAATAAATATAGTAATATATTA
ATGGTATGGACTTAACCAGGTTTATATATTATCATTTGATAAACATTAATTATAATTTTTCTATTTA
TTATTATTATTATACTGTGAGATTAATAATTATTAAAATATAATATTACATAGTACGTGATATTTGG
GGATTTTATCTTATTAATTGGCAATTAATGATTCTAATCAAATTTTATTCTCTTTAGTTTAATGGTA
GAACAATGATCTTCTAATTCATTGGTTTTAGTTCGATTCTAAAAAGAGATGAGTAAATAATTTTCT
AGATCAGAAATACTACTTTTAACTACAAAAAGCTTACGCTTTTTAACATTTTTTTGATAAATAACA
ACTATTGTTAATATTTGGCTGTCTATTGGTTTAACTAACAATTACAAAATTTTTTCAATTTTTATAAT
ATAAATTTAAATGAGAATATTAAAAAGTCATTCATTATTAAAATTAGTGAATTCTTACCTTATCGA
TGCGTCACAACCAAGTAACATTAGTTACTTGTGAAATTTTGGTTCATTATTAGCTGTTTGTTTAAT
AGTACAAATTATTACCGGTATTACATTAGCTATGCATTATAGTCCTAGTGTAATGGAAGCTTTTAA
CTCAATAGAGCATATAATGAGAGATGTTAATAACGGGTGATTAGTTCGTTATCTACATAGTAATA
CAGCTTCTGCTTTCTTTTTCTTAGTGTATTTACACATAGGAAGAGGTATATATTACGGATCATATA
GAGCTCCTCGTACTTTAGTTTGAGCTATTGGTACTGTTATATTAATATTAATGATGGCTATCGGTT
TCCTAGGTTATGTTTTACCTTATGGACAGATGTCATTATGAGGTGCTACAGTTATTACTAATCTTA
TTAGTGCTATACCTTGAATAGGGCAAGATATTGTTGAATTCATTTGAGGTGGTTTTTCTGTTAATA
ATGCCACTTTAAACAGATTTTTTGCATTACATTTTGTATTGCCTTTTGTATTAGCTGCTTTAGTTTTA
ATGCACTTAATTGCACTTCATGATACTGCTGGTTCAAGCAATCCTCTTGGTGTTTCAGGTAATTAC
GATAGAATTACATTTGCTCCATATTTTTTATTTAAAGATTTAATTACTATTTTTATATTTATTTTTGTA
TTAAGTGCTTTTGTATTCTTTATGCCTAATGTTTTAGGGGATAGTGATAATTATATTATGGCTAATC
CTATGCAAACTCCTGCTGCTATTGTACCTGAATGATACTTATTACCTTTCTATGCTATTTTAAGATC

TATACCTAATAAATTATTAGGTGTTATAGCGATGTTTAGTGCTATTTTAGCTATTATGTTATTACCT
GTTACAGATTTAGGTAGATCTAGAGGTTTACAATTTAGACCATTTAGTAAAATAGCTTTCTGAGTT
TTTGTTGCTAATTTCTTAGTTTTAATGCAATTAGGTGCTAAACACGTTGAAGATCCATTTATATTAT
TAGGTCAATTAAGTACTGTATTATACTTTAGTTATTTTGTTGCTATATTACCTTTAGCTAGTTACTT
AGATAATAGTTTAACTGATTTATCTAATAAATCTGAATTATTTTTAAATAAAACTAACTAAATATAT
TAAGATTATTATTTAATATATTTTCTATTTAAGATACTATTAATTTAGTATTTTGGGTTTTCAGTTTA
TAATTTATATTATATTATGCATTACCCTCCACCTTGCTTTGTAGTAAGCTAATCTGTTATTTCCTTTA
GTTTAATGGTAGAACAATGATCTTCTAATTCATTGGTTTTAGTTCGAATCTAAAAAGGAAATAAG
AAATATATTCTTATTATTACTTATATAATAATTATTTCTTAAAAATATACATTTTGCATTATAGCCGT
TTAGCTGTATTAAAATGTAAAATGATATAAAATAGAATAAATATTTAAATTATTCCTATGTTATATT
ATCCTATATTGCAACCATTATCAGAAGTTGTATTAATACTTGTACCTGCCTTATTAGCTGTAGCTT
ATGTTACAGTTGCTGAAAGAAAAACTATGGCTAGTATGCAAAGAAGATTAGGTCCTAATGCTGT
AGGTTACTATGGACTATTGCAAGCATTTGCTGATGCCTTAAAACTTTTATTAAAAGAATATGTAG
CTCCTACACAATCTAATATTGTTCTTTTCTTTTTAGGTCCTGTAATAACTTTAATTTTTGCATTATTA
GGTTACGCTGTTATACCCTATGGTCCTGGTTCAGGGATAAGCGACATGAATTTAGGTATATTTTA
CATGTTAGCTGTGTCATCTTTAGCTACATACGGTATTCTATTAGCTGGTTGAAGTGCGAATAGTA
AATACGCTTTTCTAGGTTCTCTTAGAAGTACAGCTCAATTAATTAGTTATGAATTAATATTAAGTT
CAGCTATATTAATAGTAATTATGATAACAGGAAATTTAAATTTAACTGTTTGTACTGAATCTCAAA
GAGCTATTTGATTTATACTACCTTTATTTCCTGTGTTTATAATATTTTTCATAGGATCTATAGCTGA
GACAAATAGAGCTCCTTTTGATTTAGCCGAGGCTAACCTGCTAATCTGGTTTGGTCTGGTTATAT
GTCACAAATTGCTAGGAAACCTTTTTATTTTAAAAACAAAAGACAATTAGCAGGAAACTTAATTT
AACCTAATTAAAAATATTAGATAATTAAACTCTTCATAGACTAAACGTGACAATTTAATATATATA
TATATTTATTTATATATATGATTAAATAAGATATAGTCAATCATCGGTGTGAATCGACTTAAAAAA
AAAAGCACATGGGTTAAACCCATCTCCCCTTATTAGGGGAATCAGAACTTGTTAGTGGGTTCAT
GACAGAGCATGCTGCCGTAGTTTTCGTATTCTTCTTTTTAGCTGAGTACGGTAGTATTGTACTAAT
GTGTATTTTAACTAGTATATTATTTATTGGTGGTTACTTATTATTTGAAATATCCTATGTTTTTACTG
TGGTAAATTATATTTTCTTTGAATTATTCTTTATAGACTGAGTAACATTTGTAGAGGTACAATCTTT
ATACACTGATTTTTTAAATAATTCTATCATTGAAGGATTATTATATGGGTTTAATCTAGGATTAAA
AAGTTCTTTAATGATATTCACATTTATTTGAGCTAGAGCATCCTTCCCTAGAATACGATTTGATCA
ACTAATGGGCTTCTGTTGAACAGTTTTATTACCTATTAATTTTGCAATTATTATATTAGTACCTTGT
GTTTTATATAGTTTTAACTTATTACCTGTAAATATACCATTGTTCTAGCTCACACACCCGCCCGCC
CTACTGCCACAAGGCTACAGTACATATGAGGAGGGGAACTAAAGATCTAGAACTATCCTAGTTA
ATAATTACACTTAATAGTATACTAAATAGACCATCTATCATACTCGAGAATAGTGATAGTGTAAT
TATACACTATTTTAACTGTATTCTACACTATTAGCATATTATTATCTTTATATGATAATAATTTTATA
ACTTAATTTATTAGGTTACATTAACAAAAAATTCGTTAACTTTTTTCACCACTTTTTAATACAAAAT
ACGAATTTTA
>YN670
ATAACAATTCTAAAGAACATAAAGAGTTATTAGATAAAAATAATTCACCTATACAGTTAATAAAT
CAACTTAAAGGGTATTTTTTCATAAATCCTTTATTAGCTTTAAGTTTAGCTATTACTATTTTCTCTTT
TGCAGGTATTCCTCCTCTTGTAGGGTTCTTTGCTAAACAGATGGTATTAAGCGCGGCTATTGATC
AAGGTTATATCTTTTTATCTTTAGTTGCAATATTAACTAGTGTTATAGGAGGGGTTTATTATTTAAA
TATAATTAAAGAAATGTTCTTTTATTCACCTGACTATAAATTAAACGAAGAAATTAAAAATAATAC
TATTAATGGTCAAATTATTAATAGAAACAATAAAATATTAAATGTTGAATTTAATTATACAAATGT
AGTTATGTCTAGTTCTGTGGCAATAACTATTTCTACTATTACATTAGTAGTTTATTATTCATGTTT

ATGAATAAAGAATGATTAAGTCTGGGTACTATATTGGTACAATCTTTATTTAGCTATTAATGAGTA
GTATGACATTATTTATAGGGTTTGTATCTGTTATAGCTATTTTATTTTTAGCCATTAATTTTATATTT
GCTCCTCATAATCCTTATCAAGAAAAATATAGTATTTTCGAGTGTGGTTTCCATAGTTTTTTAGGG
CAAAATAGAACACAATTCGGTATAAAATTCTTTATTTTTGCTTTAGTTTATTTACTTTTAGATTTAG
AAATATTATTAACTTTCCCTTTCGCTCTTAGTGAGTATGTTAATGGTATTTATGGTCTTTTAGTTAC
TTTAATTTTTATAGCTATAATAACTATAGGATTTATATTTGAATTAGGTAAAAGCGCTCTTAAAATA
GACAGCAGACAAAAATTATATATACCTAAATTGAACGTTAATTACCATACAGAGTATGTTGGAAT
AGGTAAGGTTTCTAAGTAAAGTTATAGAGGCAGAAAACCAAAAAACCTACCAAAGGGTAGCTA
ATGGGAAGCTATTAAAAATAAAAGATGATAACCTATATATAGTATAGTTACTATATATTTACTATA
ACTAGGATTATTATATATATATATATATTATCTTATTGTATATTAAGATTATTATTATTATAAGGTATA
ATTAATATAGTATCTTATTGTATAAGAATATAATATATTAACCTATAATTAATTTTATTTTTTAATTT
TTAATTATAATTTTTTTTTATATCTAGATGCTTACACATCTACAGATGTAGAAGAGAACAAAATAT
TGTTGTTATAGTAGTGGATGATATAGAAAAATATTTTTATATTATTTATTTTTGTTAGGTAGCTTTT
GAAGTGTTTGATAGAGAGGATATATGGACGGTAGGAGGGTATTCATTTTAATGAACAGTGGATA
GTTTAAATTAACCTAGTTATAGTTTATGAATTTAAATTATAATTATATTAATGTAGGTTATGATAGA
ATTATGTATTGATCCTTAAGAGTTAAGAGAGATACGCCCACGTATAATACATATTGGTTTAGGGT
TGAGTATATATACTTAAGAGTTAAGAATATATATATACAATATATAATAAATATAGTAATATATTA
ATGGTATGGACTTAACCAGGTTTATATATTATCATTTGATAAACATTAATTATAATTTTTCTATTTA
TTATTATTATTATACTGTGAGATTAATAATTATTAAAATATAATATTACATAGTACGTGATATTTGG
GGATTTTATCTTATTAATTGGCAATTAATGATTCTAATCAAATTTTATTCTCTTTAGTTTAATGGTA
GAACAATGATCTTCTAATTCATTGGTTTTAGTTCGATTCTAAAAAGAGATGAGTAAATAATTTTCT
AGATCAGAAATACTACTTTTAACTACAAAAAGCTTACGCTTTTTAACATTTTTTTGATAAATAACA
ACTATTGTTAATATTTGGCTGTCTATTGGTTTAACTAACAATTACAAAATTTTTTCAATTTTTATAAT
ATAAATTTAAATGAGAATATTAAAAAGTCATTCATTATTAAAATTAGTGAATTCTTACCTTATCGA
TGCGTCACAACCAAGTAACATTAGTTACTTGTGAAATTTTGGTTCATTATTAGCTGTTTGTTTAAT
AGTACAAATTATTACCGGTATTACATTAGCTATGCATTATAGTCCTAGTGTAATGGAAGCTTTTAA
CTCAATAGAGCATATAATGAGAGATGTTAATAACGGGTGATTAGTTCGTTATCTACATAGTAATA
CAGCTTCTGCTTTCTTTTTCTTAGTGTATTTACACATAGGAAGAGGTATATATTACGGATCATATA
GAGCTCCTCGTACTTTAGTTTGAGCTATTGGTACTGTTATATTAATATTAATGATGGCTATCGGTT
TCCTAGGTTATGTTTTACCTTATGGACAGATGTCATTATGAGGTGCTACAGTTATTACTAATCTTA
TTAGTGCTATACCTTGAATAGGGCAAGATATTGTTGAATTCATTTGAGGTGGTTTTTCTGTTAATA
ATGCCACTTTAAACAGATTTTTTGCATTACATTTTGTATTGCCTTTTGTATTAGCTGCTTTAGTTTTA
ATGCACTTAATTGCACTTCATGATACTGCTGGTTCAAGCAATCCTCTTGGTGTTTCAGGTAATTAC
GATAGAATTACATTTGCTCCATATTTTTTATTTAAAGATTTAATTACTATTTTTATATTTATTTTTGTA
TTAAGTGCTTTTGTATTCTTTATGCCTAATGTTTTAGGGGATAGTGATAATTATATTATGGCTAATC
CTATGCAAACTCCTGCTGCTATTGTACCTGAATGATACTTATTACCTTTCTATGCTATTTTAAGATC
TATACCTAATAAATTATTAGGTGTTATAGCGATGTTTAGTGCTATTTTAGCTATTATGTTATTACCT
GTTACAGATTTAGGTAGATCTAGAGGTTTACAATTTAGACCATTTAGTAAAATAGCTTTCTGAGTT
TTTGTTGCTAATTTCTTAGTTTTAATGCAATTAGGTGCTAAACACGTTGAAGATCCATTTATATTAT
TAGGTCAATTAAGTACTGTATTATACTTTAGTTATTTTGTTGCTATATTACCTTTAGCTAGTTACTT
AGATAATAGTTTAACTGATTTATCTAATAAATCTGAATTATTTTTAAATAAAACTAACTAAATATAT
TAAGATTATTATTTAATATATTTTCTATTTAAGATACTATTAATTTAGTATTTTGGGTTTTCAGTTTA
TAATTTATATTATATTATGCATTACCCTCCACCTTGCTTTGTAGTAAGCTAATCTGTTATTTCCTTTA
GTTTAATGGTAGAACAATGATCTTCTAATTCATTGGTTTTAGTTCGAATCTAAAAAGGAAATAAG

AAATATATTCTTATTATTACTTATATAATAATTATTTCTTAAAAATATACATTTTGCATTATAGCCGT
TTAGCTGTATTAAAATGTAAAATGATATAAAATAGAATAAATATTTAAATTATTCCTATGTTATATT
ATCCTATATTGCAACCATTATCAGAAGTTGTATTAATACTTGTACCTGCCTTATTAGCTGTAGCTT
ATGTTACAGTTGCTGAAAGAAAAACTATGGCTAGTATGCAAAGAAGATTAGGTCCTAATGCTGT
AGGTTACTATGGACTATTGCAAGCATTTGCTGATGCCTTAAAACTTTTATTAAAAGAATATGTAG
CTCCTACACAATCTAATATTGTTCTTTTCTTTTTAGGTCCTGTAATAACTTTAATTTTTGCATTATTA
GGTTACGCTGTTATACCCTATGGTCCTGGTTCAGGGATAAGCGACATGAATTTAGGTATATTTTA
CATGTTAGCTGTGTCATCTTTAGCTACATACGGTATTCTATTAGCTGGTTGAAGTGCGAATAGTA
AATACGCTTTTCTAGGTTCTCTTAGAAGTACAGCTCAATTAATTAGTTATGAATTAATATTAAGTT
CAGCTATATTAATAGTAATTATGATAACAGGAAATTTAAATTTAACTGTTTGTACTGAATCTCAAA
GAGCTATTTGATTTATACTACCTTTATTTCCTGTGTTTATAATATTTTTCATAGGATCTATAGCTGA
GACAAATAGAGCTCCTTTTGATTTAGCCGAGGCTAACCTGCTAATCTGGTTTGGTCTGGTTATAT
GTCACAAATTGCTAGGAAACCTTTTTATTTTAAAAACAAAAGACAATTAGCAGGAAACTTAATTT
AACCTAATTAAAAATATTAGATAATTAAACTCTTCATAGACTAAACGTGACAATTTAATATATATA
TATATTTATTTATATATATGATTAAATAAGATATAGTCAATCATCGGTGTGAATCGACTTAAAAAA
AAAAGCACATGGGTTAAACCCATCTCCCCTTATTAGGGGAATCAGAACTTGTTAGTGGGTTCAT
GACAGAGCATGCTGCCGTAGTTTTCGTATTCTTCTTTTTAGCTGAGTACGGTAGTATTGTACTAAT
GTGTATTTTAACTAGTATATTATTTATTGGTGGTTACTTATTATTTGAAATATCCTATGTTTTTACTG
TGGTAAATTATATTTTCTTTGAATTATTCTTTATAGACTGAGTAACATTTGTAGAGGTACAATCTTT
ATACACTGATTTTTTAAATAATTCTATCATTGAAGGATTATTATATGGGTTTAATCTAGGATTAAA
AAGTTCTTTAATGATATTCACATTTATTTGAGCTAGAGCATCCTTCCCTAGAATACGATTTGATCA
ACTAATGGGCTTCTGTTGAACAGTTTTATTACCTATTAATTTTGCAATTATTATATTAGTACCTTGT
GTTTTATATAGTTTTAACTTATTACCTGTAAATATACCATTGTTCTAGCTCACACACCCGCCCGCC
CTACTGCCACAAGGCTACAGTACATATGAGGAGGGGAACTAAAGATCTAGAACTATCCTAGTTA
ATAATTACACTTAATAGTATACTAAATAGACCATCTATCATACTCGAGAATAGTGATAGTGTAAT
TATACACTATTTTAACTGTATTCTACACTATTAGCATATTATTATCTTTATATGATAATAATTTTATA
ACTTAATTTATTAGGTTACATTAACAAAAAATTCGTTAACTTTTTTCACCACTTTTTAATACAAAAT
ACGAATATTA
>YN673
ATAACAATTCTAAAGAACATAAAGAGTTATTAGATAAAAATAATTCACCTATACAGTTAATAAAT
CAACTTAAAGGGTATTTTTTCATAAATCCTTTATTAGCTTTAAGTTTAGCTATTACTATTTTCTCTTT
TGCAGGTATTCCTCCTCTTGTAGGGTTCTTTGCTAAACAGATGGTATTAAGCGCGGCTATTGATC
AAGGTTATATCTTTTTATCTTTAGTTGCAATATTAACTAGTGTTATAGGAGGGGTTTATTATTTAAA
TATAATTAAAGAAATGTTCTTTTATTCACCTGACTATAAATTAAACGAAGAAATTAAAAATAATAC
TATTAATGGTCAAATTATTAATAGAAACAATAAAATATTAAATGTTGAATTTAATTATACAAATGT
AGTTATGTCTAGTTCTGTGGCAATAACTATTTCTACTATTACATTAGTAGTTTTATTATTCATGTTT
ATGAATAAAGAATGATTAAGTCTGGGTACTATATTGGTACAATCTTTATTTAGCTATTAATGAGTA
GTATGACATTATTTATAGGGTTTGTATCTGTTATAGCTATTTTATTTTTAGCCATTAATTTTATATTT
GCTCCTCATAATCCTTATCAAGAAAAATATAGTATTTTCGAGTGTGGTTTCCATAGTTTTTTAGGG
CAAAATAGAACACAATTCGGTATAAAATTCTTTATTTTTGCTTTAGTTTATTTACTTTTAGATTTAG
AAATATTATTAACTTTCCCTTTCGCTCTTAGTGAGTATGTTAATGGTATTTATGGTCTTTTAGTTAC
TTTAATTTTTATAGCTATAATAACTATAGGATTTATATTTGAATTAGGTAAAAGCGCTCTTAAAATA
GACAGCAGACAAAAATTATATATACCTAAATTGAACGTTAATTACCATACAGAGTATGTTGGAAT
AGGTAAGGTTTCTAAGTAAAGTTATAGAGGCAGAAAACCAAAAAACCTACCAAAGGGTAGCTA

ATGGGAAGCTATTAAAAATAAAAGATGATAACCTATATATAGTATAGTTACTATATATTTACTATA
ACTAGGATTATTATATATATATATATTATCTTATTGTATATTAAGATTATTATTATTATAAGGTATA
ATTAATATAGTATCTTATTGTATAAGAATATAATATATTAACCTATAATTAATTTTATTTTTTAATTT
TTAATTATAATTTTTTTTTATATCTAGATGCTTACACATCTACAGATGTAGAAGAGAACAAAATAT
TGTTGTTATAGTAGTGGATGATATAGAAAAATATTTTTATATTATTTATTTTTGTTAGGTAGCTTTT
GAAGTGTTTGATAGAGAGGATATATGGACGGTAGGAGGGTATTCATTTTAATGAACAGTGGATA
GTTTAAATTAACCTAGTTATAGTTTATGAATTTAAATTATAATTATATTAATGTAGGTTATGATAGA
ATTATGTATTGATCCTTAAGAGTTAAGAGAGATACGCCCACGTATAATACATATTGGTTTAGGGT
TGAGTATATATACTTAAGAGTTAAGAATATATATATACAATATATAATAAATATAGTAATATATTA
ATGGTATGGACTTAACCAGGTTTATATATTATCATTTGATAAACATTAATTATAATTTTTCTATTTA
TTATTATTATTATACTGTGAGATTAATAATTATTAAAATATAATATTACATAGTACGTGATATTTGG
GGATTTTATCTTATTAATTGGCAATTAATGATTCTAATCAAATTTTATTCTCTTTAGTTTAATGGTA
GAACAATGATCTTCTAATTCATTGGTTTTAGTTCGATTCTAAAAAGAGATGAGTAAATAATTTTCT
AGATCAGAAATACTACTTTTAACTACAAAAAGCTTACGCTTTTTAACATTTTTTTGATAAATAACA
ACTATTGTTAATATTTGGCTGTCTATTGGTTTAACTAACAATTACAAAATTTTTTCAATTTTTATAAT
ATAAATTTAAATGAGAATATTAAAAAGTCATTCATTATTAAAATTAGTGAATTCTTACCTTATCGA
TGCGTCACAACCAAGTAACATTAGTTACTTGTGAAATTTTGGTTCATTATTAGCTGTTTGTTTAAT
AGTACAAATTATTACCGGTATTACATTAGCTATGCATTATAGTCCTAGTGTAATGGAAGCTTTTAA
CTCAATAGAGCATATAATGAGAGATGTTAATAACGGGTGATTAGTTCGTTATCTACATAGTAATA
CAGCTTCTGCTTTCTTTTTCTTAGTGTATTTACACATAGGAAGAGGTATATATTACGGATCATATA
GAGCTCCTCGTACTTTAGTTTGAGCTATTGGTACTGTTATATTAATATTAATGATGGCTATCGGTT
TCCTAGGTTATGTTTTACCTTATGGACAGATGTCATTATGAGGTGCTACAGTTATTACTAATCTTA
TTAGTGCTATACCTTGAATAGGGCAAGATATTGTTGAATTCATTTGAGGTGGTTTTTCTGTTAATA
ATGCCACTTTAAACAGATTTTTTGCATTACATTTTGTATTGCCTTTTGTATTAGCTGCTTTAGTTTTA
ATGCACTTAATTGCACTTCATGATACTGCTGGTTCAAGCAATCCTCTTGGTGTTTCAGGTAATTAC
GATAGAATTACATTTGCTCCATATTTTTTATTTAAAGATTTAATTACTATTTTTATATTTATTTTTGTA
TTAAGTGCTTTTGTATTCTTTATGCCTAATGTTTTAGGGGATAGTGATAATTATATTATGGCTAATC
CTATGCAAACTCCTGCTGCTATTGTACCTGAATGATACTTATTACCTTTCTATGCTATTTTAAGATC
TATACCTAATAAATTATTAGGTGTTATAGCGATGTTTAGTGCTATTTTAGCTATTATGTTATTACCT
GTTACAGATTTAGGTAGATCTAGAGGTTTACAATTTAGACCATTTAGTAAAATAGCTTTCTGAGTT
TTTGTTGCTAATTTCTTAGTTTTAATGCAATTAGGTGCTAAACACGTTGAAGATCCATTTATATTAT
TAGGTCAATTAAGTACTGTATTATACTTTAGTTATTTTGTTGCTATATTACCTTTAGCTAGTTACTT
AGATAATAGTTTAACTGATTTATCTAATAAATCTGAATTATTTTTAAATAAAACTAACTAAATATAT
TAAGATTATTATTTAATATATTTTCTATTTAAGATACTATTAATTTAGTATTTTGGGTTTTCAGTTTA
TAATTTATATTATATTATGCATTACCCTCCACCTTGCTTTGTAGTAAGCTAATCTGTTATTTCCTTTA
GTTTAATGGTAGAACAATGATCTTCTAATTCATTGGTTTTAGTTCGAATCTAAAAAGGAAATAAG
AAATATATTCTTATTATTACTTATATAATAATTATTTCTTAAAAATATACATTTTGCATTATAGCCGT
TTAGCTGTATTAAAATGTAAAATGATATAAAATAGAATAAATATTTAAATTATTCCTATGTTATATT
ATCCTATATTGCAACCATTATCAGAAGTTGTATTAATACTTGTACCTGCCTTATTAGCTGTAGCTT
ATGTTACAGTTGCTGAAAGAAAAACTATGGCTAGTATGCAAAGAAGATTAGGTCCTAATGCTGT
AGGTTACTATGGACTATTGCAAGCATTTGCTGATGCCTTAAAACTTTTATTAAAAGAATATGTAG
CTCCTACACAATCTAATATTGTTCTTTTCTTTTTAGGTCCTGTAATAACTTTAATTTTTGCATTATTA
GGTTACGCTGTTATACCCTATGGTCCTGGTTCAGGGATAAGCGACATGAATTTAGGTATATTTTA
CATGTTAGCTGTGTCATCTTTAGCTACATACGGTATTCTATTAGCTGGTTGAAGTGCGAATAGTA

AATACGCTTTTCTAGGTTCTCTTAGAAGTACAGCTCAATTAATTAGTTATGAATTAATATTAAGTT
CAGCTATATTAATAGTAATTATGATAACAGGAAATTTAAATTTAACTGTTTGTACTGAATCTCAAA
GAGCTATTTGATTTATACTACCTTTATTTCCTGTGTTTATAATATTTTTCATAGGATCTATAGCTGA
GACAAATAGAGCTCCTTTTGATTTAGCCGAGGCTAACCTGCTAATCTGGTTTGGTCTGGTTATAT
GTCACAAATTGCTAGGAAACCTTTTTATTTTAAAAACAAAAGACAATTAGCAGGAAACTTAATTT
AACCTAATTAAAAATATTAGATAATTAAACTCTTCATAGACTAAACGTGACAATTTAATATATATA
TATATTTATTTATATATATGATTAAATAAGATATAGTCAATCATCGGTGTGAATCGACTTAAAAAA
AAAAGCACATGGGTTAAACCCATCTCCCCTTATTAGGGGAATCAGAACTTGTTAGTGGGTTCAT
GACAGAGCATGCTGCCGTAGTTTTCGTATTCTTCTTTTTAGCTGAGTACGGTAGTATTGTACTAAT
GTGTATTTTAACTAGTATATTATTTATTGGTGGTTACTTATTATTTGAAATATCCTATGTTTTTACTG
TGGTAAATTATATTTTCTTTGAATTATTCTTTATAGACTGAGTAACATTTGTAGAGGTACAATCTTT
ATACACTGATTTTTTAAATAATTCTATCATTGAAGGATTATTATATGGGTTTAATCTAGGATTAAA
AAGTTCTTTAATGATATTCACATTTATTTGAGCTAGAGCATCCTTCCCTAGAATACGATTTGATCA
ACTAATGGGCTTCTGTTGAACAGTTTTATTACCTATTAATTTTGCAATTATTATATTAGTACCTTGT
GTTTTATATAGTTTTAACTTATTACCTGTAAATATACCATTGTTCTAGCTCACACACCCGCCCGCC
CTACTGCCACAAGGCTACAGTACATATGAGGAGGGGAACTAAAGATCTAGAACTATCCTAGTTA
ATAATTACACTTAATAGTATACTAAATAGACCATCTATCATACTCGAGAATAGTGATAGTGTAAT
TATACACTATTTTAACTGTATTCTACACTATTAGCATATTATTATCTTTATATGATAATAATTTTATA
ACTTAATTTATTAGGTTACATTAACAAAAAATTCGTTAACTTTTTTCACCACTTTTTAATACAAAAT
ACGAATTTTA
>YN690
ATAACAATTCTAAAGAACATAAAGAGTTATTAGATAAAAATAATTCACCTATACAGTTAATAAAT
CAACTTAAAGGGTATTTTTTCATAAATCCTTTATTAGCTTTAAGTTTAGCTATTACTATTTTCTCTTT
TGCAGGTATTCCTCCTCTTGTAGGGTTCTTTGCTAAACAGATGGTATTAAGCGCGGCTATTGATC
AAGGTTATATCTTTTTATCTTTAGTTGCAATATTAACTAGTGTTATAGGAGGGGTTTATTATTTAAA
TATAATTAAAGAAATGTTCTTTTATTCACCTGACTATAAATTAAACGAAGAAATTAAAAATAATAC
TATTAATGGTCAAATTATTAATAGAAACAATAAAATATTAAATGTTGAATTTAATTATACAAATGT
AGTTATGTCTAGTTCTGTGGCAATAACTATTTCTACTATTACATTAGTAGTTTTATTATTCATGTTT
ATGAATAAAGAATGATTAAGTCTGGGTACTATATTGGTACAATCTTTATTTAGCTATTAATGAGTA
GTATGACATTATTTATAGGGTTTGTATCTGTTATAGCTATTTTATTTTTAGCCATTAATTTTATATTT
GCTCCTCATAATCCTTATCAAGAAAAATATAGTATTTTCGAGTGTGGTTTCCATAGTTTTTTAGGG
CAAAATAGAACACAATTCGGTATAAAATTCTTTATTTTTGCTTTAGTTTATTTACTTTTAGATTTAG
AAATATTATTAACTTTCCCTTTCGCTCTTAGTGAGTATGTTAATGGTATTTATGGTCTTTTAGTTAC
TTTAATTTTTATAGCTATAATAACTATAGGATTTATATTTGAATTAGGTAAAAGCGCTCTTAAAATA
GACAGCAGACAAAAATTATATATACCTAAATTGAACGTTAATTACCATACAGAGTATGTTGGAAT
AGGTAAGGTTTCTAAGTAAAGTTATAGAGGCAGAAAACCAAAAAACCTACCAAAGGGTAGCTA
ATGGGAAGCTATTAAAAATAAAAGATGATAACCTATATATAGTATAGTTACTATATATTTACTATA
ACTAGGATTATTATATATATATATATATTATCTTATTGTATATTAAGATTATTATTATTATAAGGTATA
ATTAATATAGTATCTTATTGTATAAGAATATAATATATTAACCTATAATTAATTTTATTTTTTAATTT
TTAATTATAATTTTTTTTTATATCTAGATGCTTACACATCTACAGATGTAGAAGAGAACAAAATAT
TGTTGTTATAGTAGTGGATGATATAGAAAAATATTTTTATATTATTTATTTTTGTTAGGTAGCTTTT
GAAGTGTTTGATAGAGAGGATATATGGACGGTAGGAGGGTATTCATTTTAATGAACAGTGGATA
GTTTAAATTAACCTAGTTATAGTTTATGAATTTAAATTATAATTATATTAATGTAGGTTATGATAGA
ATTATGTATTGATCCTTAAGAGTTAAGAGAGATACGCCCACGTATAATACATATTGGTTTAGGGT

TGAGTATATATACTTAAGAGTTAAGAATATATATATACAATATATAATAAATATAGTAATATATTA
ATGGTATGGACTTAACCAGGTTTATATATTATCATTTGATAAACATTAATTATAATTTTTCTATTTA
TTATTATTATTATACTGTGAGATTAATAATTATTAAAATATAATATTACATAGTACGTGATATTTGG
GGATTTTATCTTATTAATTGGCAATTAATGATTCTAATCAAATTTTATTCTCTTTAGTTTAATGGTA
GAACAATGATCTTCTAATTCATTGGTTTTAGTTCGATTCTAAAAAGAGATGAGTAAATAATTTTCT
AGATCAGAAATACTACTTTTAACTACAAAAAGCTTACGCTTTTTAACATTTTTTTGATAAATAACA
ACTATTGTTAATATTTGGCTGTCTATTGGTTTAACTAACAATTACAAAATTTTTTCAATTTTTATAAT
ATAAATTTAAATGAGAATATTAAAAAGTCATTCATTATTAAAATTAGTGAATTCTTACCTTATCGA
TGCGTCACAACCAAGTAACATTAGTTACTTGTGAAATTTTGGTTCATTATTAGCTGTTTGTTTAAT
AGTACAAATTATTACCGGTATTACATTAGCTATGCATTATAGTCCTAGTGTAATGGAAGCTTTTAA
CTCAATAGAGCATATAATGAGAGATGTTAATAACGGGTGATTAGTTCGTTATCTACATAGTAATA
CAGCTTCTGCTTTCTTTTTCTTAGTGTATTTACACATAGGAAGAGGTATATATTACGGATCATATA
GAGCTCCTCGTACTTTAGTTTGAGCTATTGGTACTGTTATATTAATATTAATGATGGCTATCGGTT
TCCTAGGTTATGTTTTACCTTATGGACAGATGTCATTATGAGGTGCTACAGTTATTACTAATCTTA
TTAGTGCTATACCTTGAATAGGGCAAGATATTGTTGAATTCATTTGAGGTGGTTTTTCTGTTAATA
ATGCCACTTTAAACAGATTTTTTGCATTACATTTTGTATTGCCTTTTGTATTAGCTGCTTTAGTTTTA
ATGCACTTAATTGCACTTCATGATACTGCTGGTTCAAGCAATCCTCTTGGTGTTTCAGGTAATTAC
GATAGAATTACATTTGCTCCATATTTTTTATTTAAAGATTTAATTACTATTTTTATATTTATTTTTGTA
TTAAGTGCTTTTGTATTCTTTATGCCTAATGTTTTAGGGGATAGTGATAATTATATTATGGCTAATC
CTATGCAAACTCCTGCTGCTATTGTACCTGAATGATACTTATTACCTTTCTATGCTATTTTAAGATC
TATACCTAATAAATTATTAGGTGTTATAGCGATGTTTAGTGCTATTTTAGCTATTATGTTATTACCT
GTTACAGATTTAGGTAGATCTAGAGGTTTACAATTTAGACCATTTAGTAAAATAGCTTTCTGAGTT
TTTGTTGCTAATTTCTTAGTTTTAATGCAATTAGGTGCTAAACACGTTGAAGATCCATTTATATTAT
TAGGTCAATTAAGTACTGTATTATACTTTAGTTATTTTGTTGCTATATTACCTTTAGCTAGTTACTT
AGATAATAGTTTAACTGATTTATCTAATAAATCTGAATTATTTTTAAATAAAACTAACTAAATATAT
TAAGATTATTATTTAATATATTTTCTATTTAAGATACTATTAATTTAGTATTTTGGGTTTTCAGTTTA
TAATTTATATTATATTATGCATTACCCTCCACCTTGCTTTGTAGTAAGCTAATCTGTTATTTCCTTTA
GTTTAATGGTAGAACAATGATCTTCTAATTCATTGGTTTTAGTTCGAATCTAAAAAGGAAATAAG
AAATATATTCTTATTATTACTTATATAATAATTATTTCTTAAAAATATACATTTTGCATTATAGCCGT
TTAGCTGTATTAAAATGTAAAATGATATAAAATAGAATAAATATTTAAATTATTCCTATGTTATATT
ATCCTATATTGCAACCATTATCAGAAGTTGTATTAATACTTGTACCTGCCTTATTAGCTGTAGCTT
ATGTTACAGTTGCTGAAAGAAAAACTATGGCTAGTATGCAAAGAAGATTAGGTCCTAATGCTGT
AGGTTACTATGGACTATTGCAAGCATTTGCTGATGCCTTAAAACTTTTATTAAAAGAATATGTAG
CTCCTACACAATCTAATATTGTTCTTTTCTTTTTAGGTCCTGTAATAACTTTAATTTTTGCATTATTA
GGTTACGCTGTTATACCCTATGGTCCTGGTTCAGGGATAAGCGACATGAATTTAGGTATATTTTA
CATGTTAGCTGTGTCATCTTTAGCTACATACGGTATTCTATTAGCTGGTTGAAGTGCGAATAGTA
AATACGCTTTTCTAGGTTCTCTTAGAAGTACAGCTCAATTAATTAGTTATGAATTAATATTAAGTT
CAGCTATATTAATAGTAATTATGATAACAGGAAATTTAAATTTAACTGTTTGTACTGAATCTCAAA
GAGCTATTTGATTTATACTACCTTTATTTCCTGTGTTTATAATATTTTTCATAGGATCTATAGCTGA
GACAAATAGAGCTCCTTTTGATTTAGCCGAGGCTAACCTGCTAATCTGGTTTGGTCTGGTTATAT
GTCACAAATTGCTAGGAAACCTTTTTATTTTAAAAACAAAAGACAATTAGCAGGAAACTTAATTT
AACCTAATTAAAAATATTAGATAATTAAACTCTTCATAGACTAAACGTGACAATTTAATATATATA
TATATTTATTTATATATATGATTAAATAAGATATAGTCAATCATCGGTGTGAATCGACTTAAAAAA
AAAAGCACATGGGTTAAACCCATCTCCCCTTATTAGGGGAATCAGAACTTGTTAGTGGGTTCAT

GACAGAGCATGCTGCCGTAGTTTTCGTATTCTTCTTTTTAGCTGAGTACGGTAGTATTGTACTAAT
GTGTATTTTAACTAGTATATTATTTATTGGTGGTTACTTATTATTTGAAATATCCTATGTTTTTACTG
TGGTAAATTATATTTTCTTTGAATTATTCTTTATAGACTGAGTAACATTTGTAGAGGTACAATCTTT
ATACACTGATTTTTTAAATAATTCTATCATTGAAGGATTATTATATGGGTTTAATCTAGGATTAAA
AAGTTCTTTAATGATATTCACATTTATTTGAGCTAGAGCATCCTTCCCTAGAATACGATTTGATCA
ACTAATGGGCTTCTGTTGAACAGTTTTATTACCTATTAATTTTGCAATTATTATATTAGTACCTTGT
GTTTTATATAGTTTTAACTTATTACCTGTAAATATACCATTGTTCTAGCTCACACACCCGCCCGCC
CTACTGCCACAAGGCTACAGTACATATGAGGAGGGGAACTAAAGATCTAGAACTATCCTAGTTA
ATAATTACACTTAATAGTATACTAAATAGACCATCTATCATACTCGAGAATAGTGATAGTGTAAT
TATACACTATTTTAACTGTATTCTACACTATTAGCATATTATTATCTTTATATGATAATAATTTTATA
ACTTAATTTATTAGGTTACATTAACAAAAAATTCGTTAACTTTTTTCACCACTTTTTAATACAAAAT
ACGAATTTTA
>YN694
ATAACAATTCTAAAGAACATAAAGAGTTATTAGATAAAAATAATTCACCTATACAGTTAATAAAT
CAACTTAAAGGGTATTTTTTCATAAATCCTTTATTAGCTTTAAGTTTAGCTATTACTATTTTCTCTTT
TGCAGGTATTCCTCCTCTTGTAGGGTTCTTTGCTAAACAGATGGTATTAAGCGCGGCTATTGATC
AAGGTTATATCTTTTTATCTTTAGTTGCAATATTAACTAGTGTTATAGGAGGGGTTTATTATTTAAA
TATAATTAAAGAAATGTTCTTTTATTCACCTGACTATAAATTAAACGAAGAAATTAAAAATAATAC
TATTAATGGTCAAATTATTAATAGAAACAATAAAATATTAAATGTTGAATTTAATTATACAAATGT
AGTTATGTCTAGTTCTGTGGCAATAACTATTTCTACTATTACATTAGTAGTTTTATTATTCATGTTT
ATGAATAAAGAATGATTAAGTCTGGGTACTATATTGGTACAATCTTTATTTAGCTATTAATGAGTA
GTATGACATTATTTATAGGGTTTGTATCTGTTATAGCTATTTTATTTTTAGCCATTAATTTTATATTT
GCTCCTCATAATCCTTATCAAGAAAAATATAGTATTTTCGAGTGTGGTTTCCATAGTTTTTTAGGG
CAAAATAGAACACAATTCGGTATAAAATTCTTTATTTTTGCTTTAGTTTATTTACTTTTAGATTTAG
AAATATTATTAACTTTCCCTTTCGCTCTTAGTGAGTATGTTAATGGTATTTATGGTCTTTTAGTTAC
TTTAATTTTTATAGCTATAATAACTATAGGATTTATATTTGAATTAGGTAAAAGCGCTCTTAAAATA
GACAGCAGACAAAAATTATATATACCTAAATTGAACGTTAATTACCATACAGAGTATGTTGGAAT
AGGTAAGGTTTCTAAGTAAAGTTATAGAGGCAGAAAACCAAAAAACCTACCAAAGGGTAGCTA
ATGGGAAGCTATTAAAAATAAAAGATGATAACCTATATATAGTATAGTTACTATATATTTACTATA
ACTAGGATTATTATATATATATATATTATCTTATTGTATATTAAGATTATTATTATTATAAGGTATA
ATTAATATAGTATCTTATTGTATAAGAATATAATATATTAACCTATAATTAATTTTATTTTTTAATTT
TTAATTATAATTTTTTTTTATATCTAGATGCTTACACATCTACAGATGTAGAAGAGAACAAAATAT
TGTTGTTATAGTAGTGGATGATATAGAAAAATATTTTTATATTATTTATTTTTGTTAGGTAGCTTTT
GAAGTGTTTGATAGAGAGGATATATGGACGGTAGGAGGGTATTCATTTTAATGAACAGTGGATA
GTTTAAATTAACCTAGTTATAGTTTATGAATTTAAATTATAATTATATTAATGTAGGTTATGATAGA
ATTATGTATTGATCCTTAAGAGTTAAGAGAGATACGCCCACGTATAATACATATTGGTTTAGGGT
TGAGTATATATACTTAAGAGTTAAGAATATATATATACAATATATAATAAATATAGTAATATATTA
ATGGTATGGACTTAACCAGGTTTATATATTATCATTTGATAAACATTAATTATAATTTTTCTATTTA
TTATTATTATTATACTGTGAGATTAATAATTATTAAAATATAATATTACATAGTACGTGATATTTGG
GGATTTTATCTTATTAATTGGCAATTAATGATTCTAATCAAATTTTATTCTCTTTAGTTTAATGGTA
GAACAATGATCTTCTAATTCATTGGTTTTAGTTCGATTCTAAAAAGAGATGAGTAAATAATTTTCT
AGATCAGAAATACTACTTTTAACTACAAAAAGCTTACGCTTTTTAACATTTTTTTGATAAATAACA
ACTATTGTTAATATTTGGCTGTCTATTGGTTTAACTAACAATTACAAAATTTTTTCAATTTTTATAAT
ATAAATTTAAATGAGAATATTAAAAAGTCATTCATTATTAAAATTAGTGAATTCTTACCTTATCGA

TGCGTCACAACCAAGTAACATTAGTTACTTGTGAAATTTTGGTTCATTATTAGCTGTTTGTTTAAT
AGTACAAATTATTACCGGTATTACATTAGCTATGCATTATAGTCCTAGTGTAATGGAAGCTTTTAA
CTCAATAGAGCATATAATGAGAGATGTTAATAACGGGTGATTAGTTCGTTATCTACATAGTAATA
CAGCTTCTGCTTTCTTTTTCTTAGTGTATTTACACATAGGAAGAGGTATATATTACGGATCATATA
GAGCTCCTCGTACTTTAGTTTGAGCTATTGGTACTGTTATATTAATATTAATGATGGCTATCGGTT
TCCTAGGTTATGTTTTACCTTATGGACAGATGTCATTATGAGGTGCTACAGTTATTACTAATCTTA
TTAGTGCTATACCTTGAATAGGGCAAGATATTGTTGAATTCATTTGAGGTGGTTTTTCTGTTAATA
ATGCCACTTTAAACAGATTTTTTGCATTACATTTTGTATTGCCTTTTGTATTAGCTGCTTTAGTTTTA
ATGCACTTAATTGCACTTCATGATACTGCTGGTTCAAGCAATCCTCTTGGTGTTTCAGGTAATTAC
GATAGAATTACATTTGCTCCATATTTTTTATTTAAAGATTTAATTACTATTTTTATATTTATTTTTGTA
TTAAGTGCTTTTGTATTCTTTATGCCTAATGTTTTAGGGGATAGTGATAATTATATTATGGCTAATC
CTATGCAAACTCCTGCTGCTATTGTACCTGAATGATACTTATTACCTTTCTATGCTATTTTAAGATC
TATACCTAATAAATTATTAGGTGTTATAGCGATGTTTAGTGCTATTTTAGCTATTATGTTATTACCT
GTTACAGATTTAGGTAGATCTAGAGGTTTACAATTTAGACCATTTAGTAAAATAGCTTTCTGAGTT
TTTGTTGCTAATTTCTTAGTTTTAATGCAATTAGGTGCTAAACACGTTGAAGATCCATTTATATTAT
TAGGTCAATTAAGTACTGTATTATACTTTAGTTATTTTGTTGCTATATTACCTTTAGCTAGTTACTT
AGATAATAGTTTAACTGATTTATCTAATAAATCTGAATTATTTTTAAATAAAACTAACTAAATATAT
TAAGATTATTATTTAATATATTTTCTATTTAAGATACTATTAATTTAGTATTTTGGGTTTTCAGTTTA
TAATTTATATTATATTATGCATTACCCTCCACCTTGCTTTGTAGTAAGCTAATCTGTTATTTCCTTTA
GTTTAATGGTAGAACAATGATCTTCTAATTCATTGGTTTTAGTTCGAATCTAAAAAGGAAATAAG
AAATATATTCTTATTATTACTTATATAATAATTATTTCTTAAAAATATACATTTTGCATTATAGCCGT
TTAGCTGTATTAAAATGTAAAATGATATAAAATAGAATAAATATTTAAATTATTCCTATGTTATATT
ATCCTATATTGCAACCATTATCAGAAGTTGTATTAATACTTGTACCTGCCTTATTAGCTGTAGCTT
ATGTTACAGTTGCTGAAAGAAAAACTATGGCTAGTATGCAAAGAAGATTAGGTCCTAATGCTGT
AGGTTACTATGGACTATTGCAAGCATTTGCTGATGCCTTAAAACTTTTATTAAAAGAATATGTAG
CTCCTACACAATCTAATATTGTTCTTTTCTTTTTAGGTCCTGTAATAACTTTAATTTTTGCATTATTA
GGTTACGCTGTTATACCCTATGGTCCTGGTTCAGGGATAAGCGACATGAATTTAGGTATATTTTA
CATGTTAGCTGTGTCATCTTTAGCTACATACGGTATTCTATTAGCTGGTTGAAGTGCGAATAGTA
AATACGCTTTTCTAGGTTCTCTTAGAAGTACAGCTCAATTAATTAGTTATGAATTAATATTAAGTT
CAGCTATATTAATAGTAATTATGATAACAGGAAATTTAAATTTAACTGTTTGTACTGAATCTCAAA
GAGCTATTTGATTTATACTACCTTTATTTCCTGTGTTTATAATATTTTTCATAGGATCTATAGCTGA
GACAAATAGAGCTCCTTTTGATTTAGCCGAGGCTAACCTGCTAATCTGGTTTGGTCTGGTTATAT
GTCACAAATTGCTAGGAAACCTTTTTATTTTAAAAACAAAAGACAATTAGCAGGAAACTTAATTT
AACCTAATTAAAAATATTAGATAATTAAACTCTTCATAGACTAAACGTGACAATTTAATATATATA
TATATTTATTTATATATATGATTAAATAAGATATAGTCAATCATCGGTGTGAATCGACTTAAAAAA
AAAAGCACATGGGTTAAACCCATCTCCCCTTATTAGGGGAATCAGAACTTGTTAGTGGGTTCAT
GACAGAGCATGCTGCCGTAGTTTTCGTATTCTTCTTTTTAGCTGAGTACGGTAGTATTGTACTAAT
GTGTATTTTAACTAGTATATTATTTATTGGTGGTTACTTATTATTTGAAATATCCTATGTTTTTACTG
TGGTAAATTATATTTTCTTTGAATTATTCTTTATAGACTGAGTAACATTTGTAGAGGTACAATCTTT
ATACACTGATTTTTTAAATAATTCTATCATTGAAGGATTATTATATGGGTTTAATCTAGGATTAAA
AAGTTCTTTAATGATATTCACATTTATTTGAGCTAGAGCATCCTTCCCTAGAATACGATTTGATCA
ACTAATGGGCTTCTGTTGAACAGTTTTATTACCTATTAATTTTGCAATTATTATATTAGTACCTTGT
GTTTTATATAGTTTTAACTTATTACCTGTAAATATACCATTGTTCTAGCTCACACACCCGCCCGCC
CTACTGCCACAAGGCTACAGTACATATGAGGAGGGGAACTAAAGATCTAGAACTATCCTAGTTA

ATAATTACACTTAATAGTATACTAAATAGACCATCTATCATACTCGAGAATAGTGATAGTGTAAT
TATACACTATTTTAACTGTATTCTACACTATTAGCATATTATTATCTTTATATGATAATAATTTTATA
ACTTAATTTATTAGGTTACATTAACAAAAAATTCGTTAACTTTTTTCACCACTTTTTAATACAAAAT
ACGAATTATA
>YN714
ATAACAATTCTAAAGAACATAAAGAGTTATTAGATAAAAATAATTCACCTATACAGTTAATAAAT
CAACTTAAAGGGTATTTTTTCATAAATCCTTTATTAGCTTTAAGTTTAGCTATTACTATTTTCTCTTT
TGCAGGTATTCCTCCTCTTGTAGGGTTCTTTGCTAAACAGATGGTATTAAGCGCGGCTATTGATC
AAGGTTATATCTTTTTATCTTTAGTTGCAATATTAACTAGTGTTATAGGAGGGGTTTATTATTTAAA
TATAATTAAAGAAATGTTCTTTTATTCACCTGACTATAAATTAAACGAAGAAATTAAAAATAATAC
TATTAATGGTCAAATTATTAATAGAAACAATAAAATATTAAATGTTGAATTTAATTATACAAATGT
AGTTATGTCTAGTTCTGTGGCAATAACTATTTCTACTATTACATTAGTAGTTTTATTATTCATGTTT
ATGAATAAAGAATGATTAAGTCTGGGTACTATATTGGTACAATCTTTATTTAGCTATTAATGAGTA
GTATGACATTATTTATAGGGTTTGTATCTGTTATAGCTATTTTATTTTTAGCCATTAATTTTATATTT
GCTCCTCATAATCCTTATCAAGAAAAATATAGTATTTTCGAGTGTGGTTTCCATAGTTTTTTAGGG
CAAAATAGAACACAATTCGGTATAAAATTCTTTATTTTTGCTTTAGTTTATTTACTTTTAGATTTAG
AAATATTATTAACTTTCCCTTTCGCTCTTAGTGAGTATGTTAATGGTATTTATGGTCTTTTAGTTAC
TTTAATTTTTATAGCTATAATAACTATAGGATTTATATTTGAATTAGGTAAAAGCGCTCTTAAAATA
GACAGCAGACAAAAATTATATATACCTAAATTGAACGTTAATTACCATACAGAGTATGTTGGAAT
AGGTAAGGTTTCTAAGTAAAGTTATAGAGGCAGAAAACCAAAAAACCTACCAAAGGGTAGCTA
ATGGGAAGCTATTAAAAATAAAAGATGATAACCTATATATAGTATAGTTACTATATATTTACTATA
ACTAGGATTATTATATATATATATATATTATCTTATTGTATATTAAGATTATTATTATTATAAGGTATA
ATTAATATAGTATCTTATTGTATAAGAATATAATATATTAACCTATAATTAATTTTATTTTTTAATTT
TTAATTATAATTTTTTTTTATATCTAGATGCTTACACATCTACAGATGTAGAAGAGAACAAAATAT
TGTTGTTATAGTAGTGGATGATATAGAAAAATATTTTTATATTATTTATTTTTGTTAGGTAGCTTTT
GAAGTGTTTGATAGAGAGGATATATGGACGGTAGGAGGGTATTCATTTTAATGAACAGTGGATA
GTTTAAATTAACCTAGTTATAGTTTATGAATTTAAATTATAATTATATTAATGTAGGTTATGATAGA
ATTATGTATTGATCCTTAAGAGTTAAGAGAGATACGCCCACGTATAATACATATTGGTTTAGGGT
TGAGTATATATACTTAAGAGTTAAGAATATATATATACAATATATAATAAATATAGTAATATATTA
ATGGTATGGACTTAACCAGGTTTATATATTATCATTTGATAAACATTAATTATAATTTTTCTATTTA
TTATTATTATTATACTGTGAGATTAATAATTATTAAAATATAATATTACATAGTACGTGATATTTGG
GGATTTTATCTTATTAATTGGCAATTAATGATTCTAATCAAATTTTATTCTCTTTAGTTTAATGGTA
GAACAATGATCTTCTAATTCATTGGTTTTAGTTCGATTCTAAAAAGAGATGAGTAAATAATTTTCT
AGATCAGAAATACTACTTTTAACTACAAAAAGCTTACGCTTTTTAACATTTTTTTGATAAATAACA
ACTATTGTTAATATTTGGCTGTCTATTGGTTTAACTAACAATTACAAAATTTTTTCAATTTTTATAAT
ATAAATTTAAATGAGAATATTAAAAAGTCATTCATTATTAAAATTAGTGAATTCTTACCTTATCGA
TGCGTCACAACCAAGTAACATTAGTTACTTGTGAAATTTTGGTTCATTATTAGCTGTTTGTTTAAT
AGTACAAATTATTACCGGTATTACATTAGCTATGCATTATAGTCCTAGTGTAATGGAAGCTTTTAA
CTCAATAGAGCATATAATGAGAGATGTTAATAACGGGTGATTAGTTCGTTATCTACATAGTAATA
CAGCTTCTGCTTTCTTTTTCTTAGTGTATTTACACATAGGAAGAGGTATATATTACGGATCATATA
GAGCTCCTCGTACTTTAGTTTGAGCTATTGGTACTGTTATATTAATATTAATGATGGCTATCGGTT
TCCTAGGTTATGTTTTACCTTATGGACAGATGTCATTATGAGGTGCTACAGTTATTACTAATCTTA
TTAGTGCTATACCTTGAATAGGGCAAGATATTGTTGAATTCATTTGAGGTGGTTTTTCTGTTAATA
ATGCCACTTTAAACAGATTTTTTGCATTACATTTTGTATTGCCTTTTGTATTAGCTGCTTTAGTTTTA

ATGCACTTAATTGCACTTCATGATACTGCTGGTTCAAGCAATCCTCTTGGTGTTTCAGGTAATTAC
GATAGAATTACATTTGCTCCATATTTTTTATTTAAAGATTTAATTACTATTTTTATATTTATTTTTGTA
TTAAGTGCTTTTGTATTCTTTATGCCTAATGTTTTAGGGGATAGTGATAATTATATTATGGCTAATC
CTATGCAAACTCCTGCTGCTATTGTACCTGAATGATACTTATTACCTTTCTATGCTATTTTAAGATC
TATACCTAATAAATTATTAGGTGTTATAGCGATGTTTAGTGCTATTTTAGCTATTATGTTATTACCT
GTTACAGATTTAGGTAGATCTAGAGGTTTACAATTTAGACCATTTAGTAAAATAGCTTTCTGAGTT
TTTGTTGCTAATTTCTTAGTTTTAATGCAATTAGGTGCTAAACACGTTGAAGATCCATTTATATTAT
TAGGTCAATTAAGTACTGTATTATACTTTAGTTATTTTGTTGCTATATTACCTTTAGCTAGTTACTT
AGATAATAGTTTAACTGATTTATCTAATAAATCTGAATTATTTTTAAATAAAACTAACTAAATATAT
TAAGATTATTATTTAATATATTTTCTATTTAAGATACTATTAATTTAGTATTTTGGGTTTTCAGTTTA
TAATTTATATTATATTATGCATTACCCTCCACCTTGCTTTGTAGTAAGCTAATCTGTTATTTCCTTTA
GTTTAATGGTAGAACAATGATCTTCTAATTCATTGGTTTTAGTTCGAATCTAAAAAGGAAATAAG
AAATATATTCTTATTATTACTTATATAATAATTATTTCTTAAAAATATACATTTTGCATTATAGCCGT
TTAGCTGTATTAAAATGTAAAATGATATAAAATAGAATAAATATTTAAATTATTCCTATGTTATATT
ATCCTATATTGCAACCATTATCAGAAGTTGTATTAATACTTGTACCTGCCTTATTAGCTGTAGCTT
ATGTTACAGTTGCTGAAAGAAAAACTATGGCTAGTATGCAAAGAAGATTAGGTCCTAATGCTGT
AGGTTACTATGGACTATTGCAAGCATTTGCTGATGCCTTAAAACTTTTATTAAAAGAATATGTAG
CTCCTACACAATCTAATATTGTTCTTTTCTTTTTAGGTCCTGTAATAACTTTAATTTTTGCATTATTA
GGTTACGCTGTTATACCCTATGGTCCTGGTTCAGGGATAAGCGACATGAATTTAGGTATATTTTA
CATGTTAGCTGTGTCATCTTTAGCTACATACGGTATTCTATTAGCTGGTTGAAGTGCGAATAGTA
AATACGCTTTTCTAGGTTCTCTTAGAAGTACAGCTCAATTAATTAGTTATGAATTAATATTAAGTT
CAGCTATATTAATAGTAATTATGATAACAGGAAATTTAAATTTAACTGTTTGTACTGAATCTCAAA
GAGCTATTTGATTTATACTACCTTTATTTCCTGTGTTTATAATATTTTTCATAGGATCTATAGCTGA
GACAAATAGAGCTCCTTTTGATTTAGCCGAGGCTAACCTGCTAATCTGGTTTGGTCTGGTTATAT
GTCACAAATTGCTAGGAAACCTTTTTATTTTAAAAACAAAAGACAATTAGCAGGAAACTTAATTT
AACCTAATTAAAAATATTAGATAATTAAACTCTTCATAGACTAAACGTGACAATTTAATATATATA
TATATTTATTTATATATATGATTAAATAAGATATAGTCAATCATCGGTGTGAATCGACTTAAAAAA
AAAAGCACATGGGTTAAACCCATCTCCCCTTATTAGGGGAATCAGAACTTGTTAGTGGGTTCAT
GACAGAGCATGCTGCCGTAGTTTTCGTATTCTTCTTTTTAGCTGAGTACGGTAGTATTGTACTAAT
GTGTATTTTAACTAGTATATTATTTATTGGTGGTTACTTATTATTTGAAATATCCTATGTTTTTACTG
TGGTAAATTATATTTTCTTTGAATTATTCTTTATAGACTGAGTAACATTTGTAGAGGTACAATCTTT
ATACACTGATTTTTTAAATAATTCTATCATTGAAGGATTATTATATGGGTTTAATCTAGGATTAAA
AAGTTCTTTAATGATATTCACATTTATTTGAGCTAGAGCATCCTTCCCTAGAATACGATTTGATCA
ACTAATGGGCTTCTGTTGAACAGTTTTATTACCTATTAATTTTGCAATTATTATATTAGTACCTTGT
GTTTTATATAGTTTTAACTTATTACCTGTAAATATACCATTGTTCTAGCTCACACACCCGCCCGCC
CTACTGCCACAAGGCTACAGTACATATGAGGAGGGGAACTAAAGATCTAGAACTATCCTAGTTA
ATAATTACACTTAATAGTATACTAAATAGACCATCTATCATACTCGAGAATAGTGATAGTGTAAT
TATACACTATTTTAACTGTATTCTACACTATTAGGATATTATTATCTTTATATGATAATAATTTTATA
ACTTAATTTATTAGGTTACATTAACAAAAAAATTCGTTAACTTTTTTCACCACTTTTTAATACAAAAT
ACGAATTTTA
>YN716
ATAACAATTCTAAAGAACATAAAGAGTTATTAGATAAAAATAATTCACCTATACAGTTAATAAAT
CAACTTAAAGGGTATTTTTTCATAAATCCTTTATTAGCTTTAAGTTTAGCTATTACTATTTTCTCTTT
TGCAGGTATTCCTCCTCTTGTAGGGTTCTTTGCTAAACAGATGGTATTAAGCGCGGCTATTGATC

AAGGTTATATCTTTTTATCTTTAGTTGCAATATTAACTAGTGTTATAGGAGGGGTTTATTATTTAAA
TATAATTAAAGAAATGTTCTTTTATTCACCTGACTATAAATTAAACGAAGAAATTAAAAATAATAC
TATTAATGGTCAAATTATTAATAGAAACAATAAAATATTAAATGTTGAATTTAATTATACAAATGT
AGTTATGTCTAGTTCTGTGGCAATAACTATTTCTACTATTACATTAGTAGTTTTATTATTCATGTTT
ATGAATAAAGAATGATTAAGTCTGGGTACTATATTGGTACAATCTTTATTTAGCTATTAATGAGTA
GTATGACATTATTTATAGGGTTTGTATCTGTTATAGCTATTTTATTTTTAGCCATTAATTTTATATTT
GCTCCTCATAATCCTTATCAAGAAAAATATAGTATTTTCGAGTGTGGTTTCCATAGTTTTTTAGGG
CAAAATAGAACACAATTCGGTATAAAATTCTTTATTTTTGCTTTAGTTTATTTACTTTTAGATTTAG
AAATATTATTAACTTTCCCTTTCGCTCTTAGTGAGTATGTTAATGGTATTTATGGTCTTTTAGTTAC
TTTAATTTTTATAGCTATAATAACTATAGGATTTATATTTGAATTAGGTAAAAGCGCTCTTAAAATA
GACAGCAGACAAAAATTATATATACCTAAATTGAACGTTAATTACCATACAGAGTATGTTGGAAT
AGGTAAGGTTTCTAAGTAAAGTTATAGAGGCAGAAAACCAAAAAACCTACCAAAGGGTAGCTA
ATGGGAAGCTATTAAAAATAAAAGATGATAACCTATATATAGTATAGTTACTATATATTTACTATA
ACTAGGATTATTATATATATATATATTATCTTATTGTATATTAAGATTATTATTATTATAAGGTATA
ATTAATATAGTATCTTATTGTATAAGAATATAATATATTAACCTATAATTAATTTTATTTTTTAATTT
TTAATTATAATTTTTTTTTATATCTAGATGCTTACACATCTACAGATGTAGAAGAGAACAAAATAT
TGTTGTTATAGTAGTGGATGATATAGAAAAATATTTTTATATTATTTATTTTTGTTAGGTAGCTTTT
GAAGTGTTTGATAGAGAGGATATATGGACGGTAGGAGGGTATTCATTTTAATGAACAGTGGATA
GTTTAAATTAACCTAGTTATAGTTTATGAATTTAAATTATAATTATATTAATGTAGGTTATGATAGA
ATTATGTATTGATCCTTAAGAGTTAAGAGAGATACGCCCACGTATAATACATATTGGTTTAGGGT
TGAGTATATATACTTAAGAGTTAAGAATATATATATACAATATATAATAAATATAGTAATATATTA
ATGGTATGGACTTAACCAGGTTTATATATTATCATTTGATAAACATTAATTATAATTTTTCTATTTA
TTATTATTATTATACTGTGAGATTAATAATTATTAAAATATAATATTACATAGTACGTGATATTTGG
GGATTTTATCTTATTAATTGGCAATTAATGATTCTAATCAAATTTTATTCTCTTTAGTTTAATGGTA
GAACAATGATCTTCTAATTCATTGGTTTTAGTTCGATTCTAAAAAGAGATGAGTAAATAATTTTCT
AGATCAGAAATACTACTTTTAACTACAAAAAGCTTACGCTTTTTAACATTTTTTTGATAAATAACA
ACTATTGTTAATATTTGGCTGTCTATTGGTTTAACTAACAATTACAAAATTTTTTCAATTTTTATAAT
ATAAATTTAAATGAGAATATTAAAAAGTCATTCATTATTAAAATTAGTGAATTCTTACCTTATCGA
TGCGTCACAACCAAGTAACATTAGTTACTTGTGAAATTTTGGTTCATTATTAGCTGTTTGTTTAAT
AGTACAAATTATTACCGGTATTACATTAGCTATGCATTATAGTCCTAGTGTAATGGAAGCTTTTAA
CTCAATAGAGCATATAATGAGAGATGTTAATAACGGGTGATTAGTTCGTTATCTACATAGTAATA
CAGCTTCTGCTTTCTTTTTCTTAGTGTATTTACACATAGGAAGAGGTATATATTACGGATCATATA
GAGCTCCTCGTACTTTAGTTTGAGCTATTGGTACTGTTATATTAATATTAATGATGGCTATCGGTT
TCCTAGGTTATGTTTTACCTTATGGACAGATGTCATTATGAGGTGCTACAGTTATTACTAATCTTA
TTAGTGCTATACCTTGAATAGGGCAAGATATTGTTGAATTCATTTGAGGTGGTTTTTCTGTTAATA
ATGCCACTTTAAACAGATTTTTTGCATTACATTTTGTATTGCCTTTTGTATTAGCTGCTTTAGTTTTA
ATGCACTTAATTGCACTTCATGATACTGCTGGTTCAAGCAATCCTCTTGGTGTTTCAGGTAATTAC
GATAGAATTACATTTGCTCCATATTTTTTATTTAAAGATTTAATTACTATTTTTATATTTATTTTTGTA
TTAAGTGCTTTTGTATTCTTTATGCCTAATGTTTTAGGGGATAGTGATAATTATATTATGGCTAATC
CTATGCAAACTCCTGCTGCTATTGTACCTGAATGATACTTATTACCTTTCTATGCTATTTTAAGATC
TATACCTAATAAATTATTAGGTGTTATAGCGATGTTTAGTGCTATTTTAGCTATTATGTTATTACCT
GTTACAGATTTAGGTAGATCTAGAGGTTTACAATTTAGACCATTTAGTAAAATAGCTTTCTGAGTT
TTTGTTGCTAATTTCTTAGTTTTAATGCAATTAGGTGCTAAACACGTTGAAGATCCATTTATATTAT
TAGGTCAATTAAGTACTGTATTATACTTTAGTTATTTTGTTGCTATATTACCTTTAGCTAGTTACTT

AGATAATAGTTTAACTGATTTATCTAATAAATCTGAATTATTTTTAAATAAAACTAACTAAATATAT
TAAGATTATTATTTAATATATTTTCTATTTAAGATACTATTAATTTAGTATTTTGGGTTTTCAGTTTA
TAATTTATATTATATTATGCATTACCCTCCACCTTGCTTTGTAGTAAGCTAATCTGTTATTTCCTTTA
GTTTAATGGTAGAACAATGATCTTCTAATTCATTGGTTTTAGTTCGAATCTAAAAAGGAAATAAG
AAATATATTCTTATTATTACTTATATAATAATTATTTCTTAAAAATATACATTTTGCATTATAGCCGT
TTAGCTGTATTAAAATGTAAAATGATATAAAATAGAATAAATATTTAAATTATTCCTATGTTATATT
ATCCTATATTGCAACCATTATCAGAAGTTGTATTAATACTTGTACCTGCCTTATTAGCTGTAGCTT
ATGTTACAGTTGCTGAAAGAAAAACTATGGCTAGTATGCAAAGAAGATTAGGTCCTAATGCTGT
AGGTTACTATGGACTATTGCAAGCATTTGCTGATGCCTTAAAACTTTTATTAAAAGAATATGTAG
CTCCTACACAATCTAATATTGTTCTTTTCTTTTTAGGTCCTGTAATAACTTTAATTTTTGCATTATTA
GGTTACGCTGTTATACCCTATGGTCCTGGTTCAGGGATAAGCGACATGAATTTAGGTATATTTTA
CATGTTAGCTGTGTCATCTTTAGCTACATACGGTATTCTATTAGCTGGTTGAAGTGCGAATAGTA
AATACGCTTTTCTAGGTTCTCTTAGAAGTACAGCTCAATTAATTAGTTATGAATTAATATTAAGTT
CAGCTATATTAATAGTAATTATGATAACAGGAAATTTAAATTTAACTGTTTGTACTGAATCTCAAA
GAGCTATTTGATTTATACTACCTTTATTTCCTGTGTTTATAATATTTTTCATAGGATCTATAGCTGA
GACAAATAGAGCTCCTTTTGATTTAGCCGAGGCTAACCTGCTAATCTGGTTTGGTCTGGTTATAT
GTCACAAATTGCTAGGAAACCTTTTTATTTTAAAAACAAAAGACAATTAGCAGGAAACTTAATTT
AACCTAATTAAAAATATTAGATAATTAAACTCTTCATAGACTAAACGTGACAATTTAATATATATA
TATATTTATTTATATATATGATTAAATAAGATATAGTCAATCATCGGTGTGAATCGACTTAAAAAA
AAAAGCACATGGGTTAAACCCATCTCCCCTTATTAGGGGAATCAGAACTTGTTAGTGGGTTCAT
GACAGAGCATGCTGCCGTAGTTTTCGTATTCTTCTTTTTAGCTGAGTACGGTAGTATTGTACTAAT
GTGTATTTTAACTAGTATATTATTTATTGGTGGTTACTTATTATTTGAAATATCCTATGTTTTTACTG
TGGTAAATTATATTTTCTTTGAATTATTCTTTATAGACTGAGTAACATTTGTAGAGGTACAATCTTT
ATACACTGATTTTTTAAATAATTCTATCATTGAAGGATTATTATATGGGTTTAATCTAGGATTAAA
AAGTTCTTTAATGATATTCACATTTATTTGAGCTAGAGCATCCTTCCCTAGAATACGATTTGATCA
ACTAATGGGCTTCTGTTGAACAGTTTTATTACCTATTAATTTTGCAATTATTATATTAGTACCTTGT
GTTTTATATAGTTTTAACTTATTACCTGTAAATATACCATTGTTCTAGCTCACACACCCGCCCGCC
CTACTGCCACAAGGCTACAGTACATATGAGGAGGGGAACTAAAGATCTAGAACTATCCTAGTTA
ATAATTACACTTAATAGTATACTAAATAGACCATCTATCATACTCGAGAATAGTGATAGTGTAAT
TATACACTATTTTAACTGTATTCTACACTATTAGCATATTATTATCTTTATATGATAATAATTTTATA
ACTTAATTTATTAGGTTACATTAACAAAAAATTCGTTAACTTTTTTCACCACTTTTTAATACAAAAT
ACGAATTTTA
>YN718
ATAACAATTCTAAAGAACATAAAGAGTTATTAGATAAAAATAATTCACCTATACAGTTAATAAAT
CAACTTAAAGGGTATTTTTTCATAAATCCTTTATTAGCTTTAAGTTTAGCTATTACTATTTTCTCTTT
TGCAGGTATTCCTCCTCTTGTAGGGTTCTTTGCTAAACAGATGGTATTAAGCGCGGCTATTGATC
AAGGTTATATCTTTTTATCTTTAGTTGCAATATTAACTAGTGTTATAGGAGGGGTTTATTATTTAAA
TATAATTAAAGAAATGTTCTTTTATTCACCTGACTATAAATTAAACGAAGAAATTAAAAATAATAC
TATTAATGGTCAAATTATTAATAGAAACAATAAAATATTAAATGTTGAATTTAATTATACAAATGT
AGTTATGTCTAGTTCTGTGGCAATAACTATTTCTACTATTACATTAGTAGTTTTATTATTCATGTTT
ATGAATAAAGAATGATTAAGTCTGGGTACTATATTGGTACAATCTTTATTTAGCTATTAATGAGTA
GTATGACATTATTTATAGGGTTTGTATCTGTTATAGCTATTTTATTTTTAGCCATTAATTTTATATTT
GCTCCTCATAATCCTTATCAAGAAAAATATAGTATTTTCGAGTGTGGTTTCCATAGTTTTTTAGGG
CAAAATAGAACACAATTCGGTATAAAATTCTTTATTTTTGCTTTAGTTTATTTACTTTTAGATTTAG

AAATATTATTAACTTTCCCTTTCGCTCTTAGTGAGTATGTTAATGGTATTTATGGTCTTTTAGTTAC
TTTAATTTTTATAGCTATAATAACTATAGGATTTATATTTGAATTAGGTAAAAGCGCTCTTAAAATA
GACAGCAGACAAAAATTATATATACCTAAATTGAACGTTAATTACCATACAGAGTATGTTGGAAT
AGGTAAGGTTTCTAAGTAAAGTTATAGAGGCAGAAAACCAAAAAACCTACCAAAGGGTAGCTA
ATGGGAAGCTATTAAAAATAAAAGATGATAACCTATATATAGTATAGTTACTATATATTTACTATA
ACTAGGATTATTATATATATATATATTATCTTATTGTATATTAAGATTATTATTATTATAAGGTATA
ATTAATATAGTATCTTATTGTATAAGAATATAATATATTAACCTATAATTAATTTTATTTTTTAATTT
TTAATTATAATTTTTTTTTATATCTAGATGCTTACACATCTACAGATGTAGAAGAGAACAAAATAT
TGTTGTTATAGTAGTGGATGATATAGAAAAATATTTTTATATTATTTATTTTTGTTAGGTAGCTTTT
GAAGTGTTTGATAGAGAGGATATATGGACGGTAGGAGGGTATTCATTTTAATGAACAGTGGATA
GTTTAAATTAACCTAGTTATAGTTTATGAATTTAAATTATAATTATATTAATGTAGGTTATGATAGA
ATTATGTATTGATCCTTAAGAGTTAAGAGAGATACGCCCACGTATAATACATATTGGTTTAGGGT
TGAGTATATATACTTAAGAGTTAAGAATATATATATACAATATATAATAAATATAGTAATATATTA
ATGGTATGGACTTAACCAGGTTTATATATTATCATTTGATAAACATTAATTATAATTTTTCTATTTA
TTATTATTATTATACTGTGAGATTAATAATTATTAAAATATAATATTACATAGTACGTGATATTTGG
GGATTTTATCTTATTAATTGGCAATTAATGATTCTAATCAAATTTTATTCTCTTTAGTTTAATGGTA
GAACAATGATCTTCTAATTCATTGGTTTTAGTTCGATTCTAAAAAGAGATGAGTAAATAATTTTCT
AGATCAGAAATACTACTTTTAACTACAAAAAGCTTACGCTTTTTAACATTTTTTTGATAAATAACA
ACTATTGTTAATATTTGGCTGTCTATTGGTTTAACTAACAATTACAAAATTTTTTCAATTTTTATAAT
ATAAATTTAAATGAGAATATTAAAAAGTCATTCATTATTAAAATTAGTGAATTCTTACCTTATCGA
TGCGTCACAACCAAGTAACATTAGTTACTTGTGAAATTTTGGTTCATTATTAGCTGTTTGTTTAAT
AGTACAAATTATTACCGGTATTACATTAGCTATGCATTATAGTCCTAGTGTAATGGAAGCTTTTAA
CTCAATAGAGCATATAATGAGAGATGTTAATAACGGGTGATTAGTTCGTTATCTACATAGTAATA
CAGCTTCTGCTTTCTTTTTCTTAGTGTATTTACACATAGGAAGAGGTATATATTACGGATCATATA
GAGCTCCTCGTACTTTAGTTTGAGCTATTGGTACTGTTATATTAATATTAATGATGGCTATCGGTT
TCCTAGGTTATGTTTTACCTTATGGACAGATGTCATTATGAGGTGCTACAGTTATTACTAATCTTA
TTAGTGCTATACCTTGAATAGGGCAAGATATTGTTGAATTCATTTGAGGTGGTTTTTCTGTTAATA
ATGCCACTTTAAACAGATTTTTTGCATTACATTTTGTATTGCCTTTTGTATTAGCTGCTTTAGTTTTA
ATGCACTTAATTGCACTTCATGATACTGCTGGTTCAAGCAATCCTCTTGGTGTTTCAGGTAATTAC
GATAGAATTACATTTGCTCCATATTTTTTATTTAAAGATTTAATTACTATTTTTATATTTATTTTTGTA
TTAAGTGCTTTTGTATTCTTTATGCCTAATGTTTTAGGGGATAGTGATAATTATATTATGGCTAATC
CTATGCAAACTCCTGCTGCTATTGTACCTGAATGATACTTATTACCTTTCTATGCTATTTTAAGATC
TATACCTAATAAATTATTAGGTGTTATAGCGATGTTTAGTGCTATTTTAGCTATTATGTTATTACCT
GTTACAGATTTAGGTAGATCTAGAGGTTTACAATTTAGACCATTTAGTAAAATAGCTTTCTGAGTT
TTTGTTGCTAATTTCTTAGTTTTAATGCAATTAGGTGCTAAACACGTTGAAGATCCATTTATATTAT
TAGGTCAATTAAGTACTGTATTATACTTTAGTTATTTTGTTGCTATATTACCTTTAGCTAGTTACTT
AGATAATAGTTTAACTGATTTATCTAATAAATCTGAATTATTTTTAAATAAAACTAACTAAATATAT
TAAGATTATTATTTAATATATTTTCTATTTAAGATACTATTAATTTAGTATTTTGGGTTTTCAGTTTA
TAATTTATATTATATTATGCATTACCCTCCACCTTGCTTTGTAGTAAGCTAATCTGTTATTTCCTTTA
GTTTAATGGTAGAACAATGATCTTCTAATTCATTGGTTTTAGTTCGAATCTAAAAAGGAAATAAG
AAATATATTCTTATTATTACTTATATAATAATTATTTCTTAAAAATATACATTTTGCATTATAGCCGT
TTAGCTGTATTAAAATGTAAAATGATATAAAATAGAATAAATATTTAAATTATTCCTATGTTATATT
ATCCTATATTGCAACCATTATCAGAAGTTGTATTAATACTTGTACCTGCCTTATTAGCTGTAGCTT
ATGTTACAGTTGCTGAAAGAAAAACTATGGCTAGTATGCAAAGAAGATTAGGTCCTAATGCTGT

AGGTTACTATGGACTATTGCAAGCATTTGCTGATGCCTTAAAACTTTTATTAAAAGAATATGTAG
CTCCTACACAATCTAATATTGTTCTTTTCTTTTTAGGTCCTGTAATAACTTTAATTTTTGCATTATTA
GGTTACGCTGTTATACCCTATGGTCCTGGTTCAGGGATAAGCGACATGAATTTAGGTATATTTTA
CATGTTAGCTGTGTCATCTTTAGCTACATACGGTATTCTATTAGCTGGTTGAAGTGCGAATAGTA
AATACGCTTTTCTAGGTTCTCTTAGAAGTACAGCTCAATTAATTAGTTATGAATTAATATTAAGTT
CAGCTATATTAATAGTAATTATGATAACAGGAAATTTAAATTTAACTGTTTGTACTGAATCTCAAA
GAGCTATTTGATTTATACTACCTTTATTTCCTGTGTTTATAATATTTTTCATAGGATCTATAGCTGA
GACAAATAGAGCTCCTTTTGATTTAGCCGAGGCTAACCTGCTAATCTGGTTTGGTCTGGTTATAT
GTCACAAATTGCTAGGAAACCTTTTTATTTTAAAAACAAAAGACAATTAGCAGGAAACTTAATTT
AACCTAATTAAAAATATTAGATAATTAAACTCTTCATAGACTAAACGTGACAATTTAATATATATA
TATATTTATTTATATATATGATTAAATAAGATATAGTCAATCATCGGTGTGAATCGACTTAAAAAA
AAAAGCACATGGGTTAAACCCATCTCCCCTTATTAGGGGAATCAGAACTTGTTAGTGGGTTCAT
GACAGAGCATGCTGCCGTAGTTTTCGTATTCTTCTTTTTAGCTGAGTACGGTAGTATTGTACTAAT
GTGTATTTTAACTAGTATATTATTTATTGGTGGTTACTTATTATTTGAAATATCCTATGTTTTTACTG
TGGTAAATTATATTTTCTTTGAATTATTCTTTATAGACTGAGTAACATTTGTAGAGGTACAATCTTT
ATACACTGATTTTTTAAATAATTCTATCATTGAAGGATTATTATATGGGTTTAATCTAGGATTAAA
AAGTTCTTTAATGATATTCACATTTATTTGAGCTAGAGCATCCTTCCCTAGAATACGATTTGATCA
ACTAATGGGCTTCTGTTGAACAGTTTTATTACCTATTAATTTTGCAATTATTATATTAGTACCTTGT
GTTTTATATAGTTTTAACTTATTACCTGTAAATATACCATTGTTCTAGCTCACACACCCGCCCGCC
CTACTGCCACAAGGCTACAGTACATATGAGGAGGGGAACTAAAGATCTAGAACTATCCTAGTTA
ATAATTACACTTAATAGTATACTAAATAGACCATCTATCATACTCGAGAATAGTGATAGTGTAAT
TATACACTATTTTAACTGTATTCTACACTATTAGCATATTATTATCTTTATATGATAATAATTTTATA
ACTTAATTTATTAGGTTACATTAACAAAAAAATTCGTTAACTTTTTTCACCACTTTTTAATACAAAAT
ACGAATTTTA
>YN735
ATAACAATTCTAAAGAACATAAAGAGTTATTAGATAAAAATAATTCACCTATACAGTTAATAAAT
CAACTTAAAGGGTATTTTTTCATAAATCCTTTATTAGCTTTAAGTTTAGCTATTACTATTTTCTCTTT
TGCAGGTATTCCTCCTCTTGTAGGGTTCTTTGCTAAACAGATGGTATTAAGCGCGGCTATTGATC
AAGGTTATATCTTTTTATCTTTAGTTGCAATATTAACTAGTGTTATAGGAGGGGTTTATTATTTAAA
TATAATTAAAGAAATGTTCTTTTATTCACCTGACTATAAATTAAACGAAGAAATTAAAAATAATAC
TATTAATGGTCAAATTATTAATAGAAACAATAAAATATTAAATGTTGAATTTAATTATACAAATGT
AGTTATGTCTAGTTCTGTGGCAATAACTATTTCTACTATTACATTAGTAGTTTTATTATTCATGTTT
ATGAATAAAGAATGATTAAGTCTGGGTACTATATTGGTACAATCTTTATTTAGCTATTAATGAGTA
GTATGACATTATTTATAGGGTTTGTATCTGTTATAGCTATTTTATTTTTAGCCATTAATTTTATATTT
GCTCCTCATAATCCTTATCAAGAAAAATATAGTATTTTCGAGTGTGGTTTCCATAGTTTTTTAGGG
CAAAATAGAACACAATTCGGTATAAAATTCTTTATTTTTGCTTTAGTTTATTTACTTTTAGATTTAG
AAATATTATTAACTTTCCCTTTCGCTCTTAGTGAGTATGTTAATGGTATTTATGGTCTTTTAGTTAC
TTTAATTTTTATAGCTATAATAACTATAGGATTTATATTTGAATTAGGTAAAAGCGCTCTTAAAATA
GACAGCAGACAAAAATTATATATACCTAAATTGAACGTTAATTACCATACAGAGTATGTTGGAAT
AGGTAAGGTTTCTAAGTAAAGTTATAGAGGCAGAAAACCAAAAAACCTACCAAAGGGTAGCTA
ATGGGAAGCTATTAAAAATAAAAGATGATAACCTATATATAGTATAGTTACTATATATTTACTATA
ACTAGGATTATTATATATATATATATTATCTTATTGTATATTAAGATTATTATTATTATAAGGTATA
ATTAATATAGTATCTTATTGTATAAGAATATAATATATTAACCTATAATTAATTTTATTTTTTAATTT
TTAATTATAATTTTTTTTTATATCTAGATGCTTACACATCTACAGATGTAGAAGAGAACAAAATAT

TGTTGTTATAGTAGTGGATGATATAGAAAAATATTTTTATATTATTTATTTTTGTTAGGTAGCTTTT
GAAGTGTTTGATAGAGAGGATATATGGACGGTAGGAGGGTATTCATTTTAATGAACAGTGGATA
GTTTAAATTAACCTAGTTATAGTTTATGAATTTAAATTATAATTATATTAATGTAGGTTATGATAGA
ATTATGTATTGATCCTTAAGAGTTAAGAGAGATACGCCCACGTATAATACATATTGGTTTAGGGT
TGAGTATATATACTTAAGAGTTAAGAATATATATATACAATATATAATAAATATAGTAATATATTA
ATGGTATGGACTTAACCAGGTTTATATATTATCATTTGATAAACATTAATTATAATTTTTCTATTTA
TTATTATTATTATACTGTGAGATTAATAATTATTAAAATATAATATTACATAGTACGTGATATTTGG
GGATTTTATCTTATTAATTGGCAATTAATGATTCTAATCAAATTTTATTCTCTTTAGTTTAATGGTA
GAACAATGATCTTCTAATTCATTGGTTTTAGTTCGATTCTAAAAAGAGATGAGTAAATAATTTTCT
AGATCAGAAATACTACTTTTAACTACAAAAAGCTTACGCTTTTTAACATTTTTTTGATAAATAACA
ACTATTGTTAATATTTGGCTGTCTATTGGTTTAACTAACAATTACAAAATTTTTTCAATTTTTATAAT
ATAAATTTAAATGAGAATATTAAAAAGTCATTCATTATTAAAATTAGTGAATTCTTACCTTATCGA
TGCGTCACAACCAAGTAACATTAGTTACTTGTGAAATTTTGGTTCATTATTAGCTGTTTGTTTAAT
AGTACAAATTATTACCGGTATTACATTAGCTATGCATTATAGTCCTAGTGTAATGGAAGCTTTTAA
CTCAATAGAGCATATAATGAGAGATGTTAATAACGGGTGATTAGTTCGTTATCTACATAGTAATA
CAGCTTCTGCTTTCTTTTTCTTAGTGTATTTACACATAGGAAGAGGTATATATTACGGATCATATA
GAGCTCCTCGTACTTTAGTTTGAGCTATTGGTACTGTTATATTAATATTAATGATGGCTATCGGTT
TCCTAGGTTATGTTTTACCTTATGGACAGATGTCATTATGAGGTGCTACAGTTATTACTAATCTTA
TTAGTGCTATACCTTGAATAGGGCAAGATATTGTTGAATTCATTTGAGGTGGTTTTTCTGTTAATA
ATGCCACTTTAAACAGATTTTTTGCATTACATTTTGTATTGCCTTTTGTATTAGCTGCTTTAGTTTTA
ATGCACTTAATTGCACTTCATGATACTGCTGGTTCAAGCAATCCTCTTGGTGTTTCAGGTAATTAC
GATAGAATTACATTTGCTCCATATTTTTTATTTAAAGATTTAATTACTATTTTTATATTTATTTTTGTA
TTAAGTGCTTTTGTATTCTTTATGCCTAATGTTTTAGGGGATAGTGATAATTATATTATGGCTAATC
CTATGCAAACTCCTGCTGCTATTGTACCTGAATGATACTTATTACCTTTCTATGCTATTTTAAGATC
TATACCTAATAAATTATTAGGTGTTATAGCGATGTTTAGTGCTATTTTAGCTATTATGTTATTACCT
GTTACAGATTTAGGTAGATCTAGAGGTTTACAATTTAGACCATTTAGTAAAATAGCTTTCTGAGTT
TTTGTTGCTAATTTCTTAGTTTTAATGCAATTAGGTGCTAAACACGTTGAAGATCCATTTATATTAT
TAGGTCAATTAAGTACTGTATTATACTTTAGTTATTTTGTTGCTATATTACCTTTAGCTAGTTACTT
AGATAATAGTTTAACTGATTTATCTAATAAATCTGAATTATTTTTAAATAAAACTAACTAAATATAT
TAAGATTATTATTTAATATATTTTCTATTTAAGATACTATTAATTTAGTATTTTGGGTTTTCAGTTTA
TAATTTATATTATATTATGCATTACCCTCCACCTTGCTTTGTAGTAAGCTAATCTGTTATTTCCTTTA
GTTTAATGGTAGAACAATGATCTTCTAATTCATTGGTTTTAGTTCGAATCTAAAAAGGAAATAAG
AAATATATTCTTATTATTACTTATATAATAATTATTTCTTAAAAATATACATTTTGCATTATAGCCGT
TTAGCTGTATTAAAATGTAAAATGATATAAAATAGAATAAATATTTAAATTATTCCTATGTTATATT
ATCCTATATTGCAACCATTATCAGAAGTTGTATTAATACTTGTACCTGCCTTATTAGCTGTAGCTT
ATGTTACAGTTGCTGAAAGAAAAACTATGGCTAGTATGCAAAGAAGATTAGGTCCTAATGCTGT
AGGTTACTATGGACTATTGCAAGCATTTGCTGATGCCTTAAAACTTTTATTAAAAGAATATGTAG
CTCCTACACAATCTAATATTGTTCTTTTCTTTTTAGGTCCTGTAATAACTTTAATTTTTGCATTATTA
GGTTACGCTGTTATACCCTATGGTCCTGGTTCAGGGATAAGCGACATGAATTTAGGTATATTTTA
CATGTTAGCTGTGTCATCTTTAGCTACATACGGTATTCTATTAGCTGGTTGAAGTGCGAATAGTA
AATACGCTTTTCTAGGTTCTCTTAGAAGTACAGCTCAATTAATTAGTTATGAATTAATATTAAGTT
CAGCTATATTAATAGTAATTATGATAACAGGAAATTTAAATTTAACTGTTTGTACTGAATCTCAAA
GAGCTATTTGATTTATACTACCTTTATTTCCTGTGTTTATAATATTTTTCATAGGATCTATAGCTGA
GACAAATAGAGCTCCTTTTGATTTAGCCGAGGCTAACCTGCTAATCTGGTTTGGTCTGGTTATAT

GTCACAAATTGCTAGGAAACCTTTTTATTTTAAAAACAAAAGACAATTAGCAGGAAACTTAATTT
AACCTAATTAAAAATATTAGATAATTAAACTCTTCATAGACTAAACGTGACAATTTAATATATATA
TATATTTATTTATATATATGATTAAATAAGATATAGTCAATCATCGGTGTGAATCGACTTAAAAAA
AAAAGCACATGGGTTAAACCCATCTCCCCTTATTAGGGGAATCAGAACTTGTTAGTGGGTTCAT
GACAGAGCATGCTGCCGTAGTTTTCGTATTCTTCTTTTTAGCTGAGTACGGTAGTATTGTACTAAT
GTGTATTTTAACTAGTATATTATTTATTGGTGGTTACTTATTATTTGAAATATCCTATGTTTTTACTG
TGGTAAATTATATTTTCTTTGAATTATTCTTTATAGACTGAGTAACATTTGTAGAGGTACAATCTTT
ATACACTGATTTTTTAAATAATTCTATCATTGAAGGATTATTATATGGGTTTAATCTAGGATTAAA
AAGTTCTTTAATGATATTCACATTTATTTGAGCTAGAGCATCCTTCCCTAGAATACGATTTGATCA
ACTAATGGGCTTCTGTTGAACAGTTTTATTACCTATTAATTTTGCAATTATTATATTAGTACCTTGT
GTTTTATATAGTTTTAACTTATTACCTGTAAATATACCATTGTTCTAGCTCACACACCCGCCCGCC
CTACTGCCACAAGGCTACAGTACATATGAGGAGGGGAACTAAAGATCTAGAACTATCCTAGTTA
ATAATTACACTTAATAGTATACTAAATAGACCATCTATCATACTCGAGAATAGTGATAGTGTAAT
TATACACTATTTTAACTGTATTCTACACTATTAGCATATTATTATCTTTATATGATAATAATTTTATA
ACTTAATTTATTAGGTTACATTAACAAAAAATTCGTTAACTTTTTTCACCACTTTTTAATACAAAAT
ACGAATTAAA
>YN745
ATAACAATTCTAAAGAACATAAAGAGTTATTAGATAAAAATAATTCACCTATACAGTTAATAAAT
CAACTTAAAGGGTATTTTTTCATAAATCCTTTATTAGCTTTAAGTTTAGCTATTACTATTTTCTCTTT
TGCAGGTATTCCTCCTCTTGTAGGGTTCTTTGCTAAACAGATGGTATTAAGCGCGGCTATTGATC
AAGGTTATATCTTTTTATCTTTAGTTGCAATATTAACTAGTGTTATAGGAGGGGTTTATTATTTAAA
TATAATTAAAGAAATGTTCTTTTATTCACCTGACTATAAATTAAACGAAGAAATTAAAAATAATAC
TATTAATGGTCAAATTATTAATAGAAACAATAAAATATTAAATGTTGAATTTAATTATACAAATGT
AGTTATGTCTAGTTCTGTGGCAATAACTATTTCTACTATTACATTAGTAGTTTTATTATTCATGTTT
ATGAATAAAGAATGATTAAGTCTGGGTACTATATTGGTACAATCTTTATTTAGCTATTAATGAGTA
GTATGACATTATTTATAGGGTTTGTATCTGTTATAGCTATTTTATTTTTAGCCATTAATTTTATATTT
GCTCCTCATAATCCTTATCAAGAAAAATATAGTATTTTCGAGTGTGGTTTCCATAGTTTTTTAGGG
CAAAATAGAACACAATTCGGTATAAAATTCTTTATTTTTGCTTTAGTTTATTTACTTTTAGATTTAG
AAATATTATTAACTTTCCCTTTCGCTCTTAGTGAGTATGTTAATGGTATTTATGGTCTTTTAGTTAC
TTTAATTTTTATAGCTATAATAACTATAGGATTTATATTTGAATTAGGTAAAAGCGCTCTTAAAATA
GACAGCAGACAAAAATTATATATACCTAAATTGAACGTTAATTACCATACAGAGTATGTTGGAAT
AGGTAAGGTTTCTAAGTAAAGTTATAGAGGCAGAAAACCAAAAAACCTACCAAAGGGTAGCTA
ATGGGAAGCTATTAAAAATAAAAGATGATAACCTATATATAGTATAGTTACTATATATTTACTATA
ACTAGGATTATTATATATATATATATTATCTTATTGTATATTAAGATTATTATTATTATAAGGTATA
ATTAATATAGTATCTTATTGTATAAGAATATAATATATTAACCTATAATTAATTTTATTTTTTAATTT
TTAATTATAATTTTTTTTTATATCTAGATGCTTACACATCTACAGATGTAGAAGAGAACAAAATAT
TGTTGTTATAGTAGTGGATGATATAGAAAAATATTTTTATATTATTTATTTTTGTTAGGTAGCTTTT
GAAGTGTTTGATAGAGAGGATATATGGACGGTAGGAGGGTATTCATTTTAATGAACAGTGGATA
GTTTAAATTAACCTAGTTATAGTTTATGAATTTAAATTATAATTATATTAATGTAGGTTATGATAGA
ATTATGTATTGATCCTTAAGAGTTAAGAGAGATACGCCCACGTATAATACATATTGGTTTAGGGT
TGAGTATATATACTTAAGAGTTAAGAATATATATATACAATATATAATAAATATAGTAATATATTA
ATGGTATGGACTTAACCAGGTTTATATATTATCATTTGATAAACATTAATTATAATTTTTCTATTTA
TTATTATTATTATACTGTGAGATTAATAATTATTAAAATATAATATTACATAGTACGTGATATTTGG
GGATTTTATCTTATTAATTGGCAATTAATGATTCTAATCAAATTTTATTCTCTTTAGTTTAATGGTA

GAACAATGATCTTCTAATTCATTGGTTTTAGTTCGATTCTAAAAAGAGATGAGTAAATAATTTTCT
AGATCAGAAATACTACTTTTAACTACAAAAAGCTTACGCTTTTTAACATTTTTTTGATAAATAACA
ACTATTGTTAATATTTGGCTGTCTATTGGTTTAACTAACAATTACAAAATTTTTTCAATTTTTATAAT
ATAAATTTAAATGAGAATATTAAAAAGTCATTCATTATTAAAATTAGTGAATTCTTACCTTATCGA
TGCGTCACAACCAAGTAACATTAGTTACTTGTGAAATTTTGGTTCATTATTAGCTGTTTGTTTAAT
AGTACAAATTATTACCGGTATTACATTAGCTATGCATTATAGTCCTAGTGTAATGGAAGCTTTTAA
CTCAATAGAGCATATAATGAGAGATGTTAATAACGGGTGATTAGTTCGTTATCTACATAGTAATA
CAGCTTCTGCTTTCTTTTTCTTAGTGTATTTACACATAGGAAGAGGTATATATTACGGATCATATA
GAGCTCCTCGTACTTTAGTTTGAGCTATTGGTACTGTTATATTAATATTAATGATGGCTATCGGTT
TCCTAGGTTATGTTTTACCTTATGGACAGATGTCATTATGAGGTGCTACAGTTATTACTAATCTTA
TTAGTGCTATACCTTGAATAGGGCAAGATATTGTTGAATTCATTTGAGGTGGTTTTTCTGTTAATA
ATGCCACTTTAAACAGATTTTTTGCATTACATTTTGTATTGCCTTTTGTATTAGCTGCTTTAGTTTTA
ATGCACTTAATTGCACTTCATGATACTGCTGGTTCAAGCAATCCTCTTGGTGTTTCAGGTAATTAC
GATAGAATTACATTTGCTCCATATTTTTTATTTAAAGATTTAATTACTATTTTTATATTTATTTTTGTA
TTAAGTGCTTTTGTATTCTTTATGCCTAATGTTTTAGGGGATAGTGATAATTATATTATGGCTAATC
CTATGCAAACTCCTGCTGCTATTGTACCTGAATGATACTTATTACCTTTCTATGCTATTTTAAGATC
TATACCTAATAAATTATTAGGTGTTATAGCGATGTTTAGTGCTATTTTAGCTATTATGTTATTACCT
GTTACAGATTTAGGTAGATCTAGAGGTTTACAATTTAGACCATTTAGTAAAATAGCTTTCTGAGTT
TTTGTTGCTAATTTCTTAGTTTTAATGCAATTAGGTGCTAAACACGTTGAAGATCCATTTATATTAT
TAGGTCAATTAAGTACTGTATTATACTTTAGTTATTTTGTTGCTATATTACCTTTAGCTAGTTACTT
AGATAATAGTTTAACTGATTTATCTAATAAATCTGAATTATTTTTAAATAAAACTAACTAAATATAT
TAAGATTATTATTTAATATATTTTCTATTTAAGATACTATTAATTTAGTATTTTGGGTTTTCAGTTTA
TAATTTATATTATATTATGCATTACCCTCCACCTTGCTTTGTAGTAAGCTAATCTGTTATTTCCTTTA
GTTTAATGGTAGAACAATGATCTTCTAATTCATTGGTTTTAGTTCGAATCTAAAAAGGAAATAAG
AAATATATTCTTATTATTACTTATATAATAATTATTTCTTAAAAATATACATTTTGCATTATAGCCGT
TTAGCTGTATTAAAATGTAAAATGATATAAAATAGAATAAATATTTAAATTATTCCTATGTTATATT
ATCCTATATTGCAACCATTATCAGAAGTTGTATTAATACTTGTACCTGCCTTATTAGCTGTAGCTT
ATGTTACAGTTGCTGAAAGAAAAACTATGGCTAGTATGCAAAGAAGATTAGGTCCTAATGCTGT
AGGTTACTATGGACTATTGCAAGCATTTGCTGATGCCTTAAAACTTTTATTAAAAGAATATGTAG
CTCCTACACAATCTAATATTGTTCTTTTCTTTTTAGGTCCTGTAATAACTTTAATTTTTGCATTATTA
GGTTACGCTGTTATACCCTATGGTCCTGGTTCAGGGATAAGCGACATGAATTTAGGTATATTTTA
CATGTTAGCTGTGTCATCTTTAGCTACATACGGTATTCTATTAGCTGGTTGAAGTGCGAATAGTA
AATACGCTTTTCTAGGTTCTCTTAGAAGTACAGCTCAATTAATTAGTTATGAATTAATATTAAGTT
CAGCTATATTAATAGTAATTATGATAACAGGAAATTTAAATTTAACTGTTTGTACTGAATCTCAAA
GAGCTATTTGATTTATACTACCTTTATTTCCTGTGTTTATAATATTTTTCATAGGATCTATAGCTGA
GACAAATAGAGCTCCTTTTGATTTAGCCGAGGCTAACCTGCTAATCTGGTTTGGTCTGGTTATAT
GTCACAAATTGCTAGGAAACCTTTTTATTTTAAAAACAAAAGACAATTAGCAGGAAACTTAATTT
AACCTAATTAAAAATATTAGATAATTAAACTCTTCATAGACTAAACGTGACAATTTAATATATATA
TATATTTATTTATATATATGATTAAATAAGATATAGTCAATCATCGGTGTGAATCGACTTAAAAAA
AAAAGCACATGGGTTAAACCCATCTCCCCTTATTAGGGGAATCAGAACTTGTTAGTGGGTTCAT
GACAGAGCATGCTGCCGTAGTTTTCGTATTCTTCTTTTTAGCTGAGTACGGTAGTATTGTACTAAT
GTGTATTTTAACTAGTATATTATTTATTGGTGGTTACTTATTATTTGAAATATCCTATGTTTTTACTG
TGGTAAATTATATTTTCTTTGAATTATTCTTTATAGACTGAGTAACATTTGTAGAGGTACAATCTTT
ATACACTGATTTTTTAAATAATTCTATCATTGAAGGATTATTATATGGGTTTAATCTAGGATTAAA

AAGTTCTTTAATGATATTCACATTTATTTGAGCTAGAGCATCCTTCCCTAGAATACGATTTGATCA
ACTAATGGGCTTCTGTTGAACAGTTTTATTACCTATTAATTTTGCAATTATTATATTAGTACCTTGT
GTTTTATATATAGTTTTAACTTATTACCTGTAAATATACCATTGTTCTAGCTCACACACCCGCCCGCC
CTACTGCCACAAGGCTACAGTACATATGAGGAGGGGAACTAAAGATCTAGAACTATCCTAGTTA
ATAATTACACTTAATAGTATACTAAATAGACCATCTATCATACTCGAGAATAGTGATAGTGTAAT
TATACACTATTTTAACTGTATTCTACACTATTAGCATATTATTATCTTTATATGATAATAATTTTATA
ACTTAATTTATTAGGTTACATTAACAAAAAATTCGTTAACTTTTTTCACCACTTTTTAATACAAAAT
ACGAATTTTA
>Z-2
ATAACAATTCTAAAGAACATAAAGAGTTATTAGATAAAAATAATTCACCTATACAGTTAATAAAT
CAACTTAAAGGGTATTTTTTCATAAATCCTTTATTAGCTTTAAGTTTAGCTATTACTATTTTCTCTTT
TGCAGGTATTCCTCCTCTTGTAGGGTTCTTTGCTAAACAGATGGTATTAAGCGCGGCTATTGATC
AAGGTTATATCTTTTTATCTTTAGTTGCAATATTAACTAGTGTTATAGGAGGGGTTTATTATTTAAA
TATAATTAAAGAAATGTTCTTTTATTCACCTGACTATAAATTAAACGAAGAAATTAAAAATAATAC
TATTAATGGTCAAATTATTAATAGAAACAATAAAATATTAAATGTTGAATTTAATTATACAAATGT
AGTTATGTCTAGTTCTGTGGCAATAACTATTTCTACTATTACATTAGTAGTTTTATTATTCATGTTT
ATGAATAAAGAATGATTAAGTCTGGGTACTATATTGGTACAATCTTTATTTAGCTATTAATGAGTA
GTATGACATTATTTATAGGGTTTGTATCTGTTATAGCTATTTTATTTTTAGCCATTAATTTTATATTT
GCTCCTCATAATCCTTATCAAGAAAAATATAGTATTTTCGAGTGTGGTTTCCATAGTTTTTTAGGG
CAAAATAGAACACAATTCGGTATAAAATTCTTTATTTTTGCTTTAGTTTATTTACTTTTAGATTTAG
AAATATTATTAACTTTCCCTTTCGCTCTTAGTGAGTATGTTAATGGTATTTATGGTCTTTTAGTTAC
TTTAATTTTTATAGCTATAATAACTATAGGATTTATATTTGAATTAGGTAAAAGCGCTCTTAAAATA
GACAGCAGACAAAAATTATATATACCTAAATTGAACGTTAATTACCATACAGAGTATGTTGGAAT
AGGTAAGGTTTCTAAGTAAAGTTATAGAGGCAGAAAACCAAAAAACCTACCAAAGGGTAGCTA
ATGGGAAGCTATTAAAAATAAAAGATGATAACCTATATATAGTATAGTTACTATATATTTACTATA
ACTAGGATTATTATATATATATATATATTATCTTATTGTATATTAAGATTATTATTATTATAAGGTATA
ATTAATATAGTATCTTATTGTATAAGAATATAATATATTAACCTATAATTAATTTTATTTTTTAATTT
TTAATTATAATTTTTTTTTATATCTAGATGCTTACACATCTACAGATGTAGAAGAGAACAAAATAT
TGTTGTTATAGTAGTGGATGATATAGAAAAATATTTTTATATTATTTATTTTTGTTAGGTAGCTTTT
GAAGTGTTTGATAGAGAGGATATATGGACGGTAGGAGGGTATTCATTTTAATGAACAGTGGATA
GTTTAAATTAACCTAGTTATAGTTTATGAATTTAAATTATAATTATATTAATGTAGGTTATGATAGA
ATTATGTATTGATCCTTAAGAGTTAAGAGAGATACGCCCACGTATAATACATATTGGTTTAGGGT
TGAGTATATATACTTAAGAGTTAAGAATATATATATACAATATATAATAAATATAGTAATATATTA
ATGGTATGGACTTAACCAGGTTTATATATTATCATTTGATAAACATTAATTATAATTTTTCTATTTA
TTATTATTATTATACTGTGAGATTAATAATTATTAAAATATAATATTACATAGTACGTGATATTTGG
GGATTTTATCTTATTAATTGGCAATTAATGATTCTAATCAAATTTTATTCTCTTTAGTTTAATGGTA
GAACAATGATCTTCTAATTCATTGGTTTTAGTTCGATTCTAAAAAGAGATGAGTAAATAATTTTCT
AGATCAGAAATACTACTTTTAACTACAAAAAGCTTACGCTTTTTAACATTTTTTTGATAAATAACA
ACTATTGTTAATATTTGGCTGTCTATTGGTTTAACTAACAATTACAAAATTTTTTCAATTTTTATAAT
ATAAATTTAAATGAGAATATTAAAAAGTCATTCATTATTAAAATTAGTGAATTCTTACCTTATCGA
TGCGTCACAACCAAGTAACATTAGTTACTTGTGAAATTTTGGTTCATTATTAGCTGTTTGTTTAAT
AGTACAAATTATTACCGGTATTACATTAGCTATGCATTATAGTCCTAGTGTAATGGAAGCTTTTAA
CTCAATAGAGCATATAATGAGAGATGTTAATAACGGGTGATTAGTTCGTTATCTACATAGTAATA
CAGCTTCTGCTTTCTTTTTCTTAGTGTATTTACACATAGGAAGAGGTATATATTACGGATCATATA

GAGCTCCTCGTACTTTAGTTTGAGCTATTGGTACTGTTATATTAATATTAATGATGGCTATCGGTT
TCCTAGGTTATGTTTTACCTTATGGACAGATGTCATTATGAGGTGCTACAGTTATTACTAATCTTA
TTAGTGCTATACCTTGAATAGGGCAAGATATTGTTGAATTCATTTGAGGTGGTTTTTCTGTTAATA
ATGCCACTTTAAACAGATTTTTTGCATTACATTTTGTATTGCCTTTTGTATTAGCTGCTTTAGTTTTA
ATGCACTTAATTGCACTTCATGATACTGCTGGTTCAAGCAATCCTCTTGGTGTTTCAGGTAATTAC
GATAGAATTACATTTGCTCCATATTTTTTATTTAAAGATTTAATTACTATTTTTATATTTATTTTTGTA
TTAAGTGCTTTTGTATTCTTTATGCCTAATGTTTTAGGGGATAGTGATAATTATATTATGGCTAATC
CTATGCAAACTCCTGCTGCTATTGTACCTGAATGATACTTATTACCTTTCTATGCTATTTTAAGATC
TATACCTAATAAATTATTAGGTGTTATAGCGATGTTTAGTGCTATTTTAGCTATTATGTTATTACCT
GTTACAGATTTAGGTAGATCTAGAGGTTTACAATTTAGACCATTTAGTAAAATAGCTTTCTGAGTT
TTTGTTGCTAATTTCTTAGTTTTAATGCAATTAGGTGCTAAACACGTTGAAGATCCATTTATATTAT
TAGGTCAATTAAGTACTGTATTATACTTTAGTTATTTTGTTGCTATATTACCTTTAGCTAGTTACTT
AGATAATAGTTTAACTGATTTATCTAATAAATCTGAATTATTTTTAAATAAAACTAACTAAATATAT
TAAGATTATTATTTAATATATTTTCTATTTAAGATACTATTAATTTAGTATTTTGGGTTTTCAGTTTA
TAATTTATATTATATTATGCATTACCCTCCACCTTGCTTTGTAGTAAGCTAATCTGTTATTTCCTTTA
GTTTAATGGTAGAACAATGATCTTCTAATTCATTGGTTTTAGTTCGAATCTAAAAAGGAAATAAG
AAATATATTCTTATTATTACTTATATAATAATTATTTCTTAAAAATATACATTTTGCATTATAGCCGT
TTAGCTGTATTAAAATGTAAAATGATATAAAATAGAATAAATATTTAAATTATTCCTATGTTATATT
ATCCTATATTGCAACCATTATCAGAAGTTGTATTAATACTTGTACCTGCCTTATTAGCTGTAGCTT
ATGTTACAGTTGCTGAAAGAAAAACTATGGCTAGTATGCAAAGAAGATTAGGTCCTAATGCTGT
AGGTTACTATGGACTATTGCAAGCATTTGCTGATGCCTTAAAACTTTTATTAAAAGAATATGTAG
CTCCTACACAATCTAATATTGTTCTTTTCTTTTTAGGTCCTGTAATAACTTTAATTTTTGCATTATTA
GGTTACGCTGTTATACCCTATGGTCCTGGTTCAGGGATAAGCGACATGAATTTAGGTATATTTTA
CATGTTAGCTGTGTCATCTTTAGCTACATACGGTATTCTATTAGCTGGTTGAAGTGCGAATAGTA
AATACGCTTTTCTAGGTTCTCTTAGAAGTACAGCTCAATTAATTAGTTATGAATTAATATTAAGTT
CAGCTATATTAATAGTAATTATGATAACAGGAAATTTAAATTTAACTGTTTGTACTGAATCTCAAA
GAGCTATTTGATTTATACTACCTTTATTTCCTGTGTTTATAATATTTTTCATAGGATCTATAGCTGA
GACAAATAGAGCTCCTTTTGATTTAGCCGAGGCTAACCTGCTAATCTGGTTTGGTCTGGTTATAT
GTCACAAATTGCTAGGAAACCTTTTTATTTTAAAAACAAAAGACAATTAGCAGGAAACTTAATTT
AACCTAATTAAAAATATTAGATAATTAAACTCTTCATAGACTAAACGTGACAATTTAATATATATA
TATATTTATTTATATATATGATTAAATAAGATATAGTCAATCATCGGTGTGAATCGACTTAAAAAA
AAAAGCACATGGGTTAAACCCATCTCCCCTTATTAGGGGAATCAGAACTTGTTAGTGGGTTCAT
GACAGAGCATGCTGCCGTAGTTTTCGTATTCTTCTTTTTAGCTGAGTACGGTAGTATTGTACTAAT
GTGTATTTTAACTAGTATATTATTTATTGGTGGTTACTTATTATTTGAAATATCCTATGTTTTTACTG
TGGTAAATTATATTTTCTTTGAATTATTCTTTATAGACTGAGTAACATTTGTAGAGGTACAATCTTT
ATACACTGATTTTTTAAATAATTCTATCATTGAAGGATTATTATATGGGTTTAATCTAGGATTAAA
AAGTTCTTTAATGATATTCACATTTATTTGAGCTAGAGCATCCTTCCCTAGAATACGATTTGATCA
ACTAATGGGCTTCTGTTGAACAGTTTTATTACCTATTAATTTTGCAATTATTATATTAGTACCTTGT
GTTTTATATAGTTTTAACTTATTACCTGTAAATATACCATTGTTCTAGCTCACACACCCGCCCGCC
CTACTGCCACAAGGCTACAGTACATATGAGGAGGGGAACTAAAGATCTAGAACTATCCTAGTTA
ATAATTACACTTAATAGTATACTAAATAGACCATCTATCATACTCGAGAATAGTGATAGTGTAAT
TATACACTATTTTAACTGTATTCTACACTATTAGCATATTATTATCTTTATATGATAATAATTTTATA
ACTTAATTTATTAGGTTACATTAACAAAAAATTCGTTAACTTTTTTCACCACTTTTTAATACAAAAT
ACGAATTTTA
