# Supplementary material for: ABCC Transporter Gene MoABC-R1 Is Associated with Pyraclostrobin Tolerance in Magnaporthe oryzae
Source: J Fungi (Basel). 2023 Sep 11;9(9):917. doi: 10.3390/jof9090917 (PMC10532721; doi:10.3390/jof9090917)
Supplement: Supplementary file 1 [file jof-09-00917-s001.zip › Supplementary Data S2_Amino acid sequence of gene CYTB.pdf]

>RB22
ITILKNIKSYIKIIHLYSINLKGIFSILYLVLLLFSLLQVFLLLGSLLNRWYARLLIKVISFYLLQYLV
LEGFIIILKKCSFIHLTINTKKLKIILLMVKLLIETIKYMLNLIIQMLCLVLWQLFLLLHFYYSCLIK
NDVWVLYWYNLYLAINEYDIIYRVCICYSYFIFSHFYICSSSLSRKIYFRVWFPFFRAKNTIRYKI
LYFCFSLFTFRFRNIINFPFRSVCWYLWSFSYFNFYSYNNYRIYIIRKRSNRQQTKIIYTIERLPYR
VCWNRGFVKLRQKTKKPTKGLMGSYKKMITYIYSYYIFTITRIIIYIYIILLYIKIIIIIRYNYSILLY
KNIIYPIINFIFFLIIIFFYIMLTHLQMKRTKYCCYSSGYRKIFLYYLFLLGSFSVRGYMDGRRVFIL
MNSGFKLTLFMNLNYNYINVGYDRIMYSLRVKRDTPTYNTYWFRVEYIYLRVKNIYIQYIINIVI
YWYGLNQVYILSFDKHLFFYLLLLLYCEINNYNIILHSTYLGILSYLAINDSNQILFSLVWNNDL
LIHWFFDSKKRVNNFLDQKYYFLQKAYAFHFFDKQLLLIFGCLLVLTITKFFQFLYKFKEYKVIH
YNILTLSMRHNQVTLVTCEILVHYLFVYKLLPVLHLCIIVLVWKLLTQSIEMLITGDFVIYIVIQL
LLSFSCIYTEEVYITDHIELLVLFELLVLLYYWLSVSVMFYLMDRCHYEVLQLLLILLVLYLEGKI
LLNSFEVVFLLIMPLTDFLHYILYCLLYLLFCTLHFMILLVQAILLVFQVITIELHLLHIFYLKILLF
LYLFLYVLLYSLCLMFGIVIIILWLILCKLLLLLYLNDTYYLSMLFDLYLINYVLRCLVLFLLCYY
LLQIVDLEVYNLDHLVKLSEFLLLISFCNVLNTLKIHLYYVNVLYYTLVILLLYYLLVTIIVLIYLI
NLNYFIKLTKYIKIIIYIFYLRYYFSILGFQFIIYIILCITLHLALANLLFPLVWNNDLLIHWFFESKK
EIRNIFLLLLILFLKNIHFALPFSCIKMNDIKNKYLNYSYVILSYIATIIRSCINTCTCLISCSLCYSC
KKNYGYAKKIRSCCRLLWTIASICCLKTFIKRICSSYTIYCSFLFRSCNNFNFCIIRLRCYTLWSW
FRDKRHEFRYILHVSCVIFSYIRYSISWLKCEIRFSRFSKYSSINLINIKFSYINSNYDNRKFKFNCL
YISKSYLIYTTFISCVYNIFHRIYSDKSSFFSRGPANLVWSGYMSQIARKPFYFKNKRQLAGNLIP
NKYIIKLFIDTQFNIYIYLFIYMIKDIVNHRCESTKKKHMGTHLPLLGESELVSGFMTEHAAVVF
VFFFLAEYGSIVLMCILTSILFIGGYLLFEISYVFTVVNYIFFELFFIDVTFVEVQSLYTDFLNNSIIE
GLLYGFNLGLKSSLMIFTFIARASFPRIRFDQLMGFCTVLLPINFAIIILVPCVLYSFNLLPVNIPLF
LTHPPALLPQGYSTYEEGNRSRTILVNNYTYTKTIYHTRECNYTLFLYSTLLAYYYLYMIIILLNL
LGYINKKFVNFFHHFLIQNTNF
>F-1
ITILKNIKSYIKIIHLYSINLKGIFSILYLVLLLFSLLQVFLLLGSLLNRWYARLLIKVISFYLLQYLV
LEGFIIILKKCSFIHLTINTKKLKIILLMVKLLIETIKYMLNLIIQMLCLVLWQLFLLLHFYYSCLIK
NDVWVLYWYNLYLAINEYDIIYRVCICYSYFIFSHFYICSSSLSRKIYFRVWFPFFRAKNTIRYKI
LYFCFSLFTFRFRNIINFPFRSVCWYLWSFSYFNFYSYNNYRIYIIRKRSNRQQTKIIYTIERLPYR
VCWNRGFVKLRQKTKKPTKGLMGSYKKMITYIYSYYIFTITRIIIYIYIILLYIKIIIIIRYNYSILLY
KNIIYPIINFIFFLIIIFFYIMLTHLQMKRTKYCCYSSGYRKIFLYYLFLLGSFSVRGYMDGRRVFIL
MNSGFKLTLFMNLNYNYINVGYDRIMYSLRVKRDTPTYNTYWFRVEYIYLRVKNIYIQYIINIVI
YWYGLNQVYILSFDKHLFFYLLLLLYCEINNYNIILHSTYLGILSYLAINDSNQILFSLVWNNDL
LIHWFFDSKKRVNNFLDQKYYFLQKAYAFHFFDKQLLLIFGCLLVLTITKFFQFLYKFKEYKVIH
YNILTLSMRHNQVTLVTCEILVHYLFVYKLLPVLHLCIIVLVWKLLTQSIEMLITGDFVIYIVIQL
LLSFSCIYTEEVYITDHIELLVLFELLVLLYYWLSVSVMFYLMDRCHYEVLQLLLILLVLYLEGKI
LLNSFEVVFLLIMPLTDFLHYILYCLLYLLFCTLHFMILLVQAILLVFQVITIELHLLHIFYLKILLF
LYLFLYVLLYSLCLMFGIVIIILWLILCKLLLLLYLNDTYYLSMLFDLYLINYVLRCLVLFLLCYY
LLQIVDLEVYNLDHLVKLSEFLLLISFCNVLNTLKIHLYYVNVLYYTLVILLLYYLLVTIIVLIYLI
NLNYFIKLTKYIKIIIYIFYLRYYFSILGFQFIIYIILCITLHLALANLLFPLVWNNDLLIHWFFESKK
EIRNIFLLLLILFLKNIHFALPFSCIKMNDIKNKYLNYSYVILSYIATIIRSCINTCTCLISCSLCYSC
KKNYGYAKKIRSCCRLLWTIASICCLKTFIKRICSSYTIYCSFLFRSCNNFNFCIIRLRCYTLWSW
FRDKRHEFRYILHVSCVIFSYIRYSISWLKCEIRFSRFSKYSSINLINIKFSYINSNYDNRKFKFNCL

YISKSYLIYTTFISCVYNIFHRIYSDKSSFFSRGPANLVWSGYMSQIARKPFYFKNKRQLAGNLIP
NKYIIKLFIDTQFNIYIYLFIYMIKDIVNHRCESTKKKHMGTHLPLLGESELVSGFMTEHAAVVF
VFFFLAEYGSIVLMCILTSILFIGGYLLFEISYVFTVVNYIFFELFFIDVTFVEVQSLYTDFLNNSIIE
GLLYGFNLGLKSSLMIFTFIARASFPRIRFDQLMGFCTVLLPINFAIIILVPCVLYSFNLLPVNIPLF
LTHPPALLPQGYSTYEEGNRSRTILVNNYTYTKTIYHTRECNYTLFLYSTLLAYYYLYMIIILLNL
LGYINKKFVNFFHHFLIQNTNF
>F-2
ITILKNIKSYIKIIHLYSINLKGIFSILYLVLLLFSLLQVFLLLGSLLNRWYARLLIKVISFYLLQYLV
LEGFIIILKKCSFIHLTINTKKLKIILLMVKLLIETIKYMLNLIIQMLCLVLWQLFLLLHFYYSCLIK
NDVWVLYWYNLYLAINEYDIIYRVCICYSYFIFSHFYICSSSLSRKIYFRVWFPFFRAKNTIRYKI
LYFCFSLFTFRFRNIINFPFRSVCWYLWSFSYFNFYSYNNYRIYIIRKRSNRQQTKIIYTIERLPYR
VCWNRGFVKLRQKTKKPTKGLMGSYKKMITYIYSYYIFTITRIIIYIYIILLYIKIIIIIRYNYSILLY
KNIIYPIINFIFFLIIIFFYIMLTHLQMKRTKYCCYSSGYRKIFLYYLFLLGSFSVRGYMDGRRVFIL
MNSGFKLTLFMNLNYNYINVGYDRIMYSLRVKRDTPTYNTYWFRVEYIYLRVKNIYIQYIINIVI
YWYGLNQVYILSFDKHLFFYLLLLLYCEINNYNIILHSTYLGILSYLAINDSNQILFSLVWNNDL
LIHWFFDSKKRVNNFLDQKYYFLQKAYAFHFFDKQLLLIFGCLLVLTITKFFQFLYKFKEYKVIH
YNILTLSMRHNQVTLVTCEILVHYLFVYKLLPVLHLCIIVLVWKLLTQSIEMLITGDFVIYIVIQL
LLSFSCIYTEEVYITDHIELLVLFELLVLLYYWLSVSVMFYLMDRCHYEVLQLLLILLVLYLEGKI
LLNSFEVVFLLIMPLTDFLHYILYCLLYLLFCTLHFMILLVQAILLVFQVITIELHLLHIFYLKILLF
LYLFLYVLLYSLCLMFGIVIIILWLILCKLLLLLYLNDTYYLSMLFDLYLINYVLRCLVLFLLCYY
LLQIVDLEVYNLDHLVKLSEFLLLISFCNVLNTLKIHLYYVNVLYYTLVILLLYYLLVTIIVLIYLI
NLNYFIKLTKYIKIIIYIFYLRYYFSILGFQFIIYIILCITLHLALANLLFPLVWNNDLLIHWFFESKK
EIRNIFLLLLILFLKNIHFALPFSCIKMNDIKNKYLNYSYVILSYIATIIRSCINTCTCLISCSLCYSC
KKNYGYAKKIRSCCRLLWTIASICCLKTFIKRICSSYTIYCSFLFRSCNNFNFCIIRLRCYTLWSW
FRDKRHEFRYILHVSCVIFSYIRYSISWLKCEIRFSRFSKYSSINLINIKFSYINSNYDNRKFKFNCL
YISKSYLIYTTFISCVYNIFHRIYSDKSSFFSRGPANLVWSGYMSQIARKPFYFKNKRQLAGNLIP
NKYIIKLFIDTQFNIYIYLFIYMIKDIVNHRCESTKKKHMGTHLPLLGESELVSGFMTEHAAVVF
VFFFLAEYGSIVLMCILTSILFIGGYLLFEISYVFTVVNYIFFELFFIDVTFVEVQSLYTDFLNNSIIE
GLLYGFNLGLKSSLMIFTFIARASFPRIRFDQLMGFCTVLLPINFAIIILVPCVLYSFNLLPVNIPLF
LTHPPALLPQGYSTYEEGNRSRTILVNNYTYTKTIYHTRECNYTLFLYSTLLAYYYLYMIIILLNL
LGYINKKFVNFFHHFLIQNTN
>H-2
ITILKNIKSYIKIIHLYSINLKGIFSILYLVLLLFSLLQVFLLLGSLLNRWYARLLIKVISFYLLQYLV
LEGFIIILKKCSFIHLTINTKKLKIILLMVKLLIETIKYMLNLIIQMLCLVLWQLFLLLHFYYSCLIK
NDVWVLYWYNLYLAINEYDIIYRVCICYSYFIFSHFYICSSSLSRKIYFRVWFPFFRAKNTIRYKI
LYFCFSLFTFRFRNIINFPFRSVCWYLWSFSYFNFYSYNNYRIYIIRKRSNRQQTKIIYTIERLPYR
VCWNRGFVKLRQKTKKPTKGLMGSYKKMITYIYSYYIFTITRIIIYIYIILLYIKIIIIIRYNYSILLY
KNIIYPIINFIFFLIIIFFYIMLTHLQMKRTKYCCYSSGYRKIFLYYLFLLGSFSVRGYMDGRRVFIL
MNSGFKLTLFMNLNYNYINVGYDRIMYSLRVKRDTPTYNTYWFRVEYIYLRVKNIYIQYIINIVI
YWYGLNQVYILSFDKHLFFYLLLLLYCEINNYNIILHSTYLGILSYLAINDSNQILFSLVWNNDL
LIHWFFDSKKRVNNFLDQKYYFLQKAYAFHFFDKQLLLIFGCLLVLTITKFFQFLYKFKEYKVIH
YNILTLSMRHNQVTLVTCEILVHYLFVYKLLPVLHLCIIVLVWKLLTQSIEMLITGDFVIYIVIQL
LLSFSCIYTEEVYITDHIELLVLFELLVLLYYWLSVSVMFYLMDRCHYEVLQLLLILLVLYLEGKI
LLNSFEVVFLLIMPLTDFLHYILYCLLYLLFCTLHFMILLVQAILLVFQVITIELHLLHIFYLKILLF

LYLFLYVLLYSLCLMFGIVIIILWLILCKLLLLLYLNDTYYLSMLFDLYLINYVLRCLVLFLLCYY
LLQIVDLEVYNLDHLVKLSEFLLLISFCNVLNTLKIHLYYVNVLYYTLVILLLYYLLVTIIVLIYLI
NLNYFIKLTKYIKIIIYIFYLRYYFSILGFQFIIYIILCITLHLALANLLFPLVWNNDLLIHWFFESKK
EIRNIFLLLLILFLKNIHFALPFSCIKMNDIKNKYLNYSYVILSYIATIIRSCINTCTCLISCSLCYSC
KKNYGYAKKIRSCCRLLWTIASICCLKTFIKRICSSYTIYCSFLFRSCNNFNFCIIRLRCYTLWSW
FRDKRHEFRYILHVSCVIFSYIRYSISWLKCEIRFSRFSKYSSINLINIKFSYINSNYDNRKFKFNCL
YISKSYLIYTTFISCVYNIFHRIYSDKSSFFSRGPANLVWSGYMSQIARKPFYFKNKRQLAGNLIP
NKYIIKLFIDTQFNIYIYLFIYMIKDIVNHRCESTKKKHMGTHLPLLGESELVSGFMTEHAAVVF
VFFFLAEYGSIVLMCILTSILFIGGYLLFEISYVFTVVNYIFFELFFIDVTFVEVQSLYTDFLNNSIIE
GLLYGFNLGLKSSLMIFTFIARASFPRIRFDQLMGFCTVLLPINFAIIILVPCVLYSFNLLPVNIPLF
LTHPPALLPQGYSTYEEGNRSRTILVNNYTYTKTIYHTRECNYTLFLYSTLLAYYYLYMIIILLNL
LGYINKKFVNFFHHFLIQNTNF
>Hei-1
ITILKNIKSYIKIIHLYSINLKGIFSILYLVLLLFSLLQVFLLLGSLLNRWYARLLIKVISFYLLQYLV
LEGFIIILKKCSFIHLTINTKKLKIILLMVKLLIETIKYMLNLIIQMLCLVLWQLFLLLHFYYSCLIK
NDVWVLYWYNLYLAINEYDIIYRVCICYSYFIFSHFYICSSSLSRKIYFRVWFPFFRAKNTIRYKI
LYFCFSLFTFRFRNIINFPFRSVCWYLWSFSYFNFYSYNNYRIYIIRKRSNRQQTKIIYTIERLPYR
VCWNRGFVKLRQKTKKPTKGLMGSYKKMITYIYSYYIFTITRIIIYIYIILLYIKIIIIIRYNYSILLY
KNIIYPIINFIFFLIIIFFYIMLTHLQMKRTKYCCYSSGYRKIFLYYLFLLGSFSVRGYMDGRRVFIL
MNSGFKLTLFMNLNYNYINVGYDRIMYSLRVKRDTPTYNTYWFRVEYIYLRVKNIYIQYIINIVI
YWYGLNQVYILSFDKHLFFYLLLLLYCEINNYNIILHSTYLGILSYLAINDSNQILFSLVWNNDL
LIHWFFDSKKRVNNFLDQKYYFLQKAYAFHFFDKQLLLIFGCLLVLTITKFFQFLYKFKEYKVIH
YNILTLSMRHNQVTLVTCEILVHYLFVYKLLPVLHLCIIVLVWKLLTQSIEMLITGDFVIYIVIQL
LLSFSCIYTEEVYITDHIELLVLFELLVLLYYWLSVSVMFYLMDRCHYEVLQLLLILLVLYLEGKI
LLNSFEVVFLLIMPLTDFLHYILYCLLYLLFCTLHFMILLVQAILLVFQVITIELHLLHIFYLKILLF
LYLFLYVLLYSLCLMFGIVIIILWLILCKLLLLLYLNDTYYLSMLFDLYLINYVLRCLVLFLLCYY
LLQIVDLEVYNLDHLVKLSEFLLLISFCNVLNTLKIHLYYVNVLYYTLVILLLYYLLVTIIVLIYLI
NLNYFIKLTKYIKIIIYIFYLRYYFSILGFQFIIYIILCITLHLALANLLFPLVWNNDLLIHWFFESKK
EIRNIFLLLLILFLKNIHFALPFSCIKMNDIKNKYLNYSYVILSYIATIIRSCINTCTCLISCSLCYSC
KKNYGYAKKIRSCCRLLWTIASICCLKTFIKRICSSYTIYCSFLFRSCNNFNFCIIRLRCYTLWSW
FRDKRHEFRYILHVSCVIFSYIRYSISWLKCEIRFSRFSKYSSINLINIKFSYINSNYDNRKFKFNCL
YISKSYLIYTTFISCVYNIFHRIYSDKSSFFSRGPANLVWSGYMSQIARKPFYFKNKRQLAGNLIP
NKYIIKLFIDTQFNIYIYLFIYMIKDIVNHRCESTKKKHMGTHLPLLGESELVSGFMTEHAAVVF
VFFFLAEYGSIVLMCILTSILFIGGYLLFEISYVFTVVNYIFFELFFIDVTFVEVQSLYTDFLNNSIIE
GLLYGFNLGLKSSLMIFTFIARASFPRIRFDQLMGFCTVLLPINFAIIILVPCVLYSFNLLPVNIPLF
LTHPPALLPQGYSTYEEGNRSRTILVNNYTYTKTIYHTRECNYTLFLYSTLLAYYYLYMIIILLNL
LGYINKKFVNFFHHFLIQNTNF
>Hei-3
ITILKNIKSYIKIIHLYSINLKGIFSILYLVLLLFSLLQVFLLLGSLLNRWYARLLIKVISFYLLQYLV
LEGFIIILKKCSFIHLTINTKKLKIILLMVKLLIETIKYMLNLIIQMLCLVLWQLFLLLHFYYSCLIK
NDVWVLYWYNLYLAINEYDIIYRVCICYSYFIFSHFYICSSSLSRKIYFRVWFPFFRAKNTIRYKI
LYFCFSLFTFRFRNIINFPFRSVCWYLWSFSYFNFYSYNNYRIYIIRKRSNRQQTKIIYTIERLPYR
VCWNRGFVKLRQKTKKPTKGLMGSYKKMITYIYSYYIFTITRIIIYIYIILLYIKIIIIIRYNYSILLY
KNIIYPIINFIFFLIIIFFYIMLTHLQMKRTKYCCYSSGYRKIFLYYLFLLGSFSVRGYMDGRRVFIL

MNSGFKLTLFMNLNYNYINVGYDRIMYSLRVKRDTPTYNTYWFRVEYIYLRVKNIYIQYIINIVI
YWYGLNQVYILSFDKHLFFYLLLLLYCEINNYNIILHSTYLGILSYLAINDSNQILFSLVWNNDL
LIHWFFDSKKRVNNFLDQKYYFLQKAYAFHFFDKQLLLIFGCLLVLTITKFFQFLYKFKEYKVIH
YNILTLSMRHNQVTLVTCEILVHYLFVYKLLPVLHLCIIVLVWKLLTQSIEMLITGDFVIYIVIQL
LLSFSCIYTEEVYITDHIELLVLFELLVLLYYWLSVSVMFYLMDRCHYEVLQLLLILLVLYLEGKI
LLNSFEVVFLLIMPLTDFLHYILYCLLYLLFCTLHFMILLVQAILLVFQVITIELHLLHIFYLKILLF
LYLFLYVLLYSLCLMFGIVIIILWLILCKLLLLLYLNDTYYLSMLFDLYLINYVLRCLVLFLLCYY
LLQIVDLEVYNLDHLVKLSEFLLLISFCNVLNTLKIHLYYVNVLYYTLVILLLYYLLVTIIVLIYLI
NLNYFIKLTKYIKIIIYIFYLRYYFSILGFQFIIYIILCITLHLALANLLFPLVWNNDLLIHWFFESKK
EIRNIFLLLLILFLKNIHFALPFSCIKMNDIKNKYLNYSYVILSYIATIIRSCINTCTCLISCSLCYSC
KKNYGYAKKIRSCCRLLWTIASICCLKTFIKRICSSYTIYCSFLFRSCNNFNFCIIRLRCYTLWSW
FRDKRHEFRYILHVSCVIFSYIRYSISWLKCEIRFSRFSKYSSINLINIKFSYINSNYDNRKFKFNCL
YISKSYLIYTTFISCVYNIFHRIYSDKSSFFSRGPANLVWSGYMSQIARKPFYFKNKRQLAGNLIP
NKYIIKLFIDTQFNIYIYLFIYMIKDIVNHRCESTKKKHMGTHLPLLGESELVSGFMTEHAAVVF
VFFFLAEYGSIVLMCILTSILFIGGYLLFEISYVFTVVNYIFFELFFIDVTFVEVQSLYTDFLNNSIIE
GLLYGFNLGLKSSLMIFTFIARASFPRIRFDQLMGFCTVLLPINFAIIILVPCVLYSFNLLPVNIPLF
LTHPPALLPQGYSTYEEGNRSRTILVNNYTYTKTIYHTRECNYTLFLYSTLLAYYYLYMIIILLNL
LGYINKKFVNFFHHFLIQNTNF
>Hu-1
ITILKNIKSYIKIIHLYSINLKGIFSILYLVLLLFSLLQVFLLLGSLLNRWYARLLIKVISFYLLQYLV
LEGFIIILKKCSFIHLTINTKKLKIILLMVKLLIETIKYMLNLIIQMLCLVLWQLFLLLHFYYSCLIK
NDVWVLYWYNLYLAINEYDIIYRVCICYSYFIFSHFYICSSSLSRKIYFRVWFPFFRAKNTIRYKI
LYFCFSLFTFRFRNIINFPFRSVCWYLWSFSYFNFYSYNNYRIYIIRKRSNRQQTKIIYTIERLPYR
VCWNRGFVKLRQKTKKPTKGLMGSYKKMITYIYSYYIFTITRIIIYIYIILLYIKIIIIIRYNYSILLY
KNIIYPIINFIFFLIIIFFYIMLTHLQMKRTKYCCYSSGYRKIFLYYLFLLGSFSVRGYMDGRRVFIL
MNSGFKLTLFMNLNYNYINVGYDRIMYSLRVKRDTPTYNTYWFRVEYIYLRVKNIYIQYIINIVI
YWYGLNQVYILSFDKHLFFYLLLLLYCEINNYNIILHSTYLGILSYLAINDSNQILFSLVWNNDL
LIHWFFDSKKRVNNFLDQKYYFLQKAYAFHFFDKQLLLIFGCLLVLTITKFFQFLYKFKEYKVIH
YNILTLSMRHNQVTLVTCEILVHYLFVYKLLPVLHLCIIVLVWKLLTQSIEMLITGDFVIYIVIQL
LLSFSCIYTEEVYITDHIELLVLFELLVLLYYWLSVSVMFYLMDRCHYEVLQLLLILLVLYLEGKI
LLNSFEVVFLLIMPLTDFLHYILYCLLYLLFCTLHFMILLVQAILLVFQVITIELHLLHIFYLKILLF
LYLFLYVLLYSLCLMFGIVIIILWLILCKLLLLLYLNDTYYLSMLFDLYLINYVLRCLVLFLLCYY
LLQIVDLEVYNLDHLVKLSEFLLLISFCNVLNTLKIHLYYVNVLYYTLVILLLYYLLVTIIVLIYLI
NLNYFIKLTKYIKIIIYIFYLRYYFSILGFQFIIYIILCITLHLALANLLFPLVWNNDLLIHWFFESKK
EIRNIFLLLLILFLKNIHFALPFSCIKMNDIKNKYLNYSYVILSYIATIIRSCINTCTCLISCSLCYSC
KKNYGYAKKIRSCCRLLWTIASICCLKTFIKRICSSYTIYCSFLFRSCNNFNFCIIRLRCYTLWSW
FRDKRHEFRYILHVSCVIFSYIRYSISWLKCEIRFSRFSKYSSINLINIKFSYINSNYDNRKFKFNCL
YISKSYLIYTTFISCVYNIFHRIYSDKSSFFSRGPANLVWSGYMSQIARKPFYFKNKRQLAGNLIP
NKYIIKLFIDTQFNIYIYLFIYMIKDIVNHRCESTKKKHMGTHLPLLGESELVSGFMTEHAAVVF
VFFFLAEYGSIVLMCILTSILFIGGYLLFEISYVFTVVNYIFFELFFIDVTFVEVQSLYTDFLNNSIIE
GLLYGFNLGLKSSLMIFTFIARASFPRIRFDQLMGFCTVLLPINFAIIILVPCVLYSFNLLPVNIPLF
LTHPPALLPQGYSTYEEGNRSRTILVNNYTYTKTIYHTRECNYTLFLYSTLLAYYYLYMIIILLNL
LGYINKKFVNFFHHFLIQNTNF
>Hu-2

ITILKNIKSYIKIIHLYSINLKGIFSILYLVLLLFSLLQVFLLLGSLLNRWYARLLIKVISFYLLQYLV
LEGFIIILKKCSFIHLTINTKKLKIILLMVKLLIETIKYMLNLIIQMLCLVLWQLFLLLHFYYSCLIK
NDVWVLYWYNLYLAINEYDIIYRVCICYSYFIFSHFYICSSSLSRKIYFRVWFPFFRAKNTIRYKI
LYFCFSLFTFRFRNIINFPFRSVCWYLWSFSYFNFYSYNNYRIYIIRKRSNRQQTKIIYTIERLPYR
VCWNRGFVKLRQKTKKPTKGLMGSYKKMITYIYSYYIFTITRIIIYIYIILLYIKIIIIIRYNYSILLY
KNIIYPIINFIFFLIIIFFYIMLTHLQMKRTKYCCYSSGYRKIFLYYLFLLGSFSVRGYMDGRRVFIL
MNSGFKLTLFMNLNYNYINVGYDRIMYSLRVKRDTPTYNTYWFRVEYIYLRVKNIYIQYIINIVI
YWYGLNQVYILSFDKHLFFYLLLLLYCEINNYNIILHSTYLGILSYLAINDSNQILFSLVWNNDL
LIHWFFDSKKRVNNFLDQKYYFLQKAYAFHFFDKQLLLIFGCLLVLTITKFFQFLYKFKEYKVIH
YNILTLSMRHNQVTLVTCEILVHYLFVYKLLPVLHLCIIVLVWKLLTQSIEMLITGDFVIYIVIQL
LLSFSCIYTEEVYITDHIELLVLFELLVLLYYWLSVSVMFYLMDRCHYEVLQLLLILLVLYLEGKI
LLNSFEVVFLLIMPLTDFLHYILYCLLYLLFCTLHFMILLVQAILLVFQVITIELHLLHIFYLKILLF
LYLFLYVLLYSLCLMFGIVIIILWLILCKLLLLLYLNDTYYLSMLFDLYLINYVLRCLVLFLLCYY
LLQIVDLEVYNLDHLVKLSEFLLLISFCNVLNTLKIHLYYVNVLYYTLVILLLYYLLVTIIVLIYLI
NLNYFIKLTKYIKIIIYIFYLRYYFSILGFQFIIYIILCITLHLALANLLFPLVWNNDLLIHWFFESKK
EIRNIFLLLLILFLKNIHFALPFSCIKMNDIKNKYLNYSYVILSYIATIIRSCINTCTCLISCSLCYSC
KKNYGYAKKIRSCCRLLWTIASICCLKTFIKRICSSYTIYCSFLFRSCNNFNFCIIRLRCYTLWSW
FRDKRHEFRYILHVSCVIFSYIRYSISWLKCEIRFSRFSKYSSINLINIKFSYINSNYDNRKFKFNCL
YISKSYLIYTTFISCVYNIFHRIYSDKSSFFSRGPANLVWSGYMSQIARKPFYFKNKRQLAGNLIP
NKYIIKLFIDTQFNIYIYLFIYMIKDIVNHRCESTKKKHMGTHLPLLGESELVSGFMTEHAAVVF
VFFFLAEYGSIVLMCILTSILFIGGYLLFEISYVFTVVNYIFFELFFIDVTFVEVQSLYTDFLNNSIIE
GLLYGFNLGLKSSLMIFTFIARASFPRIRFDQLMGFCTVLLPINFAIIILVPCVLYSFNLLPVNIPLF
LTHPPALLPQGYSTYEEGNRSRTILVNNYTYTKTIYHTRECNYTLFLYSTLLAYYYLYMIIILLNL
LGYINKKFVNFFHHFLIQNTNF
>l-1
ITILKNIKSYIKIIHLYSINLKGIFSILYLVLLLFSLLQVFLLLGSLLNRWYARLLIKVISFYLLQYLV
LEGFIIILKKCSFIHLTINTKKLKIILLMVKLLIETIKYMLNLIIQMLCLVLWQLFLLLHFYYSCLIK
NDVWVLYWYNLYLAINEYDIIYRVCICYSYFIFSHFYICSSSLSRKIYFRVWFPFFRAKNTIRYKI
LYFCFSLFTFRFRNIINFPFRSVCWYLWSFSYFNFYSYNNYRIYIIRKRSNRQQTKIIYTIERLPYR
VCWNRGFVKLRQKTKKPTKGLMGSYKKMITYIYSYYIFTITRIIIYIYIILLYIKIIIIIRYNYSILLY
KNIIYPIINFIFFLIIIFFYIMLTHLQMKRTKYCCYSSGYRKIFLYYLFLLGSFSVRGYMDGRRVFIL
MNSGFKLTLFMNLNYNYINVGYDRIMYSLRVKRDTPTYNTYWFRVEYIYLRVKNIYIQYIINIVI
YWYGLNQVYILSFDKHLFFYLLLLLYCEINNYNIILHSTYLGILSYLAINDSNQILFSLVWNNDL
LIHWFFDSKKRVNNFLDQKYYFLQKAYAFHFFDKQLLLIFGCLLVLTITKFFQFLYKFKEYKVIH
YNILTLSMRHNQVTLVTCEILVHYLFVYKLLPVLHLCIIVLVWKLLTQSIEMLITGDFVIYIVIQL
LLSFSCIYTEEVYITDHIELLVLFELLVLLYYWLSVSVMFYLMDRCHYEVLQLLLILLVLYLEGKI
LLNSFEVVFLLIMPLTDFLHYILYCLLYLLFCTLHFMILLVQAILLVFQVITIELHLLHIFYLKILLF
LYLFLYVLLYSLCLMFGIVIIILWLILCKLLLLLYLNDTYYLSMLFDLYLINYVLRCLVLFLLCYY
LLQIVDLEVYNLDHLVKLSEFLLLISFCNVLNTLKIHLYYVNVLYYTLVILLLYYLLVTIIVLIYLI
NLNYFIKLTKYIKIIIYIFYLRYYFSILGFQFIIYIILCITLHLALANLLFPLVWNNDLLIHWFFESKK
EIRNIFLLLLILFLKNIHFALPFSCIKMNDIKNKYLNYSYVILSYIATIIRSCINTCTCLISCSLCYSC
KKNYGYAKKIRSCCRLLWTIASICCLKTFIKRICSSYTIYCSFLFRSCNNFNFCIIRLRCYTLWSW
FRDKRHEFRYILHVSCVIFSYIRYSISWLKCEIRFSRFSKYSSINLINIKFSYINSNYDNRKFKFNCL
YISKSYLIYTTFISCVYNIFHRIYSDKSSFFSRGPANLVWSGYMSQIARKPFYFKNKRQLAGNLIP

NKYIIKLFIDTQFNIYIYLFIYMIKDIVNHRCESTKKKHMGTHLPLLGESELVSGFMTEHAAVVF
VFFFLAEYGSIVLMCILTSILFIGGYLLFEISYVFTVVNYIFFELFFIDVTFVEVQSLYTDFLNNSIIE
GLLYGFNLGLKSSLMIFTFIARASFPRIRFDQLMGFCTVLLPINFAIIILVPCVLYSFNLLPVNIPLF
LTHPPALLPQGYSTYEEGNRSRTILVNNYTYTKTIYHTRECNYTLFLYSTLLAYYYLYMIIILLNL
LGYINKKFVNFFHHFLIQNTNF
>LZJ-32-5
ITILKNIKSYIKIIHLYSINLKGIFSILYLVLLLFSLLQVFLLLGSLLNRWYARLLIKVISFYLLQYLV
LEGFIIILKKCSFIHLTINTKKLKIILLMVKLLIETIKYMLNLIIQMLCLVLWQLFLLLHFYYSCLIK
NDVWVLYWYNLYLAINEYDIIYRVCICYSYFIFSHFYICSSSLSRKIYFRVWFPFFRAKNTIRYKI
LYFCFSLFTFRFRNIINFPFRSVCWYLWSFSYFNFYSYNNYRIYIIRKRSNRQQTKIIYTIERLPYR
VCWNRGFVKLRQKTKKPTKGLMGSYKKMITYIYSYYIFTITRIIIYIYIILLYIKIIIIIRYNYSILLY
KNIIYPIINFIFLFLIIIFFYIMLTHLQMKRTKYCCYSSGYRKIFLYYLFLLGSFSVRGYMDGRRVFI
MNSGFKLTLFMNLNYNYINVGYDRIMYSLRVKRDTPTYNTYWFRVEYIYLRVKNIYIQYIINIVI
YWYGLNQVVYILSFDKHLFFYLLLLLYCEINNYNIILHSTYLGILSYLAINDSNQILFSLVWNNDL
LIHWFFDSKKRVNNFLDQKYYFLQKAYAFHFFDKQLLLIFGCLLVLTITKFFQFLYKFKEYKVIH
YNILTLSMRHNQVTLVTCEILVHYLFVYKLLPVLHLCIIVLVWKLLTQSIEMLITGDFVIYIVIQL
LLSFSCIYTEEVYITDHIELLVLFELLVLLYYWLSVSVMFYLMDRCHYEVLQLLLILLVLYLEGKI
LLNSFEVVFLLIMPLTDFLHYILYCLLYLLFCTLHFMILLVQAILLVFQVITIELHLLHIIYLKILLFL
YLFLYVLLYSLCLMFGIVIIILWLILCKLLLLLYLNDTYYLSMLFDLYLINYVLRCLVLFLLCYYL
LQIVDLEVYNLDHLVKLSEFLLLISFCNVLNTLKIHLYYVNVLYYTLVILLLYYLLVTIIVLIYLIN
LNYFIKLTKYIKIIIYIFYLRYYFSILGFQFIIIILCITLHLALANLLFPLVWNNDLLIHWFFESKKEIR
NIFLLLLLILFLKNIHFALPFSCIKMNDIKNKYLNYSYVILSYIATIIRSCINTCTCLISCSLCYSCKK
NYGYAKKIRSCCRLLWTIASICCLKTFIRICSSYTIYCSFLFRSCNKFNFCIIRLRCYTLWSWFRDK
RHEFRYILHVSCVIFSYIRYSISWLKCEIRFSRFSKYSSINLINIKFSYINSNYDNRKFKFNCLYISK
SYLIYTTFISCVYNIFHRIYSDKSSFFSRGPANLVWSGYMSQIARKPFYFKNKRQLAGNLIPNKYI
IKLFIDTQFNIYIYLFIYMIKDIVNHRCESTKKKHMGTHLPLLGESELVSGFMTEHAAVVFVFFFL
AEYGSIVLMCILTSILFIGGYLLFEISYVFTVVNYIFFELFFIDVTFVEVQSLYTDFLNNSIIEGLLY
GFNLGLKSSLMIFTFIARASFPRIRFDQLMGFCTVLLPINFAIIILVPCVLYSFNLLPVNIPLFLTHPP
ALLPQGYSTYEEGNRSRTILVNNYTYTKTIYHTRECNYTLFLYSTILAYYYLYMIIILLNLLGYIN
KLVNFFHHFLIQNTNF
>MT-14-2
ITILKNIKSYIKIIHQYSINLKGIFSILYLVLSLSSLLQGFLLLGSLLNRWYARLLIKVISFYLLQYLV
EEFIIIKKCSSIHLTISTKKLKIILLMVKLLIETIKHMNLIIQMSLCLVLWQLLFLLHFYYSCLIKND
VWVLYWYNLYLTINEYDIIYRVCISNSYIIFSYFYICSSSLSRKIYFRVWFPFFRAKNTIRYKILYIC
FSLFTFRFRNIINFSFRCVCWYLWSFSYFNFYSYNNYRIYIIRKRSNRQTKIIYTSKREIPYVRNRG
FVKLRQKTKKPTKGLMGSYKKMITYIYSYYIFTITRIIIYIYIILLYIKIIIIIRYNYSILLYKNIIYPIIN
FIFFLIIIFFYIMLTHLQMKRTKYCCYSSGYRKIFLYYLFLLGSFSVRGYMDGRRVFILMNSGFKL
TLFMNLNYNYINVGYDRIMYSLRVKRDTPTYNTYWFRVEYIYLRVKNIYIQYIINIVIYWYGLN
QVVYILSFDKHLFFYLLLLLYCEINNYNIILHSTYLGILSYLATNESNQNFSLVWNNDLLIHWFFDS
KKRVNNILAEKYYFLQKAYAFHFFDKQLLLIFDCLAVLTITKFNQVLSKFKEYKVIHYNILILSM
RHNQVILVTYEILVHYLFVYKLLQVLHLCIIVLVWKPLIQNIEMLITDDFVIYIVIQPQHSFSCIYT
VEVYIMDHIELLVLYELLVLYLYWPSVSVMFYLMDKCHYEVLQLLLILLVLSLEDKILLNLFEE
VFLLIMPLTDFLHYILYYLLYLLYCTHFTIQLVLVTLVFQVIMIELHLLHIIYLKILLFLYLYLYVHL
YSLCLMFGIVIIILWLILCKLLLLLCLNDIYYLSMLFDLYLINYELLCLVQFLCYYLLQIVDLEVY

NLDHLVKLSEFLLISYCNVLNTKIHLYYVNVLFYTLVTLLYYLLVTIIVQIYLINLNYYIKLTKFIK
IIIYIFYLRYYFSILGFQFIIYIILCITLHLALGNPLFPLVWNNDLLIHWFFDSKKEIRNNLLLLILFL
KNIHFALPFSCIKMNDIKNQYLNYSYVILPYFTAIFRSYINTCTCLVSCSLCYSSKKNYGYAKKIR
SRCRLLWTTTSICCLKTFIKRICSSYTIYSSFLFRSSYYFNICITRLCCYTIWTWFRIKRYEFRYLLY
VSCVIFSYIRYFISRLKCQICISRFSKYSSINLINIKFSHINSNYDNRKFKFNCMYISKSCLINIAIISC
IYNIFYRIYSNKSSFFSRGPNILVWSGYMSQIAGKPFYFKNKRQLAGNLIPNKYIIKLFIDTQFNIY
IYLFIYMIKDIVNHRCESTKKKHMGTHLPLLGESELVSGFMTEHAAVVFVFFFLAEYGSIVLMCI
LTSILFIGGYLLFEISYVFTVVNYIFFELFFIDVTFIEIQSLYTDFLNNSVIEGLLYGFNLGLKSSLMI
FTFIARASFPRIRFDQLMAFCTVLLPINFAIIILVPCVLYSFNLLPINIPLFLTHPPALLPQGYSTYEE
GNRSRTILVNNYTYTKTIYHTRECNYTLFLYSTLLAYYYLYMIIILLNLLGYINKKFVNFFHHFLI
QNTNF
>R01-1
ITILKNIKSYIKIIHLYSINLKGIFSILYLVLLLFSLLQVFLLLGSLLNRWYARLLIKVISFYLLQYLV
LEGFIIILKKCSFIHLTINTKKLKIILLMVKLLIETIKYMLNLIIQMLCLVLWQLFLLLHFYYSCLIK
NDVWVLYWYNLYLAINEYDIIYRVCICYSYFIFSHFYICSSSLSRKIYFRVWFPFFRAKNTIRYKI
LYFCFSLFTFRFRNIINFPFRSVCWYLWSFSYFNFYSYNNYRIYIIRKRSNRQQTKIIYTIERLPYR
VCWNRGFVKLRQKTKKPTKGLMGSYKKMITYIYSYYIFTITRIIIYIYIILLYIKIIIIIRYNYSILLY
KNIIYPIINFIFFLIIIFFYIMLTHLQMKRTKYCCYSSGYRKIFLYYLFLLGSFSVRGYMDGRRVFIL
MNSGFKLTLFMNLNYNYINVGYDRIMYSLRVKRDTPTYNTYWFRVEYIYLRVKNIYIQYIINIVI
YWYGLNQVYILSFDKHLFFYLLLLLYCEINNYNIILHSTYLGILSYLAINDSNQILFSLVWNNDL
LIHWFFDSKKRVNNFLDQKYYFLQKAYAFHFFDKQLLLIFGCLLVLTITKFFQFLYKFKEYKVIH
YNILTLSMRHNQVTLVTCEILVHYLFVYKLLPVLHLCIIVLVWKLLTQSIEMLITGDFVIYIVIQL
LLSFSCIYTEEVYITDHIELLVLFELLVLLYYWLSVSVMFYLMDRCHYEVLQLLLILLVLYLEGKI
LLNSFEVVFLLIMPLTDFLHYILYCLLYLLFCTLHFMILLVQAILLVFQVITIELHLLHIFYLKILLF
LYLFLYVLLYSLCLMFGIVIIILWLILCKLLLLLYLNDTYYLSMLFDLYLINYVLRCLVLFLLCYY
LLQIVDLEVYNLDHLVKLSEFLLLISFCNVLNTLKIHLYYVNVLYYTLVILLLYYLLVTIIVLIYLI
NLNYFIKLTKYIKIIIYIFYLRYYFSILGFQFIIYIILCITLHLALANLLFPLVWNNDLLIHWFFESKK
EIRNIFLLLLILFLKNIHFALPFSCIKMNDIKNKYLNYSYVILSYIATIIRSCINTCTCLISCSLCYSC
KKNYGYAKKIRSCCRLLWTIASICCLKTFIKRICSSYTIYCSFLFRSCNNFNFCIIRLRCYTLWSW
FRDKRHEFRYILHVSCVIFSYIRYSISWLKCEIRFSRFSKYSSINLINIKFSYINSNYDNRKFKFNCL
YISKSYLIYTTFISCVYNIFHRIYSDKSSFFSRGPANLVWSGYMSQIARKPFYFKNKRQLAGNLIP
NKYIIKLFIDTQFNIYIYLFIYMIKDIVNHRCESTKKKHMGTHLPLLGESELVSGFMTEHAAVVF
VFFFLAEYGSIVLMCILTSILFIGGYLLFEISYVFTVVNYIFFELFFIDVTFVEVQSLYTDFLNNSIIE
GLLYGFNLGLKSSLMIFTFIARASFPRIRFDQLMGFCTVLLPINFAIIILVPCVLYSFNLLPVNIPLF
LTHPPALLPQGYSTYEEGNRSRTILVNNYTYTKTIYHTRECNYTLFLYSTLLAYYYLYMIIILLNL
LGYINKKFVNFFHHFLIQNTNF
>S1
ITILKNIKSYIKIIHLYSINLKGIFSILYLVLLLFSLLQVFLLLGSLLNRWYARLLIKVISFYLLQYLV
LEGFIIILKKCSFIHLTINTKKLKIILLMVKLLIETIKYMLNLIIQMLCLVLWQLFLLLHFYYSCLIK
NDVWVLYWYNLYLAINEYDIIYRVCICYSYFIFSHFYICSSSLSRKIYFRVWFPFFRAKNTIRYKI
LYFCFSLFTFRFRNIINFPFRSVCWYLWSFSYFNFYSYNNYRIYIIRKRSNRQQTKIIYTIERLPYR
VCWNRGFVKLRQKTKKPTKGLMGSYKKMITYIYSYYIFTITRIIIYIYIILLYIKIIIIIRYNYSILLY
KNIIYPIINFIFFLIIIFFYIMLTHLQMKRTKYCCYSSGYRKIFLYYLFLLGSFSVRGYMDGRRVFIL
MNSGFKLTLFMNLNYNYINVGYDRIMYSLRVKRDTPTYNTYWFRVEYIYLRVKNIYIQYIINIVI

YWYGLNQVVYILSFDKHLFFYLLLLLYCEINNYNIILHSTYLGILSYLAINDSNQILFSLVWNNDL
LIHWFFDSKKRVNNFLDQKYYFLQKAYAFHFFDKQLLLIFGCLLVLTITKFFQFLYKFKEYKVIH
YNILTLSMRHNQVTLVTCEILVHYLFVYKLLPVLHLCIIVLVWKLLTQSIEMLITGDFVIYIVIQL
LLSFSCIYTEEVYITDHIELLVLFELLVLLYYWLSVSVMFYLMDRCHYEVLQLLLILLVLYLEGKI
LLNSFEVVFLLIMPLTDFLHYILYCLLYLLFCTLHFMILLVQAILLVFQVITIELHLLHIIYLKILLFL
YLFLYVLLYSLCLMFGIVIIILWLILCKLLLLLYLNDTYYLSMLFDLYLINYVLRCLVLFLLCYYL
LQIVDLEVYNLDHLVKLSEFLLLISFCNVLNTLKIHLYYVNVLYYTLVILLLYYLLVTIIVLIYLIN
LNYFIKLTKYIKIIIYIFYLRYYFSILGFQFIIYIILCITLHLALANLLFPLVWNNDLLIHWFFESKKEI
RNIFLLLLILFLKNIHFALPFSCIKMNDIKNKYLNYSYVILSYIATIIRSCINTCTCLISCSLCYSCKK
NYGYAKKIRSCCRLLWTIASICCLKTFIKRICSSYTIYCSFLFRSCNNFNFCIIRLRCYTLWSWFRD
KRHEFRYILHVSCVIFSYIRYSISWLKCEIRFSRFSKYSSINLINIKFSYINSNYDNRKFKFNCLYIS
KSYLIYTTFISCVYNIFHRIYSDKSSFFSRGPANLVWSGYMSQIARKPFYFKNKRQLAGNLIPNK
YIIKLFIDTQFNIYIYLFIYMIKDIVNHRCESTKKKHMGTHLPLLGESELVSGFMTEHAAVVFVFF
FLAEYGSIVLMCILTSILFIGGYLLFEISYVFTVVNYIFFELFFIDVTFVEVQSLYTDFLNNSIIEGL
LYGFNLGLKSSLMIFTFIARASFPRIRFDQLMGFCTVLLPINFAIIILVPCVLYSFNLLPVNIPLFLT
HPPALLPQGYSTYEEGNRSRTILVNNYTYTKTIYHTRECNYTLFLYSTLLAYYYLYMIIILLNLLG
YINKKFVNFFHHFLIQNTNF
>Su-1
ITILKNIKSYIKIIHLYSINLKGIFSILYLVLLLFSLLQVFLLLGSLLNRWYARLLIKVISFYLLQYLV
LEGFIIILKKCSFIHLTINTKKLKIILLMVKLLIETIKYMLNLIIQMLCLVLWQLFLLLHFYYSCLIK
NDVWVLYWYNLYLAINEYDIIYRVCICYSYFIFSHFYICSSSLSRKIYFRVWFPFFRAKNTIRYKI
LYFCFSLFTFRFRNIINFPFRSVCWYLWSFSYFNFYSYNNYRIYIIRKRSNRQQTKIIYTIERLPYR
VCWNRGFVKLRQKTKKPTKGLMGSYKKMITYIYSYYIFTITRIIIYIYIILLYIKIIIIIRYNYSILLY
KNIIYPIINFIFFLIIIFFYIMLTHLQMKRTKYCCYSSGYRKIFLYYLFLLGSFSVRGYMDGRRVFIL
MNSGFKLTLFMNLNYNYINVGYDRIMYSLRVKRDTPTYNTYWFRVEYIYLRVKNIYIQYIINIVI
YWYGLNQVVYILSFDKHLFFYLLLLLYCEINNYNIILHSTYLGILSYLAINDSNQILFSLVWNNDL
LIHWFFDSKKRVNNFLDQKYYFLQKAYAFHFFDKQLLLIFGCLLVLTITKFFQFLYKFKEYKVIH
YNILTLSMRHNQVTLVTCEILVHYLFVYKLLPVLHLCIIVLVWKLLTQSIEMLITGDFVIYIVIQL
LLSFSCIYTEEVYITDHIELLVLFELLVLLYYWLSVSVMFYLMDRCHYEVLQLLLILLVLYLEGKI
LLNSFEVVFLLIMPLTDFLHYILYCLLYLLFCTLHFMILLVQAILLVFQVITIELHLLHIIYLKILLFL
YLFLYVLLYSLCLMFGIVIIILWLILCKLLLLLYLNDTYYLSMLFDLYLINYVLRCLVLFLLCYYL
LQIVDLEVYNLDHLVKLSEFLLLISFCNVLNTLKIHLYYVNVLYYTLVILLLYYLLVTIIVLIYLIN
LNYFIKLTKYIKIIIYIFYLRYYFSILGFQFIIYIILCITLHLALANLLFPLVWNNDLLIHWFFESKKEI
RNIFLLLLILFLKNIHFALPFSCIKMNDIKNKYLNYSYVILSYIATIIRSCINTCTCLISCSLCYSCKK
NYGYAKKIRSCCRLLWTIASICCLKTFIKRICSSYTIYCSFLFRSCNNFNFCIIRLRCYTLWSWFRD
KRHEFRYILHVSCVIFSYIRYSISWLKCEIRFSRFSKYSSINLINIKFSYINSNYDNRKFKFNCLYIS
KSYLIYTTFISCVYNIFHRIYSDKSSFFSRGPANLVWSGYMSQIARKPFYFKNKRQLAGNLIPNK
YIIKLFIDTQFNIYIYLFIYMIKDIVNHRCESTKKKHMGTHLPLLGESELVSGFMTEHAAVVFVFF
FLAEYGSIVLMCILTSILFIGGYLLFEISYVFTVVNYIFFELFFIDVTFVEVQSLYTDFLNNSIIEGL
LYGFNLGLKSSLMIFTFIARASFPRIRFDQLMGFCTVLLPINFAIIILVPCVLYSFNLLPVNIPLFLT
HPPALLPQGYSTYEEGNRSRTILVNNYTYTKTIYHTRECNYTLFLYSTLLAYYYLYMIIILLNLLG
YINKKFVNFFHHFLIQNTNF
>T-3
ITILKNIKSYIKIIHLYSINLKGIFSILYLVLLLFSLLQVFLLLGSLLNRWYARLLIKVISFYLLQYLV

LEGFIIILKKCSFIHLTINTKKLKIILLMVKLLIETIKYMLNLIIQMLCLVLWQLFLLLHFYYSCLIK
NDVWVLYWYNLYLAINEYDIIYRVCICYSYFIFSHFYICSSSLSRKIYFRVWFPFFRAKNTIRYKI
LYFCFSLFTFRFRNIINFPFRSVCWYLWSFSYFNFYSYNNYRIYIIRKRSNRQQTKIIYTIERLPYR
VCWNRGFVKLRQKTKKPTKGLMGSYKKMITYIYSYYIFTITRIIIYIYIILLYIKIIIIIRYNYSILLY
KNIIYPIINFIFFLIIIFFYIMLTHLQMKRTKYCCYSSGYRKIFLYYLFLLGSFSVRGYMDGRRVFIL
MNSGFKLTLFMNLNYNYINVGYDRIMYSLRVKRDTPTYNTYWFRVEYIYLRVKNIYIQYIINIVI
YWYGLNQVYILSFDKHLFFYLLLLLYCEINNYNIILHSTYLGILSYLAINDSNQILFSLVWNNDL
LIHWFFDSKKRVNNFLDQKYYFLQKAYAFHFFDKQLLLIFGCLLVLTITKFFQFLYKFKEYKVIH
YNILTLSMRHNQVTLVTCEILVHYLFVYKLLPVLHLCIIVLVWKLLTQSIEMLITGDFVIYIVIQL
LLSFSCIYTEEVYITDHIELLVLFELLVLLYYWLSVSVMFYLMDRCHYEVLQLLLILLVLYLEGKI
LLNSFEVVFLLIMPLTDFLHYILYCLLYLLFCTLHFMILLVQAILLVFQVITIELHLLHIFYLKILLF
LYLFLYVLLYSLCLMFGIVIIILWLILCKLLLLLYLNDTYYLSMLFDLYLINYVLRCLVLFLLCYY
LLQIVDLEVYNLDHLVKLSEFLLLISFCNVLNTLKIHLYYVNVLYYTLVILLLYYLLVTIIVLIYLI
NLNYFIKLTKYIKIIIYIFYLRYYFSILGFQFIIYIILCITLHLALANLLFPLVWNNDLLIHWFFESKK
EIRNIFLLLLILFLKNIHFALPFSCIKMNDIKNKYLNYSYVILSYIATIIRSCINTCTCLISCSLCYSC
KKNYGYAKKIRSCCRLLWTIASICCLKTFIKRICSSYTIYCSFLFRSCNNFNFCIIRLRCYTLWSW
FRDKRHEFRYILHVSCVIFSYIRYSISWLKCEIRFSRFSKYSSINLINIKFSYINSNYDNRKFKFNCL
YISKSYLIYTTFISCVYNIFHRIYSDKSSFFSRGPANLVWSGYMSQIARKPFYFKNKRQLAGNLIP
NKYIIKLFIDTQFNIYIYLFIYMIKDIVNHRCESTKKKHMGTHLPLLGESELVSGFMTEHAAVVF
VFFFLAEYGSIVLMCILTSILFIGGYLLFEISYVFTVVNYIFFELFFIDVTFVEVQSLYTDFLNNSIIE
GLLYGFNLGLKSSLMIFTFIARASFPRIRFDQLMGFCTVLLPINFAIIILVPCVLYSFNLLPVNIPLF
LTHPPALLPQGYSTYEEGNRSRTILVNNYTYTKTIYHTRECNYTLFLYSTLLAYYYLYMIIILLNL
LGYINKKFVNFFHHFLIQNTNF
>YN010
ITILKNIKSYIKIIHLYSINLKGIFSILYLVLLLFSLLQVFLLLGSLLNRWYARLLIKVISFYLLQYLV
LEGFIIILKKCSFIHLTINTKKLKIILLMVKLLIETIKYMLNLIIQMLCLVLWQLFLLLHFYYSCLIK
NDVWVLYWYNLYLAINEYDIIYRVCICYSYFIFSHFYICSSSLSRKIYFRVWFPFFRAKNTIRYKI
LYFCFSLFTFRFRNIINFPFRSVCWYLWSFSYFNFYSYNNYRIYIIRKRSNRQQTKIIYTIERLPYR
VCWNRGFVKLRQKTKKPTKGLMGSYKKMITYIYSYYIFTITRIIIYIYIILLYIKIIIIIRYNYSILLY
KNIIYPIINFIFFLIIIFFYIMLTHLQMKRTKYCCYSSGYRKIFLYYLFLLGSFSVRGYMDGRRVFIL
MNSGFKLTLFMNLNYNYINVGYDRIMYSLRVKRDTPTYNTYWFRVEYIYLRVKNIYIQYIINIVI
YWYGLNQVYILSFDKHLFFYLLLLLYCEINNYNIILHSTYLGILSYLAINDSNQILFSLVWNNDL
LIHWFFDSKKRVNNFLDQKYYFLQKAYAFHFFDKQLLLIFGCLLVLTITKFFQFLYKFKEYKVIH
YNILTLSMRHNQVTLVTCEILVHYLFVYKLLPVLHLCIIVLVWKLLTQSIEMLITGDFVIYIVIQL
LLSFSCIYTEEVYITDHIELLVLFELLVLLYYWLSVSVMFYLMDRCHYEVLQLLLILLVLYLEGKI
LLNSFEVVFLLIMPLTDFLHYILYCLLYLLFCTLHFMILLVQAILLVFQVITIELHLLHIFYLKILLF
LYLFLYVLLYSLCLMFGIVIIILWLILCKLLLLLYLNDTYYLSMLFDLYLINYVLRCLVLFLLCYY
LLQIVDLEVYNLDHLVKLSEFLLLISFCNVLNTLKIHLYYVNVLYYTLVILLLYYLLVTIIVLIYLI
NLNYFIKLTKYIKIIIYIFYLRYYFSILGFQFIIYIILCITLHLALANLLFPLVWNNDLLIHWFFESKK
EIRNIFLLLLILFLKNIHFALPFSCIKMNDIKNKYLNYSYVILSYIATIIRSCINTCTCLISCSLCYSC
KKNYGYAKKIRSCCRLLWTIASICCLKTFIKRICSSYTIYCSFLFRSCNNFNFCIIRLRCYTLWSW
FRDKRHEFRYILHVSCVIFSYIRYSISWLKCEIRFSRFSKYSSINLINIKFSYINSNYDNRKFKFNCL
YISKSYLIYTTFISCVYNIFHRIYSDKSSFFSRGPANLVWSGYMSQIARKPFYFKNKRQLAGNLIP
NKYIIKLFIDTQFNIYIYLFIYMIKDIVNHRCESTKKKHMGTHLPLLGESELVSGFMTEHAAVVF

VFFFLAEYGSIVLMCILTSILFIGGYLLFEISYVFTVVNYIFFELFFIDVTFVEVQSLYTDFLNNSIIE
GLLYGFNLGLKSSLMIFTFIARASFPRIRFDQLMGFCTVLLPINFAIIILVPCVLYSFNLLPVNIPLF
LTHPPALLPQGYSTYEEGNRSRTILVNNYTYTKTIYHTRECNYTLFLYSTLLAYYYLYMIIILLNL
LGYINKKFVNFFHHFLIQNTNF
>YN029
ITILKNIKSYIKIIHLYSINLKGIFSILYLVLLLFSLLQVFLLLGSLLNRWYARLLIKVISFYLLQYLV
LEGFIIILKKCSFIHLTINTKKLKIILLMVKLLIETIKYMLNLIIQMLCLVLWQLFLLLHFYYSCLIK
NDVWVLYWYNLYLAINEYDIIYRVCICYSYFIFSHFYICSSSLSRKIYFRVWFPFFRAKNTIRYKI
LYFCFSLFTFRFRNIINFPPFRSVCWYLWSFSYFNFYSYNNYRIYIIRKRSNRQQTKIIYTIERLPYR
VCWNRGFVKLRQKTKKPTKGLMGSYKKMITYIYSYYIFTITRIIIYIYIILLYIKIIIIIRYNYSILLY
KNIIYPIINFIFFLIIIFFYIMLTHLQMKRTKYCCYSSGYRKIFLYYLFLLGSFSVRGYMDGRRVFIL
MNSGFKLTLFMNLNYNYINVGYDRIMYSLRVKRDTPTYNTYWFRVEYIYLRVKNIYIQYIINIVI
YWYGLNQVYILSFDKHLFFYLLLLLYCEINNYNIILHSTYLGILSYLAINDSNQILFSLVWNNDL
LIHWFFDSKKRVNNFLDQKYYFLQKAYAFHFFDKQLLLIFGCLLVLTITKFFQFLYKFKEYKVIH
YNILTLSMRHNQVTLVTCEILVHYLFVYKLLPVLHLCIIVLVWKLLTQSIEMLITGDFVIYIVIQL
LLSFSCIYTEEVYITDHIELLVLFELLVLLYYWLSVSVMFYLMDRCHYEVLQLLLILLVLYLEGKI
LLNSFEVVFLLIMPLTDFLHYILYCLLYLLFCTLHFMILLVQAILLVFQVITIELHLLHIFYLKILLF
LYLFLYVLLYSLCLMFGIVIIILWLILCKLLLLLYLNDTYYLSMLFDLYLINYVLRCLVLFLLCYY
LLQIVDLEVYNLDHLVKLSEFLLLISFCNVLNTLKIHLYYVNVLYYTLVILLLYYLLVTIIVLIYLI
NLNYFIKLTKYIKIIIYIFYLRYYFSILGFQFIIYIILCITLHLALANLLFPLVWNNDLLIHWFFESKK
EIRNIFLLLLILFLKNIHFALPFSCIKMNDIKNKYLNYSYVILSYIATIIRSCINTCTCLISCSLCYSC
KKNYGYAKKIRSCCRLLWTIASICCLKTFIKRICSSYTIYCSFLFRSCNNFNFCIIRLRCYTLWSW
FRDKRHEFRYILHVSCVIFSYIRYSISWLKCEIRFSRFSKYSSINLINIKFSYINSNYDNRKFKFNCL
YISKSYLIYTTFISCVYNIFHRIYSDKSSFFSRGPANLVWSGYMSQIARKPFYFKNKRQLAGNLIP
NKYIIKLFIDTQFNIYIYLFIYMIKDIVNHRCESTKKKHMGTHLPLLGESELVSGFMTEHAAVVF
VFFFLAEYGSIVLMCILTSILFIGGYLLFEISYVFTVVNYIFFELFFIDVTFVEVQSLYTDFLNNSIIE
GLLYGFNLGLKSSLMIFTFIARASFPRIRFDQLMGFCTVLLPINFAIIILVPCVLYSFNLLPVNIPLF
LTHPPALLPQGYSTYEEGNRSRTILVNNYTYTKTIYHTRECNYTLFLYSTLLAYYYLYMIIILLNL
LGYINKKFVNFFHHFLIQNTNF
>YN030
ITILKNIKSYIKIIHLYSINLKGIFSILYLVLLLFSLLQVFLLLGSLLNRWYARLLIKVISFYLLQYLV
LEGFIIILKKCSFIHLTINTKKLKIILLMVKLLIETIKYMLNLIIQMLCLVLWQLFLLLHFYYSCLIK
NDVWVLYWYNLYLAINEYDIIYRVCICYSYFIFSHFYICSSSLSRKIYFRVWFPFFRAKNTIRYKI
LYFCFSLFTFRFRNIINFPPFRSVCWYLWSFSYFNFYSYNNYRIYIIRKRSNRQQTKIIYTIERLPYR
VCWNRGFVKLRQKTKKPTKGLMGSYKKMITYIYSYYIFTITRIIIYIYIILLYIKIIIIIRYNYSILLY
KNIIYPIINFIFFLIIIFFYIMLTHLQMKRTKYCCYSSGYRKIFLYYLFLLGSFSVRGYMDGRRVFIL
MNSGFKLTLFMNLNYNYINVGYDRIMYSLRVKRDTPTYNTYWFRVEYIYLRVKNIYIQYIINIVI
YWYGLNQVYILSFDKHLFFYLLLLLYCEINNYNIILHSTYLGILSYLAINDSNQILFSLVWNNDL
LIHWFFDSKKRVNNFLDQKYYFLQKAYAFHFFDKQLLLIFGCLLVLTITKFFQFLYKFKEYKVIH
YNILTLSMRHNQVTLVTCEILVHYLFVYKLLPVLHLCIIVLVWKLLTQSIEMLITGDFVIYIVIQL
LLSFSCIYTEEVYITDHIELLVLFELLVLLYYWLSVSVMFYLMDRCHYEVLQLLLILLVLYLEGKI
LLNSFEVVFLLIMPLTDFLHYILYCLLYLLFCTLHFMILLVQAILLVFQVITIELHLLHIFYLKILLF
LYLFLYVLLYSLCLMFGIVIIILWLILCKLLLLLYLNDTYYLSMLFDLYLINYVLRCLVLFLLCYY
LLQIVDLEVYNLDHLVKLSEFLLLISFCNVLNTLKIHLYYVNVLYYTLVILLLYYLLVTIIVLIYLI

NLNYFIKLTKYIKIIIYIFYLRYYFSILGFQFIIYIILCITLHLALANLLFPLVWNNDLLIHWFFESKK
EIRNIFLLLLILFLKNIHFALPFSCIKMNDIKNKYLNYSYVILSYIATIIRSCINTCTCLISCSLCYSC
KKNYGYAKKIRSCCRLLWTIASICCLKTFIKRICSSYTIYCSFLFRSCNNFNFCIIRLRCYTLWSW
FRDKRHEFRYILHVSCVIFSYIRYSISWLKCEIRFSRFSKYSSINLINIKFSYINSNYDNRKFKFNCL
YISKSYLIYTTFISCVYNIFHRIYSDKSSFFSRGPANLVWSGYMSQIARKPFYFKNKRQLAGNLIP
NKYIIKLFIDTQFNIYIYLFIYMIKDIVNHRCESTKKKHMGTHLPLLGESELVSGFMTEHAAVVF
VFFFLAEYGSIVLMCILTSILFIGGYLLFEISYVFTVVNYIFFELFFIDVTFVEVQSLYTDFLNNSIIE
GLLYGFNLGLKSSLMIFTFIARASFPRIRFDQLMGFCTVLLPINFAIIILVPCVLYSFNLLPVNIPLF
LTHPPALLPQGYSTYEEGNRSRTILVNNYTYTKTIYHTRECNYTLFLYSTLLAYYYLYMIIILLNL
LGYINKKFVNFFHHFLIQNTNF
>YN040
ITILKNIKSYIKIIHLYSINLKGIFSILYLVLLLFSLLQVFLLLGSLLNRWYARLLIKVISFYLLQYLV
LEGFIIILKKCSFIHLTINTKKLKIILLMVKLLIETIKYMLNLIIQMLCLVLWQLFLLLHFYYSCLIK
NDVWVLYWYNLYLAINEYDIIYRVCICYSYFIFSHFYICSSSLSRKIYFRVWFPFFRAKNTIRYKI
LYFCFSLFTFRFRNIINFPFRSVCWYLWSFSYFNFYSYNNYRIYIIRKRSNRQQTKIIYTIERLPYR
VCWNRGFVKLRQKTKKPTKGLMGSYKKMITYIYSYYIFTITRIIIYIYIILLYIKIIIIIRYNYSILLY
KNIIYPIINFIFFLIIIFFYIMLTHLQMKRTKYCCYSSGYRKIFLYYLFLLGSFSVRGYMDGRRVFIL
MNSGFKLTLFMNLNYNYINVGYDRIMYSLRVKRDTPTYNTYWFRVEYIYLRVKNIYIQYIINIVI
YWYGLNQVYILSFDKHLFFYLLLLLYCEINNYNIILHSTYLGILSYLAINDSNQILFSLVWNNDL
LIHWFFDSKKRVNNFLDQKYYFLQKAYAFHFFDKQLLLIFGCLLVLTITKFFQFLYKFKEYKVIH
YNILTLSMRHNQVTLVTCEILVHYLFVYKLLPVLHLCIIVLVWKLLTQSIEMLITGDFVIYIVIQL
LLSFSCIYTEEVYITDHIELLVLFELLVLLYYWLSVSVMFYLMDRCHYEVLQLLLILLVLYLEGKI
LLNSFEVVFLLIMPLTDFLHYILYCLLYLLFCTLHFMILLVQAILLVFQVITIELHLLHIFYLKILLF
LYLFLYVLLYSLCLMFGIVIIILWLILCKLLLLLYLNDTYYLSMLFDLYLINYVLRCLVLFLLCYY
LLQIVDLEVYNLDHLVKLSEFLLLISFCNVLNTLKIHLYYVNVLYYTLVILLLYYLLVTIIVLIYLI
NLNYFIKLTKYIKIIIYIFYLRYYFSILGFQFIIYIILCITLHLALANLLFPLVWNNDLLIHWFFESKK
EIRNIFLLLLILFLKNIHFALPFSCIKMNDIKNKYLNYSYVILSYIATIIRSCINTCTCLISCSLCYSC
KKNYGYAKKIRSCCRLLWTIASICCLKTFIKRICSSYTIYCSFLFRSCNNFNFCIIRLRCYTLWSW
FRDKRHEFRYILHVSCVIFSYIRYSISWLKCEIRFSRFSKYSSINLINIKFSYINSNYDNRKFKFNCL
YISKSYLIYTTFISCVYNIFHRIYSDKSSFFSRGPANLVWSGYMSQIARKPFYFKNKRQLAGNLIP
NKYIIKLFIDTQFNIYIYLFIYMIKDIVNHRCESTKKKHMGTHLPLLGESELVSGFMTEHAAVVF
VFFFLAEYGSIVLMCILTSILFIGGYLLFEISYVFTVVNYIFFELFFIDVTFVEVQSLYTDFLNNSIIE
GLLYGFNLGLKSSLMIFTFIARASFPRIRFDQLMGFCTVLLPINFAIIILVPCVLYSFNLLPVNIPLF
LTHPPALLPQGYSTYEEGNRSRTILVNNYTYTKTIYHTRECNYTLFLYSTLLGYYYLYMIIILLNL
LGYINKKFVNFFHHFLIQNTNF
>YN047
ITILKNIKSYIKIIHLYSINLKGIFSILYLVLLLFSLLQVFLLLGSLLNRWYARLLIKVISFYLLQYLV
LEGFIIILKKCSFIHLTINTKKLKIILLMVKLLIETIKYMLNLIIQMLCLVLWQLFLLLHFYYSCLIK
NDVWVLYWYNLYLAINEYDIIYRVCICYSYFIFSHFYICSSSLSRKIYFRVWFPFFRAKNTIRYKI
LYFCFSLFTFRFRNIINFPFRSVCWYLWSFSYFNFYSYNNYRIYIIRKRSNRQQTKIIYTIERLPYR
VCWNRGFVKLRQKTKKPTKGLMGSYKKMITYIYSYYIFTITRIIIYIYIILLYIKIIIIIRYNYSILLY
KNIIYPIINFIFFLIIIFFYIMLTHLQMKRTKYCCYSSGYRKIFLYYLFLLGSFSVRGYMDGRRVFIL
MNSGFKLTLFMNLNYNYINVGYDRIMYSLRVKRDTPTYNTYWFRVEYIYLRVKNIYIQYIINIVI
YWYGLNQVYILSFDKHLFFYLLLLLYCEINNYNIILHSTYLGILSYLAINDSNQILFSLVWNNDL

LIHWFFDSKKRVNNFLDQKYYFLQKAYAFHFFDKQLLLIFGCLLVLTITKFFQFLYKFKEYKVIH
YNILTLSMRHNQVTLVTCEILVHYLFVYKLLPVLHLCIIVLVWKLLTQSIEMLITGDFVIYIVIQL
LLSFSCIYTEEVYITDHIELLVLFELLVLLYYWLSVSVMFYLMDRCHYEVLQLLLILLVLYLEGKI
LLNSFEVVFLLIMPLTDFLHYILYCLLYLLFCTLHFMILLVQAILLVFQVITIELHLLHIFYLKILLF
LYLFLYVLLYSLCLMFGIVIIILWLILCKLLLLLYLNDTYYLSMLFDLYLINYVLRCLVLFLLCYY
LLQIVDLEVYNLDHLVKLSEFLLLISFCNVLNTLKIHLYYVNVLYYTLVILLLYYLLVTIIVLIYLI
NLNYFIKLTKYIKIIIYIFYLRYYFSILGFQFIIYIILCITLHLALANLLFPLVWNNDLLIHWFFDSKK
EIRNIFLLLLILFLKNIHFALPFSCIKMNDIKNKYLNYSYVILSYIATIIRSCINTCTCLISCSLCYSC
KKNYGYAKKIRSCCRLLWTIASICCLKTFIKRICSSYTIYCSFLFRSCNNFNFCIIRLRCYTLWSW
FRDKRHEFRYILHVSCVIFSYIRYSISWLKCEIRFSRFSKYSSINLINIKFSYINSNYDNRKFKFNCL
YISKSYLIYTTFISCVYNIFHRIYSDKSSFFSRGPANLVWSGYMSQIARKPFYFKNKRQLAGNLIP
NKYIIKLFIDTQFNIYIYLFIYMIKDIVNHRCESTKKKHMGTHLPLLGESELVSGFMTEHAAVVF
VFFFLAEYGSIVLMCILTSILFIGGYLLFEISYVFTVVNYIFFELFFIDVTFVEVQSLYTDFLNNSIIE
GLLYGFNLGLKSSLMIFTFIARASFPRIRFDQLMGFCTVLLPINFAIIILVPCVLYSFNLLPVNIPLF
LTHPPALLPQGYSTYEEGNRSRTILVNNYTYTKTIYHTRECNYTLFLYSTLLAYYYLYMIIILLNL
LGYINKKFVNFFHHFLIQNTNF
>YN084
ITILKNIKSYIKIIHLYSINLKGIFSILYLVLLLFSLLQVFLLLGSLLNRWYARLLIKVISFYLLQYLV
LEGFIIILKKCSFIHLTINTKKLKIILLMVKLLIETIKYMLNLIIQMLCLVLWQLFLLLHFYYSCLIK
NDVWVLYWYNLYLAINEYDIIYRVCICYSYFIFSHFYICSSSLSRKIYFRVWFPFFRAKNTIRYKI
LYFCFSLFTFRFRNIINFPFRSVCWYLWSFSYFNFYSYNNYRIYIIRKRSNRQQTKIIYTIERLPYR
VCWNRGFVKLRQKTKKPTKGLMGSYKKMITYIYSYYIFTITRIIIYIYIILLYIKIIIIIRYNYSILLY
KNIIYPIINFIFFLIIIFFYIMLTHLQMKRTKYCCYSSGYRKIFLYYLFLLGSFSVRGYMDGRRVFIL
MNSGFKLTLFMNLNYNYINVGYDRIMYSLRVKRDTPTYNTYWFRVEYIYLRVKNIYIQYIINIVI
YWYGLNQVYILSFDKHLFFYLLLLLYCEINNYNIILHSTYLGILSYLAINDSNQILFSLVWNNDL
LIHWFFDSKKRVNNFLDQKYYFLQKAYAFHFFDKQLLLIFGCLLVLTITKFFQFLYKFKEYKVIH
YNILTLSMRHNQVTLVTCEILVHYLFVYKLLPVLHLCIIVLVWKLLTQSIEMLITGDFVIYIVIQL
LLSFSCIYTEEVYITDHIELLVLFELLVLLYYWLSVSVMFYLMDRCHYEVLQLLLILLVLYLEGKI
LLNSFEVVFLLIMPLTDFLHYILYCLLYLLFCTLHFMILLVQAILLVFQVITIELHLLHIFYLKILLF
LYLFLYVLLYSLCLMFGIVIIILWLILCKLLLLLYLNDTYYLSMLFDLYLINYVLRCLVLFLLCYY
LLQIVDLEVYNLDHLVKLSEFLLLISFCNVLNTLKIHLYYVNVLYYTLVILLLYYLLVTIIVLIYLI
NLNYFIKLTKYIKIIIYIFYLRYYFSILGFQFIIYIILCITLHLALANLLFPLVWNNDLLIHWFFESKK
EIRNIFLLLLILFLKNIHFALPFSCIKMNDIKNKYLNYSYVILSYIATIIRSCINTCTCLISCSLCYSC
KKNYGYAKKIRSCCRLLWTIASICCLKTFIKRICSSYTIYCSFLFRSCNNFNFCIIRLRCYTLWSW
FRDKRHEFRYILHVSCVIFSYIRYSISWLKCEIRFSRFSKYSSINLINIKFSYINSNYDNRKFKFNCL
YISKSYLIYTTFISCVYNIFHRIYSDKSSFFSRGPANLVWSGYMSQIARKPFYFKNKRQLAGNLIP
NKYIIKLFIDTQFNIYIYLFIYMIKDIVNHRCESTKKKHMGTHLPLLGESELVSGFMTEHAAVVF
VFFFLAEYGSIVLMCILTSILFIGGYLLFEISYVFTVVNYIFFELFFIDVTFVEVQSLYTDFLNNSIIE
GLLYGFNLGLKSSLMIFTFIARASFPRIRFDQLMGFCTVLLPINFAIIILVPCVLYSFNLLPVNIPLF
LTHPPALLPQGYSTYEEGNRSRTILVNNYTYTKTIYHTRECNYTLFLYSTLLAYYYLYMIIILLNL
LGYINKKFVNFFHHFLIQNTNF
>YN093
ISILKNIKSYIKIIHLYSINLKGIFSILYLVLLLFSLLQVFLLLGSLLNRWYARLLIKVISFYLLQYLV
LEGFIIILKKCSFIHLTINTKKLKIILLMVKLLIETIKYMLNLIIQMLCLVLWQLFLLLHFYYSCLIK

NDVWVLYWYNLYLAINEYDIIYRVCICYSYFIFSHFYICSSSLSRKIYFRVWFPFFRAKNTIRYKI
LYFCFSLFTFRFRNIINFPFRSVCWYLWSFSYFNFYSYNNYRIYIIRKRSNRQQTKIIYTIERLPYR
VCWNRGFVKLRQKTKKPTKGLMGSYKKMITYIYSYYIFTITRIIIYIYIILLYIKIIIIIRYNYSILLY
KNIIYPIINFIFFLIIIFFYIMLTHLQMKRTKYCCYSSGYRKIFLYYLFLLGSFSVRGYMDGRRVFIL
MNSGFKLTLFMNLNYNYINVGYDRIMYSLRVKRDTPTYNTYWFRVEYIYLRVKNIYIQYIINIVI
YWYGLNQVYILSFDKHLFFYLLLLLYCEINNYNIILHSTYLGILSYLAINDSNQILFSLVWNNDL
LIHWFFDSKKRVNNFLDQKYYFLQKAYAFHFFDKQLLLIFGCLLVLTITKFFQFLYKFKEYKVIH
YNILTLSMRHNQVTLVTCEILVHYLFVYKLLPVLHLCIIVLVWKLLTQSIEMLITGDFVIYIVIQL
LLSFSCIYTEEVYITDHIELLVLFELLVLLYYWLSVSVMFYLMDRCHYEVLQLLLILLVLYLEGKI
LLNSFEVVFLLIMPLTDFLHYILYCLLYLLFCTLHFMILLVQAILLVFQVITIELHLLHIFYLKILLF
LYLFLYVLLYSLCLMFGIVIIILWLILCKLLLLLYLNDTYYLSMLFDLYLINYVLRCLVLFLLCYY
LLQIVDLEVYNLDHLVKLSEFLLLISFCNVLNTLKIHLYYVNVLYYTLVILLLYYLLVTIIVLIYLI
NLNYFIKLTKYIKIIIYIFYLRYYFSILGFQFIIYIILCITLHLALANLLFPLVWNNDLLIHWFFESKK
EIRNIFLLLLILFLKNIHFALPFSCIKMNDIKNKYLNYSYVILSYIATIIRSCINTCTCLISCSLCYSC
KKNYGYAKKIRSCCRLLWTIASICCLKTFIKRICSSYTIYCSFLFRSCNNFNFCIIRLRCYTLWSW
FRDKRHEFRYILHVSCVIFSYIRYSISWLKCEIRFSRFSKYSSINLINIKFSYINSNYDNRKFKFNCL
YISKSYLIYTTFISCVYNIFHRIYSDKSSFFSRGPANLVWSGYMSQIARKPFYFKNKRQLAGNLIP
NKYIIKLFIDTQFNIYIYLFIYMIKDIVNHRCESTKKKHMGTHLPLLGESELVSGFMTEHAAVVF
VFFFLAEYGSIVLMCILTSILFIGGYLLFEISYVFTVVNYIFFELFFIDVTFVEVQSLYTDFLNNSIIE
GLLYGFNLGLKSSLMIFTFIARASFPRIRFDQLMGFCTVLLPINFAIIILVPCVLYSFNLLPVNIPLF
LTHPPALLPQGYSTYEEGNRSRTILVNNYTYTKTIYHTRECNYTLFLYSTLLGYYYLYMIIILLNL
LGYINKKFVNFFHHFLIQNTNF
>YN094
ITILKNIKSYIKIIHLYSINLKGIFSILYLVLLLFSLLQVFLLLGSLLNRWYARLLIKVISFYLLQYLV
LEGFIIILKKCSFIHLTINTKKLKIILLMVKLLIETIKYMLNLIIQMLCLVLWQLFLLLHFYYSCLIK
NDVWVLYWYNLYLAINEYDIIYRVCICYSYFIFSHFYICSSSLSRKIYFRVWFPFFRAKNTIRYKI
LYFCFSLFTFRFRNIINFPFRSVCWYLWSFSYFNFYSYNNYRIYIIRKRSNRQQTKIIYTIERLPYR
VCWNRGFVKLRQKTKKPTKGLMGSYKKMITYIYSYYIFTITRIIIYIYIILLYIKIIIIIRYNYSILLY
KNIIYPIINFIFFLIIIFFYIMLTHLQMKRTKYCCYSSGYRKIFLYYLFLLGSFSVRGYMDGRRVFIL
MNSGFKLTLFMNLNYNYINVGYDRIMYSLRVKRDTPTYNTYWFRVEYIYLRVKNIYIQYIINIVI
YWYGLNQVYILSFDKHLFFYLLLLLYCEINNYNIILHSTYLGILSYLAINDSNQILFSLVWNNDL
LIHWFFDSKKRVNNFLDQKYYFLQKAYAFHFFDKQLLLIFGCLLVLTITKFFQFLYKFKEYKVIH
YNILTLSMRHNQVTLVTCEILVHYLFVYKLLPVLHLCIIVLVWKLLTQSIEMLITGDFVIYIVIQL
LLSFSCIYTEEVYITDHIELLVLFELLVLLYYWLSVSVMFYLMDRCHYEVLQLLLILLVLYLEGKI
LLNSFEVVFLLIMPLTDFLHYILYCLLYLLFCTLHFMILLVQAILLVFQVITIELHLLHIFYLKILLF
LYLFLYVLLYSLCLMFGIVIIILWLILCKLLLLLYLNDTYYLSMLFDLYLINYVLRCLVLFLLCYY
LLQIVDLEVYNLDHLVKLSEFLLLISFCNVLNTLKIHLYYVNVLYYTLVILLLYYLLVTIIVLIYLI
NLNYFIKLTKYIKIIIYIFYLRYYFSILGFQFIIYIILCITLHLALANLLFPLVWNNDLLIHWFFESKK
EIRNIFLLLLILFLKNIHFALPFSCIKMNDIKNKYLNYSYVILSYIATIIRSCINTCTCLISCSLCYSC
KKNYGYAKKIRSCCRLLWTIASICCLKTFIKRICSSYTIYCSFLFRSCNNFNFCIIRLRCYTLWSW
FRDKRHEFRYILHVSCVIFSYIRYSISWLKCEIRFSRFSKYSSINLINIKFSYINSNYDNRKFKFNCL
YISKSYLIYTTFISCVYNIFHRIYSDKSSFFSRGPANLVWSGYMSQIARKPFYFKNKRQLAGNLIP
NKYIIKLFIDTQFNIYIYLFIYMIKDIVNHRCESTKKKHMGTHLPLLGESELVSGFMTEHAAVVF
VFFFLAEYGSIVLMCILTSILFIGGYLLFEISYVFTVVNYIFFELFFIDVTFVEVQSLYTDFLNNSIIE

GLLYGFNLGLKSSLMIFTFIARASFPRIRFDQLMGFCTVLLPINFAIIILVPCVLYSFNLLPVNIPLF
LTHPPALLPQGYSTYEEGNRSRTILVNNYTYTKTIYHTRECNYTLFLYSTLLGYYYLYMIIILLNL
LGYINKKFVNFFHHFLIQNTNF
>YN097
ITILKNIKSYIKIIHLYSINLKGIFSILYLVLLLFSLLQVFLLLGSLLNRWYARLLIKVISFYLLQYLV
LEGFIIILKKCSFIHLTINTKKLKIILLMVKLLIETIKYMLNLIIQMLCLVLWQLFLLLHFYYSCLIK
NDVWVLYWYNLYLAINEYDIIYRVCICYSYFIFSHFYICSSSLSRKIYFRVWFPFFRAKNTIRYKI
LYFCFSLFTFRFRNIINFPFRSVCWYLWSFSYFNFYSYNNYRIYIIRKRSNRQQTKIIYTIERLPYR
VCWNRGFVKLRQKTKKPTKGLMGSYKKMITYIYSYYIFTITRIIIYIYIILLYIKIIIIIRYNYSILLY
KNIIYPIINFIFFLIIIFFYIMLTHLQMKRTKYCCYSSGYRKIFLYYLFLLGSFSVRGYMDGRRVFIL
MNSGFKLTLFMNLNYNYINVGYDRIMYSLRVKRDTPTYNTYWFRVEYIYLRVKNIYIQYIINIVI
YWYGLNQVYILSFDKHLFFYLLLLLYCEINNYNIILHSTYLGILSYLAINDSNQILFSLVWNNDL
LIHWFFDSKKRVNNFLDQKYYFLQKAYAFHFFDKQLLLIFGCLLVLTITKFFQFLYKFKEYKVIH
YNILTLSMRHNQVTLVTCEILVHYLFVYKLLPVLHLCIIVLVWKLLTQSIEMLITGDFVIYIVIQL
LLSFSCIYTEEVYITDHIELLVLFELLVLLYYWLSVSVMFYLMDRCHYEVLQLLLILLVLYLEGKI
LLNSFEVVFLLIMPLTDFLHYILYCLLYLLFCTLHFMILLVQAILLVFQVITIELHLLHIFYLKILLF
LYLFLYVLLYSLCLMFGIVIIILWLILCKLLLLLYLNDTYYLSMLFDLYLINYVLRCLVLFLLCYY
LLQIVDLEVYNLDHLVKLSEFLLLISFCNVLNTLKIHLYYVNVLYYTLVILLLYYLLVTIIVLIYLI
NLNYFIKLTKYIKIIIYIFYLRYYFSILGFQFIIYIILCITLHLALANLLFPLVWNNDLLIHWFFESKK
EIRNIFLLLLILFLKNIHFALPFSCIKMNDIKNKYLNYSYVILSYIATIIRSCINTCTCLISCSLCYSC
KKNYGYAKKIRSCCRLLWTIASICCLKTFIKRICSSYTIYCSFLFRSCNNFNFCIIRLRCYTLWSW
FRDKRHEFRYILHVSCVIFSYIRYSISWLKCEIRFSRFSKYSSINLINIKFSYINSNYDNRKFKFNCL
YISKSYLIYTTFISCVYNIFHRIYSDKSSFFSRGPANLVWSGYMSQIARKPFYFKNKRQLAGNLIP
NKYIIKLFIDTQFNIYIYLFIYMIKDIVNHRCESTKKKHMGTHLPLLGESELVSGFMTEHAAVVF
VFFFLAEYGSIVLMCILTSILFIGGYLLFEISYVFTVVNYIFFELFFIDVTFVEVQSLYTDFLNNSIIE
GLLYGFNLGLKSSLMIFTFIARASFPRIRFDQLMGFCTVLLPINFAIIILVPCVLYSFNLLPVNIPLF
LTHPPALLPQGYSTYEEGNRSRTILVNNYTYTKTIYHTRECNYTLFLYSTLLAYYYLYMIIILLNL
LGYINKKFVNFFHHFLIQNTNF
>YN137
ITILKNIKSYIKIIHLYSINLKGIFSILYLVLLLFSLLQVFLLLGSLLNRWYARLLIKVISFYLLQYLV
LEGFIIILKKCSFIHLTINTKKLKIILLMVKLLIETIKYMLNLIIQMLCLVLWQLFLLLHFYYSCLIK
NDVWVLYWYNLYLAINEYDIIYRVCICYSYFIFSHFYICSSSLSRKIYFRVWFPFFRAKNTIRYKI
LYFCFSLFTFRFRNIINFPFRSVCWYLWSFSYFNFYSYNNYRIYIIRKRSNRQQTKIIYTIERLPYR
VCWNRGFVKLRQKTKKPTKGLMGSYKKMITYIYSYYIFTITRIIIYIYIILLYIKIIIIIRYNYSILLY
KNIIYPIINFIFFLIIIFFYIMLTHLQMKRTKYCCYSSGYRKIFLYYLFLLGSFSVRGYMDGRRVFIL
MNSGFKLTLFMNLNYNYINVGYDRIMYSLRVKRDTPTYNTYWFRVEYIYLRVKNIYIQYIINIVI
YWYGLNQVYILSFDKHLFFYLLLLLYCEINNYNIILHSTYLGILSYLAINDSNQILFSLVWNNDL
LIHWFFDSKKRVNNFLDQKYYFLQKAYAFHFFDKQLLLIFGCLLVLTITKFFQFLYKFKEYKVIH
YNILTLSMRHNQVTLVTCEILVHYLFVYKLLPVLHLCIIVLVWKLLTQSIEMLITGDFVIYIVIQL
LLSFSCIYTEEVYITDHIELLVLFELLVLLYYWLSVSVMFYLMDRCHYEVLQLLLILLVLYLEGKI
LLNSFEVVFLLIMPLTDFLHYILYCLLYLLFCTLHFMILLVQAILLVFQVITIELHLLHIFYLKILLF
LYLFLYVLLYSLCLMFGIVIIILWLILCKLLLLLYLNDTYYLSMLFDLYLINYVLRCLVLFLLCYY
LLQIVDLEVYNLDHLVKLSEFLLLISFCNVLNTLKIHLYYVNVLYYTLVILLLYYLLVTIIVLIYLI
NLNYFIKLTKYIKIIIYIFYLRYYFSILGFQFIIYIILCITLHLALANLLFPLVWNNDLLIHWFFESKK

EIRNIFLLLLILFLKNIHFALPFSCIKMNDIKNKYLNYSYVILSYIATIIRSCINTCTCLISCSLCYSC
KKNYGYAKKIRSCCRLLWTIASICCLKTFIKRICSSYTIYCSFLFRSCNNFNFCIIRLRCYTLWSW
FRDKRHEFRYILHVSCVIFSYIRYSISWLKCEIRFSRFSKYSSINLINIKFSYINSNYDNRKFKFNCL
YISKSYLIYTTFISCVYNIFHRIYSDKSSFFSRGPANLVWSGYMSQIARKPFYFKNKRQLAGNLIP
NKYIIKLFIDTQFNIYIYLFIYMIKDIVNHRCESTKKKHMGTHLPLLGESELVSGFMTEHAAVVF
VFFFLAEYGSIVLMCILTSILFIGGYLLFEISYVFTVVNYIFFELFFIDVTFVEVQSLYTDFLNNSIIE
GLLYGFNLGLKSSLMIFTFIARASFPRIRFDQLMGFCTVLLPINFAIIILVPCVLYSFNLLPVNIPLF
LTHPPALLPQGYSTYEEGNRSRTILVNNYTYTKTIYHTRECNYTLFLYSTLLAYYYLYMIIILLNL
LGYINKKFVNFFHHFLIQNTNY
>YN153
ITILKNIKSYIKIIHLYSINLKGIFSILYLVLLLFSLLQVFLLLGSLLNRWYARLLIKVISFYLLQYLV
LEGFIIILKKCSFIHLTINTKKLKIILLMVKLLIETIKYMLNLIIQMLCLVLWQLFLLLHFYYSCLIK
NDVWVLYWYNLYLAINEYDIIYRVCICYSYFIFSHFYICSSSLSRKIYFRVWFPFFRAKNTIRYKI
LYFCFSLFTFRFRNIINFPFRSVCWYLWSFSYFNFYSYNNYRIYIIRKRSNRQQTKIIYTIERLPYR
VCWNRGFVKLRQKTKKPTKGLMGSYKKMITYIYSYYIFTITRIIIYIYIILLYIKIIIIIRYNYSILLY
KNIIYPIINFIFFLIIIFFYIMLTHLQMKRTKYCCYSSGYRKIFLYYLFLLGSFSVRGYMDGRRVFIL
MNSGFKLTLFMNLNYNYINVGYDRIMYSLRVKRDTPTYNTYWFRVEYIYLRVKNIYIQYIINIVI
YWYGLNQVVYILSFDKHLFFYLLLLLYCEINNYNIILHSTYLGILSYLAINDSNQILFSLVWNNDL
LIHWFFDSKKRVNNFLDQKYYFLQKAYAFHFFDKQLLLIFGCLLVLTITKFFQFLYKFKEYKVIH
YNILTLSMRHNQVTLVTCEILVHYLFVYKLLPVLHLCIIVLVWKLLTQSIEMLITGDFVIYIVIQL
LLSFSCIYTEEVYITDHIELLVLFELLVLLYYWLSVSVMFYLMDRCHYEVLQLLLILLVLYLEGKI
LLNSFEVVFLLIMPLTDFLHYILYCLLYLLFCTLHFMILLVQAILLVFQVITIELHLLHIFYLKILLF
LYLFLYVLLYSLCLMFGIVIIILWLILCKLLLLLYLNDTYYLSMLFDLYLINYVLRCLVLFLLCYY
LLQIVDLEVYNLDHLVKLSEFLLLISFCNVLNTLKIHLYYVNVLYYTLVILLLYYLLVTIIVLIYLI
NLNYFIKLTKYIKIIIYIFYLRYYFSILGFQFIIYIILCITLHLALANLLFPLVWNNDLLIHWFFESKK
EIRNIFLLLLILFLKNIHFALPFSCIKMNDIKNKYLNYSYVILSYIATIIRSCINTCTCLISCSLCYSC
KKNYGYAKKIRSCCRLLWTIASICCLKTFIKRICSSYTIYCSFLFRSCNNFNFCIIRLRCYTLWSW
FRDKRHEFRYILHVSCVIFSYIRYSISWLKCEIRFSRFSKYSSINLINIKFSYINSNYDNRKFKFNCL
YISKSYLIYTTFISCVYNIFHRIYSDKSSFFSRGPANLVWSGYMSQIARKPFYFKNKRQLAGNLIP
NKYIIKLFIDTQFNIYIYLFIYMIKDIVNHRCESTKKKHMGTHLPLLGESELVSGFMTEHAAVVF
VFFFLAEYGSIVLMCILTSILFIGGYLLFEISYVFTVVNYIFFELFFIDVTFVEVQSLYTDFLNNSIIE
GLLYGFNLGLKSSLMIFTFIARASFPRIRFDQLMGFCTVLLPINFAIIILVPCVLYSFNLLPVNIPLF
LTHPPALLPQGYSTYEEGNRSRTILVNNYTYTKTIYHTRECNYTLFLYSTLLAYYYLYMIIILLNL
LGYINKKFVNFFHHFLIQNTNY
>YN154
ITILKNIKSYIKIIHLYSINLKGIFSILYLVLLLFSLLQVFLLLGSLLNRWYARLLIKVISFYLLQYLV
LEGFIIILKKCSFIHLTINTKKLKIILLMVKLLIETIKYMLNLIIQMLCLVLWQLFLLLHFYYSCLIK
NDVWVLYWYNLYLAINEYDIIYRVCICYSYFIFSHFYICSSSLSRKIYFRVWFPFFRAKNTIRYKI
LYFCFSLFTFRFRNIINFPFRSVCWYLWSFSYFNFYSYNNYRIYIIRKRSNRQQTKIIYTIERLPYR
VCWNRGFVKLRQKTKKPTKGLMGSYKKMITYIYSYYIFTITRIIIYIYIILLYIKIIIIIRYNYSILLY
KNIIYPIINFIFFLIIIFFYIMLTHLQMKRTKYCCYSSGYRKIFLYYLFLLGSFSVRGYMDGRRVFIL
MNSGFKLTLFMNLNYNYINVGYDRIMYSLRVKRDTPTYNTYWFRVEYIYLRVKNIYIQYIINIVI
YWYGLNQVVYILSFDKHLFFYLLLLLYCEINNYNIILHSTYLGILSYLAINDSNQILFSLVWNNDL
LIHWFFDSKKRVNNFLDQKYYFLQKAYAFHFFDKQLLLIFGCLLVLTITKFFQFLYKFKEYKVIH

YNILTLSMRHNQVTLVTCEILVHYLFVYKLLPVLHLCIIVLVWKLLTQSIEMLITGDFVIYIVIQL
LLSFSCIYTEEVYITDHIELLVLFELLVLLYYWLSVSVMFYLMDRCHYEVLQLLLILLVLYLEGKI
LLNSFEVVFLLIMPLTDFLHYILYCLLYLLFCTLHFMILLVQAILLVFQVITIELHLLHIFYLKILLF
LYLFLYVLLYSLCLMFGIVIIILWLILCKLLLLLYLNDTYYLSMLFDLYLINYVLRCLVLFLLCYY
LLQIVDLEVYNLDHLVKLSEFLLLISFCNVLNTLKIHLYYVNVLYYTLVILLLYYLLVTIIVLIYLI
NLNYFIKLTKYIKIIIYIFYLRYYFSILGFQFIIYIILCITLHLALANLLFPLVWNNDLLIHWFFESKK
EIRNIFLLLLILFLKNIHFALPFSCIKMNDIKNKYLNYSYVILSYIATIIRSCINTCTCLISCSLCYSC
KKNYGYAKKIRSCCRLLWTIASICCLKTFIKRICSSYTIYCSFLFRSCNNFNFCIIRLRCYTLWSW
FRDKRHEFRYILHVSCVIFSYIRYSISWLKCEIRFSRFSKYSSINLINIKFSYINSNYDNRKFKFNCL
YISKSYLIYTTFISCVYNIFHRIYSDKSSFFSRGPANLVWSGYMSQIARKPFYFKNKRQLAGNLIP
NKYIIKLFIDTQFNIYIYLFIYMIKDIVNHRCESTKKKHMGTHLPLLGESELVSGFMTEHAAVVF
VFFFLAEYGSIVLMCILTSILFIGGYLLFEISYVFTVVNYIFFELFFIDVTFVEVQSLYTDFLNNSIIE
GLLYGFNLGLKSSLMIFTFIARASFPRIRFDQLMGFCTVLLPINFAIIILVPCVLYSFNLLPVNIPLF
LTHPPALLPQGYSTYEEGNRSRTILVNNYTYTKTIYHTRECNYTLFLYSTLLAYYYLYMIIILLNL
LGYINKKFVNFFHHFLIQNTNF
>YN157
ITILKNIKSYIKIIHLYSINLKGIFSILYLVLLLFSLLQVFLLLGSLLNRWYARLLIKVISFYLLQYLV
LEGFIIILKKCSFIHLTINTKKLKIILLMVKLLIETIKYMLNLIIQMLCLVLWQLFLLLHFYYSCLIK
NDVWVLYWYNLYLAINEYDIIYRVCICYSYFIFSHFYICSSSLSRKIYFRVWFPFFRAKNTIRYKI
LYFCFSLFTFRFRNIINFPFRSVCWYLWSFSYFNFYSYNNYRIYIIRKRSNRQQTKIIYTIERLPYR
VCWNRGFVKLRQKTKKPTKGLMGSYKKMITYIYSYYIFTITRIIIYIYIILLYIKIIIIIRYNYSILLY
KNIIYPIINFIFFLIIIFFYIMLTHLQMKRTKYCCYSSGYRKIFLYYLFLLGSFSVRGYMDGRRVFIL
MNSGFKLTLFMNLNYNYINVGYDRIMYSLRVKRDTPTYNTYWFRVEYIYLRVKNIYIQYIINIVI
YWYGLNQVVYILSFDKHLFFYLLLLLYCEINNYNIILHSTYLGILSYLAINDSNQILFSLVWNNDL
LIHWFFDSKKRVNNFLDQKYYFLQKAYAFHFFDKQLLLIFGCLLVLTITKFFQFLYKFKEYKVIH
YNILTLSMRHNQVTLVTCEILVHYLFVYKLLPVLHLCIIVLVWKLLTQSIEMLITGDFVIYIVIQL
LLSFSCIYTEEVYITDHIELLVLFELLVLLYYWLSVSVMFYLMDRCHYEVLQLLLILLVLYLEGKI
LLNSFEVVFLLIMPLTDFLHYILYCLLYLLFCTLHFMILLVQAILLVFQVITIELHLLHIFYLKILLF
LYLFLYVLLYSLCLMFGIVIIILWLILCKLLLLLYLNDTYYLSMLFDLYLINYVLRCLVLFLLCYY
LLQIVDLEVYNLDHLVKLSEFLLLISFCNVLNTLKIHLYYVNVLYYTLVILLLYYLLVTIIVLIYLI
NLNYFIKLTKYIKIIIYIFYLRYYFSILGFQFIIYIILCITLHLALANLLFPLVWNNDLLIHWFFESKK
EIRNIFLLLLILFLKNIHFALPFSCIKMNDIKNKYLNYSYVILSYIATIIRSCINTCTCLISCSLCYSC
KKNYGYAKKIRSCCRLLWTIASICCLKTFIKRICSSYTIYCSFLFRSCNNFNFCIIRLRCYTLWSW
FRDKRHEFRYILHVSCVIFSYIRYSISWLKCEIRFSRFSKYSSINLINIKFSYINSNYDNRKFKFNCL
YISKSYLIYTTFISCVYNIFHRIYSDKSSFFSRGPANLVWSGYMSQIARKPFYFKNKRQLAGNLIP
NKYIIKLFIDTQFNIYIYLFIYMIKDIVNHRCESTKKKHMGTHLPLLGESELVSGFMTEHAAVVF
VFFFLAEYGSIVLMCILTSILFIGGYLLFEISYVFTVVNYIFFELFFIDVTFVEVQSLYTDFLNNSIIE
GLLYGFNLGLKSSLMIFTFIARASFPRIRFDQLMGFCTVLLPINFAIIILVPCVLYSFNLLPVNIPLF
LTHPPALLPQGYSTYEEGNRSRTILVNNYTYTKTIYHTRECNYTLFLYSTLLAYYYLYMIIILLNL
LGYINKKFVNFFHHFLIQNTNF
>YN158
ITILKNIKSYIKIIHLYSINLKGIFSILYLVLLLFSLLQVFLLLGSLLNRWYARLLIKVISFYLLQYLV
LEGFIIILKKCSFIHLTINTKKLKIILLMVKLLIETIKYMLNLIIQMLCLVLWQLFLLLHFYYSCLIK
NDVWVLYWYNLYLAINEYDIIYRVCICYSYFIFSHFYICSSSLSRKIYFRVWFPFFRAKNTIRYKI

LYFCFSLFTFRFRNIINFPFRSVCWYLWSFSYFNFYSYNNYRIYIIRKRSNRQQTKIIYTIERLPYR
VCWNRGFVKLRQKTKKPTKGLMGSYKKMITYIYSYYIFTITRIIIYIYIILLYIKIIIIIRYNYSILLY
KNIIYPIINFIFFLIIIFFYIMLTHLQMKRTKYCCYSSGYRKIFLYYLFLLGSFSVRGYMDGRRVFIL
MNSGFKLTLFMNLNYNYINVGYDRIMYSLRVKRDTPTYNTYWFRVEYIYLRVKNIYIQYIINIVI
YWYGLNQVYILSFDKHLFFYLLLLLYCEINNYNIILHSTYLGILSYLAINDSNQILFSLVWNNDL
LIHWFFDSKKRVNNFLDQKYYFLQKAYAFHFFDKQLLLIFGCLLVLTITKFFQFLYKFKEYKVIH
YNILTLSMRHNQVTLVTCEILVHYLFVYKLLPVLHLCIIVLVWKLLTQSIEMLITGDFVIYIVIQL
LLSFSCIYTEEVYITDHIELLVLFELLVLLYYWLSVSVMFYLMDRCHYEVLQLLLILLVLYLEGKI
LLNSFEVVFLLIMPLTDFLHYILYCLLYLLFCTLHFMILLVQAILLVFQVITIELHLLHIFYLKILLF
LYLFLYVLLYSLCLMFGIVIIILWLILCKLLLLLYLNDTYYLSMLFDLYLINYVLRCLVLFLLCYY
LLQIVDLEVYNLDHLVKLSEFLLLISFCNVLNTLKIHLYYVNVLYYTLVILLLYYLLVTIIVLIYLI
NLNYFIKLTKYIKIIIYIFYLRYYFSILGFQFIIYIILCITLHLALANLLFPLVWNNDLLIHWFFESKK
EIRNIFLLLLILFLKNIHFALPFSCIKMNDIKNKYLNYSYVILSYIATIIRSCINTCTCLISCSLCYSC
KKNYGYAKKIRSCCRLLWTIASICCLKTFIKRICSSYTIYCSFLFRSCNNFNFCIIRLRCYTLWSW
FRDKRHEFRYILHVSCVIFSYIRYSISWLKCEIRFSRFSKYSSINLINIKFSYINSNYDNRKFKFNCL
YISKSYLIYTTFISCVYNIFHRIYSDKSSFFSRGPANLVWSGYMSQIARKPFYFKNKRQLAGNLIP
NKYIIKLFIDTQFNIYIYLFIYMIKDIVNHRCESTKKKHMGTHLPLLGESELVSGFMTEHAAVVF
VFFFLAEYGSIVLMCILTSILFIGGYLLFEISYVFTVVNYIFFELFFIDVTFVEVQSLYTDFLNNSIIE
GLLYGFNLGLKSSLMIFTFIARASFPRIRFDQLMGFCTVLLPINFAIIILVPCVLYSFNLLPVNIPLF
LTHPPALLPQGYSTYEEGNRSRTILVNNYTYTKTIYHTRECNYTLFLYSTLLAYYYLYMIIILLNL
LGYINKKFVNFFHHFLIQNTNF
>YN161
ITILKNIKSYIKIIHLYSINLKGIFSILYLVLLLFSLLQVFLLLGSLLNRWYARLLIKVISFYLLQYLV
LEGFIIILKKCSFIHLTINTKKLKIILLMVKLLIETIKYMLNLIIQMLCLVLWQLFLLLHFYYSCLIK
NDVWVLYWYNLYLAINEYDIIYRVCICYSYFIFSHFYICSSSLSRKIYFRVWFPFFRAKNTIRYKI
LYFCFSLFTFRFRNIINFPFRSVCWYLWSFSYFNFYSYNNYRIYIIRKRSNRQQTKIIYTIERLPYR
VCWNRGFVKLRQKTKKPTKGLMGSYKKMITYIYSYYIFTITRIIIYIYIILLYIKIIIIIRYNYSILLY
KNIIYPIINFIFFLIIIFFYIMLTHLQMKRTKYCCYSSGYRKIFLYYLFLLGSFSVRGYMDGRRVFIL
MNSGFKLTLFMNLNYNYINVGYDRIMYSLRVKRDTPTYNTYWFRVEYIYLRVKNIYIQYIINIVI
YWYGLNQVYILSFDKHLFFYLLLLLYCEINNYNIILHSTYLGILSYLAINDSNQILFSLVWNNDL
LIHWFFDSKKRVNNFLDQKYYFLQKAYAFHFFDKQLLLIFGCLLVLTITKFFQFLYKFKEYKVIH
YNILTLSMRHNQVTLVTCEILVHYLFVYKLLPVLHLCIIVLVWKLLTQSIEMLITGDFVIYIVIQL
LLSFSCIYTEEVYITDHIELLVLFELLVLLYYWLSVSVMFYLMDRCHYEVLQLLLILLVLYLEGKI
LLNSFEVVFLLIMPLTDFLHYILYCLLYLLFCTLHFMILLVQAILLVFQVITIELHLLHIFYLKILLF
LYLFLYVLLYSLCLMFGIVIIILWLILCKLLLLLYLNDTYYLSMLFDLYLINYVLRCLVLFLLCYY
LLQIVDLEVYNLDHLVKLSEFLLLISFCNVLNTLKIHLYYVNVLYYTLVILLLYYLLVTIIVLIYLI
NLNYFIKLTKYIKIIIYIFYLRYYFSILGFQFIIYIILCITLHLALANLLFPLVWNNDLLIHWFFESKK
EIRNIFLLLLILFLKNIHFALPFSCIKMNDIKNKYLNYSYVILSYIATIIRSCINTCTCLISCSLCYSC
KKNYGYAKKIRSCCRLLWTIASICCLKTFIKRICSSYTIYCSFLFRSCNNFNFCIIRLRCYTLWSW
FRDKRHEFRYILHVSCVIFSYIRYSISWLKCEIRFSRFSKYSSINLINIKFSYINSNYDNRKFKFNCL
YISKSYLIYTTFISCVYNIFHRIYSDKSSFFSRGPANLVWSGYMSQIARKPFYFKNKRQLAGNLIP
NKYIIKLFIDTQFNIYIYLFIYMIKDIVNHRCESTKKKHMGTHLPLLGESELVSGFMTEHAAVVF
VFFFLAEYGSIVLMCILTSILFIGGYLLFEISYVFTVVNYIFFELFFIDVTFVEVQSLYTDFLNNSIIE
GLLYGFNLGLKSSLMIFTFIARASFPRIRFDQLMGFCTVLLPINFAIIILVPCVLYSFNLLPVNIPLF

LTHPPALLPQGYSTYEEGNRSRTILVNNYTYTKTIYHTRECNYTLFLYSTLLGYYYLYMIIILLNL
LGYINKKFVNFFHHFLIQNTNF
>YN166
ITILKNIKSYIKIIHLYSINLKGIFSILYLVLLLFSLLQVFLLLGSLLNRWYARLLIKVISFYLLQYLV
LEGFIIILKKCSFIHLTINTKKLKIILLMVKLLIETIKYMLNLIIQMLCLVLWQLFLLLHFYYSCLIK
NDVWVLYWYNLYLAINEYDIIYRVCICYSYFIFSHFYICSSSLSRKIYFRVWFPFFRAKNTIRYKI
LYFCFSLFTFRFRNIINFPFRSVCWYLWSFSYFNFYSYNNYRIYIIRKRSNRQQTKIIYTIERLPYR
VCWNRGFVKLRQKTKKPTKGLMGSYKKMITYIYSYYIFTITRIIIYIYIILLYIKIIIIIRYNYSILLY
KNIIYPIINFIFFLIIIFFYIMLTHLQMKRTKYCCYSSGYRKIFLYYLFLLGSFSVRGYMDGRRVFIL
MNSGFKLTLFMNLNYNYINVGYDRIMYSLRVKRDTPTYNTYWFRVEYIYLRVKNIYIQYIINIVI
YWYGLNQVYILSFDKHLFFYLLLLLYCEINNYNIILHSTYLGILSYLAINDSNQILFSLVWNNDL
LIHWFFDSKKRVNNFLDQKYYFLQKAYAFHFFDKQLLLIFGCLLVLTITKFFQFLYKFKEYKVIH
YNILTLSMRHNQVTLVTCEILVHYLFVYKLLPVLHLCIIVLVWKLLTQSIEMLITGDFVIYIVIQL
LLSFSCIYTEEVYITDHIELLVLFELLVLLYYWLSVSVMFYLMDRCHYEVLQLLLILLVLYLEGKI
LLNSFEVVFLLIMPLTDFLHYILYCLLYLLFCTLHFMILLVQAILLVFQVITIELHLLHIFYLKILLF
LYLFLYVLLYSLCLMFGIVIIILWLILCKLLLLLYLNDTYYLSMLFDLYLINYVLRCLVLFLLCYY
LLQIVDLEVYNLDHLVKLSEFLLLISFCNVLNTLKIHLYYVNVLYYTLVILLLYYLLVTIIVLIYLI
NLNYFIKLTKYIKIIIYIFYLRYYFSILGFQFIIYIILCITLHLALANLLFPLVWNNDLLIHWFFESKK
EIRNIFLLLLILFLKNIHFALPFSCIKMNDIKNKYLNYSYVILSYIATIIRSCINTCTCLISCSLCYSC
KKNYGYAKKIRSCCRLLWTIASICCLKTFIKRICSSYTIYCSFLFRSCNNFNFCIIRLRCYTLWSW
FRDKRHEFRYILHVSCVIFSYIRYSISWLKCEIRFSRFSKYSSINLINIKFSYINSNYDNRKFKFNCL
YISKSYLIYTTFISCVYNIFHRIYSDKSSFFSRGPANLVWSGYMSQIARKPFYFKNKRQLAGNLIP
NKYIIKLFIDTQFNIYIYLFIYMIKDIVNHRCESTKKKHMGTHLPLLGESELVSGFMTEHAAVVF
VFFFLAEYGSIVLMCILTSILFIGGYLLFEISYVFTVVNYIFFELFFIDVTFVEVQSLYTDFLNNSIIE
GLLYGFNLGLKSSLMIFTFIARASFPRIRFDQLMGFCTVLLPINFAIIILVPCVLYSFNLLPVNIPLF
LTHPPALLPQGYSTYEEGNRSRTILVNNYTYTKTIYHTRECNYTLFLYSTLLAYYYLYMIIILLNL
LGYINKKFVNFFHHFLIQNTNF
>YN179
ITILKNIKSYIKIIHLYSINLKGIFSILYLVLLLFSLLQVFLLLGSLLNRWYARLLIKVISFYLLQYLV
LEGFIIILKKCSFIHLTINTKKLKIILLMVKLLIETIKYMLNLIIQMLCLVLWQLFLLLHFYYSCLIK
NDVWVLYWYNLYLAINEYDIIYRVCICYSYFIFSHFYICSSSLSRKIYFRVWFPFFRAKNTIRYKI
LYFCFSLFTFRFRNIINFPFRSVCWYLWSFSYFNFYSYNNYRIYIIRKRSNRQQTKIIYTIERLPYR
VCWNRGFVKLRQKTKKPTKGLMGSYKKMITYIYSYYIFTITRIIIYIYIILLYIKIIIIIRYNYSILLY
KNIIYPIINFIFFLIIIFFYIMLTHLQMKRTKYCCYSSGYRKIFLYYLFLLGSFSVRGYMDGRRVFIL
MNSGFKLTLFMNLNYNYINVGYDRIMYSLRVKRDTPTYNTYWFRVEYIYLRVKNIYIQYIINIVI
YWYGLNQVYILSFDKHLFFYLLLLLYCEINNYNIILHSTYLGILSYLAINDSNQILFSLVWNNDL
LIHWFFDSKKRVNNFLDQKYYFLQKAYAFHFFDKQLLLIFGCLLVLTITKFFQFLYKFKEYKVIH
YNILTLSMRHNQVTLVTCEILVHYLFVYKLLPVLHLCIIVLVWKLLTQSIEMLITGDFVIYIVIQL
LLSFSCIYTEEVYITDHIELLVLFELLVLLYYWLSVSVMFYLMDRCHYEVLQLLLILLVLYLEGKI
LLNSFEVVFLLIMPLTDFLHYILYCLLYLLFCTLHFMILLVQAILLVFQVITIELHLLHIFYLKILLF
LYLFLYVLLYSLCLMFGIVIIILWLILCKLLLLLYLNDTYYLSMLFDLYLINYVLRCLVLFLLCYY
LLQIVDLEVYNLDHLVKLSEFLLLISFCNVLNTLKIHLYYVNVLYYTLVILLLYYLLVTIIVLIYLI
NLNYFIKLTKYIKIIIYIFYLRYYFSILGFQFIIYIILCITLHLALANLLFPLVWNNDLLIHWFFESKK
EIRNIFLLLLILFLKNIHFALPFSCIKMNDIKNKYLNYSYVILSYIATIIRSCINTCTCLISCSLCYSC

KKNYGYAKKIRSCCRLLWTIASICCLKTFIKRICSSYTIYCSFLFRSCNNFNFCIIRLRCYTLWSW
FRDKRHEFRYILHVSCVIFSYIRYSISWLKCEIRFSRFSKYSSINLINIKFSYINSNYDNRKFKFNCL
YISKSYLIYTTFISCVYNIFHRIYSDKSSFFSRGPANLVWSGYMSQIARKPFYFKNKRQLAGNLIP
NKYIIKLFIDTQFNIYIYLFIYMIKDIVNHRCESTKKKHMGTHLPLLGESELVSGFMTEHAAVVF
VFFFLAEYGSIVLMCILTSILFIGGYLLFEISYVFTVVNYIFFELFFIDVTFVEVQSLYTDFLNNSIIE
GLLYGFNLGLKSSLMIFTFIARASFPRIRFDQLMGFCTVLLPINFAIIILVPCVLYSFNLLPVNIPLF
LTHPPALLPQGYSTYEEGNRSRTILVNNYTYTKTIYHTRECNYTLFLYSTLLGYYYLYMIIILLNL
LGYINKKFVNFFHHFLIQNTNF

>YN199

ITILKNIKSYIKIIHLYSINLKGIFSILYLVLLLFSLLQVFLLLGSLLNRWYARLLIKVISFYLLQYLV
LEGFIIILKKCSFIHLTINTKKLKIILLMVKLLIETIKYMLNLIIQMLCLVLWQLFLLLHFYYSCLIK
NDVWVLYWYNLYLAINEYDIIYRVCICYSYFIFSHFYICSSSLSRKIYFRVWFPFFRAKNTIRYKI
LYFCFSLFTFRFRNIINFPFRSVCWYLWSFSYFNFYSYNNYRIYIIRKRSNRQQTKIIYTIERLPYR
VCWNRGFVKLRQKTKKPTKGLMGSYKKMITYIYSYYIFTITRIIIYIYIILLYIKIIIIIRYNYSILLY
KNIIYPIINFIFFLIIIFFYIMLTHLQMKRTKYCCYSSGYRKIFLYYLFLLGSFSVRGYMDGRRVFIL
MNSGFKLTLFMNLNYNYINVGYDRIMYSLRVKRDTPTYNTYWFRVEYIYLRVKNIYIQYIINIVI
YWYGLNQVYILSFDKHLFFYLLLLLYCEINNYNIILHSTYLGILSYLAINDSNQILFSLVWNNDL
LIHWFFDSKKRVNNFLDQKYYFLQKAYAFHFFDKQLLLIFGCLLVLTITKFFQFLYKFKEYKVIH
YNILTLSMRHNQVTLVTCEILVHYLFVYKLLPVLHLCIIVLVWKLLTQSIEMLITGDFVIYIVIQL
LLSFSCIYTEEVYITDHIELLVLFELLVLLYYWLSVSVMFYLMDRCHYEVLQLLLILLVLYLEGKI
LLNSFEVVFLLIMPLTDFLHYILYCLLYLLFCTLHFMILLVQAILLVFQVITIELHLLHIFYLKILLF
LYLFLYVLLYSLCLMFGIVIIILWLILCKLLLLLYLNDTYYLSMLFDLYLINYVLRCLVLFLLCYY
LLQIVDLEVYNLDHLVKLSEFLLLISFCNVLNTLKIHLYYVNVLYYTLVILLLYYLLVTIIVLIYLI
NLNYFIKLTKYIKIIIYIFYLRYYFSILGFQFIIYIILCITLHLALANLLFPLVWNNDLLIHWFFESKK
EIRNIFLLLLILFLKNIHFALPFSCIKMNDIKNKYLNYSYVILSYIATIIRSCINTCTCLISCSLCYSC
KKNYGYAKKIRSCCRLLWTIASICCLKTFIKRICSSYTIYCSFLFRSCNNFNFCIIRLRCYTLWSW
FRDKRHEFRYILHVSCVIFSYIRYSISWLKCEIRFSRFSKYSSINLINIKFSYINSNYDNRKFKFNCL
YISKSYLIYTTFISCVYNIFHRIYSDKSSFFSRGPANLVWSGYMSQIARKPFYFKNKRQLAGNLIP
NKYIIKLFIDTQFNIYIYLFIYMIKDIVNHRCESTKKKHMGTHLPLLGESELVSGFMTEHAAVVF
VFFFLAEYGSIVLMCILTSILFIGGYLLFEISYVFTVVNYIFFELFFIDVTFVEVQSLYTDFLNNSIIE
GLLYGFNLGLKSSLMIFTFIARASFPRIRFDQLMGFCTVLLPINFAIIILVPCVLYSFNLLPVNIPLF
LTHPPALLPQGYSTYEEGNRSRTILVNNYTYTKTIYHTRECNYTLFLYSTLLAYYYLYMIIILLNL
LGYINKKFVNFFHHFLIQNTNF

>YN208

ITILKNIKSYIKIIHLYSINLKGIFSILYLVLLLFSLLQVFLLLGSLLNRWYARLLIKVISFYLLQYLV
LEGFIIILKKCSFIHLTINTKKLKIILLMVKLLIETIKYMLNLIIQMLCLVLWQLFLLLHFYYSCLIK
NDVWVLYWYNLYLAINEYDIIYRVCICYSYFIFSHFYICSSSLSRKIYFRVWFPFFRAKNTIRYKI
LYFCFSLFTFRFRNIINFPFRSVCWYLWSFSYFNFYSYNNYRIYIIRKRSNRQQTKIIYTIERLPYR
VCWNRGFVKLRQKTKKPTKGLMGSYKKMITYIYSYYIFTITRIIIYIYIILLYIKIIIIIRYNYSILLY
KNIIYPIINFIFFLIIIFFYIMLTHLQMKRTKYCCYSSGYRKIFLYYLFLLGSFSVRGYMDGRRVFIL
MNSGFKLTLFMNLNYNYINVGYDRIMYSLRVKRDTPTYNTYWFRVEYIYLRVKNIYIQYIINIVI
YWYGLNQVYILSFDKHLFFYLLLLLYCEINNYNIILHSTYLGILSYLAINDSNQILFSLVWNNDL
LIHWFFDSKKRVNNFLDQKYYFLQKAYAFHFFDKQLLLIFGCLLVLTITKFFQFLYKFKEYKVIH
YNILTLSMRHNQVTLVTCEILVHYLFVYKLLPVLHLCIIVLVWKLLTQSIEMLITGDFVIYIVIQL

LLSFSCIYTEEVYITDHIELLVLFELLVLLYYWLSVSVMFYLMDRCHYEVLQLLLILLVLYLEGKI
LLNSFEVVFLLIMPLTDFLHYILYCLLYLLFCTLHFMILLVQAILLVFQVITIELHLLHIFYLKILLF
LYLFLYVLLYSLCLMFGIVIIILWLILCKLLLLLYLNDTYYLSMLFDLYLINYVLRCLVLFLLCYY
LLQIVDLEVYNLDHLVKLSEFLLLISFCNVLNTLKIHLYYVNVLYYTLVILLLYYLLVTIIVLIYLI
NLNYFIKLTKYIKIIIYIFYLRYYFSILGFQFIIYIILCITLHLALANLLFPLVWNNDLLIHWFFESKK
EIRNIFLLLLILFLKNIHFALPFSCIKMNDIKNKYLNYSYVILSYIATIIRSCINTCTCLISCSLCYSC
KKNYGYAKKIRSCCRLLWTIASICCLKTFIKRICSSYTIYCSFLFRSCNNFNFCIIRLRCYTLWSW
FRDKRHEFRYILHVSCVIFSYIRYSISWLKCEIRFSRFSKYSSINLINIKFSYINSNYDNRKFKFNCL
YISKSYLIYTTFISCVYNIFHRIYSDKSSFFSRGPANLVWSGYMSQIARKPFYFKNKRQLAGNLIP
NKYIIKLFIDTQFNIYIYLFIYMIKDIVNHRCESTKKKHMGTHLPLLGESELVSGFMTEHAAVVF
VFFFLAEYGSIVLMCILTSILFIGGYLLFEISYVFTVVNYIFFELFFIDVTFVEVQSLYTDFLNNSIIE
GLLYGFNLGLKSSLMIFTFIARASFPRIRFDQLMGFCTVLLPINFAIIILVPCVLYSFNLLPVNIPLF
LTHPPALLPQGYSTYEEGNRSRTILVNNYTYTKTIYHTRECNYTLFLYSTLLAYYYLYMIIILLNL
LGYINKKFVNFFHHFLIQNTNF
>YN220
ITILKNIKSYIKIIHLYSINLKGIFSILYLVLLLFSLLQVFLLLGSLLNRWYARLLIKVISFYLLQYLV
LEGFIIILKKCSFIHLTINTKKLKIILLMVKLLIETIKYMLNLIIQMLCLVLWQLFLLLHFYYSCLIK
NDVWVLYWYNLYLAINEYDIIYRVCICYSYFIFSHFYICSSSLSRKIYFRVWFPFFRAKNTIRYKI
LYFCFSLFTFRFRNIINFPFRSVCWYLWSFSYFNFYSYNNYRIYIIRKRSNRQQTKIIYTIERLPYR
VCWNRGFVKLRQKTKKPTKGLMGSYKKMITYIYSYYIFTITRIIIYIYIILLYIKIIIIIRYNYSILLY
KNIIYPIINFIFFLIIIFFYIMLTHLQMKRTKYCCYSSGYRKIFLYYLFLLGSFSVRGYMDGRRVFIL
MNSGFKLTLFMNLNYNYINVGYDRIMYSLRVKRDTPTYNTYWFRVEYIYLRVKNIYIQYIINIVI
YWYGLNQVYILSFDKHLFFYLLLLLYCEINNYNIILHSTYLGILSYLAINDSNQILFSLVWNNDL
LIHWFFDSKKRVNNFLDQKYYFLQKAYAFHFFDKQLLLIFGCLLVLTITKFFQFLYKFKEYKVIH
YNILTLSMRHNQVTLVTCEILVHYLFVYKLLPVLHLCIIVLVWKLLTQSIEMLITGDFVIYIVIQL
LLSFSCIYTEEVYITDHIELLVLFELLVLLYYWLSVSVMFYLMDRCHYEVLQLLLILLVLYLEGKI
LLNSFEVVFLLIMPLTDFLHYILYCLLYLLFCTLHFMILLVQAILLVFQVITIELHLLHIFYLKILLF
LYLFLYVLLYSLCLMFGIVIIILWLILCKLLLLLYLNDTYYLSMLFDLYLINYVLRCLVLFLLCYY
LLQIVDLEVYNLDHLVKLSEFLLLISFCNVLNTLKIHLYYVNVLYYTLVILLLYYLLVTIIVLIYLI
NLNYFIKLTKYIKIIIYIFYLRYYFSILGFQFIIYIILCITLHLALANLLFPLVWNNDLLIHWFFESKK
EIRNIFLLLLILFLKNIHFALPFSCIKMNDIKNKYLNYSYVILSYIATIIRSCINTCTCLISCSLCYSC
KKNYGYAKKIRSCCRLLWTIASICCLKTFIKRICSSYTIYCSFLFRSCNNFNFCIIRLRCYTLWSW
FRDKRHEFRYILHVSCVIFSYIRYSISWLKCEIRFSRFSKYSSINLINIKFSYINSNYDNRKFKFNCL
YISKSYLIYTTFISCVYNIFHRIYSDKSSFFSRGPANLVWSGYMSQIARKPFYFKNKRQLAGNLIP
NKYIIKLFIDTQFNIYIYLFIYMIKDIVNHRCESTKKKHMGTHLPLLGESELVSGFMTEHAAVVF
VFFFLAEYGSIVLMCILTSILFIGGYLLFEISYVFTVVNYIFFELFFIDVTFVEVQSLYTDFLNNSIIE
GLLYGFNLGLKSSLMIFTFIARASFPRIRFDQLMGFCTVLLPINFAIIILVPCVLYSFNLLPVNIPLF
LTHPPALLPQGYSTYEEGNRSRTILVNNYTYTKTIYHTRECNYTLFLYSTLLAYYYLYMIIILLNL
LGYINKKFVNFFHHFLIQNTNF
>YN226
ITILKNIKSYIKIIHLYSINLKGIFSILYLVLLLFSLLQVFLLLGSLLNRWYARLLIKVISFYLLQYLV
LEGFIIILKKCSFIHLTINTKKLKIILLMVKLLIETIKYMLNLIIQMLCLVLWQLFLLLHFYYSCLIK
NDVWVLYWYNLYLAINEYDIIYRVCICYSYFIFSHFYICSSSLSRKIYFRVWFPFFRAKNTIRYKI
LYFCFSLFTFRFRNIINFPFRSVCWYLWSFSYFNFYSYNNYRIYIIRKRSNRQQTKIIYTIERLPYR

VCWNRGFVKLRQKTKKPTKGLMGSYKKMITYIYSYYIFTITRIIIYIYIILLYIKIIIIIRYNYSILLY
KNIIYPIINFIFFLIIIFFYIMLTHLQMKRTKYCCYSSGYRKIFLYYLFLLGSFSVRGYMDGRRVFIL
MNSGFKLTLFMNLNYNYINVGYDRIMYSLRVKRDTPTYNTYWFRVEYIYLRVKNIYIQYIINIVI
YWYGLNQVYILSFDKHLFFYLLLLLYCEINNYNIILHSTYLGILSYLAINDSNQILFSLVWNNDL
LIHWFFDSKKRVNNFLDQKYYFLQKAYAFHFFDKQLLLIFGCLLVLTITKFFQFLYKFKEYKVIH
YNILTLSMRHNQVTLVTCEILVHYLFVYKLLPVLHLCIIVLVWKLLTQSIEMLITGDFVIYIVIQL
LLSFSCIYTEEVYITDHIELLVLFELLVLLYYWLSVSVMFYLMDRCHYEVLQLLLILLVLYLEGKI
LLNSFEVVFLLIMPLTDFLHYILYCLLYLLFCTLHFMILLVQAILLVFQVITIELHLLHIFYLKILLF
LYLFLYVLLYSLCLMFGIVIIILWLILCKLLLLLYLNDTYYLSMLFDLYLINYVLRCLVLFLLCYY
LLQIVDLEVYNLDHLVKLSEFLLLISFCNVLNTLKIHLYYVNVLYYTLVILLLYYLLVTIIVLIYLI
NLNYFIKLTKYIKIIIYIFYLRYYFSILGFQFIIYIILCITLHLALANLLFPLVWNNDLLIHWFFESKK
EIRNIFLLLLILFLKNIHFALPFSCIKMNDIKNKYLNYSYVILSYIATIIRSCINTCTCLISCSLCYSC
KKNYGYAKKIRSCCRLLWTIASICCLKTFIKRICSSYTIYCSFLFRSCNNFNFCIIRLRCYTLWSW
FRDKRHEFRYILHVSCVIFSYIRYSISWLKCEIRFSRFSKYSSINLINIKFSYINSNYDNRKFKFNCL
YISKSYLIYTTFISCVYNIFHRIYSDKSSFFSRGPANLVWSGYMSQIARKPFYFKNKRQLAGNLIP
NKYIIKLFIDTQFNIYIYLFIYMIKDIVNHRCESTKKKHMGTHLPLLGESELVSGFMTEHAAVVF
VFFFLAEYGSIVLMCILTSILFIGGYLLFEISYVFTVVNYIFFELFFIDVTFVEVQSLYTDFLNNSIIE
GLLYGFNLGLKSSLMIFTFIARASFPRIRFDQLMGFCTVLLPINFAIIILVPCVLYSFNLLPVNIPLF
LTHPPALLPQGYSTYEEGNRSRTILVNNYTYTKTIYHTRECNYTLFLYSTLLAYYYLYMIIILLNL
LGYINKKFVNFFHHFLIQNTN

>YN228

ITILKNIKSYIKIIHLYSINLKGIFSILYLVLLLFSLLQVFLLLGSLLNRWYARLLIKVISFYLLQYLV
LEGFIIILKKCSFIHLTINTKKLKIILLMVKLLIETIKYMLNLIIQMLCLVLWQLFLLLHFYYSCLIK
NDVWVLYWYNLYLAINEYDIIYRVCICYSYFIFSHFYICSSSLSRKIYFRVWFPFFRAKNTIRYKI
LYFCFSLFTFRFRNIINFPFRSVCWYLWSFSYFNFYSYNNYRIYIIRKRSNRQQTKIIYTIERLPYR
VCWNRGFVKLRQKTKKPTKGLMGSYKKMITYIYSYYIFTITRIIIYIYIILLYIKIIIIIRYNYSILLY
KNIIYPIINFIFFLIIIFFYIMLTHLQMKRTKYCCYSSGYRKIFLYYLFLLGSFSVRGYMDGRRVFIL
MNSGFKLTLFMNLNYNYINVGYDRIMYSLRVKRDTPTYNTYWFRVEYIYLRVKNIYIQYIINIVI
YWYGLNQVYILSFDKHLFFYLLLLLYCEINNYNIILHSTYLGILSYLAINDSNQILFSLVWNNDL
LIHWFFDSKKRVNNFLDQKYYFLQKAYAFHFFDKQLLLIFGCLLVLTITKFFQFLYKFKEYKVIH
YNILTLSMRHNQVTLVTCEILVHYLFVYKLLPVLHLCIIVLVWKLLTQSIEMLITGDFVIYIVIQL
LLSFSCIYTEEVYITDHIELLVLFELLVLLYYWLSVSVMFYLMDRCHYEVLQLLLILLVLYLEGKI
LLNSFEVVFLLIMPLTDFLHYILYCLLYLLFCTLHFMILLVQAILLVFQVITIELHLLHIFYLKILLF
LYLFLYVLLYSLCLMFGIVIIILWLILCKLLLLLYLNDTYYLSMLFDLYLINYVLRCLVLFLLCYY
LLQIVDLEVYNLDHLVKLSEFLLLISFCNVLNTLKIHLYYVNVLYYTLVILLLYYLLVTIIVLIYLI
NLNYFIKLTKYIKIIIYIFYLRYYFSILGFQFIIYIILCITLHLALANLLFPLVWNNDLLIHWFFESKK
EIRNIFLLLLILFLKNIHFALPFSCIKMNDIKNKYLNYSYVILSYIATIIRSCINTCTCLISCSLCYSC
KKNYGYAKKIRSCCRLLWTIASICCLKTFIKRICSSYTIYCSFLFRSCNNFNFCIIRLRCYTLWSW
FRDKRHEFRYILHVSCVIFSYIRYSISWLKCEIRFSRFSKYSSINLINIKFSYINSNYDNRKFKFNCL
YISKSYLIYTTFISCVYNIFHRIYSDKSSFFSRGPANLVWSGYMSQIARKPFYFKNKRQLAGNLIP
NKYIIKLFIDTQFNIYIYLFIYMIKDIVNHRCESTKKKHMGTHLPLLGESELVSGFMTEHAAVVF
VFFFLAEYGSIVLMCILTSILFIGGYLLFEISYVFTVVNYIFFELFFIDVTFVEVQSLYTDFLNNSIIE
GLLYGFNLGLKSSLMIFTFIARASFPRIRFDQLMGFCTVLLPINFAIIILVPCVLYSFNLLPVNIPLF
LTHPPALLPQGYSTYEEGNRSRTILVNNYTYTKTIYHTRECNYTLFLYSTLLAYYYLYMIIILLNL

LGYINKKFVNFFHHFLIQNTNY
>YN232
ITILKNIKSYIKIIHLYSINLKGIFSILYLVLLLFSLLQVFLLLGSLLNRWYARLLIKVISFYLLQYLV
LEGFIIILKKCSFIHLTINTKKLKIILLMVKLLIETIKYMLNLIIQMLCLVLWQLFLLLHFYYSCLIK
NDVWVLYWYNLYLAINEYDIIYRVCICYSYFIFSHFYICSSSLSRKIYFRVWFPFFRAKNTIRYKI
LYFCFSLFTFRFRNIINFPFRSVCWYLWSFSYFNFYSYNNYRIYIIRKRSNRQQTKIIYTIERLPYR
VCWNRGFVKLRQKTKKPTKGLMGSYKKMITYIYSYYIFTITRIIIYIYIILLYIKIIIIIRYNYSILLY
KNIIYPIINFIFFLIIIFFYIMLTHLQMKRTKYCCYSSGYRKIFLYYLFLLGSFSVRGYMDGRRVFIL
MNSGFKLTLFMNLNYNYINVGYDRIMYSLRVKRDTPTYNTYWFRVEYIYLRVKNIYIQYIINIVI
YWYGLNQVYILSFDKHLFFYLLLLLYCEINNYNIILHSTYLGILSYLAINDSNQILFSLVWNNDL
LIHWFFDSKKRVNNFLDQKYYFLQKAYAFHFFDKQLLLIFGCLLVLTITKFFQFLYKFKEYKVIH
YNILTLSMRHNQVTLVTCEILVHYLFVYKLLPVLHLCIIVLVWKLLTQSIEMLITGDFVIYIVIQL
LLSFSCIYTEEVYITDHIELLVLFELLVLLYYWLSVSVMFYLMDRCHYEVLQLLLILLVLYLEGKI
LLNSFEVVFLLIMPLTDFLHYILYCLLYLLFCTLHFMILLVQAILLVFQVITIELHLLHIFYLKILLF
LYLFLYVLLYSLCLMFGIVIIILWLILCKLLLLLYLNDTYYLSMLFDLYLINYVLRCLVLFLLCYY
LLQIVDLEVYNLDHLVKLSEFLLLISFCNVLNTLKIHLYYVNVLYYTLVILLLYYLLVTIIVLIYLI
NLNYFIKLTKYIKIIIYIFYLRYYFSILGFQFIIYIILCITLHLALANLLFPLVWNNDLLIHWFFESKK
EIRNIFLLLLILFLKNIHFALPFSCIKMNDIKNKYLNYSYVILSYIATIIRSCINTCTCLISCSLCYSC
KKNYGYAKKIRSCCRLLWTIASICCLKTFIKRICSSYTIYCSFLFRSCNNFNFCIIRLRCYTLWSW
FRDKRHEFRYILHVSCVIFSYIRYSISWLKCEIRFSRFSKYSSINLINIKFSYINSNYDNRKFKFNCL
YISKSYLIYTTFISCVYNIFHRIYSDKSSFFSRGPANLVWSGYMSQIARKPFYFKNKRQLAGNLIP
NKYIIKLFIDTQFNIYIYLFIYMIKDIVNHRCESTKKKHMGTHLPLLGESELVSGFMTEHAAVVF
VFFFLAEYGSIVLMCILTSILFIGGYLLFEISYVFTVVNYIFFELFFIDVTFVEVQSLYTDFLNNSIIE
GLLYGFNLGLKSSLMIFTFIARASFPRIRFDQLMGFCTVLLPINFAIIILVPCVLYSFNLLPVNIPLF
LTHPPALLPQGYSTYEEGNRSRTILVNNYTYTKTIYHTRECNYTLFLYSTLLAYYYLYMIIILLNL
LGYINKKFVNFFHHFLIQNTNF
>YN239
ITILKNIKSYIKIIHLYSINLKGIFSILYLVLLLFSLLQVFLLLGSLLNRWYARLLIKVISFYLLQYLV
LEGFIIILKKCSFIHLTINTKKLKIILLMVKLLIETIKYMLNLIIQMLCLVLWQLFLLLHFYYSCLIK
NDVWVLYWYNLYLAINEYDIIYRVCICYSYFIFSHFYICSSSLSRKIYFRVWFPFFRAKNTIRYKI
LYFCFSLFTFRFRNIINFPFRSVCWYLWSFSYFNFYSYNNYRIYIIRKRSNRQQTKIIYTIERLPYR
VCWNRGFVKLRQKTKKPTKGLMGSYKKMITYIYSYYIFTITRIIIYIYIILLYIKIIIIIRYNYSILLY
KNIIYPIINFIFFLIIIFFYIMLTHLQMKRTKYCCYSSGYRKIFLYYLFLLGSFSVRGYMDGRRVFIL
MNSGFKLTLFMNLNYNYINVGYDRIMYSLRVKRDTPTYNTYWFRVEYIYLRVKNIYIQYIINIVI
YWYGLNQVYILSFDKHLFFYLLLLLYCEINNYNIILHSTYLGILSYLAINDSNQILFSLVWNNDL
LIHWFFDSKKRVNNFLDQKYYFLQKAYAFHFFDKQLLLIFGCLLVLTITKFFQFLYKFKEYKVIH
YNILTLSMRHNQVTLVTCEILVHYLFVYKLLPVLHLCIIVLVWKLLTQSIEMLITGDFVIYIVIQL
LLSFSCIYTEEVYITDHIELLVLFELLVLLYYWLSVSVMFYLMDRCHYEVLQLLLILLVLYLEGKI
LLNSFEVVFLLIMPLTDFLHYILYCLLYLLFCTLHFMILLVQAILLVFQVITIELHLLHIFYLKILLF
LYLFLYVLLYSLCLMFGIVIIILWLILCKLLLLLYLNDTYYLSMLFDLYLINYVLRCLVLFLLCYY
LLQIVDLEVYNLDHLVKLSEFLLLISFCNVLNTLKIHLYYVNVLYYTLVILLLYYLLVTIIVLIYLI
NLNYFIKLTKYIKIIIYIFYLRYYFSILGFQFIIYIILCITLHLALANLLFPLVWNNDLLIHWFFESKK
EIRNIFLLLLILFLKNIHFALPFSCIKMNDIKNKYLNYSYVILSYIATIIRSCINTCTCLISCSLCYSC
KKNYGYAKKIRSCCRLLWTIASICCLKTFIKRICSSYTIYCSFLFRSCNNFNFCIIRLRCYTLWSW

FRDKRHEFRYILHVSCVIFSYIRYSISWLKCEIRFSRFSKYSSINLINIKFSYINSNYDNRKFKFNCL
YISKSYLIYTTFISCVYNIFHRIYSDKSSFFSRGPANLVWSGYMSQIARKPFYFKNKRQLAGNLIP
NKYIIKLFIDTQFNIYIYLFIYMIKDIVNHRCESTKKKHMGTHLPLLGESELVSGFMTEHAAVVF
VFFFLAEYGSIVLMCILTSILFIGGYLLFEISYVFTVVNYIFFELFFIDVTFVEVQSLYTDFLNNSIIE
GLLYGFNLGLKSSLMIFTFIARASFPRIRFDQLMGFCTVLLPINFAIIILVPCVLYSFNLLPVNIPLF
LTHPPALLPQGYSTYEEGNRSRTILVNNYTYTKTIYHTRECNYTLFLYSTLLAYYYLYMIIILLNL
LGYINKKFVNFFHHFLIQNTNF
>YN247
ITILKNIKSYIKIIHLYSINLKGIFSILYLVLLLFSLLQVFLLLGSLLNRWYARLLIKVISFYLLQYLV
LEGFIIILKKCSFIHLTINTKKLKIILLMVKLLIETIKYMLNLIIQMLCLVLWQLFLLLHFYYSCLIK
NDVWVLYWYNLYLAINEYDIIYRVCICYSYFIFSHFYICSSSLSRKIYFRVWFPFFRAKNTIRYKI
LYFCFSLFTFRFRNIINFPFRSVCWYLWSFSYFNFYSYNNYRIYIIRKRSNRQQTKIIYTIERLPYR
VCWNRGFVKLRQKTKKPTKGLMGSYKKMITYIYSYYIFTITRIIIYIYIILLYIKIIIIIRYNYSILLY
KNIIYPIINFIFFLIIIFFYIMLTHLQMKRTKYCCYSSGYRKIFLYYLFLLGSFSVRGYMDGRRVFIL
MNSGFKLTLFMNLNYNYINVGYDRIMYSLRVKRDTPTYNTYWFRVEYIYLRVKNIYIQYIINIVI
YWYGLNQVVYILSFDKHLFFYLLLLLYCEINNYNIILHSTYLGILSYLAINDSNQILFSLVWNNDL
LIHWFFDSKKRVNNFLDQKYYFLQKAYAFHFFDKQLLLIFGCLLVLTITKFFQFLYKFKEYKVIH
YNILTLSMRHNQVTLVTCEILVHYLFVYKLLPVLHLCIIVLVWKLLTQSIEMLITGDFVIYIVIQL
LLSFSCIYTEEVYITDHIELLVLFELLVLLYYWLSVSVMFYLMDRCHYEVLQLLLILLVLYLEGKI
LLNSFEVVFLLIMPLTDFLHYILYCLLYLLFCTLHFMILLVQAILLVFQVITIELHLLHIFYLKILLF
LYLFLYVLLYSLCLMFGIVIIILWLILCKLLLLLYLNDTYYLSMLFDLYLINYVLRCLVLFLLCYY
LLQIVDLEVYNLDHLVKLSEFLLLISFCNVLNTLKIHLYYVNVLYYTLVILLLYYLLVTIIVLIYLI
NLNYFIKLTKYIKIIIYIFYLRYYFSILGFQFIIYIILCITLHLALANLLFPLVWNNDLLIHWFFESKK
EIRNIFLLLLILFLKNIHFALPFSCIKMNDIKNKYLNYSYVILSYIATIIRSCINTCTCLISCSLCYSC
KKNYGYAKKIRSCCRLLWTIASICCLKTFIKRICSSYTIYCSFLFRSCNNFNFCIIRLRCYTLWSW
FRDKRHEFRYILHVSCVIFSYIRYSISWLKCEIRFSRFSKYSSINLINIKFSYINSNYDNRKFKFNCL
YISKSYLIYTTFISCVYNIFHRIYSDKSSFFSRGPANLVWSGYMSQIARKPFYFKNKRQLAGNLIP
NKYIIKLFIDTQFNIYIYLFIYMIKDIVNHRCESTKKKHMGTHLPLLGESELVSGFMTEHAAVVF
VFFFLAEYGSIVLMCILTSILFIGGYLLFEISYVFTVVNYIFFELFFIDVTFVEVQSLYTDFLNNSIIE
GLLYGFNLGLKSSLMIFTFIARASFPRIRFDQLMGFCTVLLPINFAIIILVPCVLYSFNLLPVNIPLF
LTHPPALLPQGYSTYEEGNRSRTILVNNYTYTKTIYHTRECNYTLFLYSTLLAYYYLYMIIILLNL
LGYINKKFVNFFHHFLIQNTNF
>YN268
ITILKNIKSYIKIIHLYSINLKGIFSILYLVLLLFSLLQVFLLLGSLLNRWYARLLIKVISFYLLQYLV
LEGFIIILKKCSFIHLTINTKKLKIILLMVKLLIETIKYMLNLIIQMLCLVLWQLFLLLHFYYSCLIK
NDVWVLYWYNLYLAINEYDIIYRVCICYSYFIFSHFYICSSSLSRKIYFRVWFPFFRAKNTIRYKI
LYFCFSLFTFRFRNIINFPFRSVCWYLWSFSYFNFYSYNNYRIYIIRKRSNRQQTKIIYTIERLPYR
VCWNRGFVKLRQKTKKPTKGLMGSYKKMITYIYSYYIFTITRIIIYIYIILLYIKIIIIIRYNYSILLY
KNIIYPIINFIFFLIIIFFYIMLTHLQMKRTKYCCYSSGYRKIFLYYLFLLGSFSVRGYMDGRRVFIL
MNSGFKLTLFMNLNYNYINVGYDRIMYSLRVKRDTPTYNTYWFRVEYIYLRVKNIYIQYIINIVI
YWYGLNQVVYILSFDKHLFFYLLLLLYCEINNYNIILHSTYLGILSYLAINDSNQILFSLVWNNDL
LIHWFFDSKKRVNNFLDQKYYFLQKAYAFHFFDKQLLLIFGCLLVLTITKFFQFLYKFKEYKVIH
YNILTLSMRHNQVTLVTCEILVHYLFVYKLLPVLHLCIIVLVWKLLTQSIEMLITGDFVIYIVIQL
LLSFSCIYTEEVYITDHIELLVLFELLVLLYYWLSVSVMFYLMDRCHYEVLQLLLILLVLYLEGKI

LLNSFEVVFLLIMPLTDFLHYILYCLLYLLFCTLHFMILLVQAILLVFQVITIELHLLHIFYLKILLF
LYLFLYVLLYSLCLMFGIVIIILWLILCKLLLLLYLNDTYYLSMLFDLYLINYVLRCLVLFLLCYY
LLQIVDLEVYNLDHLVKLSEFLLLISFCNVLNTLKIHLYYVNVLYYTLVILLLYYLLVTIIVLIYLI
NLNYFIKLTKYIKIIIYIFYLRYYFSILGFQFIIYIILCITLHLALANLLFPLVWNNDLLIHWFFESKK
EIRNIFLLLLILFLKNIHFALPFSCIKMNDIKNKYLNYSYVILSYIATIIRSCINTCTCLISCSLCYSC
KKNYGYAKKIRSCCRLLWTIASICCLKTFIKRICSSYTIYCSFLFRSCNNFNFCIIRLRCYTLWSW
FRDKRHEFRYILHVSCVIFSYIRYSISWLKCEIRFSRFSKYSSINLINIKFSYINSNYDNRKFKFNCL
YISKSYLIYTTFISCVYNIFHRIYSDKSSFFSRGPANLVWSGYMSQIARKPFYFKNKRQLAGNLIP
NKYIIKLFIDTQFNIYIYLFIYMIKDIVNHRCESTKKKHMGTHLPLLGESELVSGFMTEHAAVVF
VFFFLAEYGSIVLMCILTSILFIGGYLLFEISYVFTVVNYIFFELFFIDVTFVEVQSLYTDFLNNSIIE
GLLYGFNLGLKSSLMIFTFIARASFPRIRFDQLMGFCTVLLPINFAIIILVPCVLYSFNLLPVNIPLF
LTHPPALLPQGYSTYEEGNRSRTILVNNYTYTKTIYHTRECNYTLFLYSTLLAYYYLYMIIILLNL
LGYINKKFVNFFHHFLIQNTNF
>YN271
ITILKNIKSYIKIIHLYSINLKGIFSILYLVLLLFSLLQVFLLLGSLLNRWYARLLIKVISFYLLQYLV
LEGFIIILKKCSFIHLTINTKKLKIILLMVKLLIETIKYMLNLIIQMLCLVLWQLFLLLHFYYSCLIK
NDVWVLYWYNLYLAINEYDIIYRVCICYSYFIFSHFYICSSSLSRKIYFRVWFPFFRAKNTIRYKI
LYFCFSLFTFRFRNIINFPPFRSVCWYLWSFSYFNFYSYNNYRIYIIRKRSNRQQTKIIYTIERLPYR
VCWNRGFVKLRQKTKKPTKGLMGSYKKMITYIYSYYIFTITRIIIYIYIILLYIKIIIIIRYNYSILLY
KNIIYPIINFIFFLIIIFFYIMLTHLQMKRTKYCCYSSGYRKIFLYYLFLLGSFSVRGYMDGRRVFIL
MNSGFKLTLFMNLNYNYINVGYDRIMYSLRVKRDTPTYNTYWFRVEYIYLRVKNIYIQYIINIVI
YWYGLNQVVYILSFDKHLFFYLLLLLYCEINNYNIILHSTYLGILSYLAINDSNQILFSLVWNNDL
LIHWFFDSKKRVNNFLDQKYYFLQKAYAFHFFDKQLLLIFGCLLVLTITKFFQFLYKFKEYKVIH
YNILTLSMRHNQVTLVTCEILVHYLFVYKLLPVLHLCIIVLVWKLLTQSIEMLITGDFVIYIVIQL
LLSFSCIYTEEVYITDHIELLVLFELLVLLYYWLSVSVMFYLMDRCHYEVLQLLLILLVLYLEGKI
LLNSFEVVFLLIMPLTDFLHYILYCLLYLLFCTLHFMILLVQAILLVFQVITIELHLLHIFYLKILLF
LYLFLYVLLYSLCLMFGIVIIILWLILCKLLLLLYLNDTYYLSMLFDLYLINYVLRCLVLFLLCYY
LLQIVDLEVYNLDHLVKLSEFLLLISFCNVLNTLKIHLYYVNVLYYTLVILLLYYLLVTIIVLIYLI
NLNYFIKLTKYIKIIIYIFYLRYYFSILGFQFIIYIILCITLHLALANLLFPLVWNNDLLIHWFFESKK
EIRNIFLLLLILFLKNIHFALPFSCIKMNDIKNKYLNYSYVILSYIATIIRSCINTCTCLISCSLCYSC
KKNYGYAKKIRSCCRLLWTIASICCLKTFIKRICSSYTIYCSFLFRSCNNFNFCIIRLRCYTLWSW
FRDKRHEFRYILHVSCVIFSYIRYSISWLKCEIRFSRFSKYSSINLINIKFSYINSNYDNRKFKFNCL
YISKSYLIYTTFISCVYNIFHRIYSDKSSFFSRGPANLVWSGYMSQIARKPFYFKNKRQLAGNLIP
NKYIIKLFIDTQFNIYIYLFIYMIKDIVNHRCESTKKKHMGTHLPLLGESELVSGFMTEHAAVVF
VFFFLAEYGSIVLMCILTSILFIGGYLLFEISYVFTVVNYIFFELFFIDVTFVEVQSLYTDFLNNSIIE
GLLYGFNLGLKSSLMIFTFIARASFPRIRFDQLMGFCTVLLPINFAIIILVPCVLYSFNLLPVNIPLF
LTHPPALLPQGYSTYEEGNRSRTILVNNYTYTKTIYHTRECNYTLFLYSTLLAYYYLYMIIILLNL
LGYINKKFVNFFHHFLIQNTNF
>YN283
ITILKNIKSYIKIIHLYSINLKGIFSILYLVLLLFSLLQVFLLLGSLLNRWYARLLIKVISFYLLQYLV
LEGFIIILKKCSFIHLTINTKKLKIILLMVKLLIETIKYMLNLIIQMLCLVLWQLFLLLHFYYSCLIK
NDVWVLYWYNLYLAINEYDIIYRVCICYSYFIFSHFYICSSSLSRKIYFRVWFPFFRAKNTIRYKI
LYFCFSLFTFRFRNIINFPPFRSVCWYLWSFSYFNFYSYNNYRIYIIRKRSNRQQTKIIYTIERLPYR
VCWNRGFVKLRQKTKKPTKGLMGSYKKMITYIYSYYIFTITRIIIYIYIILLYIKIIIIIRYNYSILLY

KNIIYPIINFIFFLIIIFFYIMLTHLQMKRTKYCCYSSGYRKIFLYYLFLLGSFSVRGYMDGRRVFIL
MNSGFKLTLFMNLNYNYINVGYDRIMYSLRVKRDTPTYNTYWFRVEYIYLRVKNIYIQYIINIVI
YWYGLNQVYILSFDKHLFFYLLLLLYCEINNYNIILHSTYLGILSYLAINDSNQILFSLVWNNDL
LIHWFFDSKKRVNNFLDQKYYFLQKAYAFHFFDKQLLLIFGCLLVLTITKFFQFLYKFKEYKVIH
YNILTLSMRHNQVTLVTCEILVHYLFVYKLLPVLHLCIIVLVWKLLTQSIEMLITGDFVIYIVIQL
LLSFSCIYTEEVYITDHIELLVLFELLVLLYYWLSVSVMFYLMDRCHYEVLQLLLILLVLYLEGKI
LLNSFEVVFLLIMPLTDFLHYILYCLLYLLFCTLHFMILLVQAILLVFQVITIELHLLHIFYLKILLF
LYLFLYVLLYSLCLMFGIVIIILWLILCKLLLLLYLNDTYYLSMLFDLYLINYVLRCLVLFLLCYY
LLQIVDLEVYNLDHLVKLSEFLLLISFCNVLNTLKIHLYYVNVLYYTLVILLLYYLLVTIIVLIYLI
NLNYFIKLTKYIKIIIYIFYLRYYFSILGFQFIIYIILCITLHLALANLLFPLVWNNDLLIHWFFESKK
EIRNIFLLLLILFLKNIHFALPFSCIKMNDIKNKYLNYSYVILSYIATIIRSCINTCTCLISCSLCYSC
KKNYGYAKKIRSCCRLLWTIASICCLKTFIKRICSSYTIYCSFLFRSCNNFNFCIIRLRCYTLWSW
FRDKRHEFRYILHVSCVIFSYIRYSISWLKCEIRFSRFSKYSSINLINIKFSYINSNYDNRKFKFNCL
YISKSYLIYTTFISCVYNIFHRIYSDKSSFFSRGPANLVWSGYMSQIARKPFYFKNKRQLAGNLIP
NKYIIKLFIDTQFNIYIYLFIYMIKDIVNHRCESTKKKHMGTHLPLLGESELVSGFMTEHAAVVF
VFFFLAEYGSIVLMCILTSILFIGGYLLFEISYVFTVVNYIFFELFFIDVTFVEVQSLYTDFLNNSIIE
GLLYGFNLGLKSSLMIFTFIARASFPRIRFDQLMGFCTVLLPINFAIIILVPCVLYSFNLLPVNIPLF
LTHPPALLPQGYSTYEEGNRSRTILVNNYTYTKTIYHTRECNYTLFLYSTLLAYYYLYMIIILLNL
LGYINKKFVNFFHHFLIQNTNF
>YN311
ITILKNIKSYIKIIHLYSINLKGIFSILYLVLLLFSLLQVFLLLGSLLNRWYARLLIKVISFYLLQYLV
LEGFIIILKKCSFIHLTINTKKLKIILLMVKLLIETIKYMLNLIIQMLCLVLWQLFLLLHFYYSCLIK
NDVWVLYWYNLYLAINEYDIIYRVCICYSYFIFSHFYICSSSLSRKIYFRVWFPFFRAKNTIRYKI
LYFCFSLFTFRFRNIINFPFRSVCWYLWSFSYFNFYSYNNYRIYIIRKRSNRQQTKIIYTIERLPYR
VCWNRGFVKLRQKTKKPTKGLMGSYKKMITYIYSYYIFTITRIIIYIYIILLYIKIIIIIRYNYSILLY
KNIIYPIINFIFFLIIIFFYIMLTHLQMKRTKYCCYSSGYRKIFLYYLFLLGSFSVRGYMDGRRVFIL
MNSGFKLTLFMNLNYNYINVGYDRIMYSLRVKRDTPTYNTYWFRVEYIYLRVKNIYIQYIINIVI
YWYGLNQVYILSFDKHLFFYLLLLLYCEINNYNIILHSTYLGILSYLAINDSNQILFSLVWNNDL
LIHWFFDSKKRVNNFLDQKYYFLQKAYAFHFFDKQLLLIFGCLLVLTITKFFQFLYKFKEYKVIH
YNILTLSMRHNQVTLVTCEILVHYLFVYKLLPVLHLCIIVLVWKLLTQSIEMLITGDFVIYIVIQL
LLSFSCIYTEEVYITDHIELLVLFELLVLLYYWLSVSVMFYLMDRCHYEVLQLLLILLVLYLEGKI
LLNSFEVVFLLIMPLTDFLHYILYCLLYLLFCTLHFMILLVQAILLVFQVITIELHLLHIFYLKILLF
LYLFLYVLLYSLCLMFGIVIIILWLILCKLLLLLYLNDTYYLSMLFDLYLINYVLRCLVLFLLCYY
LLQIVDLEVYNLDHLVKLSEFLLLISFCNVLNTLKIHLYYVNVLYYTLVILLLYYLLVTIIVLIYLI
NLNYFIKLTKYIKIIIYIFYLRYYFSILGFQFIIYIILCITLHLALANLLFPLVWNNDLLIHWFFESKK
EIRNIFLLLLILFLKNIHFALPFSCIKMNDIKNKYLNYSYVILSYIATIIRSCINTCTCLISCSLCYSC
KKNYGYAKKIRSCCRLLWTIASICCLKTFIKRICSSYTIYCSFLFRSCNNFNFCIIRLRCYTLWSW
FRDKRHEFRYILHVSCVIFSYIRYSISWLKCEIRFSRFSKYSSINLINIKFSYINSNYDNRKFKFNCL
YISKSYLIYTTFISCVYNIFHRIYSDKSSFFSRGPANLVWSGYMSQIARKPFYFKNKRQLAGNLIP
NKYIIKLFIDTQFNIYIYLFIYMIKDIVNHRCESTKKKHMGTHLPLLGESELVSGFMTEHAAVVF
VFFFLAEYGSIVLMCILTSILFIGGYLLFEISYVFTVVNYIFFELFFIDVTFVEVQSLYTDFLNNSIIE
GLLYGFNLGLKSSLMIFTFIARASFPRIRFDQLMGFCTVLLPINFAIIILVPCVLYSFNLLPVNIPLF
LTHPPALLPQGYSTYEEGNRSRTILVNNYTYTKTIYHTRECNYTLFLYSTLLAYYYLYMIIILLNL
LGYINKKFVNFFHHFLIQNTNF

>YN314
ITILKNIKSYIKIIHLYSINLKGIFSILYLVLLLFSLLQVFLLLGSLLNRWYARLLIKVISFYLLQYLV
LEGFIIILKKCSFIHLTINTKKLKIILLMVKLLIETIKYMLNLIIQMLCLVLWQLFLLLHFYYSCLIK
NDVWVLYWYNLYLAINEYDIIYRVCICYSYFIFSHFYICSSSLSRKIYFRVWFPFFRAKNTIRYKI
LYFCFSLFTFRFRNIINFPFRSVCWYLWSFSYFNFYSYNNYRIYIIRKRSNRQQTKIIYTIERLPYR
VCWNRGFVKLRQKTKKPTKGLMGSYKKMITYIYSYYIFTITRIIIYIYIILLYIKIIIIIRYNYSILLY
KNIIYPIINFIFFLIIIFFYIMLTHLQMKRTKYCCYSSGYRKIFLYYLFLLGSFSVRGYMDGRRVFIL
MNSGFKLTLFMNLNYNYINVGYDRIMYSLRVKRDTPTYNTYWFRVEYIYLRVKNIYIQYIINIVI
YWYGLNQVYILSFDKHLFFYLLLLLYCEINNYNIILHSTYLGILSYLAINDSNQILFSLVWNNDL
LIHWFFDSKKRVNNFLDQKYYFLQKAYAFHFFDKQLLLIFGCLLVLTITKFFQFLYKFKEYKVIH
YNILTLSMRHNQVTLVTCEILVHYLFVYKLLPVLHLCIIVLVWKLLTQSIEMLITGDFVIYIVIQL
LLSFSCIYTEEVYITDHIELLVLFELLVLLYYWLSVSVMFYLMDRCHYEVLQLLLILLVLYLEGKI
LLNSFEVVFLLIMPLTDFLHYILYCLLYLLFCTLHFMILLVQAILLVFQVITIELHLLHIFYLKILLF
LYLFLYVLLYSLCLMFGIVIIILWLILCKLLLLLYLNDTYYLSMLFDLYLINYVLRCLVLFLLCYY
LLQIVDLEVYNLDHLVKLSEFLLLISFCNVLNTLKIHLYYVNVLYYTLVILLLYYLLVTIIVLIYLI
NLNYFIKLTKYIKIIIYIFYLRYYFSILGFQFIIYIILCITLHLALANLLFPLVWNNDLLIHWFFESKK
EIRNIFLLLLILFLKNIHFALPFSCIKMNDIKNKYLNYSYVILSYIATIIRSCINTCTCLISCSLCYSC
KKNYGYAKKIRSCCRLLWTIASICCLKTFIKRICSSYTIYCSFLFRSCNNFNFCIIRLRCYTLWSW
FRDKRHEFRYILHVSCVIFSYIRYSISWLKCEIRFSRFSKYSSINLINIKFSYINSNYDNRKFKFNCL
YISKSYLIYTTFISCVYNIFHRIYSDKSSFFSRGPANLVWSGYMSQIARKPFYFKNKRQLAGNLIP
NKYIIKLFIDTQFNIYIYLFIYMIKDIVNHRCESTKKKHMGTHLPLLGESELVSGFMTEHAAVVF
VFFFLAEYGSIVLMCILTSILFIGGYLLFEISYVFTVVNYIFFELFFIDVTFVEVQSLYTDFLNNSIIE
GLLYGFNLGLKSSLMIFTFIARASFPRIRFDQLMGFCTVLLPINFAIIILVPCVLYSFNLLPVNIPLF
LTHPPALLPQGYSTYEEGNRSRTILVNNYTYTKTIYHTRECNYTLFLYSTLLAYYYLYMIIILLNL
LGYINKKFVNFFHHFLIQNTNY
>YN352
ITILKNIKSYIKIIHLYSINLKGIFSILYLVLLLFSLLQVFLLLGSLLNRWYARLLIKVISFYLLQYLV
LEGFIIILKKCSFIHLTINTKKLKIILLMVKLLIETIKYMLNLIIQMLCLVLWQLFLLLHFYYSCLIK
NDVWVLYWYNLYLAINEYDIIYRVCICYSYFIFSHFYICSSSLSRKIYFRVWFPFFRAKNTIRYKI
LYFCFSLFTFRFRNIINFPFRSVCWYLWSFSYFNFYSYNNYRIYIIRKRSNRQQTKIIYTIERLPYR
VCWNRGFVKLRQKTKKPTKGLMGSYKKMITYIYSYYIFTITRIIIYIYIILLYIKIIIIIRYNYSILLY
KNIIYPIINFIFFLIIIFFYIMLTHLQMKRTKYCCYSSGYRKIFLYYLFLLGSFSVRGYMDGRRVFIL
MNSGFKLTLFMNLNYNYINVGYDRIMYSLRVKRDTPTYNTYWFRVEYIYLRVKNIYIQYIINIVI
YWYGLNQVYILSFDKHLFFYLLLLLYCEINNYNIILHSTYLGILSYLAINDSNQILFSLVWNNDL
LIHWFFDSKKRVNNFLDQKYYFLQKAYAFHFFDKQLLLIFGCLLVLTITKFFQFLYKFKEYKVIH
YNILTLSMRHNQVTLVTCEILVHYLFVYKLLPVLHLCIIVLVWKLLTQSIEMLITGDFVIYIVIQL
LLSFSCIYTEEVYITDHIELLVLFELLVLLYYWLSVSVMFYLMDRCHYEVLQLLLILLVLYLEGKI
LLNSFEVVFLLIMPLTDFLHYILYCLLYLLFCTLHFMILLVQAILLVFQVITIELHLLHIFYLKILLF
LYLFLYVLLYSLCLMFGIVIIILWLILCKLLLLLYLNDTYYLSMLFDLYLINYVLRCLVLFLLCYY
LLQIVDLEVYNLDHLVKLSEFLLLISFCNVLNTLKIHLYYVNVLYYTLVILLLYYLLVTIIVLIYLI
NLNYFIKLTKYIKIIIYIFYLRYYFSILGFQFIIYIILCITLHLALANLLFPLVWNNDLLIHWFFESKK
EIRNIFLLLLILFLKNIHFALPFSCIKMNDIKNKYLNYSYVILSYIATIIRSCINTCTCLISCSLCYSC
KKNYGYAKKIRSCCRLLWTIASICCLKTFIKRICSSYTIYCSFLFRSCNNFNFCIIRLRCYTLWSW
FRDKRHEFRYILHVSCVIFSYIRYSISWLKCEIRFSRFSKYSSINLINIKFSYINSNYDNRKFKFNCL

YISKSYLIYTTFISCVYNIFHRIYSDKSSFFSRGPANLVWSGYMSQIARKPFYFKNKRQLAGNLIP
NKYIIKLFIDTQFNIYIYLFIYMIKDIVNHRCESTKKKHMGTHLPLLGESELVSGFMTEHAAVVF
VFFFLAEYGSIVLMCILTSILFIGGYLLFEISYVFTVVNYIFFELFFIDVTFVEVQSLYTDFLNNSIIE
GLLYGFNLGLKSSLMIFTFIARASFPRIRFDQLMGFCTVLLPINFAIIILVPCVLYSFNLLPVNIPLF
LTHPPALLPQGYSTYEEGNRSRTILVNNYTYTKTIYHTRECNYTLFLYSTLLAYYYLYMIIILLNL
LGYINKKFVNFFHHFLIQNTNF
>YN355
ITILKNIKSYIKIIHLYSINLKGIFSILYLVLLLFSLLQVFLLLGSLLNRWYARLLIKVISFYLLQYLV
LEGFIIILKKCSFIHLTINTKKLKIILLMVKLLIETIKYMLNLIIQMLCLVLWQLFLLLHFYYSCLIK
NDVWVLYWYNLYLAINEYDIIYRVCICYSYFIFSHFYICSSSLSRKIYFRVWFPFFRAKNTIRYKI
LYFCFSLFTFRFRNIINFPFRSVCWYLWSFSYFNFYSYNNYRIYIIRKRSNRQQTKIIYTIERLPYR
VCWNRGFVKLRQKTKKPTKGLMGSYKKMITYIYSYYIFTITRIIIYIYIILLYIKIIIIIRYNYSILLY
KNIIYPIINFIFFLIIIFFYIMLTHLQMKRTKYCCYSSGYRKIFLYYLFLLGSFSVRGYMDGRRVFIL
MNSGFKLTLFMNLNYNYINVGYDRIMYSLRVKRDTPTYNTYWFRVEYIYLRVKNIYIQYIINIVI
YWYGLNQVYILSFDKHLFFYLLLLLYCEINNYNIILHSTYLGILSYLAINDSNQILFSLVWNNDL
LIHWFFDSKKRVNNFLDQKYYFLQKAYAFHFFDKQLLLIFGCLLVLTITKFFQFLYKFKEYKVIH
YNILTLSMRHNQVTLVTCEILVHYLFVYKLLPVLHLCIIVLVWKLLTQSIEMLITGDFVIYIVIQL
LLSFSCIYTEEVYITDHIELLVLFELLVLLYYWLSVSVMFYLMDRCHYEVLQLLLILLVLYLEGKI
LLNSFEVVFLLIMPLTDFLHYILYCLLYLLFCTLHFMILLVQAILLVFQVITIELHLLHIFYLKILLF
LYLFLYVLLYSLCLMFGIVIIILWLILCKLLLLLYLNDTYYLSMLFDLYLINYVLRCLVLFLLCYY
LLQIVDLEVYNLDHLVKLSEFLLLISFCNVLNTLKIHLYYVNVLYYTLVILLLYYLLVTIIVLIYLI
NLNYFIKLTKYIKIIIYIFYLRYYFSILGFQFIIYIILCITLHLALANLLFPLVWNNDLLIHWFFESKK
EIRNIFLLLLILFLKNIHFALPFSCIKMNDIKNKYLNYSYVILSYIATIIRSCINTCTCLISCSLCYSC
KKNYGYAKKIRSCCRLLWTIASICCLKTFIKRICSSYTIYCSFLFRSCNNFNFCIIRLRCYTLWSW
FRDKRHEFRYILHVSCVIFSYIRYSISWLKCEIRFSRFSKYSSINLINIKFSYINSNYDNRKFKFNCL
YISKSYLIYTTFISCVYNIFHRIYSDKSSFFSRGPANLVWSGYMSQIARKPFYFKNKRQLAGNLIP
NKYIIKLFIDTQFNIYIYLFIYMIKDIVNHRCESTKKKHMGTHLPLLGESELVSGFMTEHAAVVF
VFFFLAEYGSIVLMCILTSILFIGGYLLFEISYVFTVVNYIFFELFFIDVTFVEVQSLYTDFLNNSIIE
GLLYGFNLGLKSSLMIFTFIARASFPRIRFDQLMGFCTVLLPINFAIIILVPCVLYSFNLLPVNIPLF
LTHPPALLPQGYSTYEEGNRSRTILVNNYTYTKTIYHTRECNYTLFLYSTLLAYYYLYMIIILLNL
LGYINKKFVNFFHHFLIQNTNF
>YN358
ITILKNIKSYIKIIHLYSINLKGIFSILYLVLLLFSLLQVFLLLGSLLNRWYARLLIKVISFYLLQYLV
LEGFIIILKKCSFIHLTINTKKLKIILLMVKLLIETIKYMLNLIIQMLCLVLWQLFLLLHFYYSCLIK
NDVWVLYWYNLYLAINEYDIIYRVCICYSYFIFSHFYICSSSLSRKIYFRVWFPFFRAKNTIRYKI
LYFCFSLFTFRFRNIINFPFRSVCWYLWSFSYFNFYSYNNYRIYIIRKRSNRQQTKIIYTIERLPYR
VCWNRGFVKLRQKTKKPTKGLMGSYKKMITYIYSYYIFTITRIIIYIYIILLYIKIIIIIRYNYSILLY
KNIIYPIINFIFFLIIIFFYIMLTHLQMKRTKYCCYSSGYRKIFLYYLFLLGSFSVRGYMDGRRVFIL
MNSGFKLTLFMNLNYNYINVGYDRIMYSLRVKRDTPTYNTYWFRVEYIYLRVKNIYIQYIINIVI
YWYGLNQVYILSFDKHLFFYLLLLLYCEINNYNIILHSTYLGILSYLAINDSNQILFSLVWNNDL
LIHWFFDSKKRVNNFLDQKYYFLQKAYAFHFFDKQLLLIFGCLLVLTITKFFQFLYKFKEYKVIH
YNILTLSMRHNQVTLVTCEILVHYLFVYKLLPVLHLCIIVLVWKLLTQSIEMLITGDFVIYIVIQL
LLSFSCIYTEEVYITDHIELLVLFELLVLLYYWLSVSVMFYLMDRCHYEVLQLLLILLVLYLEGKI
LLNSFEVVFLLIMPLTDFLHYILYCLLYLLFCTLHFMILLVQAILLVFQVITIELHLLHIFYLKILLF

LYLFLYVLLYSLCLMFGIVIIILWLILCKLLLLLYLNDTYYLSMLFDLYLINYVLRCLVLFLLCYY
LLQIVDLEVYNLDHLVKLSEFLLLISFCNVLNTLKIHLYYVNVLYYTLVILLLYYLLVTIIVLIYLI
NLNYFIKLTKYIKIIIYIFYLRYYFSILGFQFIIYIILCITLHLALANLLFPLVWNNDLLIHWFFESKK
EIRNIFLLLLILFLKNIHFALPFSCIKMNDIKNKYLNYSYVILSYIATIIRSCINTCTCLISCSLCYSC
KKNYGYAKKIRSCCRLLWTIASICCLKTFIKRICSSYTIYCSFLFRSCNNFNFCIIRLRCYTLWSW
FRDKRHEFRYILHVSCVIFSYIRYSISWLKCEIRFSRFSKYSSINLINIKFSYINSNYDNRKFKFNCL
YISKSYLIYTTFISCVYNIFHRIYSDKSSFFSRGPANLVWSGYMSQIARKPFYFKNKRQLAGNLIP
NKYIIKLFIDTQFNIYIYLFIYMIKDIVNHRCESTKKKHMGTHLPLLGESELVSGFMTEHAAVVF
VFFFLAEYGSIVLMCILTSILFIGGYLLFEISYVFTVVNYIFFELFFIDVTFVEVQSLYTDFLNNSIIE
GLLYGFNLGLKSSLMIFTFIARASFPRIRFDQLMGFCTVLLPINFAIIILVPCVLYSFNLLPVNIPLF
LTHPPALLPQGYSTYEEGNRSRTILVNNYTYTKTIYHTRECNYTLFLYSTLLAYYYLYMIIILLNL
LGYINKKFVNFFHHFLIQNTNF
>YN359
ITILKNIKSYIKIIHLYSINLKGIFSILYLVLLLFSLLQVFLLLGSLLNRWYARLLIKVISFYLLQYLV
LEGFIIILKKCSFIHLTINTKKLKIILLMVKLLIETIKYMLNLIIQMLCLVLWQLFLLLHFYYSCLIK
NDVWVLYWYNLYLAINEYDIIYRVCICYSYFIFSHFYICSSSLSRKIYFRVWFPFFRAKNTIRYKI
LYFCFSLFTFRFRNIINFPFRSVCWYLWSFSYFNFYSYNNYRIYIIRKRSNRQQTKIIYTIERLPYR
VCWNRGFVKLRQKTKKPTKGLMGSYKKMITYIYSYYIFTITRIIIYIYIILLYIKIIIIIRYNYSILLY
KNIIYPIINFIFFLIIIFFYIMLTHLQMKRTKYCCYSSGYRKIFLYYLFLLGSFSVRGYMDGRRVFIL
MNSGFKLTLFMNLNYNYINVGYDRIMYSLRVKRDTPTYNTYWFRVEYIYLRVKNIYIQYIINIVI
YWYGLNQVYILSFDKHLFFYLLLLLYCEINNYNIILHSTYLGILSYLAINDSNQILFSLVWNNDL
LIHWFFDSKKRVNNFLDQKYYFLQKAYAFHFFDKQLLLIFGCLLVLTITKFFQFLYKFKEYKVIH
YNILTLSMRHNQVTLVTCEILVHYLFVYKLLPVLHLCIIVLVWKLLTQSIEMLITGDFVIYIVIQL
LLSFSCIYTEEVYITDHIELLVLFELLVLLYYWLSVSVMFYLMDRCHYEVLQLLLILLVLYLEGKI
LLNSFEVVFLLIMPLTDFLHYILYCLLYLLFCTLHFMILLVQAILLVFQVITIELHLLHIFYLKILLF
LYLFLYVLLYSLCLMFGIVIIILWLILCKLLLLLYLNDTYYLSMLFDLYLINYVLRCLVLFLLCYY
LLQIVDLEVYNLDHLVKLSEFLLLISFCNVLNTLKIHLYYVNVLYYTLVILLLYYLLVTIIVLIYLI
NLNYFIKLTKYIKIIIYIFYLRYYFSILGFQFIIYIILCITLHLALANLLFPLVWNNDLLIHWFFESKK
EIRNIFLLLLILFLKNIHFALPFSCIKMNDIKNKYLNYSYVILSYIATIIRSCINTCTCLISCSLCYSC
KKNYGYAKKIRSCCRLLWTIASICCLKTFIKRICSSYTIYCSFLFRSCNNFNFCIIRLRCYTLWSW
FRDKRHEFRYILHVSCVIFSYIRYSISWLKCEIRFSRFSKYSSINLINIKFSYINSNYDNRKFKFNCL
YISKSYLIYTTFISCVYNIFHRIYSDKSSFFSRGPANLVWSGYMSQIARKPFYFKNKRQLAGNLIP
NKYIIKLFIDTQFNIYIYLFIYMIKDIVNHRCESTKKKHMGTHLPLLGESELVSGFMTEHAAVVF
VFFFLAEYGSIVLMCILTSILFIGGYLLFEISYVFTVVNYIFFELFFIDVTFVEVQSLYTDFLNNSIIE
GLLYGFNLGLKSSLMIFTFIARASFPRIRFDQLMGFCTVLLPINFAIIILVPCVLYSFNLLPVNIPLF
LTHPPALLPQGYSTYEEGNRSRTILVNNYTYTKTIYHTRECNYTLFLYSTLLAYYYLYMIIILLNL
LGYINKKFVNFFHHFLIQNTNF
>YN365
ITILKNIKSYIKIIHLYSINLKGIFSILYLVLLLFSLLQVFLLLGSLLNRWYARLLIKVISFYLLQYLV
LEGFIIILKKCSFIHLTINTKKLKIILLMVKLLIETIKYMLNLIIQMLCLVLWQLFLLLHFYYSCLIK
NDVWVLYWYNLYLAINEYDIIYRVCICYSYFIFSHFYICSSSLSRKIYFRVWFPFFRAKNTIRYKI
LYFCFSLFTFRFRNIINFPFRSVCWYLWSFSYFNFYSYNNYRIYIIRKRSNRQQTKIIYTIERLPYR
VCWNRGFVKLRQKTKKPTKGLMGSYKKMITYIYSYYIFTITRIIIYIYIILLYIKIIIIIRYNYSILLY
KNIIYPIINFIFFLIIIFFYIMLTHLQMKRTKYCCYSSGYRKIFLYYLFLLGSFSVRGYMDGRRVFIL

MNSGFKLTLFMNLNYNYINVGYDRIMYSLRVKRDTPTYNTYWFRVEYIYLRVKNIYIQYIINIVI
YWYGLNQVVYILSFDKHLFFYLLLLLYCEINNYNIILHSTYLGILSYLAINDSNQILFSLVWNNDL
LIHWFFDSKKRVNNFLDQKYYFLQKAYAFHFFDKQLLLIFGCLLVLTITKFFQFLYKFKEYKVIH
YNILTLSMRHNQVTLVTCEILVHYLFVYKLLPVLHLCIIVLVWKLLTQSIEMLITGDFVIYIVIQL
LLSFSCIYTEEVYITDHIELLVLFELLVLLYYWLSVSVMFYLMDRCHYEVLQLLLILLVLYLEGKI
LLNSFEVVFLLIMPLTDFLHYILYCLLYLLFCTLHFMILLVQAILLVFQVITIELHLLHIFYLKILLF
LYLFLYVLLYSLCLMFGIVIIILWLILCKLLLLLYLNDTYYLSMLFDLYLINYVLRCLVLFLLCYY
LLQIVDLEVYNLDHLVKLSEFLLLISFCNVLNTLKIHLYYVNVLYYTLVILLLYYLLVTIIVLIYLI
NLNYFIKLTKYIKIIIYIFYLRYYFSILGFQFIIYIILCITLHLALANLLFPLVWNNDLLIHWFFESKK
EIRNIFLLLLILFLKNIHFALPFSCIKMNDIKNKYLNYSYVILSYIATIIRSCINTCTCLISCSLCYSC
KKNYGYAKKIRSCCRLLWTIASICCLKTFIKRICSSYTIYCSFLFRSCNNFNFCIIRLRCYTLWSW
FRDKRHEFRYILHVSCVIFSYIRYSISWLKCEIRFSRFSKYSSINLINIKFSYINSNYDNRKFKFNCL
YISKSYLIYTTFISCVYNIFHRIYSDKSSFFSRGPANLVWSGYMSQIARKPFYFKNKRQLAGNLIP
NKYIIKLFIDTQFNIYIYLFIYMIKDIVNHRCESTKKKHMGTHLPLLGESELVSGFMTEHAAVVF
VFFFLAEYGSIVLMCILTSILFIGGYLLFEISYVFTVVNYIFFELFFIDVTFVEVQSLYTDFLNNSIIE
GLLYGFNLGLKSSLMIFTFIARASFPRIRFDQLMGFCTVLLPINFAIIILVPCVLYSFNLLPVNIPLF
LTHPPALLPQGYSTYEEGNRSRTILVNNYTYTKTIYHTRECNYTLFLYSTLLAYYYLYMIIILLNL
LGYINKKFVNFFHHFLIQNTNF
>YN376
ITILKNIKSYIKIIHLYSINLKGIFSILYLVLLLFSLLQVFLLLGSLLNRWYARLLIKVISFYLLQYLV
LEGFIIILKKCSFIHLTINTKKLKIILLMVKLLIETIKYMLNLIIQMLCLVLWQLFLLLHFYYSCLIK
NDVWVLYWYNLYLAINEYDIIYRVCICYSYFIFSHFYICSSSLSRKIYFRVWFPFFRAKNTIRYKI
LYFCFSLFTFRFRNIINFPFRSVCWYLWSFSYFNFYSYNNYRIYIIRKRSNRQQTKIIYTIERLPYR
VCWNRGFVKLRQKTKKPTKGLMGSYKKMITYIYSYYIFTITRIIIYIYIILLYIKIIIIIRYNYSILLY
KNIIYPIINFIFFLIIIFFYIMLTHLQMKRTKYCCYSSGYRKIFLYYLFLLGSFSVRGYMDGRRVFIL
MNSGFKLTLFMNLNYNYINVGYDRIMYSLRVKRDTPTYNTYWFRVEYIYLRVKNIYIQYIINIVI
YWYGLNQVVYILSFDKHLFFYLLLLLYCEINNYNIILHSTYLGILSYLAINDSNQILFSLVWNNDL
LIHWFFDSKKRVNNFLDQKYYFLQKAYAFHFFDKQLLLIFGCLLVLTITKFFQFLYKFKEYKVIH
YNILTLSMRHNQVTLVTCEILVHYLFVYKLLPVLHLCIIVLVWKLLTQSIEMLITGDFVIYIVIQL
LLSFSCIYTEEVYITDHIELLVLFELLVLLYYWLSVSVMFYLMDRCHYEVLQLLLILLVLYLEGKI
LLNSFEVVFLLIMPLTDFLHYILYCLLYLLFCTLHFMILLVQAILLVFQVITIELHLLHIFYLKILLF
LYLFLYVLLYSLCLMFGIVIIILWLILCKLLLLLYLNDTYYLSMLFDLYLINYVLRCLVLFLLCYY
LLQIVDLEVYNLDHLVKLSEFLLLISFCNVLNTLKIHLYYVNVLYYTLVILLLYYLLVTIIVLIYLI
NLNYFIKLTKYIKIIIYIFYLRYYFSILGFQFIIYIILCITLHLALANLLFPLVWNNDLLIHWFFESKK
EIRNIFLLLLILFLKNIHFALPFSCIKMNDIKNKYLNYSYVILSYIATIIRSCINTCTCLISCSLCYSC
KKNYGYAKKIRSCCRLLWTIASICCLKTFIKRICSSYTIYCSFLFRSCNNFNFCIIRLRCYTLWSW
FRDKRHEFRYILHVSCVIFSYIRYSISWLKCEIRFSRFSKYSSINLINIKFSYINSNYDNRKFKFNCL
YISKSYLIYTTFISCVYNIFHRIYSDKSSFFSRGPANLVWSGYMSQIARKPFYFKNKRQLAGNLIP
NKYIIKLFIDTQFNIYIYLFIYMIKDIVNHRCESTKKKHMGTHLPLLGESELVSGFMTEHAAVVF
VFFFLAEYGSIVLMCILTSILFIGGYLLFEISYVFTVVNYIFFELFFIDVTFVEVQSLYTDFLNNSIIE
GLLYGFNLGLKSSLMIFTFIARASFPRIRFDQLMGFCTVLLPINFAIIILVPCVLYSFNLLPVNIPLF
LTHPPALLPQGYSTYEEGNRSRTILVNNYTYTKTIYHTRECNYTLFLYSTLLAYYYLYMIIILLNL
LGYINKKFVNFFHHFLIQNTNF
>YN390

ITILKNIKSYIKIIHLYSINLKGIFSILYLVLLLFSLLQVFLLLGSLLNRWYARLLIKVISFYLLQYLV
LEGFIIILKKCSFIHLTINTKKLKIILLMVKLLIETIKYMLNLIIQMLCLVLWQLFLLLHFYYSCLIK
NDVWVLYWYNLYLAINEYDIIYRVCICYSYFIFSHFYICSSSLSRKIYFRVWFPFFRAKNTIRYKI
LYFCFSLFTFRFRNIINFPFRSVCWYLWSFSYFNFYSYNNYRIYIIRKRSNRQQTKIIYTIERLPYR
VCWNRGFVKLRQKTKKPTKGLMGSYKKMITYIYSYYIFTITRIIIYIYIILLYIKIIIIIRYNYSILLY
KNIIYPIINFIFFLIIIFFYIMLTHLQMKRTKYCCYSSGYRKIFLYYLFLLGSFSVRGYMDGRRVFIL
MNSGFKLTLFMNLNYNYINVGYDRIMYSLRVKRDTPTYNTYWFRVEYIYLRVKNIYIQYIINIVI
YWYGLNQVYILSFDKHLFFYLLLLLYCEINNYNIILHSTYLGILSYLAINDSNQILFSLVWNNDL
LIHWFFDSKKRVNNFLDQKYYFLQKAYAFHFFDKQLLLIFGCLLVLTITKFFQFLYKFKEYKVIH
YNILTLSMRHNQVTLVTCEILVHYLFVYKLLPVLHLCIIVLVWKLLTQSIEMLITGDFVIYIVIQL
LLSFSCIYTEEVYITDHIELLVLFELLVLLYYWLSVSVMFYLMDRCHYEVLQLLLILLVLYLEGKI
LLNSFEVVFLLIMPLTDFLHYILYCLLYLLFCTLHFMILLVQAILLVFQVITIELHLLHIFYLKILLF
LYLFLYVLLYSLCLMFGIVIIILWLILCKLLLLLYLNDTYYLSMLFDLYLINYVLRCLVLFLLCYY
LLQIVDLEVYNLDHLVKLSEFLLLISFCNVLNTLKIHLYYVNVLYYTLVILLLYYLLVTIIVLIYLI
NLNYFIKLTKYIKIIIYIFYLRYYFSILGFQFIIYIILCITLHLALANLLFPLVWNNDLLIHWFFESKK
EIRNIFLLLLILFLKNIHFALPFSCIKMNDIKNKYLNYSYVILSYIATIIRSCINTCTCLISCSLCYSC
KKNYGYAKKIRSCCRLLWTIASICCLKTFIKRICSSYTIYCSFLFRSCNNFNFCIIRLRCYTLWSW
FRDKRHEFRYILHVSCVIFSYIRYSISWLKCEIRFSRFSKYSSINLINIKFSYINSNYDNRKFKFNCL
YISKSYLIYTTFISCVYNIFHRIYSDKSSFFSRGPANLVWSGYMSQIARKPFYFKNKRQLAGNLIP
NKYIIKLFIDTQFNIYIYLFIYMIKDIVNHRCESTKKKHMGTHLPLLGESELVSGFMTEHAAVVF
VFFFLAEYGSIVLMCILTSILFIGGYLLFEISYVFTVVNYIFFELFFIDVTFVEVQSLYTDFLNNSIIE
GLLYGFNLGLKSSLMIFTFIARASFPRIRFDQLMGFCTVLLPINFAIIILVPCVLYSFNLLPVNIPLF
LTHPPALLPQGYSTYEEGNRSRTILVNNYTYTKTIYHTRECNYTLFLYSTLLGYYYLYMIIILLNL
LGYINKKFVNFFHHFLIQNTNF
>YN391
ITILKNIKSYIKIIHLYSINLKGIFSILYLVLLLFSLLQVFLLLGSLLNRWYARLLIKVISFYLLQYLV
LEGFIIILKKCSFIHLTINTKKLKIILLMVKLLIETIKYMLNLIIQMLCLVLWQLFLLLHFYYSCLIK
NDVWVLYWYNLYLAINEYDIIYRVCICYSYFIFSHFYICSSSLSRKIYFRVWFPFFRAKNTIRYKI
LYFCFSLFTFRFRNIINFPFRSVCWYLWSFSYFNFYSYNNYRIYIIRKRSNRQQTKIIYTIERLPYR
VCWNRGFVKLRQKTKKPTKGLMGSYKKMITYIYSYYIFTITRIIIYIYIILLYIKIIIIIRYNYSILLY
KNIIYPIINFIFFLIIIFFYIMLTHLQMKRTKYCCYSSGYRKIFLYYLFLLGSFSVRGYMDGRRVFIL
MNSGFKLTLFMNLNYNYINVGYDRIMYSLRVKRDTPTYNTYWFRVEYIYLRVKNIYIQYIINIVI
YWYGLNQVYILSFDKHLFFYLLLLLYCEINNYNIILHSTYLGILSYLAINDSNQILFSLVWNNDL
LIHWFFDSKKRVNNFLDQKYYFLQKAYAFHFFDKQLLLIFGCLLVLTITKFFQFLYKFKEYKVIH
YNILTLSMRHNQVTLVTCEILVHYLFVYKLLPVLHLCIIVLVWKLLTQSIEMLITGDFVIYIVIQL
LLSFSCIYTEEVYITDHIELLVLFELLVLLYYWLSVSVMFYLMDRCHYEVLQLLLILLVLYLEGKI
LLNSFEVVFLLIMPLTDFLHYILYCLLYLLFCTLHFMILLVQAILLVFQVITIELHLLHIFYLKILLF
LYLFLYVLLYSLCLMFGIVIIILWLILCKLLLLLYLNDTYYLSMLFDLYLINYVLRCLVLFLLCYY
LLQIVDLEVYNLDHLVKLSEFLLLISFCNVLNTLKIHLYYVNVLYYTLVILLLYYLLVTIIVLIYLI
NLNYFIKLTKYIKIIIYIFYLRYYFSILGFQFIIYIILCITLHLALANLLFPLVWNNDLLIHWFFESKK
EIRNIFLLLLILFLKNIHFALPFSCIKMNDIKNKYLNYSYVILSYIATIIRSCINTCTCLISCSLCYSC
KKNYGYAKKIRSCCRLLWTIASICCLKTFIKRICSSYTIYCSFLFRSCNNFNFCIIRLRCYTLWSW
FRDKRHEFRYILHVSCVIFSYIRYSISWLKCEIRFSRFSKYSSINLINIKFSYINSNYDNRKFKFNCL
YISKSYLIYTTFISCVYNIFHRIYSDKSSFFSRGPANLVWSGYMSQIARKPFYFKNKRQLAGNLIP

NKYIIKLFIDTQFNIYIYLFIYMIKDIVNHRCESTKKKHMGTHLPLLGESELVSGFMTEHAAVVF
VFFFLAEYGSIVLMCILTSILFIGGYLLFEISYVFTVVNYIFFELFFIDVTFVEVQSLYTDFLNNSIIE
GLLYGFNLGLKSSLMIFTFIARASFPRIRFDQLMGFCTVLLPINFAIIILVPCVLYSFNLLPVNIPLF
LTHPPALLPQGYSTYEEGNRSRTILVNNYTYTKTIYHTRECNYTLFLYSTLLAYYYLYMIIILLNL
LGYINKKFVNFFHHFLIQNTNF
>YN397
ITILKNIKSYIKIIHLYSINLKGIFSILYLVLLLFSLLQVFLLLGSLLNRWYARLLIKVISFYLLQYLV
LEGFIIILKKCSFIHLTINTKKLKIILLMVKLLIETIKYMLNLIIQMLCLVLWQLFLLLHFYYSCLIK
NDVWVLYWYNLYLAINEYDIIYRVCICYSYFIFSHFYICSSSLSRKIYFRVWFPFFRAKNTIRYKI
LYFCFSLFTFRFRNIINFPFRSVCWYLWSFSYFNFYSYNNYRIYIIRKRSNRQQTKIIYTIERLPYR
VCWNRGFVKLRQKTKKPTKGLMGSYKKMITYIYSYYIFTITRIIIYIYIILLYIKIIIIIRYNYSILLY
KNIIYPIINFIFFLIIIFFYIMLTHLQMKRTKYCCYSSGYRKIFLYYLFLLGSFSVRGYMDGRRVFIL
MNSGFKLTLFMNLNYNYINVGYDRIMYSLRVKRDTPTYNTYWFRVEYIYLRVKNIYIQYIINIVI
YWYGLNQVVYILSFDKHLFFYLLLLLYCEINNYNIILHSTYLGILSYLAINDSNQILFSLVWNNDL
LIHWFFDSKKRVNNFLDQKYYFLQKAYAFHFFDKQLLLIFGCLLVLTITKFFQFLYKFKEYKVIH
YNILTLSMRHNQVTLVTCEILVHYLFVYKLLPVLHLCIIVLVWKLLTQSIEMLITGDFVIYIVIQL
LLSFSCIYTEEVYITDHIELLVLFELLVLLYYWLSVSVMFYLMDRCHYEVLQLLLILLVLYLEGKI
LLNSFEVVFLLIMPLTDFLHYILYCLLYLLFCTLHFMILLVQAILLVFQVITIELHLLHIFYLKILLF
LYLFLYVLLYSLCLMFGIVIIILWLILCKLLLLLYLNDTYYLSMLFDLYLINYVLRCLVLFLLCYY
LLQIVDLEVYNLDHLVKLSEFLLLISFCNVLNTLKIHLYYVNVLYYTLVILLLYYLLVTIIVLIYLI
NLNYFIKLTKYIKIIIYIFYLRYYFSILGFQFIIYIILCITLHLALANLLFPLVWNNDLLIHWFFESKK
EIRNIFLLLLILFLKNIHFALPFSCIKMNDIKNKYLNYSYVILSYIATIIRSCINTCTCLISCSLCYSC
KKNYGYAKKIRSCCRLLWTIASICCLKTFIKRICSSYTIYCSFLFRSCNNFNFCIIRLRCYTLWSW
FRDKRHEFRYILHVSCVIFSYIRYSISWLKCEIRFSRFSKYSSINLINIKFSYINSNYDNRKFKFNCL
YISKSYLIYTTFISCVYNIFHRIYSDKSSFFSRGPANLVWSGYMSQIARKPFYFKNKRQLAGNLIP
NKYIIKLFIDTQFNIYIYLFIYMIKDIVNHRCESTKKKHMGTHLPLLGESELVSGFMTEHAAVVF
VFFFLAEYGSIVLMCILTSILFIGGYLLFEISYVFTVVNYIFFELFFIDVTFVEVQSLYTDFLNNSIIE
GLLYGFNLGLKSSLMIFTFIARASFPRIRFDQLMGFCTVLLPINFAIIILVPCVLYSFNLLPVNIPLF
LTHPPALLPQGYSTYEEGNRSRTILVNNYTYTKTIYHTRECNYTLFLYSTLLAYYYLYMIIILLNL
LGYINKKFVNFFHHFLIQNTNF
>YN398
ITILKNIKSYIKIIHLYSINLKGIFSILYLVLLLFSLLQVFLLLGSLLNRWYARLLIKVISFYLLQYLV
LEGFIIILKKCSFIHLTINTKKLKIILLMVKLLIETIKYMLNLIIQMLCLVLWQLFLLLHFYYSCLIK
NDVWVLYWYNLYLAINEYDIIYRVCICYSYFIFSHFYICSSSLSRKIYFRVWFPFFRAKNTIRYKI
LYFCFSLFTFRFRNIINFPFRSVCWYLWSFSYFNFYSYNNYRIYIIRKRSNRQQTKIIYTIERLPYR
VCWNRGFVKLRQKTKKPTKGLMGSYKKMITYIYSYYIFTITRIIIYIYIILLYIKIIIIIRYNYSILLY
KNIIYPIINFIFFLIIIFFYIMLTHLQMKRTKYCCYSSGYRKIFLYYLFLLGSFSVRGYMDGRRVFIL
MNSGFKLTLFMNLNYNYINVGYDRIMYSLRVKRDTPTYNTYWFRVEYIYLRVKNIYIQYIINIVI
YWYGLNQVVYILSFDKHLFFYLLLLLYCEINNYNIILHSTYLGILSYLAINDSNQILFSLVWNNDL
LIHWFFDSKKRVNNFLDQKYYFLQKAYAFHFFDKQLLLIFGCLLVLTITKFFQFLYKFKEYKVIH
YNILTLSMRHNQVTLVTCEILVHYLFVYKLLPVLHLCIIVLVWKLLTQSIEMLITGDFVIYIVIQL
LLSFSCIYTEEVYITDHIELLVLFELLVLLYYWLSVSVMFYLMDRCHYEVLQLLLILLVLYLEGKI
LLNSFEVVFLLIMPLTDFLHYILYCLLYLLFCTLHFMILLVQAILLVFQVITIELHLLHIFYLKILLF
LYLFLYVLLYSLCLMFGIVIIILWLILCKLLLLLYLNDTYYLSMLFDLYLINYVLRCLVLFLLCYY

LLQIVDLEVYNLDHLVKLSEFLLLISFCNVLNTLKIHLYYVNVLYYTLVILLLYYLLVTIIVLIYLI
NLNYFIKLTKYIKIIIYIFYLRYYFSILGFQFIIYIILCITLHLALANLLFPLVWNNDLLIHWFFESKK
EIRNIFLLLLILFLKNIHFALPFSCIKMNDIKNKYLNYSYVILSYIATIIRSCINTCTCLISCSLCYSC
KKNYGYAKKIRSCCRLLWTIASICCLKTFIKRICSSYTIYCSFLFRSCNNFNFCIIRLRCYTLWSW
FRDKRHEFRYILHVSCVIFSYIRYSISWLKCEIRFSRFSKYSSINLINIKFSYINSNYDNRKFKFNCL
YISKSYLIYTTFISCVYNIFHRIYSDKSSFFSRGPANLVWSGYMSQIARKPFYFKNKRQLAGNLIP
NKYIIKLFIDTQFNIYIYLFIYMIKDIVNHRCESTKKKHMGTHLPLLGESELVSGFMTEHAAVVF
VFFFLAEYGSIVLMCILTSILFIGGYLLFEISYVFTVVNYIFFELFFIDVTFVEVQSLYTDFLNNSIIE
GLLYGFNLGLKSSLMIFTFIARASFPRIRFDQLMGFCTVLLPINFAIIILVPCVLYSFNLLPVNIPLF
LTHPPALLPQGYSTYEEGNRSRTILVNNYTYTKTIYHTRECNYTLFLYSTLLAYYYLYMIIILLNL
LGYINKKFVNFFHHFLIQNTNF
>YN406
ITILKNIKSYIKIIHLYSINLKGIFSILYLVLLLFSLLQVFLLLGSLLNRWYARLLIKVISFYLLQYLV
LEGFIIILKKCSFIHLTINTKKLKIILLMVKLLIETIKYMLNLIIQMLCLVLWQLFLLLHFYYSCLIK
NDVWVLYWYNLYLAINEYDIIYRVCICYSYFIFSHFYICSSSLSRKIYFRVWFPFFRAKNTIRYKI
LYFCFSLFTFRFRNIINFPPFRSVCWYLWSFSYFNFYSYNNYRIYIIRKRSNRQQTKIIYTIERLPYR
VCWNRGFVKLRQKTKKPTKGLMGSYKKMITYIYSYYIFTITRIIIYIYIILLYIKIIIIIRYNYSILLY
KNIIYPIINFIFFLIIIFFYIMLTHLQMKRTKYCCYSSGYRKIFLYYLFLLGSFSVRGYMDGRRVFIL
MNSGFKLTLFMNLNYNYINVGYDRIMYSLRVKRDTPTYNTYWFRVEYIYLRVKNIYIQYIINIVI
YWYGLNQVYILSFDKHLFFYLLLLLYCEINNYNIILHSTYLGILSYLAINDSNQILFSLVWNNDL
LIHWFFDSKKRVNNFLDQKYYFLQKAYAFHFFDKQLLLIFGCLLVLTITKFFQFLYKFKEYKVIH
YNILTLSMRHNQVTLVTCEILVHYLFVYKLLPVLHLCIIVLVWKLLTQSIEMLITGDFVIYIVIQL
LLSFSCIYTEEVYITDHIELLVLFELLVLLYYWLSVSVMFYLMDRCHYEVLQLLLILLVLYLEGKI
LLNSFEVVFLLIMPLTDFLHYILYCLLYLLFCTLHFMILLVQAILLVFQVITIELHLLHIFYLKILLF
LYLFLYVLLYSLCLMFGIVIIILWLILCKLLLLLYLNDTYYLSMLFDLYLINYVLRCLVLFLLCYY
LLQIVDLEVYNLDHLVKLSEFLLLISFCNVLNTLKIHLYYVNVLYYTLVILLLYYLLVTIIVLIYLI
NLNYFIKLTKYIKIIIYIFYLRYYFSILGFQFIIYIILCITLHLALANLLFPLVWNNDLLIHWFFESKK
EIRNIFLLLLILFLKNIHFALPFSCIKMNDIKNKYLNYSYVILSYIATIIRSCINTCTCLISCSLCYSC
KKNYGYAKKIRSCCRLLWTIASICCLKTFIKRICSSYTIYCSFLFRSCNNFNFCIIRLRCYTLWSW
FRDKRHEFRYILHVSCVIFSYIRYSISWLKCEIRFSRFSKYSSINLINIKFSYINSNYDNRKFKFNCL
YISKSYLIYTTFISCVYNIFHRIYSDKSSFFSRGPANLVWSGYMSQIARKPFYFKNKRQLAGNLIP
NKYIIKLFIDTQFNIYIYLFIYMIKDIVNHRCESTKKKHMGTHLPLLGESELVSGFMTEHAAVVF
VFFFLAEYGSIVLMCILTSILFIGGYLLFEISYVFTVVNYIFFELFFIDVTFVEVQSLYTDFLNNSIIE
GLLYGFNLGLKSSLMIFTFIARASFPRIRFDQLMGFCTVLLPINFAIIILVPCVLYSFNLLPVNIPLF
LTHPPALLPQGYSTYEEGNRSRTILVNNYTYTKTIYHTRECNYTLFLYSTLLAYYYLYMIIILLNL
LGYINKKFVNFFHHFLIQNTNY
>YN410
ITILKNIKSYIKIIHLYSINLKGIFSILYLVLLLFSLLQVFLLLGSLLNRWYARLLIKVISFYLLQYLV
LEGFIIILKKCSFIHLTINTKKLKIILLMVKLLIETIKYMLNLIIQMLCLVLWQLFLLLHFYYSCLIK
NDVWVLYWYNLYLAINEYDIIYRVCICYSYFIFSHFYICSSSLSRKIYFRVWFPFFRAKNTIRYKI
LYFCFSLFTFRFRNIINFPPFRSVCWYLWSFSYFNFYSYNNYRIYIIRKRSNRQQTKIIYTIERLPYR
VCWNRGFVKLRQKTKKPTKGLMGSYKKMITYIYSYYIFTITRIIIYIYIILLYIKIIIIIRYNYSILLY
KNIIYPIINFIFFLIIIFFYIMLTHLQMKRTKYCCYSSGYRKIFLYYLFLLGSFSVRGYMDGRRVFIL
MNSGFKLTLFMNLNYNYINVGYDRIMYSLRVKRDTPTYNTYWFRVEYIYLRVKNIYIQYIINIVI

YWYGLNQVVYILSFDKHLFFYLLLLLYCEINNYNIILHSTYLGILSYLAINDSNQILFSLVWNNDL
LIHWFFDSKKRVNNFLDQKYYFLQKAYAFHFFDKQLLLIFGCLLVLTITKFFQFLYKFKEYKVIH
YNILTLSMRHNQVTLVTCEILVHYLFVYKLLPVLHLCIIVLVWKLLTQSIEMLITGDFVIYIVIQL
LLSFSCIYTEEVYITDHIELLVLFELLVLLYYWLSVSVMFYLMDRCHYEVLQLLLILLVLYLEGKI
LLNSFEVVFLLIMPLTDFLHYILYCLLYLLFCTLHFMILLVQAILLVFQVITIELHLLHIFYLKILLF
LYLFLYVLLYSLCLMFGIVIIILWLILCKLLLLLYLNDTYYLSMLFDLYLINYVLRCLVLFLLCYY
LLQIVDLEVYNLDHLVKLSEFLLLISFCNVLNTLKIHLYYVNVLYYTLVILLLYYLLVTIIVLIYLI
NLNYFIKLTKYIKIIIYIFYLRYYFSILGFQFIIYIILCITLHLALANLLFPLVWNNDLLIHWFFESKK
EIRNIFLLLLILFLKNIHFALPFSCIKMNDIKNKYLNYSYVILSYIATIIRSCINTCTCLISCSLCYSC
KKNYGYAKKIRSCCRLLWTIASICCLKTFIKRICSSYTIYCSFLFRSCNNFNFCIIRLRCYTLWSW
FRDKRHEFRYILHVSCVIFSYIRYSISWLKCEIRFSRFSKYSSINLINIKFSYINSNYDNRKFKFNCL
YISKSYLIYTTFISCVYNIFHRIYSDKSSFFSRGPANLVWSGYMSQIARKPFYFKNKRQLAGNLIP
NKYIIKLFIDTQFNIYIYLFIYMIKDIVNHRCESTKKKHMGTHLPLLGESELVSGFMTEHAAVVF
VFFFLAEYGSIVLMCILTSILFIGGYLLFEISYVFTVVNYIFFELFFIDVTFVEVQSLYTDFLNNSIIE
GLLYGFNLGLKSSLMIFTFIARASFPRIRFDQLMGFCTVLLPINFAIIILVPCVLYSFNLLPVNIPLF
LTHPPALLPQGYSTYEEGNRSRTILVNNYTYTKTIYHTRECNYTLFLYSTLLAYYYLYMIIILLNL
LGYINKKFVNFFHHFLIQNTNF
>YN427
ITILKNIKSYIKIIHLYSINLKGIFSILYLVLLLFSLLQVFLLLGSLLNRWYARLLIKVISFYLLQYLV
LEGFIIILKKCSFIHLTINTKKLKIILLMVKLLIETIKYMLNLIIQMLCLVLWQLFLLLHFYYSCLIK
NDVWVLYWYNLYLAINEYDIIYRVCICYSYFIFSHFYICSSSLSRKIYFRVWFPFFRAKNTIRYKI
LYFCFSLFTFRFRNIINFPFRSVCWYLWSFSYFNFYSYNNYRIYIIRKRSNRQQTKIIYTIERLPYR
VCWNRGFVKLRQKTKKPTKGLMGSYKKMITYIYSYYIFTITRIIIYIYIILLYIKIIIIIRYNYSILLY
KNIIYPIINFIFFLIIIFFYIMLTHLQMKRTKYCCYSSGYRKIFLYYLFLLGSFSVRGYMDGRRVFIL
MNSGFKLTLFMNLNYNYINVGYDRIMYSLRVKRDTPTYNTYWFRVEYIYLRVKNIYIQYIINIVI
YWYGLNQVVYILSFDKHLFFYLLLLLYCEINNYNIILHSTYLGILSYLAINDSNQILFSLVWNNDL
LIHWFFDSKKRVNNFLDQKYYFLQKAYAFHFFDKQLLLIFGCLLVLTITKFFQFLYKFKEYKVIH
YNILTLSMRHNQVTLVTCEILVHYLFVYKLLPVLHLCIIVLVWKLLTQSIEMLITGDFVIYIVIQL
LLSFSCIYTEEVYITDHIELLVLFELLVLLYYWLSVSVMFYLMDRCHYEVLQLLLILLVLYLEGKI
LLNSFEVVFLLIMPLTDFLHYILYCLLYLLFCTLHFMILLVQAILLVFQVITIELHLLHIFYLKILLF
LYLFLYVLLYSLCLMFGIVIIILWLILCKLLLLLYLNDTYYLSMLFDLYLINYVLRCLVLFLLCYY
LLQIVDLEVYNLDHLVKLSEFLLLISFCNVLNTLKIHLYYVNVLYYTLVILLLYYLLVTIIVLIYLI
NLNYFIKLTKYIKIIIYIFYLRYYFSILGFQFIIYIILCITLHLALANLLFPLVWNNDLLIHWFFESKK
EIRNIFLLLLILFLKNIHFALPFSCIKMNDIKNKYLNYSYVILSYIATIIRSCINTCTCLISCSLCYSC
KKNYGYAKKIRSCCRLLWTIASICCLKTFIKRICSSYTIYCSFLFRSCNNFNFCIIRLRCYTLWSW
FRDKRHEFRYILHVSCVIFSYIRYSISWLKCEIRFSRFSKYSSINLINIKFSYINSNYDNRKFKFNCL
YISKSYLIYTTFISCVYNIFHRIYSDKSSFFSRGPANLVWSGYMSQIARKPFYFKNKRQLAGNLIP
NKYIIKLFIDTQFNIYIYLFIYMIKDIVNHRCESTKKKHMGTHLPLLGESELVSGFMTEHAAVVF
VFFFLAEYGSIVLMCILTSILFIGGYLLFEISYVFTVVNYIFFELFFIDVTFVEVQSLYTDFLNNSIIE
GLLYGFNLGLKSSLMIFTFIARASFPRIRFDQLMGFCTVLLPINFAIIILVPCVLYSFNLLPVNIPLF
LTHPPALLPQGYSTYEEGNRSRTILVNNYTYTKTIYHTRECNYTLFLYSTLLAYYYLYMIIILLNL
LGYINKKFVNFFHHFLIQNTNF
>YN435
ITILKNIKSYIKIIHLYSINLKGIFSILYLVLLLFSLLQVFLLLGSLLNRWYARLLIKVISFYLLQYLV

LEGFIIILKKCSFIHLTINTKKLKIILLMVKLLIETIKYMLNLIIQMLCLVLWQLFLLLHFYYSCLIK
NDVWVLYWYNLYLAINEYDIIYRVCICYSYFIFSHFYICSSSLSRKIYFRVWFPFFRAKNTIRYKI
LYFCFSLFTFRFRNIINFPFRSVCWYLWSFSYFNFYSYNNYRIYIIRKRSNRQQTKIIYTIERLPYR
VCWNRGFVKLRQKTKKPTKGLMGSYKKMITYIYSYYIFTITRIIIYIYIILLYIKIIIIIRYNYSILLY
KNIIYPIINFIFFLIIIFFYIMLTHLQMKRTKYCCYSSGYRKIFLYYLFLLGSFSVRGYMDGRRVFIL
MNSGFKLTLFMNLNYNYINVGYDRIMYSLRVKRDTPTYNTYWFRVEYIYLRVKNIYIQYIINIVI
YWYGLNQVYILSFDKHLFFYLLLLLYCEINNYNIILHSTYLGILSYLAINDSNQILFSLVWNNDL
LIHWFFDSKKRVNNFLDQKYYFLQKAYAFHFFDKQLLLIFGCLLVLTITKFFQFLYKFKEYKVIH
YNILTLSMRHNQVTLVTCEILVHYLFVYKLLPVLHLCIIVLVWKLLTQSIEMLITGDFVIYIVIQL
LLSFSCIYTEEVYITDHIELLVLFELLVLLYYWLSVSVMFYLMDRCHYEVLQLLLILLVLYLEGKI
LLNSFEVVFLLIMPLTDFLHYILYCLLYLLFCTLHFMILLVQAILLVFQVITIELHLLHIFYLKILLF
LYLFLYVLLYSLCLMFGIVIIILWLILCKLLLLLYLNDTYYLSMLFDLYLINYVLRCLVLFLLCYY
LLQIVDLEVYNLDHLVKLSEFLLLISFCNVLNTLKIHLYYVNVLYYTLVILLLYYLLVTIIVLIYLI
NLNYFIKLTKYIKIIIYIFYLRYYFSILGFQFIIYIILCITLHLALANLLFPLVWNNDLLIHWFFESKK
EIRNIFLLLLILFLKNIHFALPFSCIKMNDIKNKYLNYSYVILSYIATIIRSCINTCTCLISCSLCYSC
KKNYGYAKKIRSCCRLLWTIASICCLKTFIKRICSSYTIYCSFLFRSCNNFNFCIIRLRCYTLWSW
FRDKRHEFRYILHVSCVIFSYIRYSISWLKCEIRFSRFSKYSSINLINIKFSYINSNYDNRKFKFNCL
YISKSYLIYTTFISCVYNIFHRIYSDKSSFFSRGPANLVWSGYMSQIARKPFYFKNKRQLAGNLIP
NKYIIKLFIDTQFNIYIYLFIYMIKDIVNHRCESTKKKHMGTHLPLLGESELVSGFMTEHAAVVF
VFFFLAEYGSIVLMCILTSILFIGGYLLFEISYVFTVVNYIFFELFFIDVTFVEVQSLYTDFLNNSIIE
GLLYGFNLGLKSSLMIFTFIARASFPRIRFDQLMGFCTVLLPINFAIIILVPCVLYSFNLLPVNIPLF
LTHPPALLPQGYSTYEEGNRSRTILVNNYTYTKTIYHTRECNYTLFLYSTLLAYYYLYMIIILLNL
LGYINKKFVNFFHHFLIQNTNF
>YN442
ITILKNIKSYIKIIHLYSINLKGIFSILYLVLLLFSLLQVFLLLGSLLNRWYARLLIKVISFYLLQYLV
LEGFIIILKKCSFIHLTINTKKLKIILLMVKLLIETIKYMLNLIIQMLCLVLWQLFLLLHFYYSCLIK
NDVWVLYWYNLYLAINEYDIIYRVCICYSYFIFSHFYICSSSLSRKIYFRVWFPFFRAKNTIRYKI
LYFCFSLFTFRFRNIINFPFRSVCWYLWSFSYFNFYSYNNYRIYIIRKRSNRQQTKIIYTIERLPYR
VCWNRGFVKLRQKTKKPTKGLMGSYKKMITYIYSYYIFTITRIIIYIYIILLYIKIIIIIRYNYSILLY
KNIIYPIINFIFFLIIIFFYIMLTHLQMKRTKYCCYSSGYRKIFLYYLFLLGSFSVRGYMDGRRVFIL
MNSGFKLTLFMNLNYNYINVGYDRIMYSLRVKRDTPTYNTYWFRVEYIYLRVKNIYIQYIINIVI
YWYGLNQVYILSFDKHLFFYLLLLLYCEINNYNIILHSTYLGILSYLAINDSNQILFSLVWNNDL
LIHWFFDSKKRVNNFLDQKYYFLQKAYAFHFFDKQLLLIFGCLLVLTITKFFQFLYKFKEYKVIH
YNILTLSMRHNQVTLVTCEILVHYLFVYKLLPVLHLCIIVLVWKLLTQSIEMLITGDFVIYIVIQL
LLSFSCIYTEEVYITDHIELLVLFELLVLLYYWLSVSVMFYLMDRCHYEVLQLLLILLVLYLEGKI
LLNSFEVVFLLIMPLTDFLHYILYCLLYLLFCTLHFMILLVQAILLVFQVITIELHLLHIFYLKILLF
LYLFLYVLLYSLCLMFGIVIIILWLILCKLLLLLYLNDTYYLSMLFDLYLINYVLRCLVLFLLCYY
LLQIVDLEVYNLDHLVKLSEFLLLISFCNVLNTLKIHLYYVNVLYYTLVILLLYYLLVTIIVLIYLI
NLNYFIKLTKYIKIIIYIFYLRYYFSILGFQFIIYIILCITLHLALANLLFPLVWNNDLLIHWFFESKK
EIRNIFLLLLILFLKNIHFALPFSCIKMNDIKNKYLNYSYVILSYIATIIRSCINTCTCLISCSLCYSC
KKNYGYAKKIRSCCRLLWTIASICCLKTFIKRICSSYTIYCSFLFRSCNNFNFCIIRLRCYTLWSW
FRDKRHEFRYILHVSCVIFSYIRYSISWLKCEIRFSRFSKYSSINLINIKFSYINSNYDNRKFKFNCL
YISKSYLIYTTFISCVYNIFHRIYSDKSSFFSRGPANLVWSGYMSQIARKPFYFKNKRQLAGNLIP
NKYIIKLFIDTQFNIYIYLFIYMIKDIVNHRCESTKKKHMGTHLPLLGESELVSGFMTEHAAVVF

VFFFLAEYGSIVLMCILTSILFIGGYLLFEISYVFTVVNYIFFELFFIDVTFVEVQSLYTDFLNNSIIE
GLLYGFNLGLKSSLMIFTFIARASFPRIRFDQLMGFCTVLLPINFAIIILVPCVLYSFNLLPVNIPLF
LTHPPALLPQGYSTYEEGNRSRTILVNNYTYTKTIYHTRECNYTLFLYSTLLAYYYLYMIIILLNL
LGYINKKFVNFFHHFLIQNTNF
>YN455
ITILKNIKSYIKIIHLYSINLKGIFSILYLVLLLFSLLQVFLLLGSLLNRWYARLLIKVISFYLLQYLV
LEGFIIILKKCSFIHLTINTKKLKIILLMVKLLIETIKYMLNLIIQMLCLVLWQLFLLLHFYYSCLIK
NDVWVLYWYNLYLAINEYDIIYRVCICYSYFIFSHFYICSSSLSRKIYFRVWFPFFRAKNTIRYKI
LYFCFSLFTFRFRNIINFPFRSVCWYLWSFSYFNFYSYNNYRIYIIRKRSNRQQTKIIYTIERLPYR
VCWNRGFVKLRQKTKKPTKGLMGSYKKMITYIYSYYIFTITRIIIYIYIILLYIKIIIIIRYNYSILLY
KNIIYPIINFIFFLIIIFFYIMLTHLQMKRTKYCCYSSGYRKIFLYYLFLLGSFSVRGYMDGRRVFIL
MNSGFKLTLFMNLNYNYINVGYDRIMYSLRVKRDTPTYNTYWFRVEYIYLRVKNIYIQYIINIVI
YWYGLNQVYILSFDKHLFFYLLLLLYCEINNYNIILHSTYLGILSYLAINDSNQILFSLVWNNDL
LIHWFFDSKKRVNNFLDQKYYFLQKAYAFHFFDKQLLLIFGCLLVLTITKFFQFLYKFKEYKVIH
YNILTLSMRHNQVTLVTCEILVHYLFVYKLLPVLHLCIIVLVWKLLTQSIEMLITGDFVIYIVIQL
LLSFSCIYTEEVYITDHIELLVLFELLVLLYYWLSVSVMFYLMDRCHYEVLQLLLILLVLYLEGKI
LLNSFEVVFLLIMPLTDFLHYILYCLLYLLFCTLHFMILLVQAILLVFQVITIELHLLHIFYLKILLF
LYLFLYVLLYSLCLMFGIVIIILWLILCKLLLLLYLNDTYYLSMLFDLYLINYVLRCLVLFLLCYY
LLQIVDLEVYNLDHLVKLSEFLLLISFCNVLNTLKIHLYYVNVLYYTLVILLLYYLLVTIIVLIYLI
NLNYFIKLTKYIKIIIYIFYLRYYFSILGFQFIIYIILCITLHLALANLLFPLVWNNDLLIHWFFESKK
EIRNIFLLLLILFLKNIHFALPFSCIKMNDIKNKYLNYSYVILSYIATIIRSCINTCTCLISCSLCYSC
KKNYGYAKKIRSCCRLLWTIASICCLKTFIKRICSSYTIYCSFLFRSCNNFNFCIIRLRCYTLWSW
FRDKRHEFRYILHVSCVIFSYIRYSISWLKCEIRFSRFSKYSSINLINIKFSYINSNYDNRKFKFNCL
YISKSYLIYTTFISCVYNIFHRIYSDKSSFFSRGPANLVWSGYMSQIARKPFYFKNKRQLAGNLIP
NKYIIKLFIDTQFNIYIYLFIYMIKDIVNHRCESTKKKHMGTHLPLLGESELVSGFMTEHAAVVF
VFFFLAEYGSIVLMCILTSILFIGGYLLFEISYVFTVVNYIFFELFFIDVTFVEVQSLYTDFLNNSIIE
GLLYGFNLGLKSSLMIFTFIARASFPRIRFDQLMGFCTVLLPINFAIIILVPCVLYSFNLLPVNIPLF
LTHPPALLPQGYSTYEEGNRSRTILVNNYTYTKTIYHTRECNYTLFLYSTLLAYYYLYMIIILLNL
LGYINKKFVNFFHHFLIQNTNF
>YN460
ITILKNIKSYIKIIHLYSINLKGIFSILYLVLLLFSLLQVFLLLGSLLNRWYARLLIKVISFYLLQYLV
LEGFIIILKKCSFIHLTINTKKLKIILLMVKLLIETIKYMLNLIIQMLCLVLWQLFLLLHFYYSCLIK
NDVWVLYWYNLYLAINEYDIIYRVCICYSYFIFSHFYICSSSLSRKIYFRVWFPFFRAKNTIRYKI
LYFCFSLFTFRFRNIINFPFRSVCWYLWSFSYFNFYSYNNYRIYIIRKRSNRQQTKIIYTIERLPYR
VCWNRGFVKLRQKTKKPTKGLMGSYKKMITYIYSYYIFTITRIIIYIYIILLYIKIIIIIRYNYSILLY
KNIIYPIINFIFFLIIIFFYIMLTHLQMKRTKYCCYSSGYRKIFLYYLFLLGSFSVRGYMDGRRVFIL
MNSGFKLTLFMNLNYNYINVGYDRIMYSLRVKRDTPTYNTYWFRVEYIYLRVKNIYIQYIINIVI
YWYGLNQVYILSFDKHLFFYLLLLLYCEINNYNIILHSTYLGILSYLAINDSNQILFSLVWNNDL
LIHWFFDSKKRVNNFLDQKYYFLQKAYAFHFFDKQLLLIFGCLLVLTITKFFQFLYKFKEYKVIH
YNILTLSMRHNQVTLVTCEILVHYLFVYKLLPVLHLCIIVLVWKLLTQSIEMLITGDFVIYIVIQL
LLSFSCIYTEEVYITDHIELLVLFELLVLLYYWLSVSVMFYLMDRCHYEVLQLLLILLVLYLEGKI
LLNSFEVVFLLIMPLTDFLHYILYCLLYLLFCTLHFMILLVQAILLVFQVITIELHLLHIFYLKILLF
LYLFLYVLLYSLCLMFGIVIIILWLILCKLLLLLYLNDTYYLSMLFDLYLINYVLRCLVLFLLCYY
LLQIVDLEVYNLDHLVKLSEFLLLISFCNVLNTLKIHLYYVNVLYYTLVILLLYYLLVTIIVLIYLI

NLNYFIKLTKYIKIIIYIFYLRYYFSILGFQFIIYIILCITLHLALANLLFPLVWNNDLLIHWFFESKK
EIRNIFLLLLILFLKNIHFALPFSCIKMNDIKNKYLNYSYVILSYIATIIRSCINTCTCLISCSLCYSC
KKNYGYAKKIRSCCRLLWTIASICCLKTFIKRICSSYTIYCSFLFRSCNNFNFCIIRLRCYTLWSW
FRDKRHEFRYILHVSCVIFSYIRYSISWLKCEIRFSRFSKYSSINLINIKFSYINSNYDNRKFKFNCL
YISKSYLIYTTFISCVYNIFHRIYSDKSSFFSRGPANLVWSGYMSQIARKPFYFKNKRQLAGNLIP
NKYIIKLFIDTQFNIYIYLFIYMIKDIVNHRCESTKKKHMGTHLPLLGESELVSGFMTEHAAVVF
VFFFLAEYGSIVLMCILTSILFIGGYLLFEISYVFTVVNYIFFELFFIDVTFVEVQSLYTDFLNNSIIE
GLLYGFNLGLKSSLMIFTFIARASFPRIRFDQLMGFCTVLLPINFAIIILVPCVLYSFNLLPVNIPLF
LTHPPALLPQGYSTYEEGNRSRTILVNNYTYTKTIYHTRECNYTLFLYSTLLAYYYLYMIIILLNL
LGYINKKFVNFFHHFLIQNTNF
>YN462
ITILKNIKSYIKIIHLYSINLKGIFSILYLVLLLFSLLQVFLLLGSLLNRWYARLLIKVISFYLLQYLV
LEGFIIILKKCSFIHLTINTKKLKIILLMVKLLIETIKYMLNLIIQMLCLVLWQLFLLLHFYYSCLIK
NDVWVLYWYNLYLAINEYDIIYRVCICYSYFIFSHFYICSSSLSRKIYFRVWFPFFRAKNTIRYKI
LYFCFSLFTFRFRNIINFPFRSVCWYLWSFSYFNFYSYNNYRIYIIRKRSNRQQTKIIYTIERLPYR
VCWNRGFVKLRQKTKKPTKGLMGSYKKMITYIYSYYIFTITRIIIYIYIILLYIKIIIIIRYNYSILLY
KNIIYPIINFIFFLIIIFFYIMLTHLQMKRTKYCCYSSGYRKIFLYYLFLLGSFSVRGYMDGRRVFIL
MNSGFKLTLFMNLNYNYINVGYDRIMYSLRVKRDTPTYNTYWFRVEYIYLRVKNIYIQYIINIVI
YWYGLNQVYILSFDKHLFFYLLLLLYCEINNYNIILHSTYLGILSYLAINDSNQILFSLVWNNDL
LIHWFFDSKKRVNNFLDQKYYFLQKAYAFHFFDKQLLLIFGCLLVLTITKFFQFLYKFKEYKVIH
YNILTLSMRHNQVTLVTCEILVHYLFVYKLLPVLHLCIIVLVWKLLTQSIEMLITGDFVIYIVIQL
LLSFSCIYTEEVYITDHIELLVLFELLVLLYYWLSVSVMFYLMDRCHYEVLQLLLILLVLYLEGKI
LLNSFEVVFLLIMPLTDFLHYILYCLLYLLFCTLHFMILLVQAILLVFQVITIELHLLHIFYLKILLF
LYLFLYVLLYSLCLMFGIVIIILWLILCKLLLLLYLNDTYYLSMLFDLYLINYVLRCLVLFLLCYY
LLQIVDLEVYNLDHLVKLSEFLLLISFCNVLNTLKIHLYYVNVLYYTLVILLLYYLLVTIIVLIYLI
NLNYFIKLTKYIKIIIYIFYLRYYFSILGFQFIIYIILCITLHLALANLLFPLVWNNDLLIHWFFESKK
EIRNIFLLLLILFLKNIHFALPFSCIKMNDIKNKYLNYSYVILSYIATIIRSCINTCTCLISCSLCYSC
KKNYGYAKKIRSCCRLLWTIASICCLKTFIKRICSSYTIYCSFLFRSCNNFNFCIIRLRCYTLWSW
FRDKRHEFRYILHVSCVIFSYIRYSISWLKCEIRFSRFSKYSSINLINIKFSYINSNYDNRKFKFNCL
YISKSYLIYTTFISCVYNIFHRIYSDKSSFFSRGPANLVWSGYMSQIARKPFYFKNKRQLAGNLIP
NKYIIKLFIDTQFNIYIYLFIYMIKDIVNHRCESTKKKHMGTHLPLLGESELVSGFMTEHAAVVF
VFFFLAEYGSIVLMCILTSILFIGGYLLFEISYVFTVVNYIFFELFFIDVTFVEVQSLYTDFLNNSIIE
GLLYGFNLGLKSSLMIFTFIARASFPRIRFDQLMGFCTVLLPINFAIIILVPCVLYSFNLLPVNIPLF
LTHPPALLPQGYSTYEEGNRSRTILVNNYTYTKTIYHTRECNYTLFLYSTLLAYYYLYMIIILLNL
LGYINKKFVNFFHHFLIQNTNF
>YN463
ITILKNIKSYIKIIHLYSINLKGIFSILYLVLLLFSLLQVFLLLGSLLNRWYARLLIKVISFYLLQYLV
LEGFIIILKKCSFIHLTINTKKLKIILLMVKLLIETIKYMLNLIIQMLCLVLWQLFLLLHFYYSCLIK
NDVWVLYWYNLYLAINEYDIIYRVCICYSYFIFSHFYICSSSLSRKIYFRVWFPFFRAKNTIRYKI
LYFCFSLFTFRFRNIINFPFRSVCWYLWSFSYFNFYSYNNYRIYIIRKRSNRQQTKIIYTIERLPYR
VCWNRGFVKLRQKTKKPTKGLMGSYKKMITYIYSYYIFTITRIIIYIYIILLYIKIIIIIRYNYSILLY
KNIIYPIINFIFFLIIIFFYIMLTHLQMKRTKYCCYSSGYRKIFLYYLFLLGSFSVRGYMDGRRVFIL
MNSGFKLTLFMNLNYNYINVGYDRIMYSLRVKRDTPTYNTYWFRVEYIYLRVKNIYIQYIINIVI
YWYGLNQVYILSFDKHLFFYLLLLLYCEINNYNIILHSTYLGILSYLAINDSNQILFSLVWNNDL

LIHWFFDSKKRVNNFLDQKYYFLQKAYAFHFFDKQLLLIFGCLLVLTITKFFQFLYKFKEYKVIH
YNILTLSMRHNQVTLVTCEILVHYLFVYKLLPVLHLCIIVLVWKLLTQSIEMLITGDFVIYIVIQL
LLSFSCIYTEEVYITDHIELLVLFELLVLLYYWLSVSVMFYLMDRCHYEVLQLLLILLVLYLEGKI
LLNSFEVVFLLIMPLTDFLHYILYCLLYLLFCTLHFMILLVQAILLVFQVITIELHLLHIFYLKILLF
LYLFLYVLLYSLCLMFGIVIIILWLILCKLLLLLYLNDTYYLSMLFDLYLINYVLRCLVLFLLCYY
LLQIVDLEVYNLDHLVKLSEFLLLISFCNVLNTLKIHLYYVNVLYYTLVILLLYYLLVTIIVLIYLI
NLNYFIKLTKYIKIIIYIFYLRYYFSILGFQFIIYIILCITLHLALANLLFPLVWNNDLLIHWFFESKK
EIRNIFLLLLILFLKNIHFALPFSCIKMNDIKNKYLNYSYVILSYIATIIRSCINTCTCLISCSLCYSC
KKNYGYAKKIRSCCRLLWTIASICCLKTFIKRICSSYTIYCSFLFRSCNNFNFCIIRLRCYTLWSW
FRDKRHEFRYILHVSCVIFSYIRYSISWLKCEIRFSRFSKYSSINLINIKFSYINSNYDNRKFKFNCL
YISKSYLIYTTFISCVYNIFHRIYSDKSSFFSRGPANLVWSGYMSQIARKPFYFKNKRQLAGNLIP
NKYIIKLFIDTQFNIYIYLFIYMIKDIVNHRCESTKKKHMGTHLPLLGESELVSGFMTEHAAVVF
VFFFLAEYGSIVLMCILTSILFIGGYLLFEISYVFTVVNYIFFELFFIDVTFVEVQSLYTDFLNNSIIE
GLLYGFNLGLKSSLMIFTFIARASFPRIRFDQLMGFCTVLLPINFAIIILVPCVLYSFNLLPVNIPLF
LTHPPALLPQGYSTYEEGNRSRTILVNNYTYTKTIYHTRECNYTLFLYSTLLAYYYLYMIIILLNL
LGYINKKFVNFFHHFLIQNTNF
>YN468
ITILKNIKSYIKIIHLYSINLKGIFSILYLVLLLFSLLQVFLLLGSLLNRWYARLLIKVISFYLLQYLV
LEGFIIILKKCSFIHLTINTKKLKIILLMVKLLIETIKYMLNLIIQMLCLVLWQLFLLLHFYYSCLIK
NDVWVLYWYNLYLAINEYDIIYRVCICYSYFIFSHFYICSSSLSRKIYFRVWFPFFRAKNTIRYKI
LYFCFSLFTFRFRNIINFPFRSVCWYLWSFSYFNFYSYNNYRIYIIRKRSNRQQTKIIYTIERLPYR
VCWNRGFVKLRQKTKKPTKGLMGSYKKMITYIYSYYIFTITRIIIYIYIILLYIKIIIIIRYNYSILLY
KNIIYPIINFIFFLIIIFFYIMLTHLQMKRTKYCCYSSGYRKIFLYYLFLLGSFSVRGYMDGRRVFIL
MNSGFKLTLFMNLNYNYINVGYDRIMYSLRVKRDTPTYNTYWFRVEYIYLRVKNIYIQYIINIVI
YWYGLNQVYILSFDKHLFFYLLLLLYCEINNYNIILHSTYLGILSYLAINDSNQILFSLVWNNDL
LIHWFFDSKKRVNNFLDQKYYFLQKAYAFHFFDKQLLLIFGCLLVLTITKFFQFLYKFKEYKVIH
YNILTLSMRHNQVTLVTCEILVHYLFVYKLLPVLHLCIIVLVWKLLTQSIEMLITGDFVIYIVIQL
LLSFSCIYTEEVYITDHIELLVLFELLVLLYYWLSVSVMFYLMDRCHYEVLQLLLILLVLYLEGKI
LLNSFEVVFLLIMPLTDFLHYILYCLLYLLFCTLHFMILLVQAILLVFQVITIELHLLHIFYLKILLF
LYLFLYVLLYSLCLMFGIVIIILWLILCKLLLLLYLNDTYYLSMLFDLYLINYVLRCLVLFLLCYY
LLQIVDLEVYNLDHLVKLSEFLLLISFCNVLNTLKIHLYYVNVLYYTLVILLLYYLLVTIIVLIYLI
NLNYFIKLTKYIKIIIYIFYLRYYFSILGFQFIIYIILCITLHLALANLLFPLVWNNDLLIHWFFESKK
EIRNIFLLLLILFLKNIHFALPFSCIKMNDIKNKYLNYSYVILSYIATIIRSCINTCTCLISCSLCYSC
KKNYGYAKKIRSCCRLLWTIASICCLKTFIKRICSSYTIYCSFLFRSCNNFNFCIIRLRCYTLWSW
FRDKRHEFRYILHVSCVIFSYIRYSISWLKCEIRFSRFSKYSSINLINIKFSYINSNYDNRKFKFNCL
YISKSYLIYTTFISCVYNIFHRIYSDKSSFFSRGPANLVWSGYMSQIARKPFYFKNKRQLAGNLIP
NKYIIKLFIDTQFNIYIYLFIYMIKDIVNHRCESTKKKHMGTHLPLLGESELVSGFMTEHAAVVF
VFFFLAEYGSIVLMCILTSILFIGGYLLFEISYVFTVVNYIFFELFFIDVTFVEVQSLYTDFLNNSIIE
GLLYGFNLGLKSSLMIFTFIARASFPRIRFDQLMGFCTVLLPINFAIIILVPCVLYSFNLLPVNIPLF
LTHPPALLPQGYSTYEEGNRSRTILVNNYTYTKTIYHTRECNYTLFLYSTLLAYYYLYMIIILLNL
LGYINKKFVNFFHHFLIQNTNF
>YN476
ITILKNIKSYIKIIHLYSINLKGIFSILYLVLLLFSLLQVFLLLGSLLNRWYARLLIKVISFYLLQYLV
LEGFIIILKKCSFIHLTINTKKLKIILLMVKLLIETIKYMLNLIIQMLCLVLWQLFLLLHFYYSCLIK

NDVWVLYWYNLYLAINEYDIIYRVCICYSYFIFSHFYICSSSLSRKIYFRVWFPFFRAKNTIRYKI
LYFCFSLFTFRFRNIINFPFRSVCWYLWSFSYFNFYSYNNYRIYIIRKRSNRQQTKIIYTIERLPYR
VCWNRGFVKLRQKTKKPTKGLMGSYKKMITYIYSYYIFTITRIIIYIYIILLYIKIIIIIRYNYSILLY
KNIIYPIINFIFFLIIIFFYIMLTHLQMKRTKYCCYSSGYRKIFLYYLFLLGSFSVRGYMDGRRVFIL
MNSGFKLTLFMNLNYNYINVGYDRIMYSLRVKRDTPTYNTYWFRVEYIYLRVKNIYIQYIINIVI
YWYGLNQVYILSFDKHLFFYLLLLLYCEINNYNIILHSTYLGILSYLAINDSNQILFSLVWNNDL
LIHWFFDSKKRVNNFLDQKYYFLQKAYAFHFFDKQLLLIFGCLLVLTITKFFQFLYKFKEYKVIH
YNILTLSMRHNQVTLVTCEILVHYLFVYKLLPVLHLCIIVLVWKLLTQSIEMLITGDFVIYIVIQL
LLSFSCIYTEEVYITDHIELLVLFELLVLLYYWLSVSVMFYLMDRCHYEVLQLLLILLVLYLEGKI
LLNSFEVVFLLIMPLTDFLHYILYCLLYLLFCTLHFMILLVQAILLVFQVITIELHLLHIFYLKILLF
LYLFLYVLLYSLCLMFGIVIIILWLILCKLLLLLYLNDTYYLSMLFDLYLINYVLRCLVLFLLCYY
LLQIVDLEVYNLDHLVKLSEFLLLISFCNVLNTLKIHLYYVNVLYYTLVILLLYYLLVTIIVLIYLI
NLNYFIKLTKYIKIIIYIFYLRYYFSILGFQFIIYIILCITLHLALANLLFPLVWNNDLLIHWFFESKK
EIRNIFLLLLILFLKNIHFALPFSCIKMNDIKNKYLNYSYVILSYIATIIRSCINTCTCLISCSLCYSC
KKNYGYAKKIRSCCRLLWTIASICCLKTFIKRICSSYTIYCSFLFRSCNNFNFCIIRLRCYTLWSW
FRDKRHEFRYILHVSCVIFSYIRYSISWLKCEIRFSRFSKYSSINLINIKFSYINSNYDNRKFKFNCL
YISKSYLIYTTFISCVYNIFHRIYSDKSSFFSRGPANLVWSGYMSQIARKPFYFKNKRQLAGNLIP
NKYIIKLFIDTQFNIYIYLFIYMIKDIVNHRCESTKKKHMGTHLPLLGESELVSGFMTEHAAVVF
VFFFLAEYGSIVLMCILTSILFIGGYLLFEISYVFTVVNYIFFELFFIDVTFVEVQSLYTDFLNNSIIE
GLLYGFNLGLKSSLMIFTFIARASFPRIRFDQLMGFCTVLLPINFAIIILVPCVLYSFNLLPVNIPLF
LTHPPALLPQGYSTYEEGNRSRTILVNNYTYTKTIYHTRECNYTLFLYSTLLGYYYLYMIIILLNL
LGYINKKFVNFFHHFLIQNTNF

>YN477

ITILKNIKSYIKIIHLYSINLKGIFSILYLVLLLFSLLQVFLLLGSLLNRWYARLLIKVISFYLLQYLV
LEGFIIILKKCSFIHLTINTKKLKIILLMVKLLIETIKYMLNLIIQMLCLVLWQLFLLLHFYYSCLIK
NDVWVLYWYNLYLAINEYDIIYRVCICYSYFIFSHFYICSSSLSRKIYFRVWFPFFRAKNTIRYKI
LYFCFSLFTFRFRNIINFPFRSVCWYLWSFSYFNFYSYNNYRIYIIRKRSNRQQTKIIYTIERLPYR
VCWNRGFVKLRQKTKKPTKGLMGSYKKMITYIYSYYIFTITRIIIYIYIILLYIKIIIIIRYNYSILLY
KNIIYPIINFIFFLIIIFFYIMLTHLQMKRTKYCCYSSGYRKIFLYYLFLLGSFSVRGYMDGRRVFIL
MNSGFKLTLFMNLNYNYINVGYDRIMYSLRVKRDTPTYNTYWFRVEYIYLRVKNIYIQYIINIVI
YWYGLNQVYILSFDKHLFFYLLLLLYCEINNYNIILHSTYLGILSYLAINDSNQILFSLVWNNDL
LIHWFFDSKKRVNNFLDQKYYFLQKAYAFHFFDKQLLLIFGCLLVLTITKFFQFLYKFKEYKVIH
YNILTLSMRHNQVTLVTCEILVHYLFVYKLLPVLHLCIIVLVWKLLTQSIEMLITGDFVIYIVIQL
LLSFSCIYTEEVYITDHIELLVLFELLVLLYYWLSVSVMFYLMDRCHYEVLQLLLILLVLYLEGKI
LLNSFEVVFLLIMPLTDFLHYILYCLLYLLFCTLHFMILLVQAILLVFQVITIELHLLHIFYLKILLF
LYLFLYVLLYSLCLMFGIVIIILWLILCKLLLLLYLNDTYYLSMLFDLYLINYVLRCLVLFLLCYY
LLQIVDLEVYNLDHLVKLSEFLLLISFCNVLNTLKIHLYYVNVLYYTLVILLLYYLLVTIIVLIYLI
NLNYFIKLTKYIKIIIYIFYLRYYFSILGFQFIIYIILCITLHLALANLLFPLVWNNDLLIHWFFESKK
EIRNIFLLLLILFLKNIHFALPFSCIKMNDIKNKYLNYSYVILSYIATIIRSCINTCTCLISCSLCYSC
KKNYGYAKKIRSCCRLLWTIASICCLKTFIKRICSSYTIYCSFLFRSCNNFNFCIIRLRCYTLWSW
FRDKRHEFRYILHVSCVIFSYIRYSISWLKCEIRFSRFSKYSSINLINIKFSYINSNYDNRKFKFNCL
YISKSYLIYTTFISCVYNIFHRIYSDKSSFFSRGPANLVWSGYMSQIARKPFYFKNKRQLAGNLIP
NKYIIKLFIDTQFNIYIYLFIYMIKDIVNHRCESTKKKHMGTHLPLLGESELVSGFMTEHAAVVF
VFFFLAEYGSIVLMCILTSILFIGGYLLFEISYVFTVVNYIFFELFFIDVTFVEVQSLYTDFLNNSIIE

GLLYGFNLGLKSSLMIFTFIARASFPRIRFDQLMGFCTVLLPINFAIIILVPCVLYSFNLLPVNIPLF
LTHPPALLPQGYSTYEEGNRSRTILVNNYTYTKTIYHTRECNYTLFLYSTLLGYYYLYMIIILLNL
LGYINKKFVNFFHHFLIQNTNF
>YN480
ITILKNIKSYIKIIHLYSINLKGIFSILYLVLLLFSLLQVFLLLGSLLNRWYARLLIKVISFYLLQYLV
LEGFIIILKKCSFIHLTINTKKLKIILLMVKLLIETIKYMLNLIIQMLCLVLWQLFLLLHFYYSCLIK
NDVWVLYWYNLYLAINEYDIIYRVCICYSYFIFSHFYICSSSLSRKIYFRVWFPFFRAKNTIRYKI
LYFCFSLFTFRFRNIINFPFRSVCWYLWSFSYFNFYSYNNYRIYIIRKRSNRQQTKIIYTIERLPYR
VCWNRGFVKLRQKTKKPTKGLMGSYKKMITYIYSYYIFTITRIIIYIYIILLYIKIIIIIRYNYSILLY
KNIIYPIINFIFFLIIIFFYIMLTHLQMKRTKYCCYSSGYRKIFLYYLFLLGSFSVRGYMDGRRVFIL
MNSGFKLTLFMNLNYNYINVGYDRIMYSLRVKRDTPTYNTYWFRVEYIYLRVKNIYIQYIINIVI
YWYGLNQVVYILSFDKHLFFYLLLLLYCEINNYNIILHSTYLGILSYLAINDSNQILFSLVWNNDL
LIHWFFDSKKRVNNFLDQKYYFLQKAYAFHFFDKQLLLIFGCLLVLTITKFFQFLYKFKEYKVIH
YNILTLSMRHNQVTLVTCEILVHYLFVYKLLPVLHLCIIVLVWKLLTQSIEMLITGDFVIYIVIQL
LLSFSCIYTEEVYITDHIELLVLFELLVLLYYWLSVSVMFYLMDRCHYEVLQLLLILLVLYLEGKI
LLNSFEVVFLLIMPLTDFLHYILYCLLYLLFCTLHFMILLVQAILLVFQVITIELHLLHIFYLKILLF
LYLFLYVLLYSLCLMFGIVIIILWLILCKLLLLLYLNDTYYLSMLFDLYLINYVLRCLVLFLLCYY
LLQIVDLEVYNLDHLVKLSEFLLLISFCNVLNTLKIHLYYVNVLYYTLVILLLYYLLVTIIVLIYLI
NLNYFIKLTKYIKIIIYIFYLRYYFSILGFQFIIYIILCITLHLALANLLFPLVWNNDLLIHWFFESKK
EIRNIFLLLLILFLKNIHFALPFSCIKMNDIKNKYLNYSYVILSYIATIIRSCINTCTCLISCSLCYSC
KKNYGYAKKIRSCCRLLWTIASICCLKTFIKRICSSYTIYCSFLFRSCNNFNFCIIRLRCYTLWSW
FRDKRHEFRYILHVSCVIFSYIRYSISWLKCEIRFSRFSKYSSINLINIKFSYINSNYDNRKFKFNCL
YISKSYLIYTTFISCVYNIFHRIYSDKSSFFSRGPANLVWSGYMSQIARKPFYFKNKRQLAGNLIP
NKYIIKLFIDTQFNIYIYLFIYMIKDIVNHRCESTKKKHMGTHLPLLGESELVSGFMTEHAAVVF
VFFFLAEYGSIVLMCILTSILFIGGYLLFEISYVFTVVNYIFFELFFIDVTFVEVQSLYTDFLNNSIIE
GLLYGFNLGLKSSLMIFTFIARASFPRIRFDQLMGFCTVLLPINFAIIILVPCVLYSFNLLPVNIPLF
LTHPPALLPQGYSTYEEGNRSRTILVNNYTYTKTIYHTRECNYTLFLYSTLLAYYYLYMIIILLNL
LGYINKKFVNFFHHFLIQNTNF
>YN485
ITILKNIKSYIKIIHLYSINLKGIFSILYLVLLLFSLLQVFLLLGSLLNRWYARLLIKVISFYLLQYLV
LEGFIIILKKCSFIHLTINTKKLKIILLMVKLLIETIKYMLNLIIQMLCLVLWQLFLLLHFYYSCLIK
NDVWVLYWYNLYLAINEYDIIYRVCICYSYFIFSHFYICSSSLSRKIYFRVWFPFFRAKNTIRYKI
LYFCFSLFTFRFRNIINFPFRSVCWYLWSFSYFNFYSYNNYRIYIIRKRSNRQQTKIIYTIERLPYR
VCWNRGFVKLRQKTKKPTKGLMGSYKKMITYIYSYYIFTITRIIINIYIILLYIKIIIIIRYNYSILLY
KNIIYPIINFIFFLIIIFFYIMLTHLQMKRTKYCCYSSGYRKIFLYYLFLLGSFSVRGYMDGRRVFIL
MNSGFKLTLFMNLNYNYINVGYDRIMYSLRVKRDTPTYNTYWFRVEYIYLRVKNIYIQYIINIVI
YWYGLNQVVYILSFDKHLFFYLLLLLYCEINNYNIILHSTYLGILSYLAINDSNQILFSLVWNNDL
LIHWFFDSKKRVNNFLDQKYYFLQKAYAFHFFDKQLLLIFGCLLVLTITKFFQFLYKFKEYKVIH
YNILTLSMRHNQVTLVTCEILVHYLFVYKLLPVLHLCIIVLVWKLLTQSIEMLITGDFVIYIVIQL
LLSFSCIYTEEVYITDHIELLVLFELLVLLYYWLSVSVMFYLMDRCHYEVLQLLLILLVLYLEGKI
LLNSFEVVFLLIMPLTDFLHYILYCLLYLLFCTLHFMILLVQAILLVFQVITIELHLLHIFYLKILLF
LYLFLYVLLYSLCLMFGIVIIILWLILCKLLLLLYLNDTYYLSMLFDLYLINYVLRCLVLFLLCYY
LLQIVDLEVYNLDHLVKLSEFLLLISFCNVLNTLKIHLYYVNVLYYTLVILLLYYLLVTIIVLIYLI
NLNYFIKLTKYIKIIIYIFYLRYYFSILGFQFIIYIILCITLHLALANLLFPLVWNNDLLIHWFFESKK

EIRNIFLLLLILFLKNIHFALPFSCIKMNDIKNKYLNYSYVILSYIATIIRSCINTCTCLISCSLCYSC
KKNYGYAKKIRSCCRLLWTIASICCLKTFIKRICSSYTIYCSFLFRSCNNFNFCIIRLRCYTLWSW
FRDKRHEFRYILHVSCVIFSYIRYSISWLKCEIRFSRFSKYSSINLINIKFSYINSNYDNRKFKFNCL
YISKSYLIYTTFISCVYNIFHRIYSDKSSFFSRGPANLVWSGYMSQIARKPFYFKNKRQLAGNLIP
NKYIIKLFIDTQFNIYIYLFIYMIKDIVNHRCESTKKKHMGTHLPLLGESELVSGFMTEHAAVVF
VFFFLAEYGSIVLMCILTSILFIGGYLLFEISYVFTVVNYIFFELFFIDVTFVEVQSLYTDFLNNSIIE
GLLYGFNLGLKSSLMIFTFIARASFPRIRFDQLMGFCTVLLPINFAIIILVPCVLYSFNLLPVNIPLF
LTHPPALLPQGYSTYEEGNRSRTILVNNYTYTKTIYHTRECNYTLFLYSTLLAYYYLYMIIILLNL
LGYINKKFVNFFHHFLIQNTNF
>YN487
ITILKNIKSYIKIIHLYSINLKGIFSILYLVLLLFSLLQVFLLLGSLLNRWYARLLIKVISFYLLQYLV
LEGFIIILKKCSFIHLTINTKKLKIILLMVKLLIETIKYMLNLIIQMLCLVLWQLFLLLHFYYSCLIK
NDVWVLYWYNLYLAINEYDIIYRVCICYSYFIFSHFYICSSSLSRKIYFRVWFPFFRAKNTIRYKI
LYFCFSLFTFRFRNIINFPFRSVCWYLWSFSYFNFYSYNNYRIYIIRKRSNRQQTKIIYTIERLPYR
VCWNRGFVKLRQKTKKPTKGLMGSYKKMITYIYSYYIFTITRIIIYIYIILLYIKIIIIIRYNYSILLY
KNIIYPIINFIFFLIIIFFYIMLTHLQMKRTKYCCYSSGYRKIFLYYLFLLGSFSVRGYMDGRRVFIL
MNSGFKLTLFMNLNYNYINVGYDRIMYSLRVKRDTPTYNTYWFRVEYIYLRVKNIYIQYIINIVI
YWYGLNQVVYILSFDKHLFFYLLLLLYCEINNYNIILHSTYLGILSYLAINDSNQILFSLVWNNDL
LIHWFFDSKKRVNNFLDQKYYFLQKAYAFHFFDKQLLLIFGCLLVLTITKFFQFLYKFKEYKVIH
YNILTLSMRHNQVTLVTCEILVHYLFVYKLLPVLHLCIIVLVWKLLTQSIEMLITGDFVIYIVIQL
LLSFSCIYTEEVYITDHIELLVLFELLVLLYYWLSVSVMFYLMDRCHYEVLQLLLILLVLYLEGKI
LLNSFEVVFLLIMPLTDFLHYILYCLLYLLFCTLHFMILLVQAILLVFQVITIELHLLHIFYLKILLF
LYLFLYVLLYSLCLMFGIVIIILWLILCKLLLLLYLNDTYYLSMLFDLYLINYVLRCLVLFLLCYY
LLQIVDLEVYNLDHLVKLSEFLLLISFCNVLNTLKIHLYYVNVLYYTLVILLLYYLLVTIIVLIYLI
NLNYFIKLTKYIKIIIYIFYLRYYFSILGFQFIIYIILCITLHLALANLLFPLVWNNDLLIHWFFESKK
EIRNIFLLLLILFLKNIHFALPFSCIKMNDIKNKYLNYSYVILSYIATIIRSCINTCTCLISCSLCYSC
KKNYGYAKKIRSCCRLLWTIASICCLKTFIKRICSSYTIYCSFLFRSCNNFNFCIIRLRCYTLWSW
FRDKRHEFRYILHVSCVIFSYIRYSISWLKCEIRFSRFSKYSSINLINIKFSYINSNYDNRKFKFNCL
YISKSYLIYTTFISCVYNIFHRIYSDKSSFFSRGPANLVWSGYMSQIARKPFYFKNKRQLAGNLIP
NKYIIKLFIDTQFNIYIYLFIYMIKDIVNHRCESTKKKHMGTHLPLLGESELVSGFMTEHAAVVF
VFFFLAEYGSIVLMCILTSILFIGGYLLFEISYVFTVVNYIFFELFFIDVTFVEVQSLYTDFLNNSIIE
GLLYGFNLGLKSSLMIFTFIARASFPRIRFDQLMGFCTVLLPINFAIIILVPCVLYSFNLLPVNIPLF
LTHPPALLPQGYSTYEEGNRSRTILVNNYTYTKTIYHTRECNYTLFLYSTLLAYYYLYMIIILLNL
LGYINKKFVNFFHHFLIQNTNF
>YN540
ITILKNIKSYIKIIHLYSINLKGIFSILYLVLLLFSLLQVFLLLGSLLNRWYARLLIKVISFYLLQYLV
LEGFIIILKKCSFIHLTINTKKLKIILLMVKLLIETIKYMLNLIIQMLCLVLWQLFLLLHFYYSCLIK
NDVWVLYWYNLYLAINEYDIIYRVCICYSYFIFSHFYICSSSLSRKIYFRVWFPFFRAKNTIRYKI
LYFCFSLFTFRFRNIINFPFRSVCWYLWSFSYFNFYSYNNYRIYIIRKRSNRQQTKIIYTIERLPYR
VCWNRGFVKLRQKTKKPTKGLMGSYKKMITYIYSYYIFTITRIIIYIYIILLYIKIIIIIRYNYSILLY
KNIIYPIINFIFFLIIIFFYIMLTHLQMKRTKYCCYSSGYRKIFLYYLFLLGSFSVRGYMDGRRVFIL
MNSGFKLTLFMNLNYNYINVGYDRIMYSLRVKRDTPTYNTYWFRVEYIYLRVKNIYIQYIINIVI
YWYGLNQVVYILSFDKHLFFYLLLLLYCEINNYNIILHSTYLGILSYLAINDSNQILFSLVWNNDL
LIHWFFDSKKRVNNFLDQKYYFLQKAYAFHFFDKQLLLIFGCLLVLTITKFFQFLYKFKEYKVIH

YNILTLSMRHNQVTLVTCEILVHYLFVYKLLPVLHLCIIVLVWKLLTQSIEMLITGDFVIYIVIQL
LLSFSCIYTEEVYITDHIELLVLFELLVLLYYWLSVSVMFYLMDRCHYEVLQLLLILLVLYLEGKI
LLNSFEVVFLLIMPLTDFLHYILYCLLYLLFCTLHFMILLVQAILLVFQVITIELHLLHIFYLKILLF
LYLFLYVLLYSLCLMFGIVIIILWLILCKLLLLLYLNDTYYLSMLFDLYLINYVLRCLVLFLLCYY
LLQIVDLEVYNLDHLVKLSEFLLLISFCNVLNTLKIHLYYVNVLYYTLVILLLYYLLVTIIVLIYLI
NLNYFIKLTKYIKIIIYIFYLRYYFSILGFQFIIYIILCITLHLALANLLFPLVWNNDLLIHWFFESKK
EIRNIFLLLLILFLKNIHFALPFSCIKMNDIKNKYLNYSYVILSYIATIIRSCINTCTCLISCSLCYSC
KKNYGYAKKIRSCCRLLWTIASICCLKTFIKRICSSYTIYCSFLFRSCNNFNFCIIRLRCYTLWSW
FRDKRHEFRYILHVSCVIFSYIRYSISWLKCEIRFSRFSKYSSINLINIKFSYINSNYDNRKFKFNCL
YISKSYLIYTTFISCVYNIFHRIYSDKSSFFSRGPANLVWSGYMSQIARKPFYFKNKRQLAGNLIP
NKYIIKLFIDTQFNIYIYLFIYMIKDIVNHRCESTKKKHMGTHLPLLGESELVSGFMTEHAAVVF
VFFFLAEYGSIVLMCILTSILFIGGYLLFEISYVFTVVNYIFFELFFIDVTFVEVQSLYTDFLNNSIIE
GLLYGFNLGLKSSLMIFTFIARASFPRIRFDQLMGFCTVLLPINFAIIILVPCVLYSFNLLPVNIPLF
LTHPPALLPQGYSTYEEGNRSRTILVNNYTYTKTIYHTRECNYTLFLYSTLLAYYYLYMIIILLNL
LGYINKKFVNFFHHFLIQNTNN

>YN490

ITILKNIKSYIKIIHLYSINLKGIFSILYLVLLLFSLLQVFLLLGSLLNRWYARLLIKVISFYLLQYLV
LEGFIIILKKCSFIHLTINTKKLKIILLMVKLLIETIKYMLNLIIQMLCLVLWQLFLLLHFYYSCLIK
NDVWVLYWYNLYLAINEYDIIYRVCICYSYFIFSHFYICSSSLSRKIYFRVWFPFFRAKNTIRYKI
LYFCFSLFTFRFRNIINFPFRSVCWYLWSFSYFNFYSYNNYRIYIIRKRSNRQQTKIIYTIERLPYR
VCWNRGFVKLRQKTKKPTKGLMGSYKKMITYIYSYYIFTITRIIIYIYIILLYIKIIIIIRYNYSILLY
KNIIYPIINFIFFLIIIFFYIMLTHLQMKRTKYCCYSSGYRKIFLYYLFLLGSFSVRGYMDGRRVFIL
MNSGFKLTLFMNLNYNYINVGYDRIMYSLRVKRDTPTYNTYWFRVEYIYLRVKNIYIQYIINIVI
YWYGLNQVYILSFDKHLFFYLLLLLYCEINNYNIILHSTYLGILSYLAINDSNQILFSLVWNNDL
LIHWFFDSKKRVNNFLDQKYYFLQKAYAFHFFDKQLLLIFGCLLVLTITKFFQFLYKFKEYKVIH
YNILTLSMRHNQVTLVTCEILVHYLFVYKLLPVLHLCIIVLVWKLLTQSIEMLITGDFVIYIVIQL
LLSFSCIYTEEVYITDHIELLVLFELLVLLYYWLSVSVMFYLMDRCHYEVLQLLLILLVLYLEGKI
LLNSFEVVFLLIMPLTDFLHYILYCLLYLLFCTLHFMILLVQAILLVFQVITIELHLLHIFYLKILLF
LYLFLYVLLYSLCLMFGIVIIILWLILCKLLLLLYLNDTYYLSMLFDLYLINYVLRCLVLFLLCYY
LLQIVDLEVYNLDHLVKLSEFLLLISFCNVLNTLKIHLYYVNVLYYTLVILLLYYLLVTIIVLIYLI
NLNYFIKLTKYIKIIIYIFYLRYYFSILGFQFIIYIILCITLHLALANLLFPLVWNNDLLIHWFFESKK
EIRNIFLLLLILFLKNIHFALPFSCIKMNDIKNKYLNYSYVILSYIATIIRSCINTCTCLISCSLCYSC
KKNYGYAKKIRSCCRLLWTIASICCLKTFIKRICSSYTIYCSFLFRSCNNFNFCIIRLRCYTLWSW
FRDKRHEFRYILHVSCVIFSYIRYSISWLKCEIRFSRFSKYSSINLINIKFSYINSNYDNRKFKFNCL
YISKSYLIYTTFISCVYNIFHRIYSDKSSFFSRGPANLVWSGYMSQIARKPFYFKNKRQLAGNLIP
NKYIIKLFIDTQFNIYIYLFIYMIKDIVNHRCESTKKKHMGTHLPLLGESELVSGFMTEHAAVVF
VFFFLAEYGSIVLMCILTSILFIGGYLLFEISYVFTVVNYIFFELFFIDVTFVEVQSLYTDFLNNSIIE
GLLYGFNLGLKSSLMIFTFIARASFPRIRFDQLMGFCTVLLPINFAIIILVPCVLYSFNLLPVNIPLF
LTHPPALLPQGYSTYEEGNRSRTILVNNYTYTKTIYHTRECNYTLFLYSTLLAYYYLYMIIILLNL
LGYINKKFVNFFHHFLIQNTNF

>YN541

ITILKNIKSYIKIIHLYSINLKGIFSILYLVLLLFSLLQVFLLLGSLLNRWYARLLIKVISFYLLQYLV
LEGFIIILKKCSFIHLTINTKKLKIILLMVKLLIETIKYMLNLIIQMLCLVLWQLFLLLHFYYSCLIK
NDVWVLYWYNLYLAINEYDIIYRVCICYSYFIFSHFYICSSSLSRKIYFRVWFPFFRAKNTIRYKI

LYFCFSLFTFRFRNIINFPPFRSVCWYLWSFSYFNFYSYNNYRIYIIRKRSNRQQTKIIYTIERLPYR
VCWNRGFVKLRQKTKKPTKGLMGSYKKMITYIYSYYIFTITRIIIYIYIILLYIKIIIIIRYNYSILLY
KNIIYPIINFIFFLIIIFFYIMLTHLQMKRTKYCCYSSGYRKIFLYYLFLLGSFSVRGYMDGRRVFIL
MNSGFKLTLFMNLNYNYINVGYDRIMYSLRVKRDTPTYNTYWFRVEYIYLRVKNIYIQYIINIVI
YWYGLNQVVYILSFDKHLFFYLLLLLYCEINNYNIILHSTYLGILSYLAINDSNQILFSLVWNNDL
LIHWFFDSKKRVNNFLDQKYYFLQKAYAFHFFDKQLLLIFGCLLVLTITKFFQFLYKFKEYKVIH
YNILTLSMRHNQVTLVTCEILVHYLFVYKLLPVLHLCIIVLVWKLLTQSIEMLITGDFVIYIVIQL
LLSFSCIYTEEVYITDHIELLVLFELLVLLYYWLSVSVMFYLMDRCHYEVLQLLLILLVLYLEGKI
LLNSFEVVFLLIMPLTDFLHYILYCLLYLLFCTLHFMILLVQAILLVFQVITIELHLLHIFYLKILLF
LYLFLYVLLYSLCLMFGIVIIILWLILCKLLLLLYLNDTYYLSMLFDLYLINYVLRCLVLFLLCYY
LLQIVDLEVYNLDHLVKLSEFLLLISFCNVLNTLKIHLYYVNVLYYTLVILLLYYLLVTIIVLIYLI
NLNYFIKLTKYIKIIIYIFYLRYYFSILGFQFIIYIILCITLHLALANLLFPLVWNNDLLIHWFFESKK
EIRNIFLLLLILFLKNIHFALPFSCIKMNDIKNKYLNYSYVILSYIATIIRSCINTCTCLISCSLCYSC
KKNYGYAKKIRSCCRLLWTIASICCLKTFIKRICSSYTIYCSFLFRSCNNFNFCIIRLRCYTLWSW
FRDKRHEFRYILHVSCVIFSYIRYSISWLKCEIRFSRFSKYSSINLINIKFSYINSNYDNRKFKFNCL
YISKSYLIYTTFISCVYNIFHRIYSDKSSFFSRGPANLVWSGYMSQIARKPFYFKNKRQLAGNLIP
NKYIIKLFIDTQFNIYIYLFIYMIKDIVNHRCESTKKKHMGTHLPLLGESELVSGFMTEHAAVVF
VFFFLAEYGSIVLMCILTSILFIGGYLLFEISYVFTVVNYIFFELFFIDVTFVEVQSLYTDFLNNSIIE
GLLYGFNLGLKSSLMIFTFIARASFPRIRFDQLMGFCTVLLPINFAIIILVPCVLYSFNLLPVNIPLF
LTHPPALLPQGYSTYEEGNRSRTILVNNYTYTKTIYHTRECNYTLFLYSTLLAYYYLYMIIILLNL
LGYINKKFVNFFHHFLIQNTNF
>YN544
ITILKNIKSYIKIIHLYSINLKGIFSILYLVLLLFSLLQVFLLLGSLLNRWYARLLIKVISFYLLQYLV
LEGFIIILKKCSFIHLTINTKKLKIILLMVKLLIETIKYMLNLIIQMLCLVLWQLFLLLHFYYSCLIK
NDVWVLYWYNLYLAINEYDIIYRVCICYSYFIFSHFYICSSSLSRKIYFRVWFPFFRAKNTIRYKI
LYFCFSLFTFRFRNIINFPPFRSVCWYLWSFSYFNFYSYNNYRIYIIRKRSNRQQTKIIYTIERLPYR
VCWNRGFVKLRQKTKKPTKGLMGSYKKMITYIYSYYIFTITRIIIYIYIILLYIKIIIIIRYNYSILLY
KNIIYPIINFIFFLIIIFFYIMLTHLQMKRTKYCCYSSGYRKIFLYYLFLLGSFSVRGYMDGRRVFIL
MNSGFKLTLFMNLNYNYINVGYDRIMYSLRVKRDTPTYNTYWFRVEYIYLRVKNIYIQYIINIVI
YWYGLNQVVYILSFDKHLFFYLLLLLYCEINNYNIILHSTYLGILSYLAINDSNQILFSLVWNNDL
LIHWFFDSKKRVNNFLDQKYYFLQKAYAFHFFDKQLLLIFGCLLVLTITKFFQFLYKFKEYKVIH
YNILTLSMRHNQVTLVTCEILVHYLFVYKLLPVLHLCIIVLVWKLLTQSIEMLITGDFVIYIVIQL
LLSFSCIYTEEVYITDHIELLVLFELLVLLYYWLSVSVMFYLMDRCHYEVLQLLLILLVLYLEGKI
LLNSFEVVFLLIMPLTDFLHYILYCLLYLLFCTLHFMILLVQAILLVFQVITIELHLLHIFYLKILLF
LYLFLYVLLYSLCLMFGIVIIILWLILCKLLLLLYLNDTYYLSMLFDLYLINYVLRCLVLFLLCYY
LLQIVDLEVYNLDHLVKLSEFLLLISFCNVLNTLKIHLYYVNVLYYTLVILLLYYLLVTIIVLIYLI
NLNYFIKLTKYIKIIIYIFYLRYYFSILGFQFIIYIILCITLHLALANLLFPLVWNNDLLIHWFFESKK
EIRNIFLLLLILFLKNIHFALPFSCIKMNDIKNKYLNYSYVILSYIATIIRSCINTCTCLISCSLCYSC
KKNYGYAKKIRSCCRLLWTIASICCLKTFIKRICSSYTIYCSFLFRSCNNFNFCIIRLRCYTLWSW
FRDKRHEFRYILHVSCVIFSYIRYSISWLKCEIRFSRFSKYSSINLINIKFSYINSNYDNRKFKFNCL
YISKSYLIYTTFISCVYNIFHRIYSDKSSFFSRGPANLVWSGYMSQIARKPFYFKNKRQLAGNLIP
NKYIIKLFIDTQFNIYIYLFIYMIKDIVNHRCESTKKKHMGTHLPLLGESELVSGFMTEHAAVVF
VFFFLAEYGSIVLMCILTSILFIGGYLLFEISYVFTVVNYIFFELFFIDVTFVEVQSLYTDFLNNSIIE
GLLYGFNLGLKSSLMIFTFIARASFPRIRFDQLMGFCTVLLPINFAIIILVPCVLYSFNLLPVNIPLF

LTHPPALLPQGYSTYEEGNRSRTILVNNYTYTKTIYHTRECNYTLFLYSTLLAYYYLYMIIILLNL
LGYINKKFVNFFHHFLIQNTNF
>YN554
ITILKNIKSYIKIIHLYSINLKGIFSILYLVLLLFSLLQVFLLLGSLLNRWYARLLIKVISFYLLQYLV
LEGFIIILKKCSFIHLTINTKKLKIILLMVKLLIETIKYMLNLIIQMLCLVLWQLFLLLHFYYSCLIK
NDVWVLYWYNLYLAINEYDIIYRVCICYSYFIFSHFYICSSSLSRKIYFRVWFPFFRAKNTIRYKI
LYFCFSLFTFRFRNIINFPFRSVCWYLWSFSYFNFYSYNNYRIYIIRKRSNRQQTKIIYTIERLPYR
VCWNRGFVKLRQKTKKPTKGLMGSYKKMITYIYSYYIFTITRIIIYIYIILLYIKIIIIIRYNYSILLY
KNIIYPIINFIFFLIIIFFYIMLTHLQMKRTKYCCYSSGYRKIFLYYLFLLGSFSVRGYMDGRRVFIL
MNSGFKLTLFMNLNYNYINVGYDRIMYSLRVKRDTPTYNTYWFRVEYIYLRVKNIYIQYIINIVI
YWYGLNQVYILSFDKHLFFYLLLLLYCEINNYNIILHSTYLGILSYLAINDSNQILFSLVWNNDL
LIHWFFDSKKRVNNFLDQKYYFLQKAYAFHFFDKQLLLIFGCLLVLTITKFFQFLYKFKEYKVIH
YNILTLSMRHNQVTLVTCEILVHYLFVYKLLPVLHLCIIVLVWKLLTQSIEMLITGDFVIYIVIQL
LLSFSCIYTEEVYITDHIELLVLFELLVLLYYWLSVSVMFYLMDRCHYEVLQLLLILLVLYLEGKI
LLNSFEVVFLLIMPLTDFLHYILYCLLYLLFCTLHFMILLVQAILLVFQVITIELHLLHIFYLKILLF
LYLFLYVLLYSLCLMFGIVIIILWLILCKLLLLLYLNDTYYLSMLFDLYLINYVLRCLVLFLLCYY
LLQIVDLEVYNLDHLVKLSEFLLLISFCNVLNTLKIHLYYVNVLYYTLVILLLYYLLVTIIVLIYLI
NLNYFIKLTKYIKIIIYIFYLRYYFSILGFQFIIYIILCITLHLALANLLFPLVWNNDLLIHWFFDSKK
EIRNIFLLLLILFLKNIHFALPFSCIKMNDIKNKYLNYSYVILSYIATIIRSCINTCTCLISCSLCYSC
KKNYGYAKKIRSCCRLLWTIASICCLKTFIKRICSSYTIYCSFLFRSCNNFNFCIIRLRCYTLWSW
FRDKRHEFRYILHVSCVIFSYIRYSISWLKCEIRFSRFSKYSSINLINIKFSYINSNYDNRKFKFNCL
YISKSYLIYTTFISCVYNIFHRIYSDKSSFFSRGPANLVWSGYMSQIARKPFYFKNKRQLAGNLIP
NKYIIKLFIDTQFNIYIYLFIYMIKDIVNHRCESTKKKHMGTHLPLLGESELVSGFMTEHAAVVF
VFFFLAEYGSIVLMCILTSILFIGGYLLFEISYVFTVVNYIFFELFFIDVTFVEVQSLYTDFLNNSIIE
GLLYGFNLGLKSSLMIFTFIARASFPRIRFDQLMGFCTVLLPINFAIIILVPCVLYSFNLLPVNIPLF
LTHPPALLPQGYSTYEEGNRSRTILVNNYTYTKTIYHTRECNYTLFLYSTLLAYYYLYMIIILLNL
LGYINKKFVNFFHHFLIQNTNF
>YN557
ITILKNIKSYIKIIHLYSINLKGIFSILYLVLLLFSLLQVFLLLGSLLNRWYARLLIKVISFYLLQYLV
LEGFIIILKKCSFIHLTINTKKLKIILLMVKLLIETIKYMLNLIIQMLCLVLWQLFLLLHFYYSCLIK
NDVWVLYWYNLYLAINEYDIIYRVCICYSYFIFSHFYICSSSLSRKIYFRVWFPFFRAKNTIRYKI
LYFCFSLFTFRFRNIINFPFRSVCWYLWSFSYFNFYSYNNYRIYIIRKRSNRQQTKIIYTIERLPYR
VCWNRGFVKLRQKTKKPTKGLMGSYKKMITYIYSYYIFTITRIIIYIYIILLYIKIIIIIRYNYSILLY
KNIIYPIINFIFFLIIIFFYIMLTHLQMKRTKYCCYSSGYRKIFLYYLFLLGSFSVRGYMDGRRVFIL
MNSGFKLTLFMNLNYNYINVGYDRIMYSLRVKRDTPTYNTYWFRVEYIYLRVKNIYIQYIINIVI
YWYGLNQVYILSFDKHLFFYLLLLLYCEINNYNIILHSTYLGILSYLAINDSNQILFSLVWNNDL
LIHWFFDSKKRVNNFLDQKYYFLQKAYAFHFFDKQLLLIFGCLLVLTITKFFQFLYKFKEYKVIH
YNILTLSMRHNQVTLVTCEILVHYLFVYKLLPVLHLCIIVLVWKLLTQSIEMLITGDFVIYIVIQL
LLSFSCIYTEEVYITDHIELLVLFELLVLLYYWLSVSVMFYLMDRCHYEVLQLLLILLVLYLEGKI
LLNSFEVVFLLIMPLTDFLHYILYCLLYLLFCTLHFMILLVQAILLVFQVITIELHLLHIFYLKILLF
LYLFLYVLLYSLCLMFGIVIIILWLILCKLLLLLYLNDTYYLSMLFDLYLINYVLRCLVLFLLCYY
LLQIVDLEVYNLDHLVKLSEFLLLISFCNVLNTLKIHLYYVNVLYYTLVILLLYYLLVTIIVLIYLI
NLNYFIKLTKYIKIIIYIFYLRYYFSILGFQFIIYIILCITLHLALANLLFPLVWNNDLLIHWFFESKK
EIRNIFLLLLILFLKNIHFALPFSCIKMNDIKNKYLNYSYVILSYIATIIRSCINTCTCLISCSLCYSC

KKNYGYAKKIRSCCRLLWTIASICCLKTFIKRICSSYTIYCSFLFRSCNNFNFCIIRLRCYTLWSW
FRDKRHEFRYILHVSCVIFSYIRYSISWLKCEIRFSRFSKYSSINLINIKFSYINSNYDNRKFKFNCL
YISKSYLIYTTFISCVYNIFHRIYSDKSSFFSRGPANLVWSGYMSQIARKPFYFKNKRQLAGNLIP
NKYIIKLFIDTQFNIYIYLFIYMIKDIVNHRCESTKKKHMGTHLPLLGESELVSGFMTEHAAVVF
VFFFLAEYGSIVLMCILTSILFIGGYLLFEISYVFTVVNYIFFELFFIDVTFVEVQSLYTDFLNNSIIE
GLLYGFNLGLKSSLMIFTFIARASFPRIRFDQLMGFCTVLLPINFAIIILVPCVLYSFNLLPVNIPLF
LTHPPALLPQGYSTYEEGNRSRTILVNNYTYTKTIYHTRECNYTLFLYSTLLAYYYLYMIIILLNL
LGYINKKFVNFFHHFLIQNTNF
>YN561
ITILKNIKSYIKIIHLYSINLKGIFSILYLVLLLFSLLQVFLLLGSLLNRWYARLLIKVISFYLLQYLV
LEGFIIILKKCSFIHLTINTKKLKIILLMVKLLIETIKYMLNLIIQMLCLVLWQLFLLLHFYYSCLIK
NDVWVLYWYNLYLAINEYDIIYRVCICYSYFIFSHFYICSSSLSRKIYFRVWFPFFRAKNTIRYKI
LYFCFSLFTFRFRNIINFPFRSVCWYLWSFSYFNFYSYNNYRIYIIRKRSNRQQTKIIYTIERLPYR
VCWNRGFVKLRQKTKKPTKGLMGSYKKMITYIYSYYIFTITRIIIYIYIILLYIKIIIIIRYNYSILLY
KNIIYPIINFIFFLIIIFFYIMLTHLQMKRTKYCCYSSGYRKIFLYYLFLLGSFSVRGYMDGRRVFIL
MNSGFKLTLFMNLNYNYINVGYDRIMYSLRVKRDTPTYNTYWFRVEYIYLRVKNIYIQYIINIVI
YWYGLNQVYILSFDKHLFFYLLLLLYCEINNYNIILHSTYLGILSYLAINDSNQILFSLVWNNDL
LIHWFFDSKKRVNNFLDQKYYFLQKAYAFHFFDKQLLLIFGCLLVLTITKFFQFLYKFKEYKVIH
YNILTLSMRHNQVTLVTCEILVHYLFVYKLLPVLHLCIIVLVWKLLTQSIEMLITGDFVIYIVIQL
LLSFSCIYTEEVYITDHIELLVLFELLVLLYYWLSVSVMFYLMDRCHYEVLQLLLILLVLYLEGKI
LLNSFEVVFLLIMPLTDFLHYILYCLLYLLFCTLHFMILLVQAILLVFQVITIELHLLHIFYLKILLF
LYLFLYVLLYSLCLMFGIVIIILWLILCKLLLLLYLNDTYYLSMLFDLYLINYVLRCLVLFLLCYY
LLQIVDLEVYNLDHLVKLSEFLLLISFCNVLNTLKIHLYYVNVLYYTLVILLLYYLLVTIIVLIYLI
NLNYFIKLTKYIKIIIYIFYLRYYFSILGFQFIIYIILCITLHLALANLLFPLVWNNDLLIHWFFESKK
EIRNIFLLLLILFLKNIHFALPFSCIKMNDIKNKYLNYSYVILSYIATIIRSCINTCTCLISCSLCYSC
KKNYGYAKKIRSCCRLLWTIASICCLKTFIKRICSSYTIYCSFLFRSCNNFNFCIIRLRCYTLWSW
FRDKRHEFRYILHVSCVIFSYIRYSISWLKCEIRFSRFSKYSSINLINIKFSYINSNYDNRKFKFNCL
YISKSYLIYTTFISCVYNIFHRIYSDKSSFFSRGPANLVWSGYMSQIARKPFYFKNKRQLAGNLIP
NKYIIKLFIDTQFNIYIYLFIYMIKDIVNHRCESTKKKHMGTHLPLLGESELVSGFMTEHAAVVF
VFFFLAEYGSIVLMCILTSILFIGGYLLFEISYVFTVVNYIFFELFFIDVTFVEVQSLYTDFLNNSIIE
GLLYGFNLGLKSSLMIFTFIARASFPRIRFDQLMGFCTVLLPINFAIIILVPCVLYSFNLLPVNIPLF
LTHPPALLPQGYSTYEEGNRSRTILVNNYTYTKTIYHTRECNYTLFLYSTLLAYYYLYMIIILLNL
LGYINKKFVNFFHHFLIQNTNY
>YN567
ITILKNIKSYIKIIHLYSINLKGIFSILYLVLLLFSLLQVFLLLGSLLNRWYARLLIKVISFYLLQYLV
LEGFIIILKKCSFIHLTINTKKLKIILLMVKLLIETIKYMLNLIIQMLCLVLWQLFLLLHFYYSCLIK
NDVWVLYWYNLYLAINEYDIIYRVCICYSYFIFSHFYICSSSLSRKIYFRVWFPFFRAKNTIRYKI
LYFCFSLFTFRFRNIINFPFRSVCWYLWSFSYFNFYSYNNYRIYIIRKRSNRQQTKIIYTIERLPYR
VCWNRGFVKLRQKTKKPTKGLMGSYKKMITYIYSYYIFTITRIIIYIYIILLYIKIIIIIRYNYSILLY
KNIIYPIINFIFFLIIIFFYIMLTHLQMKRTKYCCYSSGYRKIFLYYLFLLGSFSVRGYMDGRRVFIL
MNSGFKLTLFMNLNYNYINVGYDRIMYSLRVKRDTPTYNTYWFRVEYIYLRVKNIYIQYIINIVI
YWYGLNQVYILSFDKHLFFYLLLLLYCEINNYNIILHSTYLGILSYLAINDSNQILFSLVWNNDL
LIHWFFDSKKRVNNFLDQKYYFLQKAYAFHFFDKQLLLIFGCLLVLTITKFFQFLYKFKEYKVIH
YNILTLSMRHNQVTLVTCEILVHYLFVYKLLPVLHLCIIVLVWKLLTQSIEMLITGDFVIYIVIQL

LLSFSCIYTEEVYITDHIELLVLFELLVLLYYWLSVSVMFYLMDRCHYEVLQLLLILLVLYLEGKI
LLNSFEVVFLLIMPLTDFLHYILYCLLYLLFCTLHFMILLVQAILLVFQVITIELHLLHIFYLKILLF
LYLFLYVLLYSLCLMFGIVIIILWLILCKLLLLLYLNDTYYLSMLFDLYLINYVLRCLVLFLLCYY
LLQIVDLEVYNLDHLVKLSEFLLLISFCNVLNTLKIHLYYVNVLYYTLVILLLYYLLVTIIVLIYLI
NLNYFIKLTKYIKIIIYIFYLRYYFSILGFQFIIYIILCITLHLALANLLFPLVWNNDLLIHWFFESKK
EIRNIFLLLLILFLKNIHFALPFSCIKMNDIKNKYLNYSYVILSYIATIIRSCINTCTCLISCSLCYSC
KKNYGYAKKIRSCCRLLWTIASICCLKTFIKRICSSYTIYCSFLFRSCNNFNFCIIRLRCYTLWSW
FRDKRHEFRYILHVSCVIFSYIRYSISWLKCEIRFSRFSKYSSINLINIKFSYINSNYDNRKFKFNCL
YISKSYLIYTTFISCVYNIFHRIYSDKSSFFSRGPANLVWSGYMSQIARKPFYFKNKRQLAGNLIP
NKYIIKLFIDTQFNIYIYLFIYMIKDIVNHRCESTKKKHMGTHLPLLGESELVSGFMTEHAAVVF
VFFFLAEYGSIVLMCILTSILFIGGYLLFEISYVFTVVNYIFFELFFIDVTFVEVQSLYTDFLNNSIIE
GLLYGFNLGLKSSLMIFTFIARASFPRIRFDQLMGFCTVLLPINFAIIILVPCVLYSFNLLPVNIPLF
LTHPPALLPQGYSTYEEGNRSRTILVNNYTYTKTIYHTRECNYTLFLYSTLLAYYYLYMIIILLNL
LGYINKKFVNFFHHFLIQNTNF
>YN604
ITILKNIKSYIKIIHLYSINLKGIFSILYLVLLLFSLLQVFLLLGSLLNRWYARLLIKVISFYLLQYLV
LEGFIIILKKCSFIHLTINTKKLKIILLMVKLLIETIKYMLNLIIQMLCLVLWQLFLLLHFYYSCLIK
NDVWVLYWYNLYLAINEYDIIYRVCICYSYFIFSHFYICSSSLSRKIYFRVWFPFFRAKNTIRYKI
LYFCFSLFTFRFRNIINFPFRSVCWYLWSFSYFNFYSYNNYRIYIIRKRSNRQQTKIIYTIERLPYR
VCWNRGFVKLRQKTKKPTKGLMGSYKKMITYIYSYYIFTITRIIIYIYIILLYIKIIIIIRYNYSILLY
KNIIYPIINFIFFLIIIFFYIMLTHLQMKRTKYCCYSSGYRKIFLYYLFLLGSFSVRGYMDGRRVFIL
MNSGFKLTLFMNLNYNYINVGYDRIMYSLRVKRDTPTYNTYWFRVEYIYLRVKNIYIQYIINIVI
YWYGLNQVYILSFDKHLFFYLLLLLYCEINNYNIILHSTYLGILSYLAINDSNQILFSLVWNNDL
LIHWFFDSKKRVNNFLDQKYYFLQKAYAFHFFDKQLLLIFGCLLVLTITKFFQFLYKFKEYKVIH
YNILTLSMRHNQVTLVTCEILVHYLFVYKLLPVLHLCIIVLVWKLLTQSIEMLITGDFVIYIVIQL
LLSFSCIYTEEVYITDHIELLVLFELLVLLYYWLSVSVMFYLMDRCHYEVLQLLLILLVLYLEGKI
LLNSFEVVFLLIMPLTDFLHYILYCLLYLLFCTLHFMILLVQAILLVFQVITIELHLLHIFYLKILLF
LYLFLYVLLYSLCLMFGIVIIILWLILCKLLLLLYLNDTYYLSMLFDLYLINYVLRCLVLFLLCYY
LLQIVDLEVYNLDHLVKLSEFLLLISFCNVLNTLKIHLYYVNVLYYTLVILLLYYLLVTIIVLIYLI
NLNYFIKLTKYIKIIIYIFYLRYYFSILGFQFIIYIILCITLHLALANLLFPLVWNNDLLIHWFFESKK
EIRNIFLLLLILFLKNIHFALPFSCIKMNDIKNKYLNYSYVILSYIATIIRSCINTCTCLISCSLCYSC
KKNYGYAKKIRSCCRLLWTIASICCLKTFIKRICSSYTIYCSFLFRSCNNFNFCIIRLRCYTLWSW
FRDKRHEFRYILHVSCVIFSYIRYSISWLKCEIRFSRFSKYSSINLINIKFSYINSNYDNRKFKFNCL
YISKSYLIYTTFISCVYNIFHRIYSDKSSFFSRGPANLVWSGYMSQIARKPFYFKNKRQLAGNLIP
NKYIIKLFIDTQFNIYIYLFIYMIKDIVNHRCESTKKKHMGTHLPLLGESELVSGFMTEHAAVVF
VFFFLAEYGSIVLMCILTSILFIGGYLLFEISYVFTVVNYIFFELFFIDVTFVEVQSLYTDFLNNSIIE
GLLYGFNLGLKSSLMIFTFIARASFPRIRFDQLMGFCTVLLPINFAIIILVPCVLYSFNLLPVNIPLF
LTHPPALLPQGYSTYEEGNRSRTILVNNYTYTKTIYHTRECNYTLFLYSTLLAYYYLYMIIILLNL
LGYINKKFVNFFHHFLIQNTNF
>YN608
ITILKNIKSYIKIIHLYSINLKGIFSILYLVLLLFSLLQVFLLLGSLLNRWYARLLIKVISFYLLQYLV
LEGFIIILKKCSFIHLTINTKKLKIILLMVKLLIETIKYMLNLIIQMLCLVLWQLFLLLHFYYSCLIK
NDVWVLYWYNLYLAINEYDIIYRVCICYSYFIFSHFYICSSSLSRKIYFRVWFPFFRAKNTIRYKI
LYFCFSLFTFRFRNIINFPFRSVCWYLWSFSYFNFYSYNNYRIYIIRKRSNRQQTKIIYTIERLPYR

VCWNRGFVKLRQKTKKPTKGLMGSYKKMITYIYSYYIFTITRIIIYIYIILLYIKIIIIIRYNYSILLY
KNIIYPIINFIFFLIIIFFYIMLTHLQMKRTKYCCYSSGYRKIFLYYLFLLGSFSVRGYMDGRRVFIL
MNSGFKLTLFMNLNYNYINVGYDRIMYSLRVKRDTPTYNTYWFRVEYIYLRVKNIYIQYIINIVI
YWYGLNQVYILSFDKHLFFYLLLLLYCEINNYNIILHSTYLGILSYLAINDSNQILFSLVWNNDL
LIHWFFDSKKRVNNFLDQKYYFLQKAYAFHFFDKQLLLIFGCLLVLTITKFFQFLYKFKEYKVIH
YNILTLSMRHNQVTLVTCEILVHYLFVYKLLPVLHLCIIVLVWKLLTQSIEMLITGDFVIYIVIQL
LLSFSCIYTEEVYITDHIELLVLFELLVLLYYWLSVSVMFYLMDRCHYEVLQLLLILLVLYLEGKI
LLNSFEVVFLLIMPLTDFLHYILYCLLYLLFCTLHFMILLVQAILLVFQVITIELHLLHIFYLKILLF
LYLFLYVLLYSLCLMFGIVIIILWLILCKLLLLLYLNDTYYLSMLFDLYLINYVLRCLVLFLLCYY
LLQIVDLEVYNLDHLVKLSEFLLLISFCNVLNTLKIHLYYVNVLYYTLVILLLYYLLVTIIVLIYLI
NLNYFIKLTKYIKIIIYIFYLRYYFSILGFQFIIYIILCITLHLALANLLFPLVWNNDLLIHWFFESKK
EIRNIFLLLLILFLKNIHFALPFSCIKMNDIKNKYLNYSYVILSYIATIIRSCINTCTCLISCSLCYSC
KKNYGYAKKIRSCCRLLWTIASICCLKTFIKRICSSYTIYCSFLFRSCNNFNFCIIRLRCYTLWSW
FRDKRHEFRYILHVSCVIFSYIRYSISWLKCEIRFSRFSKYSSINLINIKFSYINSNYDNRKFKFNCL
YISKSYLIYTTFISCVYNIFHRIYSDKSSFFSRGPANLVWSGYMSQIARKPFYFKNKRQLAGNLIP
NKYIIKLFIDTQFNIYIYLFIYMIKDIVNHRCESTKKKHMGTHLPLLGESELVSGFMTEHAAVVF
VFFFLAEYGSIVLMCILTSILFIGGYLLFEISYVFTVVNYIFFELFFIDVTFVEVQSLYTDFLNNSIIE
GLLYGFNLGLKSSLMIFTFIARASFPRIRFDQLMGFCTVLLPINFAIIILVPCVLYSFNLLPVNIPLF
LTHPPALLPQGYSTYEEGNRSRTILVNNYTYTKTIYHTRECNYTLFLYSTLLAYYYLYMIIILLNL
LGYINKKFVNFFHHFLIQNTNF
>YN617
ITILKNIKSYIKIIHLYSINLKGIFSILYLVLLLFSLLQVFLLLGSLLNRWYARLLIKVISFYLLQYLV
LEGFIIILKKCSFIHLTINTKKLKIILLMVKLLIETIKYMLNLIIQMLCLVLWQLFLLLHFYYSCLIK
NDVWVLYWYNLYLAINEYDIIYRVCICYSYFIFSHFYICSSSLSRKIYFRVWFPFFRAKNTIRYKI
LYFCFSLFTFRFRNIINFPFRSVCWYLWSFSYFNFYSYNNYRIYIIRKRSNRQQTKIIYTIERLPYR
VCWNRGFVKLRQKTKKPTKGLMGSYKKMITYIYSYYIFTITRIIIYIYIILLYIKIIIIIRYNYSILLY
KNIIYPIINFIFFLIIIFFYIMLTHLQMKRTKYCCYSSGYRKIFLYYLFLLGSFSVRGYMDGRRVFIL
MNSGFKLTLFMNLNYNYINVGYDRIMYSLRVKRDTPTYNTYWFRVEYIYLRVKNIYIQYIINIVI
YWYGLNQVYILSFDKHLFFYLLLLLYCEINNYNIILHSTYLGILSYLAINDSNQILFSLVWNNDL
LIHWFFDSKKRVNNFLDQKYYFLQKAYAFHFFDKQLLLIFGCLLVLTITKFFQFLYKFKEYKVIH
YNILTLSMRHNQVTLVTCEILVHYLFVYKLLPVLHLCIIVLVWKLLTQSIEMLITGDFVIYIVIQL
LLSFSCIYTEEVYITDHIELLVLFELLVLLYYWLSVSVMFYLMDRCHYEVLQLLLILLVLYLEGKI
LLNSFEVVFLLIMPLTDFLHYILYCLLYLLFCTLHFMILLVQAILLVFQVITIELHLLHIFYLKILLF
LYLFLYVLLYSLCLMFGIVIIILWLILCKLLLLLYLNDTYYLSMLFDLYLINYVLRCLVLFLLCYY
LLQIVDLEVYNLDHLVKLSEFLLLISFCNVLNTLKIHLYYVNVLYYTLVILLLYYLLVTIIVLIYLI
NLNYFIKLTKYIKIIIYIFYLRYYFSILGFQFIIYIILCITLHLALANLLFPLVWNNDLLIHWFFESKK
EIRNIFLLLLILFLKNIHFALPFSCIKMNDIKNKYLNYSYVILSYIATIIRSCINTCTCLISCSLCYSC
KKNYGYAKKIRSCCRLLWTIASICCLKTFIKRICSSYTIYCSFLFRSCNNFNFCIIRLRCYTLWSW
FRDKRHEFRYILHVSCVIFSYIRYSISWLKCEIRFSRFSKYSSINLINIKFSYINSNYDNRKFKFNCL
YISKSYLIYTTFISCVYNIFHRIYSDKSSFFSRGPANLVWSGYMSQIARKPFYFKNKRQLAGNLIP
NKYIIKLFIDTQFNIYIYLFIYMIKDIVNHRCESTKKKHMGTHLPLLGESELVSGFMTEHAAVVF
VFFFLAEYGSIVLMCILTSILFIGGYLLFEISYVFTVVNYIFFELFFIDVTFVEVQSLYTDFLNNSIIE
GLLYGFNLGLKSSLMIFTFIARASFPRIRFDQLMGFCTVLLPINFAIIILVPCVLYSFNLLPVNIPLF
LTHPPALLPQGYSTYEEGNRSRTILVNNYTYTKTIYHTRECNYTLFLYSTLLAYYYLYMIIILLNL

LGYINKKFVNFFHHFLIQNTN
>YN626
ITILKNIKSYIKIIHLYSINLKGIFSILYLVLLLFSLLQVFLLLGSLLNRWYARLLIKVISFYLLQYLV
LEGFIIILKKCSFIHLTINTKKLKIILLMVKLLIETIKYMLNLIIQMLCLVLWQLFLLLHFYYSCLIK
NDVWVLYWYNLYLAINEYDIIYRVCICYSYFIFSHFYICSSSLSRKIYFRVWFPFFRAKNTIRYKI
LYFCFSLFTFRFRNIINFPFRSVCWYLWSFSYFNFYSYNNYRIYIIRKRSNRQQTKIIYTIERLPYR
VCWNRGFVKLRQKTKKPTKGLMGSYKKMITYIYSYYIFTITRIIIYIYIILLYIKIIIIIRYNYSILLY
KNIIYPIINFIFFLIIIFFYIMLTHLQMKRTKYCCYSSGYRKIFLYYLFLLGSFSVRGYMDGRRVFIL
MNSGFKLTLFMNLNYNYINVGYDRIMYSLRVKRDTPTYNTYWFRVEYIYLRVKNIYIQYIINIVI
YWYGLNQVYILSFDKHLFFYLLLLLYCEINNYNIILHSTYLGILSYLAINDSNQILFSLVWNNDL
LIHWFFDSKKRVNNFLDQKYYFLQKAYAFHFFDKQLLLIFGCLLVLTITKFFQFLYKFKEYKVIH
YNILTLSMRHNQVTLVTCEILVHYLFVYKLLPVLHLCIIVLVWKLLTQSIEMLITGDFVIYIVIQL
LLSFSCIYTEEVYITDHIELLVLFELLVLLYYWLSVSVMFYLMDRCHYEVLQLLLILLVLYLEGKI
LLNSFEVVFLLIMPLTDFLHYILYCLLYLLFCTLHFMILLVQAILLVFQVITIELHLLHIFYLKILLF
LYLFLYVLLYSLCLMFGIVIIILWLILCKLLLLLYLNDTYYLSMLFDLYLINYVLRCLVLFLLCYY
LLQIVDLEVYNLDHLVKLSEFLLLISFCNVLNTLKIHLYYVNVLYYTLVILLLYYLLVTIIVLIYLI
NLNYFIKLTKYIKIIIYIFYLRYYFSILGFQFIIYIILCITLHLALANLLFPLVWNNDLLIHWFFESKK
EIRNIFLLLLILFLKNIHFALPFSCIKMNDIKNKYLNYSYVILSYIATIIRSCINTCTCLISCSLCYSC
KKNYGYAKKIRSCCRLLWTIASICCLKTFIKRICSSYTIYCSFLFRSCNNFNFCIIRLRCYTLWSW
FRDKRHEFRYILHVSCVIFSYIRYSISWLKCEIRFSRFSKYSSINLINIKFSYINSNYDNRKFKFNCL
YISKSYLIYTTFISCVYNIFHRIYSDKSSFFSRGPANLVWSGYMSQIARKPFYFKNKRQLAGNLIP
NKYIIKLFIDTQFNIYIYLFIYMIKDIVNHRCESTKKKHMGTHLPLLGESELVSGFMTEHAAVVF
VFFFLAEYGSIVLMCILTSILFIGGYLLFEISYVFTVVNYIFFELFFIDVTFVEVQSLYTDFLNNSIIE
GLLYGFNLGLKSSLMIFTFIARASFPRIRFDQLMGFCTVLLPINFAIIILVPCVLYSFNLLPVNIPLF
LTHPPALLPQGYSTYEEGNRSRTILVNNYTYTKTIYHTRECNYTLFLYSTLLAYYYLYMIIILLNL
LGYINKKFVNFFHHFLIQNTNF
>YN634
ITILKNIKSYIKIIHLYSINLKGIFSILYLVLLLFSLLQVFLLLGSLLNRWYARLLIKVISFYLLQYLV
LEGFIIILKKCSFIHLTINTKKLKIILLMVKLLIETIKYMLNLIIQMLCLVLWQLFLLLHFYYSCLIK
NDVWVLYWYNLYLAINEYDIIYRVCICYSYFIFSHFYICSSSLSRKIYFRVWFPFFRAKNTIRYKI
LYFCFSLFTFRFRNIINFPFRSVCWYLWSFSYFNFYSYNNYRIYIIRKRSNRQQTKIIYTIERLPYR
VCWNRGFVKLRQKTKKPTKGLMGSYKKMITYIYSYYIFTITRIIIYIYIILLYIKIIIIIRYNYSILLY
KNIIYPIINFIFFLIIIFFYIMLTHLQMKRTKYCCYSSGYRKIFLYYLFLLGSFSVRGYMDGRRVFIL
MNSGFKLTLFMNLNYNYINVGYDRIMYSLRVKRDTPTYNTYWFRVEYIYLRVKNIYIQYIINIVI
YWYGLNQVYILSFDKHLFFYLLLLLYCEINNYNIILHSTYLGILSYLAINDSNQILFSLVWNNDL
LIHWFFDSKKRVNNFLDQKYYFLQKAYAFHFFDKQLLLIFGCLLVLTITKFFQFLYKFKEYKVIH
YNILTLSMRHNQVTLVTCEILVHYLFVYKLLPVLHLCIIVLVWKLLTQSIEMLITGDFVIYIVIQL
LLSFSCIYTEEVYITDHIELLVLFELLVLLYYWLSVSVMFYLMDRCHYEVLQLLLILLVLYLEGKI
LLNSFEVVFLLIMPLTDFLHYILYCLLYLLFCTLHFMILLVQAILLVFQVITIELHLLHIFYLKILLF
LYLFLYVLLYSLCLMFGIVIIILWLILCKLLLLLYLNDTYYLSMLFDLYLINYVLRCLVLFLLCYY
LLQIVDLEVYNLDHLVKLSEFLLLISFCNVLNTLKIHLYYVNVLYYTLVILLLYYLLVTIIVLIYLI
NLNYFIKLTKYIKIIIYIFYLRYYFSILGFQFIIYIILCITLHLALANLLFPLVWNNDLLIHWFFESKK
EIRNIFLLLLILFLKNIHFALPFSCIKMNDIKNKYLNYSYVILSYIATIIRSCINTCTCLISCSLCYSC
KKNYGYAKKIRSCCRLLWTIASICCLKTFIKRICSSYTIYCSFLFRSCNNFNFCIIRLRCYTLWSW

FRDKRHEFRYILHVSCVIFSYIRYSISWLKCEIRFSRFSKYSSINLINIKFSYINSNYDNRKFKFNCL
YISKSYLIYTTFISCVYNIFHRIYSDKSSFFSRGPANLVWSGYMSQIARKPFYFKNKRQLAGNLIP
NKYIIKLFIDTQFNIYIYLFIYMIKDIVNHRCESTKKKHMGTHLPLLGESELVSGFMTEHAAVVF
VFFFLAEYGSIVLMCILTSILFIGGYLLFEISYVFTVVNYIFFELFFIDVTFVEVQSLYTDFLNNSIIE
GLLYGFNLGLKSSLMIFTFIARASFPRIRFDQLMGFCTVLLPINFAIIILVPCVLYSFNLLPVNIPLF
LTHPPALLPQGYSTYEEGNRSRTILVNNYTYTKTIYHTRECNYTLFLYSTLLAYYYLYMIIILLNL
LGYINKKFVNFFHHFLIQNTNF
>YN635
ITILKNIKSYIKIIHLYSINLKGIFSILYLVLLLFSLLQVFLLLGSLLNRWYARLLIKVISFYLLQYLV
LEGFIIILKKCSFIHLTINTKKLKIILLMVKLLIETIKYMLNLIIQMLCLVLWQLFLLLHFYYSCLIK
NDVWVLYWYNLYLAINEYDIIYRVCICYSYFIFSHFYICSSSLSRKIYFRVWFPFFRAKNTIRYKI
LYFCFSLFTFRFRNIINFPFRSVCWYLWSFSYFNFYSYNNYRIYIIRKRSNRQQTKIIYTIERLPYR
VCWNRGFVKLRQKTKKPTKGLMGSYKKMITYIYSYYIFTITRIIIYIYIILLYIKIIIIIRYNYSILLY
KNIIYPIINFIFFLIIIFFYIMLTHLQMKRTKYCCYSSGYRKIFLYYLFLLGSFSVRGYMDGRRVFIL
MNSGFKLTLFMNLNYNYINVGYDRIMYSLRVKRDTPTYNTYWFRVEYIYLRVKNIYIQYIINIVI
YWYGLNQVVYILSFDKHLFFYLLLLLYCEINNYNIILHSTYLGILSYLAINDSNQILFSLVWNNDL
LIHWFFDSKKRVNNFLDQKYYFLQKAYAFHFFDKQLLLIFGCLLVLTITKFFQFLYKFKEYKVIH
YNILTLSMRHNQVTLVTCEILVHYLFVYKLLPVLHLCIIVLVWKLLTQSIEMLITGDFVIYIVIQL
LLSFSCIYTEEVYITDHIELLVLFELLVLLYYWLSVSVMFYLMDRCHYEVLQLLLILLVLYLEGKI
LLNSFEVVFLLIMPLTDFLHYILYCLLYLLFCTLHFMILLVQAILLVFQVITIELHLLHIFYLKILLF
LYLFLYVLLYSLCLMFGIVIIILWLILCKLLLLLYLNDTYYLSMLFDLYLINYVLRCLVLFLLCYY
LLQIVDLEVYNLDHLVKLSEFLLLISFCNVLNTLKIHLYYVNVLYYTLVILLLYYLLVTIIVLIYLI
NLNYFIKLTKYIKIIIYIFYLRYYFSILGFQFIIYIILCITLHLALANLLFPLVWNNDLLIHWFFESKK
EIRNIFLLLLILFLKNIHFALPFSCIKMNDIKNKYLNYSYVILSYIATIIRSCINTCTCLISCSLCYSC
KKNYGYAKKIRSCCRLLWTIASICCLKTFIKRICSSYTIYCSFLFRSCNNFNFCIIRLRCYTLWSW
FRDKRHEFRYILHVSCVIFSYIRYSISWLKCEIRFSRFSKYSSINLINIKFSYINSNYDNRKFKFNCL
YISKSYLIYTTFISCVYNIFHRIYSDKSSFFSRGPANLVWSGYMSQIARKPFYFKNKRQLAGNLIP
NKYIIKLFIDTQFNIYIYLFIYMIKDIVNHRCESTKKKHMGTHLPLLGESELVSGFMTEHAAVVF
VFFFLAEYGSIVLMCILTSILFIGGYLLFEISYVFTVVNYIFFELFFIDVTFVEVQSLYTDFLNNSIIE
GLLYGFNLGLKSSLMIFTFIARASFPRIRFDQLMGFCTVLLPINFAIIILVPCVLYSFNLLPVNIPLF
LTHPPALLPQGYSTYEEGNRSRTILVNNYTYTKTIYHTRECNYTLFLYSTLLAYYYLYMIIILLNL
LGYINKKFVNFFHHFLIQNTNF
>YN640
ITILKNIKSYIKIIHLYSINLKGIFSILYLVLLLFSLLQVFLLLGSLLNRWYARLLIKVISFYLLQYLV
LEGFIIILKKCSFIHLTINTKKLKIILLMVKLLIETIKYMLNLIIQMLCLVLWQLFLLLHFYYSCLIK
NDVWVLYWYNLYLAINEYDIIYRVCICYSYFIFSHFYICSSSLSRKIYFRVWFPFFRAKNTIRYKI
LYFCFSLFTFRFRNIINFPFRSVCWYLWSFSYFNFYSYNNYRIYIIRKRSNRQQTKIIYTIERLPYR
VCWNRGFVKLRQKTKKPTKGLMGSYKKMITYIYSYYIFTITRIIIYIYIILLYIKIIIIIRYNYSILLY
KNIIYPIINFIFFLIIIFFYIMLTHLQMKRTKYCCYSSGYRKIFLYYLFLLGSFSVRGYMDGRRVFIL
MNSGFKLTLFMNLNYNYINVGYDRIMYSLRVKRDTPTYNTYWFRVEYIYLRVKNIYIQYIINIVI
YWYGLNQVVYILSFDKHLFFYLLLLLYCEINNYNIILHSTYLGILSYLAINDSNQILFSLVWNNDL
LIHWFFDSKKRVNNFLDQKYYFLQKAYAFHFFDKQLLLIFGCLLVLTITKFFQFLYKFKEYKVIH
YNILTLSMRHNQVTLVTCEILVHYLFVYKLLPVLHLCIIVLVWKLLTQSIEMLITGDFVIYIVIQL
LLSFSCIYTEEVYITDHIELLVLFELLVLLYYWLSVSVMFYLMDRCHYEVLQLLLILLVLYLEGKI

LLNSFEVVFLLIMPLTDFLHYILYCLLYLLFCTLHFMILLVQAILLVFQVITIELHLLHIFYLKILLF
LYLFLYVLLYSLCLMFGIVIIILWLILCKLLLLLYLNDTYYLSMLFDLYLINYVLRCLVLFLLCYY
LLQIVDLEVYNLDHLVKLSEFLLLISFCNVLNTLKIHLYYVNVLYYTLVILLLYYLLVTIIVLIYLI
NLNYFIKLTKYIKIIIYIFYLRYYFSILGFQFIIYIILCITLHLALANLLFPLVWNNDLLIHWFFESKK
EIRNIFLLLLILFLKNIHFALPFSCIKMNDIKNKYLNYSYVILSYIATIIRSCINTCTCLISCSLCYSC
KKNYGYAKKIRSCCRLLWTIASICCLKTFIKRICSSYTIYCSFLFRSCNNFNFCIIRLRCYTLWSW
FRDKRHEFRYILHVSCVIFSYIRYSISWLKCEIRFSRFSKYSSINLINIKFSYINSNYDNRKFKFNCL
YISKSYLIYTTFISCVYNIFHRIYSDKSSFFSRGPANLVWSGYMSQIARKPFYFKNKRQLAGNLIP
NKYIIKLFIDTQFNIYIYLFIYMIKDIVNHRCESTKKKHMGTHLPLLGESELVSGFMTEHAAVVF
VFFFLAEYGSIVLMCILTSILFIGGYLLFEISYVFTVVNYIFFELFFIDVTFVEVQSLYTDFLNNSIIE
GLLYGFNLGLKSSLMIFTFIARASFPRIRFDQLMGFCTVLLPINFAIIILVPCVLYSFNLLPVNIPLF
LTHPPALLPQGYSTYEEGNRSRTILVNNYTYTKTIYHTRECNYTLFLYSTLLAYYYLYMIIILLNL
LGYINKKFVNFFHHFLIQNTNF
>YN643
ITILKNIKSYIKIIHLYSINLKGIFSILYLVLLLFSLLQVFLLLGSLLNRWYARLLIKVISFYLLQYLV
LEGFIIILKKCSFIHLTINTKKLKIILLMVKLLIETIKYMLNLIIQMLCLVLWQLFLLLHFYYSCLIK
NDVWVLYWYNLYLAINEYDIIYRVCICYSYFIFSHFYICSSSLSRKIYFRVWFPFFRAKNTIRYKI
LYFCFSLFTFRFRNIINFPFRSVCWYLWSFSYFNFYSYNNYRIYIIRKRSNRQQTKIIYTIERLPYR
VCWNRGFVKLRQKTKKPTKGLMGSYKKMITYIYSYYIFTITRIIIYIYIILLYIKIIIIIRYNYSILLY
KNIIYPIINFIFFLIIIFFYIMLTHLQMKRTKYCCYSSGYRKIFLYYLFLLGSFSVRGYMDGRRVFIL
MNSGFKLTLFMNLNYNYINVGYDRIMYSLRVKRDTPTYNTYWFRVEYIYLRVKNIYIQYIINIVI
YWYGLNQVYILSFDKHLFFYLLLLLYCEINNYNIILHSTYLGILSYLAINDSNQILFSLVWNNDL
LIHWFFDSKKRVNNFLDQKYYFLQKAYAFHFFDKQLLLIFGCLLVLTITKFFQFLYKFKEYKVIH
YNILTLSMRHNQVTLVTCEILVHYLFVYKLLPVLHLCIIVLVWKLLTQSIEMLITGDFVIYIVIQL
LLSFSCIYTEEVYITDHIELLVLFELLVLLYYWLSVSVMFYLMDRCHYEVLQLLLILLVLYLEGKI
LLNSFEVVFLLIMPLTDFLHYILYCLLYLLFCTLHFMILLVQAILLVFQVITIELHLLHIFYLKILLF
LYLFLYVLLYSLCLMFGIVIIILWLILCKLLLLLYLNDTYYLSMLFDLYLINYVLRCLVLFLLCYY
LLQIVDLEVYNLDHLVKLSEFLLLISFCNVLNTLKIHLYYVNVLYYTLVILLLYYLLVTIIVLIYLI
NLNYFIKLTKYIKIIIYIFYLRYYFSILGFQFIIYIILCITLHLALANLLFPLVWNNDLLIHWFFESKK
EIRNIFLLLLILFLKNIHFALPFSCIKMNDIKNKYLNYSYVILSYIATIIRSCINTCTCLISCSLCYSC
KKNYGYAKKIRSCCRLLWTIASICCLKTFIKRICSSYTIYCSFLFRSCNNFNFCIIRLRCYTLWSW
FRDKRHEFRYILHVSCVIFSYIRYSISWLKCEIRFSRFSKYSSINLINIKFSYINSNYDNRKFKFNCL
YISKSYLIYTTFISCVYNIFHRIYSDKSSFFSRGPANLVWSGYMSQIARKPFYFKNKRQLAGNLIP
NKYIIKLFIDTQFNIYIYLFIYMIKDIVNHRCESTKKKHMGTHLPLLGESELVSGFMTEHAAVVF
VFFFLAEYGSIVLMCILTSILFIGGYLLFEISYVFTVVNYIFFELFFIDVTFVEVQSLYTDFLNNSIIE
GLLYGFNLGLKSSLMIFTFIARASFPRIRFDQLMGFCTVLLPINFAIIILVPCVLYSFNLLPVNIPLF
LTHPPALLPQGYSTYEEGNRSRTILVNNYTYTKTIYHTRECNYTLFLYSTLLAYYYLYMIIILLNL
LGYINKKFVNFFHHFLIQNTNF
>YN651
ITILKNIKSYIKIIHLYSINLKGIFSILYLVLLLFSLLQVFLLLGSLLNRWYARLLIKVISFYLLQYLV
LEGFIIILKKCSFIHLTINTKKLKIILLMVKLLIETIKYMLNLIIQMLCLVLWQLFLLLHFYYSCLIK
NDVWVLYWYNLYLAINEYDIIYRVCICYSYFIFSHFYICSSSLSRKIYFRVWFPFFRAKNTIRYKI
LYFCFSLFTFRFRNIINFPFRSVCWYLWSFSYFNFYSYNNYRIYIIRKRSNRQQTKIIYTIERLPYR
VCWNRGFVKLRQKTKKPTKGLMGSYKKMITYIYSYYIFTITRIIIYIYIILLYIKIIIIIRYNYSILLY

KNIIYPIINFIFFLIIIFFYIMLTHLQMKRTKYCCYSSGYRKIFLYYLFLLGSFSVRGYMDGRRVFIL
MNSGFKLTLFMNLNYNYINVGYDRIMYSLRVKRDTPTYNTYWFRVEYIYLRVKNIYIQYIINIVI
YWYGLNQVVYILSFDKHLFFYLLLLLYCEINNYNIILHSTYLGILSYLAINDSNQILFSLVWNNDL
LIHWFFDSKKRVNNFLDQKYYFLQKAYAFHFFDKQLLLIFGCLLVLTITKFFQFLYKFKEYKVIH
YNILTLSMRHNQVTLVTCEILVHYLFVYKLLPVLHLCIIVLVWKLLTQSIEMLITGDFVIYIVIQL
LLSFSCIYTEEVYITDHIELLVLFELLVLLYYWLSVSVMFYLMDRCHYEVLQLLLILLVLYLEGKI
LLNSFEVVFLLIMPLTDFLHYILYCLLYLLFCTLHFMILLVQAILLVFQVITIELHLLHIFYLKILLF
LYLFLYVLLYSLCLMFGIVIIILWLILCKLLLLLYLNDTYYLSMLFDLYLINYVLRCLVLFLLCYY
LLQIVDLEVYNLDHLVKLSEFLLLISFCNVLNTLKIHLYYVNVLYYTLVILLLYYLLVTIIVLIYLI
NLNYFIKLTKYIKIIIYIFYLRYYFSILGFQFIIYIILCITLHLALANLLFPLVWNNDLLIHWFFESKK
EIRNIFLLLLILFLKNIHFALPFSCIKMNDIKNKYLNYSYVILSYIATIIRSCINTCTCLISCSLCYSC
KKNYGYAKKIRSCCRLLWTIASICCLKTFIKRICSSYTIYCSFLFRSCNNFNFCIIRLRCYTLWSW
FRDKRHEFRYILHVSCVIFSYIRYSISWLKCEIRFSRFSKYSSINLINIKFSYINSNYDNRKFKFNCL
YISKSYLIYTTFISCVYNIFHRIYSDKSSFFSRGPANLVWSGYMSQIARKPFYFKNKRQLAGNLIP
NKYIIKLFIDTQFNIYIYLFIYMIKDIVNHRCESTKKKHMGTHLPLLGESELVSGFMTEHAAVVF
VFFFLAEYGSIVLMCILTSILFIGGYLLFEISYVFTVVNYIFFELFFIDVTFVEVQSLYTDFLNNSIIE
GLLYGFNLGLKSSLMIFTFIARASFPRIRFDQLMGFCTVLLPINFAIIILVPCVLYSFNLLPVNIPLF
LTHPPALLPQGYSTYEEGNRSRTILVNNYTYTKTIYHTRECNYTLFLYSTLLAYYYLYMIIILLNL
LGYINKKFVNFFHHFLIQNTNF
>YN657
ITILKNIKSYIKIIHLYSINLKGIFSILYLVLLLFSLLQVFLLLGSLLNRWYARLLIKVISFYLLQYLV
LEGFIIILKKCSFIHLTINTKKLKIILLMVKLLIETIKYMLNLIIQMLCLVLWQLFLLLHFYYSCLIK
NDVWVLYWYNLYLAINEYDIIYRVCICYSYFIFSHFYICSSSLSRKIYFRVWFPFFRAKNTIRYKI
LYFCFSLFTFRFRNIINFPFRSVCWYLWSFSYFNFYSYNNYRIYIIRKRSNRQQTKIIYTIERLPYR
VCWNRGFVKLRQKTKKPTKGLMGSYKKMITYIYSYYIFTITRIIIYIYIILLYIKIIIIIRYNYSILLY
KNIIYPIINFIFFLIIIFFYIMLTHLQMKRTKYCCYSSGYRKIFLYYLFLLGSFSVRGYMDGRRVFIL
MNSGFKLTLFMNLNYNYINVGYDRIMYSLRVKRDTPTYNTYWFRVEYIYLRVKNIYIQYIINIVI
YWYGLNQVVYILSFDKHLFFYLLLLLYCEINNYNIILHSTYLGILSYLAINDSNQILFSLVWNNDL
LIHWFFDSKKRVNNFLDQKYYFLQKAYAFHFFDKQLLLIFGCLLVLTITKFFQFLYKFKEYKVIH
YNILTLSMRHNQVTLVTCEILVHYLFVYKLLPVLHLCIIVLVWKLLTQSIEMLITGDFVIYIVIQL
LLSFSCIYTEEVYITDHIELLVLFELLVLLYYWLSVSVMFYLMDRCHYEVLQLLLILLVLYLEGKI
LLNSFEVVFLLIMPLTDFLHYILYCLLYLLFCTLHFMILLVQAILLVFQVITIELHLLHIFYLKILLF
LYLFLYVLLYSLCLMFGIVIIILWLILCKLLLLLYLNDTYYLSMLFDLYLINYVLRCLVLFLLCYY
LLQIVDLEVYNLDHLVKLSEFLLLISFCNVLNTLKIHLYYVNVLYYTLVILLLYYLLVTIIVLIYLI
NLNYFIKLTKYIKIIIYIFYLRYYFSILGFQFIIYIILCITLHLALANLLFPLVWNNDLLIHWFFESKK
EIRNIFLLLLILFLKNIHFALPFSCIKMNDIKNKYLNYSYVILSYIATIIRSCINTCTCLISCSLCYSC
KKNYGYAKKIRSCCRLLWTIASICCLKTFIKRICSSYTIYCSFLFRSCNNFNFCIIRLRCYTLWSW
FRDKRHEFRYILHVSCVIFSYIRYSISWLKCEIRFSRFSKYSSINLINIKFSYINSNYDNRKFKFNCL
YISKSYLIYTTFISCVYNIFHRIYSDKSSFFSRGPANLVWSGYMSQIARKPFYFKNKRQLAGNLIP
NKYIIKLFIDTQFNIYIYLFIYMIKDIVNHRCESTKKKHMGTHLPLLGESELVSGFMTEHAAVVF
VFFFLAEYGSIVLMCILTSILFIGGYLLFEISYVFTVVNYIFFELFFIDVTFVEVQSLYTDFLNNSIIE
GLLYGFNLGLKSSLMIFTFIARASFPRIRFDQLMGFCTVLLPINFAIIILVPCVLYSFNLLPVNIPLF
LTHPPALLPQGYSTYEEGNRSRTILVNNYTYTKTIYHTRECNYTLFLYSTLLAYYYLYMIIILLNL
LGYINKKFVNFFHHFLIQNTNI

>YN661
ITILKNIKSYIKIIHLYSINLKGIFSILYLVLLLFSLLQVFLLLGSLLNRWYARLLIKVISFYLLQYLV
LEGFIIILKKCSFIHLTINTKKLKIILLMVKLLIETIKYMLNLIIQMLCLVLWQLFLLLHFYYSCLIK
NDVWVLYWYNLYLAINEYDIIYRVCICYSYFIFSHFYICSSSLSRKIYFRVWFPFFRAKNTIRYKI
LYFCFSLFTFRFRNIINFPFRSVCWYLWSFSYFNFYSYNNYRIYIIRKRSNRQQTKIIYTIERLPYR
VCWNRGFVKLRQKTKKPTKGLMGSYKKMITYIYSYYIFTITRIIIYIYIILLYIKIIIIIRYNYSILLY
KNIIYPIINFIFFLIIIFFYIMLTHLQMKRTKYCCYSSGYRKIFLYYLFLLGSFSVRGYMDGRRVFIL
MNSGFKLTLFMNLNYNYINVGYDRIMYSLRVKRDTPTYNTYWFRVEYIYLRVKNIYIQYIINIVI
YWYGLNQVVYILSFDKHLFFYLLLLLYCEINNYNIILHSTYLGILSYLAINDSNQILFSLVWNNDL
LIHWFFDSKKRVNNFLDQKYYFLQKAYAFHFFDKQLLLIFGCLLVLTITKFFQFLYKFKEYKVIH
YNILTLSMRHNQVTLVTCEILVHYLFVYKLLPVLHLCIIVLVWKLLTQSIEMLITGDFVIYIVIQL
LLSFSCIYTEEVYITDHIELLVLFELLVLLYYWLSVSVMFYLMDRCHYEVLQLLLILLVLYLEGKI
LLNSFEVVFLLIMPLTDFLHYILYCLLYLLFCTLHFMILLVQAILLVFQVITIELHLLHIFYLKILLF
LYLFLYVLLYSLCLMFGIVIIILWLILCKLLLLLYLNDTYYLSMLFDLYLINYVLRCLVLFLLCYY
LLQIVDLEVYNLDHLVKLSEFLLLISFCNVLNTLKIHLYYVNVLYYTLVILLLYYLLVTIIVLIYLI
NLNYFIKLTKYIKIIIYIFYLRYYFSILGFQFIIYIILCITLHLALANLLFPLVWNNDLLIHWFFESKK
EIRNIFLLLLILFLKNIHFALPFSCIKMNDIKNKYLNYSYVILSYIATIIRSCINTCTCLISCSLCYSC
KKNYGYAKKIRSCCRLLWTIASICCLKTFIKRICSSYTIYCSFLFRSCNNFNFCIIRLRCYTLWSW
FRDKRHEFRYILHVSCVIFSYIRYSISWLKCEIRFSRFSKYSSINLINIKFSYINSNYDNRKFKFNCL
YISKSYLIYTTFISCVYNIFHRIYSDKSSFFSRGPANLVWSGYMSQIARKPFYFKNKRQLAGNLIP
NKYIIKLFIDTQFNIYIYLFIYMIKDIVNHRCESTKKKHMGTHLPLLGESELVSGFMTEHAAVVF
VFFFLAEYGSIVLMCILTSILFIGGYLLFEISYVFTVVNYIFFELFFIDVTFVEVQSLYTDFLNNSIIE
GLLYGFNLGLKSSLMIFTFIARASFPRIRFDQLMGFCTVLLPINFAIIILVPCVLYSFNLLPVNIPLF
LTHPPALLPQGYSTYEEGNRSRTILVNNYTYTKTIYHTRECNYTLFLYSTLLAYYYLYMIIILLNL
LGYINKKFVNFFHHFLIQNTNF
>YN670
ITILKNIKSYIKIIHLYSINLKGIFSILYLVLLLFSLLQVFLLLGSLLNRWYARLLIKVISFYLLQYLV
LEGFIIILKKCSFIHLTINTKKLKIILLMVKLLIETIKYMLNLIIQMLCLVLWQLFLLLHFYYSCLIK
NDVWVLYWYNLYLAINEYDIIYRVCICYSYFIFSHFYICSSSLSRKIYFRVWFPFFRAKNTIRYKI
LYFCFSLFTFRFRNIINFPFRSVCWYLWSFSYFNFYSYNNYRIYIIRKRSNRQQTKIIYTIERLPYR
VCWNRGFVKLRQKTKKPTKGLMGSYKKMITYIYSYYIFTITRIIIYIYIILLYIKIIIIIRYNYSILLY
KNIIYPIINFIFFLIIIFFYIMLTHLQMKRTKYCCYSSGYRKIFLYYLFLLGSFSVRGYMDGRRVFIL
MNSGFKLTLFMNLNYNYINVGYDRIMYSLRVKRDTPTYNTYWFRVEYIYLRVKNIYIQYIINIVI
YWYGLNQVVYILSFDKHLFFYLLLLLYCEINNYNIILHSTYLGILSYLAINDSNQILFSLVWNNDL
LIHWFFDSKKRVNNFLDQKYYFLQKAYAFHFFDKQLLLIFGCLLVLTITKFFQFLYKFKEYKVIH
YNILTLSMRHNQVTLVTCEILVHYLFVYKLLPVLHLCIIVLVWKLLTQSIEMLITGDFVIYIVIQL
LLSFSCIYTEEVYITDHIELLVLFELLVLLYYWLSVSVMFYLMDRCHYEVLQLLLILLVLYLEGKI
LLNSFEVVFLLIMPLTDFLHYILYCLLYLLFCTLHFMILLVQAILLVFQVITIELHLLHIFYLKILLF
LYLFLYVLLYSLCLMFGIVIIILWLILCKLLLLLYLNDTYYLSMLFDLYLINYVLRCLVLFLLCYY
LLQIVDLEVYNLDHLVKLSEFLLLISFCNVLNTLKIHLYYVNVLYYTLVILLLYYLLVTIIVLIYLI
NLNYFIKLTKYIKIIIYIFYLRYYFSILGFQFIIYIILCITLHLALANLLFPLVWNNDLLIHWFFESKK
EIRNIFLLLLILFLKNIHFALPFSCIKMNDIKNKYLNYSYVILSYIATIIRSCINTCTCLISCSLCYSC
KKNYGYAKKIRSCCRLLWTIASICCLKTFIKRICSSYTIYCSFLFRSCNNFNFCIIRLRCYTLWSW
FRDKRHEFRYILHVSCVIFSYIRYSISWLKCEIRFSRFSKYSSINLINIKFSYINSNYDNRKFKFNCL

YISKSYLIYTTFISCVYNIFHRIYSDKSSFFSRGPANLVWSGYMSQIARKPFYFKNKRQLAGNLIP
NKYIIKLFIDTQFNIYIYLFIYMIKDIVNHRCESTKKKHMGTHLPLLGESELVSGFMTEHAAVVF
VFFFLAEYGSIVLMCILTSILFIGGYLLFEISYVFTVVNYIFFELFFIDVTFVEVQSLYTDFLNNSIIE
GLLYGFNLGLKSSLMIFTFIARASFPRIRFDQLMGFCTVLLPINFAIIILVPCVLYSFNLLPVNIPLF
LTHPPALLPQGYSTYEEGNRSRTILVNNYTYTKTIYHTRECNYTLFLYSTLLAYYYLYMIIILLNL
LGYINKKFVNFFHHFLIQNTNI

>YN673

ITILKNIKSYIKIIHLYSINLKGIFSILYLVLLLFSLLQVFLLLGSLLNRWYARLLIKVISFYLLQYLV
LEGFIIILKKCSFIHLTINTKKLKIILLMVKLLIETIKYMLNLIIQMLCLVLWQLFLLLHFYYSCLIK
NDVWVLYWYNLYLAINEYDIIYRVCICYSYFIFSHFYICSSSLSRKIYFRVWFPFFRAKNTIRYKI
LYFCFSLFTFRFRNIINFPFRSVCWYLWSFSYFNFYSYNNYRIYIIRKRSNRQQTKIIYTIERLPYR
VCWNRGFVKLRQKTKKPTKGLMGSYKKMITYIYSYYIFTITRIIIYIYIILLYIKIIIIIRYNYSILLY
KNIIYPIINFIFFLIIIFFYIMLTHLQMKRTKYCCYSSGYRKIFLYYLFLLGSFSVRGYMDGRRVFIL
MNSGFKLTLFMNLNYNYINVGYDRIMYSLRVKRDTPTYNTYWFRVEYIYLRVKNIYIQYIINIVI
YWYGLNQVYILSFDKHLFFYLLLLLYCEINNYNIILHSTYLGILSYLAINDSNQILFSLVWNNDL
LIHWFFDSKKRVNNFLDQKYYFLQKAYAFHFFDKQLLLIFGCLLVLTITKFFQFLYKFKEYKVIH
YNILTLSMRHNQVTLVTCEILVHYLFVYKLLPVLHLCIIVLVWKLLTQSIEMLITGDFVIYIVIQL
LLSFSCIYTEEVYITDHIELLVLFELLVLLYYWLSVSVMFYLMDRCHYEVLQLLLILLVLYLEGKI
LLNSFEVVFLLIMPLTDFLHYILYCLLYLLFCTLHFMILLVQAILLVFQVITIELHLLHIFYLKILLF
LYLFLYVLLYSLCLMFGIVIIILWLILCKLLLLLYLNDTYYLSMLFDLYLINYVLRCLVLFLLCYY
LLQIVDLEVYNLDHLVKLSEFLLLISFCNVLNTLKIHLYYVNVLYYTLVILLLYYLLVTIIVLIYLI
NLNYFIKLTKYIKIIIYIFYLRYYFSILGFQFIIYIILCITLHLALANLLFPLVWNNDLLIHWFFESKK
EIRNIFLLLLILFLKNIHFALPFSCIKMNDIKNKYLNYSYVILSYIATIIRSCINTCTCLISCSLCYSC
KKNYGYAKKIRSCCRLLWTIASICCLKTFIKRICSSYTIYCSFLFRSCNNFNFCIIRLRCYTLWSW
FRDKRHEFRYILHVSCVIFSYIRYSISWLKCEIRFSRFSKYSSINLINIKFSYINSNYDNRKFKFNCL
YISKSYLIYTTFISCVYNIFHRIYSDKSSFFSRGPANLVWSGYMSQIARKPFYFKNKRQLAGNLIP
NKYIIKLFIDTQFNIYIYLFIYMIKDIVNHRCESTKKKHMGTHLPLLGESELVSGFMTEHAAVVF
VFFFLAEYGSIVLMCILTSILFIGGYLLFEISYVFTVVNYIFFELFFIDVTFVEVQSLYTDFLNNSIIE
GLLYGFNLGLKSSLMIFTFIARASFPRIRFDQLMGFCTVLLPINFAIIILVPCVLYSFNLLPVNIPLF
LTHPPALLPQGYSTYEEGNRSRTILVNNYTYTKTIYHTRECNYTLFLYSTLLAYYYLYMIIILLNL
LGYINKKFVNFFHHFLIQNTNF

>YN690

ITILKNIKSYIKIIHLYSINLKGIFSILYLVLLLFSLLQVFLLLGSLLNRWYARLLIKVISFYLLQYLV
LEGFIIILKKCSFIHLTINTKKLKIILLMVKLLIETIKYMLNLIIQMLCLVLWQLFLLLHFYYSCLIK
NDVWVLYWYNLYLAINEYDIIYRVCICYSYFIFSHFYICSSSLSRKIYFRVWFPFFRAKNTIRYKI
LYFCFSLFTFRFRNIINFPFRSVCWYLWSFSYFNFYSYNNYRIYIIRKRSNRQQTKIIYTIERLPYR
VCWNRGFVKLRQKTKKPTKGLMGSYKKMITYIYSYYIFTITRIIIYIYIILLYIKIIIIIRYNYSILLY
KNIIYPIINFIFFLIIIFFYIMLTHLQMKRTKYCCYSSGYRKIFLYYLFLLGSFSVRGYMDGRRVFIL
MNSGFKLTLFMNLNYNYINVGYDRIMYSLRVKRDTPTYNTYWFRVEYIYLRVKNIYIQYIINIVI
YWYGLNQVYILSFDKHLFFYLLLLLYCEINNYNIILHSTYLGILSYLAINDSNQILFSLVWNNDL
LIHWFFDSKKRVNNFLDQKYYFLQKAYAFHFFDKQLLLIFGCLLVLTITKFFQFLYKFKEYKVIH
YNILTLSMRHNQVTLVTCEILVHYLFVYKLLPVLHLCIIVLVWKLLTQSIEMLITGDFVIYIVIQL
LLSFSCIYTEEVYITDHIELLVLFELLVLLYYWLSVSVMFYLMDRCHYEVLQLLLILLVLYLEGKI
LLNSFEVVFLLIMPLTDFLHYILYCLLYLLFCTLHFMILLVQAILLVFQVITIELHLLHIFYLKILLF

LYLFLYVLLYSLCLMFGIVIIILWLILCKLLLLLYLNDTYYLSMLFDLYLINYVLRCLVLFLLCYY
LLQIVDLEVYNLDHLVKLSEFLLLISFCNVLNTLKIHLYYVNVLYYTLVILLLYYLLVTIIVLIYLI
NLNYFIKLTKYIKIIIYIFYLRYYFSILGFQFIIYIILCITLHLALANLLFPLVWNNDLLIHWFFESKK
EIRNIFLLLLILFLKNIHFALPFSCIKMNDIKNKYLNYSYVILSYIATIIRSCINTCTCLISCSLCYSC
KKNYGYAKKIRSCCRLLWTIASICCLKTFIKRICSSYTIYCSFLFRSCNNFNFCIIRLRCYTLWSW
FRDKRHEFRYILHVSCVIFSYIRYSISWLKCEIRFSRFSKYSSINLINIKFSYINSNYDNRKFKFNCL
YISKSYLIYTTFISCVYNIFHRIYSDKSSFFSRGPANLVWSGYMSQIARKPFYFKNKRQLAGNLIP
NKYIIKLFIDTQFNIYIYLFIYMIKDIVNHRCESTKKKHMGTHLPLLGESELVSGFMTEHAAVVF
VFFFLAEYGSIVLMCILTSILFIGGYLLFEISYVFTVVNYIFFELFFIDVTFVEVQSLYTDFLNNSIIE
GLLYGFNLGLKSSLMIFTFIARASFPRIRFDQLMGFCTVLLPINFAIIILVPCVLYSFNLLPVNIPLF
LTHPPALLPQGYSTYEEGNRSRTILVNNYTYTKTIYHTRECNYTLFLYSTLLAYYYLYMIIILLNL
LGYINKKFVNFFHHFLIQNTNF
>YN694
ITILKNIKSYIKIIHLYSINLKGIFSILYLVLLLFSLLQVFLLLGSLLNRWYARLLIKVISFYLLQYLV
LEGFIIILKKCSFIHLTINTKKLKIILLMVKLLIETIKYMLNLIIQMLCLVLWQLFLLLHFYYSCLIK
NDVWVLYWYNLYLAINEYDIIYRVCICYSYFIFSHFYICSSSLSRKIYFRVWFPFFRAKNTIRYKI
LYFCFSLFTFRFRNIINFPFRSVCWYLWSFSYFNFYSYNNYRIYIIRKRSNRQQTKIIYTIERLPYR
VCWNRGFVKLRQKTKKPTKGLMGSYKKMITYIYSYYIFTITRIIIYIYIILLYIKIIIIIRYNYSILLY
KNIIYPIINFIFFLIIIFFYIMLTHLQMKRTKYCCYSSGYRKIFLYYLFLLGSFSVRGYMDGRRVFIL
MNSGFKLTLFMNLNYNYINVGYDRIMYSLRVKRDTPTYNTYWFRVEYIYLRVKNIYIQYIINIVI
YWYGLNQVYILSFDKHLFFYLLLLLYCEINNYNIILHSTYLGILSYLAINDSNQILFSLVWNNDL
LIHWFFDSKKRVNNFLDQKYYFLQKAYAFHFFDKQLLLIFGCLLVLTITKFFQFLYKFKEYKVIH
YNILTLSMRHNQVTLVTCEILVHYLFVYKLLPVLHLCIIVLVWKLLTQSIEMLITGDFVIYIVIQL
LLSFSCIYTEEVYITDHIELLVLFELLVLLYYWLSVSVMFYLMDRCHYEVLQLLLILLVLYLEGKI
LLNSFEVVFLLIMPLTDFLHYILYCLLYLLFCTLHFMILLVQAILLVFQVITIELHLLHIFYLKILLF
LYLFLYVLLYSLCLMFGIVIIILWLILCKLLLLLYLNDTYYLSMLFDLYLINYVLRCLVLFLLCYY
LLQIVDLEVYNLDHLVKLSEFLLLISFCNVLNTLKIHLYYVNVLYYTLVILLLYYLLVTIIVLIYLI
NLNYFIKLTKYIKIIIYIFYLRYYFSILGFQFIIYIILCITLHLALANLLFPLVWNNDLLIHWFFESKK
EIRNIFLLLLILFLKNIHFALPFSCIKMNDIKNKYLNYSYVILSYIATIIRSCINTCTCLISCSLCYSC
KKNYGYAKKIRSCCRLLWTIASICCLKTFIKRICSSYTIYCSFLFRSCNNFNFCIIRLRCYTLWSW
FRDKRHEFRYILHVSCVIFSYIRYSISWLKCEIRFSRFSKYSSINLINIKFSYINSNYDNRKFKFNCL
YISKSYLIYTTFISCVYNIFHRIYSDKSSFFSRGPANLVWSGYMSQIARKPFYFKNKRQLAGNLIP
NKYIIKLFIDTQFNIYIYLFIYMIKDIVNHRCESTKKKHMGTHLPLLGESELVSGFMTEHAAVVF
VFFFLAEYGSIVLMCILTSILFIGGYLLFEISYVFTVVNYIFFELFFIDVTFVEVQSLYTDFLNNSIIE
GLLYGFNLGLKSSLMIFTFIARASFPRIRFDQLMGFCTVLLPINFAIIILVPCVLYSFNLLPVNIPLF
LTHPPALLPQGYSTYEEGNRSRTILVNNYTYTKTIYHTRECNYTLFLYSTLLAYYYLYMIIILLNL
LGYINKKFVNFFHHFLIQNTNY
>YN714
ITILKNIKSYIKIIHLYSINLKGIFSILYLVLLLFSLLQVFLLLGSLLNRWYARLLIKVISFYLLQYLV
LEGFIIILKKCSFIHLTINTKKLKIILLMVKLLIETIKYMLNLIIQMLCLVLWQLFLLLHFYYSCLIK
NDVWVLYWYNLYLAINEYDIIYRVCICYSYFIFSHFYICSSSLSRKIYFRVWFPFFRAKNTIRYKI
LYFCFSLFTFRFRNIINFPFRSVCWYLWSFSYFNFYSYNNYRIYIIRKRSNRQQTKIIYTIERLPYR
VCWNRGFVKLRQKTKKPTKGLMGSYKKMITYIYSYYIFTITRIIIYIYIILLYIKIIIIIRYNYSILLY
KNIIYPIINFIFFLIIIFFYIMLTHLQMKRTKYCCYSSGYRKIFLYYLFLLGSFSVRGYMDGRRVFIL

MNSGFKLTLFMNLNYNYINVGYDRIMYSLRVKRDTPTYNTYWFRVEYIYLRVKNIYIQYIINIVI
YWYGLNQVVYILSFDKHLFFYLLLLLYCEINNYNIILHSTYLGILSYLAINDSNQILFSLVWNNDL
LIHWFFDSKKRVNNFLDQKYYFLQKAYAFHFFDKQLLLIFGCLLVLTITKFFQFLYKFKEYKVIH
YNILTLSMRHNQVTLVTCEILVHYLFVYKLLPVLHLCIIVLVWKLLTQSIEMLITGDFVIYIVIQL
LLSFSCIYTEEVYITDHIELLVLFELLVLLYYWLSVSVMFYLMDRCHYEVLQLLLILLVLYLEGKI
LLNSFEVVFLLIMPLTDFLHYILYCLLYLLFCTLHFMILLVQAILLVFQVITIELHLLHIFYLKILLF
LYLFLYVLLYSLCLMFGIVIIILWLILCKLLLLLYLNDTYYLSMLFDLYLINYVLRCLVLFLLCYY
LLQIVDLEVYNLDHLVKLSEFLLLISFCNVLNTLKIHLYYVNVLYYTLVILLLYYLLVTIIVLIYLI
NLNYFIKLTKYIKIIIYIFYLRYYFSILGFQFIIYIILCITLHLALANLLFPLVWNNDLLIHWFFESKK
EIRNIFLLLLILFLKNIHFALPFSCIKMNDIKNKYLNYSYVILSYIATIIRSCINTCTCLISCSLCYSC
KKNYGYAKKIRSCCRLLWTIASICCLKTFIKRICSSYTIYCSFLFRSCNNFNFCIIRLRCYTLWSW
FRDKRHEFRYILHVSCVIFSYIRYSISWLKCEIRFSRFSKYSSINLINIKFSYINSNYDNRKFKFNCL
YISKSYLIYTTFISCVYNIFHRIYSDKSSFFSRGPANLVWSGYMSQIARKPFYFKNKRQLAGNLIP
NKYIIKLFIDTQFNIYIYLFIYMIKDIVNHRCESTKKKHMGTHLPLLGESELVSGFMTEHAAVVF
VFFFLAEYGSIVLMCILTSILFIGGYLLFEISYVFTVVNYIFFELFFIDVTFVEVQSLYTDFLNNSIIE
GLLYGFNLGLKSSLMIFTFIARASFPRIRFDQLMGFCTVLLPINFAIIILVPCVLYSFNLLPVNIPLF
LTHPPALLPQGYSTYEEGNRSRTILVNNYTYTKTIYHTRECNYTLFLYSTLLGYYYLYMIIILLNL
LGYINKKFVNFFHHFLIQNTNF
>YN716
ITILKNIKSYIKIIHLYSINLKGIFSILYLVLLLFSLLQVFLLLGSLLNRWYARLLIKVISFYLLQYLV
LEGFIIILKKCSFIHLTINTKKLKIILLMVKLLIETIKYMLNLIIQMLCLVLWQLFLLLHFYYSCLIK
NDVWVLYWYNLYLAINEYDIIYRVCICYSYFIFSHFYICSSSLSRKIYFRVWFPFFRAKNTIRYKI
LYFCFSLFTFRFRNIINFPFRSVCWYLWSFSYFNFYSYNNYRIYIIRKRSNRQQTKIIYTIERLPYR
VCWNRGFVKLRQKTKKPTKGLMGSYKKMITYIYSYYIFTITRIIIYIYIILLYIKIIIIIRYNYSILLY
KNIIYPIINFIFFLIIIFFYIMLTHLQMKRTKYCCYSSGYRKIFLYYLFLLGSFSVRGYMDGRRVFIL
MNSGFKLTLFMNLNYNYINVGYDRIMYSLRVKRDTPTYNTYWFRVEYIYLRVKNIYIQYIINIVI
YWYGLNQVVYILSFDKHLFFYLLLLLYCEINNYNIILHSTYLGILSYLAINDSNQILFSLVWNNDL
LIHWFFDSKKRVNNFLDQKYYFLQKAYAFHFFDKQLLLIFGCLLVLTITKFFQFLYKFKEYKVIH
YNILTLSMRHNQVTLVTCEILVHYLFVYKLLPVLHLCIIVLVWKLLTQSIEMLITGDFVIYIVIQL
LLSFSCIYTEEVYITDHIELLVLFELLVLLYYWLSVSVMFYLMDRCHYEVLQLLLILLVLYLEGKI
LLNSFEVVFLLIMPLTDFLHYILYCLLYLLFCTLHFMILLVQAILLVFQVITIELHLLHIFYLKILLF
LYLFLYVLLYSLCLMFGIVIIILWLILCKLLLLLYLNDTYYLSMLFDLYLINYVLRCLVLFLLCYY
LLQIVDLEVYNLDHLVKLSEFLLLISFCNVLNTLKIHLYYVNVLYYTLVILLLYYLLVTIIVLIYLI
NLNYFIKLTKYIKIIIYIFYLRYYFSILGFQFIIYIILCITLHLALANLLFPLVWNNDLLIHWFFESKK
EIRNIFLLLLILFLKNIHFALPFSCIKMNDIKNKYLNYSYVILSYIATIIRSCINTCTCLISCSLCYSC
KKNYGYAKKIRSCCRLLWTIASICCLKTFIKRICSSYTIYCSFLFRSCNNFNFCIIRLRCYTLWSW
FRDKRHEFRYILHVSCVIFSYIRYSISWLKCEIRFSRFSKYSSINLINIKFSYINSNYDNRKFKFNCL
YISKSYLIYTTFISCVYNIFHRIYSDKSSFFSRGPANLVWSGYMSQIARKPFYFKNKRQLAGNLIP
NKYIIKLFIDTQFNIYIYLFIYMIKDIVNHRCESTKKKHMGTHLPLLGESELVSGFMTEHAAVVF
VFFFLAEYGSIVLMCILTSILFIGGYLLFEISYVFTVVNYIFFELFFIDVTFVEVQSLYTDFLNNSIIE
GLLYGFNLGLKSSLMIFTFIARASFPRIRFDQLMGFCTVLLPINFAIIILVPCVLYSFNLLPVNIPLF
LTHPPALLPQGYSTYEEGNRSRTILVNNYTYTKTIYHTRECNYTLFLYSTLLAYYYLYMIIILLNL
LGYINKKFVNFFHHFLIQNTNF
>YN718

ITILKNIKSYIKIIHLYSINLKGIFSILYLVLLLFSLLQVFLLLGSLLNRWYARLLIKVISFYLLQYLV
LEGFIIILKKCSFIHLTINTKKLKIILLMVKLLIETIKYMLNLIIQMLCLVLWQLFLLLHFYYSCLIK
NDVWVLYWYNLYLAINEYDIIYRVCICYSYFIFSHFYICSSSLSRKIYFRVWFPFFRAKNTIRYKI
LYFCFSLFTFRFRNIINFPFRSVCWYLWSFSYFNFYSYNNYRIYIIRKRSNRQQTKIIYTIERLPYR
VCWNRGFVKLRQKTKKPTKGLMGSYKKMITYIYSYYIFTITRIIIYIYIILLYIKIIIIIRYNYSILLY
KNIIYPIINFIFFLIIIFFYIMLTHLQMKRTKYCCYSSGYRKIFLYYLFLLGSFSVRGYMDGRRVFIL
MNSGFKLTLFMNLNYNYINVGYDRIMYSLRVKRDTPTYNTYWFRVEYIYLRVKNIYIQYIINIVI
YWYGLNQVYILSFDKHLFFYLLLLLYCEINNYNIILHSTYLGILSYLAINDSNQILFSLVWNNDL
LIHWFFDSKKRVNNFLDQKYYFLQKAYAFHFFDKQLLLIFGCLLVLTITKFFQFLYKFKEYKVIH
YNILTLSMRHNQVTLVTCEILVHYLFVYKLLPVLHLCIIVLVWKLLTQSIEMLITGDFVIYIVIQL
LLSFSCIYTEEVYITDHIELLVLFELLVLLYYWLSVSVMFYLMDRCHYEVLQLLLILLVLYLEGKI
LLNSFEVVFLLIMPLTDFLHYILYCLLYLLFCTLHFMILLVQAILLVFQVITIELHLLHIFYLKILLF
LYLFLYVLLYSLCLMFGIVIIILWLILCKLLLLLYLNDTYYLSMLFDLYLINYVLRCLVLFLLCYY
LLQIVDLEVYNLDHLVKLSEFLLLISFCNVLNTLKIHLYYVNVLYYTLVILLLYYLLVTIIVLIYLI
NLNYFIKLTKYIKIIIYIFYLRYYFSILGFQFIIYIILCITLHLALANLLFPLVWNNDLLIHWFFESKK
EIRNIFLLLLILFLKNIHFALPFSCIKMNDIKNKYLNYSYVILSYIATIIRSCINTCTCLISCSLCYSC
KKNYGYAKKIRSCCRLLWTIASICCLKTFIKRICSSYTIYCSFLFRSCNNFNFCIIRLRCYTLWSW
FRDKRHEFRYILHVSCVIFSYIRYSISWLKCEIRFSRFSKYSSINLINIKFSYINSNYDNRKFKFNCL
YISKSYLIYTTFISCVYNIFHRIYSDKSSFFSRGPANLVWSGYMSQIARKPFYFKNKRQLAGNLIP
NKYIIKLFIDTQFNIYIYLFIYMIKDIVNHRCESTKKKHMGTHLPLLGESELVSGFMTEHAAVVF
VFFFLAEYGSIVLMCILTSILFIGGYLLFEISYVFTVVNYIFFELFFIDVTFVEVQSLYTDFLNNSIIE
GLLYGFNLGLKSSLMIFTFIARASFPRIRFDQLMGFCTVLLPINFAIIILVPCVLYSFNLLPVNIPLF
LTHPPALLPQGYSTYEEGNRSRTILVNNYTYTKTIYHTRECNYTLFLYSTLLAYYYLYMIIILLNL
LGYINKKFVNFFHHFLIQNTNF
>YN735
ITILKNIKSYIKIIHLYSINLKGIFSILYLVLLLFSLLQVFLLLGSLLNRWYARLLIKVISFYLLQYLV
LEGFIIILKKCSFIHLTINTKKLKIILLMVKLLIETIKYMLNLIIQMLCLVLWQLFLLLHFYYSCLIK
NDVWVLYWYNLYLAINEYDIIYRVCICYSYFIFSHFYICSSSLSRKIYFRVWFPFFRAKNTIRYKI
LYFCFSLFTFRFRNIINFPFRSVCWYLWSFSYFNFYSYNNYRIYIIRKRSNRQQTKIIYTIERLPYR
VCWNRGFVKLRQKTKKPTKGLMGSYKKMITYIYSYYIFTITRIIIYIYIILLYIKIIIIIRYNYSILLY
KNIIYPIINFIFFLIIIFFYIMLTHLQMKRTKYCCYSSGYRKIFLYYLFLLGSFSVRGYMDGRRVFIL
MNSGFKLTLFMNLNYNYINVGYDRIMYSLRVKRDTPTYNTYWFRVEYIYLRVKNIYIQYIINIVI
YWYGLNQVYILSFDKHLFFYLLLLLYCEINNYNIILHSTYLGILSYLAINDSNQILFSLVWNNDL
LIHWFFDSKKRVNNFLDQKYYFLQKAYAFHFFDKQLLLIFGCLLVLTITKFFQFLYKFKEYKVIH
YNILTLSMRHNQVTLVTCEILVHYLFVYKLLPVLHLCIIVLVWKLLTQSIEMLITGDFVIYIVIQL
LLSFSCIYTEEVYITDHIELLVLFELLVLLYYWLSVSVMFYLMDRCHYEVLQLLLILLVLYLEGKI
LLNSFEVVFLLIMPLTDFLHYILYCLLYLLFCTLHFMILLVQAILLVFQVITIELHLLHIFYLKILLF
LYLFLYVLLYSLCLMFGIVIIILWLILCKLLLLLYLNDTYYLSMLFDLYLINYVLRCLVLFLLCYY
LLQIVDLEVYNLDHLVKLSEFLLLISFCNVLNTLKIHLYYVNVLYYTLVILLLYYLLVTIIVLIYLI
NLNYFIKLTKYIKIIIYIFYLRYYFSILGFQFIIYIILCITLHLALANLLFPLVWNNDLLIHWFFESKK
EIRNIFLLLLILFLKNIHFALPFSCIKMNDIKNKYLNYSYVILSYIATIIRSCINTCTCLISCSLCYSC
KKNYGYAKKIRSCCRLLWTIASICCLKTFIKRICSSYTIYCSFLFRSCNNFNFCIIRLRCYTLWSW
FRDKRHEFRYILHVSCVIFSYIRYSISWLKCEIRFSRFSKYSSINLINIKFSYINSNYDNRKFKFNCL
YISKSYLIYTTFISCVYNIFHRIYSDKSSFFSRGPANLVWSGYMSQIARKPFYFKNKRQLAGNLIP

NKYIIKLFIDTQFNIYIYLFIYMIKDIVNHRCESTKKKHMGTHLPLLGESELVSGFMTEHAAVVF
VFFFLAEYGSIVLMCILTSILFIGGYLLFEISYVFTVVNYIFFELFFIDVTFVEVQSLYTDFLNNSIIE
GLLYGFNLGLKSSLMIFTFIARASFPRIRFDQLMGFCTVLLPINFAIIILVPCVLYSFNLLPVNIPLF
LTHPPALLPQGYSTYEEGNRSRTILVNNYTYTKTIYHTRECNYTLFLYSTLLAYYYLYMIIILLNL
LGYINKKFVNFFHHFLIQNTN
>YN745
ITILKNIKSYIKIIHLYSINLKGIFSILYLVLLLFSLLQVFLLLGSLLNRWYARLLIKVISFYLLQYLV
LEGFIIILKKCSFIHLTINTKKLKIILLMVKLLIETIKYMLNLIIQMLCLVLWQLFLLLHFYYSCLIK
NDVWVLYWYNLYLAINEYDIIYRVCICYSYFIFSHFYICSSSLSRKIYFRVWFPFFRAKNTIRYKI
LYFCFSLFTFRFRNIINFPFRSVCWYLWSFSYFNFYSYNNYRIYIIRKRSNRQQTKIIYTIERLPYR
VCWNRGFVKLRQKTKKPTKGLMGSYKKMITYIYSYYIFTITRIIIYIYIILLYIKIIIIIRYNYSILLY
KNIIYPIINFIFFLIIIFFYIMLTHLQMKRTKYCCYSSGYRKIFLYYLFLLGSFSVRGYMDGRRVFIL
MNSGFKLTLFMNLNYNYINVGYDRIMYSLRVKRDTPTYNTYWFRVEYIYLRVKNIYIQYIINIVI
YWYGLNQVVYILSFDKHLFFYLLLLLYCEINNYNIILHSTYLGILSYLAINDSNQILFSLVWNNDL
LIHWFFDSKKRVNNFLDQKYYFLQKAYAFHFFDKQLLLIFGCLLVLTITKFFQFLYKFKEYKVIH
YNILTLSMRHNQVTLVTCEILVHYLFVYKLLPVLHLCIIVLVWKLLTQSIEMLITGDFVIYIVIQL
LLSFSCIYTEEVYITDHIELLVLFELLVLLYYWLSVSVMFYLMDRCHYEVLQLLLILLVLYLEGKI
LLNSFEVVFLLIMPLTDFLHYILYCLLYLLFCTLHFMILLVQAILLVFQVITIELHLLHIFYLKILLF
LYLFLYVLLYSLCLMFGIVIIILWLILCKLLLLLYLNDTYYLSMLFDLYLINYVLRCLVLFLLCYY
LLQIVDLEVYNLDHLVKLSEFLLLISFCNVLNTLKIHLYYVNVLYYTLVILLLYYLLVTIIVLIYLI
NLNYFIKLTKYIKIIIYIFYLRYYFSILGFQFIIYIILCITLHLALANLLFPLVWNNDLLIHWFFESKK
EIRNIFLLLLILFLKNIHFALPFSCIKMNDIKNKYLNYSYVILSYIATIIRSCINTCTCLISCSLCYSC
KKNYGYAKKIRSCCRLLWTIASICCLKTFIKRICSSYTIYCSFLFRSCNNFNFCIIRLRCYTLWSW
FRDKRHEFRYILHVSCVIFSYIRYSISWLKCEIRFSRFSKYSSINLINIKFSYINSNYDNRKFKFNCL
YISKSYLIYTTFISCVYNIFHRIYSDKSSFFSRGPANLVWSGYMSQIARKPFYFKNKRQLAGNLIP
NKYIIKLFIDTQFNIYIYLFIYMIKDIVNHRCESTKKKHMGTHLPLLGESELVSGFMTEHAAVVF
VFFFLAEYGSIVLMCILTSILFIGGYLLFEISYVFTVVNYIFFELFFIDVTFVEVQSLYTDFLNNSIIE
GLLYGFNLGLKSSLMIFTFIARASFPRIRFDQLMGFCTVLLPINFAIIILVPCVLYSFNLLPVNIPLF
LTHPPALLPQGYSTYEEGNRSRTILVNNYTYTKTIYHTRECNYTLFLYSTLLAYYYLYMIIILLNL
LGYINKKFVNFFHHFLIQNTNF
>Z-2
ITILKNIKSYIKIIHLYSINLKGIFSILYLVLLLFSLLQVFLLLGSLLNRWYARLLIKVISFYLLQYLV
LEGFIIILKKCSFIHLTINTKKLKIILLMVKLLIETIKYMLNLIIQMLCLVLWQLFLLLHFYYSCLIK
NDVWVLYWYNLYLAINEYDIIYRVCICYSYFIFSHFYICSSSLSRKIYFRVWFPFFRAKNTIRYKI
LYFCFSLFTFRFRNIINFPFRSVCWYLWSFSYFNFYSYNNYRIYIIRKRSNRQQTKIIYTIERLPYR
VCWNRGFVKLRQKTKKPTKGLMGSYKKMITYIYSYYIFTITRIIIYIYIILLYIKIIIIIRYNYSILLY
KNIIYPIINFIFFLIIIFFYIMLTHLQMKRTKYCCYSSGYRKIFLYYLFLLGSFSVRGYMDGRRVFIL
MNSGFKLTLFMNLNYNYINVGYDRIMYSLRVKRDTPTYNTYWFRVEYIYLRVKNIYIQYIINIVI
YWYGLNQVVYILSFDKHLFFYLLLLLYCEINNYNIILHSTYLGILSYLAINDSNQILFSLVWNNDL
LIHWFFDSKKRVNNFLDQKYYFLQKAYAFHFFDKQLLLIFGCLLVLTITKFFQFLYKFKEYKVIH
YNILTLSMRHNQVTLVTCEILVHYLFVYKLLPVLHLCIIVLVWKLLTQSIEMLITGDFVIYIVIQL
LLSFSCIYTEEVYITDHIELLVLFELLVLLYYWLSVSVMFYLMDRCHYEVLQLLLILLVLYLEGKI
LLNSFEVVFLLIMPLTDFLHYILYCLLYLLFCTLHFMILLVQAILLVFQVITIELHLLHIFYLKILLF
LYLFLYVLLYSLCLMFGIVIIILWLILCKLLLLLYLNDTYYLSMLFDLYLINYVLRCLVLFLLCYY

LLQIVDLEVYNLDHLVKLSEFLLLISFCNVLNTLKIHLYYVNVLYYTLVILLLYYLLVTIIVLIYLI
NLNYFIKLTKYIKIIIYIFYLRYYFSILGFQFIIYIILCITLHLALANLLFPLVWNNDLLIHWFFESKK
EIRNIFLLLLILFLKNIHFALPFSCIKMNDIKNKYLNYSYVILSYIATIIRSCINTCTCLISCSLCYSC
KKNYGYAKKIRSCCRLLWTIASICCLKTFIKRICSSYTIYCSFLFRSCNNFNFCIIRLRCYTLWSW
FRDKRHEFRYILHVSCVIFSYIRYSISWLKCEIRFSRFSKYSSINLINIKFSYINSNYDNRKFKFNCL
YISKSYLIYTTFISCVYNIFHRIYSDKSSFFSRGPANLVWSGYMSQIARKPFYFKNKRQLAGNLIP
NKYIIKLFIDTQFNIYIYLFIYMIKDIVNHRCESTKKKHMGTHLPLLGESELVSGFMTEHAAVVF
VFFFLAEYGSIVLMCILTSILFIGGYLLFEISYVFTVVNYIFFELFFIDVTFVEVQSLYTDFLNNSIIE
GLLYGFNLGLKSSLMIFTFIARASFPRIRFDQLMGFCTVLLPINFAIIILVPCVLYSFNLLPVNIPLF
LTHPPALLPQGYSTYEEGNRSRTILVNNYTYTKTIYHTRECNYTLFLYSTLLAYYYLYMIIILLNL
LGYINKKFVNFFHHFLIQNTNF
