# Supplementary material for: ABCC Transporter Gene MoABC-R1 Is Associated with Pyraclostrobin Tolerance in Magnaporthe oryzae
Source: J Fungi (Basel). 2023 Sep 11;9(9):917. doi: 10.3390/jof9090917 (PMC10532721; doi:10.3390/jof9090917)
Supplement: Supplementary file 1 [file jof-09-00917-s001.zip › Supplementary data S3_Protein sequence aligment of gene MGG_04899 in 90 strains.pdf]

```
         1        10        20        30        40        50        60
1_1896   MVREDVQDSHLEGSSNVSACTSTFHLEDSSFESIDKEFLYSHTMPGNKHDSMQPHRRPES
2_4785   MVREDVQDSHLEGSSNVSACTSTFHLEDSSFESIDKEFLYSHTMPGNKHDSMQPHRRPES
3_3653   MVREDVQDSHLEGSSNVSACTSTFHLEDSSFESIDKEFLYSHTMPGNKHDSMQPHRRPES
4_8157   MVREDVQDSHLEGSSNVSACTSTFHLEDSSFESIDKEFLYSHTMPGNKHDSMQPHRRPES
5_752    MVREDVQDSHLEGSSNVSACTSTFHLEDSSFESIDKEFLYSHTMPGNKHDSMQPHRRPES
6_8853   MVREDVQDSHLEGSSNVSACTSTFHLEDSSFESIDKEFLYSHTMPGNKHDSMQPHRRPES
7_4015   MIVAFKYGPLMTLVITASVPIAMFIMRALSQMLEKAIRAQKEKQAVATKLSAAAITGIDL
8_2020   MVREDVQDSHLEGSSNVSACTSTFHLEDSSFESIDKEFLYSHTMPGNKHDSMQPHRRPES
9_1829   MVREDVQDSHLEGSSNVSACTSTFHLEDSSFESIDKEFLYSHTMPGNKHDSMQPHRRPES
10_6452  MVREDVQDSHLEGSSNVSACTSTFHLEDSSFESIDKEFLYSHTMPGNKHDSMQPHRRPES
11_8627  MVREDVQDSHLEGSSNVSACTSTFHLEDSSFESIDKEFLYSHTMPGNKHDSMQPHRRPES
12_6118  MVREDVQDSHLEGSSNVSACTSTFHLEDSSFESIDKEFLYSHTMPGNKHDSMQPHRRPES
13_2688  MVREDVQDSHLEGSSNVSACTSTFHLEDSSFESIDKEFLYSHTMPGNKHDSMQPHRRPES
14_6406  MVREDVQDSHLEGSSNVSACTSTFHLEDSSFESIDKEFLYSHTMPGNKHDSMQPHRRPES
15_9419  MVREDVQDSHLEGSSNVSACTSTFHLEDSSFESIDKEFLYSHTMPGNKHDSMQPHRRPES
16_8507  MVREDVQDSHLEGSSNVSACTSTFHLEDSSFESIDKEFLYSHTMPGNKHDSMQPHRRPES
17_5884  MVREDVQDSHLEGSSNVSACTSTFHLEDSSFESIDKEFLYSHTMPGNKHDSMQPHRRPES
18_2670  MVREDVQDSHLEGSSNVSACTSTFHLEDSSFESIDKEFLYSHTMPGNKHDSMQPHRRPES
19_5172  MVREDVQDSHLEGSSNVSACTSTFHLEDSSFESIDKEFLYSHTMPGNKHDSMQPHRRPES
20_8057  MVREDVQDSHLEGSSNVSACTSTFHLEDSSFESIDKEFLYSHTMPGNKHDSMQPHRRPES
21_4920  MTFKPGKMTFLVGRSGSGKSTITNLLVKFYEPLSGEILLDEHPLQTLNSEWVRKNVTLVQ
22_6393  MVREDVQDSHLEGSSNVSACTSTFHLEDSSFESIDKEFLYSHTMPGNKHDSMQPHRRPES
23_8263  MVREDVQDSHLEGSSNVSACTSTFHLEDSSFESIDKEFLYSHTMPGNKHDSMQPHRRPES
24_8248  MVREDVQDSHLEGSSNVSACTSTFHLEDSSFESIDKEFLYSHTMPGNKHDSMQPHRRPES
25_6733  MVREDVQDSHLEGSSNVSACTSTFHLEDSSFESIDKEFLYSHTMPGNKHDSMQPHRRPES
26_2277  MVREDVQDSHLEGSSNVSACTSTFHLEDSSFESIDKEFLYSHTMPGNKHDSMQPHRRPES
27_2623  MVREDVQDSHLEGSSNVSACTSTFHLEDSSFESIDKEFLYSHTMPGNKHDSMQPHRRPES
28_7413  MVREDVQDSHLEGSSNVSACTSTFHLEDSSFESIDKEFLYSHTMPGNKHDSMQPHRRPES
29_8980  MVREDVQDSHLEGSSNVSACTSTFHLEDSSFESIDKEFLYSHTMPGNKHDSMQPHRRPES
30_8706  MVREDVQDSHLEGSSNVSACTSTFHLEDSSFESIDKEFLYSHTMPGNKHDSMQPHRRPES
31_6378  MVREDVQDSHLEGSSNVSACTSTFHLEDSSFESIDKEFLYSHTMPGNKHDSMQPHRRPES
32_4679  MVREDVQDSHLEGSSNVSACTSTFHLEDSSFESIDKEFLYSHTMPGNKHDSMQPHRRPES
33_2437  MVREDVQDSHLEGSSNVSACTSTFHLEDSSFESIDKEFLYSHTMPGNKHDSMQPHRRPES
34_9700  MVREDVQDSHLEGSSNVSACTSTFHLEDSSFESIDKEFLYSHTMPGNKHDSMQPHRRPES
35_10218 MVREDVQDSHLEGSSNVSACTSTFHLEDSSFESIDKEFLYSHTMPGNKHDSMQPHRRPES
36_2652  MVREDVQDSHLEGSSNVSACTSTFHLEDSSFESIDKEFLYSHTMPGNKHDSMQPHRRPES
37_5779  MVREDVQDSHLEGSSNVSACTSTFHLEDSSFESIDKEFLYSHTMPGNKHDSMQPHRRPES
38_654   MVREDVQDSHLEGSSNVSACTSTFHLEDSSFESIDKEFLYSHTMPGNKHDSMQPHRRPES
39_4879  MVREDVQDSHLEGSSNVSACTSTFHLEDSSFESIDKEFLYSHTMPGNKHDSMQPHRRPES
40_8113  MVREDVQDSHLEGSSNVSACTSTFHLEDSSFESIDKEFLYSHTMPGNKHDSMQPHRRPES
41_1767  MVREDVQDSHLEGSSNVSACTSTFHLEDSSFESIDKEFLYSHTMPGNKHDSMQPHRRPES
42_8428  MVREDVQDSHLEGSSNVSACTSTFHLEDSSFESIDKEFLYSHTMPGNKHDSMQPHRRPES
43_9819  MVREDVQDSHLEGSSNVSACTSTFHLEDSSFESIDKEFLYSHTMPGNKHDSMQPHRRPES
44_5569  MVREDVQDSHLEGSSNVSACTSTFHLEDSSFESIDKEFLYSHTMPGNKHDSMQPHRRPES
45_4189  MVREDVQDSHLEGSSNVSACTSTFHLEDSSFESIDKEFLYSHTMPGNKHDSMQPHRRPES
46_1027  MVREDVQDSHLEGSSNVSACTSTFHLEDSSFESIDKEFLYSHTMPGNKHDSMQPHRRPES
47_5048  MVREDVQDSHLEGSSNVSACTSTFHLEDSSFESIDKEFLYSHTMPGNKHDSMQPHRRPES
48_10124 MVREDVQDSHLEGSSNVSACTSTFHLEDSSFESIDKEFLYSHTMPGNKHDSMQPHRRPES
49_7151  MVREDVQDSHLEGSSNVSACTSTFHLEDSSFESIDKEFLYSHTMPGNKHDSMQPHRRPES
50_3695  MVREDVQDSHLEGSSNVSACTSTFHLEDSSFESIDKEFLYSHTMPGNKHDSMQPHRRPES
51_364   MVREDVQDSHLEGSSNVSACTSTFHLEDSSFESIDKEFLYSHTMPGNKHDSMQPHRRPES
52_242   MVREDVQDSHLEGSSNVSACTSTFHLEDSSFESIDKEFLYSHTMPGNKHDSMQPHRRPES
53_5225  MVREDVQDSHLEGSSNVSACTSTFHLEDSSFESIDKEFLYSHTMPGNKHDSMQPHRRPES
54_1635  MVREDVQDSHLEGSSNVSACTSTFHLEDSSFESIDKEFLYSHTMPGNKHDSMQPHRRPES
55_5766  MVREDVQDSHLEGSSNVSACTSTFHLEDSSFESIDKEFLYSHTMPGNKHDSMQPHRRPES
56_1557  MVREDVQDSHLEGSSNVSACTSTFHLEDSSFESIDKEFLYSHTMPGNKHDSMQPHRRPES
57_10137 MVREDVQDSHLEGSSNVSACTSTFHLEDSSFESIDKEFLYSHTMPGNKHDSMQPHRRPES
58_10525 MVREDVQDSHLEGSSNVSACTSTFHLEDSSFESIDKEFLYSHTMPGNKHDSMQPHRRPES
59_3457  MVREDVQDSHLEGSSNVSACTSTFHLEDSSFESIDKEFLYSHTMPGNKHDSMQPHRRPES
60_8244  MVREDVQDSHLEGSSNVSACTSTFHLEDSSFESIDKEFLYSHTMPGNKHDSMQPHRRPES
61_7964  MVREDVQDSHLEGSSNVSACTSTFHLEDSSFESIDKEFLYSHTMPGNKHDSMQPHRRPES
62_4413  MVREDVQDSHLEGSSNVSACTSTFHLEDSSFESIDKEFLYSHTMPGNKHDSMQPHRRPES
63_6958  MVREDVQDSHLEGSSNVSACTSTFHLEDSSFESIDKEFLYSHTMPGNKHDSMQPHRRPES
64_5997  MNSENVSQDKVKAACEMALLQSTIAGLPQGLDTLVGPGGQSLSGGQRQRVALARARLRDT
65_8711  MVREDVQDSHLEGSSNVSACTSTFHLEDSSFESIDKEFLYSHTMPGNKHDSMQPHRRPES
66_9925  MVREDVQDSHLEGSSNVSACTSTFHLEDSSFESIDKEFLYSHTMPGNKHDSMQPHRRPES
67_10374 MVREDVQDSHLEGSSNVSACTSTFHLEDSSFESIDKEFLYSHTMPGNKHDSMQPHRRPES
68_3713  MVREDVQDSHLEGSSNVSACTSTFHLEDSSFESIDKEFLYSHTMPGNKHDSMQPHRRPES
69_4511  MVREDVQDSHLEGSSNVSACTSTFHLEDSSFESIDKEFLYSHTMPGNKHDSMQPHRRPES
70_6201  MVREDVQDSHLEGSSNVSACTSTFHLEDSSFESIDKEFLYSHTMPGNKHDSMQPHRRPES
71_3261  MVREDVQDSHLEGSSNVSACTSTFHLEDSSFESIDKEFLYSHTMPGNKHDSMQPHRRPES
72_8516  MVREDVQDSHLEGSSNVSACTSTFHLEDSSFESIDKEFLYSHTMPGNKHDSMQPHRRPES
73_9295  MVREDVQDSHLEGSSNVSACTSTFHLEDSSFESIDKEFLYSHTMPGNKHDSMQPHRRPES
74_809   MVREDVQDSHLEGSSNVSACTSTFHLEDSSFESIDKEFLYSHTMPGNKHDSMQPHRRPES
75_7282  MVREDVQDSHLEGSSNVSACTSTFHLEDSSFESIDKEFLYSHTMPGNKHDSMQPHRRPES
76_4910  MVREDVQDSHLEGSSNVSACTSTFHLEDSSFESIDKEFLYSHTMPGNKHDSMQPHRRPES
77_10912 MVREDVQDSHLEGSSNVSACTSTFHLEDSSFESIDKEFLYSHTMPGNKHDSMQPHRRPES
78_4020  MVREDVQDSHLEGSSNVSACTSTFHLEDSSFESIDKEFLYSHTMPGNKHDSMQPHRRPES
79_6703  MVREDVQDSHLEGSSNVSACTSTFHLEDSSFESIDKEFLYSHTMPGNKHDSMQPHRRPES
80_8619  MVREDVQDSHLEGSSNVSACTSTFHLEDSSFESIDKEFLYSHTMPGNKHDSMQPHRRPES
81_4126  MVREDVQDSHLEGSSNVSACTSTFHLEDSSFESIDKEFLYSHTMPGNKHDSMQPHRRPES
82_1363  MVREDVQDSHLEGSSNVSACTSTFHLEDSSFESIDKEFLYSHTMPGNKHDSMQPHRRPES
83_5813  MVREDVQDSHLEGSSNVSACTSTFHLEDSSFESIDKEFLYSHTMPGNKHDSMQPHRRPES
84_2044  MVREDVQDSHLEGSSNVSACTSTFHLEDSSFESIDKEFLYSHTMPGNKHDSMQPHRRPES
85_7617  MVREDVQDSHLEGSSNVSACTSTFHLEDSSFESIDKEFLYSHTMPGNKHDSMQPHRRPES
86_1530  MVREDVQDSHLEGSSNVSACTSTFHLEDSSFESIDKEFLYSHTMPGNKHDSMQPHRRPES
87_1890  MVREDVQDSHLEGSSNVSACTSTFHLEDSSFESIDKEFLYSHTMPGNKHDSMQPHRRPES
88_7049  MVREDVQDSHLEGSSNVSACTSTFHLEDSSFESIDKEFLYSHTMPGNKHDSMQPHRRPES
89_440   MVREDVQDSHLEGSSNVSACTSTFHLEDSSFESIDKEFLYSHTMPGNKHDSMQPHRRPES
90_4331  MVREDVQDSHLEGSSNVSACTSTFHLEDSSFESIDKEFLYSHTMPGNKHDSMQPHRRPES
```

```
                 70        80        90       100       110       120
1_1896    LYNMDQEKAPKRPDQSSSTLDQPKESVDSAAATWRDLFNFFNPRHLVVLVTALFASAVAV
2_4785    LYNMDQEKAPKRPDQSSSTLDQPKESVDSAAATWRDLFNFFNPRHLVVLVTALFASAVAV
3_3653    LYNMDQEKAPKRPDQSSSTLDQPKESVDSAAATWRDLFNFFNPRHLVVLVTALFASAVAV
4_8157    LYNMDQEKAPKRPDQSSSTLDQPKESVDSAAATWRDLFNFFNPRHLVVLVTALFASAVAV
5_752     LYNMDQEKAPKRPDQSSSTLDQPKESVDSAAATWRDLFNFFNPRHLVVLVTALFASAVAV
6_8853    LYNMDQEKAPKRPDQSSSTLDQPKESVDSAAATWRDLFNFFNPRHLVVLVTALFASAVAV
7_4015    VKVYNSHEHELHNFRLAAREAGKHARVQTMWNALQAAFIKIYMAVVFLSGFFFGIYQVDQ
8_2020    LYNMDQEKAPKRPDQSSSTLDQPKESVDSAAATWRDLFNFFNPRHLVVLVTALFASAVAV
9_1829    LYNMDQEKAPKRPDQSSSTLDQPKESVDSAAATWRDLFNFFNPRHLVVLVTALFASAVAV
10_6452   LYNMDQEKAPKRPDQSSSTLDQPKESVDSAAATWRDLFNFFNPRHLVVLVTALFASAVAV
11_8627   LYNMDQEKAPKRPDQSSSTLDQPKESVDSAAATWRDLFNFFNPRHLVVLVTALFASAVAV
12_6118   LYNMDQEKAPKRPDQSSSTLDQPKESVDSAAATWRDLFNFFNPRHLVVLVTALFASAVAV
13_2688   LYNMDQEKAPKRPDQSSSTLDQPKESVDSAAATWRDLFNFFNPRHLVVLVTALFASAVAV
14_6406   LYNMDQEKAPKRPDQSSSTLDQPKESVDSAAATWRDLFNFFNPRHLVVLVTALFASAVAV
15_9419   LYNMDQEKAPKRPDQSSSTLDQPKESVDSAAATWRDLFNFFNPRHLVVLVTALFASAVAV
16_8507   LYNMDQEKAPKRPDQSSSTLDQPKESVDSAAATWRDLFNFFNPRHLVVLVTALFASAVAV
17_5884   LYNMDQEKAPKRPDQSSSTLDQPKESVDSAAATWRDLFNFFNPRHLVVLVTALFASAVAV
18_2670   LYNMDQEKAPKRPDQSSSTLDQPKESVDSAAATWRDLFNFFNPRHLVVLVTALFASAVAV
19_5172   LYNMDQEKAPKRPDQSSSTLDQPKESVDSAAATWRDLFNFFNPRHLVVLVTALFASAVAV
20_8057   LYNMDQEKAPKRPDQSSSTLDQPKESVDSAAATWRDLFNFFNPRHLVVLVTALFASAVAV
21_4920   QQSTLFSDTLYANIAMNSENVSQDKVKAACEMALLQSTIAGLPQGLDTLVGPGGQSLSGG
22_6393   LYNMDQEKAPKRPDQSSSTLDQPKESVDSAAATWRDLFNFFNPRHLVVLVTALFASAVAV
23_8263   LYNMDQEKAPKRPDQSSSTLDQPKESVDSAAATWRDLFNFFNPRHLVVLVTALFASAVAV
24_8248   LYNMDQEKAPKRPDQSSSTLDQPKESVDSAAATWRDLFNFFNPRHLVVLVTALFASAVAV
25_6733   LYNMDQEKAPKRPDQSSSTLDQPKESVDSAAATWRDLFNFFNPRHLVVLVTALFASAVAV
26_2277   LYNMDQEKAPKRPDQSSSTLDQPKESVDSAAATWRDLFNFFNPRHLVVLVTALFASAVAV
27_2623   LYNMDQEKAPKRPDQSSSTLDQPKESVDSAAATWRDLFNFFNPRHLVVLVTALFASAVAV
28_7413   LYNMDQEKAPKRPDQSSSTLDQPKESVDSAAATWRDLFNFFNPRHLVVLVTALFASAVAV
29_8980   LYNMDQEKAPKRPDQSSSTLDQPKESVDSAAATWRDLFNFFNPRHLVVLVTALFASAVAV
30_8706   LYNMDQEKAPKRPDQSSSTLDQPKESVDSAAATWRDLFNFFNPRHLVVLVTALFASAVAV
31_6378   LYNMDQEKAPKRPDQSSSTLDQPKESVDSAAATWRDLFNFFNPRHLVVLVTALFASAVAV
32_4679   LYNMDQEKAPKRPDQSSSTLDQPKESVDSAAATWRDLFNFFNPRHLVVLVTALFASAVAV
33_2437   LYNMDQEKAPKRPDQSSSTLDQPKESVDSAAATWRDLFNFFNPRHLVVLVTALFASAVAV
34_9700   LYNMDQEKAPKRPDQSSSTLDQPKESVDSAAATWRDLFNFFNPRHLVVLVTALFASAVAV
35_10218  LYNMDQEKAPKRPDQSSSTLDQPKESVDSAAATWRDLFNFFNPRHLVVLVTALFASAVAV
36_2652   LYNMDQEKAPKRPDQSSSTLDQPKESVDSAAATWRDLFNFFNPRHLVVLVTALFASAVAV
37_5779   LYNMDQEKAPKRPDQSSSTLDQPKESVDSAAATWRDLFNFFNPRHLVVLVTALFASAVAV
38_654    LYNMDQEKAPKRPDQSSSTLDQPKESVDSAAATWRDLFNFFNPRHLVVLVTALFASAVAV
39_4879   LYNMDQEKAPKRPDQSSSTLDQPKESVDSAAATWRDLFNFFNPRHLVVLVTALFASAVAV
40_8113   LYNMDQEKAPKRPDQSSSTLDQPKESVDSAAATWRDLFNFFNPRHLVVLVTALFASAVAV
41_1767   LYNMDQEKAPKRPDQSSSTLDQPKESVDSAAATWRDLFNFFNPRHLVVLVTALFASAVAV
42_8428   LYNMDQEKAPKRPDQSSSTLDQPKESVDSAAATWRDLFNFFNPRHLVVLVTALFASAVAV
43_9819   LYNMDQEKAPKRPDQSSSTLDQPKESVDSAAATWRDLFNFFNPRHLVVLVTALFASAVAV
44_5569   LYNMDQEKAPKRPDQSSSTLDQPKESVDSAAATWRDLFNFFNPRHLVVLVTALFASAVAV
45_4189   LYNMDQEKAPKRPDQSSSTLDQPKESVDSAAATWRDLFNFFNPRHLVVLVTALFASAVAV
46_1027   LYNMDQEKAPKRPDQSSSTLDQPKESVDSAAATWRDLFNFFNPRHLVVLVTALFASAVAV
47_5048   LYNMDQEKAPKRPDQSSSTLDQPKESVDSAAATWRDLFNFFNPRHLVVLVTALFASAVAV
48_10124  LYNMDQEKAPKRPDQSSSTLDQPKESVDSAAATWRDLFNFFNPRHLVVLVTALFASAVAV
49_7151   LYNMDQEKAPKRPDQSSSTLDQPKESVDSAAATWRDLFNFFNPRHLVVLVTALFASAVAV
50_3695   LYNMDQEKAPKRPDQSSSTLDQPKESVDSAAATWRDLFNFFNPRHLVVLVTALFASAVAV
51_364    LYNMDQEKAPKRPDQSSSTLDQPKESVDSAAATWRDLFNFFNPRHLVVLVTALFASAVAV
52_242    LYNMDQEKAPKRPDQSSSTLDQPKESVDSAAATWRDLFNFFNPRHLVVLVTALFASAVAV
53_5225   LYNMDQEKAPKRPDQSSSTLDQPKESVDSAAATWRDLFNFFNPRHLVVLVTALFASAVAV
54_1635   LYNMDQEKAPKRPDQSSSTLDQPKESVDSAAATWRDLFNFFNPRHLVVLVTALFASAVAV
55_5766   LYNMDQEKAPKRPDQSSSTLDQPKESVDSAAATWRDLFNFFNPRHLVVLVTALFASAVAV
56_1557   LYNMDQEKAPKRPDQSSSTLDQPKESVDSAAATWRDLFNFFNPRHLVVLVTALFASAVAV
57_10137  LYNMDQEKAPKRPDQSSSTLDQPKESVDSAAATWRDLFNFFNPRHLVVLVTALFASAVAV
58_10525  LYNMDQEKAPKRPDQSSSTLDQPKESVDSAAATWRDLFNFFNPRHLVVLVTALFASAVAV
59_3457   LYNMDQEKAPKRPDQSSSTLDQPKESVDSAAATWRDLFNFFNPRHLVVLVTALFASAVAV
60_8244   LYNMDQEKAPKRPDQSSSTLDQPKESVDSAAATWRDLFNFFNPRHLVVLVTALFASAVAV
61_7964   LYNMDQEKAPKRPDQSSSTLDQPKESVDSAAATWRDLFNFFNPRHLVVLVTALFASAVAV
62_4413   LYNMDQEKAPKRPDQSSSTLDQPKESVDSAAATWRDLFNFFNPRHLVVLVTALFASAVAV
63_6958   LYNMDQEKAPKRPDQSSSTLDQPKESVDSAAATWRDLFNFFNPRHLVVLVTALFASAVAV
64_5997   PVLILDEVTSGLDPKSKLMIMDAIRYWRSGRTTIVITHDVSQVGDDDYVYVMDKAEVREE
65_8711   LYNMDQEKAPKRPDQSSSTLDQPKESVDSAAATWRDLFNFFNPRHLVVLVTALFASAVAV
66_9925   LYNMDQEKAPKRPDQSSSTLDQPKESVDSAAATWRDLFNFFNPRHLVVLVTALFASAVAV
67_10374  LYNMDQEKAPKRPDQSSSTLDQPKESVDSAAATWRDLFNFFNPRHLVVLVTALFASAVAV
68_3713   LYNMDQEKAPKRPDQSSSTLDQPKESVDSAAATWRDLFNFFNPRHLVVLVTALFASAVAV
69_4511   LYNMDQEKAPKRPDQSSSTLDQPKESVDSAAATWRDLFNFFNPRHLVVLVTALFASAVAV
70_6201   LYNMDQEKAPKRPDQSSSTLDQPKESVDSAAATWRDLFNFFNPRHLVVLVTALFASAVAV
71_3261   LYNMDQEKAPKRPDQSSSTLDQPKESVDSAAATWRDLFNFFNPRHLVVLVTALFASAVAV
72_8516   LYNMDQEKAPKRPDQSSSTLDQPKESVDSAAATWRDLFNFFNPRHLVVLVTALFASAVAV
73_9295   LYNMDQEKAPKRPDQSSSTLDQPKESVDSAAATWRDLFNFFNPRHLVVLVTALFASAVAV
74_809    LYNMDQEKAPKRPDQSSSTLDQPKESVDSAAATWRDLFNFFNPRHLVVLVTALFASAVAV
75_7282   LYNMDQEKAPKRPDQSSSTLDQPKESVDSAAATWRDLFNFFNPRHLVVLVTALFASAVAV
76_4910   LYNMDQEKAPKRPDQSSSTLDQPKESVDSAAATWRDLFNFFNPRHLVVLVTALFASAVAV
77_10912  LYNMDQEKAPKRPDQSSSTLDQPKESVDSAAATWRDLFNFFNPRHLVVLVTALFASAVAV
78_4020   LYNMDQEKAPKRPDQSSSTLDQPKESVDSAAATWRDLFNFFNPRHLVVLVTALFASAVAV
79_6703   LYNMDQEKAPKRPDQSSSTLDQPKESVDSAAATWRDLFNFFNPRHLVVLVTALFASAVAV
80_8619   LYNMDQEKAPKRPDQSSSTLDQPKESVDSAAATWRDLFNFFNPRHLVVLVTALFASAVAV
81_4126   LYNMDQEKAPKRPDQSSSTLDQPKESVDSAAATWRDLFNFFNPRHLVVLVTALFASAVAV
82_1363   LYNMDQEKAPKRPDQSSSTLDQPKESVDSAAATWRDLFNFFNPRHLVVLVTALFASAVAV
83_5813   LYNMDQEKAPKRPDQSSSTLDQPKESVDSAAATWRDLFNFFNPRHLVVLVTALFASAVAV
84_2044   LYNMDQEKAPKRPDQSSSTLDQPKESVDSAAATWRDLFNFFNPRHLVVLVTALFASAVAV
85_7617   LYNMDQEKAPKRPDQSSSTLDQPKESVDSAAATWRDLFNFFNPRHLVVLVTALFASAVAV
86_1530   LYNMDQEKAPKRPDQSSSTLDQPKESVDSAAATWRDLFNFFNPRHLVVLVTALFASAVAV
87_1890   LYNMDQEKAPKRPDQSSSTLDQPKESVDSAAATWRDLFNFFNPRHLVVLVTALFASAVAV
88_7049   LYNMDQEKAPKRPDQSSSTLDQPKESVDSAAATWRDLFNFFNPRHLVVLVTALFASAVAV
89_440    LYNMDQEKAPKRPDQSSSTLDQPKESVDSAAATWRDLFNFFNPRHLVVLVTALFASAVAV
90_4331   LYNMDQEKAPKRPDQSSSTLDQPKESVDSAAATWRDLFNFFNPRHLVVLVTALFASAVAV
```

```
                 130       140       150       160       170       180
1_1896      GADIAGVIIFGNVFQELTSYAARQLGPEEARRNVNFWVSIIAALAVTHLIAAATQMGSWI
2_4785      GADIAGVIIFGNVFQELTSYAARQLGPEEARRNVNFWVSIIAALAVTHLIAAATQMGSWI
3_3653      GADIAGVIIFGNVFQELTSYAARQLGPEEARRNVNFWVSIIAALAVTHLIAAATQMGSWI
4_8157      GADIAGVIIFGNVFQELTSYAARQLGPEEARRNVNFWVSIIAALAVTHLIAAATQMGSWI
5_752       GADIAGVIIFGNVFQELTSYAARQLGPEEARRNVNFWVSIIAALAVTHLIAAATQMGSWI
6_8853      GADIAGVIIFGNVFQELTSYAARQLGPEEARRNVNFWVSIIAALAVTHLIAAATQMGSWI
7_4015      GNMSAGDVITTFYAALSTFQGVQGLGPHILNLVRGMQAGKALKSVVFDRVVEMGGGRKPR
8_2020      GADIAGVIIFGNVFQELTSYAARQLGPEEARRNVNFWVSIIAALAVTHLIAAATQMGSWI
9_1829      GADIAGVIIFGNVFQELTSYAARQLGPEEARRNVNFWVSIIAALAVTHLIAAATQMGSWI
10_6452     GADIAGVIIFGNVFQELTSYAARQLGPEEARRNVNFWVSIIAALAVTHLIAAATQMGSWI
11_8627     GADIAGVIIFGNVFQELTSYAARQLGPEEARRNVNFWVSIIAALAVTHLIAAATQMGSWI
12_6118     GADIAGVIIFGNVFQELTSYAARQLGPEEARRNVNFWVSIIAALAVTHLIAAATQMGSWI
13_2688     GADIAGVIIFGNVFQELTSYAARQLGPEEARRNVNFWVSIIAALAVTHLIAAATQMGSWI
14_6406     GADIAGVIIFGNVFQELTSYAARQLGPEEARRNVNFWVSIIAALAVTHLIAAATQMGSWI
15_9419     GADIAGVIIFGNVFQELTSYAARQLGPEEARRNVNFWVSIIAALAVTHLIAAATQMGSWI
16_8507     GADIAGVIIFGNVFQELTSYAARQLGPEEARRNVNFWVSIIAALAVTHLIAAATQMGSWI
17_5884     GADIAGVIIFGNVFQELTSYAARQLGPEEARRNVNFWVSIIAALAVTHLIAAATQMGSWI
18_2670     GADIAGVIIFGNVFQELTSYAARQLGPEEARRNVNFWVSIIAALAVTHLIAAATQMGSWI
19_5172     GADIAGVIIFGNVFQELTSYAARQLGPEEARRNVNFWVSIIAALAVTHLIAAATQMGSWI
20_8057     GADIAGVIIFGNVFQELTSYAARQLGPEEARRNVNFWVSIIAALAVTHLIAAATQMGSWI
21_4920     QRQRVALARARLRDTPVLILDEVTSGLDPKSKLMIMDAIRYWRSGRTTIVITHDVSQVGD
22_6393     GADIAGVIIFGNVFQELTSYAARQLGPEEARRNVNFWVSIIAALAVTHLIAAATQMGSWI
23_8263     GADIAGVIIFGNVFQELTSYAARQLGPEEARRNVNFWVSIIAALAVTHLIAAATQMGSWI
24_8248     GADIAGVIIFGNVFQELTSYAARQLGPEEARRNVNFWVSIIAALAVTHLIAAATQMGSWI
25_6733     GADIAGVIIFGNVFQELTSYAARQLGPEEARRNVNFWVSIIAALAVTHLIAAATQMGSWI
26_2277     GADIAGVIIFGNVFQELTSYAARQLGPEEARRNVNFWVSIIAALAVTHLIAAATQMGSWI
27_2623     GADIAGVIIFGNVFQELTSYAARQLGPEEARRNVNFWVSIIAALAVTHLIAAATQMGSWI
28_7413     GADIAGVIIFGNVFQELTSYAARQLGPEEARRNVNFWVSIIAALAVTHLIAAATQMGSWI
29_8980     GADIAGVIIFGNVFQELTSYAARQLGPEEARRNVNFWVSIIAALAVTHLIAAATQMGSWI
30_8706     GADIAGVIIFGNVFQELTSYAARQLGPEEARRNVNFWVSIIAALAVTHLIAAATQMGSWI
31_6378     GADIAGVIIFGNVFQELTSYAARQLGPEEARRNVNFWVSIIAALAVTHLIAAATQMGSWI
32_4679     GADIAGVIIFGNVFQELTSYAARQLGPEEARRNVNFWVSIIAALAVTHLIAAATQMGSWI
33_2437     GADIAGVIIFGNVFQELTSYAARQLGPEEARRNVNFWVSIIAALAVTHLIAAATQMGSWI
34_9700     GADIAGVIIFGNVFQELTSYAARQLGPEEARRNVNFWVSIIAALAVTHLIAAATQMGSWI
35_10218    GADIAGVIIFGNVFQELTSYAARQLGPEEARRNVNFWVSIIAALAVTHLIAAATQMGSWI
36_2652     GADIAGVIIFGNVFQELTSYAARQLGPEEARRNVNFWVSIIAALAVTHLIAAATQMGSWI
37_5779     GADIAGVIIFGNVFQELTSYAARQLGPEEARRNVNFWVSIIAALAVTHLIAAATQMGSWI
38_654      GADIAGVIIFGNVFQELTSYAARQLGPEEARRNVNFWVSIIAALAVTHLIAAATQMGSWI
39_4879     GADIAGVIIFGNVFQELTSYAARQLGPEEARRNVNFWVSIIAALAVTHLIAAATQMGSWI
40_8113     GADIAGVIIFGNVFQELTSYAARQLGPEEARRNVNFWVSIIAALAVTHLIAAATQMGSWI
41_1767     GADIAGVIIFGNVFQELTSYAARQLGPEEARRNVNFWVSIIAALAVTHLIAAATQMGSWI
42_8428     GADIAGVIIFGNVFQELTSYAARQLGPEEARRNVNFWVSIIAALAVTHLIAAATQMGSWI
43_9819     GADIAGVIIFGNVFQELTSYAARQLGPEEARRNVNFWVSIIAALAVTHLIAAATQMGSWI
44_5569     GADIAGVIIFGNVFQELTSYAARQLGPEEARRNVNFWVSIIAALAVTHLIAAATQMGSWI
45_4189     GADIAGVIIFGNVFQELTSYAARQLGPEEARRNVNFWVSIIAALAVTHLIAAATQMGSWI
46_1027     GADIAGVIIFGNVFQELTSYAARQLGPEEARRNVNFWVSIIAALAVTHLIAAATQMGSWI
47_5048     GADIAGVIIFGNVFQELTSYAARQLGPEEARRNVNFWVSIIAALAVTHLIAAATQMGSWI
48_10124    GADIAGVIIFGNVFQELTSYAARQLGPEEARRNVNFWVSIIAALAVTHLIAAATQMGSWI
49_7151     GADIAGVIIFGNVFQELTSYAARQLGPEEARRNVNFWVSIIAALAVTHLIAAATQMGSWI
50_3695     GADIAGVIIFGNVFQELTSYAARQLGPEEARRNVNFWVSIIAALAVTHLIAAATQMGSWI
51_364      GADIAGVIIFGNVFQELTSYAARQLGPEEARRNVNFWVSIIAALAVTHLIAAATQMGSWI
52_242      GADIAGVIIFGNVFQELTSYAARQLGPEEARRNVNFWVSIIAALAVTHLIAAATQMGSWI
53_5225     GADIAGVIIFGNVFQELTSYAARQLGPEEARRNVNFWVSIIAALAVTHLIAAATQMGSWI
54_1635     GADIAGVIIFGNVFQELTSYAARQLGPEEARRNVNFWVSIIAALAVTHLIAAATQMGSWI
55_5766     GADIAGVIIFGNVFQELTSYAARQLGPEEARRNVNFWVSIIAALAVTHLIAAATQMGSWI
56_1557     GADIAGVIIFGNVFQELTSYAARQLGPEEARRNVNFWVSIIAALAVTHLIAAATQMGSWI
57_10137    GADIAGVIIFGNVFQELTSYAARQLGPEEARRNVNFWVSIIAALAVTHLIAAATQMGSWI
58_10525    GADIAGVIIFGNVFQELTSYAARQLGPEEARRNVNFWVSIIAALAVTHLIAAATQMGSWI
59_3457     GADIAGVIIFGNVFQELTSYAARQLGPEEARRNVNFWVSIIAALAVTHLIAAATQMGSWI
60_8244     GADIAGVIIFGNVFQELTSYAARQLGPEEARRNVNFWVSIIAALAVTHLIAAATQMGSWI
61_7964     GADIAGVIIFGNVFQELTSYAARQLGPEEARRNVNFWVSIIAALAVTHLIAAATQMGSWI
62_4413     GADIAGVIIFGNVFQELTSYAARQLGPEEARRNVNFWVSIIAALAVTHLIAAATQMGSWI
63_6958     GADIAGVIIFGNVFQELTSYAARQLGPEEARRNVNFWVSIIAALAVTHLIAAATQMGSWI
64_5997     GLCKQLLSSRDGYFLQLRALAESGADSNGPEGVMTPEDDLSSLSSSEDELDVDMSGKSFY
65_8711     GADIAGVIIFGNVFQELTSYAARQLGPEEARRNVNFWVSIIAALAVTHLIAAATQMGSWI
66_9925     GADIAGVIIFGNVFQELTSYAARQLGPEEARRNVNFWVSIIAALAVTHLIAAATQMGSWI
67_10374    GADIAGVIIFGNVFQELTSYAARQLGPEEARRNVNFWVSIIAALAVTHLIAAATQMGSWI
68_3713     GADIAGVIIFGNVFQELTSYAARQLGPEEARRNVNFWVSIIAALAVTHLIAAATQMGSWI
69_4511     GADIAGVIIFGNVFQELTSYAARQLGPEEARRNVNFWVSIIAALAVTHLIAAATQMGSWI
70_6201     GADIAGVIIFGNVFQELTSYAARQLGPEEARRNVNFWVSIIAALAVTHLIAAATQMGSWI
71_3261     GADIAGVIIFGNVFQELTSYAARQLGPEEARRNVNFWVSIIAALAVTHLIAAATQMGSWI
72_8516     GADIAGVIIFGNVFQELTSYAARQLGPEEARRNVNFWVSIIAALAVTHLIAAATQMGSWI
73_9295     GADIAGVIIFGNVFQELTSYAARQLGPEEARRNVNFWVSIIAALAVTHLIAAATQMGSWI
74_809      GADIAGVIIFGNVFQELTSYAARQLGPEEARRNVNFWVSIIAALAVTHLIAAATQMGSWI
75_7282     GADIAGVIIFGNVFQELTSYAARQLGPEEARRNVNFWVSIIAALAVTHLIAAATQMGSWI
76_4910     GADIAGVIIFGNVFQELTSYAARQLGPEEARRNVNFWVSIIAALAVTHLIAAATQMGSWI
77_10912    GADIAGVIIFGNVFQELTSYAARQLGPEEARRNVNFWVSIIAALAVTHLIAAATQMGSWI
78_4020     GADIAGVIIFGNVFQELTSYAARQLGPEEARRNVNFWVSIIAALAVTHLIAAATQMGSWI
79_6703     GADIAGVIIFGNVFQELTSYAARQLGPEEARRNVNFWVSIIAALAVTHLIAAATQMGSWI
80_8619     GADIAGVIIFGNVFQELTSYAARQLGPEEARRNVNFWVSIIAALAVTHLIAAATQMGSWI
81_4126     GADIAGVIIFGNVFQELTSYAARQLGPEEARRNVNFWVSIIAALAVTHLIAAATQMGSWI
82_1363     GADIAGVIIFGNVFQELTSYAARQLGPEEARRNVNFWVSIIAALAVTHLIAAATQMGSWI
83_5813     GADIAGVIIFGNVFQELTSYAARQLGPEEARRNVNFWVSIIAALAVTHLIAAATQMGSWI
84_2044     GADIAGVIIFGNVFQELTSYAARQLGPEEARRNVNFWVSIIAALAVTHLIAAATQMGSWI
85_7617     GADIAGVIIFGNVFQELTSYAARQLGPEEARRNVNFWVSIIAALAVTHLIAAATQMGSWI
86_1530     GADIAGVIIFGNVFQELTSYAARQLGPEEARRNVNFWVSIIAALAVTHLIAAATQMGSWI
87_1890     GADIAGVIIFGNVFQELTSYAARQLGPEEARRNVNFWVSIIAALAVTHLIAAATQMGSWI
88_7049     GADIAGVIIFGNVFQELTSYAARQLGPEEARRNVNFWVSIIAALAVTHLIAAATQMGSWI
89_440      GADIAGVIIFGNVFQELTSYAARQLGPEEARRNVNFWVSIIAALAVTHLIAAATQMGSWI
90_4331     GADIAGVIIFGNVFQELTSYAARQLGPEEARRNVNFWVSIIAALAVTHLIAAATQMGSWI
```

```
                    190       200       210       220       230       240
1_1896     KYGEMQARSARLELFRRMLSKQMGWFDSNQDGMPAMLTALQRNIRELQIATSQTLGFICH
2_4785     KYGEMQARSARLELFRRMLSKQMGWFDSNQDGMPAMLTALQRNIRELQIATSQTLGFICH
3_3653     KYGEMQARSARLELFRRMLSKQMGWFDSNQDGMPAMLTALQRNIRELQIATSQTLGFICH
4_8157     KYGEMQARSARLELFRRMLSKQMGWFDSNQDGMPAMLTALQRNIRELQIATSQTLGFICH
5_752      KYGEMQARSARLELFRRMLSKQMGWFDSNQDGMPAMLTALQRNIRELQIATSQTLGFICH
6_8853     KYGEMQARSARLELFRRMLSKQMGWFDSNQDGMPAMLTALQRNIRELQIATSQTLGFICH
7_4015     VFEGRVEVKSVSFAYPSSPAVQVLKQTSMTFKPGKMTFLVGRSGSGKSSITNLLVKFYEP
8_2020     KYGEMQARSARLELFRRMLSKQMGWFDSNQDGMPAMLTALQRNIRELQIATSQTLGFICH
9_1829     KYGEMQARSARLELFRRMLSKQMGWFDSNQDGMPAMLTALQRNIRELQIATSQTLGFICH
10_6452    KYGEMQARSARLELFRRMLSKQMGWFDSNQDGMPAMLTALQRNIRELQIATSQTLGFICH
11_8627    KYGEMQARSARLELFRRMLSKQMGWFDSNQDGMPAMLTALQRNIRELQIATSQTLGFICH
12_6118    KYGEMQARSARLELFRRMLSKQMGWFDSNQDGMPAMLTALQRNIRELQIATSQTLGFICH
13_2688    KYGEMQARSARLELFRRMLSKQMGWFDSNQDGMPAMLTALQRNIRELQIATSQTLGFICH
14_6406    KYGEMQARSARLELFRRMLSKQMGWFDSNQDGMPAMLTALQRNIRELQIATSQTLGFICH
15_9419    KYGEMQARSARLELFRRMLSKQMGWFDSNQDGMPAMLTALQRNIRELQIATSQTLGFICH
16_8507    KYGEMQARSARLELFRRMLSKQMGWFDSNQDGMPAMLTALQRNIRELQIATSQTLGFICH
17_5884    KYGEMQARSARLELFRRMLSKQMGWFDSNQDGMPAMLTALQRNIRELQIATSQTLGFICH
18_2670    KYGEMQARSARLELFRRMLSKQMGWFDSNQDGMPAMLTALQRNIRELQIATSQTLGFICH
19_5172    KYGEMQARSARLELFRRMLSKQMGWFDSNQDGMPAMLTALQRNIRELQIATSQTLGFICH
20_8057    KYGEMQARSARLELFRRMLSKQMGWFDSNQDGMPAMLTALQRNIRELQIATSQTLGFICH
21_4920    DDYVYVMDKAEVREEGLCKQLLSSRDGYFLQLRALAESGADSNGPEGVMTPEDDLSSLSS
22_6393    KYGEMQARSARLELFRRMLSKQMGWFDSNQDGMPAMLTALQRNIRELQIATSQTLGFICH
23_8263    KYGEMQARSARLELFRRMLSKQMGWFDSNQDGMPAMLTALQRNIRELQIATSQTLGFICH
24_8248    KYGEMQARSARLELFRRMLSKQMGWFDSNQDGMPAMLTALQRNIRELQIATSQTLGFICH
25_6733    KYGEMQARSARLELFRRMLSKQMGWFDSNQDGMPAMLTALQRNIRELQIATSQTLGFICH
26_2277    KYGEMQARSARLELFRRMLSKQMGWFDSNQDGMPAMLTALQRNIRELQIATSQTLGFICH
27_2623    KYGEMQARSARLELFRRMLSKQMGWFDSNQDGMPAMLTALQRNIRELQIATSQTLGFICH
28_7413    KYGEMQARSARLELFRRMLSKQMGWFDSNQDGMPAMLTALQRNIRELQIATSQTLGFICH
29_8980    KYGEMQARSARLELFRRMLSKQMGWFDSNQDGMPAMLTALQRNIRELQIATSQTLGFICH
30_8706    KYGEMQARSARLELFRRMLSKQMGWFDSNQDGMPAMLTALQRNIRELQIATSQTLGFICH
31_6378    KYGEMQARSARLELFRRMLSKQMGWFDSNQDGMPAMLTALQRNIRELQIATSQTLGFICH
32_4679    KYGEMQARSARLELFRRMLSKQMGWFDSNQDGMPAMLTALQRNIRELQIATSQTLGFICH
33_2437    KYGEMQARSARLELFRRMLSKQMGWFDSNQDGMPAMLTALQRNIRELQIATSQTLGFICH
34_9700    KYGEMQARSARLELFRRMLSKQMGWFDSNQDGMPAMLTALQRNIRELQIATSQTLGFICH
35_10218   KYGEMQARSARLELFRRMLSKQMGWFDSNQDGMPAMLTALQRNIRELQIATSQTLGFICH
36_2652    KYGEMQARSARLELFRRMLSKQMGWFDSNQDGMPAMLTALQRNIRELQIATSQTLGFICH
37_5779    KYGEMQARSARLELFRRMLSKQMGWFDSNQDGMPAMLTALQRNIRELQIATSQTLGFICH
38_654     KYGEMQARSARLELFRRMLSKQMGWFDSNQDGMPAMLTALQRNIRELQIATSQTLGFICH
39_4879    KYGEMQARSARLELFRRMLSKQMGWFDSNQDGMPAMLTALQRNIRELQIATSQTLGFICH
40_8113    KYGEMQARSARLELFRRMLSKQMGWFDSNQDGMPAMLTALQRNIRELQIATSQTLGFICH
41_1767    KYGEMQARSARLELFRRMLSKQMGWFDSNQDGMPAMLTALQRNIRELQIATSQTLGFICH
42_8428    KYGEMQARSARLELFRRMLSKQMGWFDSNQDGMPAMLTALQRNIRELQIATSQTLGFICH
43_9819    KYGEMQARSARLELFRRMLSKQMGWFDSNQDGMPAMLTALQRNIRELQIATSQTLGFICH
44_5569    KYGEMQARSARLELFRRMLSKQMGWFDSNQDGMPAMLTALQRNIRELQIATSQTLGFICH
45_4189    KYGEMQARSARLELFRRMLSKQMGWFDSNQDGMPAMLTALQRNIRELQIATSQTLGFICH
46_1027    KYGEMQARSARLELFRRMLSKQMGWFDSNQDGMPAMLTALQRNIRELQIATSQTLGFICH
47_5048    KYGEMQARSARLELFRRMLSKQMGWFDSNQDGMPAMLTALQRNIRELQIATSQTLGFICH
48_10124   KYGEMQARSARLELFRRMLSKQMGWFDSNQDGMPAMLTALQRNIRELQIATSQTLGFICH
49_7151    KYGEMQARSARLELFRRMLSKQMGWFDSNQDGMPAMLTALQRNIRELQIATSQTLGFICH
50_3695    KYGEMQARSARLELFRRMLSKQMGWFDSNQDGMPAMLTALQRNIRELQIATSQTLGFICH
51_364     KYGEMQARSARLELFRRMLSKQMGWFDSNQDGMPAMLTALQRNIRELQIATSQTLGFICH
52_242     KYGEMQARSARLELFRRMLSKQMGWFDSNQDGMPAMLTALQRNIRELQIATSQTLGFICH
53_5225    KYGEMQARSARLELFRRMLSKQMGWFDSNQDGMPAMLTALQRNIRELQIATSQTLGFICH
54_1635    KYGEMQARSARLELFRRMLSKQMGWFDSNQDGMPAMLTALQRNIRELQIATSQTLGFICH
55_5766    KYGEMQARSARLELFRRMLSKQMGWFDSNQDGMPAMLTALQRNIRELQIATSQTLGFICH
56_1557    KYGEMQARSARLELFRRMLSKQMGWFDSNQDGMPAMLTALQRNIRELQIATSQTLGFICH
57_10137   KYGEMQARSARLELFRRMLSKQMGWFDSNQDGMPAMLTALQRNIRELQIATSQTLGFICH
58_10525   KYGEMQARSARLELFRRMLSKQMGWFDSNQDGMPAMLTALQRNIRELQIATSQTLGFICH
59_3457    KYGEMQARSARLELFRRMLSKQMGWFDSNQDGMPAMLTALQRNIRELQIATSQTLGFICH
60_8244    KYGEMQARSARLELFRRMLSKQMGWFDSNQDGMPAMLTALQRNIRELQIATSQTLGFICH
61_7964    KYGEMQARSARLELFRRMLSKQMGWFDSNQDGMPAMLTALQRNIRELQIATSQTLGFICH
62_4413    KYGEMQARSARLELFRRMLSKQMGWFDSNQDGMPAMLTALQRNIRELQIATSQTLGFICH
63_6958    KYGEMQARSARLELFRRMLSKQMGWFDSNQDGMPAMLTALQRNIRELQIATSQTLGFICH
64_5997    IPEPKTTSSFGALGRMSFVPIPGTNGPIYPTPNQPGSGAYLQLHRSPRHRHREQGRPNNW
65_8711    KYGEMQARSARLELFRRMLSKQMGWFDSNQDGMPAMLTALQRNIRELQIATSQTLGFICH
66_9925    KYGEMQARSARLELFRRMLSKQMGWFDSNQDGMPAMLTALQRNIRELQIATSQTLGFICH
67_10374   KYGEMQARSARLELFRRMLSKQMGWFDSNQDGMPAMLTALQRNIRELQIATSQTLGFICH
68_3713    KYGEMQARSARLELFRRMLSKQMGWFDSNQDGMPAMLTALQRNIRELQIATSQTLGFICH
69_4511    KYGEMQARSARLELFRRMLSKQMGWFDSNQDGMPAMLTALQRNIRELQIATSQTLGFICH
70_6201    KYGEMQARSARLELFRRMLSKQMGWFDSNQDGMPAMLTALQRNIRELQIATSQTLGFICH
71_3261    KYGEMQARSARLELFRRMLSKQMGWFDSNQDGMPAMLTALQRNIRELQIATSQTLGFICH
72_8516    KYGEMQARSARLELFRRMLSKQMGWFDSNQDGMPAMLTALQRNIRELQIATSQTLGFICH
73_9295    KYGEMQARSARLELFRRMLSKQMGWFDSNQDGMPAMLTALQRNIRELQIATSQTLGFICH
74_809     KYGEMQARSARLELFRRMLSKQMGWFDSNQDGMPAMLTALQRNIRELQIATSQTLGFICH
75_7282    KYGEMQARSARLELFRRMLSKQMGWFDSNQDGMPAMLTALQRNIRELQIATSQTLGFICH
76_4910    KYGEMQARSARLELFRRMLSKQMGWFDSNQDGMPAMLTALQRNIRELQIATSQTLGFICH
77_10912   KYGEMQARSARLELFRRMLSKQMGWFDSNQDGMPAMLTALQRNIRELQIATSQTLGFICH
78_4020    KYGEMQARSARLELFRRMLSKQMGWFDSNQDGMPAMLTALQRNIRELQIATSQTLGFICH
79_6703    KYGEMQARSARLELFRRMLSKQMGWFDSNQDGMPAMLTALQRNIRELQIATSQTLGFICH
80_8619    KYGEMQARSARLELFRRMLSKQMGWFDSNQDGMPAMLTALQRNIRELQIATSQTLGFICH
81_4126    KYGEMQARSARLELFRRMLSKQMGWFDSNQDGMPAMLTALQRNIRELQIATSQTLGFICH
82_1363    KYGEMQARSARLELFRRMLSKQMGWFDSNQDGMPAMLTALQRNIRELQIATSQTLGFICH
83_5813    KYGEMQARSARLELFRRMLSKQMGWFDSNQDGMPAMLTALQRNIRELQIATSQTLGFICH
84_2044    KYGEMQARSARLELFRRMLSKQMGWFDSNQDGMPAMLTALQRNIRELQIATSQTLGFICH
85_7617    KYGEMQARSARLELFRRMLSKQMGWFDSNQDGMPAMLTALQRNIRELQIATSQTLGFICH
86_1530    KYGEMQARSARLELFRRMLSKQMGWFDSNQDGMPAMLTALQRNIRELQIATSQTLGFICH
87_1890    KYGEMQARSARLELFRRMLSKQMGWFDSNQDGMPAMLTALQRNIRELQIATSQTLGFICH
88_7049    KYGEMQARSARLELFRRMLSKQMGWFDSNQDGMPAMLTALQRNIRELQIATSQTLGFICH
89_440     KYGEMQARSARLELFRRMLSKQMGWFDSNQDGMPAMLTALQRNIRELQIATSQTLGFICH
90_4331    KYGEMQARSARLELFRRMLSKQMGWFDSNQDGMPAMLTALQRNIRELQIATSQTLGFICH
```

```
                  250       260       270       280       290       300
1_1896     DFMLSIAGTIVAFKYGPLMTLVITATVPIAMFIMRALSQMLEKAVRTQKEKQAVATKLSA
2_4785     DFMLSIAGTIVAFKYGPLMTLVITATVPIAMFIMRALSQMLEKAVRTQKEKQAVATKLSA
3_3653     DFMLSIAGTIVAFKYGPLMTLVITATVPIAMFIMRALSQMLEKAVRTQKEKQAVATKLSA
4_8157     DFMLSIAGTIVAFKYGPLMTLVITATVPIAMFIMRALSQMLEKAVRTQKEKQAVATKLSA
5_752      DFMLSIAGTIVAFKYGPLMTLVITATVPIAMFIMRALSQMLEKAVRTQKEKQAVATKLSA
6_8853     DFMLSIAGTIVAFKYGPLMTLVITATVPIAMFIMRALSQMLEKAVRTQKEKQAVATKLSA
7_4015     LSGEILLDEHPLQTLDSEWVRKNVTLVQQQSTLFSDTLYANITMNSDNVSRDKVKAACEM
8_2020     DFMLSIAGTIVAFKYGPLMTLVITATVPIAMFIMRALSQMLEKAVRTQKEKQAVATKLSA
9_1829     DFMLSIAGTIVAFKYGPLMTLVITATVPIAMFIMRALSQMLEKAVRTQKEKQAVATKLSA
10_6452    DFMLSIAGTIVAFKYGPLMTLVITATVPIAMFIMRALSQMLEKAVRTQKEKQAVATKLSA
11_8627    DFMLSIAGTIVAFKYGPLMTLVITATVPIAMFIMRALSQMLEKAVRTQKEKQAVATKLSA
12_6118    DFMLSIAGTIVAFKYGPLMTLVITATVPIAMFIMRALSQMLEKAVRTQKEKQAVATKLSA
13_2688    DFMLSIAGTIVAFKYGPLMTLVITATVPIAMFIMRALSQMLEKAVRTQKEKQAVATKLSA
14_6406    DFMLSIAGTIVAFKYGPLMTLVITATVPIAMFIMRALSQMLEKAVRTQKEKQAVATKLSA
15_9419    DFMLSIAGTIVAFKYGPLMTLVITATVPIAMFIMRALSQMLEKAVRTQKEKQAVATKLSA
16_8507    DFMLSIAGTIVAFKYGPLMTLVITATVPIAMFIMRALSQMLEKAVRTQKEKQAVATKLSA
17_5884    DFMLSIAGTIVAFKYGPLMTLVITATVPIAMFIMRALSQMLEKAVRTQKEKQAVATKLSA
18_2670    DFMLSIAGTIVAFKYGPLMTLVITATVPIAMFIMRALSQMLEKAVRTQKEKQAVATKLSA
19_5172    DFMLSIAGTIVAFKYGPLMTLVITATVPIAMFIMRALSQMLEKAVRTQKEKQAVATKLSA
20_8057    DFMLSIAGTIVAFKYGPLMTLVITATVPIAMFIMRALSQMLEKAVRTQKEKQAVATKLSA
21_4920    SEDELDVDMSGKSFYIPEPKTTSSFGALGRMSFVPIPGTNGPIYPTPNQPGSGAYLQLHR
22_6393    DFMLSIAGTIVAFKYGPLMTLVITATVPIAMFIMRALSQMLEKAVRTQKEKQAVATKLSA
23_8263    DFMLSIAGTIVAFKYGPLMTLVITATVPIAMFIMRALSQMLEKAVRTQKEKQAVATKLSA
24_8248    DFMLSIAGTIVAFKYGPLMTLVITATVPIAMFIMRALSQMLEKAVRTQKEKQAVATKLSA
25_6733    DFMLSIAGTIVAFKYGPLMTLVITATVPIAMFIMRALSQMLEKAVRTQKEKQAVATKLSA
26_2277    DFMLSIAGTIVAFKYGPLMTLVITATVPIAMFIMRALSQMLEKAVRTQKEKQAVATKLSA
27_2623    DFMLSIAGTIVAFKYGPLMTLVITATVPIAMFIMRALSQMLEKAVRTQKEKQAVATKLSA
28_7413    DFMLSIAGTIVAFKYGPLMTLVITATVPIAMFIMRALSQMLEKAVRTQKEKQAVATKLSA
29_8980    DFMLSIAGTIVAFKYGPLMTLVITATVPIAMFIMRALSQMLEKAVRTQKEKQAVATKLSA
30_8706    DFMLSIAGTIVAFKYGPLMTLVITATVPIAMFIMRALSQMLEKAVRTQKEKQAVATKLSA
31_6378    DFMLSIAGTIVAFKYGPLMTLVITATVPIAMFIMRALSQMLEKAVRTQKEKQAVATKLSA
32_4679    DFMLSIAGTIVAFKYGPLMTLVITATVPIAMFIMRALSQMLEKAVRTQKEKQAVATKLSA
33_2437    DFMLSIAGTIVAFKYGPLMTLVITATVPIAMFIMRALSQMLEKAVRTQKEKQAVATKLSA
34_9700    DFMLSIAGTIVAFKYGPLMTLVITATVPIAMFIMRALSQMLEKAVRTQKEKQAVATKLSA
35_10218   DFMLSIAGTIVAFKYGPLMTLVITATVPIAMFIMRALSQMLEKAVRTQKEKQAVATKLSA
36_2652    DFMLSIAGTIVAFKYGPLMTLVITATVPIAMFIMRALSQMLEKAVRTQKEKQAVATKLSA
37_5779    DFMLSIAGTIVAFKYGPLMTLVITATVPIAMFIMRALSQMLEKAVRTQKEKQAVATKLSA
38_654     DFMLSIAGTIVAFKYGPLMTLVITATVPIAMFIMRALSQMLEKAVRTQKEKQAVATKLSA
39_4879    DFMLSIAGTIVAFKYGPLMTLVITATVPIAMFIMRALSQMLEKAVRTQKEKQAVATKLSA
40_8113    DFMLSIAGTIVAFKYGPLMTLVITATVPIAMFIMRALSQMLEKAVRTQKEKQAVATKLSA
41_1767    DFMLSIAGTIVAFKYGPLMTLVITATVPIAMFIMRALSQMLEKAVRTQKEKQAVATKLSA
42_8428    DFMLSIAGTIVAFKYGPLMTLVITATVPIAMFIMRALSQMLEKAVRTQKEKQAVATKLSA
43_9819    DFMLSIAGTIVAFKYGPLMTLVITATVPIAMFIMRALSQMLEKAVRTQKEKQAVATKLSA
44_5569    DFMLSIAGTIVAFKYGPLMTLVITATVPIAMFIMRALSQMLEKAVRTQKEKQAVATKLSA
45_4189    DFMLSIAGTIVAFKYGPLMTLVITATVPIAMFIMRALSQMLEKAVRTQKEKQAVATKLSA
46_1027    DFMLSIAGTIVAFKYGPLMTLVITATVPIAMFIMRALSQMLEKAVRTQKEKQAVATKLSA
47_5048    DFMLSIAGTIVAFKYGPLMTLVITATVPIAMFIMRALSQMLEKAVRTQKEKQAVATKLSA
48_10124   DFMLSIAGTIVAFKYGPLMTLVITATVPIAMFIMRALSQMLEKAVRTQKEKQAVATKLSA
49_7151    DFMLSIAGTIVAFKYGPLMTLVITATVPIAMFIMRALSQMLEKAVRTQKEKQAVATKLSA
50_3695    DFMLSIAGTIVAFKYGPLMTLVITATVPIAMFIMRALSQMLEKAVRTQKEKQAVATKLSA
51_364     DFMLSIAGTIVAFKYGPLMTLVITATVPIAMFIMRALSQMLEKAVRTQKEKQAVATKLSA
52_242     DFMLSIAGTIVAFKYGPLMTLVITATVPIAMFIMRALSQMLEKAVRTQKEKQAVATKLSA
53_5225    DFMLSIAGTIVAFKYGPLMTLVITATVPIAMFIMRALSQMLEKAVRTQKEKQAVATKLSA
54_1635    DFMLSIAGTIVAFKYGPLMTLVITATVPIAMFIMRALSQMLEKAVRTQKEKQAVATKLSA
55_5766    DFMLSIAGTIVAFKYGPLMTLVITATVPIAMFIMRALSQMLEKAVRTQKEKQAVATKLSA
56_1557    DFMLSIAGTIVAFKYGPLMTLVITATVPIAMFIMRALSQMLEKAVRTQKEKQAVATKLSA
57_10137   DFMLSIAGTIVAFKYGPLMTLVITATVPIAMFIMRALSQMLEKAVRTQKEKQAVATKLSA
58_10525   DFMLSIAGTIVAFKYGPLMTLVITATVPIAMFIMRALSQMLEKAVRTQKEKQAVATKLSA
59_3457    DFMLSIAGTIVAFKYGPLMTLVITATVPIAMFIMRALSQMLEKAVRTQKEKQAVATKLSA
60_8244    DFMLSIAGTIVAFKYGPLMTLVITATVPIAMFIMRALSQMLEKAVRTQKEKQAVATKLSA
61_7964    DFMLSIAGTIVAFKYGPLMTLVITATVPIAMFIMRALSQMLEKAVRTQKEKQAVATKLSA
62_4413    DFMLSIAGTIVAFKYGPLMTLVITATVPIAMFIMRALSQMLEKAVRTQKEKQAVATKLSA
63_6958    DFMLSIAGTIVAFKYGPLMTLVITATVPIAMFIMRALSQMLEKAVRTQKEKQAVATKLSA
64_5997    QDAEVSKPRRGSIDLIQARGLSALANRSPVKRPLPQGGRIGGVLRRLSTRSSPIEDVEMS
65_8711    DFMLSIAGTIVAFKYGPLMTLVITATVPIAMFIMRALSQMLEKAVRTQKEKQAVATKLSA
66_9925    DFMLSIAGTIVAFKYGPLMTLVITATVPIAMFIMRALSQMLEKAVRTQKEKQAVATKLSA
67_10374   DFMLSIAGTIVAFKYGPLMTLVITATVPIAMFIMRALSQMLEKAVRTQKEKQAVATKLSA
68_3713    DFMLSIAGTIVAFKYGPLMTLVITATVPIAMFIMRALSQMLEKAVRTQKEKQAVATKLSA
69_4511    DFMLSIAGTIVAFKYGPLMTLVITATVPIAMFIMRALSQMLEKAVRTQKEKQAVATKLSA
70_6201    DFMLSIAGTIVAFKYGPLMTLVITATVPIAMFIMRALSQMLEKAVRTQKEKQAVATKLSA
71_3261    DFMLSIAGTIVAFKYGPLMTLVITATVPIAMFIMRALSQMLEKAVRTQKEKQAVATKLSA
72_8516    DFMLSIAGTIVAFKYGPLMTLVITATVPIAMFIMRALSQMLEKAVRTQKEKQAVATKLSA
73_9295    DFMLSIAGTIVAFKYGPLMTLVITATVPIAMFIMRALSQMLEKAVRTQKEKQAVATKLSA
74_809     DFMLSIAGTIVAFKYGPLMTLVITATVPIAMFIMRALSQMLEKAVRTQKEKQAVATKLSA
75_7282    DFMLSIAGTIVAFKYGPLMTLVITATVPIAMFIMRALSQMLEKAVRTQKEKQAVATKLSA
76_4910    DFMLSIAGTIVAFKYGPLMTLVITATVPIAMFIMRALSQMLEKAVRTQKEKQAVATKLSA
77_10912   DFMLSIAGTIVAFKYGPLMTLVITATVPIAMFIMRALSQMLEKAVRTQKEKQAVATKLSA
78_4020    DFMLSIAGTIVAFKYGPLMTLVITATVPIAMFIMRALSQMLEKAVRTQKEKQAVATKLSA
79_6703    DFMLSIAGTIVAFKYGPLMTLVITATVPIAMFIMRALSQMLEKAVRTQKEKQAVATKLSA
80_8619    DFMLSIAGTIVAFKYGPLMTLVITATVPIAMFIMRALSQMLEKAVRTQKEKQAVATKLSA
81_4126    DFMLSIAGTIVAFKYGPLMTLVITATVPIAMFIMRALSQMLEKAVRTQKEKQAVATKLSA
82_1363    DFMLSIAGTIVAFKYGPLMTLVITATVPIAMFIMRALSQMLEKAVRTQKEKQAVATKLSA
83_5813    DFMLSIAGTIVAFKYGPLMTLVITATVPIAMFIMRALSQMLEKAVRTQKEKQAVATKLSA
84_2044    DFMLSIAGTIVAFKYGPLMTLVITATVPIAMFIMRALSQMLEKAVRTQKEKQAVATKLSA
85_7617    DFMLSIAGTIVAFKYGPLMTLVITATVPIAMFIMRALSQMLEKAVRTQKEKQAVATKLSA
86_1530    DFMLSIAGTIVAFKYGPLMTLVITATVPIAMFIMRALSQMLEKAVRTQKEKQAVATKLSA
87_1890    DFMLSIAGTIVAFKYGPLMTLVITATVPIAMFIMRALSQMLEKAVRTQKEKQAVATKLSA
88_7049    DFMLSIAGTIVAFKYGPLMTLVITATVPIAMFIMRALSQMLEKAVRTQKEKQAVATKLSA
89_440     DFMLSIAGTIVAFKYGPLMTLVITATVPIAMFIMRALSQMLEKAVRTQKEKQAVATKLSA
90_4331    DFMLSIAGTIVAFKYGPLMTLVITATVPIAMFIMRALSQMLEKAVRTQKEKQAVATKLSA
```

```
                   310       320       330       340       350       360
1_1896    AAITGIDLVKVYNSHEHELHNFRLAAREAGKHARVQTMWNALQAAFIKIYMAVVFLSGFF
2_4785    AAITGIDLVKVYNSHEHELHNFRLAAREAGKHARVQTMWNALQAAFIKIYMAVVFLSGFF
3_3653    AAITGIDLVKVYNSHEHELHNFRLAAREAGKHARVQTMWNALQAAFIKIYMAVVFLSGFF
4_8157    AAITGIDLVKVYNSHEHELHNFRLAAREAGKHARVQTMWNALQAAFIKIYMAVVFLSGFF
5_752     AAITGIDLVKVYNSHEHELHNFRLAAREAGKHARVQTMWNALQAAFIKIYMAVVFLSGFF
6_8853    AAITGIDLVKVYNSHEHELHNFRLAAREAGKHARVQTMWNALQAAFIKIYMAVVFLSGFF
7_4015    ALLQSTIASLPQGLDTLVGPGGQSLSGGQRQRVALARARLRDTPVLILDEVTSGLDPKSK
8_2020    AAITGIDLVKVYNSHEHELHNFRLAAREAGKHARVQTMWNALQAAFIKIYMAVVFLSGFF
9_1829    AAITGIDLVKVYNSHEHELHNFRLAAREAGKHARVQTMWNALQAAFIKIYMAVVFLSGFF
10_6452   AAITGIDLVKVYNSHEHELHNFRLAAREAGKHARVQTMWNALQAAFIKIYMAVVFLSGFF
11_8627   AAITGIDLVKVYNSHEHELHNFRLAAREAGKHARVQTMWNALQAAFIKIYMAVVFLSGFF
12_6118   AAITGIDLVKVYNSHEHELHNFRLAAREAGKHARVQTMWNALQAAFIKIYMAVVFLSGFF
13_2688   AAITGIDLVKVYNSHEHELHNFRLAAREAGKHARVQTMWNALQAAFIKIYMAVVFLSGFF
14_6406   AAITGIDLVKVYNSHEHELHNFRLAAREAGKHARVQTMWNALQAAFIKIYMAVVFLSGFF
15_9419   AAITGIDLVKVYNSHEHELHNFRLAAREAGKHARVQTMWNALQAAFIKIYMAVVFLSGFF
16_8507   AAITGIDLVKVYNSHEHELHNFRLAAREAGKHARVQTMWNALQAAFIKIYMAVVFLSGFF
17_5884   AAITGIDLVKVYNSHEHELHNFRLAAREAGKHARVQTMWNALQAAFIKIYMAVVFLSGFF
18_2670   AAITGIDLVKVYNSHEHELHNFRLAAREAGKHARVQTMWNALQAAFIKIYMAVVFLSGFF
19_5172   AAITGIDLVKVYNSHEHELHNFRLAAREAGKHARVQTMWNALQAAFIKIYMAVVFLSGFF
20_8057   AAITGIDLVKVYNSHEHELHNFRLAAREAGKHARVQTMWNALQAAFIKIYMAVVFLSGFF
21_4920   SPRHRHREQGRPNNWQDAEVSKPRRGSIDLIQARGLSALANRSPVKRPLPQGGRIGGVLR
22_6393   AAITGIDLVKVYNSHEHELHNFRLAAREAGKHARVQTMWNALQAAFIKIYMAVVFLSGFF
23_8263   AAITGIDLVKVYNSHEHELHNFRLAAREAGKHARVQTMWNALQAAFIKIYMAVVFLSGFF
24_8248   AAITGIDLVKVYNSHEHELHNFRLAAREAGKHARVQTMWNALQAAFIKIYMAVVFLSGFF
25_6733   AAITGIDLVKVYNSHEHELHNFRLAAREAGKHARVQTMWNALQAAFIKIYMAVVFLSGFF
26_2277   AAITGIDLVKVYNSHEHELHNFRLAAREAGKHARVQTMWNALQAAFIKIYMAVVFLSGFF
27_2623   AAITGIDLVKVYNSHEHELHNFRLAAREAGKHARVQTMWNALQAAFIKIYMAVVFLSGFF
28_7413   AAITGIDLVKVYNSHEHELHNFRLAAREAGKHARVQTMWNALQAAFIKIYMAVVFLSGFF
29_8980   AAITGIDLVKVYNSHEHELHNFRLAAREAGKHARVQTMWNALQAAFIKIYMAVVFLSGFF
30_8706   AAITGIDLVKVYNSHEHELHNFRLAAREAGKHARVQTMWNALQAAFIKIYMAVVFLSGFF
31_6378   AAITGIDLVKVYNSHEHELHNFRLAAREAGKHARVQTMWNALQAAFIKIYMAVVFLSGFF
32_4679   AAITGIDLVKVYNSHEHELHNFRLAAREAGKHARVQTMWNALQAAFIKIYMAVVFLSGFF
33_2437   AAITGIDLVKVYNSHEHELHNFRLAAREAGKHARVQTMWNALQAAFIKIYMAVVFLSGFF
34_9700   AAITGIDLVKVYNSHEHELHNFRLAAREAGKHARVQTMWNALQAAFIKIYMAVVFLSGFF
35_10218  AAITGIDLVKVYNSHEHELHNFRLAAREAGKHARVQTMWNALQAAFIKIYMAVVFLSGFF
36_2652   AAITGIDLVKVYNSHEHELHNFRLAAREAGKHARVQTMWNALQAAFIKIYMAVVFLSGFF
37_5779   AAITGIDLVKVYNSHEHELHNFRLAAREAGKHARVQTMWNALQAAFIKIYMAVVFLSGFF
38_654    AAITGIDLVKVYNSHEHELHNFRLAAREAGKHARVQTMWNALQAAFIKIYMAVVFLSGFF
39_4879   AAITGIDLVKVYNSHEHELHNFRLAAREAGKHARVQTMWNALQAAFIKIYMAVVFLSGFF
40_8113   AAITGIDLVKVYNSHEHELHNFRLAAREAGKHARVQTMWNALQAAFIKIYMAVVFLSGFF
41_1767   AAITGIDLVKVYNSHEHELHNFRLAAREAGKHARVQTMWNALQAAFIKIYMAVVFLSGFF
42_8428   AAITGIDLVKVYNSHEHELHNFRLAAREAGKHARVQTMWNALQAAFIKIYMAVVFLSGFF
43_9819   AAITGIDLVKVYNSHEHELHNFRLAAREAGKHARVQTMWNALQAAFIKIYMAVVFLSGFF
44_5569   AAITGIDLVKVYNSHEHELHNFRLAAREAGKHARVQTMWNALQAAFIKIYMAVVFLSGFF
45_4189   AAITGIDLVKVYNSHEHELHNFRLAAREAGKHARVQTMWNALQAAFIKIYMAVVFLSGFF
46_1027   AAITGIDLVKVYNSHEHELHNFRLAAREAGKHARVQTMWNALQAAFIKIYMAVVFLSGFF
47_5048   AAITGIDLVKVYNSHEHELHNFRLAAREAGKHARVQTMWNALQAAFIKIYMAVVFLSGFF
48_10124  AAITGIDLVKVYNSHEHELHNFRLAAREAGKHARVQTMWNALQAAFIKIYMAVVFLSGFF
49_7151   AAITGIDLVKVYNSHEHELHNFRLAAREAGKHARVQTMWNALQAAFIKIYMAVVFLSGFF
50_3695   AAITGIDLVKVYNSHEHELHNFRLAAREAGKHARVQTMWNALQAAFIKIYMAVVFLSGFF
51_364    AAITGIDLVKVYNSHEHELHNFRLAAREAGKHARVQTMWNALQAAFIKIYMAVVFLSGFF
52_242    AAITGIDLVKVYNSHEHELHNFRLAAREAGKHARVQTMWNALQAAFIKIYMAVVFLSGFF
53_5225   AAITGIDLVKVYNSHEHELHNFRLAAREAGKHARVQTMWNALQAAFIKIYMAVVFLSGFF
54_1635   AAITGIDLVKVYNSHEHELHNFRLAAREAGKHARVQTMWNALQAAFIKIYMAVVFLSGFF
55_5766   AAITGIDLVKVYNSHEHELHNFRLAAREAGKHARVQTMWNALQAAFIKIYMAVVFLSGFF
56_1557   AAITGIDLVKVYNSHEHELHNFRLAAREAGKHARVQTMWNALQAAFIKIYMAVVFLSGFF
57_10137  AAITGIDLVKVYNSHEHELHNFRLAAREAGKHARVQTMWNALQAAFIKIYMAVVFLSGFF
58_10525  AAITGIDLVKVYNSHEHELHNFRLAAREAGKHARVQTMWNALQAAFIKIYMAVVFLSGFF
59_3457   AAITGIDLVKVYNSHEHELHNFRLAAREAGKHARVQTMWNALQAAFIKIYMAVVFLSGFF
60_8244   AAITGIDLVKVYNSHEHELHNFRLAAREAGKHARVQTMWNALQAAFIKIYMAVVFLSGFF
61_7964   AAITGIDLVKVYNSHEHELHNFRLAAREAGKHARVQTMWNALQAAFIKIYMAVVFLSGFF
62_4413   AAITGIDLVKVYNSHEHELHNFRLAAREAGKHARVQTMWNALQAAFIKIYMAVVFLSGFF
63_6958   AAITGIDLVKVYNSHEHELHNFRLAAREAGKHARVQTMWNALQAAFIKIYMAVVFLSGFF
64_5997   PFRDHDKGVKEVVRERIREAIGKGTGAGKRGVTKRQARRGKKDGPDEEATNTTGHMPAYQ
65_8711   AAITGIDLVKVYNSHEHELHNFRLAAREAGKHARVQTMWNALQAAFIKIYMAVVFLSGFF
66_9925   AAITGIDLVKVYNSHEHELHNFRLAAREAGKHARVQTMWNALQAAFIKIYMAVVFLSGFF
67_10374  AAITGIDLVKVYNSHEHELHNFRLAAREAGKHARVQTMWNALQAAFIKIYMAVVFLSGFF
68_3713   AAITGIDLVKVYNSHEHELHNFRLAAREAGKHARVQTMWNALQAAFIKIYMAVVFLSGFF
69_4511   AAITGIDLVKVYNSHEHELHNFRLAAREAGKHARVQTMWNALQAAFIKIYMAVVFLSGFF
70_6201   AAITGIDLVKVYNSHEHELHNFRLAAREAGKHARVQTMWNALQAAFIKIYMAVVFLSGFF
71_3261   AAITGIDLVKVYNSHEHELHNFRLAAREAGKHARVQTMWNALQAAFIKIYMAVVFLSGFF
72_8516   AAITGIDLVKVYNSHEHELHNFRLAAREAGKHARVQTMWNALQAAFIKIYMAVVFLSGFF
73_9295   AAITGIDLVKVYNSHEHELHNFRLAAREAGKHARVQTMWNALQAAFIKIYMAVVFLSGFF
74_809    AAITGIDLVKVYNSHEHELHNFRLAAREAGKHARVQTMWNALQAAFIKIYMAVVFLSGFF
75_7282   AAITGIDLVKVYNSHEHELHNFRLAAREAGKHARVQTMWNALQAAFIKIYMAVVFLSGFF
76_4910   AAITGIDLVKVYNSHEHELHNFRLAAREAGKHARVQTMWNALQAAFIKIYMAVVFLSGFF
77_10912  AAITGIDLVKVYNSHEHELHNFRLAAREAGKHARVQTMWNALQAAFIKIYMAVVFLSGFF
78_4020   AAITGIDLVKVYNSHEHELHNFRLAAREAGKHARVQTMWNALQAAFIKIYMAVVFLSGFF
79_6703   AAITGIDLVKVYNSHEHELHNFRLAAREAGKHARVQTMWNALQAAFIKIYMAVVFLSGFF
80_8619   AAITGIDLVKVYNSHEHELHNFRLAAREAGKHARVQTMWNALQAAFIKIYMAVVFLSGFF
81_4126   AAITGIDLVKVYNSHEHELHNFRLAAREAGKHARVQTMWNALQAAFIKIYMAVVFLSGFF
82_1363   AAITGIDLVKVYNSHEHELHNFRLAAREAGKHARVQTMWNALQAAFIKIYMAVVFLSGFF
83_5813   AAITGIDLVKVYNSHEHELHNFRLAAREAGKHARVQTMWNALQAAFIKIYMAVVFLSGFF
84_2044   AAITGIDLVKVYNSHEHELHNFRLAAREAGKHARVQTMWNALQAAFIKIYMAVVFLSGFF
85_7617   AAITGIDLVKVYNSHEHELHNFRLAAREAGKHARVQTMWNALQAAFIKIYMAVVFLSGFF
86_1530   AAITGIDLVKVYNSHEHELHNFRLAAREAGKHARVQTMWNALQAAFIKIYMAVVFLSGFF
87_1890   AAITGIDLVKVYNSHEHELHNFRLAAREAGKHARVQTMWNALQAAFIKIYMAVVFLSGFF
88_7049   AAITGIDLVKVYNSHEHELHNFRLAAREAGKHARVQTMWNALQAAFIKIYMAVVFLSGFF
89_440    AAITGIDLVKVYNSHEHELHNFRLAAREAGKHARVQTMWNALQAAFIKIYMAVVFLSGFF
90_4331   AAITGIDLVKVYNSHEHELHNFRLAAREAGKHARVQTMWNALQAAFIKIYMAVVFLSGFF
```

```
                  370           380           390           400           410           420
                    .             .             .             .             .             .
1_1896     FGIYQVDRGNMSAGDVITTFYAALSTFEGVQGLGPHILSLVRGMQASKALKSVVSDRVVE
2_4785     FGIYQVDRGNMSAGDVITTFYAALSTFEGVQGLGPHILSLVRGMQASKALKSVVSDRVVE
3_3653     FGIYQVDRGNMSAGDVITTFYAALSTFEGVQGLGPHILSLVRGMQASKALKSVVSDRVVE
4_8157     FGIYQVDRGNMSAGDVITTFYAALSTFEGVQGLGPHILSLVRGMQASKALKSVVSDRVVE
5_752      FGIYQVDRGNMSAGDVITTFYAALSTFEGVQGLGPHILSLVRGMQASKALKSVVSDRVVE
6_8853     FGIYQVDRGNMSAGDVITTFYAALSTFEGVQGLGPHILSLVRGMQASKALKSVVSDRVVE
7_4015     LMIMDAIRYWRSGRTTIVITHDISQVGDDDYVYVMDKAEVREEGLCKQLQSNRDGYFLQL
8_2020     FGIYQVDRGNMSAGDVITTFYAALSTFEGVQGLGPHILSLVRGMQASKALKSVVSDRVVE
9_1829     FGIYQVDRGNMSAGDVITTFYAALSTFEGVQGLGPHILSLVRGMQASKALKSVVSDRVVE
10_6452    FGIYQVDRGNMSAGDVITTFYAALSTFEGVQGLGPHILSLVRGMQASKALKSVVSDRVVE
11_8627    FGIYQVDRGNMSAGDVITTFYAALSTFEGVQGLGPHILSLVRGMQASKALKSVVSDRVVE
12_6118    FGIYQVDRGNMSAGDVITTFYAALSTFEGVQGLGPHILSLVRGMQASKALKSVVSDRVVE
13_2688    FGIYQVDRGNMSAGDVITTFYAALSTFEGVQGLGPHILSLVRGMQASKALKSVVSDRVVE
14_6406    FGIYQVDRGNMSAGDVITTFYAALSTFEGVQGLGPHILSLVRGMQASKALKSVVSDRVVE
15_9419    FGIYQVDRGNMSAGDVITTFYAALSTFEGVQGLGPHILSLVRGMQASKALKSVVSDRVVE
16_8507    FGIYQVDRGNMSAGDVITTFYAALSTFEGVQGLGPHILSLVRGMQASKALKSVVSDRVVE
17_5884    FGIYQVDRGNMSAGDVITTFYAALSTFEGVQGLGPHILSLVRGMQASKALKSVVSDRVVE
18_2670    FGIYQVDRGNMSAGDVITTFYAALSTFEGVQGLGPHILSLVRGMQASKALKSVVSDRVVE
19_5172    FGIYQVDRGNMSAGDVITTFYAALSTFEGVQGLGPHILSLVRGMQASKALKSVVSDRVVE
20_8057    FGIYQVDRGNMSAGDVITTFYAALSTFEGVQGLGPHILSLVRGMQASKALKSVVSDRVVE
21_4920    RLSTRSSPIEDVEMSPFRDHDKGVKEVVRERIREAIGKGTGAGKRGVTKRQARRGKKDGP
22_6393    FGIYQVDRGNMSAGDVITTFYAALSTFEGVQGLGPHILSLVRGMQASKALKSVVSDRVVE
23_8263    FGIYQVDRGNMSAGDVITTFYAALSTFEGVQGLGPHILSLVRGMQASKALKSVVSDRVVE
24_8248    FGIYQVDRGNMSAGDVITTFYAALSTFEGVQGLGPHILSLVRGMQASKALKSVVSDRVVE
25_6733    FGIYQVDRGNMSAGDVITTFYAALSTFEGVQGLGPHILSLVRGMQASKALKSVVSDRVVE
26_2277    FGIYQVDRGNMSAGDVITTFYAALSTFEGVQGLGPHILSLVRGMQASKALKSVVSDRVVE
27_2623    FGIYQVDRGNMSAGDVITTFYAALSTFEGVQGLGPHILSLVRGMQASKALKSVVSDRVVE
28_7413    FGIYQVDRGNMSAGDVITTFYAALSTFEGVQGLGPHILSLVRGMQASKALKSVVSDRVVE
29_8980    FGIYQVDRGNMSAGDVITTFYAALSTFEGVQGLGPHILSLVRGMQASKALKSVVSDRVVE
30_8706    FGIYQVDRGNMSAGDVITTFYAALSTFEGVQGLGPHILSLVRGMQASKALKSVVSDRVVE
31_6378    FGIYQVDRGNMSAGDVITTFYAALSTFEGVQGLGPHILSLVRGMQASKALKSVVSDRVVE
32_4679    FGIYQVDRGNMSAGDVITTFYAALSTFEGVQGLGPHILSLVRGMQASKALKSVVSDRVVE
33_2437    FGIYQVDRGNMSAGDVITTFYAALSTFEGVQGLGPHILSLVRGMQASKALKSVVSDRVVE
34_9700    FGIYQVDRGNMSAGDVITTFYAALSTFEGVQGLGPHILSLVRGMQASKALKSVVSDRVVE
35_10218   FGIYQVDRGNMSAGDVITTFYAALSTFEGVQGLGPHILSLVRGMQASKALKSVVSDRVVE
36_2652    FGIYQVDRGNMSAGDVITTFYAALSTFEGVQGLGPHILSLVRGMQASKALKSVVSDRVVE
37_5779    FGIYQVDRGNMSAGDVITTFYAALSTFEGVQGLGPHILSLVRGMQASKALKSVVSDRVVE
38_654     FGIYQVDRGNMSAGDVITTFYAALSTFEGVQGLGPHILSLVRGMQASKALKSVVSDRVVE
39_4879    FGIYQVDRGNMSAGDVITTFYAALSTFEGVQGLGPHILSLVRGMQASKALKSVVSDRVVE
40_8113    FGIYQVDRGNMSAGDVITTFYAALSTFEGVQGLGPHILSLVRGMQASKALKSVVSDRVVE
41_1767    FGIYQVDRGNMSAGDVITTFYAALSTFEGVQGLGPHILSLVRGMQASKALKSVVSDRVVE
42_8428    FGIYQVDRGNMSAGDVITTFYAALSTFEGVQGLGPHILSLVRGMQASKALKSVVSDRVVE
43_9819    FGIYQVDRGNMSAGDVITTFYAALSTFEGVQGLGPHILSLVRGMQASKALKSVVSDRVVE
44_5569    FGIYQVDRGNMSAGDVITTFYAALSTFEGVQGLGPHILSLVRGMQASKALKSVVSDRVVE
45_4189    FGIYQVDRGNMSAGDVITTFYAALSTFEGVQGLGPHILSLVRGMQASKALKSVVSDRVVE
46_1027    FGIYQVDRGNMSAGDVITTFYAALSTFEGVQGLGPHILSLVRGMQASKALKSVVSDRVVE
47_5048    FGIYQVDRGNMSAGDVITTFYAALSTFEGVQGLGPHILSLVRGMQASKALKSVVSDRVVE
48_10124   FGIYQVDRGNMSAGDVITTFYAALSTFEGVQGLGPHILSLVRGMQASKALKSVVSDRVVE
49_7151    FGIYQVDRGNMSAGDVITTFYAALSTFEGVQGLGPHILSLVRGMQASKALKSVVSDRVVE
50_3695    FGIYQVDRGNMSAGDVITTFYAALSTFEGVQGLGPHILSLVRGMQASKALKSVVSDRVVE
51_364     FGIYQVDRGNMSAGDVITTFYAALSTFEGVQGLGPHILSLVRGMQASKALKSVVSDRVVE
52_242     FGIYQVDRGNMSAGDVITTFYAALSTFEGVQGLGPHILSLVRGMQASKALKSVVSDRVVE
53_5225    FGIYQVDRGNMSAGDVITTFYAALSTFEGVQGLGPHILSLVRGMQASKALKSVVSDRVVE
54_1635    FGIYQVDRGNMSAGDVITTFYAALSTFEGVQGLGPHILSLVRGMQASKALKSVVSDRVVE
55_5766    FGIYQVDRGNMSAGDVITTFYAALSTFEGVQGLGPHILSLVRGMQASKALKSVVSDRVVE
56_1557    FGIYQVDRGNMSAGDVITTFYAALSTFEGVQGLGPHILSLVRGMQASKALKSVVSDRVVE
57_10137   FGIYQVDRGNMSAGDVITTFYAALSTFEGVQGLGPHILSLVRGMQASKALKSVVSDRVVE
58_10525   FGIYQVDRGNMSAGDVITTFYAALSTFEGVQGLGPHILSLVRGMQASKALKSVVSDRVVE
59_3457    FGIYQVDRGNMSAGDVITTFYAALSTFEGVQGLGPHILSLVRGMQASKALKSVVSDRVVE
60_8244    FGIYQVDRGNMSAGDVITTFYAALSTFEGVQGLGPHILSLVRGMQASKALKSVVSDRVVE
61_7964    FGIYQVDRGNMSAGDVITTFYAALSTFEGVQGLGPHILSLVRGMQASKALKSVVSDRVVE
62_4413    FGIYQVDRGNMSAGDVITTFYAALSTFEGVQGLGPHILSLVRGMQASKALKSVVSDRVVE
63_6958    FGIYQVDRGNMSAGDVITTFYAALSTFEGVQGLGPHILSLVRGMQASKALKSVVSDRVVE
64_5997    ILKTVWPALDTKHRLYAVAALFWCIVAAGCSPVFAFVFSNLLQAFWAQGSKLEAGTKWAI
65_8711    FGIYQVDRGNMSAGDVITTFYAALSTFEGVQGLGPHILSLVRGMQASKALKSVVSDRVVE
66_9925    FGIYQVDRGNMSAGDVITTFYAALSTFEGVQGLGPHILSLVRGMQASKALKSVVSDRVVE
67_10374   FGIYQVDRGNMSAGDVITTFYAALSTFEGVQGLGPHILSLVRGMQASKALKSVVSDRVVE
68_3713    FGIYQVDRGNMSAGDVITTFYAALSTFEGVQGLGPHILSLVRGMQASKALKSVVSDRVVE
69_4511    FGIYQVDRGNMSAGDVITTFYAALSTFEGVQGLGPHILSLVRGMQASKALKSVVSDRVVE
70_6201    FGIYQVDRGNMSAGDVITTFYAALSTFEGVQGLGPHILSLVRGMQASKALKSVVSDRVVE
71_3261    FGIYQVDRGNMSAGDVITTFYAALSTFEGVQGLGPHILSLVRGMQASKALKSVVSDRVVE
72_8516    FGIYQVDRGNMSAGDVITTFYAALSTFEGVQGLGPHILSLVRGMQASKALKSVVSDRVVE
73_9295    FGIYQVDRGNMSAGDVITTFYAALSTFEGVQGLGPHILSLVRGMQASKALKSVVSDRVVE
74_809     FGIYQVDRGNMSAGDVITTFYAALSTFEGVQGLGPHILSLVRGMQASKALKSVVSDRVVE
75_7282    FGIYQVDRGNMSAGDVITTFYAALSTFEGVQGLGPHILSLVRGMQASKALKSVVSDRVVE
76_4910    FGIYQVDRGNMSAGDVITTFYAALSTFEGVQGLGPHILSLVRGMQASKALKSVVSDRVVE
77_10912   FGIYQVDRGNMSAGDVITTFYAALSTFEGVQGLGPHILSLVRGMQASKALKSVVSDRVVE
78_4020    FGIYQVDRGNMSAGDVITTFYAALSTFEGVQGLGPHILSLVRGMQASKALKSVVSDRVVE
79_6703    FGIYQVDRGNMSAGDVITTFYAALSTFEGVQGLGPHILSLVRGMQASKALKSVVSDRVVE
80_8619    FGIYQVDRGNMSAGDVITTFYAALSTFEGVQGLGPHILSLVRGMQASKALKSVVSDRVVE
81_4126    FGIYQVDRGNMSAGDVITTFYAALSTFEGVQGLGPHILSLVRGMQASKALKSVVSDRVVE
82_1363    FGIYQVDRGNMSAGDVITTFYAALSTFEGVQGLGPHILSLVRGMQASKALKSVVSDRVVE
83_5813    FGIYQVDRGNMSAGDVITTFYAALSTFEGVQGLGPHILSLVRGMQASKALKSVVSDRVVE
84_2044    FGIYQVDRGNMSAGDVITTFYAALSTFEGVQGLGPHILSLVRGMQASKALKSVVSDRVVE
85_7617    FGIYQVDRGNMSAGDVITTFYAALSTFEGVQGLGPHILSLVRGMQASKALKSVVSDRVVE
86_1530    FGIYQVDRGNMSAGDVITTFYAALSTFEGVQGLGPHILSLVRGMQASKALKSVVSDRVVE
87_1890    FGIYQVDRGNMSAGDVITTFYAALSTFEGVQGLGPHILSLVRGMQASKALKSVVSDRVVE
88_7049    FGIYQVDRGNMSAGDVITTFYAALSTFEGVQGLGPHILSLVRGMQASKALKSVVSDRVVE
89_440     FGIYQVDRGNMSAGDVITTFYAALSTFEGVQGLGPHILSLVRGMQASKALKSVVSDRVVE
90_4331    FGIYQVDRGNMSAGDVITTFYAALSTFEGVQGLGPHILSLVRGMQASKALKSVVSDRVVE
```

```
          430       440       450       460       470       480
1_1896    MGGGRKPRVFEGRIEVKSVSFAYPSNPAVQVLKPTSMTFKPGKMTFLVGRSGSGKSTITN
2_4785    MGGGRKPRVFEGRIEVKSVSFAYPSNPAVQVLKPTSMTFKPGKMTFLVGRSGSGKSTITN
3_3653    MGGGRKPRVFEGRIEVKSVSFAYPSNPAVQVLKPTSMTFKPGKMTFLVGRSGSGKSTITN
4_8157    MGGGRKPRVFEGRIEVKSVSFAYPSNPAVQVLKPTSMTFKPGKMTFLVGRSGSGKSTITN
5_752     MGGGRKPRVFEGRIEVKSVSFAYPSNPAVQVLKPTSMTFKPGKMTFLVGRSGSGKSTITN
6_8853    MGGGRKPRVFEGRIEVKSVSFAYPSNPAVQVLKPTSMTFKPGKMTFLVGRSGSGKSTITN
7_4015    RALAESGADSSSQDGAMTPDDDLSSLSSSEDEPDADMSGKSFYIPEPKTTSSLGALGRMS
8_2020    MGGGRKPRVFEGRIEVKSVSFAYPSNPAVQVLKPTSMTFKPGKMTFLVGRSGSGKSTITN
9_1829    MGGGRKPRVFEGRIEVKSVSFAYPSNPAVQVLKPTSMTFKPGKMTFLVGRSGSGKSTITN
10_6452   MGGGRKPRVFEGRIEVKSVSFAYPSNPAVQVLKPTSMTFKPGKMTFLVGRSGSGKSTITN
11_8627   MGGGRKPRVFEGRIEVKSVSFAYPSNPAVQVLKPTSMTFKPGKMTFLVGRSGSGKSTITN
12_6118   MGGGRKPRVFEGRIEVKSVSFAYPSNPAVQVLKPTSMTFKPGKMTFLVGRSGSGKSTITN
13_2688   MGGGRKPRVFEGRIEVKSVSFAYPSNPAVQVLKPTSMTFKPGKMTFLVGRSGSGKSTITN
14_6406   MGGGRKPRVFEGRIEVKSVSFAYPSNPAVQVLKPTSMTFKPGKMTFLVGRSGSGKSTITN
15_9419   MGGGRKPRVFEGRIEVKSVSFAYPSNPAVQVLKPTSMTFKPGKMTFLVGRSGSGKSTITN
16_8507   MGGGRKPRVFEGRIEVKSVSFAYPSNPAVQVLKPTSMTFKPGKMTFLVGRSGSGKSTITN
17_5884   MGGGRKPRVFEGRIEVKSVSFAYPSNPAVQVLKPTSMTFKPGKMTFLVGRSGSGKSTITN
18_2670   MGGGRKPRVFEGRIEVKSVSFAYPSNPAVQVLKPTSMTFKPGKMTFLVGRSGSGKSTITN
19_5172   MGGGRKPRVFEGRIEVKSVSFAYPSNPAVQVLKPTSMTFKPGKMTFLVGRSGSGKSTITN
20_8057   MGGGRKPRVFEGRIEVKSVSFAYPSNPAVQVLKPTSMTFKPGKMTFLVGRSGSGKSTITN
21_4920   DEEATNTTGHMPAYQILKTVWPALDTKHRLYAVAALFWCIVAAGCSPVFAFVFSNLLQAF
22_6393   MGGGRKPRVFEGRIEVKSVSFAYPSNPAVQVLKPTSMTFKPGKMTFLVGRSGSGKSTITN
23_8263   MGGGRKPRVFEGRIEVKSVSFAYPSNPAVQVLKPTSMTFKPGKMTFLVGRSGSGKSTITN
24_8248   MGGGRKPRVFEGRIEVKSVSFAYPSNPAVQVLKPTSMTFKPGKMTFLVGRSGSGKSTITN
25_6733   MGGGRKPRVFEGRIEVKSVSFAYPSNPAVQVLKPTSMTFKPGKMTFLVGRSGSGKSTITN
26_2277   MGGGRKPRVFEGRIEVKSVSFAYPSNPAVQVLKPTSMTFKPGKMTFLVGRSGSGKSTITN
27_2623   MGGGRKPRVFEGRIEVKSVSFAYPSNPAVQVLKPTSMTFKPGKMTFLVGRSGSGKSTITN
28_7413   MGGGRKPRVFEGRIEVKSVSFAYPSNPAVQVLKPTSMTFKPGKMTFLVGRSGSGKSTITN
29_8980   MGGGRKPRVFEGRIEVKSVSFAYPSNPAVQVLKPTSMTFKPGKMTFLVGRSGSGKSTITN
30_8706   MGGGRKPRVFEGRIEVKSVSFAYPSNPAVQVLKPTSMTFKPGKMTFLVGRSGSGKSTITN
31_6378   MGGGRKPRVFEGRIEVKSVSFAYPSNPAVQVLKPTSMTFKPGKMTFLVGRSGSGKSTITN
32_4679   MGGGRKPRVFEGRIEVKSVSFAYPSNPAVQVLKPTSMTFKPGKMTFLVGRSGSGKSTITN
33_2437   MGGGRKPRVFEGRIEVKSVSFAYPSNPAVQVLKPTSMTFKPGKMTFLVGRSGSGKSTITN
34_9700   MGGGRKPRVFEGRIEVKSVSFAYPSNPAVQVLKPTSMTFKPGKMTFLVGRSGSGKSTITN
35_10218  MGGGRKPRVFEGRIEVKSVSFAYPSNPAVQVLKPTSMTFKPGKMTFLVGRSGSGKSTITN
36_2652   MGGGRKPRVFEGRIEVKSVSFAYPSNPAVQVLKPTSMTFKPGKMTFLVGRSGSGKSTITN
37_5779   MGGGRKPRVFEGRIEVKSVSFAYPSNPAVQVLKPTSMTFKPGKMTFLVGRSGSGKSTITN
38_654    MGGGRKPRVFEGRIEVKSVSFAYPSNPAVQVLKPTSMTFKPGKMTFLVGRSGSGKSTITN
39_4879   MGGGRKPRVFEGRIEVKSVSFAYPSNPAVQVLKPTSMTFKPGKMTFLVGRSGSGKSTITN
40_8113   MGGGRKPRVFEGRIEVKSVSFAYPSNPAVQVLKPTSMTFKPGKMTFLVGRSGSGKSTITN
41_1767   MGGGRKPRVFEGRIEVKSVLKPTSMTFKPGKMTFLVGRSGSGKSTITNLLVKFYEPLSGE
42_8428   MGGGRKPRVFEGRIEVKSVSFAYPSNPAVQVLKPTSMTFKPGKMTFLVGRSGSGKSTITN
43_9819   MGGGRKPRVFEGRIEVKSVSFAYPSNPAVQVLKPTSMTFKPGKMTFLVGRSGSGKSTITN
44_5569   MGGGRKPRVFEGRIEVKSVSFAYPSNPAVQVLKPTSMTFKPGKMTFLVGRSGSGKSTITN
45_4189   MGGGRKPRVFEGRIEVKSVSFAYPSNPAVQVLKPTSMTFKPGKMTFLVGRSGSGKSTITN
46_1027   MGGGRKPRVFEGRIEVKSVSFAYPSNPAVQVLKPTSMTFKPGKMTFLVGRSGSGKSTITN
47_5048   MGGGRKPRVFEGRIEVKSVSFAYPSNPAVQVLKPTSMTFKPGKMTFLVGRSGSGKSTITN
48_10124  MGGGRKPRVFEGRIEVKSVSFAYPSNPAVQVLKPTSMTFKPGKMTFLVGRSGSGKSTITN
49_7151   MGGGRKPRVFEGRIEVKSVSFAYPSNPAVQVLKPTSMTFKPGKMTFLVGRSGSGKSTITN
50_3695   MGGGRKPRVFEGRIEVKSVSFAYPSNPAVQVLKPTSMTFKPGKMTFLVGRSGSGKSTITN
51_364    MGGGRKPRVFEGRIEVKSVSFAYPSNPAVQVLKPTSMTFKPGKMTFLVGRSGSGKSTITN
52_242    MGGGRKPRVFEGRIEVKSVSFAYPSNPAVQVLKPTSMTFKPGKMTFLVGRSGSGKSTITN
53_5225   MGGGRKPRVFEGRIEVKSVSFAYPSNPAVQVLKPTSMTFKPGKMTFLVGRSGSGKSTITN
54_1635   MGGGRKPRVFEGRIEVKSVSFAYPSNPAVQVLKPTSMTFKPGKMTFLVGRSGSGKSTITN
55_5766   MGGGRKPRVFEGRIEVKSVSFAYPSNPAVQVLKPTSMTFKPGKMTFLVGRSGSGKSTITN
56_1557   MGGGRKPRVFEGRIEVKSVSFAYPSNPAVQVLKPTSMTFKPGKMTFLVGRSGSGKSTITN
57_10137  MGGGRKPRVFEGRIEVKSVSFAYPSNPAVQVLKPTSMTFKPGKMTFLVGRSGSGKSTITN
58_10525  MGGGRKPRVFEGRIEVKSVSFAYPSNPAVQVLKPTSMTFKPGKMTFLVGRSGSGKSTITN
59_3457   MGGGRKPRVFEGRIEVKSVSFAYPSNPAVQVLKPTSMTFKPGKMTFLVGRSGSGKSTITN
60_8244   MGGGRKPRVFEGRIEVKSVSFAYPSNPAVQVLKPTSMTFKPGKMTFLVGRSGSGKSTITN
61_7964   MGGGRKPRVFEGRIEVKSVSFAYPSNPAVQVLKPTSMTFKPGKMTFLVGRSGSGKSTITN
62_4413   MGGGRKPRVFEGRIEVKSVSFAYPSNPAVQVLKPTSMTFKPGKMTFLVGRSGSGKSTITN
63_6958   MGGGRKPRVFEGRIEVKSVSFAYPSNPAVQVLKPTSMTFKPGKMTFLVGRSGSGKSTITN
64_5997   VLGCVGILDFTAVFFTFFLAGAVAQEWVDTLKVDAFFSILCQPRAWHDRSKNSAARVCDV
65_8711   MGGGRKPRVFEGRIEVKSVSFAYPSNPAVQVLKPTSMTFKPGKMTFLVGRSGSGKSTITN
66_9925   MGGGRKPRVFEGRIEVKSVSFAYPSNPAVQVLKPTSMTFKPGKMTFLVGRSGSGKSTITN
67_10374  MGGGRKPRVFEGRIEVKSVSFAYPSNPAVQVLKPTSMTFKPGKMTFLVGRSGSGKSTITN
68_3713   MGGGRKPRVFEGRIEVKSVSFAYPSNPAVQVLKPTSMTFKPGKMTFLVGRSGSGKSTITN
69_4511   MGGGRKPRVFEGRIEVKSVSFAYPSNPAVQVLKPTSMTFKPGKMTFLVGRSGSGKSTITN
70_6201   MGGGRKPRVFEGRIEVKSVSFAYPSNPAVQVLKPTSMTFKPGKMTFLVGRSGSGKSTITN
71_3261   MGGGRKPRVFEGRIEVKSVSFAYPSNPAVQVLKPTSMTFKPGKMTFLVGRSGSGKSTITN
72_8516   MGGGRKPRVFEGRIEVKSVSFAYPSNPAVQVLKPTSMTFKPGKMTFLVGRSGSGKSTITN
73_9295   MGGGRKPRVFEGRIEVKSVSFAYPSNPAVQVLKPTSMTFKPGKMTFLVGRSGSGKSTITN
74_809    MGGGRKPRVFEGRIEVKSVSFAYPSNPAVQVLKPTSMTFKPGKMTFLVGRSGSGKSTITN
75_7282   MGGGRKPRVFEGRIEVKSVSFAYPSNPAVQVLKPTSMTFKPGKMTFLVGRSGSGKSTITN
76_4910   MGGGRKPRVFEGRIEVKSVSFAYPSNPAVQVLKPTSMTFKPGKMTFLVGRSGSGKSTITN
77_10912  MGGGRKPRVFEGRIEVKSVSFAYPSNPAVQVLKPTSMTFKPGKMTFLVGRSGSGKSTITN
78_4020   MGGGRKPRVFEGRIEVKSVSFAYPSNPAVQVLKPTSMTFKPGKMTFLVGRSGSGKSTITN
79_6703   MGGGRKPRVFEGRIEVKSVSFAYPSNPAVQVLKPTSMTFKPGKMTFLVGRSGSGKSTITN
80_8619   MGGGRKPRVFEGRIEVKSVSFAYPSNPAVQVLKPTSMTFKPGKMTFLVGRSGSGKSTITN
81_4126   MGGGRKPRVFEGRIEVKSVLKPTSMTFKPGKMTFLVGRSGSGKSTITNLLVKFYEPLSGE
82_1363   MGGGRKPRVFEGRIEVKSVSFAYPSNPAVQVLKPTSMTFKPGKMTFLVGRSGSGKSTITN
83_5813   MGGGRKPRVFEGRIEVKSVSFAYPSNPAVQVLKPTSMTFKPGKMTFLVGRSGSGKSTITN
84_2044   MGGGRKPRVFEGRIEVKSVSFAYPSNPAVQVLKPTSMTFKPGKMTFLVGRSGSGKSTITN
85_7617   MGGGRKPRVFEGRIEVKSVSFAYPSNPAVQVLKPTSMTFKPGKMTFLVGRSGSGKSTITN
86_1530   MGGGRKPRVFEGRIEVKSVSFAYPSNPAVQVLKPTSMTFKPGKMTFLVGRSGSGKSTITN
87_1890   MGGGRKPRVFEGRIEVKSVSFAYPSNPAVQVLKPTSMTFKPGKMTFLVGRSGSGKSTITN
88_7049   MGGGRKPRVFEGRIEVKSVSFAYPSNPAVQVLKPTSMTFKPGKMTFLVGRSGSGKSTITN
89_440    MGGGRKPRVFEGRIEVKSVSFAYPSNPAVQVLKPTSMTFKPGKMTFLVGRSGSGKSTITN
90_4331   MGGGRKPRVFEGRIEVKSVSFAYPSNPAVQVLKPTSMTFKPGKMTFLVGRSGSGKSTITN
```

```
                490       500       510       520       530       540
1_1896     LLVKFYEPLSGEILLDEHPLQTLNSEWVRKNVTLVQQQSTLFSDTLYANIAMNSENVSQD
2_4785     LLVKFYEPLSGEILLDEHPLQTLNSEWVRKNVTLVQQQSTLFSDTLYANIAMNSENVSQD
3_3653     LLVKFYEPLSGEILLDEHPLQTLNSEWVRKNVTLVQQQSTLFSDTLYANIAMNSENVSQD
4_8157     LLVKFYEPLSGEILLDEHPLQTLNSEWVRKNVTLVQQQSTLFSDTLYANIAMNSENVSQD
5_752      LLVKFYEPLSGEILLDEHPLQTLNSEWVRKNVTLVQQQSTLFSDTLYANIAMNSENVSQD
6_8853     LLVKFYEPLSGEILLDEHPLQTLNSEWVRKNVTLVQQQSTLFSDTLYANIAMNSENVSQD
7_4015     FVPIPGTNAAIYPTPNQPGSDAYLQLHQSPRHRHREQVRPNYRQDGEVAKPRRGSIDFIQ
8_2020     LLVKFYEPLSGEILLDEHPLQTLNSEWVRKNVTLVQQQSTLFSDTLYANIAMNSENVSQD
9_1829     LLVKFYEPLSGEILLDEHPLQTLNSEWVRKNVTLVQQQSTLFSDTLYANIAMNSENVSQD
10_6452    LLVKFYEPLSGEILLDEHPLQTLNSEWVRKNVTLVQQQSTLFSDTLYANIAMNSENVSQD
11_8627    LLVKFYEPLSGEILLDEHPLQTLNSEWVRKNVTLVQQQSTLFSDTLYANIAMNSENVSQD
12_6118    LLVKFYEPLSGEILLDEHPLQTLNSEWVRKNVTLVQQQSTLFSDTLYANIAMNSENVSQD
13_2688    LLVKFYEPLSGEILLDEHPLQTLNSEWVRKNVTLVQQQSTLFSDTLYANIAMNSENVSQD
14_6406    LLVKFYEPLSGEILLDEHPLQTLNSEWVRKNVTLVQQQSTLFSDTLYANIAMNSENVSQD
15_9419    LLVKFYEPLSGEILLDEHPLQTLNSEWVRKNVTLVQQQSTLFSDTLYANIAMNSENVSQD
16_8507    LLVKFYEPLSGEILLDEHPLQTLNSEWVRKNVTLVQQQSTLFSDTLYANIAMNSENVSQD
17_5884    LLVKFYEPLSGEILLDEHPLQTLSSEWVRKNVTLVQQQSTLFSDTLYANIAMNSENVSQD
18_2670    LLVKFYEPLSGEILLDEHPLQTLSSEWVRKNVTLVQQQSTLFSDTLYANIAMNSENVSQD
19_5172    LLVKFYEPLSGEILLDEHPLQTLNSEWVRKNVTLVQQQSTLFSDTLYANIAMNSENVSQD
20_8057    LLVKFYEPLSGEILLDEHPLQTLNSEWVRKNVTLVQQQSTLFSDTLYANIAMNSENVSQD
21_4920    WAQGSKLEAGTKWAIVLGCVGILDFTAVFFTFFLAGAVAQEWVDTLKVDAFFSILCQPRA
22_6393    LLVKFYEPLSGEILLDEHPLQTLSSEWVRKNVTLVQQQSTLFSDTLYANIAMNSENVSQD
23_8263    LLVKFYEPLSGEILLDEHPLQTLNSEWVRKNVTLVQQQSTLFSDTLYANIAMNSENVSQD
24_8248    LLVKFYEPLSGEILLDEHPLQTLSSEWVRKNVTLVQQQSTLFSDTLYANIAMNSENVSQD
25_6733    LLVKFYEPLSGEILLDEHPLQTLNSEWVRKNVTLVQQQSTLFSDTLYANIAMNSENVSQD
26_2277    LLVKFYEPLSGEILLDEHPLQTLNSEWVRKNVTLVQQQSTLFSDTLYANIAMNSENVSQD
27_2623    LLVKFYEPLSGEILLDEHPLQTLNSEWVRKNVTLVQQQSTLFSDTLYANIAMNSENVSQD
28_7413    LLVKFYEPLSGEILLDEHPLQTLNSEWVRKNVTLVQQQSTLFSDTLYANIAMNSENVSQD
29_8980    LLVKFYEPLSGEILLDEHPLQTLNSEWVRKNVTLVQQQSTLFSDTLYANIAMNSENVSQD
30_8706    LLVKFYEPLSGEILLDEHPLQTLSSEWVRKNVTLVQQQSTLFSDTLYANIAMNSENVSQD
31_6378    LLVKFYEPLSGEILLDEHPLQTLNSEWVRKNVTLVQQQSTLFSDTLYANIAMNSENVSQD
32_4679    LLVKFYEPLSGEILLDEHPLQTLSSEWVRKNVTLVQQQSTLFSDTLYANIAMNSENVSQD
33_2437    LLVKFYEPLSGEILLDEHPLQTLNSEWVRKNVTLVQQQSTLFSDTLYANIAMNSENVSQD
34_9700    LLVKFYEPLSGEILLDEHPLQTLSSEWVRKNVTLVQQQSTLFSDTLYANIAMNSENVSQD
35_10218   LLVKFYEPLSGEILLDEHPLQTLNSEWVRKNVTLVQQQSTLFSDTLYANIAMNSENVSQD
36_2652    LLVKFYEPLSGEILLDEHPLQTLSSEWVRKNVTLVQQQSTLFSDTLYANIAMNSENVSQD
37_5779    LLVKFYEPLSGEILLDEHPLQTLNSEWVRKNVTLVQQQSTLFSDTLYANIAMNSENVSQD
38_654     LLVKFYEPLSGEILLDEHPLQTLNSEWVRKNVTLVQQQSTLFSDTLYANIAMNSENVSQD
39_4879    LLVKFYEPLSGEILLDEHPLQTLSSEWVRKNVTLVQQQSTLFSDTLYANIAMNSENVSQD
40_8113    LLVKFYEPLSGEILLDEHPLQTLNSEWVRKNVTLVQQQSTLFSDTLYANIAMNSENVSQD
41_1767    ILLDEHPLQTLNSEWVRKNVTLVQQQSTLFSDTLYANIAMNSENVSQDKVKAACEMALLQ
42_8428    LLVKFYEPLSGEILLDEHPLQTLSSEWVRKNVTLVQQQSTLFSDTLYANIAMNSENVSQD
43_9819    LLVKFYEPLSGEILLDEHPLQTLNSEWVRKNVTLVQQQSTLFSDTLYANIAMNSENVSQD
44_5569    LLVKFYEPLSGEILLDEHPLQTLSSEWVRKNVTLVQQQSTLFSDTLYANIAMNSENVSQD
45_4189    LLVKFYEPLSGEILLDEHPLQTLNSEWVRKNVTLVQQQSTLFSDTLYANIAMNSENVSQD
46_1027    LLVKFYEPLSGEILLDEHPLQTLNSEWVRKNVTLVQQQSTLFSDTLYANIAMNSENVSQD
47_5048    LLVKFYEPLSGEILLDEHPLQTLNSEWVRKNVTLVQQQSTLFSDTLYANIAMNSENVSQD
48_10124   LLVKFYEPLSGEILLDEHPLQTLSSEWVRKNVTLVQQQSTLFSDTLYANIAMNSENVSQD
49_7151    LLVKFYEPLSGEILLDEHPLQTLSSEWVRKNVTLVQQQSTLFSDTLYANIAMNSENVSQD
50_3695    LLVKFYEPLSGEILLDEHPLQTLSSEWVRKNVTLVQQQSTLFSDTLYANIAMNSENVSQD
51_364     LLVKFYEPLSGEILLDEHPLQTLSSEWVRKNVTLVQQQSTLFSDTLYANIAMNSENVSQD
52_242     LLVKFYEPLSGEILLDEHPLQTLSSEWVRKNVTLVQQQSTLFSDTLYANIAMNSENVSQD
53_5225    LLVKFYEPLSGEILLDEHPLQTLSSEWVRKNVTLVQQQSTLFSDTLYANIAMNSENVSQD
54_1635    LLVKFYEPLSGEILLDEHPLQTLNSEWVRKNVTLVQQQSTLFSDTLYANIAMNSENVSQD
55_5766    LLVKFYEPLSGEILLDEHPLQTLNSEWVRKNVTLVQQQSTLFSDTLYANIAMNSENVSQD
56_1557    LLVKFYEPLSGEILLDEHPLQTLSSEWVRKNVTLVQQQSTLFSDTLYANIAMNSENVSQD
57_10137   LLVKFYEPLSGEILLDEHPLQTLNSEWVRKNVTLVQQQSTLFSDTLYANIAMNSENVSQD
58_10525   LLVKFYEPLSGEILLDEHPLQTLNSEWVRKNVTLVQQQSTLFSDTLYANIAMNSENVSQD
59_3457    LLVKFYEPLSGEILLDEHPLQTLSSEWVRKNVTLVQQQSTLFSDTLYANIAMNSENVSQD
60_8244    LLVKFYEPLSGEILLDEHPLQTLNSEWVRKNVTLVQQQSTLFSDTLYANIAMNSENVSQD
61_7964    LLVKFYEPLSGEILLDEHPLQTLNSEWVRKNVTLVQQQSTLFSDTLYANIAMNSENVSQD
62_4413    LLVKFYEPLSGEILLDEHPLQTLNSEWVRKNVTLVQQQSTLFSDTLYANIAMNSENVSQD
63_6958    LLVKFYEPLSGEILLDEHPLQTLNSEWVRKNVTLVQQQSTLFSDTLYANIAMNSENVSQD
64_5997    LDRGADEMRTIVAQFTPIALIVVIMVSSAIVWAMVILWQLTLVALAVFPFVGLCVFFSTR
65_8711    LLVKFYEPLSGEILLDEHPLQTLNSEWVRKNVTLVQQQSTLFSDTLYANIAMNSENVSQD
66_9925    LLVKFYEPLSGEILLDEHPLQTLSSEWVRKNVTLVQQQSTLFSDTLYANIAMNSENVSQD
67_10374   LLVKFYEPLSGEILLDEHPLQTLNSEWVRKNVTLVQQQSTLFSDTLYANIAMNSENVSQD
68_3713    LLVKFYEPLSGEILLDEHPLQTLNSEWVRKNVTLVQQQSTLFSDTLYANIAMNSENVSQD
69_4511    LLVKFYEPLSGEILLDEHPLQTLNSEWVRKNVTLVQQQSTLFSDTLYANIAMNSENVSQD
70_6201    LLVKFYEPLSGEILLDEHPLQTLNSEWVRKNVTLVQQQSTLFSDTLYANIAMNSENVSQD
71_3261    LLVKFYEPLSGEILLDEHPLQTLSSEWVRKNVTLVQQQSTLFSDTLYANIAMNSENVSQD
72_8516    LLVKFYEPLSGEILLDEHPLQTLSSEWVRKNVTLVQQQSTLFSDTLYANIAMNSENVSQD
73_9295    LLVKFYEPLSGEILLDEHPLQTLSSEWVRKNVTLVQQQSTLFSDTLYANIAMNSENVSQD
74_809     LLVKFYEPLSGEILLDEHPLQTLSSEWVRKNVTLVQQQSTLFSDTLYANIAMNSENVSQD
75_7282    LLVKFYEPLSGEILLDEHPLQTLSSEWVRKNVTLVQQQSTLFSDTLYANIAMNSENVSQD
76_4910    LLVKFYEPLSGEILLDEHPLQTLSSEWVRKNVTLVQQQSTLFSDTLYANIAMNSENVSQD
77_10912   LLVKFYEPLSGEILLDEHPLQTLNSEWVRKNVTLVQQQSTLFSDTLYANIAMNSENVSQD
78_4020    LLVKFYEPLSGEILLDEHPLQTLNSEWVRKNVTLVQQQSTLFSDTLYANIAMNSENVSQD
79_6703    LLVKFYEPLSGEILLDEHPLQTLNSEWVRKNVTLVQQQSTLFSDTLYANIAMNSENVSQD
80_8619    LLVKFYEPLSGEILLDEHPLQTLNSEWVRKNVTLVQQQSTLFSDTLYANIAMNSENVSQD
81_4126    ILLDEHPLQTLSSEWVRKNVTLVQQQSTLFSDTLYANIAMNSENVSQDKVKAACEMALLQ
82_1363    LLVKFYEPLSGEILLDEHPLQTLSSEWVRKNVTLVQQQSTLFSDTLYANIAMNSENVSQD
83_5813    LLVKFYEPLSGEILLDEHPLQTLSSEWVRKNVTLVQQQSTLFSDTLYANIAMNSENVSQD
84_2044    LLVKFYEPLSGEILLDEHPLQTLSSEWVRKNVTLVQQQSTLFSDTLYANIAMNSENVSQD
85_7617    LLVKFYEPLSGEILLDEHPLQTLNSEWVRKNVTLVQQQSTLFSDTLYANIAMNSENVSQD
86_1530    LLVKFYEPLSGEILLDEHPLQTLSSEWVRKNVTLVQQQSTLFSDTLYANIAMNSENVSQD
87_1890    LLVKFYEPLSGEILLDEHPLQTLSSEWVRKNVTLVQQQSTLFSDTLYANIAMNSENVSQD
88_7049    LLVKFYEPLSGEILLDEHPLQTLSSEWVRKNVTLVQQQSTLFSDTLYANIAMNSENVSQD
89_440     LLVKFYEPLSGEILLDEHPLQTLNSEWVRKNVTLVQQQSTLFSDTLYANIAMNSENVSQD
90_4331    LLVKFYEPLSGEILLDEHPLQTLNSEWVRKNVTLVQQQSTLFSDTLYANIAMNSENVSQD
```

```
                550       560       570       580       590       600
1_1896     KVKAACEMALLQSTIAGLPQGLDTLVGPGGQSLSGGQRQRVALARARLRDTPVLILDEVT
2_4785     KVKAACEMALLQSTIAGLPQGLDTLVGPGGQSLSGGQRQRVALARARLRDTPVLILDEVT
3_3653     KVKAACEMALLQSTIAGLPQGLDTLVGPGGQSLSGGQRQRVALARARLRDTPVLILDEVT
4_8157     KVKAACEMALLQSTIAGLPQGLDTLVGPGGQSLSGGQRQRVALARARLRDTPVLILDEVT
5_752      KVKAACEMALLQSTIAGLPQGLDTLVGPGGQSLSGGQRQRVALARARLRDTPVLILDEVT
6_8853     KVKAACEMALLQSTIAGLPQGLDTLVGPGGQSLSGGQRQRVALARARLRDTPVLILDEVT
7_4015     ARGLSALANRSPAKRSVPQGSGIGDVLRRLSTRSSPIEDVEMSPVRENKKGVKGVMRKKM
8_2020     KVKAACEMALLQSTIAGLPQGLDTLVGPGGQSLSGGQRQRVALARARLRDTPVLILDEVT
9_1829     KVKAACEMALLQSTIAGLPQGLDTLVGPGGQSLSGGQRQRVALARARLRDTPVLILDEVT
10_6452    KVKAACEMALLQSTIAGLPQGLDTLVGPGGQSLSGGQRQRVALARARLRDTPVLILDEVT
11_8627    KVKAACEMALLQSTIAGLPQGLDTLVGPGGQSLSGGQRQRVALARARLRDTPVLILDEVT
12_6118    KVKAACEMALLQSTIAGLPQGLDTLVGPGGQSLSGGQRQRVALARARLRDTPVLILDEVT
13_2688    KVKAACEMALLQSTIAGLPQGLDTLVGPGGQSLSGGQRQRVALARARLRDTPVLILDEVT
14_6406    KVKAACEMALLQSTIAGLPQGLDTLVGPGGQSLSGGQRQRVALARARLRDTPVLILDEVT
15_9419    KVKAACEMALLQSTIAGLPQGLDTLVGPGGQSLSGGQRQRVALARARLRDTPVLILDEVT
16_8507    KVKAACEMALLQSTIAGLPQGLDTLVGPGGQSLSGGQRQRVALARARLRDTPVLILDEVT
17_5884    KVKAACEMALLQSTIAGLPQGLDTLVGPGGQSLSGGQRQRVALARARLRDTPVLILDEVT
18_2670    KVKAACEMALLQSTIAGLPQGLDTLVGPGGQSLSGGQRQRVALARARLRDTPVLILDEVT
19_5172    KVKAACEMALLQSTIAGLPQGLDTLVGPGGQSLSGGQRQRVALARARLRDTPVLILDEVT
20_8057    KVKAACEMALLQSTIAGLPQGLDTLVGPGGQSLSGGQRQRVALARARLRDTPVLILDEVT
21_4920    WHDRSKNSAARVCDVLDRGADEMRTIVAQFTPIALIVVIMVSSAIVWAMVILWQLTLVAL
22_6393    KVKAACEMALLQSTIAGLPQGLDTLVGPGGQSLSGGQRQRVALARARLRDTPVLILDEVT
23_8263    KVKAACEMALLQSTIAGLPQGLDTLVGPGGQSLSGGQRQRVALARARLRDTPVLILDEVT
24_8248    KVKAACEMALLQSTIAGLPQGLDTLVGPGGQSLSGGQRQRVALARARLRDTPVLILDEVT
25_6733    KVKAACEMALLQSTIAGLPQGLDTLVGPGGQSLSGGQRQRVALARARLRDTPVLILDEVT
26_2277    KVKAACEMALLQSTIAGLPQGLDTLVGPGGQSLSGGQRQRVALARARLRDTPVLILDEVT
27_2623    KVKAACEMALLQSTIAGLPQGLDTLVGPGGQSLSGGQRQRVALARARLRDTPVLILDEVT
28_7413    KVKAACEMALLQSTIAGLPQGLDTLVGPGGQSLSGGQRQRVALARARLRDTPVLILDEVT
29_8980    KVKAACEMALLQSTIAGLPQGLDTLVGPGGQSLSGGQRQRVALARARLRDTPVLILDEVT
30_8706    KVKAACEMALLQSTIAGLPQGLDTLVGPGGQSLSGGQRQRVALARARLRDTPVLILDEVT
31_6378    KVKAACEMALLQSTIAGLPQGLDTLVGPGGQSLSGGQRQRVALARARLRDTPVLILDEVT
32_4679    KVKAACEMALLQSTIAGLPQGLDTLVGPGGQSLSGGQRQRVALARARLRDTPVLILDEVT
33_2437    KVKAACEMALLQSTIAGLPQGLDTLVGPGGQSLSGGQRQRVALARARLRDTPVLILDEVT
34_9700    KVKAACEMALLQSTIAGLPQGLDTLVGPGGQSLSGGQRQRVALARARLRDTPVLILDEVT
35_10218   KVKAACEMALLQSTIAGLPQGLDTLVGPGGQSLSGGQRQRVALARARLRDTPVLILDEVT
36_2652    KVKAACEMALLQSTIAGLPQGLDTLVGPGGQSLSGGQRQRVALARARLRDTPVLILDEVT
37_5779    KVKAACEMALLQSTIAGLPQGLDTLVGPGGQSLSGGQRQRVALARARLRDTPVLILDEVT
38_654     KVKAACEMALLQSTIAGLPQGLDTLVGPGGQSLSGGQRQRVALARARLRDTPVLILDEVT
39_4879    KVKAACEMALLQSTIAGLPQGLDTLVGPGGQSLSGGQRQRVALARARLRDTPVLILDEVT
40_8113    KVKAACEMALLQSTIAGLPQGLDTLVGPGGQSLSGGQRQRVALARARLRDTPVLILDEVT
41_1767    STIAGLPQGLDTLVGPGGQSLSGGQRQRVALARARLRDTPVLILDEVTSGLDPKSKLMIM
42_8428    KVKAACEMALLQSTIAGLPQGLDTLVGPGGQSLSGGQRQRVALARARLRDTPVLILDEVT
43_9819    KVKAACEMALLQSTIAGLPQGLDTLVGPGGQSLSGGQRQRVALARARLRDTPVLILDEVT
44_5569    KVKAACEMALLQSTIAGLPQGLDTLVGPGGQSLSGGQRQRVALARARLRDTPVLILDEVT
45_4189    KVKAACEMALLQSTIAGLPQGLDTLVGPGGQSLSGGQRQRVALARARLRDTPVLILDEVT
46_1027    KVKAACEMALLQSTIAGLPQGLDTLVGPGGQSLSGGQRQRVALARARLRDTPVLILDEVT
47_5048    KVKAACEMALLQSTIAGLPQGLDTLVGPGGQSLSGGQRQRVALARARLRDTPVLILDEVT
48_10124   KVKAACEMALLQSTIAGLPQGLDTLVGPGGQSLSGGQRQRVALARARLRDTPVLILDEVT
49_7151    KVKAACEMALLQSTIAGLPQGLDTLVGPGGQSLSGGQRQRVALARARLRDTPVLILDEVT
50_3695    KVKAACEMALLQSTIAGLPQGLDTLVGPGGQSLSGGQRQRVALARARLRDTPVLILDEVT
51_364     KVKAACEMALLQSTIAGLPQGLDTLVGPGGQSLSGGQRQRVALARARLRDTPVLILDEVT
52_242     KVKAACEMALLQSTIAGLPQGLDTLVGPGGQSLSGGQRQRVALARARLRDTPVLILDEVT
53_5225    KVKAACEMALLQSTIAGLPQGLDTLVGPGGQSLSGGQRQRVALARARLRDTPVLILDEVT
54_1635    KVKAACEMALLQSTIAGLPQGLDTLVGPGGQSLSGGQRQRVALARARLRDTPVLILDEVT
55_5766    KVKAACEMALLQSTIAGLPQGLDTLVGPGGQSLSGGQRQRVALARARLRDTPVLILDEVT
56_1557    KVKAACEMALLQSTIAGLPQGLDTLVGPGGQSLSGGQRQRVALARARLRDTPVLILDEVT
57_10137   KVKAACEMALLQSTIAGLPQGLDTLVGPGGQSLSGGQRQRVALARARLRDTPVLILDEVT
58_10525   KVKAACEMALLQSTIAGLPQGLDTLVGPGGQSLSGGQRQRVALARARLRDTPVLILDEVT
59_3457    KVKAACEMALLQSTIAGLPQGLDTLVGPGGQSLSGGQRQRVALARARLRDTPVLILDEVT
60_8244    KVKAACEMALLQSTIAGLPQGLDTLVGPGGQSLSGGQRQRVALARARLRDTPVLILDEVT
61_7964    KVKAACEMALLQSTIAGLPQGLDTLVGPGGQSLSGGQRQRVALARARLRDTPVLILDEVT
62_4413    KVKAACEMALLQSTIAGLPQGLDTLVGPGGQSLSGGQRQRVALARARLRDTPVLILDEVT
63_6958    KVKAACEMALLQSTIAGLPQGLDTLVGPGGQSLSGGQRQRVALARARLRDTPVLILDEVT
64_5997    TSEKWEALSNESAEATGGILSSVVSDIRVVRAFLLEKFFGDRFEAAAERAFTIGKKRGLY
65_8711    KVKAACEMALLQSTIAGLPQGLDTLVGPGGQSLSGGQRQRVALARARLRDTPVLILDEVT
66_9925    KVKAACEMALLQSTIAGLPQGLDTLVGPGGQSLSGGQRQRVALARARLRDTPVLILDEVT
67_10374   KVKAACEMALLQSTIAGLPQGLDTLVGPGGQSLSGGQRQRVALARARLRDTPVLILDEVT
68_3713    KVKAACEMALLQSTIAGLPQGLDTLVGPGGQSLSGGQRQRVALARARLRDTPVLILDEVT
69_4511    KVKAACEMALLQSTIAGLPQGLDTLVGPGGQSLSGGQRQRVALARARLRDTPVLILDEVT
70_6201    KVKAACEMALLQSTIAGLPQGLDTLVGPGGQSLSGGQRQRVALARARLRDTPVLILDEVT
71_3261    KVKAACEMALLQSTIAGLPQGLDTLVGPGGQSLSGGQRQRVALARARLRDTPVLILDEVT
72_8516    KVKAACEMALLQSTIAGLPQGLDTLVGPGGQSLSGGQRQRVALARARLRDTPVLILDEVT
73_9295    KVKAACEMALLQSTIAGLPQGLDTLVGPGGQSLSGGQRQRVALARARLRDTPVLILDEVT
74_809     KVKAACEMALLQSTIAGLPQGLDTLVGPGGQSLSGGQRQRVALARARLRDTPVLILDEVT
75_7282    KVKAACEMALLQSTIAGLPQGLDTLVGPGGQSLSGGQRQRVALARARLRDTPVLILDEVT
76_4910    KVKAACEMALLQSTIAGLPQGLDTLVGPGGQSLSGGQRQRVALARARLRDTPVLILDEVT
77_10912   KVKAACEMALLQSTIAGLPQGLDTLVGPGGQSLSGGQRQRVALARARLRDTPVLILDEVT
78_4020    KVKAACEMALLQSTIAGLPQGLDTLVGPGGQSLSGGQRQRVALARARLRDTPVLILDEVT
79_6703    KVKAACEMALLQSTIAGLPQGLDTLVGPGGQSLSGGQRQRVALARARLRDTPVLILDEVT
80_8619    KVKAACEMALLQSTIAGLPQGLDTLVGPGGQSLSGGQRQRVALARARLRDTPVLILDEVT
81_4126    STIAGLPQGLDTLVGPGGQSLSGGQRQRVALARARLRDTPVLILDEVTSGLDPKSKLMIM
82_1363    KVKAACEMALLQSTIAGLPQGLDTLVGPGGQSLSGGQRQRVALARARLRDTPVLILDEVT
83_5813    KVKAACEMALLQSTIAGLPQGLDTLVGPGGQSLSGGQRQRVALARARLRDTPVLILDEVT
84_2044    KVKAACEMALLQSTIAGLPQGLDTLVGPGGQSLSGGQRQRVALARARLRDTPVLILDEVT
85_7617    KVKAACEMALLQSTIAGLPQGLDTLVGPGGQSLSGGQRQRVALARARLRDTPVLILDEVT
86_1530    KVKAACEMALLQSTIAGLPQGLDTLVGPGGQSLSGGQRQRVALARARLRDTPVLILDEVT
87_1890    KVKAACEMALLQSTIAGLPQGLDTLVGPGGQSLSGGQRQRVALARARLRDTPVLILDEVT
88_7049    KVKAACEMALLQSTIAGLPQGLDTLVGPGGQSLSGGQRQRVALARARLRDTPVLILDEVT
89_440     KVKAACEMALLQSTIAGLPQGLDTLVGPGGQSLSGGQRQRVALARARLRDTPVLILDEVT
90_4331    KVKAACEMALLQSTIAGLPQGLDTLVGPGGQSLSGGQRQRVALARARLRDTPVLILDEVT
```

```
              610       620       630       640       650       660
1_1896    SGLDPKSKLMIMDAIRYWRSGRTTIVITHDVSQVGDDDYVYVMDKAEVREEGLCKQLLSS
2_4785    SGLDPKSKLMIMDAIRYWRSGRTTIVITHDVSQVGDDDYVYVMDKAEVREEGLCKQLLSS
3_3653    SGLDPKSKLMIMDAIRYWRSGRTTIVITHDVSQVGDDDYVYVMDKAEVREEGLCKQLLSS
4_8157    SGLDPKSKLMIMDAIRYWRSGRTTIVITHDVSQVGDDDYVYVMDKAEVREEGLCKQLLSS
5_752     SGLDPKSKLMIMDAIRYWRSGRTTIVITHDVSQVGDDDYVYVMDKAEVREEGLCKQLLSS
6_8853    SGLDPKSKLMIMDAIRYWRSGRTTIVITHDVSQVGDDDYVYVMDKAEVREEGLCKQLLSS
7_4015    KNVMGKGARGGKRGMKDGTNEEAKEETTSTTEQMSTYQILKTVWPALDAKHRLYAVVALF
8_2020    SGLDPKSKLMIMDAIRYWRSGRTTIVITHDVSQVGDDDYVYVMDKAEVREEGLCKQLLSS
9_1829    SGLDPKSKLMIMDAIRYWRSGRTTIVITHDVSQVGDDDYVYVMDKAEVREEGLCKQLLSS
10_6452   SGLDPKSKLMIMDAIRYWRSGRTTIVITHDVSQVGDDDYVYVMDKAEVREEGLCKQLLSS
11_8627   SGLDPKSKLMIMDAIRYWRSGRTTIVITHDVSQVGDDDYVYVMDKAEVREEGLCKQLLSS
12_6118   SGLDPKSKLMIMDAIRYWRSGRTTIVITHDVSQVGDDDYVYVMDKAEVREEGLCKQLLSS
13_2688   SGLDPKSKLMIMDAIRYWRSGRTTIVITHDVSQVGDDDYVYVMDKAEVREEGLCKQLLSS
14_6406   SGLDPKSKLMIMDAIRYWRSGRTTIVITHDVSQVGDDDYVYVMDKAEVREEGLCKQLLSS
15_9419   SGLDPKSKLMIMDAIRYWRSGRTTIVITHDVSQVGDDDYVYVMDKAEVREEGLCKQLLSS
16_8507   SGLDPKSKLMIMDAIRYWRSGRTTIVITHDVSQVGDDDYVYVMDKAEVREEGLCKQLLSS
17_5884   SGLDPKSKLMIMDAIRYWRSGRTTIVITHDVSQVGDDDYVYVMDKAEVREEGLCKQLLSS
18_2670   SGLDPKSKLMIMDAIRYWRSGRTTIVITHDVSQVGDDDYVYVMDKAEVREEGLCKQLLSS
19_5172   SGLDPKSKLMIMDAIRYWRSGRTTIVITHDVSQVGDDDYVYVMDKAEVREEGLCKQLLSS
20_8057   SGLDPKSKLMIMDAIRYWRSGRTTIVITHDVSQVGDDDYVYVMDKAEVREEGLCKQLLSS
21_4920   AVFPFVGLCVFFSTRTSEKWEALSNESAEATGGILSSVVSDIRVVRAFLLEKFFGDRFEA
22_6393   SGLDPKSKLMIMDAIRYWRSGRTTIVITHDVSQVGDDDYVYVMDKAEVREEGLCKQLLSS
23_8263   SGLDPKSKLMIMDAIRYWRSGRTTIVITHDVSQVGDDDYVYVMDKAEVREEGLCKQLLSS
24_8248   SGLDPKSKLMIMDAIRYWRSGRTTIVITHDVSQVGDDDYVYVMDKAEVREEGLCKQLLSS
25_6733   SGLDPKSKLMIMDAIRYWRSGRTTIVITHDVSQVGDDDYVYVMDKAEVREEGLCKQLLSS
26_2277   SGLDPKSKLMIMDAIRYWRSGRTTIVITHDVSQVGDDDYVYVMDKAEVREEGLCKQLLSS
27_2623   SGLDPKSKLMIMDAIRYWRSGRTTIVITHDVSQVGDDDYVYVMDKAEVREEGLCKQLLSS
28_7413   SGLDPKSKLMIMDAIRYWRSGRTTIVITHDVSQVGDDDYVYVMDKAEVREEGLCKQLLSS
29_8980   SGLDPKSKLMIMDAIRYWRSGRTTIVITHDVSQVGDDDYVYVMDKAEVREEGLCKQLLSS
30_8706   SGLDPKSKLMIMDAIRYWRSGRTTIVITHDVSQVGDDDYVYVMDKAEVREEGLCKQLLSS
31_6378   SGLDPKSKLMIMDAIRYWRSGRTTIVITHDVSQVGDDDYVYVMDKAEVREEGLCKQLLSS
32_4679   SGLDPKSKLMIMDAIRYWRSGRTTIVITHDVSQVGDDDYVYVMDKAEVREEGLCKQLLSS
33_2437   SGLDPKSKLMIMDAIRYWRSGRTTIVITHDVSQVGDDDYVYVMDKAEVREEGLCKQLLSS
34_9700   SGLDPKSKLMIMDAIRYWRSGRTTIVITHDVSQVGDDDYVYVMDKAEVREEGLCKQLLSS
35_10218  SGLDPKSKLMIMDAIRYWRSGRTTIVITHDVSQVGDDDYVYVMDKAEVREEGLCKQLLSS
36_2652   SGLDPKSKLMIMDAIRYWRSGRTTIVITHDVSQVGDDDYVYVMDKAEVREEGLCKQLLSS
37_5779   SGLDPKSKLMIMDAIRYWRSGRTTIVITHDVSQVGDDDYVYVMDKAEVREEGLCKQLLSS
38_654    SGLDPKSKLMIMDAIRYWRSGRTTIVITHDVSQVGDDDYVYVMDKAEVREEGLCKQLLSS
39_4879   SGLDPKSKLMIMDAIRYWRSGRTTIVITHDVSQVGDDDYVYVMDKAEVREEGLCKQLLSS
40_8113   SGLDPKSKLMIMDAIRYWRSGRTTIVITHDVSQVGDDDYVYVMDKAEVREEGLCKQLLSS
41_1767   DAIRYWRSGRTTIVITHDVSQVGDDDYVYVMDKAEVREEGLCKQLLSSRDGYFLQLRALA
42_8428   SGLDPKSKLMIMDAIRYWRSGRTTIVITHDVSQVGDDDYVYVMDKAEVREEGLCKQLLSS
43_9819   SGLDPKSKLMIMDAIRYWRSGRTTIVITHDVSQVGDDDYVYVMDKAEVREEGLCKQLLSS
44_5569   SGLDPKSKLMIMDAIRYWRSGRTTIVITHDVSQVGDDDYVYVMDKAEVREEGLCKQLLSS
45_4189   SGLDPKSKLMIMDAIRYWRSGRTTIVITHDVSQVGDDDYVYVMDKAEVREEGLCKQLLSS
46_1027   SGLDPKSKLMIMDAIRYWRSGRTTIVITHDVSQVGDDDYVYVMDKAEVREEGLCKQLLSS
47_5048   SGLDPKSKLMIMDAIRYWRSGRTTIVITHDVSQVGDDDYVYVMDKAEVREEGLCKQLLSS
48_10124  SGLDPKSKLMIMDAIRYWRSGRTTIVITHDVSQVGDDDYVYVMDKAEVREEGLCKQLLSS
49_7151   SGLDPKSKLMIMDAIRYWRSGRTTIVITHDVSQVGDDDYVYVMDKAEVREEGLCKQLLSS
50_3695   SGLDPKSKLMIMDAIRYWRSGRTTIVITHDVSQVGDDDYVYVMDKAEVREEGLCKQLLSS
51_364    SGLDPKSKLMIMDAIRYWRSGRTTIVITHDVSQVGDDDYVYVMDKAEVREEGLCKQLLSS
52_242    SGLDPKSKLMIMDAIRYWRSGRTTIVITHDVSQVGDDDYVYVMDKAEVREEGLCKQLLSS
53_5225   SGLDPKSKLMIMDAIRYWRSGRTTIVITHDVSQVGDDDYVYVMDKAEVREEGLCKQLLSS
54_1635   SGLDPKSKLMIMDAIRYWRSGRTTIVITHDVSQVGDDDYVYVMDKAEVREEGLCKQLLSS
55_5766   SGLDPKSKLMIMDAIRYWRSGRTTIVITHDVSQVGDDDYVYVMDKAEVREEGLCKQLLSS
56_1557   SGLDPKSKLMIMDAIRYWRSGRTTIVITHDVSQVGDDDYVYVMDKAEVREEGLCKQLLSS
57_10137  SGLDPKSKLMIMDAIRYWRSGRTTIVITHDVSQVGDDDYVYVMDKAEVREEGLCKQLLSS
58_10525  SGLDPKSKLMIMDAIRYWRSGRTTIVITHDVSQVGDDDYVYVMDKAEVREEGLCKQLLSS
59_3457   SGLDPKSKLMIMDAIRYWRSGRTTIVITHDVSQVGDDDYVYVMDKAEVREEGLCKQLLSS
60_8244   SGLDPKSKLMIMDAIRYWRSGRTTIVITHDVSQVGDDDYVYVMDKAEVREEGLCKQLLSS
61_7964   SGLDPKSKLMIMDAIRYWRSGRTTIVITHDVSQVGDDDYVYVMDKAEVREEGLCKQLLSS
62_4413   SGLDPKSKLMIMDAIRYWRSGRTTIVITHDVSQVGDDDYVYVMDKAEVREEGLCKQLLSS
63_6958   SGLDPKSKLMIMDAIRYWRSGRTTIVITHDVSQVGDDDYVYVMDKAEVREEGLCKQLLSS
64_5997   TGIWGGIHCSISQWLVVVIFTFGVFLLTTAESVNVGDIIQVVNLLMFTVGSATMMMSNIP
65_8711   SGLDPKSKLMIMDAIRYWRSGRTTIVITHDVSQVGDDDYVYVMDKAEVREEGLCKQLLSS
66_9925   SGLDPKSKLMIMDAIRYWRSGRTTIVITHDVSQVGDDDYVYVMDKAEVREEGLCKQLLSS
67_10374  SGLDPKSKLMIMDAIRYWRSGRTTIVITHDVSQVGDDDYVYVMDKAEVREEGLCKQLLSS
68_3713   SGLDPKSKLMIMDAIRYWRSGRTTIVITHDVSQVGDDDYVYVMDKAEVREEGLCKQLLSS
69_4511   SGLDPKSKLMIMDAIRYWRSGRTTIVITHDVSQVGDDDYVYVMDKAEVREEGLCKQLLSS
70_6201   SGLDPKSKLMIMDAIRYWRSGRTTIVITHDVSQVGDDDYVYVMDKAEVREEGLCKQLLSS
71_3261   SGLDPKSKLMIMDAIRYWRSGRTTIVITHDVSQVGDDDYVYVMDKAEVREEGLCKQLLSS
72_8516   SGLDPKSKLMIMDAIRYWRSGRTTIVITHDVSQVGDDDYVYVMDKAEVREEGLCKQLLSS
73_9295   SGLDPKSKLMIMDAIRYWRSGRTTIVITHDVSQVGDDDYVYVMDKAEVREEGLCKQLLSS
74_809    SGLDPKSKLMIMDAIRYWRSGRTTIVITHDVSQVGDDDYVYVMDKAEVREEGLCKQLLSS
75_7282   SGLDPKSKLMIMDAIRYWRSGRTTIVITHDVSQVGDDDYVYVMDKAEVREEGLCKQLLSS
76_4910   SGLDPKSKLMIMDAIRYWRSGRTTIVITHDVSQVGDDDYVYVMDKAEVREEGLCKQLLSS
77_10912  SGLDPKSKLMIMDAIRYWRSGRTTIVITHDVSQVGDDDYVYVMDKAEVREEGLCKQLLSS
78_4020   SGLDPKSKLMIMDAIRYWRSGRTTIVITHDVSQVGDDDYVYVMDKAEVREEGLCKQLLSS
79_6703   SGLDPKSKLMIMDAIRYWRSGRTTIVITHDVSQVGDDDYVYVMDKAEVREEGLCKQLLSS
80_8619   SGLDPKSKLMIMDAIRYWRSGRTTIVITHDVSQVGDDDYVYVMDKAEVREEGLCKQLLSS
81_4126   DAIRYWRSGRTTIVITHDVSQVGDDDYVYVMDKAEVREEGLCKQLLSSRDGYFLQLRALA
82_1363   SGLDPKSKLMIMDAIRYWRSGRTTIVITHDVSQVGDDDYVYVMDKAEVREEGLCKQLLSS
83_5813   SGLDPKSKLMIMDAIRYWRSGRTTIVITHDVSQVGDDDYVYVMDKAEVREEGLCKQLLSS
84_2044   SGLDPKSKLMIMDAIRYWRSGRTTIVITHDVSQVGDDDYVYVMDKAEVREEGLCKQLLSS
85_7617   SGLDPKSKLMIMDAIRYWRSGRTTIVITHDVSQVGDDDYVYVMDKAEVREEGLCKQLLSS
86_1530   SGLDPKSKLMIMDAIRYWRSGRTTIVITHDVSQVGDDDYVYVMDKAEVREEGLCKQLLSS
87_1890   SGLDPKSKLMIMDAIRYWRSGRTTIVITHDVSQVGDDDYVYVMDKAEVREEGLCKQLLSS
88_7049   SGLDPKSKLMIMDAIRYWRSGRTTIVITHDVSQVGDDDYVYVMDKAEVREEGLCKQLLSS
89_440    SGLDPKSKLMIMDAIRYWRSGRTTIVITHDVSQVGDDDYVYVMDKAEVREEGLCKQLLSS
90_4331   SGLDPKSKLMIMDAIRYWRSGRTTIVITHDVSQVGDDDYVYVMDKAEVREEGLCKQLLSS
```

```
                 670       680       690       700       710       720
1_1896     RDGYFLQLRALAESGADSNGPEGVMTPEDDLSSLSSSEDELDVDMSGKSFYIPEPKTTSS
2_4785     RDGYFLQLRALAESGADSNGPEGVMTPEDDLSSLSSSEDELDVDMSGKSFYIPEPKTTSS
3_3653     RDGYFLQLRALAESGADSNGPEGVMTPEDDLSSLSSSEDELDVDMSGKSFYIPEPKTTSS
4_8157     RDGYFLQLRALAESGADSNGPEGVMTPEDDLSSLSSSEDELDVDMSGKSFYIPEPKTTSS
5_752      RDGYFLQLRALAESGADSNGPEGVMTPEDDLSSLSSSEDELDVDMSGKSFYIPEPKTTSS
6_8853     RDGYFLQLRALAESGADSNGPEGVMTPEDDLSSLSSSEDELDVDMSGKSFYIPEPKTTSS
7_4015     WCIVAAGCSPVFAFVFSNLLQAFWAQGSKLEAGTKWAIVLGCVGILDFTAVFFTFSLAGA
8_2020     RDGYFLQLRALAESGADSNGPEGVMTPEDDLSSLSSSEDELDVDMSGKSFYIPEPKTTSS
9_1829     RDGYFLQLRALAESGADSNGPEGVMTPEDDLSSLSSSEDELDVDMSGKSFYIPEPKTTSS
10_6452    RDGYFLQLRALAESGADSNGPEGVMTPEDDLSSLSSSEDELDVDMSGKSFYIPEPKTTSS
11_8627    RDGYFLQLRALAESGADSNGPEGVMTPEDDLSSLSSSEDELDVDMSGKSFYIPEPKTTSS
12_6118    RDGYFLQLRALAESGADSNGPEGVMTPEDDLSSLSSSEDELDVDMSGKSFYIPEPKTTSS
13_2688    RDGYFLQLRALAESGADSNGPEGVMTPEDDLSSLSSSEDELDVDMSGKSFYIPEPKTTSS
14_6406    RDGYFLQLRALAESGADSNGPEGVMTPEDDLSSLSSSEDELDVDMSGKSFYIPEPKTTSS
15_9419    RDGYFLQLRALAESGADSNGPEGVMTPEDDLSSLSSSEDELDVDMSGKSFYIPEPKTTSS
16_8507    RDGYFLQLRALAESGADSNGPEGVMTPEDDLSSLSSSEDELDVDMSGKSFYIPEPKTTSS
17_5884    RDGYFLQLRALAESGADSNGPEGVMTPEDDLSSLSSSEDELDVDMSGKSFYIPEPKTTSS
18_2670    RDGYFLQLRALAESGADSNGPEGVMTPEDDLSSLSSSEDELDVDMSGKSFYIPEPKTTSS
19_5172    RDGYFLQLRALAESGADSNGPEGVMTPEDDLSSLSSSEDELDVDMSGKSFYIPEPKTTSS
20_8057    RDGYFLQLRALAESGADSNGPEGVMTPEDDLSSLSSSEDELDVDMSGKSFYIPEPKTTSS
21_4920    AAERAFTIGKKRGLYTGIWGGIHCSISQWLVVVIFTFGVFLLTTAESVNVGDIIQVVNLL
22_6393    RDGYFLQLRALAESGADSNGPEGVMTPEDDLSSLSSSEDELDVDMSGKSFYIPEPKTTSS
23_8263    RDGYFLQLRALAESGADSNGPEGVMTPEDDLSSLSSSEDELDVDMSGKSFYIPEPKTTSS
24_8248    RDGYFLQLRALAESGADSNGPEGVMTPEDDLSSLSSSEDELDVDMSGKSFYIPEPKTTSS
25_6733    RDGYFLQLRALAESGADSNGPEGVMTPEDDLSSLSSSEDELDVDMSGKSFYIPEPKTTSS
26_2277    RDGYFLQLRALAESGADSNGPEGVMTPEDDLSSLSSSEDELDVDMSGKSFYIPEPKTTSS
27_2623    RDGYFLQLRALAESGADSNGPEGVMTPEDDLSSLSSSEDELDVDMSGKSFYIPEPKTTSS
28_7413    RDGYFLQLRALAESGADSNGPEGVMTPEDDLSSLSSSEDELDVDMSGKSFYIPEPKTTSS
29_8980    RDGYFLQLRALAESGADSNGPEGVMTPEDDLSSLSSSEDELDVDMSGKSFYIPEPKTTSS
30_8706    RDGYFLQLRALAESGADSNGPEGVMTPEDDLSSLSSSEDELDVDMSGKSFYIPEPKTTSS
31_6378    RDGYFLQLRALAESGADSNGPEGVMTPEDDLSSLSSSEDELDVDMSGKSFYIPEPKTTSS
32_4679    RDGYFLQLRALAESGADSNGPEGVMTPEDDLSSLSSSEDELDVDMSGKSFYIPEPKTTSS
33_2437    RDGYFLQLRALAESGADSNGPEGVMTPEDDLSSLSSSEDELDVDMSGKSFYIPEPKTTSS
34_9700    RDGYFLQLRALAESGADSNGPEGVMTPEDDLSSLSSSEDELDVDMSGKSFYIPEPKTTSS
35_10218   RDGYFLQLRALAESGADSNGPEGVMTPEDDLSSLSSSEDELDVDMSGKSFYIPEPKTTSS
36_2652    RDGYFLQLRALAESGADSNGPEGVMTPEDDLSSLSSSEDELDVDMSGKSFYIPEPKTTSS
37_5779    RDGYFLQLRALAESGADSNGPEGVMTPEDDLSSLSSSEDELDVDMSGKSFYIPEPKTTSS
38_654     RDGYFLQLRALAESGADSNGPEGVMTPEDDLSSLSSSEDELDVDMSGKSFYIPEPKTTSS
39_4879    RDGYFLQLRALAESGADSNGPEGVMTPEDDLSSLSSSEDELDVDMSGKSFYIPEPKTTSS
40_8113    RDGYFLQLRALAESGADSNGPEGVMTPEDDLSSLSSSEDELDVDMSGKSFYIPEPKTTSS
41_1767    ESGADSNGPEGVMTPEDDLSSLSSSEDELDVDMSGKSFYIPEPKTTSSFGALGRMSFVPI
42_8428    RDGYFLQLRALAESGADSNGPEGVMTPEDDLSSLSSSEDELDVDMSGKSFYIPEPKTTSS
43_9819    RDGYFLQLRALAESGADSNGPEGVMTPEDDLSSLSSSEDELDVDMSGKSFYIPEPKTTSS
44_5569    RDGYFLQLRALAESGADSNGPEGVMTPEDDLSSLSSSEDELDVDMSGKSFYIPEPKTTSS
45_4189    RDGYFLQLRALAESGADSNGPEGVMTPEDDLSSLSSSEDELDVDMSGKSFYIPEPKTTSS
46_1027    RDGYFLQLRALAESGADSNGPEGVMTPEDDLSSLSSSEDELDVDMSGKSFYIPEPKTTSS
47_5048    RDGYFLQLRALAESGADSNGPEGVMTPEDDLSSLSSSEDELDVDMSGKSFYIPEPKTTSS
48_10124   RDGYFLQLRALAESGADSNGPEGVMTPEDDLSSLSSSEDELDVDMSGKSFYIPEPKTTSS
49_7151    RDGYFLQLRALAESGADSNGPEGVMTPEDDLSSLSSSEDELDVDMSGKSFYIPEPKTTSS
50_3695    RDGYFLQLRALAESGADSNGPEGVMTPEDDLSSLSSSEDELDVDMSGKSFYIPEPKTTSS
51_364     RDGYFLQLRALAESGADSNGPEGVMTPEDDLSSLSSSEDELDVDMSGKSFYIPEPKTTSS
52_242     RDGYFLQLRALAESGADSNGPEGVMTPEDDLSSLSSSEDELDVDMSGKSFYIPEPKTTSS
53_5225    RDGYFLQLRALAESGADSNGPEGVMTPEDDLSSLSSSEDELDVDMSGKSFYIPEPKTTSS
54_1635    RDGYFLQLRALAESGADSNGPEGVMTPEDDLSSLSSSEDELDVDMSGKSFYIPEPKTTSS
55_5766    RDGYFLQLRALAESGADSNGPEGVMTPEDDLSSLSSSEDELDVDMSGKSFYIPEPKTTSS
56_1557    RDGYFLQLRALAESGADSNGPEGVMTPEDDLSSLSSSEDELDVDMSGKSFYIPEPKTTSS
57_10137   RDGYFLQLRALAESGADSNGPEGVMTPEDDLSSLSSSEDELDVDMSGKSFYIPEPKTTSS
58_10525   RDGYFLQLRALAESGADSNGPEGVMTPEDDLSSLSSSEDELDVDMSGKSFYIPEPKTTSS
59_3457    RDGYFLQLRALAESGADSNGPEGVMTPEDDLSSLSSSEDELDVDMSGKSFYIPEPKTTSS
60_8244    RDGYFLQLRALAESGADSNGPEGVMTPEDDLSSLSSSEDELDVDMSGKSFYIPEPKTTSS
61_7964    RDGYFLQLRALAESGADSNGPEGVMTPEDDLSSLSSSEDELDVDMSGKSFYIPEPKTTSS
62_4413    RDGYFLQLRALAESGADSNGPEGVMTPEDDLSSLSSSEDELDVDMSGKSFYIPEPKTTSS
63_6958    RDGYFLQLRALAESGADSNGPEGVMTPEDDLSSLSSSEDELDVDMSGKSFYIPEPKTTSS
64_5997    QIAAAQAKAAQMLHLAKMPRDHPDQQELGKKRLLTPLPTVFNKLRFAYPGRDQQVLRNVS
65_8711    RDGYFLQLRALAESGADSNGPEGVMTPEDDLSSLSSSEDELDVDMSGKSFYIPEPKTTSS
66_9925    RDGYFLQLRALAESGADSNGPEGVMTPEDDLSSLSSSEDELDVDMSGKSFYIPEPKTTSS
67_10374   RDGYFLQLRALAESGADSNGPEGVMTPEDDLSSLSSSEDELDVDMSGKSFYIPEPKTTSS
68_3713    RDGYFLQLRALAESGADSNGPEGVMTPEDDLSSLSSSEDELDVDMSGKSFYIPEPKTTSS
69_4511    RDGYFLQLRALAESGADSNGPEGVMTPEDDLSSLSSSEDELDVDMSGKSFYIPEPKTTSS
70_6201    RDGYFLQLRALAESGADSNGPEGVMTPEDDLSSLSSSEDELDVDMSGKSFYIPEPKTTSS
71_3261    RDGYFLQLRALAESGADSNGPEGVMTPEDDLSSLSSSEDELDVDMSGKSFYIPEPKTTSS
72_8516    RDGYFLQLRALAESGADSNGPEGVMTPEDDLSSLSSSEDELDVDMSGKSFYIPEPKTTSS
73_9295    RDGYFLQLRALAESGADSNGPEGVMTPEDDLSSLSSSEDELDVDMSGKSFYIPEPKTTSS
74_809     RDGYFLQLRALAESGADSNGPEGVMTPEDDLSSLSSSEDELDVDMSGKSFYIPEPKTTSS
75_7282    RDGYFLQLRALAESGADSNGPEGVMTPEDDLSSLSSSEDELDVDMSGKSFYIPEPKTTSS
76_4910    RDGYFLQLRALAESGADSNGPEGVMTPEDDLSSLSSSEDELDVDMSGKSFYIPEPKTTSS
77_10912   RDGYFLQLRALAESGADSNGPEGVMTPEDDLSSLSSSEDELDVDMSGKSFYIPEPKTTSS
78_4020    RDGYFLQLRALAESGADSNGPEGVMTPEDDLSSLSSSEDELDVDMSGKSFYIPEPKTTSS
79_6703    RDGYFLQLRALAESGADSNGPEGVMTPEDDLSSLSSSEDELDVDMSGKSFYIPEPKTTSS
80_8619    RDGYFLQLRALAESGADSNGPEGVMTPEDDLSSLSSSEDELDVDMSGKSFYIPEPKTTSS
81_4126    ESGADSNGPEGVMTPEDDLSSLSSSEDELDVDMSGKSFYIPEPKTTSSFGALGRMSFVPI
82_1363    RDGYFLQLRALAESGADSNGPEGVMTPEDDLSSLSSSEDELDVDMSGKSFYIPEPKTTSS
83_5813    RDGYFLQLRALAESGADSNGPEGVMTPEDDLSSLSSSEDELDVDMSGKSFYIPEPKTTSS
84_2044    RDGYFLQLRALAESGADSNGPEGVMTPEDDLSSLSSSEDELDVDMSGKSFYIPEPKTTSS
85_7617    RDGYFLQLRALAESGADSNGPEGVMTPEDDLSSLSSSEDELDVDMSGKSFYIPEPKTTSS
86_1530    RDGYFLQLRALAESGADSNGPEGVMTPEDDLSSLSSSEDELDVDMSGKSFYIPEPKTTSS
87_1890    RDGYFLQLRALAESGADSNGPEGVMTPEDDLSSLSSSEDELDVDMSGKSFYIPEPKTTSS
88_7049    RDGYFLQLRALAESGADSNGPEGVMTPEDDLSSLSSSEDELDVDMSGKSFYIPEPKTTSS
89_440     RDGYFLQLRALAESGADSNGPEGVMTPEDDLSSLSSSEDELDVDMSGKSFYIPEPKTTSS
90_4331    RDGYFLQLRALAESGADSNGPEGVMTPEDDLSSLSSSEDELDVDMSGKSFYIPEPKTTSS
```

```
                  730       740       750       760       770       780
1_1896     FGALGRMSFVPIPGTNGPIYPTPNQPGSGAYLQLHRSPRHRHREQGRPNNWQDAEVSKPR
2_4785     FGALGRMSFVPIPGTNGPIYPTPNQPGSGAYLQLHRSPRHRHREQGRPNNWQDAEVSKPR
3_3653     FGALGRMSFVPIPGTNGPIYPTPNQPGSGAYLQLHRSPRHRHREQGRPNNWQDAEVSKPR
4_8157     FGALGRMSFVPIPGTNGPIYPTPNQPGSGAYLQLHRSPRHRHREQGRPNNWQDAEVSKPR
5_752      FGALGRMSFVPIPGTNGPIYPTPNQPGSGAYLQLHRSPRHRHREQGRPNNWQDAEVSKPR
6_8853     FGALGRMSFVPIPGTNGPIYPTPNQPGSGAYLQLHRSPRHRHREQGRPNNWQDAEVSKPR
7_4015     VAQEWVDTLKVDAFFSILCQPRAWHDRSKNSAARVCDVLDRGADEMRTIVAQFAPITLTV
8_2020     FGALGRMSFVPIPGTNGPIYPTPNQPGSGAYLQLHRSRRHRHREQGRPNNWQDAEVSKPR
9_1829     FGALGRMSFVPIPGTNGPIYPTPNQPGSGAYLQLHRSPRHRHREQGRPNNWQDAEVSKPR
10_6452    FGALGRMSFVPIPGTNGPIYPTPNQPGSGAYLQLHRSPRHRHREQGRPNNWQDAEVSKPR
11_8627    FGALGRMSFVPIPGTNGPIYPTPNQPGSGAYLQLHRSPRHRHREQGRPNNWQDAEVSKPR
12_6118    FGALGRMSFVPIPGTNGPIYPTPNQPGSGAYLQLHRSPRHRHREQGRPNNWQDAEVSKPR
13_2688    FGALGRMSFVPIPGTNGPIYPTPNQPGSGAYLQLHRSPRHRHREQGRPNNWQDAEVSKPR
14_6406    FGALGRMSFVPIPGTNGPIYPTPNQPGSGAYLQLHRSPRHRHREQGRPNNWQDAEVSKPR
15_9419    FGALGRMSFVPIPGTNGPIYPTPNQPGSGAYLQLHRSPRHRHREQGRPNNWQDAEVSKPR
16_8507    FGALGRMSFVPIPGTNGPIYPTPNQPGSGAYLQLHRSPRHRHREQGRPNNWQDAEVSKPR
17_5884    FGALGRMSFVPIPGTNGPIYPTPNQPGSGAYLQLHRSPRHRHREQGRPNNWQDAEVSKPR
18_2670    FGALGRMSFVPIPGTNGPIYPTPNQPGSGAYLQLHRSPRHRHREQGRPNNWQDAEVSKPR
19_5172    FGALGRMSFVPIPGTNGPIYPTPNQPGSGAYLQLHRSPRHRHREQGRPNNWQDAEVSKPR
20_8057    FGALGRMSFVPIPGTNGPIYPTPNQPGSGAYLQLHRSPRHRHREQGRPNNWQDAEVSKPR
21_4920    MFTVGSATMMMSNIPQIAAAQAKAAQMLHLAKMPRDHPDQQELGKKRLLTPLPTVFNKLR
22_6393    FGALGRMSFVPIPGTNGPIYPTPNQPGSGAYLQLHRSPRHRHREQGRPNNWQDAEVSKPR
23_8263    FGALGRMSFVPIPGTNGPIYPTPNQPGSGAYLQLHRSPRHRHREQGRPNNWQDAEVSKPR
24_8248    FGALGRMSFVPIPGTNGPIYPTPNQPGSGAYLQLHRSPRHRHREQGRPNNWQDAEVSKPR
25_6733    FGALGRMSFVPIPGTNGPIYPTPNQPGSGAYLQLHRSPRHRHREQGRPNNWQDAEVSKPR
26_2277    FGALGRMSFVPIPGTNGPIYPTPNQPGSGAYLQLHRSPRHRHREQGRPNNWQDAEVSKPR
27_2623    FGALGRMSFVPIPGTNGPIYPTPNQPGSGAYLQLHRSPRHRHREQGRPNNWQDAEVSKPR
28_7413    FGALGRMSFVPIPGTNGPIYPTPNQPGSGAYLQLHRSPRHRHREQGRPNNWQDAEVSKPR
29_8980    FGALGRMSFVPIPGTNGPIYPTPNQPGSGAYLQLHRSPRHRHREQGRPNNWQDAEVSKPR
30_8706    FGALGRMSFVPIPGTNGPIYPTPNQPGSGAYLQLHRSPRHRHREQGRPNNWQDAEVSKPR
31_6378    FGALGRMSFVPIPGTNGPIYPTPNQPGSGAYLQLHRSPRHRHREQGRPNNWQDAEVSKPR
32_4679    FGALGRMSFVPIPGTNGPIYPTPNQPGSGAYLQLHRSPRHRHREQGRPNNWQDAEVSKPR
33_2437    FGALGRMSFVPIPGTNGPIYPTPNQPGSGAYLQLHRSPRHRHREQGRPNNWQDAEVSKPR
34_9700    FGALGRMSFVPIPGTNGPIYPTPNQPGSGAYLQLHRSPRHRHREQGRPNNWQDAEVSKPR
35_10218   FGALGRMSFVPIPGTNGPIYPTPNQPGSGAYLQLHRSPRHRHREQGRPNNWQDAEVSKPR
36_2652    FGALGRMSFVPIPGTNGPIYPTPNQPGSGAYLQLHRSPRHRHREQGRPNNWQDAEVSKPR
37_5779    FGALGRMSFVPIPGTNGPIYPTPNQPGSGAYLQLHRSPRHRHREQGRPNNWQDAEVSKPR
38_654     FGALGRMSFVPIPGTNGPIYPTPNQPGSGAYLQLHRSPRHRHREQGRPNNWQDAEVSKPR
39_4879    FGALGRMSFVPIPGTNGPIYPTPNQPGSGAYLQLHRSPRHRHREQGRPNNWQDAEVSKPR
40_8113    FGALGRMSFVPIPGTNGPIYPTPNQPGSGAYLQLHRSPRHRHREQGRPNNWQDAEVSKPR
41_1767    PGTNGPIYPTPNQPGSGAYLQLHRSPRHRHREQGRPNNWQDAEVSKPRRGSIDLIQARGL
42_8428    FGALGRMSFVPIPGTNGPIYPTPNQPGSGAYLQLHRSPRHRHREQGRPNNWQDAEVSKPR
43_9819    FGALGRMSFVPIPGTNGPIYPTPNQPGSGAYLQLHRSPRHRHREQGRPNNWQDAEVSKPR
44_5569    FGALGRMSFVPIPGTNGPIYPTPNQPGSGAYLQLHRSPRHRHREQGRPNNWQDAEVSKPR
45_4189    FGALGRMSFVPIPGTNGPIYPTPNQPGSGAYLQLHRSPRHRHREQGRPNNWQDAEVSKPR
46_1027    FGALGRMSFVPIPGTNGPIYPTPNQPGSGAYLQLHRSPRHRHREQGRPNNWQDAEVSKPR
47_5048    FGALGRMSFVPIPGTNGPIYPTPNQPGSGAYLQLHRSPRHRHREQGRPNNWQDAEVSKPR
48_10124   FGALGRMSFVPIPGTNGPIYPTPNQPGSGAYLQLHRSPRHRHREQGRPNNWQDAEVSKPR
49_7151    FGALGRMSFVPIPGTNGPIYPTPNQPGSGAYLQLHRSPRHRHREQGRPNNWQDAEVSKPR
50_3695    FGALGRMSFVPIPGTNGPIYPTPNQPGSGAYLQLHRSPRHRHREQGRPNNWQDAEVSKPR
51_364     FGALGRMSFVPIPGTNGPIYPTPNQPGSGAYLQLHRSPRHRHREQGRPNNWQDAEVSKPR
52_242     FGALGRMSFVPIPGTNGPIYPTPNQPGSGAYLQLHRSPRHRHREQGRPNNWQDAEVSKPR
53_5225    FGALGRMSFVPIPGTNGPIYPTPNQPGSGAYLQLHRSPRHRHREQGRPNNWQDAEVSKPR
54_1635    FGALGRMSFVPIPGTNGPIYPTPNQPGSGAYLQLHRSPRHRHREQGRPNNWQDAEVSKPR
55_5766    FGALGRMSFVPIPGTNGPIYPTPNQPGSGAYLQLHRSPRHRHREQGRPNNWQDAEVSKPR
56_1557    FGALGRMSFVPIPGTNGPIYPTPNQPGSGAYLQLHRSPRHRHREQGRPNNWQDAEVSKPR
57_10137   FGALGRMSFVPIPGTNGPIYPTPNQPGSGAYLQLHRSPRHRHREQGRPNNWQDAEVSKPR
58_10525   FGALGRMSFVPIPGTNGPIYPTPNQPGSGAYLQLHRSPRHRHREQGRPNNWQDAEVSKPR
59_3457    FGALGRMSFVPIPGTNGPIYPTPNQPGSGAYLQLHRSPRHRHREQGRPNNWQDAEVSKPR
60_8244    FGALGRMSFVPIPGTNGPIYPTPNQPGSGAYLQLHRSPRHRHREQGRPNNWQDAEVSKPR
61_7964    FGALGRMSFVPIPGTNGPIYPTPNQPGSGAYLQLHRSPRHRHREQGRPNNWQDAEVSKPR
62_4413    FGALGRMSFVPIPGTNGPIYPTPNQPGSGAYLQLHRSPRHRHREQGRPNNWQDAEVSKPR
63_6958    FGALGRMSFVPIPGTNGPIYPTPNQPGSGAYLQLHRSPRHRHREQGRPNNWQDAEVSKPR
64_5997    LSIPSEGCTAIVGGSGCGKSTIAALILGLYEPLYDDDPSPTGGASPKASRKGNFEVISPV
65_8711    FGALGRMSFVPIPGTNGPIYPTPNQPGSGAYLQLHRSPRHRHREQGRPNNWQDAEVSKPR
66_9925    FGALGRMSFVPIPGTNGPIYPTPNQPGSGAYLQLHRSPRHRHREQGRPNNWQDAEVSKPR
67_10374   FGALGRMSFVPIPGTNGPIYPTPNQPGSGAYLQLHRSPRHRHREQGRPNNWQDAEVSKPR
68_3713    FGALGRMSFVPIPGTNGPIYPTPNQPGSGAYLQLHRSPRHRHREQGRPNNWQDAEVSKPR
69_4511    FGALGRMSFVPIPGTNGPIYPTPNQPGSGAYLQLHRSPRHRHREQGRPNNWQDAEVSKPR
70_6201    FGALGRMSFVPIPGTNGPIYPTPNQPGSGAYLQLHRSPRHRHREQGRPNNWQDAEVSKPR
71_3261    FGALGRMSFVPIPGTNGPIYPTPNQPGSGAYLQLHRSPRHRHREQGRPNNWQDAEVSKPR
72_8516    FGALGRMSFVPIPGTNGPIYPTPNQPGSGAYLQLHRSPRHRHREQGRPNNWQDAEVSKPR
73_9295    FGALGRMSFVPIPGTNGPIYPTPNQPGSGAYLQLHRSPRHRHREQGRPNNWQDAEVSKPR
74_809     FGALGRMSFVPIPGTNGPIYPTPNQPGSGAYLQLHRSPRHRHREQGRPNNWQDAEVSKPR
75_7282    FGALGRMSFVPIPGTNGPIYPTPNQPGSGAYLQLHRSPRHRHREQGRPNNWQDAEVSKPR
76_4910    FGALGRMSFVPIPGTNGPIYPTPNQPGSGAYLQLHRSPRHRHREQGRPNNWQDAEVSKPR
77_10912   FGALGRMSFVPIPGTNGPIYPTPNQPGSGAYLQLHRSPRHRHREQGRPNNWQDAEVSKPR
78_4020    FGALGRMSFVPIPGTNGPIYPTPNQPGSGAYLQLHRSPRHRHREQGRPNNWQDAEVSKPR
79_6703    FGALGRMSFVPIPGTNGPIYPTPNQPGSGAYLQLHRSPRHRHREQGRPNNWQDAEVSKPR
80_8619    FGALGRMSFVPIPGTNGPIYPTPNQPGSGAYLQLHRSPRHRHREQGRPNNWQDAEVSKPR
81_4126    PGTNGPIYPTPNQPGSGAYLQLHRSPRHRHREQGRPNNWQDAEVSKPRRGSIDLIQARGL
82_1363    FGALGRMSFVPIPGTNGPIYPTPNQPGSGAYLQLHRSPRHRHREQGRPNNWQDAEVSKPR
83_5813    FGALGRMSFVPIPGTNGPIYPTPNQPGSGAYLQLHRSPRHRHREQGRPNNWQDAEVSKPR
84_2044    FGALGRMSFVPIPGTNGPIYPTPNQPGSGAYLQLHRSPRHRHREQGRPNNWQDAEVSKPR
85_7617    FGALGRMSFVPIPGTNGPIYPTPNQPGSGAYLQLHRSPRHRHREQGRPNNWQDAEVSKPR
86_1530    FGALGRMSFVPIPGTNGPIYPTPNQPGSGAYLQLHRSPRHRHREQGRPNNWQDAEVSKPR
87_1890    FGALGRMSFVPIPGTNGPIYPTPNQPGSGAYLQLHRSPRHRHREQGRPNNWQDAEVSKPR
88_7049    FGALGRMSFVPIPGTNGPIYPTPNQPGSGAYLQLHRSPRHRHREQGRPNNWQDAEVSKPR
89_440     FGALGRMSFVPIPGTNGPIYPTPNQPGSGAYLQLHRSPRHRHREQGRPNNWQDAEVSKPR
90_4331    FGALGRMSFVPIPGTNGPIYPTPNQPGSGAYLQLHRSPRHRHREQGRPNNWQDAEVSKPR
```

```
                790       800       810       820       830       840
1_1896     RGSIDLIQARGLSALANRSPVKRPLPQGGRIGGVLRRLSTRSSPIEDVEMSPFRDHDKGV
2_4785     RGSIDLIQARGLSALANRSPVKRPLPQGGRIGGVLRRLSTRSSPIEDVEMSPFRDHDKGV
3_3653     RGSIDLIQARGLSALANRSPVKRPLPQGGRIGGVLRRLSTRSSPIEDVEMSPFRDHDKGV
4_8157     RGSIDLIQARGLSALANRSPVKRPLPQGGRIGGVLRRLSTRSSPIEDVEMSPFRDHDKGV
5_752      RGSIDLIQARGLSALANRSPVKRPLPQGGRIGGVLRRLSTRSSPIEDVEMSPFRDHDKGV
6_8853     RGSIDLIQARGLSALANRSPVKRPLPQGGRIGGVLRRLSTRSSPIEDVEMSPFRDHDKGV
7_4015     IIMISSAIIWAMAILWQLTLVALAVFPFVGLCIFSSTRTSEKWEALSNESSEATGSILSS
8_2020     RGSIDLIQARGLSALANRSPVKRPLPQGGRIGGVLRRLSTRSSPIEDVEMSPFRDHDKGV
9_1829     RGSIDLIQARGLSALANRSPVKRPLPQGGRIGGVLRRLSTRSSPIEDVEMSPFRDHDKGV
10_6452    RGSIDLIQARGLSALANRSPVKRPLPQGGRIGGVLRRLSTRSSPIEDVEMSPFRDHDKGV
11_8627    RGSIDLIQARGLSALANRSPVKRPLPQGGRIGGVLRRLSTRSSPIEDVEMSPFRDHDKGV
12_6118    RGSIDLIQARGLSALANRSPVKRPLPQGGRIGGVLRRLSTRSSPIEDVEMSPFRDHDKGV
13_2688    RGSIDLIQARGLSALANRSPVKRPLPQGGRIGGVLRRLSTRSSPIEDVEMSPFRDHDKGV
14_6406    RGSIDLIQARGLSALANRSPVKRPLPQGGRIGGVLRRLSTRSSPIEDVEMSPFRDHDKGV
15_9419    RGSIDLIQARGLSALANRSPVKRPLPQGGRIGGVLRRLSTRSSPIEDVEMSPFRDHDKGV
16_8507    RGSIDLIQARGLSALANRSPVKRPLPQGGRIGGVLRRLSTRSSPIEDVEMSPFRDHDKGV
17_5884    RGSIDLIQARGLSALANRSPVKRPLPQGGRIGGVLRRLSTRSSPIEDVEMSPFRDHDKGV
18_2670    RGSIDLIQARGLSALANRSPVKRPLPQGGRIGGVLRRLSTRSSPIEDVEMSPFRDHDKGV
19_5172    RGSIDLIQARGLSALANRSPVKRPLPQGGRIGGVLRRLSTRSSPIEDVEMSPFRDHDKGV
20_8057    RGSIDLIQARGLSALANRSPVKRPLPQGGRIGGVLRRLSTRSSPIEDVEMSPFRDHDKGV
21_4920    FAYPGRDQQVLRNVSLSIPSEGCTAIVGGSGCGKSTIAALILGLYEPLYDDDPSPTGGAS
22_6393    RGSIDLIQARGLSALANRSPVKRPLPQGGRIGGVLRRLSTRSSPIEDVEMSPFRDHDKGV
23_8263    RGSIDLIQARGLSALANRSPVKRPLPQGGRIGGVLRRLSTRSSPIEDVEMSPFRDHDKGV
24_8248    RGSIDLIQARGLSALANRSPVKRPLPQGGRIGGVLRRLSTRSSPIEDVEMSPFRDHDKGV
25_6733    RGSIDLIQARGLSALANRSPVKRPLPQGGRIGGVLRRLSTRSSPIEDVEMSPFRDHDKGV
26_2277    RGSIDLIQARGLSALANRSPVKRPLPQGGRIGGVLRRLSTRSSPIEDVEMSPFRDHDKGV
27_2623    RGSIDLIQARGLSALANRSPVKRPLPQGGRIGGVLRRLSTRSSPIEDVEMSPFRDHDKGV
28_7413    RGSIDLIQARGLSALANRSPVKRPLPQGGRIGGVLRRLSTRSSPIEDVEMSPFRDHDKGV
29_8980    RGSIDLIQARGLSALANRSPVKRPLPQGGRIGGVLRRLSTRSSPIEDVEMSPFRDHDKGV
30_8706    RGSIDLIQARGLSALANRSPVKRPLPQGGRIGGVLRRLSTRSSPIEDVEMSPFRDHDKGV
31_6378    RGSIDLIQARGLSALANRSPVKRPLPQGGRIGGVLRRLSTRSSPIEDVEMSPFRDHDKGV
32_4679    RGSIDLIQARGLSALANRSPVKRPLPQGGRIGGVLRRLSTRSSPIEDVEMSPFRDHDKGV
33_2437    RGSIDLIQARGLSALANRSPVKRPLPQGGRIGGVLRRLSTRSSPIEDVEMSPFRDHDKGV
34_9700    RGSIDLIQARGLSALANRSPVKRPLPQGGRIGGVLRRLSTRSSPIEDVEMSPFRDHDKGV
35_10218   RGSIDLIQARGLSALANRSPVKRPLPQGGRIGGVLRRLSTRSSPIEDVEMSPFRDHDKGV
36_2652    RGSIDLIQARGLSALANRSPVKRPLPQGGRIGGVLRRLSTRSSPIEDVEMSPFRDHDKGV
37_5779    RGSIDLIQARGLSALANRSPVKRPLPQGGRIGGVLRRLSTRSSPIEDVEMSPFRDHDKGV
38_654     RGSIDLIQARGLSALANRSPVKRPLPQGGRIGGVLRRLSTRSSPIEDVEMSPFRDHDKGV
39_4879    RGSIDLIQARGLSALANRSPVKRPLPQGGRIGGVLRRLSTRSSPIEDVEMSPFRDHDKGV
40_8113    RGSIDLIQARGLSALANRSPVKRPLPQGGRIGGVLRRLSTRSSPIEDVEMSPFRDHDKGV
41_1767    SALANRSPVKRPLPQGGRIGGVLRRLSTRSSPIEDVEMSPFRDHDKGVKEVVRERIREAI
42_8428    RGSIDLIQARGLSALANRSPVKRPLPQGGRIGGVLRRLSTRSSPIEDVEMSPFRDHDKGV
43_9819    RGSIDLIQARGLSALANRSPVKRPLPQGGRIGGVLRRLSTRSSPIEDVEMSPFRDHDKGV
44_5569    RGSIDLIQARGLSALANRSPVKRPLPQGGRIGGVLRRLSTRSSPIEDVEMSPFRDHDKGV
45_4189    RGSIDLIQARGLSALANRSPVKRPLPQGGRIGGVLRRLSTRSSPIEDVEMSPFRDHDKGV
46_1027    RGSIDLIQARGLSALANRSPVKRPLPQGGRIGGVLRRLSTRSSPIEDVEMSPFRDHDKGV
47_5048    RGSIDLIQARGLSALANRSPVKRPLPQGGRIGGVLRRLSTRSSPIEDVEMSPFRDHDKGV
48_10124   RGSIDLIQARGLSALANRSPVKRPLPQGGRIGGVLRRLSTRSSPIEDVEMSPFRDHDKGV
49_7151    RGSIDLIQARGLSALANRSPVKRPLPQGGRIGGVLRRLSTRSSPIEDVEMSPFRDHDKGV
50_3695    RGSIDLIQARGLSALANRSPVKRPLPQGGRIGGVLRRLSTRSSPIEDVEMSPFRDHDKGV
51_364     RGSIDLIQARGLSALANRSPVKRPLPQGGRIGGVLRRLSTRSSPIEDVEMSPFRDHDKGV
52_242     RGSIDLIQARGLSALANRSPVKRPLPQGGRIGGVLRRLSTRSSPIEDVEMSPFRDHDKGV
53_5225    RGSIDLIQARGLSALANRSPVKRPLPQGGRIGGVLRRLSTRSSPIEDVEMSPFRDHDKGV
54_1635    RGSIDLIQARGLSALANRSPVKRPLPQGGRIGGVLRRLSTRSSPIEDVEMSPFRDHDKGV
55_5766    RGSIDLIQARGLSALANRSPVKRPLPQGGRIGGVLRRLSTRSSPIEDVEMSPFRDHDKGV
56_1557    RGSIDLIQARGLSALANRSPVKRPLPQGGRIGGVLRRLSTRSSPIEDVEMSPFRDHDKGV
57_10137   RGSIDLIQARGLSALANRSPVKRPLPQGGRIGGVLRRLSTRSSPIEDVEMSPFRDHDKGV
58_10525   RGSIDLIQARGLSALANRSPVKRPLPQGGRIGGVLRRLSTRSSPIEDVEMSPFRDHDKGV
59_3457    RGSIDLIQARGLSALANRSPVKRPLPQGGRIGGVLRRLSTRSSPIEDVEMSPFRDHDKGV
60_8244    RGSIDLIQARGLSALANRSPVKRPLPQGGRIGGVLRRLSTRSSPIEDVEMSPFRDHDKGV
61_7964    RGSIDLIQARGLSALANRSPVKRPLPQGGRIGGVLRRLSTRSSPIEDVEMSPFRDHDKGV
62_4413    RGSIDLIQARGLSALANRSPVKRPLPQGGRIGGVLRRLSTRSSPIEDVEMSPFRDHDKGV
63_6958    RGSIDLIQARGLSALANRSPVKRPLPQGGRIGGVLRRLSTRSSPIEDVEMSPFRDHDKGV
64_5997    VSYPLGPVASSPLPSLVYSPPQGGTRASSHRRSGELGALTYAGFDAREISTKALRGMMAY
65_8711    RGSIDLIQARGLSALANRSPVKRPLPQGGRIGGVLRRLSTRSSPIEDVEMSPFRDHDKGV
66_9925    GGSIDLIQARGLSALANRSPVKRPLPQGGRIGGVLRRLSTRSSPIEDVEMSPFRDHDKGV
67_10374   RGSIDLIQARGLSALANRSPVKRPLPQGGRIGGVLRRLSTRSSPIEDVEMSPFRDHDKGV
68_3713    RGSIDLIQARGLSALANRSPVKRPLPQGGRIGGVLRRLSTRSSPIEDVEMSPFRDHDKGV
69_4511    RGSIDLIQARGLSALANRSPVKRPLPQGGRIGGVLRRLSTRSSPIEDVEMSPFRDHDKGV
70_6201    RGSIDLIQARGLSALANRSPVKRPLPQGGRIGGVLRRLSTRSSPIEDVEMSPFRDHDKGV
71_3261    RGSIDLIQARGLSALANRSPVKRPLPQGGRIGGVLRRLSTRSSPIEDVEMSPFRDHDKGV
72_8516    RGSIDLIQARGLSALANRSPVKRPLPQGGRIGGVLRRLSTRSSPIEDVEMSPFRDHDKGV
73_9295    RGSIDLIQARGLSALANRSPVKRPLPQGGRIGGVLRRLSTRSSPIEDVEMSPFRDHDKGV
74_809     RGSIDLIQARGLSALANRSPVKRPLPQGGRIGGVLRRLSTRSSPIEDVEMSPFRDHDKGV
75_7282    RGSIDLIQARGLSALANRSPVKRPLPQGGRIGGVLRRLSTRSSPIEDVEMSPFRDHDKGV
76_4910    RGSIDLIQARGLSALANRSPVKRPLPQGGRIGGVLRRLSTRSSPIEDVEMSPFRDHDKGV
77_10912   RGSIDLIQARGLSALANRSPVKRPLPQGGRIGGVLRRLSTRSSPIEDVEMSPFRDHDKGV
78_4020    RGSIDLIQARGLSALANRSPVKRPLPQGGRIGGVLRRLSTRSSPIEDVEMSPFRDHDKGV
79_6703    RGSIDLIQARGLSALANRSPVKRPLPQGGRIGGVLRRLSTRSSPIEDVEMSPFRDHDKGV
80_8619    RGSIDLIQARGLSALANRSPVKRPLPQGGRIGGVLRRLSTRSSPIEDVEMSPFRDHDKGV
81_4126    SALANRSPVKRPLPQGGRIGGVLRRLSTRSSPIEDVEMSPFRDHDKGVKEVVRERIREAI
82_1363    RGSIDLIQARGLSALANRSPVKRPLPQGGRIGGVLRRLSTRSSPIEDVEMSPFRDHDKGV
83_5813    RGSIDLIQARGLSALANRSPVKRPLPQGGRIGGVLRRLSTRSSPIEDVEMSPFRDHDKGV
84_2044    RGSIDLIQARGLSALANRSPVKRPLPQGGRIGGVLRRLSTRSSPIEDVEMSPFRDHDKGV
85_7617    RGSIDLIQARGLSALANRSPVKRPLPQGGRIGGVLRRLSTRSSPIEDVEMSPFRDHDKGV
86_1530    RGSIDLIQARGLSALANRSPVKRPLPQGGRIGGVLRRLSTRSSPIEDVEMSPFRDHDKGV
87_1890    GGSIDLIQARGLSALANRSPVKRPLPQGGRIGGVLRRLSTRSSPIEDVEMSPFRDHDKGV
88_7049    RGSIDLIQARGLSALANRSPVKRPLPQGGRIGGVLRRLSTRSSPIEDVEMSPFRDHDKGV
89_440     RGSIDLIQARGLSALANRSPVKRPLPQGGRIGGVLRRLSTRSSPIEDVEMSPFRDHDKGV
90_4331    RGSIDLIQARGLSALANRSPVKRPLPQGGRIGGVLRRLSTRSSPIEDVEMSPFRDHDKGV
```

```
                  850       860       870       880       890       900
1_1896    KEVVRERIREAIGKGTGAGKRGVTKRQARRGKKDGPDEEATNTTGHMPAYQILKTVWPAL
2_4785    KEVVRERIREAIGKGTGAGKRGVTKRQARRGKKDGPDEEATNTTGHMPAYQILKTVWPAL
3_3653    KEVVRERIREAIGKGTGAGKRGVTKRQARRGKKDGPDEEATNTTGHMPAYQILKTVWPAL
4_8157    KEVVRERIREAIGKGTGAGKRGVTKRQARRGKKDGPDEEATNTTGHMPAYQILKTVWPAL
5_752     KEVVRERIREAIGKGTGAGKRGVTKRQARRGKKDGPDEEATNTTGHMPAYQILKTVWPAL
6_8853    KEVVRERIREAIGKGTGAGKRGVTKRQARRGKKDGPDEEATNTTGHMPAYQILKTVWPAL
7_4015    VVSDIRVVRAFLLEKFFGDRFEAAVERAFTIGKKRGLYTGIWSGIHCSISQWLVVVIFTF
8_2020    KEVVRERIREAIGKGTGAGKRGVTKRQARRGKKDGPDEEATNTTGHMPAYQILKTVWPAL
9_1829    KEVVRERIREAIGKGTGAGKRGVTKRQARRGKKDGPDEEATNTTGHMPAYQILKTVWPAL
10_6452   KEVVRERIREAIGKGTGAGKRGVTKRQARRGKKDGPDEEATNTTGHMPAYQILKTVWPAL
11_8627   KEVVRERIREAIGKGTGAGKRGVTKRQARRGKKDGPDEEATNTTGHMPAYQILKTVWPAL
12_6118   KEVVRERIREAIGKGTGAGKRGVTKRQARRGKKDGPDEEATNTTGHMPAYQILKTVWPAL
13_2688   KEVVRERIREAIGKGTGAGKRGVTKRQARRGKKDGPDEEATNTTGHMPAYQILKTVWPAL
14_6406   KEVVRERIREAIGKGTGAGKRGVTKRQARRGKKDGPDEEATNTTGHMPAYQILKTVWPAL
15_9419   KEVVRERIREAIGKGTGAGKRGVTKRQARRGKKDGPDEEATNTTGHMPAYQILKTVWPAL
16_8507   KEVVRERIREAIGKGTGAGKRGVTKRQARRGKKDGPDEEATNTTGHMPAYQILKTVWPAL
17_5884   KEVVRERIREAIGKGTGAGKRGVTKRQARRGKKDGPDEEATNTTGHMPAYQILKTVWPAL
18_2670   KEVVRERIREAIGKGTGAGKRGVTKRQARRGKKDGPDEEATNTTGHMPAYQILKTVWPAL
19_5172   KEVVRERIREAIGKGTGAGKRGVTKRQARRGKKDGPDEEATNTTGHMPAYQILKTVWPAL
20_8057   KEVVRERIREAIGKGTGAGKRGVTKRQARRGKKDGPDEEATNTTGHMPAYQILKTVWPAL
21_4920   PKASRKGNFEVISPVVSYPLGPVASSPLPSLVYSPPQGGTRASSHRRSGELGALTYAGFD
22_6393   KEVVRERIREAIGKGTGAGKRGVTKRQARRGKKDGPDEEATNTTGHMPAYQILKTVWPAL
23_8263   KEVVRERIREAIGKGTGAGKRGVTKRQARRGKKDGPDEEATNTTGHMPAYQILKTVWPAL
24_8248   KEVVRERIREAIGKGTGAGKRGVTKRQARRGKKDGPDEEATNTTGHMPAYQILKTVWPAL
25_6733   KEVVRERIREAIGKGTGAGKRGVTKRQARRGKKDGPDEEATNTTGHMPAYQILKTVWPAL
26_2277   KEVVRERIREAIGKGTGAGKRGVTKRQARRGKKDGPDEEATNTTGHMPAYQILKTVWPAL
27_2623   KEVVRERIREAIGKGTGAGKRGVTKRQARRGKKDGPDEEATNTTGHMPAYQILKTVWPAL
28_7413   KEVVRERIREAIGKGTGAGKRGVTKRQARRGKKDGPDEEATNTTGHMPAYQILKTVWPAL
29_8980   KEVVRERIREAIGKGTGAGKRGVTKRQARRGKKDGPDEEATNTTGHMPAYQILKTVWPAL
30_8706   KEVVRERIREAIGKGTGAGKRGVTKRQARRGKKDGPDEEATNTTGHMPAYQILKTVWPAL
31_6378   KEVVRERIREAIGKGTGAGKRGVTKRQARRGKKDGPDEEATNTTGHMPAYQILKTVWPAL
32_4679   KEVVRERIREAIGKGTGAGKRGVTKRQARRGKKDGPDEEATNTTGHMPAYQILKTVWPAL
33_2437   KEVVRERIREAIGKGTGAGKRGVTKRQARRGKKDGPDEEATNTTGHMPAYQILKTVWPAL
34_9700   KEVVRERIREAIGKGTGAGKRGVTKRQARRGKKDGPDEEATNTTGHMPAYQILKTVWPAL
35_10218  KEVVRERIREAIGKGTGAGKRGVTKRQARRGKKDGPDEEATNTTGHMPAYQILKTVWPAL
36_2652   KEVVRERIREAIGKGTGAGKRGVTKRQARRGKKDGPDEEATNTTGHMPAYQILKTVWPAL
37_5779   KEVVRERIREAIGKGTGAGKRGVTKRQARRGKKDGPDEEATNTTGHMPAYQILKTVWPAL
38_654    KEVVRERIREAIGKGTGAGKRGVTKRQARRGKKDGPDEEATNTTGHMPAYQILKTVWPAL
39_4879   KEVVRERIREAIGKGTGAGKRGVTKRQARRGKKDGPDEEATNTTGHMPAYQILKTVWPAL
40_8113   KEVVRERIREAIGKGTGAGKRGVTKRQARRGKKDGPDEEATNTTGHMPAYQILKTVWPAL
41_1767   GKGTGAGKRGVTKRQARRGKKDGPDEEATNTTGHMPAYQILKTVWPALDTKHRLYAVAAL
42_8428   KEVVRERIREAIGKGTGAGKRGVTKRQARRGKKDGPDEEATNTTGHMPAYQILKTVWPAL
43_9819   KEVVRERIREAIGKGTGAGKRGVTKRQARRGKKDGPDEEATNTTGHMPAYQILKTVWPAL
44_5569   KEVVRERIREAIGKGTGAGKRGVTKRQARRGKKDGPDEEATNTTGHMPAYQILKTVWPAL
45_4189   KEVVRERIREAIGKGTGAGKRGVTKRQARRGKKDGPDEEATNTTGHMPAYQILKTVWPAL
46_1027   KEVVRERIREAIGKGTGAGKRGVTKRQARRGKKDGPDEEATNTTGHMPAYQILKTVWPAL
47_5048   KEVVRERIREAIGKGTGAGKRGVTKRQARRGKKDGPDEEATNTTGHMPAYQILKTVWPAL
48_10124  KEVVRERIREAIGKGTGAGKRGVTKRQARRGKKDGPDEEATNTTGHMPAYQILKTVWPAL
49_7151   KEVVRERIREAIGKGTGAGKRGVTKRQARRGKKDGPDEEATNTTGHMPAYQILKTVWPAL
50_3695   KEVVRERIREAIGKGTGAGKRGVTKRQARRGKKDGPDEEATNTTGHMPAYQILKTVWPAL
51_364    KEVVRERIREAIGKGTGAGKRGVTKRQARRGKKDGPDEEATNTTGHMPAYQILKTVWPAL
52_242    KEVVRERIREAIGKGTGAGKRGVTKRQARRGKKDGPDEEATNTTGHMPAYQILKTVWPAL
53_5225   KEVVRERIREAIGKGTGAGKRGVTKRQARRGKKDGPDEEATNTTGHMPAYQILKTVWPAL
54_1635   KEVVRERIREAIGKGTGAGKRGVTKRQARRGKKDGPDEEATNTTGHMPAYQILKTVWPAL
55_5766   KEVVRERIREAIGKGTGAGKRGVTKRQARRGKKDGPDEEATNTTGHMPAYQILKTVWPAL
56_1557   KEVVRERIREAIGKGTGAGKRGVTKRQARRGKKDGPDEEATNTTGHMPAYQILKTVWPAL
57_10137  KEVVRERIREAIGKGTGAGKRGVTKRQARRGKKDGPDEEATNTTGHMPAYQILKTVWPAL
58_10525  KEVVRERIREAIGKGTGAGKRGVTKRQARRGKKDGPDEEATNTTGHMPAYQILKTVWPAL
59_3457   KEVVRERIREAIGKGTGAGKRGVTKRQARRGKKDGPDEEATNTTGHMPAYQILKTVWPAL
60_8244   KEVVRERIREAIGKGTGAGKRGVTKRQARRGKKDGPDEEATNTTGHMPAYQILKTVWPAL
61_7964   KEVVRERIREAIGKGTGAGKRGVTKRQARRGKKDGPDEEATNTTGHMPAYQILKTVWPAL
62_4413   KEVVRERIREAIGKGTGAGKRGVTKRQARRGKKDGPDEEATNTTGHMPAYQILKTVWPAL
63_6958   KEVVRERIREAIGKGTGAGKRGVTKRQARRGKKDGPDEEATNTTGHMPAYQILKTVWPAL
64_5997   VPQQPFIFPGTIRENIVYGLPEDESVLRGQHNVELAAREADIHEFIISLPQGYETLVGDG
65_8711   KEVVRERIREAIGKGTGAGKRGVTKRQARRGKKDGPDEEATNTTGHMPAYQILKTVWPAL
66_9925   KEVVRERIREAIGKGTGAGKRGVTKRQARRGKKDGPDEEATNTTGHMPAYQILKTVWPAL
67_10374  KEVVRERIREAIGKGTGAGKRGVTKRQARRGKKDGPDEEATNTTGHMPAYQILKTVWPAL
68_3713   KEVVRERIREAIGKGTGAGKRGVTKRQARRGKKDGPDEEATNTTGHMPAYQILKTVWPAL
69_4511   KEVVRERIREAIGKGTGAGKRGVTKRQARRGKKDGPDEEATNTTGHMPAYQILKTVWPAL
70_6201   KEVVRERIREAIGKGTGAGKRGVTKRQARRGKKDGPDEEATNTTGHMPAYQILKTVWPAL
71_3261   KEVVRERIREAIGKGTGAGKRGVTKRQARRGKKDGPDEEATNTTGHMPAYQILKTVWPAL
72_8516   KEVVRERIREAIGKGTGAGKRGVTKRQARRGKKDGPDEEATNTTGHMPAYQILKTVWPAL
73_9295   KEVVRERIREAIGKGTGAGKRGVTKRQARRGKKDGPDEEATNTTGHMPAYQILKTVWPAL
74_809    KEVVRERIREAIGKGTGAGKRGVTKRQARRGKKDGPDEEATNTTGHMPAYQILKTVWPAL
75_7282   KEVVRERIREAIGKGTGAGKRGVTKRQARRGKKDGPDEEATNTTGHMPAYQILKTVWPAL
76_4910   KEVVRERIREAIGKGTGAGKRGVTKRQARRGKKDGPDEEATNTTGHMPAYQILKTVWPAL
77_10912  KEVVRERIREAIGKGTGAGKRGVTKRQARRGKKDGPDEEATNTTGHMPAYQILKTVWPAL
78_4020   KEVVRERIREAIGKGTGAGKRGVTKRQARRGKKDGPDEEATNTTGHMPAYQILKTVWPAL
79_6703   KEVVRERIREAIGKGTGAGKRGVTKRQARRGKKDGPDEEATNTTGHMPAYQILKTVWPAL
80_8619   KEVVRERIREAIGKGTGAGKRGVTKRQARRGKKDGPDEEATNTTGHMPAYQILKTVWPAL
81_4126   GKGTGAGKRGVTKRQARRGKKDGPDEEATNTTGHMPAYQILKTVWPALDTKHRLYAVAAL
82_1363   KEVVRERIREAIGKGTGAGKRGVTKRQARRGKKDGPDEEATNTTGHMPAYQILKTVWPAL
83_5813   KEVVRERIREAIGKGTGAGKRGVTKRQARRGKKDGPDEEATNTTGHMPAYQILKTVWPAL
84_2044   KEVVRERIREAIGKGTGAGKRGVTKRQARRGKKDGPDEEATNTTGHMPAYQILKTVWPAL
85_7617   KEVVRERIREAIGKGTGAGKRGVTKRQARRGKKDGPDEEATNTTGHMPAYQILKTVWPAL
86_1530   KEVVRERIREAIGKGTGAGKRGVTKRQARRGKKDGPDEEATNTTGHMPAYQILKTVWPAL
87_1890   KEVVRERIREAIGKGTGAGKRGVTKRQARRGKKDGPDEEATNTTGHMPAYQILKTVWPAL
88_7049   KEVVRERIREAIGKGTGAGKRGVTKRQARRGKKDGPDEEATNTTGHMPAYQILKTVWPAL
89_440    KEVVRERIREAIGKGTGAGKRGVTKRQARRGKKDGPDEEATNTTGHMPAYQILKTVWPAL
90_4331   KEVVRERIREAIGKGTGAGKRGVTKRQARRGKKDGPDEEATNTTGHMPAYQILKTVWPAL
```

```
                   910       920       930       940       950       960
1_1896     DTKHRLYAVAALFWCIVAAGCSPVFAFVFSNLLQAFWAQGSKLEAGTKWAIVLGCVGILD
2_4785     DTKHRLYAVAALFWCIVAAGCSPVFAFVFSNLLQAFWAQGSKLEAGTKWAIVLGCVGILD
3_3653     DTKHRLYAVAALFWCIVAAGCSPVFAFVFSNLLQAFWAQGSKLEAGTKWAIVLGCVGILD
4_8157     DTKHRLYAVAALFWCIVAAGCSPVFAFVFSNLLQAFWAQGSKLEAGTKWAIVLGCVGILD
5_752      DTKHRLYAVAALFWCIVAAGCSPVFAFVFSNLLQAFWAQGSKLEAGTKWAIVLGCVGILD
6_8853     DTKHRLYAVAALFWCIVAAGCSPVFAFVFSNLLQAFWAQGSKLEAGTKWAIVLGCVGILD
7_4015     GVFLLTTVETVNVGDIIQVVNLLMFTVGSATMMMSNIPQIAAAQAKATQMLHLAQMPRGH
8_2020     DTKHRLYAVAALFWCIVAAGCSPVFAFVFSNLLQAFWAQGSKLEAGTKWAIVLGCVGILD
9_1829     DTKHRLYAVAALFWCIVAAGCSPVFAFVFSNLLQAFWAQGSKLEAGTKWAIVLGCVGILD
10_6452    DTKHRLYAVAALFWCIVAAGCSPVFAFVFSNLLQAFWAQGSKLEAGTKWAIVLGCVGILD
11_8627    DTKHRLYAVAALFWCIVAAGCSPVFAFVFSNLLQAFWAQGSKLEAGTKWAIVLGCVGILD
12_6118    DTKHRLYAVAALFWCIVAAGCSPVFAFVFSNLLQAFWAQGSKLEAGTKWAIVLGCVGILD
13_2688    DTKHRLYAVAALFWCIVAAGCSPVFAFVFSNLLQAFWAQGSKLEAGTKWAIVLGCVGILD
14_6406    DTKHRLYAVAALFWCIVAAGCSPVFAFVFSNLLQAFWAQGSKLEAGTKWAIVLGCVGILD
15_9419    DTKHRLYAVAALFWCIVAAGCSPVFAFVFSNLLQAFWAQGSKLEAGTKWAIVLGCVGILD
16_8507    DTKHRLYAVAALFWCIVAAGCSPVFAFVFSNLLQAFWAQGSKLEAGTKWAIVLGCVGILD
17_5884    DTKHRLYAVAALFWCIVAAGCSPVFAFVFSNLLQAFWAQGSKLEAGTKWAIVLGCVGILD
18_2670    DTKHRLYAVAALFWCIVAAGCSPVFAFVFSNLLQAFWAQGSKLEAGTKWAIVLGCVGILD
19_5172    DTKHRLYAVAALFWCIVAAGCSPVFAFVFSNLLQAFWAQGSKLEAGTKWAIVLGCVGILD
20_8057    DTKHRLYAVAALFWCIVAAGCSPVFAFVFSNLLQAFWAQGSKLEAGTKWAIVLGCVGILD
21_4920    AREISTKALRGMMAYVPQQPFIFPGTIRENIVYGLPEDESVLRGQHNVELAAREADIHEF
22_6393    DTKHRLYAVAALFWCIVAAGCSPVFAFVFSNLLQAFWAQGSKLEAGTKWAIVLGCVGILD
23_8263    DTKHRLYAVAALFWCIVAAGCSPVFAFVFSNLLQAFWAQGSKLEAGTKWAIVLGCVGILD
24_8248    DTKHRLYAVAALFWCIVAAGCSPVFAFVFSNLLQAFWAQGSKLEAGTKWAIVLGCVGILD
25_6733    DTKHRLYAVAALFWCIVAAGCSPVFAFVFSNLLQAFWAQGSKLEAGTKWAIVLGCVGILD
26_2277    DTKHRLYAVAALFWCIVAAGCSPVFAFVFSNLLQAFWAQGSKLEAGTKWAIVLGCVGILD
27_2623    DTKHRLYAVAALFWCIVAAGCSPVFAFVFSNLLQAFWAQGSKLEAGTKWAIVLGCVGILD
28_7413    DTKHRLYAVAALFWCIVAAGCSPVFAFVFSNLLQAFWAQGSKLEAGTKWAIVLGCVGILD
29_8980    DTKHRLYAVAALFWCIVAAGCSPVFAFVFSNLLQAFWAQGSKLEAGTKWAIVLGCVGILD
30_8706    DTKHRLYAVAALFWCIVAAGCSPVFAFVFSNLLQAFWAQGSKLEAGTKWAIVLGCVGILD
31_6378    DTKHRLYAVAALFWCIVAAGCSPVFAFVFSNLLQAFWAQGSKLEAGTKWAIVLGCVGILD
32_4679    DTKHRLYAVAALFWCIVAAGCSPVFAFVFSNLLQAFWAQGSKLEAGTKWAIVLGCVGILD
33_2437    DTKHRLYAVAALFWCIVAAGCSPVFAFVFSNLLQAFWAQGSKLEAGTKWAIVLGCVGILD
34_9700    DTKHRLYAVAALFWCIVAAGCSPVFAFVFSNLLQAFWAQGSKLEAGTKWAIVLGCVGILD
35_10218   DTKHRLYAVAALFWCIVAAGCSPVFAFVFSNLLQAFWAQGSKLEAGTKWAIVLGCVGILD
36_2652    DTKHRLYAVAALFWCIVAAGCSPVFAFVFSNLLQAFWAQGSKLEAGTKWAIVLGCVGILD
37_5779    DTKHRLYAVAALFWCIVAAGCSPVFAFVFSNLLQAFWAQGSKLEAGTKWAIVLGCVGILD
38_654     DTKHRLYAVAALFWCIVAAGCSPVFAFVFSNLLQAFWAQGSKLEAGTKWAIVLGCVGILD
39_4879    DTKHRLYAVAALFWCIVAAGCSPVFAFVFSNLLQAFWAQGSKLEAGTKWAIVLGCVGILD
40_8113    DTKHRLYAVAALFWCIVAAGCSPVFAFVFSNLLQAFWAQGSKLEAGTKWAIVLGCVGILD
41_1767    FWCIVAAGCSPVFAFVFSNLLQAFWAQGSKLEAGTKWAIVLGCVGILDFTAVFFTFFLAG
42_8428    DTKHRLYAVAALFWCIVAAGCSPVFAFVFSNLLQAFWAQGSKLEAGTKWAIVLGCVGILD
43_9819    DTKHRLYAVAALFWCIVAAGCSPVFAFVFSNLLQAFWAQGSKLEAGTKWAIVLGCVGILD
44_5569    DTKHRLYAVAALFWCIVAAGCSPVFAFVFSNLLQAFWAQGSKLEAGTKWAIVLGCVGILD
45_4189    DTKHRLYAVAALFWCIVAAGCSPVFAFVFSNLLQAFWAQGSKLEAGTKWAIVLGCVGILD
46_1027    DTKHRLYAVAALFWCIVAAGCSPVFAFVFSNLLQAFWAQGSKLEAGTKWAIVLGCVGILD
47_5048    DTKHRLYAVAALFWCIVAAGCSPVFAFVFSNLLQAFWAQGSKLEAGTKWAIVLGCVGILD
48_10124   DTKHRLYAVAALFWCIVAAGCSPVFAFVFSNLLQAFWAQGSKLEAGTKWAIVLGCVGILD
49_7151    DTKHRLYAVAALFWCIVAAGCSPVFAFVFSNLLQAFWAQGSKLEAGTKWAIVLGCVGILD
50_3695    DTKHRLYAVAALFWCIVAAGCSPVFAFVFSNLLQAFWAQGSKLEAGTKWAIVLGCVGILD
51_364     DTKHRLYAVAALFWCIVAAGCSPVFAFVFSNLLQAFWAQGSKLEAGTKWAIVLGCVGILD
52_242     DTKHRLYAVAALFWCIVAAGCSPVFAFVFSNLLQAFWAQGSKLEAGTKWAIVLGCVGILD
53_5225    DTKHRLYAVAALFWCIVAAGCSPVFAFVFSNLLQAFWAQGSKLEAGTKWAIVLGCVGILD
54_1635    DTKHRLYAVAALFWCIVAAGCSPVFAFVFSNLLQAFWAQGSKLEAGTKWAIVLGCVGILD
55_5766    DTKHRLYAVAALFWCIVAAGCSPVFAFVFSNLLQAFWAQGSKLEAGTKWAIVLGCVGILD
56_1557    DTKHRLYAVAALFWCIVAAGCSPVFAFVFSNLLQAFWAQGSKLEAGTKWAIVLGCVGILD
57_10137   DTKHRLYAVAALFWCIVAAGCSPVFAFVFSNLLQAFWAQGSKLEAGTKWAIVLGCVGILD
58_10525   DTKHRLYAVAALFWCIVAAGCSPVFAFVFSNLLQAFWAQGSKLEAGTKWAIVLGCVGILD
59_3457    DTKHRLYAVAALFWCIVAAGCSPVFAFVFSNLLQAFWAQGSKLEAGTKWAIVLGCVGILD
60_8244    DTKHRLYAVAALFWCIVAAGCSPVFAFVFSNLLQAFWAQGSKLEAGTKWAIVLGCVGILD
61_7964    DTKHRLYAVAALFWCIVAAGCSPVFAFVFSNLLQAFWAQGSKLEAGTKWAIVLGCVGILD
62_4413    DTKHRLYAVAALFWCIVAAGCSPVFAFVFSNLLQAFWAQGSKLEAGTKWAIVLGCVGILD
63_6958    DTKHRLYAVAALFWCIVAAGCSPVFAFVFSNLLQAFWAQGSKLEAGTKWAIVLGCVGILD
64_5997    GVALSGGQAQRLCIARALARRPKLLVLDEPTSALDAESGQSVMDTLRTLVCRRDGDLSTA
65_8711    DTKHRLYAVAALFWCIVAAGCSPVFAFVFSNLLQAFWAQGSKLEAGTKWAIVLGCVGILD
66_9925    DTKHRLYAVAALFWCIVAAGCSPVFAFVFSNLLQAFWAQGSKLEAGTKWAIVLGCVGILD
67_10374   DTKHRLYAVAALFWCIVAAGCSPVFAFVFSNLLQAFWAQGSKLEAGTKWAIVLGCVGILD
68_3713    DTKHRLYAVAALFWCIVAAGCSPVFAFVFSNLLQAFWAQGSKLEAGTKWAIVLGCVGILD
69_4511    DTKHRLYAVAALFWCIVAAGCSPVFAFVFSNLLQAFWAQGSKLEAGTKWAIVLGCVGILD
70_6201    DTKHRLYAVAALFWCIVAAGCSPVFAFVFSNLLQAFWAQGSKLEAGTKWAIVLGCVGILD
71_3261    DTKHRLYAVAALFWCIVAAGCSPVFAFVFSNLLQAFWAQGSKLEAGTKWAIVLGCVGILD
72_8516    DTKHRLYAVAALFWCIVAAGCSPVFAFVFSNLLQAFWAQGSKLEAGTKWAIVLGCVGILD
73_9295    DTKHRLYAVAALFWCIVAAGCSPVFAFVFSNLLQAFWAQGSKLEAGTKWAIVLGCVGILD
74_809     DTKHRLYAVAALFWCIVAAGCSPVFAFVFSNLLQAFWAQGSKLEAGTKWAIVLGCVGILD
75_7282    DTKHRLYAVAALFWCIVAAGCSPVFAFVFSNLLQAFWAQGSKLEAGTKWAIVLGCVGILD
76_4910    DTKHRLYAVAALFWCIVAAGCSPVFAFVFSNLLQAFWAQGSKLEAGTKWAIVLGCVGILD
77_10912   DTKHRLYAVAALFWCIVAAGCSPVFAFVFSNLLQAFWAQGSKLEAGTKWAIVLGCVGILD
78_4020    DTKHRLYAVAALFWCIVAAGCSPVFAFVFSNLLQAFWAQGSKLEAGTKWAIVLGCVGILD
79_6703    DTKHRLYAVAALFWCIVAAGCSPVFAFVFSNLLQAFWAQGSKLEAGTKWAIVLGCVGILD
80_8619    DTKHRLYAVAALFWCIVAAGCSPVFAFVFSNLLQAFWAQGSKLEAGTKWAIVLGCVGILD
81_4126    FWCIVAAGCSPVFAFVFSNLLQAFWAQGSKLEAGTKWAIVLGCVGILDFTAVFFTFFLAG
82_1363    DTKHRLYAVAALFWCIVAAGCSPVFAFVFSNLLQAFWAQGSKLEAGTKWAIVLGCVGILD
83_5813    DTKHRLYAVAALFWCIVAAGCSPVFAFVFSNLLQAFWAQGSKLEAGTKWAIVLGCVGILD
84_2044    DTKHRLYAVAALFWCIVAAGCSPVFAFVFSNLLQAFWAQGSKLEAGTKWAIVLGCVGILD
85_7617    DTKHRLYAVAALFWCIVAAGCSPVFAFVFSNLLQAFWAQGSKLEAGTKWAIVLGCVGILD
86_1530    DTKHRLYAVAALFWCIVAAGCSPVFAFVFSNLLQAFWAQGSKLEAGTKWAIVLGCVGILD
87_1890    DTKHRLYAVAALFWCIVAAGCSPVFAFVFSNLLQAFWAQGSKLEAGTKWAIVLGCVGILD
88_7049    DTKHRLYAVAALFWCIVAAGCSPVFAFVFSNLLQAFWAQGSKLEAGTKWAIVLGCVGILD
89_440     DTKHRLYAVAALFWCIVAAGCSPVFAFVFSNLLQAFWAQGSKLEAGTKWAIVLGCVGILD
90_4331    DTKHRLYAVAALFWCIVAAGCSPVFAFVFSNLLQAFWAQGSKLEAGTKWAIVLGCVGILD
```

```
                     970       980       990       1000      1010      1020
1_1896      FTAVFFTFFLAGAVAQEWVDTLKVDAFFSILCQPRAWHDRSKNSAARVCDVLDRGADEMR
2_4785      FTAVFFTFFLAGAVAQEWVDTLKVDAFFSILCQPRAWHDRSKNSAARVCDVLDRGADEMR
3_3653      FTAVFFTFFLAGAVAQEWVDTLKVDAFFSILCQPRAWHDRSKNSAARVCDVLDRGADEMR
4_8157      FTAVFFTFFLAGAVAQEWVDTLKVDAFFSILCQPRAWHDRSKNSAARVCDVLDRGADEMR
5_752       FTAVFFTFFLAGAVAQEWVDTLKVDAFFSILCQPRAWHDRSKNSAARVCDVLDRGADEMR
6_8853      FTAVFFTFFLAGAVAQEWVDTLKVDAFFSILCQPRAWHDRSKNSAARVCDVLDRGADEMR
7_4015      SEQQELGKKRILAPFPIVFNKLRFAYPGRDQQVLRNVSLSIPSEGCTAIVGGSGCGKSTI
8_2020      FTAVFFTFFLAGAVAQEWVDTLKVDAFFSILCQPRAWHDRSKNSAARVCDVLDRGADEMR
9_1829      FTAVFFTFFLAGAVAQEWVDTLKVDAFFSILCQPRAWHDRSKNSAARVCDVLDRGADEMR
10_6452     FTAVFFTFFLAGAVAQEWVDTLKVDAFFSILCQPRAWHDRSKNSAARVCDVLDRGADEMR
11_8627     FTAVFFTFFLAGAVAQEWVDTLKVDAFFSILCQPRAWHDRSKNSAARVCDVLDRGADEMR
12_6118     FTAVFFTFFLAGAVAQEWVDTLKVDAFFSILCQPRAWHDRSKNSAARVCDVLDRGADEMR
13_2688     FTAVFFTFFLAGAVAQEWVDTLKVDAFFSILCQPRAWHDRSKNSAARVCDVLDRGADEMR
14_6406     FTAVFFTFFLAGAVAQEWVDTLKVDAFFSILCQPRAWHDRSKNSAARVCDVLDRGADEMR
15_9419     FTAVFFTFFLAGAVAQEWVDTLKVDAFFSILCQPRAWHDRSKNSAARVCDVLDRGADEMR
16_8507     FTAVFFTFFLAGAVAQEWVDTLKVDAFFSILCQPRAWHDRSKNSAARVCDVLDRGADEMR
17_5884     FTAVFFTFFLAGAVAQEWVDTLKVDAFFSILCQPRAWHDRSKNSAARVCDVLDRGADEMR
18_2670     FTAVFFTFFLAGAVAQEWVDTLKVDAFFSILCQPRAWHDRSKNSAARVCDVLDRGADEMR
19_5172     FTAVFFTFFLAGAVAQEWVDTLKVDAFFSILCQPRAWHDRSKNSAARVCDVLDRGADEMR
20_8057     FTAVFFTFFLAGAVAQEWVDTLKVDAFFSILCQPRAWHDRSKNSAARVCDVLDRGADEMR
21_4920     IISLPQGYETLVGDGGVALSGGQAQRLCIARALARRPKLLVLDEPTSALDAESGQSVMDT
22_6393     FTAVFFTFFLAGAVAQEWVDTLKVDAFFSILCQPRAWHDRSKNSAARVCDVLDRGADEMR
23_8263     FTAVFFTFFLAGAVAQEWVDTLKVDAFFSILCQPRAWHDRSKNSAARVCDVLDRGADEMR
24_8248     FTAVFFTFFLAGAVAQEWVDTLKVDAFFSILCQPRAWHDRSKNSAARVCDVLDRGADEMR
25_6733     FTAVFFTFFLAGAVAQEWVDTLKVDAFFSILCQPRAWHDRSKNSAARVCDVLDRGADEMR
26_2277     FTAVFFTFFLAGAVAQEWVDTLKVDAFFSILCQPRAWHDRSKNSAARVCDVLDRGADEMR
27_2623     FTAVFFTFFLAGAVAQEWVDTLKVDAFFSILCQPRAWHDRSKNSAARVCDVLDRGADEMR
28_7413     FTAVFFTFFLAGAVAQEWVDTLKVDAFFSILCQPRAWHDRSKNSAARVCDVLDRGADEMR
29_8980     FTAVFFTFFLAGAVAQEWVDTLKVDAFFSILCQPRAWHDRSKNSAARVCDVLDRGADEMR
30_8706     FTAVFFTFFLAGAVAQEWVDTLKVDAFFSILCQPRAWHDRSKNSAARVCDVLDRGADEMR
31_6378     FTAVFFTFFLAGAVAQEWVDTLKVDAFFSILCQPRAWHDRSKNSAARVCDVLDRGADEMR
32_4679     FTAVFFTFFLAGAVAQEWVDTLKVDAFFSILCQPRAWHDRSKNSAARVCDVLDRGADEMR
33_2437     FTAVFFTFFLAGAVAQEWVDTLKVDAFFSILCQPRAWHDRSKNSAARVCDVLDRGADEMR
34_9700     FTAVFFTFFLAGAVAQEWVDTLKVDAFFSILCQPRAWHDRSKNSAARVCDVLDRGADEMR
35_10218    FTAVFFTFFLAGAVAQEWVDTLKVDAFFSILCQPRAWHDRSKNSAARVCDVLDRGADEMR
36_2652     FTAVFFTFFLAGAVAQEWVDTLKVDAFFSILCQPRAWHDRSKNSAARVCDVLDRGADEMR
37_5779     FTAVFFTFFLAGAVAQEWVDTLKVDAFFSILCQPRAWHDRSKNSAARVCDVLDRGADEMR
38_654      FTAVFFTFFLAGAVAQEWVDTLKVDAFFSILCQPRAWHDRSKNSAARVCDVLDRGADEMR
39_4879     FTAVFFTFFLAGAVAQEWVDTLKVDAFFSILCQPRAWHDRSKNSAARVCDVLDRGADEMR
40_8113     FTAVFFTFFLAGAVAQEWVDTLKVDAFFSILCQPRAWHDRSKNSAARVCDVLDRGADEMR
41_1767     AVAQEWVDTLKVDAFFSILCQPRAWHDRSKNSAARVCDVLDRGADEMRTIVAQFTPIALI
42_8428     FTAVFFTFFLAGAVAQEWVDTLKVDAFFSILCQPRAWHDRSKNSAARVCDVLDRGADEMR
43_9819     FTAVFFTFFLAGAVAQEWVDTLKVDAFFSILCQPRAWHDRSKNSAARVCDVLDRGADEMR
44_5569     FTAVFFTFFLAGAVAQEWVDTLKVDAFFSILCQPRAWHDRSKNSAARVCDVLDRGADEMR
45_4189     FTAVFFTFFLAGAVAQEWVDTLKVDAFFSILCQPRAWHDRSKNSAARVCDVLDRGADEMR
46_1027     FTAVFFTFFLAGAVAQEWVDTLKVDAFFSILCQPRAWHDRSKNSAARVCDVLDRGADEMR
47_5048     FTAVFFTFFLAGAVAQEWVDTLKVDAFFSILCQPRAWHDRSKNSAARVCDVLDRGADEMR
48_10124    FTAVFFTFFLAGAVAQEWVDTLKVDAFFSILCQPRAWHDRSKNSAARVCDVLDRGADEMR
49_7151     FTAVFFTFFLAGAVAQEWVDTLKVDAFFSILCQPRAWHDRSKNSAARVCDVLDRGADEMR
50_3695     FTAVFFTFFLAGAVAQEWVDTLKVDAFFSILCQPRAWHDRSKNSAARVCDVLDRGADEMR
51_364      FTAVFFTFFLAGAVAQEWVDTLKVDAFFSILCQPRAWHDRSKNSAARVCDVLDRGADEMR
52_242      FTAVFFTFFLAGAVAQEWVDTLKVDAFFSILCQPRAWHDRSKNSAARVCDVLDRGADEMR
53_5225     FTAVFFTFFLAGAVAQEWVDTLKVDAFFSILCQPRAWHDRSKNSAARVCDVLDRGADEMR
54_1635     FTAVFFTFFLAGAVAQEWVDTLKVDAFFSILCQPRAWHDRSKNSAARVCDVLDRGADEMR
55_5766     FTAVFFTFFLAGAVAQEWVDTLKVDAFFSILCQPRAWHDRSKNSAARVCDVLDRGADEMR
56_1557     FTAVFFTFFLAGAVAQEWVDTLKVDAFFSILCQPRAWHDRSKNSAARVCDVLDRGADEMR
57_10137    FTAVFFTFFLAGAVAQEWVDTLKVDAFFSILCQPRAWHDRSKNSAARVCDVLDRGADEMR
58_10525    FTAVFFTFFLAGAVAQEWVDTLKVDAFFSILCQPRAWHDRSKNSAARVCDVLDRGADEMR
59_3457     FTAVFFTFFLAGAVAQEWVDTLKVDAFFSILCQPRAWHDRSKNSAARVCDVLDRGADEMR
60_8244     FTAVFFTFFLAGAVAQEWVDTLKVDAFFSILCQPRAWHDRSKNSAARVCDVLDRGADEMR
61_7964     FTAVFFTFFLAGAVAQEWVDTLKVDAFFSILCQPRAWHDRSKNSAARVCDVLDRGADEMR
62_4413     FTAVFFTFFLAGAVAQEWVDTLKVDAFFSILCQPRAWHDRSKNSAARVCDVLDRGADEMR
63_6958     FTAVFFTFFLAGAVAQEWVDTLKVDAFFSILCQPRAWHDRSKNSAARVCDVLDRGADEMR
64_5997     AASPLLRKAGGTLSPYSPQHPDPYNWEGNFFRKSEGRSFSYVHGAAAGTQHRQPAVVVIT
65_8711     FTAVFFTFFLAGAVAQEWVDTLKVDAFFSILCQPRAWHDRSKNSAARVCDVLDRGADEMR
66_9925     FTAVFFTFFLAGAVAQEWVDTLKVDAFFSILCQPRAWHDRSKNSAARVCDVLDRGADEMR
67_10374    FTAVFFTFFLAGAVAQEWVDTLKVDAFFSILCQPRAWHDRSKNSAARVCDVLDRGADEMR
68_3713     FTAVFFTFFLAGAVAQEWVDTLKVDAFFSILCQPRAWHDRSKNSAARVCDVLDRGADEMR
69_4511     FTAVFFTFFLAGAVAQEWVDTLKVDAFFSILCQPRAWHDRSKNSAARVCDVLDRGADEMR
70_6201     FTAVFFTFFLAGAVAQEWVDTLKVDAFFSILCQPRAWHDRSKNSAARVCDVLDRGADEMR
71_3261     FTAVFFTFFLAGAVAQEWVDTLKVDAFFSILCQPRAWHDRSKNSAARVCDVLDRGADEMR
72_8516     FTAVFFTFFLAGAVAQEWVDTLKVDAFFSILCQPRAWHDRSKNSAARVCDVLDRGADEMR
73_9295     FTAVFFTFFLAGAVAQEWVDTLKVDAFFSILCQPRAWHDRSKNSAARVCDVLDRGADEMR
74_809      FTAVFFTFFLAGAVAQEWVDTLKVDAFFSILCQPRAWHDRSKNSAARVCDVLDRGADEMR
75_7282     FTAVFFTFFLAGAVAQEWVDTLKVDAFFSILCQPRAWHDRSKNSAARVCDVLDRGADEMR
76_4910     FTAVFFTFFLAGAVAQEWVDTLKVDAFFSILCQPRAWHDRSKNSAARVCDVLDRGADEMR
77_10912    FTAVFFTFFLAGAVAQEWVDTLKVDAFFSILCQPRAWHDRSKNSAARVCDVLDRGADEMR
78_4020     FTAVFFTFFLAGAVAQEWVDTLKVDAFFSILCQPRAWHDRSKNSAARVCDVLDRGADEMR
79_6703     FTAVFFTFFLAGAVAQEWVDTLKVDAFFSILCQPRAWHDRSKNSAARVCDVLDRGADEMR
80_8619     FTAVFFTFFLAGAVAQEWVDTLKVDAFFSILCQPRAWHDRSKNSAARVCDVLDRGADEMR
81_4126     AVAQEWVDTLKVDAFFSILCQPRAWHDRSKNSAARVCDVLDRGADEMRTIVAQFTPIALI
82_1363     FTAVFFTFFLAGAVAQEWVDTLKVDAFFSILCQPRAWHDRSKNSAARVCDVLDRGADEMR
83_5813     FTAVFFTFFLAGAVAQEWVDTLKVDAFFSILCQPRAWHDRSKNSAARVCDVLDRGADEMR
84_2044     FTAVFFTFFLAGAVAQEWVDTLKVDAFFSILCQPRAWHDRSKNSAARVCDVLDRGADEMR
85_7617     FTAVFFTFFLAGAVAQEWVDTLKVDAFFSILCQPRAWHDRSKNSAARVCDVLDRGADEMR
86_1530     FTAVFFTFFLAGAVAQEWVDTLKVDAFFSILCQPRAWHDRSKNSAARVCDVLDRGADEMR
87_1890     FTAVFFTFFLAGAVAQEWVDTLKVDAFFSILCQPRAWHDRSKNSAARVCDVLDRGADEMR
88_7049     FTAVFFTFFLAGAVAQEWVDTLKVDAFFSILCQPRAWHDRSKNSAARVCDVLDRGADEMR
89_440      FTAVFFTFFLAGAVAQEWVDTLKVDAFFSILCQPRAWHDRSKNSAARVCDVLDRGADEMR
90_4331     FTAVFFTFFLAGAVAQEWVDTLKVDAFFSILCQPRAWHDRSKNSAARVCDVLDRGADEMR
```

	1030 1040 1050 1060 1070 1080
1_1896	TIVAQFTPIALIVVIMVSSAIVWAMVILWQLTLVALAVFPFVGLCVFFSTRTSEKWEALS
2_4785	TIVAQFTPIALIVVIMVSSAIVWAMVILWQLTLVALAVFPFVGLCVFFSTRTSEKWEALS
3_3653	TIVAQFTPIALIVVIMVSSAIVWAMVILWQLTLVALAVFPFVGLCVFFSTRTSEKWEALS
4_8157	TIVAQFTPIALIVVIMVSSAIVWAMVILWQLTLVALAVFPFVGLCVFFSTRTSEKWEALS
5_752	TIVAQFTPIALIVVIMVSSAIVWAMVILWQLTLVALAVFPFVGLCVFFSTRTSEKWEALS
6_8853	TIVAQFTPIALIVVIMVSSAIVWAMVILWQLTLVALAVFPFVGLCVFFSTRTSEKWEALS
7_4015	AALILGLYEPLCDDDRSPVGGASPKAFRKGNFEVASPVSYPLGPVASSPLPSLVYSLPQG
8_2020	TIVAQFTPIALIVVIMVSSAIVWAMVILWQLTLVALAVFPFVGLCVFFSTRTSEKWEALS
9_1829	TIVAQFTPIALIVVIMVSSAIVWAMVILWQLTLVALAVFPFVGLCVFFSTRTSEKWEALS
10_6452	TIVAQFTPIALIVVIMVSSAIVWAMVILWQLTLVALAVFPFVGLCVFFSTRTSEKWEALS
11_8627	TIVAQFTPIALIVVIMVSSAIVWAMVILWQLTLVALAVFPFVGLCVFFSTRTSEKWEALS
12_6118	TIVAQFTPIALIVVIMVSSAIVWAMVILWQLTLVALAVFPFVGLCVFFSTRTSEKWEALS
13_2688	TIVAQFTPIALIVVIMVSSAIVWAMVILWQLTLVALAVFPFVGLCVFFSTRTSEKWEALS
14_6406	TIVAQFTPIALIVVIMVSSAIVWAMVILWQLTLVALAVFPFVGLCVFFSTRTSEKWEALS
15_9419	TIVAQFTPIALIVVIMVSSAIVWAMVILWQLTLVALAVFPFVGLCVFFSTRTSEKWEALS
16_8507	TIVAQFTPIALIVVIMVSSAIVWAMVILWQLTLVALAVFPFVGLCVFFSTRTSEKWEALS
17_5884	TIVAQFTPIALIVVIMVSSAIVWAMVILWQLTLVALAVFPFVGLCVFFSTRTSEKWEALS
18_2670	TIVAQFTPIALIVVIMVSSAIVWAMVILWQLTLVALAVFPFVGLCVFFSTRTSEKWEALS
19_5172	TIVAQFTPIALIVVIMVSSAIVWAMVILWQLTLVALAVFPFVGLCVFFSTRTSEKWEALS
20_8057	TIVAQFTPIALIVVIMVSSAIVWAMVILWQLTLVALAVFPFVGLCVFFSTRTSEKWEALS
21_4920	LRTLVCRRDGDLSTAAASPLLRKAGGTLSPYSPQHPDPYNWEGNFFRKSEGRSFSYVHGA
22_6393	TIVAQFTPIALIVVIMVSSAIVWAMVILWQLTLVALAVFPFVGLCVFFSTRTSEKWEALS
23_8263	TIVAQFTPIALIVVIMVSSAIVWAMVILWQLTLVALAVFPFVGLCVFFSTRTSEKWEALS
24_8248	TIVAQFTPIALIVVIMVSSAIVWAMVILWQLTLVALAVFPFVGLCVFFSTRTSEKWEALS
25_6733	TIVAQFTPIALIVVIMVSSAIVWAMVILWQLTLVALAVFPFVGLCVFFSTRTSEKWEALS
26_2277	TIVAQFTPIALIVVIMVSSAIVWAMVILWQLTLVALAVFPFVGLCVFFSTRTSEKWEALS
27_2623	TIVAQFTPIALIVVIMVSSAIVWAMVILWQLTLVALAVFPFVGLCVFFSTRTSEKWEALS
28_7413	TIVAQFTPIALIVVIMVSSAIVWAMVILWQLTLVALAVFPFVGLCVFFSTRTSEKWEALS
29_8980	TIVAQFTPIALIVVIMVSSAIVWAMVILWQLTLVALAVFPFVGLCVFFSTRTSEKWEALS
30_8706	TIVAQFTPIALIVVIMVSSAIVWAMVILWQLTLVALAVFPFVGLCVFFSTRTSEKWEALS
31_6378	TIVAQFTPIALIVVIMVSSAIVWAMVILWQLTLVALAVFPFVGLCVFFSTRTSEKWEALS
32_4679	TIVAQFTPIALIVVIMVSSAIVWAMVILWQLTLVALAVFPFVGLCVFFSTRTSEKWEALS
33_2437	TIVAQFTPIALIVVIMVSSAIVWAMVILWQLTLVALAVFPFVGLCVFFSTRTSEKWEALS
34_9700	TIVAQFTPIALIVVIMVSSAIVWAMVILWQLTLVALAVFPFVGLCVFFSTRTSEKWEALS
35_10218	TIVAQFTPIALIVVIMVSSAIVWAMVILWQLTLVALAVFPFVGLCVFFSTRTSEKWEALS
36_2652	TIVAQFTPIALIVVIMVSSAIVWAMVILWQLTLVALAVFPFVGLCVFFSTRTSEKWEALS
37_5779	TIVAQFTPIALIVVIMVSSAIVWAMVILWQLTLVALAVFPFVGLCVFFSTRTSEKWEALS
38_654	TIVAQFTPIALIVVIMVSSAIVWAMVILWQLTLVALAVFPFVGLCVFFSTRTSEKWEALS
39_4879	TIVAQFTPIALIVVIMVSSAIVWAMVILWQLTLVALAVFPFVGLCVFFSTRTSEKWEALS
40_8113	TIVAQFTPIALIVVIMVSSAIVWAMVILWQLTLVALAVFPFVGLCVFFSTRTSEKWEALS
41_1767	VVIMVSSAIVWAMVILWQLTLVALAVFPFVGLCVFFSTRTSEKWEALSNESAEATGGILS
42_8428	TIVAQFTPIALIVVIMVSSAIVWAMVILWQLTLVALAVFPFVGLCVFFSTRTSEKWEALS
43_9819	TIVAQFTPIALIVVIMVSSAIVWAMVILWQLTLVALAVFPFVGLCVFFSTRTSEKWEALS
44_5569	TIVAQFTPIALIVVIMVSSAIVWAMVILWQLTLVALAVFPFVGLCVFFSTRTSEKWEALS
45_4189	TIVAQFTPIALIVVIMVSSAIVWAMVILWQLTLVALAVFPFVGLCVFFSTRTSEKWEALS
46_1027	TIVAQFTPIALIVVIMVSSAIVWAMVILWQLTLVALAVFPFVGLCVFFSTRTSEKWEALS
47_5048	TIVAQFTPIALIVVIMVSSAIVWAMVILWQLTLVALAVFPFVGLCVFFSTRTSEKWEALS
48_10124	TIVAQFTPIALIVVIMVSSAIVWAMVILWQLTLVALAVFPFVGLCVFFSTRTSEKWEALS
49_7151	TIVAQFTPIALIVVIMVSSAIVWAMVILWQLTLVALAVFPFVGLCVFFSTRTSEKWEALS
50_3695	TIVAQFTPIALIVVIMVSSAIVWAMVILWQLTLVALAVFPFVGLCVFFSTRTSEKWEALS
51_364	TIVAQFTPIALIVVIMVSSAIVWAMVILWQLTLVALAVFPFVGLCVFFSTRTSEKWEALS
52_242	TIVAQFTPIALIVVIMVSSAIVWAMVILWQLTLVALAVFPFVGLCVFFSTRTSEKWEALS
53_5225	TIVAQFTPIALIVVIMVSSAIVWAMVILWQLTLVALAVFPFVGLCVFFSTRTSEKWEALS
54_1635	TIVAQFTPIALIVVIMVSSAIVWAMVILWQLTLVALAVFPFVGLCVFFSTRTSEKWEALS
55_5766	TIVAQFTPIALIVVIMVSSAIVWAMVILWQLTLVALAVFPFVGLCVFFSTRTSEKWEALS
56_1557	TIVAQFTPIALIVVIMVSSAIVWAMVILWQLTLVALAVFPFVGLCVFFSTRTSEKWEALS
57_10137	TIVAQFTPIALIVVIMVSSAIVWAMVILWQLTLVALAVFPFVGLCVFFSTRTSEKWEALS
58_10525	TIVAQFTPIALIVVIMVSSAIVWAMVILWQLTLVALAVFPFVGLCVFFSTRTSEKWEALS
59_3457	TIVAQFTPIALIVVIMVSSAIVWAMVILWQLTLVALAVFPFVGLCVFFSTRTSEKWEALS
60_8244	TIVAQFTPIALIVVIMVSSAIVWAMVILWQLTLVALAVFPFVGLCVFFSTRTSEKWEALS
61_7964	TIVAQFTPIALIVVIMVSSAIVWAMVILWQLTLVALAVFPFVGLCVFFSTRTSEKWEALS
62_4413	TIVAQFTPIALIVVIMVSSAIVWAMVILWQLTLVALAVFPFVGLCVFFSTRTSEKWEALS
63_6958	TIVAQFTPIALIVVIMVSSAIVWAMVILWQLTLVALAVFPFVGLCVFFSTRTSEKWEALS
64_5997	HSREMMKMADRLVVIDNGCVAETGTYEYLMAAGDSRLAELLDGGYRAPAAKSSGVPMENT
65_8711	TIVAQFTPIALIVVIMVSSAIVWAMVILWQLTLVALAVFPFVGLCVFFSTRTSEKWEALS
66_9925	TIVAQFTPIALIVVIMVSSAIVWAMVILWQLTLVALAVFPFVGLCVFFSTRTSEKWEALS
67_10374	TIVAQFTPIALIVVIMVSSAIVWAMVILWQLTLVALAVFPFVGLCVFFSTRTSEKWEALS
68_3713	TIVAQFTPIALIVVIMVSSAIVWAMVILWQLTLVALAVFPFVGLCVFFSTRTSEKWEALS
69_4511	TIVAQFTPIALIVVIMVSSAIVWAMVILWQLTLVALAVFPFVGLCVFFSTRTSEKWEALS
70_6201	TIVAQFTPIALIVVIMVSSAIVWAMVILWQLTLVALAVFPFVGLCVFFSTRTSEKWEALS
71_3261	TIVAQFTPIALIVVIMVSSAIVWAMVILWQLTLVALAVFPFVGLCVFFSTRTSEKWEALS
72_8516	TIVAQFTPIALIVVIMVSSAIVWAMVILWQLTLVALAVFPFVGLCVFFSTRTSEKWEALS
73_9295	TIVAQFTPIALIVVIMVSSAIVWAMVILWQLTLVALAVFPFVGLCVFFSTRTSEKWEALS
74_809	TIVAQFTPIALIVVIMVSSAIVWAMVILWQLTLVALAVFPFVGLCVFFSTRTSEKWEALS
75_7282	TIVAQFTPIALIVVIMVSSAIVWAMVILWQLTLVALAVFPFVGLCVFFSTRTSEKWEALS
76_4910	TIVAQFTPIALIVVIMVSSAIVWAMVILWQLTLVALAVFPFVGLCVFFSTRTSEKWEALS
77_10912	TIVAQFTPIALIVVIMVSSAIVWAMVILWQLTLVALAVFPFVGLCVFFSTRTSEKWEALS
78_4020	TIVAQFTPIALIVVIMVSSAIVWAMVILWQLTLVALAVFPFVGLCVFFSTRTSEKWEALS
79_6703	TIVAQFTPIALIVVIMVSSAIVWAMVILWQLTLVALAVFPFVGLCVFFSTRTSEKWEALS
80_8619	TIVAQFTPIALIVVIMVSSAIVWAMVILWQLTLVALAVFPFVGLCVFFSTRTSEKWEALS
81_4126	VVIMVSSAIVWAMVILWQLTLVALAVFPFVGLCVFFSTRTSEKWEALSNESAEATGGILS
82_1363	TIVAQFTPIALIVVIMVSSAIVWAMVILWQLTLVALAVFPFVGLCVFFSTRTSEKWEALS
83_5813	TIVAQFTPIALIVVIMVSSAIVWAMVILWQLTLVALAVFPFVGLCVFFSTRTSEKWEALS
84_2044	TIVAQFTPIALIVVIMVSSAIVWAMVILWQLTLVALAVFPFVGLCVFFSTRTSEKWEALS
85_7617	TIVAQFTPIALIVVIMVSSAIVWAMVILWQLTLVALAVFPFVGLCVFFSTRTSEKWEALS
86_1530	TIVAQFTPIALIVVIMVSSAIVWAMVILWQLTLVALAVFPFVGLCVFFSTRTSEKWEALS
87_1890	TIVAQFTPIALIVVIMVSSAIVWAMVILWQLTLVALAVFPFVGLCVFFSTRTSEKWEALS
88_7049	TIVAQFTPIALIVVIMVSSAIVWAMVILWQLTLVALAVFPFVGLCVFFSTRTSEKWEALS
89_440	TIVAQFTPIALIVVIMVSSAIVWAMVILWQLTLVALAVFPFVGLCVFFSTRTSEKWEALS
90_4331	TIVAQFTPIALIVVIMVSSAIVWAMVILWQLTLVALAVFPFVGLCVFFSTRTSEKWEALS

```
                  1090      1100      1110      1120      1130      1140
1_1896    NESAEATGGILSSVVSDIRVVRAFLLEKFFGDRFEAAAERAFTIGKKRGLYTGIWGGIHC
2_4785    NESAEATGGILSSVVSDIRVVRAFLLEKFFGDRFEAAAERAFTIGKKRGLYTGIWGGIHC
3_3653    NESAEATGGILSSVVSDIRVVRAFLLEKFFGDRFEAAAERAFTIGKKRGLYTGIWGGIHC
4_8157    NESAEATGGILSSVVSDIRVVRAFLLEKFFGDRFEAAAERAFTIGKKRGLYTGIWGGIHC
5_752     NESAEATGGILSSVVSDIRVVRAFLLEKFFGDRFEAAAERAFTIGKKRGLYTGIWGGIHC
6_8853    NESAEATGGILSSVVSDIRVVRAFLLEKFFGDRFEAAAERAFTIGKKRGLYTGIWGGIHC
7_4015    VMRAGSYRRSGELGALTYAGFDSREISTKALRSMMAYVPQHPFLFPGTIQGNIAYGLPED
8_2020    NESAEATGGILSSVVSDIRVVRAFLLEKFFGDRFEAAAERAFTIGKKRGLYTGIWGGIHC
9_1829    NESAEATGGILSSVVSDIRVVRAFLLEKFFGDRFEAAAERAFTIGKKRGLYTGIWGGIHC
10_6452   NESAEATGGILSSVVSDIRVVRAFLLEKFFGDRFEAAAERAFTIGKKRGLYTGIWGGIHC
11_8627   NESAEATGGILSSVVSDIRVVRAFLLEKFFGDRFEAAAERAFTIGKKRGLYTGIWGGIHC
12_6118   NESAEATGGILSSVVSDIRVVRAFLLEKFFGDRFEAAAERAFTIGKKRGLYTGIWGGIHC
13_2688   NESAEATGGILSSVVSDIRVVRAFLLEKFFGDRFEAAAERAFTIGKKRGLYTGIWGGIHC
14_6406   NESAEATGGILSSVVSDIRVVRAFLLEKFFGDRFEAAAERAFTIGKKRGLYTGIWGGIHC
15_9419   NESAEATGGILSSVVSDIRVVRAFLLEKFFGDRFEAAAERAFTIGKKRGLYTGIWGGIHC
16_8507   NESAEATGGILSSVVSDIRVVRAFLLEKFFGDRFEAAAERAFTIGKKRGLYTGIWGGIHC
17_5884   NESAEATGGILSSVVSDIRVVRAFLLEKFFGDRFEAAAERAFTIGKKRGLYTGIWGGIHC
18_2670   NESAEATGGILSSVVSDIRVVRAFLLEKFFGDRFEAAAERAFTIGKKRGLYTGIWGGIHC
19_5172   NESAEATGGILSSVVSDIRVVRAFLLEKFFGDRFEAAAERAFTIGKKRGLYTGIWGGIHC
20_8057   NESAEATGGILSSVVSDIRVVRAFLLEKFFGDRFEAAAERAFTIGKKRGLYTGIWGGIHC
21_4920   AAGTQHRQPAVVVITHSREMMKMADRLVVIDNGCVAETGTYEYLMAAGDSRLAELLDGGY
22_6393   NESAEATGGILSSVVSDIRVVRAFLLEKFFGDRFEAAAERAFTIGKKRGLYTGIWGGIHC
23_8263   NESAEATGGILSSVVSDIRVVRAFLLEKFFGDRFEAAAERAFTIGKKRGLYTGIWGGIHC
24_8248   NESAEATGGILSSVVSDIRVVRAFLLEKFFGDRFEAAAERAFTIGKKRGLYTGIWGGIHC
25_6733   NESAEATGGILSSVVSDIRVVRAFLLEKFFGDRFEAAAERAFTIGKKRGLYTGIWGGIHC
26_2277   NESAEATGGILSSVVSDIRVVRAFLLEKFFGDRFEAAAERAFTIGKKRGLYTGIWGGIHC
27_2623   NESAEATGGILSSVVSDIRVVRAFLLEKFFGDRFEAAAERAFTIGKKRGLYTGIWGGIHC
28_7413   NESAEATGGILSSVVSDIRVVRAFLLEKFFGDRFEAAAERAFTIGKKRGLYTGIWGGIHC
29_8980   NESAEATGGILSSVVSDIRVVRAFLLEKFFGDRFEAAAERAFTIGKKRGLYTGIWGGIHC
30_8706   NESAEATGGILSSVVSDIRVVRAFLLEKFFGDRFEAAAERAFTIGKKRGLYTGIWGGIHC
31_6378   NESAEATGGILSSVVSDIRVVRAFLLEKFFGDRFEAAAERAFTIGKKRGLYTGIWGGIHC
32_4679   NESAEATGGILSSVVSDIRVVRAFLLEKFFGDRFEAAAERAFTIGKKRGLYTGIWGGIHC
33_2437   NESAEATGGILSSVVSDIRVVRAFLLEKFFGDRFEAAAERAFTIGKKRGLYTGIWGGIHC
34_9700   NESAEATGGILSSVVSDIRVVRAFLLEKFFGDRFEAAAERAFTIGKKRGLYTGIWGGIHC
35_10218  NESAEATGGILSSVVSDIRVVRAFLLEKFFGDRFEAAAERAFTIGKKRGLYTGIWGGIHC
36_2652   NESAEATGGILSSVVSDIRVVRAFLLEKFFGDRFEAAAERAFTIGKKRGLYTGIWGGIHC
37_5779   NESAEATGGILSSVVSDIRVVRAFLLEKFFGDRFEAAAERAFTIGKKRGLYTGIWGGIHC
38_654    NESAEATGGILSSVVSDIRVVRAFLLEKFFGDRFEAAAERAFTIGKKRGLYTGIWGGIHC
39_4879   NESAEATGGILSSVVSDIRVVRAFLLEKFFGDRFEAAAERAFTIGKKRGLYTGIWGGIHC
40_8113   NESAEATGGILSSVVSDIRVVRAFLLEKFFGDRFEAAAERAFTIGKKRGLYTGIWGGIHC
41_1767   SVVSDIRVVRAFLLEKFFGDRFEAAAERAFTIGKKRGLYTGIWGGIHCSISQWLVVVIFT
42_8428   NESAEATGGILSSVVSDIRVVRAFLLEKFFGDRFEAAAERAFTIGKKRGLYTGIWGGIHC
43_9819   NESAEATGGILSSVVSDIRVVRAFLLEKFFGDRFEAAAERAFTIGKKRGLYTGIWGGIHC
44_5569   NESAEATGGILSSVVSDIRVVRAFLLEKFFGDRFEAAAERAFTIGKKRGLYTGIWGGIHC
45_4189   NESAEATGGILSSVVSDIRVVRAFLLEKFFGDRFEAAAERAFTIGKKRGLYTGIWGGIHC
46_1027   NESAEATGGILSSVVSDIRVVRAFLLEKFFGDRFEAAAERAFTIGKKRGLYTGIWGGIHC
47_5048   NESAEATGGILSSVVSDIRVVRAFLLEKFFGDRFEAAAERAFTIGKKRGLYTGIWGGIHC
48_10124  NESAEATGGILSSVVSDIRVVRAFLLEKFFGDRFEAAAERAFTIGKKRGLYTGIWGGIHC
49_7151   NESAEATGGILSSVVSDIRVVRAFLLEKFFGDRFEAAAERAFTIGKKRGLYTGIWGGIHC
50_3695   NESAEATGGILSSVVSDIRVVRAFLLEKFFGDRFEAAAERAFTIGKKRGLYTGIWGGIHC
51_364    NESAEATGGILSSVVSDIRVVRAFLLEKFFGDRFEAAAERAFTIGKKRGLYTGIWGGIHC
52_242    NESAEATGGILSSVVSDIRVVRAFLLEKFFGDRFEAAAERAFTIGKKRGLYTGIWGGIHC
53_5225   NESAEATGGILSSVVSDIRVVRAFLLEKFFGDRFEAAAERAFTIGKKRGLYTGIWGGIHC
54_1635   NESAEATGGILSSVVSDIRVVRAFLLEKFFGDRFEAAAERAFTIGKKRGLYTGIWGGIHC
55_5766   NESAEATGGILSSVVSDIRVVRAFLLEKFFGDRFEAAAERAFTIGKKRGLYTGIWGGIHC
56_1557   NESAEATGGILSSVVSDIRVVRAFLLEKFFGDRFEAAAERAFTIGKKRGLYTGIWGGIHC
57_10137  NESAEATGGILSSVVSDIRVVRAFLLEKFFGDRFEAAAERAFTIGKKRGLYTGIWGGIHC
58_10525  NESAEATGGILSSVVSDIRVVRAFLLEKFFGDRFEAAAERAFTIGKKRGLYTGIWGGIHC
59_3457   NESAEATGGILSSVVSDIRVVRAFLLEKFFGDRFEAAAERAFTIGKKRGLYTGIWGGIHC
60_8244   NESAEATGGILSSVVSDIRVVRAFLLEKFFGDRFEAAAERAFTIGKKRGLYTGIWGGIHC
61_7964   NESAEATGGILSSVVSDIRVVRAFLLEKFFGDRFEAAAERAFTIGKKRGLYTGIWGGIHC
62_4413   NESAEATGGILSSVVSDIRVVRAFLLEKFFGDRFEAAAERAFTIGKKRGLYTGIWGGIHC
63_6958   NESAEATGGILSSVVSDIRVVRAFLLEKFFGDRFEAAAERAFTIGKKRGLYTGIWGGIHC
64_5997   TSLEPVPVSPRTLTRVVSPSPEQYRRNNRSSPGGIPQRQQRPGMNMEDMFLPQVAWQEDD
65_8711   NESAEATGGILSSVVSDIRVVRAFLLEKFFGDRFEAAAERAFTIGKKRGLYTGIWGGIHC
66_9925   NESAEATGGILSSVVSDIRVVRAFLLEKFFGDRFEAAAERAFTIGKKRGLYTGIWGGIHC
67_10374  NESAEATGGILSSVVSDIRVVRAFLLEKFFGDRFEAAAERAFTIGKKRGLYTGIWGGIHC
68_3713   NESAEATGGILSSVVSDIRVVRAFLLEKFFGDRFEAAAERAFTIGKKRGLYTGIWGGIHC
69_4511   NESAEATGGILSSVVSDIRVVRAFLLEKFFGDRFEAAAERAFTIGKKRGLYTGIWGGIHC
70_6201   NESAEATGGILSSVVSDIRVVRAFLLEKFFGDRFEAAAERAFTIGKKRGLYTGIWGGIHC
71_3261   NESAEATGGILSSVVSDIRVVRAFLLEKFFGDRFEAAAERAFTIGKKRGLYTGIWGGIHC
72_8516   NESAEATGGILSSVVSDIRVVRAFLLEKFFGDRFEAAAERAFTIGKKRGLYTGIWGGIHC
73_9295   NESAEATGGILSSVVSDIRVVRAFLLEKFFGDRFEAAAERAFTIGKKRGLYTGIWGGIHC
74_809    NESAEATGGILSSVVSDIRVVRAFLLEKFFGDRFEAAAERAFTIGKKRGLYTGIWGGIHC
75_7282   NESAEATGGILSSVVSDIRVVRAFLLEKFFGDRFEAAAERAFTIGKKRGLYTGIWGGIHC
76_4910   NESAEATGGILSSVVSDIRVVRAFLLEKFFGDRFEAAAERAFTIGKKRGLYTGIWGGIHC
77_10912  NESAEATGGILSSVVSDIRVVRAFLLEKFFGDRFEAAAERAFTIGKKRGLYTGIWGGIHC
78_4020   NESAEATGGILSSVVSDIRVVRAFLLEKFFGDRFEAAAERAFTIGKKRGLYTGIWGGIHC
79_6703   NESAEATGGILSSVVSDIRVVRAFLLEKFFGDRFEAAAERAFTIGKKRGLYTGIWGGIHC
80_8619   NESAEATGGILSSVVSDIRVVRAFLLEKFFGDRFEAAAERAFTIGKKRGLYTGIWGGIHC
81_4126   SVVSDIRVVRAFLLEKFFGDRFEAAAERAFTIGKKRGLYTGIWGGIHCSISQWLVVVIFT
82_1363   NESAEATGGILSSVVSDIRVVRAFLLEKFFGDRFEAAAERAFTIGKKRGLYTGIWGGIHC
83_5813   NESAEATGGILSSVVSDIRVVRAFLLEKFFGDRFEAAAERAFTIGKKRGLYTGIWGGIHC
84_2044   NESAEATGGILSSVVSDIRVVRAFLLEKFFGDRFEAAAERAFTIGKKRGLYTGIWGGIHC
85_7617   NESAEATGGILSSVVSDIRVVRAFLLEKFFGDRFEAAAERAFTIGKKRGLYTGIWGGIHC
86_1530   NESAEATGGILSSVVSDIRVVRAFLLEKFFGDRFEAAAERAFTIGKKRGLYTGIWGGIHC
87_1890   NESAEATGGILSSVVSDIRVVRAFLLEKFFGDRFEAAAERAFTIGKKRGLYTGIWGGIHC
88_7049   NESAEATGGILSSVVSDIRVVRAFLLEKFFGDRFEAAAERAFTIGKKRGLYTGIWGGIHC
89_440    NESAEATGGILSSVVSDIRVVRAFLLEKFFGDRFEAAAERAFTIGKKRGLYTGIWGGIHC
90_4331   NESAEATGGILSSVVSDIRVVRAFLLEKFFGDRFEAAAERAFTIGKKRGLYTGIWGGIHC
```

```
                1150      1160      1170      1180      1190      1200
1_1896     SISQWLVVVIFTFGVFLLTTAESVNVGDIIQVVNLLMFTVGSATMMMSNIPQIAAAQAKA
2_4785     SISQWLVVVIFTFGVFLLTTAESVNVGDIIQVVNLLMFTVGSATMMMSNIPQIAAAQAKA
3_3653     SISQWLVVVIFTFGVFLLTTAESVNVGDIIQVVNLLMFTVGSATMMMSNIPQIAAAQAKA
4_8157     SISQWLVVVIFTFGVFLLTTAESVNVGDIIQVVNLLMFTVGSATMMMSNIPQIAAAQAKA
5_752      SISQWLVVVIFTFGVFLLTTAESVNVGDIIQVVNLLMFTVGSATMMMSNIPQIAAAQAKA
6_8853     SISQWLVVVIFTFGVFLLTTAESVNVGDIIQVVNLLMFTVGSATMMMSNIPQIAAAQAKA
7_4015     EIVLRGQHNIELAAREADIHEFIISLPQGYETLVGDGGVALSGGQAQRLCIARALARRPK
8_2020     SISQWLVVVIFTFGVFLLTTAESVNVGDIIQVVNLLMFTVGSATMMMSNIPQIAAAQAKA
9_1829     SISQWLVVVIFTFGVFLLTTAESVNVGDIIQVVNLLMFTVGSATMMMSNIPQIAAAQAKA
10_6452    SISQWLVVVIFTFGVFLLTTAESVNVGDIIQVVNLLMFTVGSATMMMSNIPQIAAAQAKA
11_8627    SISQWLVVVIFTFGVFLLTTAESVNVGDIIQVVNLLMFTVGSATMMMSNIPQIAAAQAKA
12_6118    SISQWLVVVIFTFGVFLLTTAESVNVGDIIQVVNLLMFTVGSATMMMSNIPQIAAAQAKA
13_2688    SISQWLVVVIFTFGVFLLTTAESVNVGDIIQVVNLLMFTVGSATMMMSNIPQIAAAQAKA
14_6406    SISQWLVVVIFTFGVFLLTTAESVNVGDIIQVVNLLMFTVGSATMMMSNIPQIAAAQAKA
15_9419    SISQWLVVVIFTFGVFLLTTAESVNVGDIIQVVNLLMFTVGSATMMMSNIPQIAAAQAKA
16_8507    SISQWLVVVIFTFGVFLLTTAESVNVGDIIQVVNLLMFTVGSATMMMSNIPQIAAAQAKA
17_5884    SISQWLVVVIFTFGVFLLTTAESVNVGDIIQVVNLLMFTVGSATMMMSNIPQIAAAQAKA
18_2670    SISQWLVVVIFTFGVFLLTTAESVNVGDIIQVVNLLMFTVGSATMMMSNIPQIAAAQAKA
19_5172    SISQWLVVVIFTFGVFLLTTAESVNVGDIIQVVNLLMFTVGSATMMMSNIPQIAAAQAKA
20_8057    SISQWLVVVIFTFGVFLLTTAESVNVGDIIQVVNLLMFTVGSATMMMSNIPQIAAAQAKA
21_4920    RAPAAKSSGVPMEKTTSLEPVPVSPRTLTRVVSPSPEQYRRNNRSSPGGIPQRQQRPGMN
22_6393    SISQWLVVVIFTFGVFLLTTAESVNVGDIIQVVNLLMFTVGSATMMMSNIPQIAAAQAKA
23_8263    SISQWLVVVIFTFGVFLLTTAESVNVGDIIQVVNLLMFTVGSATMMMSNIPQIAAAQAKA
24_8248    SISQWLVVVIFTFGVFLLTTAESVNVGDIIQVVNLLMFTVGSATMMMSNIPQIAAAQAKA
25_6733    SISQWLVVVIFTFGVFLLTTAESVNVGDIIQVVNLLMFTVGSATMMMSNIPQIAAAQAKA
26_2277    SISQWLVVVIFTFGVFLLTTAESVNVGDIIQVVNLLMFTVGSATMMMSNIPQIAAAQAKA
27_2623    SISQWLVVVIFTFGVFLLTTAESVNVGDIIQVVNLLMFTVGSATMMMSNIPQIAAAQAKA
28_7413    SISQWLVVVIFTFGVFLLTTAESVNVGDIIQVVNLLMFTVGSATMMMSNIPQIAAAQAKA
29_8980    SISQWLVVVIFTFGVFLLTTAESVNVGDIIQVVNLLMFTVGSATMMMSNIPQIAAAQAKA
30_8706    SISQWLVVVIFTFGVFLLTTAESVNVGDIIQVVNLLMFTVGSATMMMSNIPQIAAAQAKA
31_6378    SISQWLVVVIFTFGVFLLTTAESVNVGDIIQVVNLLMFTVGSATMMMSNIPQIAAAQAKA
32_4679    SISQWLVVVIFTFGVFLLTTAESVNVGDIIQVVNLLMFTVGSATMMMSNIPQIAAAQAKA
33_2437    SISQWLVVVIFTFGVFLLTTAESVNVGDIIQVVNLLMFTVGSATMMMSNIPQIAAAQAKA
34_9700    SISQWLVVVIFTFGVFLLTTAESVNVGDIIQVVNLLMFTVGSATMMMSNIPQIAAAQAKA
35_10218   SISQWLVVVIFTFGVFLLTTAESVNVGDIIQVVNLLMFTVGSATMMMSNIPQIAAAQAKA
36_2652    SISQWLVVVIFTFGVFLLTTAESVNVGDIIQVVNLLMFTVGSATMMMSNIPQIAAAQAKA
37_5779    SISQWLVVVIFTFGVFLLTTAESVNVGDIIQVVNLLMFTVGSATMMMSNIPQIAAAQAKA
38_654     SISQWLVVVIFTFGVFLLTTAESVNVGDIIQVVNLLMFTVGSATMMMSNIPQIAAAQAKA
39_4879    SISQWLVVVIFTFGVFLLTTAESVNVGDIIQVVNLLMFTVGSATMMMSNIPQIAAAQAKA
40_8113    SISQWLVVVIFTFGVFLLTTAESVNVGDIIQVVNLLMFTVGSATMMMSNIPQIAAAQAKA
41_1767    FGVFLLTTAESVNVGDIIQVVNLLMFTVGSATMMMSNIPQIAAAQAKAAQMLHLAKMPRD
42_8428    SISQWLVVVIFTFGVFLLTTAESVNVGDIIQVVNLLMFTVGSATMMMSNIPQIAAAQAKA
43_9819    SISQWLVVVIFTFGVFLLTTAESVNVGDIIQVVNLLMFTVGSATMMMSNIPQIAAAQAKA
44_5569    SISQWLVVVIFTFGVFLLTTAESVNVGDIIQVVNLLMFTVGSATMMMSNIPQIAAAQAKA
45_4189    SISQWLVVVIFTFGVFLLTTAESVNVGDIIQVVNLLMFTVGSATMMMSNIPQIAAAQAKA
46_1027    SISQWLVVVIFTFGVFLLTTAESVNVGDIIQVVNLLMFTVGSATMMMSNIPQIAAAQAKA
47_5048    SISQWLVVVIFTFGVFLLTTAESVNVGDIIQVVNLLMFTVGSATMMMSNIPQIAAAQAKA
48_10124   SISQWLVVVIFTFGVFLLTTAESVNVGDIIQVVNLLMFTVGSATMMMSNIPQIAAAQAKA
49_7151    SISQWLVVVIFTFGVFLLTTAESVNVGDIIQVVNLLMFTVGSATMMMSNIPQIAAAQAKA
50_3695    SISQWLVVVIFTFGVFLLTTAESVNVGDIIQVVNLLMFTVGSATMMMSNIPQIAAAQAKA
51_364     SISQWLVVVIFTFGVFLLTTAESVNVGDIIQVVNLLMFTVGSATMMMSNIPQIAAAQAKA
52_242     SISQWLVVVIFTFGVFLLTTAESVNVGDIIQVVNLLMFTVGSATMMMSNIPQIAAAQAKA
53_5225    SISQWLVVVIFTFGVFLLTTAESVNVGDIIQVVNLLMFTVGSATMMMSNIPQIAAAQAKA
54_1635    SISQWLVVVIFTFGVFLLTTAESVNVGDIIQVVNLLMFTVGSATMMMSNIPQIAAAQAKA
55_5766    SISQWLVVVIFTFGVFLLTTAESVNVGDIIQVVNLLMFTVGSATMMMSNIPQIAAAQAKA
56_1557    SISQWLVVVIFTFGVFLLTTAESVNVGDIIQVVNLLMFTVGSATMMMSNIPQIAAAQAKA
57_10137   SISQWLVVVIFTFGVFLLTTAESVNVGDIIQVVNLLMFTVGSATMMMSNIPQIAAAQAKA
58_10525   SISQWLVVVIFTFGVFLLTTAESVNVGDIIQVVNLLMFTVGSATMMMSNIPQIAAAQAKA
59_3457    SISQWLVVVIFTFGVFLLTTAESVNVGDIIQVVNLLMFTVGSATMMMSNIPQIAAAQAKA
60_8244    SISQWLVVVIFTFGVFLLTTAESVNVGDIIQVVNLLMFTVGSATMMMSNIPQIAAAQAKA
61_7964    SISQWLVVVIFTFGVFLLTTAESVNVGDIIQVVNLLMFTVGSATMMMSNIPQIAAAQAKA
62_4413    SISQWLVVVIFTFGVFLLTTAESVNVGDIIQVVNLLMFTVGSATMMMSNIPQIAAAQAKA
63_6958    SISQWLVVVIFTFGVFLLTTAESVNVGDIIQVVNLLMFTVGSATMMMSNIPQIAAAQAKA
64_5997    RS..........................................................
65_8711    SISQWLVVVIFTFGVFLLTTAESVNVGDIIQVVNLLMFTVGSATMMMSNIPQIAAAQAKA
66_9925    SISQWLVVVIFTFGVFLLTTAESVNVGDIIQVVNLLMFTVGSATMMMSNIPQIAAAQAKA
67_10374   SISQWLVVVIFTFGVFLLTTAESVNVGDIIQVVNLLMFTVGSATMMMSNIPQIAAAQAKA
68_3713    SISQWLVVVIFTFGVFLLTTAESVNVGDIIQVVNLLMFTVGSATMMMSNIPQIAAAQAKA
69_4511    SISQWLVVVIFTFGVFLLTTAESVNVGDIIQVVNLLMFTVGSATMMMSNIPQIAAAQAKA
70_6201    SISQWLVVVIFTFGVFLLTTAESVNVGDIIQVVNLLMFTVGSATMMMSNIPQIAAAQAKA
71_3261    SISQWLVVVIFTFGVFLLTTAESVNVGDIIQVVNLLMFTVGSATMMMSNIPQIAAAQAKA
72_8516    SISQWLVVVIFTFGVFLLTTAESVNVGDIIQVVNLLMFTVGSATMMMSNIPQIAAAQAKA
73_9295    SISQWLVVVIFTFGVFLLTTAESVNVGDIIQVVNLLMFTVGSATMMMSNIPQIAAAQAKA
74_809     SISQWLVVVIFTFGVFLLTTAESVNVGDIIQVVNLLMFTVGSATMMMSNIPQIAAAQAKA
75_7282    SISQWLVVVIFTFGVFLLTTAESVNVGDIIQVVNLLMFTVGSATMMMSNIPQIAAAQAKA
76_4910    SISQWLVVVIFTFGVFLLTTAESVNVGDIIQVVNLLMFTVGSATMMMSNIPQIAAAQAKA
77_10912   SISQWLVVVIFTFGVFLLTTAESVNVGDIIQVVNLLMFTVGSATMMMSNIPQIAAAQAKA
78_4020    SISQWLVVVIFTFGVFLLTTAESVNVGDIIQVVNLLMFTVGSATMMMSNIPQIAAAQAKA
79_6703    SISQWLVVVIFTFGVFLLTTAESVNVGDIIQVVNLLMFTVGSATMMMSNIPQIAAAQAKA
80_8619    SISQWLVVVIFTFGVFLLTTAESVNVGDIIQVVNLLMFTVGSATMMMSNIPQIAAAQAKA
81_4126    FGVFLLTTAESVNVGDIIQVVNLLMFTVGSATMMMSNIPQIAAAQAKAAQMLHLAKMPRD
82_1363    SISQWLVVVIFTFGVFLLTTAESVNVGDIIQVVNLLMFTVGSATMMMSNIPQIAAAQAKA
83_5813    SISQWLVVVIFTFGVFLLTTAESVNVGDIIQVVNLLMFTVGSATMMMSNIPQIAAAQAKA
84_2044    SISQWLVVVIFTFGVFLLTTAESVNVGDIIQVVNLLMFTVGSATMMMSNIPQIAAAQAKA
85_7617    SISQWLVVVIFTFGVFLLTTAESVNVGDIIQVVNLLMFTVGSATMMMSNIPQIAAAQAKA
86_1530    SISQWLVVVIFTFGVFLLTTAESVNVGDIIQVVNLLMFTVGSATMMMSNIPQIAAAQAKA
87_1890    SISQWLVVVIFTFGVFLLTTAESVNVGDIIQVVNLLMFTVGSATMMMSNIPQIAAAQAKA
88_7049    SISQWLVVVIFTFGVFLLTTAESVNVGDIIQVVNLLMFTVGSATMMMSNIPQIAAAQAKA
89_440     SISQWLVVVIFTFGVFLLTTAESVNVGDIIQVVNLLMFTVGSATMMMSNIPQIAAAQAKA
90_4331    SISQWLVVVIFTFGVFLLTTAESVNVGDIIQVVNLLMFTVGSATMMMSNIPQIAAAQAKA
```

```
             1210      1220      1230      1240      1250      1260
1_1896    AQMLHLAKMPRDHPDQQELGKKRLLTPLPTVFNKLRFAYPGRDQQVLRNVSLSIPSEGCT
2_4785    AQMLHLAKMPRDHPDQQELGKKRLLTPLPTVFNKLRFAYPGRDQQVLRNVSLSIPSEGCT
3_3653    AQMLHLAKMPRDHPDQQELGKKRLLTPLPTVFNKLRFAYPGRDQQVLRNVSLSIPSEGCT
4_8157    AQMLHLAKMPRDHPDQQELGKKRLLTPLPTVFNKLRFAYPGRDQQVLRNVSLSIPSEGCT
5_752     AQMLHLAKMPRDHPDQQELGKKRLLTPLPTVFNKLRFAYPGRDQQVLRNVSLSIPSEGCT
6_8853    AQMLHLAKMPRDHPDQQELGKKRLLTPLPTVFNKLRFAYPGRDQQVLRNVSLSIPSEGCT
7_4015    LLVLDEPTSALDAESGQSVMDTLRTLVCRRDGGLSAAASPLLRRGDGTLSPYSPQHPDPY
8_2020    AQMLHLAKMPRDHPDQQELGKKRLLTPLPIVFNKLRFAYPGRDQQVLRNVSLSIPSEGCT
9_1829    AQMLHLAKMPRDHPDQQELGKKRLLTPLPTVFNKLRFAYPGRDQQVLRNVSLSIPSEGCT
10_6452   AQMLHLAKMPRDHPDQQELGKKRLLTPLPTVFNKLRFAYPGRDQQVLRNVSLSIPSEGCT
11_8627   AQMLHLAKMPRDHPDQQELGKKRLLTPLPTVFNKLRFAYPGRDQQVLRNVSLSIPSEGCT
12_6118   AQMLHLAKMPRDHPDQQELGKKRLLTPLPTVFNKLRFAYPGRDQQVLRNVSLSIPSEGCT
13_2688   AQMLHLAKMPRDHPDQQELGKKRLLTPLPTVFNKLRFAYPGRDQQVLRNVSLSIPSEGCT
14_6406   AQMLHLAKMPRDHPDQQELGKKRLLTPLPTVFNKLRFAYPGRDQQVLRNVSLSIPSEGCT
15_9419   AQMLHLAKMPRDHPDQQELGKKRLLTPLPTVFNKLRFAYPGRDQQVLRNVSLSIPSEGCT
16_8507   AQMLHLAKMPRDHPDQQELGKKRLLTPLPTVFNKLRFAYPGRDQQVLRNVSLSIPSEGCT
17_5884   AQMLHLAKMPRDHPDQQELGKKRLLTPLPTVFNKLRFAYPGRDQQVLRNVSLSIPSEGCT
18_2670   AQMLHLAKMPRDHPDQQELGKKRLLTPLPTVFNKLRFAYPGRDQQVLRNVSLSIPSEGCT
19_5172   AQMLHLAKMPRDHPDQQELGKKRLLTPLPTVFNKLRFAYPGRDQQVLRNVSLSIPSEGCT
20_8057   AQMLHLAKMPRDHPDQQELGKKRLLTPLPTVFNKLRFAYPGRDQQVLRNVSLSIPSEGCT
21_4920   MEDMFLPQVAWQEDDRS...........................................
22_6393   AQMLHLAKMPRDHPDQQELGKKRLLTPLPTVFNKLRFAYPGRDQQVLRNVSLSIPSEGCT
23_8263   AQMLHLAKMPRDHPDQQELGKKRLLTPLPTVFNKLRFAYPGRDQQVLRNVSLSIPSEGCT
24_8248   AQMLHLAKMPRDHPDQQELGKKRLLTPLPTVFNKLRFAYPGRDQQVLRNVSLSIPSEGCT
25_6733   AQMLHLAKMPRDHPDQQELGKKRLLTPLPTVFNKLRFAYPGRDQQVLRNVSLSIPSEGCT
26_2277   AQMLHLAKMPRDHPDQQELGKKRLLTPLPTVFNKLRFAYPGRDQQVLRNVSLSIPSEGCT
27_2623   AQMLHLAKMPRDHPDQQELGKKRLLTPLPTVFNKLRFAYPGRDQQVLRNVSLSIPSEGCT
28_7413   AQMLHLAKMPRDHPDQQELGKKRLLTPLPTVFNKLRFAYPGRDQQVLRNVSLSIPSEGCT
29_8980   AQMLHLAKMPRDHPDQQELGKKRLLTPLPTVFNKLRFAYPGRDQQVLRNVSLSIPSEGCT
30_8706   AQMLHLAKMPRDHPDQQELGKKRLLTPLPTVFNKLRFAYPGRDQQVLRNVSLSIPSEGCT
31_6378   AQMLHLAKMPRDHPDQQELGKKRLLTPLPTVFNKLRFAYPGRDQQVLRNVSLSIPSEGCT
32_4679   AQMLHLAKMPRDHPDQQELGKKRLLTPLPTVFNKLRFAYPGRDQQVLRNVSLSIPSEGCT
33_2437   AQMLHLAKMPRDHPDQQELGKKRLLTPLPTVFNKLRFAYPGRDQQVLRNVSLSIPSEGCT
34_9700   AQMLHLAKMPRDHPDQQELGKKRLLTPLPTVFNKLRFAYPGRDQQVLRNVSLSIPSEGCT
35_10218  AQMLHLAKMPRDHPDQQELGKKRLLTPLPTVFNKLRFAYPGRDQQVLRNVSLSIPSEGCT
36_2652   AQMLHLAKMPRDHPDQQELGKKRLLTPLPTVFNKLRFAYPGRDQQVLRNVSLSIPSEGCT
37_5779   AQMLHLAKMPRDHPDQQELGKKRLLTPLPTVFNKLRFAYPGRDQQVLRNVSLSIPSEGCT
38_654    AQMLHLAKMPRDHPDQQELGKKRLLTPLPTVFNKLRFAYPGRDQQVLRNVSLSIPSEGCT
39_4879   AQMLHLAKMPRDHPDQQELGKKRLLTPLPTVFNKLRFAYPGRDQQVLRNVSLSIPSEGCT
40_8113   AQMLHLAKMPRDHPDQQELGKKRLLTPLPTVFNKLRFAYPGRDQQVLRNVSLSIPSEGCT
41_1767   HPDQQELGKKRLLTPLPTVFNKLRFAYPGRDQQVLRNVSLSIPSEGCTAIVGGSGCGKST
42_8428   AQMLHLAKMPRDHPDQQELGKKRLLTPLPTVFNKLRFAYPGRDQQVLRNVSLSIPSEGCT
43_9819   AQMLHLAKMPRDHPDQQELGKKRLLTPLPTVFNKLRFAYPGRDQQVLRNVSLSIPSEGCT
44_5569   AQMLHLAKMPRDHPDQQELGKKRLLTPLPTVFNKLRFAYPGRDQQVLRNVSLSIPSEGCT
45_4189   AQMLHLAKMPRDHPDQQELGKKRLLTPLPTVFNKLRFAYPGRDQQVLRNVSLSIPSEGCT
46_1027   AQMLHLAKMPRDHPDQQELGKKRLLTPLPTVFNKLRFAYPGRDQQVLRNVSLSIPSEGCT
47_5048   AQMLHLAKMPRDHPDQQELGKKRLLTPLPTVFNKLRFAYPGRDQQVLRNVSLSIPSEGCT
48_10124  AQMLHLAKMPRDHPDQQELGKKRLLTPLPTVFNKLRFAYPGRDQQVLRNVSLSIPSEGCT
49_7151   AQMLHLAKMPRDHPDQQELGKKRLLTPLPTVFNKLRFAYPGRDQQVLRNVSLSIPSEGCT
50_3695   AQMLHLAKMPRDHPDQQELGKKRLLTPLPTVFNKLRFAYPGRDQQVLRNVSLSIPSEGCT
51_364    AQMLHLAKMPRDHPDQQELGKKRLLTPLPTVFNKLRFAYPGRDQQVLRNVSLSIPSEGCT
52_242    AQMLHLAKMPRDHPDQQELGKKRLLTPLPTVFNKLRFAYPGRDQQVLRNVSLSIPSEGCT
53_5225   AQMLHLAKMPRDHPDQQELGKKRLLTPLPTVFNKLRFAYPGRDQQVLRNVSLSIPSEGCT
54_1635   AQMLHLAKMPRDHPDQQELGKKRLLTPLPTVFNKLRFAYPGRDQQVLRNVSLSIPSEGCT
55_5766   AQMLHLAKMPRDHPDQQELGKKRLLTPLPTVFNKLRFAYPGRDQQVLRNVSLSIPSEGCT
56_1557   AQMLHLAKMPRDHPDQQELGKKRLLTPLPTVFNKLRFAYPGRDQQVLRNVSLSIPSEGCT
57_10137  AQMLHLAKMPRDHPDQQELGKKRLLTPLPTVFNKLRFAYPGRDQQVLRNVSLSIPSEGCT
58_10525  AQMLHLAKMPRDHPDQQELGKKRLLTPLPTVFNKLRFAYPGRDQQVLRNVSLSIPSEGCT
59_3457   AQMLHLAKMPRDHPDQQELGKKRLLTPLPTVFNKLRFAYPGRDQQVLRNVSLSIPSEGCT
60_8244   AQMLHLAKMPRDHPDQQELGKKRLLTPLPTVFNKLRFAYPGRDQQVLRNVSLSIPSEGCT
61_7964   AQMLHLAKMPRDHPDQQELGKKRLLTPLPTVFNKLRFAYPGRDQQVLRNVSLSIPSEGCT
62_4413   AQMLHLAKMPRDHPDQQELGKKRLLTPLPTVFNKLRFAYPGRDQQVLRNVSLSIPSEGCT
63_6958   AQMLHLAKMPRDHPDQQELGKKRLLTPLPTVFNKLRFAYPGRDQQVLRNVSLSIPSEGCT
64_5997   ............................................................
65_8711   AQMLHLAKMPRDHPDQQELGKKRLLTPLPTVFNKLRFAYPGRDQQVLRNVSLSIPSEGCT
66_9925   AQMLHLAKMPRDHPDQQELGKKRLLTPLPTVFNKLRFAYPGRDQQVLRNVSLSIPSEGCT
67_10374  AQMLHLAKMPRDHPDQQELGKKRLLTPLPTVFNKLRFAYPGRDQQVLRNVSLSIPSEGCT
68_3713   AQMLHLAKMPRDHPDQQELGKKRLLTPLPTVFNKLRFAYPGRDQQVLRNVSLSIPSEGCT
69_4511   AQMLHLAKMPRDHPDQQELGKKRLLTPLPTVFNKLRFAYPGRDQQVLRNVSLSIPSEGCT
70_6201   AQMLHLAKMPRDHPDQQELGKKRLLTPLPTVFNKLRFAYPGRDQQVLRNVSLSIPSEGCT
71_3261   AQMLHLAKMPRDHPDQQELGKKRLLTPLPTVFNKLRFAYPGRDQQVLRNVSLSIPSEGCT
72_8516   AQMLHLAKMPRDHPDQQELGKKRLLTPLPTVFNKLRFAYPGRDQQVLRNVSLSIPSEGCT
73_9295   AQMLHLAKMPRDHPDQQELGKKRLLTPLPTVFNKLRFAYPGRDQQVLRNVSLSIPSEGCT
74_809    AQMLHLAKMPRDHPDQQELGKKRLLTPLPTVFNKLRFAYPGRDQQVLRNVSLSIPSEGCT
75_7282   AQMLHLAKMPRDHPDQQELGKKRLLTPLPTVFNKLRFAYPGRDQQVLRNVSLSIPSEGCT
76_4910   AQMLHLAKMPRDHPDQQELGKKRLLTPLPTVFNKLRFAYPGRDQQVLRNVSLSIPSEGCT
77_10912  AQMLHLAKMPRDHPDQQELGKKRLLTPLPTVFNKLRFAYPGRDQQVLRNVSLSIPSEGCT
78_4020   AQMLHLAKMPRDHPDQQELGKKRLLTPLPTVFNKLRFAYPGRDQQVLRNVSLSIPSEGCT
79_6703   AQMLHLAKMPRDHPDQQELGKKRLLTPLPTVFNKLRFAYPGRDQQVLRNVSLSIPSEGCT
80_8619   AQMLHLAKMPRDHPDQQELGKKRLLTPLPTVFNKLRFAYPGRDQQVLRNVSLSIPSEGCT
81_4126   HPDQQELGKKRLLTPLPTVFNKLRFAYPGRDQQVLRNVSLSIPSEGCTAIVGGSGCGKST
82_1363   AQMLHLAKMPRDHPDQQELGKKRLLTPLPTVFNKLRFAYPGRDQQVLRNVSLSIPSEGCT
83_5813   AQMLHLAKMPRDHPDQQELGKKRLLTPLPTVFNKLRFAYPGRDQQVLRNVSLSIPSEGCT
84_2044   AQMLHLAKMPRDHPDQQELGKKRLLTPLPTVFNKLRFAYPGRDQQVLRNVSLSIPSEGCT
85_7617   AQMLHLAKMPRDHPDQQELGKKRLLTPLPTVFNKLRFAYPGRDQQVLRNVSLSIPSEGCT
86_1530   AQMLHLAKMPRDHPDQQELGKKRLLTPLPTVFNKLRFAYPGRDQQVLRNVSLSIPSEGCT
87_1890   AQMLHLAKMPRDHPDQQELGKKRLLTPLPTVFNKLRFAYPGRDQQVLRNVSLSIPSEGCT
88_7049   AQMLHLAKMPRDHPDQQELGKKRLLTPLPTVFNKLRFAYPGRDQQVLRNVSLSIPSEGCT
89_440    AQMLHLAKMPRDHPDQQELGKKRLLTPLPTVFNKLRFAYPGRDQQVLRNVSLSIPSEGCT
90_4331   AQMLHLAKMPRDHPDQQELGKKRLLTPLPTVFNKLRFAYPGRDQQVLRNVSLSIPSEGCT
```

	1270 . . . 1280 . . . 1290 . . . 1300 . . . 1310 . . . 1320
1_1896	AIVGGSGCGKSTIAALILGLYEPLYDDDPSPTGGASPKASRKGNFEVISPVVSYPLGPVA
2_4785	AIVGGSGCGKSTIAALILGLYEPLYDDDPSPTGGASPKASRKGNFEVISPVVSYPLGPVA
3_3653	AIVGGSGCGKSTIAALILGLYEPLYDDDPSPTGGASPKASRKGNFEVISPVVSYPLGPVA
4_8157	AIVGGSGCGKSTIAALILGLYEPLYDDDPSPTGGASPKASRKGNFEVISPVVSYPLGPVA
5_752	AIVGGSGCGKSTIAALILGLYEPLYDDDPSPTGGASPKASRKGNFEVISPVVSYPLGPVA
6_8853	AIVGGSGCGKSTIAALILGLYEPLYDDDPSPTGGASPKASRKGNFEVISPVVSYPLGPVA
7_4015	NWEGNFFRKSEGRPFSYVHGAGASAQHRAPAVVVITHSREMMKMADRLVVIDNGCVAETG
8_2020	AIVGGSGCGKSTIAALILGLYEPLYDDDPSPTGGASPKASRKGNFEVISPVVSYPLGPVA
9_1829	AIVGGSGCGKSTIAALILGLYEPLYDDDPSPTGGASPKASRKGNFEVISPVVSYPLGPVA
10_6452	AIVGGSGCGKSTIAALILGLYEPLYDDDPSPTGGASPKASRKGNFEVISPVVSYPLGPVA
11_8627	AIVGGSGCGKSTIAALILGLYEPLYDDDPSPTGGASPKASRKGNFEVISPVVSYPLGPVA
12_6118	AIVGGSGCGKSTIAALILGLYEPLYDDDPSPTGGASPKASRKGNFEVISPVVSYPLGPVA
13_2688	AIVGGSGCGKSTIAALILGLYEPLYDDDPSPTGGASPKASRKGNFEVISPVVSYPLGPVA
14_6406	AIVGGSGCGKSTIAALILGLYEPLYDDDPSPTGGASPKASRKGNFEVISPVVSYPLGPVA
15_9419	AIVGGSGCGKSTIAALILGLYEPLYDDDPSPTGGASPKASRKGNFEVISPVVSYPLGPVA
16_8507	AIVGGSGCGKSTIAALILGLYEPLYDDDPSPTGGASPKASRKGNFEVISPVVSYPLGPVA
17_5884	AIVGGSGCGKSTIAALILGLYEPLYDDDPSPTGGASPKASRKGNFEVISPVVSYPLGPVA
18_2670	AIVGGSGCGKSTIAALILGLYEPLYDDDPSPTGGASPKASRKGNFEVISPVVSYPLGPVA
19_5172	AIVGGSGCGKSTIAALILGLYEPLYDDDPSPTGGASPKASRKGNFEVISPVVSYPLGPVA
20_8057	AIVGGSGCGKSTIAALILGLYEPLYDDDPSPTGGASPKASRKGNFEVISPVVSYPLGPVA
21_4920	....
22_6393	AIVGGSGCGKSTIAALILGLYEPLYDDDPSPTGGASPKASRKGNFEVISPVVSYPLGPVA
23_8263	AIVGGSGCGKSTIAALILGLYEPLYDDDPSPTGGASPKASRKGNFEVISPVVSYPLGPVA
24_8248	AIVGGSGCGKSTIAALILGLYEPLYDDDPSPTGGASPKASRKGNFEVISPVVSYPLGPVA
25_6733	AIVGGSGCGKSTIAALILGLYEPLYDDDPSPTGGASPKASRKGNFEVISPVVSYPLGPVA
26_2277	AIVGGSGCGKSTIAALILGLYEPLYDDDPSPTGGASPKASRKGNFEVISPVVSYPLGPVA
27_2623	AIVGGSGCGKSTIAALILGLYEPLYDDDPSPTGGASPKASRKGNFEVISPVVSYPLGPVA
28_7413	AIVGGSGCGKSTIAALILGLYEPLYDDDPSPTGGASPKASRKGNFEVISPVVSYPLGPVA
29_8980	AIVGGSGCGKSTIAALILGLYEPLYDDDPSPTGGASPKASRKGNFEVISPVVSYPLGPVA
30_8706	AIVGGSGCGKSTIAALILGLYEPLYDDDPSPTGGASPKASRKGNFEVISPVVSYPLGPVA
31_6378	AIVGGSGCGKSTIAALILGLYEPLYDDDPSPTGGASPKASRKGNFEVISPVVSYPLGPVA
32_4679	AIVGGSGCGKSTIAALILGLYEPLYDDDPSPTGGASPKASRKGNFEVISPVVSYPLGPVA
33_2437	AIVGGSGCGKSTIAALILGLYEPLYDDDPSPTGGASPKASRKGNFEVISPVVSYPLGPVA
34_9700	AIVGGSGCGKSTIAALILGLYEPLYDDDPSPTGGASPKASRKGNFEVISPVVSYPLGPVA
35_10218	AIVGGSGCGKSTIAALILGLYEPLYDDDPSPTGGASPKASRKGNFEVISPVVSYPLGPVA
36_2652	AIVGGSGCGKSTIAALILGLYEPLYDDDPSPTGGASPKASRKGNFEVISPVVSYPLGPVA
37_5779	AIVGGSGCGKSTIAALILGLYEPLYDDDPSPTGGASPKASRKGNFEVISPVVSYPLGPVA
38_654	AIVGGSGCGKSTIAALILGLYEPLYDDDPSPTGGASPKASRKGNFEVISPVVSYPLGPVA
39_4879	AIVGGSGCGKSTIAALILGLYEPLYDDDPSPTGGASPKASRKGNFEVISPVVSYPLGPVA
40_8113	AIVGGSGCGKSTIAALILGLYEPLYDDDPSPTGGASPKASRKGNFEVISPVVSYPLGPVA
41_1767	IAALILGLYEPLYDDDPSPTGGASPKASRKGNFEVISPVVSYPLGPVASSPLPSLVYSPP
42_8428	AIVGGSGCGKSTIAALILGLYEPLYDDDPSPTGGASPKASRKGNFEVISPVVSYPLGPVA
43_9819	AIVGGSGCGKSTIAALILGLYEPLYDDDPSPTGGASPKASRKGNFEVISPVVSYPLGPVA
44_5569	AIVGGSGCGKSTIAALILGLYEPLYDDDPSPTGGASPKASRKGNFEVISPVVSYPLGPVA
45_4189	AIVGGSGCGKSTIAALILGLYEPLYDDDPSPTGGASPKASRKGNFEVISPVVSYPLGPVA
46_1027	AIVGGSGCGKSTIAALILGLYEPLYDDDPSPTGGASPKASRKGNFEVISPVVSYPLGPVA
47_5048	AIVGGSGCGKSTIAALILGLYEPLYDDDPSPTGGASPKASRKGNFEVISPVVSYPLGPVA
48_10124	AIVGGSGCGKSTIAALILGLYEPLYDDDPSPTGGASPKASRKGNFEVISPVVSYPLGPVA
49_7151	AIVGGSGCGKSTIAALILGLYEPLYDDDPSPTGGASPKASRKGNFEVISPVVSYPLGPVA
50_3695	AIVGGSGCGKSTIAALILGLYEPLYDDDPSPTGGASPKASRKGNFEVISPVVSYPLGPVA
51_364	AIVGGSGCGKSTIAALILGLYEPLYDDDPSPTGGASPKASRKGNFEVISPVVSYPLGPVA
52_242	AIVGGSGCGKSTIAALILGLYEPLYDDDPSPTGGASPKASRKGNFEVISPVVSYPLGPVA
53_5225	AIVGGSGCGKSTIAALILGLYEPLYDDDPSPTGGASPKASRKGNFEVISPVVSYPLGPVA
54_1635	AIVGGSGCGKSTIAALILGLYEPLYDDDPSPTGGASPKASRKGNFEVISPVVSYPLGPVA
55_5766	AIVGGSGCGKSTIAALILGLYEPLYDDDPSPTGGASPKASRKGNFEVISPVVSYPLGPVA
56_1557	AIVGGSGCGKSTIAALILGLYEPLYDDDPSPTGGASPKASRKGNFEVISPVVSYPLGPVA
57_10137	AIVGGSGCGKSTIAALILGLYEPLYDDDPSPTGGASPKASRKGNFEVISPVVSYPLGPVA
58_10525	AIVGGSGCGKSTIAALILGLYEPLYDDDPSPTGGASPKASRKGNFEVISPVVSYPLGPVA
59_3457	AIVGGSGCGKSTIAALILGLYEPLYDDDPSPTGGASPKASRKGNFEVISPVVSYPLGPVA
60_8244	AIVGGSGCGKSTIAALILGLYEPLYDDDPSPTGGASPKASRKGNFEVISPVVSYPLGPVA
61_7964	AIVGGSGCGKSTIAALILGLYEPLYDDDPSPTGGASPKASRKGNFEVISPVVSYPLGPVA
62_4413	AIVGGSGCGKSTIAALILGLYEPLYDDDPSPTGGASPKASRKGNFEVISPVVSYPLGPVA
63_6958	AIVGGSGCGKSTIAALILGLYEPLYDDDPSPTGGASPKASRKGNFEVISPVVSYPLGPVA
64_5997	....
65_8711	AIVGGSGCGKSTIAALILGLYEPLYDDDPSPTGGASPKASRKGNFEVISPVVSYPLGPVA
66_9925	AIVGGSGCGKSTIAALILGLYEPLYDDDPSPTGGASPKASRKGNFEVISPVVSYPLGPVA
67_10374	AIVGGSGCGKSTIAALILGLYEPLYDDDPSPTGGASPKASRKGNFEVISPVVSYPLGPVA
68_3713	AIVGGSGCGKSTIAALILGLYEPLYDDDPSPTGGASPKASRKGNFEVISPVVSYPLGPVA
69_4511	AIVGGSGCGKSTIAALILGLYEPLYDDDPSPTGGASPKASRKGNFEVISPVVSYPLGPVA
70_6201	AIVGGSGCGKSTIAALILGLYEPLYDDDPSPTGGASPKASRKGNFEVISPVVSYPLGPVA
71_3261	AIVGGSGCGKSTIAALILGLYEPLYDDDPSPTGGASPKASRKGNFEVISPVVSYPLGPVA
72_8516	AIVGGSGCGKSTIAALILGLYEPLYDDDPSPTGGASPKASRKGNFEVISPVVSYPLGPVA
73_9295	AIVGGSGCGKSTIAALILGLYEPLYDDDPSPTGGASPKASRKGNFEVISPVVSYPLGPVA
74_809	AIVGGSGCGKSTIAALILGLYEPLYDDDPSPTGGASPKASRKGNFEVISPVVSYPLGPVA
75_7282	AIVGGSGCGKSTIAALILGLYEPLYDDDPSPTGGASPKASRKGNFEVISPVVSYPLGPVA
76_4910	AIVGGSGCGKSTIAALILGLYEPLYDDDPSPTGGASPKASRKGNFEVISPVVSYPLGPVA
77_10912	AIVGGSGCGKSTIAALILGLYEPLYDDDPSPTGGASPKASRKGNFEVISPVVSYPLGPVA
78_4020	AIVGGSGCGKSTIAALILGLYEPLYDDDPSPTGGASPKASRKGNFEVISPVVSYPLGPVA
79_6703	AIVGGSGCGKSTIAALILGLYEPLYDDDPSPTGGASPKASRKGNFEVISPVVSYPLGPVA
80_8619	AIVGGSGCGKSTIAALILGLYEPLYDDDPSPTGGASPKASRKGNFEVISPVVSYPLGPVA
81_4126	IAALILGLYEPLYDDDPSPTGGASPKASRKGNFEVISPVVSYPLGPVASSPLPSLVYSPP
82_1363	AIVGGSGCGKSTIAALILGLYEPLYDDDPSPTGGASPKASRKGNFEVISPVVSYPLGPVA
83_5813	AIVGGSGCGKSTIAALILGLYEPLYDDDPSPTGGASPKASRKGNFEVISPVVSYPLGPVA
84_2044	AIVGGSGCGKSTIAALILGLYEPLYDDDPSPTGGASPKASRKGNFEVISPVVSYPLGPVA
85_7617	AIVGGSGCGKSTIAALILGLYEPLYDDDPSPTGGASPKASRKGNFEVISPVVSYPLGPVA
86_1530	AIVGGSGCGKSTIAALILGLYEPLYDDDPSPTGGASPKASRKGNFEVISPVVSYPLGPVA
87_1890	AIVGGSGCGKSTIAALILGLYEPLYDDDPSPTGGASPKASRKGNFEVISPVVSYPLGPVA
88_7049	AIVGGSGCGKSTIAALILGLYEPLYDDDPSPTGGASPKASRKGNFEVISPVVSYPLGPVA
89_440	AIVGGSGCGKSTIAALILGLYEPLYDDDPSPTGGASPKASRKGNFEVISPVVSYPLGPVA
90_4331	AIVGGSGCGKSTIAALILGLYEPLYDDDPSPTGGASPKASRKGNFEVISPVVSYPLGPVA

```
                1330      1340      1350      1360      1370      1380
1_1896     SSPLPSLVYSPPQGGTRASSHRRSGELGALTYAGFDAREISTKALRGMMAYVPQQPFIFP
2_4785     SSPLPSLVYSPPQGGTRASSHRRSGELGALTYAGFDAREISTKALRGMMAYVPQQPFIFP
3_3653     SSPLPSLVYSPPQGGTRASSHRRSGELGALTYAGFDAREISTKALRGMMAYVPQQPFIFP
4_8157     SSPLPSLVYSPPQGGTRASSHRRSGELGALTYAGFDAREISTKALRGMMAYVPQQPFIFP
5_752      SSPLPSLVYSPPQGGTRASSHRRSGELGALTYAGFDAREISTKALRGMMAYVPQQPFIFP
6_8853     SSPLPSLVYSPPQGGTRASSHRRSGELGALTYAGFDAREISTKALRGMMAYVPQQPFIFP
7_4015     TYEYLMAVGDSRLAELLDGAYRSPAAKSSGVPREKTTSLEPVPVSPRTLTRVVSPSPEQY
8_2020     SSPLPSLVYSPPQGGTRASSHRRSGELGALTYAGFDAREISTKALRGMMAYVPQQPFIFP
9_1829     SSPLPSLVYSPPQGGTRASSHRRSGELGALTYAGFDAREISTKALRGMMAYVPQQPFIFP
10_6452    SSPLPSLVYSPPQGGTRASSHRRSGELGALTYAGFDAREISTKALRGMMAYVPQQPFIFP
11_8627    SSPLPSLVYSPPQGGTRASSHRRSGELGALTYAGFDAREISTKALRGMMAYVPQQPFIFP
12_6118    SSPLPSLVYSPPQGGTRASSHRRSGELGALTYAGFDAREISTKALRGMMAYVPQQPFIFP
13_2688    SSPLPSLVYSPPQGGTRASSHRRSGELGALTYAGFDAREISTKALRGMMAYVPQQPFIFP
14_6406    SSPLPSLVYSPPQGGTRASSHRRSGELGALTYAGFDAREISTKALRGMMAYVPQQPFIFP
15_9419    SSPLPSLVYSPPQGGTRASSHRRSGELGALTYAGFDAREISTKALRGMMAYVPQQPFIFP
16_8507    SSPLPSLVYSPPQGGTRASSHRRSGELGALTYAGFDAREISTKALRGMMAYVPQQPFIFP
17_5884    SSPLPSLVYSPPQGGTRASSHRRSGELGALTYAGFDAREISTKALRGMMAYVPQQPFIFP
18_2670    SSPLPSLVYSPPQGGTRASSHRRSGELGALTYAGFDAREISTKALRGMMAYVPQQPFIFP
19_5172    SSPLPSLVYSPPQGGTRASSHRRSGELGALTYAGFDAREISTKALRGMMAYVPQQPFIFP
20_8057    SSPLPSLVYSPPQGGTRASSHRRSGELGALTYAGFDAREISTKALRGMMAYVPQQPFIFP
21_4920    ............................................................
22_6393    SSPLPSLVYSPPQGGTRASSHRRSGELGALTYAGFDAREISTKALRGMMAYVPQQPFIFP
23_8263    SSPLPSLVYSPPQGGTRASSHRRSGELGALTYAGFDAREISTKALRGMMAYVPQQPFIFP
24_8248    SSPLPSLVYSPPQGGTRASSHRRSGELGALTYAGFDAREISTKALRGMMAYVPQQPFIFP
25_6733    SSPLPSLVYSPPQGGTRASSHRRSGELGALTYAGFDAREISTKALRGMMAYVPQQPFIFP
26_2277    SSPLPSLVYSPPQGGTRASSHRRSGELGALTYAGFDAREISTKALRGMMAYVPQQPFIFP
27_2623    SSPLPSLVYSPPQGGTRASSHRRSGELGALTYAGFDAREISTKALRGMMAYVPQQPFIFP
28_7413    SSPLPSLVYSPPQGGTRASSHRRSGELGALTYAGFDAREISTKALRGMMAYVPQQPFIFP
29_8980    SSPLPSLVYSPPQGGTRASSHRRSGELGALTYAGFDAREISTKALRGMMAYVPQQPFIFP
30_8706    SSPLPSLVYSPPQGGTRASSHRRSGELGALTYAGFDAREISTKALRGMMAYVPQQPFIFP
31_6378    SSPLPSLVYSPPQGGTRASSHRRSGELGALTYAGFDAREISTKALRGMMAYVPQQPFIFP
32_4679    SSPLPSLVYSPPQGGTRASSHRRSGELGALTYAGFDAREISTKALRGMMAYVPQQPFIFP
33_2437    SSPLPSLVYSPPQGGTRASSHRRSGELGALTYAGFDAREISTKALRGMMAYVPQQPFIFP
34_9700    SSPLPSLVYSPPQGGTRASSHRRSGELGALTYAGFDAREISTKALRGMMAYVPQQPFIFP
35_10218   SSPLPSLVYSPPQGGTRASSHRRSGELGALTYAGFDAREISTKALRGMMAYVPQQPFIFP
36_2652    SSPLPSLVYSPPQGGTRASSHRRSGELGALTYAGFDAREISTKALRGMMAYVPQQPFIFP
37_5779    SSPLPSLVYSPPQGGTRASSHRRSGELGALTYAGFDAREISTKALRGMMAYVPQQPFIFP
38_654     SSPLPSLVYSPPQGGTRASSHRRSGELGALTYAGFDAREISTKALRGMMAYVPQQPFIFP
39_4879    SSPLPSLVYSPPQGGTRASSHRRSGELGALTYAGFDAREISTKALRGMMAYVPQQPFIFP
40_8113    SSPLPSLVYSPPQGGTRASSHRRSGELGALTYAGFDAREISTKALRGMMAYVPQQPFIFP
41_1767    QGGTRASSHRRSGELGALTYAGFDAREISTKALRGMMAYVPQQPFIFPGTIRENIVYGLP
42_8428    SSPLPSLVYSPPQGGTRASSHRRSGELGALTYAGFDAREISTKALRGMMAYVPQQPFIFP
43_9819    SSPLPSLVYSPPQGGTRASSHRRSGELGALTYAGFDAREISTKALRGMMAYVPQQPFIFP
44_5569    SSPLPSLVYSPPQGGTRASSHRRSGELGALTYAGFDAREISTKALRGMMAYVPQQPFIFP
45_4189    SSPLPSLVYSPPQGGTRASSHRRSGELGALTYAGFDAREISTKALRGMMAYVPQQPFIFP
46_1027    SSPLPSLVYSPPQGGTRASSHRRSGELGALTYAGFDAREISTKALRGMMAYVPQQPFIFP
47_5048    SSPLPSLVYSPPQGGTRASSHRRSGELGALTYAGFDAREISTKALRGMMAYVPQQPFIFP
48_10124   SSPLPSLVYSPPQGGTRASSHRRSGELGALTYAGFDAREISTKALRGMMAYVPQQPFIFP
49_7151    SSPLPSLVYSPPQGGTRASSHRRSGELGALTYAGFDAREISTKALRGMMAYVPQQPFIFP
50_3695    SSPLPSLVYSPPQGGTRASSHRRSGELGALTYAGFDAREISTKALRGMMAYVPQQPFIFP
51_364     SSPLPSLVYSPPQGGTRASSHRRSGELGALTYAGFDAREISTKALRGMMAYVPQQPFIFP
52_242     SSPLPSLVYSPPQGGTRASSHRRSGELGALTYAGFDAREISTKALRGMMAYVPQQPFIFP
53_5225    SSPLPSLVYSPPQGGTRASSHRRSGELGALTYAGFDAREISTKALRGMMAYVPQQPFIFP
54_1635    SSPLPSLVYSPPQGGTRASSHRRSGELGALTYAGFDAREISTKALRGMMAYVPQQPFIFP
55_5766    SSPLPSLVYSPPQGGTRASSHRRSGELGALTYAGFDAREISTKALRGMMAYVPQQPFIFP
56_1557    SSPLPSLVYSPPQGGTRASSHRRSGELGALTYAGFDAREISTKALRGMMAYVPQQPFIFP
57_10137   SSPLPSLVYSPPQGGTRASSHRRSGELGALTYAGFDAREISTKALRGMMAYVPQQPFIFP
58_10525   SSPLPSLVYSPPQGGTRASSHRRSGELGALTYAGFDAREISTKALRGMMAYVPQQPFIFP
59_3457    SSPLPSLVYSPPQGGTRASSHRRSGELGALTYAGFDAREISTKALRGMMAYVPQQPFIFP
60_8244    SSPLPSLVYSPPQGGTRASSHRRSGELGALTYAGFDAREISTKALRGMMAYVPQQPFIFP
61_7964    SSPLPSLVYSPPQGGTRASSHRRSGELGALTYAGFDAREISTKALRGMMAYVPQQPFIFP
62_4413    SSPLPSLVYSPPQGGTRASSHRRSGELGALTYAGFDAREISTKALRGMMAYVPQQPFIFP
63_6958    SSPLPSLVYSPPQGGTRASSHRRSGELGALTYAGFDAREISTKALRGMMAYVPQQPFIFP
64_5997    ............................................................
65_8711    SSPLPSLVYSPPQGGTRASSHRRSGELGALTYAGFDAREISTKALRGMMAYVPQQPFIFP
66_9925    SSPLPSLVYSPPQGGTRASSHRRSGELGALTYAGFDAREISTKALRGMMAYVPQQPFIFP
67_10374   SSPLPSLVYSPPQGGTRASSHRRSGELGALTYAGFDAREISTKALRGMMAYVPQQPFIFP
68_3713    SSPLPSLVYSPPQGGTRASSHRRSGELGALTYAGFDAREISTKALRGMMAYVPQQPFIFP
69_4511    SSPLPSLVYSPPQGGTRASSHRRSGELGALTYAGFDAREISTKALRGMMAYVPQQPFIFP
70_6201    SSPLPSLVYSPPQGGTRASSHRRSGELGALTYAGFDAREISTKALRGMMAYVPQQPFIFP
71_3261    SSPLPSLVYSPPQGGTRASSHRRSGELGALTYAGFDAREISTKALRGMMAYVPQQPFIFP
72_8516    SSPLPSLVYSPPQGGTRASSHRRSGELGALTYAGFDAREISTKALRGMMAYVPQQPFIFP
73_9295    SSPLPSLVYSPPQGGTRASSHRRSGELGALTYAGFDAREISTKALRGMMAYVPQQPFIFP
74_809     SSPLPSLVYSPPQGGTRASSHRRSGELGALTYAGFDAREISTKALRGMMAYVPQQPFIFP
75_7282    SSPLPSLVYSPPQGGTRASSHRRSGELGALTYAGFDAREISTKALRGMMAYVPQQPFIFP
76_4910    SSPLPSLVYSPPQGGTRASSHRRSGELGALTYAGFDAREISTKALRGMMAYVPQQPFIFP
77_10912   SSPLPSLVYSPPQGGTRASSHRRSGELGALTYAGFDAREISTKALRGMMAYVPQQPFIFP
78_4020    SSPLPSLVYSPPQGGTRASSHRRSGELGALTYAGFDAREISTKALRGMMAYVPQQPFIFP
79_6703    SSPLPSLVYSPPQGGTRASSHRRSGELGALTYAGFDAREISTKALRGMMAYVPQQPFIFP
80_8619    SSPLPSLVYSPPQGGTRASSHRRSGELGALTYAGFDAREISTKALRGMMAYVPQQPFIFP
81_4126    QGGTRASSHRRSGELGALTYAGFDAREISTKALRGMMAYVPQQPFIFPGTIRENIVYGLP
82_1363    SSPLPSLVYSPPQGGTRASSHRRSGELGALTYAGFDAREISTKALRGMMAYVPQQPFIFP
83_5813    SSPLPSLVYSPPQGGTRASSHRRSGELGALTYAGFDAREISTKALRGMMAYVPQQPFIFP
84_2044    SSPLPSLVYSPPQGGTRASSHRRSGELGALTYAGFDAREISTKALRGMMAYVPQQPFIFP
85_7617    SSPLPSLVYSPPQGGTRASSHRRSGELGALTYAGFDAREISTKALRGMMAYVPQQPFIFP
86_1530    SSPLPSLVYSPPQGGTRASSHRRSGELGALTYAGFDAREISTKALRGMMAYVPQQPFIFP
87_1890    SSPLPSLVYSPPQGGTRASSHRRSGELGALTYAGFDAREISTKALRGMMAYVPQQPFIFP
88_7049    SSPLPSLVYSPPQGGTRASSHRRSGELGALTYAGFDAREISTKALRGMMAYVPQQPFIFP
89_440     SSPLPSLVYSPPQGGTRASSHRRSGELGALTYAGFDAREISTKALRGMMAYVPQQPFIFP
90_4331    SSPLPSLVYSPPQGGTRASSHRRSGELGALTYAGFDAREISTKALRGMMAYVPQQPFIFP
```

```
                 1390      1400      1410      1420      1430      1440
1_1896    GTIRENIVYGLPEDESVLRGQHNVELAAREADIHELIISLPQGYETLVGDGGVALSGGQA
2_4785    GTIRENIVYGLPEDESVLRGQHNVELAAREADIHEFIISLPQGYETLVGDGGVALSGGQA
3_3653    GTIRENIVYGLPEDESVLRGQHNVELAAREADIHEFIISLPQGYETLVGDGGVALSGGQA
4_8157    GTIRENIVYGLPEDESVLRGQHNVELAAREADIHEFIISLPQGYETLVGDGGVALSGGQA
5_752     GTIRENIVYGLPEDESVLRGQHNVELAAREADIHEFIISLPQGYETLVGDGGVALSGGQA
6_8853    GTIRENIVYGLPEDESVLRGQHNVELAAREADIHEFIISLPQGYETLVGDGGVALSGGQA
7_4015    RRNNHSSPGGIPQRQQRPGRNIEDMFLPQVDWQGDRR.......................
8_2020    GTIRENIVYGLPEDESVLRGQHNVELAAREADIHEFIISLPQGYETLVGDGGVALSGGQA
9_1829    GTIRENIVYGLPEDESVLRGQHNVELAAREADIHEFIISLPQGYETLVGDGGVALSGGQA
10_6452   GTIRENIVYGLPEDESVLRGQHNVELAAREADIHEFIISLPQGYETLVGDGGVALSGGQA
11_8627   GTIRENIVYGLPEDESVLRGQHNVELAAREADIHELIISLPQGYETLVGDGGVALSGGQA
12_6118   GTIRENIVYGLPEDESVLRGQHNVELAAREADIHEFIISLPQGYETLVGDGGVALSGGQA
13_2688   GTIRENIVYGLPEDESVLRGQHNVELAAREADIHELIISLPQGYETLVGDGGVALSGGQA
14_6406   GTIRENIVYGLPEDESVLRGQHNVELAAREADIHEFIISLPQGYETLVGDGGVALSGGQA
15_9419   GTIRENIVYGLPEDESVLRGQHNVELAAREADIHELIISLPQGYETLVGDGGVALSGGQA
16_8507   GTIRENIVYGLPEDESVLRGQHNVELAAREADIHELIISLPQGYETLVGDGGVALSGGQA
17_5884   GTIRENIVYGLPEDESVLRGQHNVELAAREADIHEFIISLPQGYETLVGDGGVALSGGQA
18_2670   GTIRENIVYGLPEDESVLRGQHNVELAAREADIHEFIISLPQGYETLVGDGGVALSGGQA
19_5172   GTIRENIVYGLPEDESVLRGQHNVELAAREADIHEFIISLPQGYETLVGDGGVALSGGQA
20_8057   GTIRENIVYGLPEDESVLRGQHNVELAAREADIHEFIISLPQGYETLVGDGGVALSGGQA
21_4920   ............................................................
22_6393   GTIRENIVYGLPEDESVLRGQHNVELAAREADIHEFIISLPQGYETLVGDGGVALSGGQA
23_8263   GTIRENIVYGLPEDESVLRGQHNVELAAREADIHELIISLPQGYETLVGDGGVALSGGQA
24_8248   GTIRENIVYGLPEDESVLRGQHNVELAAREADIHEFIISLPQGYETLVGDGGVALSGGQA
25_6733   GTIRENIVYGLPEDESVLRGQHNVELAAREADIHELIISLPQGYETLVGDGGVALSGGQA
26_2277   GTIRENIVYGLPEDESVLRGQHNVELAAREADIHELIISLPQGYETLVGDGGVALSGGQA
27_2623   GTIRENIVYGLPEDESVLRGQHNVELAAREADIHEFIISLPQGYETLVGDGGVALSGGQA
28_7413   GTIRENIVYGLPEDESVLRGQHNVELAAREADIHEFIISLPQGYETLVGDGGVALSGGQA
29_8980   GTIRENIVYGLPEDESVLRGQHNVELAAREADIHEFIISLPQGYETLVGDGGVALSGGQA
30_8706   GTIRENIVYGLPEDESVLRGQHNVELAAREADIHEFIISLPQGYETLVGDGGVALSGGQA
31_6378   GTIRENIVYGLPEDESVLRGQHNVELAAREADIHEFIISLPQGYETLVGDGGVALSGGQA
32_4679   GTIRENIVYGLPEDESVLRGQHNVELAAREADIHEFIISLPQGYETLVGDGGVALSGGQA
33_2437   GTIRENIVYGLPEDESVLRGQHNVELAAREADIHEFIISLPQGYETLVGDGGVALSGGQA
34_9700   GTIRENIVYGLPEDESVLRGQHNVELAAREADIHEFIISLPQGYETLVGDGGVALSGGQA
35_10218  GTIRENIVYGLPEDESVLRGQHNVELAAREADIHEFIISLPQGYETLVGDGGVALSGGQA
36_2652   GTIRENIVYGLPEDESVLRGQHNVELAAREADIHEFIISLPQGYETLVGDGGVALSGGQA
37_5779   GTIRENIVYGLPEDESVLRGQHNVELAAREADIHEFIISLPQGYETLVGDGGVALSGGQA
38_654    GTIRENIVYGLPEDESVLRGQHNVELAAREADIHEFIISLPQGYETLVGDGGVALSGGQA
39_4879   GTIRENIVYGLPEDESVLRGQHNVELAAREADIHEFIISLPQGYETLVGDGGVALSGGQA
40_8113   GTIRENIVYGLPEDESVLRGQHNVELAAREADIHEFIISLPQGYETLVGDGGVALSGGQA
41_1767   EDESVLRGQHNVELAAREADIHEFIISLPQGYETLVGDGGVALSGGQAQRLCIARALARR
42_8428   GTIRENIVYGLPEDESVLRGQHNVELAAREADIHEFIISLPQGYETLVGDGGVALSGGQA
43_9819   GTIRENIVYGLPEDESVLRGQHNVELAAREADIHELIISLPQGYETLVGDGGVALSGGQA
44_5569   GTIRENIVYGLPEDESVLRGQHNVELAAREADIHEFIISLPQGYETLVGDGGVALSGGQA
45_4189   GTIRENIVYGLPEDESVLRGQHNVELAAREADIHELIISLPQGYETLVGDGGVALSGGQA
46_1027   GTIRENIVYGLPEDESVLRGQHNVELAAREADIHEFIISLPQGYETLVGDGGVALSGGQA
47_5048   GTIRENIVYGLPEDESVLRGQHNVELAAREADIHEFIISLPQGYETLVGDGGVALSGGQA
48_10124  GTIRENIVYGLPEDESVLRGQHNVELAAREADIHEFIISLPQGYETLVGDGGVALSGGQA
49_7151   GTIRENIVYGLPEDESVLRGQHNVELAAREADIHEFIISLPQGYETLVGDGGVALSGGQA
50_3695   GTIRENIVYGLPEDESVLRGQHNVELAAREADIHEFIISLPQGYETLVGDGGVALSGGQA
51_364    GTIRENIVYGLPEDESVLRGQHNVELAAREADIHEFIISLPQGYETLVGDGGVALSGGQA
52_242    GTIRENIVYGLPEDESVLRGQHNVELAAREADIHEFIISLPQGYETLVGDGGVALSGGQA
53_5225   GTIRENIVYGLPEDESVLRGQHNVELAAREADIHEFIISLPQGYETLVGDGGVALSGGQA
54_1635   GTIRENIVYGLPEDESVLRGQHNVELAAREADIHEFIISLPQGYETLVGDGGVALSGGQA
55_5766   GTIRENIVYGLPEDESVLRGQHNVELAAREADIHEFIISLPQGYETLVGDGGVALSGGQA
56_1557   GTIRENIVYGLPEDESVLRGQHNVELAAREADIHEFIISLPQGYETLVGDGGVALSGGQA
57_10137  GTIRENIVYGLPEDESVLRGQHNVELAAREADIHELIISLPQGYETLVGDGGVALSGGQA
58_10525  GTIRENIVYGLPEDESVLRGQHNVELAAREADIHELIISLPQGYETLVGDGGVALSGGQA
59_3457   GTIRENIVYGLPEDESVLRGQHNVELAAREADIHEFIISLPQGYETLVGDGGVALSGGQA
60_8244   GTIRENIVYGLPEDESVLRGQHNVELAAREADIHEFIISLPQGYETLVGDGGVALSGGQA
61_7964   GTIRENIVYGLPEDESVLRGQHNVELAAREADIHEFIISLPQGYETLVGDGGVALSGGQA
62_4413   GTIRENIVYGLPEDESVLRGQHNVELAAREADIHEFIISLPQGYETLVGDGGVALSGGQA
63_6958   GTIRENIVYGLPEDESVLRGQHNVELAAREADIHEFIISLPQGYETLVGDGGVALSGGQA
64_5997   ............................................................
65_8711   GTIRENIVYGLPEDESVLRGQHNVELAAREADIHEFIISLPQGYETLVGDGGVALSGGQA
66_9925   GTIRENIVYGLPEDESVLRGQHNVELAAREADIHEFIISLPQGYETLVGDGGVALSGGQA
67_10374  GTIRENIVYGLPEDESVLRGQHNVELAAREADIHEFIISLPQGYETLVGDGGVALSGGQA
68_3713   GTIRENIVYGLPEDESVLRGQHNVELAAREADIHEFIISLPQGYETLVGDGGVALSGGQA
69_4511   GTIRENIVYGLPEDESVLRGQHNVELAAREADIHEFIISLPQGYETLVGDGGVALSGGQA
70_6201   GTIRENIVYGLPEDESVLRGQHNVELAAREADIHEFIISLPQGYETLVGDGGVALSGGQA
71_3261   GTIRENIVYGLPEDESVLRGQHNVELAAREADIHEFIISLPQGYETLVGDGGVALSGGQA
72_8516   GTIRENIVYGLPEDESVLRGQHNVELAAREADIHEFIISLPQGYETLVGDGGVALSGGQA
73_9295   GTIRENIVYGLPEDESVLRGQHNVELAAREADIHEFIISLPQGYETLVGDGGVALSGGQA
74_809    GTIRENIVYGLPEDESVLRGQHNVELAAREADIHEFIISLPQGYETLVGDGGVALSGGQA
75_7282   GTIRENIVYGLPEDESVLRGQHNVELAAREADIHEFIISLPQGYETLVGDGGVALSGGQA
76_4910   GTIRENIVYGLPEDESVLRGQHNVELAAREADIHEFIISLPQGYETLVGDGGVALSGGQA
77_10912  GTIRENIVYGLPEDESVLRGQHNVELAAREADIHEFIISLPQGYETLVGDGGVALSGGQA
78_4020   GTIRENIVYGLPEDESVLRGQHNVELAAREADIHEFIISLPQGYETLVGDGGVALSGGQA
79_6703   GTIRENIVYGLPEDESVLRGQHNVELAAREADIHEFIISLPQGYETLVGDGGVALSGGQA
80_8619   GTIRENIVYGLPEDESVLRGQHNVELAAREADIHEFIISLPQGYETLVGDGGVALSGGQA
81_4126   EDESVLRGQHNVELAAREADIHEFIISLPQGYETLVGDGGVALSGGQAQRLCIARALARR
82_1363   GTIRENIVYGLPEDESVLRGQHNVELAAREADIHEFIISLPQGYETLVGDGGVALSGGQA
83_5813   GTIRENIVYGLPEDESVLRGQHNVELAAREADIHEFIISLPQGYETLVGDGGVALSGGQA
84_2044   GTIRENIVYGLPEDESVLRGQHNVELAAREADIHEFIISLPQGYETLVGDGGVALSGGQA
85_7617   GTIRENIVYGLPEDESVLRGQHNVELAAREADIHELIISLPQGYETLVGDGGVALSGGQA
86_1530   GTIRENIVYGLPEDESVLRGQHNVELAAREADIHEFIISLPQGYETLVGDGGVALSGGQA
87_1890   GTIRENIVYGLPEDESVLRGQHNVELAAREADIHEFIISLPQGYETLVGDGGVALSGGQA
88_7049   GTIRENIVYGLPEDESVLRGQHNVELAAREADIHEFIISLPQGYETLVGDGGVALSGGQA
89_440    GTIRENIVYGLPEDESVLRGQHNVELAAREADIHEFIISLPQGYETLVGDGGVALSGGQA
90_4331   GTIRENIVYGLPEDESVLRGQHNVELAAREADIHEFIISLPQGYETLVGDGGVALSGGQA
```

```
           1450      1460      1470      1480      1490      1500
1_1896     QRLCIARALARRPKLLVLDEPTSALDAESGQSVMDTLRTLVCRRDGDLSTAAASPLLRKA
2_4785     QRLCIARALARRPKLLVLDEPTSALDAESGQSVMDTLRTLVCRRDGDLSTAAASPLLRKA
3_3653     QRLCIARALARRPKLLVLDEPTSALDAESGQSVMDTLRTLVCRRDGDLSTAAASPLLRKA
4_8157     QRLCIARALARRPKLLVLDEPTSALDAESGQSVMDTLRTLVCRRDGDLSTAAASPLLRKA
5_752      QRLCIARALARRPKLLVLDEPTSALDAESGQSVMDTLRTLVCRRDGDLSTAAASPLLRKA
6_8853     QRLCIARALARRPKLLVLDEPTSALDAESGQSVMDTLRTLVCRRDGDLSTAAASPLLRKA
7_4015     ............................................................
8_2020     QRLCIARALARRPKLLVLDEPTSALDAESGQSVMDTLRTLVCRRDGDLSTAAASPLLRKA
9_1829     QRLCIARALARRPKLLVLDEPTSALDAESGQSVMDTLRTLVCRRDGDLSTAAASPLLRKA
10_6452    QRLCIARALARRPKLLVLDEPTSALDAESGQSVMDTLRTLVCRRDGDLSTAAASPLLRKA
11_8627    QRLCIARALARRPKLLVLDEPTSALDAESGQSVMDTLRTLVCRRDGDLSTAAASPLLRKA
12_6118    QRLCIARALARRPKLLVLDEPTSALDAESGQSVMDTLRTLVCRRDGDLSTAAASPLLRKA
13_2688    QRLCIARALARRPKLLVLDEPTSALDAESGQSVMDTLRTLVCRRDGDLSTAAASPLLRKA
14_6406    QRLCIARALARRPKLLVLDEPTSALDAESGQSVMDTLRTLVCRRDGDLSTAAASPLLRKA
15_9419    QRLCIARALARRPKLLVLDEPTSALDAESGQSVMDTLRTLVCRRDGDLSTAAASPLLRKA
16_8507    QRLCIARALARRPKLLVLDEPTSALDAESGQSVMDTLRTLVCRRDGDLSTAAASPLLRKA
17_5884    QRLCIARALARRPKLLVLDEPTSALDAESGQSVMDTLRTLVCRRDGDLSTAAASPLLRKA
18_2670    QRLCIARALARRPKLLVLDEPTSALDAESGQSVMDTLRTLVCRRDGDLSTAAASPLLRKA
19_5172    QRLCIARALARRPKLLVLDEPTSALDAESGQSVMDTLRTLVCRRDGDLSTAAASPLLRKA
20_8057    QRLCIARALARRPKLLVLDEPTSALDAESGQSVMDTLRTLVCRRDGDLSTAAASPLLRKA
21_4920    ............................................................
22_6393    QRLCIARALARRPKLLVLDEPTSALDAESGQSVMDTLRTLVCRRDGDLSTAAASPLLRKA
23_8263    QRLCIARALARRPKLLVLDEPTSALDAESGQSVMDTLRTLVCRRDGDLSTAAASPLLRKA
24_8248    QRLCIARALARRPKLLVLDEPTSALDAESGQSVMDTLRTLVCRRDGDLSTAAASPLLRKA
25_6733    QRLCIARALARRPKLLVLDEPTSALDAESGQSVMDTLRTLVCRRDGDLSTAAASPLLRKA
26_2277    QRLCIARALARRPKLLVLDEPTSALDAESGQSVMDTLRTLVCRRDGDLSTAAASPLLRKA
27_2623    QRLCIARALARRPKLLVLDEPTSALDAESGQSVMDTLRTLVCRRDGDLSTAAASPLLRKA
28_7413    QRLCIARALARRPKLLVLDEPTSALDAESGQSVMDTLRTLVCRRDGDLSTAAASPLLRKA
29_8980    QRLCIARALARRPKLLVLDEPTSALDAESGQSVMDTLRTLVCRRDGDLSTAAASPLLRKA
30_8706    QRLCIARALARRPKLLVLDEPTSALDAESGQSVMDTLRTLVCRRDGDLSTAAASPLLRKA
31_6378    QRLCIARALARRPKLLVLDEPTSALDAESGQSVMDTLRTLVCRRDGDLSTAAASPLLRKA
32_4679    QRLCIARALARRPKLLVLDEPTSALDAESGQSVMDTLRTLVCRRDGDLSTAAASPLLRKA
33_2437    QRLCIARALARRPKLLVLDEPTSALDAESGQSVMDTLRTLVCRRDGDLSTAAASPLLRKA
34_9700    QRLCIARALARRPKLLVLDEPTSALDAESGQSVMDTLRTLVCRRDGDLSTAAASPLLRKA
35_10218   QRLCIARALARRPKLLVLDEPTSALDAESGQSVMDTLRTLVCRRDGDLSTAAASPLLRKA
36_2652    QRLCIARALARRPKLLVLDEPTSALDAESGQSVMDTLRTLVCRRDGDLSTAAASPLLRKA
37_5779    QRLCIARALARRPKLLVLDEPTSALDAESGQSVMDTLRTLVCRRDGDLSTAAASPLLRKA
38_654     QRLCIARALARRPKLLVLDEPTSALDAESGQSVMDTLRTLVCRRDGDLSTAAASPLLRKA
39_4879    QRLCIARALARRPKLLVLDEPTSALDAESGQSVMDTLRTLVCRRDGDLSTAAASPLLRKA
40_8113    QRLCIARALARRPKLLVLDEPTSALDAESGQSVMDTLRTLVCRRDGDLSTAAASPLLRKA
41_1767    PKLLVLDEPTSALDAESGQSVMDTLRTLVCRRDGDLXPGRRSQHRRRLPLAKKSGWYPQP
42_8428    QRLCIARALARRPKLLVLDEPTSALDAESGQSVMDTLRTLVCRRDGDLSTAAASPLLRKA
43_9819    QRLCIARALARRPKLLVLDEPTSALDAESGQSVMDTLRTLVCRRDGDLSTAAASPLLRKA
44_5569    QRLCIARALARRPKLLVLDEPTSALDAESGQSVMDTLRTLVCRRDGDLSTAAASPLLRKA
45_4189    QRLCIARALARRPKLLVLDEPTSALDAESGQSVMDTLRTLVCRRDGDLSTAAASPLLRKA
46_1027    QRLCIARALARRPKLLVLDEPTSALDAESGQSVMDTLRTLVCRRDGDLSTAAASPLLRKA
47_5048    QRLCIARALARRPKLLVLDEPTSALDAESGQSVMDTLRTLVCRRDGDLSTAAASPLLRKA
48_10124   QRLCIARALARRPKLLVLDEPTSALDAESGQSVMDTLRTLVCRRDGDLSTAAASPLLRKA
49_7151    QRLCIARALARRPKLLVLDEPTSALDAESGQSVMDTLRTLVCRRDGDLSTAAASPLLRKA
50_3695    QRLCIARALARRPKLLVLDEPTSALDAESGQSVMDTLRTLVCRRDGDLSTAAASPLLRKA
51_364     QRLCIARALARRPKLLVLDEPTSAXGHSEDPAGGTLSPYSPQHPDPYNWEGNFFRKSEGR
52_242     QRLCIARALARRPKLLVLDEPTSALDAESGQSVMDTLRTLVCRRDGDLSTAAASPLLRKA
53_5225    QRLCIARALARRPKLLVLDEPTSALDAESGQSVMDTLRTLVCRRDGDLSTAAASPLLRKA
54_1635    QRLCIARALARRPKLLVLDEPTSALDAESGQSVMDTLRTLVCRRDGDLSTAAASPLLRKA
55_5766    QRLCIARALARRPKLLVLDEPTSALDAESGQSVMDTLRTLVCRRDGDLSTAAASPLLRKA
56_1557    QRLCIARALARRPKLLVLDEPTSALDAESGQSVMDTLRTLVCRRDGDLSTAAASPLLRKA
57_10137   QRLCIARALARRPKLLVLDEPTSALDAESGQSVMDTLRTLVCRRDGDLSTAAASPLLRKA
58_10525   QRLCIARALARRPKLLVLDEPTSALDAESGQSVMDTLRTLVCRRDGDLSTAAASPLLRKA
59_3457    QRLCIARALARRPKLLVLDEPTSALDAESGQSVMDTLRTLVCRRDGDLSTAAASPLLRKA
60_8244    QRLCIARALARRPKLLVLDEPTSALDAESGQSVMDTLRTLVCRRDGDLSTAAASPLLRKA
61_7964    QRLCIARALARRPKLLVLDEPTSALDAESGQSVMDTLRTLVCRRDGDLSTAAASPLLRKA
62_4413    QRLCIARALARRPKLLVLDEPTSALDAESGQSVMDTLRTLVCRRDGDLSTAAASPLLRKA
63_6958    QRLCIARALARRPKLLVLDEPTSALDAESGQSVMDTLRTLVCRRDGDLSTAAASPLLRKA
64_5997    ............................................................
65_8711    QRLCIARALARRPKLLVLDEPTSALDAESGQSVMDTLRTLVCRRDGDLSTAAASPLLRKA
66_9925    QRLCIARALARRPKLLVLDEPTSALDAESGQSVMDTLRTLVCRRDGDLSTAAASPLLRKA
67_10374   QRLCIARALARRPKLLVLDEPTSALDAESGQSVMDTLRTLVCRRDGDLSTAAASPLLRKA
68_3713    QRLCIARALARRPKLLVLDEPTSALDAESGQSVMDTLRTLVCRRDGDLSTAAASPLLRKA
69_4511    QRLCIARALARRPKLLVLDEPTSALDAESGQSVMDTLRTLVCRRDGDLSTAAASPLLRKA
70_6201    QRLCIARALARRPKLLVLDEPTSALDAESGQSVMDTLRTLVCRRDGDLSTAAASPLLRKA
71_3261    QRLCIARALARRPKLLVLDEPTSALDAESGQSVMDTLRTLVCRRDGDLSTAAASPLLRKA
72_8516    QRLCIARALARRPKLLVLDEPTSALDAESGQSVMDTLRTLVCRRDGDLSTAAASPLLRKA
73_9295    QRLCIARALARRPKLLVLDEPTSALDAESGQSVMDTLRTLVCRRDGDLSTAAASPLLRKA
74_809     QRLCIARALARRPKLLVLDEPTSALDAESGQSVMDTLRTLVCRRDGDLSTAAASPLLRKA
75_7282    QRLCIARALARRPKLLVLDEPTSALDAESGQSVMDTLRTLVCRRDGDLSTAAASPLLRKA
76_4910    QRLCIARALARRPKLLVLDEPTSALDAESGQSVMDTLRTLVCRRDGDLSTAAASPLLRKA
77_10912   QRLCIARALARRPKLLVLDEPTSALDAESGQSVMDTLRTLVCRRDGDLSTAAASPLLRKA
78_4020    QRLCIARALARRPKLLVLDEPTSALDAESGQSVMDTLRTLVCRRDGDLSTAAASPLLRKA
79_6703    QRLCIARALARRPKLLVLDEPTSALDAESGQSVMDTLRTLVCRRDGDLSTAAASPLLRKA
80_8619    QRLCIARALARRPKLLVLDEPTSALDAESGQSVMDTLRTLVCRRDGDLSTAAASPLLRKA
81_4126    PKLLVLDEPTSALDAESGQSVMDTLRTLVCRRDGDLSTAAASPLLRKAGGTLSPYSPQHP
82_1363    QRLCIARALARRPKLLVLDEPTSALDAESGQSVMDTLRTLVCRRDGDLSTAAASPLLRKA
83_5813    QRLCIARALARRPKLLVLDEPTSALDAESGQSVMDTLRTLVCRRDGDLSTAAASPLLRKA
84_2044    QRLCIARALARRPKLLVLDEPTSALDAESGQSVMDTLRTLVCRRDGDLSTAAASPLLRKA
85_7617    QRLCIARALARRPKLLVLDEPTSALDAESGQSVMDTLRTLVCRRDGDLSTAAASPLLRKA
86_1530    QRLCIARALARRPKLLVLDEPTSALDAESGQSVMDTLRTLVCRRDGDLSTAAASPLLRKA
87_1890    QRLCIARALARRPKLLVLDEPTSALDAESGQSVMDTLRTLVCRRDGDLSTAAASPLLRKA
88_7049    QRLCIARALARRPKLLVLDEPTSALDAESGQSVMDTLRTLVCRRDGDLSTAAASPLLRKA
89_440     QRLCIARALARRPKLLVLDEPTSALDAESGQSVMDTLRTLVCRRDGDLSTAAASPLLRKA
90_4331    QRLCIARALARRPKLLVLDEPTSALDAESGQSVMDTLRTLVCRRDGDLSTAAASPLLRKA
```

```
                1510      1520      1530      1540      1550      1560
1_1896    GGTLSPYSPQHPDPYNWEGNFFRKSEGRSFSYVHGAAAGTQHRQPAVVVITHSREMMKMA
2_4785    GGTLSPYSPQHPDPYNWEGNFFRKSEGRSFSYVHGAAAGTQHRQPAVVVITHSREMMKMA
3_3653    GGTLSPYSPQHPDPYNWEGNFFRKSEGRSFSYVHGAAAGTQHRQPAVVVITHSREMMKMA
4_8157    GGTLSPYSPQHPDPYNWEGNFFRKSEGRSFSYVHGAAAGTQHRQPAVVVITHSREMMKMA
5_752     GGTLSPYSPQHPDPYNWEGNFFRKSEGRSFSYVHGAAAGTQHRQPAVVVITHSREMMKMA
6_8853    GGTLSPYSPQHPDPYNWEGNFFRKSEGRSFSYVHGAAAGTQHRQPAVVVITHSREMMKMA
7_4015    ............................................................
8_2020    GGTLSPYSPQHPDPYNWEGNFFRKSEGRSFSYVHGAAAGTQHRQPAVVVITHSREMMKMA
9_1829    GGTLSPYSPQHPDPYNWEGNFFRKSEGRSFSYVHGAAAGTQHRQPAVVVITHSREMMKMA
10_6452   GGTLSPYSPQHPDPYNWEGNFFRKSEGRSFSYVHGAAAGTQHRQPAVVVITHSREMMKMA
11_8627   GGTLSPYSPQHPDPYNWEGNFFRKSEGRSFSYVHGAAAGTQHRQPAVVVITHSREMMKMA
12_6118   GGTLSPYSPQHPDPYNWEGNFFRKSEGRSFSYVHGAAAGTQHRQPAVVVITHSREMMKMA
13_2688   GGTLSPYSPQHPDPYNWEGNFFRKSEGRSFSYVHGAAAGTQHRQPAVVVITHSREMMKMA
14_6406   GGTLSPYSPQHPDPYNWEGNFFRKSEGRSFSYVHGAAAGTQHRQPAVVVITHSREMMKMA
15_9419   GGTLSPYSPQHPDPYNWEGNFFRKSEGRSFSYVHGAAAGTQHRQPAVVVITHSREMMKMA
16_8507   GGTLSPYSPQHPDPYNWEGNFFRKSEGRSFSYVHGAAAGTQHRQPAVVVITHSREMMKMA
17_5884   GGTLSPYSPQHPDPYNWEGNFFRKSEGRSFSYVHGAAAGTQHRQPAVVVITHSREMMKMA
18_2670   GGTLSPYSPQHPDPYNWEGNFFRKSEGRSFSYVHGAAAGTQHRQPAVVVITHSREMMKMA
19_5172   GGTLSPYSPQHPDPYNWEGNFFRKSEGRSFSYVHGAAAGTQHRQPAVVVITHSREMMKMA
20_8057   GGTLSPYSPQHPDPYNWEGNFFRKSEGRSFSYVHGAAAGTQHRQPAVVVITHSREMMKMA
21_4920   ............................................................
22_6393   GGTLSPYSPQHPDPYNWEGNFFRKSEGRSFSYVHGAAAGTQHRQPAVVVITHSREMMKMA
23_8263   GGTLSPYSPQHPDPYNWEGNFFRKSEGRSFSYVHGAAAGTQHRQPAVVVITHSREMMKMA
24_8248   GGTLSPYSPQHPDPYNWEGNFFRKSEGRSFSYVHGAAAGTQHRQPAVVVITHSREMMKMA
25_6733   GGTLSPYSPQHPDPYNWEGNFFRKSEGRSFSYVHGAAAGTQHRQPAVVVITHSREMMKMA
26_2277   GGTLSPYSPQHPDPYNWEGNFFRKSEGRSFSYVHGAAAGTQHRQPAVVVITHSREMMKMA
27_2623   GGTLSPYSPQHPDPYNWEGNFFRKSEGRSFSYVHGAAAGTQHRQPAVVVITHSREMMKMA
28_7413   GGTLSPYSPQHPDPYNWEGNFFRKSEGRSFSYVHGAAAGTQHRQPAVVVITHSREMMKMA
29_8980   GGTLSPYSPQHPDPYNWEGNFFRKSEGRSFSYVHGAAAGTQHRQPAVVVITHSREMMKMA
30_8706   GGTLSPYSPQHPDPYNWEGNFFRKSEGRSFSYVHGAAAGTQHRQPAVVVITHSREMMKMA
31_6378   GGTLSPYSPQHPDPYNWEGNFFRKSEGRSFSYVHGAAAGTQHRQPAVVVITHSREMMKMA
32_4679   GGTLSPYSPQHPDPYNWEGNFFRKSEGRSFSYVHGAAAGTQHRQPAVVVITHSREMMKMA
33_2437   GGTLSPYSPQHPDPYNWEGNFFRKSEGRSFSYVHGAAAGTQHRQPAVVVITHSREMMKMA
34_9700   GGTLSPYSPQHPDPYNWEGNFFRKSEGRSFSYVHGAAAGTQHRQPAVVVITHSREMMKMA
35_10218  GGTLSPYSPQHPDPYNWEGNFFRKSEGRSFSYVHGAAAGTQHRQPAVVVITHSREMMKMA
36_2652   GGTLSPYSPQHPDPYNWEGNFFRKSEGRSFSYVHGAAAGTQHRQPAVVVITHSREMMKMA
37_5779   GGTLSPYSPQHPDPYNWEGNFFRKSEGRSFSYVHGAAAGTQHRQPAVVVITHSREMMKMA
38_654    GGTLSPYSPQHPDPYNWEGNFFRKSEGRSFSYVHGAAAGTQHRQPAVVVITHSREMMKMA
39_4879   GGTLSPYSPQHPDPYNWEGNFFRKSEGRSFSYVHGAAAGTQHRQPAVVVITHSREMMKMA
40_8113   GGTLSPYSPQHPDPYNWEGNFFRKSEGRSFSYVHGAAAGTQHRQPAVVVITHSREMMKMA
41_1767   LQPAAPRPVQLGGQLLPQERGQVLQLRPRSCRRHTAPAAGRRGHHAFARDDEDGGPPRRH
42_8428   GGTLSPYSPQHPDPYNWEGNFFRKSEGRSFSYVHGAAAGTQHRQPAVVVITHSREMMKMA
43_9819   GGTLSPYSPQHPDPYNWEGNFFRKSEGRSFSYVHGAAAGTQHRQPAVVVITHSREMMKMA
44_5569   GGTLSPYSPQHPDPYNWEGNFFRKSEGRSFSYVHGAAAGTQHRQPAVVVITHSREMMKMA
45_4189   GGTLSPYSPQHPDPYNWEGNFFRKSEGRSFSYVHGAAAGTQHRQPAVVVITHSREMMKMA
46_1027   GGTLSPYSPQHPDPYNWEGNFFRKSEGRSFSYVHGAAAGTQHRQPAVVVITHSREMMKMA
47_5048   GGTLSPYSPQHPDPYNWEGNFFRKSEGRSFSYVHGAAAGTQHRQPAVVVITHSREMMKMA
48_10124  GGTLSPYSPQHPDPYNWEGNFFRKSEGRSFSYVHGAAAGTQHRQPAVVVITHSREMMKMA
49_7151   GGTLSPYSPQHPDPYNWEGNFFRKSEGRSFSYVHGAAAGTQHRQPAVVVITHSREMMKMA
50_3695   GGTLSPYSPQHPDPYNWEGNFFRKSEGRSFSYVHGAAAGTQHRQPAVVVITHSREMMKMA
51_364    SFSYVHGAAAGTQHRQPAVVVITHSREMMKMADRLVVIDNGCVAETGTYEYLMAAGDSRL
52_242    GGTLSPYSPQHPDPYNWEGNFFRKSEGRSFSYVHGAAAGTQHRQPAVVVITHSREMMKMA
53_5225   GGTLSPYSPQHPDPYNWEGNFFRKSEGRSFSYVHGAAAGTQHRQPAVVVITHSREMMKMA
54_1635   GGTLSPYSPQHPDPYNWEGNFFRKSEGRSFSYVHGAAAGTQHRQPAVVVITHSREMMKMA
55_5766   GGTLSPYSPQHPDPYNWEGNFFRKSEGRSFSYVHGAAAGTQHRQPAVVVITHSREMMKMA
56_1557   GGTLSPYSPQHPDPYNWEGNFFRKSEGRSFSYVHGAAAGTQHRQPAVVVITHSREMMKMA
57_10137  GGTLSPYSPQHPDPYNWEGNFFRKSEGRSFSYVHGAAAGTQHRQPAVVVITHSREMMKMA
58_10525  GGTLSPYSPQHPDPYNWEGNFFRKSEGRSFSYVHGAAAGTQHRQPAVVVITHSREMMKMA
59_3457   GGTLSPYSPQHPDPYNWEGNFFRKSEGRSFSYVHGAAAGTQHRQPAVVVITHSREMMKMA
60_8244   GGTLSPYSPQHPDPYNWEGNFFRKSEGRSFSYVHGAAAGTQHRQPAVVVITHSREMMKMA
61_7964   GGTLSPYSPQHPDPYNWEGNFFRKSEGRSFSYVHGAAAGTQHRQPAVVVITHSREMMKMA
62_4413   GGTLSPYSPQHPDPYNWEGNFFRKSEGRSFSYVHGAAAGTQHRQPAVVVITHSREMMKMA
63_6958   GGTLSPYSPQHPDPYNWEGNFFRKSEGRSFSYVHGAAAGTQHRQPAVVVITHSREMMKMA
64_5997   ............................................................
65_8711   GGTLSPYSPQHPDPYNWEGNFFRKSEGRSFSYVHGAAAGTQHRQPAVVVITHSREMMKMA
66_9925   GGTLSPYSPQHPDPYNWEGNFFRKSEGRSFSYVHGAAAGTQHRQPAVVVITHSREMMKMA
67_10374  GGTLSPYSPQHPDPYNWEGNFFRKSEGRSFSYVHGAAAGTQHRQPAVVVITHSREMMKMA
68_3713   GGTLSPYSPQHPDPYNWEGNFFRKSEGRSFSYVHGAAAGTQHRQPAVVVITHSREMMKMA
69_4511   GGTLSPYSPQHPDPYNWEGNFFRKSEGRSFSYVHGAAAGTQHRQPAVVVITHSREMMKMA
70_6201   GGTLSPYSPQHPDPYNWEGNFFRKSEGRSFSYVHGAAAGTQHRQPAVVVITHSREMMKMA
71_3261   GGTLSPYSPQHPDPYNWEGNFFRKSEGRSFSYVHGAAAGTQHRQPAVVVITHSREMMKMA
72_8516   GGTLSPYSPQHPDPYNWEGNFFRKSEGRSFSYVHGAAAGTQHRQPAVVVITHSREMMKMA
73_9295   GGTLSPYSPQHPDPYNWEGNFFRKSEGRSFSYVHGAAAGTQHRQPAVVVITHSREMMKMA
74_809    GGTLSPYSPQHPDPYNWEGNFFRKSEGRSFSYVHGAAAGTQHRQPAVVVITHSREMMKMA
75_7282   GGTLSPYSPQHPDPYNWEGNFFRKSEGRSFSYVHGAAAGTQHRQPAVVVITHSREMMKMA
76_4910   GGTLSPYSPQHPDPYNWEGNFFRKSEGRSFSYVHGAAAGTQHRQPAVVVITHSREMMKMA
77_10912  GGTLSPYSPQHPDPYNWEGNFFRKSEGRSFSYVHGAAAGTQHRQPAVVVITHSREMMKMA
78_4020   GGTLSPYSPQHPDPYNWEGNFFRKSEGRSFSYVHGAAAGTQHRQPAVVVITHSREMMKMA
79_6703   GGTLSPYSPQHPDPYNWEGNFFRKSEGRSFSYVHGAAAGTQHRQPAVVVITHSREMMKMA
80_8619   GGTLSPYSPQHPDPYNWEGNFFRKSEGRSFSYVHGAAAGTQHRQPAVVVITHSREMMKMA
81_4126   DPYNWEGNFFRKSEGRSFSYVHGAAAGTQHRQPAVVVITHSREMMKMADRLVVIDNGCVA
82_1363   GGTLSPYSPQHPDPYNWEGNFFRKSEGRSFSYVHGAAAGTQHRQPAVVVITHSREMMKMA
83_5813   GGTLSPYSPQHPDPYNWEGNFFRKSEGRSFSYVHGAAAGTQHRQPAVVVITHSREMMKMA
84_2044   GGTLSPYSPQHPDPYNWEGNFFRKSEGRSFSYVHGAAAGTQHRQPAVVVITHSREMMKMA
85_7617   GGTLSPYSPQHPDPYNWEGNFFRKSEGRSFSYVHGAAAGTQHRQPAVVVITHSREMMKMA
86_1530   GGTLSPYSPQHPDPYNWEGNFFRKSEGRSFSYVHGAAAGTQHRQPAVVVITHSREMMKMA
87_1890   GGTLSPYSPQHPDPYNWEGNFFRKSEGRSFSYVHGAAAGTQHRQPAVVVITHSREMMKMA
88_7049   GGTLSPYSPQHPDPYNWEGNFFRKSEGRSFSYVHGAAAGTQHRQPAVVVITHSREMMKMA
89_440    GGTLSPYSPQHPDPYNWEGNFFRKSEGRSFSYVHGAAAGTQHRQPAVVVITHSREMMKMA
90_4331   GGTLSPYSPQHPDPYNWEGNFFRKSEGRSFSYVHGAAAGTQHRQPAVVVITHSREMMKMA
```

```
              1570      1580      1590      1600      1610      1620
1_1896    DRLVVIDNGCVAETGTYEYLMAAGDSRLAELLDGGYRAPAAKSSGVPMEKTTSLEPVPVS
2_4785    DRLVVIDNGCVAETGTYEYLMAAGDSRLAELLDGGYRAPAAKSSGVPMEKTTSLEPVPVS
3_3653    DRLVVIDNGCVAETGTYEYLMAAGDSRLAELLDGGYRAPAAKSSGVPMEKTTSLEPVPVS
4_8157    DRLVVIDNGCVAETGTYEYLMAAGDSRLAELLDGGYRAPAAKSSGVPMEKTTSLEPVPVS
5_752     DRLVVIDNGCVAETGTYEYLMAAGDSRLAELLDGGYRAPAAKSSGVPMEKTTSLEPVPVS
6_8853    DRLVVIDNGCVAETGTYEYLMAAGDSRLAELLDGGYRAPAAKSSGVPMEKTTSLEPVPVS
7_4015    ............................................................
8_2020    DRLVVIDNGCVAETGTYEYLMAAGDSRLAELLDGGYRAPAAKSSGVPMEKTTSLEPVPVS
9_1829    DRLVVIDNGCVAETGTYEYLMAAGDSRLAELLDGGYRAPAAKSSGVPMEKTTSLEPVPVS
10_6452   DRLVVIDNGCVAETGTYEYLMAAGDSRLAELLDGGYRAPAAKSSGVPMEKTTSLEPVPVS
11_8627   DRLVVIDNGCVAETGTYEYLMAAGDSRLAELLDGGYRAPAAKSSGVPMEKTTSLEPVPVS
12_6118   DRLVVIDNGCVAETGTYEYLMAAGDSRLAELLDGGYRAPAAKSSGVPMEKTTSLEPVPVS
13_2688   DRLVVIDNGCVAETGTYEYLMAAGDSRLAELLDGGYRAPAAKSSGVPMEKTTSLEPVPVS
14_6406   DRLVVIDNGCVAETGTYEYLMAAGDSRLAELLDGGYRAPAAKSSGVPMEKTTSLEPVPVS
15_9419   DRLVVIDNGCVAETGTYEYLMAAGDSRLAELLDGGYRAPAAKSSGVPMEKTTSLEPVPVS
16_8507   DRLVVIDNGCVAETGTYEYLMAAGDSRLAELLDGGYRAPAAKSSGVPMEKTTSLEPVPVS
17_5884   DRLVVIDNGCVAETGTYEYLMAAGDSRLAELLDGGYRAPAAKSSGVPMENTTSLEPVPVS
18_2670   DRLVVIDNGCVAETGTYEYLMAAGDSRLAELLDGGYRAPAAKSSGVPMENTTSLEPVPVS
19_5172   DRLVVIDNGCVAETGTYEYLMAAGDSRLAELLDGGYRAPAAKSSGVPMEKTTSLEPVPVS
20_8057   DRLVVIDNGCVAETGTYEYLMAAGDSRLAELLDGGYRAPAAKSSGVPMEKTTSLEPVPVS
21_4920   ............................................................
22_6393   DRLVVIDNGCVAETGTYEYLMAAGDSRLAELLDGGYRAPAAKSSGVPMENTTSLEPVPVS
23_8263   DRLVVIDNGCVAETGTYEYLMAAGDSRLAELLDGGYRAPAAKSSGVPMEKTTSLEPVPVS
24_8248   DRLVVIDNGCVAETGTYEYLMAAGDSRLAELLDGGYRAPAAKSSGVPMENTTSLEPVPVS
25_6733   DRLVVIDNGCVAETGTYEYLMAAGDSRLAELLDGGYRAPAAKSSGVPMEKTTSLEPVPVS
26_2277   DRLVVIDNGCVAETGTYEYLMAAGDSRLAELLDGGYRAPAAKSSGVPMEKTTSLEPVPVS
27_2623   DRLVVIDNGCVAETGTYEYLMAAGDSRLAELLDGGYRAPAAKSSGVPMEKTTSLEPVPVS
28_7413   DRLVVIDNGCVAETGTYEYLMAAGDSRLAELLDGGYRAPAAKSSGVPMEKTTSLEPVPVS
29_8980   DRLVVIDNGCVAETGTYEYLMAAGDSRLAELLDGGYRAPAAKSSGVPMEKTTSLEPVPVS
30_8706   DRLVVIDNGCVAETGTYEYLMAAGDSRLAELLDGGYRAPAAKSSGVPMENTTSLEPVPVS
31_6378   DRLVVIDNGCVAETGTYEYLMAAGDSRLAELLDGGYRAPAAKSSGVPMEKTTSLEPVPVS
32_4679   DRLVVIDNGCVAETGTYEYLMAAGDSRLAELLDGGYRAPAAKSSGVPMENTTSLEPVPVS
33_2437   DRLVVIDNGCVAETGTYEYLMAAGDSRLAELLDGGYRAPAAKSSGVPMEKTTSLEPVPVS
34_9700   DRLVVIDNGCVAETGTYEYLMAAGDSRLAELLDGGYRAPAAKSSGVPMENTTSLEPVPVS
35_10218  DRLVVIDNGCVAETGTYEYLMAAGDSRLAELLDGGYRAPAAKSSGVPMEKTTSLEPVPVS
36_2652   DRLVVIDNGCVAETGTYEYLMAAGDSRLAELLDGGYRAPAAKSSGVPMENTTSLEPVPVS
37_5779   DRLVVIDNGCVAETGTYEYLMAAGDSRLAELLDGGYRAPAAKSSGVPMEKTTSLEPVPVS
38_654    DRLVVIDNGCVAETGTYEYLMAAGDSRLAELLDGGYRAPAAKSSGVPMEKTTSLEPVPVS
39_4879   DRLVVIDNGCVAETGTYEYLMAAGDSRLAELLDGGYRAPAAKSSGVPMENTTSLEPVPVS
40_8113   DRLVVIDNGCVAETGTYEYLMAAGDSRLAELLDGGYRAPAAKSSGVPMEKTTSLEPVPVS
41_1767   RQRLRGRDGHL.................................................
42_8428   DRLVVIDNGCVAETGTYEYLMAAGDSRLAELLDGGYRAPAAKSSGVPMENTTSLEPVPVS
43_9819   DRLVVIDNGCVAETGTYEYLMAAGDSRLAELLDGGYRAPAAKSSGVPMEKTTSLEPVPVS
44_5569   DRLVVIDNGCVAETGTYEYLMAAGDSRLAELLDGGYRAPAAKSSGVPMENTTSLEPVPVS
45_4189   DRLVVIDNGCVAETGTYEYLMAAGDSRLAELLDGGYRAPAAKSSGVPMEKTTSLEPVPVS
46_1027   DRLVVIDNGCVAETGTYEYLMAAGDSRLAELLDGGYRAPAAKSSGVPMEKTTSLEPVPVS
47_5048   DRLVVIDNGCVAETGTYEYLMAAGDSRLAELLDGGYRAPAAKSSGVPMEKTTSLEPVPVS
48_10124  DRLVVIDNGCVAETGTYEYLMAAGDSRLAELLDGGYRAPAAKSSGVPMENTTSLEPVPVS
49_7151   DRLVVIDNGCVAETGTYEYLMAAGDSRLAELLDGGYRAPAAKSSGVPMENTTSLEPVPVS
50_3695   DRLVVIDNGCVAETGTYEYLMAAGDSRLAELLDGGYRAPAAKSSGVPMENTTSLEPVPVS
51_364    AELLDGGYRAPAAKSSGVPMENTTSLEPVPVSPRTLTRVVSPSPEQYRRNNRSSPGGIPQ
52_242    DRLVVIDNGCVAETGTYEYLMAAGDSRLAELLDGGYRAPAAKSSGVPMENTTSLEPVPVS
53_5225   DRLVVIDNGCVAETGTYEYLMAAGDSRLAELLDGGYRAPAAKSSGVPMENTTSLEPVPVS
54_1635   DRLVVIDNGCVAETGTYEYLMAAGDSRLAELLDGGYRAPAAKSSGVPMEKTTSLEPVPVS
55_5766   DRLVVIDNGCVAETGTYEYLMAAGDSRLAELLDGGYRAPAAKSSGVPMEKTTSLEPVPVS
56_1557   DRLVVIDNGCVAETGTYEYLMAAGDSRLAELLDGGYRAPAAKSSGVPMENTTSLEPVPVS
57_10137  DRLVVIDNGCVAETGTYEYLMAAGDSRLAELLDGGYRAPAAKSSGVPMEKTTSLEPVPVS
58_10525  DRLVVIDNGCVAETGTYEYLMAAGDSRLAELLDGGYRAPAAKSSGVPMEKTTSLEPVPVS
59_3457   DRLVVIDNGCVAETGTYEYLMAAGDSRLAELLDGGYRAPAAKSSGVPMENTTSLEPVPVS
60_8244   DRLVVIDNGCVAETGTYEYLMAAGDSRLAELLDGGYRAPAAKSSGVPMEKTTSLEPVPVS
61_7964   DRLVVIDNGCVAETGTYEYLMAAGDSRLAELLDGGYRAPAAKSSGVPMEKTTSLEPVPVS
62_4413   DRLVVIDNGCVAETGTYEYLMAAGDSRLAELLDGGYRAPAAKSSGVPMEKTTSLEPVPVS
63_6958   DRLVVIDNGCVAETGTYEYLMAAGDSRLAELLDGGYRAPAAKSSGVPMEKTTSLEPVPVS
64_5997   ............................................................
65_8711   DRLVVIDNGCVAETGTYEYLMAAGDSRLAELLDGGYRAPAAKSSGVPMEKTTSLEPVPVS
66_9925   DRLVVIDNGCVAETGTYEYLMAAGDSRLAELLDGGYRAPAAKSSGVPMENTTSLEPVPVS
67_10374  DRLVVIDNGCVAETGTYEYLMAAGDSRLAELLDGGYRAPAAKSSGVPMEKTTSLEPVPVS
68_3713   DRLVVIDNGCVAETGTYEYLMAAGDSRLAELLDGGYRAPAAKSSGVPMEKTTSLEPVPVS
69_4511   DRLVVIDNGCVAETGTYEYLMAAGDSRLAELLDGGYRAPAAKSSGVPMEKTTSLEPVPVS
70_6201   DRLVVIDNGCVAETGTYEYLMAAGDSRLAELLDGGYRAPAAKSSGVPMEKTTSLEPVPVS
71_3261   DRLVVIDNGCVAETGTYEYLMAAGDSRLAELLDGGYRAPAAKSSGVPMENTTSLEPVPVS
72_8516   DRLVVIDNGCVAETGTYEYLMAAGDSRLAELLDGGYRAPAAKSSGVPMENTTSLEPVPVS
73_9295   DRLVVIDNGCVAETGTYEYLMAAGDSRLAELLDGGYRAPAAKSSGVPMENTTSLEPVPVS
74_809    DRLVVIDNGCVAETGTYEYLMAAGDSRLAELLDGGYRAPAAKSSGVPMENTTSLEPVPVS
75_7282   DRLVVIDNGCVAETGTYEYLMAAGDSRLAELLDGGYRAPAAKSSGVPMENTTSLEPVPVS
76_4910   DRLVVIDNGCVAETGTYEYLMAAGDSRLAELLDGGYRAPAAKSSGVPMENTTSLEPVPVS
77_10912  DRLVVIDNGCVAETGTYEYLMAAGDSRLAELLDGGYRAPAAKSSGVPMEKTTSLEPVPVS
78_4020   DRLVVIDNGCVAETGTYEYLMAAGDSRLAELLDGGYRAPAAKSSGVPMEKTTSLEPVPVS
79_6703   DRLVVIDNGCVAETGTYEYLMAAGDSRLAELLDGGYRAPAAKSSGVPMEKTTSLEPVPVS
80_8619   DRLVVIDNGCVAETGTYEYLMAAGDSRLAELLDGGYRAPAAKSSGVPMEKTTSLEPVPVS
81_4126   ETGTYEYLMAAGDSRLAELLDGGYRAPAAKSSGVPMENTTSLEPVPVSPRTLTRVVSPSP
82_1363   DRLVVIDNGCVAETGTYEYLMAAGDSRLAELLDGGYRAPAAKSSGVPMENTTSLEPVPVS
83_5813   DRLVVIDNGCVAETGTYEYLMAAGDSRLAELLDGGYRAPAAKSSGVPMENTTSLEPVPVS
84_2044   DRLVVIDNGCVAETGTYEYLMAAGDSRLAELLDGGYRAPAAKSSGVPMENTTSLEPVPVS
85_7617   DRLVVIDNGCVAETGTYEYLMAAGDSRLAELLDGGYRAPAAKSSGVPMEKTTSLEPVPVS
86_1530   DRLVVIDNGCVAETGTYEYLMAAGDSRLAELLDGGYRAPAAKSSGVPMENTTSLEPVPVS
87_1890   DRLVVIDNGCVAETGTYEYLMAAGDSRLAELLDGGYRAPAAKSSGVPMENTTSLEPVPVS
88_7049   DRLVVIDNGCVAETGTYEYLMAAGDSRLAELLDGGYRAPAAKSSGVPMENTTSLEPVPVS
89_440    DRLVVIDNGCVAETGTYEYLMAAGDSRLAELLDGGYRAPAAKSSGVPMEKTTSLEPVPVS
90_4331   DRLVVIDNGCVAETGTYEYLMAAGDSRLAELLDGGYRAPAAKSSGVPMEKTTSLEPVPVS
```

```
               1630      1640      1650      1660      1670
1_1896    PRTLTRVVSPSPEQYRRNNRSSPGGIPQRQQRPGMNMEDMFLPQVAWQEDDRS
2_4785    PRTLTRVVSPSPEQYRRNNRSSPGGIPQRQQRPGMNMEDMFLPQVAWQEDDRS
3_3653    PRTLTRVVSPSPEQYRRNNRSSPGGIPQRQQRPGMNMEDMFLPQVAWQEDDRS
4_8157    PRTLTRVVSPSPEQYRRNNRSSPGGIPQRQQRPGMNMEDMFLPQVAWQEDDRS
5_752     PRTLTRVVSPSPEQYRRNNRSSPGGIPQRQQRPGMNMEDMFLPQVAWQEDDRS
6_8853    PRTLTRVVSPSPEQYRRNNRSSPGGIPQRQQRPGMNMEDMFLPQVAWQEDDRS
7_4015    .....................................................
8_2020    PRTLTRVVSPSPEQYRRNNRSSPGGIPQRQQRPGMNMEDMFLPQVAWQEDDRS
9_1829    PRTLTRVVSPSPEQYRRNNRSSPGGIPQRQQRPGMNMEDMFLPQVAWQEDDRS
10_6452   PRTLTRVVSPSPEQYRRNNRSSPGGIPQRQQRPGMNMEDMFLPQVAWQEDDRS
11_8627   PRTLTRVVSPSPEQYRRNNRSSPGGIPQRQQRPGMNMEDMFLPQVAWQEDDRS
12_6118   PRTLTRVVSPSPEQYRRNNRSSPGGIPQRQQRPGMNMEDMFLPQVAWQEDDRS
13_2688   PRTLTRVVSPSPEQYRRNNRSSPGGIPQRQQRPGMNMEDMFLPQVAWQEDDRS
14_6406   PRTLTRVVSPSPEQYRRNNRSSPGGIPQRQQRPGMNMEDMFLPQVAWQEDDRS
15_9419   PRTLTRVVSPSPEQYRRNNRSSPGGIPQRQQRPGMNMEDMFLPQVAWQEDDRS
16_8507   PRTLTRVVSPSPEQYRRNNRSSPGGIPQRQQRPGMNMEDMFLPQVAWQEDDRS
17_5884   PRTLTRVVSPSPEQYRRNNRSSPGGIPQRQQRPGMNMEDMFLPQVAWQEDDRS
18_2670   PRTLTRVVSPSPEQYRRNNRSSPGGIPQRQQRPGMNMEDMFLPQVAWQEDDRS
19_5172   PRTLTRVVSPSPEQYRRNNRSSPGGIPQRQQRPGMNMEDMFLPQVAWQEDDRS
20_8057   PRTLTRVVSPSPEQYRRNNRSSPGGIPQRQQRPGMNMEDMFLPQVAWQEDDRS
21_4920   .....................................................
22_6393   PRTLTRVVSPSPEQYRRNNRSSPGGIPQRQQRPGMNMEDMFLPQVAWQEDDRS
23_8263   PRTLTRVVSPSPEQYRRNNRSSPGGIPQRQQRPGMNMEDMFLPQVAWQEDDRS
24_8248   PRTLTRVVSPSPEQYRRNNRSSPGGIPQRQQRPGMNMEDMFLPQVAWQEDDRS
25_6733   PRTLTRVVSPSPEQYRRNNRSSPGGIPQRQQRPGMNMEDMFLPQVAWQEDDRS
26_2277   PRTLTRVVSPSPEQYRRNNRSSPGGIPQRQQRPGMNMEDMFLPQVAWQEDDRS
27_2623   PRTLTRVVSPSPEQYRRNNRSSPGGIPQRQQRPGMNMEDMFLPQVAWQEDDRS
28_7413   PRTLTRVVSPSPEQYRRNNRSSPGGIPQRQQRPGMNMEDMFLPQVAWQEDDRS
29_8980   PRTLTRVVSPSPEQYRRNNRSSPGGIPQRQQRPGMNMEDMFLPQVAWQEDDRS
30_8706   PRTLTRVVSPSPEQYRRNNRSSPGGIPQRQQRPGMNMEDMFLPQVAWQEDDRS
31_6378   PRTLTRVVSPSPEQYRRNNRSSPGGIPQRQQRPGMNMEDMFLPQVAWQEDDRS
32_4679   PRTLTRVVSPSPEQYRRNNRSSPGGIPQRQQRPGMNMEDMFLPQVAWQEDDRS
33_2437   PRTLTRVVSPSPEQYRRNNRSSPGGIPQRQQRPGMNMEDMFLPQVAWQEDDRS
34_9700   PRTLTRVVSPSPEQYRRNNRSSPGGIPQRQQRPGMNMEDMFLPQVAWQEDDRS
35_10218  PRTLTRVVSPSPEQYRRNNRSSPGGIPQRQQRPGMNMEDMFLPQVAWQEDDRS
36_2652   PRTLTRVVSPSPEQYRRNNRSSPGGIPQRQQRPGMNMEDMFLPQVAWQEDDRS
37_5779   PRTLTRVVSPSPEQYRRNNRSSPGGIPQRQQRPGMNMEDMFLPQVAWQEDDRS
38_654    PRTLTRVVSPSPEQYRRNNRSSPGGIPQRQQRPGMNMEDMFLPQVAWQEDDRS
39_4879   PRTLTRVVSPSPEQYRRNNRSSPGGIPQRQQRPGMNMEDMFLPQVAWQEDDRS
40_8113   PRTLTRVVSPSPEQYRRNNRSSPGGIPQRQQRPGMNMEDMFLPQVAWQEDDRS
41_1767   .....................................................
42_8428   PRTLTRVVSPSPEQYRRNNRSSPGGIPQRQQRPGMNMEDMFLPQVAWQEDDRS
43_9819   PRTLTRVVSPSPEQYRRNNRSSPGGIPQRQQRPGMNMEDMFLPQVAWQEDDRS
44_5569   PRTLTRVVSPSPEQYRRNNRSSPGGIPQRQQRPGMNMEDMFLPQVAWQEDDRS
45_4189   PRTLTRVVSPSPEQYRRNNRSSPGGIPQRQQRPGMNMEDMFLPQVAWQEDDRS
46_1027   PRTLTRVVSPSPEQYRRNNRSSPGGIPQRQQRPGMNMEDMFLPQVAWQEDDRS
47_5048   PRTLTRVVSPSPEQYRRNNRSSPGGIPQRQQRPGMNMEDMFLPQVAWQEDDRS
48_10124  PRTLTRVVSPSPEQYRRNNRSSPGGIPQRQQRPGMNMEDMFLPQVAWQEDDRS
49_7151   PRTLTRVVSPSPEQYRRNNRSSPGGIPQRQQRPGMNMEDMFLPQVAWQEDDRS
50_3695   PRTLTRVVSPSPEQYRRNNRSSPGGIPQRQQRPGMNMEDMFLPQVAWQEDDRS
51_364    RQQRPGMNMEDMFLPQVAWQEDDRS............................
52_242    PRTLTRVVSPSPEQYRRNNRSSPGGIPQRQQRPGMNMEDMFLPQVAWQEDDRS
53_5225   PRTLTRVVSPSPEQYRRNNRSSPGGIPQRQQRPGMNMEDMFLPQVAWQEDDRS
54_1635   PRTLTRVVSPSPEQYRRNNRSSPGGIPQRQQRPGMNMEDMFLPQVAWQEDDRS
55_5766   PRTLTRVVSPSPEQYRRNNRSSPGGIPQRQQRPGMNMEDMFLPQVAWQEDDRS
56_1557   PRTLTRVVSPSPEQYRRNNRSSPGGIPQRQQRPGMNMEDMFLPQVAWQEDDRS
57_10137  PRTLTRVVSPSPEQYRRNNRSSPGGIPQRQQRPGMNMEDMFLPQVAWQEDDRS
58_10525  PRTLTRVVSPSPEQYRRNNRSSPGGIPQRQQRPGMNMEDMFLPQVAWQEDDRS
59_3457   PRTLTRVVSPSPEQYRRNNRSSPGGIPQRQQRPGMNMEDMFLPQVAWQEDDRS
60_8244   PRTLTRVVSPSPEQYRRNNRSSPGGIPQRQQRPGMNMEDMFLPQVAWQEDDRS
61_7964   PRTLTRVVSPSPEQYRRNNRSSPGGIPQRQQRPGMNMEDMFLPQVAWQEDDRS
62_4413   PRTLTRVVSPSPEQYRRNNRSSPGGIPQRQQRPGMNMEDMFLPQVAWQEDDRS
63_6958   PRTLTRVVSPSPEQYRRNNRSSPGGIPQRQQRPGMNMEDMFLPQVAWQEDDRS
64_5997   .....................................................
65_8711   PRTLTRVVSPSPEQYRRNNRSSPGGIPQRQQRPGMNMEDMFLPQVAWQEDDRS
66_9925   PRTLTRVVSPSPEQYRRNNRSSPGGIPQRQQRPGMNMEDMFLPQVAWQEDDRS
67_10374  PRTLTRVVSPSPEQYRRNNRSSPGGIPQRQQRPGMNMEDMFLPQVAWQEDDRS
68_3713   PRTLTRVVSPSPEQYRRNNRSSPGGIPQRQQRPGMNMEDMFLPQVAWQEDDRS
69_4511   PRTLTRVVSPSPEQYRRNNRSSPGGIPQRQQRPGMNMEDMFLPQVAWQEDDRS
70_6201   PRTLTRVVSPSPEQYRRNNRSSPGGIPQRQQRPGMNMEDMFLPQVAWQEDDRS
71_3261   PRTLTRVVSPSPEQYRRNNRSSPGGIPQRQQRPGMNMEDMFLPQVAWQEDDRS
72_8516   PRTLTRVVSPSPEQYRRNNRSSPGGIPQRQQRPGMNMEDMFLPQVAWQEDDRS
73_9295   PRTLTRVVSPSPEQYRRNNRSSPGGIPQRQQRPGMNMEDMFLPQVAWQEDDRS
74_809    PRTLTRVVSPSPEQYRRNNRSSPGGIPQRQQRPGMNMEDMFLPQVAWQEDDRS
75_7282   PRTLTRVVSPSPEQYRRNNRSSPGGIPQRQQRPGMNMEDMFLPQVAWQEDDRS
76_4910   PRTLTRVVSPSPEQYRRNNRSSPGGIPQRQQRPGMNMEDMFLPQVAWQEDDRS
77_10912  PRTLTRVVSPSPEQYRRNNRSSPGGIPQRQQRPGMNMEDMFLPQVAWQEDDRS
78_4020   PRTLTRVVSPSPEQYRRNNRSSPGGIPQRQQRPGMNMEDMFLPQVAWQEDDRS
79_6703   PRTLTRVVSPSPEQYRRNNRSSPGGIPQRQQRPGMNMEDMFLPQVAWQEDDRS
80_8619   PRTLTRVVSPSPEQYRRNNRSSPGGIPQRQQRPGMNMEDMFLPQVAWQEDDRS
81_4126   EQYRRNNRSSPGGIPQRQQRPGMNMEDMFLPQVAWQEDDRS............
82_1363   PRTLTRVVSPSPEQYRRNNRSSPGGIPQRQQRPGMNMEDMFLPQVAWQEDDRS
83_5813   PRTLTRVVSPSPEQYRRNNRSSPGGIPQRQQRPGMNMEDMFLPQVAWQEDDRS
84_2044   PRTLTRVVSPSPEQYRRNNRSSPGGIPQRQQRPGMNMEDMFLPQVAWQEDDRS
85_7617   PRTLTRVVSPSPEQYRRNNRSSPGGIPQRQQRPGMNMEDMFLPQVAWQEDDRS
86_1530   PRTLTRVVSPSPEQYRRNNRSSPGGIPQRQQRPGMNMEDMFLPQVAWQEDDRS
87_1890   PRTLTRVVSPSPEQYRRNNRSSPGGIPQRQQRPGMNMEDMFLPQVAWQEDDRS
88_7049   PRTLTRVVSPSPEQYRRNNRSSPGGIPQRQQRPGMNMEDMFLPQVAWQEDDRS
89_440    PRTLTRVVSPSPEQYRRNNRSSPGGIPQRQQRPGMNMEDMFLPQVAWQEDDRS
90_4331   PRTLTRVVSPSPEQYRRNNRSSPGGIPQRQQRPGMNMEDMFLPQVAWQEDDRS
```
