# Supplementary material for: ABCC Transporter Gene MoABC-R1 Is Associated with Pyraclostrobin Tolerance in Magnaporthe oryzae
Source: J Fungi (Basel). 2023 Sep 11;9(9):917. doi: 10.3390/jof9090917 (PMC10532721; doi:10.3390/jof9090917)
Supplement: Supplementary file 1 [file jof-09-00917-s001.zip › Supplementary data S4-Protein sequence alignment of gene MoABC-R1 in 90 strains.pdf]

```
           1        10        20        30        40        50        60
1_4160     MECPADADRVFGPAIQGCRSDFDFTLLFQDSVLGILPSSVLIILAAARLVFLARRHAVAS
2_6143     MECPADADRVFGPAIQGCRSDFDFTLLFQDSVLGILPSSVLIILAAARLVFLARRHAVAS
3_6554     MECPADADRVFGPAIQGCRSDFDFTLLFQDSVLGILPSSVLIILAAARLVFLARRHAVAS
4_111      MECPADADRVFGPAIQGCRSDFDFTLLFQDSVLGILPSSVLIILAAARLVFLARRHAVAS
5_5742     MECPADADRVFGPAIQGCRSDFDFTLLFQDSVLGILPSSVLIILAAARLVFLARRHAVAS
6_9490     MECPADADRVFGPAIQGCRSDFDFTLLFQDSVLGILPSSVLIILAAARLVFLARRHAVAS
7_6313     MISDVQRIVGGLVYYVHEMWAGVLETALATYLLQRVMGVSSVAMLGLALAYSTITLSPLLV
8_2552     MECPADADRVFGPAIQGCRSDFDFTLLFQDSVLGILPSSVLIILAAARLVFLARRHAVAS
9_7745     MECPADADRVFGPAIQGCRSDFDFTLLFQDSVLGILPSSVLIILAAARLVFLARRHAVAS
10_2315    MECPADADRVFGPAIQGCRSDFDFTLLFQDSVLGILPSSVLIILAAARLVFLARRHAVAS
11_2035    MECPADADRVFGPAIQGCRSDFDFTLLFQDSVLGILPSSVLIILAAARLVFLARRHAVAS
12_9640    MECPADADRVFGPAIQGCRSDFDFTLLFQDSVLGILPSSVLIILAAARLVFLARRHAVAS
13_3674    MECPADADRVFGPAIQGCRSDFDFTLLFQDSVLGILPSSVLIILAAARLVFLARRHAVAS
14_6548    MECPADADRVFGPAIQGCRSDFDFTLLFQDSVLGILPSSVLIILAAARLVFLARRHAVAS
15_10828   MECPADADRVFGPAIQGCRSDFDFTLLFQDSVLGILPSSVLIILAAARLVFLARRHAVAS
16_6578    MECPADADRVFGPAIQGCRSDFDFTLLFQDSVLGILPSSVLIILAAARLVFLARRHAVAS
17_10233   MECPADADRVFGPAIQGCRSDFDFTLLFQDSVLGILPSSVLIILAAARLVFLARRHAVAS
18_9261    MECPADADRVFGPAIQGCRSDFDFTLLFQDSVLGILPSSVLIILAAARLVFLARRHAVAS
19_8450    MECPADADRVFGPAIQGCRSDFDFTLLFQDSVLGILPSSVLIILAAARLVFLARRHAVAS
20_1727    MECPADADRVFGPAIQGCRSDFDFTLLFQDSVLGILPSSVLIILAAARLVFLARRHAVAS
21_206     MECPADADRVFGPAIQGCRSDFDFTLLFQDSVLGILPSSVLIILAAARLVFLARRHAVAS
22_7439    MECPADADRVFGPAIQGCRSDFDFTLLFQDSVLGILPSSVLIILAAARLVFLARRHAVAS
23_3780    MECPADADRVFGPAIQGCRSDFDFTLLFQDSVLGILPSSVLIILAAARLVFLARRHAVAS
24_967     MECPADADRVFGPAIQGCRSDFDFTLLFQDSVLGILPSSVLIILAAARLVFLARRHAVAS
25_6595    MECPADADRVFGPAIQGCRSDFDFTLLFQDSVLGILPSSVLIILAAARLVFLARRHAVAS
26_8391    MECPADADRVFGPAIQGCRSDFDFTLLFQDSVLGILPSSVLIILAAARLVFLARRHAVAS
27_8170    MECPADADRVFGPAIQGCRSDFDFTLLFQDSVLGILPSSVLIILAAARLVFLARRHAVAS
28_5209    MECPADADRVFGPAIQGCRSDFDFTLLFQDSVLGILPSSVLIILAAARLVFLARRHAVAS
29_2181    MECPADADRVFGPAIQGCRSDFDFTLLFQDSVLGILPSSVLIILAAARLVFLARRHAVAS
30_8564    MECPADADRVFGPAIQGCRSDFDFTLLFQDSVLGILPSSVLIILAAARLVFLARRHAVAS
31_1582    MECPADADRVFGPAIQGCRSDFDFTLLFQDSVLGILPSSVLIILAAARLVFLARRHAVAS
32_4537    MECPADADRVFGPAIQGCRSDFDFTLLFQDSVLGILPSSVLIILAAARLVFLARRHAVAS
33_9418    MECPADADRVFGPAIQGCRSDFDFTLLFQDSVLGILPSSVLIILAAARLVFLARRHAVAS
34_1782    MECPADADRVFGPAIQGCRSDFDFTLLFQDSVLGILPSSVLIILAAARLVFLARRHAVAS
35_10360   MECPADADRVFGPAIQGCRSDFDFTLLFQDSVLGILPSSVLIILAAARLVFLARRHAVAS
36_468     MECPADADRVFGPAIQGCRSDFDFTLLFQDSVLGILPSSVLIILAAARLVFLARRHAVAS
37_4956    MECPADADRVFGPAIQGCRSDFDFTLLFQDSVLGILPSSVLIILAAARLVFLARRHAVAS
38_4305    MECPADADRVFGPAIQGCRSDFDFTLLFQDSVLGILPSSVLIILAAARLVFLARRHAVAS
39_4737    MECPADADRVFGPAIQGCRSDFDFTLLFQDSVLGILPSSVLIILAAARLVFLARRHAVAS
40_9543    MECPADADRVFGPAIQGCRSDFDFTLLFQDSVLGILPSSVLIILAAARLVFLARRHAVAS
41_6669    MECPADADRVFGPAIQGCRSDFDFTLLFQDSVLGILPSSVLIILAAARLVFLARRHAVAS
42_10558   MECPADADRVFGPAIQGCRSDFDFTLLFQDSVLGILPSSVLIILAAARLVFLARRHAVAS
43_8051    MECPADADRVFGPAIQGCRSDFDFTLLFQDSVLGILPSSVLIILAAARLVFLARRHAVAS
44_10454   MECPADADRVFGPAIQGCRSDFDFTLLFQDSVLGILPSSVLIILAAARLVFLARRHAVAS
45_10126   MECPADADRVFGPAIQGCRSDFDFTLLFQDSVLGILPSSVLIILAAARLVFLARRHAVAS
46_1547    MECPADADRVFGPAIQGCRSDFDFTLLFQDSVLGILPSSVLIILAAARLVFLARRHAVAS
47_6749    MECPADADRVFGPAIQGCRSDFDFTLLFQDSVLGILPSSVLIILAAARLVFLARRHAVAS
48_9982    MECPADADRVFGPAIQGCRSDFDFTLLFQDSVLGILPSSVLIILAAARLVFLARRHAVAS
49_7293    MECPADADRVFGPAIQGCRSDFDFTLLFQDSVLGILPSSVLIILAAARLVFLARRHAVAS
50_5993    MECPADADRVFGPAIQGCRSDFDFTLLFQDSVLGILPSSVLIILAAARLVFLARRHAVAS
51_4791    MECPADADRVFGPAIQGCRSDFDFTLLFQDSVLGILPSSVLIILAAARLVFLARRHAVAS
52_121     MECPADADRVFGPAIQGCRSDFDFTLLFQDSVLGILPSSVLIILAAARLVFLARRHAVAS
53_1667    MECPADADRVFGPAIQGCRSDFDFTLLFQDSVLGILPSSVLIILAAARLVFLARRHAVAS
54_10488   MECPADADRVFGPAIQGCRSDFDFTLLFQDSVLGILPSSVLIILAAARLVFLARRHAVAS
55_7733    MECPADADRVFGPAIQGCRSDFDFTLLFQDSVLGILPSSVLIILAAARLVFLARRHAVAS
56_3982    MECPADADRVFGPAIQGCRSDFDFTLLFQDSVLGILPSSVLIILAAARLVFLARRHAVAS
57_392     MECPADADRVFGPAIQGCRSDFDFTLLFQDSVLGILPSSVLIILAAARLVFLARRHAVAS
58_10291   MECPADADRVFGPAIQGCRSDFDFTLLFQDSVLGILPSSVLIILAAARLVFLARRHAVAS
59_5792    MECPADADRVFGPAIQGCRSDFDFTLLFQDSVLGILPSSVLIILAAARLVFLARRHAVAS
60_8712    MECPADADRVFGPAIQGCRSDFDFTLLFQDSVLGILPSSVLIILAAARLVFLARRHAVAS
61_6716    MECPADADRVFGPAIQGCRSDFDFTLLFQDSVLGILPSSVLIILAAARLVFLARRHAVAS
62_5599    MECPADADRVFGPAIQGCRSDFDFTLLFQDSVLGILPSSVLIILAAARLVFLARRHAVAS
63_304     MECPADADRVFGPAIQGCRSDFDFTLLFQDSVLGILPSSVLIILAAARLVFLARRHAVAS
64_10473   MNRFNQDLMFVDTRLPIDLFNTSIDFFITIIQLILVVLVSKEALAILPVVFGALYLIQKV
65_6310    MECPADADRVFGPAIQGCRSDFDFTLLFQDSVLGILPSSVLIILAAARLVFLARRHAVAS
66_8714    MECPADADRVFGPAIQGCRSDFDFTLLFQDSVLGILPSSVLIILAAARLVFLARRHAVAS
67_2350    MECPADADRVFGPAIQGCRSDFDFTLLFQDSVLGILPSSVLIILAAARLVFLARRHAVAS
68_2447    MECPADADRVFGPAIQGCRSDFDFTLLFQDSVLGILPSSVLIILAAARLVFLARRHAVAS
69_5658    MECPADADRVFGPAIQGCRSDFDFTLLFQDSVLGILPSSVLIILAAARLVFLARRHAVAS
70_1277    MECPADADRVFGPAIQGCRSDFDFTLLFQDSVLGILPSSVLIILAAARLVFLARRHAVAS
71_3717    MECPADADRVFGPAIQGCRSDFDFTLLFQDSVLGILPSSVLIILAAARLVFLARRHAVAS
72_7846    MECPADADRVFGPAIQGCRSDFDFTLLFQDSVLGILPSSVLIILAAARLVFLARRHAVAS
73_10528   MECPADADRVFGPAIQGCRSDFDFTLLFQDSVLGILPSSVLIILAAARLVFLARRHAVAS
74_4899    MECPADADRVFGPAIQGCRSDFDFTLLFQDSVLGILPSSVLIILAAARLVFLARRHAVAS
75_5507    MECPADADRVFGPAIQGCRSDFDFTLLFQDSVLGILPSSVLIILAAARLVFLARRHAVAS
76_5432    MECPADADRVFGPAIQGCRSDFDFTLLFQDSVLGILPSSVLIILAAARLVFLARRHAVAS
77_10770   MECPADADRVFGPAIQGCRSDFDFTLLFQDSVLGILPSSVLIILAAARLVFLARRHAVAS
78_5904    MECPADADRVFGPAIQGCRSDFDFTLLFQDSVLGILPSSVLIILAAARLVFLARRHAVAS
79_9653    MECPADADRVFGPAIQGCRSDFDFTLLFQDSVLGILPSSVLIILAAARLVFLARRHAVAS
80_4462    MECPADADRVFGPAIQGCRSDFDFTLLFQDSVLGILPSSVLIILAAARLVFLARRHAVAS
81_7194    MECPADADRVFGPAIQGCRSDFDFTLLFQDSVLGILPSSVLIILAAARLVFLARRHAVAS
82_1022    MECPADADRVFGPAIQGCRSDFDFTLLFQDSVLGILPSSVLIILAAARLVFLARRHAVAS
83_9813    MECPADADRVFGPAIQGCRSDFDFTLLFQDSVLGILPSSVLIILAAARLVFLARRHAVAS
84_1902    MECPADADRVFGPAIQGCRSDFDFTLLFQDSVLGILPSSVLIILAAARLVFLARRHAVAS
85_5152    MECPADADRVFGPAIQGCRSDFDFTLLFQDSVLGILPSSVLIILAAARLVFLARRHAVAS
86_3385    MECPADADRVFGPAIQGCRSDFDFTLLFQDSVLGILPSSVLIILAAARLVFLARRHAVAS
87_10760   MECPADADRVFGPAIQGCRSDFDFTLLFQDSVLGILPSSVLIILAAARLVFLARRHAVAS
88_1588    MECPADADRVFGPAIQGCRSDFDFTLLFQDSVLGILPSSVLIILAAARLVFLARRHAVAS
89_1609    MECPADADRVFGPAIQGCRSDFDFTLLFQDSVLGILPSSVLIILAAARLVFLARRHAVAS
90_4023    MECPADADRVFGPAIQGCRSDFDFTLLFQDSVLGILPSSVLIILAAARLVFLARRHAVAS
```

```
                  70        80        90       100       110       120
1_4160    LNWLYHSKIGFNILSFALQLTILVQRCRIQDLETSAAIPWATLGLAATCALFLLSAVEHR
2_6143    LNWLYHSKIGFNILSFALQLTILVQRCRIQDLETSAAIPWATLGLAATCALFLLSAVEHR
3_6554    LNWLYHSKIGFNILSFALQLTILVQRCRIQDLETSAAIPWATLGLAATCALFLLSAVEHR
4_111     LNWLYHSKIGFNILSFALQLTILVQRCRIQDLETSAAIPWATLGLAATCALFLLSAVEHR
5_5742    LNWLYHSKIGFNILSFALQLTILVQRCRIQDLETSAAIPWATLGLAATCALFLLSAVEHR
6_9490    LNWLYHSKIGFNILSFALQLTILVQRCRIQDLETSAAIPWATLGLAATCALFLLSAVEHR
7_6313    FAAYIGVNGDDENLDSATMFSSLVLISLLGSPLVHLFQAMPALGSAQGCFKRIHAFLETP
8_2552    LNWLYHSKIGFNILSFALQLTILVQRCRIQDLETSAAIPWATLGLAATCALFLLSAVEHR
9_7745    LNWLYHSKIGFNILSFALQLTILVQRCRIQDLETSAAIPWATLGLAATCALFLLSAVEHR
10_2315   LNWLYHSKIGFNILSFALQLTILVQRCRIQDLETSAAIPWATLGLAATCALFLLSAVEHR
11_2035   LNWLYHSKIGFNILSFALQLTILVQRCRIQDLETSAAIPWATLGLAATCALFLLSAVEHR
12_9640   LNWLYHSKIGFNILSFALQLTILVQRCRIQDLETSAAIPWATLGLAATCALFLLSAVEHR
13_3674   LNWLYHSKIGFNILSFALQLTILVQRCRIQDLETSAAIPWATLGLAATCALFLLSAVEHR
14_6548   LNWLYHSKIGFNILSFALQLTILVQRCRIQDLETSAAIPWATLGLAATCALFLLSAVEHR
15_10828  LNWLYHSKIGFNILSFALQLTILVQRCRIQDLETSAAIPWATLGLAATCALFLLSAVEHR
16_6578   LNWLYHSKIGFNILSFALQLTILVQRCRIQDLETSAAIPWATLGLAATCALFLLSAVEHR
17_10233  LNWLYHSKIGFNILSFALQLTILVQRCRIQDLETSAAIPWATLGLAATCALFLLSAVEHR
18_9261   LNWLYHSKIGFNILSFALQLTILVQRCRIQDLETSAAIPWATLGLAATCALFLLSAVEHR
19_8450   LNWLYHSKIGFNILSFALQLTILVQRCRIQDLETSAAIPWATLGLAATCALFLLSAVEHR
20_1727   LNWLYHSKIGFNILSFALQLTILVQRCRIQDLETSAAIPWATLGLAATCALFLLSAVEHR
21_206    LNWLYHSKIGFNILSFALQLTILVQRCRIQDLETSAAIPWATLGLAATCALFLLSAVEHR
22_7439   LNWLYHSKIGFNILSFALQLTILVQRCRIQDLETSAAIPWATLGLAATCALFLLSAVEHR
23_3780   LNWLYHSKIGFNILSFALQLTILVQRCRIQDLETSAAIPWATLGLAATCALFLLSAVEHR
24_967    LNWLYHSKIGFNILSFALQLTILVQRCRIQDLETSAAIPWATLGLAATCALFLLSAVEHR
25_6595   LNWLYHSKIGFNILSFALQLTILVQRCRIQDLETSAAIPWATLGLAATCALFLLSAVEHR
26_8391   LNWLYHSKIGFNILSFALQLTILVQRCRIQDLETSAAIPWATLGLAATCALFLLSAVEHR
27_8170   LNWLYHSKIGFNILSFALQLTILVQRCRIQDLETSAAIPWATLGLAATCALFLLSAVEHR
28_5209   LNWLYHSKIGFNILSFALQLTILVQRCRIQDLETSAAIPWATLGLAATCALFLLSAVEHR
29_2181   LNWLYHSKIGFNILSFALQLTILVQRCRIQDLETSAAIPWATLGLAATCALFLLSAVEHR
30_8564   LNWLYHSKIGFNILSFALQLTILVQRCRIQDLETSAAIPWATLGLAATCALFLLSAVEHR
31_1582   LNWLYHSKIGFNILSFALQLTILVQRCRIQDLETSAAIPWATLGLAATCALFLLSAVEHR
32_4537   LNWLYHSKIGFNILSFALQLTILVQRCRIQDLETSAAIPWATLGLAATCALFLLSAVEHR
33_9418   LNWLYHSKIGFNILSFALQLTILVQRCRIQDLETSAAIPWATLGLAATCALFLLSAVEHR
34_1782   LNWLYHSKIGFNILSFALQLTILVQRCRIQDLETSAAIPWATLGLAATCALFLLSAVEHR
35_10360  LNWLYHSKIGFNILSFALQLTILVQRCRIQDLETSAAIPWATLGLAATCALFLLSAVEHR
36_468    LNWLYHSKIGFNILSFALQLTILVQRCRIQDLETSAAIPWATLGLAATCALFLLSAVEHR
37_4956   LNWLYHSKIGFNILSFALQLTILVQRCRIQDLETSAAIPWATLGLAATCALFLLSAVEHR
38_4305   LNWLYHSKIGFNILSFALQLTILVQRCRIQDLETSAAIPWATLGLAATCALFLLSAVEHR
39_4737   LNWLYHSKIGFNILSFALQLTILVQRCRIQDLETSAAIPWATLGLAATCALFLLSAVEHR
40_9543   LNWLYHSKIGFNILSFALQLTILVQRCRIQDLETSAAIPWATLGLAATCALFLLSAVEHR
41_6669   LNWLYHSKIGFNILSFALQLTILVQRCRIQDLETSAAIPWATLGLAATCALFLLSAVEHR
42_10558  LNWLYHSKIGFNILSFALQLTILVQRCRIQDLETSAAIPWATLGLAATCALFLLSAVEHR
43_8051   LNWLYHSKIGFNILSFALQLTILVQRCRIQDLETSAAIPWATLGLAATCALFLLSAVEHR
44_10454  LNWLYHSKIGFNILSFALQLTILVQRCRIQDLETSAAIPWATLGLAATCALFLLSAVEHR
45_10126  LNWLYHSKIGFNILSFALQLTILVQRCRIQDLETSAAIPWATLGLAATCALFLLSAVEHR
46_1547   LNWLYHSKIGFNILSFALQLTILVQRCRIQDLETSAAIPWATLGLAATCALFLLSAVEHR
47_6749   LNWLYHSKIGFNILSFALQLTILVQRCRIQDLETSAAIPWATLGLAATCALFLLSAVEHR
48_9982   LNWLYHSKIGFNILSFALQLTILVQRCRIQDLETSAAIPWATLGLAATCALFLLSAVEHR
49_7293   LNWLYHSKIGFNILSFALQLTILVQRCRIQDLETSAAIPWATLGLAATCALFLLSAVEHR
50_5993   LNWLYHSKIGFNILSFALQLTILVQRCRIQDLETSAAIPWATLGLAATCALFLLSAVEHR
51_4791   LNWLYHSKIGFNILSFALQLTILVQRCRIQDLETSAAIPWATLGLAATCALFLLSAVEHR
52_121    LNWLYHSKIGFNILSFALQLTILVQRCRIQDLETSAAIPWATLGLAATCALFLLSAVEHR
53_1667   LNWLYHSKIGFNILSFALQLTILVQRCRIQDLETSAAIPWATLGLAATCALFLLSAVEHR
54_10488  LNWLYHSKIGFNILSFALQLTILVQRCRIQDLETSAAIPWATLGLAATCALFLLSAVEHR
55_7733   LNWLYHSKIGFNILSFALQLTILVQRCRIQDLETSAAIPWATLGLAATCALFLLSAVEHR
56_3982   LNWLYHSKIGFNILSFALQLTILVQRCRIQDLETSAAIPWATLGLAATCALFLLSAVEHR
57_392    LNWLYHSKIGFNILSFALQLTILVQRCRIQDLETSAAIPWATLGLAATCALFLLSAVEHR
58_10291  LNWLYHSKIGFNILSFALQLTILVQRCRIQDLETSAAIPWATLGLAATCALFLLSAVEHR
59_5792   LNWLYHSKIGFNILSFALQLTILVQRCRIQDLETSAAIPWATLGLAATCALFLLSAVEHR
60_8712   LNWLYHSKIGFNILSFALQLTILVQRCRIQDLETSAAIPWATLGLAATCALFLLSAVEHR
61_6716   LNWLYHSKIGFNILSFALQLTILVQRCRIQDLETSAAIPWATLGLAATCALFLLSAVEHR
62_5599   LNWLYHSKIGFNILSFALQLTILVQRCRIQDLETSAAIPWATLGLAATCALFLLSAVEHR
63_304    LNWLYHSKIGFNILSFALQLTILVQRCRIQDLETSAAIPWATLGLAATCALFLLSAVEHR
64_10473  YLRSSKQLRLLDLDWKADLHTAFGETTAGLSVIRANGWLDPMRAKFAEKLDRSQEPFYLL
65_6310   LNWLYHSKIGFNILSFALQLTILVQRCRIQDLETSAAIPWATLGLAATCALFLLSAVEHR
66_8714   LNWLYHSKIGFNILSFALQLTILVQRCRIQDLETSAAIPWATLGLAATCALFLLSAVEHR
67_2350   LNWLYHSKIGFNILSFALQLTILVQRCRIQDLETSAAIPWATLGLAATCALFLLSAVEHR
68_2447   LNWLYHSKIGFNILSFALQLTILVQRCRIQDLETSAAIPWATLGLAATCALFLLSAVEHR
69_5658   LNWLYHSKIGFNILSFALQLTILVQRCRIQDLETSAAIPWATLGLAATCALFLLSAVEHR
70_1277   LNWLYHSKIGFNILSFALQLTILVQRCRIQDLETSAAIPWATLGLAATCALFLLSAVEHR
71_3717   LNWLYHSKIGFNILSFALQLTILVQRCRIQDLETSAAIPWATLGLAATCALFLLSAVEHR
72_7846   LNWLYHSKIGFNILSFALQLTILVQRCRIQDLETSAAIPWATLGLAATCALFLLSAVEHR
73_10528  LNWLYHSKIGFNILSFALQLTILVQRCRIQDLETSAAIPWATLGLAATCALFLLSAVEHR
74_4899   LNWLYHSKIGFNILSFALQLTILVQRCRIQDLETSAAIPWATLGLAATCALFLLSAVEHR
75_5507   LNWLYHSKIGFNILSFALQLTILVQRCRIQDLETSAAIPWATLGLAATCALFLLSAVEHR
76_5432   LNWLYHSKIGFNILSFALQLTILVQRCRIQDLETSAAIPWATLGLAATCALFLLSAVEHR
77_10770  LNWLYHSKIGFNILSFALQLTILVQRCRIQDLETSAAIPWATLGLAATCALFLLSAVEHR
78_5904   LNWLYHSKIGFNILSFALQLTILVQRCRIQDLETSAAIPWATLGLAATCALFLLSAVEHR
79_9653   LNWLYHSKIGFNILSFALQLTILVQRCRIQDLETSAAIPWATLGLAATCALFLLSAVEHR
80_4462   LNWLYHSKIGFNILSFALQLTILVQRCRIQDLETSAAIPWATLGLAATCALFLLSAVEHR
81_7194   LNWLYHSKIGFNILSFALQLTILVQRCRIQDLETSAAIPWATLGLAATCALFLLSAVEHR
82_1022   LNWLYHSKIGFNILSFALQLTILVQRCRIQDLETSAAIPWATLGLAATCALFLLSAVEHR
83_9813   LNWLYHSKIGFNILSFALQLTILVQRCRIQDLETSAAIPWATLGLAATCALFLLSAVEHR
84_1902   LNWLYHSKIGFNILSFALQLTILVQRCRIQDLETSAAIPWATLGLAATCALFLLSAVEHR
85_5152   LNWLYHSKIGFNILSFALQLTILVQRCRIQDLETSAAIPWATLGLAATCALFLLSAVEHR
86_3385   LNWLYHSKIGFNILSFALQLTILVQRCRIQDLETSAAIPWATLGLAATCALFLLSAVEHR
87_10760  LNWLYHSKIGFNILSFALQLTILVQRCRIQDLETSAAIPWATLGLAATCALFLLSAVEHR
88_1588   LNWLYHSKIGFNILSFALQLTILVQRCRIQDLETSAAIPWATLGLAATCALFLLSAVEHR
89_1609   LNWLYHSKIGFNILSFALQLTILVQRCRIQDLETSAAIPWATLGLAATCALFLLSAVEHR
90_4023   LNWLYHSKIGFNILSFALQLTILVQRCRIQDLETSAAIPWATLGLAATCALFLLSAVEHR
```

```
                 130       140       150       160       170       180
1_4160     RSARPSSLIVAYLFIFVLTEATRARTYYLKRQTVAASITAANCCVKFVLLILEEQKKTLR
2_6143     RSARPSSLIVAYLFIFVLTEATRARTYYLKRQTVAASITAANCCVKFVLLILEEQKKTLR
3_6554     RSARPSSLIVAYLFIFVLTEATRARTYYLKRQTVAASITAANCCVKFVLLILEEQKKTLR
4_111      RSARPSSLIVAYLFIFVLTEATRARTYYLKRQTVAASITAANCCVKFVLLILEEQKKTLR
5_5742     RSARPSSLIVAYLFIFVLTEATRARTYYLKRQTVAASITAANCCVKFVLLILEEQKKTLR
6_9490     RSARPSSLIVAYLFIFVLTEATRARTYYLKRQTVAASITAANCCVKFVLLILEEQKKTLR
7_6313     EKPLVIKHKPEIITTCDEETKNAHLQLSEIEETALSIRNASIGWSPDEQPLLRDINLRIQ
8_2552     RSARPSSLIVAYLFIFVLTEATRARTYYLKRQTVAASITAANCCVKFVLLILEEQKKTLR
9_7745     RSARPSSLIVAYLFIFVLTEATRARTYYLKRQTVAASITAANCCVKFVLLILEEQKKTLR
10_2315    RSARPSSLIVAYLFIFVLTEATRARTYYLKRQTVAASITAANCCVKFVLLILEEQKKTLR
11_2035    RSARPSSLIVAYLFIFVLTEATRARTYYLKRQTVAASITAANCCVKFVLLILEEQKKTLR
12_9640    RSARPSSLIVAYLFIFVLTEATRARTYYLKRQTVAASITAANCCVKFVLLILEEQKKTLR
13_3674    RSARPSSLIVAYLFIFVLTEATRARTYYLKRQTVAASITAANCCVKFVLLILEEQKKTLR
14_6548    RSARPSSLIVAYLFIFVLTEATRARTYYLKRQTVAASITAANCCVKFVLLILEEQKKTLR
15_10828   RSARPSSLIVAYLFIFVLTEATRARTYYLKRQTVAASITAANCCVKFVLLILEEQKKTLR
16_6578    RSARPSSLIVAYLFIFVLTEATRARTYYLKRQTVAASITAANCCVKFVLLILEEQKKTLR
17_10233   RSARPSSLIVAYLFIFVLTEATRARTYYLKRQTVAASITAANCCVKFVLLILEEQKKTLR
18_9261    RSARPSSLIVAYLFIFVLTEATRARTYYLKRQTVAASITAANCCVKFVLLILEEQKKTLR
19_8450    RSARPSSLIVAYLFIFVLTEATRARTYYLKRQTVAASITAANCCVKFVLLILEEQKKTLR
20_1727    RSARPSSLIVAYLFIFVLTEATRARTYYLKRQTVAASITAANCCVKFVLLILEEQKKTLR
21_206     RSARPSSLIVAYLFIFVLTEATRARTYYLKRQTVAASITAANCCVKFVLLILEEQKKTLR
22_7439    RSARPSSLIVAYLFIFVLTEATRARTYYLKRQTVAASITAANCCVKFVLLILEEQKKTLR
23_3780    RSARPSSLIVAYLFIFVLTEATRARTYYLKRQTVAASITAANCCVKFVLLILEEQKKTLR
24_967     RSARPSSLIVAYLFIFVLTEATRARTYYLKRQTVAASITAANCCVKFVLLILEEQKKTLR
25_6595    RSARPSSLIVAYLFIFVLTEATRARTYYLKRQTVAASITAANCCVKFVLLILEEQKKTLR
26_8391    RSARPSSLIVAYLFIFVLTEATRARTYYLKRQTVAASITAANCCVKFVLLILEEQKKTLR
27_8170    RSARPSSLIVAYLFIFVLTEATRARTYYLKRQTVAASITAANCCVKFVLLILEEQKKTLR
28_5209    RSARPSSLIVAYLFIFVLTEATRARTYYLKRQTVAASITAANCCVKFVLLILEEQKKTLR
29_2181    RSARPSSLIVAYLFIFVLTEATRARTYYLKRQTVAASITAANCCVKFVLLILEEQKKTLR
30_8564    RSARPSSLIVAYLFIFVLTEATRARTYYLKRQTVAASITAANCCVKFVLLILEEQKKTLR
31_1582    RSARPSSLIVAYLFIFVLTEATRARTYYLKRQTVAASITAANCCVKFVLLILEEQKKTLR
32_4537    RSARPSSLIVAYLFIFVLTEATRARTYYLKRQTVAASITAANCCVKFVLLILEEQKKTLR
33_9418    RSARPSSLIVAYLFIFVLTEATRARTYYLKRQTVAASITAANCCVKFVLLILEEQKKTLR
34_1782    RSARPSSLIVAYLFIFVLTEATRARTYYLKRQTVAASITAANCCVKFVLLILEEQKKTLR
35_10360   RSARPSSLIVAYLFIFVLTEATRARTYYLKRQTVAASITAANCCVKFVLLILEEQKKTLR
36_468     RSARPSSLIVAYLFIFVLTEATRARTYYLKRQTVAASITAANCCVKFVLLILEEQKKTLR
37_4956    RSARPSSLIVAYLFIFVLTEATRARTYYLKRQTVAASITAANCCVKFVLLILEEQKKTLR
38_4305    RSARPSSLIVAYLFIFVLTEATRARTYYLKRQTVAASITAANCCVKFVLLILEEQKKTLR
39_4737    RSARPSSLIVAYLFIFVLTEATRARTYYLKRQTVAASITAANCCVKFVLLILEEQKKTLR
40_9543    RSARPSSLIVAYLFIFVLTEATRARTYYLKRQTVAASITAANCCVKFVLLILEEQKKTLR
41_6669    RSARPSSLIVAYLFIFVLTEATRARTYYLKRQTVAASITAANCCVKFVLLILEEQKKTLR
42_10558   RSARPSSLIVAYLFIFVLTEATRARTYYLKRQTVAASITAANCCVKFVLLILEEQKKTLR
43_8051    RSARPSSLIVAYLFIFVLTEATRARTYYLKRQTVAASITAANCCVKFVLLILEEQKKTLR
44_10454   RSARPSSLIVAYLFIFVLTEATRARTYYLKRQTVAASITAANCCVKFVLLILEEQKKTLR
45_10126   RSARPSSLIVAYLFIFVLTEATRARTYYLKRQTVAASITAANCCVKFVLLILEEQKKTLR
46_1547    RSARPSSLIVAYLFIFVLTEATRARTYYLKRQTVAASITAANCCVKFVLLILEEQKKTLR
47_6749    RSARPSSLIVAYLFIFVLTEATRARTYYLKRQTVAASITAANCCVKFVLLILEEQKKTLR
48_9982    RSARPSSLIVAYLFIFVLTEATRARTYYLKRQTVAASITAANCCVKFVLLILEEQKKTLR
49_7293    RSARPSSLIVAYLFIFVLTEATRARTYYLKRQTVAASITAANCCVKFVLLILEEQKKTLR
50_5993    RSARPSSLIVAYLFIFVLTEATRARTYYLKRQTVAASITAANCCVKFVLLILEEQKKTLR
51_4791    RSARPSSLIVAYLFIFVLTEATRARTYYLKRQTVAASITAANCCVKFVLLILEEQKKTLR
52_121     RSARPSSLIVAYLFIFVLTEATRARTYYLKRQTVAASITAANCCVKFVLLILEEQKKTLR
53_1667    RSARPSSLIVAYLFIFVLTEATRARTYYLKRQTVAASITAANCCVKFVLLILEEQKKTLR
54_10488   RSARPSSLIVAYLFIFVLTEATRARTYYLKRQTVAASITAANCCVKFVLLILEEQKKTLR
55_7733    RSARPSSLIVAYLFIFVLTEATRARTYYLKRQTVAASITAANCCVKFVLLILEEQKKTLR
56_3982    RSARPSSLIVAYLFIFVLTEATRARTYYLKRQTVAASITAANCCVKFVLLILEEQKKTLR
57_392     RSARPSSLIVAYLFIFVLTEATRARTYYLKRQTVAASITAANCCVKFVLLILEEQKKTLR
58_10291   RSARPSSLIVAYLFIFVLTEATRARTYYLKRQTVAASITAANCCVKFVLLILEEQKKTLR
59_5792    RSARPSSLIVAYLFIFVLTEATRARTYYLKRQTVAASITAANCCVKFVLLILEEQKKTLR
60_8712    RSARPSSLIVAYLFIFVLTEATRARTYYLKRQTVAASITAANCCVKFVLLILEEQKKTLR
61_6716    RSARPSSLIVAYLFIFVLTEATRARTYYLKRQTVAASITAANCCVKFVLLILEEQKKTLR
62_5599    RSARPSSLIVAYLFIFVLTEATRARTYYLKRQTVAASITAANCCVKFVLLILEEQKKTLR
63_304     RSARPSSLIVAYLFIFVLTEATRARTYYLKRQTVAASITAANCCVKFVLLILEEQKKTLR
64_10473   YMVQRWLQLVLNLVVAGLAIAIAGVAIGLRDKVAAGAVGVALLNTTTLGETLTNFIMSWT
65_6310    RSARPSSLIVAYLFIFVLTEATRARTYYLKRQTVAASITAANCCVKFVLLILEEQKKTLR
66_8714    RSARPSSLIVAYLFIFVLTEATRARTYYLKRQTVAASITAANCCVKFVLLILEEQKKTLR
67_2350    RSARPSSLIVAYLFIFVLTEATRARTYYLKRQTVAASITAANCCVKFVLLILEEQKKTLR
68_2447    RSARPSSLIVAYLFIFVLTEATRARTYYLKRQTVAASITAANCCVKFVLLILEEQKKTLR
69_5658    RSARPSSLIVAYLFIFVLTEATRARTYYLKRQTVAASITAANCCVKFVLLILEEQKKTLR
70_1277    RSARPSSLIVAYLFIFVLTEATRARTYYLKRQTVAASITAANCCVKFVLLILEEQKKTLR
71_3717    RSARPSSLIVAYLFIFVLTEATRARTYYLKRQTVAASITAANCCVKFVLLILEEQKKTLR
72_7846    RSARPSSLIVAYLFIFVLTEATRARTYYLKRQTVAASITAANCCVKFVLLILEEQKKTLR
73_10528   RSARPSSLIVAYLFIFVLTEATRARTYYLKRQTVAASITAANCCVKFVLLILEEQKKTLR
74_4899    RSARPSSLIVAYLFIFVLTEATRARTYYLKRQTVAASITAANCCVKFVLLILEEQKKTLR
75_5507    RSARPSSLIVAYLFIFVLTEATRARTYYLKRQTVAASITAANCCVKFVLLILEEQKKTLR
76_5432    RSARPSSLIVAYLFIFVLTEATRARTYYLKRQTVAASITAANCCVKFVLLILEEQKKTLR
77_10770   RSARPSSLIVAYLFIFVLTEATRARTYYLKRQTVAASITAANCCVKFVLLILEEQKKTLR
78_5904    RSARPSSLIVAYLFIFVLTEATRARTYYLKRQTVAASITAANCCVKFVLLILEEQKKTLR
79_9653    RSARPSSLIVAYLFIFVLTEATRARTYYLKRQTVAASITAANCCVKFVLLILEEQKKTLR
80_4462    RSARPSSLIVAYLFIFVLTEATRARTYYLKRQTVAASITAANCCVKFVLLILEEQKKTLR
81_7194    RSARPSSLIVAYLFIFVLTEATRARTYYLKRQTVAASITAANCCVKFVLLILEEQKKTLR
82_1022    RSARPSSLIVAYLFIFVLTEATRARTYYLKRQTVAASITAANCCVKFVLLILEEQKKTLR
83_9813    RSARPSSLIVAYLFIFVLTEATRARTYYLKRQTVAASITAANCCVKFVLLILEEQKKTLR
84_1902    RSARPSSLIVAYLFIFVLTEATRARTYYLKRQTVAASITAANCCVKFVLLILEEQKKTLR
85_5152    RSARPSSLIVAYLFIFVLTEATRARTYYLKRQTVAASITAANCCVKFVLLILEEQKKTLR
86_3385    RSARPSSLIVAYLFIFVLTEATRARTYYLKRQTVAASITAANCCVKFVLLILEEQKKTLR
87_10760   RSARPSSLIVAYLFIFVLTEATRARTYYLKRQTVAASITAANCCVKFVLLILEEQKKTLR
88_1588    RSARPSSLIVAYLFIFVLTEATRARTYYLKRQTVAASITAANCCVKFVLLILEEQKKTLR
89_1609    RSARPSSLIVAYLFIFVLTEATRARTYYLKRQTVAASITAANCCVKFVLLILEEQKKTLR
90_4023    RSARPSSLIVAYLFIFVLTEATRARTYYLKRQTVAASITAANCCVKFVLLILEEQKKTLR
```

	190 200 210 220 230 240
1_4160	SGSDGSRKKAASEDLAGPTNQTFFLWLNRLFLTGYRRAFTTTDLELISSPLYVKSIRAQF
2_6143	SGSDGSRKKAASEDLAGPTNQTFFLWLNRLFLTGYRRAFTTTDLELISSPLYVKSIRAQF
3_6554	SGSDGSRKKAASEDLAGPTNQTFFLWLNRLFLTGYRRAFTTTDLELISSPLYVKSIRAQF
4_111	SGSDGSRKKAASEDLAGPTNQTFFLWLNRLFLTGYRRAFTTTDLELISSPLYVKSIRAQF
5_5742	SGSDGSRKKAASEDLAGPTNQTFFLWLNRLFLTGYRRAFTTTDLELISSPLYVKSIRAQF
6_9490	SGSDGSRKKAASEDLAGPTNQTFFLWLNRLFLTGYRRAFTTTDLELISSPLYVKSIRAQF
7_6313	KGSFVALVGKTGSGKSLLLKSTIGEGGHVSGSIHIPLDKVAAGRIGSGGSRLSGGQKQRL
8_2552	SGSDGSRKKAASEDLAGPTNQTFFLWLNRLFLTGYRRAFTTTDLELISSPLYVKSIRAQF
9_7745	SGSDGSRKKAASEDLAGPTNQTFFLWLNRLFLTGYRRAFTTTDLELISSPLYVKSIRAQF
10_2315	SGSDGSRKKAASEDLAGPTNQTFFLWLNRLFLTGYRRAFTTTDLELISSPLYVKSIRAQF
11_2035	SGSDGSRKKAASEDLAGPTNQTFFLWLNRLFLTGYRRAFTTTDLELISSPLYVKSIRAQF
12_9640	SGSDGSRKKAASEDLAGPTNQTFFLWLNRLFLTGYRRAFTTTDLELISSPLYVKSIRAQF
13_3674	SGSDGSRKKAASEDLAGPTNQTFFLWLNRLFLTGYRRAFTTTDLELISSPLYVKSIRAQF
14_6548	SGSDGSRKKAASEDLAGPTNQTFFLWLNRLFLTGYRRAFTTTDLELISSPLYVKSIRAQF
15_10828	SGSDGSRKKAASEDLAGPTNQTFFLWLNRLFLTGYRRAFTTTDLELISSPLYVKSIRAQF
16_6578	SGSDGSRKKAASEDLAGPTNQTFFLWLNRLFLTGYRRAFTTTDLELISSPLYVKSIRAQF
17_10233	SGSDGSRKKAASEDLAGPTNQTFFLWLNRLFLTGYRRAFTTTDLELISSPLYVKSIRAQF
18_9261	SGSDGSRKKAASEDLAGPTNQTFFLWLNRLFLTGYRRAFTTTDLELISSPLYVKSIRAQF
19_8450	SGSDGSRKKAASEDLAGPTNQTFFLWLNRLFLTGYRRAFTTTDLELISSPLYVKSIRAQF
20_1727	SGSDGSRKKAASEDLAGPTNQTFFLWLNRLFLTGYRRAFTTTDLELISSPLYVKSIRAQF
21_206	SGSDGSRKKAASEDLAGPTNQTFFLWLNRLFLTGYRRAFTTTDLELISSPLYVKSIRAQF
22_7439	SGSDGSRKKAASEDLAGPTNQTFFLWLNRLFLTGYRRAFTTTDLELISSPLYVKSIRAQF
23_3780	SGSDGSRKKAASEDLAGPTNQTFFLWLNRLFLTGYRRAFTTTDLELISSPLYVKSIRAQF
24_967	SGSDGSRKKAASEDLAGPTNQTFFLWLNRLFLTGYRRAFTTTDLELISSPLYVKSIRAQF
25_6595	SGSDGSRKKAASEDLAGPTNQTFFLWLNRLFLTGYRRAFTTTDLELISSPLYVKSIRAQF
26_8391	SGSDGSRKKAASEDLAGPTNQTFFLWLNRLFLTGYRRAFTTTDLELISSPLYVKSIRAQF
27_8170	SGSDGSRKKAASEDLAGPTNQTFFLWLNRLFLTGYRRAFTTTDLELISSPLYVKSIRAQF
28_5209	SGSDGSRKKAASEDLAGPTNQTFFLWLNRLFLTGYRRAFTTTDLELISSPLYVKSIRAQF
29_2181	SGSDGSRKKAASEDLAGPTNQTFFLWLNRLFLTGYRRAFTTTDLELISSPLYVKSIRAQF
30_8564	SGSDGSRKKAASEDLAGPTNQTFFLWLNRLFLTGYRRAFTTTDLELISSPLYVKSIRAQF
31_1582	SGSDGSRKKAASEDLAGPTNQTFFLWLNRLFLTGYRRAFTTTDLELISSPLYVKSIRAQF
32_4537	SGSDGSRKKAASEDLAGPTNQTFFLWLNRLFLTGYRRAFTTTDLELISSPLYVKSIRAQF
33_9418	SGSDGSRKKAASEDLAGPTNQTFFLWLNRLFLTGYRRAFTTTDLELISSPLYVKSIRAQF
34_1782	SGSDGSRKKAASEDLAGPTNQTFFLWLNRLFLTGYRRAFTTTDLELISSPLYVKSIRAQF
35_10360	SGSDGSRKKAASEDLAGPTNQTFFLWLNRLFLTGYRRAFTTTDLELISSPLYVKSIRAQF
36_468	SGSDGSRKKAASEDLAGPTNQTFFLWLNRLFLTGYRRAFTTTDLELISSPLYVKSIRAQF
37_4956	SGSDGSRKKAASEDLAGPTNQTFFLWLNRLFLTGYRRAFTTTDLELISSPLYVKSIRAQF
38_4305	SGSDGSRKKAASEDLAGPTNQTFFLWLNRLFLTGYRRAFTTTDLELISSPLYVKSIRAQF
39_4737	SGSDGSRKKAASEDLAGPTNQTFFLWLNRLFLTGYRRAFTTTDLELISSPLYVKSIRAQF
40_9543	SGSDGSRKKAASEDLAGPTNQTFFLWLNRLFLTGYRRAFTTTDLELISSPLYVKSIRAQF
41_6669	SGSDGSRKKAASEDLAGPTNQTFFLWLNRLFLTGYRRAFTTTDLELISSPLYVKSIRAQF
42_10558	SGSDGSRKKAASEDLAGPTNQTFFLWLNRLFLTGYRRAFTTTDLELISSPLYVKSIRAQF
43_8051	SGSDGSRKKAASEDLAGPTNQTFFLWLNRLFLTGYRRAFTTTDLELISSPLYVKSIRAQF
44_10454	SGSDGSRKKAASEDLAGPTNQTFFLWLNRLFLTGYRRAFTTTDLELISSPLYVKSIRAQF
45_10126	SGSDGSRKKAASEDLAGPTNQTFFLWLNRLFLTGYRRAFTTTDLELISSPLYVKSIRAQF
46_1547	SGSDGSRKKAASEDLAGPTNQTFFLWLNRLFLTGYRRAFTTTDLELISSPLYVKSIRAQF
47_6749	SGSDGSRKKAASEDLAGPTNQTFFLWLNRLFLTGYRRAFTTTDLELISSPLYVKSIRAQF
48_9982	SGSDGSRKKAASEDLAGPTNQTFFLWLNRLFLTGYRRAFTTTDLELISSPLYVKSIRAQF
49_7293	SGSDGSRKKAASEDLAGPTNQTFFLWLNRLFLTGYRRAFTTTDLELISSPLYVKSIRAQF
50_5993	SGSDGSRKKAASEDLAGPTNQTFFLWLNRLFLTGYRRAFTTTDLELISSPLYVKSIRAQF
51_4791	SGSDGSRKKAASEDLAGPTNQTFFLWLNRLFLTGYRRAFTTTDLELISSPLYVKSIRAQF
52_121	SGSDGSRKKAASEDLAGPTNQTFFLWLNRLFLTGYRRAFTTTDLELISSPLYVKSIRAQF
53_1667	SGSDGSRKKAASEDLAGPTNQTFFLWLNRLFLTGYRRAFTTTDLELISSPLYVKSIRAQF
54_10488	SGSDGSRKKAASEDLAGPTNQTFFLWLNRLFLTGYRRAFTTTDLELISSPLYVKSIRAQF
55_7733	SGSDGSRKKAASEDLAGPTNQTFFLWLNRLFLTGYRRAFTTTDLELISSPLYVKSIRAQF
56_3982	SGSDGSRKKAASEDLAGPTNQTFFLWLNRLFLTGYRRAFTTTDLELISSPLYVKSIRAQF
57_392	SGSDGSRKKAASEDLAGPTNQTFFLWLNRLFLTGYRRAFTTTDLELISSPLYVKSIRAQF
58_10291	SGSDGSRKKAASEDLAGPTNQTFFLWLNRLFLTGYRRAFTTTDLELISSPLYVKSIRAQF
59_5792	SGSDGSRKKAASEDLAGPTNQTFFLWLNRLFLTGYRRAFTTTDLELISSPLYVKSIRAQF
60_8712	SGSDGSRKKAASEDLAGPTNQTFFLWLNRLFLTGYRRAFTTTDLELISSPLYVKSIRAQF
61_6716	SGSDGSRKKAASEDLAGPTNQTFFLWLNRLFLTGYRRAFTTTDLELISSPLYVKSIRAQF
62_5599	SGSDGSRKKAASEDLAGPTNQTFFLWLNRLFLTGYRRAFTTTDLELISSPLYVKSIRAQF
63_304	SGSDGSRKKAASEDLAGPTNQTFFLWLNRLFLTGYRRAFTTTDLELISSPLYVKSIRAQF
64_10473	SLETSLGAIARVCTFEQDTPREREEPSTTDLPDNRPGAGQISFENVWATYEDEGCGSNWG
65_6310	SGSDGSRKKAASEDLAGPTNQTFFLWLNRLFLTGYRRAFTTTDLELISSPLYVKSIRAQF
66_8714	SGSDGSRKKAASEDLAGPTNQTFFLWLNRLFLTGYRRAFTTTDLELISSPLYVKSIRAQF
67_2350	SGSDGSRKKAASEDLAGPTNQTFFLWLNRLFLTGYRRAFTTTDLELISSPLYVKSIRAQF
68_2447	SGSDGSRKKAASEDLAGPTNQTFFLWLNRLFLTGYRRAFTTTDLELISSPLYVKSIRAQF
69_5658	SGSDGSRKKAASEDLAGPTNQTFFLWLNRLFLTGYRRAFTTTDLELISSPLYVKSIRAQF
70_1277	SGSDGSRKKAASEDLAGPTNQTFFLWLNRLFLTGYRRAFTTTDLELISSPLYVKSIRAQF
71_3717	SGSDGSRKKAASEDLAGPTNQTFFLWLNRLFLTGYRRAFTTTDLELISSPLYVKSIRAQF
72_7846	SGSDGSRKKAASEDLAGPTNQTFFLWLNRLFLTGYRRAFTTTDLELISSPLYVKSIRAQF
73_10528	SGSDGSRKKAASEDLAGPTNQTFFLWLNRLFLTGYRRAFTTTDLELISSPLYVKSIRAQF
74_4899	SSSDGSRKKAASEDLAGPTNQTFFLWLNRLFLTGYRRAFTTTDLELISSPLYVKSIRAQF
75_5507	SGSDGSRKKAASEDLAGPTNQTFFLWLNRLFLTGYRRAFTTTDLELISSPLYVKSIRAQF
76_5432	SGSDGSRKKAASEDLAGPTNQTFFLWLNRLFLTGYRRAFTTTDLELISSPLYVKSIRAQF
77_10770	SGSDGSRKKAASEDLAGPTNQTFFLWLNRLFLTGYRRAFTTTDLELISSPLYVKSIRAQF
78_5904	SGSDGSRKKAASEDLAGPTNQTFFLWLNRLFLTGYRRAFTTTDLELISSPLYVKSIRAQF
79_9653	SGSDGSRKKAASEDLAGPTNQTFFLWLNRLFLTGYRRAFTTTDLELISSPLYVKSIRAQF
80_4462	SGSDGSRKKAASEDLAGPTNQTFFLWLNRLFLTGYRRAFTTTDLELISSPLYVKSIRAQF
81_7194	SGSDGSRKKAASEDLAGPTNQTFFLWLNRLFLTGYRRAFTTTDLELISSPLYVKSIRAQF
82_1022	SGSDGSRKKAASEDLAGPTNQTFFLWLNRLFLTGYRRAFTTTDLELISSPLYVKSIRAQF
83_9813	SGSDGSRKKAASEDLAGPTNQTFFLWLNRLFLTGYRRAFTTTDLELISSPLYVKSIRAQF
84_1902	SGSDGSRKKAASEDLAGPTNQTFFLWLNRLFLTGYRRAFTTTDLELISSPLYVKSIRAQF
85_5152	SGSDGSRKKAASEDLAGPTNQTFFLWLNRLFLTGYRRAFTTTDLELISSPLYVKSIRAQF
86_3385	SGSDGSRKKAASEDLAGPTNQTFFLWLNRLFLTGYRRAFTTTDLELISSPLYVKSIRAQF
87_10760	SGSDGSRKKAASEDLAGPTNQTFFLWLNRLFLTGYRRAFTTTDLELISSPLYVKSIRAQF
88_1588	SGSDGSRKKAASEDLAGPTNQTFFLWLNRLFLTGYRRAFTTTDLELISSPLYVKSIRAQF
89_1609	SGSDGSRKKAASEDLAGPTNQTFFLWLNRLFLTGYRRAFTTTDLELISSPLYVKSIRAQF
90_4023	SGSDGSRKKAASEDLAGPTNQTFFLWLNRLFLTGYRRAFTTTDLELISSPLYVKSIRAQF

```
                    250       260       270       280       290       300
1_4160     HGMTNGQTANGHSLFSSSTNTFALQAFTSLGSYALAPVIPRLAVTGFTFSQSFLVTALLD
2_6143     HGMTNGQTANGHSLFSSSTNTFALQAFTSLGSYALAPVIPRLAVTGFTFSQSFLVTALLD
3_6554     HGMTNGQTANGHSLFSSSTNTFALQAFTSLGSYALAPVIPRLAVTGFTFSQSFLVTALLD
4_111      HGMTNGQTANGHSLFSSSTNTFALQAFTSLGSYALAPVIPRLAVTGFTFSQSFLVTALLD
5_5742     HGMTNGQTANGHSLFSSSTNTFALQAFTSLGSYALAPVIPRLAVTGFTFSQSFLVTALLD
6_9490     HGMTNGQTANGHSLFSSSTNTFALQAFTSLGSYALAPVIPRLAVTGFTFSQSFLVTALLD
7_6313     QDGFSDPRGCCEDWEQQFYLPLTTVTNLADEVYEINVDRMLTPVPVRKPVANGEVAKYED
8_2552     HGMTNGQTANGHSLFSSSTNTFALQAFTSLGSYALAPVIPRLAVTGFTFSQSFLVTALLD
9_7745     HGMTNGQTANGHSLFSSSTNTFALQAFTSLGSYALAPVIPRLAVTGFTFSQSFLVTALLD
10_2315    HGMTNGQTANGHSLFSSSTNTFALQAFTSLGSYALAPVIPRLAVTGFTFSQSFLVTALLD
11_2035    HGMTNGQTANGHSLFSSSTNTFALQAFTSLGSYALAPVIPRLAVTGFTFSQSFLVTALLD
12_9640    HGMTNGQTANGHSLFSSSTNTFALQAFTSLGSYALAPVIPRLAVTGFTFSQSFLVTALLD
13_3674    HGMTNGQTANGHSLFSSSTNTFALQAFTSLGSYALAPVIPRLAVTGFTFSQSFLVTALLD
14_6548    HGMTNGQTANGHSLFSSSTNTFALQAFTSLGSYALAPVIPRLAVTGFTFSQSFLVTALLD
15_10828   HGMTNGQTANGHSLFSSSTNTFALQAFTSLGSYALAPVIPRLAVTGFTFSQSFLVTALLD
16_6578    HGMTNGQTANGHSLFSSSTNTFALQAFTSLGSYALAPVIPRLAVTGFTFSQSFLVTALLD
17_10233   HGMTNGQTANGHSLFSSSTNTFALLAFTSLGSYALAPVIPRLAVTGFTFSQSFLVTALLD
18_9261    HGMTNGQTANGHSLFSSSTNTFALLAFTSLGSYALAPVIPRLAVTGFTFSQSFLVTALLD
19_8450    HGMTNGQTANGHSLFSSSTNTFALQAFTSLGSYALAPVIPRLAVTGFTFSQSFLVTALLD
20_1727    HGMTNGQTANGHSLFSSSTNTFALQAFTSLGSYALAPVIPRLAVTGFTFSQSFLVTALLD
21_206     HGMTNGQTANGHSLFSSSTNTFALQAFTSLGSYALAPVIPRLAVTGFTFSQSFLVTALLD
22_7439    HGMTNGQTANGHSLFSSSTNTFALLAFTSLGSYALAPVIPRLAVTGFTFSQSFLVTALLD
23_3780    HGMTNGQTANGHSLFSSSTNTFALQAFTSLGSYALAPVIPRLAVTGFTFSQSFLVTALLD
24_967     HGMTNGQTANGHSLFSSSTNTFALLAFTSLGSYALAPVIPRLAVTGFTFSQSFLVTALLD
25_6595    HGMTNGQTANGHSLFSSSTNTFALQAFTSLGSYALAPVIPRLAVTGFTFSQSFLVTALLD
26_8391    HGMTNGQTANGHSLFSSSTNTFALQAFTSLGSYALAPVIPRLAVTGFTFSQSFLVTALLD
27_8170    HGMTNGQTANGHSLFSSSTNTFALQAFTSLGSYALAPVIPRLAVTGFTFSQSFLVTALLD
28_5209    HGMTNGQTANGHSLFSSSTNTFALQAFTSLGSYALAPVIPRLAVTGFTFSQSFLVTALLD
29_2181    HGMTNGQTANGHSLFSSSTNTFALQAFTSLGSYALAPVIPRLAVTGFTFSQSFLVTALLD
30_8564    HGMTNGQTANGHSLFSSSTNTFALLAFTSLGSYALAPVIPRLAVTGFTFSQSFLVTALLD
31_1582    HGMTNGQTANGHSLFSSSTNTFALQAFTSLGSYALAPVIPRLAVTGFTFSQSFLVTALLD
32_4537    HGMTNGQTANGHSLFSSSTNTFALLAFTSLGSYALAPVIPRLAVTGFTFSQSFLVTALLD
33_9418    HGMTNGQTANGHSLFSSSTNTFALQAFTSLGSYALAPVIPRLAVTGFTFSQSFLVTALLD
34_1782    HGMTNGQTANGHSLFSSSTNTFALLAFTSLGSYALAPVIPRLAVTGFTFSQSFLVTALLD
35_10360   HGMTNGQTANGHSLFSSSTNTFALQAFTSLGSYALAPVIPRLAVTGFTFSQSFLVTALLD
36_468     HGMTNGQTANGHSLFSSSTNTFALLAFTSLGSYALAPVIPRLAVTGFTFSQSFLVTALLD
37_4956    HGMTNGQTANGHSLFSSSTNTFALQAFTSLGSYALAPVIPRLAVTGFTFSQSFLVTALLD
38_4305    HGMTNGQTANGHSLFSSSTNTFALQAFTSLGSYALAPVIPRLAVTGFTFSQSFLVTALLD
39_4737    HGMTNGQTANGHSLFSSSTNTFALLAFTSLGSYALAPVIPRLAVTGFTFSQSFLVTALLD
40_9543    HGMTNGQTANGHSLFSSSTNTFALQAFTSLGSYALAPVIPRLAVTGFTFSQSFLVTALLD
41_6669    HGMTNGQTANGHSLFSSSTNTFALQAFTSLGSYALAPVIPRLAVTGFTFSQSFLVTALLD
42_10558   HGMTNGQTANGHSLFSSSTNTFALLAFTSLGSYALAPVIPRLAVTGFTFSQSFLVTALLD
43_8051    HGMTNGQTANGHSLFSSSTNTFALQAFTSLGSYALAPVIPRLAVTGFTFSQSFLVTALLD
44_10454   HGMTNGQTANGHSLFSSSTNTFALLAFTSLGSYALAPVIPRLAVTGFTFSQSFLVTALLD
45_10126   HGMTNGQTANGHSLFSSSTNTFALQAFTSLGSYALAPVIPRLAVTGFTFSQSFLVTALLD
46_1547    HGMTNGQTANGHSLFSSSTNTFALQAFTSLGSYALAPVIPRLAVTGFTFSQSFLVTALLD
47_6749    HGMTNGQTANGHSLFSSSTNTFALQAFTSLGSYALAPVIPRLAVTGFTFSQSFLVTALLD
48_9982    HGMTNGQTANGHSLFSSSTNTFALLAFTSLGSYALAPVIPRLAVTGFTFSQSFLVTALLD
49_7293    HGMTNGQTANGHSLFSSSTNTFALLAFTSLGSYALAPVIPRLAVTGFTFSQSFLVTALLD
50_5993    HGMTNGQTANGHSLFSSSTNTFALLAFTSLGSYALAPVIPRLAVTGFTFSQSFLVTALLD
51_4791    HGMTNGQTANGHSLFSSSTNTFALLAFTSLGSYALAPVIPRLAVTGFTFSQSFLVTALLD
52_121     HGMTNGQTANGHSLFSSSTNTFALLAFTSLGSYALAPVIPRLAVTGFTFSQSFLVTALLD
53_1667    HGMTNGQTANGHSLFSSSTNTFALLAFTSLGSYALAPVIPRLAVTGFTFSQSFLVTALLD
54_10488   HGMTNGQTANGHSLFSSSTNTFALQAFTSLGSYALAPVIPRLAVTGFTFSQSFLVTALLD
55_7733    HGMTNGQTANGHSLFSSSTNTFALQAFTSLGSYALAPVIPRLAVTGFTFSQSFLVTALLD
56_3982    HGMTNGQTANGHSLFSSSTNTFALLAFTSLGSYALAPVIPRLAVTGFTFSQSFLVTALLD
57_392     HGMTNGQTANGHSLFSSSTNTFALQAFTSLGSYALAPVIPRLAVTGFTFSQSFLVTALLD
58_10291   HGMTNGQTANGHSLFSSSTNTFALQAFTSLGSYALAPVIPRLAVTGFTFSQSFLVTALLD
59_5792    HGMTNGQTANGHSLFSSSTNTFALLAFTSLGSYALAPVIPRLAVTGFTFSQSFLVTALLD
60_8712    HGMTNGQTANGHSLFSSSTNTFALQAFTSLGSYALAPVIPRLAVTGFTFSQSFLVTALLD
61_6716    HGMTNGQTANGHSLFSSSTNTFALQAFTSLGSYALAPVIPRLAVTGFTFSQSFLVTALLD
62_5599    HGMTNGQTANGHSLFSSSTNTFALQAFTSLGSYALAPVIPRLAVTGFTFSQSFLVTALLD
63_304     HGMTNGQTANGHSLFSSSTNTFALQAFTSLGSYALAPVIPRLAVTGFTFSQSFLVTALLD
64_10473   LSGITLAVQPGERVAVCGRTGSGKSTLLLALLGMLHTPAGSIRIDGVDTSTLPIDVLRRR
65_6310    HGMTNGQTANGHSLFSSSTNTFALQAFTSLGSYALAPVIPRLAVTGFTFSQSFLVTALLD
66_8714    HGMTNGQTANGHSLFSSSTNTFALLAFTSLGSYALAPVIPRLAVTGFTFSQSFLVTALLD
67_2350    HGMTNGQTANGHSLFSSSTNTFALQAFTSLGSYALAPVIPRLAVTGFTFSQSFLVTALLD
68_2447    HGMTNGQTANGHSLFSSSTNTFALQAFTSLGSYALAPVIPRLAVTGFTFSQSFLVTALLD
69_5658    HGMTNGQTANGHSLFSSSTNTFALQAFTSLGSYALAPVIPRLAVTGFTFSQSFLVTALLD
70_1277    HGMTNGQTANGHSLFSSSTNTFALQAFTSLGSYALAPVIPRLAVTGFTFSQSFLVTALLD
71_3717    HGMTNGQTANGHSLFSSSTNTFALLAFTSLGSYALAPVIPRLAVTGFTFSQSFLVTALLD
72_7846    HGMTNGQTANGHSLFSSSTNTFALLAFTSLGSYALAPVIPRLAVTGFTFSQSFLVTALLD
73_10528   HGMTNGQTANGHSLFSSSTNTFALLAFTSLGSYALAPVIPRLAVTGFTFSQSFLVTALLD
74_4899    HGMTNGQTANGHSLFSSSTNTFALLAFTSLGSYALAPVIPRLAVTGFTFSQSFLVTALLD
75_5507    HGMTNGQTANGHSLFSSSTNTFALLAFTSLGSYALAPVIPRLAVTGFTFSQSFLVTALLD
76_5432    HGMTNGQTANGHSLFSSSTNTFALLAFTSLGSYALAPVIPRLAVTGFTFSQSFLVTALLD
77_10770   HGMTNGQTANGHSLFSSSTNTFALLAFTSLGSYALAPVIPRLAVTGFTFSQSFLVTALLD
78_5904    HGMTNGQTANGHSLFSSSTNTFALQAFTSLGSYALAPVIPRLAVTGFTFSQSFLVTALLD
79_9653    HGMTNGQTANGHSLFSSSTNTFALQAFTSLGSYALAPVIPRLAVTGFTFSQSFLVTALLD
80_4462    HGMTNGQTANGHSLFSSSTNTFALQAFTSLGSYALAPVIPRLAVTGFTFSQSFLVTALLD
81_7194    HGMTNGQTANGHSLFSSSTNTFALLAFTSLGSYALAPVIPRLAVTGFTFSQSFLVTALLD
82_1022    HGMTNGQTANGHSLFSSSTNTFALLAFTSLGSYALAPVIPRLAVTGFTFSQSFLVTALLD
83_9813    HGMTNGQTANGHSLFSSSTNTFALLAFTSLGSYALAPVIPRLAVTGFTFSQSFLVTALLD
84_1902    HGMTNGQTANGHSLFSSSTNTFALLAFTSLGSYALAPVIPRLAVTGFTFSQSFLVTALLD
85_5152    HGMTNGQTANGHSLFSSSTNTFALQAFTSLGSYALAPVIPRLAVTGFTFSQSFLVTALLD
86_3385    HGMTNGQTANGHSLFSSSTNTFALLAFTSLGSYALAPVIPRLAVTGFTFSQSFLVTALLD
87_10760   HGMTNGQTANGHSLFSSSTNTFALLAFTSLGSYALAPVIPRLAVTGFTFSQSFLVTALLD
88_1588    HGMTNGQTANGHSLFSSSTNTFALLAFTSLGSYALAPVIPRLAVTGFTFSQSFLVTALLD
89_1609    HGMTNGQTANGHSLFSSSTNTFALQAFTSLGSYALAPVIPRLAVTGFTFSQSFLVTALLD
90_4023    HGMTNGQTANGHSLFSSSTNTFALQAFTSLGSYALAPVIPRLAVTGFTFSQSFLVTALLD
```

```
                   310       320       330       340       350       360
1_4160     YLENGRDRPASHGYGLLGAYAFVYIGIAVSNSWYSRHTYKSVSIIRGGLIVSIFEKVLRL
2_6143     YLENGRDRPASHGYGLLGAYAFVYIGIAVSNSWYSRHTYKSVSIIRGGLIVSIFEKVLRL
3_6554     YLENGRDRPASHGYGLLGAYAFVYIGIAVSNSWYSRHTYKSVSIIRGGLIVSIFEKVLRL
4_111      YLENGRDRPASHGYGLLGAYAFVYIGIAVSNSWYSRHTYKSVSIIRGGLIVSIFEKVLRL
5_5742     YLENGRDRPASHGYGLLGAYAFVYIGIAVSNSWYSRHTYKSVSIIRGGLIVSIFEKVLRL
6_9490     YLENGRDRPASHGYGLLGAYAFVYIGIAVSNSWYSRHTYKSVSIIRGGLIVSIFEKVLRL
7_6313     SDAKDTATDASNNEETSTAPDPGTASVTQNANNGDQGVGTPVSDKQVYLRYARAMGFRNA
8_2552     YLENGRDRPASHGYGLLGAYAFVYIGIAVSNSWYSRHTYKSVSIIRGGLIVSIFEKVLRL
9_7745     YLENGRDRPASHGYGLLGAYAFVYIGIAVSNSWYSRHTYKSVSIIRGGLIVSIFEKVLRL
10_2315    YLENGRDRPASHGYGLLGAYAFVYIGIAVSNSWYSRHTYKSVSIIRGGLIVSIFEKVLRL
11_2035    YLENGRDRPASHGYGLLGAYAFVYIGIAVSNSWYSRHTYKSVSIIRGGLIVSIFEKVLRL
12_9640    YLENGRDRPASHGYGLLGAYAFVYIGIAVSNSWYSRHTYKSVSIIRGGLIVSIFEKVLRL
13_3674    YLENGRDRPASHGYGLLGAYAFVYIGIAVSNSWYSRHTYKSVSIIRGGLIVSIFEKVLRL
14_6548    YLENGRDRPASHGYGLLGAYAFVYIGIAVSNSWYSRHTYKSVSIIRGGLIVSIFEKVLRL
15_10828   YLENGRDRPASHGYGLLGAYAFVYIGIAVSNSWYSRHTYKSVSIIRGGLIVSIFEKVLRL
16_6578    YLENGRDRPASHGYGLLGAYAFVYIGIAVSNSWYSRHTYKSVSIIRGGLIVSIFEKVLRL
17_10233   YLENGRDRPASHGYGLLGAYAFVYIGIAVSNSWYSRHTYKSVSIIRGGLIVSIFEKVLRL
18_9261    YLENGRDRPASHGYGLLGAYAFVYIGIAVSNSWYSRHTYKSVSIIRGGLIVSIFEKVLRL
19_8450    YLENGRDRPASHGYGLLGAYAFVYIGIAVSNSWYSRHTYKSVSIIRGGLIVSIFEKVLRL
20_1727    YLENGRDRPASHGYGLLGAYAFVYIGIAVSNSWYSRHTYKSVSIIRGGLIVSIFEKVLRL
21_206     YLENGRDRPASHGYGLLGAYAFVYIGIAVSNSWYSRHTYKSVSIIRGGLIVSIFEKVLRL
22_7439    YLENGRDRPASHGYGLLGAYAFVYIGIAVSNSWYSRHTYKSVSIIRGGLIVSIFEKVLRL
23_3780    YLENGRDRPASHGYGLLGAYAFVYIGIAVSNSWYSRHTYKSVSIIRGGLIVSIFEKVLRL
24_967     YLENGRDRPASHGYGLLGAYAFVYIGIAVSNSWYSRHTYKSVSIIRGGLIVSIFEKVLRL
25_6595    YLENGRDRPASHGYGLLGAYAFVYIGIAVSNSWYSRHTYKSVSIIRGGLIVSIFEKVLRL
26_8391    YLENGRDRPASHGYGLLGAYAFVYIGIAVSNSWYSRHTYKSVSIIRGGLIVSIFEKVLRL
27_8170    YLENGRDRPASHGYGLLGAYAFVYIGIAVSNSWYSRHTYKSVSIIRGGLIVSIFEKVLRL
28_5209    YLENGRDRPASHGYGLLGAYAFVYIGIAVSNSWYSRHTYKSVSIIRGGLIVSIFEKVLRL
29_2181    YLENGRDRPASHGYGLLGAYAFVYIGIAVSNSWYSRHTYKSVSIIRGGLIVSIFEKVLRL
30_8564    YLENGRDRPASHGYGLLGAYAFVYIGIAVSNSWYSRHTYKSVSIIRGGLIVSIFEKVLRL
31_1582    YLENGRDRPASHGYGLLGAYAFVYIGIAVSNSWYSRHTYKSVSIIRGGLIVSIFEKVLRL
32_4537    YLENGRDRPASHGYGLLGAYAFVYIGIAVSNSWYSRHTYKSVSIIRGGLIVSIFEKVLRL
33_9418    YLENGRDRPASHGYGLLGAYAFVYIGIAVSNSWYSRHTYKSVSIIRGGLIVSIFEKVLRL
34_1782    YLENGRDRPASHGYGLLGAYAFVYIGIAVSNSWYSRHTYKSVSIIRGGLIVSIFEKVLRL
35_10360   YLENGRDRPASHGYGLLGAYAFVYIGIAVSNSWYSRHTYKSVSIIRGGLIVSIFEKVLRL
36_468     YLENGRDRPASHGYGLLGAYAFVYIGIAVSNSWYSRHTYKSVSIIRGGLIVSIFEKVLRL
37_4956    YLENGRDRPASHGYGLLGAYAFVYIGIAVSNSWYSRHTYKSVSIIRGGLIVSIFEKVLRL
38_4305    YLENGRDRPASHGYGLLGAYAFVYIGIAVSNSWYSRHTYKSVSIIRGGLIVSIFEKVLRL
39_4737    YLENGRDRPASHGYGLLGAYAFVYIGIAVSNSWYSRHTYKSVSIIRGGLIVSIFEKVLRL
40_9543    YLENGRDRPASHGYGLLGAYAFVYIGIAVSNSWYSRHTYKSVSIIRGGLIVSIFEKVLRL
41_6669    YLENGRDRPASHGYGLLGAYAFVYIGIAVSNSWYSRHTYKSVSIIRGGLIVSIFEKVLRL
42_10558   YLENGRDRPASHGYGLLGAYAFVYIGIAVSNSWYSRHTYKSVSIIRGGLIVSIFEKVLRL
43_8051    YLENGRDRPASHGYGLLGAYAFVYIGIAVSNSWYSRHTYKSVSIIRGGLIVSIFEKVLRL
44_10454   YLENGRDRPASHGYGLLGAYAFVYIGIAVSNSWYSRHTYKSVSIIRGGLIVSIFEKVLRL
45_10126   YLENGRDRPASHGYGLLGAYAFVYIGIAVSNSWYSRHTYKSVSIIRGGLIVSIFEKVLRL
46_1547    YLENGRDRPASHGYGLLGAYAFVYIGIAVSNSWYSRHTYKSVSIIRGGLIVSIFEKVLRL
47_6749    YLENGRDRPASHGYGLLGAYAFVYIGIAVSNSWYSRHTYKSVSIIRGGLIVSIFEKVLRL
48_9982    YLENGRDRPASHGYGLLGAYAFVYIGIAVSNSWYSRHTYKSVSIIRGGLIVSIFEKVLRL
49_7293    YLENGRDRPASHGYGLLGAYAFVYIGIAVSNSWYSRHTYKSVSIIRGGLIVSIFEKVLRL
50_5993    YLENGRDRPASHGYGLLGAYAFVYIGIAVSNSWYSRHTYKSVSIIRGGLIVSIFEKVLRL
51_4791    YLENGRDRPASHGYGLLGAYAFVYIGIAVSNSWYSRHTYKSVSIIRGGLIVSIFEKVLRL
52_121     YLENGRDRPASHGYGLLGAYAFVYIGIAVSNSWYSRHTYKSVSIIRGGLIVSIFEKVLRL
53_1667    YLENGRDRPASHGYGLLGAYAFVYIGIAVSNSWYSRHTYKSVSIIRGGLIVSIFEKVLRL
54_10488   YLENGRDRPASHGYGLLGAYAFVYIGIAVSNSWYSRHTYKSVSIIRGGLIVSIFEKVLRL
55_7733    YLENGRDRPASHGYGLLGAYAFVYIGIAVSNSWYSRHTYKSVSIIRGGLIVSIFEKVLRL
56_3982    YLENGRDRPASHGYGLLGAYAFVYIGIAVSNSWYSRHTYKSVSIIRGGLIVSIFEKVLRL
57_392     YLENGRDRPASHGYGLLGAYAFVYIGIAVSNSWYSRHTYKSVSIIRGGLIVSIFEKVLRL
58_10291   YLENGRDRPASHGYGLLGAYAFVYIGIAVSNSWYSRHTYKSVSIIRGGLIVSIFEKVLRL
59_5792    YLENGRDRPASHGYGLLGAYAFVYIGIAVSNSWYSRHTYKSVSIIRGGLIVSIFEKVLRL
60_8712    YLENGRDRPASHGYGLLGAYAFVYIGIAVSNSWYSRHTYKSVSIIRGGLIVSIFEKVLRL
61_6716    YLENGRDRPASHGYGLLGAYAFVYIGIAVSNSWYSRHTYKSVSIIRGGLIVSIFEKVLRL
62_5599    YLENGRDRPASHGYGLLGAYAFVYIGIAVSNSWYSRHTYKSVSIIRGGLIVSIFEKVLRL
63_304     YLENGRDRPASHGYGLLGAYAFVYIGIAVSNSWYSRHTYKSVSIIRGGLIVSIFEKVLRL
64_10473   FTVVSQDSFFEPTSTFRQELDPSGDMSDQIIEEVLRECRAWEIVDGSGGLGGKRADANLS
65_6310    YLENGRDRPASHGYGLLGAYAFVYIGIAVSNSWYSRHTYKSVSIIRGGLIVSIFEKVLRL
66_8714    YLENGRDRPASHGYGLLGAYAFVYIGIAVSNSWYSRHTYKSVSIIRGGLIVSIFEKVLRL
67_2350    YLENGRDRPASHGYGLLGAYAFVYIGIAVSNSWYSRHTYKSVSIIRGGLIVSIFEKVLRL
68_2447    YLENGRDRPASHGYGLLGAYAFVYIGIAVSNSWYSRHTYKSVSIIRGGLIVSIFEKVLRL
69_5658    YLENGRDRPASHGYGLLGAYAFVYIGIAVSNSWYSRHTYKSVSIIRGGLIVSIFEKVLRL
70_1277    YLENGRDRPASHGYGLLGAYAFVYIGIAVSNSWYSRHTYKSVSIIRGGLIVSIFEKVLRL
71_3717    YLENGRDRPASHGYGLLGAYAFVYIGIAVSNSWYSRHTYKSVSIIRGGLIVSIFEKVLRL
72_7846    YLENGRDRPASHGYGLLGAYAFVYIGIAVSNSWYSRHTYKSVSIIRGGLIVSIFEKVLRL
73_10528   YLENGRDRPASHGYGLLGAYAFVYIGIAVSNSWYSRHTYKSVSIIRGGLIVSIFEKVLRL
74_4899    YLENGRDRPASHGYGLLGAYAFVYIGIAVSNSWYSRHTYKSVSIIRGGLIVSIFEKVLRL
75_5507    YLENGRDRPASHGYGLLGAYAFVYIGIAVSNSWYSRHTYKSVSIIRGGLIVSIFEKVLRL
76_5432    YLENGRDRPASHGYGLLGAYAFVYIGIAVSNSWYSRHTYKSVSIIRGGLIVSIFEKVLRL
77_10770   YLENGRDRPASHGYGLLGAYAFVYIGIAVSNSWYSRHTYKSVSIIRGGLIVSIFEKVLRL
78_5904    YLENGRDRPASHGYGLLGAYAFVYIGIAVSNSWYSRHTYKSVSIIRGGLIVSIFEKVLRL
79_9653    YLENGRDRPASHGYGLLGAYAFVYIGIAVSNSWYSRHTYKSVSIIRGGLIVSIFEKVLRL
80_4462    YLENGRDRPASHGYGLLGAYAFVYIGIAVSNSWYSRHTYKSVSIIRGGLIVSIFEKVLRL
81_7194    YLENGRDRPASHGYGLLGAYAFVYIGIAVSNSWYSRHTYKSVSIIRGGLIVSIFEKVLRL
82_1022    YLENGRDRPASHGYGLLGAYAFVYIGIAVSNSWYSRHTYKSVSIIRGGLIVSIFEKVLRL
83_9813    YLENGRDRPASHGYGLLGAYAFVYIGIAVSNSWYSRHTYKSVSIIRGGLIVSIFEKVLRL
84_1902    YLENGRDRPASHGYGLLGAYAFVYIGIAVSNSWYSRHTYKSVSIIRGGLIVSIFEKVLRL
85_5152    YLENGRDRPASHGYGLLGAYAFVYIGIAVSNSWYSRHTYKSVSIIRGGLIVSIFEKVLRL
86_3385    YLENGRDRPASHGYGLLGAYAFVYIGIAVSNSWYSRHTYKSVSIIRGGLIVSIFEKVLRL
87_10760   YLENGRDRPASHGYGLLGAYAFVYIGIAVSNSWYSRHTYKSVSIIRGGLIVSIFEKVLRL
88_1588    YLENGRDRPASHGYGLLGAYAFVYIGIAVSNSWYSRHTYKSVSIIRGGLIVSIFEKVLRL
89_1609    YLENGRDRPASHGYGLLGAYAFVYIGIAVSNSWYSRHTYKSVSIIRGGLIVSIFEKVLRL
90_4023    YLENGRDRPASHGYGLLGAYAFVYIGIAVSNSWYSRHTYKSVSIIRGGLIVSIFEKVLRL
```

	370 380 390 400 410 420
1_4160	GEDSSIEAKATTLMISDVQRIVGGLVYIHEVWAGVLETALATYLLQRVMGVSSVAMLGLA
2_6143	GEDSSIEAKATTLMISDVQRIVGGLVYIHEVWAGVLETALATYLLQRVMGVSSVAMLGLA
3_6554	GEDSSIEAKATTLMISDVQRIVGGLVYIHEVWAGVLETALATYLLQRVMGVSSVAMLGLA
4_111	GEDSSIEAKATTLMISDVQRIVGGLVYIHEVWAGVLETALATYLLQRVMGVSSVAMLGLA
5_5742	GEDSSIEAKATTLMISDVQRIVGGLVYIHEVWAGVLETALATYLLQRVMGVSSVAMLGLA
6_9490	GEDSSIEAKATTLMISDVQRIVGGLVYIHEVWAGVLETALATYLLQRVMGVSSVAMLGLA
7_6313	GVFLLLMIGCAITLKMPDLWVQWWSTAMKEGDTYGNSYWIGILALLEVLPLLMLCFSLLH
8_2552	GEDSSIEAKATTLMISDVQRIVGGLVYIHEVWAGVLETALATYLLQRVMGVSSVAMLGLA
9_7745	GEDSSIEAKATTLMISDVQRIVGGLVYIHEVWAGVLETALATYLLQRVMGVSSVAMLGLA
10_2315	GEDSSIEAKATTLMISDVQRIVGGLVYIHEVWAGVLETALATYLLQRVMGVSSVAMLGLA
11_2035	GEDSSIEAKATTLMISDVQRIVGGLVYIHEVWAGVLETALATYLLQRVMGVSSVAMLGLA
12_9640	GEDSSIEAKATTLMISDVQRIVGGLVYIHEVWAGVLETALATYLLQRVMGVSSVAMLGLA
13_3674	GEDSSIEAKATTLMISDVQRIVGGLVYIHEVWAGVLETALATYLLQRVMGVSSVAMLGLA
14_6548	GEDSSIEAKATTLMISDVQRIVGGLVYIHEVWAGVLETALATYLLQRVMGVSSVAMLGLA
15_10828	GEDSSIEAKATTLMISDVQRIVGGLVYIHEVWAGVLETALATYLLQRVMGVSSVAMLGLA
16_6578	GEDSSIEAKATTLMISDVQRIVGGLVYIHEVWAGVLETALATYLLQRVMGVSSVAMLGLA
17_10233	GEDSSIEAKATTLMISDVQRIVGGLVYIHEVWAGVLETALATYLLQRVMGVSSVAMLGLA
18_9261	GEDSSIEAKATTLMISDVQRIVGGLVYIHEVWAGVLETALATYLLQRVMGVSSVAMLGLA
19_8450	GEDSSIEAKATTLMISDVQRIVGGLVYIHEVWAGVLETALATYLLQRVMGVSSVAMLGLA
20_1727	GEDSSIEAKATTLMISDVQRIVGGLVYIHEVWAGVLETALATYLLQRVMGVSSVAMLGLA
21_206	GEDSSIEAKATTLMISDVQRIVGGLVYIHEVWAGVLETALATYLLQRVMGVSSVAMLGLA
22_7439	GEDSSIEAKATTLMISDVQRIVGGLVYIHEVWAGVLETALATYLLQRVMGVSSVAMLGLA
23_3780	GEDSSIEAKATTLMISDVQRIVGGLVYIHEVWAGVLETALATYLLQRVMGVSSVAMLGLA
24_967	GEDSSIEAKATTLMISDVQRIVGGLVYIHEVWAGVLETALATYLLQRVMGVSSVAMLGLA
25_6595	GEDSSIEAKATTLMISDVQRIVGGLVYIHEVWAGVLETALATYLLQRVMGVSSVAMLGLA
26_8391	GEDSSIEAKATTLMISDVQRIVGGLVYIHEVWAGVLETALATYLLQRVMGVSSVAMLGLA
27_8170	GEDSSIEAKATTLMISDVQRIVGGLVYIHEVWAGVLETALATYLLQRVMGVSSVAMLGLA
28_5209	GEDSSIEAKATTLMISDVQRIVGGLVYIHEVWAGVLETALATYLLQRVMGVSSVAMLGLA
29_2181	GEDSSIEAKATTLMISDVQRIVGGLVYIHEVWAGVLETALATYLLQRVMGVSSVAMLGLA
30_8564	GEDSSIEAKATTLMISDVQRIVGGLVYIHEVWAGVLETALATYLLQRVMGVSSVAMLGLA
31_1582	GEDSSIEAKATTLMISDVQRIVGGLVYIHEVWAGVLETALATYLLQRVMGVSSVAMLGLA
32_4537	GEDSSIEAKATTLMISDVQRIVGGLVYIHEVWAGVLETALATYLLQRVMGVSSVAMLGLA
33_9418	GEDSSIEAKATTLMISDVQRIVGGLVYIHEVWAGVLETALATYLLQRVMGVSSVAMLGLA
34_1782	GEDSSIEAKATTLMISDVQRIVGGLVYIHEVWAGVLETALATYLLQRVMGVSSVAMLGLA
35_10360	GEDSSIEAKATTLMISDVQRIVGGLVYIHEVWAGVLETALATYLLQRVMGVSSVAMLGLA
36_468	GEDSSIEAKATTLMISDVQRIVGGLVYIHEVWAGVLETALATYLLQRVMGVSSVAMLGLA
37_4956	GEDSSIEAKATTLMISDVQRIVGGLVYIHEVWAGVLETALATYLLQRVMGVSSVAMLGLA
38_4305	GEDSSIEAKATTLMISDVQRIVGGLVYIHEVWAGVLETALATYLLQRVMGVSSVAMLGLA
39_4737	GEDSSIEAKATTLMISDVQRIVGGLVYIHEVWAGVLETALATYLLQRVMGVSSVAMLGLA
40_9543	GEDSSIEAKATTLMISDVQRIVGGLVYIHEVWAGVLETALATYLLQRVMGVSSVAMLGLA
41_6669	GEDSSIEAKATTLMISDVQRIVGGLVYIHEVWAGVLETALATYLLQRVMGVSSVAMLGLA
42_10558	GEDSSIEAKATTLMISDVQRIVGGLVYIHEVWAGVLETALATYLLQRVMGVSSVAMLGLA
43_8051	GEDSSIEAKATTLMISDVQRIVGGLVYIHEVWAGVLETALATYLLQRVMGVSSVAMLGLA
44_10454	GEDSSIEAKATTLMISDVQRIVGGLVYIHEVWAGVLETALATYLLQRVMGVSSVAMLGLA
45_10126	GEDSSIEAKATTLMISDVQRIVGGLVYIHEVWAGVLETALATYLLQRVMGVSSVAMLGLA
46_1547	GEDSSIEAKATTLMISDVQRIVGGLVYIHEVWAGVLETALATYLLQRVMGVSSVAMLGLA
47_6749	GEDSSIEAKATTLMISDVQRIVGGLVYIHEVWAGVLETALATYLLQRVMGVSSVAMLGLA
48_9982	GEDSSIEAKATTLMISDVQRIVGGLVYIHEVWAGVLETALATYLLQRVMGVSSVAMLGLA
49_7293	GEDSSIEAKATTLMISDVQRIVGGLVYIHEVWAGVLETALATYLLQRVMGVSSVAMLGLA
50_5993	GEDSSIEAKATTLMISDVQRIVGGLVYIHEVWAGVLETALATYLLQRVMGVSSVAMLGLA
51_4791	GEDSSIEAKATTLMISDVQRIVGGLVYIHEVWAGVLETALATYLLQRVMGVSSVAMLGLA
52_121	GEDSSIEAKATTLMISDVQRIVGGLVYIHEVWAGVLETALATYLLQRVMGVSSVAMLGLA
53_1667	GEDSSIEAKATTLMISDVQRIVGGLVYIHEVWAGVLETALATYLLQRVMGVSSVAMLGLA
54_10488	GEDSSIEAKATTLMISDVQRIVGGLVYIHEVWAGVLETALATYLLQRVMGVSSVAMLGLA
55_7733	GEDSSIEAKATTLMISDVQRIVGGLVYIHEVWAGVLETALATYLLQRVMGVSSVAMLGLA
56_3982	GEDSSIEAKATTLMISDVQRIVGGLVYIHEVWAGVLETALATYLLQRVMGVSSVAMLGLA
57_392	GEDSSIEAKATTLMISDVQRIVGGLVYIHEVWAGVLETALATYLLQRVMGVSSVAMLGLA
58_10291	GEDSSIEAKATTLMISDVQRIVGGLVYIHEVWAGVLETALATYLLQRVMGVSSVAMLGLA
59_5792	GEDSSIEAKATTLMISDVQRIVGGLVYIHEVWAGVLETALATYLLQRVMGVSSVAMLGLA
60_8712	GEDSSIEAKATTLMISDVQRIVGGLVYIHEVWAGVLETALATYLLQRVMGVSSVAMLGLA
61_6716	GEDSSIEAKATTLMISDVQRIVGGLVYIHEVWAGVLETALATYLLQRVMGVSSVAMLGLA
62_5599	GEDSSIEAKATTLMISDVQRIVGGLVYIHEVWAGVLETALATYLLQRVMGVSSVAMLGLA
63_304	GEDSSIEAKATTLMISDVQRIVGGLVYIHEVWAGVLETALATYLLQRVMGVSSVAMLGLA
64_10473	AGEVQLLAIARLVLQWQSQPAGSGGIILLDEATSNLDRQTEVLVESIMAARLQHATVVSV
65_6310	GEDSSIEAKATTLMISDVQRIVGGLVYIHEVWAGVLETALATYLLQRVMGVSSVAMLGLA
66_8714	GEDSSIEAKATTLMISDVQRIVGGLVYIHEVWAGVLETALATYLLQRVMGVSSVAMLGLA
67_2350	GEDSSIEAKATTLMISDVQRIVGGLVYIHEVWAGVLETALATYLLQRVMGVSSVAMLGLA
68_2447	GEDSSIEAKATTLMISDVQRIVGGLVYIHEVWAGVLETALATYLLQRVMGVSSVAMLGLA
69_5658	GEDSSIEAKATTLMISDVQRIVGGLVYIHEVWAGVLETALATYLLQRVMGVSSVAMLGLA
70_1277	GEDSSIEAKATTLMISDVQRIVGGLVYIHEVWAGVLETALATYLLQRVMGVSSVAMLGLA
71_3717	GEDSSIEAKATTLMISDVQRIVGGLVYIHEVWAGVLETALATYLLQRVMGVSSVAMLGLA
72_7846	GEDSSIEAKATTLMISDVQRIVGGLVYIHEVWAGVLETALATYLLQRVMGVSSVAMLGLA
73_10528	GEDSSIEAKATTLMISDVQRIVGGLVYIHEVWAGVLETALATYLLQRVMGVSSVAMLGLA
74_4899	GEDSSIEAKATTLMISDVQRIVGGLVYIHEVWAGVLETALATYLLQRVMGVSSVAMLGLA
75_5507	GEDSSIEAKATTLMISDVQRIVGGLVYIHEVWAGVLETALATYLLQRVMGVSSVAMLGLA
76_5432	GEDSSIEAKATTLMISDVQRIVGGLVYIHEVWAGVLETALATYLLQRVMGVSSVAMLGLA
77_10770	GEDSSIEAKATTLMISDVQRIVGGLVYIHEVWAGVLETALATYLLQRVMGVSSVAMLGLA
78_5904	GEDSSIEAKATTLMISDVQRIVGGLVYIHEVWAGVLETALATYLLQRVMGVSSVAMLGLA
79_9653	GEDSSIEAKATTLMISDVQRIVGGLVYIHEVWAGVLETALATYLLQRVMGVSSVAMLGLA
80_4462	GEDSSIEAKATTLMISDVQRIVGGLVYIHEVWAGVLETALATYLLQRVMGVSSVAMLGLA
81_7194	GEDSSIEAKATTLMISDVQRIVGGLVYIHEVWAGVLETALATYLLQRVMGVSSVAMLGLA
82_1022	GEDSSIEAKATTLMISDVQRIVGGLVYIHEVWAGVLETALATYLLQRVMGVSSVAMLGLA
83_9813	GEDSSIEAKATTLMISDVQRIVGGLVYIHEVWAGVLETALATYLLQRVMGVSSVAMLGLA
84_1902	GEDSSIEAKATTLMISDVQRIVGGLVYIHEVWAGVLETALATYLLQRVMGVSSVAMLGLA
85_5152	GEDSSIEAKATTLMISDVQRIVGGLVYIHEVWAGVLETALATYLLQRVMGVSSVAMLGLA
86_3385	GEDSSIEAKATTLMISDVQRIVGGLVYIHEVWAGVLETALATYLLQRVMGVSSVAMLGLA
87_10760	GEDSSIEAKATTLMISDVQRIVGGLVYIHEVWAGVLETALATYLLQRVMGVSSVAMLGLA
88_1588	GEDSSIEAKATTLMISDVQRIVGGLVYIHEVWAGVLETALATYLLQRVMGVSSVAMLGLA
89_1609	GEDSSIEAKATTLMISDVQRIVGGLVYIHEVWAGVLETALATYLLQRVMGVSSVAMLGLA
90_4023	GEDSSIEAKATTLMISDVQRIVGGLVYIHEVWAGVLETALATYLLQRVMGVSSVAMLGLA

```
                430       440       450       460       470       480
1_4160     LSCGVGAYFVAGNMSLQQGKWLKAMEQRIDATKRLCDSLKAVKMRGAETRVSRVVNELRR
2_6143     LSCGVGAYFVAGNMSLQQGKWLKAMEQRIDATKRLCDSLKAVKMRGAETRVSRVVNELRR
3_6554     LSCGVGAYFVAGNMSLQQGKWLKAMEQRIDATKRLCDSLKAVKMRGAETRVSRVVNELRR
4_111      LSCGVGAYFVAGNMSLQQGKWLKAMEQRIDATKRLCDSLKAVKMRGAETRVSRVVNELRR
5_5742     LSCGVGAYFVAGNMSLQQGKWLKAMEQRIDATKRLCDSLKAVKMRGAETRVSRVVNELRR
6_9490     LSCGVGAYFVAGNMSLQQGKWLKAMEQRIDATKRLCDSLKAVKMRGAETRVSRVVNELRR
7_6313     LMFWVVPQSASALHASLLRTVLLAPFGFISRVDTGSLLNRFNQDLMFVDTRLPLDLFNTC
8_2552     LSCGVGAYFVAGNMSLQQGKWLKAMEQRIDATKRLCDSLKAVKMRGAETRVSRVVNELRR
9_7745     LSCGVGAYFVAGNMSLQQGKWLKAMEQRIDATKRLCDSLKAVKMRGAETRVSRVVNELRR
10_2315    LSCGVGAYFVAGNMSLQQGKWLKAMEQRIDATKRLCDSLKAVKMRGAETRVSRVVNELRR
11_2035    LSCGVGAYFVAGNMSLQQGKWLKAMEQRIDATKRLCDSLKAVKMRGAETRVSRVVNELRR
12_9640    LSCGVGAYFVAGNMSLQQGKWLKAMEQRIDATKRLCDSLKAVKMRGAETRVSRVVNELRR
13_3674    LSCGVGAYFVAGNMSLQQGKWLKAMEQRIDATKRLCDSLKAVKMRGAETRVSRVVNELRR
14_6548    LSCGVGAYFVAGNMSLQQGKWLKAMEQRIDATKRLCDSLKAVKMRGAETRVSRVVNELRR
15_10828   LSCGVGAYFVAGNMSLQQGKWLKAMEQRIDATKRLCDSLKAVKMRGAETRVSRVVNELRR
16_6578    LSCGVGAYFVAGNMSLQQGKWLKAMEQRIDATKRLCDSLKAVKMRGAETRVSRVVNELRR
17_10233   LSCGVGAYFVAGNMSLQQGKWLKAMEQRIDATKRLCDSLKAVKMRGAETRVSRVVNELRR
18_9261    LSCGVGAYFVAGNMSLQQGKWLKAMEQRIDATKRLCDSLKAVKMRGAETRVSRVVNELRR
19_8450    LSCGVGAYFVAGNMSLQQGKWLKAMEQRIDATKRLCDSLKAVKMRGAETRVSRVVNELRR
20_1727    LSCGVGAYFVAGNMSLQQGKWLKAMEQRIDATKRLCDSLKAVKMRGAETRVSRVVNELRR
21_206     LSCGVGAYFVAGNMSLQQGKWLKAMEQRIDATKRLCDSLKAVKMRGAETRVSRVVNELRR
22_7439    LSCGVGAYFVAGNMSLQQGKWLKAMEQRIDATKRLCDSLKAVKMRGAETRVSRVVNELRR
23_3780    LSCGVGAYFVAGNMSLQQGKWLKAMEQRIDATKRLCDSLKAVKMRGAETRVSRVVNELRR
24_967     LSCGVGAYFVAGNMSLQQGKWLKAMEQRIDATKRLCDSLKAVKMRGAETRVSRVVNELRR
25_6595    LSCGVGAYFVAGNMSLQQGKWLKAMEQRIDATKRLCDSLKAVKMRGAETRVSRVVNELRR
26_8391    LSCGVGAYFVAGNMSLQQGKWLKAMEQRIDATKRLCDSLKAVKMRGAETRVSRVVNELRR
27_8170    LSCGVGAYFVAGNMSLQQGKWLKAMEQRIDATKRLCDSLKAVKMRGAETRVSRVVNELRR
28_5209    LSCGVGAYFVAGNMSLQQGKWLKAMEQRIDATKRLCDSLKAVKMRGAETRVSRVVNELRR
29_2181    LSCGVGAYFVAGNMSLQQGKWLKAMEQRIDATKRLCDSLKAVKMRGAETRVSRVVNELRR
30_8564    LSCGVGAYFVAGNMSLQQGKWLKAMEQRIDATKRLCDSLKAVKMRGAETRVSRVVNELRR
31_1582    LSCGVGAYFVAGNMSLQQGKWLKAMEQRIDATKRLCDSLKAVKMRGAETRVSRVVNELRR
32_4537    LSCGVGAYFVAGNMSLQQGKWLKAMEQRIDATKRLCDSLKAVKMRGAETRVSRVVNELRR
33_9418    LSCGVGAYFVAGNMSLQQGKWLKAMEQRIDATKRLCDSLKAVKMRGAETRVSRVVNELRR
34_1782    LSCGVGAYFVAGNMSLQQGKWLKAMEQRIDATKRLCDSLKAVKMRGAETRVSRVVNELRR
35_10360   LSCGVGAYFVAGNMSLQQGKWLKAMEQRIDATKRLCDSLKAVKMRGAETRVSRVVNELRR
36_468     LSCGVGAYFVAGNMSLQQGKWLKAMEQRIDATKRLCDSLKAVKMRGAETRVSRVVNELRR
37_4956    LSCGVGAYFVAGNMSLQQGKWLKAMEQRIDATKRLCDSLKAVKMRGAETRVSRVVNELRR
38_4305    LSCGVGAYFVAGNMSLQQGKWLKAMEQRIDATKRLCDSLKAVKMRGAETRVSRVVNELRR
39_4737    LSCGVGAYFVAGNMSLQQGKWLKAMEQRIDATKRLCDSLKAVKMRGAETRVSRVVNELRR
40_9543    LSCGVGAYFVAGNMSLQQGKWLKAMEQRIDATKRLCDSLKAVKMRGAETRVSRVVNELRR
41_6669    LSCGVGAYFVAGNMSLQQGKWLKAMEQRIDATKRLCDSLKAVKMRGAETRVSRVVNELRR
42_10558   LSCGVGAYFVAGNMSLQQGKWLKAMEQRIDATKRLCDSLKAVKMRGAETRVSRVVNELRR
43_8051    LSCGVGAYFVAGNMSLQQGKWLKAMEQRIDATKRLCDSLKAVKMRGAETRVSRVVNELRR
44_10454   LSCGVGAYFVAGNMSLQQGKWLKAMEQRIDATKRLCDSLKAVKMRGAETRVSRVVNELRR
45_10126   LSCGVGAYFVAGNMSLQQGKWLKAMEQRIDATKRLCDSLKAVKMRGAETRVSRVVNELRR
46_1547    LSCGVGAYFVAGNMSLQQGKWLKAMEQRIDATKRLCDSLKAVKMRGAETRVSRVVNELRR
47_6749    LSCGVGAYFVAGNMSLQQGKWLKAMEQRIDATKRLCDSLKAVKMRGAETRVSRVVNELRR
48_9982    LSCGVGAYFVAGNMSLQQGKWLKAMEQRIDATKRLCDSLKAVKMRGAETRVSRVVNELRR
49_7293    LSCGVGAYFVAGNMSLQQGKWLKAMEQRIDATKRLCDSLKAVKMRGAETRVSRVVNELRR
50_5993    LSCGVGAYFVAGNMSLQQGKWLKAMEQRIDATKRLCDSLKAVKMRGAETRVSRVVNELRR
51_4791    LSCGVGAYFVAGNMSLQQGKWLKAMEQRIDATKRLCDSLKAVKMRGAETRVSRVVNELRR
52_121     LSCGVGAYFVAGNMSLQQGKWLKAMEQRIDATKRLCDSLKAVKMRGAETRVSRVVNELRR
53_1667    LSCGVGAYFVAGNMSLQQGKWLKAMEQRIDATKRLCDSLKAVKMRGAETRVSRVVNELRR
54_10488   LSCGVGAYFVAGNMSLQQGKWLKAMEQRIDATKRLCDSLKAVKMRGAETRVSRVVNELRR
55_7733    LSCGVGAYFVAGNMSLQQGKWLKAMEQRIDATKRLCDSLKAVKMRGAETRVSRVVNELRR
56_3982    LSCGVGAYFVAGNMSLQQGKWLKAMEQRIDATKRLCDSLKAVKMRGAETRVSRVVNELRR
57_392     LSCGVGAYFVAGNMSLQQGKWLKAMEQRIDATKRLCDSLKAVKMRGAETRVSRVVNELRR
58_10291   LSCGVGAYFVAGNMSLQQGKWLKAMEQRIDATKRLCDSLKAVKMRGAETRVSRVVNELRR
59_5792    LSCGVGAYFVAGNMSLQQGKWLKAMEQRIDATKRLCDSLKAVKMRGAETRVSRVVNELRR
60_8712    LSCGVGAYFVAGNMSLQQGKWLKAMEQRIDATKRLCDSLKAVKMRGAETRVSRVVNELRR
61_6716    LSCGVGAYFVAGNMSLQQGKWLKAMEQRIDATKRLCDSLKAVKMRGAETRVSRVVNELRR
62_5599    LSCGVGAYFVAGNMSLQQGKWLKAMEQRIDATKRLCDSLKAVKMRGAETRVSRVVNELRR
63_304     LSCGVGAYFVAGNMSLQQGKWLKAMEQRIDATKRLCDSLKAVKMRGAETRVSRVVNELRR
64_10473   MHRLEAVAAYDKVAVLDKGVLVDFGPVTDVMARCELFTG.....................
65_6310    LSCGVGAYFVAGNMSLQQGKWLKAMEQRIDATKRLCDSLKAVKMRGAETRVSRVVNELRR
66_8714    LSCGVGAYFVAGNMSLQQGKWLKAMEQRIDATKRLCDSLKAVKMRGAETRVSRVVNELRR
67_2350    LSCGVGAYFVAGNMSLQQGKWLKAMEQRIDATKRLCDSLKAVKMRGAETRVSRVVNELRR
68_2447    LSCGVGAYFVAGNMSLQQGKWLKAMEQRIDATKRLCDSLKAVKMRGAETRVSRVVNELRR
69_5658    LSCGVGAYFVAGNMSLQQGKWLKAMEQRIDATKRLCDSLKAVKMRGAETRVSRVVNELRR
70_1277    LSCGVGAYFVAGNMSLQQGKWLKAMEQRIDATKRLCDSLKAVKMRGAETRVSRVVNELRR
71_3717    LSCGVGAYFVAGNMSLQQGKWLKAMEQRIDATKRLCDSLKAVKMRGAETRVSRVVNELRR
72_7846    LSCGVGAYFVAGNMSLQQGKWLKAMEQRIDATKRLCDSLKAVKMRGAETRVSRVVNELRR
73_10528   LSCGVGAYFVAGNMSLQQGKWLKAMEQRIDATKRLCDSLKAVKMRGAETRVSRVVNELRR
74_4899    LSCGVGAYFVAGNMSLQQGKWLKAMEQRIDATKRLCDSLKAVKMRGAETRVSRVVNELRR
75_5507    LSCGVGAYFVAGNMSLQQGKWLKAMEQRIDATKRLCDSLKAVKMRGAETRVSRVVNELRR
76_5432    LSCGVGAYFVAGNMSLQQGKWLKAMEQRIDATKRLCDSLKAVKMRGAETRVSRVVNELRR
77_10770   LSCGVGAYFVAGNMSLQQGKWLKAMEQRIDATKRLCDSLKAVKMRGAETRVSRVVNELRR
78_5904    LSCGVGAYFVAGNMSLQQGKWLKAMEQRIDATKRLCDSLKAVKMRGAETRVSRVVNELRR
79_9653    LSCGVGAYFVAGNMSLQQGKWLKAMEQRIDATKRLCDSLKAVKMRGAETRVSRVVNELRR
80_4462    LSCGVGAYFVAGNMSLQQGKWLKAMEQRIDATKRLCDSLKAVKMRGAETRVSRVVNELRR
81_7194    LSCGVGAYFVAGNMSLQQGKWLKAMEQRIDATKRLCDSLKAVKMRGAETRVSRVVNELRR
82_1022    LSCGVGAYFVAGNMSLQQGKWLKAMEQRIDATKRLCDSLKAVKMRGAETRVSRVVNELRR
83_9813    LSCGVGAYFVAGNMSLQQGKWLKAMEQRIDATKRLCDSLKAVKMRGAETRVSRVVNELRR
84_1902    LSCGVGAYFVAGNMSLQQGKWLKAMEQRIDATKRLCDSLKAVKMRGAETRVSRVVNELRR
85_5152    LSCGVGAYFVAGNMSLQQGKWLKAMEQRIDATKRLCDSLKAVKMRGAETRVSRVVNELRR
86_3385    LSCGVGAYFVAGNMSLQQGKWLKAMEQRIDATKRLCDSLKAVKMRGAETRVSRVVNELRR
87_10760   LSCGVGAYFVAGNMSLQQGKWLKAMEQRIDATKRLCDSLKAVKMRGAETRVSRVVNELRR
88_1588    LSCGVGAYFVAGNMSLQQGKWLKAMEQRIDATKRLCDSLKAVKMRGAETRVSRVVNELRR
89_1609    LSCGVGAYFVAGNMSLQQGKWLKAMEQRIDATKRLCDSLKAVKMRGAETRVSRVVNELRR
90_4023    LSCGVGAYFVAGNMSLQQGKWLKAMEQRIDATKRLCDSLKAVKMRGAETRVSRVVNELRR
```

```
                       490       500       510       520       530       540
                         .         .         .         .         .         .
1_4160    LEIQAARPFRALITASVILSYSTMTLSPLLVFAAYIGVNGNDDNLDSATMFSSLVLIALL
2_6143    LEIQAARPFRALITASVILSYSTMTLSPLLVFAAYIGVNGNDDNLDSATMFSSLVLIALL
3_6554    LEIQAARPFRALITASVILSYSTMTLSPLLVFAAYIGVNGNDDNLDSATMFSSLVLIALL
4_111     LEIQAARPFRALITASVILSYSTMTLSPLLVFAAYIGVNGNDDNLDSATMFSSLVLIALL
5_5742    LEIQAARPFRALITASVILSYSTMTLSPLLVFAAYIGVNGNDDNLDSATMFSSLVLIALL
6_9490    LEIQAARPFRALITASVILSYSTMTLSPLLVFAAYIGVNGNDDNLDSATMFSSLVLIALL
7_6313    SEMFVSIFQLVLVVVVSKEALAILPVVFGALYLIQKVYLRSSKQLRLLDLGWKADLHTKF
8_2552    LEIQAARPFRALITASVILSYSTMTLSPLLVFAAYIGVNGNDDNLDSATMFSSLVLIALL
9_7745    LEIQAARPFRALITASVILSYSTMTLSPLLVFAAYIGVNGNDDNLDSATMFSSLVLIALL
10_2315   LEIQAARPFRALITASVILSYSTMTLSPLLVFAAYIGVNGNDDNLDSATMFSSLVLIALL
11_2035   LEIQAARPFRALITASVILSYSTMTLSPLLVFAAYIGVNGNDDNLDSATMFSSLVLIALL
12_9640   LEIQAARPFRALITASVILSYSTMTLSPLLVFAAYIGVNGNDDNLDSATMFSSLVLIALL
13_3674   LEIQAARPFRALITASVILSYSTMTLSPLLVFAAYIGVNGNDDNLDSATMFSSLVLIALL
14_6548   LEIQAARPFRALITASVILSYSTMTLSPLLVFAAYIGVNGNDDNLDSATMFSSLVLIALL
15_10828  LEIQAARPFRALITASVILSYSTMTLSPLLVFAAYIGVNGNDDNLDSATMFSSLVLIALL
16_6578   LEIQAARPFRALITASVILSYSTMTLSPLLVFAAYIGVNGNDDNLDSATMFSSLVLIALL
17_10233  LEIQAARPFRALITASVILSYSTMTLSPLLVFAAYIGVNGNDDNLDSATMFSSLVLIALL
18_9261   LEIQAARPFRALITASVILSYSTMTLSPLLVFAAYIGVNGNDDNLDSATMFSSLVLIALL
19_8450   LEIQAARPFRALITASVILSYSTMTLSPLLVFAAYIGVNGNDDNLDSATMFSSLVLIALL
20_1727   LEIQAARPFRALITASVILSYSTMTLSPLLVFAAYIGVNGNDDNLDSATMFSSLVLIALL
21_206    LEIQAARPFRALITASVILSYSTMTLSPLLVFAAYIGVNGNDDNLDSATMFSSLVLIALL
22_7439   LEIQAARPFRALITASVILSYSTMTLSPLLVFAAYIGVNGNDDNLDSATMFSSLVLIALL
23_3780   LEIQAARPFRALITASVILSYSTMTLSPLLVFAAYIGVNGNDDNLDSATMFSSLVLIALL
24_967    LEIQAARPFRALITASVILSYSTMTLSPLLVFAAYIGVNGNDDNLDSATMFSSLVLIALL
25_6595   LEIQAARPFRALITASVILSYSTMTLSPLLVFAAYIGVNGNDDNLDSATMFSSLVLIALL
26_8391   LEIQAARPFRALITASVILSYSTMTLSPLLVFAAYIGVNGNDDNLDSATMFSSLVLIALL
27_8170   LEIQAARPFRALITASVILSYSTMTLSPLLVFAAYIGVNGNDDNLDSATMFSSLVLIALL
28_5209   LEIQAARPFRALITASVILSYSTMTLSPLLVFAAYIGVNGNDDNLDSATMFSSLVLIALL
29_2181   LEIQAARPFRALITASVILSYSTMTLSPLLVFAAYIGVNGNDDNLDSATMFSSLVLIALL
30_8564   LEIQAARPFRALITASVILSYSTMTLSPLLVFAAYIGVNGNDDNLDSATMFSSLVLIALL
31_1582   LEIQAARPFRALITASVILSYSTMTLSPLLVFAAYIGVNGNDDNLDSATMFSSLVLIALL
32_4537   LEIQAARPFRALITASVILSYSTMTLSPLLVFAAYIGVNGNDDNLDSATMFSSLVLIALL
33_9418   LEIQAARPFRALITASVILSYSTMTLSPLLVFAAYIGVNGNDDNLDSATMFSSLVLIALL
34_1782   LEIQAARPFRALITASVILSYSTMTLSPLLVFAAYIGVNGNDDNLDSATMFSSLVLIALL
35_10360  LEIQAARPFRALITASVILSYSTMTLSPLLVFAAYIGVNGNDDNLDSATMFSSLVLIALL
36_468    LEIQAARPFRALITASVILSYSTMTLSPLLVFAAYIGVNGNDDNLDSATMFSSLVLIALL
37_4956   LEIQAARPFRALITASVILSYSTMTLSPLLVFAAYIGVNGNDDNLDSATMFSSLVLIALL
38_4305   LEIQAARPFRALITASVILSYSTMTLSPLLVFAAYIGVNGNDDNLDSATMFSSLVLIALL
39_4737   LEIQAARPFRALITASVILSYSTMTLSPLLVFAAYIGVNGNDDNLDSATMFSSLVLIALL
40_9543   LEIQAARPFRALITASVILSYSTMTLSPLLVFAAYIGVNGNDDNLDSATMFSSLVLIALL
41_6669   LEIQAARPFRALITASVILSYSTMTLSPLLVFAAYIGVNGNDDNLDSATMFSSLVLIALL
42_10558  LEIQAARPFRALITASVILSYSTMTLSPLLVFAAYIGVNGNDDNLDSATMFSSLVLIALL
43_8051   LEIQAARPFRALITASVILSYSTMTLSPLLVFAAYIGVNGNDDNLDSATMFSSLVLIALL
44_10454  LEIQAARPFRALITASVILSYSTMTLSPLLVFAAYIGVNGNDDNLDSATMFSSLVLIALL
45_10126  LEIQAARPFRALITASVILSYSTMTLSPLLVFAAYIGVNGNDDNLDSATMFSSLVLIALL
46_1547   LEIQAARPFRALITASVILSYSTMTLSPLLVFAAYIGVNGNDDNLDSATMFSSLVLIALL
47_6749   LEIQAARPFRALITASVILSYSTMTLSPLLVFAAYIGVNGNDDNLDSATMFSSLVLIALL
48_9982   LEIQAARPFRALITASVILSYSTMTLSPLLVFAAYIGVNGNDDNLDSATMFSSLVLIALL
49_7293   LEIQAARPFRALITASVILSYSTMTLSPLLVFAAYIGVNGNDDNLDSATMFSSLVLIALL
50_5993   LEIQAARPFRALITASVILSYSTMTLSPLLVFAAYIGVNGNDDNLDSATMFSSLVLIALL
51_4791   LEIQAARPFRALITASVILSYSTMTLSPLLVFAAYIGVNGNDDNLDSATMFSSLVLIALL
52_121    LEIQAARPFRALITASVILSYSTMTLSPLLVFAAYIGVNGNDDNLDSATMFSSLVLIALL
53_1667   LEIQAARPFRALITASVILSYSTMTLSPLLVFAAYIGVNGNDDNLDSATMFSSLVLIALL
54_10488  LEIQAARPFRALITASVILSYSTMTLSPLLVFAAYIGVNGNDDNLDSATMFSSLVLIALL
55_7733   LEIQAARPFRALITASVILSYSTMTLSPLLVFAAYIGVNGNDDNLDSATMFSSLVLIALL
56_3982   LEIQAARPFRALITASVILSYSTMTLSPLLVFAAYIGVNGNDDNLDSATMFSSLVLIALL
57_392    LEIQAARPFRALITASVILSYSTMTLSPLLVFAAYIGVNGNDDNLDSATMFSSLVLIALL
58_10291  LEIQAARPFRALITASVILSYSTMTLSPLLVFAAYIGVNGNDDNLDSATMFSSLVLIALL
59_5792   LEIQAARPFRALITASVILSYSTMTLSPLLVFAAYIGVNGNDDNLDSATMFSSLVLIALL
60_8712   LEIQAARPFRALITASVILSYSTMTLSPLLVFAAYIGVNGNDDNLDSATMFSSLVLIALL
61_6716   LEIQAARPFRALITASVILSYSTMTLSPLLVFAAYIGVNGNDDNLDSATMFSSLVLIALL
62_5599   LEIQAARPFRALITASVILSYSTMTLSPLLVFAAYIGVNGNDDNLDSATMFSSLVLIALL
63_304    LEIQAARPFRALITASVILSYSTMTLSPLLVFAAYIGVNGNDDNLDSATMFSSLVLIALL
64_10473  ............................................................
65_6310   LEIQAARPFRALITASVILSYSTMTLSPLLVFAAYIGVNGNDDNLDSATMFSSLVLIALL
66_8714   LEIQAARPFRALITASVILSYSTMTLSPLLVFAAYIGVNGNDDNLDSATMFSSLVLIALL
67_2350   LEIQAARPFRALITASVILSYSTMTLSPLLVFAAYIGVNGNDDNLDSATMFSSLVLIALL
68_2447   LEIQAARPFRALITASVILSYSTMTLSPLLVFAAYIGVNGNDDNLDSATMFSSLVLIALL
69_5658   LEIQAARPFRALITASVILSYSTMTLSPLLVFAAYIGVNGNDDNLDSATMFSSLVLIALL
70_1277   LEIQAARPFRALITASVILSYSTMTLSPLLVFAAYIGVNGNDDNLDSATMFSSLVLIALL
71_3717   LEIQAARPFRALITASVILSYSTMTLSPLLVFAAYIGVNGNDDNLDSATMFSSLVLIALL
72_7846   LEIQAARPFRALITASVILSYSTMTLSPLLVFAAYIGVNGNDDNLDSATMFSSLVLIALL
73_10528  LEIQAARPFRALITASVILSYSTMTLSPLLVFAAYIGVNGNDDNLDSATMFSSLVLIALL
74_4899   LEIQAARPFRALITASVILSYSTMTLSPLLVFAAYIGVNGNDDNLDSATMFSSLVLIALL
75_5507   LEIQAARPFRALITASVILSYSTMTLSPLLVFAAYIGVNGNDDNLDSATMFSSLVLIALL
76_5432   LEIQAARPFRALITASVILSYSTMTLSPLLVFAAYIGVNGNDDNLDSATMFSSLVLIALL
77_10770  LEIQAARPFRALITASVILSYSTMTLSPLLVFAAYIGVNGNDDNLDSATMFSSLVLIALL
78_5904   LEIQAARPFRALITASVILSYSTMTLSPLLVFAAYIGVNGNDDNLDSATMFSSLVLIALL
79_9653   LEIQAARPFRALITASVILSYSTMTLSPLLVFAAYIGVNGNDDNLDSATMFSSLVLIALL
80_4462   LEIQAARPFRALITASVILSYSTMTLSPLLVFAAYIGVNGNDDNLDSATMFSSLVLIALL
81_7194   LEIQAARPFRALITASVILSYSTMTLSPLLVFAAYIGVNGNDDNLDSATMFSSLVLIALL
82_1022   LEIQAARPFRALITASVILSYSTMTLSPLLVFAAYIGVNGNDDNLDSATMFSSLVLIALL
83_9813   LEIQAARPFRALITASVILSYSTMTLSPLLVFAAYIGVNGNDDNLDSATMFSSLVLIALL
84_1902   LEIQAARPFRALITASVILSYSTMTLSPLLVFAAYIGVNGNDDNLDSATMFSSLVLIALL
85_5152   LEIQAARPFRALITASVILSYSTMTLSPLLVFAAYIGVNGNDDNLDSATMFSSLVLIALL
86_3385   LEIQAARPFRALITASVILSYSTMTLSPLLVFAAYIGVNGNDDNLDSATMFSSLVLIALL
87_10760  LEIQAARPFRALITASVILSYSTMTLSPLLVFAAYIGVNGNDDNLDSATMFSSLVLIALL
88_1588   LEIQAARPFRALITASVILSYSTMTLSPLLVFAAYIGVNGNDDNLDSATMFSSLVLIALL
89_1609   LEIQAARPFRALITASVILSYSTMTLSPLLVFAAYIGVNGNDDNLDSATMFSSLVLIALL
90_4023   LEIQAARPFRALITASVILSYSTMTLSPLLVFAAYIGVNGNDDNLDSATMFSSLVLIALL
```

```
                550       560       570       580       590       600
1_4160     GSPLVHLFQAMPALGSAHGCFERILAFLKTPEKPLTIKNEPETMPESNRGTKDASIQRSE
2_6143     GSPLVHLFQAMPALGSAHGCFERILAFLKTPEKPLTIKNEPETMPESNRGTKDASIQRSE
3_6554     GSPLVHLFQAMPALGSAHGCFERILAFLKTPEKPLTIKNEPETMPESNRGTKDASIQRSE
4_111      GSPLVHLFQAMPALGSAHGCFERILAFLKTPEKPLTIKNEPETMPESNRGTKDASIQRSE
5_5742     GSPLVHLFQAMPALGSAHGCFERILAFLKTPEKPLTIKNEPETMPESNRGTKDASIQRSE
6_9490     GSPLVHLFQAMPALGSAHGCFERILAFLKTPEKPLTIKNEPETMPESNRGTKDASIQRSE
7_6313     GETAAGLSVIRANGWLGPMRGKFDEKLDRSQEPFYLLYMVQRWLQLVLNLVVAGLAIAIA
8_2552     GSPLVHLFQAMPALGSAHGCFERILAFLKTPEKPLTIKNEPETMPESNRGTKDASIQRSE
9_7745     GSPLVHLFQAMPALGSAHGCFERILAFLKTPEKPLTIKNEPETMPESNRGTKDASIQRSE
10_2315    GSPLVHLFQAMPALGSAHGCFERILAFLKTPEKPLTIKNEPETMPESNRGTKDASIQRSE
11_2035    GSPLVHLFQAMPALGSAHGCFERILAFLKTPEKPLTIKNEPETMPESNRGTKDASIQRSE
12_9640    GSPLVHLFQAMPALGSAHGCFERILAFLKTPEKPLTIKNEPETMPESNRGTKDASIQRSE
13_3674    GSPLVHLFQAMPALGSAHGCFERILAFLKTPEKPLTIKNEPETMPESNRGTKDASIQRSE
14_6548    GSPLVHLFQAMPALGSAHGCFERILAFLKTPEKPLTIKNEPETMPESNRGTKDASIQRSE
15_10828   GSPLVHLFQAMPALGSAHGCFERILAFLKTPEKPLTIKNEPETMPESNRGTKDASIQRSE
16_6578    GSPLVHLFQAMPALGSAHGCFERILAFLKTPEKPLTIKNEPETMPESNRGTKDASIQRSE
17_10233   GSPLVHLFQAMPALGSAHGCFERILAFLKTPEKPLTIKNEPETMPESNRGTKDASIQRSE
18_9261    GSPLVHLFQAMPALGSAHGCFERILAFLKTPEKPLTIKNEPETMPESNRGTKDASIQRSE
19_8450    GSPLVHLFQAMPALGSAHGCFERILAFLKTPEKPLTIKNEPETMPESNRGTKDASIQRSE
20_1727    GSPLVHLFQAMPALGSAHGCFERILAFLKTPEKPLTIKNEPETMPESNRGTKDASIQRSE
21_206     GSPLVHLFQAMPALGSAHGCFERILAFLKTPEKPLTIKNEPETMPESNRGTKDASIQRSE
22_7439    GSPLVHLFQAMPALGSAHGCFERILAFLKTPEKPLTIKNEPETMPESNRGTKDASIQRSE
23_3780    GSPLVHLFQAMPALGSAHGCFERILAFLKTPEKPLTIKNEPETMPESNRGTKDASIQRSE
24_967     GSPLVHLFQAMPALGSAHGCFERILAFLKTPEKPLTIKNEPETMPESNRGTKDASIQRSE
25_6595    GSPLVHLFQAMPALGSAHGCFERILAFLKTPEKPLTIKNEPETMPESNRGTKDASIQRSE
26_8391    GSPLVHLFQAMPALGSAHGCFERILAFLKTPEKPLTIKNEPETMPESNRGTKDASIQRSE
27_8170    GSPLVHLFQAMPALGSAHGCFERILAFLKTPEKPLTIKNEPETMPESNRGTKDASIQRSE
28_5209    GSPLVHLFQAMPALGSAHGCFERILAFLKTPEKPLTIKNEPETMPESNRGTKDASIQRSE
29_2181    GSPLVHLFQAMPALGSAHGCFERILAFLKTPEKPLTIKNEPETMPESNRGTKDASIQRSE
30_8564    GSPLVHLFQAMPALGSAHGCFERILAFLKTPEKPLTIKNEPETMPESNRGTKDASIQRSE
31_1582    GSPLVHLFQAMPALGSAHGCFERILAFLKTPEKPLTIKNEPETMPESNRGTKDASIQRSE
32_4537    GSPLVHLFQAMPALGSAHGCFERILAFLKTPEKPLTIKNEPETMPESNRGTKDASIQRSE
33_9418    GSPLVHLFQAMPALGSAHGCFERILAFLKTPEKPLTIKNEPETMPESNRGTKDASIQRSE
34_1782    GSPLVHLFQAMPALGSAHGCFERILAFLKTPEKPLTIKNEPETMPESNRGTKDASIQRSE
35_10360   GSPLVHLFQAMPALGSAHGCFERILAFLKTPEKPLTIKNEPETMPESNRGTKDASIQRSE
36_468     GSPLVHLFQAMPALGSAHGCFERILAFLKTPEKPLTIKNEPETMPESNRGTKDASIQRSE
37_4956    GSPLVHLFQAMPALGSAHGCFERILAFLKTPEKPLTIKNEPETMPESNRGTKDASIQRSE
38_4305    GSPLVHLFQAMPALGSAHGCFERILAFLKTPEKPLTIKNEPETMPESNRGTKDASIQRSE
39_4737    GSPLVHLFQAMPALGSAHGCFERILAFLKTPEKPLTIKNEPETMPESNRGTKDASIQRSE
40_9543    GSPLVHLFQAMPALGSAHGCFERILAFLKTPEKPLTIKNEPETMPESNRGTKDASIQRSE
41_6669    GSPLVHLFQAMPALGSAHGCFERILAFLKTPEKPLTIKNEPETMPESNRGTKDASIQRSE
42_10558   GSPLVHLFQAMPALGSAHGCFERILAFLKTPEKPLTIKNEPETMPESNRGTKDASIQRSE
43_8051    GSPLVHLFQAMPALGSAHGCFERILAFLKTPEKPLTIKNEPETMPESNRGTKDASIQRSE
44_10454   GSPLVHLFQAMPALGSAHGCFERILAFLKTPEKPLTIKNEPETMPESNRGTKDASIQRSE
45_10126   GSPLVHLFQAMPALGSAHGCFERILAFLKTPEKPLTIKNEPETMPESNRGTKDASIQRSE
46_1547    GSPLVHLFQAMPALGSAHGCFERILAFLKTPEKPLTIKNEPETMPESNRGTKDASIQRSE
47_6749    GSPLVHLFQAMPALGSAHGCFERILAFLKTPEKPLTIKNEPETMPESNRGTKDASIQRSE
48_9982    GSPLVHLFQAMPALGSAHGCFERILAFLKTPEKPLTIKNEPETMPESNRGTKDASIQRSE
49_7293    GSPLVHLFQAMPALGSAHGCFERILAFLKTPEKPLTIKNEPETMPESNRGTKDASIQRSE
50_5993    GSPLVHLFQAMPALGSAHGCFERILAFLKTPEKPLTIKNEPETMPESNRGTKDASIQRSE
51_4791    GSPLVHLFQAMPALGSAHGCFERILAFLKTPEKPLTIKNEPETMPESNRGTKDASIQRSE
52_121     GSPLVHLFQAMPALGSAHGCFERILAFLKTPEKPLTIKNEPETMPESNRGTKDASIQRSE
53_1667    GSPLVHLFQAMPALGSAHGCFERILAFLKTPEKPLTIKNEPETMPESNRGTKDASIQRSE
54_10488   GSPLVHLFQAMPALGSAHGCFERILAFLKTPEKPLTIKNEPETMPESNRGTKDASIQRSE
55_7733    GSPLVHLFQAMPALGSAHGCFERILAFLKTPEKPLTIKNEPETMPESNRGTKDASIQRSE
56_3982    GSPLVHLFQAMPALGSAHGCFERILAFLKTPEKPLTIKNEPETMPESNRGTKDASIQRSE
57_392     GSPLVHLFQAMPALGSAHGCFERILAFLKTPEKPLTIKNEPETMPESNRGTKDASIQRSE
58_10291   GSPLVHLFQAMPALGSAHGCFERILAFLKTPEKPLTIKNEPETMPESNRGTKDASIQRSE
59_5792    GSPLVHLFQAMPALGSAHGCFERILAFLKTPEKPLTIKNEPETMPESNRGTKDASIQRSE
60_8712    GSPLVHLFQAMPALGSAHGCFERILAFLKTPEKPLTIKNEPETMPESNRGTKDASIQRSE
61_6716    GSPLVHLFQAMPALGSAHGCFERILAFLKTPEKPLTIKNEPETMPESNRGTKDASIQRSE
62_5599    GSPLVHLFQAMPALGSAHGCFERILAFLKTPEKPLTIKNEPETMPESNRGTKDASIQRSE
63_304     GSPLVHLFQAMPALGSAHGCFERILAFLKTPEKPLTIKNEPETMPESNRGTKDASIQRSE
64_10473   ............................................................
65_6310    GSPLVHLFQAMPALGSAHGCFERILAFLKTPEKPLTIKNEPETMPESNRGTKDASIQRSE
66_8714    GSPLVHLFQAMPALGSAHGCFERILAFLKTPEKPLTIKNEPETMPESNRGTKDASIQRSE
67_2350    GSPLVHLFQAMPALGSAHGCFERILAFLKTPEKPLTIKNEPETMPESNRGTKDASIQRSE
68_2447    GSPLVHLFQAMPALGSAHGCFERILAFLKTPEKPLTIKNEPETMPESNRGTKDASIQRSE
69_5658    GSPLVHLFQAMPALGSAHGCFERILAFLKTPEKPLTIKNEPETMPESNRGTKDASIQRSE
70_1277    GSPLVHLFQAMPALGSAHGCFERILAFLKTPEKPLTIKNEPETMPESNRGTKDASIQRSE
71_3717    GSPLVHLFQAMPALGSAHGCFERILAFLKTPEKPLTIKNEPETMPESNRGTKDASIQRSE
72_7846    GSPLVHLFQAMPALGSAHGCFERILAFLKTPEKPLTIKNEPETMPESNRGTKDASIQRSE
73_10528   GSPLVHLFQAMPALGSAHGCFERILAFLKTPEKPLTIKNEPETMPESNRGTKDASIQRSE
74_4899    GSPLVHLFQAMPALGSAHGCFERILAFLKTPEKPLTIKNEPETMPESNRGTKDASIQRSE
75_5507    GSPLVHLFQAMPALGSAHGCFERILAFLKTPEKPLTIKNEPETMPESNRGTKDASIQRSE
76_5432    GSPLVHLFQAMPALGSAHGCFERILAFLKTPEKPLTIKNEPETMPESNRGTKDASIQRSE
77_10770   GSPLVHLFQAMPALGSAHGCFERILAFLKTPEKPLTIKNEPETMPESNRGTKDASIQRSE
78_5904    GSPLVHLFQAMPALGSAHGCFERILAFLKTPEKPLTIKNEPETMPESNRGTKDASIQRSE
79_9653    GSPLVHLFQAMPALGSAHGCFERILAFLKTPEKPLTIKNEPETMPESNRGTKDASIQRSE
80_4462    GSPLVHLFQAMPALGSAHGCFERILAFLKTPEKPLTIKNEPETMPESNRGTKDASIQRSE
81_7194    GSPLVHLFQAMPALGSAHGCFERILAFLKTPEKPLTIKNEPETMPESNRGTKDASIQRSE
82_1022    GSPLVHLFQAMPALGSAHGCFERILAFLKTPEKPLTIKNEPETMPESNRGTKDASIQRSE
83_9813    GSPLVHLFQAMPALGSAHGCFERILAFLKTPEKPLTIKNEPETMPESNRGTKDASIQRSE
84_1902    GSPLVHLFQAMPALGSAHGCFERILAFLKTPEKPLTIKNEPETMPESNRGTKDASIQRSE
85_5152    GSPLVHLFQAMPALGSAHGCFERILAFLKTPEKPLTIKNEPETMPESNRGTKDASIQRSE
86_3385    GSPLVHLFQAMPALGSAHGCFERILAFLKTPEKPLTIKNEPETMPESNRGTKDASIQRSE
87_10760   GSPLVHLFQAMPALGSAHGCFERILAFLKTPEKPLTIKNEPETMPESNRGTKDASIQRSE
88_1588    GSPLVHLFQAMPALGSAHGCFERILAFLKTPEKPLTIKNEPETMPESNRGTKDASIQRSE
89_1609    GSPLVHLFQAMPALGSAHGCFERILAFLKTPEKPLTIKNEPETMPESNRGTKDASIQRSE
90_4023    GSPLVHLFQAMPALGSAHGCFERILAFLKTPEKPLTIKNEPETMPESNRGTKDASIQRSE
```

```
                    610       620       630       640       650       660
1_4160     ANETALSIRHASIGWSPDEPVLKDINLQIQKGSFVALVGKTGSGKSLLLKSAIGEGGHVS
2_6143     ANETALSIRHASIGWSPDEPVLKDINLQIQKGSFVALVGKTGSGKSLLLKSAIGEGGHVS
3_6554     ANETALSIRHASIGWSPDEPVLKDINLQIQKGSFVALVGKTGSGKSLLLKSAIGEGGHVS
4_111      ANETALSIRHASIGWSPDEPVLKDINLQIQKGSFVALVGKTGSGKSLLLKSAIGEGGHVS
5_5742     ANETALSIRHASIGWSPDEPVLKDINLQIQKGSFVALVGKTGSGKSLLLKSAIGEGGHVS
6_9490     ANETALSIRHASIGWSPDEPVLKDINLQIQKGSFVALVGKTGSGKSLLLKSAIGEGGHVS
7_6313     GVAIGLREQVAAAAVGVALLNTTTLGETLTNFIVSWTSLETSLGAIARVCAFEQDTPRER
8_2552     ANETALSIRHASIGWSPDEPVLKDINLQIQKGSFVALVGKTGSGKSLLLKSAIGEGGHVS
9_7745     ANETALSIRHASIGWSPDEPVLKDINLQIQKGSFVALVGKTGSGKSLLLKSAIGEGGHVS
10_2315    ANETALSIRHASIGWSPDEPVLKDINLQIQKGSFVALVGKTGSGKSLLLKSAIGEGGHVS
11_2035    ANETALSIRHASIGWSPDEPVLKDINLQIQKGSFVALVGKTGSGKSLLLKSAIGEGGHVS
12_9640    ANETALSIRHASIGWSPDEPVLKDINLQIQKGSFVALVGKTGSGKSLLLKSAIGEGGHVS
13_3674    ANETALSIRHASIGWSPDEPVLKDINLQIQKGSFVALVGKTGSGKSLLLKSAIGEGGHVS
14_6548    ANETALSIRHASIGWSPDEPVLKDINLQIQKGSFVALVGKTGSGKSLLLKSAIGEGGHVS
15_10828   ANETALSIRHASIGWSPDEPVLKDINLQIQKGSFVALVGKTGSGKSLLLKSAIGEGGHVS
16_6578    ANETALSIRHASIGWSPDEPVLKDINLQIQKGSFVALVGKTGSGKSLLLKSAIGEGGHVS
17_10233   ANETALSIRHASIGWSPDEPVLKDINLQIQKGSFVALVGKTGSGKSLLLKSAIGEGGHVS
18_9261    ANETALSIRHASIGWSPDEPVLKDINLQIQKGSFVALVGKTGSGKSLLLKSAIGEGGHVS
19_8450    ANETALSIRHASIGWSPDEPVLKDINLQIQKGSFVALVGKTGSGKSLLLKSAIGEGGHVS
20_1727    ANETALSIRHASIGWSPDEPVLKDINLQIQKGSFVALVGKTGSGKSLLLKSAIGEGGHVS
21_206     ANETALSIRHASIGWSPDEPVLKDINLQIQKGSFVALVGKTGSGKSLLLKSAIGEGGHVS
22_7439    ANETALSIRHASIGWSPDEPVLKDINLQIQKGSFVALVGKTGSGKSLLLKSAIGEGGHVS
23_3780    ANETALSIRHASIGWSPDEPVLKDINLQIQKGSFVALVGKTGSGKSLLLKSAIGEGGHVS
24_967     ANETALSIRHASIGWSPDEPVLKDINLQIQKGSFVALVGKTGSGKSLLLKSAIGEGGHVS
25_6595    ANETALSIRHASIGWSPDEPVLKDINLQIQKGSFVALVGKTGSGKSLLLKSAIGEGGHVS
26_8391    ANETALSIRHASIGWSPDEPVLKDINLQIQKGSFVALVGKTGSGKSLLLKSAIGEGGHVS
27_8170    ANETALSIRHASIGWSPDEPVLKDINLQIQKGSFVALVGKTGSGKSLLLKSAIGEGGHVS
28_5209    ANETALSIRHASIGWSPDEPVLKDINLQIQKGSFVALVGKTGSGKSLLLKSAIGEGGHVS
29_2181    ANETALSIRHASIGWSPDEPVLKDINLQIQKGSFVALVGKTGSGKSLLLKSAIGEGGHVS
30_8564    ANETALSIRHASIGWSPDEPVLKDINLQIQKGSFVALVGKTGSGKSLLLKSAIGEGGHVS
31_1582    ANETALSIRHASIGWSPDEPVLKDINLQIQKGSFVALVGKTGSGKSLLLKSAIGEGGHVS
32_4537    ANETALSIRHASIGWSPDEPVLKDINLQIQKGSFVALVGKTGSGKSLLLKSAIGEGGHVS
33_9418    ANETALSIRHASIGWSPDEPVLKDINLQIQKGSFVALVGKTGSGKSLLLKSAIGEGGHVS
34_1782    ANETALSIRHASIGWSPDEPVLKDINLQIQKGSFVALVGKTGSGKSLLLKSAIGEGGHVS
35_10360   ANETALSIRHASIGWSPDEPVLKDINLQIQKGSFVALVGKTGSGKSLLLKSAIGEGGHVS
36_468     ANETALSIRHASIGWSPDEPVLKDINLQIQKGSFVALVGKTGSGKSLLLKSAIGEGGHVS
37_4956    ANETALSIRHASIGWSPDEPVLKDINLQIQKGSFVALVGKTGSGKSLLLKSAIGEGGHVS
38_4305    ANETALSIRHASIGWSPDEPVLKDINLQIQKGSFVALVGKTGSGKSLLLKSAIGEGGHVS
39_4737    ANETALSIRHASIGWSPDEPVLKDINLQIQKGSFVALVGKTGSGKSLLLKSAIGEGGHVS
40_9543    ANETALSIRHASIGWSPDEPVLKDINLQIQKGSFVALVGKTGSGKSLLLKSAIGEGGHVS
41_6669    ANETALSIRHASIGWSPDEPVLKDINLQIQKGSFVALVGKTGSGKSLLLKSAIGEGGHVS
42_10558   ANETALSIRHASIGWSPDEPVLKDINLQIQKGSFVALVGKTGSGKSLLLKSAIGEGGHVS
43_8051    ANETALSIRHASIGWSPDEPVLKDINLQIQKGSFVALVGKTGSGKSLLLKSAIGEGGHVS
44_10454   ANETALSIRHASIGWSPDEPVLKDINLQIQKGSFVALVGKTGSGKSLLLKSAIGEGGHVS
45_10126   ANETALSIRHASIGWSPDEPVLKDINLQIQKGSFVALVGKTGSGKSLLLKSAIGEGGHVS
46_1547    ANETALSIRHASIGWSPDEPVLKDINLQIQKGSFVALVGKTGSGKSLLLKSAIGEGGHVS
47_6749    ANETALSIRHASIGWSPDEPVLKDINLQIQKGSFVALVGKTGSGKSLLLKSAIGEGGHVS
48_9982    ANETALSIRHASIGWSPDEPVLKDINLQIQKGSFVALVGKTGSGKSLLLKSAIGEGGHVS
49_7293    ANETALSIRHASIGWSPDEPVLKDINLQIQKGSFVALVGKTGSGKSLLLKSAIGEGGHVS
50_5993    ANETALSIRHASIGWSPDEPVLKDINLQIQKGSFVALVGKTGSGKSLLLKSAIGEGGHVS
51_4791    ANETALSIRHASIGWSPDEPVLKDINLQIQKGSFVALVGKTGSGKSLLLKSAIGEGGHVS
52_121     ANETALSIRHASIGWSPDEPVLKDINLQIQKGSFVALVGKTGSGKSLLLKSAIGEGGHVS
53_1667    ANETALSIRHASIGWSPDEPVLKDINLQIQKGSFVALVGKTGSGKSLLLKSAIGEGGHVS
54_10488   ANETALSIRHASIGWSPDEPVLKDINLQIQKGSFVALVGKTGSGKSLLLKSAIGEGGHVS
55_7733    ANETALSIRHASIGWSPDEPVLKDINLQIQKGSFVALVGKTGSGKSLLLKSAIGEGGHVS
56_3982    ANETALSIRHASIGWSPDEPVLKDINLQIQKGSFVALVGKTGSGKSLLLKSAIGEGGHVS
57_392     ANETALSIRHASIGWSPDEPVLKDINLQIQKGSFVALVGKTGSGKSLLLKSAIGEGGHVS
58_10291   ANETALSIRHASIGWSPDEPVLKDINLQIQKGSFVALVGKTGSGKSLLLKSAIGEGGHVS
59_5792    ANETALSIRHASIGWSPDEPVLKDINLQIQKGSFVALVGKTGSGKSLLLKSAIGEGGHVS
60_8712    ANETALSIRHASIGWSPDEPVLKDINLQIQKGSFVALVGKTGSGKSLLLKSAIGEGGHVS
61_6716    ANETALSIRHASIGWSPDEPVLKDINLQIQKGSFVALVGKTGSGKSLLLKSAIGEGGHVS
62_5599    ANETALSIRHASIGWSPDEPVLKDINLQIQKGSFVALVGKTGSGKSLLLKSAIGEGGHVS
63_304     ANETALSIRHASIGWSPDEPVLKDINLQIQKGSFVALVGKTGSGKSLLLKSAIGEGGHVS
64_10473   ............................................................
65_6310    ANETALSIRHASIGWSPDEPVLKDINLQIQKGSFVALVGKTGSGKSLLLKSAIGEGGHVS
66_8714    ANETALSIRHASIGWSPDEPVLKDINLQIQKGSFVALVGKTGSGKSLLLKSAIGEGGHVS
67_2350    ANETALSIRHASIGWSPDEPVLKDINLQIQKGSFVALVGKTGSGKSLLLKSAIGEGGHVS
68_2447    ANETALSIRHASIGWSPDEPVLKDINLQIQKGSFVALVGKTGSGKSLLLKSAIGEGGHVS
69_5658    ANETALSIRHASIGWSPDEPVLKDINLQIQKGSFVALVGKTGSGKSLLLKSAIGEGGHVS
70_1277    ANETALSIRHASIGWSPDEPVLKDINLQIQKGSFVALVGKTGSGKSLLLKSAIGEGGHVS
71_3717    ANETALSIRHASIGWSPDEPVLKDINLQIQKGSFVALVGKTGSGKSLLLKSAIGEGGHVS
72_7846    ANETALSIRHASIGWSPDEPVLKDINLQIQKGSFVALVGKTGSGKSLLLKSAIGEGGHVS
73_10528   ANETALSIRHASIGWSPDEPVLKDINLQIQKGSFVALVGKTGSGKSLLLKSAIGEGGHVS
74_4899    ANETALSIRHASIGWSPDEPVLKDINLQIQKGSFVALVGKTGSGKSLLLKSAIGEGGHVS
75_5507    ANETALSIRHASIGWSPDEPVLKDINLQIQKGSFVALVGKTGSGKSLLLKSAIGEGGHVS
76_5432    ANETALSIRHASIGWSPDEPVLKDINLQIQKGSFVALVGKTGSGKSLLLKSAIGEGGHVS
77_10770   ANETALSIRHASIGWSPDEPVLKDINLQIQKGSFVALVGKTGSGKSLLLKSAIGEGGHVS
78_5904    ANETALSIRHASIGWSPDEPVLKDINLQIQKGSFVALVGKTGSGKSLLLKSAIGEGGHVS
79_9653    ANETALSIRHASIGWSPDEPVLKDINLQIQKGSFVALVGKTGSGKSLLLKSAIGEGGHVS
80_4462    ANETALSIRHASIGWSPDEPVLKDINLQIQKGSFVALVGKTGSGKSLLLKSAIGEGGHVS
81_7194    ANETALSIRHASIGWSPDEPVLKDINLQIQKGSFVALVGKTGSGKSLLLKSAIGEGGHVS
82_1022    ANETALSIRHASIGWSPDEPVLKDINLQIQKGSFVALVGKTGSGKSLLLKSAIGEGGHVS
83_9813    ANETALSIRHASIGWSPDEPVLKDINLQIQKGSFVALVGKTGSGKSLLLKSAIGEGGHVS
84_1902    ANETALSIRHASIGWSPDEPVLKDINLQIQKGSFVALVGKTGSGKSLLLKSAIGEGGHVS
85_5152    ANETALSIRHASIGWSPDEPVLKDINLQIQKGSFVALVGKTGSGKSLLLKSAIGEGGHVS
86_3385    ANETALSIRHASIGWSPDEPVLKDINLQIQKGSFVALVGKTGSGKSLLLKSAIGEGGHVS
87_10760   ANETALSIRHASIGWSPDEPVLKDINLQIQKGSFVALVGKTGSGKSLLLKSAIGEGGHVS
88_1588    ANETALSIRHASIGWSPDEPVLKDINLQIQKGSFVALVGKTGSGKSLLLKSAIGEGGHVS
89_1609    ANETALSIRHASIGWSPDEPVLKDINLQIQKGSFVALVGKTGSGKSLLLKSAIGEGGHVS
90_4023    ANETALSIRHASIGWSPDEPVLKDINLQIQKGSFVALVGKTGSGKSLLLKSAIGEGGHVS
```

```
                670       680       690       700       710       720
1_4160     GSIDISLDKVAYCSQSPWLENISAEHTWTQFGESGDAKWLAGVIDACCLDDLTGLPDYRT
2_6143     GSIDISLDKVAYCSQSPWLENISAEHTWTQFGESGDAKWLAGVIDACCLDDLTGLPDYRT
3_6554     GSIDISLDKVAYCSQSPWLENISAEHTWTQFGESGDAKWLAGVIDACCLDDLTGLPDYRT
4_111      GSIDISLDKVAYCSQSPWLENISAEHTWTQFGESGDAKWLAGVIDACCLDDLTGLPDYRT
5_5742     GSIDISLDKVAYCSQSPWLENISAEHTWTQFGESGDAKWLAGVIDACCLDDLTGLPDYRT
6_9490     GSIDISLDKVAYCSQSPWLENISAEHTWTQFGESGDAKWLAGVIDACCLDDLTGLPDYRT
7_6313     EEPATTDLPDNWPGAGQISFENVWATYDEEGCDGNWGLSGITLGIQPGERVAVCGRTGSG
8_2552     GSIDISLDKVAYCSQSPWLENISAEHTWTQFGESGDAKWLAGVIDACCLDDLTGLPDYRT
9_7745     GSIDISLDKVAYCSQSPWLENISAEHTWTQFGESGDAKWLAGVIDACCLDDLTGLPDYRT
10_2315    GSIDISLDKVAYCSQSPWLENISAEHTWTQFGESGDAKWLAGVIDACCLDDLTGLPDYRT
11_2035    GSIDISLDKVAYCSQSPWLENISAEHTWTQFGESGDAKWLAGVIDACCLDDLTGLPDYRT
12_9640    GSIDISLDKVAYCSQSPWLENISAEHTWTQFGESGDAKWLAGVIDACCLDDLTGLPDYRT
13_3674    GSIDISLDKVAYCSQSPWLENISAEHTWTQFGESGDAKWLAGVIDACCLDDLTGLPDYRT
14_6548    GSIDISLDKVAYCSQSPWLENISAEHTWTQFGESGDAKWLAGVIDACCLDDLTGLPDYRT
15_10828   GSIDISLDKVAYCSQSPWLENISAEHTWTQFGESGDAKWLAGVIDACCLDDLTGLPDYRT
16_6578    GSIDISLDKVAYCSQSPWLENISAEHTWTQFGESGDAKWLAGVIDACCLDDLTGLPDYRT
17_10233   GSIDISLDKVAYCSQSPWLENISAEHTWTQFGESGDAKWLAGVIDACCLDDLTGLPDYRT
18_9261    GSIDISLDKVAYCSQSPWLENISAEHTWTQFGESGDAKWLAGVIDACCLDDLTGLPDYRT
19_8450    GSIDISLDKVAYCSQSPWLENISAEHTWTQFGESGDAKWLAGVIDACCLDDLTGLPDYRT
20_1727    GSIDISLDKVAYCSQSPWLENISAEHTWTQFGESGDAKWLAGVIDACCLDDLTGLPDYRT
21_206     GSIDISLDKVAYCSQSPWLENISAEHTWTQFGESGDAKWLAGVIDACCLDDLTGLPDYRT
22_7439    GSIDISLDKVAYCSQSPWLENISAEHTWTQFGESGDAKWLAGVIDACCLDDLTGLPDYRT
23_3780    GSIDISLDKVAYCSQSPWLENISAEHTWTQFGESGDAKWLAGVIDACCLDDLTGLPDYRT
24_967     GSIDISLDKVAYCSQSPWLENISAEHTWTQFGESGDAKWLAGVIDACCLDDLTGLPDYRT
25_6595    GSIDISLDKVAYCSQSPWLENISAEHTWTQFGESGDAKWLAGVIDACCLDDLTGLPDYRT
26_8391    GSIDISLDKVAYCSQSPWLENISAEHTWTQFGESGDAKWLAGVIDACCLDDLTGLPDYRT
27_8170    GSIDISLDKVAYCSQSPWLENISAEHTWTQFGESGDAKWLAGVIDACCLDDLTGLPDYRT
28_5209    GSIDISLDKVAYCSQSPWLENISAEHTWTQFGESGDAKWLAGVIDACCLDDLTGLPDYRT
29_2181    GSIDISLDKVAYCSQSPWLENISAEHTWTQFGESGDAKWLAGVIDACCLDDLTGLPDYRT
30_8564    GSIDISLDKVAYCSQSPWLENISAEHTWTQFGESGDAKWLAGVIDACCLDDLTGLPDYRT
31_1582    GSIDISLDKVAYCSQSPWLENISAEHTWTQFGESGDAKWLAGVIDACCLDDLTGLPDYRT
32_4537    GSIDISLDKVAYCSQSPWLENISAEHTWTQFGESGDAKWLAGVIDACCLDDLTGLPDYRT
33_9418    GSIDISLDKVAYCSQSPWLENISAEHTWTQFGESGDAKWLAGVIDACCLDDLTGLPDYRT
34_1782    GSIDISLDKVAYCSQSPWLENISAEHTWTQFGESGDAKWLAGVIDACCLDDLTGLPDYRT
35_10360   GSIDISLDKVAYCSQSPWLENISAEHTWTQFGESGDAKWLAGVIDACCLDDLTGLPDYRT
36_468     GSIDISLDKVAYCSQSPWLENISAEHTWTQFGESGDAKWLAGVIDACCLDDLTGLPDYRT
37_4956    GSIDISLDKVAYCSQSPWLENISAEHTWTQFGESGDAKWLAGVIDACCLDDLTGLPDYRT
38_4305    GSIDISLDKVAYCSQSPWLENISAEHTWTQFGESGDAKWLAGVIDACCLDDLTGLPDYRT
39_4737    GSIDISLDKVAYCSQSPWLENISAEHTWTQFGESGDAKWLAGVIDACCLDDLTGLPDYRT
40_9543    GSIDISLDKVAYCSQSPWLENISAEHTWTQFGESGDAKWLAGVIDACCLDDLTGLPDYRT
41_6669    GSIDISLDKVAYCSQSPWLENISAEHTWTQFGESGDAKWLAGVIDACCLDDLTGLPDYRT
42_10558   GSIDISLDKVAYCSQSPWLENISAEHTWTQFGESGDAKWLAGVIDACCLDDLTGLPDYRT
43_8051    GSIDISLDKVAYCSQSPWLENISAEHTWTQFGESGDAKWLAGVIDACCLDDLTGLPDYRT
44_10454   GSIDISLDKVAYCSQSPWLENISAEHTWTQFGESGDAKWLAGVIDACCLDDLTGLPDYRT
45_10126   GSIDISLDKVAYCSQSPWLENISAEHTWTQFGESGDAKWLAGVIDACCLDDLTGLPDYRT
46_1547    GSIDISLDKVAYCSQSPWLENISAEHTWTQFGESGDAKWLAGVIDACCLDDLTGLPDYRT
47_6749    GSIDISLDKVAYCSQSPWLENISAEHTWTQFGESGDAKWLAGVIDACCLDDLTGLPDYRT
48_9982    GSIDISLDKVAYCSQSPWLENISAEHTWTQFGESGDAKWLAGVIDACCLDDLTGLPDYRT
49_7293    GSIDISLDKVAYCSQSPWLENISAEHTWTQFGESGDAKWLAGVIDACCLDDLTGLPDYRT
50_5993    GSIDISLDKVAYCSQSPWLENISAEHTWTQFGESGDAKWLAGVIDACCLDDLTGLPDYRT
51_4791    GSIDISLDKVAYCSQSPWLENISAEHTWTQFGESGDAKWLAGVIDACCLDDLTGLPDYRT
52_121     GSIDISLDKVAYCSQSPWLENISAEHTWTQFGESGDAKWLAGVIDACCLDDLTGLPDYRT
53_1667    GSIDISLDKVAYCSQSPWLENISAEHTWTQFGESGDAKWLAGVIDACCLDDLTGLPDYRT
54_10488   GSIDISLDKVAYCSQSPWLENISAEHTWTQFGESGDAKWLAGVIDACCLDDLTGLPDYRT
55_7733    GSIDISLDKVAYCSQSPWLENISAEHTWTQFGESGDAKWLAGVIDACCLDDLTGLPDYRT
56_3982    GSIDISLDKVAYCSQSPWLENISAEHTWTQFGESGDAKWLAGVIDACCLDDLTGLPDYRT
57_392     GSIDISLDKVAYCSQSPWLENISAEHTWTQFGESGDAKWLAGVIDACCLDDLTGLPDYRT
58_10291   GSIDISLDKVAYCSQSPWLENISAEHTWTQFGESGDAKWLAGVIDACCLDDLTGLPDYRT
59_5792    GSIDISLDKVAYCSQSPWLENISAEHTWTQFGESGDAKWLAGVIDACCLDDLTGLPDYRT
60_8712    GSIDISLDKVAYCSQSPWLENISAEHTWTQFGESGDAKWLAGVIDACCLDDLTGLPDYRT
61_6716    GSIDISLDKVAYCSQSPWLENISAEHTWTQFGESGDAKWLAGVIDACCLDDLTGLPDYRT
62_5599    GSIDISLDKVAYCSQSPWLENISAEHTWTQFGESGDAKWLAGVIDACCLDDLTGLPDYRT
63_304     GSIDISLDKVAYCSQSPWLENISAEHTWTQFGESGDAKWLAGVIDACCLDDLTGLPDYRT
64_10473   ............................................................
65_6310    GSIDISLDKVAYCSQSPWLENISAEHTWTQFGESGDAKWLAGVIDACCLDDLTGLPDYRT
66_8714    GSIDISLDKVAYCSQSPWLENISAEHTWTQFGESGDAKWLAGVIDACCLDDLTGLPDYRT
67_2350    GSIDISLDKVAYCSQSPWLENISAEHTWTQFGESGDAKWLAGVIDACCLDDLTGLPDYRT
68_2447    GSIDISLDKVAYCSQSPWLENISAEHTWTQFGESGDAKWLAGVIDACCLDDLTGLPDYRT
69_5658    GSIDISLDKVAYCSQSPWLENISAEHTWTQFGESGDAKWLAGVIDACCLDDLTGLPDYRT
70_1277    GSIDISLDKVAYCSQSPWLENISAEHTWTQFGESGDAKWLAGVIDACCLDDLTGLPDYRT
71_3717    GSIDISLDKVAYCSQSPWLENISAEHTWTQFGESGDAKWLAGVIDACCLDDLTGLPDYRT
72_7846    GSIDISLDKVAYCSQSPWLENISAEHTWTQFGESGDAKWLAGVIDACCLDDLTGLPDYRT
73_10528   GSIDISLDKVAYCSQSPWLENISAEHTWTQFGESGDAKWLAGVIDACCLDDLTGLPDYRT
74_4899    GSIDISLDKVAYCSQSPWLENISAEHTWTQFGESGDAKWLAGVIDACCLDDLTGLPDYRT
75_5507    GSIDISLDKVAYCSQSPWLENISAEHTWTQFGESGDAKWLAGVIDACCLDDLTGLPDYRT
76_5432    GSIDISLDKVAYCSQSPWLENISAEHTWTQFGESGDAKWLAGVIDACCLDDLTGLPDYRT
77_10770   GSIDISLDKVAYCSQSPWLENISAEHTWTQFGESGDAKWLAGVIDACCLDDLTGLPDYRT
78_5904    GSIDISLDKVAYCSQSPWLENISAEHTWTQFGESGDAKWLAGVIDACCLDDLTGLPDYRT
79_9653    GSIDISLDKVAYCSQSPWLENISAEHTWTQFGESGDAKWLAGVIDACCLDDLTGLPDYRT
80_4462    GSIDISLDKVAYCSQSPWLENISAEHTWTQFGESGDAKWLAGVIDACCLDDLTGLPDYRT
81_7194    GSIDISLDKVAYCSQSPWLENISAEHTWTQFGESGDAKWLAGVIDACCLDDLTGLPDYRT
82_1022    GSIDISLDKVAYCSQSPWLENISAEHTWTQFGESGDAKWLAGVIDACCLDDLTGLPDYRT
83_9813    GSIDISLDKVAYCSQSPWLENISAEHTWTQFGESGDAKWLAGVIDACCLDDLTGLPDYRT
84_1902    GSIDISLDKVAYCSQSPWLENISAEHTWTQFGESGDAKWLAGVIDACCLDDLTGLPDYRT
85_5152    GSIDISLDKVAYCSQSPWLENISAEHTWTQFGESGDAKWLAGVIDACCLDDLTGLPDYRT
86_3385    GSIDISLDKVAYCSQSPWLENISAEHTWTQFGESGDAKWLAGVIDACCLDDLTGLPDYRT
87_10760   GSIDISLDKVAYCSQSPWLENISAEHTWTQFGESGDAKWLAGVIDACCLDDLTGLPDYRT
88_1588    GSIDISLDKVAYCSQSPWLENISAEHTWTQFGESGDAKWLAGVIDACCLDDLTGLPDYRT
89_1609    GSIDISLDKVAYCSQSPWLENISAEHTWTQFGESGDAKWLAGVIDACCLDDLTGLPDYRT
90_4023    GSIDISLDKVAYCSQSPWLENISAEHTWTQFGESGDAKWLAGVIDACCLDDLTGLPDYRT
```

```
                   730       740       750       760       770       780
1_4160     GRIGSGGARLSGGQKQRLALARAIATRKDIVLLDDVFSALDRTTKHHIATRLLGPEGLLR
2_6143     GRIGSGGARLSGGQKQRLALARAIATRKDIVLLDDVFSALDRTTKHHIATRLLGPEGLLR
3_6554     GRIGSGGARLSGGQKQRLALARAIATRKDIVLLDDVFSALDRTTKHHIATRLLGPEGLLR
4_111      GRIGSGGARLSGGQKQRLALARAIATRKDIVLLDDVFSALDRTTKHHIATRLLGPEGLLR
5_5742     GRIGSGGARLSGGQKQRLALARAIATRKDIVLLDDVFSALDRTTKHHIATRLLGPEGLLR
6_9490     GRIGSGGARLSGGQKQRLALARAIATRKDIVLLDDVFSALDRTTKHHIATRLLGPEGLLR
7_6313     KSTLLLALLGMLHVPAGSIRLDDMDTSTLPISVLRRRFTVVSQDSFFEPTSTFRQELDPS
8_2552     GRIGSGGARLSGGQKQRLALARAIATRKDIVLLDDVFSALDRTTKHHIATRLLGPEGLLR
9_7745     GRIGSGGARLSGGQKQRLALARAIATRKDIVLLDDVFSALDRTTKHHIATRLLGPEGLLR
10_2315    GRIGSGGARLSGGQKQRLALARAIATRKDIVLLDDVFSALDRTTKHHIATRLLGPEGLLR
11_2035    GRIGSGGARLSGGQKQRLALARAIATRKDIVLLDDVFSALDRTTKHHIATRLLGPEGLLR
12_9640    GRIGSGGARLSGGQKQRLALARAIATRKDIVLLDDVFSALDRTTKHHIATRLLGPEGLLR
13_3674    GRIGSGGARLSGGQKQRLALARAIATRKDIVLLDDVFSALDRTTKHHIATRLLGPEGLLR
14_6548    GRIGSGGARLSGGQKQRLALARAIATRKDIVLLDDVFSALDRTTKHHIATRLLGPEGLLR
15_10828   GRIGSGGARLSGGQKQRLALARAIATRKDIVLLDDVFSALDRTTKHHIATRLLGPEGLLR
16_6578    GRIGSGGARLSGGQKQRLALARAIATRKDIVLLDDVFSALDRTTKHHIATRLLGPEGLLR
17_10233   GRIGSGGARLSGGQKQRLALARAIATRKDIVLLDDVFSALDRTTKHHIATRLLGPEGLLR
18_9261    GRIGSGGARLSGGQKQRLALARAIATRKDIVLLDDVFSALDRTTKHHIATRLLGPEGLLR
19_8450    GRIGSGGARLSGGQKQRLALARAIATRKDIVLLDDVFSALDRTTKHHIATRLLGPEGLLR
20_1727    GRIGSGGARLSGGQKQRLALARAIATRKDIVLLDDVFSALDRTTKHHIATRLLGPEGLLR
21_206     GRIGSGGARLSGGQKQRLALARAIATRKDIVLLDDVFSALDRTTKHHIATRLLGPEGLLR
22_7439    GRIGSGGARLSGGQKQRLALARAIATRKDIVLLDDVFSALDRTTKHHIATRLLGPEGLLR
23_3780    GRIGSGGARLSGGQKQRLALARAIATRKDIVLLDDVFSALDRTTKHHIATRLLGPEGLLR
24_967     GRIGSGGARLSGGQKQRLALARAIATRKDIVLLDDVFSALDRTTKHHIATRLLGPEGLLR
25_6595    GRIGSGGARLSGGQKQRLALARAIATRKDIVLLDDVFSALDRTTKHHIATRLLGPEGLLR
26_8391    GRIGSGGARLSGGQKQRLALARAIATRKDIVLLDDVFSALDRTTKHHIATRLLGPEGLLR
27_8170    GRIGSGGARLSGGQKQRLALARTIATRKDIVLLDDVFSALDRTTKHHIATRLLGPEGLLR
28_5209    GRIGSGGARLSGGQKQRLALARAIATRKDIVLLDDVFSALDRTTKHHIATRLLGPEGLLR
29_2181    GRIGSGGARLSGGQKQRLALARTIATRKDIVLLDDVFSALDRTTKHHIATRLLGPEGLLR
30_8564    GRIGSGGARLSGGQKQRLALARAIATRKDIVLLDDVFSALDRTTKHHIATRLLGPEGLLR
31_1582    GRIGSGGARLSGGQKQRLALARAIATRKDIVLLDDVFSALDRTTKHHIATRLLGPEGLLR
32_4537    GRIGSGGARLSGGQKQRLALARAIATRKDIVLLDDVFSALDRTTKHHIATRLLGPEGLLR
33_9418    GRIGSGGARLSGGQKQRLALARAIATRKDIVLLDDVFSALDRTTKHHIATRLLGPEGLLR
34_1782    GRIGSGGARLSGGQKQRLALARAIATRKDIVLLDDVFSALDRTTKHHIATRLLGPEGLLR
35_10360   GRIGSGGARLSGGQKQRLALARAIATRKDIVLLDDVFSALDRTTKHHIATRLLGPEGLLR
36_468     GRIGSGGARLSGGQKQRLALARAIATRKDIVLLDDVFSALDRTTKHHIATRLLGPEGLLR
37_4956    GRIGSGGARLSGGQKQRLALARAIATRKDIVLLDDVFSALDRTTKHHIATRLLGPEGLLR
38_4305    GRIGSGGARLSGGQKQRLALARAIATRKDIVLLDDVFSALDRTTKHHIATRLLGPEGLLR
39_4737    GRIGSGGARLSGGQKQRLALARAIATRKDIVLLDDVFSALDRTTKHHIATRLLGPEGLLR
40_9543    GRIGSGGARLSGGQKQRLALARAIATRKDIVLLDDVFSALDRTTKHHIATRLLGPEGLLR
41_6669    GRIGSGGARLSGGQKQRLALARAIATRKDIVLLDDVFSALDRTTKHHIATRLLGPEGLLR
42_10558   GRIGSGGARLSGGQKQRLALARAIATRKDIVLLDDVFSALDRTTKHHIATRLLGPEGLLR
43_8051    GRIGSGGARLSGGQKQRLALARAIATRKDIVLLDDVFSALDRTTKHHIATRLLGPEGLLR
44_10454   GRIGSGGARLSGGQKQRLALARAIATRKDIVLLDDVFSALDRTTKHHIATRLLGPEGLLR
45_10126   GRIGSGGARLSGGQKQRLALARAIATRKDIVLLDDVFSALDRTTKHHIATRLLGPEGLLR
46_1547    GRIGSGGARLSGGQKQRLALARAIATRKDIVLLDDVFSALDRTTKHHIATRLLGPEGLLR
47_6749    GRIGSGGARLSGGQKQRLALARAIATRKDIVLLDDVFSALDRTTKHHIATRLLGPEGLLR
48_9982    GRIGSGGARLSGGQKQRLALARAIATRKDIVLLDDVFSALDRTTKHHIATRLLGPEGLLR
49_7293    GRIGSGGARLSGGQKQRLALARAIATRKDIVLLDDVFSALDRTTKHHIATRLLGPEGLLR
50_5993    GRIGSGGARLSGGQKQRLALARAIATRKDIVLLDDVFSALDRTTKHHIATRLLGPEGLLR
51_4791    GRIGSGGARLSGGQKQRLALARAIATRKDIVLLDDVFSALDRTTKHHIATRLLGPEGLLR
52_121     GRIGSGGARLSGGQKQRLALARAIATRKDIVLLDDVFSALDRTTKHHIATRLLGPEGLLR
53_1667    GRIGSGGARLSGGQKQRLALARAIATRKDIVLLDDVFSALDRTTKHHIATRLLGPEGLLR
54_10488   GRIGSGGARLSGGQKQRLALARAIATRKDIVLLDDVFSALDRTTKHHIATRLLGPEGLLR
55_7733    GRIGSGGARLSGGQKQRLALARAIATRKDIVLLDDVFSALDRTTKHHIATRLLGPEGLLR
56_3982    GRIGSGGARLSGGQKQRLALARAIATRKDIVLLDDVFSALDRTTKHHIATRLLGPEGLLR
57_392     GRIGSGGARLSGGQKQRLALARAIATRKDIVLLDDVFSALDRTTKHHIATRLLGPEGLLR
58_10291   GRIGSGGARLSGGQKQRLALARAIATRKDIVLLDDVFSALDRTTKHHIATRLLGPEGLLR
59_5792    GRIGSGGARLSGGQKQRLALARAIATRKDIVLLDDVFSALDRTTKHHIATRLLGPEGLLR
60_8712    GRIGSGGARLSGGQKQRLALARAIATRKDIVLLDDVFSALDRTTKHHIATRLLGPEGLLR
61_6716    GRIGSGGARLSGGQKQRLALARAIATRKDIVLLDDVFSALDRTTKHHIATRLLGPEGLLR
62_5599    GRIGSGGARLSGGQKQRLALARAIATRKDIVLLDDVFSALDRTTKHHIATRLLGPEGLLR
63_304     GRIGSGGARLSGGQKQRLALARAIATRKDIVLLDDVFSALDRTTKHHIATRLLGPEGLLR
64_10473   ............................................................
65_6310    GRIGSGGARLSGGQKQRLALARAIATRKDIVLLDDVFSALDRTTKHHIATRLLGPEGLLR
66_8714    GRIGSGGARLSGGQKQRLALARAIATRKDIVLLDDVFSALDRTTKHHIATRLLGPEGLLR
67_2350    GRIGSGGARLSGGQKQRLALARAIATRKDIVLLDDVFSALDRTTKHHIATRLLGPEGLLR
68_2447    GRIGSGGARLSGGQKQRLALARAIATRKDIVLLDDVFSALDRTTKHHIATRLLGPEGLLR
69_5658    GRIGSGGARLSGGQKQRLALARAIATRKDIVLLDDVFSALDRTTKHHIATRLLGPEGLLR
70_1277    GRIGSGGARLSGGQKQRLALARAIATRKDIVLLDDVFSALDRTTKHHIATRLLGPEGLLR
71_3717    GRIGSGGARLSGGQKQRLALARAIATRKDIVLLDDVFSALDRTTKHHIATRLLGPEGLLR
72_7846    GRIGSGGARLSGGQKQRLALARAIATRKDIVLLDDVFSALDRTTKHHIATRLLGPEGLLR
73_10528   GRIGSGGARLSGGQKQRLALARAIATRKDIVLLDDVFSALDRTTKHHIATRLLGPEGLLR
74_4899    GRIGSGGARLSGGQKQRLALARAIATRKDIVLLDDVFSALDRTTKHHIATRLLGPEGLLR
75_5507    GRIGSGGARLSGGQKQRLALARAIATRKDIVLLDDVFSALDRTTKHHIATRLLGPEGLLR
76_5432    GRIGSGGARLSGGQKQRLALARAIATRKDIVLLDDVFSALDRTTKHHIATRLLGPEGLLR
77_10770   GRIGSGGARLSGGQKQRLALARAIATRKDIVLLDDVFSALDRTTKHHIATRLLGPEGLLR
78_5904    GRIGSGGARLSGGQKQRLALARAIATRKDIVLLDDVFSALDRTTKHHIATRLLGPEGLLR
79_9653    GRIGSGGARLSGGQKQRLALARAIATRKDIVLLDDVFSALDRTTKHHIATRLLGPEGLLR
80_4462    GRIGSGGARLSGGQKQRLALARAIATRKDIVLLDDVFSALDRTTKHHIATRLLGPEGLLR
81_7194    GRIGSGGARLSGGQKQRLPHGFSGPRGFCEGWERQYYLQLTTVTNLADQVYEITVDGILT
82_1022    GRIGSGGARLSGGQKQRLALARAIATRKDIVLLDDVFSALDRTTKHHIATRLLGPEGLLR
83_9813    GRIGSGGARLSGGQKQRLALARAIATRKDIVLLDDVFSALDRTTKHHIATRLLGPEGLLR
84_1902    GRIGSGGARLSGGQKQRLALARAIATRKDIVLLDDVFSALDRTTKHHIATRLLGPEGLLR
85_5152    GRIGSGGARLSGGQKQRLALARAIATRKDIVLLDDVFSALDRTTKHHIATRLLGPEGLLR
86_3385    GRIGSGGARLSGGQKQRLALARAIATRKDIVLLDDVFSALDRTTKHHIATRLLGPEGLLR
87_10760   GRIGSGGARLSGGQKQRLALARAIATRKDIVLLDDVFSALDRTTKHHIATRLLGPEGLLR
88_1588    GRIGSGGARLSGGQKQRLALARAIATRKDIVLLDDVFSALDRTTKHHIATRLLGPEGLLR
89_1609    GRIGSGGARLSGGQKQRLALARAIATRKDIVLLDDVFSALDRTTKHHIATRLLGPEGLLR
90_4023    GRIGSGGARLSGGQKQRLALARAIATRKDIVLLDDVFSALDRTTKHHIATRLLGPEGLLR
```

```
                   790       800       810       820       830       840
1_4160    RLGTTVLFATHDSSIANLADQVYEITVDGILTPVLVQKPADDEGTKHEDSDAKYTVTDAS
2_6143    RLGTTVLFATHDSSIANLADQVYEITVDGILTPVLVQKPADDEGTKHEDSDAKYTVTDAS
3_6554    RLGTTVLFATHDSSIANLADQVYEITVDGILTPVLVQKPADDEGTKHEDSDAKYTVTDAS
4_111     RLGTTVLFATHDSSIANLADQVYEITVDGILTPVLVQKPADDEGTKHEDSDAKYTVTDAS
5_5742    RLGTTVLFATHDSSIANLADQVYEITVDGILTPVLVQKPADDEGTKHEDSDAKYTVTDAS
6_9490    RLGTTVLFATHDSSIANLADQVYEITVDGILTPVLVQKPADDEGTKHEDSDAKYTVTDAS
7_6313    GDISDQVMEEMLRECRVWEVVENSGGLGAKRADSKLSAGETQLLAIARLLLQWQSQPAGS
8_2552    RLGTTVLFATHDSSIANLADQVYEITVDGILTPVLVQKPADDEGTKHEDSDAKYTVTDAS
9_7745    RLGTTVLFATHDSSIANLADQVYEITVDGILTPVLVQKPADDEGTKHEDSDAKYTVTDAS
10_2315   RLGTTVLFATHDSSIANLADQVYEITVDGILTPVLVQKPADDEGTKHEDSDAKYTVTDAS
11_2035   RLGTTVLFATHDSSIANLADQVYEITVDGILTPVLVQKPADDEGTKHEDSDAKYTVTDAS
12_9640   RLGTTVLFATHDSSIANLADQVYEITVDGILTPVLVQKPADDEGTKHEDSDAKYTVTDAS
13_3674   RLGTTVLFATHDSSIANLADQVYEITVDGILTPVLVQKPADDEGTKHEDSDAKYTVTDAS
14_6548   RLGTTVLFATHDSSIANLADQVYEITVDGILTPVLVQKPADDEGTKHEDSDAKYTVTDAS
15_10828  RLGTTVLFATHDSSIANLADQVYEITVDGILTPVLVQKPADDEGTKHEDSDAKYTVTDAS
16_6578   RLGTTVLFATHDSSIANLADQVYEITVDGILTPVLVQKPADDEGTKHEDSDAKYTVTDAS
17_10233  RLGTTVLFATHDSSIANLADQVYEITVDGILTPVLVQKPADDEGTKHEDSDAKYTVTDAS
18_9261   RLGTTVLFATHDSSIANLADQVYEITVDGILTPVLVQKPADDEGTKHEDSDAKYTVTDAS
19_8450   RLGTTVLFATHDSSIANLADQVYEITVDGILTPVLVQKPADDEGTKHEDSDAKYTVTDAS
20_1727   RLGTTVLFATHDSSIANLADQVYEITVDGILTPVLVQKPADDEGTKHEDSDAKYTVTDAS
21_206    RLGTTVLFATHDSSIANLADQVYEITVDGILTPVLVQKPADDEGTKHEDSDAKYTVTDAS
22_7439   RLGTTVLFATHDSSIANLADQVYEITVDGILTPVLVQKPADDEGTKHEDSDAKYTVTDAS
23_3780   RLGTTVLFATHDSSIANLADQVYEITVDGILTPVLVQKPADDEGTKHEDSDAKYTVTDAS
24_967    RLGTTVLFATHDSSIANLADQVYEITVDGILTPVLVQKPADDEGTKHEDSDAKYTVTDAS
25_6595   RLGTTVLFATHDSSIANLADQVYEITVDGILTPVLVQKPADDEGTKHEDSDAKYTVTDAS
26_8391   RLGTTVLFATHDSSIANLADQVYEITVDGILTPVLVQKPADDEGTKHEDSDAKYTVTDAS
27_8170   RLGTTVLFATHDSSIANLADQVYEITVDGILTPVLVQKPADDEGTKHEDSDAKYTVTDAS
28_5209   RLGTTVLFATHDSSIANLADQVYEITVDGILTPVLVQKPADDEGTKHEDSDAKYTVTDAS
29_2181   RLGTTVLFATHDSSIANLADQVYEITVDGILTPVLVQKPADDEGTKHEDSDAKYTVTDAS
30_8564   RLGTTVLFATHDSSIANLADQVYEITVDGILTPVLVQKPADDEGTKHEDSDAKYTVTDAS
31_1582   RLGTTVLFATHDSSIANLADQVYEITVDGILTPVLVQKPADDEGTKHEDSDAKYTVTDAS
32_4537   RLGTTVLFATHDSSIANLADQVYEITVDGILTPVLVQKPADDEGTKHEDSDAKYTVTDAS
33_9418   RLGTTVLFATHDSSIANLADQVYEITVDGILTPVLVQKPADDEGTKHEDSDAKYTVTDAS
34_1782   RLGTTVLFATHDSSIANLADQVYEITVDGILTPVLVQKPADDEGTKHEDSDAKYTVTDAS
35_10360  RLGTTVLFATHDSSIANLADQVYEITVDGILTPVLVQKPADDEGTKHEDSDAKYTVTDAS
36_468    RLGTTVLFATHDSSIANLADQVYEITVDGILTPVLVQKPADDEGTKHEDSDAKYTVTDAS
37_4956   RLGTTVLFATHDSSIANLADQVYEITVDGILTPVLVQKPADDEGTKHEDSDAKYTVTDAS
38_4305   RLGTTVLFATHDSSIANLADQVYEITVDGILTPVLVQKPADDEGTKHEDSDAKYTVTDAS
39_4737   RLGTTVLFATHDSSIANLADQVYEITVDGILTPVLVQKPADDEGTKHEDSDAKYTVTDAS
40_9543   RLGTTVLFATHDSSIANLADQVYEITVDGILTPVLVQKPADDEGTKHEDSDAKYTVTDAS
41_6669   RLGTTVLFATHDSSIANLADQVYEITVDGILTPVLVQKPADDEGTKHEDSDAKYTVTDAS
42_10558  RLGTTVLFATHDSSIANLADQVYEITVDGILTPVLVQKPADDEGTKHEDSDAKYTVTDAS
43_8051   RLGTTVLFATHDSSIANLADQVYEITVDGILTPVLVQKPADDEGTKHEDSDAKYTVTDAS
44_10454  RLGTTVLFATHDSSIANLADQVYEITVDGILTPVLVQKPADDEGTKHEDSDAKYTVTDAS
45_10126  RLGTTVLFATHDSSIANLADQVYEITVDGILTPVLVQKPADDEGTKHEDSDAKYTVTDAS
46_1547   RLGTTVLFATHDSSIANLADQVYEITVDGILTPVLVQKPADDEGTKHEDSDAKYTVTDAS
47_6749   RLGTTVLFATHDSSIANLADQVYEITVDGILTPVLVQKPADDEGTKHEDSDAKYTVTDAS
48_9982   RLGTTVLFATHDSSIANLADQVYEITVDGILTPVLVQKPADDEGTKHEDSDAKYTVTDAS
49_7293   RLGTTVLFATHDSSIANLADQVYEITVDGILTPVLVQKPADDEGTKHEDSDAKYTVTDAS
50_5993   RLGTTVLFATHDSSIANLADQVYEITVDGILTPVLVQKPADDEGTKHEDSDAKYTVTDAS
51_4791   RLGTTVLFATHDSSIANLADQVYEITVDGILTPVLVQKPADDEGTKHEDSDAKYTVTDAS
52_121    RLGTTVLFATHDSSIANLADQVYEITVDGILTPVLVQKPADDEGTKHEDSDAKYTVTDAS
53_1667   RLGTTVLFATHDSSIANLADQVYEITVDGILTPVLVQKPADDEGTKHEDSDAKYTVTDAS
54_10488  RLGTTVLFATHDSSIANLADQVYEITVDGILTPVLVQKPADDEGTKHEDSDAKYTVTDAS
55_7733   RLGTTVLFATHDSSIANLADQVYEITVDGILTPVLVQKPADDEGTKHEDSDAKYTVTDAS
56_3982   RLGTTVLFATHDSSIANLADQVYEITVDGILTPVLVQKPADDEGTKHEDSDAKYTVTDAS
57_392    RLGTTVLFATHDSSIANLADQVYEITVDGILTPVLVQKPADDEGTKHEDSDAKYTVTDAS
58_10291  RLGTTVLFATHDSSIANLADQVYEITVDGILTPVLVQKPADDEGTKHEDSDAKYTVTDAS
59_5792   RLGTTVLFATHDSSIANLADQVYEITVDGILTPVLVQKPADDEGTKHEDSDAKYTVTDAS
60_8712   RLGTTVLFATHDSSIANLADQVYEITVDGILTPVLVQKPADDEGTKHEDSDAKYTVTDAS
61_6716   RLGTTVLFATHDSSIANLADQVYEITVDGILTPVLVQKPADDEGTKHEDSDAKYTVTDAS
62_5599   RLGTTVLFATHDSSIANLADQVYEITVDGILTPVLVQKPADDEGTKHEDSDAKYTVTDAS
63_304    RLGTTVLFATHDSSIANLADQVYEITVDGILTPVLVQKPADDEGTKHEDSDAKYTVTDAS
64_10473  ............................................................
65_6310   RLGTTVLFATHDSSIANLADQVYEITVDGILTPVLVQKPADDEGTKHEDSDAKYTVTDAS
66_8714   RLGTTVLFATHDSSIANLADQVYEITVDGILTPVLVQKPADDEGTKHEDSDAKYTVTDAS
67_2350   RLGTTVLFATHDSSIANLADQVYEITVDGILTPVLVQKPADDEGTKHEDSDAKYTVTDAS
68_2447   RLGTTVLFATHDSSIANLADQVYEITVDGILTPVLVQKPADDEGTKHEDSDAKYTVTDAS
69_5658   RLGTTVLFATHDSSIANLADQVYEITVDGILTPVLVQKPADDEGTKHEDSDAKYTVTDAS
70_1277   RLGTTVLFATHDSSIANLADQVYEITVDGILTPVLVQKPADDEGTKHEDSDAKYTVTDAS
71_3717   RLGTTVLFATHDSSIANLADQVYEITVDGILTPVLVQKPADDEGTKHEDSDAKYTVTDAS
72_7846   RLGTTVLFATHDSSIANLADQVYEITVDGILTPVLVQKPADDEGTKHEDSDAKYTVTDAS
73_10528  RLGTTVLFATHDSSIANLADQVYEITVDGILTPVLVQKPADDEGTKHEDSDAKYTVTDAS
74_4899   RLGTTVLFATHDSSIANLADQVYEITVDGILTPVLVQKPADDEGTKHEDSDAKYTVTDAS
75_5507   RLGTTVLFATHDSSIANLADQVYEITVDGILTPVLVQKPADDEGTKHEDSDAKYTVTDAS
76_5432   RLGTTVLFATHDSSIANLADQVYEITVDGILTPVLVQKPADDEGTKHEDSDAKYTVTDAS
77_10770  RLGTTVLFATHDSSIANLADQVYEITVDGILTPVLVQKPADDEGTKHEDSDAKYTVTDAS
78_5904   RLGTTVLFATHDSSIANLADQVYEITVDGILTPVLVQKPADDEGTKHEDSDAKYTVTDAS
79_9653   RLGTTVLFATHDSSIANLADQVYEITVDGILTPVLVQKPADDEGTKHEDSDAKYTVTDAS
80_4462   RLGTTVLFATHDSSIANLADQVYEITVDGILTPVLVQKPADDEGTKHEDSDAKYTVTDAS
81_7194   PVLVQKPADDEGTKHEDSDAKYTVTDASNDEKTSTAPDHGTNTVMTHKVENGGEATGTSV
82_1022   RLGTTVLFATHDSSIANLADQVYEITVDGILTPVLVQKPADDEGTKHEDSDAKYTVTDAS
83_9813   RLGTTVLFATHDSSIANLADQVYEITVDGILTPVLVQKPADDEGTKHEDSDAKYTVTDAS
84_1902   RLGTTVLFATHDSSIANLADQVYEITVDGILTPVLVQKPADDEGTKHEDSDAKYTVTDAS
85_5152   RLGTTVLFATHDSSIANLADQVYEITVDGILTPVLVQKPADDEGTKHEDSDAKYTVTDAS
86_3385   RLGTTVLFATHDSSIANLADQVYEITVDGILTPVLVQKPADDEGTKHEDSDAKYTVTDAS
87_10760  RLGTTVLFATHDSSIANLADQVYEITVDGILTPVLVQKPADDEGTKHEDSDAKYTVTDAS
88_1588   RLGTTVLFATHDSSIANLADQVYEITVDGILTPVLVQKPADDEGTKHEDSDAKYTVTDAS
89_1609   RLGTTVLFATHDSSIANLADQVYEITVDGILTPVLVQKPADDEGTKHEDSDAKYTVTDAS
90_4023   RLGTTVLFATHDSSIANLADQVYEITVDGILTPVLVQKPADDEGTKHEDSDAKYTVTDAS
```

```
                850       860       870       880       890       900
                  .         .         .         .         .         .
1_4160    NDEKTSTAPDHGTNTVMTHKVENGGEATGTSVSDKKVYLRYARAMGFKNAATFLFLVMGC
2_6143    NDEKTSTAPDHGTNTVMTHKVENGGEATGTSVSDKKVYLRYARAMGFKNAATFLFLVMGC
3_6554    NDEKTSTAPDHGTNTVMTHKVENGGEATGTSVSDKKVYLRYARAMGFKNAATFLFLVMGC
4_111     NDEKTSTAPDHGTNTVMTHKVENGGEATGTSVSDKKVYLRYARAMGFKNAATFLFLVMGC
5_5742    NDEKTSTAPDHGTNTVMTHKVENGGEATGTSVSDKKVYLRYARAMGFKNAATFLFLVMGC
6_9490    NDEKTSTAPDHGTNTVMTHKVENGGEATGTSVSDKKVYLRYARAMGFKNAATFLFLVMGC
7_6313    GGIILLDEATSNLDRQTEVHLESVMAARLQHATVVSVMHRLEAVAAYDKVAILDKGVLVD
8_2552    NDEKTSTAPDHGTNTVMTHKVENGGEATGTSVSDKKVYLRYARAMGFKNAATFLFLVMGC
9_7745    NDEKTSTAPDHGTNTVMTHKVENGGEATGTSVSDKKVYLRYARAMGFKNAATFLFLVMGC
10_2315   NDEKTSTAPDHGTNTVMTHKVENGGEATGTSVSDKKVYLRYARAMGFKNAATFLFLVMGC
11_2035   NDEKTSTAPDHGTNTVMTHKVENGGEATGTSVSDKKVYLRYARAMGFKNAATFLFLVMGC
12_9640   NDEKTSTAPDHGTNTVMTHKVENGGEATGTSVSDKKVYLRYARAMGFKNAATFLFLVMGC
13_3674   NDEKTSTAPDHGTNTVMTHKVENGGEATGTSVSDKKVYLRYARAMGFKNAATFLFLVMGC
14_6548   NDEKTSTAPDHGTNTVMTHKVENGGEATGTSVSDKKVYLRYARAMGFKNAATFLFLVMGC
15_10828  NDEKTSTAPDHGTNTVMTHKVENGGEATGTSVSDKKVYLRYARAMGFKNAATFLFLVMGC
16_6578   NDEKTSTAPDHGTNTVMTHKVENGGEATGTSVSDKKVYLRYARAMGFKNAATFLFLVMGC
17_10233  NDEKTSTAPDHGTNTVMTHKVENGGEATGTSVSDKKVYLRYARAMGFKNAATFLFLVMGC
18_9261   NDEKTSTAPDHGTNTVMTHKVENGGEATGTSVSDKKVYLRYARAMGFKNAATFLFLVMGC
19_8450   NDEKTSTAPDHGTNTVMTHKVENGGEATGTSVSDKKVYLRYARAMGFKNAATFLFLVMGC
20_1727   NDEKTSTAPDHGTNTVMTHKVENGGEATGTSVSDKKVYLRYARAMGFKNAATFLFLVMGC
21_206    NDEKTSTAPDHGTNTVMTHKVENGGEATGTSVSDKKVYLRYARAMGFKNAATFLFLVMGC
22_7439   NDEKTSTAPDHGTNTVMTHKVENGGEATGTSVSDKKVYLRYARAMGFKNAATFLFLVMGC
23_3780   NDEKTSTAPDHGTNTVMTHKVENGGEATGTSVSDKKVYLRYARAMGFKNAATFLFLVMGC
24_967    NDEKTSTAPDHGTNTVMTHKVENGGEATGTSVSDKKVYLRYARAMGFKNAATFLFLVMGC
25_6595   NDEKTSTAPDHGTNTVMTHKVENGGEATGTSVSDKKVYLRYARAMGFKNAATFLFLVMGC
26_8391   NDEKTSTAPDHGTNTVMTHKVENGGEATGTSVSDKKVYLRYARAMGFKNAATFLFLVMGC
27_8170   NDEKTSTAPDHGTNTVMTHKVENGGEATGTSVSDKKVYLRYARAMGFKNAATFLFLVMGC
28_5209   NDEKTSTAPDHGTNTVMTHKVENGGEATGTSVSDKKVYLRYARAMGFKNAATFLFLVMGC
29_2181   NDEKTSTAPDHGTNTVMTHKVENGGEATGTSVSDKKVYLRYARAMGFKNAATFLFLVMGC
30_8564   NDEKTSTAPDHGTNTVMTHKVENGGEATGTSVSDKKVYLRYARAMGFKNAATFLFLVMGC
31_1582   NDEKTSTAPDHGTNTVMTHKVENGGEATGTSVSDKKVYLRYARAMGFKNAATFLFLVMGC
32_4537   NDEKTSTAPDHGTNTVMTHKVENGGEATGTSVSDKKVYLRYARAMGFKNAATFLFLVMGC
33_9418   NDEKTSTAPDHGTNTVMTHKVENGGEATGTSVSDKKVYLRYARAMGFKNAATFLFLVMGC
34_1782   NDEKTSTAPDHGTNTVMTHKVENGGEATGTSVSDKKVYLRYARAMGFKNAATFLFLVMGC
35_10360  NDEKTSTAPDHGTNTVMTHKVENGGEATGTSVSDKKVYLRYARAMGFKNAATFLFLVMGC
36_468    NDEKTSTAPDHGTNTVMTHKVENGGEATGTSVSDKKVYLRYARAMGFKNAATFLFLVMGC
37_4956   NDEKTSTAPDHGTNTVMTHKVENGGEATGTSVSDKKVYLRYARAMGFKNAATFLFLVMGC
38_4305   NDEKTSTAPDHGTNTVMTHKVENGGEATGTSVSDKKVYLRYARAMGFKNAATFLFLVMGC
39_4737   NDEKTSTAPDHGTNTVMTHKVENGGEATGTSVSDKKVYLRYARAMGFKNAATFLFLVMGC
40_9543   NDEKTSTAPDHGTNTVMTHKVENGGEATGTSVSDKKVYLRYARAMGFKNAATFLFLVMGC
41_6669   NDEKTSTAPDHGTNTVMTHKVENGGEATGTSVSDKKVYLRYARAMGFKNAATFLFLVMGC
42_10558  NDEKTSTAPDHGTNTVMTHKVENGGEATGTSVSDKKVYLRYARAMGFKNAATFLFLVMGC
43_8051   NDEKTSTAPDHGTNTVMTHKVENGGEATGTSVSDKKVYLRYARAMGFKNAATFLFLVMGC
44_10454  NDEKTSTAPDHGTNTVMTHKVENGGEATGTSVSDKKVYLRYARAMGFKNAATFLFLVMGC
45_10126  NDEKTSTAPDHGTNTVMTHKVENGGEATGTSVSDKKVYLRYARAMGFKNAATFLFLVMGC
46_1547   NDEKTSTAPDHGTNTVMTHKVENGGEATGTSVSDKKVYLRYARAMGFKNAATFLFLVMGC
47_6749   NDEKTSTAPDHGTNTVMTHKVENGGEATGTSVSDKKVYLRYARAMGFKNAATFLFLVMGC
48_9982   NDEKTSTAPDHGTNTVMTHKVENGGEATGTSVSDKKVYLRYARAMGFKNAATFLFLVMGC
49_7293   NDEKTSTAPDHGTNTVMTHKVENGGEATGTSVSDKKVYLRYARAMGFKNAATFLFLVMGC
50_5993   NDEKTSTAPDHGTNTVMTHKVENGGEATGTSVSDKKVYLRYARAMGFKNAATFLFLVMGC
51_4791   NDEKTSTAPDHGTNTVMTHKVENGGEATGTSVSDKKVYLRYARAMGFKNAATFLFLVMGC
52_121    NDEKTSTAPDHGTNTVMTHKVENGGEATGTSVSDKKVYLRYARAMGFKNAATFLFLVMGC
53_1667   NDEKTSTAPDHGTNTVMTHKVENGGEATGTSVSDKKVYLRYARAMGFKNAATFLFLVMGC
54_10488  NDEKTSTAPDHGTNTVMTHKVENGGEATGTSVSDKKVYLRYARAMGFKNAATFLFLVMGC
55_7733   NDEKTSTAPDHGTNTVMTHKVENGGEATGTSVSDKKVYLRYARAMGFKNAATFLFLVMGC
56_3982   NDEKTSTAPDHGTNTVMTHKVENGGEATGTSVSDKKVYLRYARAMGFKNAATFLFLVMGC
57_392    NDEKTSTAPDHGTNTVMTHKVENGGEATGTSVSDKKVYLRYARAMGFKNAATFLFLVMGC
58_10291  NDEKTSTAPDHGTNTVMTHKVENGGEATGTSVSDKKVYLRYARAMGFKNAATFLFLVMGC
59_5792   NDEKTSTAPDHGTNTVMTHKVENGGEATGTSVSDKKVYLRYARAMGFKNAATFLFLVMGC
60_8712   NDEKTSTAPDHGTNTVMTHKVENGGEATGTSVSDKKVYLRYARAMGFKNAATFLFLVMGC
61_6716   NDEKTSTAPDHGTNTVMTHKVENGGEATGTSVSDKKVYLRYARAMGFKNAATFLFLVMGC
62_5599   NDEKTSTAPDHGTNTVMTHKVENGGEATGTSVSDKKVYLRYARAMGFKNAATFLFLVMGC
63_304    NDEKTSTAPDHGTNTVMTHKVENGGEATGTSVSDKKVYLRYARAMGFKNAATFLFLVMGC
64_10473  ............................................................
65_6310   NDEKTSTAPDHGTNTVMTHKVENGGEATGTSVSDKKVYLRYARAMGFKNAATFLFLVMGC
66_8714   NDEKTSTAPDHGTNTVMTHKVENGGEATGTSVSDKKVYLRYARAMGFKNAATFLFLVMGC
67_2350   NDEKTSTAPDHGTNTVMTHKVENGGEATGTSVSDKKVYLRYARAMGFKNAATFLFLVMGC
68_2447   NDEKTSTAPDHGTNTVMTHKVENGGEATGTSVSDKKVYLRYARAMGFKNAATFLFLVMGC
69_5658   NDEKTSTAPDHGTNTVMTHKVENGGEATGTSVSDKKVYLRYARAMGFKNAATFLFLVMGC
70_1277   NDEKTSTAPDHGTNTVMTHKVENGGEATGTSVSDKKVYLRYARAMGFKNAATFLFLVMGC
71_3717   NDEKTSTAPDHGTNTVMTHKVENGGEATGTSVSDKKVYLRYARAMGFKNAATFLFLVMGC
72_7846   NDEKTSTAPDHGTNTVMTHKVENGGEATGTSVSDKKVYLRYARAMGFKNAATFLFLVMGC
73_10528  NDEKTSTAPDHGTNTVMTHKVENGGEATGTSVSDKKVYLRYARAMGFKNAATFLFLVMGC
74_4899   NDEKTSTAPDHGTNTVMTHKVENGGEATGTSVSDKKVYLRYARAMGFKNAATFLFLVMGC
75_5507   NDEKTSTAPDHGTNTVMTHKVENGGEATGTSVSDKKVYLRYARAMGFKNAATFLFLVMGC
76_5432   NDEKTSTAPDHGTNTVMTHKVENGGEATGTSVSDKKVYLRYARAMGFKNAATFLFLVMGC
77_10770  NDEKTSTAPDHGTNTVMTHKVENGGEATGTSVSDKKVYLRYARAMGFKNAATFLFLVMGC
78_5904   NDEKTSTAPDHGTNTVMTHKVENGGEATGTSVSDKKVYLRYARAMGFKNAATFLFLVMGC
79_9653   NDEKTSTAPDHGTNTVMTHKVENGGEATGTSVSDKKVYLRYARAMGFKNAATFLFLVMGC
80_4462   NDEKTSTAPDHGTNTVMTHKVENGGEATGTSVSDKKVYLRYARAMGFKNAATFLFLVMGC
81_7194   SDKKVYLRYARAMGFKNAATFLFLVMGCAVCFKIPDLWVQWWSTAIKQGTTYSSSYWIGI
82_1022   NDEKTSTAPDHGTNTVMTHKVENGGEATGTSVSDKKVYLRYARAMGFKNAATFLFLVMGC
83_9813   NDEKTSTAPDHGTNTVMTHKVENGGEATGTSVSDKKVYLRYARAMGFKNAATFLFLVMGC
84_1902   NDEKTSTAPDHGTNTVMTHKVENGGEATGTSVSDKKVYLRYARAMGFKNAATFLFLVMGC
85_5152   NDEKTSTAPDHGTNTVMTHKVENGGEATGTSVSDKKVYLRYARAMGFKNAATFLFLVMGC
86_3385   NDEKTSTAPDHGTNTVMTHKVENGGEATGTSVSDKKVYLRYARAMGFKNAATFLFLVMGC
87_10760  NDEKTSTAPDHGTNTVMTHKVENGGEATGTSVSDKKVYLRYARAMGFKNAATFLFLVMGC
88_1588   NDEKTSTAPDHGTNTVMTHKVENGGEATGTSVSDKKVYLRYARAMGFKNAATFLFLVMGC
89_1609   NDEKTSTAPDHGTNTVMTHKVENGGEATGTSVSDKKVYLRYARAMGFKNAATFLFLVMGC
90_4023   NDEKTSTAPDHGTNTVMTHKVENGGEATGTSVSDKKVYLRYARAMGFKNAATFLFLVMGC
```

```
                   910       920       930       940       950       960
                     .         .         .         .         .         .
1_4160    AVCFKIPDLWVQWWSTAIKQGTTYSSSYWIGILALLEVLPLLMLWLSLFHVLFFIVPRSA
2_6143    AVCFKIPDLWVQWWSTAIKQGTTYSSSYWIGILALLEVLPLLMLWLSLFHVLFFIVPRSA
3_6554    AVCFKIPDLWVQWWSTAIKQGTTYSSSYWIGILALLEVLPLLMLWLSLFHVLFFIVPRSA
4_111     AVCFKIPDLWVQWWSTAIKQGTTYSSSYWIGILALLEVLPLLMLWLSLFHVLFFIVPRSA
5_5742    AVCFKIPDLWVQWWSTAIKQGTTYSSSYWIGILALLEVLPLLMLWLSLFHVLFFIVPRSA
6_9490    AVCFKIPDLWVQWWSTAIKQGTTYSSSYWIGILALLEVLPLLMLWLSLFHVLFFIVPRSA
7_6313    FGPVADVMARCELFGA............................................
8_2552    AVCFKIPDLWVQWWSTAIKQGTTYSSSYWIAILALLEVLPLLMLWLSLFHVLFFIVPRSA
9_7745    AVCFKIPDLWVQWWSTAIKQGTTYSSSYWIGILALLEVLPLLMLWLSLFHVLFFIVPRSA
10_2315   AVCFKIPDLWVQWWSTAIKQGTTYSSSYWIGILALLEVLPLLMLWLSLFHVLFFIVPRSA
11_2035   AVCFKIPDLWVQWWSTAIKQGTTYSSSYWIGILALLEVLPLLMLWLSLFHVLFFIVPRSA
12_9640   AVCFKIPDLWVQWWSTAIKQGTTYSSSYWIGILALLEVLPLLMLWLSLFHVLFFIVPRSA
13_3674   AVCFKIPDLWVQWWSTAIKQGTTYSSSYWIGILALLEVLPLLMLWLSLFHVLFFIVPRSA
14_6548   AVCFKIPDLWVQWWSTAIKQGTTYSSSYWIGILALLEVLPLLMLWLSLFHVLFFIVPRSA
15_10828  AVCFKIPDLWVQWWSTAIKQGTTYSSSYWIGILALLEVLPLLMLWLSLFHVLFFIVPRSA
16_6578   AVCFKIPDLWVQWWSTAIKQGTTYSSSYWIGILALLEVLPLLMLWLSLFHVLFFIVPRSA
17_10233  AVCFKIPDLWVQWWSTAIKQGTTYSSSYWIGILALLEVLPLLMLWLSLFHVLFFIVPRSA
18_9261   AVCFKIPDLWVQWWSTAIKQGTTYSSSYWIGILALLEVLPLLMLWLSLFHVLFFIVPRSA
19_8450   AVCFKIPDLWVQWWSTAIKQGTTYSSSYWIGILALLEVLPLLMLWLSLFHVLFFIVPRSA
20_1727   AVCFKIPDLWVQWWSTAIKQGTTYSSSYWIGILALLEVLPLLMLWLSLFHVLFFIVPRSA
21_206    AVCFKIPDLWVQWWSTAIKQGTTYSSSYWIGILALLEVLPLLMLWLSLFHVLFFIVPRSA
22_7439   AVCFKIPDLWVQWWSTAIKQGTTYSSSYWIGILALLEVLPLLMLWLSLFHVLFFIVPRSA
23_3780   AVCFKIPDLWVQWWSTAIKQGTTYSSSYWIGILALLEVLPLLMLWLSLFHVLFFIVPRSA
24_967    AVCFKIPDLWVQWWSTAIKQGTTYSSSYWIGILALLEVLPLLMLWLSLFHVLFFIVPRSA
25_6595   AVCFKIPDLWVQWWSTAIKQGTTYSSSYWIGILALLEVLPLLMLWLSLFHVLFFIVPRSA
26_8391   AVCFKIPDLWVQWWSTAIKQGTTYSSSYWIGILALLEVLPLLMLWLSLFHVLFFIVPRSA
27_8170   AVCFKIPDLWVQWWSTAIKQGTTYSSSYWIGILALLEVLPLLMLWLSLFHVLFFIVPRSA
28_5209   AVCFKIPDLWVQWWSTAIKQGTTYSSSYWIGILALLEVLPLLMLWLSLFHVLFFIVPRSA
29_2181   AVCFKIPDLWVQWWSTAIKQGTTYSSSYWIGILALLEVLPLLMLWLSLFHVLFFIVPRSA
30_8564   AVCFKIPDLWVQWWSTAIKQGTTYSSSYWIGILALLEVLPLLMLWLSLFHVLFFIVPRSA
31_1582   AVCFKIPDLWVQWWSTAIKQGTTYSSSYWIGILALLEVLPLLMLWLSLFHVLFFIVPRSA
32_4537   AVCFKIPDLWVQWWSTAIKQGTTYSSSYWIGILALLEVLPLLMLWLSLFHVLFFIVPRSA
33_9418   AVCFKIPDLWVQWWSTAIKQGTTYSSSYWIGILALLEVLPLLMLWLSLFHVLFFIVPRSA
34_1782   AVCFKIPDLWVQWWSTAIKQGTTYSSSYWIGILALLEVLPLLMLWLSLFHVLFFIVPRSA
35_10360  AVCFKIPDLWVQWWSTAIKQGTTYSSSYWIGILALLEVLPLLMLWLSLFHVLFFIVPRSA
36_468    AVCFKIPDLWVQWWSTAIKQGTTYSSSYWIGILALLEVLPLLMLWLSLFHVLFFIVPRSA
37_4956   AVCFKIPDLWVQWWSTAIKQGTTYSSSYWIGILALLEVLPLLMLWLSLFHVLFFIVPRSA
38_4305   AVCFKIPDLWVQWWSTAIKQGTTYSSSYWIGILALLEVLPLLMLWLSLFHVLFFIVPRSA
39_4737   AVCFKIPDLWVQWWSTAIKQGTTYSSSYWIGILALLEVLPLLMLWLSLFHVLFFIVPRSA
40_9543   AVCFKIPDLWVQWWSTAIKQGTTYSSSYWIGILALLEVLPLLMLWLSLFHVLFFIVPRSA
41_6669   AVCFKIPDLWVQWWSTAIKQGTTYSSSYWIGILALLEVLPLLMLWLSLFHVLFFIVPRSA
42_10558  AVCFKIPDLWVQWWSTAIKQGTTYSSSYWIGILALLEVLPLLMLWLSLFHVLFFIVPRSA
43_8051   AVCFKIPDLWVQWWSTAIKQGTTYSSSYWIGILALLEVLPLLMLWLSLFHVLFFIVPRSA
44_10454  AVCFKIPDLWVQWWSTAIKQGTTYSSSYWIGILALLEVLPLLMLWLSLFHVLFFIVPRSA
45_10126  AVCFKIPDLWVQWWSTAIKQGTTYSSSYWIGILALLEVLPLLMLWLSLFHVLFFIVPRSA
46_1547   AVCFKIPDLWVQWWSTAIKQGTTYSSSYWIGILALLEVLPLLMLWLSLFHVLFFIVPRSA
47_6749   AVCFKIPDLWVQWWSTAIKQGTTYSSSYWIGILALLEVLPLLMLWLSLFHVLFFIVPRSA
48_9982   AVCFKIPDLWVQWWSTAIKQGTTYSSSYWIGILALLEVLPLLMLWLSLFHVLFFIVPRSA
49_7293   AVCFKIPDLWVQWWSTAIKQGTTYSSSYWIGILALLEVLPLLMLWLSLFHVLFFIVPRSA
50_5993   AVCFKIPDLWVQWWSTAIKQGTTYSSSYWIGILALLEVLPLLMLWLSLFHVLFFIVPRSA
51_4791   AVCFKIPDLWVQWWSTAIKQGTTYSSSYWIGILALLEVLPLLMLWLSLFHVLFFIVPRSA
52_121    AVCFKIPDLWVQWWSTAIKQGTTYSSSYWIGILALLEVLPLLMLWLSLFHVLFFIVPRSA
53_1667   AVCFKIPDLWVQWWSTAIKQGTTYSSSYWIGILALLEVLPLLMLWLSLFHVLFFIVPRSA
54_10488  AVCFKIPDLWVQWWSTAIKQGTTYSSSYWIGILALLEVLPLLMLWLSLFHVLFFIVPRSA
55_7733   AVCFKIPDLWVQWWSTAIKQGTTYSSSYWIGILALLEVLPLLMLWLSLFHVLFFIVPRSA
56_3982   AVCFKIPDLWVQWWSTAIKQGTTYSSSYWIGILALLEVLPLLMLWLSLFHVLFFIVPRSA
57_392    AVCFKIPDLWVQWWSTAIKQGTTYSSSYWIGILALLEVLPLLMLWLSLFHVLFFIVPRSA
58_10291  AVCFKIPDLWVQWWSTAIKQGTTYSSSYWIGILALLEVLPLLMLWLSLFHVLFFIVPRSA
59_5792   AVCFKIPDLWVQWWSTAIKQGTTYSSSYWIGILALLEVLPLLMLWLSLFHVLFFIVPRSA
60_8712   AVCFKIPDLWVQWWSTAIKQGTTYSSSYWIGILALLEVLPLLMLWLSLFHVLFFIVPRSA
61_6716   AVCFKIPDLWVQWWSTAIKQGTTYSSSYWIGILALLEVLPLLMLWLSLFHVLFFIVPRSA
62_5599   AVCFKIPDLWVQWWSTAIKQGTTYSSSYWIGILALLEVLPLLMLWLSLFHVLFFIVPRSA
63_304    AVCFKIPDLWVQWWSTAIKQGTTYSSSYWIGILALLEVLPLLMLWLSLFHVLFFIVPRSA
64_10473  ............................................................
65_6310   AVCFKIPDLWVQWWSTAIKQGTTYSSSYWIGILALLEVLPLLMLWLSLFHVLFFIVPRSA
66_8714   AVCFKIPDLWVQWWSTAIKQGTTYSSSYWIGILALLEVLPLLMLWLSLFHVLFFIVPRSA
67_2350   AVCFKIPDLWVQWWSTAIKQGTTYSSSYWIGILALLEVLPLLMLWLSLFHVLFFIVPRSA
68_2447   AVCFKIPDLWVQWWSTAIKQGTTYSSSYWIGILALLEVLPLLMLWLSLFHVLFFIVPRSA
69_5658   AVCFKIPDLWVQWWSTAIKQGTTYSSSYWIGILALLEVLPLLMLWLSLFHVLFFIVPRSA
70_1277   AVCFKIPDLWVQWWSTAIKQGTTYSSSYWIGILALLEVLPLLMLWLSLFHVLFFIVPRSA
71_3717   AVCFKIPDLWVQWWSTAIKQGTTYSSSYWIGILALLEVLPLLMLWLSLFHVLFFIVPRSA
72_7846   AVCFKIPDLWVQWWSTAIKQGTTYSSSYWIGILALLEVLPLLMLWLSLFHVLFFIVPRSA
73_10528  AVCFKIPDLWVQWWSTAIKQGTTYSSSYWIGILALLEVLPLLMLWLSLFHVLFFIVPRSA
74_4899   AVCFKIPDLWVQWWSTAIKQGTTYSSSYWIGILALLEVLPLLMLWLSLFHVLFFIVPRSA
75_5507   AVCFKIPDLWVQWWSTAIKQGTTYSSSYWIGILALLEVLPLLMLWLSLFHVLFFIVPRSA
76_5432   AVCFKIPDLWVQWWSTAIKQGTTYSSSYWIGILALLEVLPLLMLWLSLFHVLFFIVPRSA
77_10770  AVCFKIPDLWVQWWSTAIKQGTTYSSSYWIGILALLEVLPLLMLWLSLFHVLFFIVPRSA
78_5904   AVCFKIPDLWVQWWSTAIKQGTTYSSSYWIGILALLEVLPLLMLWLSLFHVLFFIVPRSA
79_9653   AVCFKIPDLWVQWWSTAIKQGTTYSSSYWIGILALLEVLPLLMLWLSLFHVLFFIVPRSA
80_4462   AVCFKIPDLWVQWWSTAIKQGTTYSSSYWIGILALLEVLPLLMLWLSLFHVLFFIVPRSA
81_7194   LALLEVLPLLMLWLSLFHVLFFIVPRSASTMHDSLLRTVLLAPFGFISRVDTGSLMNRFN
82_1022   AVCFKIPDLWVQWWSTAIKQGTTYSSSYWIGILALLEVLPLLMLWLSLFHVLFFIVPRSA
83_9813   AVCFKIPDLWVQWWSTAIKQGTTYSSSYWIGILALLEVLPLLMLWLSLFHVLFFIVPRSA
84_1902   AVCFKIPDLWVQWWSTAIKQGTTYSSSYWIGILALLEVLPLLMLWLSLFHVLFFIVPRSA
85_5152   AVCFKIPDLWVQWWSTAIKQGTTYSSSYWIGILALLEVLPLLMLWLSLFHVLFFIVPRSA
86_3385   AVCFKIPDLWVQWWSTAIKQGTTYSSSYWIGILALLEVLPLLMLWLSLFHVLFFIVPRSA
87_10760  AVCFKIPDLWVQWWSTAIKQGTTYSSSYWIGILALLEVLPLLMLWLSLFHVLFFIVPRSA
88_1588   AVCFKIPDLWVQWWSTAIKQGTTYSSSYWIGILALLEVLPLLMLWLSLFHVLFFIVPRSA
89_1609   AVCFKIPDLWVQWWSTAIKQGTTYSSSYWIGILALLEVLPLLMLWLSLFHVLFFIVPRSA
90_4023   AVCFKIPDLWVQWWSTAIKQGTTYSSSYWIGILALLEVLPLLMLWLSLFHVLFFIVPRSA
```

```
                970       980       990       1000      1010      1020
1_4160    STMHDSLLRTVLLAPFGFISRVDTGSLMNRFNQDLMFVDTRLPIDLFNTSIDFFITIIQL
2_6143    STMHDSLLRTVLLAPFGFISRVDTGSLMNRFNQDLMFVDTRLPIDLFNTSIDFFITIIQL
3_6554    STMHDSLLRTVLLAPFGFISRVDTGSLMNRFNQDLMFVDTRLPIDLFNTSIDFFITIIQL
4_111     STMHDSLLRTVLLAPFGFISRVDTGSLMNRFNQDLMFVDTRLPIDLFNTSIDFFITIIQL
5_5742    STMHDSLLRTVLLAPFGFISRVDTGSLMNRFNQDLMFVDTRLPIDLFNTSIDFFITIIQL
6_9490    STMHDSLLRTVLLAPFGFISRVDTGSLMNRFNQDLMFVDTRLPIDLFNTSIDFFITIIQL
7_6313    ............................................................
8_2552    STMHDSLLRTVLLAPFGFISRVDTGSLMNRFNQDLMFVDTRLPIDLFNTSIDFFITIIQL
9_7745    STMHDSLLRTVLLAPFGFISRVDTGSLMNRFNQDLMFVDTRLPIDLFNTSIDFFITIIQL
10_2315   STMHDSLLRTVLLAPFGFISRVDTGSLMNRFNQDLMFVDTRLPIDLFNTSIDFFITIIQL
11_2035   STMHDSLLRTVLLAPFGFISRVDTGSLMNRFNQDLMFVDTRLPIDLFNTSIDFFITIIQL
12_9640   STMHDSLLRTVLLAPFGFISRVDTGSLMNRFNQDLMFVDTRLPIDLFNTSIDFFITIIQL
13_3674   STMHDSLLRTVLLAPFGFISRVDTGSLMNRFNQDLMFVDTRLPIDLFNTSIDFFITIIQL
14_6548   STMHDSLLRTVLLAPFGFISRVDTGSLMNRFNQDLMFVDTRLPIDLFNTSIDFFITIIQL
15_10828  STMHDSLLRTVLLAPFGFISRVDTGSLMNRFNQDLMFVDTRLPIDLFNTSIDFFITIIQL
16_6578   STMHDSLLRTVLLAPFGFISRVDTGSLMNRFNQDLMFVDTRLPIDLFNTSIDFFITIIQL
17_10233  STMHNSLLRTVLLAPFGFISRVDTGSLMNRFNQDLMFVDTRLPIDLFNTSIDFFITIIQL
18_9261   STMHDSLLRTVLLAPFGFISRVDTGSLMNRFNQDLMFVDTRLPIDLFNTSIDFFITIIQL
19_8450   STMHDSLLRTVLLAPFGFISRVDTGSLMNRFNQDLMFVDTRLPIDLFNTSIDFFITIIQL
20_1727   STMHDSLLRTVLLAPFGFISRVDTGSLMNRFNQDLMFVDTRLPIDLFNTSIDFFITIIQL
21_206    STMHDSLLRTVLLAPFGFISRVDTGSLMNRFNQDLMFVDTRLPIDLFNTSIDFFITIIQL
22_7439   STMHDSLLRTVLLAPFGFISRVDTGSLMNRFNQDLMFVDTRLPIDLFNTSIDFFITIIQL
23_3780   STMHDSLLRTVLLAPFGFISRVDTGSLMNRFNQDLMFVDTRLPIDLFNTSIDFFITIIQL
24_967    STMHNSLLRTVLLAPFGFISRVDTGSLMNRFNQDLMFVDTRLPIDLFNTSIDFFITIIQL
25_6595   STMHDSLLRTVLLAPFGFISRVDTGSLMNRFNQDLMFVDTRLPIDLFNTSIDFFITIIQL
26_8391   STMHDSLLRTVLLAPFGFISRVDTGSLMNRFNQDLMFVDTRLPIDLFNTSIDFFITIIQL
27_8170   STMHDSLLRTVLLAPFGFISRVDTGSLMNRFNQDLMFVDTRLPIDLFNTSIDFFITIIQL
28_5209   STMHDSLLRTVLLAPFGFISRVDTGSLMNRFNQDLMFVDTRLPIDLFNTSIDFFITIIQL
29_2181   STMHDSLLRTVLLAPFGFISRVDTGSLMNRFNQDLMFVDTRLPIDLFNTSIDFFITIIQL
30_8564   STMHDSLLRTVLLAPFGFISRVDTGSLMNRFNQDLMFVDTRLPIDLFNTSIDFFITIIQL
31_1582   STMHDSLLRTVLLAPFGFISRVDTGSLMNRFNQDLMFVDTRLPIDLFNTSIDFFITIIQL
32_4537   STMHDSLLRTVLLAPFGFISRVDTGSLMNRFNQDLMFVDTRLPIDLFNTSIDFFITIIQL
33_9418   STMHDSLLRTVLLAPFGFISRVDTGSLMNRFNQDLMFVDTRLPIDLFNTSIDFFITIIQL
34_1782   STMHDSLLRTVLLAPFGFISRVDTGSLMNRFNQDLMFVDTRLPIDLFNTSIDFFITIIQL
35_10360  STMHDSLLRTVLLAPFGFISRVDTGSLMNRFNQDLMFVDTRLPIDLFNTSIDFFITIIQL
36_468    STMHDSLLRTVLLAPFGFISRVDTGSLMNRFNQDLMFVDTRLPIDLFNTSIDFFITIIQL
37_4956   STMHDSLLRTVLLAPFGFISRVDTGSLMNRFNQDLMFVDTRLPIDLFNTSIDFFITIIQL
38_4305   STMHDSLLRTVLLAPFGFISRVDTGSLMNRFNQDLMFVDTRLPIDLFNTSIDFFITIIQL
39_4737   STMHDSLLRTVLLAPFGFISRVDTGSLMNRFNQDLMFVDTRLPIDLFNTSIDFFITIIQL
40_9543   STMHDSLLRTVLLAPFGFISRVDTGSLMNRFNQDLMFVDTRLPIDLFNTSIDFFITIIQL
41_6669   STMHDSLLRTVLLAPFGFISRVDTGSLMNRFNQDLMFVDTRLPIDLFNTSIDFFITIIQL
42_10558  STMHDSLLRTVLLAPFGFISRVDTGSLMNRFNQDLMFVDTRLPIDLFNTSIDFFITIIQL
43_8051   STMHDSLLRTVLLAPFGFISRVDTGSLMNRFNQDLMFVDTRLPIDLFNTSIDFFITIIQL
44_10454  STMHDSLLRTVLLAPFGFISRVDTGSLMNRFNQDLMFVDTRLPIDLFNTSIDFFITIIQL
45_10126  STMHDSLLRTVLLAPFGFISRVDTGSLMNRFNQDLMFVDTRLPIDLFNTSIDFFITIIQL
46_1547   STMHDSLLRTVLLAPFGFISRVDTGSLMNRFNQDLMFVDTRLPIDLFNTSIDFFITIIQL
47_6749   STMHDSLLRTVLLAPFGFISRVDTGSLMNRFNQDLMFVDTRLPIDLFNTSIDFFITIIQL
48_9982   STMHDSLLRTVLLAPFGFISRVDTGSLMNRFNQDLMFVDTRLPIDLFNTSIDFFITIIQL
49_7293   STMHDSLLRTVLLAPFGFISRVDTGSLMNRFNQDLMFVDTRLPIDLFNTSIDFFITIIQL
50_5993   STMHDSLLRTVLLAPFGFISRVDTGSLMNRFNQDLMFVDTRLPIDLFNTSIDFFITIIQL
51_4791   STMHDSLLRTVLLAPFGFISRVDTGSLMNRFNQDLMFVDTRLPIDLFNTSIDFFITIIQL
52_121    STMHDSLLRTVLLAPFGFISRVDTGSLMNRFNQDLMFVDTRLPIDLFNTSIDFFITIIQL
53_1667   STMHDSLLRTVLLAPFGFISRVDTGSLMNRFNQDLMFVDTRLPIDLFNTSIDFFITIIQL
54_10488  STMHDSLLRTVLLAPFGFISRVDTGSLMNRFNQDLMFVDTRLPIDLFNTSIDFFITIIQL
55_7733   STMHDSLLRTVLLAPFGFISRVDTGSLMNRFNQDLMFVDTRLPIDLFNTSIDFFITIIQL
56_3982   STMHDSLLRTVLLAPFGFISRVDTGSLMNRFNQDLMFVDTRLPIDLFNTSIDFFITIIQL
57_392    STMHDSLLRTVLLAPFGFISRVDTGSLMNRFNQDLMFVDTRLPIDLFNTSIDFFITIIQL
58_10291  STMHDSLLRTVLLAPFGFISRVDTGSLMNRFNQDLMFVDTRLPIDLFNTSIDFFITIIQL
59_5792   STMHDSLLRTVLLAPFGFISRVDTGSLMNRFNQDLMFVDTRLPIDLFNTSIDFFITIIQL
60_8712   STMHDSLLRTVLLAPFGFISRVDTGSLMNRFNQDLMFVDTRLPIDLFNTSIDFFITIIQL
61_6716   STMHDSLLRTVLLAPFGFISRVDTGSLMNRFNQDLMFVDTRLPIDLFNTSIDFFITIIQL
62_5599   STMHDSLLRTVLLAPFGFISRVDTGSLMNRFNQDLMFVDTRLPIDLFNTSIDFFITIIQL
63_304    STMHDSLLRTVLLAPFGFISRVDTGSLMNRFNQDLMFVDTRLPIDLFNTSIDFFITIIQL
64_10473  ............................................................
65_6310   STMHDSLLRTVLLAPFGFISRVDTGSLMNRFNQDLMFVDTRLPIDLFNTSIDFFITIIQL
66_8714   STMHDSLLRTVLLAPFGFISRVDTGSLMNRFNQDLMFVDTRLPIDLFNTSIDFFITIIQL
67_2350   STMHDSLLRTVLLAPFGFISRVDTGSLMNRFNQDLMFVDTRLPIDLFNTSIDFFITIIQL
68_2447   STMHDSLLRTVLLAPFGFISRVDTGSLMNRFNQDLMFVDTRLPIDLFNTSIDFFITIIQL
69_5658   STMHDSLLRTVLLAPFGFISRVDTGSLMNRFNQDLMFVDTRLPIDLFNTSIDFFITIIQL
70_1277   STMHDSLLRTVLLAPFGFISRVDTGSLMNRFNQDLMFVDTRLPIDLFNTSIDFFITIIQL
71_3717   STMHDSLLRTVLLAPFGFISRVDTGSLMNRFNQDLMFVDTRLPIDLFNTSIDFFITIIQL
72_7846   STMHDSLLRTVLLAPFGFISRVDTGSLMNRFNQDLMFVDTRLPIDLFNTSIDFFITIIQL
73_10528  STMHDSLLRTVLLAPFGFISRVDTGSLMNRFNQDLMFVDTRLPIDLFNTSIDFFITIIQL
74_4899   STMHDSLLRTVLLAPFGFISRVDTGSLMNRFNQDLMFVDTRLPIDLFNTSIDFFITIIQL
75_5507   STMHDSLLRTVLLAPFGFISRVDTGSLMNRFNQDLMFVDTRLPIDLFNTSIDFFITIIQL
76_5432   STMHDSLLRTVLLAPFGFISRVDTGSLMNRFNQDLMFVDTRLPIDLFNTSIDFFITIIQL
77_10770  STMHDSLLRTVLLAPFGFISRVDTGSLMNRFNQDLMFVDTRLPIDLFNTSIDFFITIIQL
78_5904   STMHDSLLRTVLLAPFGFISRVDTGSLMNRFNQDLMFVDTRLPIDLFNTSIDFFITIIQL
79_9653   STMHDSLLRTVLLAPFGFISRVDTGSLMNRFNQDLMFVDTRLPIDLFNTSIDFFITIIQL
80_4462   STMHDSLLRTVLLAPFGFISRVDTGSLMNRFNQDLMFVDTRLPIDLFNTSIDFFITIIQL
81_7194   QDLMFVDTRLPIDLFNTSIDFFITIIQLILVVLVSKEALAILPVVFGALYLIQKVYLRSS
82_1022   STMHDSLLRTVLLAPFGFISRVDTGSLMNRFNQDLMFVDTRLPIDLFNTSIDFFITIIQL
83_9813   STMHDSLLRTVLLAPFGFISRVDTGSLMNRFNQDLMFVDTRLPIDLFNTSIDFFITIIQL
84_1902   STMHDSLLRTVLLAPFGFISRVDTGSLMNRFNQDLMFVDTRLPIDLFNTSIDFFITIIQL
85_5152   STMHDSLLRTVLLAPFGFISRVDTGSLMNRFNQDLMFVDTRLPIDLFNTSIDFFITIIQL
86_3385   STMHDSLLRTVLLAPFGFISRVDTGSLMNRFNQDLMFVDTRLPIDLFNTSIDFFITIIQL
87_10760  STMHDSLLRTVLLAPFGFISRVDTGSLMNRFNQDLMFVDTRLPIDLFNTSIDFFITIIQL
88_1588   STMHDSLLRTVLLAPFGFISRVDTGSLMNRFNQDLMFVDTRLPIDLFNTSIDFFITIIQL
89_1609   STMHDSLLRTVLLAPFGFISRVDTGSLMNRFNQDLMFVDTRLPIDLFNTSIDFFITIIQL
90_4023   STMHDSLLRTVLLAPFGFISRVDTGSLMNRFNQDLMFVDTRLPIDLFNTSIDFFITIIQL
```

```
                  1030      1040      1050      1060      1070      1080
                     .         .         .         .         .         .
1_4160      ILVVLVSKEALAILPVVFGALYLIQKVYLRSSKQLRLLDLDWKADLHTAFGETTAGLSVI
2_6143      ILVVLVSKEALAILPVVFGALYLIQKVYLRSSKQLRLLDLDWKADLHTAFGETTAGLSVI
3_6554      ILVVLVSKEALAILPVVFGALYLIQKVYLRSSKQLRLLDLDWKADLHTAFGETTAGLSVI
4_111       ILVVLVSKEALAILPVVFGALYLIQKVYLRSSKQLRLLDLDWKADLHTAFGETTAGLSVI
5_5742      ILVVLVSKEALAILPVVFGALYLIQKVYLRSSKQLRLLDLDWKADLHTAFGETTAGLSVI
6_9490      ILVVLVSKEALAILPVVFGALYLIQKVYLRSSKQLRLLDLDWKADLHTAFGETTAGLSVI
7_6313      ............................................................
8_2552      ILVVLVSKEALAILPVVFGALYLIQKVYLRSSKQLRLLDLDWKADLHTAFGETTAGLSVI
9_7745      ILVVLVSKEALAILPVVFGALYLIQKVYLRSSKQLRLLDLDWKADLHTAFGETTAGLSVI
10_2315     ILVVLVSKEALAILPVVFGALYLIQKVYLRSSKQLRLLDLDWKADLHTAFGETTAGLSVI
11_2035     ILVVLVSKEALAILPVVFGALYLIQKVYLRSSKQLRLLDLDWKADLHTAFGETTAGLSVI
12_9640     ILVVLVSKEALAILPVVFGALYLIQKVYLRSSKQLRLLDLDWKADLHTAFGETTAGLSVI
13_3674     ILVVLVSKEALAILPVVFGALYLIQKVYLRSSKQLRLLDLDWKADLHTAFGETTAGLSVI
14_6548     ILVVLVSKEALAILPVVFGALYLIQKVYLRSSKQLRLLDLDWKADLHTAFGETTAGLSVI
15_10828    ILVVLVSKEALAILPVVFGALYLIQKVYLRSSKQLRLLDLDWKADLHTAFGETTAGLSVI
16_6578     ILVVLVSKEALAILPVVFGALYLIQKVYLRSSKQLRLLDLDWKADLHTAFGETTAGLSVI
17_10233    ILVVLVSKEALAILPVVFGALYLIQKVYLRSSKQLRLLDLDWKADLHTAFGETTAGLSVI
18_9261     ILVVLVSKEALAILPVVFGALYLIQKVYLRSSKQLRLLDLDWKADLHTAFGETTAGLSVI
19_8450     ILVVLVSKEALAILPVVFGALYLIQKVYLRSSKQLRLLDLDWKADLHTAFGETTAGLSVI
20_1727     ILVVLVSKEALAILPVVFGALYLIQKVYLRSSKQLRLLDLDWKADLHTAFGETTAGLSVI
21_206      ILVVLVSKEALAILPVVFGALYLIQKVYLRSSKQLRLLDLDWKADLHTAFGETTAGLSVI
22_7439     ILVVLVSKEALAILPVVFGALYLIQKVYLRSSKQLRLLDLDWKADLHTAFGETTAGLSVI
23_3780     ILVVLVSKEALAILPVVFGALYLIQKVYLRSSKQLRLLDLDWKADLHTAFGETTAGLSVI
24_967      ILVVLVSKEALAILPVVFGALYLIQKVYLRSSKQLRLLDLDWKADLHTAFGETTAGLSVI
25_6595     ILVVLVSKEALAILPVVFGALYLIQKVYLRSSKQLRLLDLDWKADLHTAFGETTAGLSVI
26_8391     ILVVLVSKEALAILPVVFGALYLIQKVYLRSSKQLRLLDLDWKADLHTAFGETTAGLSVI
27_8170     ILVVLVSKEALAILPVVFGALYLIQKVYLRSSKQLRLLDLDWKADLHTAFGETTAGLSVI
28_5209     ILVVLVSKEALAILPVVFGALYLIQKVYLRSSKQLRLLDLDWKADLHTAFGETTAGLSVI
29_2181     ILVVLVSKEALAILPVVFGALYLIQKVYLRSSKQLRLLDLDWKADLHTAFGETTAGLSVI
30_8564     ILVVLVSKEALAILPVVFGALYLIQKVYLRSSKQLRLLDLDWKADLHTAFGETTAGLSVI
31_1582     ILVVLVSKEALAILPVVFGALYLIQKVYLRSSKQLRLLDLDWKADLHTAFGETTAGLSVI
32_4537     ILVVLVSKEALAILPVVFGALYLIQKVYLRSSKQLRLLDLDWKADLHTAFGETTAGLSVI
33_9418     ILVVLVSKEALAILPVVFGALYLIQKVYLRSSKQLRLLDLDWKADLHTAFGETTAGLSVI
34_1782     ILVVLVSKEALAILPVVFGALYLIQKVYLRSSKQLRLLDLDWKADLHTAFGETTAGLSVI
35_10360    ILVVLVSKEALAILPVVFGALYLIQKVYLRSSKQLRLLDLDWKADLHTAFGETTAGLSVI
36_468      ILVVLVSKEALAILPVVFGALYLIQKVYLRSSKQLRLLDLDWKADLHTAFGETTAGLSVI
37_4956     ILVVLVSKEALAILPVVFGALYLIQKVYLRSSKQLRLLDLDWKADLHTAFGETTAGLSVI
38_4305     ILVVLVSKEALAILPVVFGALYLIQKVYLRSSKQLRLLDLDWKADLHTAFGETTAGLSVI
39_4737     ILVVLVSKEALAILPVVFGALYLIQKVYLRSSKQLRLLDLDWKADLHTAFGETTAGLSVI
40_9543     ILVVLVSKEALAILPVVFGALYLIQKVYLRSSKQLRLLDLDWKADLHTAFGETTAGLSVI
41_6669     ILVVLVSKEALAILPVVFGALYLIQKVYLRSSKQLRLLDLDWKADLHTAFGETTAGLSVI
42_10558    ILVVLVSKEALAILPVVFGALYLIQKVYLRSSKQLRLLDLDWKADLHTAFGETTAGLSVI
43_8051     ILVVLVSKEALAILPVVFGALYLIQKVYLRSSKQLRLLDLDWKADLHTAFGETTAGLSVI
44_10454    ILVVLVSKEALAILPVVFGALYLIQKVYLRSSKQLRLLDLDWKADLHTAFGETTAGLSVI
45_10126    ILVVLVSKEALAILPVVFGALYLIQKVYLRSSKQLRLLDLDWKADLHTAFGETTAGLSVI
46_1547     ILVVLVSKEALAILPVVFGALYLIQKVYLRSSKQLRLLDLDWKADLHTAFGETTAGLSVI
47_6749     ILVVLVSKEALAILPVVFGALYLIQKVYLRSSKQLRLLDLDWKADLHTAFGETTAGLSVI
48_9982     ILVVLVSKEALAILPVVFGALYLIQKVYLRSSKQLRLLDLDWKADLHTAFGETTAGLSVI
49_7293     ILVVLVSKEALAILPVVFGALYLIQKVYLRSSKQLRLLDLDWKADLHTAFGETTAGLSVI
50_5993     ILVVLVSKEALAILPVVFGALYLIQKVYLRSSKQLRLLDLDWKADLHTAFGETTAGLSVI
51_4791     ILVVLVSKEALAILPVVFGALYLIQKVYLRSSKQLRLLDLDWKADLHTAFGETTAGLSVI
52_121      ILVVLVSKEALAILPVVFGALYLIQKVYLRSSKQLRLLDLDWKADLHTAFGETTAGLSVI
53_1667     ILVVLVSKEALAILPVVFGALYLIQKVYLRSSKQLRLLDLDWKADLHTAFGETTAGLSVI
54_10488    ILVVLVSKEALAILPVVFGALYLIQKVYLRSSKQLRLLDLDWKADLHTAFGETTAGLSVI
55_7733     ILVVLVSKEALAILPVVFGALYLIQKVYLRSSKQLRLLDLDWKADLHTAFGETTAGLSVI
56_3982     ILVVLVSKEALAILPVVFGALYLIQKVYLRSSKQLRLLDLDWKADLHTAFGETTAGLSVI
57_392      ILVVLVSKEALAILPVVFGALYLIQKVYLRSSKQLRLLDLDWKADLHTAFGETTAGLSVI
58_10291    ILVVLVSKEALAILPVVFGALYLIQKVYLRSSKQLRLLDLDWKADLHTAFGETTAGLSVI
59_5792     ILVVLVSKEALAILPVVFGALYLIQKVYLRSSKQLRLLDLDWKADLHTAFGETTAGLSVI
60_8712     ILVVLVSKEALAILPVVFGALYLIQKVYLRSSKQLRLLDLDWKADLHTAFGETTAGLSVI
61_6716     ILVVLVSKEALAILPVVFGALYLIQKVYLRSSKQLRLLDLDWKADLHTAFGETTAGLSVI
62_5599     ILVVLVSKEALAILPVVFGALYLIQKVYLRSSKQLRLLDLDWKADLHTAFGETTAGLSVI
63_304      ILVVLVSKEALAILPVVFGALYLIQKVYLRSSKQLRLLDLDWKADLHTAFGETTAGLSVI
64_10473    ............................................................
65_6310     ILVVLVSKEALAILPVVFGALYLIQKVYLRSSKQLRLLDLDWKADLHTAFGETTAGLSVI
66_8714     ILVVLVSKEALAILPVVFGALYLIQKVYLRSSKQLRLLDLDWKADLHTAFGETTAGLSVI
67_2350     ILVVLVSKEALAILPVVFGALYLIQKVYLRSSKQLRLLDLDWKADLHTAFGETTAGLSVI
68_2447     ILVVLVSKEALAILPVVFGALYLIQKVYLRSSKQLRLLDLDWKADLHTAFGETTAGLSVI
69_5658     ILVVLVSKEALAILPVVFGALYLIQKVYLRSSKQLRLLDLDWKADLHTAFGETTAGLSVI
70_1277     ILVVLVSKEALAILPVVFGALYLIQKVYLRSSKQLRLLDLDWKADLHTAFGETTAGLSVI
71_3717     ILVVLVSKEALAILPVVFGALYLIQKVYLRSSKQLRLLDLDWKADLHTAFGETTAGLSVI
72_7846     ILVVLVSKEALAILPVVFGALYLIQKVYLRSSKQLRLLDLDWKADLHTAFGETTAGLSVI
73_10528    ILVVLVSKEALAILPVVFGALYLIQKVYLRSSKQLRLLDLDWKADLHTAFGETTAGLSVI
74_4899     ILVVLVSKEALAILPVVFGALYLIQKVYLRSSKQLRLLDLDWKADLHTAFGETTAGLSVI
75_5507     ILVVLVSKEALAILPVVFGALYLIQKVYLRSSKQLRLLDLDWKADLHTAFGETTAGLSVI
76_5432     ILVVLVSKEALAILPVVFGALYLIQKVYLRSSKQLRLLDLDWKADLHTAFGETTAGLSVI
77_10770    ILVVLVSKEALAILPVVFGALYLIQKVYLRSSKQLRLLDLDWKADLHTAFGETTAGLSVI
78_5904     ILVVLVSKEALAILPVVFGALYLIQKVYLRSSKQLRLLDLDWKADLHTAFGETTAGLSVI
79_9653     ILVVLVSKEALAILPVVFGALYLIQKVYLRSSKQLRLLDLDWKADLHTAFGETTAGLSVI
80_4462     ILVVLVSKEALAILPVVFGALYLIQKVYLRSSKQLRLLDLDWKADLHTAFGETTAGLSVI
81_7194     KQLRLLDLDWKADLHTAFGETTAGLSVIRANGWLDPMRAKFAEKLDRSQEPFYLLYMVQR
82_1022     ILVVLVSKEALAILPVVFGALYLIQKVYLRSSKQLRLLDLDWKADLHTAFGETTAGLSVI
83_9813     ILVVLVSKEALAILPVVFGALYLIQKVYLRSSKQLRLLDLDWKADLHTAFGETTAGLSVI
84_1902     ILVVLVSKEALAILPVVFGALYLIQKVYLRSSKQLRLLDLDWKADLHTAFGETTAGLSVI
85_5152     ILVVLVSKEALAILPVVFGALYLIQKVYLRSSKQLRLLDLDWKADLHTAFGETTAGLSVI
86_3385     ILVVLVSKEALAILPVVFGALYLIQKVYLRSSKQLRLLDLDWKADLHTAFGETTAGLSVI
87_10760    ILVVLVSKEALAILPVVFGALYLIQKVYLRSSKQLRLLDLDWKADLHTAFGETTAGLSVI
88_1588     ILVVLVSKEALAILPVVFGALYLIQKVYLRSSKQLRLLDLDWKADLHTAFGETTAGLSVI
89_1609     ILVVLVSKEALAILPVVFGALYLIQKVYLRSSKQLRLLDLDWKADLHTAFGETTAGLSVI
90_4023     ILVVLVSKEALAILPVVFGALYLIQKVYLRSSKQLRLLDLDWKADLHTAFGETTAGLSVI
```

```
                   1090      1100      1110      1120      1130      1140
1_4160    RANGWLDPMRAKFAEKLDRSQEPFYLLYMVQRWLQLVLNLVVAGLAIAIAGVAIGLRDKV
2_6143    RANGWLDPMRAKFAEKLDRSQEPFYLLYMVQRWLQLVLNLVVAGLAIAIAGVAIGLRDKV
3_6554    RANGWLDPMRAKFAEKLDRSQEPFYLLYMVQRWLQLVLNLVVAGLAIAIAGVAIGLRDKV
4_111     RANGWLDPMRAKFAEKLDRSQEPFYLLYMVQRWLQLVLNLVVAGLAIAIAGVAIGLRDKV
5_5742    RANGWLDPMRAKFAEKLDRSQEPFYLLYMVQRWLQLVLNLVVAGLAIAIAGVAIGLRDKV
6_9490    RANGWLDPMRAKFAEKLDRSQEPFYLLYMVQRWLQLVLNLVVAGLAIAIAGVAIGLRDKV
7_6313    ............................................................
8_2552    RANGWLDPMRAKFAEKLDRSQEPFYLLYMVQRWLQLVLNLVVAGLAIAIAGVAIGLRDKV
9_7745    RANGWLDPMRAKFAEKLDRSQEPFYLLYMVQRWLQLVLNLVVAGLAIAIAGVAIGLRDKV
10_2315   RANGWLDPMRAKFAEKLDRSQEPFYLLYMVQRWLQLVLNLVVAGLAIAIAGVAIGLRDKV
11_2035   RANGWLDPMRAKFAEKLDRSQEPFYLLYMVQRWLQLVLNLVVAGLAIAIAGVAIGLRDKV
12_9640   RANGWLDPMRAKFAEKLDRSQEPFYLLYMVQRWLQLVLNLVVAGLAIAIAGVAIGLRDKV
13_3674   RANGWLDPMRAKFAEKLDRSQEPFYLLYMVQRWLQLVLNLVVAGLAIAIAGVAIGLRDKV
14_6548   RANGWLDPMRAKFAEKLDRSQEPFYLLYMVQRWLQLVLNLVVAGLAIAIAGVAIGLRDKV
15_10828  RANGWLDPMRAKFAEKLDRSQEPFYLLYMVQRWLQLVLNLVVAGLAIAIAGVAIGLRDKV
16_6578   RANGWLDPMRAKFAEKLDRSQEPFYLLYMVQRWLQLVLNLVVAGLAIAIAGVAIGLRDKV
17_10233  RANGWLDPMRAKFAEKLDRSQEPFYLLYMVQRWLQLVLNLVVAGLAIAIAGVAIGLRDKV
18_9261   RANGWLDPMRAKFAEKLDRSQEPFYLLYMVQRWLQLVLNLVVAGLAIAIAGVAIGLRDKV
19_8450   RANGWLDPMRAKFAEKLDRSQEPFYLLYMVQRWLQLVLNLVVAGLAIAIAGVAIGLRDKV
20_1727   RANGWLDPMRAKFAEKLDRSQEPFYLLYMVQRWLQLVLNLVVAGLAIAIAGVAIGLRDKV
21_206    RANGWLDPMRAKFAEKLDRSQEPFYLLYMVQRWLQLVLNLVVAGLAIAIAGVAIGLRDKV
22_7439   RANGWLDPMRAKFAEKLDRSQEPFYLLYMVQRWLQLVLNLVVAGLAIAIAGVAIGLRDKV
23_3780   RANGWLDPMRAKFAEKLDRSQEPFYLLYMVQRWLQLVLNLVVAGLAIAIAGVAIGLRDKV
24_967    RANGWLDPMRAKFAEKLDRSQEPFYLLYMVQRWLQLVLNLVVAGLAIAIAGVAIGLRDKV
25_6595   RANGWLDPMRAKFAEKLDRSQEPFYLLYMVQRWLQLVLNLVVAGLAIAIAGVAIGLRDKV
26_8391   RANGWLDPMRAKFAEKLDRSQEPFYLLYMVQRWLQLVLNLVVAGLAIAIAGVAIGLRDKV
27_8170   RANGWLDPMRAKFAEKLDRSQEPFYLLYMVQRWLQLVLNLVVAGLAIAIAGVAIGLRDKV
28_5209   RANGWLDPMRAKFAEKLDRSQEPFYLLYMVQRWLQLVLNLVVAGLAIAIAGVAIGLRDKV
29_2181   RANGWLDPMRAKFAEKLDRSQEPFYLLYMVQRWLQLVLNLVVAGLAIAIAGVAIGLRDKV
30_8564   RANGWLDPMRAKFAEKLDRSQEPFYLLYMVQRWLQLVLNLVVAGLAIAIAGVAIGLRDKV
31_1582   RANGWLDPMRAKFAEKLDRSQEPFYLLYMVQRWLQLVLNLVVAGLAIAIAGVAIGLRDKV
32_4537   RANGWLDPMRAKFAEKLDRSQEPFYLLYMVQRWLQLVLNLVVAGLAIAIAGVAIGLRDKV
33_9418   RANGWLDPMRAKFAEKLDRSQEPFYLLYMVQRWLQLVLNLVVAGLAIAIAGVAIGLRDKV
34_1782   RANGWLDPMRAKFAEKLDRSQEPFYLLYMVQRWLQLVLNLVVAGLAIAIAGVAIGLRDKV
35_10360  RANGWLDPMRAKFAEKLDRSQEPFYLLYMVQRWLQLVLNLVVAGLAIAIAGVAIGLRDKV
36_468    RANGWLDPMRAKFAEKLDRSQEPFYLLYMVQRWLQLVLNLVVAGLAIAIAGVAIGLRDKV
37_4956   RANGWLDPMRAKFAEKLDRSQEPFYLLYMVQRWLQLVLNLVVAGLAIAIAGVAIGLRDKV
38_4305   RANGWLDPMRAKFAEKLDRSQEPFYLLYMVQRWLQLVLNLVVAGLAIAIAGVAIGLRDKV
39_4737   RANGWLDPMRAKFAEKLDRSQEPFYLLYMVQRWLQLVLNLVVAGLAIAIAGVAIGLRDKV
40_9543   RANGWLDPMRAKFAEKLDRSQEPFYLLYMVQRWLQLVLNLVVAGLAIAIAGVAIGLRDKV
41_6669   RANGWLDPMRAKFAEKLDRSQEPFYLLYMVQRWLQLVLNLVVAGLAIAIAGVAIGLRDKV
42_10558  RANGWLDPMRAKFAEKLDRSQEPFYLLYMVQRWLQLVLNLVVAGLAIAIAGVAIGLRDKV
43_8051   RANGWLDPMRAKFAEKLDRSQEPFYLLYMVQRWLQLVLNLVVAGLAIAIAGVAIGLRDKV
44_10454  RANGWLDPMRAKFAEKLDRSQEPFYLLYMVQRWLQLVLNLVVAGLAIAIAGVAIGLRDKV
45_10126  RANGWLDPMRAKFAEKLDRSQEPFYLLYMVQRWLQLVLNLVVAGLAIAIAGVAIGLRDKV
46_1547   RANGWLDPMRAKFAEKLDRSQEPFYLLYMVQRWLQLVLNLVVAGLAIAIAGVAIGLRDKV
47_6749   RANGWLDPMRAKFAEKLDRSQEPFYLLYMVQRWLQLVLNLVVAGLAIAIAGVAIGLRDKV
48_9982   RANGWLDPMRAKFAEKLDRSQEPFYLLYMVQRWLQLVLNLVVAGLAIAIAGVAIGLRDKV
49_7293   RANGWLDPMRAKFAEKLDRSQEPFYLLYMVQRWLQLVLNLVVAGLAIAIAGVAIGLRDKV
50_5993   RANGWLDPMRAKFAEKLDRSQEPFYLLYMVQRWLQLVLNLVVAGLAIAIAGVAIGLRDKV
51_4791   RANGWLDPMRAKFAEKLDRSQEPFYLLYMVQRWLQLVLNLVVAGLAIAIAGVAIGLRDKV
52_121    RANGWLDPMRAKFAEKLDRSQEPFYLLYMVQRWLQLVLNLVVAGLAIAIAGVAIGLRDKV
53_1667   RANGWLDPMRAKFAEKLDRSQEPFYLLYMVQRWLQLVLNLVVAGLAIAIAGVAIGLRDKV
54_10488  RANGWLDPMRAKFAEKLDRSQEPFYLLYMVQRWLQLVLNLVVAGLAIAIAGVAIGLRDKV
55_7733   RANGWLDPMRAKFAEKLDRSQEPFYLLYMVQRWLQLVLNLVVAGLAIAIAGVAIGLRDKV
56_3982   RANGWLDPMRAKFAEKLDRSQEPFYLLYMVQRWLQLVLNLVVAGLAIAIAGVAIGLRDKV
57_392    RANGWLDPMRAKFAEKLDRSQEPFYLLYMVQRWLQLVLNLVVAGLAIAIAGVAIGLRDKV
58_10291  RANGWLDPMRAKFAEKLDRSQEPFYLLYMVQRWLQLVLNLVVAGLAIAIAGVAIGLRDKV
59_5792   RANGWLDPMRAKFAEKLDRSQEPFYLLYMVQRWLQLVLNLVVAGLAIAIAGVAIGLRDKV
60_8712   RANGWLDPMRAKFAEKLDRSQEPFYLLYMVQRWLQLVLNLVVAGLAIAIAGVAIGLRDKV
61_6716   RANGWLDPMRAKFAEKLDRSQEPFYLLYMVQRWLQLVLNLVVAGLAIAIAGVAIGLRDKV
62_5599   RANGWLDPMRAKFAEKLDRSQEPFYLLYMVQRWLQLVLNLVVAGLAIAIAGVAIGLRDKV
63_304    RANGWLDPMRAKFAEKLDRSQEPFYLLYMVQRWLQLVLNLVVAGLAIAIAGVAIGLRDKV
64_10473  ............................................................
65_6310   RANGWLDPMRAKFAEKLDRSQEPFYLLYMVQRWLQLVLNLVVAGLAIAIAGVAIGLRDKV
66_8714   RANGWLDPMRAKFAEKLDRSQEPFYLLYMVQRWLQLVLNLVVAGLAIAIAGVAIGLRDKV
67_2350   RANGWLDPMRAKFAEKLDRSQEPFYLLYMVQRWLQLVLNLVVAGLAIAIAGVAIGLRDKV
68_2447   RANGWLDPMRAKFAEKLDRSQEPFYLLYMVQRWLQLVLNLVVAGLAIAIAGVAIGLRDKV
69_5658   RANGWLDPMRAKFAEKLDRSQEPFYLLYMVQRWLQLVLNLVVAGLAIAIAGVAIGLRDKV
70_1277   RANGWLDPMRAKFAEKLDRSQEPFYLLYMVQRWLQLVLNLVVAGLAIAIAGVAIGLRDKV
71_3717   RANGWLDPMRAKFAEKLDRSQEPFYLLYMVQRWLQLVLNLVVAGLAIAIAGVAIGLRDKV
72_7846   RANGWLDPMRAKFAEKLDRSQEPFYLLYMVQRWLQLVLNLVVAGLAIAIAGVAIGLRDKV
73_10528  RANGWLDPMRAKFAEKLDRSQEPFYLLYMVQRWLQLVLNLVVAGLAIAIAGVAIGLRDKV
74_4899   RANGWLDPMRAKFAEKLDRSQEPFYLLYMVQRWLQLVLNLVVAGLAIAIAGVAIGLRDKV
75_5507   RANGWLDPMRAKFAEKLDRSQEPFYLLYMVQRWLQLVLNLVVAGLAIAIAGVAIGLRDKV
76_5432   RANGWLDPMRAKFAEKLDRSQEPFYLLYMVQRWLQLVLNLVVAGLAIAIAGVAIGLRDKV
77_10770  RANGWLDPMRAKFAEKLDRSQEPFYLLYMVQRWLQLVLNLVVAGLAIAIAGVAIGLRDKV
78_5904   RANGWLDPMRAKFAEKLDRSQEPFYLLYMVQRWLQLVLNLVVAGLAIAIAGVAIGLRDKV
79_9653   RANGWLDPMRAKFAEKLDRSQEPFYLLYMVQRWLQLVLNLVVAGLAIAIAGVAIGLRDKV
80_4462   RANGWLDPMRAKFAEKLDRSQEPFYLLYMVQRWLQLVLNLVVAGLAIAIAGVAIGLRDKV
81_7194   WLQLVLNLVVAGLAIAIAGVAIGLRDKVAAGAVGVALLNTTTLGETLTNFIMSWTSLETS
82_1022   RANGWLDPMRAKFAEKLDRSQEPFYLLYMVQRWLQLVLNLVVAGLAIAIAGVAIGLRDKV
83_9813   RANGWLDPMRAKFAEKLDRSQEPFYLLYMVQRWLQLVLNLVVAGLAIAIAGVAIGLRDKV
84_1902   RANGWLDPMRAKFAEKLDRSQEPFYLLYMVQRWLQLVLNLVVAGLAIAIAGVAIGLRDKV
85_5152   RANGWLDPMRAKFAEKLDRSQEPFYLLYMVQRWLQLVLNLVVAGLAIAIAGVAIGLRDKV
86_3385   RANGWLDPMRAKFAEKLDRSQEPFYLLYMVQRWLQLVLNLVVAGLAIAIAGVAIGLRDKV
87_10760  RANGWLDPMRAKFAEKLDRSQEPFYLLYMVQRWLQLVLNLVVAGLAIAIAGVAIGLRDKV
88_1588   RANGWLDPMRAKFAEKLDRSQEPFYLLYMVQRWLQLVLNLVVAGLAIAIAGVAIGLRDKV
89_1609   RANGWLDPMRAKFAEKLDRSQEPFYLLYMVQRWLQLVLNLVVAGLAIAIAGVAIGLRDKV
90_4023   RANGWLDPMRAKFAEKLDRSQEPFYLLYMVQRWLQLVLNLVVAGLAIAIAGVAIGLRDKV
```

```
                  1150      1160      1170      1180      1190      1200
                     .         .         .         .         .         .
1_4160   AAGAVGVALLNTTTLGETLTNFIMSWTSLETSLGAIARVCTFEQDTPREREEPSTTDLPD
2_6143   AAGAVGVALLNTTTLGETLTNFIMSWTSLETSLGAIARVCTFEQDTPREREEPSTTDLPD
3_6554   AAGAVGVALLNTTTLGETLTNFIMSWTSLETSLGAIARVCTFEQDTPREREEPSTTDLPD
4_111    AAGAVGVALLNTTTLGETLTNFIMSWTSLETSLGAIARVCTFEQDTPREREEPSTTDLPD
5_5742   AAGAVGVALLNTTTLGETLTNFIMSWTSLETSLGAIARVCTFEQDTPREREEPSTTDLPD
6_9490   AAGAVGVALLNTTTLGETLTNFIMSWTSLETSLGAIARVCTFEQDTPREREEPSTTDLPD
7_6313   ............................................................
8_2552   AAGAVGVALLNTTTLGETLTNFIMSWTSLETSLGAIARVCTFEQDTPREREEPSTTDLPD
9_7745   AAGAVGVALLNTTTLGETLTNFIMSWTSLETSLGAIARVCTFEQDTPREREEPSTTDLPD
10_2315  AAGAVGVALLNTTTLGETLTNFIMSWTSLETSLGAIARVCTFEQDTPREREEPSTTDLPD
11_2035  AAGAVGVALLNTTTLGETLTNFIMSWTSLETSLGAIARVCTFEQDTPREREEPSTTDLPD
12_9640  AAGAVGVALLNTTTLGETLTNFIMSWTSLETSLGAIARVCTFEQDTPREREEPSTTDLPD
13_3674  AAGAVGVALLNTTTLGETLTNFIMSWTSLETSLGAIARVCTFEQDTPREREEPSTTDLPD
14_6548  AAGAVGVALLNTTTLGETLTNFIMSWTSLETSLGAIARVCTFEQDTPREREEPSTTDLPD
15_10828 AAGAVGVALLNTTTLGETLTNFIMSWTSLETSLGAIARVCTFEQDTPREREEPSTTDLPD
16_6578  AAGAVGVALLNTTTLGETLTNFIMSWTSLETSLGAIARVCTFEQDTPREREEPSTTDLPD
17_10233 AAGAVGVALLNTTTLGETLTNFIMSWTSLETSLGAIARVCTFEQDTPREREEPSTTDLPD
18_9261  AAGAVGVALLNTTTLGETLTNFIMSWTSLETSLGAIARVCTFEQDTPREREEPSTTDLPD
19_8450  AAGAVGVALLNTTTLGETLTNFIMSWTSLETSLGAIARVCTFEQDTPREREEPSTTDLPD
20_1727  AAGAVGVALLNTTTLGETLTNFIMSWTSLETSLGAIARVCTFEQDTPREREEPSTTDLPD
21_206   AAGAVGVALLNTTTLGETLTNFIMSWTSLETSLGAIARVCTFEQDTPREREEPSTTDLPD
22_7439  AAGAVGVALLNTTTLGETLTNFIMSWTSLETSLGAIARVCTFEQDTPREREEPSTTDLPD
23_3780  AAGAVGVALLNTTTLGETLTNFIMSWTSLETSLGAIARVCTFEQDTPREREEPSTTDLPD
24_967   AAGAVGVALLNTTTLGETLTNFIMSWTSLETSLGAIARVCTFEQDTPREREEPSTTDLPD
25_6595  AAGAVGVALLNTTTLGETLTNFIMSWTSLETSLGAIARVCTFEQDTPREREEPSTTDLPD
26_8391  AAGAVGVALLNTTTLGETLTNFIMSWTSLETSLGAIARVCTFEQDTPREREEPSTTDLPD
27_8170  AAGAVGVALLNTTTLGETLTNFIMSWTSLETSLGAIARVCTFEQDTPREREEPSTTDLPD
28_5209  AAGAVGVALLNTTTLGETLTNFIMSWTSLETSLGAIARVCTFEQDTPREREEPSTTDLPD
29_2181  AAGAVGVALLNTTTLGETLTNFIMSWTSLETSLGAIARVCTFEQDTPREREEPSTTDLPD
30_8564  AAGAVGVALLNTTTLGETLTNFIMSWTSLETSLGAIARVCTFEQDTPREREEPSTTDLPD
31_1582  AAGAVGVALLNTTTLGETLTNFIMSWTSLETSLGAIARVCTFEQDTPREREEPSTTDLPD
32_4537  AAGAVGVALLNTTTLGETLTNFIMSWTSLETSLGAIARVCTFEQDTPREREEPSTTDLPD
33_9418  AAGAVGVALLNTTTLGETLTNFIMSWTSLETSLGAIARVCTFEQDTPREREEPSTTDLPD
34_1782  AAGAVGVALLNTTTLGETLTNFIMSWTSLETSLGAIARVCTFEQDTPREREEPSTTDLPD
35_10360 AAGAVGVALLNTTTLGETLTNFIMSWTSLETSLGAIARVCTFEQDTPREREEPSTTDLPD
36_468   AAGAVGVALLNTTTLGETLTNFIMSWTSLETSLGAIARVCTFEQDTPREREEPSTTDLPD
37_4956  AAGAVGVALLNTTTLGETLTNFIMSWTSLETSLGAIARVCTFEQDTPREREEPSTTDLPD
38_4305  AAGAVGVALLNTTTLGETLTNFIMSWTSLETSLGAIARVCTFEQDTPREREEPSTTDLPD
39_4737  AAGAVGVALLNTTTLGETLTNFIMSWTSLETSLGAIARVCTFEQDTPREREEPSTTDLPD
40_9543  AAGAVGVALLNTTTLGETLTNFIMSWTSLETSLGAIARVCTFEQDTPREREEPSTTDLPD
41_6669  AAGAVGVALLNTTTLGETLTNFIMSWTSLETSLGAIARVCTFEQDTPREREEPSTTDLPD
42_10558 AAGAVGVALLNTTTLGETLTNFIMSWTSLETSLGAIARVCTFEQDTPREREEPSTTDLPD
43_8051  AAGAVGVALLNTTTLGETLTNFIMSWTSLETSLGAIARVCTFEQDTPREREEPSTTDLPD
44_10454 AAGAVGVALLNTTTLGETLTNFIMSWTSLETSLGAIARVCTFEQDTPREREEPSTTDLPD
45_10126 AAGAVGVALLNTTTLGETLTNFIMSWTSLETSLGAIARVCTFEQDTPREREEPSTTDLPD
46_1547  AAGAVGVALLNTTTLGETLTNFIMSWTSLETSLGAIARVCTFEQDTPREREEPSTTDLPD
47_6749  AAGAVGVALLNTTTLGETLTNFIMSWTSLETSLGAIARVCTFEQDTPREREEPSTTDLPD
48_9982  AAGAVGVALLNTTTLGETLTNFIMSWTSLETSLGAIARVCTFEQDTPREREEPSTTDLPD
49_7293  AAGAVGVALLNTTTLGETLTNFIMSWTSLETSLGAIARVCTFEQDTPREREEPSTTDLPD
50_5993  AAGAVGVALLNTTTLGETLTNFIMSWTSLETSLGAIARVCTFEQDTPREREEPSTTDLPD
51_4791  AAGAVGVALLNTTTLGETLTNFIMSWTSLETSLGAIARVCTFEQDTPREREEPSTTDLPD
52_121   AAGAVGVALLNTTTLGETLTNFIMSWTSLETSLGAIARVCTFEQDTPREREEPSTTDLPD
53_1667  AAGAVGVALLNTTTLGETLTNFIMSWTSLETSLGAIARVCTFEQDTPREREEPSTTDLPD
54_10488 AAGAVGVALLNTTTLGETLTNFIMSWTSLETSLGAIARVCTFEQDTPREREEPSTTDLPD
55_7733  AAGAVGVALLNTTTLGETLTNFIMSWTSLETSLGAIARVCTFEQDTPREREEPSTTDLPD
56_3982  AAGAVGVALLNTTTLGETLTNFIMSWTSLETSLGAIARVCTFEQDTPREREEPSTTDLPD
57_392   AAGAVGVALLNTTTLGETLTNFIMSWTSLETSLGAIARVCTFEQDTPREREEPSTTDLPD
58_10291 AAGAVGVALLNTTTLGETLTNFIMSWTSLETSLGAIARVCTFEQDTPREREEPSTTDLPD
59_5792  AAGAVGVALLNTTTLGETLTNFIMSWTSLETSLGAIARVCTFEQDTPREREEPSTTDLPD
60_8712  AAGAVGVALLNTTTLGETLTNFIMSWTSLETSLGAIARVCTFEQDTPREREEPSTTDLPD
61_6716  AAGAVGVALLNTTTLGETLTNFIMSWTSLETSLGAIARVCTFEQDTPREREEPSTTDLPD
62_5599  AAGAVGVALLNTTTLGETLTNFIMSWTSLETSLGAIARVCTFEQDTPREREEPSTTDLPD
63_304   AAGAVGVALLNTTTLGETLTNFIMSWTSLETSLGAIARVCTFEQDTPREREEPSTTDLPD
64_10473 ............................................................
65_6310  AAGAVGVALLNTTTLGETLTNFIMSWTSLETSLGAIARVCTFEQDTPREREEPSTTDLPD
66_8714  AAGAVGVALLNTTTLGETLTNFIMSWTSLETSLGAIARVCTFEQDTPREREEPSTTDLPD
67_2350  AAGAVGVALLNTTTLGETLTNFIMSWTSLETSLGAIARVCTFEQDTPREREEPSTTDLPD
68_2447  AAGAVGVALLNTTTLGETLTNFIMSWTSLETSLGAIARVCTFEQDTPREREEPSTTDLPD
69_5658  AAGAVGVALLNTTTLGETLTNFIMSWTSLETSLGAIARVCTFEQDTPREREEPSTTDLPD
70_1277  AAGAVGVALLNTTTLGETLTNFIMSWTSLETSLGAIARVCTFEQDTPREREEPSTTDLPD
71_3717  AAGAVGVALLNTTTLGETLTNFIMSWTSLETSLGAIARVCTFEQDTPREREEPSTTDLPD
72_7846  AAGAVGVALLNTTTLGETLTNFIMSWTSLETSLGAIARVCTFEQDTPREREEPSTTDLPD
73_10528 AAGAVGVALLNTTTLGETLTNFIMSWTSLETSLGAIARVCTFEQDTPREREEPSTTDLPD
74_4899  AAGAVGVALLNTTTLGETLTNFIMSWTSLETSLGAIARVCTFEQDTPREREEPSTTDLPD
75_5507  AAGAVGVALLNTTTLGETLTNFIMSWTSLETSLGAIARVCTFEQDTPREREEPSTTDLPD
76_5432  AAGAVGVALLNTTTLGETLTNFIMSWTSLETSLGAIARVCTFEQDTPREREEPSTTDLPD
77_10770 AAGAVGVALLNTTTLGETLTNFIMSWTSLETSLGAIARVCTFEQDTPREREEPSTTDLPD
78_5904  AAGAVGVALLNTTTLGETLTNFIMSWTSLETSLGAIARVCTFEQDTPREREEPSTTDLPD
79_9653  AAGAVGVALLNTTTLGETLTNFIMSWTSLETSLGAIARVCTFEQDTPREREEPSTTDLPD
80_4462  AAGAVGVALLNTTTLGETLTNFIMSWTSLETSLGAIARVCTFEQDTPREREEPSTTDLPD
81_7194  LGAIARVCTFEQDTPREREEPSTTDLPDNRPGAGQISFENVWATYEDEGCGSNWGLSGIT
82_1022  AAGAVGVALLNTTTLGETLTNFIMSWTSLETSLGAIARVCTFEQDTPREREEPSTTDLPD
83_9813  AAGAVGVALLNTTTLGETLTNFIMSWTSLETSLGAIARVCTFEQDTPREREEPSTTDLPD
84_1902  AAGAVGVALLNTTTLGETLTNFIMSWTSLETSLGAIARVCTFEQDTPREREEPSTTDLPD
85_5152  AAGAVGVALLNTTTLGETLTNFIMSWTSLETSLGAIARVCTFEQDTPREREEPSTTDLPD
86_3385  AAGAVGVALLNTTTLGETLTNFIMSWTSLETSLGAIARVCTFEQDTPREREEPSTTDLPD
87_10760 AAGAVGVALLNTTTLGETLTNFIMSWTSLETSLGAIARVCTFEQDTPREREEPSTTDLPD
88_1588  AAGAVGVALLNTTTLGETLTNFIMSWTSLETSLGAIARVCTFEQDTPREREEPSTTDLPD
89_1609  AAGAVGVALLNTTTLGETLTNFIMSWTSLETSLGAIARVCTFEQDTPREREEPSTTDLPD
90_4023  AAGAVGVALLNTTTLGETLTNFIMSWTSLETSLGAIARVCTFEQDTPREREEPSTTDLPD
```

```
                  1210      1220      1230      1240      1250      1260
1_4160      NRPGAGQISFENVWATYEDEGCGSNWGLSGITLAVQPGERVAVCGRTGSGKSTLLLALLG
2_6143      NRPGAGQISFENVWATYEDEGCGSNWGLSGITLAVQPGERVAVCGRTGSGKSTLLLALLG
3_6554      NRPGAGQISFENVWATYEDEGCGSNWGLSGITLAVQPGERVAVCGRTGSGKSTLLLALLG
4_111       NRPGAGQISFENVWATYEDEGCGSNWGLSGITLAVQPGERVAVCGRTGSGKSTLLLALLG
5_5742      NRPGAGQISFENVWATYEDEGCGSNWGLSGITLAVQPGERVAVCGRTGSGKSTLLLALLG
6_9490      NRPGAGQISFENVWATYEDEGCGSNWGLSGITLAVQPGERVAVCGRTGSGKSTLLLALLG
7_6313      ............................................................
8_2552      NRPGAGQISFENVWATYEDEGCGSNWGLSGITLAVQPGERVAVCGRTGSGKSTLLLALLG
9_7745      NRPGAGQISFENVWATYEDEGCGSNWGLSGITLAVQPGERVAVCGRTGSGKSTLLLALLG
10_2315     NRPGAGQISFENVWATYEDEGCGSNWGLSGITLAVQPGERVAVCGRTGSGKSTLLLALLG
11_2035     NRPGAGQISFENVWATYEDEGCGSNWGLSGITLAVQPGERVAVCGRTGSGKSTLLLALLG
12_9640     NRPGAGQISFENVWATYEDEGCGSNWGLSGITLAVQPGERVAVCGRTGSGKSTLLLALLG
13_3674     NRPGAGQISFENVWATYEDEGCGSNWGLSGITLAVQPGERVAVCGRTGSGKSTLLLALLG
14_6548     NRPGAGQISFENVWATYEDEGCGSNWGLSGITLAVQPGERVAVCGRTGSGKSTLLLALLG
15_10828    NRPGAGQISFENVWATYEDEGCGSNWGLSGITLAVQPGERVAVCGRTGSGKSTLLLALLG
16_6578     NRPGAGQISFENVWATYEDEGCGSNWGLSGITLAVQPGERVAVCGRTGSGKSTLLLALLG
17_10233    NRPGAGQISFENVWATYEDEGCGSNWGLSGITLAVQPGERVAVCGRTGSGKSTLLLALLG
18_9261     NRPGAGQISFENVWATYEDEGCGSNWGLSGITLAVQPGERVAVCGRTGSGKSTLLLALLG
19_8450     NRPGAGQISFENVWATYEDEGCGSNWGLSGITLAVQPGERVAVCGRTGSGKSTLLLALLG
20_1727     NRPGAGQISFENVWATYEDEGCGSNWGLSGITLAVQPGERVAVCGRTGSGKSTLLLALLG
21_206      NRPGAGQISFENVWATYEDEGCGSNWGLSGITLAVQPGERVAVCGRTGSGKSTLLLALLG
22_7439     NRPGAGQISFENVWATYEDEGCGSNWGLSGITLAVQPGERVAVCGRTGSGKSTLLLALLG
23_3780     NRPGAGQISFENVWATYEDEGCGSNWGLSGITLAVQPGERVAVCGRTGSGKSTLLLALLG
24_967      NRPGAGQISFENVWATYEDEGCGSNWGLSGITLAVQPGERVAVCGRTGSGKSTLLLALLG
25_6595     NRPGAGQISFENVWATYEDEGCGSNWGLSGITLAVQPGERVAVCGRTGSGKSTLLLALLG
26_8391     NRPGAGQISFENVWATYEDEGCGSNWGLSGITLAVQPGERVAVCGRTGSGKSTLLLALLG
27_8170     NRPGAGQISFENVWATYEDEGCGSNWGLSGITLAVQPGERVAVCGRTGSGKSTLLLALLG
28_5209     NRPGAGQISFENVWATYEDEGCGSNWGLSGITLAVQPGERVAVCGRTGSGKSTLLLALLG
29_2181     NRPGAGQISFENVWATYEDEGCGSNWGLSGITLAVQPGERVAVCGRTGSGKSTLLLALLG
30_8564     NRPGAGQISFENVWATYEDEGCGSNWGLSGITLAVQPGERVAVCGRTGSGKSTLLLALLG
31_1582     NRPGAGQISFENVWATYEDEGCGSNWGLSGITLAVQPGERVAVCGRTGSGKSTLLLALLG
32_4537     NRPGAGQISFENVWATYEDEGCGSNWGLSGITLAVQPGERVAVCGRTGSGKSTLLLALLG
33_9418     NRPGAGQISFENVWATYEDEGCGSNWGLSGITLAVQPGERVAVCGRTGSGKSTLLLALLG
34_1782     NRPGAGQISFENVWATYEDEGCGSNWGLSGITLAVQPGERVAVCGRTGSGKSTLLLALLG
35_10360    NRPGAGQISFENVWATYEDEGCGSNWGLSGITLAVQPGERVAVCGRTGSGKSTLLLALLG
36_468      NRPGAGQISFENVWATYEDEGCGSNWGLSGITLAVQPGERVAVCGRTGSGKSTLLLALLG
37_4956     NRPGAGQISFENVWATYEDEGCGSNWGLSGITLAVQPGERVAVCGRTGSGKSTLLLALLG
38_4305     NRPGAGQISFENVWATYEDEGCGSNWGLSGITLAVQPGERVAVCGRTGSGKSTLLLALLG
39_4737     NRPGAGQISFENVWATYEDEGCGSNWGLSGITLAVQPGERVAVCGRTGSGKSTLLLALLG
40_9543     NRPGAGQISFENVWATYEDEGCGSNWGLSGITLAVQPGERVAVCGRTGSGKSTLLLALLG
41_6669     NRPGAGQISFENVWATYEDEGCGSNWGLSGITLAVQPGERVAVCGRTGSGKSTLLLALLG
42_10558    NRPGAGQISFENVWATYEDEGCGSNWGLSGITLAVQPGERVAVCGRTGSGKSTLLLALLG
43_8051     NRPGAGQISFENVWATYEDEGCGSNWGLSGITLAVQPGERVAVCGRTGSGKSTLLLALLG
44_10454    NRPGAGQISFENVWATYEDEGCGSNWGLSGITLAVQPGERVAVCGRTGSGKSTLLLALLG
45_10126    NRPGAGQISFENVWATYEDEGCGSNWGLSGITLAVQPGERVAVCGRTGSGKSTLLLALLG
46_1547     NRPGAGQISFENVWATYEDEGCGSNWGLSGITLAVQPGERVAVCGRTGSGKSTLLLALLG
47_6749     NRPGAGQISFENVWATYEDEGCGSNWGLSGITLAVQPGERVAVCGRTGSGKSTLLLALLG
48_9982     NRPGAGQISFENVWATYEDEGCGSNWGLSGITLAVQPGERVAVCGRTGSGKSTLLLALLG
49_7293     NRPGAGQISFENVWATYEDEGCGSNWGLSGITLAVQPGERVAVCGRTGSGKSTLLLALLG
50_5993     NRPGAGQISFENVWATYEDEGCGSNWGLSGITLAVQPGERVAVCGRTGSGKSTLLLALLG
51_4791     NRPGAGQISFENVWATYEDEGCGSNWGLSGITLAVQPGERVAVCGRTGSGKSTLLLALLG
52_121      NRPGAGQISFENVWATYEDEGCGSNWGLSGITLAVQPGERVAVCGRTGSGKSTLLLALLG
53_1667     NRPGAGQISFENVWATYEDEGCGSNWGLSGITLAVQPGERVAVCGRTGSGKSTLLLALLG
54_10488    NRPGAGQISFENVWATYEDEGCGSNWGLSGITLAVQPGERVAVCGRTGSGKSTLLLALLG
55_7733     NRPGAGQISFENVWATYEDEGCGSNWGLSGITLAVQPGERVAVCGRTGSGKSTLLLALLG
56_3982     NRPGAGQISFENVWATYEDEGCGSNWGLSGITLAVQPGERVAVCGRTGSGKSTLLLALLG
57_392      NRPGAGQISFENVWATYEDEGCGSNWGLSGITLAVQPGERVAVCGRTGSGKSTLLLALLG
58_10291    NRPGAGQISFENVWATYEDEGCGSNWGLSGITLAVQPGERVAVCGRTGSGKSTLLLALLG
59_5792     NRPGAGQISFENVWATYEDEGCGSNWGLSGITLAVQPGERVAVCGRTGSGKSTLLLALLG
60_8712     NRPGAGQISFENVWATYEDEGCGSNWGLSGITLAVQPGERVAVCGRTGSGKSTLLLALLG
61_6716     NRPGAGQISFENVWATYEDEGCGSNWGLSGITLAVQPGERVAVCGRTGSGKSTLLLALLG
62_5599     NRPGAGQISFENVWATYEDEGCGSNWGLSGITLAVQPGERVAVCGRTGSGKSTLLLALLG
63_304      NRPGAGQISFENVWATYEDEGCGSNWGLSGITLAVQPGERVAVCGRTGSGKSTLLLALLG
64_10473    ............................................................
65_6310     NRPGAGQISFENVWATYEDEGCGSNWGLSGITLAVQPGERVAVCGRTGSGKSTLLLALLG
66_8714     NRPGAGQISFENVWATYEDEGCGSNWGLSGITLAVQPGERVAVCGRTGSGKSTLLLALLG
67_2350     NRPGAGQISFENVWATYEDEGCGSNWGLSGITLAVQPGERVAVCGRTGSGKSTLLLALLG
68_2447     NRPGAGQISFENVWATYEDEGCGSNWGLSGITLAVQPGERVAVCGRTGSGKSTLLLALLG
69_5658     NRPGAGQISFENVWATYEDEGCGSNWGLSGITLAVQPGERVAVCGRTGSGKSTLLLALLG
70_1277     NRPGAGQISFENVWATYEDEGCGSNWGLSGITLAVQPGERVAVCGRTGSGKSTLLLALLG
71_3717     NRPGAGQISFENVWATYEDEGCGSNWGLSGITLAVQPGERVAVCGRTGSGKSTLLLALLG
72_7846     NRPGAGQISFENVWATYEDEGCGSNWGLSGITLAVQPGERVAVCGRTGSGKSTLLLALLG
73_10528    NRPGAGQISFENVWATYEDEGCGSNWGLSGITLAVQPGERVAVCGRTGSGKSTLLLALLG
74_4899     NRPGAGQISFENVWATYEDEGCGSNWGLSGITLAVQPGERVAVCGRTGSGKSTLLLALLG
75_5507     NRPGAGQISFENVWATYEDEGCGSNWGLSGITLAVQPGERVAVCGRTGSGKSTLLLALLG
76_5432     NRPGAGQISFENVWATYEDEGCGSNWGLSGITLAVQPGERVAVCGRTGSGKSTLLLALLG
77_10770    NRPGAGQISFENVWATYEDEGCGSNWGLSGITLAVQPGERVAVCGRTGSGKSTLLLALLG
78_5904     NRPGAGQISFENVWATYEDEGCGSNWGLSGITLAVQPGERVAVCGRTGSGKSTLLLALLG
79_9653     NRPGAGQISFENVWATYEDEGCGSNWGLSGITLAVQPGERVAVCGRTGSGKSTLLLALLG
80_4462     NRPGAGQISFENVWATYEDEGCGSNWGLSGITLAVQPGERVAVCGRTGSGKSTLLLALLG
81_7194     LAVQPGERVAVCGRTGSGKSTLLLALLGMLHTPAGSIRIDGVDTSTLPIDVLRRRFTVVS
82_1022     NRPGAGQISFENVWATYEDEGCGSNWGLSGITLAVQPGERVAVCGRTGSGKSTLLLALLG
83_9813     NRPGAGQISFENVWATYEDEGCGSNWGLSGITLAVQPGERVAVCGRTGSGKSTLLLALLG
84_1902     NRPGAGQISFENVWATYEDEGCGSNWGLSGITLAVQPGERVAVCGRTGSGKSTLLLALLG
85_5152     NRPGAGQISFENVWATYEDEGCGSNWGLSGITLAVQPGERVAVCGRTGSGKSTLLLALLG
86_3385     NRPGAGQISFENVWATYEDEGCGSNWGLSGITLAVQPGERVAVCGRTGSGKSTLLLALLG
87_10760    NRPGAGQISFENVWATYEDEGCGSNWGLSGITLAVQPGERVAVCGRTGSGKSTLLLALLG
88_1588     NRPGAGQISFENVWATYEDEGCGSNWGLSGITLAVQPGERVAVCGRTGSGKSTLLLALLG
89_1609     NRPGAGQISFENVWATYEDEGCGSNWGLSGITLAVQPGERVAVCGRTGSGKSTLLLALLG
90_4023     NRPGAGQISFENVWATYEDEGCGSNWGLSGITLAVQPGERVAVCGRTGSGKSTLLLALLG
```

```
              1270      1280      1290      1300      1310      1320
1_4160    MLHTPAGSIRIDGVDTSTLPIDVLRRRFTVVSQDSFFEPTSTFRQELDPSGDMSDQIIEE
2_6143    MLHTPAGSIRIDGVDTSTLPIDVLRRRFTVVSQDSFFEPTSTFRQELDPSGDMSDQIIEE
3_6554    MLHTPAGSIRIDGVDTSTLPIDVLRRRFTVVSQDSFFEPTSTFRQELDPSGDMSDQIIEE
4_111     MLHTPAGSIRIDGVDTSTLPIDVLRRRFTVVSQDSFFEPTSTFRQELDPSGDMSDQIIEE
5_5742    MLHTPAGSIRIDGVDTSTLPIDVLRRRFTVVSQDSFFEPTSTFRQELDPSGDMSDQIIEE
6_9490    MLHTPAGSIRIDGVDTSTLPIDVLRRRFTVVSQDSFFEPTSTFRQELDPSGDMSDQIIEE
7_6313    ............................................................
8_2552    MLHTPAGSIRIDGVDTSTLPIDVLRRRFTVVSQDSFFEPTSTFRQELDPSGDMSDQIIEE
9_7745    MLHTPAGSIRIDGVDTSTLPIDVLRRRFTVVSQDSFFEPTSTFRQELDPSGDMSDQIIEE
10_2315   MLHTPAGSIRIDGVDTSTLPIDVLRRRFTVVSQDSFFEPTSTFRQELDPSGDMSDQIIEE
11_2035   MLHTPAGSIRIDGVDTSTLPIDVLRRRFTVVSQDSFFEPTSTFRQELDPSGDMSDQIIEE
12_9640   MLHTPAGSIRIDGVDTSTLPIDVLRRRFTVVSQDSFFEPTSTFRQELDPSGDMSDQIIEE
13_3674   MLHTPAGSIRIDGVDTSTLPIDVLRRRFTVVSQDSFFEPTSTFRQELDPSGDMSDQIIEE
14_6548   MLHTPAGSIRIDGVDTSTLPIDVLRRRFTVVSQDSFFEPTSTFRQELDPSGDMSDQIIEE
15_10828  MLHTPAGSIRIDGVDTSTLPIDVLRRRFTVVSQDSFFEPTSTFRQELDPSGDMSDQIIEE
16_6578   MLHTPAGSIRIDGVDTSTLPIDVLRRRFTVVSQDSFFEPTSTFRQELDPSGDMSDQIIEE
17_10233  MLHTPAGSIRIDGVDTSTLPIDVLRRRFTVVSQDSFFEPTSTFRQELDPSGDMSDQIIEE
18_9261   MLHTPAGSIRIDGVDTSTLPIDVLRRRFTVVSQDSFFEPTSTFRQELDPSGDMSDQIIEE
19_8450   MLHTPAGSIRIDGVDTSTLPIDVLRRRFTVVSQDSFFEPTSTFRQELDPSGDMSDQIIEE
20_1727   MLHTPAGSIRIDGVDTSTLPIDVLRRRFTVVSQDSFFEPTSTFRQELDPSGDMSDQIIEE
21_206    MLHTPAGSIRIDGVDTSTLPIDVLRRRFTVVSQDSFFEPTSTFRQELDPSGDMSDQIIEE
22_7439   MLHTPAGSIRIDGVDTSTLPIDVLRRRFTVVSQDSFFEPTSTFRQELDPSGDMSDQIIEE
23_3780   MLHTPAGSIRIDGVDTSTLPIDVLRRRFTVVSQDSFFEPTSTFRQELDPSGDMSDQIIEE
24_967    MLHTPAGSIRIDGVDTSTLPIDVLRRRFTVVSQDSFFEPTSTFRQELDPSGDMSDQIIEE
25_6595   MLHTPAGSIRIDGVDTSTLPIDVLRRRFTVVSQDSFFEPTSTFRQELDPSGDMSDQIIEE
26_8391   MLHTPAGSIRIDGVDTSTLPIDVLRRRFTVVSQDSFFEPTSTFRQELDPSGDMSDQIIEE
27_8170   MLHTPAGSIRIDGVDTSTLPIDVLRRRFTVVSQDSFFEPTSTFRQELDPSGDMSDQIIEE
28_5209   MLHTPAGSIRIDGVDTSTLPIDVLRRRFTVVSQDSFFEPTSTFRQELDPSGDMSDQIIEE
29_2181   MLHTPAGSIRIDGVDTSTLPIDVLRRRFTVVSQDSFFEPTSTFRQELDPSGDMSDQIIEE
30_8564   MLHTPAGSIRIDGVDTSTLPIDVLRRRFTVVSQDSFFEPTSTFRQELDPSGDMSDQIIEE
31_1582   MLHTPAGSIRIDGVDTSTLPIDVLRRRFTVVSQDSFFEPTSTFRQELDPSGDMSDQIIEE
32_4537   MLHTPAGSIRIDGVDTSTLPIDVLRRRFTVVSQDSFFEPTSTFRQELDPSGDMSDQIIEE
33_9418   MLHTPAGSIRIDGVDTSTLPIDVLRRRFTVVSQDSFFEPTSTFRQELDPSGDMSDQIIEE
34_1782   MLHTPAGSIRIDGVDTSTLPIDVLRRRFTVVSQDSFFEPTSTFRQELDPSGDMSDQIIEE
35_10360  MLHTPAGSIRIDGVDTSTLPIDVLRRRFTVVSQDSFFEPTSTFRQELDPSGDMSDQIIEE
36_468    MLHTPAGSIRIDGVDTSTLPIDVLRRRFTVVSQDSFFEPTSTFRQELDPSGDMSDQIIEE
37_4956   MLHTPAGSIRIDGVDTSTLPIDVLRRRFTVVSQDSFFEPTSTFRQELDPSGDMSDQIIEE
38_4305   MLHTPAGSIRIDGVDTSTLPIDVLRRRFTVVSQDSFFEPTSTFRQELDPSGDMSDQIIEE
39_4737   MLHTPAGSIRIDGVDTSTLPIDVLRRRFTVVSQDSFFEPTSTFRQELDPSGDMSDQIIEE
40_9543   MLHTPAGSIRIDGVDTSTLPIDVLRRRFTVVSQDSFFEPTSTFRQELDPSGDMSDQIIEE
41_6669   MLHTPAGSIRIDGVDTSTLPIDVLRRRFTVVSQDSFFEPTSTFRQELDPSGDMSDQIIEE
42_10558  MLHTPAGSIRIDGVDTSTLPIDVLRRRFTVVSQDSFFEPTSTFRQELDPSGDMSDQIIEE
43_8051   MLHTPAGSIRIDGVDTSTLPIDVLRRRFTVVSQDSFFEPTSTFRQELDPSGDMSDQIIEE
44_10454  MLHTPAGSIRIDGVDTSTLPIDVLRRRFTVVSQDSFFEPTSTFRQELDPSGDMSDQIIEE
45_10126  MLHTPAGSIRIDGVDTSTLPIDVLRRRFTVVSQDSFFEPTSTFRQELDPSGDMSDQIIEE
46_1547   MLHTPAGSIRIDGVDTSTLPIDVLRRRFTVVSQDSFFEPTSTFRQELDPSGDMSDQIIEE
47_6749   MLHTPAGSIRIDGVDTSTLPIDVLRRRFTVVSQDSFFEPTSTFRQELDPSGDMSDQIIEE
48_9982   MLHTPAGSIRIDGVDTSTLPIDVLRRRFTVVSQDSFFEPTSTFRQELDPSGDMSDQIIEE
49_7293   MLHTPAGSIRIDGVDTSTLPIDVLRRRFTVVSQDSFFEPTSTFRQELDPSGDMSDQIIEE
50_5993   MLHTPAGSIRIDGVDTSTLPIDVLRRRFTVVSQDSFFEPTSTFRQELDPSGDMSDQIIEE
51_4791   MLHTPAGSIRIDGVDTSTLPIDVLRRRFTVVSQDSFFEPTSTFRQELDPSGDMSDQIIEE
52_121    MLHTPAGSIRIDGVDTSTLPIDVLRRRFTVVSQDSFFEPTSTFRQELDPSGDMSDQIIEE
53_1667   MLHTPAGSIRIDGVDTSTLPIDVLRRRFTVVSQDSFFEPTSTFRQELDPSGDMSDQIIEE
54_10488  MLHTPAGSIRIDGVDTSTLPIDVLRRRFTVVSQDSFFEPTSTFRQELDPSGDMSDQIIEE
55_7733   MLHTPAGSIRIDGVDTSTLPIDVLRRRFTVVSQDSFFEPTSTFRQELDPSGDMSDQIIEE
56_3982   MLHTPAGSIRIDGVDTSTLPIDVLRRRFTVVSQDSFFEPTSTFRQELDPSGDMSDQIIEE
57_392    MLHTPAGSIRIDGVDTSTLPIDVLRRRFTVVSQDSFFEPTSTFRQELDPSGDMSDQIIEE
58_10291  MLHTPAGSIRIDGVDTSTLPIDVLRRRFTVVSQDSFFEPTSTFRQELDPSGDMSDQIIEE
59_5792   MLHTPAGSIRIDGVDTSTLPIDVLRRRFTVVSQDSFFEPTSTFRQELDPSGDMSDQIIEE
60_8712   MLHTPAGSIRIDGVDTSTLPIDVLRRRFTVVSQDSFFEPTSTFRQELDPSGDMSDQIIEE
61_6716   MLHTPAGSIRIDGVDTSTLPIDVLRRRFTVVSQDSFFEPTSTFRQELDPSGDMSDQIIEE
62_5599   MLHTPAGSIRIDGVDTSTLPIDVLRRRFTVVSQDSFFEPTSTFRQELDPSGDMSDQIIEE
63_304    MLHTPAGSIRIDGVDTSTLPIDVLRRRFTVVSQDSFFEPTSTFRQELDPSGDMSDQIIEE
64_10473  ............................................................
65_6310   MLHTPAGSIRIDGVDTSTLPIDVLRRRFTVVSQDSFFEPTSTFRQELDPSGDMSDQIIEE
66_8714   MLHTPAGSIRIDGVDTSTLPIDVLRRRFTVVSQDSFFEPTSTFRQELDPSGDMSDQIIEE
67_2350   MLHTPAGSIRIDGVDTSTLPIDVLRRRFTVVSQDSFFEPTSTFRQELDPSGDMSDQIIEE
68_2447   MLHTPAGSIRIDGVDTSTLPIDVLRRRFTVVSQDSFFEPTSTFRQELDPSGDMSDQIIEE
69_5658   MLHTPAGSIRIDGVDTSTLPIDVLRRRFTVVSQDSFFEPTSTFRQELDPSGDMSDQIIEE
70_1277   MLHTPAGSIRIDGVDTSTLPIDVLRRRFTVVSQDSFFEPTSTFRQELDPSGDMSDQIIEE
71_3717   MLHTPAGSIRIDGVDTSTLPIDVLRRRFTVVSQDSFFEPTSTFRQELDPSGDMSDQIIEE
72_7846   MLHTPAGSIRIDGVDTSTLPIDVLRRRFTVVSQDSFFEPTSTFRQELDPSGDMSDQIIEE
73_10528  MLHTPAGSIRIDGVDTSTLPIDVLRRRFTVVSQDSFFEPTSTFRQELDPSGDMSDQIIEE
74_4899   MLHTPAGSIRIDGVDTSTLPIDVLRRRFTVVSQDSFFEPTSTFRQELDPSGDMSDQIIEE
75_5507   MLHTPAGSIRIDGVDTSTLPIDVLRRRFTVVSQDSFFEPTSTFRQELDPSGDMSDQIIEE
76_5432   MLHTPAGSIRIDGVDTSTLPIDVLRRRFTVVSQDSFFEPTSTFRQELDPSGDMSDQIIEE
77_10770  MLHTPAGSIRIDGVDTSTLPIDVLRRRFTVVSQDSFFEPTSTFRQELDPSGDMSDQIIEE
78_5904   MLHTPAGSIRIDGVDTSTLPIDVLRRRFTVVSQDSFFEPTSTFRQELDPSGDMSDQIIEE
79_9653   MLHTPAGSIRIDGVDTSTLPIDVLRRRFTVVSQDSFFEPTSTFRQELDPSGDMSDQIIEE
80_4462   MLHTPAGSIRIDGVDTSTLPIDVLRRRFTVVSQDSFFEPTSTFRQELDPSGDMSDQIIEE
81_7194   QDSFFEPTSTFRQELDPSGDMSDQIIEEVLRECRAWEIVDGSGGLGGKRADANLSAGEVQ
82_1022   MLHTPAGSIRIDGVDTSTLPIDVLRRRFTVVSQDSFFEPTSTFRQELDPSGDMSDQIIEE
83_9813   MLHTPAGSIRIDGVDTSTLPIDVLRRRFTVVSQDSFFEPTSTFRQELDPSGDMSDQIIEE
84_1902   MLHTPAGSIRIDGVDTSTLPIDVLRRRFTVVSQDSFFEPTSTFRQELDPSGDMSDQIIEE
85_5152   MLHTPAGSIRIDGVDTSTLPIDVLRRRFTVVSQDSFFEPTSTFRQELDPSGDMSDQIIEE
86_3385   MLHTPAGSIRIDGVDTSTLPIDVLRRRFTVVSQDSFFEPTSTFRQELDPSGDMSDQIIEE
87_10760  MLHTPAGSIRIDGVDTSTLPIDVLRRRFTVVSQDSFFEPTSTFRQELDPSGDMSDQIIEE
88_1588   MLHTPAGSIRIDGVDTSTLPIDVLRRRFTVVSQDSFFEPTSTFRQELDPSGDMSDQIIEE
89_1609   MLHTPAGSIRIDGVDTSTLPIDVLRRRFTVVSQDSFFEPTSTFRQELDPSGDMSDQIIEE
90_4023   MLHTPAGSIRIDGVDTSTLPIDVLRRRFTVVSQDSFFEPTSTFRQELDPSGDMSDQIIEE
```

```
             1330      1340      1350      1360      1370      1380
1_4160    VLRECRAWEIVDGSGGLGGKRADANLSAGEVQLLAIARLVLQWQSQPAGSGGIILLDEAT
2_6143    VLRECRAWEIVDGSGGLGGKRADANLSAGEVQLLAIARLVLQWQSQPAGSGGIILLDEAT
3_6554    VLRECRAWEIVDGSGGLGGKRADANLSAGEVQLLAIARLVLQWQSQPAGSGGIILLDEAT
4_111     VLRECRAWEIVDGSGGLGGKRADANLSAGEVQLLAIARLVLQWQSQPAGSGGIILLDEAT
5_5742    VLRECRAWEIVDGSGGLGGKRADANLSAGEVQLLAIARLVLQWQSQPAGSGGIILLDEAT
6_9490    VLRECRAWEIVDGSGGLGGKRADANLSAGEVQLLAIARLVLQWQSQPAGSGGIILLDEAT
7_6313    ............................................................
8_2552    VLRECRAWEIVDGSGGLGGKRADANLSAGEVQLLAIARLVLQWQSQPAGSGGIILLDEAT
9_7745    VLRECRAWEIVDGSGGLGGKRADANLSAGEVQLLAIARLVLQWQSQPAGSGGIILLDEAT
10_2315   VLRECRAWEIVDGSGGLGGKRADANLSAGEVQLLAIARLVLQWQSQPAGSGGIILLDEAT
11_2035   VLRECRAWEIVDGSGGLGGKRADANLSAGEVQLLAIARLVLQWQSQPAGSGGIILLDEAT
12_9640   VLRECRAWEIVDGSGGLGGKRADANLSAGEVQLLAIARLVLQWQSQPAGSGGIILLDEAT
13_3674   VLRECRAWEIVDGSGGLGGKRADANLSAGEVQLLAIARLVLQWQSQPAGSGGIILLDEAT
14_6548   VLRECRAWEIVDGSGGLGGKRADANLSAGEVQLLAIARLVLQWQSQPAGSGGIILLDEAT
15_10828  VLRECRAWEIVDGSGGLGGKRADANLSAGEVQLLAIARLVLQWQSQPAGSGGIILLDEAT
16_6578   VLRECRAWEIVDGSGGLGGKRADANLSAGEVQLLAIARLVLQWQSQPAGSGGIILLDEAT
17_10233  VLRECRAWEIVDGSGGLGGKRADANLSAGEVQLLAIARLVLQWQSQPAGSGGIILLDEAT
18_9261   VLRECRAWEIVDGSGGLGGKRADANLSAGEVQLLAIARLVLQWQSQPAGSGGIILLDEAT
19_8450   VLRECRAWEIVDGSGGLGGKRADANLSAGEVQLLAIARLVLQWQSQPAGSGGIILLDEAT
20_1727   VLRECRAWEIVDGSGGLGGKRADANLSAGEVQLLAIARLVLQWQSQPAGSGGIILLDEAT
21_206    VLRECRAWEIVDGSGGLGGKRADANLSAGEVQLLAIARLVLQWQSQPAGSGGIILLDEAT
22_7439   VLRECRAWEIVDGSGGLGGKRADANLSAGEVQLLAIARLVLQWQSQPAGSGGIILLDEAT
23_3780   VLRECRAWEIVDGSGGLGGKRADANLSAGEVQLLAIARLVLQWQSQPAGSGGIILLDEAT
24_967    VLRECRAWEIVDGSGGLGGKRADANLSAGEVQLLAIARLVLQWQSQPAGSGGIILLDEAT
25_6595   VLRECRAWEIVDGSGGLGGKRADANLSAGEVQLLAIARLVLQWQSQPAGSGGIILLDEAT
26_8391   VLRECRAWEIVDGSGGLGGKRADANLSAGEVQLLAIARLVLQWQSQPAGSGGIILLDEAT
27_8170   VLRECRAWEIVDGSGGLGGKRADANLSAGEVQLLAIARLVLQWQSQPAGSGGIILLDEAT
28_5209   VLRECRAWEIVDGSGGLGGKRADANLSAGEVQLLAIARLVLQWQSQPAGSGGIILLDEAT
29_2181   VLRECRAWEIVDGSGGLGGKRADANLSAGEVQLLAIARLVLQWQSQPAGSGGIILLDEAT
30_8564   VLRECRAWEIVDGSGGLGGKRADANLSAGEVQLLAIARLVLQWQSQPAGSGGIILLDEAT
31_1582   VLRECRAWEIVDGSGGLGGKRADANLSAGEVQLLAIARLVLQWQSQPAGSGGIILLDEAT
32_4537   VLRECRAWEIVDGSGGLGGKRADANLSAGEVQLLAIARLVLQWQSQPAGSGGIILLDEAT
33_9418   VLRECRAWEIVDGSGGLGGKRADANLSAGEVQLLAIARLVLQWQSQPAGSGGIILLDEAT
34_1782   VLRECRAWEIVDGSGGLGGKRADANLSAGEVQLLAIARLVLQWQSQPAGSGGIILLDEAT
35_10360  VLRECRAWEIVDGSGGLGGKRADANLSAGEVQLLAIARLVLQWQSQPAGSGGIILLDEAT
36_468    VLRECRAWEIVDGSGGLGGKRADANLSAGEVQLLAIARLVLQWQSQPAGSGGIILLDEAT
37_4956   VLRECRAWEIVDGSGGLGGKRADANLSAGEVQLLAIARLVLQWQSQPAGSGGIILLDEAT
38_4305   VLRECRAWEIVDGSGGLGGKRADANLSAGEVQLLAIARLVLQWQSQPAGSGGIILLDEAT
39_4737   VLRECRAWEIVDGSGGLGGKRADANLSAGEVQLLAIARLVLQWQSQPAGSGGIILLDEAT
40_9543   VLRECRAWEIVDGSGGLGGKRADANLSAGEVQLLAIARLVLQWQSQPAGSGGIILLDEAT
41_6669   VLRECRAWEIVDGSGGLGGKRADANLSAGEVQLLAIARLVLQWQSQPAGSGGIILLDEAT
42_10558  VLRECRAWEIVDGSGGLGGKRADANLSAGEVQLLAIARLVLQWQSQPAGSGGIILLDEAT
43_8051   VLRECRAWEIVDGSGGLGGKRADANLSAGEVQLLAIARLVLQWQSQPAGSGGIILLDEAT
44_10454  VLRECRAWEIVDGSGGLGGKRADANLSAGEVQLLAIARLVLQWQSQPAGSGGIILLDEAT
45_10126  VLRECRAWEIVDGSGGLGGKRADANLSAGEVQLLAIARLVLQWQSQPAGSGGIILLDEAT
46_1547   VLRECRAWEIVDGSGGLGGKRADANLSAGEVQLLAIARLVLQWQSQPAGSGGIILLDEAT
47_6749   VLRECRAWEIVDGSGGLGGKRADANLSAGEVQLLAIARLVLQWQSQPAGSGGIILLDEAT
48_9982   VLRECRAWEIVDGSGGLGGKRADANLSAGEVQLLAIARLVLQWQSQPAGSGGIILLDEAT
49_7293   VLRECRAWEIVDGSGGLGGKRADANLSAGEVQLLAIARLVLQWQSQPAGSGGIILLDEAT
50_5993   VLRECRAWEIVDGSGGLGGKRADANLSAGEVQLLAIARLVLQWQSQPAGSGGIILLDEAT
51_4791   VLRECRAWEIVDGSGXRKPLGGRGSGGIILLDEATSNLDRQTEVLVESIMAARLQHATVV
52_121    VLRECRAWEIVDGSGGLGGKRADANLSAGEVQLLAIARLVLQWQSQPAGSGGIILLDEAT
53_1667   VLRECRAWEIVDGSGGLGGKRADANLSAGEVQLLAIARLVLQWQSQPAGSGGIILLDEAT
54_10488  VLRECRAWEIVDGSGGLGGKRADANLSAGEVQLLAIARLVLQWQSQPAGSGGIILLDEAT
55_7733   VLRECRAWEIVDGSGGLGGKRADANLSAGEVQLLAIARLVLQWQSQPAGSGGIILLDEAT
56_3982   VLRECRAWEIVDGSGGLGGKRADANLSAGEVQLLAIARLVLQWQSQPAGSGGIILLDEAT
57_392    VLRECRAWEIVDGSGGLGGKRADANLSAGEVQLLAIARLVLQWQSQPAGSGGIILLDEAT
58_10291  VLRECRAWEIVDGSGGLGGKRADANLSAGEVQLLAIARLVLQWQSQPAGSGGIILLDEAT
59_5792   VLRECRAWEIVDGSGGLGGKRADANLSAGEVQLLAIARLVLQWQSQPAGSGGIILLDEAT
60_8712   VLRECRAWEIVDGSGGLGGKRADANLSAGEVQLLAIARLVLQWQSQPAGSGGIILLDEAT
61_6716   VLRECRAWEIVDGSGGLGGKRADANLSAGEVQLLAIARLVLQWQSQPAGSGGIILLDEAT
62_5599   VLRECRAWEIVDGSGGLGGKRADANLSAGEVQLLAIARLVLQWQSQPAGSGGIILLDEAT
63_304    VLRECRAWEIVDGSGGLGGKRADANLSAGEVQLLAIARLVLQWQSQPAGSGGIILLDEAT
64_10473  ............................................................
65_6310   VLRECRAWEIVDGSGGLGGKRADANLSAGEVQLLAIARLVLQWQSQPAGSGGIILLDEAT
66_8714   VLRECRAWEIVDGSGGLGGKRADANLSAGEVQLLAIARLVLQWQSQPAGSGGIILLDEAT
67_2350   VLRECRAWEIVDGSGGLGGKRADANLSAGEVQLLAIARLVLQWQSQPAGSGGIILLDEAT
68_2447   VLRECRAWEIVDGSGGLGGKRADANLSAGEVQLLAIARLVLQWQSQPAGSGGIILLDEAT
69_5658   VLRECRAWEIVDGSGGLGGKRADANLSAGEVQLLAIARLVLQWQSQPAGSGGIILLDEAT
70_1277   VLRECRAWEIVDGSGGLGGKRADANLSAGEVQLLAIARLVLQWQSQPAGSGGIILLDEAT
71_3717   VLRECRAWEIVDGSGGLGGKRADANLSAGEVQLLAIARLVLQWQSQPAGSGGIILLDEAT
72_7846   VLRECRAWEIVDGSGGLGGKRADANLSAGEVQLLAIARLVLQWQSQPAGSGGIILLDEAT
73_10528  VLRECRAWEIVDGSGGLGGKRADANLSAGEVQLLAIARLVLQWQSQPAGSGGIILLDEAT
74_4899   VLRECRAWEIVDGSGGLGGKRADANLSAGEVQLLAIARLVLQWQSQPAGSGGIILLDEAT
75_5507   VLRECRAWEIVDGSGGLGGKRADANLSAGEVQLLAIARLVLQWQSQPAGSGGIILLDEAT
76_5432   VLRECRAWEIVDGSGGLGGKRADANLSAGEVQLLAIARLVLQWQSQPAGSGGIILLDEAT
77_10770  VLRECRAWEIVDGSGGLGGKRADANLSAGEVQLLAIARLVLQWQSQPAGSGGIILLDEAT
78_5904   VLRECRAWEIVDGSGGLGGKRADANLSAGEVQLLAIARLVLQWQSQPAGSGGIILLDEAT
79_9653   VLRECRAWEIVDGSGGLGGKRADANLSAGEVQLLAIARLVLQWQSQPAGSGGIILLDEAT
80_4462   VLRECRAWEIVDGSGGLGGKRADANLSAGEVQLLAIARLVLQWQSQPAGSGGIILLDEAT
81_7194   LLAIARLVLQWQSQPAGSGGIILLDEATSNLDRQTEVLVESIMAARLQHATVVSVMHRLE
82_1022   VLRECRAWEIVDGSGGLGGKRADANLSAGEVQLLAIARLVLQWQSQPAGSGGIILLDEAT
83_9813   VLRECRAWEIVDGSGGLGGKRADANLSAGEVQLLAIARLVLQWQSQPAGSGGIILLDEAT
84_1902   VLRECRAWEIVDGSGGLGGKRADANLSAGEVQLLAIARLVLQWQSQPAGSGGIILLDEAT
85_5152   VLRECRAWEIVDGSGGLGGKRADANLSAGEVQLLAIARLVLQWQSQPAGSGGIILLDEAT
86_3385   VLRECRAWEIVDGSGGLGGKRADANLSAGEVQLLAIARLVLQWQSQPAGSGGIILLDEAT
87_10760  VLRECRAWEIVDGSGGLGGKRADANLSAGEVQLLAIARLVLQWQSQPAGSGGIILLDEAT
88_1588   VLRECRAWEIVDGSGGLGGKRADANLSAGEVQLLAIARLVLQWQSQPAGSGGIILLDEAT
89_1609   VLRECRAWEIVDGSGGLGGKRADANLSAGEVQLLAIARLVLQWQSQPAGSGGIILLDEAT
90_4023   VLRECRAWEIVDGSGGLGGKRADANLSAGEVQLLAIARLVLQWQSQPAGSGGIILLDEAT
```

```
                 1390      1400      1410      1420      1430      1440
                    .         .         .         .         .         .
1_4160    SNLDRQTEVLVESIMAARLQHATVVSVMHRLEAVAAYDKVAVLDKGVLVDFGPVTDVMAR
2_6143    SNLDRQTEVLVESIMAARLQHATVVSVMHRLEAVAAYDKVAVLDKGVLVDFGPVTDVMAR
3_6554    SNLDRQTEVLVESIMAARLQHATVVSVMHRLEAVAAYDKVAVLDKGVLVDFGPVTDVMAR
4_111     SNLDRQTEVLVESIMAARLQHATVVSVMHRLEAVAAYDKVAVLDKGVLVDFGPVTDVMAR
5_5742    SNLDRQTEVLVESIMAARLQHATVVSVMHRLEAVAAYDKVAVLDKGVLVDFGPVTDVMAR
6_9490    SNLDRQTEVLVESIMAARLQHATVVSVMHRLEAVAAYDKVAVLDKGVLVDFGPVTDVMAR
7_6313    ............................................................
8_2552    SNLDRQTEVLVESIMAARLQHATVVSVMHRLEAVAAYDKVAVLDKGVLVDFGPVTDVMAR
9_7745    SNLDRQTEVLVESIMAARLQHATVVSVMHRLEAVAAYDKVAVLDKGVLVDFGPVTDVMAR
10_2315   SNLDRQTEVLVESIMAARLQHATVVSVMHRLEAVAAYDKVAVLDKGVLVDFGPVTDVMAR
11_2035   SNLDRQTEVLVESIMAARLQHATVVSVMHRLEAVAAYDKVAVLDKGVLVDFGPVTDVMAR
12_9640   SNLDRQTEVLVESIMAARLQHATVVSVMHRLEAVAAYDKVAVLDKGVLVDFGPVTDVMAR
13_3674   SNLDRQTEVLVESIMAARLQHATVVSVMHRLEAVAAYDKVAVLDKGVLVDFGPVTDVMAR
14_6548   SNLDRQTEVLVESIMAARLQHATVVSVMHRLEAVAAYDKVAVLDKGVLVDFGPVTDVMAR
15_10828  SNLDRQTEVLVESIMAARLQHATVVSVMHRLEAVAAYDKVAVLDKGVLVDFGPVTDVMAR
16_6578   SNLDRQTEVLVESIMAARLQHATVVSVMHRLEAVAAYDKVAVLDKGVLVDFGPVTDVMAR
17_10233  SNLDRQTEVLVESIMAARLQHATVVSVMHRLEAVAAYDKVAVLDKGVLVDFGPVTDVMAR
18_9261   SNLDRQTEVLVESIMAARLQHATVVSVMHRLEAVAAYDKVAVLDKGVLVDFGPVTDVMAR
19_8450   SNLDRQTEVLVESIMAARLQHATVVSVMHRLEAVAAYDKVAVLDKGVLVDFGPVTDVMAR
20_1727   SNLDRQTEVLVESIMAARLQHATVVSVMHRLEAVAAYDKVAVLDKGVLVDFGPVTDVMAR
21_206    SNLDRQTEVLVESIMAARLQHATVVSVMHRLEAVAAYDKVAVLDKGVLVDFGPVTDVMAR
22_7439   SNLDRQTEVLVESIMAARLQHATVVSVMHRLEAVAAYDKVAVLDKGVLVDFGPVTDVMAR
23_3780   SNLDRQTEVLVESIMAARLQHATVVSVMHRLEAVAAYDKVAVLDKGVLVDFGPVTDVMAR
24_967    SNLDRQTEVLVESIMAARLQHATVVSVMHRLEAVAAYDKVAVLDKGVLVDFGPVTDVMAR
25_6595   SNLDRQTEVLVESIMAARLQHATVVSVMHRLEAVAAYDKVAVLDKGVLVDFGPVTDVMAR
26_8391   SNLDRQTEVLVESIMAARLQHATVVSVMHRLEAVAAYDKVAVLDKGVLVDFGPVTDVMAR
27_8170   SNLDRQTEVLVESIMAARLQHATVVSVMHRLEAVAAYDKVAVLDKGVLVDFGPVTDVMAR
28_5209   SNLDRQTEVLVESIMAARLQHATVVSVMHRLEAVAAYDKVAVLDKGVLVDFGPVTDVMAR
29_2181   SNLDRQTEVLVESIMAARLQHATVVSVMHRLEAVAAYDKVAVLDKGVLVDFGPVTDVMAR
30_8564   SNLDRQTEVLVESIMAARLQHATVVSVMHRLEAVAAYDKVAVLDKGVLVDFGPVTDVMAR
31_1582   SNLDRQTEVLVESIMAARLQHATVVSVMHRLEAVAAYDKVAVLDKGVLVDFGPVTDVMAR
32_4537   SNLDRQTEVLVESIMAARLQHATVVSVMHRLEAVAAYDKVAVLDKGVLVDFGPVTDVMAR
33_9418   SNLDRQTEVLVESIMAARLQHATVVSVMHRLEAVAAYDKVAVLDKGVLVDFGPVTDVMAR
34_1782   SNLDRQTEVLVESIMAARLQHATVVSVMHRLEAVAAYDKVAVLDKGVLVDFGPVTDVMAR
35_10360  SNLDRQTEVLVESIMAARLQHATVVSVMHRLEAVAAYDKVAVLDKGVLVDFGPVTDVMAR
36_468    SNLDRQTEVLVESIMAARLQHATVVSVMHRLEAVAAYDKVAVLDKGVLVDFGPVTDVMAR
37_4956   SNLDRQTEVLVESIMAARLQHATVVSVMHRLEAVAAYDKVAVLDKGVLVDFGPVTDVMAR
38_4305   SNLDRQTEVLVESIMAARLQHATVVSVMHRLEAVAAYDKVAVLDKGVLVDFGPVTDVMAR
39_4737   SNLDRQTEVLVESIMAARLQHATVVSVMHRLEAVAAYDKVAVLDKGVLVDFGPVTDVMAR
40_9543   SNLDRQTEVLVESIMAARLQHATVVSVMHRLEAVAAYDKVAVLDKGVLVDFGPVTDVMAR
41_6669   SNLDRQTEVLVESIMAARLQHATVVSVMHRLEAVAAYDKVAVLDKGVLVDFGPVTDVMAR
42_10558  SNLDRQTEVLVESIMAARLQHATVVSVMHRLEAVAAYDKVAVLDKGVLVDFGPVTDVMAR
43_8051   SNLDRQTEVLVESIMAARLQHATVVSVMHRLEAVAAYDKVAVLDKGVLVDFGPVTDVMAR
44_10454  SNLDRQTEVLVESIMAARLQHATVVSVMHRLEAVAAYDKVAVLDKGVLVDFGPVTDVMAR
45_10126  SNLDRQTEVLVESIMAARLQHATVVSVMHRLEAVAAYDKVAVLDKGVLVDFGPVTDVMAR
46_1547   SNLDRQTEVLVESIMAARLQHATVVSVMHRLEAVAAYDKVAVLDKGVLVDFGPVTDVMAR
47_6749   SNLDRQTEVLVESIMAARLQHATVVSVMHRLEAVAAYDKVAVLDKGVLVDFGPVTDVMAR
48_9982   SNLDRQTEVLVESIMAARLQHATVVSVMHRLEAVAAYDKVAVLDKGVLVDFGPVTDVMAR
49_7293   SNLDRQTEVLVESIMAARLQHATVVSVMHRLEAVAAYDKVAVLDKGVLVDFGPVTDVMAR
50_5993   SNLDRQTEVLVESIMAARLQHATVVSVMHRLEAVAAYDKVAVLDKGVLVDFGPVTDVMAR
51_4791   SVMHRLEAVAAYDKVAVLDKGVLVDFGPVTDVMARCELFTG...................
52_121    SNLDRQTEVLVESIMAARLQHATVVSVMHRLEAVAAYDKVAVLDKGVLVDFGPVTDVMAR
53_1667   SNLDRQTEVLVESIMAARLQHATVVSVMHRLEAVAAYDKVAVLDKGVLVDFGPVTDVMAR
54_10488  SNLDRQTEVLVESIMAARLQHATVVSVMHRLEAVAAYDKVAVLDKGVLVDFGPVTDVMAR
55_7733   SNLDRQTEVLVESIMAARLQHATVVSVMHRLEAVAAYDKVAVLDKGVLVDFGPVTDVMAR
56_3982   SNLDRQTEVLVESIMAARLQHATVVSVMHRLEAVAAYDKVAVLDKGVLVDFGPVTDVMAR
57_392    SNLDRQTEVLVESIMAARLQHATVVSVMHRLEAVAAYDKVAVLDKGVLVDFGPVTDVMAR
58_10291  SNLDRQTEVLVESIMAARLQHATVVSVMHRLEAVAAYDKVAVLDKGVLVDFGPVTDVMAR
59_5792   SNLDRQTEVLVESIMAARLQHATVVSVMHRLEAVAAYDKVAVLDKGVLVDFGPVTDVMAR
60_8712   SNLDRQTEVLVESIMAARLQHATVVSVMHRLEAVAAYDKVAVLDKGVLVDFGPVTDVMAR
61_6716   SNLDRQTEVLVESIMAARLQHATVVSVMHRLEAVAAYDKVAVLDKGVLVDFGPVTDVMAR
62_5599   SNLDRQTEVLVESIMAARLQHATVVSVMHRLEAVAAYDKVAVLDKGVLVDFGPVTDVMAR
63_304    SNLDRQTEVLVESIMAARLQHATVVSVMHRLEAVAAYDKVAVLDKGVLVDFGPVTDVMAR
64_10473  ............................................................
65_6310   SNLDRQTEVLVESIMAARLQHATVVSVMHRLEAVAAYDKVAVLDKGVLVDFGPVTDVMAR
66_8714   SNLDRQTEVLVESIMAARLQHATVVSVMHRLEAVAAYDKVAVLDKGVLVDFGPVTDVMAR
67_2350   SNLDRQTEVLVESIMAARLQHATVVSVMHRLEAVAAYDKVAVLDKGVLVDFGPVTDVMAR
68_2447   SNLDRQTEVLVESIMAARLQHATVVSVMHRLEAVAAYDKVAVLDKGVLVDFGPVTDVMAR
69_5658   SNLDRQTEVLVESIMAARLQHATVVSVMHRLEAVAAYDKVAVLDKGVLVDFGPVTDVMAR
70_1277   SNLDRQTEVLVESIMAARLQHATVVSVMHRLEAVAAYDKVAVLDKGVLVDFGPVTDVMAR
71_3717   SNLDRQTEVLVESIMAARLQHATVVSVMHRLEAVAAYDKVAVLDKGVLVDFGPVTDVMAR
72_7846   SNLDRQTEVLVESIMAARLQHATVVSVMHRLEAVAAYDKVAVLDKGVLVDFGPVTDVMAR
73_10528  SNLDRQTEVLVESIMAARLQHATVVSVMHRLEAVAAYDKVAVLDKGVLVDFGPVTDVMAR
74_4899   SNLDRQTEVLVESIMAARLQHATVVSVMHRLEAVAAYDKVAVLDKGVLVDFGPVTDVMAR
75_5507   SNLDRQTEVLVESIMAARLQHATVVSVMHRLEAVAAYDKVAVLDKGVLVDFGPVTDVMAR
76_5432   SNLDRQTEVLVESIMAARLQHATVVSVMHRLEAVAAYDKVAVLDKGVLVDFGPVTDVMAR
77_10770  SNLDRQTEVLVESIMAARLQHATVVSVMHRLEAVAAYDKVAVLDKGVLVDFGPVTDVMAR
78_5904   SNLDRQTEVLVESIMAARLQHATVVSVMHRLEAVAAYDKVAVLDKGVLVDFGPVTDVMAR
79_9653   SNLDRQTEVLVESIMAARLQHATVVSVMHRLEAVAAYDKVAVLDKGVLVDFGPVTDVMAR
80_4462   SNLDRQTEVLVESIMAARLQHATVVSVMHRLEAVAAYDKVAVLDKGVLVDFGPVTDVMAR
81_7194   AVAAYDKVAVLDKGVLVDFGPVTDVMARCELFTG..........................
82_1022   SNLDRQTEVLVESIMAARLQHATVVSVMHRLEAVAAYDKVAVLDKGVLVDFGPVTDVMAR
83_9813   SNLDRQTEVLVESIMAARLQHATVVSVMHRLEAVAAYDKVAVLDKGVLVDFGPVTDVMAR
84_1902   SNLDRQTEVLVESIMAARLQHATVVSVMHRLEAVAAYDKVAVLDKGVLVDFGPVTDVMAR
85_5152   SNLDRQTEVLVESIMAARLQHATVVSVMHRLEAVAAYDKVAVLDKGVLVDFGPVTDVMAR
86_3385   SNLDRQTEVLVESIMAARLQHATVVSVMHRLEAVAAYDKVAVLDKGVLVDFGPVTDVMAR
87_10760  SNLDRQTEVLVESIMAARLQHATVVSVMHRLEAVAAYDKVAVLDKGVLVDFGPVTDVMAR
88_1588   SNLDRQTEVLVESIMAARLQHATVVSVMHRLEAVAAYDKVAVLDKGVLVDFGPVTDVMAR
89_1609   SNLDRQTEVLVESIMAARLQHATVVSVMHRLEAVAAYDKVAVLDKGVLVDFGPVTDVMAR
90_4023   SNLDRQTEVLVESIMAARLQHATVVSVMHRLEAVAAYDKVAVLDKGVLVDFGPVTDVMAR
```

1_4160	CELFTG
2_6143	CELFTG
3_6554	CELFTG
4_111	CELFTG
5_5742	CELFTG
6_9490	CELFTG
7_6313	......
8_2552	CELFTG
9_7745	CELFTG
10_2315	CELFTG
11_2035	CELFTG
12_9640	CELFTG
13_3674	CELFTG
14_6548	CELFTG
15_10828	CELFTG
16_6578	CELFTG
17_10233	CELFTG
18_9261	CELFTG
19_8450	CELFTG
20_1727	CELFTG
21_206	CELFTG
22_7439	CELFTG
23_3780	CELFTG
24_967	CELFTG
25_6595	CELFTG
26_8391	CELFTG
27_8170	CELFTG
28_5209	CELFTG
29_2181	CELFTG
30_8564	CELFTG
31_1582	CELFTG
32_4537	CELFTG
33_9418	CELFTG
34_1782	CELFTG
35_10360	CELFTG
36_468	CELFTG
37_4956	CELFTG
38_4305	CELFTG
39_4737	CELFTG
40_9543	CELFTG
41_6669	CELFTG
42_10558	CELFTG
43_8051	CELFTG
44_10454	CELFTG
45_10126	CELFTG
46_1547	CELFTG
47_6749	CELFTG
48_9982	CELFTG
49_7293	CELFTG
50_5993	CELFTG
51_4791	......
52_121	CELFTG
53_1667	CELFTG
54_10488	CELFTG
55_7733	CELFTG
56_3982	CELFTG
57_392	CELFTG
58_10291	CELFTG
59_5792	CELFTG
60_8712	CELFTG
61_6716	CELFTG
62_5599	CELFTG
63_304	CELFTG
64_10473	......
65_6310	CELFTG
66_8714	CELFTG
67_2350	CELFTG
68_2447	CELFTG
69_5658	CELFTG
70_1277	CELFTG
71_3717	CELFTG
72_7846	CELFTG
73_10528	CELFTG
74_4899	CELFTG
75_5507	CELFTG
76_5432	CELFTG
77_10770	CELFTG
78_5904	CELFTG
79_9653	CELFTG
80_4462	CELFTG
81_7194	......
82_1022	CELFTG
83_9813	CELFTG
84_1902	CELFTG
85_5152	CELFTG
86_3385	CELFTG
87_10760	CELFTG
88_1588	CELFTG
89_1609	CELFTG
90_4023	CELFTG
