# Supplementary material for: ABCC Transporter Gene MoABC-R1 Is Associated with Pyraclostrobin Tolerance in Magnaporthe oryzae
Source: J Fungi (Basel). 2023 Sep 11;9(9):917. doi: 10.3390/jof9090917 (PMC10532721; doi:10.3390/jof9090917)
Supplement: Supplementary file 1 [file jof-09-00917-s001.zip › Supplementary dataS5_Sequence aligment for the homology protein of MoABC-R1.pdf]

Supplementary data 3 Sequence alignment for the homology protein of MoABC-R1.

catrA *Botrytis cinerea*	----------------------------MFYTHAERMYAERAIEGREGAQIPLHSDNYRGEPIQPHDSSSSESEINNDGTWGEHEQGGPVNCSTAMHEYEDLRHQLTHLSKTRSQKSVATARKASAMKRTMTNASRRSRATAGEDIEAQE
BcatrB *Botrytis cinerea*	--MAAIEPEGFSSIVR--------PHEEHGNALTRALSSSSAFSDRKR----------------------------QRAYDSSDEDNKKE
FgABCC15 *Fusarium graminearum*	---MAVSGCTNDNSFGPIVQGCRGDFDFTLRF--------QNIILGILPAAIFILLALTR--------------------------------------VATLAFRS
FgABCC9 *Fusarium graminearum*	---MPDPVDKELQPSDTQAAP---LAPTNFER
FgABCG6 *Fusarium graminearum*	---------------------------------------MALPEANMSSTRSEQSSRSHDTIVGNEQPHSEKPAASAPGDQMSSDDEDEGPQTEEMIRRHSIVRDLARNYTNT-----------------------SHHFTGSSADLFNA
FgABC1 *Fusarium graminearum*	---------------------------------------MALPEANMSSTRSEQSSRSHDTIVGNEQPHSEKPAASAPGDQMSSDDEDEGPQTEEMIRRHSIVRDLARNYTNT-----------------------SHHFTGSSADLFNA
FcABC1 *Fusarium culmorum*	---------------------------------------MALPEANMSSTRSEQSSRSHDTIVGNEQPHSEKPAASAPGDQMSSDDEDEGPQTEEMIRRHSIVRDLARNYTNT-----------------------SHHFSGSSADLFNA
AtrB *Botryotinia fuckeliana*	---MSTLTVGDTALPRESRETMV---SIQGIRIVNYSSRR
AtrA *Botryotinia fuckeliana*	---MGVPDELPPGSSETDTIV-----------SSSQPTNRSPMDLISEAESLNLRRIATNQSKAQCRP----------------GSAAVPSHDNPPND
Pdr5p *Saccharomyces cerevisiae*	-----------------------------------MPEAKLN-------------NNVNDVTSYSSASSSTENAAD--------LHNYNGFDEHTEARIQKLARTLTAQSMQNSTQSAPNKSDAQSIFSS------GVEGVNPIFSDPEA
Snq2p *Saccharomyces cerevisiae*	----------------------------MSNIKSTQDSSHNAVARSSSASFAASEESFTGITHDKDEQSDTPADKL--------TKMLTGPARDTASQISATVSEMAPDVVS------------------------KVESFADALSRHTT
Cdr1p *Candida albicans*	---MSDSKMSSQDESKLEKAISQDSSSENHS-----------INEYHGFDAHTSENIQNLARTFTHDSFKDDSSAGLLKYLT------------HMSEVPGVNPYEHE
PMR1 *Penicillium digitatum*	---MEARRHDPTASVNTEDTAN------------EKSEVGDEKYTDAEVTRLAQQLTRQSTR------------------------FSVSPQNAENPFIE
ABC1 *Magnaporthe oryzae*	MSQPVEDPSHDQARNDNAQTTTDTGNASMPKTNGHDQESSATGISSSPADTLMDKEKQAAPTPEPSTEKAAVPDPAASVASDTAEDEFFDDNDSEQRRRNEMVQQLARTYTSRSNASAA-----------------ADEYGNANPFLIAS
ABC2 *Magnaporthe oryzae*	----------------------------------MDQKANDVASLEEGNKAPSTANTAVGSLPRSADPSINLEELRVAAHQKNPNGTSNVGSGISVARAEADFAELQRELTGMSRASR------------------RKSHASQRDAEKAN
ABC3 *Magnaporthe oryzae*	--MTAATPAD
ABC4 *Magnaporthe oryzae*	---MAFIRQTITLVKK
MoABC5 *Magnaporthe oryzae*	--MSVVLGVKTILLVL--
CaABC1 *Colletotrichum acutatum*	---MSLENEKPVGASEKST---EVAEKTAS
MoABC7 *Magnaporthe oryzae*	---MGGVGQCQWPVWQVDDLTPCF--------QHDYLRILLPAVVIGLSVLNLGFRSARHAAS---------------------RSKSPSTHAYAPVS
MoABC-R1 *Magnaporthe oryzae*	--MECPADADRVFGPAIQGCRSDFDFTLLF--------QDSVLGILPSSVLIILAAAR--------------------------------------LVFLARRH

BcatrA *Botrytis cinerea*	EEEKLEEAAADEDEFVLGDFLK--DGHFEKRNTKGSAKKVGVIYKNLTVQGVGATSTYVKTLPSAIMGTFGPDLYKLLTRFIPALPKPGSNGQRRDLIHDFTGCVRDGE----MLLVLGRPGSGCSTFLKAISNKR-GDYAGVTGEVSYG
BcatrB *Botrytis cinerea*	KSMAADWSLMPEL----------QAMQQQSDKDQAKRRDLGVTWKNLTVKGIGADAAINENVGSQ-------------FNIPKLIKEGRTKPPLRTLVDNSHGCVKPGE----MLLVLGRPGAGCTTLLKMLANTRGG-YAEVTGDVHFG
FgABCC15 *Fusarium graminearum*	RIVGGKVLQFTKLAVIAATAALQLVLLILSSTSNDESVDGDTFAVANSALGFSQW----------------------IVTLALSFAEHSRAPRPSSILTLYL-----------LLQIL----------------------LDVTRCRSYW
FgABCC9 *Fusarium graminearum*	SGESDE------------------DQIKESEEKRSAERRQSEEFNE---KNESRLADLEEVNTGASEAQ-------TISRATSG--------------------------
FgABCG6 *Fusarium graminearum*	ADANSPLNPSSEN-FNARAWARAMAKTMGENGSGF--RQSGLCFQDMNVFGYGAETDYQKDVGNV---------WLGLPDMVHQMISPNANKRRIDILRGFDGVINAGE----MCVVLGPPGSGCSTFLKSISGETNGIYIDDSTYFNYN
FgABC1 *Fusarium graminearum*	ADANSPLNPSSEN-FNARAWARAMAKTMGENGSGF--RQSGLCFQDMNVFGYGAETDYQKDVGNV---------WLGLPDMVHQMISPNANKRRIDILRGFDGVINAGE----MCVVLGPPGSGCSTFLKSISGETNGIYIDDSTYFNYN
FcABC1 *Fusarium culmorum*	ADANSPLNPSSEN-FNARAWARAMAKTMGENGSGF--RQSGLCFQDMNVFGYGAETDYQKDVGNV---------WLGLPNMVQQMIKPNAGKRRIDILRGFDGVVNAGE----MLVVLGPPGSGCSTFLKSISGETNGIYVDDSTYFNYN
AtrB *Botryotinia fuckeliana*	GFRFHNKIQMSAVPMAGLLMPQVKEQNEREAESGFKRRELGVTWQNLSVEVVSADAAVQEN-------------FLSQFNVPKLARESRNKPPLRTILDNSHGCVKPGE----MLLVLGRPGSGCTTLLKMLANQRLG-YKAVQGDVRYG
AtrA *Botryotinia fuckeliana*	DLEDATLDPNSAS-FSLEKWLR--AAVSDASQQGLSTPSGGIPLQKSDRPRAQTAALQLQPTVGS---------VLTAPLRSRVCCDTGEYEPRR-ILHGFDGVMKTGE----LLLVLGRPGTGCSTFLKAVCGETNGLHIDADSVLHYN
Pdr5p *Saccharomyces cerevisiae*	PGYDPKLDPNSEN-FSSAAWVKNMAHLSAADPDFYKPYSLGCAWKNLSASGASADVAYQSTVVNI---------PYKILKSGLRKFQRSKETNTFQILKPMDGCLNPGE----LLVVLGRPGSGCTTLLKSISSNTHGFDLGADTKISYS
Snq2p *Saccharomyces cerevisiae*	RSGAFNMDSDSDDGFDAHAIFE--SFVRDADEQGIHIRKAGVTIEDVSAKGVDASALEGATFGNI----------LCLPLTIFKGIKAKRHQKMRQIISNVNALAEAGE----MILVLGRPGAGCSSFLKVTAGEIDQFAGGVSGEVAYD
Cdr1p *Candida albicans*	EINNDQLNPDSEN-FNAKFWVKNLRKLFESDPEYYKPSKLGIGYRNLRAYGVANDSDYQPTVTNA---------LWKLATEGFRHFQKDDDSRYFDILKSMDAIMRPGE----LTVVLGRPGAGCSTLLKTIAVNTYGFHIGKESQITYD
PMR1 *Penicillium digitatum*	THEDSTLNPHSGN-FKAKNWMKNLLAIQSRDPERYPKRQAGLAFKNLSVHGFGSPTDYQKDVANS---------VLEIGAFFRTMAGTGK--QKIQILRDFDGLVKNGE----MLIVLGRPGSGCSTFLKTIAGEMNGIFKDANSHMNYQ
ABC1 *Magnaporthe oryzae*	EDPDSPLNPSGNN-FKAYAWAKAIAGMVAAEGGSF--RTIGICFQNMNVFGFGAATDFQKTVSNV---------WLEAANMLRTAVGMGKTTR-IDILRGFNGVVRNGE----MLVVLGPPGSGCSTFLKTIAGETNGLNVDQSAYFNYQ
ABC2 *Magnaporthe oryzae*	GEDLGETSSSESEPFDLESWIR--GGIQAEREAGIRPKHIGVYWDELTVKGMSAFTNYVETFPDAVIR------FFDYYTPIKNKLGLGGKAPEATLLDSFRGVCKPGE----MVLVLGKPGSGCTTFLKNITNQRYG-YTGVEGDVLYG
ABC3 *Magnaporthe oryzae*	GEKGFKLNP---KHLLAIHNFKRILTYGT---
ABC4 *Magnaporthe oryzae*	NFLIILWRHSTATVLRAFLLPIFLAAFLSFARFFFVPPARNGVADSLAIRSLGDSLRV-------------------------------AGETSRNTVVFVDGGHKTGD-------------------IDRVINNLTS-QVEDAGKTVFR
MoABC5 *Magnaporthe oryzae*	---EAVPKTIIAATIPGTAQNGQ---
CaABC1 *Colletotrichum acutatum*	NGHSDRASSHGDS---TEVDASIQPKN---------------------------------QTANITSN
MoABC7 *Magnaporthe oryzae*	NGDNSRPGAHRTD------ISPDDDAIAQDDEDDDEGLAIGGGRLAL------------------------------VKTATKGSIVQADTPPAQTLSVVVEELAIAGLVAVYVIALLSPKAHGSYTLTGTIIGLTTWVYVLVLATLRLF
MoABC-R1 *Magnaporthe oryzae*	AVASLNWLYHSKIGFNILSFALQLTILVQRCRIQDLETSAAIPWATL---GLAATCAL----------------------FLLSAVEHRRSARPSSLIVAYL-----------FIFVLTEATRARTYYLKRQTVAAS--ITAANCCVKFV

BcatrA *Botrytis cinerea*	GISAEEQAKHYRGEVNYNEEDDQHFPSLTVEQTLDFSLLNKTKKHEKGDI-------PTIITALLKMFGISHTRHTLVGDAFVRGVSGGERKRVSIAETLATKSTVVSWDNSTRGLDASTALDYANSLRVMTDISNRTTLVTLYQAGEQI
BcatrB *Botrytis cinerea*	SLNHTEAHQ-YRGQIVMNTEEELFFPTLTVGQTIDFATRMKVPFHRPSNSGSPEEYQQANRDFLLKSMGISHTHETKVGNEYVRGVSGGERKRVSIIEMLASRGSVMCWDNSTRGLDASSALDYTKAIRAMTDIFGMASIVTLYQAGNGI
FgABCC15 *Fusarium graminearum*	LLATSFQITRYAGVFTATIGLKVITLLLELQNKARWMTWRKEDHSPEETS---SLFNLGVYFW------LIGIFRNGFKKTLSIKDDLYLLDHEMQSKI-----
FgABCC9 *Fusarium graminearum*	--PKDPPKKSWHKKM----------------NPLRWGSPPPVPEKSVKSR---EHEAGFLSKLTFQW------MSPLMHAGYRRPLE-PNDIWTVNPDRSVEPLTLKM
FgABCG6 *Fusarium graminearum*	GIPAEEMHKSHAGETIYTAEVDIHFPMLSVGDTLTFAARARCPQNLPPGI-DHNLYSEHMRDVVMAMYGISHTINTQVGDNYIRGVSGGERKRVTIAEATLSNAPFQCWDNSTRGLDSANAIEFCKTLRLQSELFGQTCAVSIYQAPQTA
FgABC1 *Fusarium graminearum*	GIPAEEMHKSHAGETIYTAEVDIHFPMLSVGDTLTFAARARCPQNLPPGI-DHNLYSEHMRDVVMAMYGISHTINTQVGDNYIRGVSGGERKRVTIAEATLSNAPFQCWDNSTRGLDSANAIEFCKTLRLQSELFGQTCAVSIYQAPQTA
FcABC1 *Fusarium culmorum*	GITADEMHKYHAGETIYTAEVDIHFPMLSVGDTLTFAARARCPQNLPAGI-NHNLYSQHMRDVVMAMYGISHTVNTQVGDNYIRGVSGGERKRVTIAEATLSNAPFQCWDNSTRGLDSANAIEFCKTLRLQSELFGQTCAVSIYQAPQTA
AtrB *Botryotinia fuckeliana*	SMTAKEAEQ-YRGQIVMNTQEELFFPSLTVGETMDFATRLKVPNRLPNGVESPEAYREEYKKFLLQSMGISHTVDTKVGNEFIRGVSGGERKRVSIIECLGTRASVFCWDNSTRGLDASTALEWTKTIRTMTDVLGLSTIVTLYQAGNGI
AtrA *Botryotinia fuckeliana*	GVSQQRMMKEFKGEVVYNQEVDKHFPHLTVRQTLEFGAAARTPAHRFQNM-SRDEFASYAASVVMAIFGLSHTHNTKVGNDFVRGVSGGERKRVSIAEMALAMTPFAAWDNSSRGLDSATALKFVQALRLSADLAGAAHAVAIYQASQSI
Pdr5p *Saccharomyces cerevisiae*	GYSGDDIKKHFRGEVVYNAEADVHLPHLTVFETLVTVARLKTPQNRIKGV-DRESYANHLAEVAMATYGLSHTRNTKVGNDIVRGVSGGERKRVSIAEVSICGSKFQCWDNATRGLDSATALEFIRALKTQADISNTSATVAIYQCSQDA
Snq2p *Saccharomyces cerevisiae*	GIPQEEMMKRYKADVIYNGELDVHFPYLTVKQTLDFAIACKTPALRVNNV-SKKEYIASRRDLYATIFGLRHTYNTKVGNDFVRGVSGGERKRVSIAEALAAKGSIYCWDNATRGLDASTALEYAKAIRIMTNLLKSTAFVTIYQASENI
Cdr1p *Candida albicans*	GLSPHDIERHYRGDVIYSAETDVHFPHLSVGDTLEFAARLRTPQNRGEGI-DRETYAKHMASVYMATYGLSHTRNTNVGNDFVRGVSGGERKRVSIAEASLSGANIQCWDNATRGLDSATALEFIRALKTSAVILDTTPLIAIYQCSQDA
PMR1 *Penicillium digitatum*	GISDKEMRNQFRGEAIYTAETDVHFPQLSVGNTLKFAALARAPRNRLPGV-SRDQYAEHMRDVVMAMLGLSHTINTRVGNDFIRGVSGGERKRVSIAEATLCGSPLQCWDNSTRGLDSANALEFCKTLNLMSKYSGTTCAVAIYQASQSA
ABC1 *Magnaporthe oryzae*	GLSAEEMHKRHRGEAIYTAEVDVHFPQLSVGDTLTFAANARAPRRAPPGV-SKTLFANHIRDVVMAIFGISHTINTRVGNEYIRGVSGGERKRVTIAEAALSGAPLQCWDNSTRGLDSANAIEFCKTLRVCTRLFQTTACVSIYQAPQSA
ABC2 *Magnaporthe oryzae*	PFTAKEFEK-YRGEAVYNQEDDIHHATLTVEQTLGFALDCKVPGKLPAGI-TKAQFKKDVITMLLKMFNIEHTRNTVVGGSLVRGVSGGERKRVSVAEMMITSGSILAWDNSTRGLDASTALDFIKSLRIQTNLYKTATFVSLYQASENI
ABC3 *Magnaporthe oryzae*	---KWDKIVLGVS---------------------------------
ABC4 *Magnaporthe oryzae*	LDRENEIQRVCKSSLR--------------GVSQCYGAAVFNGSPDFGGI--WNYTLR-MDGA-------------------------------
MoABC5 *Magnaporthe oryzae*	--EEKQESTDKRG-------------------------KRQAPHEQTAGV---YARRSFWW------LNRILWTGFRNSKLKADDLYEIDDDIKTERYSQAL
CaABC1 *Colletotrichum acutatum*	LEKADSK-----------------------------AIAPKDPSDDPYEH---LPANEAAILKRQVFTPEVKAGI---MTLYR-----
MoABC7 *Magnaporthe oryzae*	LGNTQ-----WRVPHLWNHTAAIYSCQWLFLIGI-FRSAMVHPSSKLAQI-------LVIVEFALTSLLFFMAITTRKGNKTVLLEWEDGIPPARENLASLFSSFTFSW------VDQIVWQGYKEPFEMGKVWNLLPKDKAATVLSH--
MoABC-R1 *Magnaporthe oryzae*	LLILEEQKKTLRSGSD--------------------GSRKKAASEDLAGP---TNQTFFLW------LNRLFLTGYRRAFTTTD------------------

BcatrA *Botrytis cinerea*	YELMDKVLVIDEGRMVYSGPAGDAKKYFERIGYYCPPRQTTADFLTACTDPAE-RRFQKDF--EGP-IPKGPVELEKAFRESEDYRLLQEDVESYERMLHETDHADARQ-FKASVEETKSKTVGPRSPYTVSFFRQVLACTKREVELTLG
BcatrB *Botrytis cinerea*	YNLFDKVLVLDEGKQIYYGPMKQARPFMEELGFICDDSANVADFLTGVTVPTE-RKIRDEF--QNR-FPRTAGEILAAYNRHSIKNEMEKEYDYPTTAIAKERTED----FRTSVQHEKNPKLGKDSPLTTSFMTQVKACVIRQYQIIWG
FgABCC15 *Fusarium graminearum*	--LLDRLTISFAKSSANVG----------------KRFRLAKALGRALIVPLI--------------LPVVPRIAMIGFQYAQP--FFIHALLEYL-IHKDVPK
FgABCC9 *Fusarium graminearum*	KESFQRRVESGEKNPLFWAMHETFKTEFWTGG-ACALYTSIIQVISPFTLRYL--------------IQFAADAYVANQTGGPPPHIGK--GVGLAVGITLMQITQSLGT
FgABCG6 *Fusarium graminearum*	YDLFDKALVIYEGRQIFFGPADEAKAYFINLGFECPDRQTTPDFLTSMTAPSE-RVVRPGW--ENK-VPRTPDEFHARWKESQQYQIVRAEIESYKSLYPLNGSSADA--FRENKHSAQAKGQRLKSPFTLSYMQQVQLCLWRGFRRLLG
FgABC1 *Fusarium graminearum*	YDLFDKALVIYEGRQIFFGPADEAKAYFINLGFECPDRQTTPDFLTSMTAPSE-RVVRPGW--ENK-VPRTPDEFHARWKESQQYQIVRAEIESYKSLYPLNGSSADA--FRENKHSAQAKGQRLKSPFTLSYMQQVQLCLWRGFRRLLG
FcABC1 *Fusarium culmorum*	YDLFDKALVIYEGRQIFFGPADEAKAYFINLGFECPDRQTTPDFLTSMTAPSE-RVVRPGF--ENK-VPRTPDEFHACWKQSQQYQIVRTEIESYKSLYPINGSSADA--FRENKHSAQAKGQRLKSPFTLSYMQQVQLCLWRGFRRLLG
AtrB *Botryotinia fuckeliana*	YDLFDKVLVLDEAKQIYYGPMTQARPYMETLDFVCREGSNVADFLTGVTVPTE-RKIRSGF--EAR-FPRNADAMLEEYNKSAVKADMISEYDYPDSEYAKLRTED----FKQAIAEEKAKQLPKSSPFTVDFMNQVKICVTRQYQILWG
AtrA *Botryotinia fuckeliana*	YEVFDKVTVLYEGRMIFFGPTGTAKEYFERMGWVCPARQTTGDFLTSITNPLE-RKARAGM--EDV-VPKTPKDFEIYWRQSPEYKTLLGEMTGVRDQHPTGNDEQASAELRARKENSQSRNSRAASPYILSIPMQIKLNTKRAYQRIWN
Pdr5p *Saccharomyces cerevisiae*	YDLFNKVCVLDDGYQIYYGPADKAKKYFEDMGYVCPSRQTTADFLTSVTSPSE-RTLNKDMLKKGIHIPQTPKEMNDYWVKSPNYKELMKEVD----QRLLNDDEASREAIKEAHIAKQSKRARPSSPYTVSYMMQVKYLLIRNMWRLRN
Snq2p *Saccharomyces cerevisiae*	YETFDKVTVLYSGKQIYFGLIHEAKPYFAKMGYLCPPRQATAEFLTALTDPNGFHLIKPGY--ENK-VPRTAEEFETYWLNSPEFAQMKKDIAAYKEKVNTEKTKEV---YDESMAQEKSKYTRKKSYYTVSYWEQVKLCTQRGFQRIYG
Cdr1p *Candida albicans*	YDLFDKVVVLYEGYQIFFGKATKAKEYFEKMGWKCPQRQTTADFLTSLTNPAE-REPLPGY--EDK-VPRTAQEFETYWKNSPEYAELTKEIDEYFVECERSNTRET---YRESHVAKQSNNTRPASPYTVSFFMQVRYGVARNFLRMKG
PMR1 *Penicillium digitatum*	YDVFDKVTVLYEGRQIYFGRTTEAREFFTNMGFHCPDRQTTADFLTSLTSPAE-RVVKPGF--EKM-VPRTPDEFAKGWKNSAAYKELQKEIDDYNTQYPIGGESFQQ--FVESRKAMQSKGQRAKSPYTLSVAEQVQICVTRGFQRLKS
ABC1 *Magnaporthe oryzae*	YDMFDKAVVLYEGYQIYFGPADEAKQYFVNLGFECPAR-TTPDFLTSMTAPHE-RIVRPGF--EGK-APRTPEEFAIAWENSAEYTALQADIEEYKSSHPINGPDAEA--FRKSRAAQQGRGQRPNSPYTLSFYQQTKLCLWRGWKRLLG
ABC2 *Magnaporthe oryzae*	YKLFDKVLVIDAGRQVYFGPATEARGYFEGLGFLPRPRQTTPDYVTGCTDEYE-RAYSEGYSPDN--APHSPETLAEAFKKSDFAKRLDNEMVEYRESLKEDQQKYED--FKIAVKEGKRTGAK-KSVYTVGFHRQVWALMKRQTVLKLQ
ABC3 *Magnaporthe oryzae*	---------------------------------------TVSSVATGLTIPLMVVV-----------FARLIGIFTDFYRQGSTVTGAQ---FSSQVNQCVYNIIYLFVA
ABC4 *Magnaporthe oryzae*	LGPLGRIDVESNTRDAQI--------------FVLPFQRAVDAAITGLNSSSN----------SSP-LSNSHEYVFTSLTQSERDEEIR--V
MoABC5 *Magnaporthe oryzae*	ASAWGRAD-------------------------KSKKRALLRSTLSVLKWPLL--------------IPALPRFAQIFLNLAQPLLLLR--LLQHVSSPPSAAGTEAEAQER
CaABC1 *Colletotrichum acutatum*	YSSTNDLLILSIS--------------------------AFTSIVVGAALPLMTVI-----------FGNLQGTFQNYFAGTMSKADFNSEMA--S
MoABC7 *Magnaporthe oryzae*	YRRVKKTTKLYWHLLRYF----------------------KGDLLSQAGWAVM----------SGM-FTFAPTMLLKAILEYVEEPESA--------------------------------------PRSVVWLYVILLPVTDIIRSLGD
MoABC-R1 *Magnaporthe oryzae*	LELISSPLYVKSIRAQFHGMTNGQTANGHSL-FSSSTNTFALQAFTSLGSYAL--------------APVIPRLAVTGFTFSQ--SFLVTALLDYLENGRDRPA

BcatrA *Botrytis cinerea*	DKTTLYTKFFIIISNSLIVGSLFHGQ--SSNTLGN-----------FSRGGTLFFSILFLGWL---------QLSELMKAVGGRPIISRHKDYA-FYRPSAVVVARVVQDFPLLLLQVVPFSIVV-YFITGLDVDAGKYFIYVLFIYLTT
BcatrB *Botrytis cinerea*	DKATFIIKQLSTLAQALIAGSLFYNA--PANSSGL-----------FVKSGALFLSLLFNALL---------AMSEVTDSFSGRPVLAKHKAFA-FYHPAAFCIAQIAADIPVLLVQVSHFSLVM-YFMVGLRQDAGAFFTYWILIFAAT
FgABCC15 *Fusarium graminearum*	NDGYGLIGAALIIYAGIAISDALFWY--FHQRCLY-----------MARGC--LASYVYRSTT-------------------QGKMTDIGDAAVLTLMSTDVERIIYGFHGLHDFWANIIEIGLGC-FLLQRQLG--LAFISPIVVILLCV
FgABCC9 *Fusarium graminearum*	NHYIYRGMTVGGQSRGVLIGLIYEKS--LVISGRA-----------KAEGA--LQSNVRDAEDDDQAKKKAKKAKKAKPDASDGTGWGNGRITA-LQSVDTYRVDQASALFHMVWTSPILCLLTL-ALL--LVNITYSALAGYGLLVIGM
FgABCG6 *Fusarium graminearum*	SPGVTIFQLIANTAVAFIASSLFYNM--KPETGDF-----------FKRGATLFLAVLSNAFA---------SALEILTQYSQRPIVEKQARYA-FYHPSAEAFASILVDMPYKITNSILFNVTL-YFMTNLNRDAGAFFFFLLVSFIMV
FgABC1 *Fusarium graminearum*	SPGVTIFQLIANTAVAFIASSLFYNM--KPETGDF-----------FKRGATLFLAVLSNAFA---------SALEILTQYSQRPIVEKQARYA-FYHPSAEAFASILVDMPYKITNSILFNVTL-YFMTNLNRDAGAFFFFLLVSFIMV
FcABC1 *Fusarium culmorum*	SPGVTIFQLIANTVVAFIASSLFYNM--KPETGDF-----------FKRGATLFLAVLSNAFA---------SALEILTQYSQRPIVEKHARYA-FYHASAESFASILVDMPYKILNSILFNVTL-YFMTNLNRDAGAFFFFLFVSFIMV
AtrB *Botryotinia fuckeliana*	DKATFIIKQVSTLIQALIAGSLFYDA--PNNSGGL-----------FVKSGALFFSLLYNSLL---------AMAEVTESFQGRPVLIKHKSFA-FFHPAAFCIAQIAADIPVLIFQVTIFALPV-YFMVGLEMDAGVFFTYWILVFATT
AtrA *Botryotinia fuckeliana*	DMSSTMSTVVGQIVIALITGVVFYDS--PNTTAGF-----------QSKGGTLFYAVLLNALT---------AMSEITSLYSQRPIVEKQASYA-FYHPATEAIAGVVSDVPVKFLLAVAFNVIM-YFLANLRREPAQFFIYFLMSFTVM
Pdr5p *Saccharomyces cerevisiae*	NIGFTLFMILGNCSMALILGSMFFKIMKKGDTSTF-----------YFRGSAMFFAILFNAFS---------SLLEIFSLYEARPITEKHRTYS-LYHPSADAFASVLSEIPSKLIIAVCFNIIF-YFLVDFRRNGGVFFFYLLINIVAV
Snq2p *Saccharomyces cerevisiae*	NKSYTVINVCSAIIQSFITGSLFYNT--PSSTSGA-----------FSRGGVLYFALLYYSLM---------GLANI--SFEHRPILQKHKGYS-LYHPSAEAIGSTLASFPFRMIGLTCFFIIL-FFLSGLHRTAGSFFTIYLFLTMCS
Cdr1p *Candida albicans*	DPSIPIFSVFGQLVMGLILSSVFYNL--SQTTGSF-----------YYRGAAMFFAVLFNAFS---------SLLEIMSLFEARPIVEKHKKYA-LYRPSADALASIISELPVKLAMSMSFNFVF-YFMVNFRRNPGRFFFYWLMCIWCT
PMR1 *Penicillium digitatum*	DYSLTISALIGNTIMALIVGSVFYQL--PDDVTSF-----------YSRGALLFFAVLLNSFS---------SALEILTLYAQRPIVEKQARYA-MYHPFAEAISSMLCDMPYKILNAITFNVTL-YFMTGLRQNAGAFFTFMLFSFVTT
ABC1 *Magnaporthe oryzae*	DPTLTVGALFANTLMALVISSIFFNL--QMTTSSF-----------FQRGALLFFACLLNGFA---------AALEILILFAQRPIVEKHDRYA-LYHPSAEAVASMLCDMPYKVFNAIVFNLVL-YFMANLRREPGAFFFYLLISFATV
ABC2 *Magnaporthe oryzae*	DRMALFLAWMRTILIAIVVGTLYINL--GQTSATS-----------FSKGGLMFISLLFNAFE---------AFAELGSTMLGRGIVNKHKAYA-FHRPSALWIGQIFVDQAFGVPRVLAFSIIV-YFMTNLFRSAGAFFMFFLFIMLGN
ABC3 *Magnaporthe oryzae*	RIIFSYISNLGFRMFSLRISSTIRTV---------------------------YLRSLFALPI---------SVIDAIPAGQTAAIVTGTASL--LQVGISEKLGGGIASLASVASSVVVA-----LVFNWLLT----FVTIAGLAFIA-
ABC4 *Magnaporthe oryzae*	RYQQAVTNFMGVAFILAIIGVCYHLT-------GF-----------MATEREIGMSTLIESMM-------------------PTRQRWQAQAARLLSYHIAFDVI---------------------YLPGWIIGALILQLGVFRHTSTAM
MoABC5 *Magnaporthe oryzae*	RVGQGLIIATILVYFGLAVFNGYYGS--KINRAVV-----------LVRGS--LIDLIYRHML---------------ELDLTTCKTTAASGPAGLVTMDVERVDLAVDKLHALWAAPIEVGLGI-FLIARQVG--WACIGTAVVAAGCT
CaABC1 *Colletotrichum acutatum*	LVLYFVYLAIGVFVCQYITTVGFITG--EHISAKI---------------REHYLQSCMRQNI---------GFFDKLGAGEVTTRITADTNL--IQDGISEKVGLTLAAIATFISAFVIG-----FVHYWKLT-----LILLSTVVALL
MoABC7 *Magnaporthe oryzae*	NRALWIGRKICINVRAILVGEIYAKALRRKAATGKDTVLGSEKKEDKPKGG--FISKIKKMLCLGDNDESEDGKDGDKDKEDSSDEQANHGTIINLMSVDSFNVSEVTSYLHFLFASAPTQLLVSVVLLYQVLG--MSAIPGFVVMVLLL
MoABC-R1 *Magnaporthe oryzae*	SHGYGLLGAYAFVYIGIAVSNSWYSR--HTYKSVS-----------IIRGG--LIVSIFEKVL---------------RLGEDSSIEAKATT---LMISDVQRIVGGLVYIHEVWAGVLETALAT-YLLQRVMG--VSSVAMLGLALSCG

BcatrA *Botrytis cinerea*	ICITSLYRMFAALSPSIDDAVRFSGIGLNLLIIYTGYVIPKPQLVSEYIWFGWLYYINPLSYSFEAVISDEFYNKNITCAPDQIVPSG--PGYTNPE--FQGCAST-GAEVGSLSVSGARYLEQSFN-YSRSHLWRNFGVVIAWTVLYII
BcatrB *Botrytis cinerea*	MCMTALFRAVGAGFSTFDAASKVSGFLVSALIMYTGYMIQKPDMHP---WFVWIYWIDPLAYGFSAILANEFKGQIIPCVANNLVPNG--PGYADLA--FQACAGVGGALPGATSVTGEQYLNSLS--YSSSNIWRNFGILWAFWVLFVV
FgABCC15 *Fusarium graminearum*	AATTAIAWAIGERQSRWMAKIESRVGLTSSIISNIKSLRI----SG---ITAPVRDLVQKMREQELSIGNKFRWLLIMTATVAFVPSA--------------------MSPVVAFAFTNEQLDTLKI-------FVSFSFITLLTNPLGA
FgABCC9 *Fusarium graminearum*	PFLTRAIRSLFHRRRAINLITDQRVSLTQEILQSVRFVKY----FG---WEKAFLERLGDLRNKEIRAIQILLAIRNALNAVSM-----------------------SLPIFASMLSFICYSLTHNG-LTAAEVFSSLALFNGLRIPLNL
FgABCG6 *Fusarium graminearum*	LAMSGVFRSIASLSRTLSQAMVPASLLILALVIFAGFVVPVDYMLG---WCRWINYLDPVAYGFESLMVNEFSGRNFTCT--AFVPNAQIPGYADVGGLNRACSTV-GAIPGQSYVNGDAYINLEYK-YFHAHKWRNVGILIAMTIFNHV
FgABC1 *Fusarium graminearum*	LAMSGVFRSIASLSRTLSQAMVPASLLILALVIFAGFVVPVDYMLG---WCRWINYLDPVAYGFESLMVNEFSGRNFTCT--AFVPNAQIPGYADVGGLNRACSTV-GAIPGQSYVNGDAYINLEYK-YFHAHKWRNVGILIAMTIFNHV
FcABC1 *Fusarium culmorum*	LAMSGIFRSIASLSRTLSQAMVPASLLILALVIFAGFVVPVDYMLG---WCRWINYLDPVAYAFESLMVNEFSGRNFTCT--AFVPNPQIPGYADVGALNRACSTV-GAIPGQSYVNGDAYINLEYK-YFHSNKWRNVGILIAMMIFNHV
AtrB *Botryotinia fuckeliana*	MAMTAVFRACGAAFKTFDDASKVSGFLISALIMYTGYMIRKPEMHP---WFVWIYWIDPLAYGFDALLSNEFHGKIIPCVGTNLVPAG--PGYENAT--TQSCTGVGGSIPGRNYVTGDDYLASLS--YSHGHVWRNFGILWAWWALFVV
AtrA *Botryotinia fuckeliana*	FVMSAVFRTMAAVTKNAAQAMGLAGVLMLALVVYTGYVLPVPSMHP---WFEWIHYLNPIYYAFEAMIANEFHGRDFDCI--AFVPSM-----QIWTGDSFSCSSL-GSVAGERMVSGDSYINFNYT-YTYSHVWRNFGVLLAFLIGFMA
Pdr5p *Saccharomyces cerevisiae*	FSMSHLFRCVGSLTKTLSEAMVPASMLLLALSMYTGFAIPKKKILR---WSKWIWYINPLAYLFESLLINEFHGIKFPCA--EYVPRG--PAYANISSTESVCTVV-GAVPGQDYVLGDDFIRGTYQ-YYHKDKWRGFGIGMAYVVFFFF
Snq2p *Saccharomyces cerevisiae*	EAINGLFEMVSSVCDTLSQANSISGILMMSISMYSTYMIQLPSMHP---WFKWISYVLPIRYAFESMLNAEFHGRHMDCA-NTLVPSG--GDYDNLSDDYKVCAFV-GSKPGQSYVLGDDYLKNQFQ-YVYKHTWRNFGILWCFLLGYVV
Cdr1p *Candida albicans*	FVMSHLFRSIGAVSTSISGAMTPATVLLLAMVIYTGFVIPTPSMLG---WSRWINYINPVGYVFESLMVNEFHGREFQCA--QYVPSG--PGYENISRSNQVCTAV-GSVPGNEMVSGTNYLAGAYQ-YYNSHKWRNLGITIGFAVFFLA
PMR1 *Penicillium digitatum*	LTMSMIFRTIASYSRTLSQALVPAAILILGLVIYTGFTIPTRNMLG---WSRWMNYIDPIAYGFETLIVNEFHGRNFPCNPESFIPAG--DSYADVGRFNKICSAK-GAVAGQNFVSGEAYYTASFQ-YSNSHRWRNMGIMIGFMVFFMV
ABC1 *Magnaporthe oryzae*	LAMSMMFRTIASMSRSLSQAMVPAAAIILILIIFTGFVIPLDYMLP---WCRWLNYIDILAYSFESLLINEFAGQRYTCT--EFVPRAEFPGYGDLSGTNRVCQAV-GSVAGQPFVKGEDYLYSSFR-YESANKWRNFGILIAFMIFFCS
ABC2 *Magnaporthe oryzae*	IAMTLFFRIIGCISIDFDYAVKFAVVTITLLITTSGYIIQYQSQQV---WLRWIFWINPLGLMFSSMMGNEFSRIDMTCTAESLIPSG--PGYNNPE--NQVCTLP-GSKPGSLEVSGSDYIRTGFA-YDPNDIWRNFGIVMGLVAFFLI
ABC3 *Magnaporthe oryzae*	IVYVIFTPLVGKKALEVHEADVKASSIATEAFTSVRMLAA----CG---AENKVARRYAVHVDESYQKGLRMAWLVGVQQMFVFFGVY-------------------ATFALAFYFAFRMYNTSITT--TPEDLIVVLLCVMMMATSIGQ
ABC4 *Magnaporthe oryzae*	YVFYNITAGLALSSMSIFGAAFFKKAQLSGVVVTIVYC-----------VLAIVAQAISYPSTAQVTI---LSLLFAPCNYVYFIANT-------------------ARWEAKKWPTDLTDVPPNSAWDIPAIWLWVFLIIQIIVYP--I
MoABC5 *Magnaporthe oryzae*	LATTRISRLLPTRQKAWNQSVQRRVALTSTALNNIKSIKMM---GG---FERHVADRLQAAREAELDASASFRKCMTIVNTLVMVPRQ-------------------LSAPLVLTLYALGVNNNNGTLLTASQAFTTLALIEIVAVPMGL
CaABC1 *Colletotrichum acutatum*	LSMGGGSTFIVKYSKQSIESYAHGGSLADEVISSIRNAVA----FG---TQDRLAKQYDAH-----LTKAEFFGYKVKSAIGVMVALMMTILYLNYGL---------AFWQGSKFLIDDG--------IKLSNILIIMMSIMIGAFNLGN
MoABC7 *Magnaporthe oryzae*	PVNIGFGRAFNSTQKKIMACSDKRIHSTNEVLQNIRIIKY----FA---WEHRFSESVDEKRKAELKALRA-RYMVWACAVAVWNTVP----------------------LLITFFSFLMYTTVEKRPLHPSIAFTSISLFMLLRHPLDQ
MoABC-R1 *Magnaporthe oryzae*	VGAYFVAGNMSLQQGKWLKAMEQRIDATKRLCDSLKAVKM----RG---AETRVSRVVNELRRLEIQAARPFRALITASVILSYSTMT--------------------LSPLLVFAAYIGVNGNDDN-LDSATMFSSLVLIALLGSPLVH

BcatrA *Botrytis cinerea*	VTAIATEVFDFTTGGG---GALEFK----RSKAAKNKVKAENATPDEENSPASTSPVPTSGASSSNTLEPPQEEALK--DITGSESVFTWE-----------------------------NVEYTVPYLGGERKILNGVNGYAKPGIMVA
BcatrB *Botrytis cinerea*	LTIYYTSNWSANGGKS---GILLIPREKAK-KNTA--ILKAANAGDEESQAIEEKRQVQSRPASQDTKVAEESDD----QLMRNTSVFTWK-----------------------------NLTYTVKTPSGDRVLLDNVQGWVKPGMLGA
FgABCC15 *Fusarium graminearum*	MFQSVPAVIAAFVCLE---RIQKFL----EIEPRVDYRKSSRPLSPADGSLLEQDDEKPLGLGQGASKS----------LISIVDGSFGW---------------------------------------TSEKMTLTGISVDVPVGKLTL
FgABCC9 *Fusarium graminearum*	LPMVLGQVIDAWGSVQ---RIEEFL------LQEE--T-----VEDTVFDAKGDD------------------------AIRLEDASFTWEKSHKEEAGKEENGKKEKTKQAPPPQVESSGDDTSTLVEEREPFKLQDLNFDVKRNELVA
FgABCG6 *Fusarium graminearum*	VYIVATEFISAKKSKG---EVLVFR------------RSNMPSKAKSDPEASSSRPIPTTEKNNNEVA-----------NIQGSTSVFHWN-----------------------------DVCYDIKIKGEPRRILDHVDGWVKPGTLTA
FgABC1 *Fusarium graminearum*	VYIVATEFISAKKSKG---EVLVFR------------RSNMPSKAKSDPEASSSRPIPTTEKNNNEVA-----------NIQGSTSVFHWN-----------------------------DVCYDIKIKGEPRRILDHVDGWVKPGTLTA
FcABC1 *Fusarium culmorum*	VYIVATEYISAKKSKG---EVLVFR------------RSNMPSKAKSDPEAASSRPIPTTEKNNNEVA-----------NIQGSTSVFHWN-----------------------------DVCYDIKIKGEPRRILDHVDGWVKPGTLTA
AtrB *Botryotinia fuckeliana*	VTIIATSRWKGASENG---PSLLIP----R-ESVEKHRQHGHRDEESQSNEKTSTKGKSEGVQDSSDIDN---------QLVRNTSVFTWK-----------------------------DLCYTVKTPSGDRQLLDHVYGWVKPGMLGA
AtrA *Botryotinia fuckeliana*	IYFLASELNSSTTSTA---EALVFR----R-GHVPEYMRPGYTRPTDEEKAVTQSDIKPSSPSPTNPIL----------PLPPQRDIFTWK-----------------------------DISYDIEIKGEPRRLLDDVSGWVKPGTLTA
Pdr5p *Saccharomyces cerevisiae*	VYLFLCEYNEGAKQKG---EILVFPRSIVK-RMKK--RGVLTEKNANDPENVGERSDLSSDRKMLQESSEEESDTYGEIGLSKSEAIFHWR-----------------------------NLCYEVQIKAETRRILNNVDGWVKPGTLTA
Snq2p *Saccharomyces cerevisiae*	LKVIFTEYKRPVKGGG---DALIFK----K-GSKRFIAHADEESPDNVNDIDAKEQFSSESSGANDEVF----------DDLEAKGVFIWK-----------------------------DVCFTIPYEGGKRMLLDNVSGYCIPGTMTA
Cdr1p *Candida albicans*	IYIALTEFNKGAMQKG---EIVLFLKGSLK-KHKR--KTAASNKGDIEAGPVAGKLDYQDEAEAVNNEKFTEKGSTGSVDFPENREIFFWR-----------------------------DLTYQVKIKKEDRVILDHVDGWVKPGQITA
PMR1 *Penicillium digitatum*	TYLVGTEYISEAKSKG---EVLLFR----R-GYAP--KNSGNSDGDVEQTHGVSSAEKKDGAGSGGEQESA--------AIQRQTSIFQWQ-----------------------------DVCYDVHIKNEERRILDHVDGWVKPGTCTA
ABC1 *Magnaporthe oryzae*	RTWLRPRMCERKKSKG---EVLVFR----R-GQRPAAIKDAKTDPEAGPPKVGGAVVAANMTGENAG------------FIQRQTSTFGWR-----------------------------DVCYEVQIKKETRRILDHVDGWVKPGTLTA
ABC2 *Magnaporthe oryzae*	MNVVLGEVIEFGMGGN---SALVYQ----K-PNKE--RKELNEKLVAKREAQRSSKSEAQGSD----------------LKIESKRVLTWE-----------------------------NLTYDVPVPGGNRRLLNNVFGYVKPGQLTA
ABC3 *Magnaporthe oryzae*	ITAPLAAAQQAAEACGIFHTIIDFP----K-------PVYGSARGEHEVRADG--------------------------DIVLMNVNFAY-------------------------------------PTRPEVKVLDNLSLVFPAGKVTA
ABC4 *Magnaporthe oryzae*	VGALIERYLYGTATKG---RNI---------------VNGGEMGGEAAVRLEGFTKIY---------------------KPGFIRRMFSF-----------------------------------ISKPKPAVRAVDGLTLSAGRGQILA
MoABC5 *Magnaporthe oryzae*	LLQALPNITSSLACLD---RIQAFLLSKARVDHRG--QLQTTADMDEKPKNVTTTAISTIDVTTGVSTTVDKTPNL---MASFKNASFFY-----------------------------------ATGEKDASFSLSQISMVLPRGSITI
CaABC1 *Colletotrichum acutatum*	VAPNVQAFTTAIAAAA---KIYNTI----D-RVSP--LDPSRDDGTKLEQVEG--------------------------TIRLENIKHIY-------------------------------------PSRPEVTVMDDVSLTIPAGKTTA
MoABC7 *Magnaporthe oryzae*	LGDMLAHVQEAKVSID---RIEEFL------SEEE--TDKFIQLGEDNVNEEGTR------------------------IIALKDAAFIW------------------------------GGKDIIAADGSQAFRLLDIDTEFMIGKLNV
MoABC-R1 *Magnaporthe oryzae*	LFQAMPALGSAHGCFE---RILAFL----K-TPEK--PLTIKNEPETMPESNRGTKDASIQRSEANET-----------ALSIRHASIGW---------------------------------------SPDEPVLKDINLQIQKGSFVA

Walker A: GxSGxGKS

ABC signature: SGGQ

BcatrA *Botrytis cinerea*	LMGASGAGKTTLLNTLSQRQKTG----------VVT-GDMLVDGRP--------LGTEFQRGTGFCEQMDLHDGTATIREALEFSAIL-RQEHTVPRAEKIEYVDKII--DLLEL-------GDMQDALVRSLG----VEQRKRVTIGVE
BcatrB *Botrytis cinerea*	LMGSSGAGKTTLLDVLAQRKTDG----------TIK-GSILVDGRP--------LNVSFQRSAGYCEQLDVHEPLATVREALEFSALL-RQSRTVPDAEKLRYVDTII--DLLEM-------HDMENTLIGNTGAGLSVEQRKRLTIGVE
FgABCC15 *Fusarium graminearum*	VVGPVASGKSTFCKALLG--EVP----------FSS-GEVIVPSTE--------------APIGYCEQTPFL-KNGSIRDNIIWHSVY-NQTRY------EEVLDACL--LRTDL----DILPEGDATTIGSNGIMLSGGQKQRVSLARA
FgABCC9 *Fusarium graminearum*	VIGSVGSGKSSLLSALAG--DMR----------KTN-GQVTFGSSR-----------------AFCPQYAWI-QNTTLKNNIIFGKDIDKAWY-------NKVIQACA--LQADI----DMLPNGDLTEIGERGITISGGQKQRLNIARA
FgABCG6 *Fusarium graminearum*	LMGVSGAGKTTLLDCLADRISMG----------VIT-GEMLVDGKI--------RDSSFQRRTGYVQQQDLHLETSTVREALTFSALL-RQPASTPREEKIAYVDEVI--KLLDM-------QEYADAVVGVLGEGLNVEQRKRLTIGVE
FgABC1 *Fusarium graminearum*	LMGVSGAGKTTLLDCLADRISMG----------VIT-GEMLVDGKI--------RDSSFQRRTGYVQQQDLHLETSTVREALTFSALL-RQPASTPREEKIAYVDEVI--KLLDM-------QEYADAVVGVLGEGLNVEQRKRLTIGVE
FcABC1 *Fusarium culmorum*	LMGVSGAGKTTLLDCLADRISMG----------VIT-GEMLVDGKI--------RDDSFQRRTGYVQQQDLHLETSTVREALTFSALL-RQPASTPRDEKIAYVDEVI--KLLDM-------QEYADAVVGVLGEGLNVEQRKRLTIGVE
AtrB *Botryotinia fuckeliana*	LMGSSGAGKTTLLDVLAQRKTAG----------TIQ-GSVLVDGRP--------LPVSFQRSAGYCEQFDVHEPYATVREALEFSALL-RQPRTTPREEKLKYVDVII--DLLEL-------HDIADTLIGRVGAGLSVEQRKRVTIGVE
AtrA *Botryotinia fuckeliana*	LMGVSGAGKTTLLDVLAHRTTMG----------VIT-GDMFVNGKG--------LDASFQRKTGYVQQQDLHLETATVRESLRFSALL-RQPASVSIREKHDYVESVI--EMLGM-------GDFCRACCGTPGEGLNVEQRKLLTIGVE
Pdr5p *Saccharomyces cerevisiae*	LMGASGAGKTTLLDCLAERVTMG----------VIT-GDILVNGIP--------RDKSFPRSIGYCQQQDLHLKTATVRESLRFSAYL-RQPAEVSIEEKNRYVEEVI--KILEM-------EKYADAVVGVAGEGLNVEQRKRLTIGVE
Snq2p *Saccharomyces cerevisiae*	LMGESGAGKTTLLNTLAQR-NVG----------IIT-GDMLVNGRP--------IDASFERRTGYVQQQDIHIAELTVRESLQFSARM-RRPQHLPDSEKMDYVEKII--RVLGM-------EEYAEALVGEVGCGLNVEQRKKLSIGVE
Cdr1p *Candida albicans*	LMGASGAGKTTLLNCLSERVTTG----------IITDGERLVNGHA--------LDSSFQRSIGYVQQQDVHLPTSTVREALQFSAYL-RQSNKISKKEKDDYVDYVI--DLLEM-------TDYADALVGVAGEGLNVEQRKRLTIGVE
PMR1 *Penicillium digitatum*	LMGVSGAGKTTLLDVLATRVTMG----------VVS-GEMLVDGRP--------RDQSFQRKTGYVQQQDLHLHTTTVREALRFSAIL-RQPRHVSHQEKLDYVEEVI--KLLGM-------EHYADAVVGVPGEGLNVEQRKRLTIGVE
ABC1 *Magnaporthe oryzae*	LMGVSGAGKTTLLDCLADRTSMG----------VIT-GEMLVDGHQ--------RDASFQRKTGYVQQQDLHLQTTTVREALNFSALL-RQPAHVPRAEKLAYVDEVI--RLLDM-------QEYADAVVGVPGEGLNVEQRKRLTIGVE
ABC2 *Magnaporthe oryzae*	LMGASGAGKTTLLDVLASRKNIG----------VIG-GDILVDGKK--------PGKEFQRSTSYAEQLDVHEPTQTVREALRFSADL-RQPFDTPQEEKYAFVEEMI--TLLEL-------EDLADSIIGWPEFGLTVEQRKRVTIGVE
ABC3 *Magnaporthe oryzae*	IVGPSGSGKSTIVGILERWYEFNGDPVLNPLVLYLRNGFVSVGGRL----LTEIDVKWWRNQIGLVQQDNVL-FNTTIYKNVEHGLIG-TLWEHESDEKKAMLIETACRDAFADE--FINRLPDRYQTTVGESGIKLSGGQRQRLAIARA
ABC4 *Magnaporthe oryzae*	LLGANGSGKSTSLDAIAG---IN----------RLTSGSITIDGTG---------------GLGIAPQKNVLWDDVTVEEHIRIFNSL-KAPANKSSKEEIRQLVEAV--DLTKK--------------IKARSKTLSGGQKRKLQLGMM
MoABC5 *Magnaporthe oryzae*	VAGPVGSGKSTLLKAILG--ELE----------LVA-GEVDVNPGP----------------IGFCDQNPWI-SNGTVRQCIQGNSEY-DSELYDDVVRTCALDQDIM--RWGEE---GSGSGNGSDMNVGSRGMALSEGQKHRLALARA
CaABC1 *Colletotrichum acutatum*	LVGASGSGKSTIV-GLVERFYDP----------VR--GSVFLDGHD----ISTLNLRWLRQQMALVSQEPTL-FATTIYQNIRYGLIG-TQHENASEEEQKKLIEEAA--KMANAHDFISGLPEGYLTNVGERGFLLSGGQKQRIAIARA
MoABC7 *Magnaporthe oryzae*	IAGPTGSGKTSLLMALLG--EMT----------LLK-GRVYLPGGRSREDVRPDPETGLAETCAYVAQQAWL-VNANIKDNILFSARF-DEKRY------RDVIVACA--LERDL----EILDNGDETLVGEKGITLSGGQKQRISLARA
MoABC-R1 *Magnaporthe oryzae*	LVGKTGSGKSLLLKSAIG--EGG----------HVS-GSIDISLDK----------------VAYCSQSPWLENISAEHTWTQFGESGDAKWL-------AGVIDACCLDDLTGL-------PDYRTGRIGSGGARLSGGQKQRLALARA

Walker B: LxxDExxSALD

BcatrA *Botrytis cinerea*	LAAKPNLLLFLDEPTSGLDSQSAYSIVRF-----LKKLS-AAGQAIVCTIHQPSS-VLIQEFDMILALNPG------GNTFYFGPVGENGSA------------------------VVKYFADRGV-QCPPQKNVAEFI---------LE
BcatrB *Botrytis cinerea*	LVSKPSILIFLDEPTSGLDGQAAFNTVRF-----LRKLA-DVGQAILVTIHQPSA-QLFAQFDSLLLLAKG------GKTVYFGDIGEDSKT------------------------IKEYFARYDA-PCPESSNPAEHM---------ID
FgABCC15 *Fusarium graminearum*	LYLETDFLL-IDDILSGLDNSTGNDVFRRVFGP-NGLMRRRNATAILCT-HAIRY---LPLADHIIILATD------GTVGEQGPFDKLAETGI-------------------------YIPTLGLSDADDASSTNSSI--------PVE
FgABCC9 *Fusarium graminearum*	IYFDADIVL-MDDPLSAVDAHVGRHIFDN------AILGLLKDKCRILATHQL---WVLSRCDRIIWMEHG--KIQAIDTFEKLMRDHKGFQT---------LM
FgABCG6 *Fusarium graminearum*	LAAKPPLLLFVDEPTSGLDSQTSWAILDL-----LEKLS-KAGQSILCTIHQPSA-MLFQRFDRLLFLAKG------GRTIYFGDIGKNSET------------------------LTNYFVKHGSQECPNGENPAEWM---------LE
FgABC1 *Fusarium graminearum*	LAAKPPLLLFVDEPTSGLDSQTSWAILDL-----LEKLS-KAGQSILCTIHQPSA-MLFQRFDRLLFLAKG------GRTIYFGDIGKNSET------------------------LTNYFVKHGSQECPNGENPAEWM---------LE
FcABC1 *Fusarium culmorum*	LAAKPPLLLFVDEPTSGLDSQTSWAILDL-----LEKLS-KAGQSILCTIHQPSA-MLFQRFDRLLFLAKG------GRTIYFGDIGKNSET------------------------LTNYFVKHGSEECPNGENPAEWM---------LE
AtrB *Botryotinia fuckeliana*	LVSKPSILIFLDEPTSGLDGQSAYNTVRF-----LRKLA-DVGQAVLVTIHQPSA-QLFGEFDSLLLLAKG------GKMVYFGDIGDNGST------------------------VKEYFGRHGA-PCPPNANPGEHM---------ID
AtrA *Botryotinia fuckeliana*	LPPSPKLLLFLDEPTSGLDSQSSWAICTF-----LRKLA-DSGQAVLCTIHQPSA-ILFQEFDQLLFLAKG------GKTVYFGPIGPNSRT------------------------LLDYFESNGARKCDEAENPAEYM---------IE
Pdr5p *Saccharomyces cerevisiae*	LTAKPKLLVFLDEPTSGLDSQTAWSICQL-----MKKLA-NHGQAILCTIHQPSA-ILMQEFDRLLFMQRG------GKTVYFGDLGEGCKT------------------------MIDYFESHGAHKCPADANPAEWM---------LE
Snq2p *Saccharomyces cerevisiae*	LVAKPDLLLFLDEPTSGLDSQSSWAIIQL-----LRKLS-KAGQSILCTIHQPSA-TLFEEFDRLLLLRKG------GQTVYFGDIGKNSAT------------------------ILNYFERNGARKCDSSENPAEYI---------LE
Cdr1p *Candida albicans*	LVAKPKLLLFLDEPTSGLDSQTAWSICKL-----MRKLA-DHGQAILCTIHQPSA-LIMAEFDRLLFLQKG------GRTAYFGELGENCQT------------------------MINYFEKYGADPCPKEANPAEWM---------LQ
PMR1 *Penicillium digitatum*	LAAKPQLLLFLDEPTSGLDSQTSWSILDL-----IDTLT-KHGQAILCTIHQPSA-MLFQRFDRLLFLAKG------GRTVYFGEIGEHSST------------------------LSNYFERNGAPKLSPEANPAEWM---------LE
ABC1 *Magnaporthe oryzae*	LAAKPPLLLFVDEPTSGLDSQTSWAILDL-----LEKLT-KSGQAILCTIHQPSA-MLFQRFDRLLFLAKG------GKTVYFGDIGENSKI------------------------MTDYFERNGGFPCPHDANPAEWM---------LE
ABC2 *Magnaporthe oryzae*	LAAKPELLLFLDEPTSGLDSQSAFNIVRF-----LKKLA-NAGQAILCTIHQPNS-ALFENFDRLLLLQRG------GRCVYFGDIGKDANV------------------------LRDYLKRHGAEASP-TDNVAEYM---------LE
ABC3 *Magnaporthe oryzae*	IVKQPKILI-LDEATSAIDVRSEQIVQAA-----LERAS--RGRTTVVIAHRLGT---VKKADKIIVLSKGQVVQEGTHDELRRQRGSAYYM----------------LANAQSLNVRRRSSRMSIDQTPDEEDDSGYFRTTSMHDGDSD
ABC4 *Magnaporthe oryzae*	LTGGSAVCC-VDEVSSGLDPLSRRKIWDI-------LLAERGKRTMILTTHFLDEADLL--ADHIAVLSKGTLRAEGSSVELKNRLGGGYRIHFLKTKTLQDGPVVEGVTKKVSFDIISYIAPSSALAAEVIRTLEAHD---------IR
MoABC5 *Magnaporthe oryzae*	LYSRAPLLI-LDNTFQALDSRTEATIRDRLLAPTSGWAA-RHGTTVMLATTSCDN-VLLGQADQVVVLDQN------GKM---VPREASTL---------LF
CaABC1 *Colletotrichum acutatum*	VVSNPKILL-LDEATSALDTKSEGVVQAA-----LEVAS--QGRTTITIAHRLST---IKDAHNIVVMSSGRIVEQGTHNDLLAKQGAYYNL--------------------VTAQNIARVNEMDAEEEEAIDAEDDEI---------IR
MoABC7 *Magnaporthe oryzae*	VYSNSKHLL-LDDCLSAVDSHTAQWIFNN-----CIRGPLMKDRTCIMVTHNIP--LCVPHSDFVVVMDNGRITHQ-GRALEVITSGALGEE----------------------------IAQKAKSETPNISRIPSRV---------PS
MoABC-R1 *Magnaporthe oryzae*	IATRKDIVL-LDDVFSALDRTTKHHIATRLLGP-EGLLR-RLGTTVLFATHDSSIANL---ADQVYEITVD---------------GILTPV------------------------LVQ--KPADDEGTKHEDSDAKYT---------VT

BcatrA *Botrytis cinerea*	TAAKGGKRRDGKKINWNEEWLNSNENKTVMQEIAR---IKSERGKIAAPE---------------ASSQREFASPVTL
BcatrB *Botrytis cinerea*	VVSGTLSKGK----DWNQVWLNSPEYEYTVKELDR---IIETAAAAPPGT---------------VDDGFEFATPLWQ
FgABCC15 *Fusarium graminearum*	ETVAAT--------------MPVPLTAFSTVSIGH---LEAESRSTGD-----------------AKVYRHYYQSVN-
FgABCC9 *Fusarium graminearum*	ETTAIEEKREEVEKPIDGDEPTADEKKKKKKKGAA---LMTQEERASASVS--------------WSVYGAYIKASGS
FgABCG6 *Fusarium graminearum*	VIGAAPGSHTDI--DWHQTWRDSSEYQAVQTELQR---LKAEGSANSVDQKSD------------PESYREFAAPFGQ
FgABC1 *Fusarium graminearum*	VIGAAPGSHTDI--DWHQTWRDSSEYQAVQTELQR---LKAEGSANSVDQKSD------------PESYREFAAPFGQ
FcABC1 *Fusarium culmorum*	VIGAAPGSHTDI--DWHQTWRDSSEYQAVQTELQR---LKAEGSANGVDEKSD------------PESYREFAAPFGQ
AtrB *Botryotinia fuckeliana*	VVSGSLSQGR----DWHEVWKASPEHTNAQKELDR---IISEAGSKPPGT---------------VDDGHEFAMPLWQ
AtrA *Botryotinia fuckeliana*	VVNAEVNDHGT---DWFDVWKGSKECQAVKEEIER---IHEKKRGTAGAIEETDD----------GSTKSEFADAILV
Pdr5p *Saccharomyces cerevisiae*	VVGAAPGSHANQ--DYYEVWRNSEEYRAVQSELDW---MERELPKKGSITA--------------AEDKHEFSQSIIY
Snq2p *Saccharomyces cerevisiae*	AIGAGATASVKE--DWHEKWLNSVEFEQTKEKVQD---LINDLSKQETKSEV-------------GDKPSKYATSYAY
Cdr1p *Candida albicans*	VVGAAPGSHAKQ--DYFEVWRNSSEYQAVREEINR---MEAELSKLPRDND--------------PEALLKYAAPLWK
PMR1 *Penicillium digitatum*	VIGAAPGTHSDI--DWPAVWRESPERKAVQNHLAE---LRNNLSLKPVATTDND-----------PAGFNEFAAPFAV
ABC1 *Magnaporthe oryzae*	VIGASPGTTSDI--DWHQAWRESPECADVHAELDR---LKEQVPNTPPPTED-------------KASYREFAAPFHQ
ABC2 *Magnaporthe oryzae*	AVGAGSAPRVGDR-DWADIWEESPEHANVKDTISQ---LKEERKAEVANDSN-------------SALEKEYASPISH
ABC3 *Magnaporthe oryzae*	HTAHSSNYGSDEEDDFIMERPRVRARDDVGVEMSTSTI--HTAHTPVSDGPPDDAAKIQVVEIQDHWLGGFAELLAEQGS
ABC4 *Magnaporthe oryzae*	DYRFSGPTIEDVFLQLAEEVKGETDAASRGSHLASASLQGANAEKTYSSEMVTPDADKDNLELMSGNKVGIPRQALVLFRKRCTVFKHNWFPSFAAFIIPILAAGLCTLFIRGQSPAGCSTQETRSGQDIFSNDDNDGNSLLLLYGPTSP
MoABC5 *Magnaporthe oryzae*	SEEQSPPKNEQIQQQPVEQARPKLNKPDALNDKKT---PPTQPAASKESSKT-------------ASTYRFFFGPVG-
CaABC1 *Colletotrichum acutatum*	KASRASDKGYVA--DPEDNMAAKLQRTNTSKSLSS---IALQNRKDDGEVKYSL-----------WTLIKLIASFNQK
MoABC7 *Magnaporthe oryzae*	SVGEGSGNTLLD--TDGDDHLSKPKNAKKAKKAEA---MEETKATGAVK----------------WPVMKLYLASMGS
MoABC-R1 *Magnaporthe oryzae*	DASNDEKTST------------APDHGTNTVMTHK---VENGGEATGTSVSD-------------KKVYLRYARAMG-

BcatrA *Botrytis cinerea*	QTTELTKRLFTQY----------------------WRDPS--------------------------------YLY--------GKLFTSVIIG---IFNGFTFWQLGHSIID-----------------------------MQNRMFTSF
BcatrB *Botrytis cinerea*	QIKLVTNRMNVAI----------------------YRNTD--------------------------------YIN--------NKFALHIGSA---LFNGFSFWMIKHSVGG-----------------------------LQLRLFTVF
FgABCC15 *Fusarium graminearum*	-GWATMTCLLSGIMYAVGRNFPSIWM-G-------WWGSN------------------------SFDRTSSFYIG---------------IMG---LFRGLQIISLFLCATA------------VVIFMTTQSGKLLHSEILNTLVNAPL
FgABCC9 *Fusarium graminearum*	-ILNAPLVLFLLIISQGANIVTSLWL-S-------YWTSD------------------------KFNLSTGVYIG--------IYAALGVVQAI--LMFAFSVVLSILGTKS---------------------SKVMLRIAVTRVLRAPM
FgABCG6 *Fusarium graminearum*	QLLIATKRVFEQY----------------------WRTPS--------------------------------YIY--------SKAALCIQVG---LFLGLVFLNAPLSLRG-----------------------------LQNQMFAIF
FgABC1 *Fusarium graminearum*	QLLIATKRVFEQY----------------------WRTPS--------------------------------YIY--------SKAALCIQVG---LFLGLVFLNAPLSLRG-----------------------------LQNQMFAIF
FcABC1 *Fusarium culmorum*	QLLIATQRVFQQY----------------------WRTPS--------------------------------YIY--------SKAALCIQVG---LFIGLVFLNAPLSLRG-----------------------------LQNQMFAIF
AtrB *Botryotinia fuckeliana*	QTVIVTKRTCLGV----------------------YRNTD--------------------------------YVN--------NKLALHIGSA---LFNGFSFWKMGASVGE-----------------------------LQFKLFVLF
AtrA *Botryotinia fuckeliana*	PAVCRHVRVFQQY----------------------WRMPE--------------------------------YII--------SKGALAIVAG---LFIGFSFYDAKTSLAG-----------------------------LQTLVFSLF
Pdr5p *Saccharomyces cerevisiae*	QTKLVSIRLFQQY----------------------WRSPD--------------------------------YLW--------SKFILTIFNQ---LFIGFTFFKAGTSLQG-----------------------------LQNQMLAVF
Snq2p *Saccharomyces cerevisiae*	QFRYVLIRTSTSF----------------------WRSLN--------------------------------YIM--------SKMMLMLVGG---LYIGFTFFNVGKSYVG-----------------------------LQNAMFAAF
Cdr1p *Candida albicans*	QYLLVSWRTIVQD----------------------WRSPG--------------------------------YIY--------SKIFLVVSAA---LFNGFSFFKAKNNMQG-----------------------------LQNQMFSVF
PMR1 *Penicillium digitatum*	QLWQCLIRVFSQY----------------------WRTPI--------------------------------YIY--------SKTALCSLTA---LYVGFSFFHAQNSMQG-----------------------------LQNQMFSIF
ABC1 *Magnaporthe oryzae*	QIYAVTHRVFQQY----------------------WRTPS--------------------------------YIY--------AKAALCAVTA---LFIGFVFYDAPNTQQG-----------------------------LQNQMFAIF
ABC2 *Magnaporthe oryzae*	QLKVTSHRTNLAL----------------------WRSPN--------------------------------YLF--------TRLFNHVVIA---IVTGLTYLQLDNSRSS-----------------------------LQYKVFIMF
ABC3 *Magnaporthe oryzae*	RWKLYFVIIIGAIGAGASTPVQAYLF-ATLLNLFSFRGPQ-------------------------VNQLANFFCL--------MFVVLAAGVGISHLFLGWSTTRLGFGLTR-----------------------FYRKEYFKNMISRPA
ABC4 *Magnaporthe oryzae*	TITAALNRTIALIRNGPGDSTGSSAG-S-------PNGGQSSSLFNIESATSFADFNRGIIDNRKNVTPAGFWLGDTNNPPTLAYTSVPEMQN---AFFGQNLMNMLLTNTTLSAKYAAFDT-------PWQDNTGNSLQLLVYICLALS
MoABC5 *Magnaporthe oryzae*	-FFRLAVLVLGVISLAVLTRIQRIWV-Q-------WWTEA------------------------SSRDRYPTFVG--------VYFTFAVGGC---TSFAFVFWWLFMSVTP-------------------ITAGGLHQQLVSTIFSAPL
CaABC1 *Colletotrichum acutatum*	EWKLMLIGLLFSIICGGGNPTQAVFFAKQIMTLSVPITPQ-----------------TTQETLHQMKKDSDFWSA--------MYLMLAGVQFIAFVIQGIAFAKCSERLIH-----------------------RVRDQAFRTMLRQDV
MoABC7 *Magnaporthe oryzae*	-WWFWVVAGCIFISQQASDVVSNLWI-K-------QWASQYTTEVSSVPISTSSHMYGTQSFAPTYMVPVQTYVKDQLARNG-NATALDIVVN---SEVNAQYYLVVLAIIGLAGSLTAFLRDLWIFFGSLTASAKIHTRLLNTVTRAKF
MoABC-R1 *Magnaporthe oryzae*	-FKNAATFLFLVMGCAVCFKIPDLWV-Q-------WWSTA---------------------IKQGTTYSSSYWIG--------ILALLEVLPL---LMLWLSLFHVLFFIVP-------------------RSASTMHDSLLRTVLLAPF

BcatrA *Botrytis cinerea*	LIILIPPTIVNAVVPKFYQNRALWEARELPSRIYGWVAFCTANI----------VAEIP--IAIV-----GAVIYWALWYW-------------------------PTGLPSDSS-------TSGYV--------------------FL-
BcatrB *Botrytis cinerea*	NFIFVAPGVMAQLQPLFLERRDIYETREKKSKMYSWWAFATGNV----------VSELP--YLVI-----CAVLYFVCWYY-------------------------TVGFPSDSS------KAGSVL--------------------FVM
FgABCC15 *Fusarium graminearum*	RFFTTTD--FGAVTNLFSQDMTLIDGELPISLLNTVIQIFDVFA-----MAVVVAVGAP--WLAIAYPVVFSIIYMLQMFYLRTSRQLRLLDLEAKSPLYAHFLDTIRGIATVRAQKLRDQEILFNQ-DLLNLSQRPAYLL-AMAQRFLA
FgABCC9 *Fusarium graminearum*	SFFDTTP--LGRITNRFSRDVDVMDN----NLSDALRMFLLTMGMITSVFILIIAFYY---YFVIALVPLYVAFVVAAMYYRASAREVKRFESVLRSHVFAKFGEGLTGVASIRAYGLQNRFINELR-DSIDEMNGAYYIT-FANQRWLS
FgABCG6 *Fusarium graminearum*	QMLTVFGQLVQMQMPHFVTQRSLYEVRERPSKTYSWKVFMLSQI----------IAEIP--WNTL-----MSVFLFVCIYY-------------------------PVGFNKNAEFAGQTAERGGLM--------------------WL-
FgABC1 *Fusarium graminearum*	QMLTVFGQLVQMQMPHFVTQRSLYEVRERPSKTYSWKVFMLSQI----------IAEIP--WNTL-----MSVFLFVCIYY-------------------------PVGFNKNAEFAGQTAERGGLM--------------------WL-
FcABC1 *Fusarium culmorum*	QMLTVFGQLVQMQMPHFVTQRSLYEVRERPSKTYSWKVFMLSQI----------MAEIP--WNTL-----MSIFLFVCIYY-------------------------PVGFQKNAEFAGQTAERGGLM--------------------WL-
AtrB *Botryotinia fuckeliana*	NFIFAAPGGIGQVQALFIERRDIYDAREKKSRIFSWVGFVTGLI----------VSELP--YLVL-----CAVLYFVCFYY-------------------------QTGLPTSSD-------KAGAV--------------------FF-
AtrA *Botryotinia fuckeliana*	MVCALFAPLVNQIMPLFITQRSLYEVRERPSKAIPGKLPDCNIL-----------VEIP--YQVL-----MGILTFVCYYY-------------------------PLSVPAKDQ-------TERAL--------------------VLL
Pdr5p *Saccharomyces cerevisiae*	MFTVIFNPILQQYLPSFVQQRDLYEARERPSRTFSWISFIFAQI----------FVEVP--WNIL-----AGTIAYFIYYY-------------------------PIGFYSNASAAGQLHERGALF--------------------WL-
Snq2p *Saccharomyces cerevisiae*	ISIILSAPAMNQIQGRAIASRELFEVRESQSNMFHWSLVLITQY----------LSELP--YHLF-----FSTIFFVSSYF-------------------------PLRIFFEAS-------RSAVY--------------------FLN
Cdr1p *Candida albicans*	MFFIPFNTLVQQMLPYFVKQRDVYEVREAPSRTFSWFAFIAGQI----------TSEIP--YQVA-----VGTIAFFCWYY-------------------------PLGLYNNATPTDSVNPRGVLM--------------------WM-
PMR1 *Penicillium digitatum*	MLMTIFGNLVQQIMPHFVTQRSLYEVRERPSKTYSWQAFMSANI----------LVELP--WNAL-----MSVLIFLCWYY-------------------------PVGLQRNAS-ADDLHERGALM--------------------WL-
ABC1 *Magnaporthe oryzae*	NILTVFGQLVQQTMPHFVIQRDLYEVRERPSKVYSWKVFMLSQI----------IVEIP--WNSL-----MAVIMFFCWYY-------------------------PVGLERNAILADQVTERGALA--------------------FL-
ABC2 *Magnaporthe oryzae*	QVTVLPALILSQVEAMYHVKRGIF-FRESSSKMYNTSAFAASML----------LAELP--YVVL-----CAVAFFLPLYY-------------------------MPGFTYDSS-------RAGYQ--------------------FL-
ABC3 *Magnaporthe oryzae*	SFFDEEDHTVGSLTARLATDPTQLQQLLGVNMAFVLVSIFNVIG----CCIVGFVFGWK--LTIVSLASTMPIIVVAMAYRVRHEVRLEAEASKVFAEGARFASESIAAIRTVSSLTMEDGVGTRYE-ELLNKHVRQA----FSKARWSL
ABC4 *Magnaporthe oryzae*	AYPAFFALYPNVERRRFVRGLQYSNGVRQFPLWIAYVLFDFINVVISSAIVIGLFVAIADVWYHAGYLFLILVLYGLCAILLSYN-------ISIFASNQLSAYAFAAGFNAVTFLVYLIGYMATITFARVDRVDSSLLVVNFVVSAFAP
MoABC5 *Magnaporthe oryzae*	SYFSGID--SGIILNRFSQDMAVLNGMLPISIFQTTSTGALIIG----QLALI-MYGAN--YMSISLPFTILALWLLANFYLRTSRQLRVLELELKAPIYTLLTETMEGLSTIRAFGWQSWYLGRCH-VRVDGSKRAIYLL-FMIQRWLG
CaABC1 *Colletotrichum acutatum*	AFFDKDSNTAGALTSFLSTETTHLAGLSGVTLGTLLMVTTTLVA----ALALAIAIGWK--LALV-CTATIPILIGCGFFRFWMLAHFQRRSKTAYSNSASYASEAISAIRTVASLTREDDVIRQYQTSLAIQQRAS----------LIS
MoABC7 *Magnaporthe oryzae*	RFFDVTP--LGQMMNRFSKDMEAVDQEVAPIAIGILSCALGITV-----TVVLIASITP--GFLIAAVFITIAYVLLAKFYLASSRDLKRLESVQRSPLFQQFGETLSGVTTIRAYGDERRFVRDNL-TRINGQLRPMIYL-WATNRWLA
MoABC-R1 *Magnaporthe oryzae*	GFISRVD--TGSLMNRFNQDLMFVDTRLPIDLFNTSIDFFITII----QLILVVLVSKE--ALAIL-PVVFGALYLIQKVYLRSSKQLRLLDLDWKADLHTAFGETTAGLSVIRANGWLDPMRAKFA-EKLDRSQEPFYLL-YMVQRWLQ

BcatrA *Botrytis cinerea*	MTMLFFLFQASWGQWICAFAPSFTVISNVL----PFFFVMFGLFNGVV--RPYSQIS--VFWRY-----------WLYYVNPATY----------WIG
BcatrB *Botrytis cinerea*	ICYEFI--YTGIGQFVAAYAPNVVFASLVN----PLVIGTLVSFCGVL--VPYAQIT--EFWRY-----------WMYYLNPFNY----------LMG
FgABCC15 *Fusarium graminearum*	TFLNLIVMVLAVGVVAISTQLRTN--SGFA----GSSLVTLMSWGESI--SSLIQYYTQVEVSIGAVSRLKAF-------------------AQNVPSENLDQEDLEPA--EEWPTQGD--------IAIRGVSASYKNDDESPTSEDDE
FgABCC9 *Fusarium graminearum*	MRIDLIGVLLVFVTAILVVTSRFSINPSIG----GLVLSYILSIVGMM--QFSVRQLAEVENAMNAVERLYYY----------------------GTELEEEAPSHTVEVRKSWPEKGE--------IVFDNVEMRY------------R
FgABCG6 *Fusarium graminearum*	LIWQFLIFTCTFAHAAIAITDTAEAGGNLA----NVVFMMSLFFCGVL--AAPDKMP--GFWI------------WMYRVSPFTY----------LVS
FgABC1 *Fusarium graminearum*	LIWQFLIFTCTFAHAAIAITDTAEAGGNLA----NVVFMMSLFFCGVL--AAPDKMP--GFWI------------WMYRVSPFTY----------LVS
FcABC1 *Fusarium culmorum*	LIWQFLIFTCTFAHAAIAITDTAEAGGNLA----NVVFMMSLFFCGVL--AAPDKMP--GFWI------------WMYRVSPFTY----------LVS
AtrB *Botryotinia fuckeliana*	VMLLYEGLYTGIGQFISAYAPNAVFATLTN----PLVIGTLVSFCGVL--VPYGQIQ--EFWRY-----------WIYWLNPFNY----------LMG
AtrA *Botryotinia fuckeliana*	FCIQFYVYASTFAHMCIAAMPNAETASPIV----ILLFSMCLTFCGVM--QPPDALP--GFWI------------FMYRVSPFTY----------WVA
Pdr5p *Saccharomyces cerevisiae*	FSCAFYVYVGSMGLLVISFNQVAESAANLA----SLLFTMSLSFCGVM--TTPSAMP--RFWI------------FMYRVSPLTY----------FIQ
Snq2p *Saccharomyces cerevisiae*	YCIMFQLYYVGLGLMILYMSPNLPSANVIL----GLCLSFMLSFCGVT--QPVSLMP--GFWT------------FMWKASPYTY----------FVQ
Cdr1p *Candida albicans*	LVTAFYVYTATMGQLCMSFSELADNAANLA----TLLFTMCLNFCGVL--AGPDVLP--GFWI------------FMYRCNPFTY----------LVQ
PMR1 *Penicillium digitatum*	LILTFMLFTSTFSHMMIAGIELAETGGNLA----NLLFSLCLIFCGVL--ATPDKMP--HFWI------------FMYRVSPFTY----------LVS
ABC1 *Magnaporthe oryzae*	YLWGFLIFTSTFTDLMIAGFETAEAGGNIA----NLFFSLCLIFCGVL--ANPDTMP--RFWI------------FMYRVSPFTY----------IVS
ABC2 *Magnaporthe oryzae*	MILITEFFSITLAQALSSITPSTFISSQLD----PFLMITFSLFCGVT--IPFPQMP--DGYK------------WLYQLDPFTR----------LIG
ABC3 *Magnaporthe oryzae*	LLFSF-SDSISFLCMAFVLWYGGRLLASRE----YSPFQYVIVYIAVVQGAMSAGQWLSFGPNIAHATAAADR--------------------VLDMREADDELDRGLPLIDPNEDAMLEEKEGAE-VELRDVWFSY------------P
ABC4 *Magnaporthe oryzae*	IGSAVRGFFIALNLFSTTCQDQELAPNPGGFLQYGSPIMYLIIQAILL---FLLLLWLDSGTIRATWQRLFGRKNKNANQQAADISDEEVAGELVRVTSSATDPSRRRQ--DTKGADGVSSSDKDG-LQVVHVTKTF-------------
MoABC5 *Magnaporthe oryzae*	FVLDCTVAALATLMVILATQLRESTNSASL----GVGLSSVIGFSVLV--AQLVATYTEVENSLGAVDRIRDC-------------------VEMVPSEEDESRLVQPP--AEWPTRGH--------LEFRNVAAGY---------TGKD
CaABC1 *Colletotrichum acutatum*	ILKSSLLFAASQSFMFLAFALGFWYGGTLIANGEYNMFQFFVCFSAVIFGAQSAGSIFSFAPDMGKAHQAANELK---------------------------ILFDRKPTIDTWSKEGASLDAVDGTLEFRDVHFRY-----------PT
MoABC7 *Magnaporthe oryzae*	FRTDLLGDFVSFFAGVFVILSIGVIDAGWA----GISLSYAIGFAENI--LWLVRLYSINEQNMNAVERIKEY-------------------LEVEQEAAPICEKNRPP--QNWPAQGS--------VEFINYTTSY------------R
MoABC-R1 *Magnaporthe oryzae*	LVLNLVVAGLAIAIAGVAIGLRDKVAAGAV----GVALLNTTTLGETL--TNFIMSWTSLETSLGAIARVCTF-------------------EQDTPREREEPSTTDLP--DNRPGAGQ--------ISFENVWATY---------EDEG

Walker A: GxSGxGKS

BcatrA *Botrytis cinerea*	GIIAATLSNVPIECASNEAAYFNPPSGQTCSSYA---------------SDFVT----------SAGVGYLT---NPDATTN--------------------------------CGYC------------------------------
BcatrB *Botrytis cinerea*	SLLVFTSWDTPVNCHESEFAIFNPANG-TCGEYL---------------SSYLQGM---------GAAANLI---NPDATEG--------------------------------CRVC------------------------------
FgABCC15 *Fusarium graminearum*	ESPNLALEDLTILVKPGQKVALCGRTGSGKSSIILLLLRLLDPLSNQSENIVIDGVPYNRVNRSILRRRLIAVPQDPVFLPDGSSIKENLDPFNVAS--------DEECLAVLEDVRLTKFATDHGSI----------------------
FgABCC9 *Fusarium graminearum*	AGLPLVLSGLTMHVKGGERIGIVGRTGAGKSSIMSTLFRLVE---ISGGKITIDGLDISTLGLHDLRSRLAIIPQDPTLFRG--TVRSNLDPFSEHT--------DLELWYALRKADLVSADAETPED------------ARRTNDPSR
FgABCG6 *Fusarium graminearum*	AILSTGIANAEVKCAANELTTFNPTNGTTCGEYM---------------NSYIK-----------AAGGYLT---NPDATSD--------------------------------CKFC------------------------------
FgABC1 *Fusarium graminearum*	AILSTGIANAEVKCAANELTTFNPTNGTTCGEYM---------------NSYIK-----------AAGGYLT---NPDATSD--------------------------------CKFC------------------------------
FcABC1 *Fusarium culmorum*	AILSTGIANAEVKCAANELTTFNPPNG-TCGDYL---------------ESYIK-----------AAGGYLT---NPDATSD--------------------------------CKFC------------------------------
AtrB *Botryotinia fuckeliana*	SLLTFTIFDVDIKCRESEFATFDPPNGSSCIDYL---------------STIFKGW---------GVSANLI---NPDATSQ--------------------------------CQVC------------------------------
AtrA *Botryotinia fuckeliana*	GMATTQVHGREVVCGENELSIFDPPTNQTCGQYM---------------ERYIS-----------VAGGQVL---NPSATAG--------------------------------CEYC------------------------------
Pdr5p *Saccharomyces cerevisiae*	ALLAVGVANVDVKCADYELLEFTPPSGMTCGQYM---------------EPYLQ----------LAKTGYLT---DENATDT--------------------------------CSFC------------------------------
Snq2p *Saccharomyces cerevisiae*	NLVGIMLHKKPVVCKKKELNYFNPPNGSTCGEYM---------------KPFLE-----------KATGYIE---NPDATSD--------------------------------CAYC------------------------------
Cdr1p *Candida albicans*	AMLSTGLANTFVKCAEREYVSVKPPNGESCSTYL---------------DPYIK-----------FAGGYFETRNDGS------------------------------------CAFC------------------------------
PMR1 *Penicillium digitatum*	AMLSTGTSGAKVECESVELLHFEPTAGKTCFEYM---------------NTYMNGLVVNGTQVAAPAGGYLV---DNNATSN--------------------------------CAFC------------------------------
ABC1 *Magnaporthe oryzae*	GLLSVAVANSEVRCASNEFLHFDPLNG-TCAEFM---------------RNYINGTTIPGLGRIPGAGGYLR-PDTESSRSN--------------------------------CAFC------------------------------
ABC2 *Magnaporthe oryzae*	GMVTTALHDLDVKCASIELNKFTPANGSTCEEYM---------------RPFFN----------AGGNGYIV---DPSSTAQ--------------------------------CDYC------------------------------
ABC3 *Magnaporthe oryzae*	TRPGTILKGLDIKVERGQFAAIVGPSGSGKTTVISLLERFYG---ADSGQVLYNGHDVLDLEPSAYRSNVSLVAQEPHLLSG--SMRDNV-LLGIEDE---STVVHADIYAACQEAGLHDFISSLPEG----------------------
ABC4 *Magnaporthe oryzae*	-GKNTAVDNVTFGVKHGEVFALLGPNGAGKSTSLNMIRGDLQP--SPGGDVFVEGVSVSK-QLAAARANLGVCPQYDAIDVM--TVTEHLRFYARVRG---IADVDRQVEAVIRAVGLELFRDRQAF-----------------------
MoABC5 *Magnaporthe oryzae*	GVTTKVIQDLTFSVEPGQKVWLCGRTGSGKSTILNLLLRLMD---ASSGEILVDGEDMARISPSAVKKAFAVIPQSPFFLPG-TTVRISLGLYASSSEGDLAQIPDEEMISVLNEVGLWDHIRSQAGE----------------------
CaABC1 *Colletotrichum acutatum*	RPEQPVLRGLNLVIRPGQYVALVGASGCGKSTTIALLERFYD---PLSGAIFVDGKEISTLNVNEYRSFIALVSQEPTLYQG--TIKENI-LLGAHTE-----VTDEAIEFACREANIYDFIVSMPEG----------------------
MoABC7 *Magnaporthe oryzae*	KELDPVLRNVTFKISPQEKVGIVGRTGAGKSSLALAIFRALE---ADGGKILIDGIDIGLIGLRDLREAITIVPQEPTLFTG--TIRSNLDPFHLFT--------DEQIYKSLQRVQLIGPDETIPTADASPVLPASPTTPGGASNKNIF
MoABC-R1 *Magnaporthe oryzae*	CGSNWGLSGITLAVQPGERVAVCGRTGSGKSTLLLALLGMLH---TPAGSIRIDGVDTSTLPIDVLRRRFTVVSQDSFFEPT-STFRQELDPSGDMS--------DQIIEEVLRECRAWEIVDGSGGL------------GGKRADA--

BcatrA *Botrytis cinerea*	------------PYASGEEYMKTLNVT-PQD-------------------------------------KWRNFGIFLAFCISNWALVY--FFIYTVRIRGWSFGFATLFGGLGKM------VGKVKSAFKGKEKKSVTESK---------
BcatrB *Botrytis cinerea*	------------EYTVGNDYLKGLNLKTYSY-------------------------------------GWRDAGICALF----VFSGY-GLVFLLMKLRTKKTKGAE---
FgABCC15 *Fusarium graminearum*	-----HAGIRADELSAGQKQLFSLGRAVLRRRVKQRLFSIHGGVLLLDEVSSSVDSATDNLVQEIIKEEFADYTIVMVSHRLNIVMEYFDSVVVLDRGRVVETGDPRDLAKTEGSWFSQLWAMEKNLVLLSRTQHLRLQCLNLANSPRAS
FgABCC9 *Fusarium graminearum*	IHLDTAVEEDGLNFSLGQRQLMALARALVRG----------AQIIVCDEATSSVDMETDDKIQATMAVGFRGKTLLCIAHRLRTIIGY-DRICVMDTGRIAELDTPLQLWKQGGIFRSMCDRSGIRMEDIHGAREELSSAVGESSRQ---
FgABCG6 *Fusarium graminearum*	------------TIKSTNVYLKALSAS-YDD-------------------------------------RWRNFGIGMVYIVVNIVGAL--FLYWLIRMPKNK--------------------------------NKKKTA----------
FgABC1 *Fusarium graminearum*	------------TIKSTNVYLKALSAS-YDD-------------------------------------RWRNFGIGMVYIVVNIVGAL--FLYWLIRMPKNKNKKKTA--
FcABC1 *Fusarium culmorum*	------------TIKDTNVYLKALSAS-YDD-------------------------------------RWRNFGIGMVYIVVNIVGAL--FLYWLVRMPKNKNKKKA---
AtrB *Botryotinia fuckeliana*	------------QYTRGSDYLYSLNLKDYYY-------------------------------------GWRDTAIVALFVLSSYA-----LVYGLMKLRTKA--------------------------------SKKAEE----------
AtrA *Botryotinia fuckeliana*	------------SLTVADEYLAASQIY-WSD-------------------------------------RWRNFGLIWVYIGFNIFVAT--AVYYLFRVKKWN--------------------------------GRRKK-----------
Pdr5p *Saccharomyces cerevisiae*	------------QISTTNDYLANVNSF-YSE-------------------------------------RWRNYGIFICYIAFNYIAGV--FFYWLARVPKKN--------------------------------GKLSKK----------
Snq2p *Saccharomyces cerevisiae*	------------IYEVGDNYLTHISSK-YSY-------------------------------------LWRNFGIFWIYIFFNIIAMV--CVYYLFHVRQSSFLSP--------------------VSILNKIKNIRKKKQ---------
Cdr1p *Candida albicans*	------------QMSSTNTFLKSVNSL-YSE-------------------------------------RWRNFGIFIAFIAINIILTV--IFYWLARVPKGN-------------------------------REKKNKK----------
PMR1 *Penicillium digitatum*	------------TIADTDTYLASVLSY-YKD-------------------------------------AWRNFGIMWAFIIFNIFGAV--CIYWLARVPKGT-------------------------------RSKKTKTA---------
ABC1 *Magnaporthe oryzae*	------------PIKDTNIFLQGAHAN-YND-------------------------------------RWRNFGLIFVYIIFNIIAAL--FVYWAVRVPKKKLGGKDAAAGVGAG--------------AGAARASASNEKGKMQREKGE
ABC2 *Magnaporthe oryzae*	------------AYSSGDQFYTPLGMT-FDN-------------------------------------RWRDLGIYLAFCASNIV-----IIFVANRFLNFNKR--
ABC3 *Magnaporthe oryzae*	--YSTEVGARGVALSGGQKQRLSIARALIRR----------PALLLLDEATSALDSETERAVQETFEATKGSRTMIVVAHRLATVKNA-DVIFVMADGKVIEQG------------------DHVSLLERRGVYYEMCQSQALDR-----
ABC4 *Magnaporthe oryzae*	------------ALSGGNKRKLSLGIALMGN----------PSVILLDEPSSGLDAAAKRIMWRTLAGTVPGRSILLTTHSMEEADALAGRVGILAK-RMLAMGSADNLRHRFGD------LLHVHIVLKGAPRTSDADAERVRNWIVQT
MoABC5 *Magnaporthe oryzae*	--LGLGLNMDKLPLSHGQRQLFGFAMTMLKV---KRQ---GSKIVLMDEPASGCDQETMMEMSALIRRAFESCTVLAVTHSSDIISEY-DMVVSLDN---
CaABC1 *Colletotrichum acutatum*	--FNTVVGSKGALLSGGQKQRIAIARALIRD----------PKILLLDEATSALDSESEHVVQAALDKAAKGRTTIAVAHRLSTIQKA-DIIYVFDQGRIVEQG------------------THAELMKKNGRYAELVNLQSLEKNS---
MoABC7 *Magnaporthe oryzae*	LNLSSKVSESGSNLSQGQRQLLCLARALLKE----------PRVLVMDEATASIDYATDSKIQDTIR-EMKDTTIITIAHRLQTIADY-DKVLVLDKGEVVEYAHPWELMRKGEG------GSFKSMCDMSGDTELLAKAAKKAFDAKKL
MoABC-R1 *Magnaporthe oryzae*	------------NLSAGEVQLLAIARLVLQWQSQPAG---SGGIILLDEATSNLDRQTEVLVESIMAARLQHATVVSVMHRLEAVAAY-DKVAVLDKGVLVDFG---------------------------PVTDVMARCELFTG-----

BcatrA *Botrytis cinerea*	--
BcatrB *Botrytis cinerea*	--
FgABCC15 *Fusarium graminearum*	ISYKYSNYLASELHSAHRDNTNILRGLNKKPSGFQLQSLQTYTVEFIRALHKAFFEQATSQSPFCYSRKAVMETAMRVFAWPPSLRLISSPEDRDLRYTTQTLVRHFTFMDRAI---
FgABCC9 *Fusarium graminearum*	--
FgABCG6 *Fusarium graminearum*	--
FgABC1 *Fusarium graminearum*	--
FcABC1 *Fusarium culmorum*	--
AtrB *Botryotinia fuckeliana*	--
AtrA *Botryotinia fuckeliana*	--
Pdr5p *Saccharomyces cerevisiae*	--
Snq2p *Saccharomyces cerevisiae*	--
Cdr1p *Candida albicans*	--
PMR1 *Penicillium digitatum*	--
ABC1 *Magnaporthe oryzae*	VEGLTTAVLGTSVAGSDAPMTTTTEGEGERAKRRTSGDEVVR---
ABC2 *Magnaporthe oryzae*	--
ABC3 *Magnaporthe oryzae*	--
ABC4 *Magnaporthe oryzae*	LPGAEVEEKVYHGQLRFSVPASVVSGQKQRTTEGGEGDEITREGQAAASSRSSQSAVGKLVVMLEENKHLGIEHYAVSPTTLDQVFLTIVGKHNVKEEGYNEDEKPTGWKRLFSRRK
MoABC5 *Magnaporthe oryzae*	--
CaABC1 *Colletotrichum acutatum*	--
MoABC7 *Magnaporthe oryzae*	IDVDDEAADAAP---
MoABC-R1 *Magnaporthe oryzae*	--
